# Silencing of *HvGSK1.1*—A GSK3/SHAGGY-Like Kinase–Enhances Barley (*Hordeum vulgare* L.) Growth in Normal and in Salt Stress Conditions

**DOI:** 10.3390/ijms21186616

**Published:** 2020-09-10

**Authors:** Yuliya Kloc, Marta Dmochowska-Boguta, Andrzej Zielezinski, Anna Nadolska-Orczyk, Wojciech M. Karlowski, Waclaw Orczyk

**Affiliations:** 1 Department of Genetic Engineering, Plant Breeding and Acclimatization, Institute–National Research Institute, Radzikow, 05-870 Blonie, Poland; y.kloc@ihar.edu.pl (Y.K.); m.dmochowska@ihar.edu.pl (M.D.-B.); 2 Department of Computational Biology, Institute of Molecular Biology and Biotechnology, Faculty of Biology, Adam Mickiewicz University, Uniwersytetu Poznańskiego 6, 61-614 Poznan, Poland; andrzejz@amu.edu.pl (A.Z.); wmk@amu.edu.pl (W.M.K.); 3 Department of Functional Genomics, Plant Breeding and Acclimatization, Institute–National Research Institute, Radzikow, 05-870 Blonie, Poland; a.orczyk@ihar.edu.pl

**Keywords:** brassinosteroids, *HvGSK*, *HvGSK* expression, RNAi, gene silencing, biomass, kernel weight, salt stress

## Abstract

Glycogen synthase kinase 3 (GSK3) is a highly conserved kinase present in all eukaryotes and functions as a key regulator of a wide range of physiological and developmental processes. The kinase, known in land plants as GSK3/SHAGGY-like kinase (GSK), is a key player in the brassinosteroid (BR) signaling pathway. The *GSK* genes, through the BRs, affect diverse developmental processes and modulate responses to environmental factors. In this work, we describe functional analysis of *HvGSK1.1*, which is one of the *GSK3/SHAGGY*-like orthologs in barley. The RNAi-mediated silencing of the target *HvGSK1.1* gene was associated with modified expression of its paralogs *HvGSK1.2*, *HvGSK2.1*, *HvGSK3.1*, and *HvGSK4.1* in plants grown in normal and in salt stress conditions. Low nucleotide similarity between the silencing fragment and barley *GSK* genes and the presence of BR-dependent transcription factors’ binding sites in promoter regions of barley and rice *GSK* genes imply an innate mechanism responsible for co-regulation of the genes. The results of the leaf inclination assay indicated that silencing of *HvGSK1.1* and the changes of *GSK* paralogs enhanced the BR-dependent signaling in the plants. The strongest phenotype of transgenic lines with downregulated *HvGSK1.1* and *GSK* paralogs had greater biomass of the seedlings grown in normal conditions and salt stress as well as elevated kernel weight of plants grown in normal conditions. Both traits showed a strong negative correlation with the transcript level of the target gene and the paralogs. The characteristics of barley lines with silenced expression of *HvGSK1.1* are compatible with the expected phenotypes of plants with enhanced BR signaling. The results show that manipulation of the GSK-encoding genes provides data to explore their biological functions and confirm it as a feasible strategy to generate plants with improved agricultural traits.

## 1. Introduction

Glycogen synthase kinase 3 (GSK3) is a highly conserved kinase present in all eukaryotes and functioning as a key regulator of a wide range of physiological and developmental processes. The kinase, known in land plants as GSK3/SHAGGY-like kinase (GSK), is a key player in the brassinosteroid (BR) signaling pathway and, through the BRs, the GSKs affect diverse developmental processes and modulate responses to environmental factors [1]. The regulatory circuits connecting BR-dependent receptors at the cell surface with genetic effectors regulating more than 1000 genes have been defined in the last decade (for comprehensive reviews, see Zhu et al. [2], Belkhadir, and Jaillais [3]). Briefly, BR-dependent signaling is initiated by binding of the brassinolide (BL) molecule by BRI1/BRL1-BRL3 kinases. This step triggers the phosphorylation cascade of several components including BSK kinase and BSU1 phosphatase. The former specifically dephosphorylates and inactivates BIN2 kinase [4], which is a homologous protein showing high sequence identity to GSKs. The BIN2/GSK3 phosphorylates the two BR-dependent transcription factors (TF) BZR1 and BES1/BZR2 [2,5,6]. This consecutively inhibits their nuclear localization and DNA-binding activity [7,8] and, consequently, restrains BR-dependent gene regulation. BZR1 and BZR2 regulate expression of thousands of genes by interacting with other transcription regulators and binding to BR-responsive promoters [9,10]. Thus, BIN2/GSK3 functions as a negative regulator of BR signaling [6,11]. Initiation of the signaling cascade by BRs leads to inhibition of BIN2/GSK3 kinase, accumulation of TFs BZR1/BZR, and promotion of BR-dependent genes and traits.

Zhu et al. [12] added KIB1 as another component of the signaling cascade. This protein interacts in a BR-dependent mode with BIN2 and directs it to ubiquitination. Thus, KIB1 functions as an important positive regulator of BR signaling, which also acts under the BRs’ control. The activity of BIN2/GSK3, accumulation of BZR1, and BR-dependent growth stimulation are regulated by the Target of Rapamycin1 (TOR1). The TOR1 protein, functioning in the sugar signaling pathway, integrates nutrient and energy signaling with growth homeostasis. This step intertwines sugar-dependent and BR-dependent signaling and provides balanced regulation of steroid-promoted growth with carbon and energy availability [13,14]. GSK3/SHAGGY-like kinases (GSK) are ancient non-receptor Ser/Thr protein kinases found in all eukaryotes. Mammalian GSK3 exists in two isoforms, GSK3α and GSK3β, differing in the localization of their phosphate-binding pocket [15]. This domain in land plants’ GSKs is identical to mammalian GSK3β [16]. Additionally, in plants, the GSK proteins are encoded by multigene families.

There are 10 *Arabidopsis* GSK3/SHAGGY-like kinases (AtSK or ASK) assigned to four functional/phylogenetic groups [16]. They are involved in regulating diverse cellular and developmental processes including plant growth [6], flower [17], stomatal [18,19] and root development [20], seed size [21], and responses to environmental stresses [22,23]. At least 7 of the 10 *Arabidopsis AtSKs* (i.e., *AtSK11*, *AtSK12*, *AtSK13* from group I, *AtSK21*, *AtSK22*, *AtSK23* from group II and *AtSK31* from group III) function in BR-dependent regulation, as it was shown in genetic screens and application of GSK3-specific inhibitor bikinin [24,25]. Since no data indicate similar roles for *AtSK32*, *AtSK41*, and *AtSK42*, their detailed functions remain to be elucidated. The structure of the ATP-binding pocket and sensitivity to bikinin indicate involvement in BR-dependent signaling. In contrast, insensitivity to bikinin, observed in AtSK42, argues against such a function [24]. The function related to carbohydrate metabolism and salt tolerance of group IV GSKs may be anticipated based on *MsK4*, which is the *Medicago sativa* ortholog of *AtSK41*. The gene was reported to regulate salt tolerance by adjusting carbohydrate metabolism in response to environmental stress [26,27].

Screening of genetic mutants and the results of exogenous application of BRs indicate that this group of phytohormones plays an important role in plant tolerance to abiotic stresses. BR application enhanced tolerance to freezing or chilling stress, a water deficit, and salt stress [28,29,30,31,32]. Treatments with BRs alleviated injuries caused by herbicide or insecticide treatments [33,34]. Zhu et al. [35] reported that enhanced salt tolerance observed in *Nicotiana benthamiana*, as a result of treatment with 0.1 µM brassinolide (BL), was induced via the nitric oxide signaling pathway. Exogenous BL application to rice improved plant growth and salinity tolerance by better osmoprotection, which is an increased level of proline and higher activities of antioxidant enzymes [36]. Compatible results, presented by Yusuf et al. [37], indicated that 24-epibrassinolide (EBL) treatment of wheat alleviated aluminum and salt stress injuries by modifying proline metabolism and improving Reactive Oxygen Species (ROS) scavenging. The ubiquitin proteasome system, known to participate in plant responses to environmental stresses was reported by Cui et al. [38] to be tightly connected with BR-dependent mechanisms of growth and salt tolerance. A number of reports on BR-dependent traits of cultivated plants have addressed the molecular mechanisms of BR-dependent regulation with the focus on *GSK* participation. Grain length locus *GL3.3* in rice was annotated as *OsGSK41* (*OsGSK5*). Plants with the *gl3.3* loss-of-function mutation had increased cell length in spikelet hulls and elevated grain weight, which indicates the negative regulation of the trait by *OsGSK41*. Another mechanism of the effect of *OsGSK41* on grain weight was reported by Hu et al. [39]. Investigating the *qTGW3* locus and the *qTGW3*-encoded OsGSK41, they observed an interaction of this kinase with AUXIN RESPONSE FACTOR 4 and auxin-dependent signaling. The grain weight, although BR-dependent, requires tight interaction of BRs with auxin. Several *GS* loci (such as *gs3* and *GS9*) further enhanced the trait by epistatic interaction with *gl3.3* by producing extra-long grains [40]. The opposite mode of the *OsGSK5* function was reported by Thitisaksakul et al. [41], where overexpression of the gene correlated with higher starch accumulation and better growth under severe salinity stress. As the authors highlighted, the results were compatible with another possible role of the *GSKs* assigned to group IV, namely, regulation of carbohydrate metabolism and the osmotic stress response.

Developmental processes and plant architecture pose another important target for modifications. The proper size of rice mesocotyls, which is a key trait facilitating rice cultivation, was found by Sun et al. [42] to be under strong selection during rice domestication. The authors revealed that *OsGSK2* determine mesocotyl length variation and certain variants were selected as a result of rice domestication. BRs regulate leaf erectness, which is a trait with a potential strong impact on rice productivity. In this case, the signaling involves GSK3-like kinases and U-type cyclin [43]. Coordinated, organ-specific expression of cotton GSK3-like kinases and their involvement in the regulation of cotton fiber development and stress responses were reported by Wang et al. [44]. In barley, based on encoded amino acid sequences and protein kinase domains, seven transcriptionally active GSK-encoding genes were assigned to four groups, which is similar to *AtSKs* in *Arabidopsis* [45].

In this work, we describe the functional analysis of *HvGSK1.1*, which is one of the *GSK3/SHAGGY*-*like* orthologs in barley. RNAi-mediated silencing of the target *HvGSK1.1* gene was associated with modified expression of its paralogs *HvGSK1.2*, *HvGSK2.1*, *HvGSK3.1*, and *HvGSK4.1* in plants grown in normal conditions and in salt stress conditions. The expression changes of the target gene and its paralogs is discussed as the mutual co-regulation of genes belonging to one gene family. The conclusion is supported by the analysis of transcription factor binding sites identified in the promoter regions of the genes. The strongest phenotype of transgenic lines with downregulated *HvGSK1.1* was greater biomass of the seedlings and greater weight of 1000 kernels. Both traits showed a strong negative correlation with the transcript level of the target gene and the selected paralogs. The characteristics of the plants are in agreement with the traits known to be regulated by BRs.

## 2. Results

The target *HvGSK1.1* gene was annotated in our earlier study [45]. It was selected for the silencing experiments because of its similarity to rice *OsGSK1*, which is a knock-out mutation that enhanced plant tolerance to abiotic stresses [46]. The barley *HvGSK1.1* has 12 exons and a similar exon-intron structure to the rice *OsGSK1*. The transcripts of *OsGSK1* and *HvGSK1.1* showed 73% nucleotide similarity. Kinase domains of the peptides encoded by the genes showed 84.2% identity and the identical pattern of the Lys-containing ATP-binding site and Asp-containing kinase active sites (Figure S1). The proteins encoded by the genes *HvGSK1.1* (HORVU3Hr1G034440) and *OsGSK1* (Os01g10840) showed a high level of conservation, e.g., very similar sequence length (406 and 403 aa residues, respectively), 84.2% identity, and 93% similarity. Very similar location of the protein kinase domain (Pfam AC: PF00069) (71–356 and 65–349 respectively) and the same pattern of ATP binding and kinase active site additionally confirm the close relation of both proteins (Figure S1).

### 2.1. Silencing Vector Construction and Agro-Based Transformation of Immature Barley Embryos

The 262 bp long open reading frame (ORF) fragment of *HvGSK1.1* used for silencing had a relatively low nucleotide similarity with other barley GSK paralogs. The longest identical stretches were 17 nt, 16 nt, 14 nt, and 12 nt long with *HvGSK1.2*, *HvGSK2.1*, *HvGSK3.1*, and *HvGSK4.1*, respectively. In all cases, the identical stretches were much shorter than the 21 nt necessary for the efficient activation of cross-silencing.

Inoculation of 1975 scutella with *Agrobacterium* AGL1: pSoup and pBract207 with silencing cassettes and culture on hygromycin-containing media resulted in regeneration of 14 plants from independent explants. The presence of T-DNA with the silencing cassette was confirmed in 11 T_0_ plants. Transgene segregation in T_1_ seedlings indicated that T-DNA was integrated in a single locus in 10 lines and multiple loci in 1 line. Individual plants representing 10 lines with single transgene integration were analyzed for hemi-/homozygosity using qPCR and gDNA as a template. This approach allowed for fast selection of homozygous plants in the T_1_–T_2_ generations (Table 1).

### 2.2. Silencing of HvGSK1.1 Modifies Expression of GSK Paralogs in Plants Grown in Normal and Salt Stress Conditions

**Figure 1:**
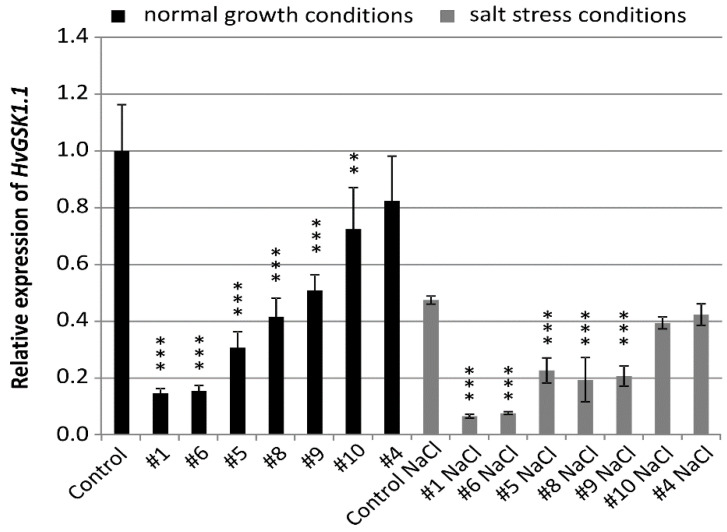
Relative expression of *HvGSK1.1* (HORVU3Hr1G034440.2) in leaves of T_4_ plants grown in normal (Hoagland medium) and in salt stress conditions (Hoagland medium supplemented with NaCl 200 mM). Relative expression of *HvGSK1.1* in leaves of the non-transgenic control plants grown in normal conditions was assumed as 1.0. The ADP-ribosylation factor (ARF) AJ508228 was used as the reference gene. Data represent mean values and standard deviation of at least three biological replicates with three technical repetitions. Indicated are significant differences between the control and the line grown in the same conditions: ** *p* < 0.01, *** *p* < 0.001.

The seeds of the T_4_ generation of selected homozygous plants were used for all further molecular and phenotype analysis. The relative transcript level of the targeted *HvGSK1.1* gene in plants grown in normal conditions was significantly lower at *p* < 0.001 in five lines and *p* < 0.01 in one line. Relative expression was 0.15 (line #1 and #6), 0.31 (line #5), 0.42 (line #8), 0.51 (line #9), 0.72 (line #10), and 0.82 (line #4) (Figure 1).

Expression of *HvGSK1.1* in salt stress was lower when compared to plants in normal growth conditions. Relative expression in the non-transgenic control in salt stress was 0.47 of expression in normal conditions. In 5 lines #1 (0.06), #6 (0.07), #5 (0.22), #8 (0.19), and line #9 (0.20)-relative expression in salt stress conditions was significantly (*p* < 0.001) lower (Figure 1).

**Figure 2:**
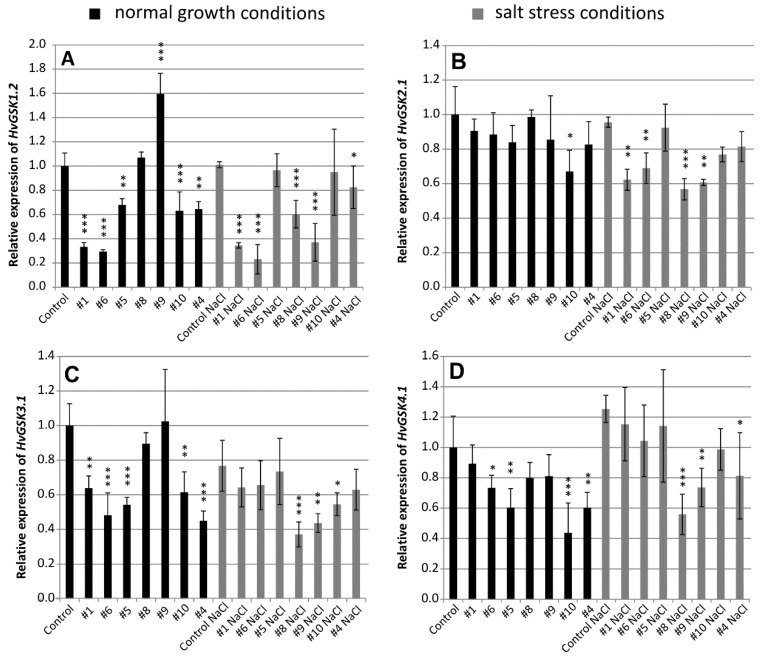
Relative expression of *HvGSK* paralogs *HvGSK1.2* (**A**), *HvGSK2.1* (**B**), *HvGSK3.1* (**C**), and *HvGSK4.1* (**D**) in leaves of T_4_ plants of transgenic lines grown in normal (Hoagland medium, black plots) and in salt stress conditions (Hoagland medium supplemented with NaCl 200 mM, grey plots). Relative expression of each of the tested paralogs in non-transgenic control plants grown in normal conditions was assumed as 1.0. The ADP-ribosylation factor (ARF) AJ508228 was used as the reference gene. Data represent mean values and standard deviation of at least three biological replicates with three technical repetitions. Indicated are significant differences between the control and the line grown in the same conditions: * *p* < 0.05, ** *p* < 0.01, *** *p* < 0.001.

Silencing of the target *HvGSK1.1* modified expression of four *GSK* paralogs *HvGSK1.2*, *HvGSK2.1*, *HvGSK3.1*, and *HvGSK4.1*. Relative expression of the paralogs varied depending on the line and the growth conditions. Relative expression of *HvGSK1.2* in the five lines: #1 (0.33), #6 (0.29), #5 (0.68), #10 (0.63), and #4 (0.64) grown in normal conditions was significantly (*p* < 0.001 and *p* < 0.01) lower than in the non-transgenic control (Figure 2A). Expression in salt stress conditions in the five lines #1 (0.35), #6 (0.23), #8 (0.60), #9 (0.37), and # 4 (0.81) was significantly (*p* < 0.001 and *p* < 0.05) lower than in the non-transgenic control (Figure 2A). Expression of *HvGSK2.1* was slightly downregulated in transgenic lines grown in normal conditions (Figure 2B). In salt stress, however, relative expression of the four lines #1 (0.62), #6 (0.69), #8 (0.57), and #9 (0.61) was significantly (*p* < 0.01 and *p* < 0.001) lower when compared to the non-transgenic control (Figure 2B).

Expression of *HvGSK3.1* was significantly downregulated in most of the transgenic lines grown in either of the tested conditions. In normal conditions, expression in lines #1 (0.64), #6 (0.48), #5 (0.54), #10 (0.62), and #4 (0.45) was significantly (*p* < 0.001 and *p* < 0.01) lower when compared with the non-transgenic control (Figure 2C). In salt stress conditions, expression was downregulated in three lines: #8 (0.37), #9 (0.44), and #10 (0.55) (Figure 2C). Expression of *HvGSK4.1* in normal conditions was significantly (*p* < 0.05, *p* < 0.01 and *p* < 0.001) downregulated in four transgenic lines #6 (0.71), #5 (0.6), #10 (0.44), and #4 (0.6) (Figure 2D). In salt stress conditions, the three lines #8 (0.56), #9 (0.74), and #4 (0.81) were significantly downregulated (Figure 2D). Northern blot hybridization confirmed the presence of the siRNA molecules complementary to the ORF fragment present in the silencing cassette. The small RNAs were detected in transgenic plants of selected lines in the T_2_ generation. They were not detectable in the non-transgenic control (Figure S2).

### 2.3. Leaf Inclination Biotest

**Figure 3:**
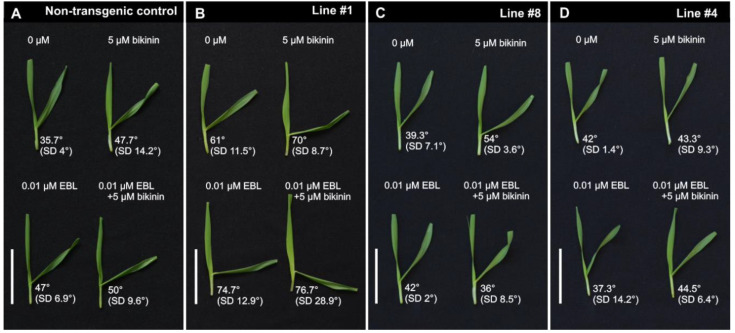
Representative pictures of leaf fragments treated with 24-epibrassinolide (EBL) 0.01 µM, bikinin 5 µM, and EBL (0.01 µM) and bikinin (5 µM) applied together. (**A**)–the non-transgenic control plants, (**B**)–line #1 with strong silencing of *HvGSK1.1*, (**C**)–line #8 with intermediate silencing of *HvGSK1.1*, and (**D**)–line #4 with alleviated silencing of *HvGSK1.1*. Indicated are medium inclination angles and standard deviation (SD) of at least five plants. Bar represents 5 cm.

The leaf inclination biotest was developed as a semi-quantitative assay of BRs [47] and BR-dependent signaling [48]. The test was adapted for barley and showed that higher concentrations of EBL and of bikinin were associated with bigger lamina joint angles (Figure S3A,B). The inclination angle in the control was 36.8° (SD 3°). After treatment with EBL (0.01 µM), it was 40.7° (SD 4.9°), and, after bikinin (5 µM) treatment, it was 37° (SD 7.6°). After simultaneous treatment with both compounds EBL (0.01 µM) and bikinin (5 µM), the angle was 59.2° (SD 14°) (Figure S3C). The concentrations 0.01 µM EBL and 5µM bikinin were used to test the response of the non-transgenic control (Figure 3A) and transgenic lines #1, #8, and #4, which represented strong, medium, and weak silencing of *HvGSK1.1* (Figure 3B–D, Figure S4). The inclination angles in water-incubated line #1 was 61°. After 0.01 µM EBL, the angle was 74.7°. After 5 µM bikinin, the angle was 70° and the combined treatment with both compounds increased the angle to 76.7° (Figure 3B, Figure S4). The inclination angle in water-incubated line #8 was 39.3°. After 0.01 µM EBL, the angle was 42°. After 5 µM bikinin, the angle was 54° and the combined treatment with both compounds gave a 36° angle (Figure 3C, Figure S4). The inclination angles in the water-incubated line #4 was 42°. After 0.01 µM EBL, the angle was 37.3°. After 5 µM bikinin, the angle was 43.3° and combined treatment with both compounds elevated the angle to 44.5° (Figure 3D, Figure S4). The inclination angles of line #1 in the reaction for either a single or both compounds were greater than in the non-transgenic control. The reaction of lines #4 and #8 with the weaker silencing of *HvGSK1.1* was similar to the non-transgenic control.

### 2.4. Silencing of HvGSK1.1 Increases Biomass of Plants Grown in Normal and Salt Stress Conditions and Kernel Weight

**Figure 4:**
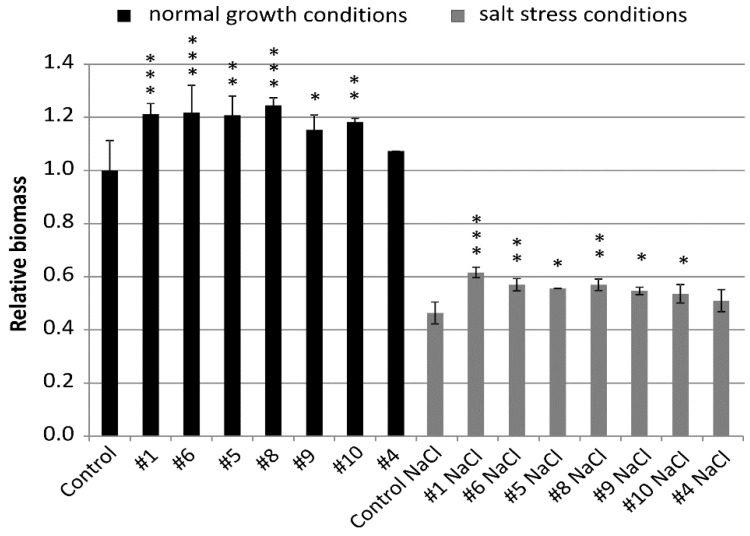
Relative biomass of the non-transgenic control and the transgenic plants of T_4_ generation grown in normal (Hoagland medium) and in salt stress conditions (Hoagland medium supplemented with NaCl 200 mM). Data represent mean values and standard deviation of at least six plants grown in normal and salt stress conditions. Indicated are significant differences between the control and the line grown in the same conditions: * *p* < 0.05, ** *p* < 0.01, *** *p* < 0.001.

**Figure 5:**
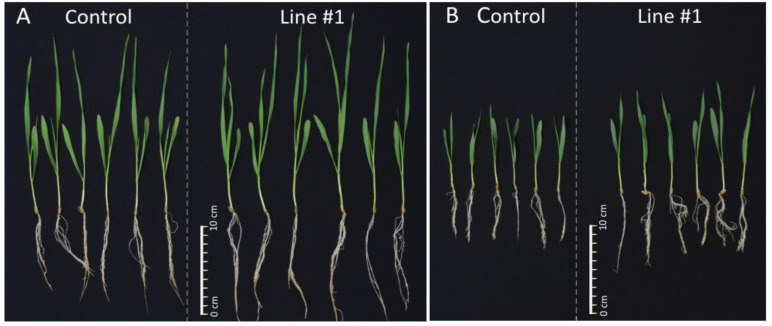
Representative pictures of 14-days-old seedlings of the non-transgenic control and the line #1. (**A**) 14-days-old seedlings of the non-transgenic control and the line #1 grown in normal conditions (Hoagland medium). (**B**) 14-days-old seedlings of the non-transgenic control and the line #1 grown in salt stress conditions (Hoagland medium supplemented with NaCl 200 mM).

Relative biomass of all lines was greater than biomass of the control from the same conditions and, for six lines, the values were significantly different (Figure 4 and Figure 5). Lines #1 (1.21), #6 (1.22), and #8 (1.25) exceeded the biomass of the control by 21% to 25% with high statistical significance *p* < 0.001. Biomass of lines #5 (1.21) and #10 (1.18) was greater than the control with *p* < 0.01 and, for line #9 (1.15), the significance was *p* < 0.05 (Figure 4).

Biomass of the plants grown in salt stress was smaller and, for the non-transgenic control, it was 0.46 of the biomass in normal conditions. However, relative biomass of all lines #1 (0.62), #6 (0.57), #5 (0.55), #8 (0.57), #9 (0.55), #10 (0.54), and #4 (0.51) was greater than the control from the same conditions. The values shown by three lines #1, #6, and #8 exceeded the control by 24% to 35% with significance at *p* < 0.01 and *p* < 0.001. The values of another lines #5, #9, and #10 exceeded the control by 17% to 20% with a significance at *p* < 0.05 (Figure 4).

**Figure 6:**
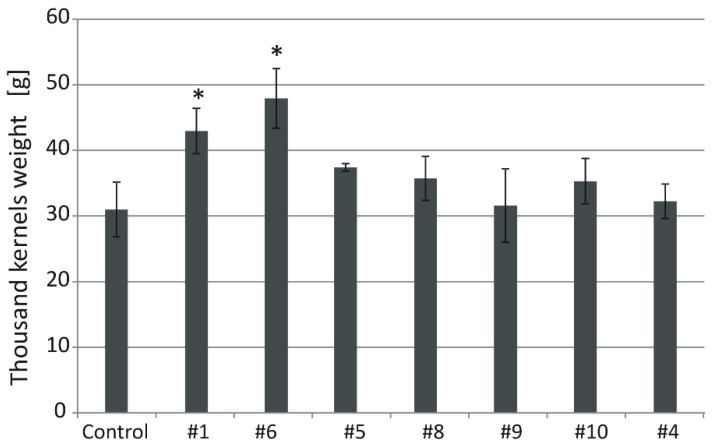
Thousand kernels weight (TKW) in the non-transgenic control and the transgenic plants grown in soil in normal conditions. Data represent mean values and standard deviation. Indicated are significant differences (* *p* < 0.05) between the control and the tested lines.

**Figure 7:**
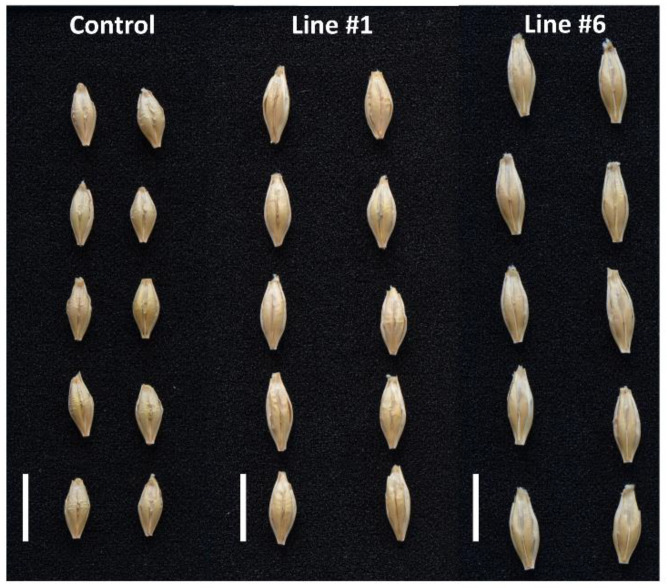
Representative pictures of kernels of the non-transgenic control and the transgenic lines #1 and #6.

Thousand kernel weight (TKW) of the control plants grown in normal conditions was 31 g (Figure 6). In transgenic lines, the values were: #1 (43), #6 (47.9), #5 (37.4), #8 (35.8), #9 (31.6), #10 (35.3), and #4 (32). The TKW values of #1 and #6 were significantly (*p* < 0.05) higher than those of the control (Figure 6 and Figure 7).

### 2.5. Silencing of HvGSK1.1 Correlates with Expression of GSK Paralogs, Plant Biomass, and Thousand Kernel Weight

**Figure 8:**
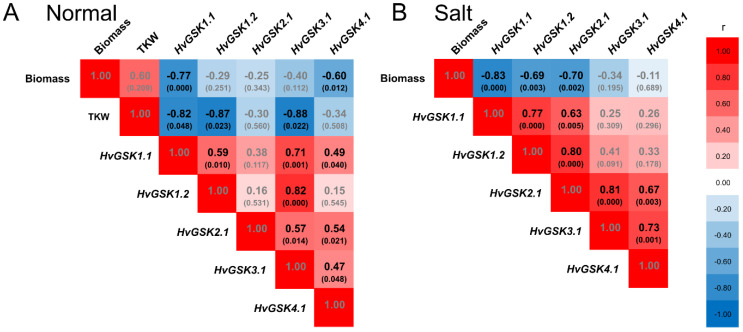
Heat map of the Pearson correlations between expression of *HvGSK1.1*, expression of *HvGSK* paralogs, and values of thousand kernel weight (TKW) measured in the non-transgenic control and the transgenic lines grown in normal conditions (**A**) and in salt stress conditions (Hoagland with NaCl 200 mM) (**B**). Indicated are *r* values and *p* values in brackets. Black font indicates significant correlations for *p* < 0.05. Red blocks indicate a positive correlation and blue blocks indicate a negative correlation.

Expression of *HvGSK1.1* in plants grown in normal conditions correlated with expression of three paralogs: *HvGSK1.2* (*r* = 0.59), *HvGSK3.1* (*r* = 0.71), and *HvGSK4.1* (*r* = 0.49). In salt stress, it correlated with *HvGSK1.2* (*r* = 0.77) and *HvGSK2.1* (*r* = 0.63) (Figure 8). Expression of the four paralogs showed a net of mutual positive correlations, which were different in normal and salt stress conditions (Figure S5). The most clear-cut characteristics of all transgenic lines had greater biomass of the seedlings grown in normal and in salt stress conditions (Figure 4 and Figure 5). Statistical analysis revealed a strong negative correlation between the biomass and the expression of *HvGSK1.1* in plants grown in normal conditions (*r* = −0.77) and in salt stress (*r* = −0.83) (Figure 8). The biomass was also negatively correlated with the expression of *HvGSK4.1* (*r* = −0.60) in normal conditions and with expression of *HvGSK1.2* (*r* = −0.69) and *HvGSK2.1* (*r* = −0.70) in salt stress conditions (Figure 8). This was consistent with the positive mutual correlations between the paralogs’ expressions (Figure S5). Kernel weight, checked in plants in normal conditions and shown as thousand kernel weight (TKW), showed a strong negative correlation with *HvGSK1.1* (*r* = −0.82), *HvGSK1.2* (*r* = −0.87), and *HvGSK3.1* (*r* = −0.88) (Figure 8, Figure S5).

## 3. Discussion

The *HvGSK1.1* gene was the first barley GSK-encoding gene identified by us. It was selected for RNAi-based silencing because of its high sequence similarity to rice *OsGSK1*, whose knockout mutation conferred elevated tolerance to environmental stresses [46]. The ORF fragment of *HvGSK1.1* selected for construction of the silencing cassette had a low-level similarity to the cDNA sequences of the *GSK* paralogs. The longest identical stretches between the silencing cassette and the paralogs, from 12 to 17 nt, excluded efficient activation of cross-silencing. The Gateway strategy proved to be very useful for constructing pBract207-based silencing cassette using the fragment of the target gene. As a result of *Agro*-based transformation, the T-DNA was integrated as a single locus in 10 plants. Only a single plant contained the T-DNA integrated into more than one locus. The 1:10 rate of multiple vs. single integration (Table 1) is lower when compared with the average 46% rate of multiple integrations reported by Bartlett et al. [49] in a large group of transgenic barley plants. The relatively high rate of lines with single integration is an advantage considering the expected higher stability of the transgene expression in the subsequent generations. As found by Zalewski et al. [50], this is particularly important when the targeted gene is developmentally regulated and it is involved in hormone regulation. Results of the Northern blot (Figure S2) confirmed that short (22–25 nt) RNA molecules, complementary to the silencing fragment of the target gene, were present in the selected transgenic lines and they were undetectable in the non-transgenic control plants. The presence of this class of short RNA molecules, complementary to the silencing cassette, confirms the synthesis of the hpRNA transcript and its processing into siRNAs. The analysis of target gene expression in T_2_ plants showed that the silencing was strong and ranged from 0.1 to 0.6 in all transgenic lines Figure S6). Seed multiplication necessary for all experiments required two additional generations of self-pollinated plants. In T_4_ generation plants, the target gene silencing remained in seven lines. These lines represented a gradient of *HvGSK1.1* silencing ranging from 0.15 to 0.82 (Figure 1) and a set of molecular and phenotype changes.

The *HvGSK1.1* silencing was associated with modified expression of the GSK-encoding paralogs. The original silencing of the target gene associated with this modified expression of the paralogs showed a net of mutually correlated changes present in both growth conditions (Figure 8, Figure S5). A low level of nucleotide similarity between the silencing cassette and the paralogs excluded cross silencing as the process responsible for the co-regulation. We propose that such an extensive net of correlations, which was further strengthened by the salt stress, implies an innate mechanism of *GSK* gene regulation. Although, at this point, there are no experimental data to explain it, we think that the pattern of transcription factor binding sites (TFBS) found in promoter regions of the genes indicates such a mechanism. The promoter regions analysis of several barley *GSKs* showed a presence in multiple binding sites of the two BR-dependent transcription factors BZR1 and BZR2 (Table S1). The two BR-dependent TFs are the targets of the GSK-dependent phosphorylation and inactivation [2,51]. This combination of positive transcriptional regulation and a negative post-transcriptional regulation indicates a possible feedback mechanism coordinating expression of the *GSK* genes. We think that the net of mutual co-regulation could be the result of such a mechanism. This notion is further confirmed by (i) the presence of the BZR1/BZR2 binding sites in promoter regions of *OsSK11* and *OsGSK1*, the two rice orthologs of *HvGSK1.1* (Table S1), and (ii) a large number of common TFBS present in the promoter regions of the selected barley, rice, and *Arabidopsis GSK* genes (Figure S7, Table S2). These observations are in agreement with extensive and strong co-regulation of the *GSK* genes found in generative organs in barley [45]. They are also compatible with the results of chromatin immuno-precipitation showing that several GSK-encoding (*AtSK12*, *AtSK13*, *AtSK21*, and *AtSK41*) and BZR2-encoding genes are among the specific targets of the BZR1 transcription factor [9]. The presence of BR-specific TFBS in at least four GSK-encoding genes in *Arabidopsis* indicates a complex network of co-regulation of the *GSK* paralogs. Our results showing coordinated regulation of the *GSKs* in barley provide novel data on the mechanisms of BR homeostasis in plants. A similar data, concerning RNAi-based silencing of *TaCKX1* and associated with this co-regulation of *CKX* paralogs, were reported by Jablonski et al. [52]. The authors found that diverse levels of target gene silencing were associated with different models of co-expression with other *TaCKX* genes and parameters of yield-related traits in wheat.

Bikinin was identified as a GSK3 inhibiting molecule. In *Arabidopsis*, 7 out of the 10 ASKs were explicitly inhibited by this compound when enhancing BR-dependent signaling [24,53]. We hypothesized that *HvGSK1.1* silencing would enhance the BR-dependent response and mimic the effect of the bikinin treatment. To verify this, we adapted the procedure of leaf inclination for barley based on the original test developed for rice [47] and confirmed by the findings of Yamamuro et al. [54] that leaf inclination angle and internode elongation were specifically regulated by BRs. The test adapted for barley showed that higher concentrations of EBL were associated with greater angles of the lamina joints. Taking into account the function, bikinin could be considered as the physiological equivalent of the silencing of the GSK-encoding genes. This effect was clearly visible in seedling fragments treated with the tested concentrations of EBL and bikinin (Figure S3A,B). The simultaneous treatment with EBL and bikinin showed a stronger effect than the impact of either of the compounds applied separately (Figure S3C). The changes observed after EBL and bikinin treatment in leaves of the non-transgenic control and the three lines #1, #8, and #4 indicated that silencing of *HvGSK1.1* enhanced the reaction for exogenous BL in a similar way as the bikinin treatment. Based on these results, we concluded that silencing of *HvGSK1.1* enhanced the BR-dependent signaling in the plants. The final effect was the result of the experimental silencing of *HvGSK1.1* and correlated with this down-regulation of other *GSK* paralogs. It agrees with findings that bikinin inhibiting the GSKs acts as an activator of BR signaling and mimics the exogenous application of BRs [24]. We propose that both the original silencing of *HvGSK1.1* correlated with this down-regulation of *GSK* paralogs enhanced the BR-dependent signaling and, in that way, promoted certain traits regulated by BRs.

The most notable feature of all transgenic lines found in normal and in salt stress conditions was their greater biomass compared with the non-transgenic control. The trait showed a strong negative correlation with the expression of the *HvGSK1.1* and the *GSK* paralogs. The observation agrees with the reported greater weight of *Arabidopsis* plants grown in normal and in salt stress growth conditions after EBL treatment [28]. Similar results of growth promotion and alleviation of salt stress have been presented in several other studies [36,55,56], and this may involve auxin-dependent regulation [57]. Another trait associated with the experimental silencing of *HvGSK1.1* and correlated with this down-regulation of the *GSKs* was increased weight of the kernels. The trait, shown as TKW, was significantly bigger in lines #1 and #6 with the strongest *HvGSK1.1* silencing (Figure 6 and Figure 7). It showed a strong negative correlation with *HvGSK1.1* (*r* = −0.82), *HvGSK1.2* (*r* = −0.87), and *HvGSK3.1* (*r* = −0.88) (Figure 8, Figure S5). This set of results fully agrees with articles showing that productivity-related traits are stimulated by BRs and BR-dependent signaling. These include significantly increased seed number in a BR-enhanced mutant [58], smaller seeds in BR-deficient mutants, and confirmation that seed size, mass, and shape in *Arabidopsis* were regulated by BRs [59]. It agrees with a finding by Che et al. [21] that mutation of *grain lenths2* loci in rice activated BR-dependent responses and, as a consequence, increased the weight of rice grains by 27%. The former authors concluded that modulating specific brassinosteroid responses could improve plant productivity. Wu et al. reported compatible results of significantly increased grain yield and weight in rice with enhanced production of BRs [60]. Enhanced barley growth observed after silencing of *HvGSK1.1* is also compatible with results of Wan et al. [61]. They reported that *PeGSK1* acts as a negative regulator of cell growth in Moso bamboo, which is one of the fastest growing monocots.

The characteristics of barley lines with silenced expression of *HvGSK1.1* are compatible with the expected phenotypes of plants with enhanced BR signaling. The results show that manipulation of the GSK3-encoding genes provides data to explore their biological functions and, at the same time, indicate it as a feasible strategy to generate plants with improved agricultural traits.

## 4. Materials and Methods

### 4.1. Bioinformatics of HvGSK1.1 and Rice Orthologs

The pairwise alignment between protein sequences of HvGSK1.1 (HORVU3Hr1G034440) and OsGSK1 (Os01g10840) was performed in the needle program from the EMBOSS package [PMID: 30976793]. The identification of protein domains in HvGSK1.1 and OsGSK1 was performed in InterProScan [PMID: 30398656]. The prediction of transcription factors’ binding sites (TFBS) in the promoter regions (up to 4000 nucleotides) of GSK-encoding genes in barley, rice, and Arabidopsis was carried out by the Match™ program [PMID: 12824369] and the TRANSFAC^®^ database (Release 2020.1) [PMID: 18436575]. Match™ was set to use only high-quality TFBS matrices to minimize false positives. The predicted TFBS were also confirmed by the CiiiDER tool (with default parameters) [PMID: 31483836] and non-redundant transcription factor plant models from the JASPAR database (Release JASPAR2020) [62].

### 4.2. Plant Material and Growth in Normal and Salt Stress Conditions

The barley (*Hordeum vulgare* L.) cultivar Golden Promise was used as a source of plant material in all experiments. Barley kernels, after 48 h of imbibition, were planted in pots (14-cm diameter) filled with peat substrate mixed with sand in a 10:3 *v*/*v* ratio. The seedlings were cultivated in a growth chamber with a 16-h photoperiod at 21 °C in the day and 18 °C in the night. The relative humidity was in the range of 60–80%, and the light intensity was 130–230 μmol photons m^−2^ s^−1^. Plants were irrigated twice a week and fertilized once a week with multi-component soil fertilizer Florovit (http://florovit.pl/), according to the manufacturer’s instructions. All experiments of transcript quantification and phenotype characterization were done using homogenous seed material of T_4_ generation obtained after self-pollination of selected T_2_ homozygotes grown in the same conditions as the donor plants. Germinating seeds of the tested lines and the non-transgenic control, after 48 h imbibition in Hoagland solution [63] or Hoagland supplemented with 25 mM NaCl, were placed on filter paper, which was tightly rolled, saturated with Hoagland solution, or Hoagland supplemented with 200 mM NaCl and placed in a growth chamber in the same conditions as the donor plants. Cultivation in Hoagland or Hoagland supplemented with NaCl is referred as growth in normal or in salt stress conditions, respectively. The 14-day old seedlings grown in normal and in salt stress conditions were used for biomass quantification and for leaf collection, RNA extraction, and transcript quantification. The plants cultivated in soil in the same conditions as the donor plants were used for kernel characterization.

### 4.3. Construction of Silencing Vector and Agrobacterium-Mediated Transformation of Barley

**Figure 9:**
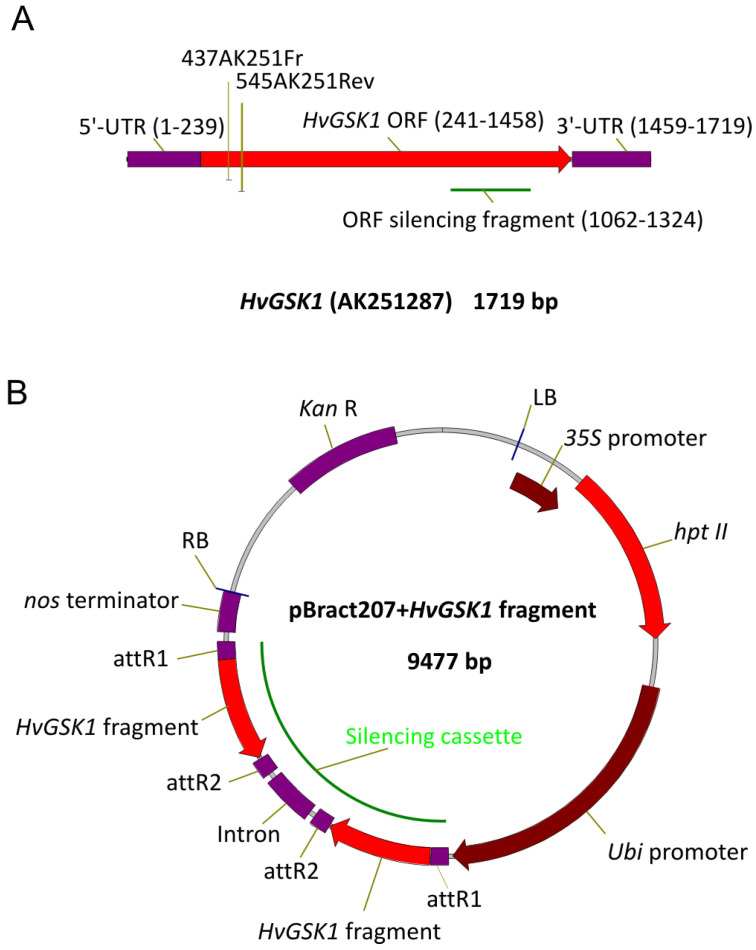
Schematic representation of cDNA of *HvGSK1.1* gene (**A**) and pBract207 vector with *HvGSK1.1* fragment built into the silencing cassette (**B**). Open reading frame (ORF) fragment used for construction of the silencing cassette in the pBract207 vector is indicated (**A**). The most important functional regions of the pBRACT207 vector are indicated (**B**). The open reading frame (ORF) fragment of *HvGSK1.1* used for gene silencing was amplified with AmpliTaq Gold DNA Polymerase (ThermoFisher Scientific) with the primers ORFgskFR/ORFgskRe (Table 2), and the vector pCR2.1TOPO with cloned *HvGSK1.1* cDNA as a template. Gel purified amplicon was blunt-end cloned into the pIPKTA38 [64]. The obtained plasmid pIPK:ORF was used as the entry clone for Gateway recombination (Invitrogen) with destination vector pBRACT207 (John Innes Center, Norwich, UK, www.bract.org).

A barley clone representing a cDNA of *HvGSK1.1* transcript (HORVU3Hr1G034440.2) was amplified using AmpliTaq Gold DNA Polymerase (Thermo-Fisher Scientific, Waltham, MA, USA), the primers 1FRGSK1 and 4RevGSK1 (Table 2), and cDNA of barley cv. Golden Promise as a template. The amplified fragment was cloned to pCR2.1TOPO (Invitrogen by Thermo Fisher Scientific, Waltham, MA, USA), sequenced (Genomed, Warsaw, Poland), and assigned as *HvGSK1.1* (Figure 9A).

Gateway recombination was performed in 5 μL of a reaction mixture containing entry and destination molecules in a 2:1 molar ratio, total DNA 75–100 ng, and 1 μL of LR-Clonase 5x enzyme mix (Life Technologies by Thermo Fisher Scientific, Waltham, MA, USA). The 1 μL of 5x water-diluted reaction mix was electroporated (2.5 kV, 25 μF, 200 Ω, Gene Pulser II, Bio-Rad, Hercules, CA, USA) into 50 μL of electro-competent *E. coli* OmniMAX2-T1 strain (Life Technologies) and cultured on LB with kanamycin (50 mg/L). Silencing cassettes were confirmed by sequencing using primers anchored in the *Ubi* promoter, intron, and *nos* terminator of pBRACT207 (Figure 9B). The plasmid with an expected sequence and structure of the silencing cassette was electroporated into *Agrobacterium tumefaciens* AGL1 strain containing a pSoup helper plasmid [65]. Immature embryos, isolated from barley cv. Golden Promise, were inoculated with *Agrobacterium* AGL1 strain containing the pBRACT207-based construct and were in vitro cultured, according to the modified procedure [49,66]. Regenerated plants were transferred to soil and grown in conditions identical to barley donor plants. In vitro regenerated, non-transgenic plants were used as controls for all phenotypic observations and all molecular analyses.

### 4.4. Nucleic Acid Isolation, Transcript Quantification Leaf Inclination Test

Genomic DNA (gDNA) was isolated from plant samples, according to the modified CTAB procedure [67]. Total RNA was extracted from leaves of 14-day-old plants grown in normal conditions (Hoagland solution) and in salt stress conditions (Hoagland with 200 mM NaCl) using RNA 3-zone reagent solution (Novazym, Poznan, Poland). RNA concentration and A260/280 ratio (always higher than 1.8) were measured using a NanoDrop spectrophotometer (NanoDrop Technologies, Wilmington, DE, USA). The RNA quality was further determined in agarose gel. Isolated total RNA was treated with 2 U of DNase RNase-free (Roche Diagnostics, Mannheim, Germany) and 2 U of Protector RNase inhibitor followed by DNase inactivation, according to the manufacturer’s protocol. Genomic DNA impurities were checked by polymerase chain reaction (PCR) with primers qAct1 and qAct2a (Table 2) specific to the β-actin encoding gene, and 100 ng of DNase treated RNA as a template. Complete removal of gDNA was confirmed by a lack of detectable amplicon after 36 cycles of amplification. Two micrograms of RNA (non-degraded, DNase-treated with undetectable gDNA impurities) were used as a template for the reverse transcription reaction with oligo d(T) primers and RevertAid First Strand cDNA Synthesis Kit (ThermoFisher Scientific, USA). Obtained cDNA was diluted five-fold and used directly as a template for quantitative PCR (qPCR). The standard qPCR reaction mix was composed of 2.2 µL of 5x HOT FIREPol EvaGreen qPCR Mix Plus (ROX) (Solis Biodyne, Tartu, Estonia), 0.25 µL of primer F (10 µM), 0.25 µL of primer R (10 µM), 1 µL of cDNA, and water to 11 µL. The reaction was performed in a Rotor-Gene 6000 model 5-plex thermocycler (Corbett Life Science, Chadstone, Australia). The efficiencies of amplification for all primer pairs were in the range of 0.85 to 1.00 and R^2^ 0.99875–1.00000. The specificity of amplification was verified by melting curve analysis. Template concentrations ranging from 10^4^ to 10^8^ copies of analyzed amplicon and 10^4^–10^8^ copies of the reference gene ADP-ribosylation factor (ADP RF) AJ508228 per reaction were used as the standards for qPCR. Primers for transcript quantification were designed based on the sequences deposited in NCBI and Ensemble Plants (Table 2). Threshold line, Ct values, standard curves, and relative quantifications were determined using the proprietary Rotor-Gene 6000 software v 1.7 (Corbett Life Science, Chadstone, Australia). The transcripts’ quantification data represent a medium of at least three biological replicates with three technical repetitions each. The whole procedure of RNA isolation, reverse transcription, qPCR conditions, and data analysis met the Minimum Information for Publication of Quantitative Real-Time PCR Experiments (MIQE) criteria outlined by Bustin et al. [68].

Identification of transgene hemi-zygotes and homo-zygotes in the T_1_ plants was made using a qPCR reaction with primers designed for *hptII* gene, primers designed for β-actin-encoding gene, which served as a reference, and using barley genomic DNA (gDNA) isolated from the tested T_1_ plants as a template. The reaction was carried out in a 11 μL mixture containing 2.2 µL of HOT EvaGreen qPCR Mix Plus and 0.25 µL (10 µM) of each primer for *hptII* (pBr_Hyg_F2/pBr_Hyg_R2) or β-actin (qAct1/qAct2a) amplification (Table 2), 7.3 µL of H_2_O, and 1 µL containing 50 ng of gDNA as a template. Each reaction was run in triplicate. The homozygotes were discriminated from hemizygotes based on the values of *hptII* to actin ratio calculated for at least six plants representing the T_1_ progeny of each transgenic line.

The fraction of small RNA was obtained from total RNA after precipitating high molecular weight RNA using 10% PEG (MW 8000) and 1 M NaCl and collecting small RNAs using a standard isopropanol method [69]. 10 µg of samples of fractionated RNA were separated by PAGE (15% acrylamide:bis acrylamide 19:1 *v*/*v*) in TBE denaturing buffer, transferred to nylon membrane positively charged using a semidry electroblotter (Bio-Rad, Hercules, CA, USA), and immobilized by UV cross-linking. The ORF-derived silencing fragment was used as a template for probe labeling using the PCR DIG Probe synthesis kit. Hybridization and chemiluminescent detection were done using a DIG detection kit. Labeling, hybridization, and detection were done, according to the manufacturer’s protocols (Roche Diagnostics, Mannheim, Germany).

The leaf inclination biotest was adapted from the protocol of Wada et al. [47]. The excised upper part of the seedlings with the first leaf developed and the second leaf emerging were incubated for 72 h in the tested concentrations of 24-epibrassinolide (EBL), (OlChemIm, Olomouc, Czech Republic) and bikinin (Sigma-Aldrich, Steinheim, Germany, a kind gift from prof. R. Malinowski). After incubation, the leaf fragments were used to assess leaf inclination angles. At least five plants of lines #1, #8, and #4 representing strong, medium, and weak *HvGSK1.1* silencing, respectively, were used. At least six seedlings representing transgenic lines and the non-transgenic control were used to assess the biomass of the plants grown in normal conditions and salt stress conditions. The kernels collected from at least three plants of transgenic lines and non-transgenic control grown in soil in the same conditions as donor plants were used to calculate the thousand kernel weight (TKW).

### 4.5. Statistical Analyses

The Pearson correlation coefficient was calculated (Statistica version 13, Tibco, Palo Alto, CA, USA) between the values of the relative expressions of the analyzed genes, the biomass of the seedlings, and the values of thousand kernel weight (TKW). The correlation coefficient was significant at *p* < 0.05. The values between 0 and ǀ0.3ǀ were defined as a weak (low) correlation, the values between ǀ0.3ǀ and ǀ0.7ǀ were defined as a medium (moderate), and the values between ǀ0.7ǀ and ǀ1ǀ were defined as a strong (high) correlation. Data are presented as the mean value with the standard deviation. Quantitative RT-PCR data represented the medium of at least three independent biological replications with each done using the three technical repetitions. Standard deviation represents the variation between the biological repetitions. The statistical significance of the differences was analyzed using the ANOVA test, which was followed by the LSD post-hoc test (STATISTICA 10, StatSoft). The differences were considered statistically significant at *p* < 0.05.

## Figures and Tables

**Table 1:** Agrobacterium-mediated transformation and in vitro culture of immature barley embryos.

Vector	Cultured Immature Embryos	Regenerated Plants	Plants with Confirmed t-DNA	Plants with Single T-DNA Integration Locus	Plants with Multiple T-DNA Integration Loci
T-DNA of silencing vector	1975	14	11	10	1
Control of in vitro culture and plant regeneration	107	Good plant regeneration on hygromycin-free medium, 20–25 shoots from one immature embryo.			
Control of in vitro culture and hygromycin selection	107	No shoots were regenerated on hygromycin-containing medium.			

**Table 2:** List of primers and reaction conditions used in this study.

Gene Name *Ensemble Plants* ID NCBI ID	Primers		Amplicon Length and Reaction Conditions
	Symbol	Sequence	
*HvGSK1.1* HORVU3Hr1G034440.2 AK251287	1FRGSK1	GGTGGTGGTAGAGTAGGAGTA	1719 bp, 95 °C 5 min, 35 cycles (95 °C 30 s, 60 °C 30 s, 72 °C 30 s) 72 °C 5 min.
	4RevGSK1	TTACAGATCAGCTATGGCAAT	
	ORFgskFR	TGGTGAAAGTGGTGTGGACC	262 bp, 95 °C 5 min, 35 cycles (95 °C 30 s, 56 °C 30 s, 72 °C 30 s) 72 °C 5 min.
	ORFgskRe	GGTCCCGAAGCTCATCAAAG	
	437AK251_Fr	AGGGAACAGAGACTGGTCACAT	108 bp, 95 °C 15 min, 45 cycles (95 °C 25 s, 60 °C 25 s, 72 °C 25 s) 72 °C 1 min.
	545AK251_Rev	AATGAACCTTGACCAACAATCC	
*HvGSK1.2* HORVU5Hr1G117030.1 AK368391	AK368_FR	TCTGGGCACACCTACAAGGG	139 bp, 95 °C 15 min, 45 cycles (95 °C 25 s, 60 °C 25 s, 72 °C 25 s) 72 °C 1 min.
	AK368_Rev	TGGAGACCAGGTCCACTGCT	
*HvGSK2.1* HORVU3Hr1G026020.1 AK364823	403AL364_FR	AGTGCTTGGAGACTGGAGAGAC	122 bp, 95 °C 15 min, 45 cycles (95 °C 25 s, 60 °C 25 s, 72 °C 25 s) 72 °C 1 min.
	524AK364_Re	GTGCTTCAGAGAGACGACATTG	
*HvGSK3.1* HORVU1Hr1G048580.7AK362547	1270AK362_Fr	AAAGTGGCGTTGATCAGTTGG	123 bp, 95 °C 15 min, 45 cycles (95 °C 25 s, 60 °C 25 s, 72 °C 25 s) 72 °C 1 min.
	1393AK362_Rev	CAGGGATGAGCTTTTATCTGAGG	
*HvGSK4.1* HORVU5Hr1G119790.18AK358344	AK358_FR	GCGAGAAGGCAGAACCTGTT	133 bp, 95 °C 15 min, 45 cycles (95 °C 25 s, 60 °C 25 s, 72 °C 25 s) 72 °C 1 min.
	AK358_Rev	TGTCACCCACCCACACAAAG	
ARF AJ508228	Ref2_FR	GCTCTCCAACAACATTGCCAAC	162 bp, 95 °C 15 min, 45 cycles (95 °C 25 s, 60 °C 25 s, 72 °C 25 s) 72 °C 1 min.
	Ref2_Rev	GCTTCTGCCTGTCACATACGC	
ACTB (actin) AY145451	qAct1	AGCAACTGGGATGACATGGAG	172 bp, 95 °C 15 min, 45 cycles (95 °C 25 s, 60 °C 25 s, 72 °C 25 s) 72 °C 1 min.
	qAct2a	CGTACATGGCAGGAACATTG	
*hptII*	pBr_Hyg_F2	GACGGCAATTTCGATGATG	205 bp, 95 °C 15 min, 45 cycles (95 °C 25 s, 60 °C 25 s, 72 °C 25 s) 72 °C 1 min.
	pBr_Hyg_R2	CCGGTCGGCATCTACTCTAT	

## Supplementary Materials

Supplementary Materials can be found at https://www.mdpi.com/1422-0067/21/18/6616/s1. Figure S1: Protein domain organization of GSK orthologs encoded by: *HvGSK1.1* (HORVU3Hr1G034440.2) and *OsGSK1* (Os01g10840). The amino acid defining features of the kinase domains i.e. protein kinase domain (Pfam AC: PF00069), ATP binding site and kinase active site are indicated. Figure S2: Northern blot detection of low molecular weight RNA in barley leaves in T_2_ generation using DIG labeled probe complementary to *HvGSK1.1* ORF fragment in the silencing cassette. Indicated are U6 and 25 nt marker (lane 1), RNA from non-transgenic control plants (lanes 5, 9), RNA from lines #1, #2, #4 and #6. Lanes 2, 3, 4 and 6 were not loaded with the RNA to avoid cross contamination of neighboring lanes with the RNA samples. Figure S3: Representative pictures of leaf fragments from wild type barley cv. Golden Promise treated with indicated concentrations of 24-epibrassinolide (EBL) (A), bikinin (B). Pictures of leaf fragments treated with threshold concentrations of 0.01μM EBL and 5μM bikinin applied separately and together (C). Indicated are medium inclination angles and standard deviation (SD) of at least five plants. Bar represents 5 cm. Figure S4: Leaf inclination angles of nontransgenic control and transgenic lines #1, #8 and #4, which represented strong, medium and weak silencing of *HvGSK1.1*. Leaf fragments were treated with 24-epibrassinolide (EBL) 0.01 μM, bikinin 5 μM and EBL (0.01 μM) and bikinin (5 μM) applied together. Data represent medium inclination angles and standard deviation (SD) of at least five plants. Figure S5: Graphical representation of Pearson correlation net between expression of *HvGSK1.1* and *HvGSK* paralogs, and the values of thousand kernel weight (TKW) measured in the non-transgenic control and the transgenic lines grown in normal conditions (A) and in salt stress conditions (Hoagland with NaCl 200 mM) (B). Positive and negative correlations are indicated with red and blue respectively. Bar width denotes the r values according to the scale. Figure S6: Relative expression of *HvGSK1.1* in leaves of T_2_ plants grown in normal (Hoagland medium) and in salt stress conditions (Hoagland medium supplemented with NaCl 200mM). Relative expression of *HvGSK1.1* in leaves of non-transgenic control plants grown in normal conditions was assumed as 1.0. Figure S7: The Upset plot shows the number of shared transcription factor binding sites (TFBS) in the promoter regions of GSK-encoding genes from barley (*HvGSK1.1*, *HvGSK1.2*, *HvGSK1.3*, *HvGSK2.1*, *HvGSK3.1*, *HvGSK4.1*) and rice (*OsGSK1* and *OsGSK1.1*). Promoter region of *HvGSK1.1* showed the highest number of shared TFBS with (n = 280) and *OsGSK1* (n = 249). Table S1: The number of binding sites for brassinosteroid-dependent transcription factors BZR1 and BZR2 identified in the GSK-encoding genes based on TRANSFAC. Table S2: The list of all transcription factors binding sites (TFBS) in promoter regions of GSK-encoding genes in barley, rice and *Arabidopsis*.
