# Supplementary material for: Silencing of HvGSK1.1—A GSK3/SHAGGY-Like Kinase–Enhances Barley (Hordeum vulgare L.) Growth in Normal and in Salt Stress Conditions
Source: Int J Mol Sci. 2020 Sep 10;21(18):6616. doi: 10.3390/ijms21186616 (PMC7554974; doi:10.3390/ijms21186616)
Supplement: Supplementary file 1 [file ijms-21-06616-s001.pdf]

Supplementary Materials

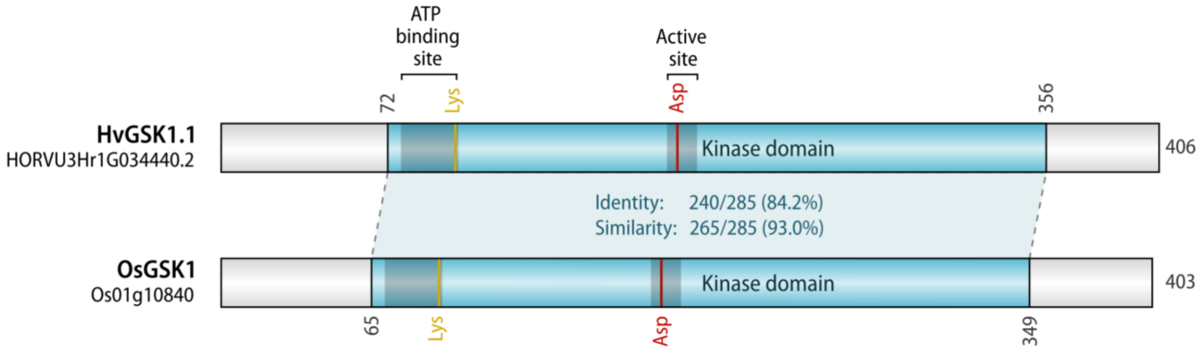

Figure S1. Protein domain organization of GSK orthologs encoded by: *HvGSK1.1* (HORVU3Hr1G034440.2) and *OsGSK1* (Os01g10840). The amino acid defining features of the kinase domains i.e. protein kinase domain (Pfam AC: PF00069), ATP binding site and kinase active site are indicated.

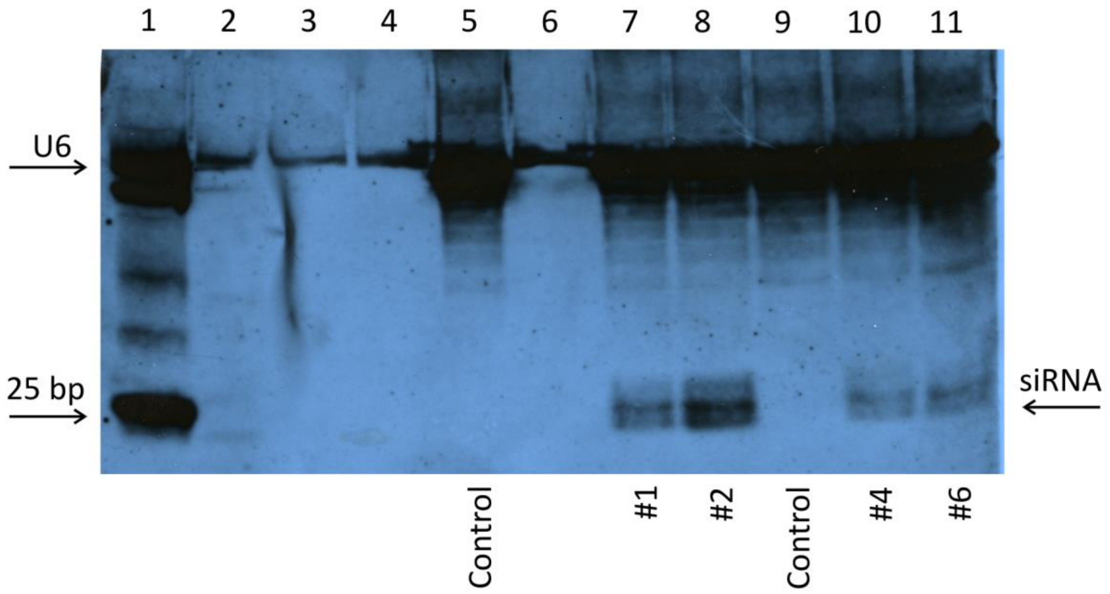

Figure S2. *Northern* blot detection of low molecular weight RNA in barley leaves in T_2 generation using DIG labeled probe complementary to *HvGSK1.1* ORF fragment in the silencing cassette. Indicated are U6 and 25 nt marker (lane 1), RNA from non-transgenic control plants (lanes 5, 9), RNA from lines #1, #2, #4 and #6. Lanes 2, 3, 4 and 6 were not loaded with the RNA to avoid cross contamination of neighboring lanes with the RNA samples.

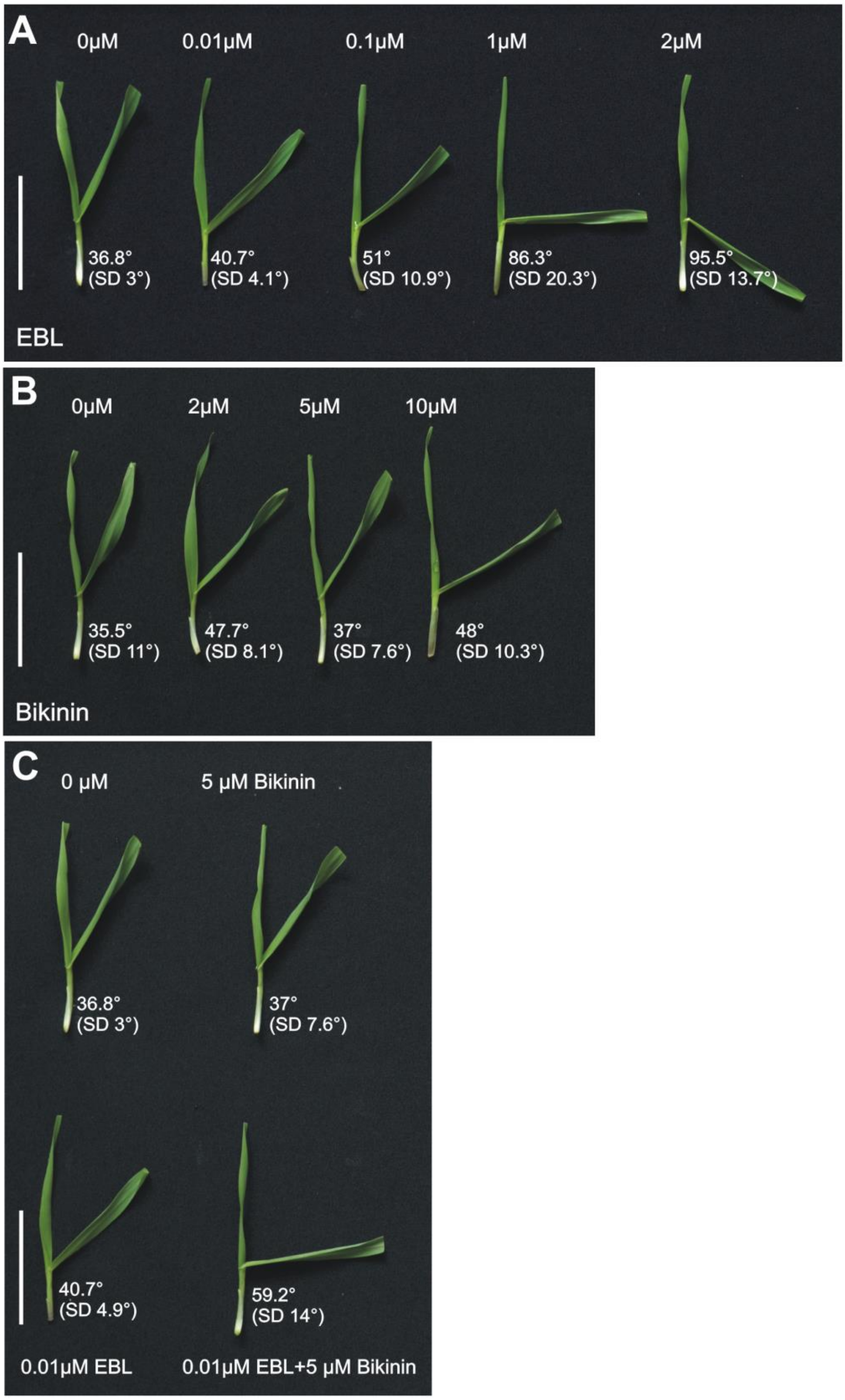

Figure S3. Representative pictures of leaf fragments from wild type barley cv. Golden Promise treated with indicated concentrations of 24-epibrassinolide (EBL) (A), bikinin (B). Pictures of leaf fragments treated with threshold concentrations of 0.01µM EBL and 5µM bikinin applied separately and together (C). Indicated are medium inclination angles and standard deviation (SD) of at least five plants. Bar represents 5 cm.

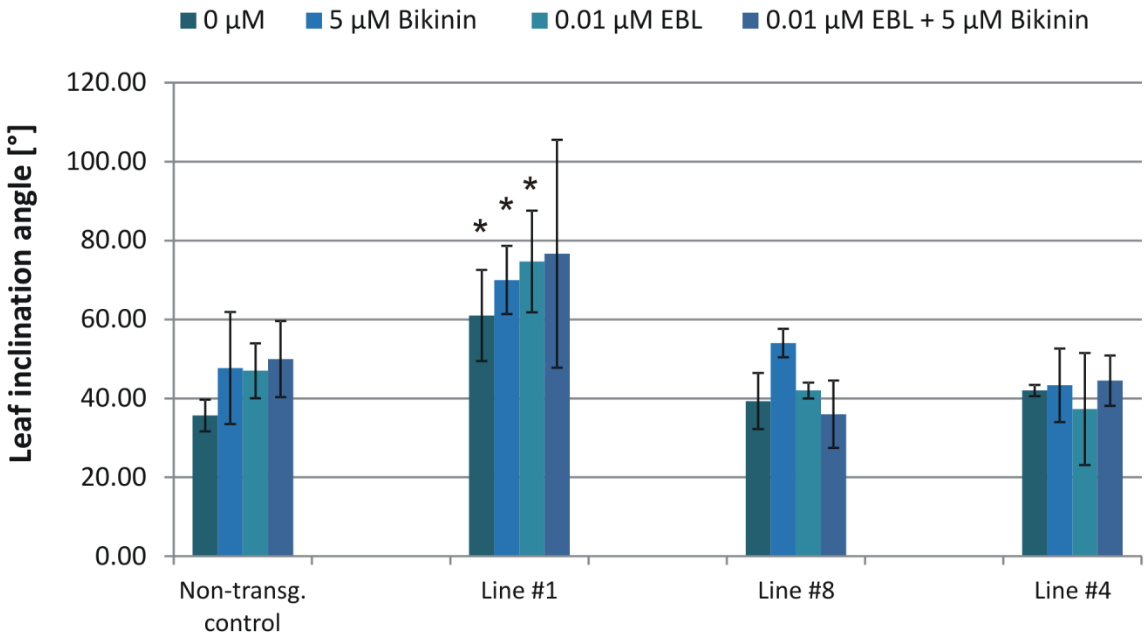

Figure S4. Leaf inclination angles of nontransgenic control and transgenic lines #1, #8 and #4, which represented strong, medium and weak silencing of HvGSK1.1. Leaf fragments were treated with 24-epibrassinolide (EBL) 0.01 µM, bikinin 5 µM and EBL (0.01 µM) and bikinin (5 µM) applied together. Data represent medium inclination angles and standard deviation (SD) of at least five plants.

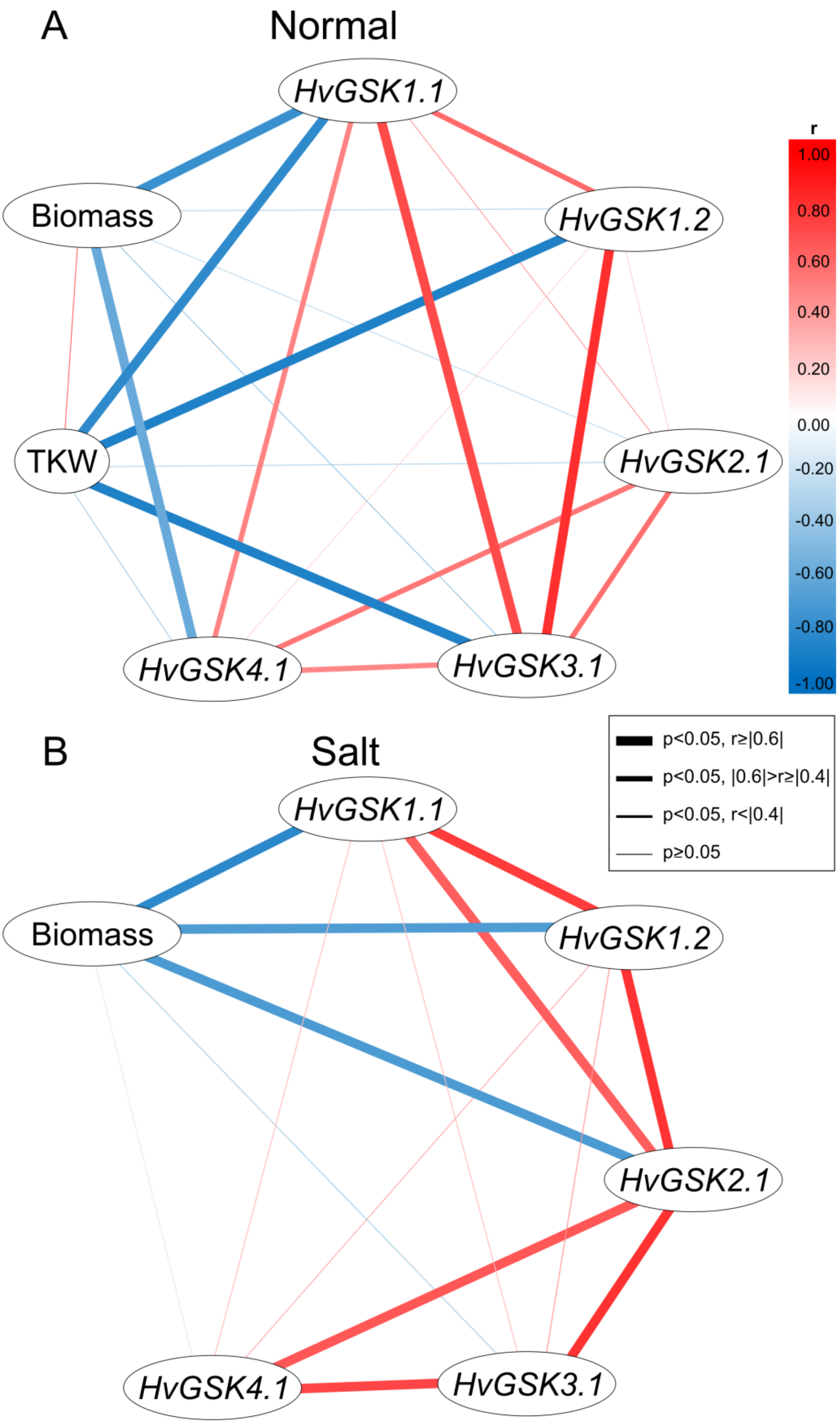

Figure S5. Graphical representation of Pearson correlation net between expression of *HvGSK1.1* and *HvGSK* paralogs, and the values of thousand kernel weight (TKW) measured in the non-transgenic control and the transgenic lines grown in normal conditions (A) and in salt stress conditions (Hoagland with NaCl 200 mM) (B). Positive and negative correlations are indicated with red and blue respectively. Bar width denotes the r values according to the scale.

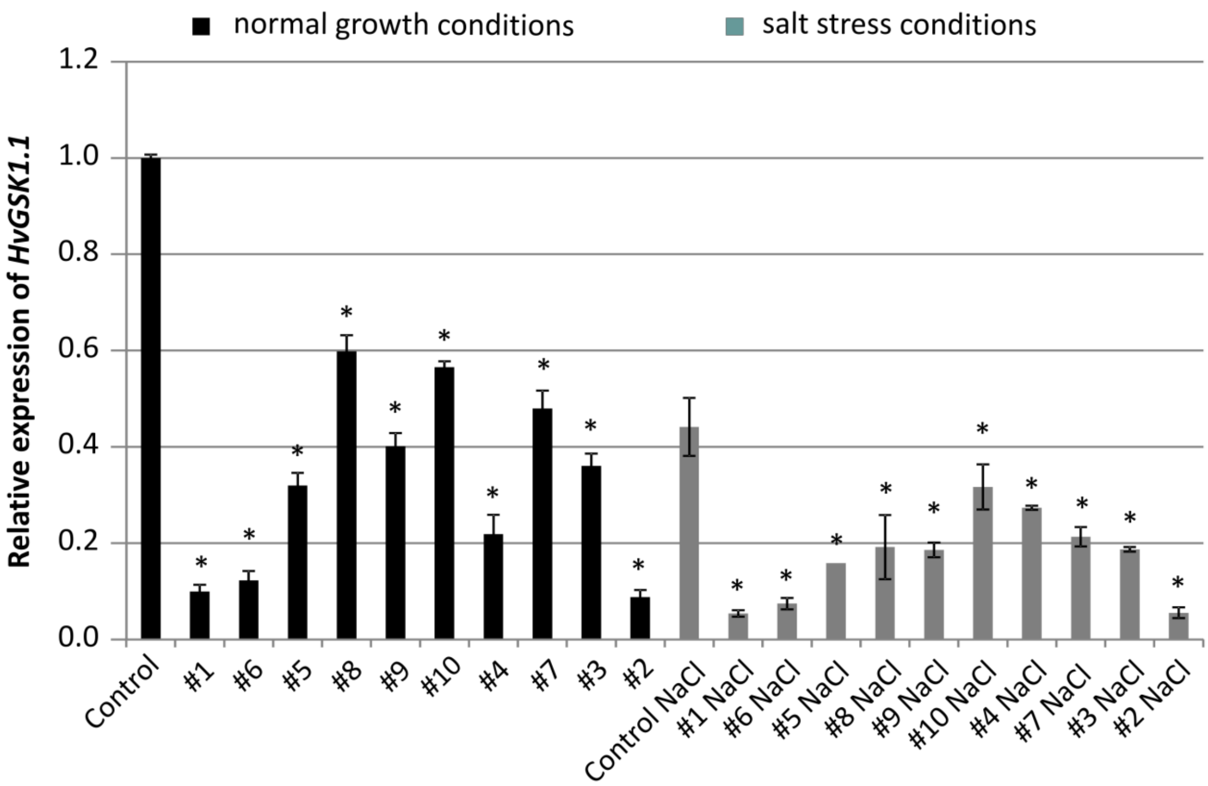

Figure S6. Relative expression of *HvGSK1.1* in leaves of T2 plants grown in normal (Hoagland medium) and in salt stress conditions (Hoagland medium supplemented with NaCl 200mM). Relative expression of *HvGSK1.1* in leaves of non-transgenic control plants grown in normal conditions was assumed as 1.0.

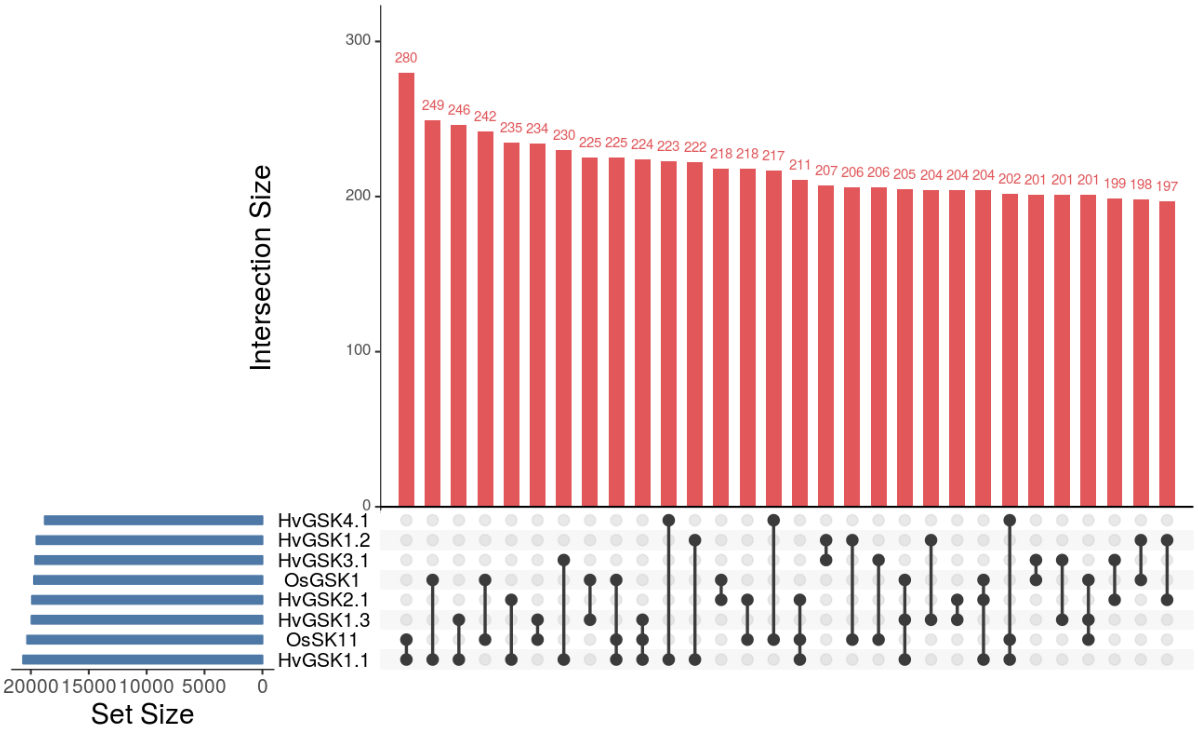

Figure S7. The Upset plot shows the number of shared transcription factor binding sites (TFBS) in the promoter regions of GSK-encoding genes from barley (*HvGSK1.1*, *HvGSK1.2*, *HvGSK1.3*, *HvGSK2.1*, *HvGSK3.1*, *HvGSK4.1*) and rice (*OsGSK1* and *OsGSK1.1*). Promoter region of *HvGSK1.1* showed the highest number of shared TFBS with (n = 280) and OsGSK1 (n = 249).

Table S1. The number of binding sites for brassinosteroid-dependent transcription factors BZR1 and BZR2 identified in the GSK-encoding genes based on TRANSFAC.

Gene name	BZR1	BZR2
HvGSK1.1	6	1
HvGSK1.2	5	0
HvGSK1.3	3	0
HvGSK2.1	2	1
HvGSK3.1	6	0
HvGSK4.1	6	2
OsGSK1	3	1
OsSK11	6	1

Supplementary Table 2. The list of all transcriptiopn factors binding sites (TFBS) in promoter regions of GSK-encoding genes in barley, rice and Arabidopsis.

Gene	TF_id	TF_name	start	end	strand	core_match_score	matrix_match_score	sequence
HvGSK1.1	P$MYB3_01	MYB3	16	27	1	1	0.862	aaaTAGGTata
HvGSK1.1	P$AT1G14580_01	AT1G14580	22	33	1	1	0.951	gtataGGGATa
HvGSK1.1	P$AT5G66730_01	AT5G66730	22	33	1	1	0.933	gtataGGGATa
HvGSK1.1	P$CBF3_02	CBF3	28	42	1	1	0.961	ggataCCGACgatc
HvGSK1.1	P$CBF1_04	CBF1	29	41	1	1	0.941	gataCCGACgat
HvGSK1.1	P$RAV2_01	RAV2	30	39	1	1	0.872	atACCGAcg
HvGSK1.1	P$AT1G77200_03	AT1G77200	30	44	1	1	0.859	ataCCGACgatcga
HvGSK1.1	P$ARF1_01	ARF1	32	40	1	1	0.897	aCCGACga
HvGSK1.1	P$ARF5_01	ARF5	32	40	1	1	0.898	aCCGACga
HvGSK1.1	P$DREB1B_01	DREB1B	33	38	1	1	1	CCGAC
HvGSK1.1	P$RAV2_01	RAV2	60	69	1	1	0.875	atACCGAag
HvGSK1.1	P$AT2G41690_01	AT2G41690	63	69	1	1	0.988	CCGAAg
HvGSK1.1	P$PBF_Q2	BF	71	77	1	1	0.958	cAAAGG
HvGSK1.1	P$ARR1_01	ARR1	95	105	1	1	0.944	tatGAATCct
HvGSK1.1	P$AT5G04240_01	AT5G04240	105	111	1	1	0.938	tGGCAC
HvGSK1.1	P$GAMYB_01	GAMYB	118	126	1	1	0.926	CAACCgat
HvGSK1.1	P$GATA9_01	GATA9	124	135	1	1	0.98	ataAGATCttc
HvGSK1.1	P$AGP1_01	AGP1	125	135	1	1	0.942	taAGATCttc
HvGSK1.1	P$ARR2_01	ARR2	125	135	1	1	0.898	taagATCTTc
HvGSK1.1	P$GATA10_01	GATA10	126	134	1	1	0.912	aAGATCtt
HvGSK1.1	P$GATA11_01	GATA11	126	134	1	1	0.949	aaGATCTt
HvGSK1.1	P$GATA8_01	GATA8	126	135	1	1	0.987	aaGATCTtc
HvGSK1.1	P$ARR10_01	ARR10	127	134	1	1	0.956	AGATCtt
HvGSK1.1	P$PCF5_01	CF5	165	175	1	1	0.885	caGGTCCcgc
HvGSK1.1	P$HSFA4A_01	HSFA4A	173	179	1	1	0.91	gCTATT
HvGSK1.1	P$WRKY11_Q2	WRKY11	183	191	1	1	0.974	aTTGACcg
HvGSK1.1	P$ZAP1_01	ZAP1	184	194	1	1	0.985	TTGACcgagg
HvGSK1.1	P$EDF3_02	EDF3	185	194	1	1	1	tGACCGagg
HvGSK1.1	P$RAV2_01	RAV2	185	194	1	1	1	tgACCGAgg
HvGSK1.1	P$O2_Q4	O2	202	213	1	1	0.88	gggtCATGTct
HvGSK1.1	P$ATHSFA1D_01	ATHSFA1D	210	216	1	1	0.941	tCTACA
HvGSK1.1	P$SPF1_Q2	SPF1	213	223	1	1	0.913	acATAGTtct
HvGSK1.1	P$ARR1_01	ARR1	221	231	1	1	0.957	ctcGAATCcg
HvGSK1.1	P$ABI3_01	ABI3	228	237	1	1	0.886	ccGCATGgt
HvGSK1.1	P$RAV2_01	RAV2	290	299	1	1	0.861	tcACCGAag
HvGSK1.1	P$AT2G41690_01	AT2G41690	293	299	1	1	0.988	CCGAAg
HvGSK1.1	P$GATA9_01	GATA9	316	327	1	1	0.879	atgAGATCacg
HvGSK1.1	P$AGP1_01	AGP1	317	327	1	1	0.851	tgAGATCacg
HvGSK1.1	P$ARR10_01	ARR10	319	326	1	1	0.869	AGATCac
HvGSK1.1	P$HBP1B_Q6	HBP1B	321	335	1	1	0.88	atcacggaCGTCAt
HvGSK1.1	P$TGA1B_01	TGA1B	325	335	1	1	0.89	cgGACGTcat
HvGSK1.1	P$HBPA1_Q6_01	HBPA1	325	335	1	1	0.86	cggaCGTCAt
HvGSK1.1	P$TGA1A_01	TGA1A	327	334	1	1	0.975	gACGTCa
HvGSK1.1	P$TGA2_Q2	TGA2	328	338	1	1	0.881	aCGTCAtgag
HvGSK1.1	P$GT1_Q6	GT1	359	366	1	1	0.912	GTGAAga
HvGSK1.1	P$KNOX3_01	KNOX3	363	375	1	1	0.963	agatTGACAtat
HvGSK1.1	P$WRKY11_Q2	WRKY11	365	373	1	1	0.903	aTTGACat
HvGSK1.1	P$ATH1_01	ATH1	367	375	1	1	0.935	TGACAtat
HvGSK1.1	P$UIF1_01	UIF1	399	409	1	1	1	aaaGATTCcg
HvGSK1.1	P$ATSPL8_01	ATSPL8	418	434	1	1	0.866	ggcagTGTACcgggag
HvGSK1.1	P$MYB89_01	MYB89	423	434	1	1	0.914	tgTACCGggag
HvGSK1.1	P$TGA1_01	TGA1	431	442	1	1	0.931	gagTGACGaat
HvGSK1.1	P$TGA7_01	TGA7	432	442	1	1	0.883	agTGACGaat
HvGSK1.1	P$TGA5_01	TGA5	433	441	1	1	0.865	gTGACGaa
HvGSK1.1	P$ALFIN1_Q2	ALFIN1	456	471	1	1	0.854	caccggGTGGGccca
HvGSK1.1	P$TCP11_01	TCP11	461	473	1	1	0.989	gGTGGGcccacc
HvGSK1.1	P$TCP15_01	TCP15	462	472	1	1	0.962	GTGGGcccac
HvGSK1.1	P$TCP20_01	TCP20	462	472	1	1	0.987	GTGGGcccac
HvGSK1.1	P$TCP11_02	TCP11	462	472	1	1	0.969	GTGGGcccac
HvGSK1.1	P$TCP7_01	TCP7	462	473	1	1	0.853	GTGGGcccacc
HvGSK1.1	P$OJ1581_01	OJ1581	462	472	1	1	1	gtGGGCCcac
HvGSK1.1	P$TCP2_01	TCP2	462	472	1	1	1	gtGGGCCcac
HvGSK1.1	P$PCF2_01	CF2	462	472	1	1	0.997	gtgggCCCAC
HvGSK1.1	P$TCP19_01	TCP19	462	472	1	1	0.987	gtgggCCCAC
HvGSK1.1	P$TCP20L_01	TCP20L	463	472	1	1	0.994	tgggCCCAC
HvGSK1.1	P$OSI_01	OSI	464	472	1	1	1	gggCCCAC
HvGSK1.1	P$TCP20_02	TCP20	464	474	1	1	1	gggCCCACcc
HvGSK1.1	P$ARALY495258_02	ARALY495258	464	472	1	1	1	gggCCCAC
HvGSK1.1	P$ARALY493022_04	ARALY493022	464	472	1	1	1	gggCCCAC
HvGSK1.1	P$ARALY484486_05	ARALY484486	464	472	1	1	1	gggCCCAC
HvGSK1.1	P$ASR1_01	ASR1	470	475	1	1	1	ACCCA
HvGSK1.1	P$RAV1_02	RAV1	471	483	1	1	0.987	cccACCTGggag
HvGSK1.1	P$EDT1_01	EDT1	514	524	1	1	0.913	cccTTAATgg
HvGSK1.1	P$BBM_01	BBM	551	561	1	1	0.863	tGGCGCcaca
HvGSK1.1	P$E2FA_02	E2FA	551	561	1	1	0.997	tggCGCCAca

HvGSK1.1	P$FLC_01	FLC	551	572	1	1	0.857	tggcgccacaaggAGAAAaaa
HvGSK1.1	P$BPC1_Q2	BPC1	564	570	1	1	0.997	AGAAAa
HvGSK1.1	P$AT4G36620_01	AT4G36620	567	575	1	1	0.919	aaaAACCA
HvGSK1.1	P$HSFA2_01	HSFA2	572	578	1	1	0.933	CCAAAg
HvGSK1.1	P$PBF_Q2	BF	573	579	1	1	0.958	cAAAGG
HvGSK1.1	P$PBF_01	BF	576	587	1	1	0.966	aggAAAAGaaa
HvGSK1.1	P$DOF_Q2	DOF	576	587	1	1	0.96	aggAAAAGaaa
HvGSK1.1	P$AT3G51080_01	AT3G51080	577	584	1	1	0.918	GGAAAag
HvGSK1.1	P$CDF2_01	CDF2	577	587	1	1	0.95	ggAAAAGaaa
HvGSK1.1	P$CDF3_01	CDF3	578	587	1	1	0.971	gAAAAGaaa
HvGSK1.1	P$BPC1_Q2	BPC1	582	588	1	1	0.997	AGAAAa
HvGSK1.1	P$ALFIN1_Q2	ALFIN1	599	614	1	1	0.915	agggagGTGGGaagg
HvGSK1.1	P$HSFA2_01	HSFA2	633	639	1	1	0.941	CCAAAc
HvGSK1.1	P$AT4G12750_01	AT4G12750	634	644	1	1	0.911	caaACCGAat
HvGSK1.1	P$AT2G41690_01	AT2G41690	638	644	1	1	0.974	CCGAAt
HvGSK1.1	P$ALFIN1_Q2	ALFIN1	642	657	1	1	0.856	attgggGTGGGagtc
HvGSK1.1	P$WRKY44_01	WRKY44	653	662	1	1	1	AGTCCacct
HvGSK1.1	P$LIM1_01	LIM1	656	668	1	1	0.866	CCACCtcctccc
HvGSK1.1	P$CBF3_02	CBF3	668	682	1	1	0.955	atcggCCGACgccc
HvGSK1.1	P$CBF1_04	CBF1	669	681	1	1	0.954	tcggCCGACgcc
HvGSK1.1	P$ERF3_02	ERF3	670	680	1	1	0.858	cGGCCGacgc
HvGSK1.1	P$DREB1E_01	DREB1E	670	680	1	1	0.875	cGGCCGacgc
HvGSK1.1	P$ERF2_04	ERF2	670	680	1	1	0.867	cGGCCGacgc
HvGSK1.1	P$ERF3_03	ERF3	670	680	1	1	0.866	cGGCCGacgc
HvGSK1.1	P$ATERF12_01	ATERF12	670	680	1	1	0.861	cGGCCGacgc
HvGSK1.1	P$AT1G53910_01	AT1G53910	670	680	1	1	0.97	cGGCCGacgc
HvGSK1.1	P$AT5G67000_01	AT5G67000	670	680	1	1	0.873	cgGCCGAcgc
HvGSK1.1	P$ERF5_02	ERF5	670	680	1	1	0.906	cgGCCGAcgc
HvGSK1.1	P$ERF1_04	ERF1	670	680	1	1	0.901	cgGCCGAcgc
HvGSK1.1	P$AT2G20350_01	AT2G20350	670	680	1	1	0.858	cgGCCGAcgc
HvGSK1.1	P$DREB1G_02	DREB1G	670	680	1	1	0.901	cggCCGACgc
HvGSK1.1	P$ARF1_01	ARF1	672	680	1	1	0.855	gCCGACgc
HvGSK1.1	P$ARF5_01	ARF5	672	680	1	1	0.894	gCCGACgc
HvGSK1.1	P$DREB1B_01	DREB1B	673	678	1	1	1	CCGAC
HvGSK1.1	P$ANAC013_Q2	ANAC013	691	705	1	1	0.876	cctgagcccCCAAGg
HvGSK1.1	P$MYB24_01	MYB24	738	747	1	1	0.859	gttTTAGGg
HvGSK1.1	P$GAMYB_Q2	GAMYB	752	765	1	1	0.867	gagacACAACttt
HvGSK1.1	P$PIL5_01	IL5	760	774	1	1	0.963	actttgccACGTGt
HvGSK1.1	P$PIF3_01	IF3	761	779	1	1	0.88	ctttgcCACGTgtaactc
HvGSK1.1	P$PIF3_02	IF3	762	779	1	1	0.866	tttgcCACGTgtaactc
HvGSK1.1	P$HY5_02	HY5	762	778	1	1	0.925	tttgcCACGTgtaact
HvGSK1.1	P$ABF2_01	ABF2	762	775	1	1	0.954	tttgcCACGTgta
HvGSK1.1	P$ABF4_Q2	ABF4	762	776	1	1	0.944	tttgccACGTGtaa
HvGSK1.1	P$O2_Q4	O2	763	774	1	1	0.893	ttgcCACGTgt
HvGSK1.1	P$BZR1_02	BZR1	763	777	1	1	0.967	ttgcCACGTgtaac
HvGSK1.1	P$HBI1_01	HBI1	763	775	1	1	0.94	ttgcCACGTgta
HvGSK1.1	P$ABZ1_01	ABZ1	763	777	1	1	0.923	ttgccACGTGtaac
HvGSK1.1	P$GBP_Q6	GBP	764	776	1	1	0.95	tgcCACGTgtaa
HvGSK1.1	P$GBF_Q2	GBF	764	776	1	1	0.907	tgcCACGTgtaa
HvGSK1.1	P$PIF3_03	IF3	764	774	1	1	0.99	tgcCACGTgt
HvGSK1.1	P$ABI5_01	ABI5	764	774	1	1	0.979	tgcCACGTgt
HvGSK1.1	P$ABF4_01	ABF4	764	776	1	1	0.96	tgcCACGTgtaa
HvGSK1.1	P$BZR1_03	BZR1	764	784	1	1	0.871	tgccACGTGtaactcaaacc
HvGSK1.1	P$EMBP1_Q2	EMBP1	765	775	1	1	0.997	gcCACGTgta
HvGSK1.1	P$CPRF_Q2	CPRF	765	775	1	1	0.98	gcCACGTgta
HvGSK1.1	P$CPRF3_Q2	CPRF3	765	775	1	1	0.99	gcCACGTgta
HvGSK1.1	P$CPRF2_Q2	CPRF2	765	775	1	1	0.996	gcCACGTgta
HvGSK1.1	P$O2_02	O2	765	775	1	1	0.982	gcCACGTgta
HvGSK1.1	P$TGA1B_Q2	TGA1B	765	775	1	1	0.929	gcCACGTgta
HvGSK1.1	P$TGA1A_Q2	TGA1A	765	775	1	1	0.979	gcCACGTgta
HvGSK1.1	P$CG1_Q6	CG1	765	776	1	1	0.855	gcCACGTgtaa
HvGSK1.1	P$CPRF1_01	CPRF1	765	775	1	1	1	gcCACGTgta
HvGSK1.1	P$CPRF3_01	CPRF3	765	775	1	1	0.988	gcCACGTgta
HvGSK1.1	P$CPRF2_01	CPRF2	765	775	1	1	0.997	gcCACGTgta
HvGSK1.1	P$TGA1B_01	TGA1B	765	775	1	1	0.928	gcCACGTgta
HvGSK1.1	P$BES1_01	BES1	765	776	1	1	0.961	gcCACGTgtaa
HvGSK1.1	P$PIF3_04	IF3	765	775	1	1	0.881	gcCACGTgta
HvGSK1.1	P$BEE2_01	BEE2	765	775	1	1	0.999	gcCACGTgta
HvGSK1.1	P$BIM2_01	BIM2	765	775	1	1	0.994	gcCACGTgta
HvGSK1.1	P$BIM3_01	BIM3	765	775	1	1	0.991	gcCACGTgta
HvGSK1.1	P$PHYPA143875_02	HYPA143875	765	775	1	1	0.997	gcCACGTgta
HvGSK1.1	P$PHYPA72483_07	HYPA72483	765	775	1	1	0.997	gcCACGTgta
HvGSK1.1	P$SPT_01	SPT	765	774	1	1	0.997	gcCACGTgt
HvGSK1.1	P$GBF1F_Q2	GBF1F	765	776	1	1	0.998	gcCACGTgtaa
HvGSK1.1	P$HBP1A_Q2	HBP1A	765	775	1	1	0.939	gccACGTGta
HvGSK1.1	P$TAF1_Q2	TAF1	765	775	1	1	0.974	gccACGTGta
HvGSK1.1	P$EMBP1_02	EMBP1	765	775	1	1	0.889	gccACGTGta
HvGSK1.1	P$TAF1_01	TAF1	765	775	1	1	0.984	gccACGTGta

HvGSK1.1	P$PIF1_01	IF1	765	775	1	1	0.99	gccACGTGta
HvGSK1.1	P$RITA1_01	RITA1	766	773	1	1	0.997	cCACGTg
HvGSK1.1	P$BHLH66_01	BHLH66	766	774	1	1	0.917	cCACGTgt
HvGSK1.1	P$PIF5_01	IF5	766	774	1	1	0.931	cCACGTgt
HvGSK1.1	P$MYC2_01	MYC2	766	774	1	1	0.953	cCACGTgt
HvGSK1.1	P$MYC3_01	MYC3	766	774	1	1	0.994	cCACGTgt
HvGSK1.1	P$BHLH34_01	BHLH34	766	774	1	1	0.961	cCACGTgt
HvGSK1.1	P$PHYPA48267_08	HYPA48267	766	774	1	1	0.978	cCACGTgt
HvGSK1.1	P$OJ1058_01	OJ1058	766	774	1	1	0.973	cCACGTgt
HvGSK1.1	P$UNE10_01	UNE10	766	774	1	1	0.988	cCACGTgt
HvGSK1.1	P$BHLH3_01	BHLH3	766	774	1	1	0.952	cCACGTgt
HvGSK1.1	P$TRAB1_Q2	TRAB1	766	777	1	1	0.885	ccACGTGtaac
HvGSK1.1	P$GBF1_01	GBF1	766	774	1	1	0.976	ccACGTGt
HvGSK1.1	P$MYC4_01	MYC4	766	774	1	1	0.956	ccACGTGt
HvGSK1.1	P$BIM1_02	BIM1	766	776	1	1	0.994	ccACGTGtaa
HvGSK1.1	P$BHLH13_01	BHLH13	766	774	1	1	0.947	ccACGTGt
HvGSK1.1	P$ABF4_02	ABF4	766	776	1	1	0.994	ccACGTGtaa
HvGSK1.1	P$OCSBF1_01	OCSBF1	767	772	1	1	1	CACGT
HvGSK1.1	P$PIF4_01	IF4	767	775	1	1	0.958	CACGTgta
HvGSK1.1	P$ABI5_Q2	ABI5	768	774	1	1	0.979	ACGTGt
HvGSK1.1	P$ABF2_01	ABF2	784	797	1	1	0.918	tatacCACGTagt
HvGSK1.1	P$O2_Q4	O2	785	796	1	1	0.887	atacCACGTag
HvGSK1.1	P$BZR1_02	BZR1	785	799	1	1	0.853	atacCACGTagttc
HvGSK1.1	P$GBP_Q6	GBP	786	798	1	1	0.9	tacCACGTagtt
HvGSK1.1	P$ABI5_01	ABI5	786	796	1	1	0.913	tacCACGTag
HvGSK1.1	P$ABF4_01	ABF4	786	798	1	1	0.904	tacCACGTagtt
HvGSK1.1	P$EMBP1_Q2	EMBP1	787	797	1	1	0.883	acCACGTagt
HvGSK1.1	P$CPRF3_Q2	CPRF3	787	797	1	1	0.92	acCACGTagt
HvGSK1.1	P$CPRF2_Q2	CPRF2	787	797	1	1	0.949	acCACGTagt
HvGSK1.1	P$O2_02	O2	787	797	1	1	0.982	acCACGTagt
HvGSK1.1	P$TGA1B_Q2	TGA1B	787	797	1	1	0.895	acCACGTagt
HvGSK1.1	P$TGA1A_Q2	TGA1A	787	797	1	1	0.962	acCACGTagt
HvGSK1.1	P$CPRF3_01	CPRF3	787	797	1	1	0.923	acCACGTagt
HvGSK1.1	P$CPRF2_01	CPRF2	787	797	1	1	0.951	acCACGTagt
HvGSK1.1	P$TGA1B_01	TGA1B	787	797	1	1	0.861	acCACGTagt
HvGSK1.1	P$PIF3_04	IF3	787	797	1	1	0.864	acCACGTagt
HvGSK1.1	P$BEE2_01	BEE2	787	797	1	1	0.915	acCACGTagt
HvGSK1.1	P$BIM2_01	BIM2	787	797	1	1	0.851	acCACGTagt
HvGSK1.1	P$BIM3_01	BIM3	787	797	1	1	0.887	acCACGTagt
HvGSK1.1	P$PHYPA143875_02	HYPA143875	787	797	1	1	0.876	acCACGTagt
HvGSK1.1	P$SPT_01	SPT	787	796	1	1	0.942	acCACGTag
HvGSK1.1	P$GBF1F_Q2	GBF1F	787	798	1	1	0.919	acCACGTagtt
HvGSK1.1	P$RITA1_01	RITA1	788	795	1	1	0.987	cCACGTa
HvGSK1.1	P$OCSBF1_01	OCSBF1	789	794	1	1	1	CACGT
HvGSK1.1	P$GATA9_01	GATA9	803	814	1	1	0.905	tctAGATCgga
HvGSK1.1	P$AGP1_01	AGP1	804	814	1	1	0.861	ctAGATCgga
HvGSK1.1	P$GATA10_01	GATA10	805	813	1	1	0.928	tAGATCgg
HvGSK1.1	P$ARR10_01	ARR10	806	813	1	1	0.934	AGATCgg
HvGSK1.1	P$DOF2_01	DOF2	828	839	1	1	0.979	gggcAAAGCcc
HvGSK1.1	P$DOF3_01	DOF3	828	839	1	1	0.984	gggcAAAGCcc
HvGSK1.1	P$TSAR2_01	TSAR2	839	849	1	1	0.931	tGCACGagca
HvGSK1.1	P$BHLH78_01	BHLH78	840	848	1	1	0.875	GCACGagc
HvGSK1.1	P$GATA9_01	GATA9	845	856	1	1	0.887	agcAGATCatc
HvGSK1.1	P$AGP1_01	AGP1	846	856	1	1	0.86	gcAGATCatc
HvGSK1.1	P$ARR10_01	ARR10	848	855	1	1	0.913	AGATCat
HvGSK1.1	P$LIM1_01	LIM1	857	869	1	1	0.925	CCACCactggca
HvGSK1.1	P$AT5G04240_01	AT5G04240	864	870	1	1	0.938	tGGCAC
HvGSK1.1	P$RRTF1_02	RRTF1	867	877	1	1	0.861	caCGCCGtca
HvGSK1.1	P$ERF112_02	ERF112	867	877	1	1	0.975	caCGCCGtca
HvGSK1.1	P$CRF4_01	CRF4	868	876	1	1	0.924	aCGCCGtc
HvGSK1.1	P$ERF4_04	ERF4	868	876	1	1	0.952	aCGCCGtc
HvGSK1.1	P$ERF069_01	ERF069	868	877	1	1	0.994	aCGCCGtca
HvGSK1.1	P$ERF11_01	ERF11	868	878	1	1	0.995	aCGCCGtcac
HvGSK1.1	P$ERF8_01	ERF8	869	879	1	1	0.992	CGCCGtcacg
HvGSK1.1	P$ERF3_04	ERF3	869	877	1	1	0.969	CGCCGtca
HvGSK1.1	P$ANAC042_01	ANAC042	869	889	1	1	0.96	cGCCGTcacgctgccggtga
HvGSK1.1	P$ANAC094_01	ANAC094	869	887	1	1	0.935	cGCCGTcacgctgccggt
HvGSK1.1	P$CBF1_01	CBF1	879	889	1	1	0.888	cTGCCGgtga
HvGSK1.1	P$ERF019_01	ERF019	879	889	1	1	0.912	cTGCCGgtga
HvGSK1.1	P$DREBIII4_01	DREBIII4	879	889	1	1	0.892	cTGCCGgtga
HvGSK1.1	P$JERF3_01	JERF3	879	889	1	1	0.862	cTGCCGgtga
HvGSK1.1	P$CEF1_01	CEF1	879	889	1	1	0.862	cTGCCGgtga
HvGSK1.1	P$JERF1_01	JERF1	879	889	1	1	0.907	cTGCCGgtga
HvGSK1.1	P$CBF1_03	CBF1	879	889	1	1	0.918	cTGCCGgtga
HvGSK1.1	P$DREB1F_01	DREB1F	879	889	1	1	0.858	cTGCCGgtga
HvGSK1.1	P$AT1G33760_01	AT1G33760	879	889	1	1	0.89	cTGCCGgtga
HvGSK1.1	P$AT1G71520_01	AT1G71520	879	889	1	1	0.919	cTGCCGgtga
HvGSK1.1	P$ORA47_01	ORA47	879	889	1	1	0.888	cTGCCGgtga

HvGSK1.1	P$RAP21_02	RAP21	879	892	1	1	0.926	ctgcCGGTGaact
HvGSK1.1	P$GATA9_01	GATA9	929	940	1	1	0.88	cggAGATCgtc
HvGSK1.1	P$AGP1_01	AGP1	930	940	1	1	0.852	ggAGATCgtc
HvGSK1.1	P$ARR10_01	ARR10	932	939	1	1	0.913	AGATCgt
HvGSK1.1	P$TGA2_Q2	TGA2	935	945	1	1	0.929	tCGTCAtcga
HvGSK1.1	P$ATSPL8_01	ATSPL8	942	958	1	1	0.873	cgagtTGTACgtgtgc
HvGSK1.1	P$SPL11_01	SPL11	944	956	1	1	0.902	agttGTACGtgt
HvGSK1.1	P$HMG1_01	HMG1	945	954	1	1	0.86	GTTGTacgt
HvGSK1.1	P$ABZ1_01	ABZ1	945	959	1	1	0.922	gttgtACGTGtgct
HvGSK1.1	P$SPL5_01	SPL5	946	955	1	1	0.973	ttGTACGtg
HvGSK1.1	P$POPTR_01	OPTR	947	954	1	1	0.93	tGTACGt
HvGSK1.1	P$SPL12_01	SPL12	947	955	1	1	0.977	tGTACGtg
HvGSK1.1	P$SPL4_01	SPL4	947	956	1	1	0.992	tGTACGtgt
HvGSK1.1	P$HBP1A_Q2	HBP1A	947	957	1	1	0.858	tgtACGTGtg
HvGSK1.1	P$TAF1_Q2	TAF1	947	957	1	1	0.912	tgtACGTGtg
HvGSK1.1	P$TAF1_01	TAF1	947	957	1	1	0.937	tgtACGTGtg
HvGSK1.1	P$GBF1_01	GBF1	948	956	1	1	0.959	gtACGTGt
HvGSK1.1	P$BIM1_02	BIM1	948	958	1	1	0.946	gtACGTGtgc
HvGSK1.1	P$ABF4_02	ABF4	948	958	1	1	0.981	gtACGTGtgc
HvGSK1.1	P$ABI5_Q2	ABI5	950	956	1	1	0.979	ACGTGt
HvGSK1.1	P$MYB3R1_01	MYB3R1	968	983	1	1	0.855	tgtgccgtCCGTTcg
HvGSK1.1	P$CBF1_01	CBF1	969	979	1	1	0.918	gTGCCGtccg
HvGSK1.1	P$ERF019_01	ERF019	969	979	1	1	0.898	gTGCCGtccg
HvGSK1.1	P$JERF3_01	JERF3	969	979	1	1	0.909	gTGCCGtccg
HvGSK1.1	P$CEF1_01	CEF1	969	979	1	1	0.912	gTGCCGtccg
HvGSK1.1	P$JERF1_01	JERF1	969	979	1	1	0.929	gTGCCGtccg
HvGSK1.1	P$CBF1_03	CBF1	969	979	1	1	0.919	gTGCCGtccg
HvGSK1.1	P$AT1G71450_01	AT1G71450	969	979	1	1	0.865	gTGCCGtccg
HvGSK1.1	P$DREB1F_01	DREB1F	969	979	1	1	0.942	gTGCCGtccg
HvGSK1.1	P$AT1G33760_01	AT1G33760	969	979	1	1	0.895	gTGCCGtccg
HvGSK1.1	P$DREB1E_02	DREB1E	969	979	1	1	0.853	gTGCCGtccg
HvGSK1.1	P$DREB2F_01	DREB2F	969	979	1	1	0.861	gTGCCGtccg
HvGSK1.1	P$RAP210_02	RAP210	969	979	1	1	0.907	gTGCCGtccg
HvGSK1.1	P$ANAC042_01	ANAC042	970	990	1	1	0.942	tGCCGTccgttcggcgctag
HvGSK1.1	P$ANAC094_01	ANAC094	970	988	1	1	0.911	tGCCGTccgttcggcgct
HvGSK1.1	P$AT1G68550_03	AT1G68550	978	987	1	1	0.957	gttCGGCGc
HvGSK1.1	P$GATA9_01	GATA9	985	996	1	1	0.905	gctAGATCgga
HvGSK1.1	P$AGP1_01	AGP1	986	996	1	1	0.861	ctAGATCgga
HvGSK1.1	P$GATA10_01	GATA10	987	995	1	1	0.928	tAGATCgg
HvGSK1.1	P$ARR10_01	ARR10	988	995	1	1	0.934	AGATCgg
HvGSK1.1	P$RAP21_02	RAP21	1008	1021	1	1	0.914	acgaCGGTGattt
HvGSK1.1	P$ARR1_01	ARR1	1018	1028	1	1	0.941	tttGAATCac
HvGSK1.1	P$HAT1_01	HAT1	1020	1030	1	1	0.857	tgAATCAcga
HvGSK1.1	P$ATSPL8_01	ATSPL8	1028	1044	1	1	0.924	gaagaTGTACcactac
HvGSK1.1	P$TEIL_01	TEIL	1032	1040	1	1	0.881	ATGTAcca
HvGSK1.1	P$ATHSFA1D_01	ATHSFA1D	1039	1045	1	1	1	aCTACA
HvGSK1.1	P$GAMYB_01	GAMYB	1046	1054	1	1	0.935	CAACCgcg
HvGSK1.1	P$CMTA2_01	CMTA2	1046	1055	1	1	1	caacCGCGT
HvGSK1.1	P$CAMTA1_02	CAMTA1	1046	1058	1	1	0.966	caacCGCGTttc
HvGSK1.1	P$CMTA3_01	CMTA3	1049	1058	1	1	1	cCGCGTttc
HvGSK1.1	P$GT1_Q6_02	GT1	1052	1064	1	1	0.948	cgtttcTTAACg
HvGSK1.1	P$AT1G66560_01	AT1G66560	1055	1065	1	1	0.895	ttcTTAACgc
HvGSK1.1	P$WRKY21_01	WRKY21	1056	1065	1	1	0.881	tcTTAACgc
HvGSK1.1	P$WRKY43_01	WRKY43	1056	1065	1	1	0.876	tcTTAACgc
HvGSK1.1	P$AT4G11070_01	AT4G11070	1056	1065	1	1	0.948	tcTTAACgc
HvGSK1.1	P$AT1G18860_01	AT1G18860	1056	1065	1	1	0.897	tcTTAACgc
HvGSK1.1	P$AT1G64000_01	AT1G64000	1056	1065	1	1	0.874	tcTTAACgc
HvGSK1.1	P$AT1G66600_01	AT1G66600	1056	1065	1	1	0.891	tcTTAACgc
HvGSK1.1	P$AT1G68150_01	AT1G68150	1056	1065	1	1	0.858	tcTTAACgc
HvGSK1.1	P$AT5G41570_01	AT5G41570	1056	1065	1	1	0.874	tcTTAACgc
HvGSK1.1	P$AT5G15130_01	AT5G15130	1056	1065	1	1	0.893	tcTTAACgc
HvGSK1.1	P$WRKY46_01	WRKY46	1056	1065	1	1	0.904	tcTTAACgc
HvGSK1.1	P$AT2G24570_01	AT2G24570	1056	1065	1	1	0.871	tcTTAACgc
HvGSK1.1	P$WRKY7_01	WRKY7	1056	1065	1	1	0.886	tcTTAACgc
HvGSK1.1	P$E2F_Q2	E2F	1062	1073	1	1	0.875	cgcTTCCCgct
HvGSK1.1	P$GATA8_01	GATA8	1075	1084	1	1	0.99	gcGATCTac
HvGSK1.1	P$ATHSFA1D_01	ATHSFA1D	1079	1085	1	1	0.941	tCTACA
HvGSK1.1	P$MYBAS1_01	MYBAS1	1096	1107	1	1	0.984	atCCAACctcc
HvGSK1.1	P$GAMYB_01	GAMYB	1099	1107	1	1	0.933	CAACCtcc
HvGSK1.1	P$MYB3_01	MYB3	1129	1140	1	1	0.902	tgaTAGGTctt
HvGSK1.1	P$MYB4_01	MYB4	1130	1138	1	1	0.871	gaTAGGTc
HvGSK1.1	P$RAV1_02	RAV1	1186	1198	1	1	0.901	gggACCTGtgcc
HvGSK1.1	P$GAMYB_Q2	GAMYB	1202	1215	1	1	0.885	ccagaACAACatg
HvGSK1.1	P$RAV1_01	RAV1	1205	1217	1	1	0.917	gaaCAACAtgca
HvGSK1.1	P$LEC2_01	LEC2	1209	1220	1	1	0.988	aaCATGCatct
HvGSK1.1	P$FUS3_Q2	FUS3	1210	1221	1	1	0.891	aCATGCatcta
HvGSK1.1	P$IDEF1_Q2	IDEF1	1211	1223	1	1	0.901	CATGCatctagg
HvGSK1.1	P$E2L_Q2	E2L	1226	1233	1	1	1	tGGCGGg

HvGSK1.1	P$HSF3_01	HSF3	1228	1234	1	1	0.945	gCGGGG
HvGSK1.1	P$TCP16_03	TCP16	1228	1239	1	1	0.975	gcggggTCCAC
HvGSK1.1	P$PCF5_01	CF5	1230	1240	1	1	0.855	ggGGTCCact
HvGSK1.1	P$BHLH112_01	BHLH112	1234	1243	1	1	1	tccACTTGt
HvGSK1.1	P$WRKY18_02	WRKY18	1238	1248	1	1	0.947	cttGTCAAtg
HvGSK1.1	P$WRKY21_02	WRKY21	1238	1248	1	1	0.954	cttGTCAAtg
HvGSK1.1	P$WRKY48_02	WRKY48	1238	1248	1	1	0.986	cttGTCAAtg
HvGSK1.1	P$WRKY57_01	WRKY57	1238	1248	1	1	0.959	cttGTCAAtg
HvGSK1.1	P$WRKY60_01	WRKY60	1238	1249	1	1	0.894	cttGTCAAtgg
HvGSK1.1	P$WRKY15_01	WRKY15	1239	1249	1	1	0.961	ttGTCAAtgg
HvGSK1.1	P$WRKY2_01	WRKY2	1239	1247	1	1	0.904	ttGTCAAt
HvGSK1.1	P$WRKY25_02	WRKY25	1239	1247	1	1	0.888	ttGTCAAt
HvGSK1.1	P$WRKY40_01	WRKY40	1239	1247	1	1	0.977	ttGTCAAt
HvGSK1.1	P$WRKY43_02	WRKY43	1239	1249	1	1	0.952	ttGTCAAtgg
HvGSK1.1	P$WRKY62_01	WRKY62	1239	1247	1	1	0.87	ttGTCAAt
HvGSK1.1	P$WRKY63_01	WRKY63	1239	1247	1	1	0.886	ttGTCAAt
HvGSK1.1	P$WRKY75_01	WRKY75	1239	1247	1	1	0.927	ttGTCAAt
HvGSK1.1	P$WRKY8_01	WRKY8	1239	1248	1	1	0.978	ttGTCAAtg
HvGSK1.1	P$WRKY30_01	WRKY30	1240	1250	1	1	0.911	tGTCAAtgga
HvGSK1.1	P$WRKY18_Q2	WRKY18	1241	1250	1	1	0.945	GTCAAtgga
HvGSK1.1	P$PBF_Q2	BF	1251	1257	1	1	0.958	cAAAGG
HvGSK1.1	P$BPC1_Q2	BPC1	1270	1276	1	1	0.997	AGAAAa
HvGSK1.1	P$HSFA2_01	HSFA2	1325	1331	1	1	0.922	CCAAAt
HvGSK1.1	P$GT1_Q6	GT1	1337	1344	1	1	1	GTAAAta
HvGSK1.1	P$WRKY48_01	WRKY48	1341	1350	1	1	0.865	atatAACAA
HvGSK1.1	P$ATMYB15_Q2	ATMYB15	1344	1350	1	1	1	TAACAa
HvGSK1.1	P$ALFIN1_Q2	ALFIN1	1361	1376	1	1	0.917	caatttGTGGGggaa
HvGSK1.1	P$ARALY897773_01	ARALY897773	1370	1380	1	1	0.911	ggggaACCAC
HvGSK1.1	P$AT4G36620_01	AT4G36620	1371	1379	1	1	0.865	gggAACCA
HvGSK1.1	P$PBF_Q2	BF	1385	1391	1	1	0.958	cAAAGG
HvGSK1.1	P$RAV1_01	RAV1	1397	1409	1	1	0.966	gagCAACAtttt
HvGSK1.1	P$SEP3_01	wrz-03	1402	1413	1	1	0.925	acatttTTTTGg
HvGSK1.1	P$SQUA_01	SQUA	1403	1413	1	1	0.908	catTTTTTgg
HvGSK1.1	P$SOC1_01	SOC1	1425	1440	1	1	0.879	atttccagtTTTTGg
HvGSK1.1	P$AGL15_03	AGL15	1426	1441	1	1	0.874	TTTCCagtttttggc
HvGSK1.1	P$SEP3_01	wrz-03	1429	1440	1	1	0.872	ccagtTTTTGg
HvGSK1.1	P$SQUA_01	SQUA	1430	1440	1	1	0.887	cagTTTTTgg
HvGSK1.1	P$PBF_Q2	BF	1458	1464	1	1	0.958	cAAAGG
HvGSK1.1	P$GT1_Q6	GT1	1502	1509	1	1	1	GTGAAta
HvGSK1.1	P$ATHB6_01	ATHB6	1503	1512	1	1	0.972	tgAATAAta
HvGSK1.1	P$ATHB5_04	ATHB5	1503	1514	1	1	0.882	tgAATAAtagc
HvGSK1.1	P$ATHB1_03	ATHB1	1503	1514	1	1	0.88	tgAATAAtagc
HvGSK1.1	P$ATHB16_01	ATHB16	1504	1512	1	1	0.862	gAATAAta
HvGSK1.1	P$CRF2_01	CRF2	1510	1518	1	1	0.884	taGCCGCa
HvGSK1.1	P$ERF098_01	ERF098	1510	1518	1	1	0.897	taGCCGCa
HvGSK1.1	P$ERF7_02	ERF7	1511	1521	1	1	0.95	aGCCGCattg
HvGSK1.1	P$AT4G36620_01	AT4G36620	1531	1539	1	1	0.898	tcaAACCA
HvGSK1.1	P$HSFA2_01	HSFA2	1562	1568	1	1	0.922	CCAAAt
HvGSK1.1	P$E2FA_02	E2FA	1566	1576	1	1	0.99	atgCGCCAtc
HvGSK1.1	P$BZR1_01	BZR1	1594	1600	1	1	0.915	CGTGCa
HvGSK1.1	P$PCF5_01	CF5	1603	1613	1	1	0.89	agGGTCCctc
HvGSK1.1	P$MYB24_01	MYB24	1611	1620	1	1	0.961	tcaTTAGGt
HvGSK1.1	P$MYB131_01	MYB131	1611	1622	1	1	0.952	tcaTTAGGtaa
HvGSK1.1	P$MYB3_01	MYB3	1612	1623	1	1	0.877	catTAGGTaat
HvGSK1.1	P$MYB4_01	MYB4	1613	1621	1	1	0.875	atTAGGTa
HvGSK1.1	P$WRKY11_Q2	WRKY11	1652	1660	1	1	0.999	aTTGACca
HvGSK1.1	P$ZAP1_01	ZAP1	1653	1663	1	1	0.948	TTGACcaagc
HvGSK1.1	P$O2_Q2	O2	1688	1701	1	1	0.924	catacGACGTaca
HvGSK1.1	P$ATSPL3_01	ATSPL3	1690	1706	1	1	0.955	tacgaCGTACatggtg
HvGSK1.1	P$SPL14_03	SPL14	1693	1704	1	1	0.855	gaCGTACatgg
HvGSK1.1	P$CBNAC_01	CBNAC	1705	1711	1	1	0.979	gTGCTT
HvGSK1.1	P$CBNAC_02	CBNAC	1705	1721	1	1	0.928	gTGCTTaccttgtaac
HvGSK1.1	P$BHLH112_01	BHLH112	1720	1729	1	1	0.916	cctACTTGa
HvGSK1.1	P$MYBAS1_01	MYBAS1	1759	1770	1	1	0.963	caCCAACgcca
HvGSK1.1	P$AT5G54070_01	AT5G54070	1761	1767	1	1	1	cCAACG
HvGSK1.1	P$GAMYB_Q2	GAMYB	1764	1777	1	1	0.939	acgccACAACaca
HvGSK1.1	P$RAV1_01	RAV1	1767	1779	1	1	0.958	ccaCAACAcaac
HvGSK1.1	P$GAMYB_Q2	GAMYB	1769	1782	1	1	0.877	acaacACAACaat
HvGSK1.1	P$RAV1_01	RAV1	1772	1784	1	1	0.928	acaCAACAatgt
HvGSK1.1	P$ARF8_01	ARF8	1779	1788	1	1	0.955	aaTGTCGat
HvGSK1.1	P$DRE1C_01	DRE1C	1780	1788	1	1	0.862	ATGTCgat
HvGSK1.1	P$O2_03	O2	1785	1795	1	1	0.93	GATGAactgg
HvGSK1.1	P$MYBAS1_01	MYBAS1	1824	1835	1	1	0.982	ccCCAACtaac
HvGSK1.1	P$C1_Q2	C1	1826	1837	1	1	0.973	ccAACTAacga
HvGSK1.1	P$WEREWOLF_Q2_01	WEREWOLF	1828	1837	1	1	0.949	aACTAAcga
HvGSK1.1	P$MYBAS1_01	MYBAS1	1828	1839	1	1	0.978	aaCTAACgatc
HvGSK1.1	P$SPL14_02	SPL14	1835	1854	1	1	0.913	gatccgCCGTAccaaacac
HvGSK1.1	P$ERF112_02	ERF112	1837	1847	1	1	0.936	tcCGCCGtac

HvGSK1.1	P$ATSPL3_01	ATSPL3	1837	1853	1	1	0.95	tccgcCGTACcaaaca
HvGSK1.1	P$AT3G63350_01	AT3G63350	1838	1844	1	1	0.866	CCGCCg
HvGSK1.1	P$CRF4_01	CRF4	1838	1846	1	1	0.869	cCGCCGta
HvGSK1.1	P$ERF4_04	ERF4	1838	1846	1	1	0.881	cCGCCGta
HvGSK1.1	P$ERF069_01	ERF069	1838	1847	1	1	0.99	cCGCCGtac
HvGSK1.1	P$ERF11_01	ERF11	1838	1848	1	1	0.973	cCGCCGtacc
HvGSK1.1	P$ERF8_01	ERF8	1839	1849	1	1	0.949	CGCCGtacca
HvGSK1.1	P$ANAC042_01	ANAC042	1839	1859	1	1	0.957	cGCCGTaccaaacacgccct
HvGSK1.1	P$ANAC094_01	ANAC094	1839	1857	1	1	0.992	cGCCGTaccaaacacgcc
HvGSK1.1	P$SPL3_01	SPL3	1840	1848	1	1	0.97	gCCGTAcc
HvGSK1.1	P$SPL14_03	SPL14	1840	1851	1	1	0.96	gcCGTACcaaa
HvGSK1.1	P$SPL14_01	SPL14	1841	1848	1	1	0.944	CCGTAcc
HvGSK1.1	P$HSFA2_01	HSFA2	1846	1852	1	1	0.941	CCAAAc
HvGSK1.1	P$MYB1L_01	MYB1L	1853	1863	1	1	0.961	cgCCCTAtca
HvGSK1.1	P$TRB2_01	TRB2	1853	1861	1	1	0.973	cgCCCTAt
HvGSK1.1	P$LEC2_01	LEC2	1870	1881	1	1	0.934	caCATGCttca
HvGSK1.1	P$CBNAC_01	CBNAC	1873	1879	1	1	0.968	aTGCTT
HvGSK1.1	P$CBNAC_02	CBNAC	1873	1889	1	1	0.87	aTGCTTcatcactatc
HvGSK1.1	P$SPF1_Q2	SPF1	1887	1897	1	1	0.924	tcATAGTaag
HvGSK1.1	P$AT3G51080_01	AT3G51080	1911	1918	1	1	0.893	GGAAAat
HvGSK1.1	P$GAMYB_Q2	GAMYB	1919	1932	1	1	0.926	catctACAACaaa
HvGSK1.1	P$ATHSFA1D_01	ATHSFA1D	1921	1927	1	1	0.941	tCTACA
HvGSK1.1	P$RAV1_01	RAV1	1922	1934	1	1	0.948	ctaCAACAaaca
HvGSK1.1	P$LEC2_01	LEC2	1933	1944	1	1	0.936	atCATGCtata
HvGSK1.1	P$PDF2_01	DF2	1938	1949	1	1	0.942	gctaTAAATga
HvGSK1.1	P$AT4G36620_01	AT4G36620	1945	1953	1	1	0.98	atgAACCA
HvGSK1.1	P$LIM1_01	LIM1	1950	1962	1	1	0.91	CCACCaatagat
HvGSK1.1	P$RIN_01	RIN	1950	1960	1	1	0.898	ccaccAATAG
HvGSK1.1	P$ARR18_01	ARR18	1954	1967	1	1	0.895	caatAGATAgaaa
HvGSK1.1	P$BPC1_Q2	BPC1	1962	1968	1	1	0.99	AGAAAt
HvGSK1.1	P$HAT1_01	HAT1	1963	1973	1	1	0.853	gaAATCAcac
HvGSK1.1	P$ATHB7_01	ATHB7	1982	1992	1	1	0.873	caAATCAaca
HvGSK1.1	P$HAT1_01	HAT1	1982	1992	1	1	0.867	caAATCAaca
HvGSK1.1	P$RAV1_01	RAV1	1984	1996	1	1	0.953	aatCAACAcaag
HvGSK1.1	P$AT1G19490_01	AT1G19490	2001	2010	1	1	0.9	GGTTTaact
HvGSK1.1	P$HSFA4A_01	HSFA4A	2019	2025	1	1	1	aCTATT
HvGSK1.1	P$EDT1_01	EDT1	2020	2030	1	1	0.851	ctaTTAATct
HvGSK1.1	P$LEC2_01	LEC2	2037	2048	1	1	0.932	gaCATGCttga
HvGSK1.1	P$CBNAC_01	CBNAC	2040	2046	1	1	0.968	aTGCTT
HvGSK1.1	P$MYBAS1_01	MYBAS1	2050	2061	1	1	0.951	atCTAACccta
HvGSK1.1	P$MYB1L_01	MYB1L	2054	2064	1	1	0.992	aaCCCTAcca
HvGSK1.1	P$TRB2_01	TRB2	2054	2062	1	1	0.957	aaCCCTAc
HvGSK1.1	P$P_01		2056	2065	1	1	0.951	ccCTACCac
HvGSK1.1	P$AT4G36620_01	AT4G36620	2069	2077	1	1	0.903	tgcAACCA
HvGSK1.1	P$GAMYB_01	GAMYB	2071	2079	1	1	0.901	CAACCaca
HvGSK1.1	P$ATHB7_01	ATHB7	2084	2094	1	1	0.948	gcAATCAacg
HvGSK1.1	P$HAT1_01	HAT1	2084	2094	1	1	0.883	gcAATCAacg
HvGSK1.1	P$AT5G54070_01	AT5G54070	2088	2094	1	1	0.91	tCAACG
HvGSK1.1	P$ZAT1_01	ZAT1	2098	2109	1	1	0.856	gagttcACAAA
HvGSK1.1	P$AT4G36620_01	AT4G36620	2104	2112	1	1	0.902	acaAACCA
HvGSK1.1	P$REF6_01	REF6	2106	2117	1	1	0.862	aaacCAGAGac
HvGSK1.1	P$ATHB7_01	ATHB7	2116	2126	1	1	0.882	ctAATCAacc
HvGSK1.1	P$HAT1_01	HAT1	2116	2126	1	1	0.877	ctAATCAacc
HvGSK1.1	P$AT4G36620_01	AT4G36620	2119	2127	1	1	1	atcAACCA
HvGSK1.1	P$GAMYB_01	GAMYB	2121	2129	1	1	0.878	CAACCatg
HvGSK1.1	P$LEC2_01	LEC2	2123	2134	1	1	0.989	acCATGCatct
HvGSK1.1	P$FUS3_Q2	FUS3	2124	2135	1	1	0.901	cCATGCatcta
HvGSK1.1	P$ATHSFA1D_01	ATHSFA1D	2131	2137	1	1	0.941	tCTACA
HvGSK1.1	P$MYB1L_01	MYB1L	2140	2150	1	1	1	aaCCCTAact
HvGSK1.1	P$TRB2_01	TRB2	2140	2148	1	1	0.973	aaCCCTAa
HvGSK1.1	P$MYBAS1_01	MYBAS1	2142	2153	1	1	0.973	ccCTAACtgca
HvGSK1.1	P$GATA9_01	GATA9	2154	2165	1	1	0.984	ctcAGATCtag
HvGSK1.1	P$AGP1_01	AGP1	2155	2165	1	1	1	tcAGATCtag
HvGSK1.1	P$GATA10_01	GATA10	2156	2164	1	1	0.916	cAGATCta
HvGSK1.1	P$GATA11_01	GATA11	2156	2164	1	1	0.939	caGATCTa
HvGSK1.1	P$GATA8_01	GATA8	2156	2165	1	1	1	caGATCTag
HvGSK1.1	P$ARR10_01	ARR10	2157	2164	1	1	0.913	AGATCta
HvGSK1.1	P$BPC1_Q2	BPC1	2180	2186	1	1	1	AGAAAg
HvGSK1.1	P$PBF_Q2	BF	2181	2187	1	1	0.965	gAAAGG
HvGSK1.1	P$AT5G66730_01	AT5G66730	2182	2193	1	1	0.864	aaaggGGGATt
HvGSK1.1	P$PBF_Q2	BF	2200	2206	1	1	0.958	cAAAGG
HvGSK1.1	P$TEIL_01	TEIL	2212	2220	1	1	0.94	ATGTAgct
HvGSK1.1	P$AT1G53910_01	AT1G53910	2236	2246	1	1	0.853	cGGCCGtaga
HvGSK1.1	P$SPL14_01	SPL14	2239	2246	1	1	0.851	CCGTAga
HvGSK1.1	P$GATA9_01	GATA9	2243	2254	1	1	0.884	agaAGATCatc
HvGSK1.1	P$AGP1_01	AGP1	2244	2254	1	1	0.851	gaAGATCatc
HvGSK1.1	P$ARR10_01	ARR10	2246	2253	1	1	0.913	AGATCat
HvGSK1.1	P$ERF112_02	ERF112	2251	2261	1	1	0.905	atCGCCGgtc

HvGSK1.1	P$ERF4_04	ERF4	2252	2260	1	1	0.855	tCGCCGgt
HvGSK1.1	P$ERF069_01	ERF069	2252	2261	1	1	0.988	tCGCCGgtc
HvGSK1.1	P$ERF11_01	ERF11	2252	2262	1	1	0.962	tCGCCGgtcg
HvGSK1.1	P$ERF8_01	ERF8	2253	2263	1	1	0.941	CGCCGgtcga
HvGSK1.1	P$CBF1_02	CBF1	2267	2277	1	1	0.853	ggaCCGCCca
HvGSK1.1	P$AT5G46350_01	AT5G46350	2269	2278	1	1	0.851	ACCGCccat
HvGSK1.1	P$AT3G01030_01	AT3G01030	2269	2278	1	1	0.857	ACCGCccat
HvGSK1.1	P$AT3G63350_01	AT3G63350	2270	2276	1	1	1	CCGCCc
HvGSK1.1	P$LEC2_01	LEC2	2273	2284	1	1	0.934	ccCATGCtgtg
HvGSK1.1	P$TCP24_01	TCP24	2279	2291	1	1	0.898	ctgtGGACCact
HvGSK1.1	P$ARALY897773_01	ARALY897773	2280	2290	1	1	0.903	tgtggACCAC
HvGSK1.1	P$TCP4_01	TCP4	2282	2290	1	1	0.951	tGGACCac
HvGSK1.1	P$TCP5_01	TCP5	2282	2290	1	1	0.866	tGGACCac
HvGSK1.1	P$ARALY496250_03	ARALY496250	2282	2290	1	1	0.877	tGGACCac
HvGSK1.1	P$CBF2_02	CBF2	2290	2301	1	1	0.854	tGGCCGatgaa
HvGSK1.1	P$AT1G53910_01	AT1G53910	2290	2300	1	1	0.886	tGGCCGatga
HvGSK1.1	P$AT2G20350_01	AT2G20350	2290	2300	1	1	0.952	tgGCCGAtga
HvGSK1.1	P$RRTF1_02	RRTF1	2303	2313	1	1	0.877	tgCGCCGctt
HvGSK1.1	P$RAP26_03	RAP26	2303	2313	1	1	0.854	tgCGCCGctt
HvGSK1.1	P$RAP210_04	RAP210	2303	2313	1	1	0.906	tgCGCCGctt
HvGSK1.1	P$ERF112_02	ERF112	2303	2313	1	1	0.929	tgCGCCGctt
HvGSK1.1	P$CRF4_01	CRF4	2304	2312	1	1	0.929	gCGCCGct
HvGSK1.1	P$ERF4_04	ERF4	2304	2312	1	1	0.897	gCGCCGct
HvGSK1.1	P$ERF069_01	ERF069	2304	2313	1	1	0.994	gCGCCGctt
HvGSK1.1	P$ERF11_01	ERF11	2304	2314	1	1	0.972	gCGCCGctta
HvGSK1.1	P$AT1G77200_01	AT1G77200	2304	2314	1	1	0.899	gcGCCGCtta
HvGSK1.1	P$ATERF14_01	ATERF14	2304	2314	1	1	0.86	gcGCCGCtta
HvGSK1.1	P$DREBIII3_01	DREBIII3	2304	2314	1	1	0.901	gcGCCGCtta
HvGSK1.1	P$DREBIII2_01	DREBIII2	2304	2314	1	1	0.898	gcGCCGCtta
HvGSK1.1	P$ERF4_02	ERF4	2304	2314	1	1	0.859	gcGCCGCtta
HvGSK1.1	P$DREBIII1_01	DREBIII1	2304	2314	1	1	0.901	gcGCCGCtta
HvGSK1.1	P$AT2G44940_01	AT2G44940	2304	2314	1	1	0.88	gcGCCGCtta
HvGSK1.1	P$ERF1B_03	ERF1B	2304	2314	1	1	0.857	gcGCCGCtta
HvGSK1.1	P$DBF2_01	DBF2	2304	2314	1	1	0.913	gcGCCGCtta
HvGSK1.1	P$AT5G43410_01	AT5G43410	2304	2314	1	1	0.866	gcGCCGCtta
HvGSK1.1	P$TINY2_02	TINY2	2304	2314	1	1	0.888	gcGCCGCtta
HvGSK1.1	P$AT3G16280_01	AT3G16280	2304	2314	1	1	0.907	gcGCCGCtta
HvGSK1.1	P$CRF2_01	CRF2	2304	2312	1	1	0.949	gcGCCGCt
HvGSK1.1	P$ERF098_01	ERF098	2304	2312	1	1	0.895	gcGCCGCt
HvGSK1.1	P$ERF8_01	ERF8	2305	2315	1	1	0.951	CGCCGcttac
HvGSK1.1	P$ERF7_02	ERF7	2305	2315	1	1	0.948	cGCCGCttac
HvGSK1.1	P$RAV1_02	RAV1	2310	2322	1	1	0.912	cttACCTGctcg
HvGSK1.1	P$DREB1A_04	DREB1A	2319	2329	1	1	0.965	tcGTCGGtgt
HvGSK1.1	P$ERF039_01	ERF039	2319	2329	1	1	0.993	tcGTCGGtgt
HvGSK1.1	P$PHYPA182268_05	HYPA182268	2319	2329	1	1	0.99	tcGTCGGtgt
HvGSK1.1	P$PHYPA64121_06	HYPA64121	2319	2332	1	1	0.864	tcgTCGGTgtcga
HvGSK1.1	P$RAP21_02	RAP21	2319	2332	1	1	0.95	tcgtCGGTGtcga
HvGSK1.1	P$ERF043_01	ERF043	2320	2328	1	1	0.892	cGTCGGtg
HvGSK1.1	P$PHYPA173530_04	HYPA173530	2320	2328	1	1	0.957	cGTCGGtg
HvGSK1.1	P$PHYPA28324_10	HYPA28324	2320	2328	1	1	0.98	cGTCGGtg
HvGSK1.1	P$ARF8_01	ARF8	2324	2333	1	1	0.952	ggTGTCGat
HvGSK1.1	P$CMTA2_01	CMTA2	2330	2339	1	1	0.992	gatgCGCGT
HvGSK1.1	P$CAMTA1_02	CAMTA1	2330	2342	1	1	0.936	gatgCGCGTcgg
HvGSK1.1	P$RRTF1_05	RRTF1	2332	2347	1	1	0.863	tgcgcgtCGGCGgga
HvGSK1.1	P$CMTA3_01	CMTA3	2333	2342	1	1	0.982	gCGCGTcgg
HvGSK1.1	P$DREB1A_04	DREB1A	2335	2345	1	1	0.968	gcGTCGGcgg
HvGSK1.1	P$ERF039_01	ERF039	2335	2345	1	1	0.984	gcGTCGGcgg
HvGSK1.1	P$PHYPA182268_05	HYPA182268	2335	2345	1	1	0.878	gcGTCGGcgg
HvGSK1.1	P$PHYPA173530_04	HYPA173530	2336	2344	1	1	0.875	cGTCGGcg
HvGSK1.1	P$PHYPA28324_10	HYPA28324	2336	2344	1	1	0.92	cGTCGGcg
HvGSK1.1	P$AT1G28160_02	AT1G28160	2336	2351	1	1	0.862	cgtCGGCGggaaagc
HvGSK1.1	P$RAP26_06	RAP26	2336	2351	1	1	0.854	cgtCGGCGggaaagc
HvGSK1.1	P$AT1G68550_03	AT1G68550	2336	2345	1	1	0.959	cgtCGGCGg
HvGSK1.1	P$E2L_Q2_01	E2L	2337	2351	1	1	0.895	gtcggCGGGAaagc
HvGSK1.1	P$E2L_Q2	E2L	2339	2346	1	1	0.928	cGGCGGg
HvGSK1.1	P$DOF2_01	DOF2	2342	2353	1	1	0.982	cgggAAAGCtc
HvGSK1.1	P$DOF3_01	DOF3	2342	2353	1	1	0.974	cgggAAAGCtc
HvGSK1.1	P$PBF_Q2	BF	2353	2359	1	1	0.965	gAAAGG
HvGSK1.1	P$TGA1_01	TGA1	2369	2380	1	1	0.956	tggTGACGgga
HvGSK1.1	P$TGA7_01	TGA7	2370	2380	1	1	0.897	ggTGACGgga
HvGSK1.1	P$TGA5_01	TGA5	2371	2379	1	1	0.864	gTGACGgg
HvGSK1.1	P$RAP21_02	RAP21	2384	2397	1	1	0.93	ggggCGGTGaggg
HvGSK1.1	P$AT1G68550_03	AT1G68550	2416	2425	1	1	0.949	actCGGCGa
HvGSK1.1	P$AT2G41690_01	AT2G41690	2433	2439	1	1	0.978	CCGAAa
HvGSK1.1	P$ERF112_02	ERF112	2443	2453	1	1	0.921	tcCGCCGttt
HvGSK1.1	P$AT3G63350_01	AT3G63350	2444	2450	1	1	0.866	CCGCCg
HvGSK1.1	P$CRF4_01	CRF4	2444	2452	1	1	0.865	cCGCCGtt
HvGSK1.1	P$ERF4_04	ERF4	2444	2452	1	1	0.871	cCGCCGtt

HvGSK1.1	P$ERF069_01	ERF069	2444	2453	1	1	0.989	cCGCCGttt
HvGSK1.1	P$ERF11_01	ERF11	2444	2454	1	1	0.97	cCGCCGtttt
HvGSK1.1	P$ERF8_01	ERF8	2445	2455	1	1	0.945	CGCCGttttt
HvGSK1.1	P$ANAC042_01	ANAC042	2445	2465	1	1	0.976	cGCCGTtttttctacgacgc
HvGSK1.1	P$ANAC094_01	ANAC094	2445	2463	1	1	0.987	cGCCGTtttttctacgac
HvGSK1.1	P$BZR1_01	BZR1	2474	2480	1	1	0.897	CGTGCc
HvGSK1.1	P$ABI3_01	ABI3	2490	2499	1	1	0.861	tcGCATGag
HvGSK1.1	P$FUS3_01	FUS3	2491	2500	1	1	0.855	cGCATGagg
HvGSK1.1	P$CMTA2_01	CMTA2	2503	2512	1	1	0.994	gaagCGCGT
HvGSK1.1	P$CAMTA1_02	CAMTA1	2503	2515	1	1	0.973	gaagCGCGTcgc
HvGSK1.1	P$CMTA3_01	CMTA3	2506	2515	1	1	0.982	gCGCGTcgc
HvGSK1.1	P$AT2G41690_01	AT2G41690	2530	2536	1	1	1	CCGAAc
HvGSK1.1	P$HSFA4A_01	HSFA4A	2547	2553	1	1	0.91	gCTATT
HvGSK1.1	P$ARF8_01	ARF8	2550	2559	1	1	0.958	atTGTCGat
HvGSK1.1	P$WRKY11_Q2	WRKY11	2576	2584	1	1	0.978	tTTGACct
HvGSK1.1	P$ZAP1_01	ZAP1	2577	2587	1	1	0.858	TTGACctgaa
HvGSK1.1	P$RAV1_02	RAV1	2577	2589	1	1	0.911	ttgACCTGaagg
HvGSK1.1	P$CBNAC_01	CBNAC	2588	2594	1	1	0.979	gTGCTT
HvGSK1.1	P$EDT1_01	EDT1	2590	2600	1	1	0.95	gctTTAATgt
HvGSK1.1	P$GT1_01	GT1	2630	2638	1	1	0.862	ggTAACCa
HvGSK1.1	P$AT4G36620_01	AT4G36620	2630	2638	1	1	0.874	ggtAACCA
HvGSK1.1	P$HSFA2_01	HSFA2	2635	2641	1	1	0.941	CCAAAc
HvGSK1.1	P$MYBAS1_01	MYBAS1	2653	2664	1	1	0.96	tcCCAACcgaa
HvGSK1.1	P$GAMYB_01	GAMYB	2656	2664	1	1	0.948	CAACCgaa
HvGSK1.1	P$AT2G41690_01	AT2G41690	2659	2665	1	1	1	CCGAAc
HvGSK1.1	P$AT4G36620_01	AT4G36620	2659	2667	1	1	0.866	ccgAACCA
HvGSK1.1	P$SBF1_01	SBF1	2669	2683	1	1	0.854	ctgggcTTAATaga
HvGSK1.1	P$AT4G36620_01	AT4G36620	2683	2691	1	1	0.865	gggAACCA
HvGSK1.1	P$HSFA2_01	HSFA2	2688	2694	1	1	0.941	CCAAAc
HvGSK1.1	P$ABF2_01	ABF2	2688	2701	1	1	0.948	ccaaaCACGTcct
HvGSK1.1	P$O2_Q4	O2	2689	2700	1	1	0.892	caaaCACGTcc
HvGSK1.1	P$BZR1_02	BZR1	2689	2703	1	1	0.854	caaaCACGTcctaa
HvGSK1.1	P$GBP_Q6	GBP	2690	2702	1	1	0.918	aaaCACGTccta
HvGSK1.1	P$ABI5_01	ABI5	2690	2700	1	1	0.927	aaaCACGTcc
HvGSK1.1	P$ABF4_01	ABF4	2690	2702	1	1	0.91	aaaCACGTccta
HvGSK1.1	P$CPRF3_Q2	CPRF3	2691	2701	1	1	0.945	aaCACGTcct
HvGSK1.1	P$CPRF2_Q2	CPRF2	2691	2701	1	1	0.951	aaCACGTcct
HvGSK1.1	P$O2_02	O2	2691	2701	1	1	0.934	aaCACGTcct
HvGSK1.1	P$TGA1B_Q2	TGA1B	2691	2701	1	1	0.903	aaCACGTcct
HvGSK1.1	P$TGA1A_Q2	TGA1A	2691	2701	1	1	0.973	aaCACGTcct
HvGSK1.1	P$CPRF3_01	CPRF3	2691	2701	1	1	0.96	aaCACGTcct
HvGSK1.1	P$CPRF2_01	CPRF2	2691	2701	1	1	0.953	aaCACGTcct
HvGSK1.1	P$TGA1B_01	TGA1B	2691	2701	1	1	0.901	aaCACGTcct
HvGSK1.1	P$BEE2_01	BEE2	2691	2701	1	1	0.907	aaCACGTcct
HvGSK1.1	P$BIM3_01	BIM3	2691	2701	1	1	0.879	aaCACGTcct
HvGSK1.1	P$PHYPA143875_02	HYPA143875	2691	2701	1	1	0.873	aaCACGTcct
HvGSK1.1	P$SPT_01	SPT	2691	2700	1	1	0.917	aaCACGTcc
HvGSK1.1	P$RITA1_01	RITA1	2692	2699	1	1	0.963	aCACGTc
HvGSK1.1	P$OCSBF1_01	OCSBF1	2693	2698	1	1	1	CACGT
HvGSK1.1	P$TGA1A_01	TGA1A	2693	2700	1	1	0.861	cACGTCc
HvGSK1.1	P$MYBAS1_01	MYBAS1	2697	2708	1	1	0.994	tcCTAACcgcc
HvGSK1.1	P$WRKY25_01	WRKY25	2697	2706	1	1	0.92	tccTAACCg
HvGSK1.1	P$WRKY33_01	WRKY33	2697	2706	1	1	0.943	tccTAACCg
HvGSK1.1	P$AT1G29860_01	AT1G29860	2697	2706	1	1	0.89	tccTAACCg
HvGSK1.1	P$AT3G62340_01	AT3G62340	2697	2706	1	1	0.918	tccTAACCg
HvGSK1.1	P$AT1G69310_01	AT1G69310	2697	2706	1	1	0.913	tccTAACCg
HvGSK1.1	P$WRKY26_01	WRKY26	2697	2706	1	1	0.942	tccTAACCg
HvGSK1.1	P$AT3G01030_01	AT3G01030	2702	2711	1	1	0.889	ACCGCcaca
HvGSK1.1	P$AT3G63350_01	AT3G63350	2703	2709	1	1	0.882	CCGCCa
HvGSK1.1	P$ASR1_01	ASR1	2741	2746	1	1	1	ACCCA
HvGSK1.1	P$HSFA2_01	HSFA2	2743	2749	1	1	0.922	CCAAAt
HvGSK1.1	P$SPL14_02	SPL14	2745	2764	1	1	0.925	aaatctCCGTActcaagac
HvGSK1.1	P$ATSPL3_01	ATSPL3	2747	2763	1	1	0.945	atctcCGTACtcaaga
HvGSK1.1	P$SPL3_01	SPL3	2750	2758	1	1	0.998	tCCGTAct
HvGSK1.1	P$SPL14_03	SPL14	2750	2761	1	1	0.986	tcCGTACtcaa
HvGSK1.1	P$SPL14_01	SPL14	2751	2758	1	1	0.944	CCGTAct
HvGSK1.1	P$AMS_01	AMS	2766	2776	1	1	0.99	agCATGTgtc
HvGSK1.1	P$MYB24_01	MYB24	2781	2790	1	1	0.932	cacTTAGGt
HvGSK1.1	P$MYB131_01	MYB131	2781	2792	1	1	0.913	cacTTAGGtct
HvGSK1.1	P$CBNAC_01	CBNAC	2800	2806	1	1	0.973	cTGCTT
HvGSK1.1	P$HAT1_01	HAT1	2815	2825	1	1	0.851	aaAATCAgct
HvGSK1.1	P$DOF43_01	DOF43	2835	2846	1	1	0.885	aaatccACTTT
HvGSK1.1	P$ATMYB77_01	ATMYB77	2844	2857	1	1	0.858	ttagaaCAGTTtc
HvGSK1.1	P$AT4G36620_01	AT4G36620	2867	2875	1	1	0.903	tgcAACCA
HvGSK1.1	P$GAMYB_01	GAMYB	2869	2877	1	1	0.869	CAACCatt
HvGSK1.1	P$PBF_01	BF	2876	2887	1	1	0.963	ttcAAAAGaat
HvGSK1.1	P$DOF_Q2	DOF	2876	2887	1	1	0.935	ttcAAAAGaat
HvGSK1.1	P$CDF2_01	CDF2	2877	2887	1	1	0.946	tcAAAAGaat

HvGSK1.1	P$CDF3_01	CDF3	2878	2887	1	1	0.968	cAAAAGaat
HvGSK1.1	P$WRKY11_Q2	WRKY11	2888	2896	1	1	0.927	tTTGACtc
HvGSK1.1	P$AT4G04450_01	AT4G04450	2897	2906	1	1	0.866	catTTAGCt
HvGSK1.1	P$AT3G51080_01	AT3G51080	2920	2927	1	1	0.893	GGAAAat
HvGSK1.1	P$GT1_Q6	GT1	2930	2937	1	1	0.971	GTAAAaa
HvGSK1.1	P$GATA15_01	GATA15	2935	2944	1	1	0.999	aaTGATCta
HvGSK1.1	P$GATA8_01	GATA8	2936	2945	1	1	0.986	atGATCTat
HvGSK1.1	P$HSFA4A_01	HSFA4A	2940	2946	1	1	0.914	tCTATT
HvGSK1.1	P$PEND_01	END	2961	2969	1	1	0.892	tAAGAAac
HvGSK1.1	P$BPC1_Q2	BPC1	2963	2969	1	1	0.99	AGAAAc
HvGSK1.1	P$ALFIN1_Q2	ALFIN1	2992	3007	1	1	0.879	gcagtgGTGGGtaat
HvGSK1.1	P$HSFA2_01	HSFA2	3013	3019	1	1	0.922	CCAAAt
HvGSK1.1	P$HMG1_01	HMG1	3092	3101	1	1	0.924	GTTGTtttg
HvGSK1.1	P$WRKY11_01	WRKY11	3126	3140	1	1	0.865	tcgtTTGACttaca
HvGSK1.1	P$WRKY11_Q2	WRKY11	3129	3137	1	1	0.931	tTTGACtt
HvGSK1.1	P$RAV1_02	RAV1	3150	3162	1	1	0.907	cggACCTGaatt
HvGSK1.1	P$REF6_01	REF6	3166	3177	1	1	0.992	gaaaCAGAGca
HvGSK1.1	P$SPL14_02	SPL14	3212	3231	1	1	0.925	aaatctCCGTActcaagac
HvGSK1.1	P$ATSPL3_01	ATSPL3	3214	3230	1	1	0.945	atctcCGTACtcaaga
HvGSK1.1	P$SPL3_01	SPL3	3217	3225	1	1	0.998	tCCGTAct
HvGSK1.1	P$SPL14_03	SPL14	3217	3228	1	1	0.986	tcCGTACtcaa
HvGSK1.1	P$SPL14_01	SPL14	3218	3225	1	1	0.944	CCGTAct
HvGSK1.1	P$AMS_01	AMS	3233	3243	1	1	0.99	agCATGTgtc
HvGSK1.1	P$PCF2_01	CF2	3257	3267	1	1	0.997	ctggcCCCAC
HvGSK1.1	P$TCP19_01	TCP19	3257	3267	1	1	0.985	ctggcCCCAC
HvGSK1.1	P$TCP20L_01	TCP20L	3258	3267	1	1	0.991	tggcCCCAC
HvGSK1.1	P$OSI_01	OSI	3259	3267	1	1	0.959	ggcCCCAC
HvGSK1.1	P$TCP20_02	TCP20	3259	3269	1	1	0.998	ggcCCCACcc
HvGSK1.1	P$ARALY495258_02	ARALY495258	3259	3267	1	1	1	ggcCCCAC
HvGSK1.1	P$ARALY493022_04	ARALY493022	3259	3267	1	1	0.973	ggcCCCAC
HvGSK1.1	P$ARALY484486_05	ARALY484486	3259	3267	1	1	1	ggcCCCAC
HvGSK1.1	P$HSF3_01	HSF3	3275	3281	1	1	0.94	tCGGGG
HvGSK1.1	P$AT4G12750_01	AT4G12750	3306	3316	1	1	0.874	gagACCGAtg
HvGSK1.1	P$EDF3_02	EDF3	3307	3316	1	1	0.943	aGACCGatg
HvGSK1.1	P$RAV2_01	RAV2	3307	3316	1	1	0.979	agACCGAtg
HvGSK1.1	P$GATA15_01	GATA15	3321	3330	1	1	0.999	caTGATCtc
HvGSK1.1	P$GATA8_01	GATA8	3322	3331	1	1	0.979	atGATCTca
HvGSK1.1	P$AT3G60580_01	AT3G60580	3328	3335	1	1	0.851	tcATCCC
HvGSK1.1	P$AT1G53910_01	AT1G53910	3336	3346	1	1	0.882	gGGCCGctgt
HvGSK1.1	P$DREBI5_01	DREBI5	3336	3346	1	1	0.886	ggGCCGCtgt
HvGSK1.1	P$AT2G47520_01	AT2G47520	3336	3346	1	1	0.852	ggGCCGCtgt
HvGSK1.1	P$AT1G77200_01	AT1G77200	3336	3346	1	1	0.899	ggGCCGCtgt
HvGSK1.1	P$ATERF14_01	ATERF14	3336	3346	1	1	0.884	ggGCCGCtgt
HvGSK1.1	P$DREBIII3_01	DREBIII3	3336	3346	1	1	0.88	ggGCCGCtgt
HvGSK1.1	P$DREBIII2_01	DREBIII2	3336	3346	1	1	0.878	ggGCCGCtgt
HvGSK1.1	P$ERF4_02	ERF4	3336	3346	1	1	0.86	ggGCCGCtgt
HvGSK1.1	P$DREBIII1_01	DREBIII1	3336	3346	1	1	0.88	ggGCCGCtgt
HvGSK1.1	P$DREBII1_01	DREBII1	3336	3346	1	1	0.885	ggGCCGCtgt
HvGSK1.1	P$AT2G44940_01	AT2G44940	3336	3346	1	1	0.89	ggGCCGCtgt
HvGSK1.1	P$DBF2_01	DBF2	3336	3346	1	1	0.918	ggGCCGCtgt
HvGSK1.1	P$CBF16_01	CBF16	3336	3346	1	1	0.88	ggGCCGCtgt
HvGSK1.1	P$CBF17_01	CBF17	3336	3346	1	1	0.886	ggGCCGCtgt
HvGSK1.1	P$CBF_01	CBF	3336	3346	1	1	0.889	ggGCCGCtgt
HvGSK1.1	P$AT3G61630_01	AT3G61630	3336	3346	1	1	0.857	ggGCCGCtgt
HvGSK1.1	P$AT5G43410_01	AT5G43410	3336	3346	1	1	0.889	ggGCCGCtgt
HvGSK1.1	P$TINY2_02	TINY2	3336	3346	1	1	0.932	ggGCCGCtgt
HvGSK1.1	P$AT3G16280_01	AT3G16280	3336	3346	1	1	0.909	ggGCCGCtgt
HvGSK1.1	P$CRF2_01	CRF2	3336	3344	1	1	0.887	ggGCCGCt
HvGSK1.1	P$ERF098_01	ERF098	3336	3344	1	1	0.893	ggGCCGCt
HvGSK1.1	P$ERF7_02	ERF7	3337	3347	1	1	0.938	gGCCGCtgtc
HvGSK1.1	P$ARF8_01	ARF8	3341	3350	1	1	0.955	gcTGTCGag
HvGSK1.1	P$UIF1_01	UIF1	3351	3361	1	1	0.994	caaGATTCgt
HvGSK1.1	P$DREB1A_04	DREB1A	3357	3367	1	1	0.966	tcGTCGGtga
HvGSK1.1	P$ERF039_01	ERF039	3357	3367	1	1	0.994	tcGTCGGtga
HvGSK1.1	P$PHYPA182268_05	HYPA182268	3357	3367	1	1	0.99	tcGTCGGtga
HvGSK1.1	P$PHYPA64121_06	HYPA64121	3357	3370	1	1	0.864	tcgTCGGTgaatt
HvGSK1.1	P$RAP21_02	RAP21	3357	3370	1	1	0.947	tcgtCGGTGaatt
HvGSK1.1	P$ERF043_01	ERF043	3358	3366	1	1	0.892	cGTCGGtg
HvGSK1.1	P$PHYPA173530_04	HYPA173530	3358	3366	1	1	0.957	cGTCGGtg
HvGSK1.1	P$PHYPA28324_10	HYPA28324	3358	3366	1	1	0.98	cGTCGGtg
HvGSK1.1	P$AT5G04240_01	AT5G04240	3378	3384	1	1	0.938	tGGCAC
HvGSK1.1	P$ASR1_01	ASR1	3382	3387	1	1	1	ACCCA
HvGSK1.1	P$MYB89_01	MYB89	3396	3407	1	1	0.861	ttTACCGcaca
HvGSK1.1	P$AT5G46350_01	AT5G46350	3399	3408	1	1	0.999	ACCGCacag
HvGSK1.1	P$ABF2_01	ABF2	3419	3432	1	1	0.962	agttaCACGTagc
HvGSK1.1	P$O2_Q4	O2	3420	3431	1	1	0.906	gttaCACGTag
HvGSK1.1	P$GBP_Q6	GBP	3421	3433	1	1	0.964	ttaCACGTagca
HvGSK1.1	P$GBF_Q2	GBF	3421	3433	1	1	0.891	ttaCACGTagca

HvGSK1.1	P$ABI5_01	ABI5	3421	3431	1	1	0.938	ttaCACGTag
HvGSK1.1	P$ABF4_01	ABF4	3421	3433	1	1	0.934	ttaCACGTagca
HvGSK1.1	P$EMBP1_Q2	EMBP1	3422	3432	1	1	0.864	taCACGTagc
HvGSK1.1	P$CPRF3_Q2	CPRF3	3422	3432	1	1	0.926	taCACGTagc
HvGSK1.1	P$CPRF2_Q2	CPRF2	3422	3432	1	1	0.946	taCACGTagc
HvGSK1.1	P$O2_02	O2	3422	3432	1	1	0.963	taCACGTagc
HvGSK1.1	P$TGA1B_Q2	TGA1B	3422	3432	1	1	0.912	taCACGTagc
HvGSK1.1	P$TGA1A_Q2	TGA1A	3422	3432	1	1	0.964	taCACGTagc
HvGSK1.1	P$CG1_Q6	CG1	3422	3433	1	1	0.863	taCACGTagca
HvGSK1.1	P$CPRF1_01	CPRF1	3422	3432	1	1	0.853	taCACGTagc
HvGSK1.1	P$CPRF3_01	CPRF3	3422	3432	1	1	0.929	taCACGTagc
HvGSK1.1	P$CPRF2_01	CPRF2	3422	3432	1	1	0.947	taCACGTagc
HvGSK1.1	P$TGA1B_01	TGA1B	3422	3432	1	1	0.88	taCACGTagc
HvGSK1.1	P$BEE2_01	BEE2	3422	3432	1	1	0.915	taCACGTagc
HvGSK1.1	P$BIM2_01	BIM2	3422	3432	1	1	0.852	taCACGTagc
HvGSK1.1	P$BIM3_01	BIM3	3422	3432	1	1	0.887	taCACGTagc
HvGSK1.1	P$PHYPA143875_02	HYPA143875	3422	3432	1	1	0.875	taCACGTagc
HvGSK1.1	P$SPT_01	SPT	3422	3431	1	1	0.914	taCACGTag
HvGSK1.1	P$GBF1F_Q2	GBF1F	3422	3433	1	1	0.875	taCACGTagca
HvGSK1.1	P$RITA1_01	RITA1	3423	3430	1	1	0.974	aCACGTa
HvGSK1.1	P$OCSBF1_01	OCSBF1	3424	3429	1	1	1	CACGT
HvGSK1.1	P$RAV1_02	RAV1	3429	3441	1	1	0.98	agcACCTGctgg
HvGSK1.1	P$BPC1_Q2	BPC1	3446	3452	1	1	0.997	AGAAAa
HvGSK1.1	P$LEC2_01	LEC2	3482	3493	1	1	0.942	caCATGCtcag
HvGSK1.1	P$CBF1_01	CBF1	3554	3564	1	1	0.98	cTGCCGccca
HvGSK1.1	P$ERF019_01	ERF019	3554	3564	1	1	0.974	cTGCCGccca
HvGSK1.1	P$DREB6_01	DREB6	3554	3564	1	1	0.964	cTGCCGccca
HvGSK1.1	P$DREBIII4_01	DREBIII4	3554	3564	1	1	0.853	cTGCCGccca
HvGSK1.1	P$JERF3_01	JERF3	3554	3564	1	1	0.954	cTGCCGccca
HvGSK1.1	P$DREB1_01	DREB1	3554	3564	1	1	0.963	cTGCCGccca
HvGSK1.1	P$CEF1_01	CEF1	3554	3564	1	1	0.954	cTGCCGccca
HvGSK1.1	P$JERF1_01	JERF1	3554	3564	1	1	0.989	cTGCCGccca
HvGSK1.1	P$CBF1_03	CBF1	3554	3564	1	1	0.98	cTGCCGccca
HvGSK1.1	P$AT1G71450_01	AT1G71450	3554	3564	1	1	0.999	cTGCCGccca
HvGSK1.1	P$DREB1F_01	DREB1F	3554	3564	1	1	0.988	cTGCCGccca
HvGSK1.1	P$AT1G33760_01	AT1G33760	3554	3564	1	1	0.999	cTGCCGccca
HvGSK1.1	P$AT1G71520_01	AT1G71520	3554	3564	1	1	0.973	cTGCCGccca
HvGSK1.1	P$AT4G28140_01	AT4G28140	3554	3564	1	1	0.919	cTGCCGccca
HvGSK1.1	P$DREB1E_02	DREB1E	3554	3564	1	1	1	cTGCCGccca
HvGSK1.1	P$ORA47_01	ORA47	3554	3564	1	1	0.914	cTGCCGccca
HvGSK1.1	P$DREB2F_01	DREB2F	3554	3564	1	1	0.999	cTGCCGccca
HvGSK1.1	P$RAP210_02	RAP210	3554	3564	1	1	0.978	cTGCCGccca
HvGSK1.1	P$ERF13_01	ERF13	3554	3564	1	1	0.947	ctGCCGCcca
HvGSK1.1	P$ABI4_03	ABI4	3554	3564	1	1	0.966	ctGCCGCcca
HvGSK1.1	P$WRAF1_01	WRAF1	3554	3564	1	1	0.88	ctGCCGCcca
HvGSK1.1	P$PTI5_01	TI5	3554	3564	1	1	0.956	ctGCCGCcca
HvGSK1.1	P$DREBI5_01	DREBI5	3554	3564	1	1	0.949	ctGCCGCcca
HvGSK1.1	P$AT2G47520_01	AT2G47520	3554	3564	1	1	0.962	ctGCCGCcca
HvGSK1.1	P$DREB2B_01	DREB2B	3554	3564	1	1	0.968	ctGCCGCcca
HvGSK1.1	P$CRF1_02	CRF1	3554	3564	1	1	0.971	ctGCCGCcca
HvGSK1.1	P$AT1G77200_01	AT1G77200	3554	3564	1	1	0.887	ctGCCGCcca
HvGSK1.1	P$OPBP1_01	OPBP1	3554	3564	1	1	0.945	ctGCCGCcca
HvGSK1.1	P$ATERF14_01	ATERF14	3554	3564	1	1	0.97	ctGCCGCcca
HvGSK1.1	P$DREB2A_02	DREB2A	3554	3564	1	1	1	ctGCCGCcca
HvGSK1.1	P$ERF1_02	ERF1	3554	3564	1	1	0.962	ctGCCGCcca
HvGSK1.1	P$DREBIII3_01	DREBIII3	3554	3564	1	1	0.858	ctGCCGCcca
HvGSK1.1	P$DREBIII2_01	DREBIII2	3554	3564	1	1	0.858	ctGCCGCcca
HvGSK1.1	P$ERF4_02	ERF4	3554	3564	1	1	0.975	ctGCCGCcca
HvGSK1.1	P$AT5G25390_01	AT5G25390	3554	3564	1	1	0.999	ctGCCGCcca
HvGSK1.1	P$DREBIII1_01	DREBIII1	3554	3564	1	1	0.858	ctGCCGCcca
HvGSK1.1	P$EREBP1_01	EREBP1	3554	3564	1	1	0.959	ctGCCGCcca
HvGSK1.1	P$ERF110_02	ERF110	3554	3564	1	1	0.863	ctGCCGCcca
HvGSK1.1	P$CBF3_01	CBF3	3554	3564	1	1	0.986	ctGCCGCcca
HvGSK1.1	P$DREBII1_01	DREBII1	3554	3564	1	1	0.954	ctGCCGCcca
HvGSK1.1	P$DREB1A_01	DREB1A	3554	3564	1	1	0.967	ctGCCGCcca
HvGSK1.1	P$AT2G44940_01	AT2G44940	3554	3564	1	1	0.865	ctGCCGCcca
HvGSK1.1	P$TSRF1_01	TSRF1	3554	3564	1	1	0.936	ctGCCGCcca
HvGSK1.1	P$DRF13_01	DRF13	3554	3564	1	1	0.999	ctGCCGCcca
HvGSK1.1	P$ERF4_03	ERF4	3554	3564	1	1	0.98	ctGCCGCcca
HvGSK1.1	P$ERF2_03	ERF2	3554	3564	1	1	0.968	ctGCCGCcca
HvGSK1.1	P$ERF1B_03	ERF1B	3554	3564	1	1	0.966	ctGCCGCcca
HvGSK1.1	P$DBF2_01	DBF2	3554	3564	1	1	0.863	ctGCCGCcca
HvGSK1.1	P$RAP26_02	RAP26	3554	3564	1	1	0.887	ctGCCGCcca
HvGSK1.1	P$CBF5_01	CBF5	3554	3564	1	1	0.945	ctGCCGCcca
HvGSK1.1	P$CBF16_01	CBF16	3554	3564	1	1	0.953	ctGCCGCcca
HvGSK1.1	P$DREB1A_02	DREB1A	3554	3564	1	1	0.894	ctGCCGCcca
HvGSK1.1	P$CBF17_01	CBF17	3554	3564	1	1	0.946	ctGCCGCcca
HvGSK1.1	P$BD1_01	BD1	3554	3564	1	1	0.875	ctGCCGCcca

HvGSK1.1	P$ERF1_05	ERF1	3554	3564	1	1	0.979	ctGCCGCcca
HvGSK1.1	P$AT5G25190_01	AT5G25190	3554	3564	1	1	0.989	ctGCCGCcca
HvGSK1.1	P$ERF105_01	ERF105	3554	3564	1	1	0.981	ctGCCGCcca
HvGSK1.1	P$CBF_01	CBF	3554	3564	1	1	0.951	ctGCCGCcca
HvGSK1.1	P$AT5G11190_01	AT5G11190	3554	3564	1	1	1	ctGCCGCcca
HvGSK1.1	P$AT1G68550_01	AT1G68550	3554	3564	1	1	1	ctGCCGCcca
HvGSK1.1	P$AT1G77640_01	AT1G77640	3554	3564	1	1	0.977	ctGCCGCcca
HvGSK1.1	P$AT1G28160_01	AT1G28160	3554	3564	1	1	0.887	ctGCCGCcca
HvGSK1.1	P$ERF016_01	ERF016	3554	3564	1	1	0.914	ctGCCGCcca
HvGSK1.1	P$AT3G61630_01	AT3G61630	3554	3564	1	1	0.951	ctGCCGCcca
HvGSK1.1	P$AT5G43410_01	AT5G43410	3554	3564	1	1	0.972	ctGCCGCcca
HvGSK1.1	P$RAP26L_02	RAP26L	3554	3564	1	1	0.856	ctGCCGCcca
HvGSK1.1	P$AT5G07310_01	AT5G07310	3554	3564	1	1	0.954	ctGCCGCcca
HvGSK1.1	P$AT3G16280_01	AT3G16280	3554	3564	1	1	0.913	ctGCCGCcca
HvGSK1.1	P$DREB1A_03	DREB1A	3554	3564	1	1	0.984	ctGCCGCcca
HvGSK1.1	P$AT1G49120_01	AT1G49120	3554	3564	1	1	0.987	ctGCCGCcca
HvGSK1.1	P$DREB2D_01	DREB2D	3554	3564	1	1	0.985	ctGCCGCcca
HvGSK1.1	P$LEP_01	LEP	3554	3564	1	1	0.877	ctGCCGCcca
HvGSK1.1	P$AT3G25890_01	AT3G25890	3554	3564	1	1	1	ctGCCGCcca
HvGSK1.1	P$AT4G23750_01	AT4G23750	3554	3564	1	1	0.998	ctGCCGCcca
HvGSK1.1	P$AT4G27950_01	AT4G27950	3554	3564	1	1	0.995	ctGCCGCcca
HvGSK1.1	P$RRTF1_01	RRTF1	3554	3564	1	1	0.943	ctGCCGCcca
HvGSK1.1	P$CEJ1_02	CEJ1	3554	3564	1	1	0.981	ctGCCGCcca
HvGSK1.1	P$ERF1_Q2_01	ERF1	3554	3568	1	1	0.873	ctGCCGCccattac
HvGSK1.1	P$CRF2_01	CRF2	3554	3562	1	1	0.933	ctGCCGCc
HvGSK1.1	P$ERF096_01	ERF096	3554	3564	1	1	0.983	ctGCCGCcca
HvGSK1.1	P$ERF098_01	ERF098	3554	3562	1	1	0.997	ctGCCGCc
HvGSK1.1	P$DREB2C_01	DREB2C	3554	3564	1	1	0.904	ctgCCGCCca
HvGSK1.1	P$CBF1_02	CBF1	3554	3564	1	1	0.942	ctgCCGCCca
HvGSK1.1	P$ERF1B_06	ERF1B	3555	3563	1	1	0.938	tGCCGCcc
HvGSK1.1	P$ERF7_02	ERF7	3555	3565	1	1	0.984	tGCCGCccat
HvGSK1.1	P$ERF094_01	ERF094	3555	3563	1	1	0.972	tGCCGCcc
HvGSK1.1	P$ERF2_01	ERF2	3555	3562	1	1	0.929	tgCCGCC
HvGSK1.1	P$ERF13_02	ERF13	3555	3563	1	1	0.974	tgCCGCCc
HvGSK1.1	P$AT3G63350_01	AT3G63350	3557	3563	1	1	1	CCGCCc
HvGSK1.1	P$MYB3R5_01	MYB3R5	3558	3573	1	1	0.886	cgcccattaCCGTTc
HvGSK1.1	P$MYB3R1_01	MYB3R1	3559	3574	1	1	0.916	gcccattaCCGTTca
HvGSK1.1	P$MYB3R4_01	MYB3R4	3559	3574	1	1	0.915	gcccattaCCGTTca
HvGSK1.1	P$MYB89_01	MYB89	3563	3574	1	1	0.874	atTACCGttca
HvGSK1.1	P$RAV1_02	RAV1	3570	3582	1	1	0.986	ttcACCTGcccg
HvGSK1.1	P$AT3G63350_01	AT3G63350	3579	3585	1	1	0.867	CCGCCt
HvGSK1.1	P$AT4G36620_01	AT4G36620	3590	3598	1	1	0.898	tcaAACCA
HvGSK1.1	P$HSFA2_01	HSFA2	3595	3601	1	1	0.941	CCAAAc
HvGSK1.1	P$AT4G36620_01	AT4G36620	3595	3603	1	1	0.878	ccaAACCA
HvGSK1.1	P$HSFA2_01	HSFA2	3600	3606	1	1	1	CCAAAa
HvGSK1.1	P$GATA9_01	GATA9	3612	3623	1	1	0.992	gccAGATCtac
HvGSK1.1	P$AGP1_01	AGP1	3613	3623	1	1	0.997	ccAGATCtac
HvGSK1.1	P$GATA10_01	GATA10	3614	3622	1	1	0.916	cAGATCta
HvGSK1.1	P$GATA11_01	GATA11	3614	3622	1	1	0.939	caGATCTa
HvGSK1.1	P$GATA8_01	GATA8	3614	3623	1	1	0.999	caGATCTac
HvGSK1.1	P$ARR10_01	ARR10	3615	3622	1	1	0.913	AGATCta
HvGSK1.1	P$P_01		3617	3626	1	1	0.923	atCTACCca
HvGSK1.1	P$ASR1_01	ASR1	3621	3626	1	1	1	ACCCA
HvGSK1.1	P$MYBAS1_01	MYBAS1	3621	3632	1	1	0.945	acCCAACccag
HvGSK1.1	P$GAMYB_01	GAMYB	3624	3632	1	1	0.88	CAACCcag
HvGSK1.1	P$ASR1_01	ASR1	3626	3631	1	1	1	ACCCA
HvGSK1.1	P$ASR1_01	ASR1	3634	3639	1	1	1	ACCCA
HvGSK1.1	P$ASR1_01	ASR1	3640	3645	1	1	1	ACCCA
HvGSK1.1	P$E2L_Q2	E2L	3649	3656	1	1	0.928	gGGCGGg
HvGSK1.1	P$HSF3_01	HSF3	3651	3657	1	1	0.945	gCGGGG
HvGSK1.1	P$HBP1B_Q6	HBP1B	3656	3670	1	1	0.869	ggcagcccCGTCAt
HvGSK1.1	P$HBPA1_Q6_01	HBPA1	3660	3670	1	1	0.883	gcccCGTCAt
HvGSK1.1	P$TGA2_Q2	TGA2	3663	3673	1	1	0.899	cCGTCAtttc
HvGSK1.1	P$GL15_01	GL15	3667	3677	1	1	0.859	catttCCCCC
HvGSK1.1	P$GAMYB_Q2	GAMYB	3673	3686	1	1	0.929	cccccACAACcta
HvGSK1.1	P$GAMYB_01	GAMYB	3679	3687	1	1	0.933	CAACCtac
HvGSK1.1	P$ARR1_01	ARR1	3686	3696	1	1	0.995	ctcGAATCtt
HvGSK1.1	P$ARR2_01	ARR2	3687	3697	1	1	0.998	tcgaATCTTt
HvGSK1.1	P$PHYPA64121_06	HYPA64121	3693	3706	1	1	0.874	cttTCGGTcctct
HvGSK1.1	P$TGA1_01	TGA1	3702	3713	1	1	0.93	ctcTGACGcgg
HvGSK1.1	P$TGA7_01	TGA7	3703	3713	1	1	0.857	tcTGACGcgg
HvGSK1.1	P$TGA5_01	TGA5	3704	3712	1	1	0.877	cTGACGcg
HvGSK1.1	P$GT1_Q6	GT1	3712	3719	1	1	0.971	GTGAAaa
HvGSK1.1	P$PBF_01	BF	3712	3723	1	1	0.972	gtgAAAAGaaa
HvGSK1.1	P$DOF_Q2	DOF	3712	3723	1	1	0.958	gtgAAAAGaaa
HvGSK1.1	P$CDF2_01	CDF2	3713	3723	1	1	0.95	tgAAAAGaaa
HvGSK1.1	P$CDF3_01	CDF3	3714	3723	1	1	0.971	gAAAAGaaa
HvGSK1.1	P$PI_02	I	3716	3730	1	1	0.852	aaagaaagGGAAAc

HvGSK1.1	P$BPC1_Q2	BPC1	3718	3724	1	1	1	AGAAAg
HvGSK1.1	P$PBF_Q2	BF	3719	3725	1	1	0.965	gAAAGG
HvGSK1.1	P$REF6_01	REF6	3725	3736	1	1	0.976	gaaaCAGAGag
HvGSK1.1	P$RAMOSA1_01	RAMOSA1	3725	3739	1	1	0.881	gaaacaGAGAGaga
HvGSK1.1	P$RAMOSA1_01	RAMOSA1	3727	3741	1	1	0.897	aacagaGAGAGagg
HvGSK1.1	P$RAMOSA1_01	RAMOSA1	3729	3743	1	1	0.922	cagagaGAGAGgga
HvGSK1.1	P$AT3G51080_01	AT3G51080	3746	3753	1	1	1	GGAAAaa
HvGSK1.1	P$ESR1_01	ESR1	3760	3770	1	1	0.866	tGGCCGcaca
HvGSK1.1	P$ERF3_01	ERF3	3760	3770	1	1	0.875	tGGCCGcaca
HvGSK1.1	P$ERF4_01	ERF4	3760	3770	1	1	0.874	tGGCCGcaca
HvGSK1.1	P$ERF3_02	ERF3	3760	3770	1	1	0.878	tGGCCGcaca
HvGSK1.1	P$ERF2_04	ERF2	3760	3770	1	1	0.877	tGGCCGcaca
HvGSK1.1	P$ERF3_03	ERF3	3760	3770	1	1	0.876	tGGCCGcaca
HvGSK1.1	P$ATERF12_01	ATERF12	3760	3770	1	1	0.867	tGGCCGcaca
HvGSK1.1	P$AT1G24590_01	AT1G24590	3760	3770	1	1	0.87	tGGCCGcaca
HvGSK1.1	P$ATERF11_01	ATERF11	3760	3770	1	1	0.88	tGGCCGcaca
HvGSK1.1	P$ATERF9_01	ATERF9	3760	3770	1	1	0.871	tGGCCGcaca
HvGSK1.1	P$AT1G44830_01	AT1G44830	3760	3770	1	1	0.852	tGGCCGcaca
HvGSK1.1	P$ATERF8_01	ATERF8	3760	3770	1	1	0.87	tGGCCGcaca
HvGSK1.1	P$AT1G53910_01	AT1G53910	3760	3770	1	1	0.889	tGGCCGcaca
HvGSK1.1	P$ERF13_01	ERF13	3760	3770	1	1	0.865	tgGCCGCaca
HvGSK1.1	P$PTI5_01	TI5	3760	3770	1	1	0.852	tgGCCGCaca
HvGSK1.1	P$AT2G47520_01	AT2G47520	3760	3770	1	1	0.856	tgGCCGCaca
HvGSK1.1	P$ATERF14_01	ATERF14	3760	3770	1	1	0.921	tgGCCGCaca
HvGSK1.1	P$ERF4_02	ERF4	3760	3770	1	1	0.88	tgGCCGCaca
HvGSK1.1	P$DREBII1_01	DREBII1	3760	3770	1	1	0.85	tgGCCGCaca
HvGSK1.1	P$DREB1A_01	DREB1A	3760	3770	1	1	0.858	tgGCCGCaca
HvGSK1.1	P$ERF4_03	ERF4	3760	3770	1	1	0.905	tgGCCGCaca
HvGSK1.1	P$ERF1B_03	ERF1B	3760	3770	1	1	0.852	tgGCCGCaca
HvGSK1.1	P$BD1_01	BD1	3760	3770	1	1	0.861	tgGCCGCaca
HvGSK1.1	P$ERF105_01	ERF105	3760	3770	1	1	0.862	tgGCCGCaca
HvGSK1.1	P$CBF_01	CBF	3760	3770	1	1	0.853	tgGCCGCaca
HvGSK1.1	P$AT1G28160_01	AT1G28160	3760	3770	1	1	0.878	tgGCCGCaca
HvGSK1.1	P$AT3G61630_01	AT3G61630	3760	3770	1	1	0.85	tgGCCGCaca
HvGSK1.1	P$AT5G43410_01	AT5G43410	3760	3770	1	1	0.92	tgGCCGCaca
HvGSK1.1	P$AT3G16280_01	AT3G16280	3760	3770	1	1	0.868	tgGCCGCaca
HvGSK1.1	P$AT1G49120_01	AT1G49120	3760	3770	1	1	0.851	tgGCCGCaca
HvGSK1.1	P$LEP_01	LEP	3760	3770	1	1	0.865	tgGCCGCaca
HvGSK1.1	P$AT4G23750_01	AT4G23750	3760	3770	1	1	0.861	tgGCCGCaca
HvGSK1.1	P$CRF2_01	CRF2	3760	3768	1	1	0.881	tgGCCGCa
HvGSK1.1	P$ERF098_01	ERF098	3760	3768	1	1	0.897	tgGCCGCa
HvGSK1.1	P$ERF7_02	ERF7	3761	3771	1	1	0.945	gGCCGCacac
HvGSK1.1	P$NAC92_01	NAC92	3765	3777	1	1	0.985	gcACACGgcacc
HvGSK1.1	P$AT5G04240_01	AT5G04240	3770	3776	1	1	0.976	cGGCAC
HvGSK1.1	P$HBPA1_Q6_01	HBPA1	3773	3783	1	1	0.86	caccCGTCAc
HvGSK1.1	P$FAR1_01	FAR1	3776	3791	1	1	0.851	ccgtcACGCGtcccc
HvGSK1.1	P$FHY3_01	FHY3	3778	3790	1	1	0.858	gtcACGCGtccc
HvGSK1.1	P$CMTA2_01	CMTA2	3778	3787	1	1	0.986	gtcaCGCGT
HvGSK1.1	P$CAMTA1_02	CAMTA1	3778	3790	1	1	0.879	gtcaCGCGTccc
HvGSK1.1	P$TRAB1_Q2	TRAB1	3779	3790	1	1	0.927	tcACGCGtccc
HvGSK1.1	P$CMTA3_01	CMTA3	3781	3790	1	1	0.985	aCGCGTccc
HvGSK1.1	P$ABF2_01	ABF2	3788	3801	1	1	0.892	cccgtCACGTccc
HvGSK1.1	P$O2_Q4	O2	3789	3800	1	1	0.872	ccgtCACGTcc
HvGSK1.1	P$GBP_Q6	GBP	3790	3802	1	1	0.954	cgtCACGTcccc
HvGSK1.1	P$ABI5_01	ABI5	3790	3800	1	1	0.92	cgtCACGTcc
HvGSK1.1	P$ABF4_01	ABF4	3790	3802	1	1	0.873	cgtCACGTcccc
HvGSK1.1	P$EMBP1_Q2	EMBP1	3791	3801	1	1	0.897	gtCACGTccc
HvGSK1.1	P$CPRF3_Q2	CPRF3	3791	3801	1	1	0.965	gtCACGTccc
HvGSK1.1	P$CPRF2_Q2	CPRF2	3791	3801	1	1	0.956	gtCACGTccc
HvGSK1.1	P$O2_02	O2	3791	3801	1	1	0.944	gtCACGTccc
HvGSK1.1	P$TGA1B_Q2	TGA1B	3791	3801	1	1	0.964	gtCACGTccc
HvGSK1.1	P$TGA1A_Q2	TGA1A	3791	3801	1	1	0.98	gtCACGTccc
HvGSK1.1	P$CPRF3_01	CPRF3	3791	3801	1	1	0.973	gtCACGTccc
HvGSK1.1	P$CPRF2_01	CPRF2	3791	3801	1	1	0.956	gtCACGTccc
HvGSK1.1	P$TGA1B_01	TGA1B	3791	3801	1	1	0.984	gtCACGTccc
HvGSK1.1	P$BEE2_01	BEE2	3791	3801	1	1	0.907	gtCACGTccc
HvGSK1.1	P$BIM3_01	BIM3	3791	3801	1	1	0.88	gtCACGTccc
HvGSK1.1	P$PHYPA143875_02	HYPA143875	3791	3801	1	1	0.873	gtCACGTccc
HvGSK1.1	P$SPT_01	SPT	3791	3800	1	1	0.921	gtCACGTcc
HvGSK1.1	P$GBF1F_Q2	GBF1F	3791	3802	1	1	0.888	gtCACGTcccc
HvGSK1.1	P$RITA1_01	RITA1	3792	3799	1	1	0.98	tCACGTc
HvGSK1.1	P$OCSBF1_01	OCSBF1	3793	3798	1	1	1	CACGT
HvGSK1.1	P$TGA1A_01	TGA1A	3793	3800	1	1	0.861	cACGTCc
HvGSK1.1	P$O2_Q4	O2	3814	3825	1	1	0.862	gggcCATGTga
HvGSK1.1	P$AMS_01	AMS	3816	3826	1	1	0.86	gcCATGTgat
HvGSK1.1	P$CBF1_01	CBF1	3824	3834	1	1	0.853	aTGCCGggcc
HvGSK1.1	P$ERF019_01	ERF019	3824	3834	1	1	0.872	aTGCCGggcc
HvGSK1.1	P$JERF3_01	JERF3	3824	3834	1	1	0.885	aTGCCGggcc

HvGSK1.1	P$CEF1_01	CEF1	3824	3834	1	1	0.886	aTGCCGggcc
HvGSK1.1	P$JERF1_01	JERF1	3824	3834	1	1	0.891	aTGCCGggcc
HvGSK1.1	P$CBF1_03	CBF1	3824	3834	1	1	0.884	aTGCCGggcc
HvGSK1.1	P$AT1G33760_01	AT1G33760	3824	3834	1	1	0.868	aTGCCGggcc
HvGSK1.1	P$OJ1581_01	OJ1581	3827	3837	1	1	0.975	ccGGGCCcgc
HvGSK1.1	P$TCP2_01	TCP2	3827	3837	1	1	0.956	ccGGGCCcgc
HvGSK1.1	P$CMTA2_01	CMTA2	3832	3841	1	1	0.99	cccgCGCGT
HvGSK1.1	P$CAMTA1_02	CAMTA1	3832	3844	1	1	0.887	cccgCGCGTcgg
HvGSK1.1	P$CMTA3_01	CMTA3	3835	3844	1	1	0.982	gCGCGTcgg
HvGSK1.1	P$DREB1A_04	DREB1A	3837	3847	1	1	0.968	gcGTCGGcga
HvGSK1.1	P$ERF039_01	ERF039	3837	3847	1	1	0.984	gcGTCGGcga
HvGSK1.1	P$PHYPA182268_05	HYPA182268	3837	3847	1	1	0.878	gcGTCGGcga
HvGSK1.1	P$PHYPA173530_04	HYPA173530	3838	3846	1	1	0.875	cGTCGGcg
HvGSK1.1	P$PHYPA28324_10	HYPA28324	3838	3846	1	1	0.92	cGTCGGcg
HvGSK1.1	P$AT1G68550_03	AT1G68550	3838	3847	1	1	0.953	cgtCGGCGa
HvGSK1.1	P$RAV2_01	RAV2	3849	3858	1	1	0.866	ggACCGAta
HvGSK1.1	P$DOF1_01	DOF1	3853	3864	1	1	0.975	cgaTAAAGcag
HvGSK1.1	P$DOF2_01	DOF2	3853	3864	1	1	0.999	cgatAAAGCag
HvGSK1.1	P$DOF3_01	DOF3	3853	3864	1	1	0.978	cgatAAAGCag
HvGSK1.1	P$AMS_01	AMS	3859	3869	1	1	0.899	agCAGGTgag
HvGSK1.1	P$PCF2_01	CF2	3879	3889	1	1	0.88	ggcgaCCCAC
HvGSK1.1	P$TCP19_01	TCP19	3879	3889	1	1	0.863	ggcgaCCCAC
HvGSK1.1	P$TCP20L_01	TCP20L	3880	3889	1	1	0.858	gcgaCCCAC
HvGSK1.1	P$TCP20_02	TCP20	3881	3891	1	1	0.893	cgaCCCACca
HvGSK1.1	P$ASR1_01	ASR1	3883	3888	1	1	1	ACCCA
HvGSK1.1	P$LIM1_01	LIM1	3885	3897	1	1	0.934	CCACCaaggcag
HvGSK1.1	P$PCF5_01	CF5	3911	3921	1	1	0.886	cgGGTCCcgc
HvGSK1.1	P$WRKY11_Q2	WRKY11	3924	3932	1	1	0.974	tTTGACcg
HvGSK1.1	P$ZAP1_01	ZAP1	3925	3935	1	1	0.933	TTGACcggtc
HvGSK1.1	P$WRKY18_02	WRKY18	3929	3939	1	1	0.996	ccgGTCAAaa
HvGSK1.1	P$WRKY21_02	WRKY21	3929	3939	1	1	0.968	ccgGTCAAaa
HvGSK1.1	P$WRKY48_02	WRKY48	3929	3939	1	1	0.998	ccgGTCAAaa
HvGSK1.1	P$WRKY57_01	WRKY57	3929	3939	1	1	0.971	ccgGTCAAaa
HvGSK1.1	P$WRKY60_01	WRKY60	3929	3940	1	1	0.978	ccgGTCAAaag
HvGSK1.1	P$WRKY15_01	WRKY15	3930	3940	1	1	0.985	cgGTCAAaag
HvGSK1.1	P$WRKY2_01	WRKY2	3930	3938	1	1	0.991	cgGTCAAa
HvGSK1.1	P$WRKY25_02	WRKY25	3930	3938	1	1	0.979	cgGTCAAa
HvGSK1.1	P$WRKY40_01	WRKY40	3930	3938	1	1	1	cgGTCAAa
HvGSK1.1	P$WRKY43_02	WRKY43	3930	3940	1	1	0.977	cgGTCAAaag
HvGSK1.1	P$WRKY62_01	WRKY62	3930	3938	1	1	0.91	cgGTCAAa
HvGSK1.1	P$WRKY63_01	WRKY63	3930	3938	1	1	0.991	cgGTCAAa
HvGSK1.1	P$WRKY75_01	WRKY75	3930	3938	1	1	0.975	cgGTCAAa
HvGSK1.1	P$WRKY8_01	WRKY8	3930	3939	1	1	0.992	cgGTCAAaa
HvGSK1.1	P$WRKY23_01	WRKY23	3931	3939	1	1	0.854	gGTCAAaa
HvGSK1.1	P$WRKY30_01	WRKY30	3931	3941	1	1	0.916	gGTCAAaagg
HvGSK1.1	P$WRKY18_Q2	WRKY18	3932	3941	1	1	0.954	GTCAAaagg
HvGSK1.1	P$PBF_01	BF	3932	3943	1	1	0.96	gtcAAAAGgcg
HvGSK1.1	P$DOF_Q2	DOF	3932	3943	1	1	0.922	gtcAAAAGgcg
HvGSK1.1	P$CDF2_01	CDF2	3933	3943	1	1	0.944	tcAAAAGgcg
HvGSK1.1	P$CDF3_01	CDF3	3934	3943	1	1	0.968	cAAAAGgcg
HvGSK1.1	P$PBF_Q2	BF	3935	3941	1	1	1	aAAAGG
HvGSK1.1	P$BZIP68_01	BZIP68	3938	3947	1	1	0.947	aggCGTGGc
HvGSK1.1	P$MYBAS1_01	MYBAS1	3944	3955	1	1	0.949	ggCCAACcaaa
HvGSK1.1	P$AT4G36620_01	AT4G36620	3945	3953	1	1	0.886	gccAACCA
HvGSK1.1	P$GAMYB_01	GAMYB	3947	3955	1	1	0.901	CAACCaaa
HvGSK1.1	P$HSFA2_01	HSFA2	3950	3956	1	1	0.941	CCAAAc
HvGSK1.1	P$AT3G60580_01	AT3G60580	3970	3977	1	1	0.874	cgATCCC
HvGSK1.1	P$LEC2_01	LEC2	3982	3993	1	1	0.962	ccCATGCgccc
HvGSK1.1	P$PCF2_01	CF2	3987	3997	1	1	0.896	gcgccCCCAC
HvGSK1.1	P$TCP19_01	TCP19	3987	3997	1	1	0.902	gcgccCCCAC
HvGSK1.1	P$TCP20L_01	TCP20L	3988	3997	1	1	0.881	cgccCCCAC
HvGSK1.1	P$TCP20_02	TCP20	3989	3999	1	1	0.902	gccCCCACga
HvGSK1.1	P$ARALY495258_02	ARALY495258	3989	3997	1	1	0.933	gccCCCAC
HvGSK1.1	P$ARALY484486_05	ARALY484486	3989	3997	1	1	0.933	gccCCCAC
HvGSK1.2	P$AT4G36620_01	AT4G36620	8	16	1	1	0.866	gcgAACCA
HvGSK1.2	P$ARR2_01	ARR2	11	21	1	1	0.862	aaccATCTTt
HvGSK1.2	P$AT4G36620_01	AT4G36620	22	30	1	1	0.903	tgcAACCA
HvGSK1.2	P$GAMYB_01	GAMYB	24	32	1	1	0.888	CAACCaag
HvGSK1.2	P$ANTL_01	ANTL	30	40	1	1	0.851	aGTTACatgt
HvGSK1.2	P$O2_Q4	O2	31	42	1	1	0.925	gttaCATGTga
HvGSK1.2	P$AMS_01	AMS	33	43	1	1	0.871	taCATGTgag
HvGSK1.2	P$ATSPL8_01	ATSPL8	56	72	1	1	0.889	tctgtTGTACatggac
HvGSK1.2	P$BPC1_Q2	BPC1	73	79	1	1	1	AGAAAg
HvGSK1.2	P$PBF_Q2	BF	74	80	1	1	0.965	gAAAGG
HvGSK1.2	P$AT1G53910_01	AT1G53910	87	97	1	1	0.928	gGGCCGcttc
HvGSK1.2	P$AT2G47520_01	AT2G47520	87	97	1	1	0.884	ggGCCGCttc
HvGSK1.2	P$AT1G77200_01	AT1G77200	87	97	1	1	0.885	ggGCCGCttc
HvGSK1.2	P$ATERF14_01	ATERF14	87	97	1	1	0.891	ggGCCGCttc

HvGSK1.2	P$ERF1_02	ERF1	87	97	1	1	0.879	ggGCCGCttc
HvGSK1.2	P$DREBIII3_01	DREBIII3	87	97	1	1	0.874	ggGCCGCttc
HvGSK1.2	P$DREBIII2_01	DREBIII2	87	97	1	1	0.873	ggGCCGCttc
HvGSK1.2	P$DREBIII1_01	DREBIII1	87	97	1	1	0.875	ggGCCGCttc
HvGSK1.2	P$EREBP1_01	EREBP1	87	97	1	1	0.874	ggGCCGCttc
HvGSK1.2	P$AT2G44940_01	AT2G44940	87	97	1	1	0.885	ggGCCGCttc
HvGSK1.2	P$ERF2_03	ERF2	87	97	1	1	0.871	ggGCCGCttc
HvGSK1.2	P$DBF2_01	DBF2	87	97	1	1	0.903	ggGCCGCttc
HvGSK1.2	P$AT3G61630_01	AT3G61630	87	97	1	1	0.871	ggGCCGCttc
HvGSK1.2	P$AT5G43410_01	AT5G43410	87	97	1	1	0.896	ggGCCGCttc
HvGSK1.2	P$TINY2_02	TINY2	87	97	1	1	0.887	ggGCCGCttc
HvGSK1.2	P$AT3G16280_01	AT3G16280	87	97	1	1	0.906	ggGCCGCttc
HvGSK1.2	P$CRF2_01	CRF2	87	95	1	1	0.887	ggGCCGCt
HvGSK1.2	P$ERF098_01	ERF098	87	95	1	1	0.893	ggGCCGCt
HvGSK1.2	P$ERF7_02	ERF7	88	98	1	1	0.937	gGCCGCttca
HvGSK1.2	P$AT3G60580_01	AT3G60580	95	102	1	1	0.851	tcATCCC
HvGSK1.2	P$AT2G41690_01	AT2G41690	111	117	1	1	0.974	CCGAAt
HvGSK1.2	P$ARR18_01	ARR18	115	128	1	1	0.942	atgaAGATAagac
HvGSK1.2	P$ARR2_01	ARR2	138	148	1	1	0.866	agcaATCTTc
HvGSK1.2	P$AT5G54070_01	AT5G54070	151	157	1	1	0.91	tCAACG
HvGSK1.2	P$BZR1_01	BZR1	181	187	1	1	0.915	CGTGCa
HvGSK1.2	P$O2_Q4	O2	181	192	1	1	0.854	cgtgCATGTta
HvGSK1.2	P$ABI3_01	ABI3	182	191	1	1	0.861	gtGCATGtt
HvGSK1.2	P$ATHSFA1D_01	ATHSFA1D	192	198	1	1	0.941	tCTACA
HvGSK1.2	P$MYB305_Q3	MYB305	198	211	1	1	0.875	catagACCTAgct
HvGSK1.2	P$HSFA4A_01	HSFA4A	220	226	1	1	1	aCTATT
HvGSK1.2	P$MYB24_01	MYB24	221	230	1	1	0.881	ctaTTAGGg
HvGSK1.2	P$ASR1_01	ASR1	264	269	1	1	1	ACCCA
HvGSK1.2	P$GATA9_01	GATA9	266	277	1	1	0.895	ccaAGATCaat
HvGSK1.2	P$AGP1_01	AGP1	267	277	1	1	0.903	caAGATCaat
HvGSK1.2	P$ARR10_01	ARR10	269	276	1	1	0.869	AGATCaa
HvGSK1.2	P$ATHSFA1D_01	ATHSFA1D	311	317	1	1	0.941	tCTACA
HvGSK1.2	P$AT3G18650_01	AT3G18650	317	328	1	1	0.877	tacccTTGTAg
HvGSK1.2	P$GATA9_01	GATA9	323	334	1	1	0.894	tgtAGATCgat
HvGSK1.2	P$AGP1_01	AGP1	324	334	1	1	0.902	gtAGATCgat
HvGSK1.2	P$GATA10_01	GATA10	325	333	1	1	0.882	tAGATCga
HvGSK1.2	P$ARR10_01	ARR10	326	333	1	1	0.869	AGATCga
HvGSK1.2	P$BPC1_Q2	BPC1	348	354	1	1	0.99	AGAAAc
HvGSK1.2	P$SPF1_Q2	SPF1	352	362	1	1	0.907	acATAGTtga
HvGSK1.2	P$TGA1A_01	TGA1A	370	377	1	1	0.871	aACGTCt
HvGSK1.2	P$ATHB7_01	ATHB7	384	394	1	1	0.924	tcAATCAcaa
HvGSK1.2	P$HAT1_01	HAT1	384	394	1	1	0.868	tcAATCAcaa
HvGSK1.2	P$MYBAS1_01	MYBAS1	405	416	1	1	0.939	atCCAACcatg
HvGSK1.2	P$AT4G36620_01	AT4G36620	406	414	1	1	0.905	tccAACCA
HvGSK1.2	P$GAMYB_01	GAMYB	408	416	1	1	0.878	CAACCatg
HvGSK1.2	P$ERF112_02	ERF112	430	440	1	1	0.932	agCGCCGaac
HvGSK1.2	P$CRF4_01	CRF4	431	439	1	1	0.875	gCGCCGaa
HvGSK1.2	P$ERF4_04	ERF4	431	439	1	1	0.865	gCGCCGaa
HvGSK1.2	P$ERF069_01	ERF069	431	440	1	1	0.987	gCGCCGaac
HvGSK1.2	P$ERF11_01	ERF11	431	441	1	1	0.971	gCGCCGaacg
HvGSK1.2	P$ERF8_01	ERF8	432	442	1	1	0.946	CGCCGaacga
HvGSK1.2	P$AT2G41690_01	AT2G41690	434	440	1	1	1	CCGAAc
HvGSK1.2	P$TGA1_01	TGA1	474	485	1	1	0.949	cgaTGACGcct
HvGSK1.2	P$TGA7_01	TGA7	475	485	1	1	0.905	gaTGACGcct
HvGSK1.2	P$TGA5_01	TGA5	476	484	1	1	0.884	aTGACGcc
HvGSK1.2	P$AT4G00870_01	AT4G00870	489	503	1	1	0.935	caCCTCGattagcg
HvGSK1.2	P$ABI3_01	ABI3	535	544	1	1	0.862	taGCATGac
HvGSK1.2	P$TGA1_01	TGA1	537	548	1	1	0.941	gcaTGACGaca
HvGSK1.2	P$TGA7_01	TGA7	538	548	1	1	0.915	caTGACGaca
HvGSK1.2	P$TGA5_01	TGA5	539	547	1	1	0.87	aTGACGac
HvGSK1.2	P$AT1G53910_02	AT1G53910	542	563	1	1	0.977	acgacatggtgGCGGCggtgg
HvGSK1.2	P$RRTF1_05	RRTF1	547	562	1	1	0.954	atggtggCGGCGgtg
HvGSK1.2	P$E2L_Q2	E2L	551	558	1	1	0.91	tGGCGGc
HvGSK1.2	P$AT1G28160_02	AT1G28160	551	566	1	1	0.978	tggCGGCGgtggtga
HvGSK1.2	P$RAP26_06	RAP26	551	566	1	1	0.972	tggCGGCGgtggtga
HvGSK1.2	P$AT1G68550_03	AT1G68550	551	560	1	1	0.999	tggCGGCGg
HvGSK1.2	P$ERF1_Q2	ERF1	552	560	1	1	0.951	GGCGGcgg
HvGSK1.2	P$RAP21_02	RAP21	553	566	1	1	0.95	gcggCGGTGgtga
HvGSK1.2	P$ERF1_Q2	ERF1	555	563	1	1	0.861	GGCGGtgg
HvGSK1.2	P$MYB1L_01	MYB1L	580	590	1	1	0.977	agCCCTAtcg
HvGSK1.2	P$TRB2_01	TRB2	580	588	1	1	0.981	agCCCTAt
HvGSK1.2	P$RAMOSA1_01	RAMOSA1	614	628	1	1	0.938	acgagaGAGAGaga
HvGSK1.2	P$RAMOSA1_01	RAMOSA1	616	630	1	1	1	gagagaGAGAGaga
HvGSK1.2	P$RAMOSA1_01	RAMOSA1	618	632	1	1	0.924	gagagaGAGAGatg
HvGSK1.2	P$ARF8_01	ARF8	637	646	1	1	0.95	tgTGTCGtg
HvGSK1.2	P$BZIP68_01	BZIP68	639	648	1	1	0.945	tgtCGTGGc
HvGSK1.2	P$PTI6_01	TI6	702	712	1	1	0.932	gGGCCGgcac
HvGSK1.2	P$TSI1_01	TSI1	702	712	1	1	0.925	gGGCCGgcac

HvGSK1.2	P$AT1G53910_01	AT1G53910	702	712	1	1	0.984	gGGCCGgcac
HvGSK1.2	P$ERF6_02	ERF6	702	712	1	1	0.981	ggGCCGGcac
HvGSK1.2	P$ERF105_02	ERF105	703	711	1	1	0.944	gGCCGGca
HvGSK1.2	P$AT5G04240_01	AT5G04240	706	712	1	1	0.976	cGGCAC
HvGSK1.2	P$MYB1L_01	MYB1L	709	719	1	1	0.984	caCCCTAggg
HvGSK1.2	P$TRB2_01	TRB2	709	717	1	1	0.963	caCCCTAg
HvGSK1.2	P$MYB1L_01	MYB1L	721	731	1	1	0.956	tcCCCTAggg
HvGSK1.2	P$TRB2_01	TRB2	721	729	1	1	0.941	tcCCCTAg
HvGSK1.2	P$ALFIN1_Q2	ALFIN1	725	740	1	1	0.946	ctagggGTGGGgcga
HvGSK1.2	P$ANAC013_01	ANAC013	753	769	1	1	0.972	CTTGGctctcaaggaa
HvGSK1.2	P$MYBAS1_01	MYBAS1	836	847	1	1	0.979	agCCAACccac
HvGSK1.2	P$GAMYB_01	GAMYB	839	847	1	1	0.945	CAACCcac
HvGSK1.2	P$ASR1_01	ASR1	841	846	1	1	1	ACCCA
HvGSK1.2	P$LIM1_01	LIM1	843	855	1	1	0.93	CCACCaggggct
HvGSK1.2	P$E2FA_02	E2FA	856	866	1	1	0.986	gtgCGCCAcc
HvGSK1.2	P$ROM_Q2	ROM	860	869	1	1	0.864	gcCACCTct
HvGSK1.2	P$LFY_Q2	LFY	872	883	1	1	0.862	agCCAATgggg
HvGSK1.2	P$OJ1581_01	OJ1581	878	888	1	1	0.981	tgGGGCCctc
HvGSK1.2	P$TCP2_01	TCP2	878	888	1	1	0.948	tgGGGCCctc
HvGSK1.2	P$E2L_Q2	E2L	890	897	1	1	0.957	aGGCGGg
HvGSK1.2	P$ERF1_Q2	ERF1	891	899	1	1	0.887	GGCGGgtg
HvGSK1.2	P$AT4G36620_01	AT4G36620	930	938	1	1	0.895	caaAACCA
HvGSK1.2	P$HSFA4A_01	HSFA4A	937	943	1	1	1	aCTATT
HvGSK1.2	P$SQUA_01	SQUA	938	948	1	1	0.931	ctaTTTTTtg
HvGSK1.2	P$MYBAS1_01	MYBAS1	968	979	1	1	0.985	taCCAACttcc
HvGSK1.2	P$ARR2_01	ARR2	984	994	1	1	0.867	atcaATCTTt
HvGSK1.2	P$AT3G62240_01	AT3G62240	999	1009	1	1	0.852	cgggcCATTC
HvGSK1.2	P$O2_Q4	O2	1018	1029	1	1	0.858	tcatCATGTcc
HvGSK1.2	P$GATA8_01	GATA8	1029	1038	1	1	0.98	ggGATCTca
HvGSK1.2	P$ARR2_01	ARR2	1050	1060	1	1	0.884	aacaATCTTc
HvGSK1.2	P$ATHB7_01	ATHB7	1058	1068	1	1	0.922	tcAATCAcca
HvGSK1.2	P$HAT1_01	HAT1	1058	1068	1	1	0.868	tcAATCAcca
HvGSK1.2	P$MYBAS1_01	MYBAS1	1063	1074	1	1	0.954	caCCAACataa
HvGSK1.2	P$RAV1_01	RAV1	1063	1075	1	1	0.972	cacCAACAtaat
HvGSK1.2	P$ATHB6_01	ATHB6	1070	1079	1	1	0.909	atAATAAct
HvGSK1.2	P$AT5G26170_01	AT5G26170	1076	1085	1	1	0.883	acTCAACaa
HvGSK1.2	P$RAV1_01	RAV1	1076	1088	1	1	0.932	actCAACAatac
HvGSK1.2	P$ARR2_01	ARR2	1104	1114	1	1	0.868	gttaATCTTg
HvGSK1.2	P$EDF3_02	EDF3	1146	1155	1	1	0.889	tGACCGaga
HvGSK1.2	P$RAV2_01	RAV2	1146	1155	1	1	0.887	tgACCGAga
HvGSK1.2	P$WRKY25_01	WRKY25	1167	1176	1	1	0.88	ccaTAACCa
HvGSK1.2	P$AT1G69310_01	AT1G69310	1167	1176	1	1	0.855	ccaTAACCa
HvGSK1.2	P$AT4G36620_01	AT4G36620	1168	1176	1	1	0.892	catAACCA
HvGSK1.2	P$HSF3_01	HSF3	1179	1185	1	1	0.945	gCGGGG
HvGSK1.2	P$ATHSFA1D_01	ATHSFA1D	1205	1211	1	1	0.985	cCTACA
HvGSK1.2	P$ATHSFA1D_01	ATHSFA1D	1214	1220	1	1	0.941	tCTACA
HvGSK1.2	P$GATA9_01	GATA9	1218	1229	1	1	0.98	caaAGATCttt
HvGSK1.2	P$AGP1_01	AGP1	1219	1229	1	1	0.938	aaAGATCttt
HvGSK1.2	P$ARR2_01	ARR2	1219	1229	1	1	0.899	aaagATCTTt
HvGSK1.2	P$GATA10_01	GATA10	1220	1228	1	1	0.912	aAGATCtt
HvGSK1.2	P$GATA11_01	GATA11	1220	1228	1	1	0.949	aaGATCTt
HvGSK1.2	P$GATA8_01	GATA8	1220	1229	1	1	0.987	aaGATCTtt
HvGSK1.2	P$ARR10_01	ARR10	1221	1228	1	1	0.956	AGATCtt
HvGSK1.2	P$AT4G36620_01	AT4G36620	1234	1242	1	1	0.976	ttgAACCA
HvGSK1.2	P$WRKY18_02	WRKY18	1243	1253	1	1	0.948	gatGTCAAgg
HvGSK1.2	P$WRKY21_02	WRKY21	1243	1253	1	1	0.95	gatGTCAAgg
HvGSK1.2	P$WRKY48_02	WRKY48	1243	1253	1	1	0.985	gatGTCAAgg
HvGSK1.2	P$WRKY57_01	WRKY57	1243	1253	1	1	0.959	gatGTCAAgg
HvGSK1.2	P$WRKY60_01	WRKY60	1243	1254	1	1	0.888	gatGTCAAgga
HvGSK1.2	P$WRKY15_01	WRKY15	1244	1254	1	1	0.959	atGTCAAgga
HvGSK1.2	P$WRKY2_01	WRKY2	1244	1252	1	1	0.9	atGTCAAg
HvGSK1.2	P$WRKY25_02	WRKY25	1244	1252	1	1	0.878	atGTCAAg
HvGSK1.2	P$WRKY40_01	WRKY40	1244	1252	1	1	0.977	atGTCAAg
HvGSK1.2	P$WRKY43_02	WRKY43	1244	1254	1	1	0.951	atGTCAAgga
HvGSK1.2	P$WRKY63_01	WRKY63	1244	1252	1	1	0.888	atGTCAAg
HvGSK1.2	P$WRKY75_01	WRKY75	1244	1252	1	1	0.919	atGTCAAg
HvGSK1.2	P$WRKY8_01	WRKY8	1244	1253	1	1	0.977	atGTCAAgg
HvGSK1.2	P$WRKY30_01	WRKY30	1245	1255	1	1	0.904	tGTCAAggat
HvGSK1.2	P$WRKY18_Q2	WRKY18	1246	1255	1	1	0.949	GTCAAggat
HvGSK1.2	P$AT3G60580_01	AT3G60580	1260	1267	1	1	0.905	caATCCC
HvGSK1.2	P$SED_Q2	SED	1274	1284	1	1	0.985	gtccCCTTTa
HvGSK1.2	P$PBF_Q2_01	BF	1278	1284	1	1	0.998	CCTTTa
HvGSK1.2	P$UIF1_01	UIF1	1305	1315	1	1	0.978	cgaGATTCga
HvGSK1.2	P$HSFA4A_01	HSFA4A	1332	1338	1	1	0.964	cCTATT
HvGSK1.2	P$ARR2_01	ARR2	1337	1347	1	1	0.866	ttcaATCTTg
HvGSK1.2	P$KNOX3_01	KNOX3	1347	1359	1	1	0.975	ttacTGACAagt
HvGSK1.2	P$ATH1_01	ATH1	1351	1359	1	1	0.931	TGACAagt
HvGSK1.2	P$NAC6_01	NAC6	1372	1378	1	1	0.854	tCGTAA

HvGSK1.2	P$GATA9_01	GATA9	1379	1390	1	1	0.899	acaAGATCcca
HvGSK1.2	P$AGP1_01	AGP1	1380	1390	1	1	0.869	caAGATCcca
HvGSK1.2	P$ARR10_01	ARR10	1382	1389	1	1	0.934	AGATCcc
HvGSK1.2	P$AT3G60580_01	AT3G60580	1382	1389	1	1	0.852	agATCCC
HvGSK1.2	P$WEREWOLF_Q2_01	WEREWOLF	1391	1400	1	1	0.906	gACTAActc
HvGSK1.2	P$MYBAS1_01	MYBAS1	1391	1402	1	1	0.987	gaCTAACtcct
HvGSK1.2	P$CBNAC_01	CBNAC	1410	1416	1	1	1	tTGCTT
HvGSK1.2	P$CBNAC_02	CBNAC	1410	1426	1	1	0.902	tTGCTTgcaagattat
HvGSK1.2	P$ATHB1_01	ATHB1	1416	1430	1	1	0.869	gcaagATTATtgtg
HvGSK1.2	P$ATSPL8_01	ATSPL8	1429	1445	1	1	0.935	gatgtTGTACtaccta
HvGSK1.2	P$HMG1_01	HMG1	1432	1441	1	1	0.923	GTTGTacta
HvGSK1.2	P$P_01		1436	1445	1	1	0.874	taCTACCta
HvGSK1.2	P$TCP11_01	TCP11	1444	1456	1	1	0.954	aGTGGGccctga
HvGSK1.2	P$TCP15_01	TCP15	1445	1455	1	1	0.963	GTGGGccctg
HvGSK1.2	P$TCP20_01	TCP20	1445	1455	1	1	0.98	GTGGGccctg
HvGSK1.2	P$TCP11_02	TCP11	1445	1455	1	1	0.968	GTGGGccctg
HvGSK1.2	P$OJ1581_01	OJ1581	1445	1455	1	1	0.983	gtGGGCCctg
HvGSK1.2	P$TCP2_01	TCP2	1445	1455	1	1	0.97	gtGGGCCctg
HvGSK1.2	P$UIF1_01	UIF1	1453	1463	1	1	0.979	tgaGATTCct
HvGSK1.2	P$TGA2_Q2	TGA2	1465	1475	1	1	0.87	cCGTCAtacg
HvGSK1.2	P$ATMYB15_Q2	ATMYB15	1478	1484	1	1	1	TAACAa
HvGSK1.2	P$AT3G60580_01	AT3G60580	1482	1489	1	1	0.883	aaATCCC
HvGSK1.2	P$LEC2_01	LEC2	1497	1508	1	1	0.943	tcCATGCcata
HvGSK1.2	P$MYBAS1_01	MYBAS1	1506	1517	1	1	0.973	taCCAACaaac
HvGSK1.2	P$RAV1_01	RAV1	1506	1518	1	1	0.954	tacCAACAaaca
HvGSK1.2	P$LEC2_01	LEC2	1514	1525	1	1	0.936	aaCATGCtcga
HvGSK1.2	P$ARR18_01	ARR18	1521	1534	1	1	0.942	tcgaAGATAccta
HvGSK1.2	P$ARR2_01	ARR2	1536	1546	1	1	0.962	gagcATCTTt
HvGSK1.2	P$GT1_Q6_01	GT1	1543	1555	1	1	0.893	TTTGTaatcatc
HvGSK1.2	P$ATHB7_01	ATHB7	1546	1556	1	1	0.92	gtAATCAtcc
HvGSK1.2	P$HAT1_01	HAT1	1546	1556	1	1	0.982	gtAATCAtcc
HvGSK1.2	P$TGA1_01	TGA1	1563	1574	1	1	0.979	ttgTGACGttt
HvGSK1.2	P$STF1_02	STF1	1564	1576	1	1	0.934	tgTGACGtttga
HvGSK1.2	P$TGA3_Q2	TGA3	1564	1573	1	1	0.934	tgTGACGtt
HvGSK1.2	P$TGA6_01	TGA6	1564	1574	1	1	0.948	tgTGACGttt
HvGSK1.2	P$TGA7_01	TGA7	1564	1574	1	1	0.947	tgTGACGttt
HvGSK1.2	P$BZIP14_01	BZIP14	1564	1574	1	1	0.959	tgTGACGttt
HvGSK1.2	P$STF1_01	STF1	1564	1576	1	1	0.946	tgtGACGTttga
HvGSK1.2	P$TGA5_01	TGA5	1565	1573	1	1	0.994	gTGACGtt
HvGSK1.2	P$TGA1B_01	TGA1B	1565	1575	1	1	0.888	gtGACGTttg
HvGSK1.2	P$AT3G62240_01	AT3G62240	1582	1592	1	1	0.911	gaaggCATTC
HvGSK1.2	P$ABI3_01	ABI3	1608	1617	1	1	0.875	ttGCATGat
HvGSK1.2	P$GATA15_01	GATA15	1611	1620	1	1	0.999	caTGATCtc
HvGSK1.2	P$GATA8_01	GATA8	1612	1621	1	1	0.979	atGATCTca
HvGSK1.2	P$MYB24_01	MYB24	1623	1632	1	1	0.853	gtcTTAGGa
HvGSK1.2	P$ARR18_01	ARR18	1630	1643	1	1	0.95	gaacAGATAcatt
HvGSK1.2	P$KNOX3_01	KNOX3	1638	1650	1	1	0.964	acatTGACAtat
HvGSK1.2	P$WRKY11_Q2	WRKY11	1640	1648	1	1	0.903	aTTGACat
HvGSK1.2	P$ATH1_01	ATH1	1642	1650	1	1	0.935	TGACAtat
HvGSK1.2	P$SPF1_Q2	SPF1	1646	1656	1	1	0.898	atATAGTaag
HvGSK1.2	P$AT3G20750_01	AT3G20750	1664	1672	1	1	0.882	gTAAACtt
HvGSK1.2	P$OSRR22_01	OSRR22	1673	1683	1	1	0.88	gtGATACgat
HvGSK1.2	P$CBNAC_01	CBNAC	1691	1697	1	1	0.968	aTGCTT
HvGSK1.2	P$CBNAC_02	CBNAC	1691	1707	1	1	0.909	aTGCTTacaattcgag
HvGSK1.2	P$ATHB7_01	ATHB7	1717	1727	1	1	0.923	caAATCAttt
HvGSK1.2	P$HAT1_01	HAT1	1717	1727	1	1	0.983	caAATCAttt
HvGSK1.2	P$ATHB4_02	ATHB4	1718	1728	1	1	0.924	aaATCATttt
HvGSK1.2	P$EDT1_01	EDT1	1726	1736	1	1	0.908	ttcTTAATga
HvGSK1.2	P$HAHB4_01	HAHB4	1730	1739	1	1	0.94	tAATGAtgt
HvGSK1.2	P$GATA15_01	GATA15	1736	1745	1	1	0.999	tgTGATCtc
HvGSK1.2	P$GATA8_01	GATA8	1737	1746	1	1	0.979	gtGATCTcg
HvGSK1.2	P$KNOX3_01	KNOX3	1750	1762	1	1	0.969	caaaTGACAact
HvGSK1.2	P$GAMYB_Q2	GAMYB	1751	1764	1	1	0.896	aaatgACAACtta
HvGSK1.2	P$ATH1_01	ATH1	1754	1762	1	1	0.926	TGACAact
HvGSK1.2	P$AT1G66560_01	AT1G66560	1780	1790	1	1	0.911	accTTAACca
HvGSK1.2	P$WRKY21_01	WRKY21	1781	1790	1	1	0.919	ccTTAACca
HvGSK1.2	P$AT1G18860_01	AT1G18860	1781	1790	1	1	0.907	ccTTAACca
HvGSK1.2	P$AT1G64000_01	AT1G64000	1781	1790	1	1	0.93	ccTTAACca
HvGSK1.2	P$WRKY6_01	WRKY6	1781	1790	1	1	0.869	ccTTAACca
HvGSK1.2	P$AT1G66600_01	AT1G66600	1781	1790	1	1	0.912	ccTTAACca
HvGSK1.2	P$AT1G68150_01	AT1G68150	1781	1790	1	1	0.895	ccTTAACca
HvGSK1.2	P$AT5G41570_01	AT5G41570	1781	1790	1	1	0.93	ccTTAACca
HvGSK1.2	P$AT5G15130_01	AT5G15130	1781	1790	1	1	0.903	ccTTAACca
HvGSK1.2	P$WRKY46_01	WRKY46	1781	1790	1	1	0.873	ccTTAACca
HvGSK1.2	P$AT2G24570_01	AT2G24570	1781	1790	1	1	0.916	ccTTAACca
HvGSK1.2	P$WRKY7_01	WRKY7	1781	1790	1	1	0.925	ccTTAACca
HvGSK1.2	P$WRKY25_01	WRKY25	1781	1790	1	1	0.944	cctTAACCa
HvGSK1.2	P$WRKY33_01	WRKY33	1781	1790	1	1	0.879	cctTAACCa

HvGSK1.2	P$AT1G29860_01	AT1G29860	1781	1790	1	1	0.907	cctTAACCa
HvGSK1.2	P$AT3G62340_01	AT3G62340	1781	1790	1	1	0.888	cctTAACCa
HvGSK1.2	P$AT1G69310_01	AT1G69310	1781	1790	1	1	0.916	cctTAACCa
HvGSK1.2	P$WRKY26_01	WRKY26	1781	1790	1	1	0.882	cctTAACCa
HvGSK1.2	P$GT1_01	GT1	1782	1790	1	1	0.968	ctTAACCa
HvGSK1.2	P$AT4G36620_01	AT4G36620	1782	1790	1	1	0.966	cttAACCA
HvGSK1.2	P$ARR2_01	ARR2	1785	1795	1	1	0.862	aaccATCTTt
HvGSK1.2	P$GATA15_01	GATA15	1792	1801	1	1	1	ttTGATCaa
HvGSK1.2	P$AT5G54070_01	AT5G54070	1797	1803	1	1	0.91	tCAACG
HvGSK1.2	P$MYB24_01	MYB24	1821	1830	1	1	0.882	ttaTTAGGg
HvGSK1.2	P$MRP1_Q2	MRP1	1839	1851	1	1	0.871	tgTCTATgtatc
HvGSK1.2	P$TEIL_01	TEIL	1844	1852	1	1	0.872	ATGTAtcc
HvGSK1.2	P$O2_Q4	O2	1851	1862	1	1	0.861	cacaCATGTag
HvGSK1.2	P$TEIL_01	TEIL	1856	1864	1	1	0.883	ATGTAgtt
HvGSK1.2	P$ATHB7_01	ATHB7	1870	1880	1	1	0.96	ccAATCAata
HvGSK1.2	P$HAT1_01	HAT1	1870	1880	1	1	0.895	ccAATCAata
HvGSK1.2	P$GAMYB_Q2	GAMYB	1874	1887	1	1	0.867	tcaatACAACtat
HvGSK1.2	P$C1_Q2	C1	1879	1890	1	1	0.937	acAACTAtagc
HvGSK1.2	P$ABI3_01	ABI3	1886	1895	1	1	0.873	taGCATGga
HvGSK1.2	P$AT3G20750_01	AT3G20750	1897	1905	1	1	0.907	aTAAACaa
HvGSK1.2	P$ATHB6_01	ATHB6	1924	1933	1	1	0.981	atAATAAta
HvGSK1.2	P$ATHB5_04	ATHB5	1924	1935	1	1	0.902	atAATAAtaac
HvGSK1.2	P$ATHB1_03	ATHB1	1924	1935	1	1	0.888	atAATAAtaac
HvGSK1.2	P$ATHB16_01	ATHB16	1925	1933	1	1	0.915	tAATAAta
HvGSK1.2	P$ATHB6_01	ATHB6	1927	1936	1	1	0.903	atAATAAcc
HvGSK1.2	P$AT4G36620_01	AT4G36620	1929	1937	1	1	0.916	aatAACCA
HvGSK1.2	P$ATHB1_01	ATHB1	1936	1950	1	1	0.879	acttcATTATtgcc
HvGSK1.2	P$ATHB5_01	ATHB5	1939	1948	1	1	0.866	tcaTTATTg
HvGSK1.2	P$SED_Q2	SED	1944	1954	1	1	0.983	attgCCTTTa
HvGSK1.2	P$PBF_Q2_01	BF	1948	1954	1	1	0.998	CCTTTa
HvGSK1.2	P$MYB24_01	MYB24	1948	1957	1	1	0.858	cctTTAGGg
HvGSK1.2	P$HSFA2_01	HSFA2	1964	1970	1	1	1	CCAAAa
HvGSK1.2	P$C1_Q2	C1	1966	1977	1	1	0.929	aaAACTAgatg
HvGSK1.2	P$ABI3_01	ABI3	1992	2001	1	1	0.866	tgGCATGgt
HvGSK1.2	P$RAV1_02	RAV1	2008	2020	1	1	0.923	agaACCTGaagc
HvGSK1.2	P$TGA1_01	TGA1	2019	2030	1	1	0.935	ccgTGACGaag
HvGSK1.2	P$TGA7_01	TGA7	2020	2030	1	1	0.877	cgTGACGaag
HvGSK1.2	P$TGA5_01	TGA5	2021	2029	1	1	0.865	gTGACGaa
HvGSK1.2	P$DOF2_01	DOF2	2034	2045	1	1	0.981	tggcAAAGCag
HvGSK1.2	P$DOF3_01	DOF3	2034	2045	1	1	0.975	tggcAAAGCag
HvGSK1.2	P$RAV1_01	RAV1	2042	2054	1	1	0.955	cagCAACAagaa
HvGSK1.2	P$HBP1A_Q2	HBP1A	2050	2060	1	1	0.859	agaACGTGaa
HvGSK1.2	P$TAF1_Q2	TAF1	2050	2060	1	1	0.917	agaACGTGaa
HvGSK1.2	P$EMBP1_02	EMBP1	2050	2060	1	1	0.858	agaACGTGaa
HvGSK1.2	P$TAF1_01	TAF1	2050	2060	1	1	0.94	agaACGTGaa
HvGSK1.2	P$GBF1_01	GBF1	2051	2059	1	1	0.897	gaACGTGa
HvGSK1.2	P$BIM1_02	BIM1	2051	2061	1	1	0.949	gaACGTGaac
HvGSK1.2	P$ABF4_02	ABF4	2051	2061	1	1	0.948	gaACGTGaac
HvGSK1.2	P$ABI5_Q2	ABI5	2053	2059	1	1	0.936	ACGTGa
HvGSK1.2	P$P_01		2073	2082	1	1	0.953	tcCTACCac
HvGSK1.2	P$LEC2_01	LEC2	2079	2090	1	1	0.99	caCATGCacgt
HvGSK1.2	P$HBP1B_Q6	HBP1B	2079	2093	1	1	0.856	cacatgcaCGTCAt
HvGSK1.2	P$FUS3_Q2	FUS3	2080	2091	1	1	0.921	aCATGCacgtc
HvGSK1.2	P$ABF2_01	ABF2	2080	2093	1	1	0.896	acatgCACGTcat
HvGSK1.2	P$O2_Q4	O2	2081	2092	1	1	0.96	catgCACGTca
HvGSK1.2	P$BZR1_02	BZR1	2081	2095	1	1	0.863	catgCACGTcataa
HvGSK1.2	P$HBI1_01	HBI1	2081	2093	1	1	0.865	catgCACGTcat
HvGSK1.2	P$GBP_Q6	GBP	2082	2094	1	1	0.914	atgCACGTcata
HvGSK1.2	P$ABI5_01	ABI5	2082	2092	1	1	0.888	atgCACGTca
HvGSK1.2	P$ABF4_01	ABF4	2082	2094	1	1	0.874	atgCACGTcata
HvGSK1.2	P$EMBP1_Q2	EMBP1	2083	2093	1	1	0.85	tgCACGTcat
HvGSK1.2	P$CPRF3_Q2	CPRF3	2083	2093	1	1	0.952	tgCACGTcat
HvGSK1.2	P$CPRF2_Q2	CPRF2	2083	2093	1	1	0.954	tgCACGTcat
HvGSK1.2	P$O2_02	O2	2083	2093	1	1	0.95	tgCACGTcat
HvGSK1.2	P$TGA1B_Q2	TGA1B	2083	2093	1	1	0.922	tgCACGTcat
HvGSK1.2	P$TGA1A_Q2	TGA1A	2083	2093	1	1	0.981	tgCACGTcat
HvGSK1.2	P$CPRF3_01	CPRF3	2083	2093	1	1	0.967	tgCACGTcat
HvGSK1.2	P$CPRF2_01	CPRF2	2083	2093	1	1	0.956	tgCACGTcat
HvGSK1.2	P$TGA1B_01	TGA1B	2083	2093	1	1	0.89	tgCACGTcat
HvGSK1.2	P$BEE2_01	BEE2	2083	2093	1	1	0.907	tgCACGTcat
HvGSK1.2	P$BIM3_01	BIM3	2083	2093	1	1	0.88	tgCACGTcat
HvGSK1.2	P$PHYPA143875_02	HYPA143875	2083	2093	1	1	0.873	tgCACGTcat
HvGSK1.2	P$SPT_01	SPT	2083	2092	1	1	0.927	tgCACGTca
HvGSK1.2	P$GBF1F_Q2	GBF1F	2083	2094	1	1	0.863	tgCACGTcata
HvGSK1.2	P$HBPA1_Q6_01	HBPA1	2083	2093	1	1	0.899	tgcaCGTCAt
HvGSK1.2	P$RITA1_01	RITA1	2084	2091	1	1	0.954	gCACGTc
HvGSK1.2	P$OCSBF1_01	OCSBF1	2085	2090	1	1	1	CACGT
HvGSK1.2	P$TGA1A_01	TGA1A	2085	2092	1	1	0.989	cACGTCa

HvGSK1.2	P$TGA2_Q2	TGA2	2086	2096	1	1	0.94	aCGTCAtaaa
HvGSK1.2	P$AT3G20750_01	AT3G20750	2091	2099	1	1	0.938	aTAAACtt
HvGSK1.2	P$ID1_01	ID1	2102	2113	1	1	0.878	tTTGTCaactt
HvGSK1.2	P$WRKY18_02	WRKY18	2102	2112	1	1	0.946	tttGTCAAct
HvGSK1.2	P$WRKY21_02	WRKY21	2102	2112	1	1	0.983	tttGTCAAct
HvGSK1.2	P$WRKY48_02	WRKY48	2102	2112	1	1	0.989	tttGTCAAct
HvGSK1.2	P$WRKY57_01	WRKY57	2102	2112	1	1	0.974	tttGTCAAct
HvGSK1.2	P$WRKY60_01	WRKY60	2102	2113	1	1	0.914	tttGTCAActt
HvGSK1.2	P$WRKY15_01	WRKY15	2103	2113	1	1	0.976	ttGTCAActt
HvGSK1.2	P$WRKY2_01	WRKY2	2103	2111	1	1	0.919	ttGTCAAc
HvGSK1.2	P$WRKY25_02	WRKY25	2103	2111	1	1	0.913	ttGTCAAc
HvGSK1.2	P$WRKY40_01	WRKY40	2103	2111	1	1	0.977	ttGTCAAc
HvGSK1.2	P$WRKY43_02	WRKY43	2103	2113	1	1	0.961	ttGTCAActt
HvGSK1.2	P$WRKY62_01	WRKY62	2103	2111	1	1	0.938	ttGTCAAc
HvGSK1.2	P$WRKY63_01	WRKY63	2103	2111	1	1	0.895	ttGTCAAc
HvGSK1.2	P$WRKY75_01	WRKY75	2103	2111	1	1	0.959	ttGTCAAc
HvGSK1.2	P$WRKY8_01	WRKY8	2103	2112	1	1	0.986	ttGTCAAct
HvGSK1.2	P$WRKY23_01	WRKY23	2104	2112	1	1	0.889	tGTCAAct
HvGSK1.2	P$WRKY30_01	WRKY30	2104	2114	1	1	0.975	tGTCAActtt
HvGSK1.2	P$WRKY18_Q2	WRKY18	2105	2114	1	1	0.973	GTCAActtt
HvGSK1.2	P$SED_Q2	SED	2112	2122	1	1	0.956	ttacCCTTTg
HvGSK1.2	P$PBF_Q2_01	BF	2116	2122	1	1	0.988	CCTTTg
HvGSK1.2	P$ATHB6_01	ATHB6	2125	2134	1	1	0.926	acAATAAaa
HvGSK1.2	P$CBNAC_01	CBNAC	2137	2143	1	1	0.968	aTGCTT
HvGSK1.2	P$WRKY18_02	WRKY18	2147	2157	1	1	0.946	ggtGTCAAga
HvGSK1.2	P$WRKY21_02	WRKY21	2147	2157	1	1	0.945	ggtGTCAAga
HvGSK1.2	P$WRKY48_02	WRKY48	2147	2157	1	1	0.985	ggtGTCAAga
HvGSK1.2	P$WRKY57_01	WRKY57	2147	2157	1	1	0.953	ggtGTCAAga
HvGSK1.2	P$WRKY60_01	WRKY60	2147	2158	1	1	0.883	ggtGTCAAgaa
HvGSK1.2	P$WRKY15_01	WRKY15	2148	2158	1	1	0.956	gtGTCAAgaa
HvGSK1.2	P$WRKY2_01	WRKY2	2148	2156	1	1	0.902	gtGTCAAg
HvGSK1.2	P$WRKY25_02	WRKY25	2148	2156	1	1	0.88	gtGTCAAg
HvGSK1.2	P$WRKY40_01	WRKY40	2148	2156	1	1	0.977	gtGTCAAg
HvGSK1.2	P$WRKY43_02	WRKY43	2148	2158	1	1	0.949	gtGTCAAgaa
HvGSK1.2	P$WRKY63_01	WRKY63	2148	2156	1	1	0.887	gtGTCAAg
HvGSK1.2	P$WRKY75_01	WRKY75	2148	2156	1	1	0.918	gtGTCAAg
HvGSK1.2	P$WRKY8_01	WRKY8	2148	2157	1	1	0.975	gtGTCAAga
HvGSK1.2	P$WRKY30_01	WRKY30	2149	2159	1	1	0.897	tGTCAAgaat
HvGSK1.2	P$WRKY18_Q2	WRKY18	2150	2159	1	1	0.949	GTCAAgaat
HvGSK1.2	P$KNOX3_01	KNOX3	2154	2166	1	1	0.959	agaaTGACAtgc
HvGSK1.2	P$ATH1_01	ATH1	2158	2166	1	1	0.932	TGACAtgc
HvGSK1.2	P$LEC2_01	LEC2	2159	2170	1	1	0.937	gaCATGCcaga
HvGSK1.2	P$RAV1_01	RAV1	2168	2180	1	1	0.957	gagCAACAtgat
HvGSK1.2	P$KNOX3_01	KNOX3	2181	2193	1	1	0.951	atatTGACAatc
HvGSK1.2	P$WRKY11_Q2	WRKY11	2183	2191	1	1	0.924	aTTGACaa
HvGSK1.2	P$ATH1_01	ATH1	2185	2193	1	1	0.906	TGACAatc
HvGSK1.2	P$AT3G20750_01	AT3G20750	2217	2225	1	1	0.943	aTAAACgt
HvGSK1.2	P$RAV1_01	RAV1	2233	2245	1	1	0.924	tatCAACActtg
HvGSK1.2	P$ATHSFA1D_01	ATHSFA1D	2245	2251	1	1	0.985	cCTACA
HvGSK1.2	P$PBF_01	BF	2253	2264	1	1	0.961	agcAAAAGgaa
HvGSK1.2	P$DOF_Q2	DOF	2253	2264	1	1	0.941	agcAAAAGgaa
HvGSK1.2	P$CDF2_01	CDF2	2254	2264	1	1	0.952	gcAAAAGgaa
HvGSK1.2	P$CDF3_01	CDF3	2255	2264	1	1	0.972	cAAAAGgaa
HvGSK1.2	P$PBF_Q2	BF	2256	2262	1	1	1	aAAAGG
HvGSK1.2	P$AT3G51080_01	AT3G51080	2260	2267	1	1	1	GGAAAaa
HvGSK1.2	P$WRKY11_Q2	WRKY11	2267	2275	1	1	0.925	cTTGACtc
HvGSK1.2	P$KNOX3_01	KNOX3	2312	2324	1	1	0.969	tcttTGACAatt
HvGSK1.2	P$WRKY11_Q2	WRKY11	2314	2322	1	1	0.924	tTTGACaa
HvGSK1.2	P$ATH1_01	ATH1	2316	2324	1	1	0.919	TGACAatt
HvGSK1.2	P$PEND_02	END	2320	2330	1	1	0.88	aaTTCTTaag
HvGSK1.2	P$SBF1_01	SBF1	2332	2346	1	1	0.855	tgcggtTTAATaaa
HvGSK1.2	P$AT1G19490_01	AT1G19490	2335	2344	1	1	0.899	GGTTTaata
HvGSK1.2	P$ATHB6_01	ATHB6	2338	2347	1	1	0.91	ttAATAAaa
HvGSK1.2	P$PBF_01	BF	2340	2351	1	1	0.984	aatAAAAGcaa
HvGSK1.2	P$DOF_Q2	DOF	2340	2351	1	1	0.951	aatAAAAGcaa
HvGSK1.2	P$DOF2_01	DOF2	2340	2351	1	1	0.995	aataAAAGCaa
HvGSK1.2	P$DOF3_01	DOF3	2340	2351	1	1	0.994	aataAAAGCaa
HvGSK1.2	P$CDF2_01	CDF2	2341	2351	1	1	0.962	atAAAAGcaa
HvGSK1.2	P$CDF3_01	CDF3	2342	2351	1	1	0.978	tAAAAGcaa
HvGSK1.2	P$GATA9_01	GATA9	2350	2361	1	1	0.974	aagAGATCtca
HvGSK1.2	P$AGP1_01	AGP1	2351	2361	1	1	0.932	agAGATCtca
HvGSK1.2	P$GATA10_01	GATA10	2352	2360	1	1	0.92	gAGATCtc
HvGSK1.2	P$GATA11_01	GATA11	2352	2360	1	1	0.949	gaGATCTc
HvGSK1.2	P$GATA8_01	GATA8	2352	2361	1	1	0.991	gaGATCTca
HvGSK1.2	P$ARR10_01	ARR10	2353	2360	1	1	0.913	AGATCtc
HvGSK1.2	P$SBF1_01	SBF1	2378	2392	1	1	0.908	aatttaTTAAAatt
HvGSK1.2	P$WRKY18_02	WRKY18	2389	2399	1	1	0.946	attGTCAAac
HvGSK1.2	P$WRKY21_02	WRKY21	2389	2399	1	1	0.956	attGTCAAac

HvGSK1.2	P$WRKY48_02	WRKY48	2389	2399	1	1	0.987	attGTCAAac
HvGSK1.2	P$WRKY57_01	WRKY57	2389	2399	1	1	0.965	attGTCAAac
HvGSK1.2	P$WRKY60_01	WRKY60	2389	2400	1	1	0.899	attGTCAAaca
HvGSK1.2	P$WRKY15_01	WRKY15	2390	2400	1	1	0.964	ttGTCAAaca
HvGSK1.2	P$WRKY2_01	WRKY2	2390	2398	1	1	0.909	ttGTCAAa
HvGSK1.2	P$WRKY25_02	WRKY25	2390	2398	1	1	0.892	ttGTCAAa
HvGSK1.2	P$WRKY40_01	WRKY40	2390	2398	1	1	0.977	ttGTCAAa
HvGSK1.2	P$WRKY43_02	WRKY43	2390	2400	1	1	0.954	ttGTCAAaca
HvGSK1.2	P$WRKY62_01	WRKY62	2390	2398	1	1	0.895	ttGTCAAa
HvGSK1.2	P$WRKY63_01	WRKY63	2390	2398	1	1	0.886	ttGTCAAa
HvGSK1.2	P$WRKY75_01	WRKY75	2390	2398	1	1	0.936	ttGTCAAa
HvGSK1.2	P$WRKY8_01	WRKY8	2390	2399	1	1	0.98	ttGTCAAac
HvGSK1.2	P$WRKY30_01	WRKY30	2391	2401	1	1	0.901	tGTCAAacat
HvGSK1.2	P$WRKY18_Q2	WRKY18	2392	2401	1	1	0.931	GTCAAacat
HvGSK1.2	P$SEP3_01	wrz-03	2425	2436	1	1	0.865	gccaaTTTTGc
HvGSK1.2	P$RAV1_01	RAV1	2432	2444	1	1	0.994	ttgCAACAtaaa
HvGSK1.2	P$WRKY11_Q2	WRKY11	2448	2456	1	1	0.927	aTTGACtg
HvGSK1.2	P$ZAP1_01	ZAP1	2449	2459	1	1	0.866	TTGACtgcgt
HvGSK1.2	P$PCF2_01	CF2	2497	2507	1	1	0.883	atagcCCCAC
HvGSK1.2	P$TCP19_01	TCP19	2497	2507	1	1	0.859	atagcCCCAC
HvGSK1.2	P$TCP20L_01	TCP20L	2498	2507	1	1	0.857	tagcCCCAC
HvGSK1.2	P$TCP20_02	TCP20	2499	2509	1	1	0.902	agcCCCACat
HvGSK1.2	P$ARALY495258_02	ARALY495258	2499	2507	1	1	0.85	agcCCCAC
HvGSK1.2	P$ARALY484486_05	ARALY484486	2499	2507	1	1	0.85	agcCCCAC
HvGSK1.2	P$O2_Q4	O2	2502	2513	1	1	0.854	cccaCATGTaa
HvGSK1.2	P$PEND_01	END	2510	2518	1	1	0.92	tAAGAAga
HvGSK1.2	P$HBP1B_Q6	HBP1B	2512	2526	1	1	0.943	agaagacaCGTCAc
HvGSK1.2	P$ABF2_01	ABF2	2513	2526	1	1	0.951	gaagaCACGTcac
HvGSK1.2	P$O2_Q4	O2	2514	2525	1	1	0.955	aagaCACGTca
HvGSK1.2	P$BZR1_02	BZR1	2514	2528	1	1	0.858	aagaCACGTcaccc
HvGSK1.2	P$NAC92_01	NAC92	2515	2527	1	1	0.989	agACACGtcacc
HvGSK1.2	P$GBP_Q6	GBP	2515	2527	1	1	0.961	agaCACGTcacc
HvGSK1.2	P$ABI5_01	ABI5	2515	2525	1	1	0.957	agaCACGTca
HvGSK1.2	P$GBF6_01	GBF6	2515	2530	1	1	0.92	agaCACGTcacccat
HvGSK1.2	P$ABF4_01	ABF4	2515	2527	1	1	0.915	agaCACGTcacc
HvGSK1.2	P$EMBP1_Q2	EMBP1	2516	2526	1	1	0.927	gaCACGTcac
HvGSK1.2	P$CPRF3_Q2	CPRF3	2516	2526	1	1	0.974	gaCACGTcac
HvGSK1.2	P$CPRF2_Q2	CPRF2	2516	2526	1	1	0.966	gaCACGTcac
HvGSK1.2	P$O2_02	O2	2516	2526	1	1	0.953	gaCACGTcac
HvGSK1.2	P$TGA1B_Q2	TGA1B	2516	2526	1	1	0.996	gaCACGTcac
HvGSK1.2	P$TGA1A_Q2	TGA1A	2516	2526	1	1	0.989	gaCACGTcac
HvGSK1.2	P$CPRF3_01	CPRF3	2516	2526	1	1	0.983	gaCACGTcac
HvGSK1.2	P$CPRF2_01	CPRF2	2516	2526	1	1	0.966	gaCACGTcac
HvGSK1.2	P$TGA1B_01	TGA1B	2516	2526	1	1	0.992	gaCACGTcac
HvGSK1.2	P$BEE2_01	BEE2	2516	2526	1	1	0.906	gaCACGTcac
HvGSK1.2	P$BIM3_01	BIM3	2516	2526	1	1	0.876	gaCACGTcac
HvGSK1.2	P$PHYPA143875_02	HYPA143875	2516	2526	1	1	0.871	gaCACGTcac
HvGSK1.2	P$SPT_01	SPT	2516	2525	1	1	0.917	gaCACGTca
HvGSK1.2	P$GBF1F_Q2	GBF1F	2516	2527	1	1	0.897	gaCACGTcacc
HvGSK1.2	P$BZIP43_01	BZIP43	2516	2528	1	1	0.85	gaCACGTcaccc
HvGSK1.2	P$BZIP48_01	BZIP48	2516	2530	1	1	0.899	gaCACGTcacccat
HvGSK1.2	P$HBPA1_Q6_01	HBPA1	2516	2526	1	1	0.995	gacaCGTCAc
HvGSK1.2	P$RITA1_01	RITA1	2517	2524	1	1	0.963	aCACGTc
HvGSK1.2	P$OCSBF1_01	OCSBF1	2518	2523	1	1	1	CACGT
HvGSK1.2	P$TGA1A_01	TGA1A	2518	2525	1	1	0.989	cACGTCa
HvGSK1.2	P$TGA2_Q2	TGA2	2519	2529	1	1	0.897	aCGTCAccca
HvGSK1.2	P$O2_Q4	O2	2523	2534	1	1	0.874	caccCATGTat
HvGSK1.2	P$ASR1_01	ASR1	2524	2529	1	1	1	ACCCA
HvGSK1.2	P$TCP16_01	TCP16	2532	2542	1	1	0.885	atGGACCcgc
HvGSK1.2	P$KNOX3_01	KNOX3	2539	2551	1	1	0.964	cgctTGACAaca
HvGSK1.2	P$GAMYB_Q2	GAMYB	2540	2553	1	1	0.904	gcttgACAACatc
HvGSK1.2	P$WRKY11_Q2	WRKY11	2541	2549	1	1	0.921	cTTGACaa
HvGSK1.2	P$ATH1_01	ATH1	2543	2551	1	1	0.927	TGACAaca
HvGSK1.2	P$RAV1_01	RAV1	2543	2555	1	1	0.916	tgaCAACAtcca
HvGSK1.2	P$O2_Q4	O2	2549	2560	1	1	0.977	catcCATGTca
HvGSK1.2	P$DRE1C_01	DRE1C	2554	2562	1	1	0.862	ATGTCagc
HvGSK1.2	P$SBF1_01	SBF1	2557	2571	1	1	0.852	tcagcaTTAAAtta
HvGSK1.2	P$ML1_01	ML1	2559	2571	1	1	0.874	agcatTAAATta
HvGSK1.2	P$PDF2_01	DF2	2560	2571	1	1	0.892	gcatTAAATta
HvGSK1.2	P$AGL12_01	AGL12	2562	2574	1	1	0.861	attAAATTagat
HvGSK1.2	P$GATA9_01	GATA9	2567	2578	1	1	0.902	attAGATCcac
HvGSK1.2	P$AGP1_01	AGP1	2568	2578	1	1	0.931	ttAGATCcac
HvGSK1.2	P$GATA10_01	GATA10	2569	2577	1	1	0.884	tAGATCca
HvGSK1.2	P$ARR10_01	ARR10	2570	2577	1	1	0.934	AGATCca
HvGSK1.2	P$O2_Q4	O2	2573	2584	1	1	0.904	tccaCATGTca
HvGSK1.2	P$ATHB6_01	ATHB6	2599	2608	1	1	0.929	ccAATAAga
HvGSK1.2	P$RAV1_01	RAV1	2608	2620	1	1	0.943	catCAACAcaga
HvGSK1.2	P$BPC1_Q2	BPC1	2617	2623	1	1	0.99	AGAAAt

HvGSK1.2	P$ATHB6_01	ATHB6	2618	2627	1	1	0.9	gaAATAAca
HvGSK1.2	P$ATMYB15_Q2	ATMYB15	2622	2628	1	1	1	TAACAa
HvGSK1.2	P$PCF2_01	CF2	2631	2641	1	1	0.997	atggcCCCAC
HvGSK1.2	P$TCP19_01	TCP19	2631	2641	1	1	0.984	atggcCCCAC
HvGSK1.2	P$TCP20L_01	TCP20L	2632	2641	1	1	0.991	tggcCCCAC
HvGSK1.2	P$OSI_01	OSI	2633	2641	1	1	0.959	ggcCCCAC
HvGSK1.2	P$TCP20_02	TCP20	2633	2643	1	1	0.998	ggcCCCACat
HvGSK1.2	P$ARALY495258_02	ARALY495258	2633	2641	1	1	1	ggcCCCAC
HvGSK1.2	P$ARALY493022_04	ARALY493022	2633	2641	1	1	0.973	ggcCCCAC
HvGSK1.2	P$ARALY484486_05	ARALY484486	2633	2641	1	1	1	ggcCCCAC
HvGSK1.2	P$O2_Q4	O2	2636	2647	1	1	0.854	cccaCATGTaa
HvGSK1.2	P$AT4G36620_01	AT4G36620	2646	2654	1	1	0.907	agcAACCA
HvGSK1.2	P$GAMYB_01	GAMYB	2648	2656	1	1	0.878	CAACCatg
HvGSK1.2	P$O2_Q4	O2	2648	2659	1	1	0.941	caacCATGTca
HvGSK1.2	P$DRE1C_01	DRE1C	2653	2661	1	1	0.862	ATGTCagc
HvGSK1.2	P$ARR2_01	ARR2	2657	2667	1	1	0.96	cagcATCTTc
HvGSK1.2	P$WRKY18_02	WRKY18	2675	2685	1	1	0.945	tatGTCAAta
HvGSK1.2	P$WRKY21_02	WRKY21	2675	2685	1	1	0.949	tatGTCAAta
HvGSK1.2	P$WRKY48_02	WRKY48	2675	2685	1	1	0.986	tatGTCAAta
HvGSK1.2	P$WRKY57_01	WRKY57	2675	2685	1	1	0.96	tatGTCAAta
HvGSK1.2	P$WRKY60_01	WRKY60	2675	2686	1	1	0.882	tatGTCAAtag
HvGSK1.2	P$WRKY15_01	WRKY15	2676	2686	1	1	0.959	atGTCAAtag
HvGSK1.2	P$WRKY2_01	WRKY2	2676	2684	1	1	0.902	atGTCAAt
HvGSK1.2	P$WRKY25_02	WRKY25	2676	2684	1	1	0.879	atGTCAAt
HvGSK1.2	P$WRKY40_01	WRKY40	2676	2684	1	1	0.977	atGTCAAt
HvGSK1.2	P$WRKY43_02	WRKY43	2676	2686	1	1	0.954	atGTCAAtag
HvGSK1.2	P$WRKY63_01	WRKY63	2676	2684	1	1	0.885	atGTCAAt
HvGSK1.2	P$WRKY75_01	WRKY75	2676	2684	1	1	0.927	atGTCAAt
HvGSK1.2	P$WRKY8_01	WRKY8	2676	2685	1	1	0.977	atGTCAAta
HvGSK1.2	P$WRKY30_01	WRKY30	2677	2687	1	1	0.904	tGTCAAtagg
HvGSK1.2	P$WRKY18_Q2	WRKY18	2678	2687	1	1	0.94	GTCAAtagg
HvGSK1.2	P$WRKY11_Q2	WRKY11	2687	2695	1	1	0.974	aTTGACcg
HvGSK1.2	P$ZAP1_01	ZAP1	2688	2698	1	1	0.933	TTGACcggtc
HvGSK1.2	P$WRKY18_02	WRKY18	2692	2702	1	1	0.998	ccgGTCAAag
HvGSK1.2	P$WRKY21_02	WRKY21	2692	2702	1	1	0.972	ccgGTCAAag
HvGSK1.2	P$WRKY48_02	WRKY48	2692	2702	1	1	0.998	ccgGTCAAag
HvGSK1.2	P$WRKY57_01	WRKY57	2692	2702	1	1	0.973	ccgGTCAAag
HvGSK1.2	P$WRKY60_01	WRKY60	2692	2703	1	1	0.983	ccgGTCAAagc
HvGSK1.2	P$WRKY15_01	WRKY15	2693	2703	1	1	0.987	cgGTCAAagc
HvGSK1.2	P$WRKY2_01	WRKY2	2693	2701	1	1	0.991	cgGTCAAa
HvGSK1.2	P$WRKY25_02	WRKY25	2693	2701	1	1	0.979	cgGTCAAa
HvGSK1.2	P$WRKY40_01	WRKY40	2693	2701	1	1	1	cgGTCAAa
HvGSK1.2	P$WRKY43_02	WRKY43	2693	2703	1	1	0.977	cgGTCAAagc
HvGSK1.2	P$WRKY62_01	WRKY62	2693	2701	1	1	0.91	cgGTCAAa
HvGSK1.2	P$WRKY63_01	WRKY63	2693	2701	1	1	0.991	cgGTCAAa
HvGSK1.2	P$WRKY75_01	WRKY75	2693	2701	1	1	0.975	cgGTCAAa
HvGSK1.2	P$WRKY8_01	WRKY8	2693	2702	1	1	0.993	cgGTCAAag
HvGSK1.2	P$WRKY23_01	WRKY23	2694	2702	1	1	0.893	gGTCAAag
HvGSK1.2	P$WRKY30_01	WRKY30	2694	2704	1	1	0.924	gGTCAAagct
HvGSK1.2	P$DOF2_01	DOF2	2694	2705	1	1	0.98	ggtcAAAGCtg
HvGSK1.2	P$DOF3_01	DOF3	2694	2705	1	1	0.98	ggtcAAAGCtg
HvGSK1.2	P$WRKY18_Q2	WRKY18	2695	2704	1	1	0.95	GTCAAagct
HvGSK1.2	P$O2_Q2	O2	2699	2712	1	1	0.969	aagctGACGTcga
HvGSK1.2	P$TGA1_01	TGA1	2700	2711	1	1	0.986	agcTGACGtcg
HvGSK1.2	P$TGA7_02	TGA7	2700	2717	1	1	0.855	agctgACGTCgacttat
HvGSK1.2	P$STF1_02	STF1	2701	2713	1	1	0.958	gcTGACGtcgac
HvGSK1.2	P$TGA3_Q2	TGA3	2701	2710	1	1	0.953	gcTGACGtc
HvGSK1.2	P$TGA6_01	TGA6	2701	2711	1	1	0.975	gcTGACGtcg
HvGSK1.2	P$TGA7_01	TGA7	2701	2711	1	1	0.949	gcTGACGtcg
HvGSK1.2	P$TGA6_02	TGA6	2701	2716	1	1	0.855	gcTGACGtcgactta
HvGSK1.2	P$BZIP14_01	BZIP14	2701	2711	1	1	0.955	gcTGACGtcg
HvGSK1.2	P$STF1_01	STF1	2701	2713	1	1	0.977	gctGACGTcgac
HvGSK1.2	P$TGA5_01	TGA5	2702	2710	1	1	0.993	cTGACGtc
HvGSK1.2	P$TGA1B_01	TGA1B	2702	2712	1	1	0.879	ctGACGTcga
HvGSK1.2	P$TGA1A_01	TGA1A	2704	2711	1	1	0.857	gACGTCg
HvGSK1.2	P$AT1G68550_03	AT1G68550	2729	2738	1	1	0.949	cctCGGCGa
HvGSK1.2	P$GAMYB_Q2	GAMYB	2734	2747	1	1	0.887	gcgatACAACcta
HvGSK1.2	P$GAMYB_01	GAMYB	2740	2748	1	1	0.868	CAACCtag
HvGSK1.2	P$PCF2_01	CF2	2749	2759	1	1	0.882	gcagaCCCAC
HvGSK1.2	P$TCP19_01	TCP19	2749	2759	1	1	0.857	gcagaCCCAC
HvGSK1.2	P$TCP20L_01	TCP20L	2750	2759	1	1	0.852	cagaCCCAC
HvGSK1.2	P$TCP20_02	TCP20	2751	2761	1	1	0.895	agaCCCACat
HvGSK1.2	P$ASR1_01	ASR1	2753	2758	1	1	1	ACCCA
HvGSK1.2	P$DOF2_01	DOF2	2758	2769	1	1	0.986	catgAAAGCaa
HvGSK1.2	P$DOF3_01	DOF3	2758	2769	1	1	0.987	catgAAAGCaa
HvGSK1.2	P$AT4G36620_01	AT4G36620	2764	2772	1	1	0.907	agcAACCA
HvGSK1.2	P$HBP1B_Q6	HBP1B	2764	2778	1	1	0.899	agcaaccaCGTCAg
HvGSK1.2	P$ABF2_01	ABF2	2765	2778	1	1	0.904	gcaacCACGTcag

HvGSK1.2	P$GAMYB_01	GAMYB	2766	2774	1	1	0.888	CAACCacg
HvGSK1.2	P$O2_Q4	O2	2766	2777	1	1	0.941	caacCACGTca
HvGSK1.2	P$BZR1_02	BZR1	2766	2780	1	1	0.868	caacCACGTcagta
HvGSK1.2	P$GBP_Q6	GBP	2767	2779	1	1	0.902	aacCACGTcagt
HvGSK1.2	P$ABI5_01	ABI5	2767	2777	1	1	0.906	aacCACGTca
HvGSK1.2	P$GBF6_01	GBF6	2767	2782	1	1	0.889	aacCACGTcagtaat
HvGSK1.2	P$ABF4_01	ABF4	2767	2779	1	1	0.884	aacCACGTcagt
HvGSK1.2	P$EMBP1_Q2	EMBP1	2768	2778	1	1	0.892	acCACGTcag
HvGSK1.2	P$CPRF3_Q2	CPRF3	2768	2778	1	1	0.963	acCACGTcag
HvGSK1.2	P$CPRF2_Q2	CPRF2	2768	2778	1	1	0.968	acCACGTcag
HvGSK1.2	P$O2_02	O2	2768	2778	1	1	0.977	acCACGTcag
HvGSK1.2	P$TGA1B_Q2	TGA1B	2768	2778	1	1	0.95	acCACGTcag
HvGSK1.2	P$TGA1A_Q2	TGA1A	2768	2778	1	1	0.989	acCACGTcag
HvGSK1.2	P$CPRF3_01	CPRF3	2768	2778	1	1	0.976	acCACGTcag
HvGSK1.2	P$CPRF2_01	CPRF2	2768	2778	1	1	0.969	acCACGTcag
HvGSK1.2	P$TGA1B_01	TGA1B	2768	2778	1	1	0.898	acCACGTcag
HvGSK1.2	P$BEE2_01	BEE2	2768	2778	1	1	0.906	acCACGTcag
HvGSK1.2	P$BIM3_01	BIM3	2768	2778	1	1	0.876	acCACGTcag
HvGSK1.2	P$PHYPA143875_02	HYPA143875	2768	2778	1	1	0.871	acCACGTcag
HvGSK1.2	P$SPT_01	SPT	2768	2777	1	1	0.944	acCACGTca
HvGSK1.2	P$GBF1F_Q2	GBF1F	2768	2779	1	1	0.918	acCACGTcagt
HvGSK1.2	P$BZIP43_01	BZIP43	2768	2780	1	1	0.885	acCACGTcagta
HvGSK1.2	P$BZIP48_01	BZIP48	2768	2782	1	1	0.895	acCACGTcagtaat
HvGSK1.2	P$HBPA1_Q6_01	HBPA1	2768	2778	1	1	0.921	accaCGTCAg
HvGSK1.2	P$RITA1_01	RITA1	2769	2776	1	1	0.976	cCACGTc
HvGSK1.2	P$OCSBF1_01	OCSBF1	2770	2775	1	1	1	CACGT
HvGSK1.2	P$TGA1A_01	TGA1A	2770	2777	1	1	0.989	cACGTCa
HvGSK1.2	P$TCP11_01	TCP11	2782	2794	1	1	0.998	tGTGGGccccat
HvGSK1.2	P$TCP15_01	TCP15	2783	2793	1	1	1	GTGGGcccca
HvGSK1.2	P$TCP20_01	TCP20	2783	2793	1	1	0.99	GTGGGcccca
HvGSK1.2	P$TCP11_02	TCP11	2783	2793	1	1	0.998	GTGGGcccca
HvGSK1.2	P$TCP7_01	TCP7	2783	2794	1	1	0.991	GTGGGccccat
HvGSK1.2	P$OJ1581_01	OJ1581	2783	2793	1	1	0.994	gtGGGCCcca
HvGSK1.2	P$TCP2_01	TCP2	2783	2793	1	1	0.974	gtGGGCCcca
HvGSK1.2	P$ARR1_01	ARR1	2797	2807	1	1	0.953	tgaGAATCat
HvGSK1.2	P$ATHB7_01	ATHB7	2799	2809	1	1	0.922	agAATCAttg
HvGSK1.2	P$HAT1_01	HAT1	2799	2809	1	1	0.981	agAATCAttg
HvGSK1.2	P$ATHB4_02	ATHB4	2800	2810	1	1	0.96	gaATCATtga
HvGSK1.2	P$GATA15_01	GATA15	2805	2814	1	1	0.999	atTGATCgg
HvGSK1.2	P$WRKY18_02	WRKY18	2810	2820	1	1	0.997	tcgGTCAAac
HvGSK1.2	P$WRKY21_02	WRKY21	2810	2820	1	1	0.969	tcgGTCAAac
HvGSK1.2	P$WRKY48_02	WRKY48	2810	2820	1	1	0.998	tcgGTCAAac
HvGSK1.2	P$WRKY57_01	WRKY57	2810	2820	1	1	0.971	tcgGTCAAac
HvGSK1.2	P$WRKY60_01	WRKY60	2810	2821	1	1	0.98	tcgGTCAAacc
HvGSK1.2	P$WRKY15_01	WRKY15	2811	2821	1	1	0.985	cgGTCAAacc
HvGSK1.2	P$WRKY2_01	WRKY2	2811	2819	1	1	0.991	cgGTCAAa
HvGSK1.2	P$WRKY25_02	WRKY25	2811	2819	1	1	0.979	cgGTCAAa
HvGSK1.2	P$WRKY40_01	WRKY40	2811	2819	1	1	1	cgGTCAAa
HvGSK1.2	P$WRKY43_02	WRKY43	2811	2821	1	1	0.976	cgGTCAAacc
HvGSK1.2	P$WRKY62_01	WRKY62	2811	2819	1	1	0.91	cgGTCAAa
HvGSK1.2	P$WRKY63_01	WRKY63	2811	2819	1	1	0.991	cgGTCAAa
HvGSK1.2	P$WRKY75_01	WRKY75	2811	2819	1	1	0.975	cgGTCAAa
HvGSK1.2	P$WRKY8_01	WRKY8	2811	2820	1	1	0.992	cgGTCAAac
HvGSK1.2	P$WRKY23_01	WRKY23	2812	2820	1	1	0.854	gGTCAAac
HvGSK1.2	P$WRKY30_01	WRKY30	2812	2822	1	1	0.918	gGTCAAaccc
HvGSK1.2	P$WRKY18_Q2	WRKY18	2813	2822	1	1	0.935	GTCAAaccc
HvGSK1.2	P$RAV2_01	RAV2	2838	2847	1	1	0.859	tgACCGAtt
HvGSK1.2	P$TGA2_Q2	TGA2	2848	2858	1	1	0.899	tCGTCAtaaa
HvGSK1.2	P$ARR2_01	ARR2	2859	2869	1	1	0.884	aacaATCTTa
HvGSK1.2	P$GAMYB_Q2	GAMYB	2865	2878	1	1	0.921	cttagACAACata
HvGSK1.2	P$RAV1_01	RAV1	2868	2880	1	1	0.957	agaCAACAtatt
HvGSK1.2	P$KNOX3_01	KNOX3	2889	2901	1	1	0.979	tagcTGACAttc
HvGSK1.2	P$AT3G62240_01	AT3G62240	2891	2901	1	1	0.871	gctgaCATTC
HvGSK1.2	P$ATH1_01	ATH1	2893	2901	1	1	0.92	TGACAttc
HvGSK1.2	P$TGA2_Q2	TGA2	2905	2915	1	1	0.896	gCGTCAccaa
HvGSK1.2	P$AP3_01	AP3	2909	2924	1	1	0.897	cacCAAAAatggtca
HvGSK1.2	P$MADSB_Q2	MADSB	2909	2924	1	1	0.865	caccAAAAAtggtca
HvGSK1.2	P$AGL9_01	AGL9	2909	2924	1	1	0.953	caccaaaAATGGtca
HvGSK1.2	P$HSFA2_01	HSFA2	2911	2917	1	1	1	CCAAAa
HvGSK1.2	P$AP1_01	AP1	2911	2924	1	1	0.873	ccAAAAAtggtca
HvGSK1.2	P$PHV_02	HV	2920	2935	1	1	0.89	gtcATCATtgccaat
HvGSK1.2	P$ATHB4_02	ATHB4	2921	2931	1	1	0.914	tcATCATtgc
HvGSK1.2	P$SOC1_01	SOC1	2925	2940	1	1	0.908	cattgccaaTTTTGg
HvGSK1.2	P$AGL1_02	AGL1	2926	2944	1	1	0.869	aTTGCCaattttgggttc
HvGSK1.2	P$SEP3_01	wrz-03	2929	2940	1	1	0.947	gccaaTTTTGg
HvGSK1.2	P$GATA15_01	GATA15	2955	2964	1	1	1	caTGATCat
HvGSK1.2	P$LEC2_01	LEC2	2959	2970	1	1	0.956	atCATGCgtga
HvGSK1.2	P$KNOX3_01	KNOX3	2963	2975	1	1	0.984	tgcgTGACAcag

HvGSK1.2	P$ATH1_01	ATH1	2967	2975	1	1	0.904	TGACAcag
HvGSK1.2	P$WRKY18_02	WRKY18	2983	2993	1	1	0.946	atcGTCAAta
HvGSK1.2	P$WRKY21_02	WRKY21	2983	2993	1	1	0.948	atcGTCAAta
HvGSK1.2	P$WRKY48_02	WRKY48	2983	2993	1	1	0.987	atcGTCAAta
HvGSK1.2	P$WRKY57_01	WRKY57	2983	2993	1	1	0.957	atcGTCAAta
HvGSK1.2	P$WRKY60_01	WRKY60	2983	2994	1	1	0.889	atcGTCAAtat
HvGSK1.2	P$WRKY15_01	WRKY15	2984	2994	1	1	0.96	tcGTCAAtat
HvGSK1.2	P$WRKY2_01	WRKY2	2984	2992	1	1	0.905	tcGTCAAt
HvGSK1.2	P$WRKY25_02	WRKY25	2984	2992	1	1	0.888	tcGTCAAt
HvGSK1.2	P$WRKY40_01	WRKY40	2984	2992	1	1	0.981	tcGTCAAt
HvGSK1.2	P$WRKY43_02	WRKY43	2984	2994	1	1	0.955	tcGTCAAtat
HvGSK1.2	P$WRKY62_01	WRKY62	2984	2992	1	1	0.87	tcGTCAAt
HvGSK1.2	P$WRKY63_01	WRKY63	2984	2992	1	1	0.886	tcGTCAAt
HvGSK1.2	P$WRKY75_01	WRKY75	2984	2992	1	1	0.928	tcGTCAAt
HvGSK1.2	P$WRKY8_01	WRKY8	2984	2993	1	1	0.978	tcGTCAAta
HvGSK1.2	P$WRKY30_01	WRKY30	2985	2995	1	1	0.907	cGTCAAtatc
HvGSK1.2	P$WRKY18_Q2	WRKY18	2986	2995	1	1	0.964	GTCAAtatc
HvGSK1.2	P$ARR2_01	ARR2	2988	2998	1	1	0.903	caatATCTTa
HvGSK1.2	P$GT1_Q6_01	GT1	2998	3010	1	1	0.855	TTTTTgataatg
HvGSK1.2	P$ALFIN1_Q2	ALFIN1	3018	3033	1	1	0.942	gtgattGTGGGggtg
HvGSK1.2	P$DREB1A_04	DREB1A	3034	3044	1	1	0.931	tgGTCGGaaa
HvGSK1.2	P$ERF039_01	ERF039	3034	3044	1	1	0.942	tgGTCGGaaa
HvGSK1.2	P$PHYPA28324_10	HYPA28324	3035	3043	1	1	0.865	gGTCGGaa
HvGSK1.2	P$AT3G51080_01	AT3G51080	3039	3046	1	1	0.89	GGAAAac
HvGSK1.2	P$TGA1A_01	TGA1A	3043	3050	1	1	0.871	aACGTCt
HvGSK1.2	P$RIN_Q2	RIN	3075	3086	1	1	0.867	tgttTTTAAgt
HvGSK1.2	P$AT4G36620_01	AT4G36620	3132	3140	1	1	0.884	cgcAACCA
HvGSK1.2	P$GAMYB_01	GAMYB	3134	3142	1	1	0.879	CAACCaat
HvGSK1.2	P$LEC2_01	LEC2	3141	3152	1	1	0.994	tcCATGCacct
HvGSK1.2	P$ROM_Q2	ROM	3145	3154	1	1	0.861	tgCACCTca
HvGSK1.2	P$CBF3_02	CBF3	3157	3171	1	1	0.959	gtcagCCGACgatg
HvGSK1.2	P$CBF1_04	CBF1	3158	3170	1	1	0.954	tcagCCGACgat
HvGSK1.2	P$ERF5_02	ERF5	3159	3169	1	1	0.94	caGCCGAcga
HvGSK1.2	P$ERF1_04	ERF1	3159	3169	1	1	0.934	caGCCGAcga
HvGSK1.2	P$DREB1G_02	DREB1G	3159	3169	1	1	0.9	cagCCGACga
HvGSK1.2	P$ARF1_01	ARF1	3161	3169	1	1	0.855	gCCGACga
HvGSK1.2	P$ARF5_01	ARF5	3161	3169	1	1	0.903	gCCGACga
HvGSK1.2	P$DREB1B_01	DREB1B	3162	3167	1	1	1	CCGAC
HvGSK1.2	P$HSFA4A_01	HSFA4A	3189	3195	1	1	0.91	gCTATT
HvGSK1.2	P$O2_Q4	O2	3215	3226	1	1	0.885	tcccCATGTct
HvGSK1.2	P$LIM1_01	LIM1	3243	3255	1	1	0.958	CCACCacaacaa
HvGSK1.2	P$GAMYB_Q2	GAMYB	3243	3256	1	1	0.912	ccaccACAACaac
HvGSK1.2	P$RAV1_01	RAV1	3246	3258	1	1	0.91	ccaCAACAaccc
HvGSK1.2	P$GAMYB_Q2	GAMYB	3246	3259	1	1	0.931	ccacaACAACccg
HvGSK1.2	P$GAMYB_01	GAMYB	3252	3260	1	1	0.865	CAACCcgg
HvGSK1.2	P$ATSPL8_01	ATSPL8	3257	3273	1	1	0.884	cggtaTGTACgcgcct
HvGSK1.2	P$SPL11_01	SPL11	3259	3271	1	1	0.884	gtatGTACGcgc
HvGSK1.2	P$FAR1_01	FAR1	3260	3275	1	1	0.886	tatgtACGCGcctcg
HvGSK1.2	P$SPL5_01	SPL5	3261	3270	1	1	0.963	atGTACGcg
HvGSK1.2	P$POPTR_01	OPTR	3262	3269	1	1	0.928	tGTACGc
HvGSK1.2	P$SPL12_01	SPL12	3262	3270	1	1	0.97	tGTACGcg
HvGSK1.2	P$SPL4_01	SPL4	3262	3271	1	1	0.988	tGTACGcgc
HvGSK1.2	P$FHY3_01	FHY3	3262	3274	1	1	0.857	tgtACGCGcctc
HvGSK1.2	P$TRAB1_Q2	TRAB1	3263	3274	1	1	0.907	gtACGCGcctc
HvGSK1.2	P$C1_Q2	C1	3273	3284	1	1	0.956	cgAACTAggcc
HvGSK1.2	P$CBF1_01	CBF1	3287	3297	1	1	0.927	cTGCCGctgg
HvGSK1.2	P$ERF019_01	ERF019	3287	3297	1	1	0.928	cTGCCGctgg
HvGSK1.2	P$DREBIII4_01	DREBIII4	3287	3297	1	1	0.894	cTGCCGctgg
HvGSK1.2	P$JERF1_01	JERF1	3287	3297	1	1	0.887	cTGCCGctgg
HvGSK1.2	P$CBF1_03	CBF1	3287	3297	1	1	0.938	cTGCCGctgg
HvGSK1.2	P$DREB1F_01	DREB1F	3287	3297	1	1	0.874	cTGCCGctgg
HvGSK1.2	P$AT1G33760_01	AT1G33760	3287	3297	1	1	0.899	cTGCCGctgg
HvGSK1.2	P$AT1G71520_01	AT1G71520	3287	3297	1	1	0.894	cTGCCGctgg
HvGSK1.2	P$ORA47_01	ORA47	3287	3297	1	1	0.988	cTGCCGctgg
HvGSK1.2	P$ABI4_03	ABI4	3287	3297	1	1	0.861	ctGCCGCtgg
HvGSK1.2	P$DREBI5_01	DREBI5	3287	3297	1	1	0.921	ctGCCGCtgg
HvGSK1.2	P$DREB2E_01	DREB2E	3287	3298	1	1	0.928	ctGCCGCtgga
HvGSK1.2	P$AT1G77200_01	AT1G77200	3287	3297	1	1	0.973	ctGCCGCtgg
HvGSK1.2	P$ATERF14_01	ATERF14	3287	3297	1	1	0.853	ctGCCGCtgg
HvGSK1.2	P$DREBIII3_01	DREBIII3	3287	3297	1	1	0.953	ctGCCGCtgg
HvGSK1.2	P$DREBIII2_01	DREBIII2	3287	3297	1	1	0.952	ctGCCGCtgg
HvGSK1.2	P$DREBIII1_01	DREBIII1	3287	3297	1	1	0.952	ctGCCGCtgg
HvGSK1.2	P$CBF3_01	CBF3	3287	3297	1	1	0.878	ctGCCGCtgg
HvGSK1.2	P$DREBII1_01	DREBII1	3287	3297	1	1	0.919	ctGCCGCtgg
HvGSK1.2	P$AT2G44940_01	AT2G44940	3287	3297	1	1	0.96	ctGCCGCtgg
HvGSK1.2	P$DBF2_01	DBF2	3287	3297	1	1	0.982	ctGCCGCtgg
HvGSK1.2	P$CBF5_01	CBF5	3287	3297	1	1	0.919	ctGCCGCtgg
HvGSK1.2	P$CBF16_01	CBF16	3287	3297	1	1	0.92	ctGCCGCtgg

HvGSK1.2	P$CBF17_01	CBF17	3287	3297	1	1	0.92	ctGCCGCtgg
HvGSK1.2	P$CBF_01	CBF	3287	3297	1	1	0.92	ctGCCGCtgg
HvGSK1.2	P$ERF016_01	ERF016	3287	3297	1	1	0.927	ctGCCGCtgg
HvGSK1.2	P$AT3G61630_01	AT3G61630	3287	3297	1	1	0.853	ctGCCGCtgg
HvGSK1.2	P$AT5G43410_01	AT5G43410	3287	3297	1	1	0.86	ctGCCGCtgg
HvGSK1.2	P$TINY2_02	TINY2	3287	3297	1	1	0.985	ctGCCGCtgg
HvGSK1.2	P$AT3G16280_01	AT3G16280	3287	3297	1	1	0.937	ctGCCGCtgg
HvGSK1.2	P$DREB1A_03	DREB1A	3287	3297	1	1	0.877	ctGCCGCtgg
HvGSK1.2	P$CRF2_01	CRF2	3287	3295	1	1	0.887	ctGCCGCt
HvGSK1.2	P$ERF098_01	ERF098	3287	3295	1	1	0.892	ctGCCGCt
HvGSK1.2	P$ERF7_02	ERF7	3288	3298	1	1	0.938	tGCCGCtgga
HvGSK1.2	P$ARR1_01	ARR1	3293	3303	1	1	0.943	ctgGAATCcg
HvGSK1.2	P$HSF3_01	HSF3	3307	3313	1	1	0.969	aCGGGG
HvGSK1.2	P$ERF112_02	ERF112	3401	3411	1	1	0.919	tcCGCCGgtg
HvGSK1.2	P$AT3G63350_01	AT3G63350	3402	3408	1	1	0.866	CCGCCg
HvGSK1.2	P$CRF4_01	CRF4	3402	3410	1	1	0.861	cCGCCGgt
HvGSK1.2	P$ERF4_04	ERF4	3402	3410	1	1	0.869	cCGCCGgt
HvGSK1.2	P$ERF069_01	ERF069	3402	3411	1	1	0.989	cCGCCGgtg
HvGSK1.2	P$ERF11_01	ERF11	3402	3412	1	1	0.965	cCGCCGgtgg
HvGSK1.2	P$RAP21_02	RAP21	3402	3415	1	1	0.917	ccgcCGGTGggtc
HvGSK1.2	P$ERF8_01	ERF8	3403	3413	1	1	0.94	CGCCGgtggg
HvGSK1.2	P$TCP11_01	TCP11	3407	3419	1	1	0.85	gGTGGGtctcct
HvGSK1.2	P$ALFIN1_Q2	ALFIN1	3413	3428	1	1	0.918	tctcctGTGGGgtag
HvGSK1.2	P$PIL5_01	IL5	3422	3436	1	1	0.881	gggtagagACGTGa
HvGSK1.2	P$ABZ1_01	ABZ1	3425	3439	1	1	0.895	tagagACGTGatcg
HvGSK1.2	P$STF1_01	STF1	3426	3438	1	1	0.855	agaGACGTgatc
HvGSK1.2	P$TGA1B_01	TGA1B	3427	3437	1	1	0.954	gaGACGTgat
HvGSK1.2	P$HBP1A_Q2	HBP1A	3427	3437	1	1	0.896	gagACGTGat
HvGSK1.2	P$TAF1_Q2	TAF1	3427	3437	1	1	0.942	gagACGTGat
HvGSK1.2	P$EMBP1_02	EMBP1	3427	3437	1	1	0.908	gagACGTGat
HvGSK1.2	P$TAF1_01	TAF1	3427	3437	1	1	0.957	gagACGTGat
HvGSK1.2	P$GBF1_01	GBF1	3428	3436	1	1	0.934	agACGTGa
HvGSK1.2	P$BIM1_02	BIM1	3428	3438	1	1	0.948	agACGTGatc
HvGSK1.2	P$ABF4_02	ABF4	3428	3438	1	1	0.949	agACGTGatc
HvGSK1.2	P$ABI5_Q2	ABI5	3430	3436	1	1	0.936	ACGTGa
HvGSK1.2	P$GATA15_01	GATA15	3431	3440	1	1	0.999	cgTGATCgc
HvGSK1.2	P$PCF5_01	CF5	3456	3466	1	1	0.884	tgGGTCCctg
HvGSK1.2	P$GT1_Q6	GT1	3505	3512	1	1	0.971	GTGAAaa
HvGSK1.2	P$GATA9_01	GATA9	3516	3527	1	1	0.887	ataAGATCaat
HvGSK1.2	P$AGP1_01	AGP1	3517	3527	1	1	0.907	taAGATCaat
HvGSK1.2	P$ARR10_01	ARR10	3519	3526	1	1	0.869	AGATCaa
HvGSK1.2	P$GT1_Q6_01	GT1	3543	3555	1	1	0.885	TTTTTttttgag
HvGSK1.2	P$GT1_Q6_01	GT1	3544	3556	1	1	0.919	TTTTTtttgaga
HvGSK1.2	P$BPC1_Q2	BPC1	3553	3559	1	1	0.997	AGAAAa
HvGSK1.2	P$CBNAC_01	CBNAC	3560	3566	1	1	1	tTGCTT
HvGSK1.2	P$CBNAC_02	CBNAC	3560	3576	1	1	0.94	tTGCTTaaccaacaaa
HvGSK1.2	P$AT1G66560_01	AT1G66560	3561	3571	1	1	0.911	tgcTTAACca
HvGSK1.2	P$WRKY21_01	WRKY21	3562	3571	1	1	0.919	gcTTAACca
HvGSK1.2	P$AT1G18860_01	AT1G18860	3562	3571	1	1	0.91	gcTTAACca
HvGSK1.2	P$AT1G64000_01	AT1G64000	3562	3571	1	1	0.922	gcTTAACca
HvGSK1.2	P$WRKY6_01	WRKY6	3562	3571	1	1	0.873	gcTTAACca
HvGSK1.2	P$AT1G66600_01	AT1G66600	3562	3571	1	1	0.911	gcTTAACca
HvGSK1.2	P$AT1G68150_01	AT1G68150	3562	3571	1	1	0.898	gcTTAACca
HvGSK1.2	P$AT5G41570_01	AT5G41570	3562	3571	1	1	0.922	gcTTAACca
HvGSK1.2	P$AT5G15130_01	AT5G15130	3562	3571	1	1	0.905	gcTTAACca
HvGSK1.2	P$WRKY46_01	WRKY46	3562	3571	1	1	0.868	gcTTAACca
HvGSK1.2	P$AT2G24570_01	AT2G24570	3562	3571	1	1	0.915	gcTTAACca
HvGSK1.2	P$WRKY7_01	WRKY7	3562	3571	1	1	0.924	gcTTAACca
HvGSK1.2	P$WRKY25_01	WRKY25	3562	3571	1	1	0.942	gctTAACCa
HvGSK1.2	P$WRKY33_01	WRKY33	3562	3571	1	1	0.877	gctTAACCa
HvGSK1.2	P$AT1G29860_01	AT1G29860	3562	3571	1	1	0.904	gctTAACCa
HvGSK1.2	P$AT3G62340_01	AT3G62340	3562	3571	1	1	0.888	gctTAACCa
HvGSK1.2	P$AT1G69310_01	AT1G69310	3562	3571	1	1	0.914	gctTAACCa
HvGSK1.2	P$WRKY26_01	WRKY26	3562	3571	1	1	0.88	gctTAACCa
HvGSK1.2	P$GT1_01	GT1	3563	3571	1	1	0.968	ctTAACCa
HvGSK1.2	P$AT4G36620_01	AT4G36620	3563	3571	1	1	0.966	cttAACCA
HvGSK1.2	P$MYBAS1_01	MYBAS1	3566	3577	1	1	0.948	aaCCAACaaaa
HvGSK1.2	P$RAV1_01	RAV1	3566	3578	1	1	0.967	aacCAACAaaag
HvGSK1.2	P$RIN_Q2_01	RIN	3567	3579	1	1	0.894	accaacAAAAGg
HvGSK1.2	P$PBF_01	BF	3570	3581	1	1	0.98	aacAAAAGggt
HvGSK1.2	P$DOF_Q2	DOF	3570	3581	1	1	0.928	aacAAAAGggt
HvGSK1.2	P$CDF2_01	CDF2	3571	3581	1	1	0.954	acAAAAGggt
HvGSK1.2	P$CDF3_01	CDF3	3572	3581	1	1	0.981	cAAAAGggt
HvGSK1.2	P$PBF_Q2	BF	3573	3579	1	1	1	aAAAGG
HvGSK1.2	P$SED_Q2	SED	3590	3600	1	1	0.897	tattCCTTTa
HvGSK1.2	P$PBF_Q2_01	BF	3594	3600	1	1	0.998	CCTTTa
HvGSK1.2	P$O2_03	O2	3613	3623	1	1	0.93	GATGAattgg
HvGSK1.2	P$BZR1_01	BZR1	3625	3631	1	1	0.915	CGTGCa

HvGSK1.2	P$O2_Q4	O2	3625	3636	1	1	0.863	cgtgCATGTga
HvGSK1.2	P$ABI3_01	ABI3	3626	3635	1	1	0.858	gtGCATGtg
HvGSK1.2	P$AMS_01	AMS	3627	3637	1	1	0.894	tgCATGTgag
HvGSK1.2	P$AT5G54070_01	AT5G54070	3642	3648	1	1	0.91	tCAACG
HvGSK1.2	P$HSFA4A_01	HSFA4A	3669	3675	1	1	1	aCTATT
HvGSK1.2	P$SQUA_01	SQUA	3670	3680	1	1	0.9	ctaTTTTTtc
HvGSK1.2	P$ABI3_01	ABI3	3691	3700	1	1	0.98	ttGCATGca
HvGSK1.2	P$FUS3_01	FUS3	3692	3701	1	1	0.965	tGCATGcaa
HvGSK1.2	P$LEC2_01	LEC2	3692	3703	1	1	0.982	tgCATGCaacg
HvGSK1.2	P$FUS3_Q2	FUS3	3693	3704	1	1	0.851	gCATGCaacga
HvGSK1.2	P$AT5G54070_01	AT5G54070	3697	3703	1	1	0.915	gCAACG
HvGSK1.2	P$DREB1A_04	DREB1A	3748	3758	1	1	0.97	tcGTCGGcaa
HvGSK1.2	P$ERF039_01	ERF039	3748	3758	1	1	0.982	tcGTCGGcaa
HvGSK1.2	P$PHYPA28324_10	HYPA28324	3749	3757	1	1	0.879	cGTCGGca
HvGSK1.2	P$MADSB_Q2	MADSB	3779	3794	1	1	0.858	gccaAAAAAtcaaaa
HvGSK1.2	P$HSFA2_01	HSFA2	3780	3786	1	1	1	CCAAAa
HvGSK1.2	P$ATHB7_01	ATHB7	3784	3794	1	1	0.873	aaAATCAaaa
HvGSK1.2	P$HAT1_01	HAT1	3784	3794	1	1	0.865	aaAATCAaaa
HvGSK1.2	P$AP1_01	AP1	3819	3832	1	1	0.896	acAAAAAaagata
HvGSK1.2	P$PBF_01	BF	3821	3832	1	1	0.974	aaaAAAAGata
HvGSK1.2	P$DOF_Q2	DOF	3821	3832	1	1	0.984	aaaAAAAGata
HvGSK1.2	P$CDF2_01	CDF2	3822	3832	1	1	0.974	aaAAAAGata
HvGSK1.2	P$CDF3_01	CDF3	3823	3832	1	1	0.974	aAAAAGata
HvGSK1.2	P$ARR18_01	ARR18	3823	3836	1	1	0.912	aaaaAGATAtaga
HvGSK1.2	P$ATHB6_01	ATHB6	3853	3862	1	1	0.997	tcAATAAta
HvGSK1.2	P$ATHB5_04	ATHB5	3853	3864	1	1	0.924	tcAATAAtaat
HvGSK1.2	P$ATHB1_03	ATHB1	3853	3864	1	1	0.905	tcAATAAtaat
HvGSK1.2	P$ATHB16_01	ATHB16	3854	3862	1	1	0.878	cAATAAta
HvGSK1.2	P$ATHB6_01	ATHB6	3856	3865	1	1	0.981	atAATAAta
HvGSK1.2	P$ATHB5_04	ATHB5	3856	3867	1	1	0.91	atAATAAtaaa
HvGSK1.2	P$ATHB1_03	ATHB1	3856	3867	1	1	0.902	atAATAAtaaa
HvGSK1.2	P$ATHB16_01	ATHB16	3857	3865	1	1	0.915	tAATAAta
HvGSK1.2	P$ATHB6_01	ATHB6	3859	3868	1	1	0.91	atAATAAaa
HvGSK1.2	P$PBF_01	BF	3863	3874	1	1	0.984	taaAAAAGggc
HvGSK1.2	P$DOF_Q2	DOF	3863	3874	1	1	0.979	taaAAAAGggc
HvGSK1.2	P$CDF2_01	CDF2	3864	3874	1	1	0.984	aaAAAAGggc
HvGSK1.2	P$CDF3_01	CDF3	3865	3874	1	1	0.988	aAAAAGggc
HvGSK1.2	P$PBF_Q2	BF	3866	3872	1	1	1	aAAAGG
HvGSK1.2	P$OJ1581_01	OJ1581	3868	3878	1	1	0.983	aaGGGCCcac
HvGSK1.2	P$TCP2_01	TCP2	3868	3878	1	1	0.971	aaGGGCCcac
HvGSK1.2	P$PCF2_01	CF2	3868	3878	1	1	0.997	aagggCCCAC
HvGSK1.2	P$TCP19_01	TCP19	3868	3878	1	1	0.984	aagggCCCAC
HvGSK1.2	P$TCP20L_01	TCP20L	3869	3878	1	1	0.992	agggCCCAC
HvGSK1.2	P$OSI_01	OSI	3870	3878	1	1	1	gggCCCAC
HvGSK1.2	P$TCP20_02	TCP20	3870	3880	1	1	1	gggCCCACaa
HvGSK1.2	P$ARALY495258_02	ARALY495258	3870	3878	1	1	1	gggCCCAC
HvGSK1.2	P$ARALY493022_04	ARALY493022	3870	3878	1	1	1	gggCCCAC
HvGSK1.2	P$ARALY484486_05	ARALY484486	3870	3878	1	1	1	gggCCCAC
HvGSK1.2	P$RAV2_01	RAV2	3906	3915	1	1	0.861	cgACCGAtc
HvGSK1.2	P$LEC2_01	LEC2	3913	3924	1	1	0.994	tcCATGCacct
HvGSK1.2	P$FUS3_Q2	FUS3	3914	3925	1	1	0.856	cCATGCacctg
HvGSK1.2	P$RAV1_02	RAV1	3917	3929	1	1	0.988	tgcACCTGaccg
HvGSK1.2	P$CBF3_02	CBF3	3929	3943	1	1	0.911	gccatCCGACgccc
HvGSK1.2	P$CBF1_04	CBF1	3930	3942	1	1	0.901	ccatCCGACgcc
HvGSK1.2	P$ARF5_01	ARF5	3933	3941	1	1	0.88	tCCGACgc
HvGSK1.2	P$DREB1B_01	DREB1B	3934	3939	1	1	1	CCGAC
HvGSK1.2	P$AT2G41690_01	AT2G41690	3955	3961	1	1	0.988	CCGAAg
HvGSK1.2	P$SEP3_01	wrz-03	3960	3971	1	1	0.881	ggctaTTTTGg
HvGSK1.2	P$HSFA4A_01	HSFA4A	3961	3967	1	1	0.91	gCTATT
HvGSK1.2	P$SED_Q2	SED	3978	3988	1	1	0.999	attcCCTTTt
HvGSK1.2	P$PBF_Q2_01	BF	3982	3988	1	1	1	CCTTTt
HvGSK1.3	P$MYB3R1_01	MYB3R1	10	25	1	1	0.869	gttaacatCCGTTtg
HvGSK1.3	P$MYB3R4_01	MYB3R4	10	25	1	1	0.861	gttaacatCCGTTtg
HvGSK1.3	P$WRKY48_01	WRKY48	22	31	1	1	0.907	ttgtAACAA
HvGSK1.3	P$ATMYB15_Q2	ATMYB15	25	31	1	1	1	TAACAa
HvGSK1.3	P$GATA15_01	GATA15	29	38	1	1	0.999	aaTGATCga
HvGSK1.3	P$ABI3_01	ABI3	46	55	1	1	0.965	gtGCATGct
HvGSK1.3	P$FUS3_01	FUS3	47	56	1	1	0.955	tGCATGcta
HvGSK1.3	P$LEC2_01	LEC2	47	58	1	1	0.937	tgCATGCtatg
HvGSK1.3	P$ZAT1_01	ZAT1	64	75	1	1	0.875	aagctcACAAA
HvGSK1.3	P$PBF_01	BF	69	80	1	1	0.966	cacAAAAGttc
HvGSK1.3	P$DOF_Q2	DOF	69	80	1	1	0.923	cacAAAAGttc
HvGSK1.3	P$CDF2_01	CDF2	70	80	1	1	0.965	acAAAAGttc
HvGSK1.3	P$CDF3_01	CDF3	71	80	1	1	0.984	cAAAAGttc
HvGSK1.3	P$AT1G77950_01	AT1G77950	77	88	1	1	0.88	ttcacTTTAAa
HvGSK1.3	P$SBF1_01	SBF1	77	91	1	1	0.853	ttcactTTAAAttg
HvGSK1.3	P$ATHB6_01	ATHB6	100	109	1	1	0.911	aaAATAAgt
HvGSK1.3	P$O2_Q4	O2	122	133	1	1	0.897	aagaCATGTga

HvGSK1.3	P$GT1_Q6	GT1	129	136	1	1	0.971	GTGAAaa
HvGSK1.3	P$BPC1_Q2	BPC1	144	150	1	1	0.99	AGAAAc
HvGSK1.3	P$AT4G36620_01	AT4G36620	144	152	1	1	0.9	agaAACCA
HvGSK1.3	P$WRKY18_02	WRKY18	151	161	1	1	0.999	atgGTCAAgg
HvGSK1.3	P$WRKY21_02	WRKY21	151	161	1	1	0.963	atgGTCAAgg
HvGSK1.3	P$WRKY48_02	WRKY48	151	161	1	1	0.996	atgGTCAAgg
HvGSK1.3	P$WRKY57_01	WRKY57	151	161	1	1	0.969	atgGTCAAgg
HvGSK1.3	P$WRKY60_01	WRKY60	151	162	1	1	0.974	atgGTCAAggt
HvGSK1.3	P$WRKY15_01	WRKY15	152	162	1	1	0.98	tgGTCAAggt
HvGSK1.3	P$WRKY2_01	WRKY2	152	160	1	1	0.982	tgGTCAAg
HvGSK1.3	P$WRKY25_02	WRKY25	152	160	1	1	0.968	tgGTCAAg
HvGSK1.3	P$WRKY40_01	WRKY40	152	160	1	1	1	tgGTCAAg
HvGSK1.3	P$WRKY43_02	WRKY43	152	162	1	1	0.972	tgGTCAAggt
HvGSK1.3	P$WRKY62_01	WRKY62	152	160	1	1	0.932	tgGTCAAg
HvGSK1.3	P$WRKY63_01	WRKY63	152	160	1	1	0.993	tgGTCAAg
HvGSK1.3	P$WRKY75_01	WRKY75	152	160	1	1	0.959	tgGTCAAg
HvGSK1.3	P$WRKY8_01	WRKY8	152	161	1	1	0.989	tgGTCAAgg
HvGSK1.3	P$WRKY23_01	WRKY23	153	161	1	1	0.875	gGTCAAgg
HvGSK1.3	P$WRKY30_01	WRKY30	153	163	1	1	0.922	gGTCAAggtt
HvGSK1.3	P$WRKY18_Q2	WRKY18	154	163	1	1	0.989	GTCAAggtt
HvGSK1.3	P$GATA9_01	GATA9	164	175	1	1	0.979	gagAGATCtaa
HvGSK1.3	P$AGP1_01	AGP1	165	175	1	1	0.988	agAGATCtaa
HvGSK1.3	P$GATA10_01	GATA10	166	174	1	1	0.92	gAGATCta
HvGSK1.3	P$GATA11_01	GATA11	166	174	1	1	0.944	gaGATCTa
HvGSK1.3	P$GATA8_01	GATA8	166	175	1	1	1	gaGATCTaa
HvGSK1.3	P$ARR10_01	ARR10	167	174	1	1	0.913	AGATCta
HvGSK1.3	P$DOF1_01	DOF1	169	180	1	1	0.986	atcTAAAGgcc
HvGSK1.3	P$PBF_Q2	BF	172	178	1	1	0.986	tAAAGG
HvGSK1.3	P$TCP16_01	TCP16	180	190	1	1	0.881	atGGACCcaa
HvGSK1.3	P$ASR1_01	ASR1	184	189	1	1	1	ACCCA
HvGSK1.3	P$MYBAS1_01	MYBAS1	184	195	1	1	0.955	acCCAACccta
HvGSK1.3	P$GAMYB_01	GAMYB	187	195	1	1	0.883	CAACCcta
HvGSK1.3	P$MYB1L_01	MYB1L	188	198	1	1	1	aaCCCTAgat
HvGSK1.3	P$TRB2_01	TRB2	188	196	1	1	0.971	aaCCCTAg
HvGSK1.3	P$GATA9_01	GATA9	191	202	1	1	0.898	cctAGATCatg
HvGSK1.3	P$AGP1_01	AGP1	192	202	1	1	0.857	ctAGATCatg
HvGSK1.3	P$GATA10_01	GATA10	193	201	1	1	0.868	tAGATCat
HvGSK1.3	P$ARR10_01	ARR10	194	201	1	1	0.913	AGATCat
HvGSK1.3	P$MYB1L_01	MYB1L	206	216	1	1	0.948	tcCCCTAccg
HvGSK1.3	P$TRB2_01	TRB2	206	214	1	1	0.927	tcCCCTAc
HvGSK1.3	P$P_01		208	217	1	1	0.948	ccCTACCgc
HvGSK1.3	P$AT3G63350_01	AT3G63350	213	219	1	1	0.867	CCGCCt
HvGSK1.3	P$MYBAS1_01	MYBAS1	241	252	1	1	0.989	ccCCAACcacc
HvGSK1.3	P$AT4G36620_01	AT4G36620	242	250	1	1	0.885	cccAACCA
HvGSK1.3	P$GAMYB_01	GAMYB	244	252	1	1	0.953	CAACCacc
HvGSK1.3	P$AT4G36620_01	AT4G36620	254	262	1	1	0.879	gcaAACCA
HvGSK1.3	P$HSFA2_01	HSFA2	259	265	1	1	0.922	CCAAAt
HvGSK1.3	P$ALFIN1_Q2	ALFIN1	270	285	1	1	0.893	aagtgtGTGGGttgt
HvGSK1.3	P$HMG1_01	HMG1	280	289	1	1	0.898	GTTGTcata
HvGSK1.3	P$SPF1_Q2	SPF1	284	294	1	1	0.918	tcATAGTggt
HvGSK1.3	P$SPF1_Q2	SPF1	307	317	1	1	0.962	tcATAGTata
HvGSK1.3	P$DOF2_01	DOF2	327	338	1	1	0.981	tcccAAAGCat
HvGSK1.3	P$DOF3_01	DOF3	327	338	1	1	0.979	tcccAAAGCat
HvGSK1.3	P$HSFA2_01	HSFA2	329	335	1	1	0.933	CCAAAg
HvGSK1.3	P$ABI3_01	ABI3	332	341	1	1	0.854	aaGCATGgc
HvGSK1.3	P$ASR1_01	ASR1	380	385	1	1	1	ACCCA
HvGSK1.3	P$WRKY40_03	WRKY40	383	393	1	1	0.998	caAGTCAacg
HvGSK1.3	P$WRKY18_02	WRKY18	383	393	1	1	0.983	caaGTCAAcg
HvGSK1.3	P$WRKY21_02	WRKY21	383	393	1	1	1	caaGTCAAcg
HvGSK1.3	P$WRKY48_02	WRKY48	383	393	1	1	0.997	caaGTCAAcg
HvGSK1.3	P$WRKY57_01	WRKY57	383	393	1	1	0.997	caaGTCAAcg
HvGSK1.3	P$WRKY60_01	WRKY60	383	394	1	1	0.925	caaGTCAAcga
HvGSK1.3	P$WRKY15_01	WRKY15	384	394	1	1	0.995	aaGTCAAcga
HvGSK1.3	P$WRKY2_01	WRKY2	384	392	1	1	0.948	aaGTCAAc
HvGSK1.3	P$WRKY25_02	WRKY25	384	392	1	1	0.933	aaGTCAAc
HvGSK1.3	P$WRKY40_01	WRKY40	384	392	1	1	0.996	aaGTCAAc
HvGSK1.3	P$WRKY43_02	WRKY43	384	394	1	1	1	aaGTCAAcga
HvGSK1.3	P$WRKY62_01	WRKY62	384	392	1	1	0.918	aaGTCAAc
HvGSK1.3	P$WRKY63_01	WRKY63	384	392	1	1	0.914	aaGTCAAc
HvGSK1.3	P$WRKY75_01	WRKY75	384	392	1	1	1	aaGTCAAc
HvGSK1.3	P$WRKY8_01	WRKY8	384	393	1	1	0.999	aaGTCAAcg
HvGSK1.3	P$WRKY23_01	WRKY23	385	393	1	1	1	aGTCAAcg
HvGSK1.3	P$WRKY30_01	WRKY30	385	395	1	1	0.998	aGTCAAcgag
HvGSK1.3	P$WRKY18_Q2	WRKY18	386	395	1	1	0.918	GTCAAcgag
HvGSK1.3	P$AT5G54070_01	AT5G54070	387	393	1	1	0.91	tCAACG
HvGSK1.3	P$MYB1L_01	MYB1L	394	404	1	1	0.973	gaCCCTAtgg
HvGSK1.3	P$TRB2_01	TRB2	394	402	1	1	0.947	gaCCCTAt
HvGSK1.3	P$MYBAS1_01	MYBAS1	411	422	1	1	0.978	tgCCAACtcat

HvGSK1.3	P$ARR18_01	ARR18	431	444	1	1	0.895	ggatAGATAgacc
HvGSK1.3	P$EDF3_02	EDF3	439	448	1	1	0.904	aGACCGtgg
HvGSK1.3	P$BZIP68_01	BZIP68	440	449	1	1	0.922	gacCGTGGt
HvGSK1.3	P$ABI3_01	ABI3	448	457	1	1	0.861	tgGCATGag
HvGSK1.3	P$PBF_Q2	BF	473	479	1	1	0.958	cAAAGG
HvGSK1.3	P$C1_Q2	C1	478	489	1	1	0.939	gtAACTAacta
HvGSK1.3	P$WEREWOLF_Q2_01	WEREWOLF	480	489	1	1	0.928	aACTAActa
HvGSK1.3	P$MYBAS1_01	MYBAS1	480	491	1	1	0.94	aaCTAACtaag
HvGSK1.3	P$C1_Q2	C1	482	493	1	1	0.962	ctAACTAagcc
HvGSK1.3	P$MYB24_01	MYB24	497	506	1	1	0.932	aacTTAGGt
HvGSK1.3	P$MYB131_01	MYB131	497	508	1	1	0.967	aacTTAGGtat
HvGSK1.3	P$MYB3_01	MYB3	498	509	1	1	0.877	actTAGGTatc
HvGSK1.3	P$MYB4_01	MYB4	499	507	1	1	0.855	ctTAGGTa
HvGSK1.3	P$HSFA4A_01	HSFA4A	508	514	1	1	0.964	cCTATT
HvGSK1.3	P$TGA1_02	TGA1	512	527	1	1	0.89	ttggttcACGTCatg
HvGSK1.3	P$HBP1B_Q6	HBP1B	512	526	1	1	0.894	ttggttcaCGTCAt
HvGSK1.3	P$ABF2_01	ABF2	513	526	1	1	0.889	tggttCACGTcat
HvGSK1.3	P$O2_Q4	O2	514	525	1	1	0.928	ggttCACGTca
HvGSK1.3	P$TGA7_02	TGA7	514	531	1	1	0.889	ggttcACGTCatgtgag
HvGSK1.3	P$GBP_Q6	GBP	515	527	1	1	0.903	gttCACGTcatg
HvGSK1.3	P$ABI5_01	ABI5	515	525	1	1	0.894	gttCACGTca
HvGSK1.3	P$ABF4_01	ABF4	515	527	1	1	0.874	gttCACGTcatg
HvGSK1.3	P$CPRF3_Q2	CPRF3	516	526	1	1	0.966	ttCACGTcat
HvGSK1.3	P$CPRF2_Q2	CPRF2	516	526	1	1	0.967	ttCACGTcat
HvGSK1.3	P$O2_02	O2	516	526	1	1	0.967	ttCACGTcat
HvGSK1.3	P$TGA1B_Q2	TGA1B	516	526	1	1	0.936	ttCACGTcat
HvGSK1.3	P$TGA1A_Q2	TGA1A	516	526	1	1	0.985	ttCACGTcat
HvGSK1.3	P$CPRF3_01	CPRF3	516	526	1	1	0.983	ttCACGTcat
HvGSK1.3	P$CPRF2_01	CPRF2	516	526	1	1	0.969	ttCACGTcat
HvGSK1.3	P$TGA1B_01	TGA1B	516	526	1	1	0.906	ttCACGTcat
HvGSK1.3	P$BEE2_01	BEE2	516	526	1	1	0.906	ttCACGTcat
HvGSK1.3	P$BIM3_01	BIM3	516	526	1	1	0.877	ttCACGTcat
HvGSK1.3	P$PHYPA143875_02	HYPA143875	516	526	1	1	0.871	ttCACGTcat
HvGSK1.3	P$SPT_01	SPT	516	525	1	1	0.921	ttCACGTca
HvGSK1.3	P$GBF1F_Q2	GBF1F	516	527	1	1	0.851	ttCACGTcatg
HvGSK1.3	P$HBPA1_Q6_01	HBPA1	516	526	1	1	0.909	ttcaCGTCAt
HvGSK1.3	P$RITA1_01	RITA1	517	524	1	1	0.98	tCACGTc
HvGSK1.3	P$OCSBF1_01	OCSBF1	518	523	1	1	1	CACGT
HvGSK1.3	P$TGA1A_01	TGA1A	518	525	1	1	0.989	cACGTCa
HvGSK1.3	P$TGA2_Q2	TGA2	519	529	1	1	0.881	aCGTCAtgtg
HvGSK1.3	P$O2_Q4	O2	519	530	1	1	0.858	acgtCATGTga
HvGSK1.3	P$AMS_01	AMS	521	531	1	1	0.88	gtCATGTgag
HvGSK1.3	P$PDF2_01	DF2	566	577	1	1	0.861	atggTAAATga
HvGSK1.3	P$PBF_Q2	BF	587	593	1	1	0.965	gAAAGG
HvGSK1.3	P$ROM_Q2	ROM	595	604	1	1	0.864	gcCACCTcg
HvGSK1.3	P$AT4G00870_01	AT4G00870	597	611	1	1	0.899	caCCTCGactacta
HvGSK1.3	P$AT3G60580_01	AT3G60580	613	620	1	1	0.902	ggATCCC
HvGSK1.3	P$AT5G66730_01	AT5G66730	624	635	1	1	0.879	aaagaGGGATc
HvGSK1.3	P$GATA8_01	GATA8	629	638	1	1	0.98	ggGATCTca
HvGSK1.3	P$AT5G26170_01	AT5G26170	633	642	1	1	0.937	tcTCAACca
HvGSK1.3	P$AT4G36620_01	AT4G36620	634	642	1	1	0.976	ctcAACCA
HvGSK1.3	P$GAMYB_01	GAMYB	636	644	1	1	0.891	CAACCata
HvGSK1.3	P$ARR2_01	ARR2	644	654	1	1	0.861	caccATCTTc
HvGSK1.3	P$DOF2_01	DOF2	666	677	1	1	0.979	gtgcAAAGCgg
HvGSK1.3	P$DOF3_01	DOF3	666	677	1	1	0.982	gtgcAAAGCgg
HvGSK1.3	P$AT2G41690_01	AT2G41690	691	697	1	1	0.978	CCGAAa
HvGSK1.3	P$GAMYB_01	GAMYB	705	713	1	1	0.948	CAACCgaa
HvGSK1.3	P$RAV2_01	RAV2	705	714	1	1	0.861	caACCGAag
HvGSK1.3	P$AT2G41690_01	AT2G41690	708	714	1	1	0.988	CCGAAg
HvGSK1.3	P$ATHB7_01	ATHB7	739	749	1	1	0.99	gcAATCAtgt
HvGSK1.3	P$HAT1_01	HAT1	739	749	1	1	0.992	gcAATCAtgt
HvGSK1.3	P$O2_Q4	O2	740	751	1	1	0.854	caatCATGTag
HvGSK1.3	P$AZF3_01	AZF3	748	759	1	1	0.9	tAGTATcttgt
HvGSK1.3	P$ARR2_01	ARR2	748	758	1	1	0.974	tagtATCTTg
HvGSK1.3	P$SED_Q2	SED	762	772	1	1	0.95	cccaCCTTTa
HvGSK1.3	P$PBF_Q2_01	BF	766	772	1	1	0.998	CCTTTa
HvGSK1.3	P$PBF_Q2	BF	775	781	1	1	0.965	gAAAGG
HvGSK1.3	P$ABI3_01	ABI3	779	788	1	1	0.951	ggGCATGcc
HvGSK1.3	P$FUS3_01	FUS3	780	789	1	1	0.94	gGCATGcct
HvGSK1.3	P$LEC2_01	LEC2	780	791	1	1	0.947	ggCATGCctct
HvGSK1.3	P$SEP3_01	wrz-03	789	800	1	1	0.89	ctcaaTTTTGg
HvGSK1.3	P$DOF1_01	DOF1	806	817	1	1	0.981	ataTAAAGtga
HvGSK1.3	P$WRKY11_Q2	WRKY11	825	833	1	1	0.952	aTTGACta
HvGSK1.3	P$ABF2_01	ABF2	846	859	1	1	0.889	gttttCACGTtct
HvGSK1.3	P$O2_Q4	O2	847	858	1	1	0.883	ttttCACGTtc
HvGSK1.3	P$GBP_Q6	GBP	848	860	1	1	0.879	tttCACGTtctt
HvGSK1.3	P$ABI5_01	ABI5	848	858	1	1	0.894	tttCACGTtc
HvGSK1.3	P$ABF4_01	ABF4	848	860	1	1	0.864	tttCACGTtctt

HvGSK1.3	P$CPRF3_Q2	CPRF3	849	859	1	1	0.909	ttCACGTtct
HvGSK1.3	P$CPRF2_Q2	CPRF2	849	859	1	1	0.929	ttCACGTtct
HvGSK1.3	P$O2_02	O2	849	859	1	1	0.936	ttCACGTtct
HvGSK1.3	P$TGA1B_Q2	TGA1B	849	859	1	1	0.868	ttCACGTtct
HvGSK1.3	P$TGA1A_Q2	TGA1A	849	859	1	1	0.96	ttCACGTtct
HvGSK1.3	P$CPRF3_01	CPRF3	849	859	1	1	0.92	ttCACGTtct
HvGSK1.3	P$CPRF2_01	CPRF2	849	859	1	1	0.931	ttCACGTtct
HvGSK1.3	P$BEE2_01	BEE2	849	859	1	1	0.92	ttCACGTtct
HvGSK1.3	P$BIM2_01	BIM2	849	859	1	1	0.857	ttCACGTtct
HvGSK1.3	P$BIM3_01	BIM3	849	859	1	1	0.89	ttCACGTtct
HvGSK1.3	P$PHYPA143875_02	HYPA143875	849	859	1	1	0.887	ttCACGTtct
HvGSK1.3	P$SPT_01	SPT	849	858	1	1	0.917	ttCACGTtc
HvGSK1.3	P$RITA1_01	RITA1	850	857	1	1	0.969	tCACGTt
HvGSK1.3	P$MYC3_01	MYC3	850	858	1	1	0.852	tCACGTtc
HvGSK1.3	P$OCSBF1_01	OCSBF1	851	856	1	1	1	CACGT
HvGSK1.3	P$AT5G65330_01	AT5G65330	869	880	1	1	0.904	CGATAtttatt
HvGSK1.3	P$SED_Q2	SED	877	887	1	1	0.984	atttCCTTTc
HvGSK1.3	P$PBF_Q2_01	BF	881	887	1	1	0.985	CCTTTc
HvGSK1.3	P$ARR2_01	ARR2	887	897	1	1	0.952	ttggATCTTc
HvGSK1.3	P$GATA11_01	GATA11	888	896	1	1	0.867	tgGATCTt
HvGSK1.3	P$GATA8_01	GATA8	888	897	1	1	0.976	tgGATCTTc
HvGSK1.3	P$ARR18_01	ARR18	893	906	1	1	0.94	cttcAGATAagtc
HvGSK1.3	P$DREB1A_04	DREB1A	901	911	1	1	0.982	aaGTCGGtac
HvGSK1.3	P$ERF039_01	ERF039	901	911	1	1	0.99	aaGTCGGtac
HvGSK1.3	P$PHYPA182268_05	HYPA182268	901	911	1	1	0.937	aaGTCGGtac
HvGSK1.3	P$PHYPA64121_06	HYPA64121	901	914	1	1	0.885	aagTCGGTacttt
HvGSK1.3	P$ERF043_01	ERF043	902	910	1	1	0.868	aGTCGGta
HvGSK1.3	P$PHYPA173530_04	HYPA173530	902	910	1	1	0.923	aGTCGGta
HvGSK1.3	P$PHYPA28324_10	HYPA28324	902	910	1	1	0.939	aGTCGGta
HvGSK1.3	P$EDT1_01	EDT1	910	920	1	1	0.957	cttTTAATgc
HvGSK1.3	P$CBF3_02	CBF3	916	930	1	1	0.919	atgccCCGACcaaa
HvGSK1.3	P$CBF1_04	CBF1	917	929	1	1	0.902	tgccCCGACcaa
HvGSK1.3	P$ARF5_01	ARF5	920	928	1	1	0.921	cCCGACca
HvGSK1.3	P$DREB1B_01	DREB1B	921	926	1	1	1	CCGAC
HvGSK1.3	P$HSFA2_01	HSFA2	925	931	1	1	1	CCAAAa
HvGSK1.3	P$AT1G19490_01	AT1G19490	937	946	1	1	0.909	GGTTTactc
HvGSK1.3	P$PBF_01	BF	1031	1042	1	1	0.969	tagAAAAGaag
HvGSK1.3	P$DOF_Q2	DOF	1031	1042	1	1	0.957	tagAAAAGaag
HvGSK1.3	P$BPC1_Q2	BPC1	1032	1038	1	1	0.997	AGAAAa
HvGSK1.3	P$CDF2_01	CDF2	1032	1042	1	1	0.95	agAAAAGaag
HvGSK1.3	P$CDF3_01	CDF3	1033	1042	1	1	0.97	gAAAAGaag
HvGSK1.3	P$ATSPL8_01	ATSPL8	1038	1054	1	1	0.936	gaagaTGTACcttact
HvGSK1.3	P$TEIL_01	TEIL	1042	1050	1	1	1	ATGTAcct
HvGSK1.3	P$RAV1_01	RAV1	1059	1071	1	1	0.985	gagCAACAtatc
HvGSK1.3	P$DREB1A_04	DREB1A	1080	1090	1	1	0.946	aaGTCGGgat
HvGSK1.3	P$ERF039_01	ERF039	1080	1090	1	1	0.944	aaGTCGGgat
HvGSK1.3	P$PHYPA28324_10	HYPA28324	1081	1089	1	1	0.856	aGTCGGga
HvGSK1.3	P$RAV1_02	RAV1	1094	1106	1	1	0.913	catACCTGcccc
HvGSK1.3	P$GL15_01	GL15	1098	1108	1	1	0.904	cctgcCCCCC
HvGSK1.3	P$RAV2_01	RAV2	1114	1123	1	1	0.879	gaACCGAtg
HvGSK1.3	P$WRKY44_01	WRKY44	1131	1140	1	1	0.85	AGTCCaaca
HvGSK1.3	P$MYBAS1_01	MYBAS1	1132	1143	1	1	0.958	gtCCAACagta
HvGSK1.3	P$RAV1_01	RAV1	1132	1144	1	1	0.958	gtcCAACAgtaa
HvGSK1.3	P$DOF1_01	DOF1	1138	1149	1	1	0.98	cagTAAAGttc
HvGSK1.3	P$KNOX3_01	KNOX3	1150	1162	1	1	0.962	ctctTGACAcac
HvGSK1.3	P$WRKY11_Q2	WRKY11	1152	1160	1	1	0.897	cTTGACac
HvGSK1.3	P$ATH1_01	ATH1	1154	1162	1	1	0.902	TGACAcac
HvGSK1.3	P$ABF2_01	ABF2	1154	1167	1	1	0.949	tgacaCACGTctg
HvGSK1.3	P$O2_Q4	O2	1155	1166	1	1	0.908	gacaCACGTct
HvGSK1.3	P$GBP_Q6	GBP	1156	1168	1	1	0.891	acaCACGTctgt
HvGSK1.3	P$ABI5_01	ABI5	1156	1166	1	1	0.928	acaCACGTct
HvGSK1.3	P$ABF4_01	ABF4	1156	1168	1	1	0.922	acaCACGTctgt
HvGSK1.3	P$EMBP1_Q2	EMBP1	1157	1167	1	1	0.857	caCACGTctg
HvGSK1.3	P$CPRF3_Q2	CPRF3	1157	1167	1	1	0.954	caCACGTctg
HvGSK1.3	P$CPRF2_Q2	CPRF2	1157	1167	1	1	0.962	caCACGTctg
HvGSK1.3	P$O2_02	O2	1157	1167	1	1	0.942	caCACGTctg
HvGSK1.3	P$TGA1B_Q2	TGA1B	1157	1167	1	1	0.924	caCACGTctg
HvGSK1.3	P$TGA1A_Q2	TGA1A	1157	1167	1	1	0.988	caCACGTctg
HvGSK1.3	P$CPRF3_01	CPRF3	1157	1167	1	1	0.97	caCACGTctg
HvGSK1.3	P$CPRF2_01	CPRF2	1157	1167	1	1	0.963	caCACGTctg
HvGSK1.3	P$TGA1B_01	TGA1B	1157	1167	1	1	0.871	caCACGTctg
HvGSK1.3	P$BEE2_01	BEE2	1157	1167	1	1	0.906	caCACGTctg
HvGSK1.3	P$BIM3_01	BIM3	1157	1167	1	1	0.874	caCACGTctg
HvGSK1.3	P$PHYPA143875_02	HYPA143875	1157	1167	1	1	0.871	caCACGTctg
HvGSK1.3	P$SPT_01	SPT	1157	1166	1	1	0.92	caCACGTct
HvGSK1.3	P$ABF3_01	ABF3	1158	1166	1	1	0.875	ACACGtct
HvGSK1.3	P$RITA1_01	RITA1	1158	1165	1	1	0.963	aCACGTc
HvGSK1.3	P$OCSBF1_01	OCSBF1	1159	1164	1	1	1	CACGT

HvGSK1.3	P$TGA1A_01	TGA1A	1159	1166	1	1	0.871	cACGTCt
HvGSK1.3	P$MYBAS1_01	MYBAS1	1177	1188	1	1	0.985	tgCCAACatcc
HvGSK1.3	P$RAV1_01	RAV1	1177	1189	1	1	0.923	tgcCAACAtcca
HvGSK1.3	P$AMS_01	AMS	1187	1197	1	1	0.97	caCATGTgcc
HvGSK1.3	P$SPL14_01	SPL14	1197	1204	1	1	0.851	CCGTAta
HvGSK1.3	P$ATHB4_02	ATHB4	1205	1215	1	1	0.875	gcATCATtgt
HvGSK1.3	P$ARF8_01	ARF8	1228	1237	1	1	0.953	cgTGTCGcg
HvGSK1.3	P$ERF1_Q2	ERF1	1240	1248	1	1	1	GGCGGctc
HvGSK1.3	P$KNOX3_01	KNOX3	1264	1276	1	1	0.976	gcccTGACAaac
HvGSK1.3	P$ATH1_01	ATH1	1268	1276	1	1	0.909	TGACAaac
HvGSK1.3	P$MYB305_Q3	MYB305	1269	1282	1	1	0.896	gacaaACCTAccg
HvGSK1.3	P$MYB61_01	MYB61	1271	1286	1	1	0.923	caaACCTAccgattc
HvGSK1.3	P$P_01		1274	1283	1	1	0.994	acCTACCga
HvGSK1.3	P$MYB305_Q3	MYB305	1281	1294	1	1	0.87	gattcACCTAtct
HvGSK1.3	P$MYB61_01	MYB61	1283	1298	1	1	0.884	ttcACCTAtctgttc
HvGSK1.3	P$MYB3R5_01	MYB3R5	1293	1308	1	1	0.918	tgttcctagCCGTTg
HvGSK1.3	P$MYB3R1_01	MYB3R1	1294	1309	1	1	0.925	gttcctagCCGTTgc
HvGSK1.3	P$MYB3R4_01	MYB3R4	1294	1309	1	1	0.948	gttcctagCCGTTgc
HvGSK1.3	P$KNOX3_01	KNOX3	1308	1320	1	1	0.986	cgtgTGACAtct
HvGSK1.3	P$ATH1_01	ATH1	1312	1320	1	1	0.939	TGACAtct
HvGSK1.3	P$MYB1L_01	MYB1L	1325	1335	1	1	0.974	gaCCCTAcat
HvGSK1.3	P$TRB2_01	TRB2	1325	1333	1	1	0.947	gaCCCTAc
HvGSK1.3	P$ATHSFA1D_01	ATHSFA1D	1328	1334	1	1	0.985	cCTACA
HvGSK1.3	P$GAMYB_Q2	GAMYB	1331	1344	1	1	0.915	acataACAACact
HvGSK1.3	P$ATMYB15_Q2	ATMYB15	1334	1340	1	1	1	TAACAa
HvGSK1.3	P$RAV1_01	RAV1	1334	1346	1	1	0.939	taaCAACActaa
HvGSK1.3	P$GT1_Q6_01	GT1	1359	1371	1	1	0.855	TTTGTcctcatt
HvGSK1.3	P$ID1_01	ID1	1359	1370	1	1	0.94	tTTGTCctcat
HvGSK1.3	P$GT1_Q6_01	GT1	1369	1381	1	1	0.857	TTTGTgtgtacc
HvGSK1.3	P$ATSPL8_01	ATSPL8	1370	1386	1	1	0.94	ttgtgTGTACcacaga
HvGSK1.3	P$GATA9_01	GATA9	1380	1391	1	1	0.889	cacAGATCaac
HvGSK1.3	P$AGP1_01	AGP1	1381	1391	1	1	0.913	acAGATCaac
HvGSK1.3	P$ARR10_01	ARR10	1383	1390	1	1	0.869	AGATCaa
HvGSK1.3	P$WRKY40_03	WRKY40	1394	1404	1	1	0.995	ctAGTCAatt
HvGSK1.3	P$WRKY18_02	WRKY18	1394	1404	1	1	0.978	ctaGTCAAtt
HvGSK1.3	P$WRKY21_02	WRKY21	1394	1404	1	1	0.963	ctaGTCAAtt
HvGSK1.3	P$WRKY48_02	WRKY48	1394	1404	1	1	0.993	ctaGTCAAtt
HvGSK1.3	P$WRKY57_01	WRKY57	1394	1404	1	1	0.977	ctaGTCAAtt
HvGSK1.3	P$WRKY60_01	WRKY60	1394	1405	1	1	0.9	ctaGTCAAttc
HvGSK1.3	P$WRKY15_01	WRKY15	1395	1405	1	1	0.974	taGTCAAttc
HvGSK1.3	P$WRKY2_01	WRKY2	1395	1403	1	1	0.935	taGTCAAt
HvGSK1.3	P$WRKY25_02	WRKY25	1395	1403	1	1	0.917	taGTCAAt
HvGSK1.3	P$WRKY40_01	WRKY40	1395	1403	1	1	0.996	taGTCAAt
HvGSK1.3	P$WRKY43_02	WRKY43	1395	1405	1	1	0.986	taGTCAAttc
HvGSK1.3	P$WRKY62_01	WRKY62	1395	1403	1	1	0.897	taGTCAAt
HvGSK1.3	P$WRKY63_01	WRKY63	1395	1403	1	1	0.905	taGTCAAt
HvGSK1.3	P$WRKY75_01	WRKY75	1395	1403	1	1	0.968	taGTCAAt
HvGSK1.3	P$WRKY8_01	WRKY8	1395	1404	1	1	0.988	taGTCAAtt
HvGSK1.3	P$WRKY23_01	WRKY23	1396	1404	1	1	0.862	aGTCAAtt
HvGSK1.3	P$WRKY30_01	WRKY30	1396	1406	1	1	0.92	aGTCAAttcc
HvGSK1.3	P$WRKY18_Q2	WRKY18	1397	1406	1	1	0.944	GTCAAttcc
HvGSK1.3	P$ATHSFA1D_01	ATHSFA1D	1418	1424	1	1	0.94	gCTACA
HvGSK1.3	P$HSFA2_01	HSFA2	1426	1432	1	1	0.941	CCAAAc
HvGSK1.3	P$GT1_Q6_02	GT1	1430	1442	1	1	0.874	acaaacTTAACt
HvGSK1.3	P$AT1G66560_01	AT1G66560	1433	1443	1	1	0.957	aacTTAACtt
HvGSK1.3	P$WRKY21_01	WRKY21	1434	1443	1	1	0.927	acTTAACtt
HvGSK1.3	P$AT2G34830_01	AT2G34830	1434	1443	1	1	0.931	acTTAACtt
HvGSK1.3	P$AT1G18860_01	AT1G18860	1434	1443	1	1	0.987	acTTAACtt
HvGSK1.3	P$AT1G64000_01	AT1G64000	1434	1443	1	1	0.911	acTTAACtt
HvGSK1.3	P$AT4G22070_01	AT4G22070	1434	1443	1	1	0.947	acTTAACtt
HvGSK1.3	P$WRKY6_01	WRKY6	1434	1443	1	1	0.946	acTTAACtt
HvGSK1.3	P$AT1G66600_01	AT1G66600	1434	1443	1	1	0.959	acTTAACtt
HvGSK1.3	P$AT1G68150_01	AT1G68150	1434	1443	1	1	0.962	acTTAACtt
HvGSK1.3	P$AT5G41570_01	AT5G41570	1434	1443	1	1	0.91	acTTAACtt
HvGSK1.3	P$AT1G69810_01	AT1G69810	1434	1443	1	1	0.963	acTTAACtt
HvGSK1.3	P$AT5G15130_01	AT5G15130	1434	1443	1	1	0.987	acTTAACtt
HvGSK1.3	P$WRKY46_01	WRKY46	1434	1443	1	1	0.917	acTTAACtt
HvGSK1.3	P$AT1G30650_01	AT1G30650	1434	1443	1	1	0.934	acTTAACtt
HvGSK1.3	P$AT2G24570_01	AT2G24570	1434	1443	1	1	0.927	acTTAACtt
HvGSK1.3	P$AT4G23550_01	AT4G23550	1434	1443	1	1	0.928	acTTAACtt
HvGSK1.3	P$WRKY7_01	WRKY7	1434	1443	1	1	0.923	acTTAACtt
HvGSK1.3	P$SED_Q2	SED	1446	1456	1	1	0.897	tgttCCTTTc
HvGSK1.3	P$PBF_Q2_01	BF	1450	1456	1	1	0.985	CCTTTc
HvGSK1.3	P$AT2G41690_01	AT2G41690	1466	1472	1	1	0.978	CCGAAa
HvGSK1.3	P$ATHB6_01	ATHB6	1470	1479	1	1	0.908	aaAATAAga
HvGSK1.3	P$ABF2_01	ABF2	1476	1489	1	1	0.896	agacgCACGTtgt
HvGSK1.3	P$HBI1_01	HBI1	1477	1489	1	1	0.864	gacgCACGTtgt
HvGSK1.3	P$GBP_Q6	GBP	1478	1490	1	1	0.883	acgCACGTtgtt

HvGSK1.3	P$ABI5_01	ABI5	1478	1488	1	1	0.885	acgCACGTtg
HvGSK1.3	P$ABF4_01	ABF4	1478	1490	1	1	0.885	acgCACGTtgtt
HvGSK1.3	P$CPRF3_Q2	CPRF3	1479	1489	1	1	0.907	cgCACGTtgt
HvGSK1.3	P$CPRF2_Q2	CPRF2	1479	1489	1	1	0.93	cgCACGTtgt
HvGSK1.3	P$O2_02	O2	1479	1489	1	1	0.934	cgCACGTtgt
HvGSK1.3	P$TGA1B_Q2	TGA1B	1479	1489	1	1	0.883	cgCACGTtgt
HvGSK1.3	P$TGA1A_Q2	TGA1A	1479	1489	1	1	0.964	cgCACGTtgt
HvGSK1.3	P$CPRF3_01	CPRF3	1479	1489	1	1	0.913	cgCACGTtgt
HvGSK1.3	P$CPRF2_01	CPRF2	1479	1489	1	1	0.931	cgCACGTtgt
HvGSK1.3	P$BEE2_01	BEE2	1479	1489	1	1	0.92	cgCACGTtgt
HvGSK1.3	P$BIM2_01	BIM2	1479	1489	1	1	0.855	cgCACGTtgt
HvGSK1.3	P$BIM3_01	BIM3	1479	1489	1	1	0.888	cgCACGTtgt
HvGSK1.3	P$PHYPA143875_02	HYPA143875	1479	1489	1	1	0.888	cgCACGTtgt
HvGSK1.3	P$SPT_01	SPT	1479	1488	1	1	0.928	cgCACGTtg
HvGSK1.3	P$RITA1_01	RITA1	1480	1487	1	1	0.943	gCACGTt
HvGSK1.3	P$MYC3_01	MYC3	1480	1488	1	1	0.857	gCACGTtg
HvGSK1.3	P$OCSBF1_01	OCSBF1	1481	1486	1	1	1	CACGT
HvGSK1.3	P$PHV_02	HV	1501	1516	1	1	0.876	tttATCATttctatt
HvGSK1.3	P$ATHB4_02	ATHB4	1502	1512	1	1	0.889	ttATCATttc
HvGSK1.3	P$HSFA4A_01	HSFA4A	1510	1516	1	1	0.914	tCTATT
HvGSK1.3	P$WRKY_Q2	WRKY	1511	1522	1	1	0.901	ctattaAGTCA
HvGSK1.3	P$WRKY40_03	WRKY40	1515	1525	1	1	0.998	taAGTCAagg
HvGSK1.3	P$WRKY18_02	WRKY18	1515	1525	1	1	0.982	taaGTCAAgg
HvGSK1.3	P$WRKY21_02	WRKY21	1515	1525	1	1	0.963	taaGTCAAgg
HvGSK1.3	P$WRKY48_02	WRKY48	1515	1525	1	1	0.993	taaGTCAAgg
HvGSK1.3	P$WRKY57_01	WRKY57	1515	1525	1	1	0.979	taaGTCAAgg
HvGSK1.3	P$WRKY60_01	WRKY60	1515	1526	1	1	0.899	taaGTCAAggt
HvGSK1.3	P$WRKY15_01	WRKY15	1516	1526	1	1	0.975	aaGTCAAggt
HvGSK1.3	P$WRKY2_01	WRKY2	1516	1524	1	1	0.931	aaGTCAAg
HvGSK1.3	P$WRKY25_02	WRKY25	1516	1524	1	1	0.907	aaGTCAAg
HvGSK1.3	P$WRKY40_01	WRKY40	1516	1524	1	1	0.996	aaGTCAAg
HvGSK1.3	P$WRKY43_02	WRKY43	1516	1526	1	1	0.987	aaGTCAAggt
HvGSK1.3	P$WRKY63_01	WRKY63	1516	1524	1	1	0.907	aaGTCAAg
HvGSK1.3	P$WRKY75_01	WRKY75	1516	1524	1	1	0.96	aaGTCAAg
HvGSK1.3	P$WRKY8_01	WRKY8	1516	1525	1	1	0.988	aaGTCAAgg
HvGSK1.3	P$WRKY23_01	WRKY23	1517	1525	1	1	0.91	aGTCAAgg
HvGSK1.3	P$WRKY30_01	WRKY30	1517	1527	1	1	0.92	aGTCAAggtg
HvGSK1.3	P$WRKY18_Q2	WRKY18	1518	1527	1	1	0.984	GTCAAggtg
HvGSK1.3	P$CBF3_02	CBF3	1548	1562	1	1	0.97	tcaaaCCGACctgg
HvGSK1.3	P$AT4G12750_01	AT4G12750	1549	1559	1	1	0.909	caaACCGAcc
HvGSK1.3	P$CBF1_04	CBF1	1549	1561	1	1	0.944	caaaCCGACctg
HvGSK1.3	P$ARF1_01	ARF1	1552	1560	1	1	0.887	aCCGACct
HvGSK1.3	P$ARF5_01	ARF5	1552	1560	1	1	0.918	aCCGACct
HvGSK1.3	P$DREB1B_01	DREB1B	1553	1558	1	1	1	CCGAC
HvGSK1.3	P$RAV1_02	RAV1	1553	1565	1	1	0.905	ccgACCTGgaaa
HvGSK1.3	P$CBF3_02	CBF3	1561	1575	1	1	0.927	gaaacCCGACaaac
HvGSK1.3	P$CBF1_04	CBF1	1562	1574	1	1	0.922	aaacCCGACaaa
HvGSK1.3	P$DREB1G_02	DREB1G	1563	1573	1	1	0.892	aacCCGACaa
HvGSK1.3	P$AT1G77200_03	AT1G77200	1563	1577	1	1	0.875	aacCCGACaaactc
HvGSK1.3	P$ARF1_01	ARF1	1565	1573	1	1	0.931	cCCGACaa
HvGSK1.3	P$ARF5_01	ARF5	1565	1573	1	1	0.99	cCCGACaa
HvGSK1.3	P$DREB1B_01	DREB1B	1566	1571	1	1	1	CCGAC
HvGSK1.3	P$MYB61_01	MYB61	1636	1651	1	1	0.877	atcACCTActttgct
HvGSK1.3	P$CBNAC_01	CBNAC	1646	1652	1	1	1	tTGCTT
HvGSK1.3	P$CBNAC_02	CBNAC	1646	1662	1	1	0.902	tTGCTTaacatgagat
HvGSK1.3	P$AT1G66560_01	AT1G66560	1647	1657	1	1	0.925	tgcTTAACat
HvGSK1.3	P$WRKY21_01	WRKY21	1648	1657	1	1	0.898	gcTTAACat
HvGSK1.3	P$AT1G18860_01	AT1G18860	1648	1657	1	1	0.931	gcTTAACat
HvGSK1.3	P$AT1G64000_01	AT1G64000	1648	1657	1	1	0.89	gcTTAACat
HvGSK1.3	P$WRKY6_01	WRKY6	1648	1657	1	1	0.868	gcTTAACat
HvGSK1.3	P$AT1G66600_01	AT1G66600	1648	1657	1	1	0.929	gcTTAACat
HvGSK1.3	P$AT1G68150_01	AT1G68150	1648	1657	1	1	0.901	gcTTAACat
HvGSK1.3	P$AT5G41570_01	AT5G41570	1648	1657	1	1	0.89	gcTTAACat
HvGSK1.3	P$AT5G15130_01	AT5G15130	1648	1657	1	1	0.926	gcTTAACat
HvGSK1.3	P$WRKY46_01	WRKY46	1648	1657	1	1	0.909	gcTTAACat
HvGSK1.3	P$AT2G24570_01	AT2G24570	1648	1657	1	1	0.894	gcTTAACat
HvGSK1.3	P$WRKY7_01	WRKY7	1648	1657	1	1	0.896	gcTTAACat
HvGSK1.3	P$AT3G60580_01	AT3G60580	1669	1676	1	1	0.901	gcATCCC
HvGSK1.3	P$AT5G04240_01	AT5G04240	1696	1702	1	1	1	gGGCAC
HvGSK1.3	P$GATA15_01	GATA15	1729	1738	1	1	0.999	taTGATCca
HvGSK1.3	P$O2_Q4	O2	1732	1743	1	1	0.948	gatcCATGTct
HvGSK1.3	P$WRKY11_Q2	WRKY11	1742	1750	1	1	0.974	tTTGACcg
HvGSK1.3	P$ZAP1_01	ZAP1	1743	1753	1	1	0.92	TTGACcgtat
HvGSK1.3	P$SPL14_01	SPL14	1747	1754	1	1	0.851	CCGTAta
HvGSK1.3	P$PBF_01	BF	1751	1762	1	1	0.974	ataAAAAGtac
HvGSK1.3	P$DOF_Q2	DOF	1751	1762	1	1	0.997	ataAAAAGtac
HvGSK1.3	P$CDF2_01	CDF2	1752	1762	1	1	0.997	taAAAAGtac
HvGSK1.3	P$CDF3_01	CDF3	1753	1762	1	1	0.991	aAAAAGtac

HvGSK1.3	P$ATHB7_01	ATHB7	1786	1796	1	1	0.854	aaAATCActa
HvGSK1.3	P$HAT1_01	HAT1	1786	1796	1	1	0.863	aaAATCActa
HvGSK1.3	P$ARF8_01	ARF8	1794	1803	1	1	0.953	taTGTCGtc
HvGSK1.3	P$DRE1C_01	DRE1C	1795	1803	1	1	0.862	ATGTCgtc
HvGSK1.3	P$GAMYB_Q2	GAMYB	1803	1816	1	1	0.928	ctcttACAACcga
HvGSK1.3	P$CBF3_02	CBF3	1807	1821	1	1	0.973	tacaaCCGACacca
HvGSK1.3	P$CBF1_04	CBF1	1808	1820	1	1	0.963	acaaCCGACacc
HvGSK1.3	P$GAMYB_01	GAMYB	1809	1817	1	1	1	CAACCgac
HvGSK1.3	P$DREB1G_02	DREB1G	1809	1819	1	1	0.913	caaCCGACac
HvGSK1.3	P$AT1G77200_03	AT1G77200	1809	1823	1	1	0.95	caaCCGACaccatg
HvGSK1.3	P$ARF1_01	ARF1	1811	1819	1	1	1	aCCGACac
HvGSK1.3	P$ARF5_01	ARF5	1811	1819	1	1	0.985	aCCGACac
HvGSK1.3	P$DREB1B_01	DREB1B	1812	1817	1	1	1	CCGAC
HvGSK1.3	P$LEC2_01	LEC2	1817	1828	1	1	0.966	acCATGCggct
HvGSK1.3	P$GAMYB_Q2	GAMYB	1829	1842	1	1	0.92	accacACAACaca
HvGSK1.3	P$RAV1_01	RAV1	1832	1844	1	1	0.953	acaCAACAcata
HvGSK1.3	P$AT3G60580_01	AT3G60580	1851	1858	1	1	0.905	caATCCC
HvGSK1.3	P$GAMYB_Q2	GAMYB	1858	1871	1	1	0.928	cctctACAACaaa
HvGSK1.3	P$ATHSFA1D_01	ATHSFA1D	1860	1866	1	1	0.941	tCTACA
HvGSK1.3	P$RAV1_01	RAV1	1861	1873	1	1	0.955	ctaCAACAaata
HvGSK1.3	P$AT3G18650_01	AT3G18650	1925	1936	1	1	0.88	gatggTTGTAt
HvGSK1.3	P$HMG1_01	HMG1	1929	1938	1	1	1	GTTGTattc
HvGSK1.3	P$AT3G62240_01	AT3G62240	1938	1948	1	1	0.865	caagcCATTC
HvGSK1.3	P$GT1_Q6	GT1	1965	1972	1	1	0.912	GTGAAca
HvGSK1.3	P$WRKY18_02	WRKY18	1979	1989	1	1	0.946	tttGTCAAga
HvGSK1.3	P$WRKY21_02	WRKY21	1979	1989	1	1	0.945	tttGTCAAga
HvGSK1.3	P$WRKY48_02	WRKY48	1979	1989	1	1	0.985	tttGTCAAga
HvGSK1.3	P$WRKY57_01	WRKY57	1979	1989	1	1	0.955	tttGTCAAga
HvGSK1.3	P$WRKY60_01	WRKY60	1979	1990	1	1	0.889	tttGTCAAgac
HvGSK1.3	P$WRKY15_01	WRKY15	1980	1990	1	1	0.956	ttGTCAAgac
HvGSK1.3	P$WRKY2_01	WRKY2	1980	1988	1	1	0.902	ttGTCAAg
HvGSK1.3	P$WRKY25_02	WRKY25	1980	1988	1	1	0.887	ttGTCAAg
HvGSK1.3	P$WRKY40_01	WRKY40	1980	1988	1	1	0.977	ttGTCAAg
HvGSK1.3	P$WRKY43_02	WRKY43	1980	1990	1	1	0.95	ttGTCAAgac
HvGSK1.3	P$WRKY62_01	WRKY62	1980	1988	1	1	0.87	ttGTCAAg
HvGSK1.3	P$WRKY63_01	WRKY63	1980	1988	1	1	0.888	ttGTCAAg
HvGSK1.3	P$WRKY75_01	WRKY75	1980	1988	1	1	0.919	ttGTCAAg
HvGSK1.3	P$WRKY8_01	WRKY8	1980	1989	1	1	0.976	ttGTCAAga
HvGSK1.3	P$WRKY30_01	WRKY30	1981	1991	1	1	0.897	tGTCAAgaca
HvGSK1.3	P$WRKY18_Q2	WRKY18	1982	1991	1	1	0.957	GTCAAgaca
HvGSK1.3	P$BPC1_Q2	BPC1	2000	2006	1	1	0.997	AGAAAa
HvGSK1.3	P$MYBAS1_01	MYBAS1	2007	2018	1	1	0.952	tcCTAACccga
HvGSK1.3	P$WRKY25_01	WRKY25	2007	2016	1	1	0.923	tccTAACCc
HvGSK1.3	P$WRKY33_01	WRKY33	2007	2016	1	1	0.945	tccTAACCc
HvGSK1.3	P$AT1G29860_01	AT1G29860	2007	2016	1	1	0.892	tccTAACCc
HvGSK1.3	P$AT3G62340_01	AT3G62340	2007	2016	1	1	0.922	tccTAACCc
HvGSK1.3	P$AT1G69310_01	AT1G69310	2007	2016	1	1	0.913	tccTAACCc
HvGSK1.3	P$WRKY26_01	WRKY26	2007	2016	1	1	0.945	tccTAACCc
HvGSK1.3	P$AT2G41690_01	AT2G41690	2014	2020	1	1	0.974	CCGAAt
HvGSK1.3	P$AMS_01	AMS	2019	2029	1	1	0.876	tgCAGGTgca
HvGSK1.3	P$MYB1L_01	MYB1L	2028	2038	1	1	0.956	acCCCTAgcc
HvGSK1.3	P$TRB2_01	TRB2	2028	2036	1	1	0.939	acCCCTAg
HvGSK1.3	P$MYB1L_01	MYB1L	2085	2095	1	1	0.936	ctCCCTAttg
HvGSK1.3	P$TRB2_01	TRB2	2085	2093	1	1	0.92	ctCCCTAt
HvGSK1.3	P$HSFA4A_01	HSFA4A	2088	2094	1	1	0.964	cCTATT
HvGSK1.3	P$SPL14_01	SPL14	2112	2119	1	1	0.851	CCGTAta
HvGSK1.3	P$AT4G04450_01	AT4G04450	2117	2126	1	1	0.955	tatTTAGCc
HvGSK1.3	P$ARR18_01	ARR18	2125	2138	1	1	0.94	cattAGATAagaa
HvGSK1.3	P$PEND_01	END	2132	2140	1	1	0.892	tAAGAAac
HvGSK1.3	P$BPC1_Q2	BPC1	2134	2140	1	1	0.99	AGAAAc
HvGSK1.3	P$ARR18_01	ARR18	2137	2150	1	1	0.889	aacaAGATAatga
HvGSK1.3	P$TGA2_Q2	TGA2	2166	2176	1	1	0.899	cCGTCAtata
HvGSK1.3	P$SPF1_Q2	SPF1	2171	2181	1	1	0.883	atATAGTtta
HvGSK1.3	P$GL15_01	GL15	2191	2201	1	1	0.858	tatatCCCCC
HvGSK1.3	P$AT3G60580_01	AT3G60580	2192	2199	1	1	0.951	atATCCC
HvGSK1.3	P$RAV1_01	RAV1	2211	2223	1	1	0.916	gctCAACAgcgt
HvGSK1.3	P$MYB1L_01	MYB1L	2230	2240	1	1	0.999	aaCCCTAata
HvGSK1.3	P$TRB2_01	TRB2	2230	2238	1	1	0.973	aaCCCTAa
HvGSK1.3	P$ATHB6_01	ATHB6	2234	2243	1	1	0.978	ctAATAAtc
HvGSK1.3	P$ATHB5_04	ATHB5	2234	2245	1	1	0.898	ctAATAAtcct
HvGSK1.3	P$ATHB1_03	ATHB1	2234	2245	1	1	0.887	ctAATAAtcct
HvGSK1.3	P$ATHB16_01	ATHB16	2235	2243	1	1	0.908	tAATAAtc
HvGSK1.3	P$ALFIN1_Q2	ALFIN1	2246	2261	1	1	0.882	atgtgtGTGGGaact
HvGSK1.3	P$C1_Q2	C1	2255	2266	1	1	0.94	ggAACTAttag
HvGSK1.3	P$HSFA4A_01	HSFA4A	2258	2264	1	1	1	aCTATT
HvGSK1.3	P$MYB24_01	MYB24	2259	2268	1	1	0.884	ctaTTAGGa
HvGSK1.3	P$HMG1_01	HMG1	2292	2301	1	1	0.933	GTTGTattg
HvGSK1.3	P$TEIL_01	TEIL	2315	2323	1	1	0.865	ATGTAtcg

HvGSK1.3	P$CBNAC_01	CBNAC	2334	2340	1	1	0.968	aTGCTT
HvGSK1.3	P$EDT1_01	EDT1	2347	2357	1	1	0.976	tatTTAATga
HvGSK1.3	P$GT1_Q6	GT1	2366	2373	1	1	0.971	GTGAAaa
HvGSK1.3	P$BPC1_Q2	BPC1	2381	2387	1	1	0.99	AGAAAt
HvGSK1.3	P$WEREWOLF_Q2	WEREWOLF	2399	2408	1	1	0.895	tgGTTAGtg
HvGSK1.3	P$ALFIN1_Q2	ALFIN1	2399	2414	1	1	0.855	tggttaGTGGGaatt
HvGSK1.3	P$WRKY11_Q2	WRKY11	2411	2419	1	1	0.927	aTTGACtc
HvGSK1.3	P$HSF3_01	HSF3	2417	2423	1	1	0.94	tCGGGG
HvGSK1.3	P$MYB24_01	MYB24	2421	2430	1	1	0.998	gggTTAGGt
HvGSK1.3	P$MYB131_01	MYB131	2421	2432	1	1	0.963	gggTTAGGtaa
HvGSK1.3	P$MYB3_01	MYB3	2422	2433	1	1	0.947	ggtTAGGTaag
HvGSK1.3	P$MYB4_01	MYB4	2423	2431	1	1	0.914	gtTAGGTa
HvGSK1.3	P$ARR18_01	ARR18	2429	2442	1	1	0.888	taagAGATAataa
HvGSK1.3	P$ATHB6_01	ATHB6	2435	2444	1	1	0.905	atAATAAca
HvGSK1.3	P$ATMYB15_Q2	ATMYB15	2439	2445	1	1	1	TAACAa
HvGSK1.3	P$ATHB7_01	ATHB7	2443	2453	1	1	0.913	aaAATCAtga
HvGSK1.3	P$HAT1_01	HAT1	2443	2453	1	1	0.975	aaAATCAtga
HvGSK1.3	P$PBF_01	BF	2451	2462	1	1	0.954	gatAAAAGgtt
HvGSK1.3	P$DOF_Q2	DOF	2451	2462	1	1	0.93	gatAAAAGgtt
HvGSK1.3	P$CDF2_01	CDF2	2452	2462	1	1	0.952	atAAAAGgtt
HvGSK1.3	P$CDF3_01	CDF3	2453	2462	1	1	0.971	tAAAAGgtt
HvGSK1.3	P$PBF_Q2	BF	2454	2460	1	1	1	aAAAGG
HvGSK1.3	P$MYB24_01	MYB24	2457	2466	1	1	0.921	aggTTAGGa
HvGSK1.3	P$ATHB1_01	ATHB1	2461	2475	1	1	0.931	taggaATTATtcac
HvGSK1.3	P$ATHB5_01	ATHB5	2464	2473	1	1	0.919	gaaTTATTc
HvGSK1.3	P$ATHB6_01	ATHB6	2476	2485	1	1	0.897	aaAATAAcc
HvGSK1.3	P$PH4_01	H4	2479	2488	1	1	0.889	ataACCCCc
HvGSK1.3	P$O2_Q4	O2	2509	2520	1	1	0.898	ctccCATGTta
HvGSK1.3	P$GAMYB_Q2	GAMYB	2521	2534	1	1	0.863	taaacACAACatg
HvGSK1.3	P$RAV1_01	RAV1	2524	2536	1	1	0.929	acaCAACAtgag
HvGSK1.3	P$SBF1_01	SBF1	2530	2544	1	1	0.87	catgagTTAAAttc
HvGSK1.3	P$PDF2_01	DF2	2533	2544	1	1	0.872	gagtTAAATtc
HvGSK1.3	P$O2_Q4	O2	2547	2558	1	1	0.951	ttgcCATGTct
HvGSK1.3	P$AT3G60580_01	AT3G60580	2559	2566	1	1	0.951	atATCCC
HvGSK1.3	P$EDT1_01	EDT1	2564	2574	1	1	0.863	ccaTTAATct
HvGSK1.3	P$O2_Q4	O2	2570	2581	1	1	0.96	atctCATGTca
HvGSK1.3	P$WRKY18_02	WRKY18	2574	2584	1	1	0.944	catGTCAAtt
HvGSK1.3	P$WRKY21_02	WRKY21	2574	2584	1	1	0.95	catGTCAAtt
HvGSK1.3	P$WRKY48_02	WRKY48	2574	2584	1	1	0.986	catGTCAAtt
HvGSK1.3	P$WRKY57_01	WRKY57	2574	2584	1	1	0.959	catGTCAAtt
HvGSK1.3	P$WRKY60_01	WRKY60	2574	2585	1	1	0.883	catGTCAAtta
HvGSK1.3	P$WRKY15_01	WRKY15	2575	2585	1	1	0.958	atGTCAAtta
HvGSK1.3	P$WRKY2_01	WRKY2	2575	2583	1	1	0.902	atGTCAAt
HvGSK1.3	P$WRKY25_02	WRKY25	2575	2583	1	1	0.879	atGTCAAt
HvGSK1.3	P$WRKY40_01	WRKY40	2575	2583	1	1	0.977	atGTCAAt
HvGSK1.3	P$WRKY43_02	WRKY43	2575	2585	1	1	0.952	atGTCAAtta
HvGSK1.3	P$WRKY63_01	WRKY63	2575	2583	1	1	0.885	atGTCAAt
HvGSK1.3	P$WRKY75_01	WRKY75	2575	2583	1	1	0.927	atGTCAAt
HvGSK1.3	P$WRKY8_01	WRKY8	2575	2584	1	1	0.977	atGTCAAtt
HvGSK1.3	P$WRKY30_01	WRKY30	2576	2586	1	1	0.904	tGTCAAttaa
HvGSK1.3	P$WRKY18_Q2	WRKY18	2577	2586	1	1	0.94	GTCAAttaa
HvGSK1.3	P$ATHB6_01	ATHB6	2583	2592	1	1	0.904	taAATAAct
HvGSK1.3	P$MYB3R5_01	MYB3R5	2585	2600	1	1	0.949	aataacttaCCGTTt
HvGSK1.3	P$MYB3R1_01	MYB3R1	2586	2601	1	1	0.958	ataacttaCCGTTta
HvGSK1.3	P$MYB3R4_01	MYB3R4	2586	2601	1	1	0.951	ataacttaCCGTTta
HvGSK1.3	P$MYB24_01	MYB24	2595	2604	1	1	0.939	cgtTTAGGt
HvGSK1.3	P$MYB131_01	MYB131	2595	2606	1	1	0.9	cgtTTAGGtta
HvGSK1.3	P$AT3G60580_01	AT3G60580	2605	2612	1	1	0.951	atATCCC
HvGSK1.3	P$MYBAS1_01	MYBAS1	2617	2628	1	1	0.955	ttCCAACccca
HvGSK1.3	P$GAMYB_01	GAMYB	2620	2628	1	1	0.893	CAACCcca
HvGSK1.3	P$MYBAS1_01	MYBAS1	2623	2634	1	1	0.954	ccCCAACcaaa
HvGSK1.3	P$AT4G36620_01	AT4G36620	2624	2632	1	1	0.885	cccAACCA
HvGSK1.3	P$GAMYB_01	GAMYB	2626	2634	1	1	0.901	CAACCaaa
HvGSK1.3	P$HSFA2_01	HSFA2	2629	2635	1	1	0.941	CCAAAc
HvGSK1.3	P$AT5G04340_01	AT5G04340	2679	2691	1	1	0.889	tgatAGAAAcag
HvGSK1.3	P$BPC1_Q2	BPC1	2683	2689	1	1	0.99	AGAAAc
HvGSK1.3	P$AT4G12750_01	AT4G12750	2716	2726	1	1	0.878	tacACCGAtg
HvGSK1.3	P$RAV2_01	RAV2	2717	2726	1	1	0.879	acACCGAtg
HvGSK1.3	P$AT3G18650_01	AT3G18650	2722	2733	1	1	0.88	gatgtTTGTAt
HvGSK1.3	P$ARR18_01	ARR18	2754	2767	1	1	0.903	aagaAGATAtttc
HvGSK1.3	P$CBF3_02	CBF3	2764	2778	1	1	0.964	ttctgCCGACtata
HvGSK1.3	P$CBF1_04	CBF1	2765	2777	1	1	0.952	tctgCCGACtat
HvGSK1.3	P$CBF1_01	CBF1	2766	2776	1	1	0.897	cTGCCGacta
HvGSK1.3	P$ERF019_01	ERF019	2766	2776	1	1	0.874	cTGCCGacta
HvGSK1.3	P$JERF3_01	JERF3	2766	2776	1	1	0.943	cTGCCGacta
HvGSK1.3	P$CEF1_01	CEF1	2766	2776	1	1	0.945	cTGCCGacta
HvGSK1.3	P$JERF1_01	JERF1	2766	2776	1	1	0.968	cTGCCGacta
HvGSK1.3	P$CBF1_03	CBF1	2766	2776	1	1	0.899	cTGCCGacta

HvGSK1.3	P$DREB1F_01	DREB1F	2766	2776	1	1	0.912	cTGCCGacta
HvGSK1.3	P$AT1G33760_01	AT1G33760	2766	2776	1	1	0.876	cTGCCGacta
HvGSK1.3	P$AT1G71520_01	AT1G71520	2766	2776	1	1	0.859	cTGCCGacta
HvGSK1.3	P$DREB1E_02	DREB1E	2766	2776	1	1	0.855	cTGCCGacta
HvGSK1.3	P$ERF5_02	ERF5	2766	2776	1	1	0.954	ctGCCGActa
HvGSK1.3	P$ERF1_04	ERF1	2766	2776	1	1	0.959	ctGCCGActa
HvGSK1.3	P$DREB1G_02	DREB1G	2766	2776	1	1	0.92	ctgCCGACta
HvGSK1.3	P$ARF5_01	ARF5	2768	2776	1	1	0.903	gCCGACta
HvGSK1.3	P$DREB1B_01	DREB1B	2769	2774	1	1	1	CCGAC
HvGSK1.3	P$FHY3_01	FHY3	2781	2793	1	1	0.888	tgcACGCGttta
HvGSK1.3	P$CMTA2_01	CMTA2	2781	2790	1	1	0.986	tgcaCGCGT
HvGSK1.3	P$CAMTA1_02	CAMTA1	2781	2793	1	1	0.889	tgcaCGCGTtta
HvGSK1.3	P$CMTA3_01	CMTA3	2784	2793	1	1	0.985	aCGCGTtta
HvGSK1.3	P$ATHB6_01	ATHB6	2795	2804	1	1	0.911	aaAATAAgt
HvGSK1.3	P$GT1_Q6_01	GT1	2811	2823	1	1	0.862	TTTGTatgtaag
HvGSK1.3	P$ATSPL8_01	ATSPL8	2828	2844	1	1	0.928	gcagtTGTACcttgtt
HvGSK1.3	P$ATHB7_01	ATHB7	2871	2881	1	1	0.921	gaAATCAttc
HvGSK1.3	P$HAT1_01	HAT1	2871	2881	1	1	0.982	gaAATCAttc
HvGSK1.3	P$ATHB4_02	ATHB4	2872	2882	1	1	0.922	aaATCATtcc
HvGSK1.3	P$ASR1_01	ASR1	2886	2891	1	1	1	ACCCA
HvGSK1.3	P$GATA15_01	GATA15	2891	2900	1	1	0.999	gcTGATCca
HvGSK1.3	P$MYBAS1_01	MYBAS1	2895	2906	1	1	0.941	atCCAACactg
HvGSK1.3	P$RAV1_01	RAV1	2895	2907	1	1	0.934	atcCAACActgg
HvGSK1.3	P$TGA1B_01	TGA1B	2904	2914	1	1	0.92	tgGACGTctc
HvGSK1.3	P$TGA1A_01	TGA1A	2906	2913	1	1	0.857	gACGTCt
HvGSK1.3	P$AT3G63350_01	AT3G63350	2914	2920	1	1	0.867	CCGCCt
HvGSK1.3	P$BZR1_01	BZR1	2924	2930	1	1	1	CGTGCg
HvGSK1.3	P$PIL5_01	IL5	2924	2938	1	1	0.873	cgtgcgaaACGTGt
HvGSK1.3	P$ABF4_Q2	ABF4	2926	2940	1	1	0.915	tgcgaaACGTGtcc
HvGSK1.3	P$ABZ1_01	ABZ1	2927	2941	1	1	0.901	gcgaaACGTGtcca
HvGSK1.3	P$GBF1_Q2_01	GBF1	2928	2939	1	1	0.92	cgaaACGTGtc
HvGSK1.3	P$HBP1A_Q2	HBP1A	2929	2939	1	1	0.928	gaaACGTGtc
HvGSK1.3	P$TAF1_Q2	TAF1	2929	2939	1	1	0.936	gaaACGTGtc
HvGSK1.3	P$EMBP1_02	EMBP1	2929	2939	1	1	0.888	gaaACGTGtc
HvGSK1.3	P$TAF1_01	TAF1	2929	2939	1	1	0.952	gaaACGTGtc
HvGSK1.3	P$HY5_01	HY5	2930	2940	1	1	0.872	aaACGTGtcc
HvGSK1.3	P$GBF1_01	GBF1	2930	2938	1	1	0.963	aaACGTGt
HvGSK1.3	P$BIM1_02	BIM1	2930	2940	1	1	0.948	aaACGTGtcc
HvGSK1.3	P$ABF4_02	ABF4	2930	2940	1	1	0.999	aaACGTGtcc
HvGSK1.3	P$GBF1_Q2	GBF1	2931	2940	1	1	0.871	aACGTGtcc
HvGSK1.3	P$ABI5_Q2	ABI5	2932	2938	1	1	0.979	ACGTGt
HvGSK1.3	P$O2_Q4	O2	2935	2946	1	1	0.853	tgtcCATGTgg
HvGSK1.3	P$AMS_01	AMS	2937	2947	1	1	0.874	tcCATGTgga
HvGSK1.3	P$AT4G36620_01	AT4G36620	2943	2951	1	1	0.884	tggAACCA
HvGSK1.3	P$SED_Q2	SED	2961	2971	1	1	0.999	atatCCTTTt
HvGSK1.3	P$PBF_Q2_01	BF	2965	2971	1	1	1	CCTTTt
HvGSK1.3	P$SQUA_01	SQUA	2965	2975	1	1	0.859	cctTTTTTcc
HvGSK1.3	P$GT1_Q6_01	GT1	2968	2980	1	1	0.897	TTTTTccttatc
HvGSK1.3	P$CBNAC_01	CBNAC	2979	2985	1	1	0.973	cTGCTT
HvGSK1.3	P$AT2G41690_01	AT2G41690	2986	2992	1	1	1	CCGAAc
HvGSK1.3	P$MYBAS1_01	MYBAS1	3001	3012	1	1	0.98	tcCCAACtcat
HvGSK1.3	P$LIM1_01	LIM1	3027	3039	1	1	0.975	CCACCatcacct
HvGSK1.3	P$ROM_Q2	ROM	3032	3041	1	1	0.861	atCACCTca
HvGSK1.3	P$WRKY48_01	WRKY48	3046	3055	1	1	0.887	ctgtAACAA
HvGSK1.3	P$ATMYB15_Q2	ATMYB15	3049	3055	1	1	1	TAACAa
HvGSK1.3	P$AT2G20350_01	AT2G20350	3059	3069	1	1	0.884	caGCCGAtcg
HvGSK1.3	P$ERF73_01	ERF73	3060	3081	1	1	0.912	agccgatCGCCGccacgttct
HvGSK1.3	P$ERF4_05	ERF4	3063	3078	1	1	0.908	cgatCGCCGccacgt
HvGSK1.3	P$RRTF1_02	RRTF1	3065	3075	1	1	0.948	atCGCCGcca
HvGSK1.3	P$RAP26_03	RAP26	3065	3075	1	1	0.932	atCGCCGcca
HvGSK1.3	P$RAP210_04	RAP210	3065	3075	1	1	0.915	atCGCCGcca
HvGSK1.3	P$ERF112_02	ERF112	3065	3075	1	1	0.987	atCGCCGcca
HvGSK1.3	P$CRF4_01	CRF4	3066	3074	1	1	0.978	tCGCCGcc
HvGSK1.3	P$ERF4_04	ERF4	3066	3074	1	1	0.986	tCGCCGcc
HvGSK1.3	P$ERF069_01	ERF069	3066	3075	1	1	0.999	tCGCCGcca
HvGSK1.3	P$ERF11_01	ERF11	3066	3076	1	1	0.997	tCGCCGccac
HvGSK1.3	P$ABI4_03	ABI4	3066	3076	1	1	0.92	tcGCCGCcac
HvGSK1.3	P$WRAF1_01	WRAF1	3066	3076	1	1	0.986	tcGCCGCcac
HvGSK1.3	P$WRAF2_01	WRAF2	3066	3076	1	1	0.985	tcGCCGCcac
HvGSK1.3	P$PTI5_01	TI5	3066	3076	1	1	0.985	tcGCCGCcac
HvGSK1.3	P$DREBI5_01	DREBI5	3066	3076	1	1	0.88	tcGCCGCcac
HvGSK1.3	P$AT2G47520_01	AT2G47520	3066	3076	1	1	0.961	tcGCCGCcac
HvGSK1.3	P$DREB2B_01	DREB2B	3066	3076	1	1	0.862	tcGCCGCcac
HvGSK1.3	P$CRF1_02	CRF1	3066	3076	1	1	0.913	tcGCCGCcac
HvGSK1.3	P$OPBP1_01	OPBP1	3066	3076	1	1	0.96	tcGCCGCcac
HvGSK1.3	P$ATERF14_01	ATERF14	3066	3076	1	1	0.942	tcGCCGCcac
HvGSK1.3	P$ERF112_01	ERF112	3066	3076	1	1	0.987	tcGCCGCcac
HvGSK1.3	P$ERF1_02	ERF1	3066	3076	1	1	0.95	tcGCCGCcac

HvGSK1.3	P$ERF4_02	ERF4	3066	3076	1	1	0.962	tcGCCGCcac
HvGSK1.3	P$AT5G25390_01	AT5G25390	3066	3076	1	1	0.901	tcGCCGCcac
HvGSK1.3	P$EREBP1_01	EREBP1	3066	3076	1	1	0.965	tcGCCGCcac
HvGSK1.3	P$ERF110_02	ERF110	3066	3076	1	1	0.983	tcGCCGCcac
HvGSK1.3	P$CBF3_01	CBF3	3066	3076	1	1	0.85	tcGCCGCcac
HvGSK1.3	P$DREBII1_01	DREBII1	3066	3076	1	1	0.863	tcGCCGCcac
HvGSK1.3	P$DREB1A_01	DREB1A	3066	3076	1	1	0.873	tcGCCGCcac
HvGSK1.3	P$TSRF1_01	TSRF1	3066	3076	1	1	0.959	tcGCCGCcac
HvGSK1.3	P$DRF13_01	DRF13	3066	3076	1	1	0.877	tcGCCGCcac
HvGSK1.3	P$ERF4_03	ERF4	3066	3076	1	1	0.854	tcGCCGCcac
HvGSK1.3	P$ERF2_03	ERF2	3066	3076	1	1	0.954	tcGCCGCcac
HvGSK1.3	P$ERF1B_03	ERF1B	3066	3076	1	1	0.95	tcGCCGCcac
HvGSK1.3	P$RAP26_02	RAP26	3066	3076	1	1	0.982	tcGCCGCcac
HvGSK1.3	P$CBF16_01	CBF16	3066	3076	1	1	0.859	tcGCCGCcac
HvGSK1.3	P$CBF17_01	CBF17	3066	3076	1	1	0.879	tcGCCGCcac
HvGSK1.3	P$ERF1_05	ERF1	3066	3076	1	1	0.912	tcGCCGCcac
HvGSK1.3	P$AT5G25190_01	AT5G25190	3066	3076	1	1	0.913	tcGCCGCcac
HvGSK1.3	P$ERF105_01	ERF105	3066	3076	1	1	0.911	tcGCCGCcac
HvGSK1.3	P$CBF_01	CBF	3066	3076	1	1	0.884	tcGCCGCcac
HvGSK1.3	P$AT5G11190_01	AT5G11190	3066	3076	1	1	0.895	tcGCCGCcac
HvGSK1.3	P$AT1G68550_01	AT1G68550	3066	3076	1	1	0.971	tcGCCGCcac
HvGSK1.3	P$AT1G77640_01	AT1G77640	3066	3076	1	1	0.889	tcGCCGCcac
HvGSK1.3	P$AT3G61630_01	AT3G61630	3066	3076	1	1	0.99	tcGCCGCcac
HvGSK1.3	P$AT5G43410_01	AT5G43410	3066	3076	1	1	0.95	tcGCCGCcac
HvGSK1.3	P$RAP26L_02	RAP26L	3066	3076	1	1	0.98	tcGCCGCcac
HvGSK1.3	P$AT5G07310_01	AT5G07310	3066	3076	1	1	0.985	tcGCCGCcac
HvGSK1.3	P$DREB1A_03	DREB1A	3066	3076	1	1	0.855	tcGCCGCcac
HvGSK1.3	P$AT1G49120_01	AT1G49120	3066	3076	1	1	0.886	tcGCCGCcac
HvGSK1.3	P$DREB2D_01	DREB2D	3066	3076	1	1	0.882	tcGCCGCcac
HvGSK1.3	P$AT3G25890_01	AT3G25890	3066	3076	1	1	0.885	tcGCCGCcac
HvGSK1.3	P$AT4G23750_01	AT4G23750	3066	3076	1	1	0.857	tcGCCGCcac
HvGSK1.3	P$AT4G27950_01	AT4G27950	3066	3076	1	1	0.902	tcGCCGCcac
HvGSK1.3	P$RRTF1_01	RRTF1	3066	3076	1	1	1	tcGCCGCcac
HvGSK1.3	P$ERF1_Q2_01	ERF1	3066	3080	1	1	0.888	tcGCCGCcacgttc
HvGSK1.3	P$CRF2_01	CRF2	3066	3074	1	1	0.988	tcGCCGCc
HvGSK1.3	P$ERF096_01	ERF096	3066	3076	1	1	1	tcGCCGCcac
HvGSK1.3	P$ERF098_01	ERF098	3066	3074	1	1	0.999	tcGCCGCc
HvGSK1.3	P$CBF1_02	CBF1	3066	3076	1	1	0.882	tcgCCGCCac
HvGSK1.3	P$ERF8_01	ERF8	3067	3077	1	1	0.998	CGCCGccacg
HvGSK1.3	P$ERF3_04	ERF3	3067	3075	1	1	1	CGCCGcca
HvGSK1.3	P$OS05G0497200_01	OS05G0497200	3067	3075	1	1	1	CGCCGcca
HvGSK1.3	P$ERF1B_06	ERF1B	3067	3075	1	1	1	cGCCGCca
HvGSK1.3	P$ERF7_02	ERF7	3067	3077	1	1	0.999	cGCCGCcacg
HvGSK1.3	P$ERF094_01	ERF094	3067	3075	1	1	0.995	cGCCGCca
HvGSK1.3	P$ERF2_01	ERF2	3067	3074	1	1	1	cgCCGCC
HvGSK1.3	P$ERF13_02	ERF13	3067	3075	1	1	1	cgCCGCCa
HvGSK1.3	P$ABF2_01	ABF2	3068	3081	1	1	0.905	gccgcCACGTtct
HvGSK1.3	P$AT3G63350_01	AT3G63350	3069	3075	1	1	0.882	CCGCCa
HvGSK1.3	P$BZR1_02	BZR1	3069	3083	1	1	0.854	ccgcCACGTtctca
HvGSK1.3	P$GBP_Q6	GBP	3070	3082	1	1	0.907	cgcCACGTtctc
HvGSK1.3	P$ABI5_01	ABI5	3070	3080	1	1	0.936	cgcCACGTtc
HvGSK1.3	P$ABF4_01	ABF4	3070	3082	1	1	0.875	cgcCACGTtctc
HvGSK1.3	P$EMBP1_Q2	EMBP1	3071	3081	1	1	0.936	gcCACGTtct
HvGSK1.3	P$CPRF3_Q2	CPRF3	3071	3081	1	1	0.915	gcCACGTtct
HvGSK1.3	P$CPRF2_Q2	CPRF2	3071	3081	1	1	0.93	gcCACGTtct
HvGSK1.3	P$O2_02	O2	3071	3081	1	1	0.95	gcCACGTtct
HvGSK1.3	P$TGA1B_Q2	TGA1B	3071	3081	1	1	0.903	gcCACGTtct
HvGSK1.3	P$TGA1A_Q2	TGA1A	3071	3081	1	1	0.961	gcCACGTtct
HvGSK1.3	P$CPRF3_01	CPRF3	3071	3081	1	1	0.916	gcCACGTtct
HvGSK1.3	P$CPRF2_01	CPRF2	3071	3081	1	1	0.931	gcCACGTtct
HvGSK1.3	P$TGA1B_01	TGA1B	3071	3081	1	1	0.891	gcCACGTtct
HvGSK1.3	P$BEE2_01	BEE2	3071	3081	1	1	0.92	gcCACGTtct
HvGSK1.3	P$BIM2_01	BIM2	3071	3081	1	1	0.855	gcCACGTtct
HvGSK1.3	P$BIM3_01	BIM3	3071	3081	1	1	0.889	gcCACGTtct
HvGSK1.3	P$PHYPA143875_02	HYPA143875	3071	3081	1	1	0.887	gcCACGTtct
HvGSK1.3	P$SPT_01	SPT	3071	3080	1	1	0.941	gcCACGTtc
HvGSK1.3	P$GBF1F_Q2	GBF1F	3071	3082	1	1	0.977	gcCACGTtctc
HvGSK1.3	P$RITA1_01	RITA1	3072	3079	1	1	0.966	cCACGTt
HvGSK1.3	P$MYC3_01	MYC3	3072	3080	1	1	0.857	cCACGTtc
HvGSK1.3	P$OCSBF1_01	OCSBF1	3073	3078	1	1	1	CACGT
HvGSK1.3	P$BZR1_01	BZR1	3091	3097	1	1	0.902	CGTGCt
HvGSK1.3	P$CBNAC_01	CBNAC	3092	3098	1	1	0.979	gTGCTT
HvGSK1.3	P$CBF3_02	CBF3	3107	3121	1	1	0.979	tcgcaCCGACaatc
HvGSK1.3	P$CBF1_04	CBF1	3108	3120	1	1	0.966	cgcaCCGACaat
HvGSK1.3	P$DREB1G_02	DREB1G	3109	3119	1	1	0.915	gcaCCGACaa
HvGSK1.3	P$AT1G77200_03	AT1G77200	3109	3123	1	1	0.986	gcaCCGACaatcgt
HvGSK1.3	P$ARF1_01	ARF1	3111	3119	1	1	1	aCCGACaa
HvGSK1.3	P$ARF5_01	ARF5	3111	3119	1	1	0.994	aCCGACaa

HvGSK1.3	P$DREB1B_01	DREB1B	3112	3117	1	1	1	CCGAC
HvGSK1.3	P$ERF112_02	ERF112	3125	3135	1	1	0.964	ttCGCCGgcg
HvGSK1.3	P$CRF4_01	CRF4	3126	3134	1	1	0.92	tCGCCGgc
HvGSK1.3	P$ERF4_04	ERF4	3126	3134	1	1	0.948	tCGCCGgc
HvGSK1.3	P$ERF069_01	ERF069	3126	3135	1	1	0.992	tCGCCGgcg
HvGSK1.3	P$ERF11_01	ERF11	3126	3136	1	1	0.985	tCGCCGgcga
HvGSK1.3	P$ERF6_02	ERF6	3126	3136	1	1	0.99	tcGCCGGcga
HvGSK1.3	P$ERF8_01	ERF8	3127	3137	1	1	0.983	CGCCGgcgag
HvGSK1.3	P$ERF3_04	ERF3	3127	3135	1	1	0.956	CGCCGgcg
HvGSK1.3	P$ERF105_02	ERF105	3127	3135	1	1	0.97	cGCCGGcg
HvGSK1.3	P$AT1G68550_03	AT1G68550	3127	3136	1	1	0.953	cgcCGGCGa
HvGSK1.3	P$HSFA1E_01	HSFA1E	3129	3135	1	1	1	cCGGCG
HvGSK1.3	P$MYB24_01	MYB24	3134	3143	1	1	0.923	gagTTAGGa
HvGSK1.3	P$UIF1_01	UIF1	3138	3148	1	1	0.856	tagGATTCta
HvGSK1.3	P$P_01		3143	3152	1	1	0.876	ttCTACCgt
HvGSK1.3	P$SED_Q2	SED	3148	3158	1	1	0.949	ccgtCCTTTc
HvGSK1.3	P$PBF_Q2_01	BF	3152	3158	1	1	0.985	CCTTTc
HvGSK1.3	P$SED_Q2	SED	3153	3163	1	1	0.992	ctttCCTTTt
HvGSK1.3	P$PBF_Q2_01	BF	3157	3163	1	1	1	CCTTTt
HvGSK1.3	P$HSFA4A_01	HSFA4A	3164	3170	1	1	0.91	gCTATT
HvGSK1.3	P$SQUA_01	SQUA	3165	3175	1	1	0.911	ctaTTTTTct
HvGSK1.3	P$CBNAC_01	CBNAC	3197	3203	1	1	1	tTGCTT
HvGSK1.3	P$CBNAC_02	CBNAC	3197	3213	1	1	0.922	tTGCTTttcaggcgag
HvGSK1.3	P$ABI3_01	ABI3	3211	3220	1	1	0.851	agGCATGac
HvGSK1.3	P$ABI3_01	ABI3	3224	3233	1	1	0.982	cgGCATGct
HvGSK1.3	P$FUS3_01	FUS3	3225	3234	1	1	0.943	gGCATGctt
HvGSK1.3	P$LEC2_01	LEC2	3225	3236	1	1	0.943	ggCATGCttct
HvGSK1.3	P$IDEF1_Q2	IDEF1	3227	3239	1	1	0.851	CATGCttctgga
HvGSK1.3	P$CBNAC_01	CBNAC	3228	3234	1	1	0.968	aTGCTT
HvGSK1.3	P$CBNAC_02	CBNAC	3228	3244	1	1	0.867	aTGCTTctggaatagc
HvGSK1.3	P$RAP21_02	RAP21	3274	3287	1	1	0.954	ttggCGGTGatgt
HvGSK1.3	P$E2L_Q2	E2L	3275	3282	1	1	0.892	tGGCGGt
HvGSK1.3	P$RAV1_01	RAV1	3296	3308	1	1	0.946	aggCAACAtcgg
HvGSK1.3	P$HSFA4A_01	HSFA4A	3329	3335	1	1	0.914	tCTATT
HvGSK1.3	P$PBF_01	BF	3363	3374	1	1	0.954	tgcAAAAGttc
HvGSK1.3	P$DOF_Q2	DOF	3363	3374	1	1	0.918	tgcAAAAGttc
HvGSK1.3	P$CDF2_01	CDF2	3364	3374	1	1	0.964	gcAAAAGttc
HvGSK1.3	P$CDF3_01	CDF3	3365	3374	1	1	0.984	cAAAAGttc
HvGSK1.3	P$PEND_02	END	3369	3379	1	1	0.903	agTTCTTttg
HvGSK1.3	P$RAV1_01	RAV1	3376	3388	1	1	0.955	ttgCAACAtcac
HvGSK1.3	P$PBF_01	BF	3396	3407	1	1	0.991	caaAAAAGtga
HvGSK1.3	P$DOF_Q2	DOF	3396	3407	1	1	0.984	caaAAAAGtga
HvGSK1.3	P$CDF2_01	CDF2	3397	3407	1	1	1	aaAAAAGtga
HvGSK1.3	P$CDF3_01	CDF3	3398	3407	1	1	1	aAAAAGtga
HvGSK1.3	P$GT1_Q6	GT1	3403	3410	1	1	0.912	GTGAAga
HvGSK1.3	P$AT3G51080_01	AT3G51080	3411	3418	1	1	0.893	GGAAAat
HvGSK1.3	P$ATHB6_01	ATHB6	3413	3422	1	1	0.905	aaAATAAaa
HvGSK1.3	P$GAMYB_Q2	GAMYB	3421	3434	1	1	0.907	attctACAACacg
HvGSK1.3	P$ATHSFA1D_01	ATHSFA1D	3423	3429	1	1	0.941	tCTACA
HvGSK1.3	P$RAV1_01	RAV1	3424	3436	1	1	0.923	ctaCAACAcgat
HvGSK1.3	P$GATA8_01	GATA8	3431	3440	1	1	0.99	acGATCTat
HvGSK1.3	P$SQUA_01	SQUA	3444	3454	1	1	0.867	caaTTTTTtt
HvGSK1.3	P$E2F_Q2	E2F	3450	3461	1	1	0.976	tttTTCCCgca
HvGSK1.3	P$RAV1_01	RAV1	3456	3468	1	1	0.986	ccgCAACAcaac
HvGSK1.3	P$GAMYB_Q2	GAMYB	3458	3471	1	1	0.852	gcaacACAACctg
HvGSK1.3	P$RAV1_02	RAV1	3463	3475	1	1	0.914	acaACCTGtgtt
HvGSK1.3	P$NAC6_01	NAC6	3485	3491	1	1	0.854	tCGTAA
HvGSK1.3	P$ATMYB15_Q2	ATMYB15	3488	3494	1	1	1	TAACAa
HvGSK1.3	P$SED_Q2	SED	3492	3502	1	1	0.902	aaaaCCTTTg
HvGSK1.3	P$PBF_Q2_01	BF	3496	3502	1	1	0.988	CCTTTg
HvGSK1.3	P$ATMYB15_Q2	ATMYB15	3517	3523	1	1	1	TAACAa
HvGSK1.3	P$PBF_01	BF	3534	3545	1	1	0.963	caaAAAAGgcg
HvGSK1.3	P$DOF_Q2	DOF	3534	3545	1	1	0.978	caaAAAAGgcg
HvGSK1.3	P$CDF2_01	CDF2	3535	3545	1	1	0.975	aaAAAAGgcg
HvGSK1.3	P$CDF3_01	CDF3	3536	3545	1	1	0.974	aAAAAGgcg
HvGSK1.3	P$PBF_Q2	BF	3537	3543	1	1	1	aAAAGG
HvGSK1.3	P$P_01		3551	3560	1	1	0.876	tgCTACCgt
HvGSK1.3	P$AT1G53910_02	AT1G53910	3571	3592	1	1	0.86	cggctcgtcaaGCGGCggatc
HvGSK1.3	P$HBPA1_Q6_01	HBPA1	3572	3582	1	1	0.861	ggctCGTCAa
HvGSK1.3	P$WRKY18_02	WRKY18	3574	3584	1	1	0.948	ctcGTCAAgc
HvGSK1.3	P$WRKY21_02	WRKY21	3574	3584	1	1	0.946	ctcGTCAAgc
HvGSK1.3	P$WRKY48_02	WRKY48	3574	3584	1	1	0.987	ctcGTCAAgc
HvGSK1.3	P$WRKY57_01	WRKY57	3574	3584	1	1	0.952	ctcGTCAAgc
HvGSK1.3	P$WRKY60_01	WRKY60	3574	3585	1	1	0.89	ctcGTCAAgcg
HvGSK1.3	P$WRKY15_01	WRKY15	3575	3585	1	1	0.958	tcGTCAAgcg
HvGSK1.3	P$WRKY2_01	WRKY2	3575	3583	1	1	0.903	tcGTCAAg
HvGSK1.3	P$WRKY25_02	WRKY25	3575	3583	1	1	0.887	tcGTCAAg
HvGSK1.3	P$WRKY40_01	WRKY40	3575	3583	1	1	0.981	tcGTCAAg

HvGSK1.3	P$WRKY43_02	WRKY43	3575	3585	1	1	0.951	tcGTCAAgcg
HvGSK1.3	P$WRKY62_01	WRKY62	3575	3583	1	1	0.87	tcGTCAAg
HvGSK1.3	P$WRKY63_01	WRKY63	3575	3583	1	1	0.888	tcGTCAAg
HvGSK1.3	P$WRKY75_01	WRKY75	3575	3583	1	1	0.92	tcGTCAAg
HvGSK1.3	P$WRKY8_01	WRKY8	3575	3584	1	1	0.977	tcGTCAAgc
HvGSK1.3	P$WRKY30_01	WRKY30	3576	3586	1	1	0.903	cGTCAAgcgg
HvGSK1.3	P$RRTF1_05	RRTF1	3576	3591	1	1	0.888	cgtcaagCGGCGgat
HvGSK1.3	P$WRKY18_Q2	WRKY18	3577	3586	1	1	0.949	GTCAAgcgg
HvGSK1.3	P$AT1G28160_02	AT1G28160	3580	3595	1	1	0.889	aagCGGCGgatcttt
HvGSK1.3	P$RAP26_06	RAP26	3580	3595	1	1	0.869	aagCGGCGgatcttt
HvGSK1.3	P$AT1G68550_03	AT1G68550	3580	3589	1	1	0.995	aagCGGCGg
HvGSK1.3	P$ERF1_Q2	ERF1	3584	3592	1	1	0.892	GGCGGatc
HvGSK1.3	P$ARR2_01	ARR2	3585	3595	1	1	0.987	gcggATCTTt
HvGSK1.3	P$GATA8_01	GATA8	3586	3595	1	1	0.976	cgGATCTTt
HvGSK1.3	P$AT4G36620_01	AT4G36620	3594	3602	1	1	0.898	tcaAACCA
HvGSK1.3	P$SED_Q2	SED	3610	3620	1	1	0.909	cataCCTTTc
HvGSK1.3	P$PBF_Q2_01	BF	3614	3620	1	1	0.985	CCTTTc
HvGSK1.3	P$GATA15_01	GATA15	3637	3646	1	1	0.999	agTGATCgc
HvGSK1.3	P$ARF8_01	ARF8	3660	3669	1	1	0.958	ctTGTCGat
HvGSK1.3	P$GATA8_01	GATA8	3664	3673	1	1	0.99	tcGATCTat
HvGSK1.3	P$ARF8_01	ARF8	3674	3683	1	1	0.959	gtTGTCGac
HvGSK1.3	P$ANTL_02	ANTL	3676	3686	1	1	0.921	tgtCGACAtt
HvGSK1.3	P$ATMYB77_01	ATMYB77	3738	3751	1	1	0.86	ttgtatCGGTTag
HvGSK1.3	P$PHYPA64121_06	HYPA64121	3740	3753	1	1	0.867	gtaTCGGTtagtt
HvGSK1.3	P$WEREWOLF_Q2	WEREWOLF	3744	3753	1	1	0.937	cgGTTAGtt
HvGSK1.3	P$BPC1_Q2	BPC1	3771	3777	1	1	0.997	AGAAAa
HvGSK1.3	P$SBF1_01	SBF1	3811	3825	1	1	0.861	atcgtaTTAAAaaa
HvGSK1.3	P$PBF_01	BF	3819	3830	1	1	0.969	aaaAAAAGgta
HvGSK1.3	P$DOF_Q2	DOF	3819	3830	1	1	0.982	aaaAAAAGgta
HvGSK1.3	P$CDF2_01	CDF2	3820	3830	1	1	0.979	aaAAAAGgta
HvGSK1.3	P$CDF3_01	CDF3	3821	3830	1	1	0.977	aAAAAGgta
HvGSK1.3	P$PBF_Q2	BF	3822	3828	1	1	1	aAAAGG
HvGSK1.3	P$ATHB6_01	ATHB6	3847	3856	1	1	0.905	aaAATAAaa
HvGSK1.3	P$O2_Q4	O2	3856	3867	1	1	0.866	tacaCATGTag
HvGSK1.3	P$PCF2_01	CF2	3878	3888	1	1	0.997	acggcCCCAC
HvGSK1.3	P$TCP19_01	TCP19	3878	3888	1	1	0.982	acggcCCCAC
HvGSK1.3	P$TCP20L_01	TCP20L	3879	3888	1	1	0.991	cggcCCCAC
HvGSK1.3	P$OSI_01	OSI	3880	3888	1	1	0.959	ggcCCCAC
HvGSK1.3	P$TCP20_02	TCP20	3880	3890	1	1	0.998	ggcCCCACac
HvGSK1.3	P$ARALY495258_02	ARALY495258	3880	3888	1	1	1	ggcCCCAC
HvGSK1.3	P$ARALY493022_04	ARALY493022	3880	3888	1	1	0.973	ggcCCCAC
HvGSK1.3	P$ARALY484486_05	ARALY484486	3880	3888	1	1	1	ggcCCCAC
HvGSK1.3	P$KNOX3_01	KNOX3	3890	3902	1	1	0.974	cacaTGACAcgg
HvGSK1.3	P$ATH1_01	ATH1	3894	3902	1	1	0.912	TGACAcgg
HvGSK1.3	P$BIM1_01	BIM1	3894	3906	1	1	0.894	tgACACGgggag
HvGSK1.3	P$HSF3_01	HSF3	3898	3904	1	1	0.969	aCGGGG
HvGSK1.3	P$ERF1_Q2	ERF1	3906	3914	1	1	0.946	GGCGGccc
HvGSK1.3	P$HSF3_01	HSF3	3912	3918	1	1	1	cCGGGG
HvGSK1.3	P$SED_Q2	SED	3920	3930	1	1	0.916	agaaCCTTTc
HvGSK1.3	P$PBF_Q2_01	BF	3924	3930	1	1	0.985	CCTTTc
HvGSK1.3	P$AT5G54070_01	AT5G54070	3928	3934	1	1	0.91	tCAACG
HvGSK1.3	P$WRKY18_02	WRKY18	3931	3941	1	1	0.997	acgGTCAAac
HvGSK1.3	P$WRKY21_02	WRKY21	3931	3941	1	1	0.969	acgGTCAAac
HvGSK1.3	P$WRKY48_02	WRKY48	3931	3941	1	1	0.998	acgGTCAAac
HvGSK1.3	P$WRKY57_01	WRKY57	3931	3941	1	1	0.974	acgGTCAAac
HvGSK1.3	P$WRKY60_01	WRKY60	3931	3942	1	1	0.98	acgGTCAAacg
HvGSK1.3	P$WRKY15_01	WRKY15	3932	3942	1	1	0.985	cgGTCAAacg
HvGSK1.3	P$WRKY2_01	WRKY2	3932	3940	1	1	0.991	cgGTCAAa
HvGSK1.3	P$WRKY25_02	WRKY25	3932	3940	1	1	0.979	cgGTCAAa
HvGSK1.3	P$WRKY40_01	WRKY40	3932	3940	1	1	1	cgGTCAAa
HvGSK1.3	P$WRKY43_02	WRKY43	3932	3942	1	1	0.976	cgGTCAAacg
HvGSK1.3	P$WRKY62_01	WRKY62	3932	3940	1	1	0.91	cgGTCAAa
HvGSK1.3	P$WRKY63_01	WRKY63	3932	3940	1	1	0.991	cgGTCAAa
HvGSK1.3	P$WRKY75_01	WRKY75	3932	3940	1	1	0.975	cgGTCAAa
HvGSK1.3	P$WRKY8_01	WRKY8	3932	3941	1	1	0.992	cgGTCAAac
HvGSK1.3	P$WRKY23_01	WRKY23	3933	3941	1	1	0.854	gGTCAAac
HvGSK1.3	P$WRKY30_01	WRKY30	3933	3943	1	1	0.918	gGTCAAacgg
HvGSK1.3	P$WRKY18_Q2	WRKY18	3934	3943	1	1	0.942	GTCAAacgg
HvGSK1.3	P$SBF1_01	SBF1	3951	3965	1	1	0.89	tactaaTTAATtaa
HvGSK1.3	P$EDT1_01	EDT1	3954	3964	1	1	0.899	taaTTAATta
HvGSK1.3	P$EDT1_01	EDT1	3958	3968	1	1	0.884	taaTTAATca
HvGSK1.3	P$ATHB7_01	ATHB7	3961	3971	1	1	0.882	ttAATCAacg
HvGSK1.3	P$HAT1_01	HAT1	3961	3971	1	1	0.875	ttAATCAacg
HvGSK1.3	P$AT5G54070_01	AT5G54070	3965	3971	1	1	0.91	tCAACG
HvGSK1.3	P$PDF2_01	DF2	3969	3980	1	1	0.899	cgagTAAATga
HvGSK1.3	P$AT4G36620_01	AT4G36620	3976	3984	1	1	0.98	atgAACCA
HvGSK1.3	P$MYBAS1_01	MYBAS1	3979	3990	1	1	0.939	aaCCAACcagg
HvGSK1.3	P$AT4G36620_01	AT4G36620	3980	3988	1	1	0.909	accAACCA

HvGSK1.3	P$GAMYB_01	GAMYB	3982	3990	1	1	0.873	CAACCagg
HvGSK1.3	P$EDT1_01	EDT1	3988	3998	1	1	0.859	ggaTTAATta
HvGSK1.3	P$EDT1_01	EDT1	3992	4002	1	1	0.899	taaTTAATta
HvGSK2.1	P$GAMYB_Q2	GAMYB	4	17	1	1	0.91	ttcatACAACccc
HvGSK2.1	P$GAMYB_01	GAMYB	10	18	1	1	0.871	CAACCcct
HvGSK2.1	P$MYBAS1_01	MYBAS1	36	47	1	1	0.951	ctCTAACtata
HvGSK2.1	P$C1_Q2	C1	38	49	1	1	0.959	ctAACTAtact
HvGSK2.1	P$HSFA4A_01	HSFA4A	46	52	1	1	1	aCTATT
HvGSK2.1	P$TEIL_01	TEIL	62	70	1	1	0.887	ATGTAagt
HvGSK2.1	P$ID1_01	ID1	71	82	1	1	0.894	tTTGTCtttga
HvGSK2.1	P$ARR1_01	ARR1	95	105	1	1	0.985	ctaGAATCtc
HvGSK2.1	P$AT5G38620_01	AT5G38620	104	120	1	1	0.926	cattagcATGGTaatt
HvGSK2.1	P$ABI3_01	ABI3	107	116	1	1	0.866	taGCATGgt
HvGSK2.1	P$AT3G18650_01	AT3G18650	125	136	1	1	0.972	gacacTTGTAg
HvGSK2.1	P$BPC1_Q2	BPC1	153	159	1	1	0.997	AGAAAa
HvGSK2.1	P$FLC_01	FLC	155	176	1	1	0.878	aaaatataaaactAGAAAaag
HvGSK2.1	P$C1_Q2	C1	162	173	1	1	0.917	aaAACTAgaaa
HvGSK2.1	P$AT5G04340_01	AT5G04340	164	176	1	1	0.877	aactAGAAAaag
HvGSK2.1	P$RIN_Q2_01	RIN	165	177	1	1	0.924	actagaAAAAGa
HvGSK2.1	P$BPC1_Q2	BPC1	168	174	1	1	0.997	AGAAAa
HvGSK2.1	P$PBF_01	BF	168	179	1	1	0.955	agaAAAAGaat
HvGSK2.1	P$DOF_Q2	DOF	168	179	1	1	0.995	agaAAAAGaat
HvGSK2.1	P$CDF2_01	CDF2	169	179	1	1	0.977	gaAAAAGaat
HvGSK2.1	P$CDF3_01	CDF3	170	179	1	1	0.974	aAAAAGaat
HvGSK2.1	P$ARR1_01	ARR1	172	182	1	1	0.952	aaaGAATCac
HvGSK2.1	P$ATHB7_01	ATHB7	174	184	1	1	0.854	agAATCActt
HvGSK2.1	P$HAT1_01	HAT1	174	184	1	1	0.862	agAATCActt
HvGSK2.1	P$GATA15_01	GATA15	182	191	1	1	0.999	ttTGATCtt
HvGSK2.1	P$ARR2_01	ARR2	182	192	1	1	0.853	tttgATCTTc
HvGSK2.1	P$GATA11_01	GATA11	183	191	1	1	0.854	ttGATCTt
HvGSK2.1	P$GATA8_01	GATA8	183	192	1	1	0.975	ttGATCTtc
HvGSK2.1	P$HSFA4A_01	HSFA4A	198	204	1	1	0.914	tCTATT
HvGSK2.1	P$AT4G36620_01	AT4G36620	212	220	1	1	0.969	ctaAACCA
HvGSK2.1	P$PCF2_01	CF2	226	236	1	1	0.89	gcgatCCCAC
HvGSK2.1	P$TCP19_01	TCP19	226	236	1	1	0.905	gcgatCCCAC
HvGSK2.1	P$AT3G60580_01	AT3G60580	227	234	1	1	0.874	cgATCCC
HvGSK2.1	P$TCP20L_01	TCP20L	227	236	1	1	0.887	cgatCCCAC
HvGSK2.1	P$TCP20_02	TCP20	228	238	1	1	0.908	gatCCCACac
HvGSK2.1	P$ARALY495258_02	ARALY495258	228	236	1	1	0.918	gatCCCAC
HvGSK2.1	P$ARALY484486_05	ARALY484486	228	236	1	1	0.918	gatCCCAC
HvGSK2.1	P$LEC2_01	LEC2	242	253	1	1	0.938	gtCATGCtctc
HvGSK2.1	P$ABF2_01	ABF2	260	273	1	1	0.898	ctttgCACGTcgg
HvGSK2.1	P$O2_Q4	O2	261	272	1	1	0.949	tttgCACGTcg
HvGSK2.1	P$HBI1_01	HBI1	261	273	1	1	0.864	tttgCACGTcgg
HvGSK2.1	P$GBP_Q6	GBP	262	274	1	1	0.897	ttgCACGTcggt
HvGSK2.1	P$ABI5_01	ABI5	262	272	1	1	0.89	ttgCACGTcg
HvGSK2.1	P$ABF4_01	ABF4	262	274	1	1	0.892	ttgCACGTcggt
HvGSK2.1	P$EMBP1_Q2	EMBP1	263	273	1	1	0.857	tgCACGTcgg
HvGSK2.1	P$CPRF3_Q2	CPRF3	263	273	1	1	0.952	tgCACGTcgg
HvGSK2.1	P$CPRF2_Q2	CPRF2	263	273	1	1	0.955	tgCACGTcgg
HvGSK2.1	P$O2_02	O2	263	273	1	1	0.954	tgCACGTcgg
HvGSK2.1	P$TGA1B_Q2	TGA1B	263	273	1	1	0.908	tgCACGTcgg
HvGSK2.1	P$TGA1A_Q2	TGA1A	263	273	1	1	0.977	tgCACGTcgg
HvGSK2.1	P$CPRF3_01	CPRF3	263	273	1	1	0.962	tgCACGTcgg
HvGSK2.1	P$CPRF2_01	CPRF2	263	273	1	1	0.956	tgCACGTcgg
HvGSK2.1	P$TGA1B_01	TGA1B	263	273	1	1	0.863	tgCACGTcgg
HvGSK2.1	P$BEE2_01	BEE2	263	273	1	1	0.907	tgCACGTcgg
HvGSK2.1	P$BIM3_01	BIM3	263	273	1	1	0.879	tgCACGTcgg
HvGSK2.1	P$PHYPA143875_02	HYPA143875	263	273	1	1	0.873	tgCACGTcgg
HvGSK2.1	P$SPT_01	SPT	263	272	1	1	0.927	tgCACGTcg
HvGSK2.1	P$GBF1F_Q2	GBF1F	263	274	1	1	0.85	tgCACGTcggt
HvGSK2.1	P$RITA1_01	RITA1	264	271	1	1	0.954	gCACGTc
HvGSK2.1	P$OCSBF1_01	OCSBF1	265	270	1	1	1	CACGT
HvGSK2.1	P$TGA1A_01	TGA1A	265	272	1	1	0.871	cACGTCg
HvGSK2.1	P$DREB1A_04	DREB1A	266	276	1	1	0.982	acGTCGGtgt
HvGSK2.1	P$ERF039_01	ERF039	266	276	1	1	0.993	acGTCGGtgt
HvGSK2.1	P$PHYPA182268_05	HYPA182268	266	276	1	1	0.99	acGTCGGtgt
HvGSK2.1	P$PHYPA64121_06	HYPA64121	266	279	1	1	0.864	acgTCGGTgttgc
HvGSK2.1	P$RAP21_02	RAP21	266	279	1	1	0.948	acgtCGGTGttgc
HvGSK2.1	P$ERF043_01	ERF043	267	275	1	1	0.892	cGTCGGtg
HvGSK2.1	P$PHYPA173530_04	HYPA173530	267	275	1	1	0.957	cGTCGGtg
HvGSK2.1	P$PHYPA28324_10	HYPA28324	267	275	1	1	0.98	cGTCGGtg
HvGSK2.1	P$RRTF1_05	RRTF1	305	320	1	1	0.877	agccggtCGGCGgtg
HvGSK2.1	P$DREB1A_04	DREB1A	308	318	1	1	0.967	cgGTCGGcgg
HvGSK2.1	P$ERF039_01	ERF039	308	318	1	1	0.98	cgGTCGGcgg
HvGSK2.1	P$PHYPA182268_05	HYPA182268	308	318	1	1	0.883	cgGTCGGcgg
HvGSK2.1	P$PHYPA173530_04	HYPA173530	309	317	1	1	0.894	gGTCGGcg
HvGSK2.1	P$PHYPA28324_10	HYPA28324	309	317	1	1	0.929	gGTCGGcg

HvGSK2.1	P$AT1G28160_02	AT1G28160	309	324	1	1	0.927	ggtCGGCGgtggtgc
HvGSK2.1	P$RAP26_06	RAP26	309	324	1	1	0.911	ggtCGGCGgtggtgc
HvGSK2.1	P$AT1G68550_03	AT1G68550	309	318	1	1	0.959	ggtCGGCGg
HvGSK2.1	P$RAP21_02	RAP21	311	324	1	1	0.946	tcggCGGTGgtgc
HvGSK2.1	P$ERF1_Q2	ERF1	313	321	1	1	0.861	GGCGGtgg
HvGSK2.1	P$LEC2_01	LEC2	326	337	1	1	0.933	ctCATGCtgtc
HvGSK2.1	P$ABI3_01	ABI3	340	349	1	1	0.965	gtGCATGct
HvGSK2.1	P$FUS3_01	FUS3	341	350	1	1	0.955	tGCATGctt
HvGSK2.1	P$LEC2_01	LEC2	341	352	1	1	0.936	tgCATGCttca
HvGSK2.1	P$CBNAC_01	CBNAC	344	350	1	1	0.968	aTGCTT
HvGSK2.1	P$CBNAC_02	CBNAC	344	360	1	1	0.856	aTGCTTcaactggaga
HvGSK2.1	P$GAMYB_Q2	GAMYB	356	369	1	1	0.871	gagacACAACctg
HvGSK2.1	P$RAV1_02	RAV1	361	373	1	1	0.917	acaACCTGcaac
HvGSK2.1	P$GAMYB_01	GAMYB	362	370	1	1	0.919	CAACCtgc
HvGSK2.1	P$AT5G54070_01	AT5G54070	368	374	1	1	0.915	gCAACG
HvGSK2.1	P$PBF_01	BF	394	405	1	1	0.962	tgcAAAAGcta
HvGSK2.1	P$DOF_Q2	DOF	394	405	1	1	0.917	tgcAAAAGcta
HvGSK2.1	P$DOF2_01	DOF2	394	405	1	1	0.99	tgcaAAAGCta
HvGSK2.1	P$DOF3_01	DOF3	394	405	1	1	0.979	tgcaAAAGCta
HvGSK2.1	P$CDF2_01	CDF2	395	405	1	1	0.955	gcAAAAGcta
HvGSK2.1	P$CDF3_01	CDF3	396	405	1	1	0.976	cAAAAGcta
HvGSK2.1	P$WRKY18_02	WRKY18	406	416	1	1	0.948	accGTCAAtg
HvGSK2.1	P$WRKY21_02	WRKY21	406	416	1	1	0.953	accGTCAAtg
HvGSK2.1	P$WRKY48_02	WRKY48	406	416	1	1	0.988	accGTCAAtg
HvGSK2.1	P$WRKY57_01	WRKY57	406	416	1	1	0.958	accGTCAAtg
HvGSK2.1	P$WRKY60_01	WRKY60	406	417	1	1	0.895	accGTCAAtgc
HvGSK2.1	P$WRKY15_01	WRKY15	407	417	1	1	0.962	ccGTCAAtgc
HvGSK2.1	P$WRKY2_01	WRKY2	407	415	1	1	0.907	ccGTCAAt
HvGSK2.1	P$WRKY25_02	WRKY25	407	415	1	1	0.894	ccGTCAAt
HvGSK2.1	P$WRKY40_01	WRKY40	407	415	1	1	0.981	ccGTCAAt
HvGSK2.1	P$WRKY43_02	WRKY43	407	417	1	1	0.955	ccGTCAAtgc
HvGSK2.1	P$WRKY63_01	WRKY63	407	415	1	1	0.887	ccGTCAAt
HvGSK2.1	P$WRKY75_01	WRKY75	407	415	1	1	0.928	ccGTCAAt
HvGSK2.1	P$WRKY8_01	WRKY8	407	416	1	1	0.979	ccGTCAAtg
HvGSK2.1	P$WRKY30_01	WRKY30	408	418	1	1	0.916	cGTCAAtgct
HvGSK2.1	P$WRKY18_Q2	WRKY18	409	418	1	1	0.937	GTCAAtgct
HvGSK2.1	P$SBF1_01	SBF1	417	431	1	1	0.873	tgcaacTTAATatg
HvGSK2.1	P$KNOX3_01	KNOX3	427	439	1	1	0.984	tatgTGACAtgc
HvGSK2.1	P$ATH1_01	ATH1	431	439	1	1	0.932	TGACAtgc
HvGSK2.1	P$LEC2_01	LEC2	432	443	1	1	0.935	gaCATGCtatg
HvGSK2.1	P$TGA1_01	TGA1	438	449	1	1	0.919	ctaTGACGacc
HvGSK2.1	P$TGA7_01	TGA7	439	449	1	1	0.883	taTGACGacc
HvGSK2.1	P$TGA5_01	TGA5	440	448	1	1	0.87	aTGACGac
HvGSK2.1	P$HSFA4A_01	HSFA4A	447	453	1	1	0.964	cCTATT
HvGSK2.1	P$KNOX3_01	KNOX3	448	460	1	1	0.971	ctatTGACAgtg
HvGSK2.1	P$SIZF2_01	SIZF2	449	459	1	1	0.906	tatTGACAgt
HvGSK2.1	P$WRKY11_Q2	WRKY11	450	458	1	1	0.899	aTTGACag
HvGSK2.1	P$ZAP1_01	ZAP1	451	461	1	1	0.864	TTGACagtgc
HvGSK2.1	P$ATH1_01	ATH1	452	460	1	1	0.975	TGACAgtg
HvGSK2.1	P$CBNAC_01	CBNAC	457	463	1	1	0.979	gTGCTT
HvGSK2.1	P$MYB1L_01	MYB1L	466	476	1	1	0.981	gaCCCTAaca
HvGSK2.1	P$TRB2_01	TRB2	466	474	1	1	0.964	gaCCCTAa
HvGSK2.1	P$MYBAS1_01	MYBAS1	468	479	1	1	0.952	ccCTAACaacg
HvGSK2.1	P$GAMYB_Q2	GAMYB	468	481	1	1	0.943	ccctaACAACggt
HvGSK2.1	P$ATMYB15_Q2	ATMYB15	471	477	1	1	1	TAACAa
HvGSK2.1	P$AT1G69560_01	AT1G69560	472	484	1	1	0.868	aacAACGGtgtt
HvGSK2.1	P$AT5G54070_01	AT5G54070	473	479	1	1	0.958	aCAACG
HvGSK2.1	P$CBF1_01	CBF1	500	510	1	1	0.932	gTGCCGcaga
HvGSK2.1	P$ERF019_01	ERF019	500	510	1	1	0.894	gTGCCGcaga
HvGSK2.1	P$DREB6_01	DREB6	500	510	1	1	0.872	gTGCCGcaga
HvGSK2.1	P$DREB1_01	DREB1	500	510	1	1	0.872	gTGCCGcaga
HvGSK2.1	P$JERF1_01	JERF1	500	510	1	1	0.893	gTGCCGcaga
HvGSK2.1	P$CBF1_03	CBF1	500	510	1	1	0.959	gTGCCGcaga
HvGSK2.1	P$DREB1F_01	DREB1F	500	510	1	1	0.876	gTGCCGcaga
HvGSK2.1	P$AT1G33760_01	AT1G33760	500	510	1	1	0.908	gTGCCGcaga
HvGSK2.1	P$AT1G71520_01	AT1G71520	500	510	1	1	0.903	gTGCCGcaga
HvGSK2.1	P$DREB1E_02	DREB1E	500	510	1	1	0.865	gTGCCGcaga
HvGSK2.1	P$ORA47_01	ORA47	500	510	1	1	0.889	gTGCCGcaga
HvGSK2.1	P$ABI4_03	ABI4	500	510	1	1	0.886	gtGCCGCaga
HvGSK2.1	P$DREBI5_01	DREBI5	500	510	1	1	0.932	gtGCCGCaga
HvGSK2.1	P$DREB2B_01	DREB2B	500	510	1	1	0.863	gtGCCGCaga
HvGSK2.1	P$AT1G77200_01	AT1G77200	500	510	1	1	0.867	gtGCCGCaga
HvGSK2.1	P$CBF3_01	CBF3	500	510	1	1	0.898	gtGCCGCaga
HvGSK2.1	P$DREBII1_01	DREBII1	500	510	1	1	0.929	gtGCCGCaga
HvGSK2.1	P$DBF2_01	DBF2	500	510	1	1	0.857	gtGCCGCaga
HvGSK2.1	P$CBF5_01	CBF5	500	510	1	1	0.929	gtGCCGCaga
HvGSK2.1	P$CBF16_01	CBF16	500	510	1	1	0.929	gtGCCGCaga
HvGSK2.1	P$CBF17_01	CBF17	500	510	1	1	0.93	gtGCCGCaga

HvGSK2.1	P$CBF_01	CBF	500	510	1	1	0.932	gtGCCGCaga
HvGSK2.1	P$ERF016_01	ERF016	500	510	1	1	0.906	gtGCCGCaga
HvGSK2.1	P$TINY2_02	TINY2	500	510	1	1	0.861	gtGCCGCaga
HvGSK2.1	P$AT3G16280_01	AT3G16280	500	510	1	1	0.85	gtGCCGCaga
HvGSK2.1	P$DREB1A_03	DREB1A	500	510	1	1	0.898	gtGCCGCaga
HvGSK2.1	P$DREB2D_01	DREB2D	500	510	1	1	0.851	gtGCCGCaga
HvGSK2.1	P$CRF2_01	CRF2	500	508	1	1	0.884	gtGCCGCa
HvGSK2.1	P$ERF098_01	ERF098	500	508	1	1	0.897	gtGCCGCa
HvGSK2.1	P$ERF7_02	ERF7	501	511	1	1	0.946	tGCCGCagag
HvGSK2.1	P$O2_03	O2	529	539	1	1	0.93	GATGAattgg
HvGSK2.1	P$AT5G54070_01	AT5G54070	538	544	1	1	0.915	gCAACG
HvGSK2.1	P$GT1_Q6	GT1	568	575	1	1	0.912	GTGAAca
HvGSK2.1	P$ABI3_01	ABI3	596	605	1	1	0.876	cgGCATGtg
HvGSK2.1	P$AMS_01	AMS	597	607	1	1	0.971	ggCATGTggc
HvGSK2.1	P$E2L_Q2	E2L	603	610	1	1	1	tGGCGGg
HvGSK2.1	P$ERF1_Q2	ERF1	604	612	1	1	0.887	GGCGGgtt
HvGSK2.1	P$LEC2_01	LEC2	625	636	1	1	0.934	acCATGCtgca
HvGSK2.1	P$RAV1_01	RAV1	631	643	1	1	0.965	ctgCAACAgtcg
HvGSK2.1	P$DREB1A_04	DREB1A	637	647	1	1	0.929	caGTCGGgga
HvGSK2.1	P$ERF039_01	ERF039	637	647	1	1	0.946	caGTCGGgga
HvGSK2.1	P$PHYPA182268_05	HYPA182268	637	647	1	1	0.87	caGTCGGgga
HvGSK2.1	P$PHYPA173530_04	HYPA173530	638	646	1	1	0.862	aGTCGGgg
HvGSK2.1	P$PHYPA28324_10	HYPA28324	638	646	1	1	0.897	aGTCGGgg
HvGSK2.1	P$HSF3_01	HSF3	640	646	1	1	0.94	tCGGGG
HvGSK2.1	P$AT1G53910_02	AT1G53910	658	679	1	1	0.884	cgctatgagtgGCGGCtgaca
HvGSK2.1	P$E2L_Q2	E2L	667	674	1	1	0.91	tGGCGGc
HvGSK2.1	P$ERF1_Q2	ERF1	668	676	1	1	0.994	GGCGGctg
HvGSK2.1	P$KNOX3_01	KNOX3	670	682	1	1	0.979	cggcTGACActa
HvGSK2.1	P$ATH1_01	ATH1	674	682	1	1	0.913	TGACActa
HvGSK2.1	P$P_01		677	686	1	1	0.871	caCTACCgt
HvGSK2.1	P$MYB89_01	MYB89	678	689	1	1	0.886	acTACCGtgag
HvGSK2.1	P$E2L_Q2	E2L	689	696	1	1	0.957	aGGCGGg
HvGSK2.1	P$ERF1_Q2	ERF1	690	698	1	1	0.887	GGCGGgtg
HvGSK2.1	P$CBF3_02	CBF3	710	724	1	1	0.925	ggagcCCGACgttt
HvGSK2.1	P$CBF1_04	CBF1	711	723	1	1	0.914	gagcCCGACgtt
HvGSK2.1	P$ARF5_01	ARF5	714	722	1	1	0.886	cCCGACgt
HvGSK2.1	P$DREB1B_01	DREB1B	715	720	1	1	1	CCGAC
HvGSK2.1	P$ARR1_01	ARR1	729	739	1	1	0.958	gacGAATCcc
HvGSK2.1	P$AT3G60580_01	AT3G60580	732	739	1	1	0.932	gaATCCC
HvGSK2.1	P$TGA2_Q2	TGA2	752	762	1	1	0.87	cCGTCAtagt
HvGSK2.1	P$SPF1_Q2	SPF1	755	765	1	1	0.932	tcATAGTgtt
HvGSK2.1	P$LEC2_01	LEC2	767	778	1	1	0.978	taCATGCatgg
HvGSK2.1	P$FUS3_Q2	FUS3	768	779	1	1	0.96	aCATGCatggg
HvGSK2.1	P$ABI3_01	ABI3	770	779	1	1	0.864	atGCATGgg
HvGSK2.1	P$TGA1A_01	TGA1A	794	801	1	1	0.871	aACGTCt
HvGSK2.1	P$AT5G26170_01	AT5G26170	802	811	1	1	0.867	ccTCAACtt
HvGSK2.1	P$EDF3_02	EDF3	811	820	1	1	0.904	cGACCGtgg
HvGSK2.1	P$BZIP68_01	BZIP68	812	821	1	1	0.944	gacCGTGGc
HvGSK2.1	P$OSRR22_01	OSRR22	840	850	1	1	0.855	gcGATACgta
HvGSK2.1	P$MYB3_01	MYB3	845	856	1	1	0.901	acgTAGGTcga
HvGSK2.1	P$MYB4_01	MYB4	846	854	1	1	0.917	cgTAGGTc
HvGSK2.1	P$MYB3R5_01	MYB3R5	860	875	1	1	0.895	ttgtgcaaaCCGTTa
HvGSK2.1	P$MYB3R1_01	MYB3R1	861	876	1	1	0.927	tgtgcaaaCCGTTag
HvGSK2.1	P$MYB3R4_01	MYB3R4	861	876	1	1	0.914	tgtgcaaaCCGTTag
HvGSK2.1	P$WEREWOLF_Q2	WEREWOLF	869	878	1	1	0.925	ccGTTAGtg
HvGSK2.1	P$ABI3_01	ABI3	875	884	1	1	0.875	gtGCATGga
HvGSK2.1	P$ABF2_01	ABF2	897	910	1	1	0.893	tgcgtCACGTtgt
HvGSK2.1	P$GBP_Q6	GBP	899	911	1	1	0.905	cgtCACGTtgtg
HvGSK2.1	P$ABI5_01	ABI5	899	909	1	1	0.921	cgtCACGTtg
HvGSK2.1	P$ABF4_01	ABF4	899	911	1	1	0.886	cgtCACGTtgtg
HvGSK2.1	P$EMBP1_Q2	EMBP1	900	910	1	1	0.896	gtCACGTtgt
HvGSK2.1	P$CPRF3_Q2	CPRF3	900	910	1	1	0.928	gtCACGTtgt
HvGSK2.1	P$CPRF2_Q2	CPRF2	900	910	1	1	0.944	gtCACGTtgt
HvGSK2.1	P$O2_02	O2	900	910	1	1	0.952	gtCACGTtgt
HvGSK2.1	P$TGA1B_Q2	TGA1B	900	910	1	1	0.917	gtCACGTtgt
HvGSK2.1	P$TGA1A_Q2	TGA1A	900	910	1	1	0.965	gtCACGTtgt
HvGSK2.1	P$CPRF3_01	CPRF3	900	910	1	1	0.932	gtCACGTtgt
HvGSK2.1	P$CPRF2_01	CPRF2	900	910	1	1	0.944	gtCACGTtgt
HvGSK2.1	P$TGA1B_01	TGA1B	900	910	1	1	0.907	gtCACGTtgt
HvGSK2.1	P$BEE2_01	BEE2	900	910	1	1	0.92	gtCACGTtgt
HvGSK2.1	P$BIM2_01	BIM2	900	910	1	1	0.854	gtCACGTtgt
HvGSK2.1	P$BIM3_01	BIM3	900	910	1	1	0.885	gtCACGTtgt
HvGSK2.1	P$PHYPA143875_02	HYPA143875	900	910	1	1	0.885	gtCACGTtgt
HvGSK2.1	P$SPT_01	SPT	900	909	1	1	0.918	gtCACGTtg
HvGSK2.1	P$GBF1F_Q2	GBF1F	900	911	1	1	0.88	gtCACGTtgtg
HvGSK2.1	P$RITA1_01	RITA1	901	908	1	1	0.969	tCACGTt
HvGSK2.1	P$OCSBF1_01	OCSBF1	902	907	1	1	1	CACGT
HvGSK2.1	P$ABI3_01	ABI3	917	926	1	1	0.85	aaGCATGag

HvGSK2.1	P$ANTL_02	ANTL	938	948	1	1	0.898	tttCGACAca
HvGSK2.1	P$TGA1_01	TGA1	953	964	1	1	0.915	attTGACGgtg
HvGSK2.1	P$WRKY11_Q2	WRKY11	954	962	1	1	0.899	tTTGACgg
HvGSK2.1	P$TGA5_01	TGA5	955	963	1	1	0.855	tTGACGgt
HvGSK2.1	P$RAP21_02	RAP21	955	968	1	1	0.947	ttgaCGGTGatgg
HvGSK2.1	P$GAMYB_01	GAMYB	969	977	1	1	0.948	CAACCgca
HvGSK2.1	P$AT3G01030_01	AT3G01030	971	980	1	1	0.913	ACCGCaagt
HvGSK2.1	P$AT5G54070_01	AT5G54070	1001	1007	1	1	0.91	tCAACG
HvGSK2.1	P$AT2G33710_01	AT2G33710	1018	1033	1	1	0.872	tcgacccCGCCGagg
HvGSK2.1	P$ERF112_02	ERF112	1023	1033	1	1	0.919	ccCGCCGagg
HvGSK2.1	P$AT3G63350_01	AT3G63350	1024	1030	1	1	0.866	CCGCCg
HvGSK2.1	P$CRF4_01	CRF4	1024	1032	1	1	0.863	cCGCCGag
HvGSK2.1	P$ERF4_04	ERF4	1024	1032	1	1	0.864	cCGCCGag
HvGSK2.1	P$ERF069_01	ERF069	1024	1033	1	1	0.987	cCGCCGagg
HvGSK2.1	P$ERF11_01	ERF11	1024	1034	1	1	0.959	cCGCCGaggg
HvGSK2.1	P$ERF8_01	ERF8	1025	1035	1	1	0.934	CGCCGagggt
HvGSK2.1	P$GATA15_01	GATA15	1047	1056	1	1	1	taTGATCga
HvGSK2.1	P$STZ_01	STZ	1067	1079	1	1	0.944	tgcTAGTAaatg
HvGSK2.1	P$PDF2_01	DF2	1069	1080	1	1	0.884	ctagTAAATgg
HvGSK2.1	P$LEC2_01	LEC2	1086	1097	1	1	0.933	gcCATGCtagc
HvGSK2.1	P$HSFA2_01	HSFA2	1101	1107	1	1	0.933	CCAAAg
HvGSK2.1	P$ARR1_01	ARR1	1114	1124	1	1	0.978	gttGAATCta
HvGSK2.1	P$FAR1_01	FAR1	1125	1140	1	1	0.975	gtgtcACGCGcatct
HvGSK2.1	P$FHY3_01	FHY3	1127	1139	1	1	0.96	gtcACGCGcatc
HvGSK2.1	P$TRAB1_Q2	TRAB1	1128	1139	1	1	0.861	tcACGCGcatc
HvGSK2.1	P$PEND_01	END	1146	1154	1	1	0.92	tAAGAAgc
HvGSK2.1	P$ATMYB77_01	ATMYB77	1159	1172	1	1	0.947	catcgaCGGTTcc
HvGSK2.1	P$ASR1_01	ASR1	1181	1186	1	1	1	ACCCA
HvGSK2.1	P$HSFA2_01	HSFA2	1213	1219	1	1	1	CCAAAa
HvGSK2.1	P$HDG9_01	HDG9	1216	1230	1	1	0.879	aaatTAAATgctgc
HvGSK2.1	P$PDF2_01	DF2	1216	1227	1	1	0.93	aaatTAAATgc
HvGSK2.1	P$MYBAS1_01	MYBAS1	1227	1238	1	1	0.974	tgCCAACaaat
HvGSK2.1	P$RAV1_01	RAV1	1227	1239	1	1	0.958	tgcCAACAaatg
HvGSK2.1	P$ERF112_02	ERF112	1272	1282	1	1	0.924	ttCGCCGgag
HvGSK2.1	P$CRF4_01	CRF4	1273	1281	1	1	0.854	tCGCCGga
HvGSK2.1	P$ERF4_04	ERF4	1273	1281	1	1	0.865	tCGCCGga
HvGSK2.1	P$ERF069_01	ERF069	1273	1282	1	1	0.989	tCGCCGgag
HvGSK2.1	P$ERF11_01	ERF11	1273	1283	1	1	0.97	tCGCCGgagt
HvGSK2.1	P$ERF8_01	ERF8	1274	1284	1	1	0.947	CGCCGgagtt
HvGSK2.1	P$SPL11_01	SPL11	1285	1297	1	1	0.881	tttgGTACGcct
HvGSK2.1	P$SPL5_01	SPL5	1287	1296	1	1	0.965	tgGTACGcc
HvGSK2.1	P$POPTR_01	OPTR	1288	1295	1	1	0.929	gGTACGc
HvGSK2.1	P$SPL12_01	SPL12	1288	1296	1	1	0.97	gGTACGcc
HvGSK2.1	P$SPL4_01	SPL4	1288	1297	1	1	0.988	gGTACGcct
HvGSK2.1	P$AT1G66560_01	AT1G66560	1293	1303	1	1	0.9	gccTTAACcg
HvGSK2.1	P$WRKY21_01	WRKY21	1294	1303	1	1	0.911	ccTTAACcg
HvGSK2.1	P$AT1G18860_01	AT1G18860	1294	1303	1	1	0.9	ccTTAACcg
HvGSK2.1	P$AT1G64000_01	AT1G64000	1294	1303	1	1	0.925	ccTTAACcg
HvGSK2.1	P$WRKY6_01	WRKY6	1294	1303	1	1	0.865	ccTTAACcg
HvGSK2.1	P$AT1G66600_01	AT1G66600	1294	1303	1	1	0.901	ccTTAACcg
HvGSK2.1	P$AT1G68150_01	AT1G68150	1294	1303	1	1	0.888	ccTTAACcg
HvGSK2.1	P$AT5G41570_01	AT5G41570	1294	1303	1	1	0.925	ccTTAACcg
HvGSK2.1	P$AT5G15130_01	AT5G15130	1294	1303	1	1	0.895	ccTTAACcg
HvGSK2.1	P$WRKY46_01	WRKY46	1294	1303	1	1	0.879	ccTTAACcg
HvGSK2.1	P$AT2G24570_01	AT2G24570	1294	1303	1	1	0.908	ccTTAACcg
HvGSK2.1	P$WRKY7_01	WRKY7	1294	1303	1	1	0.916	ccTTAACcg
HvGSK2.1	P$WRKY25_01	WRKY25	1294	1303	1	1	0.939	cctTAACCg
HvGSK2.1	P$WRKY33_01	WRKY33	1294	1303	1	1	0.874	cctTAACCg
HvGSK2.1	P$AT1G29860_01	AT1G29860	1294	1303	1	1	0.903	cctTAACCg
HvGSK2.1	P$AT3G62340_01	AT3G62340	1294	1303	1	1	0.883	cctTAACCg
HvGSK2.1	P$AT1G69310_01	AT1G69310	1294	1303	1	1	0.913	cctTAACCg
HvGSK2.1	P$WRKY26_01	WRKY26	1294	1303	1	1	0.877	cctTAACCg
HvGSK2.1	P$GT1_01	GT1	1295	1303	1	1	0.946	ctTAACCg
HvGSK2.1	P$AT3G01030_01	AT3G01030	1299	1308	1	1	0.857	ACCGCgaat
HvGSK2.1	P$ATHB6_01	ATHB6	1303	1312	1	1	0.902	cgAATAAag
HvGSK2.1	P$DOF1_01	DOF1	1304	1315	1	1	0.976	gaaTAAAGatt
HvGSK2.1	P$SBF1_01	SBF1	1307	1321	1	1	0.879	taaagaTTAATtac
HvGSK2.1	P$EDT1_01	EDT1	1310	1320	1	1	0.861	agaTTAATta
HvGSK2.1	P$FBP24_01	FBP24	1360	1378	1	1	0.871	gaatTAAACgtttacttg
HvGSK2.1	P$FBP28_01	FBP28	1360	1378	1	1	0.9	gaattaAACGTttacttg
HvGSK2.1	P$AT3G20750_01	AT3G20750	1363	1371	1	1	0.912	tTAAACgt
HvGSK2.1	P$TGA1_01	TGA1	1373	1384	1	1	0.928	actTGACGgca
HvGSK2.1	P$WRKY11_Q2	WRKY11	1374	1382	1	1	0.897	cTTGACgg
HvGSK2.1	P$TGA7_01	TGA7	1374	1384	1	1	0.89	ctTGACGgca
HvGSK2.1	P$TGA5_01	TGA5	1375	1383	1	1	0.856	tTGACGgc
HvGSK2.1	P$ABI3_01	ABI3	1379	1388	1	1	0.89	cgGCATGaa
HvGSK2.1	P$C1_Q2	C1	1384	1395	1	1	0.963	tgAACTAcacg
HvGSK2.1	P$PIL5_01	IL5	1384	1398	1	1	0.976	tgaactacACGTGg

HvGSK2.1	P$PIF3_01	IF3	1385	1403	1	1	0.879	gaactaCACGTggttgat
HvGSK2.1	P$PIF3_02	IF3	1386	1403	1	1	0.9	aactaCACGTggttgat
HvGSK2.1	P$ABF2_01	ABF2	1386	1399	1	1	0.996	aactaCACGTggt
HvGSK2.1	P$ATHSFA1D_01	ATHSFA1D	1387	1393	1	1	1	aCTACA
HvGSK2.1	P$BZR1_02	BZR1	1387	1401	1	1	0.96	actaCACGTggttg
HvGSK2.1	P$HBI1_01	HBI1	1387	1399	1	1	0.935	actaCACGTggt
HvGSK2.1	P$ABZ1_01	ABZ1	1387	1401	1	1	0.932	actacACGTGgttg
HvGSK2.1	P$GBP_Q6	GBP	1388	1400	1	1	0.906	ctaCACGTggtt
HvGSK2.1	P$PIF3_03	IF3	1388	1398	1	1	0.928	ctaCACGTgg
HvGSK2.1	P$ABI5_01	ABI5	1388	1398	1	1	0.974	ctaCACGTgg
HvGSK2.1	P$ABF4_01	ABF4	1388	1400	1	1	0.997	ctaCACGTggtt
HvGSK2.1	P$GBF1_Q2_01	GBF1	1388	1399	1	1	0.882	ctacACGTGgt
HvGSK2.1	P$BZR1_03	BZR1	1388	1408	1	1	0.886	ctacACGTGgttgatgatac
HvGSK2.1	P$EMBP1_Q2	EMBP1	1389	1399	1	1	0.907	taCACGTggt
HvGSK2.1	P$CPRF_Q2	CPRF	1389	1399	1	1	0.965	taCACGTggt
HvGSK2.1	P$CPRF3_Q2	CPRF3	1389	1399	1	1	0.982	taCACGTggt
HvGSK2.1	P$CPRF2_Q2	CPRF2	1389	1399	1	1	0.995	taCACGTggt
HvGSK2.1	P$O2_02	O2	1389	1399	1	1	0.97	taCACGTggt
HvGSK2.1	P$TGA1B_Q2	TGA1B	1389	1399	1	1	0.903	taCACGTggt
HvGSK2.1	P$TGA1A_Q2	TGA1A	1389	1399	1	1	0.976	taCACGTggt
HvGSK2.1	P$CPRF1_01	CPRF1	1389	1399	1	1	0.971	taCACGTggt
HvGSK2.1	P$CPRF3_01	CPRF3	1389	1399	1	1	0.984	taCACGTggt
HvGSK2.1	P$CPRF2_01	CPRF2	1389	1399	1	1	0.997	taCACGTggt
HvGSK2.1	P$TGA1B_01	TGA1B	1389	1399	1	1	0.89	taCACGTggt
HvGSK2.1	P$BES1_01	BES1	1389	1400	1	1	0.965	taCACGTggtt
HvGSK2.1	P$PIF3_04	IF3	1389	1399	1	1	0.878	taCACGTggt
HvGSK2.1	P$BEE2_01	BEE2	1389	1399	1	1	0.999	taCACGTggt
HvGSK2.1	P$BIM2_01	BIM2	1389	1399	1	1	0.994	taCACGTggt
HvGSK2.1	P$BIM3_01	BIM3	1389	1399	1	1	0.991	taCACGTggt
HvGSK2.1	P$PHYPA143875_02	HYPA143875	1389	1399	1	1	0.997	taCACGTggt
HvGSK2.1	P$PHYPA72483_07	HYPA72483	1389	1399	1	1	0.997	taCACGTggt
HvGSK2.1	P$SPT_01	SPT	1389	1398	1	1	0.969	taCACGTgg
HvGSK2.1	P$GBF1F_Q2	GBF1F	1389	1400	1	1	0.868	taCACGTggtt
HvGSK2.1	P$HBP1A_Q2	HBP1A	1389	1399	1	1	0.92	tacACGTGgt
HvGSK2.1	P$TAF1_Q2	TAF1	1389	1399	1	1	0.972	tacACGTGgt
HvGSK2.1	P$EMBP1_02	EMBP1	1389	1399	1	1	0.985	tacACGTGgt
HvGSK2.1	P$TAF1_01	TAF1	1389	1399	1	1	0.983	tacACGTGgt
HvGSK2.1	P$PIF1_01	IF1	1389	1399	1	1	0.985	tacACGTGgt
HvGSK2.1	P$ABF3_01	ABF3	1390	1398	1	1	0.875	ACACGtgg
HvGSK2.1	P$RITA1_01	RITA1	1390	1397	1	1	0.984	aCACGTg
HvGSK2.1	P$BHLH66_01	BHLH66	1390	1398	1	1	0.93	aCACGTgg
HvGSK2.1	P$PIF5_01	IF5	1390	1398	1	1	0.93	aCACGTgg
HvGSK2.1	P$MYC2_01	MYC2	1390	1398	1	1	0.953	aCACGTgg
HvGSK2.1	P$MYC3_01	MYC3	1390	1398	1	1	0.994	aCACGTgg
HvGSK2.1	P$BHLH34_01	BHLH34	1390	1398	1	1	0.972	aCACGTgg
HvGSK2.1	P$PHYPA48267_08	HYPA48267	1390	1398	1	1	0.971	aCACGTgg
HvGSK2.1	P$OJ1058_01	OJ1058	1390	1398	1	1	1	aCACGTgg
HvGSK2.1	P$UNE10_01	UNE10	1390	1398	1	1	0.98	aCACGTgg
HvGSK2.1	P$BHLH3_01	BHLH3	1390	1398	1	1	0.952	aCACGTgg
HvGSK2.1	P$HY5_01	HY5	1390	1400	1	1	0.95	acACGTGgtt
HvGSK2.1	P$GBF1_01	GBF1	1390	1398	1	1	0.973	acACGTGg
HvGSK2.1	P$MYC4_01	MYC4	1390	1398	1	1	0.953	acACGTGg
HvGSK2.1	P$BIM1_02	BIM1	1390	1400	1	1	0.995	acACGTGgtt
HvGSK2.1	P$BHLH13_01	BHLH13	1390	1398	1	1	0.947	acACGTGg
HvGSK2.1	P$ABF4_02	ABF4	1390	1400	1	1	0.979	acACGTGgtt
HvGSK2.1	P$BZIP68_01	BZIP68	1390	1399	1	1	0.976	acaCGTGGt
HvGSK2.1	P$OCSBF1_01	OCSBF1	1391	1396	1	1	1	CACGT
HvGSK2.1	P$PIF4_01	IF4	1391	1399	1	1	0.966	CACGTggt
HvGSK2.1	P$CPRF1_Q2	CPRF1	1391	1401	1	1	0.962	cACGTGgttg
HvGSK2.1	P$ABI5_Q2	ABI5	1392	1398	1	1	1	ACGTGg
HvGSK2.1	P$MYB3R5_01	MYB3R5	1398	1413	1	1	0.892	ttgatgataCCGTTt
HvGSK2.1	P$MYB3R1_01	MYB3R1	1399	1414	1	1	0.917	tgatgataCCGTTtc
HvGSK2.1	P$MYB3R4_01	MYB3R4	1399	1414	1	1	0.898	tgatgataCCGTTtc
HvGSK2.1	P$RAV1_01	RAV1	1410	1422	1	1	0.903	tttCAACAcccg
HvGSK2.1	P$AP2A_01	AP2A	1410	1420	1	1	0.881	tttcaACACC
HvGSK2.1	P$BPC1_Q2	BPC1	1446	1452	1	1	1	AGAAAg
HvGSK2.1	P$BPC1_Q2	BPC1	1450	1456	1	1	0.997	AGAAAa
HvGSK2.1	P$SED_Q2	SED	1453	1463	1	1	0.916	aaaaCCTTTc
HvGSK2.1	P$PBF_Q2_01	BF	1457	1463	1	1	0.985	CCTTTc
HvGSK2.1	P$AT2G15660_01	AT2G15660	1460	1471	1	1	0.909	TTCTCgtaaaa
HvGSK2.1	P$NAC6_01	NAC6	1463	1469	1	1	0.854	tCGTAA
HvGSK2.1	P$ANTL_02	ANTL	1477	1487	1	1	0.897	actCGACAat
HvGSK2.1	P$ATHB6_01	ATHB6	1482	1491	1	1	0.921	acAATAAca
HvGSK2.1	P$ATMYB15_Q2	ATMYB15	1486	1492	1	1	1	TAACAa
HvGSK2.1	P$TGA1_01	TGA1	1489	1500	1	1	0.935	caaTGACGaga
HvGSK2.1	P$TGA7_01	TGA7	1490	1500	1	1	0.887	aaTGACGaga
HvGSK2.1	P$TGA5_01	TGA5	1491	1499	1	1	0.869	aTGACGag
HvGSK2.1	P$HSFA4A_01	HSFA4A	1502	1508	1	1	0.91	gCTATT

HvGSK2.1	P$DREB1A_04	DREB1A	1522	1532	1	1	0.968	taGTCGGcct
HvGSK2.1	P$ERF039_01	ERF039	1522	1532	1	1	0.979	taGTCGGcct
HvGSK2.1	P$PHYPA28324_10	HYPA28324	1523	1531	1	1	0.871	aGTCGGcc
HvGSK2.1	P$AT5G26170_01	AT5G26170	1529	1538	1	1	0.867	ccTCAACtt
HvGSK2.1	P$BHLH112_01	BHLH112	1531	1540	1	1	0.934	tcaACTTGa
HvGSK2.1	P$BPC1_Q2	BPC1	1570	1576	1	1	0.997	AGAAAa
HvGSK2.1	P$HSFA4A_01	HSFA4A	1593	1599	1	1	1	aCTATT
HvGSK2.1	P$AT3G20750_01	AT3G20750	1601	1609	1	1	1	aTAAACat
HvGSK2.1	P$GT1_Q6_01	GT1	1614	1626	1	1	0.923	TTTTTttataaa
HvGSK2.1	P$GT1_Q6_01	GT1	1615	1627	1	1	0.91	TTTTTtataaat
HvGSK2.1	P$PDF2_01	DF2	1618	1629	1	1	0.856	tttaTAAATac
HvGSK2.1	P$E2F_Q2	E2F	1629	1640	1	1	0.892	aaaTTCCCgcg
HvGSK2.1	P$AT3G60580_01	AT3G60580	1640	1647	1	1	0.883	aaATCCC
HvGSK2.1	P$PDF2_01	DF2	1647	1658	1	1	0.867	ggtaTAAATat
HvGSK2.1	P$SBF1_01	SBF1	1657	1671	1	1	0.865	tataaaTTAAAtat
HvGSK2.1	P$E2L1_Q2	E2L1	1668	1680	1	1	0.865	taTTGGCggtaa
HvGSK2.1	P$E2L_Q2	E2L	1671	1678	1	1	0.892	tGGCGGt
HvGSK2.1	P$ABI3_01	ABI3	1679	1688	1	1	0.865	atGCATGgc
HvGSK2.1	P$MYBAS1_01	MYBAS1	1685	1696	1	1	0.952	ggCTAACgtcg
HvGSK2.1	P$TGA1A_01	TGA1A	1689	1696	1	1	0.871	aACGTCg
HvGSK2.1	P$O2_Q2	O2	1696	1709	1	1	0.882	cagtaGACGTagg
HvGSK2.1	P$MYB3_01	MYB3	1702	1713	1	1	0.901	acgTAGGTtta
HvGSK2.1	P$MYB4_01	MYB4	1703	1711	1	1	0.917	cgTAGGTt
HvGSK2.1	P$ARR2_01	ARR2	1708	1718	1	1	0.861	gtttATCTTt
HvGSK2.1	P$AT1G77950_01	AT1G77950	1710	1721	1	1	0.895	ttatcTTTAAt
HvGSK2.1	P$SBF1_01	SBF1	1710	1724	1	1	0.859	ttatctTTAATtcg
HvGSK2.1	P$EDT1_01	EDT1	1713	1723	1	1	0.883	tctTTAATtc
HvGSK2.1	P$AT3G60580_01	AT3G60580	1725	1732	1	1	0.873	ccATCCC
HvGSK2.1	P$WRKY11_Q2	WRKY11	1734	1742	1	1	0.976	cTTGACct
HvGSK2.1	P$ZAP1_01	ZAP1	1735	1745	1	1	0.883	TTGACcttcc
HvGSK2.1	P$MYBAS1_01	MYBAS1	1753	1764	1	1	0.997	cgCTAACcgct
HvGSK2.1	P$GT1_01	GT1	1754	1762	1	1	0.85	gcTAACCg
HvGSK2.1	P$ANTL_01	ANTL	1770	1780	1	1	0.973	tGTTACcgct
HvGSK2.1	P$AT4G12750_01	AT4G12750	1786	1796	1	1	0.895	tagACCGAtt
HvGSK2.1	P$RAV2_01	RAV2	1787	1796	1	1	0.859	agACCGAtt
HvGSK2.1	P$AT3G51080_01	AT3G51080	1824	1831	1	1	0.89	GGAAAac
HvGSK2.1	P$GAMYB_Q2	GAMYB	1824	1837	1	1	0.886	ggaaaACAACttg
HvGSK2.1	P$BHLH112_01	BHLH112	1829	1838	1	1	0.934	acaACTTGg
HvGSK2.1	P$BPC1_Q2	BPC1	1845	1851	1	1	0.99	AGAAAt
HvGSK2.1	P$ATHB7_01	ATHB7	1846	1856	1	1	0.874	gaAATCAaga
HvGSK2.1	P$HAT1_01	HAT1	1846	1856	1	1	0.872	gaAATCAaga
HvGSK2.1	P$GATA9_01	GATA9	1850	1861	1	1	0.903	tcaAGATCcac
HvGSK2.1	P$AGP1_01	AGP1	1851	1861	1	1	0.926	caAGATCcac
HvGSK2.1	P$ARR10_01	ARR10	1853	1860	1	1	0.934	AGATCca
HvGSK2.1	P$MYB80_01	MYB80	1871	1882	1	1	0.859	tgGAATAtctt
HvGSK2.1	P$ARR2_01	ARR2	1873	1883	1	1	0.904	gaatATCTTg
HvGSK2.1	P$GATA15_01	GATA15	1879	1888	1	1	0.999	ctTGATCtc
HvGSK2.1	P$GATA11_01	GATA11	1880	1888	1	1	0.854	ttGATCTc
HvGSK2.1	P$GATA8_01	GATA8	1880	1889	1	1	0.979	ttGATCTca
HvGSK2.1	P$RAV1_01	RAV1	1884	1896	1	1	0.944	tctCAACAcatg
HvGSK2.1	P$MYB3_01	MYB3	1895	1906	1	1	0.907	gagTAGGTggt
HvGSK2.1	P$MYB4_01	MYB4	1896	1904	1	1	0.961	agTAGGTg
HvGSK2.1	P$AMS_01	AMS	1970	1980	1	1	0.869	ggCAGGTgga
HvGSK2.1	P$TEIL_01	TEIL	1986	1994	1	1	0.922	ATGTAtat
HvGSK2.1	P$HSFA2_01	HSFA2	2000	2006	1	1	1	CCAAAa
HvGSK2.1	P$MYB3R5_01	MYB3R5	2003	2018	1	1	0.953	aaaatccaaCCGTTa
HvGSK2.1	P$MYB3R1_01	MYB3R1	2004	2019	1	1	0.961	aaatccaaCCGTTac
HvGSK2.1	P$MYB3R4_01	MYB3R4	2004	2019	1	1	0.947	aaatccaaCCGTTac
HvGSK2.1	P$MYB118_01	MYB118	2005	2022	1	1	0.981	aatccaaccGTTACaac
HvGSK2.1	P$MYBAS1_01	MYBAS1	2006	2017	1	1	0.985	atCCAACcgtt
HvGSK2.1	P$GAMYB_01	GAMYB	2009	2017	1	1	0.916	CAACCgtt
HvGSK2.1	P$GAMYB_Q2	GAMYB	2012	2025	1	1	0.881	ccgttACAACatt
HvGSK2.1	P$RAV1_01	RAV1	2015	2027	1	1	0.943	ttaCAACAttat
HvGSK2.1	P$ATHB1_01	ATHB1	2017	2031	1	1	0.871	acaacATTATtgtc
HvGSK2.1	P$ATHB5_01	ATHB5	2020	2029	1	1	0.862	acaTTATTg
HvGSK2.1	P$MYBAS1_01	MYBAS1	2028	2039	1	1	0.941	gtCCAACtcgg
HvGSK2.1	P$WRKY18_02	WRKY18	2035	2045	1	1	0.997	tcgGTCAAac
HvGSK2.1	P$WRKY21_02	WRKY21	2035	2045	1	1	0.969	tcgGTCAAac
HvGSK2.1	P$WRKY48_02	WRKY48	2035	2045	1	1	0.998	tcgGTCAAac
HvGSK2.1	P$WRKY57_01	WRKY57	2035	2045	1	1	0.971	tcgGTCAAac
HvGSK2.1	P$WRKY60_01	WRKY60	2035	2046	1	1	0.98	tcgGTCAAacc
HvGSK2.1	P$WRKY15_01	WRKY15	2036	2046	1	1	0.985	cgGTCAAacc
HvGSK2.1	P$WRKY2_01	WRKY2	2036	2044	1	1	0.991	cgGTCAAa
HvGSK2.1	P$WRKY25_02	WRKY25	2036	2044	1	1	0.979	cgGTCAAa
HvGSK2.1	P$WRKY40_01	WRKY40	2036	2044	1	1	1	cgGTCAAa
HvGSK2.1	P$WRKY43_02	WRKY43	2036	2046	1	1	0.976	cgGTCAAacc
HvGSK2.1	P$WRKY62_01	WRKY62	2036	2044	1	1	0.91	cgGTCAAa
HvGSK2.1	P$WRKY63_01	WRKY63	2036	2044	1	1	0.991	cgGTCAAa

HvGSK2.1	P$WRKY75_01	WRKY75	2036	2044	1	1	0.975	cgGTCAAa
HvGSK2.1	P$WRKY8_01	WRKY8	2036	2045	1	1	0.992	cgGTCAAac
HvGSK2.1	P$WRKY23_01	WRKY23	2037	2045	1	1	0.854	gGTCAAac
HvGSK2.1	P$WRKY30_01	WRKY30	2037	2047	1	1	0.918	gGTCAAaccg
HvGSK2.1	P$WRKY18_Q2	WRKY18	2038	2047	1	1	0.935	GTCAAaccg
HvGSK2.1	P$AT4G12750_01	AT4G12750	2040	2050	1	1	0.911	caaACCGAag
HvGSK2.1	P$RAV2_01	RAV2	2041	2050	1	1	0.86	aaACCGAag
HvGSK2.1	P$AT2G41690_01	AT2G41690	2044	2050	1	1	0.988	CCGAAg
HvGSK2.1	P$SEP3_01	wrz-03	2087	2098	1	1	0.887	gcaacTTTTGg
HvGSK2.1	P$E2L1_Q2	E2L1	2092	2104	1	1	0.914	ttTTGGCgtttc
HvGSK2.1	P$AT4G12750_01	AT4G12750	2107	2117	1	1	0.874	gagACCGAta
HvGSK2.1	P$RAV2_01	RAV2	2108	2117	1	1	0.866	agACCGAta
HvGSK2.1	P$ARR2_01	ARR2	2118	2128	1	1	0.893	ataaATCTTg
HvGSK2.1	P$WRKY11_01	WRKY11	2139	2153	1	1	0.854	ttggTTGACctagg
HvGSK2.1	P$WRKY11_Q2	WRKY11	2142	2150	1	1	0.979	gTTGACct
HvGSK2.1	P$ZAP1_01	ZAP1	2143	2153	1	1	0.932	TTGACctagg
HvGSK2.1	P$HSFA2_01	HSFA2	2160	2166	1	1	0.941	CCAAAc
HvGSK2.1	P$AT5G04240_01	AT5G04240	2170	2176	1	1	0.938	tGGCAC
HvGSK2.1	P$GT1_Q6	GT1	2182	2189	1	1	0.971	GTAAAaa
HvGSK2.1	P$ATHB6_01	ATHB6	2185	2194	1	1	0.905	aaAATAAaa
HvGSK2.1	P$WEREWOLF_Q2	WEREWOLF	2228	2237	1	1	0.895	tgGTTAGtg
HvGSK2.1	P$SEP3_01	wrz-03	2234	2245	1	1	0.868	gtgttTTTTGg
HvGSK2.1	P$ATHB7_01	ATHB7	2252	2262	1	1	0.873	aaAATCAaac
HvGSK2.1	P$HAT1_01	HAT1	2252	2262	1	1	0.864	aaAATCAaac
HvGSK2.1	P$AT5G04240_01	AT5G04240	2265	2271	1	1	0.938	tGGCAC
HvGSK2.1	P$AT1G53910_01	AT1G53910	2319	2329	1	1	0.851	gGGCCGattt
HvGSK2.1	P$AT2G20350_01	AT2G20350	2319	2329	1	1	0.928	ggGCCGAttt
HvGSK2.1	P$AT5G04240_01	AT5G04240	2340	2346	1	1	0.938	tGGCAC
HvGSK2.1	P$AT2G41690_01	AT2G41690	2345	2351	1	1	0.974	CCGAAt
HvGSK2.1	P$MYB24_01	MYB24	2355	2364	1	1	1	gagTTAGGt
HvGSK2.1	P$MYB131_01	MYB131	2355	2366	1	1	0.95	gagTTAGGttt
HvGSK2.1	P$MYB3_01	MYB3	2356	2367	1	1	0.918	agtTAGGTttt
HvGSK2.1	P$MYB4_01	MYB4	2357	2365	1	1	0.901	gtTAGGTt
HvGSK2.1	P$AT1G53910_02	AT1G53910	2396	2417	1	1	0.852	gagagtttttgGCGGCtaact
HvGSK2.1	P$E2L1_Q2	E2L1	2402	2414	1	1	0.993	ttTTGGCggcta
HvGSK2.1	P$E2L_Q2	E2L	2405	2412	1	1	0.91	tGGCGGc
HvGSK2.1	P$ERF1_Q2	ERF1	2406	2414	1	1	0.983	GGCGGcta
HvGSK2.1	P$MYBAS1_01	MYBAS1	2409	2420	1	1	0.953	ggCTAACtcaa
HvGSK2.1	P$DOF2_01	DOF2	2414	2425	1	1	0.985	actcAAAGCac
HvGSK2.1	P$DOF3_01	DOF3	2414	2425	1	1	0.983	actcAAAGCac
HvGSK2.1	P$ARR18_01	ARR18	2441	2454	1	1	0.903	tcatAGATAtctc
HvGSK2.1	P$SED_Q2	SED	2452	2462	1	1	0.953	tcatCCTTTt
HvGSK2.1	P$PBF_Q2_01	BF	2456	2462	1	1	1	CCTTTt
HvGSK2.1	P$SQUA_01	SQUA	2456	2466	1	1	0.895	cctTTTTTaa
HvGSK2.1	P$SBF1_01	SBF1	2456	2470	1	1	0.85	ccttttTTAATagt
HvGSK2.1	P$GT1_Q6_01	GT1	2458	2470	1	1	0.885	TTTTTtaatagt
HvGSK2.1	P$SPF1_Q2	SPF1	2463	2473	1	1	0.987	taATAGTatt
HvGSK2.1	P$RIN_Q2	RIN	2555	2566	1	1	0.873	tgtaTTTAAgg
HvGSK2.1	P$GATA8_01	GATA8	2564	2573	1	1	0.979	ggGATCTcc
HvGSK2.1	P$AMS_01	AMS	2587	2597	1	1	0.99	agCATGTggc
HvGSK2.1	P$E2L_Q2	E2L	2593	2600	1	1	1	tGGCGGg
HvGSK2.1	P$ERF1_Q2	ERF1	2594	2602	1	1	0.887	GGCGGgtt
HvGSK2.1	P$WRKY11_Q2	WRKY11	2611	2619	1	1	1	gTTGACca
HvGSK2.1	P$ZAP1_01	ZAP1	2612	2622	1	1	0.92	TTGACcatgc
HvGSK2.1	P$LEC2_01	LEC2	2615	2626	1	1	0.934	acCATGCtgca
HvGSK2.1	P$RRTF1_05	RRTF1	2624	2639	1	1	0.872	caatagtCGGCGatg
HvGSK2.1	P$DREB1A_04	DREB1A	2627	2637	1	1	0.969	taGTCGGcga
HvGSK2.1	P$ERF039_01	ERF039	2627	2637	1	1	0.981	taGTCGGcga
HvGSK2.1	P$PHYPA182268_05	HYPA182268	2627	2637	1	1	0.881	taGTCGGcga
HvGSK2.1	P$PHYPA173530_04	HYPA173530	2628	2636	1	1	0.88	aGTCGGcg
HvGSK2.1	P$PHYPA28324_10	HYPA28324	2628	2636	1	1	0.92	aGTCGGcg
HvGSK2.1	P$AT1G68550_03	AT1G68550	2628	2637	1	1	0.953	agtCGGCGa
HvGSK2.1	P$CBF3_02	CBF3	2658	2672	1	1	0.976	ggccaCCGACacta
HvGSK2.1	P$CBF1_04	CBF1	2659	2671	1	1	0.97	gccaCCGACact
HvGSK2.1	P$DREB1G_02	DREB1G	2660	2670	1	1	0.916	ccaCCGACac
HvGSK2.1	P$AT1G77200_03	AT1G77200	2660	2674	1	1	0.998	ccaCCGACactacc
HvGSK2.1	P$ARF1_01	ARF1	2662	2670	1	1	1	aCCGACac
HvGSK2.1	P$ARF5_01	ARF5	2662	2670	1	1	0.985	aCCGACac
HvGSK2.1	P$DREB1B_01	DREB1B	2663	2668	1	1	1	CCGAC
HvGSK2.1	P$P_01		2667	2676	1	1	0.871	caCTACCgt
HvGSK2.1	P$MYB89_01	MYB89	2668	2679	1	1	0.886	acTACCGtgag
HvGSK2.1	P$AMS_01	AMS	2680	2690	1	1	0.882	ggCATGTgcg
HvGSK2.1	P$ARR1_01	ARR1	2715	2725	1	1	0.958	gacGAATCcc
HvGSK2.1	P$AT3G60580_01	AT3G60580	2718	2725	1	1	0.932	gaATCCC
HvGSK2.1	P$E2F_Q2	E2F	2725	2736	1	1	0.87	gagTTCCCgct
HvGSK2.1	P$MYB3R5_01	MYB3R5	2729	2744	1	1	0.882	tcccgctagCCGTTa
HvGSK2.1	P$MYB3R1_01	MYB3R1	2730	2745	1	1	0.912	cccgctagCCGTTat
HvGSK2.1	P$MYB3R4_01	MYB3R4	2730	2745	1	1	0.924	cccgctagCCGTTat

HvGSK2.1	P$SPF1_Q2	SPF1	2741	2751	1	1	0.894	ttATAGTgtt
HvGSK2.1	P$LEC2_01	LEC2	2753	2764	1	1	0.955	taCATGCgtgg
HvGSK2.1	P$ALFIN1_Q2	ALFIN1	2754	2769	1	1	0.9	acatgcGTGGGtgcg
HvGSK2.1	P$BZIP68_01	BZIP68	2756	2765	1	1	0.927	atgCGTGGg
HvGSK2.1	P$HSFA4A_01	HSFA4A	2768	2774	1	1	0.91	gCTATT
HvGSK2.1	P$ICE1_01	ICE1	2792	2805	1	1	0.856	gaCCGCGactata
HvGSK2.1	P$O2_03	O2	2820	2830	1	1	0.945	GATGAaatga
HvGSK2.1	P$OSRR22_01	OSRR22	2826	2836	1	1	0.867	atGATACgta
HvGSK2.1	P$MYB3_01	MYB3	2831	2842	1	1	0.901	acgTAGGTcga
HvGSK2.1	P$MYB4_01	MYB4	2832	2840	1	1	0.917	cgTAGGTc
HvGSK2.1	P$UIF1_01	UIF1	2839	2849	1	1	0.979	cgaGATTCtg
HvGSK2.1	P$PIL5_01	IL5	2847	2861	1	1	0.854	tgtgaaaaACGTGg
HvGSK2.1	P$GT1_Q6	GT1	2848	2855	1	1	0.971	GTGAAaa
HvGSK2.1	P$ABZ1_01	ABZ1	2850	2864	1	1	0.943	gaaaaACGTGggcg
HvGSK2.1	P$GBF1_Q2_01	GBF1	2851	2862	1	1	0.87	aaaaACGTGgg
HvGSK2.1	P$ALFIN1_Q2	ALFIN1	2851	2866	1	1	0.888	aaaaacGTGGGcgca
HvGSK2.1	P$HBP1A_Q2	HBP1A	2852	2862	1	1	0.874	aaaACGTGgg
HvGSK2.1	P$TAF1_Q2	TAF1	2852	2862	1	1	0.934	aaaACGTGgg
HvGSK2.1	P$EMBP1_02	EMBP1	2852	2862	1	1	0.942	aaaACGTGgg
HvGSK2.1	P$TAF1_01	TAF1	2852	2862	1	1	0.958	aaaACGTGgg
HvGSK2.1	P$HY5_01	HY5	2853	2863	1	1	0.942	aaACGTGggc
HvGSK2.1	P$GBF1_01	GBF1	2853	2861	1	1	0.957	aaACGTGg
HvGSK2.1	P$BIM1_02	BIM1	2853	2863	1	1	0.946	aaACGTGggc
HvGSK2.1	P$ABF4_02	ABF4	2853	2863	1	1	0.973	aaACGTGggc
HvGSK2.1	P$BZIP68_01	BZIP68	2853	2862	1	1	0.977	aaaCGTGGg
HvGSK2.1	P$CPRF1_Q2	CPRF1	2854	2864	1	1	0.925	aACGTGggcg
HvGSK2.1	P$ABI5_Q2	ABI5	2855	2861	1	1	1	ACGTGg
HvGSK2.1	P$ABI3_01	ABI3	2861	2870	1	1	0.864	gcGCATGga
HvGSK2.1	P$TSAR2_01	TSAR2	2906	2916	1	1	0.93	aGCACGagca
HvGSK2.1	P$BHLH78_01	BHLH78	2907	2915	1	1	0.875	GCACGagc
HvGSK2.1	P$AT5G04240_01	AT5G04240	2929	2935	1	1	0.976	cGGCAC
HvGSK2.1	P$GATA11_01	GATA11	2938	2946	1	1	0.878	agGATCTg
HvGSK2.1	P$GATA8_01	GATA8	2938	2947	1	1	0.987	agGATCTga
HvGSK2.1	P$RRTF1_05	RRTF1	2940	2955	1	1	0.904	gatctgaCGGCGatg
HvGSK2.1	P$TGA1_01	TGA1	2941	2952	1	1	0.926	atcTGACGgcg
HvGSK2.1	P$TGA7_01	TGA7	2942	2952	1	1	0.872	tcTGACGgcg
HvGSK2.1	P$TGA5_01	TGA5	2943	2951	1	1	0.863	cTGACGgc
HvGSK2.1	P$AT1G28160_02	AT1G28160	2944	2959	1	1	0.866	tgaCGGCGatgcgcg
HvGSK2.1	P$AT1G68550_03	AT1G68550	2944	2953	1	1	0.964	tgaCGGCGa
HvGSK2.1	P$HSFA1E_01	HSFA1E	2946	2952	1	1	0.87	aCGGCG
HvGSK2.1	P$AT3G01030_01	AT3G01030	2959	2968	1	1	0.913	ACCGCaagt
HvGSK2.1	P$ATMYB77_01	ATMYB77	2987	3000	1	1	0.946	gatcgaCGGTTcc
HvGSK2.1	P$WRKY11_Q2	WRKY11	3005	3013	1	1	0.974	aTTGACcg
HvGSK2.1	P$ZAP1_01	ZAP1	3006	3016	1	1	0.98	TTGACcgcgc
HvGSK2.1	P$AT5G46350_01	AT5G46350	3009	3018	1	1	0.971	ACCGCgccg
HvGSK2.1	P$ERF112_02	ERF112	3011	3021	1	1	0.916	cgCGCCGagg
HvGSK2.1	P$CRF4_01	CRF4	3012	3020	1	1	0.872	gCGCCGag
HvGSK2.1	P$ERF4_04	ERF4	3012	3020	1	1	0.854	gCGCCGag
HvGSK2.1	P$ERF069_01	ERF069	3012	3021	1	1	0.987	gCGCCGagg
HvGSK2.1	P$ERF11_01	ERF11	3012	3022	1	1	0.959	gCGCCGaggg
HvGSK2.1	P$ERF8_01	ERF8	3013	3023	1	1	0.934	CGCCGagggt
HvGSK2.1	P$GATA15_01	GATA15	3035	3044	1	1	1	taTGATCga
HvGSK2.1	P$RAV1_01	RAV1	3081	3093	1	1	0.953	tatCAACAcaaa
HvGSK2.1	P$PBF_01	BF	3087	3098	1	1	0.969	cacAAAAGcat
HvGSK2.1	P$DOF_Q2	DOF	3087	3098	1	1	0.935	cacAAAAGcat
HvGSK2.1	P$DOF2_01	DOF2	3087	3098	1	1	0.991	cacaAAAGCat
HvGSK2.1	P$DOF3_01	DOF3	3087	3098	1	1	0.989	cacaAAAGCat
HvGSK2.1	P$CDF2_01	CDF2	3088	3098	1	1	0.959	acAAAAGcat
HvGSK2.1	P$CDF3_01	CDF3	3089	3098	1	1	0.977	cAAAAGcat
HvGSK2.1	P$LEC2_01	LEC2	3115	3126	1	1	0.994	ctCATGCaaat
HvGSK2.1	P$AT4G27900_01	AT4G27900	3147	3169	1	1	0.894	gatcAACGGttccgatcaatcg
HvGSK2.1	P$ATMYB77_01	ATMYB77	3147	3160	1	1	0.876	gatcaaCGGTTcc
HvGSK2.1	P$AT5G54070_01	AT5G54070	3149	3155	1	1	0.91	tCAACG
HvGSK2.1	P$HSFA2_01	HSFA2	3201	3207	1	1	1	CCAAAa
HvGSK2.1	P$P_01		3212	3221	1	1	0.876	cgCTACCaa
HvGSK2.1	P$MYBAS1_01	MYBAS1	3215	3226	1	1	0.973	taCCAACaaat
HvGSK2.1	P$RAV1_01	RAV1	3215	3227	1	1	0.959	tacCAACAaatg
HvGSK2.1	P$ERF112_02	ERF112	3259	3269	1	1	0.924	ttCGCCGgag
HvGSK2.1	P$CRF4_01	CRF4	3260	3268	1	1	0.854	tCGCCGga
HvGSK2.1	P$ERF4_04	ERF4	3260	3268	1	1	0.865	tCGCCGga
HvGSK2.1	P$ERF069_01	ERF069	3260	3269	1	1	0.989	tCGCCGgag
HvGSK2.1	P$ERF11_01	ERF11	3260	3270	1	1	0.97	tCGCCGgagt
HvGSK2.1	P$ERF8_01	ERF8	3261	3271	1	1	0.947	CGCCGgagtt
HvGSK2.1	P$SPL11_01	SPL11	3272	3284	1	1	0.881	tgtgGTACGcct
HvGSK2.1	P$SPL5_01	SPL5	3274	3283	1	1	0.965	tgGTACGcc
HvGSK2.1	P$POPTR_01	OPTR	3275	3282	1	1	0.929	gGTACGc
HvGSK2.1	P$SPL12_01	SPL12	3275	3283	1	1	0.97	gGTACGcc
HvGSK2.1	P$SPL4_01	SPL4	3275	3284	1	1	0.988	gGTACGcct

HvGSK2.1	P$AT1G66560_01	AT1G66560	3280	3290	1	1	0.868	gccTTAACta
HvGSK2.1	P$AT1G18860_01	AT1G18860	3281	3290	1	1	0.894	ccTTAACta
HvGSK2.1	P$AT1G64000_01	AT1G64000	3281	3290	1	1	0.856	ccTTAACta
HvGSK2.1	P$AT1G66600_01	AT1G66600	3281	3290	1	1	0.87	ccTTAACta
HvGSK2.1	P$AT1G68150_01	AT1G68150	3281	3290	1	1	0.857	ccTTAACta
HvGSK2.1	P$AT5G41570_01	AT5G41570	3281	3290	1	1	0.855	ccTTAACta
HvGSK2.1	P$AT5G15130_01	AT5G15130	3281	3290	1	1	0.89	ccTTAACta
HvGSK2.1	P$WRKY7_01	WRKY7	3281	3290	1	1	0.853	ccTTAACta
HvGSK2.1	P$C1_Q2	C1	3283	3294	1	1	0.952	ttAACTActaa
HvGSK2.1	P$PBF_01	BF	3291	3302	1	1	0.96	taaAAAAGatt
HvGSK2.1	P$DOF_Q2	DOF	3291	3302	1	1	0.975	taaAAAAGatt
HvGSK2.1	P$CDF2_01	CDF2	3292	3302	1	1	0.974	aaAAAAGatt
HvGSK2.1	P$CDF3_01	CDF3	3293	3302	1	1	0.973	aAAAAGatt
HvGSK2.1	P$GT1_Q6_02	GT1	3294	3306	1	1	0.852	aaaagaTTAACt
HvGSK2.1	P$C1_Q2	C1	3300	3311	1	1	0.958	ttAACTAcatc
HvGSK2.1	P$ATHSFA1D_01	ATHSFA1D	3303	3309	1	1	1	aCTACA
HvGSK2.1	P$AT3G18650_01	AT3G18650	3315	3326	1	1	0.851	tataaTTGTAa
HvGSK2.1	P$GT1_Q6	GT1	3322	3329	1	1	0.912	GTAAAca
HvGSK2.1	P$AT3G20750_01	AT3G20750	3322	3330	1	1	0.85	gTAAACaa
HvGSK2.1	P$FBP24_01	FBP24	3347	3365	1	1	0.871	gaatTAAACgtttacttg
HvGSK2.1	P$FBP28_01	FBP28	3347	3365	1	1	0.9	gaattaAACGTttacttg
HvGSK2.1	P$AT3G20750_01	AT3G20750	3350	3358	1	1	0.912	tTAAACgt
HvGSK2.1	P$RRTF1_05	RRTF1	3359	3374	1	1	0.883	tacttgaCGGCGtga
HvGSK2.1	P$TGA1_01	TGA1	3360	3371	1	1	0.922	actTGACGgcg
HvGSK2.1	P$WRKY11_Q2	WRKY11	3361	3369	1	1	0.897	cTTGACgg
HvGSK2.1	P$TGA7_01	TGA7	3361	3371	1	1	0.861	ctTGACGgcg
HvGSK2.1	P$ZAP1_01	ZAP1	3362	3372	1	1	0.866	TTGACggcgt
HvGSK2.1	P$TGA5_01	TGA5	3362	3370	1	1	0.856	tTGACGgc
HvGSK2.1	P$AT1G68550_03	AT1G68550	3363	3372	1	1	0.964	tgaCGGCGt
HvGSK2.1	P$HSFA1E_01	HSFA1E	3365	3371	1	1	0.87	aCGGCG
HvGSK2.1	P$C1_Q2	C1	3371	3382	1	1	0.948	tgAACTAaatg
HvGSK2.1	P$PDF2_01	DF2	3372	3383	1	1	0.908	gaacTAAATgt
HvGSK2.1	P$MYB3R5_01	MYB3R5	3397	3412	1	1	0.866	tttcaatgtCCGTTc
HvGSK2.1	P$MYB3R1_01	MYB3R1	3398	3413	1	1	0.887	ttcaatgtCCGTTcc
HvGSK2.1	P$MYB3R4_01	MYB3R4	3398	3413	1	1	0.891	ttcaatgtCCGTTcc
HvGSK2.1	P$DRE1C_01	DRE1C	3402	3410	1	1	0.862	ATGTCcgt
HvGSK2.1	P$MYB3R5_01	MYB3R5	3402	3417	1	1	0.854	atgtccgttCCGTTg
HvGSK2.1	P$MYB3R1_01	MYB3R1	3403	3418	1	1	0.871	tgtccgttCCGTTgt
HvGSK2.1	P$MYB3R4_01	MYB3R4	3403	3418	1	1	0.867	tgtccgttCCGTTgt
HvGSK2.1	P$HMG1_01	HMG1	3413	3422	1	1	0.913	GTTGTgatg
HvGSK2.1	P$BPC1_Q2	BPC1	3433	3439	1	1	0.997	AGAAAa
HvGSK2.1	P$AT2G15660_01	AT2G15660	3445	3456	1	1	0.909	TTCTCgtaaaa
HvGSK2.1	P$NAC6_01	NAC6	3448	3454	1	1	0.854	tCGTAA
HvGSK2.1	P$ANTL_02	ANTL	3463	3473	1	1	0.918	agtCGACAat
HvGSK2.1	P$ATHB6_01	ATHB6	3468	3477	1	1	0.921	acAATAAca
HvGSK2.1	P$ATMYB15_Q2	ATMYB15	3472	3478	1	1	1	TAACAa
HvGSK2.1	P$TGA1_01	TGA1	3475	3486	1	1	0.935	caaTGACGaga
HvGSK2.1	P$TGA7_01	TGA7	3476	3486	1	1	0.887	aaTGACGaga
HvGSK2.1	P$TGA5_01	TGA5	3477	3485	1	1	0.869	aTGACGag
HvGSK2.1	P$SEP3_01	wrz-03	3487	3498	1	1	0.866	cgcttTTTTGt
HvGSK2.1	P$GATA15_01	GATA15	3506	3515	1	1	0.999	tcTGATCgg
HvGSK2.1	P$AT5G26170_01	AT5G26170	3515	3524	1	1	0.867	ccTCAACtt
HvGSK2.1	P$BHLH112_01	BHLH112	3517	3526	1	1	0.934	tcaACTTGa
HvGSK2.1	P$SQUA_01	SQUA	3545	3555	1	1	0.886	caaTTTTTaa
HvGSK2.1	P$SBF1_01	SBF1	3545	3559	1	1	0.866	caatttTTAATaga
HvGSK2.1	P$BPC1_Q2	BPC1	3556	3562	1	1	0.997	AGAAAa
HvGSK2.1	P$HSFA4A_01	HSFA4A	3579	3585	1	1	1	aCTATT
HvGSK2.1	P$AT3G20750_01	AT3G20750	3587	3595	1	1	0.938	aTAAACtt
HvGSK2.1	P$ID1_01	ID1	3592	3603	1	1	0.868	cTTGTCgattt
HvGSK2.1	P$ARF8_01	ARF8	3592	3601	1	1	0.958	ctTGTCGat
HvGSK2.1	P$SQUA_01	SQUA	3597	3607	1	1	0.871	cgaTTTTTtt
HvGSK2.1	P$GT1_Q6_01	GT1	3600	3612	1	1	0.905	TTTTTttataga
HvGSK2.1	P$ARR18_01	ARR18	3605	3618	1	1	0.953	ttatAGATAcaaa
HvGSK2.1	P$AT3G60580_01	AT3G60580	3615	3622	1	1	0.883	aaATCCC
HvGSK2.1	P$DOF2_01	DOF2	3622	3633	1	1	0.981	cgcgAAAGCct
HvGSK2.1	P$DOF3_01	DOF3	3622	3633	1	1	0.975	cgcgAAAGCct
HvGSK2.1	P$PDF2_01	DF2	3650	3661	1	1	0.882	aaatTAAATgg
HvGSK2.1	P$E2L_Q2	E2L	3661	3668	1	1	0.892	tGGCGGt
HvGSK2.1	P$ABI3_01	ABI3	3669	3678	1	1	0.865	atGCATGgc
HvGSK2.1	P$MYBAS1_01	MYBAS1	3675	3686	1	1	0.952	ggCCAACgtcg
HvGSK2.1	P$AT5G54070_01	AT5G54070	3677	3683	1	1	1	cCAACG
HvGSK2.1	P$TGA1A_01	TGA1A	3679	3686	1	1	0.871	aACGTCg
HvGSK2.1	P$MYB3_01	MYB3	3692	3703	1	1	0.9	ccgTAGGTtta
HvGSK2.1	P$MYB4_01	MYB4	3693	3701	1	1	0.917	cgTAGGTt
HvGSK2.1	P$ARR2_01	ARR2	3698	3708	1	1	0.861	gtttATCTTt
HvGSK2.1	P$AT1G77950_01	AT1G77950	3700	3711	1	1	0.895	ttatcTTTAAt
HvGSK3.1	P$KNOX3_01	KNOX3	18	30	1	1	0.961	ttcaTGACAata
HvGSK3.1	P$ATH1_01	ATH1	22	30	1	1	0.92	TGACAata

HvGSK3.1	P$ATHB6_01	ATHB6	24	33	1	1	0.923	acAATAAac
HvGSK3.1	P$MYBAS1_01	MYBAS1	35	46	1	1	0.976	atCCAACacat
HvGSK3.1	P$RAV1_01	RAV1	35	47	1	1	0.957	atcCAACAcatc
HvGSK3.1	P$SED_Q2	SED	70	80	1	1	0.936	ccgtCCTTTg
HvGSK3.1	P$PBF_Q2_01	BF	74	80	1	1	0.988	CCTTTg
HvGSK3.1	P$ATHB9_01	ATHB9	107	126	1	1	0.865	gaagggaATGATaaccttg
HvGSK3.1	P$BPC1_Q2	BPC1	126	132	1	1	0.99	AGAAAc
HvGSK3.1	P$ARR2_01	ARR2	153	163	1	1	0.903	ccacATCTTt
HvGSK3.1	P$CBNAC_01	CBNAC	161	167	1	1	1	tTGCTT
HvGSK3.1	P$CBNAC_02	CBNAC	161	177	1	1	0.861	tTGCTTccatgatctt
HvGSK3.1	P$GATA15_01	GATA15	168	177	1	1	0.999	caTGATCtt
HvGSK3.1	P$ARR2_01	ARR2	168	178	1	1	0.872	catgATCTTg
HvGSK3.1	P$GATA8_01	GATA8	169	178	1	1	0.976	atGATCTtg
HvGSK3.1	P$NAC078_Q2	NAC078	173	188	1	1	0.89	tctTGGAGaccaaca
HvGSK3.1	P$MYBAS1_01	MYBAS1	180	191	1	1	0.956	gaCCAACaaca
HvGSK3.1	P$RAV1_01	RAV1	180	192	1	1	0.933	gacCAACAacaa
HvGSK3.1	P$GAMYB_Q2	GAMYB	180	193	1	1	0.961	gaccaACAACaag
HvGSK3.1	P$RAV1_01	RAV1	183	195	1	1	0.911	caaCAACAagcg
HvGSK3.1	P$AT4G36620_01	AT4G36620	262	270	1	1	0.907	aagAACCA
HvGSK3.1	P$SPF1_Q2	SPF1	272	282	1	1	0.87	tcATAGTtag
HvGSK3.1	P$CBNAC_01	CBNAC	290	296	1	1	0.968	aTGCTT
HvGSK3.1	P$O2_03	O2	316	326	1	1	0.925	GATGAtgtgc
HvGSK3.1	P$MYBAS1_01	MYBAS1	326	337	1	1	0.977	tcCCAACaatc
HvGSK3.1	P$RAV1_01	RAV1	326	338	1	1	0.932	tccCAACAatct
HvGSK3.1	P$ARR2_01	ARR2	330	340	1	1	0.885	aacaATCTTt
HvGSK3.1	P$AT5G04240_01	AT5G04240	339	345	1	1	0.938	tGGCAC
HvGSK3.1	P$NAC083_01	NAC083	353	363	1	1	0.943	ggACGCAagt
HvGSK3.1	P$RAV1_01	RAV1	372	384	1	1	0.918	cttCAACAggga
HvGSK3.1	P$DOF2_01	DOF2	400	411	1	1	0.981	gaacAAAGCag
HvGSK3.1	P$DOF3_01	DOF3	400	411	1	1	0.993	gaacAAAGCag
HvGSK3.1	P$REF6_01	REF6	404	415	1	1	0.865	aaagCAGAGtg
HvGSK3.1	P$HMG1_01	HMG1	420	429	1	1	0.852	GTTGTttgt
HvGSK3.1	P$GT1_Q6_02	GT1	422	434	1	1	0.959	tgtttgTTAACc
HvGSK3.1	P$WRKY21_01	WRKY21	426	435	1	1	0.852	tgTTAACct
HvGSK3.1	P$WRKY43_01	WRKY43	426	435	1	1	0.912	tgTTAACct
HvGSK3.1	P$AT4G11070_01	AT4G11070	426	435	1	1	0.855	tgTTAACct
HvGSK3.1	P$AT1G64000_01	AT1G64000	426	435	1	1	0.87	tgTTAACct
HvGSK3.1	P$AT5G41570_01	AT5G41570	426	435	1	1	0.87	tgTTAACct
HvGSK3.1	P$WRKY46_01	WRKY46	426	435	1	1	0.87	tgTTAACct
HvGSK3.1	P$WRKY7_01	WRKY7	426	435	1	1	0.852	tgTTAACct
HvGSK3.1	P$WRKY25_01	WRKY25	426	435	1	1	0.858	tgtTAACCt
HvGSK3.1	P$WRKY33_01	WRKY33	426	435	1	1	0.881	tgtTAACCt
HvGSK3.1	P$AT1G29860_01	AT1G29860	426	435	1	1	0.873	tgtTAACCt
HvGSK3.1	P$AT3G62340_01	AT3G62340	426	435	1	1	0.861	tgtTAACCt
HvGSK3.1	P$AT1G69310_01	AT1G69310	426	435	1	1	0.865	tgtTAACCt
HvGSK3.1	P$WRKY26_01	WRKY26	426	435	1	1	0.882	tgtTAACCt
HvGSK3.1	P$GT1_01	GT1	427	435	1	1	0.977	gtTAACCt
HvGSK3.1	P$CBNAC_01	CBNAC	452	458	1	1	1	tTGCTT
HvGSK3.1	P$CBNAC_02	CBNAC	452	468	1	1	0.903	tTGCTTggatcgtgcg
HvGSK3.1	P$BZR1_01	BZR1	462	468	1	1	1	CGTGCg
HvGSK3.1	P$MYB1L_01	MYB1L	474	484	1	1	0.948	acCCCTActc
HvGSK3.1	P$TRB2_01	TRB2	474	482	1	1	0.925	acCCCTAc
HvGSK3.1	P$ANTL_02	ANTL	480	490	1	1	0.897	actCGACAat
HvGSK3.1	P$HSFA2_01	HSFA2	493	499	1	1	0.933	CCAAAg
HvGSK3.1	P$HMG1_01	HMG1	498	507	1	1	0.953	GTTGTgttt
HvGSK3.1	P$HMG1_01	HMG1	508	517	1	1	0.925	GTTGTggtt
HvGSK3.1	P$PIL5_01	IL5	517	531	1	1	0.879	caaatgagACGTGa
HvGSK3.1	P$O2_Q2	O2	519	532	1	1	0.877	aatgaGACGTgat
HvGSK3.1	P$ABZ1_01	ABZ1	520	534	1	1	0.888	atgagACGTGatac
HvGSK3.1	P$TGA1B_01	TGA1B	522	532	1	1	0.954	gaGACGTgat
HvGSK3.1	P$HBP1A_Q2	HBP1A	522	532	1	1	0.896	gagACGTGat
HvGSK3.1	P$TAF1_Q2	TAF1	522	532	1	1	0.942	gagACGTGat
HvGSK3.1	P$EMBP1_02	EMBP1	522	532	1	1	0.908	gagACGTGat
HvGSK3.1	P$TAF1_01	TAF1	522	532	1	1	0.957	gagACGTGat
HvGSK3.1	P$TRAB1_Q2	TRAB1	523	534	1	1	0.856	agACGTGatac
HvGSK3.1	P$GBF1_01	GBF1	523	531	1	1	0.934	agACGTGa
HvGSK3.1	P$BIM1_02	BIM1	523	533	1	1	0.948	agACGTGata
HvGSK3.1	P$ABF4_02	ABF4	523	533	1	1	0.95	agACGTGata
HvGSK3.1	P$ABI5_Q2	ABI5	525	531	1	1	0.936	ACGTGa
HvGSK3.1	P$ASR1_01	ASR1	539	544	1	1	1	ACCCA
HvGSK3.1	P$ATHB7_01	ATHB7	550	560	1	1	0.921	aaAATCAttc
HvGSK3.1	P$HAT1_01	HAT1	550	560	1	1	0.98	aaAATCAttc
HvGSK3.1	P$ATHB4_02	ATHB4	551	561	1	1	0.92	aaATCATtct
HvGSK3.1	P$GT1_Q6_02	GT1	554	566	1	1	0.968	tcattcTTAACa
HvGSK3.1	P$AT1G66560_01	AT1G66560	557	567	1	1	0.916	ttcTTAACaa
HvGSK3.1	P$WRKY21_01	WRKY21	558	567	1	1	0.895	tcTTAACaa
HvGSK3.1	P$WRKY43_01	WRKY43	558	567	1	1	0.879	tcTTAACaa
HvGSK3.1	P$AT4G11070_01	AT4G11070	558	567	1	1	0.962	tcTTAACaa

HvGSK3.1	P$AT1G18860_01	AT1G18860	558	567	1	1	0.914	tcTTAACaa
HvGSK3.1	P$AT1G64000_01	AT1G64000	558	567	1	1	0.876	tcTTAACaa
HvGSK3.1	P$AT1G66600_01	AT1G66600	558	567	1	1	0.912	tcTTAACaa
HvGSK3.1	P$AT1G68150_01	AT1G68150	558	567	1	1	0.875	tcTTAACaa
HvGSK3.1	P$AT5G41570_01	AT5G41570	558	567	1	1	0.876	tcTTAACaa
HvGSK3.1	P$AT5G15130_01	AT5G15130	558	567	1	1	0.91	tcTTAACaa
HvGSK3.1	P$WRKY46_01	WRKY46	558	567	1	1	0.908	tcTTAACaa
HvGSK3.1	P$AT2G24570_01	AT2G24570	558	567	1	1	0.884	tcTTAACaa
HvGSK3.1	P$WRKY7_01	WRKY7	558	567	1	1	0.899	tcTTAACaa
HvGSK3.1	P$WRKY48_01	WRKY48	558	567	1	1	0.872	tcttAACAA
HvGSK3.1	P$ATMYB15_Q2	ATMYB15	561	567	1	1	1	TAACAa
HvGSK3.1	P$MYB24_01	MYB24	566	575	1	1	0.862	agtTTAGGa
HvGSK3.1	P$AT3G25990_01	AT3G25990	579	600	1	1	0.859	gaccatGGTTAacaatatgcg
HvGSK3.1	P$ATMYB15_Q2	ATMYB15	588	594	1	1	1	TAACAa
HvGSK3.1	P$ALFIN1_Q2	ALFIN1	627	642	1	1	0.877	tgggagGTGGGacac
HvGSK3.1	P$TCP7_01	TCP7	633	644	1	1	0.857	GTGGGacacat
HvGSK3.1	P$LEC2_01	LEC2	639	650	1	1	0.986	caCATGCaaag
HvGSK3.1	P$PBF_Q2	BF	645	651	1	1	0.958	cAAAGG
HvGSK3.1	P$PCF2_01	CF2	653	663	1	1	0.88	ttcgcCCCAC
HvGSK3.1	P$TCP19_01	TCP19	653	663	1	1	0.861	ttcgcCCCAC
HvGSK3.1	P$TCP20L_01	TCP20L	654	663	1	1	0.856	tcgcCCCAC
HvGSK3.1	P$TCP20_02	TCP20	655	665	1	1	0.9	cgcCCCACtc
HvGSK3.1	P$ARALY495258_02	ARALY495258	655	663	1	1	0.85	cgcCCCAC
HvGSK3.1	P$ARALY484486_05	ARALY484486	655	663	1	1	0.85	cgcCCCAC
HvGSK3.1	P$GATA11_01	GATA11	663	671	1	1	0.854	tcGATCTc
HvGSK3.1	P$GATA8_01	GATA8	663	672	1	1	0.981	tcGATCTct
HvGSK3.1	P$GT1_Q6_01	GT1	679	691	1	1	0.876	TTTGTttctatc
HvGSK3.1	P$MRP1_Q2	MRP1	683	695	1	1	0.854	ttTCTATcagta
HvGSK3.1	P$SED_Q2	SED	701	711	1	1	0.98	ttttCCTTTt
HvGSK3.1	P$AGL15_03	AGL15	702	717	1	1	0.905	TTTCCttttcaggat
HvGSK3.1	P$PBF_Q2_01	BF	705	711	1	1	1	CCTTTt
HvGSK3.1	P$AT4G36620_01	AT4G36620	723	731	1	1	0.957	ctgAACCA
HvGSK3.1	P$HSFA2_01	HSFA2	728	734	1	1	0.933	CCAAAg
HvGSK3.1	P$PBF_Q2	BF	729	735	1	1	0.958	cAAAGG
HvGSK3.1	P$GT1_01	GT1	742	750	1	1	0.958	atTAACCa
HvGSK3.1	P$AT4G36620_01	AT4G36620	742	750	1	1	0.99	attAACCA
HvGSK3.1	P$PEND_01	END	764	772	1	1	0.901	gAAGAAgt
HvGSK3.1	P$ARR2_01	ARR2	786	796	1	1	0.896	ggaaATCTTt
HvGSK3.1	P$WRKY11_Q2	WRKY11	805	813	1	1	0.978	tTTGACct
HvGSK3.1	P$ZAP1_01	ZAP1	806	816	1	1	0.908	TTGACctcga
HvGSK3.1	P$PDF2_01	DF2	827	838	1	1	0.921	gactTAAATgt
HvGSK3.1	P$EDF3_02	EDF3	867	876	1	1	0.89	cGACCGaga
HvGSK3.1	P$RAV2_01	RAV2	867	876	1	1	0.887	cgACCGAga
HvGSK3.1	P$MYBAS1_01	MYBAS1	899	910	1	1	0.948	ccCCAACccgg
HvGSK3.1	P$GAMYB_01	GAMYB	902	910	1	1	0.865	CAACCcgg
HvGSK3.1	P$SBF1_01	SBF1	942	956	1	1	0.881	aaacccTTAATaaa
HvGSK3.1	P$ATHB6_01	ATHB6	948	957	1	1	0.911	ttAATAAag
HvGSK3.1	P$DOF1_01	DOF1	949	960	1	1	0.979	taaTAAAGaga
HvGSK3.1	P$PBF_Q2	BF	979	985	1	1	0.965	gAAAGG
HvGSK3.1	P$WRKY11_01	WRKY11	991	1005	1	1	0.87	atggTTGACtagag
HvGSK3.1	P$WRKY11_Q2	WRKY11	994	1002	1	1	0.953	gTTGACta
HvGSK3.1	P$MYBAS1_01	MYBAS1	1014	1025	1	1	0.977	agCCAACtaac
HvGSK3.1	P$C1_Q2	C1	1016	1027	1	1	0.974	ccAACTAacaa
HvGSK3.1	P$WEREWOLF_Q2_01	WEREWOLF	1018	1027	1	1	0.991	aACTAAcaa
HvGSK3.1	P$MYBAS1_01	MYBAS1	1018	1029	1	1	0.975	aaCTAACaagt
HvGSK3.1	P$ATMYB15_Q2	ATMYB15	1021	1027	1	1	1	TAACAa
HvGSK3.1	P$SBF1_01	SBF1	1036	1050	1	1	0.862	ttttctTTAAAcaa
HvGSK3.1	P$WRKY48_01	WRKY48	1041	1050	1	1	0.914	tttaAACAA
HvGSK3.1	P$AT3G20750_01	AT3G20750	1042	1050	1	1	0.876	tTAAACaa
HvGSK3.1	P$MYB24_01	MYB24	1048	1057	1	1	0.964	aaaTTAGGt
HvGSK3.1	P$MYB131_01	MYB131	1048	1059	1	1	0.947	aaaTTAGGttt
HvGSK3.1	P$MYB4_01	MYB4	1050	1058	1	1	0.862	atTAGGTt
HvGSK3.1	P$C1_Q2	C1	1063	1074	1	1	0.923	aaAACTAggtc
HvGSK3.1	P$ARR18_01	ARR18	1074	1087	1	1	0.959	gtctAGATAtgca
HvGSK3.1	P$C1_Q2	C1	1084	1095	1	1	0.935	gcAACTAggag
HvGSK3.1	P$GAMYB_01	GAMYB	1097	1105	1	1	0.86	CAACCtat
HvGSK3.1	P$MYBAS1_01	MYBAS1	1109	1120	1	1	0.955	tgCTAACaaca
HvGSK3.1	P$GAMYB_Q2	GAMYB	1109	1122	1	1	0.965	tgctaACAACaac
HvGSK3.1	P$ATMYB15_Q2	ATMYB15	1112	1118	1	1	1	TAACAa
HvGSK3.1	P$RAV1_01	RAV1	1112	1124	1	1	0.925	taaCAACAacaa
HvGSK3.1	P$GAMYB_Q2	GAMYB	1112	1125	1	1	0.943	taacaACAACaat
HvGSK3.1	P$RAV1_01	RAV1	1115	1127	1	1	0.942	caaCAACAataa
HvGSK3.1	P$ATHB6_01	ATHB6	1120	1129	1	1	0.926	acAATAAaa
HvGSK3.1	P$RIN_Q2_01	RIN	1120	1132	1	1	0.933	acaataAAAAGc
HvGSK3.1	P$PBF_01	BF	1123	1134	1	1	0.982	ataAAAAGcaa
HvGSK3.1	P$DOF_Q2	DOF	1123	1134	1	1	0.996	ataAAAAGcaa
HvGSK3.1	P$DOF2_01	DOF2	1123	1134	1	1	0.994	ataaAAAGCaa
HvGSK3.1	P$DOF3_01	DOF3	1123	1134	1	1	0.982	ataaAAAGCaa

HvGSK3.1	P$CDF2_01	CDF2	1124	1134	1	1	0.987	taAAAAGcaa
HvGSK3.1	P$CDF3_01	CDF3	1125	1134	1	1	0.984	aAAAAGcaa
HvGSK3.1	P$ATHB6_01	ATHB6	1130	1139	1	1	0.932	gcAATAAgt
HvGSK3.1	P$GT1_Q6	GT1	1137	1144	1	1	1	GTAAAta
HvGSK3.1	P$PEND_01	END	1154	1162	1	1	0.972	tAAGAAat
HvGSK3.1	P$BPC1_Q2	BPC1	1156	1162	1	1	0.99	AGAAAt
HvGSK3.1	P$ATHB6_01	ATHB6	1157	1166	1	1	0.898	gaAATAAcc
HvGSK3.1	P$AT4G36620_01	AT4G36620	1159	1167	1	1	0.916	aatAACCA
HvGSK3.1	P$AT4G12750_01	AT4G12750	1172	1182	1	1	0.943	gaaACCGAtg
HvGSK3.1	P$RAV2_01	RAV2	1173	1182	1	1	0.879	aaACCGAtg
HvGSK3.1	P$AT2G41690_01	AT2G41690	1199	1205	1	1	0.988	CCGAAg
HvGSK3.1	P$SED_Q2	SED	1208	1218	1	1	0.993	cttcCCTTTt
HvGSK3.1	P$RIN_Q2	RIN	1211	1222	1	1	0.963	ccctTTTAAgg
HvGSK3.1	P$PBF_Q2_01	BF	1212	1218	1	1	1	CCTTTt
HvGSK3.1	P$SPL11_01	SPL11	1221	1233	1	1	0.9	ggaaGTACGtct
HvGSK3.1	P$SPL5_01	SPL5	1223	1232	1	1	0.972	aaGTACGtc
HvGSK3.1	P$POPTR_01	OPTR	1224	1231	1	1	0.93	aGTACGt
HvGSK3.1	P$SPL12_01	SPL12	1224	1232	1	1	0.977	aGTACGtc
HvGSK3.1	P$SPL4_01	SPL4	1224	1233	1	1	0.992	aGTACGtct
HvGSK3.1	P$MYB3R5_01	MYB3R5	1224	1239	1	1	0.901	agtacgtctCCGTTg
HvGSK3.1	P$MYB3R1_01	MYB3R1	1225	1240	1	1	0.912	gtacgtctCCGTTgg
HvGSK3.1	P$MYB3R4_01	MYB3R4	1225	1240	1	1	0.919	gtacgtctCCGTTgg
HvGSK3.1	P$TGA1A_01	TGA1A	1226	1233	1	1	0.882	tACGTCt
HvGSK3.1	P$AT5G04240_01	AT5G04240	1250	1256	1	1	0.939	aGGCAC
HvGSK3.1	P$ARF8_01	ARF8	1316	1325	1	1	0.95	agTGTCGtg
HvGSK3.1	P$AT4G36620_01	AT4G36620	1352	1360	1	1	0.884	ggcAACCA
HvGSK3.1	P$GAMYB_01	GAMYB	1354	1362	1	1	0.886	CAACCaga
HvGSK3.1	P$SED_Q2	SED	1358	1368	1	1	0.907	cagaCCTTTa
HvGSK3.1	P$PBF_Q2_01	BF	1362	1368	1	1	0.998	CCTTTa
HvGSK3.1	P$GAMYB_Q2	GAMYB	1362	1375	1	1	0.889	cctttACAACaag
HvGSK3.1	P$RAV1_01	RAV1	1365	1377	1	1	0.911	ttaCAACAaggt
HvGSK3.1	P$GAMYB_Q2	GAMYB	1385	1398	1	1	0.926	tctccACAACtca
HvGSK3.1	P$MYBAS1_01	MYBAS1	1407	1418	1	1	0.949	tcCCAACaaaa
HvGSK3.1	P$RAV1_01	RAV1	1407	1419	1	1	0.964	tccCAACAaaac
HvGSK3.1	P$AT4G36620_01	AT4G36620	1413	1421	1	1	0.895	caaAACCA
HvGSK3.1	P$PBF_Q2	BF	1449	1455	1	1	0.958	cAAAGG
HvGSK3.1	P$ASR1_01	ASR1	1483	1488	1	1	1	ACCCA
HvGSK3.1	P$PEND_01	END	1486	1494	1	1	0.854	cAAGAAct
HvGSK3.1	P$ATHB6_01	ATHB6	1498	1507	1	1	0.905	ggAATAAat
HvGSK3.1	P$PDF2_01	DF2	1498	1509	1	1	0.885	ggaaTAAATtt
HvGSK3.1	P$SED_Q2	SED	1506	1516	1	1	0.953	ttttCCTTTg
HvGSK3.1	P$PBF_Q2_01	BF	1510	1516	1	1	0.988	CCTTTg
HvGSK3.1	P$GATA9_01	GATA9	1523	1534	1	1	0.989	tgtAGATCtag
HvGSK3.1	P$AGP1_01	AGP1	1524	1534	1	1	0.985	gtAGATCtag
HvGSK3.1	P$GATA10_01	GATA10	1525	1533	1	1	0.954	tAGATCta
HvGSK3.1	P$GATA11_01	GATA11	1525	1533	1	1	0.964	taGATCTa
HvGSK3.1	P$GATA8_01	GATA8	1525	1534	1	1	1	taGATCTag
HvGSK3.1	P$ARR10_01	ARR10	1526	1533	1	1	0.913	AGATCta
HvGSK3.1	P$GAMYB_Q2	GAMYB	1554	1567	1	1	0.867	gcaatACAACaag
HvGSK3.1	P$RAV1_01	RAV1	1557	1569	1	1	0.917	ataCAACAagtt
HvGSK3.1	P$GATA9_01	GATA9	1583	1594	1	1	0.886	gagAGATCggg
HvGSK3.1	P$AGP1_01	AGP1	1584	1594	1	1	0.86	agAGATCggg
HvGSK3.1	P$GATA10_01	GATA10	1585	1593	1	1	0.893	gAGATCgg
HvGSK3.1	P$ARR10_01	ARR10	1586	1593	1	1	0.934	AGATCgg
HvGSK3.1	P$BPC1_Q2	BPC1	1596	1602	1	1	0.99	AGAAAc
HvGSK3.1	P$RAMOSA1_01	RAMOSA1	1617	1631	1	1	0.887	ggtggaGAGAGaga
HvGSK3.1	P$RAMOSA1_01	RAMOSA1	1619	1633	1	1	0.91	tggagaGAGAGagg
HvGSK3.1	P$RAMOSA1_01	RAMOSA1	1621	1635	1	1	0.909	gagagaGAGAGgta
HvGSK3.1	P$ARR18_01	ARR18	1633	1646	1	1	0.94	taagAGATAagag
HvGSK3.1	P$BPC1_Q2	BPC1	1655	1661	1	1	0.997	AGAAAa
HvGSK3.1	P$AT4G36620_01	AT4G36620	1687	1695	1	1	0.912	tatAACCA
HvGSK3.1	P$GAMYB_Q2	GAMYB	1692	1705	1	1	0.869	ccattACAACtat
HvGSK3.1	P$C1_Q2	C1	1697	1708	1	1	0.927	acAACTAtttt
HvGSK3.1	P$HSFA4A_01	HSFA4A	1700	1706	1	1	1	aCTATT
HvGSK3.1	P$SQUA_01	SQUA	1701	1711	1	1	0.897	ctaTTTTTca
HvGSK3.1	P$RAP21_02	RAP21	1712	1725	1	1	0.908	tggaCGGTGcaac
HvGSK3.1	P$GAMYB_01	GAMYB	1721	1729	1	1	0.911	CAACCggt
HvGSK3.1	P$AT1G53910_01	AT1G53910	1733	1743	1	1	0.919	aGGCCGgtac
HvGSK3.1	P$ARALY897773_01	ARALY897773	1736	1746	1	1	0.889	ccggtACCAC
HvGSK3.1	P$GAMYB_Q2	GAMYB	1762	1775	1	1	0.912	gcggaACAACtgg
HvGSK3.1	P$MYBAS1_01	MYBAS1	1773	1784	1	1	0.984	ggCCAACcacc
HvGSK3.1	P$AT4G36620_01	AT4G36620	1774	1782	1	1	0.886	gccAACCA
HvGSK3.1	P$GAMYB_01	GAMYB	1776	1784	1	1	0.953	CAACCacc
HvGSK3.1	P$ABI4_01	ABI4	1777	1788	1	1	0.942	aaccaCCGCCc
HvGSK3.1	P$AT5G46350_01	AT5G46350	1781	1790	1	1	0.871	ACCGCccaa
HvGSK3.1	P$AT3G01030_01	AT3G01030	1781	1790	1	1	0.855	ACCGCccaa
HvGSK3.1	P$AT3G63350_01	AT3G63350	1782	1788	1	1	1	CCGCCc
HvGSK3.1	P$MYBAS1_01	MYBAS1	1784	1795	1	1	0.985	gcCCAACaacc

HvGSK3.1	P$RAV1_01	RAV1	1784	1796	1	1	0.918	gccCAACAaccg
HvGSK3.1	P$GAMYB_Q2	GAMYB	1784	1797	1	1	0.959	gcccaACAACcgg
HvGSK3.1	P$GAMYB_01	GAMYB	1790	1798	1	1	0.92	CAACCggg
HvGSK3.1	P$FLC_01	FLC	1793	1814	1	1	0.946	ccgggccaaaaacAGAAAgtc
HvGSK3.1	P$AP3_01	AP3	1796	1811	1	1	0.917	ggcCAAAAacagaaa
HvGSK3.1	P$HSFA2_01	HSFA2	1798	1804	1	1	1	CCAAAa
HvGSK3.1	P$AP1_01	AP1	1798	1811	1	1	0.962	ccAAAAAcagaaa
HvGSK3.1	P$BPC1_Q2	BPC1	1806	1812	1	1	1	AGAAAg
HvGSK3.1	P$DREB1A_04	DREB1A	1809	1819	1	1	0.985	aaGTCGGccc
HvGSK3.1	P$ERF039_01	ERF039	1809	1819	1	1	0.979	aaGTCGGccc
HvGSK3.1	P$PHYPA28324_10	HYPA28324	1810	1818	1	1	0.871	aGTCGGcc
HvGSK3.1	P$LIM1_01	LIM1	1826	1838	1	1	0.959	CCACCagccacg
HvGSK3.1	P$AT1G53910_01	AT1G53910	1836	1846	1	1	0.884	cGGCCGctgg
HvGSK3.1	P$DREBI5_01	DREBI5	1836	1846	1	1	0.888	cgGCCGCtgg
HvGSK3.1	P$DREB2E_01	DREB2E	1836	1847	1	1	0.887	cgGCCGCtggc
HvGSK3.1	P$AT2G47520_01	AT2G47520	1836	1846	1	1	0.857	cgGCCGCtgg
HvGSK3.1	P$AT1G77200_01	AT1G77200	1836	1846	1	1	0.9	cgGCCGCtgg
HvGSK3.1	P$ATERF14_01	ATERF14	1836	1846	1	1	0.882	cgGCCGCtgg
HvGSK3.1	P$DREBIII3_01	DREBIII3	1836	1846	1	1	0.88	cgGCCGCtgg
HvGSK3.1	P$DREBIII2_01	DREBIII2	1836	1846	1	1	0.878	cgGCCGCtgg
HvGSK3.1	P$ERF4_02	ERF4	1836	1846	1	1	0.861	cgGCCGCtgg
HvGSK3.1	P$DREBIII1_01	DREBIII1	1836	1846	1	1	0.88	cgGCCGCtgg
HvGSK3.1	P$DREBII1_01	DREBII1	1836	1846	1	1	0.886	cgGCCGCtgg
HvGSK3.1	P$AT2G44940_01	AT2G44940	1836	1846	1	1	0.891	cgGCCGCtgg
HvGSK3.1	P$DBF2_01	DBF2	1836	1846	1	1	0.92	cgGCCGCtgg
HvGSK3.1	P$CBF5_01	CBF5	1836	1846	1	1	0.85	cgGCCGCtgg
HvGSK3.1	P$CBF16_01	CBF16	1836	1846	1	1	0.882	cgGCCGCtgg
HvGSK3.1	P$CBF17_01	CBF17	1836	1846	1	1	0.889	cgGCCGCtgg
HvGSK3.1	P$CBF_01	CBF	1836	1846	1	1	0.891	cgGCCGCtgg
HvGSK3.1	P$AT3G61630_01	AT3G61630	1836	1846	1	1	0.879	cgGCCGCtgg
HvGSK3.1	P$AT5G43410_01	AT5G43410	1836	1846	1	1	0.887	cgGCCGCtgg
HvGSK3.1	P$TINY2_02	TINY2	1836	1846	1	1	0.934	cgGCCGCtgg
HvGSK3.1	P$AT3G16280_01	AT3G16280	1836	1846	1	1	0.91	cgGCCGCtgg
HvGSK3.1	P$CRF2_01	CRF2	1836	1844	1	1	0.892	cgGCCGCt
HvGSK3.1	P$ERF098_01	ERF098	1836	1844	1	1	0.893	cgGCCGCt
HvGSK3.1	P$ERF7_02	ERF7	1837	1847	1	1	0.937	gGCCGCtggc
HvGSK3.1	P$BZIP68_01	BZIP68	1843	1852	1	1	0.948	tggCGTGGc
HvGSK3.1	P$GAMYB_Q2	GAMYB	1862	1875	1	1	0.885	gcggtACAACagc
HvGSK3.1	P$RAV1_01	RAV1	1865	1877	1	1	0.926	gtaCAACAgctt
HvGSK3.1	P$ABI4_01	ABI4	1878	1889	1	1	0.92	gaacaCCGCCa
HvGSK3.1	P$AT3G01030_01	AT3G01030	1882	1891	1	1	0.981	ACCGCcagt
HvGSK3.1	P$AT3G63350_01	AT3G63350	1883	1889	1	1	0.882	CCGCCa
HvGSK3.1	P$MYB89_01	MYB89	1888	1899	1	1	0.91	agTACCGgcta
HvGSK3.1	P$SBF1_01	SBF1	1896	1910	1	1	0.932	ctataaTTAATata
HvGSK3.1	P$EDT1_01	EDT1	1899	1909	1	1	0.892	taaTTAATat
HvGSK3.1	P$GAMYB_Q2	GAMYB	1910	1923	1	1	0.872	gcggtACAACcgg
HvGSK3.1	P$GAMYB_01	GAMYB	1916	1924	1	1	0.92	CAACCggg
HvGSK3.1	P$LIM1_01	LIM1	1924	1936	1	1	0.974	CCACCaccgccc
HvGSK3.1	P$ABI4_01	ABI4	1925	1936	1	1	0.941	caccaCCGCCc
HvGSK3.1	P$AT5G46350_01	AT5G46350	1929	1938	1	1	0.871	ACCGCccaa
HvGSK3.1	P$AT3G01030_01	AT3G01030	1929	1938	1	1	0.855	ACCGCccaa
HvGSK3.1	P$AT3G63350_01	AT3G63350	1930	1936	1	1	1	CCGCCc
HvGSK3.1	P$MYBAS1_01	MYBAS1	1932	1943	1	1	0.985	gcCCAACaacc
HvGSK3.1	P$RAV1_01	RAV1	1932	1944	1	1	0.918	gccCAACAaccg
HvGSK3.1	P$GAMYB_Q2	GAMYB	1932	1945	1	1	0.959	gcccaACAACcgt
HvGSK3.1	P$GAMYB_01	GAMYB	1938	1946	1	1	0.938	CAACCgta
HvGSK3.1	P$BHLH64_02	BHLH64	1962	1968	1	1	1	ACCAGt
HvGSK3.1	P$AT1G53910_01	AT1G53910	1982	1992	1	1	0.918	gGGCCGgtac
HvGSK3.1	P$GAMYB_Q2	GAMYB	1985	1998	1	1	0.885	ccggtACAACcgc
HvGSK3.1	P$GAMYB_01	GAMYB	1991	1999	1	1	1	CAACCgcc
HvGSK3.1	P$AT5G46350_01	AT5G46350	1993	2002	1	1	0.936	ACCGCccag
HvGSK3.1	P$AT3G01030_01	AT3G01030	1993	2002	1	1	0.853	ACCGCccag
HvGSK3.1	P$AT3G63350_01	AT3G63350	1994	2000	1	1	1	CCGCCc
HvGSK3.1	P$CDC5_01	CDC5	2003	2014	1	1	0.853	tgtTCAGCggt
HvGSK3.1	P$GAMYB_Q2	GAMYB	2009	2022	1	1	0.881	gcggtACAACcgc
HvGSK3.1	P$GAMYB_01	GAMYB	2015	2023	1	1	1	CAACCgcc
HvGSK3.1	P$AT5G46350_01	AT5G46350	2017	2026	1	1	0.851	ACCGCccat
HvGSK3.1	P$AT3G01030_01	AT3G01030	2017	2026	1	1	0.857	ACCGCccat
HvGSK3.1	P$AT3G63350_01	AT3G63350	2018	2024	1	1	1	CCGCCc
HvGSK3.1	P$ATMYB15_Q2	ATMYB15	2025	2031	1	1	0.865	TAACAg
HvGSK3.1	P$ATMYB77_01	ATMYB77	2038	2051	1	1	0.851	atcgatCAGTTag
HvGSK3.1	P$AT5G04340_01	AT5G04340	2045	2057	1	1	0.893	agttAGAAAaac
HvGSK3.1	P$BPC1_Q2	BPC1	2049	2055	1	1	0.997	AGAAAa
HvGSK3.1	P$PBF_01	BF	2096	2107	1	1	0.968	tgcAAAAGtgt
HvGSK3.1	P$DOF_Q2	DOF	2096	2107	1	1	0.921	tgcAAAAGtgt
HvGSK3.1	P$CDF2_01	CDF2	2097	2107	1	1	0.969	gcAAAAGtgt
HvGSK3.1	P$CDF3_01	CDF3	2098	2107	1	1	0.993	cAAAAGtgt
HvGSK3.1	P$PEND_01	END	2111	2119	1	1	0.859	gAAGAAtt

HvGSK3.1	P$ASR1_01	ASR1	2124	2129	1	1	1	ACCCA
HvGSK3.1	P$LFY_Q2	LFY	2134	2145	1	1	0.922	ttCCAATgtgg
HvGSK3.1	P$AT3G60580_01	AT3G60580	2143	2150	1	1	0.902	ggATCCC
HvGSK3.1	P$SPL15_01	SPL15	2154	2168	1	1	0.854	tagtaGTACGggat
HvGSK3.1	P$SPL11_01	SPL11	2155	2167	1	1	0.956	agtaGTACGgga
HvGSK3.1	P$SPL5_02	SPL5	2155	2167	1	1	0.868	agtaGTACGgga
HvGSK3.1	P$BHLH28_01	BHLH28	2155	2167	1	1	0.935	agtaGTACGgga
HvGSK3.1	P$SPL5_01	SPL5	2157	2166	1	1	0.998	taGTACGgg
HvGSK3.1	P$POPTR_01	OPTR	2158	2165	1	1	0.999	aGTACGg
HvGSK3.1	P$SPL12_01	SPL12	2158	2166	1	1	0.998	aGTACGgg
HvGSK3.1	P$SPL4_01	SPL4	2158	2167	1	1	0.999	aGTACGgga
HvGSK3.1	P$AT3G60580_01	AT3G60580	2164	2171	1	1	0.902	ggATCCC
HvGSK3.1	P$MYB1L_01	MYB1L	2166	2176	1	1	0.952	atCCCTAcga
HvGSK3.1	P$TRB2_01	TRB2	2166	2174	1	1	0.928	atCCCTAc
HvGSK3.1	P$HSFA2_01	HSFA2	2176	2182	1	1	0.933	CCAAAg
HvGSK3.1	P$ATHB6_01	ATHB6	2182	2191	1	1	0.899	agAATAAac
HvGSK3.1	P$AT3G20750_01	AT3G20750	2185	2193	1	1	0.888	aTAAACac
HvGSK3.1	P$ABF2_01	ABF2	2196	2209	1	1	0.951	gggaaCACGTctt
HvGSK3.1	P$O2_Q4	O2	2197	2208	1	1	0.862	ggaaCACGTct
HvGSK3.1	P$GBP_Q6	GBP	2198	2210	1	1	0.91	gaaCACGTcttc
HvGSK3.1	P$ABI5_01	ABI5	2198	2208	1	1	0.928	gaaCACGTct
HvGSK3.1	P$ABF4_01	ABF4	2198	2210	1	1	0.922	gaaCACGTcttc
HvGSK3.1	P$EMBP1_Q2	EMBP1	2199	2209	1	1	0.859	aaCACGTctt
HvGSK3.1	P$CPRF3_Q2	CPRF3	2199	2209	1	1	0.954	aaCACGTctt
HvGSK3.1	P$CPRF2_Q2	CPRF2	2199	2209	1	1	0.961	aaCACGTctt
HvGSK3.1	P$O2_02	O2	2199	2209	1	1	0.942	aaCACGTctt
HvGSK3.1	P$TGA1B_Q2	TGA1B	2199	2209	1	1	0.91	aaCACGTctt
HvGSK3.1	P$TGA1A_Q2	TGA1A	2199	2209	1	1	0.977	aaCACGTctt
HvGSK3.1	P$CPRF3_01	CPRF3	2199	2209	1	1	0.97	aaCACGTctt
HvGSK3.1	P$CPRF2_01	CPRF2	2199	2209	1	1	0.963	aaCACGTctt
HvGSK3.1	P$TGA1B_01	TGA1B	2199	2209	1	1	0.909	aaCACGTctt
HvGSK3.1	P$BEE2_01	BEE2	2199	2209	1	1	0.906	aaCACGTctt
HvGSK3.1	P$BIM3_01	BIM3	2199	2209	1	1	0.875	aaCACGTctt
HvGSK3.1	P$PHYPA143875_02	HYPA143875	2199	2209	1	1	0.871	aaCACGTctt
HvGSK3.1	P$SPT_01	SPT	2199	2208	1	1	0.917	aaCACGTct
HvGSK3.1	P$GBF1F_Q2	GBF1F	2199	2210	1	1	0.851	aaCACGTcttc
HvGSK3.1	P$ABF3_01	ABF3	2200	2208	1	1	0.875	ACACGtct
HvGSK3.1	P$RITA1_01	RITA1	2200	2207	1	1	0.963	aCACGTc
HvGSK3.1	P$OCSBF1_01	OCSBF1	2201	2206	1	1	1	CACGT
HvGSK3.1	P$TGA1A_01	TGA1A	2201	2208	1	1	0.871	cACGTCt
HvGSK3.1	P$ARR2_01	ARR2	2207	2217	1	1	0.854	ttcgATCTTt
HvGSK3.1	P$GATA11_01	GATA11	2208	2216	1	1	0.854	tcGATCTt
HvGSK3.1	P$GATA8_01	GATA8	2208	2217	1	1	0.979	tcGATCTtt
HvGSK3.1	P$MYB305_Q3	MYB305	2228	2241	1	1	0.905	gggatACCTAacc
HvGSK3.1	P$MYB61_01	MYB61	2230	2245	1	1	0.874	gatACCTAaccgtct
HvGSK3.1	P$MYBAS1_01	MYBAS1	2233	2244	1	1	0.99	acCTAACcgtc
HvGSK3.1	P$WRKY25_01	WRKY25	2233	2242	1	1	0.912	accTAACCg
HvGSK3.1	P$AT1G69310_01	AT1G69310	2233	2242	1	1	0.885	accTAACCg
HvGSK3.1	P$MYBAS1_01	MYBAS1	2248	2259	1	1	0.976	atCTAACatat
HvGSK3.1	P$RAV1_02	RAV1	2259	2271	1	1	0.909	gatACCTGcata
HvGSK3.1	P$MYBPH3_01	MYBPH3	2274	2287	1	1	0.864	gatacccGGTTag
HvGSK3.1	P$ATMYB77_01	ATMYB77	2274	2287	1	1	0.861	gatacccGGTTag
HvGSK3.1	P$ARR18_01	ARR18	2281	2294	1	1	0.888	ggttAGATAacat
HvGSK3.1	P$SPF1_Q2	SPF1	2290	2300	1	1	0.894	acATAGTgag
HvGSK3.1	P$HMG1_01	HMG1	2299	2308	1	1	0.975	GTTGTcatc
HvGSK3.1	P$PHV_02	HV	2302	2317	1	1	0.861	gtcATCATcaccaaa
HvGSK3.1	P$HSFA2_01	HSFA2	2312	2318	1	1	1	CCAAAa
HvGSK3.1	P$ARR18_01	ARR18	2343	2356	1	1	0.951	atttAGATAcatc
HvGSK3.1	P$AT3G60580_01	AT3G60580	2351	2358	1	1	0.852	acATCCC
HvGSK3.1	P$MYB1L_01	MYB1L	2353	2363	1	1	0.952	atCCCTAtgt
HvGSK3.1	P$TRB2_01	TRB2	2353	2361	1	1	0.928	atCCCTAt
HvGSK3.1	P$PEND_01	END	2393	2401	1	1	0.892	tAAGAAaa
HvGSK3.1	P$BPC1_Q2	BPC1	2395	2401	1	1	0.997	AGAAAa
HvGSK3.1	P$ATHB6_01	ATHB6	2397	2406	1	1	0.902	aaAATAAac
HvGSK3.1	P$AT3G20750_01	AT3G20750	2400	2408	1	1	0.888	aTAAACac
HvGSK3.1	P$AT2G15660_01	AT2G15660	2408	2419	1	1	0.958	TTCTCgttgaa
HvGSK3.1	P$SED_Q2	SED	2421	2431	1	1	0.98	tttaCCTTTt
HvGSK3.1	P$PBF_Q2_01	BF	2425	2431	1	1	1	CCTTTt
HvGSK3.1	P$HSFA2_01	HSFA2	2435	2441	1	1	0.922	CCAAAt
HvGSK3.1	P$AT3G49930_01	AT3G49930	2453	2467	1	1	0.864	tgctcataTATTGg
HvGSK3.1	P$GATA15_01	GATA15	2493	2502	1	1	0.999	gaTGATCcc
HvGSK3.1	P$AT3G60580_01	AT3G60580	2495	2502	1	1	0.852	tgATCCC
HvGSK3.1	P$CBNAC_01	CBNAC	2523	2529	1	1	0.968	aTGCTT
HvGSK3.1	P$CBNAC_02	CBNAC	2523	2539	1	1	0.864	aTGCTTgaaggtggat
HvGSK3.1	P$CBNAC_01	CBNAC	2541	2547	1	1	1	tTGCTT
HvGSK3.1	P$CBNAC_02	CBNAC	2541	2557	1	1	0.9	tTGCTTtcgtacggcg
HvGSK3.1	P$AT2G38090_01	AT2G38090	2543	2555	1	1	0.943	gctTTCGTacgg
HvGSK3.1	P$ATSPL3_01	ATSPL3	2543	2559	1	1	0.931	gctttCGTACggcggg

HvGSK3.1	P$SPL15_01	SPL15	2544	2558	1	1	0.862	ctttcGTACGgcgg
HvGSK3.1	P$SMZ_01	SMZ	2545	2553	1	1	0.876	ttTCGTAc
HvGSK3.1	P$SPL11_01	SPL11	2545	2557	1	1	0.978	tttcGTACGgcg
HvGSK3.1	P$SPL5_02	SPL5	2545	2557	1	1	0.872	tttcGTACGgcg
HvGSK3.1	P$BHLH28_01	BHLH28	2545	2557	1	1	0.948	tttcGTACGgcg
HvGSK3.1	P$RRTF1_05	RRTF1	2545	2560	1	1	0.868	tttcgtaCGGCGgga
HvGSK3.1	P$SPL1_01	SPL1	2546	2556	1	1	0.899	ttCGTACggc
HvGSK3.1	P$SPL14_03	SPL14	2546	2557	1	1	0.878	ttCGTACggcg
HvGSK3.1	P$SPL5_01	SPL5	2547	2556	1	1	0.999	tcGTACGgc
HvGSK3.1	P$POPTR_01	OPTR	2548	2555	1	1	1	cGTACGg
HvGSK3.1	P$SPL12_01	SPL12	2548	2556	1	1	0.999	cGTACGgc
HvGSK3.1	P$SPL4_01	SPL4	2548	2557	1	1	1	cGTACGgcg
HvGSK3.1	P$AT1G28160_02	AT1G28160	2549	2564	1	1	0.856	gtaCGGCGggaagat
HvGSK3.1	P$AT1G68550_03	AT1G68550	2549	2558	1	1	0.967	gtaCGGCGg
HvGSK3.1	P$E2L_Q2_01	E2L	2550	2564	1	1	0.887	tacggCGGGAagat
HvGSK3.1	P$HSFA1E_01	HSFA1E	2551	2557	1	1	0.87	aCGGCG
HvGSK3.1	P$E2L_Q2	E2L	2552	2559	1	1	0.928	cGGCGGg
HvGSK3.1	P$ARR18_01	ARR18	2556	2569	1	1	0.94	gggaAGATAagaa
HvGSK3.1	P$PEND_01	END	2563	2571	1	1	0.875	tAAGAAca
HvGSK3.1	P$PEND_01	END	2573	2581	1	1	0.854	cAAGAAct
HvGSK3.1	P$C1_Q2	C1	2575	2586	1	1	0.917	agAACTAaaat
HvGSK3.1	P$HSFA2_01	HSFA2	2592	2598	1	1	0.933	CCAAAg
HvGSK3.1	P$TGA1A_Q2_01	TGA1A	2596	2618	1	1	0.913	agaATGACatgatgaaccattt
HvGSK3.1	P$KNOX3_01	KNOX3	2596	2608	1	1	0.961	agaaTGACAtga
HvGSK3.1	P$ATH1_01	ATH1	2600	2608	1	1	0.946	TGACAtga
HvGSK3.1	P$AT4G36620_01	AT4G36620	2607	2615	1	1	0.98	atgAACCA
HvGSK3.1	P$SQUA_01	SQUA	2612	2622	1	1	0.9	ccaTTTTTcc
HvGSK3.1	P$GT1_Q6_01	GT1	2615	2627	1	1	0.857	TTTTTcctcatc
HvGSK3.1	P$AZF3_01	AZF3	2635	2646	1	1	0.934	aAGTATatctt
HvGSK3.1	P$ARR2_01	ARR2	2637	2647	1	1	0.887	gtatATCTTc
HvGSK3.1	P$MYB118_01	MYB118	2638	2655	1	1	0.902	tatatcttcGTTACttt
HvGSK3.1	P$WRKY18_02	WRKY18	2664	2674	1	1	0.948	actGTCAAgg
HvGSK3.1	P$WRKY21_02	WRKY21	2664	2674	1	1	0.95	actGTCAAgg
HvGSK3.1	P$WRKY48_02	WRKY48	2664	2674	1	1	0.985	actGTCAAgg
HvGSK3.1	P$WRKY57_01	WRKY57	2664	2674	1	1	0.957	actGTCAAgg
HvGSK3.1	P$WRKY60_01	WRKY60	2664	2675	1	1	0.895	actGTCAAgga
HvGSK3.1	P$WRKY15_01	WRKY15	2665	2675	1	1	0.959	ctGTCAAgga
HvGSK3.1	P$WRKY2_01	WRKY2	2665	2673	1	1	0.904	ctGTCAAg
HvGSK3.1	P$WRKY25_02	WRKY25	2665	2673	1	1	0.893	ctGTCAAg
HvGSK3.1	P$WRKY40_01	WRKY40	2665	2673	1	1	0.977	ctGTCAAg
HvGSK3.1	P$WRKY43_02	WRKY43	2665	2675	1	1	0.949	ctGTCAAgga
HvGSK3.1	P$WRKY63_01	WRKY63	2665	2673	1	1	0.889	ctGTCAAg
HvGSK3.1	P$WRKY75_01	WRKY75	2665	2673	1	1	0.919	ctGTCAAg
HvGSK3.1	P$WRKY8_01	WRKY8	2665	2674	1	1	0.977	ctGTCAAgg
HvGSK3.1	P$WRKY30_01	WRKY30	2666	2676	1	1	0.904	tGTCAAggaa
HvGSK3.1	P$WRKY18_Q2	WRKY18	2667	2676	1	1	0.949	GTCAAggaa
HvGSK3.1	P$MYB1L_01	MYB1L	2674	2684	1	1	1	aaCCCTAgtc
HvGSK3.1	P$TRB2_01	TRB2	2674	2682	1	1	0.971	aaCCCTAg
HvGSK3.1	P$TEIL_01	TEIL	2687	2695	1	1	0.931	ATGTAtgt
HvGSK3.1	P$PCF2_01	CF2	2693	2703	1	1	0.89	gtgacCCCAC
HvGSK3.1	P$TCP19_01	TCP19	2693	2703	1	1	0.909	gtgacCCCAC
HvGSK3.1	P$TCP20L_01	TCP20L	2694	2703	1	1	0.89	tgacCCCAC
HvGSK3.1	P$TCP20_02	TCP20	2695	2705	1	1	0.914	gacCCCACac
HvGSK3.1	P$ARALY495258_02	ARALY495258	2695	2703	1	1	0.956	gacCCCAC
HvGSK3.1	P$ARALY484486_05	ARALY484486	2695	2703	1	1	0.956	gacCCCAC
HvGSK3.1	P$MYBAS1_01	MYBAS1	2702	2713	1	1	0.981	caCCAACatat
HvGSK3.1	P$RAV1_01	RAV1	2702	2714	1	1	0.967	cacCAACAtata
HvGSK3.1	P$AT3G01030_01	AT3G01030	2717	2726	1	1	0.855	ACCGCatgc
HvGSK3.1	P$ABI3_01	ABI3	2718	2727	1	1	0.982	ccGCATGct
HvGSK3.1	P$FUS3_01	FUS3	2719	2728	1	1	0.972	cGCATGcta
HvGSK3.1	P$LEC2_01	LEC2	2719	2730	1	1	0.938	cgCATGCtata
HvGSK3.1	P$GAMYB_Q2	GAMYB	2735	2748	1	1	0.867	ggattACAACtgt
HvGSK3.1	P$ASR1_01	ASR1	2757	2762	1	1	1	ACCCA
HvGSK3.1	P$ARR2_01	ARR2	2767	2777	1	1	0.898	tccaATCTTg
HvGSK3.1	P$TGA1_01	TGA1	2772	2783	1	1	0.929	tctTGACGcag
HvGSK3.1	P$WRKY11_Q2	WRKY11	2773	2781	1	1	0.897	cTTGACgc
HvGSK3.1	P$TGA7_01	TGA7	2773	2783	1	1	0.855	ctTGACGcag
HvGSK3.1	P$TGA5_01	TGA5	2774	2782	1	1	0.87	tTGACGca
HvGSK3.1	P$AT1G55110_01	AT1G55110	2783	2795	1	1	0.856	attGGCGGcaca
HvGSK3.1	P$E2L_Q2	E2L	2785	2792	1	1	0.91	tGGCGGc
HvGSK3.1	P$ERF1_Q2	ERF1	2786	2794	1	1	0.957	GGCGGcac
HvGSK3.1	P$AT5G04240_01	AT5G04240	2788	2794	1	1	0.976	cGGCAC
HvGSK3.1	P$ASR1_01	ASR1	2808	2813	1	1	1	ACCCA
HvGSK3.1	P$MYBAS1_01	MYBAS1	2808	2819	1	1	0.981	acCCAACccgc
HvGSK3.1	P$GAMYB_01	GAMYB	2811	2819	1	1	0.93	CAACCcgc
HvGSK3.1	P$CMTA2_01	CMTA2	2812	2821	1	1	0.996	aaccCGCGT
HvGSK3.1	P$CAMTA1_02	CAMTA1	2812	2824	1	1	0.935	aaccCGCGTgcc
HvGSK3.1	P$CMTA3_01	CMTA3	2815	2824	1	1	1	cCGCGTgcc

HvGSK3.1	P$BZR1_01	BZR1	2818	2824	1	1	0.897	CGTGCc
HvGSK3.1	P$HAT1_01	HAT1	2825	2835	1	1	0.854	caAATCAcct
HvGSK3.1	P$ARR18_01	ARR18	2840	2853	1	1	0.895	taatAGATAgaac
HvGSK3.1	P$SBF1_01	SBF1	2852	2866	1	1	0.88	caatatTTAATtag
HvGSK3.1	P$EDT1_01	EDT1	2855	2865	1	1	0.892	tatTTAATta
HvGSK3.1	P$PHV_02	HV	2869	2884	1	1	0.855	tttATCATttcaaaa
HvGSK3.1	P$ATHB4_02	ATHB4	2870	2880	1	1	0.889	ttATCATttc
HvGSK3.1	P$MADSB_Q2	MADSB	2877	2892	1	1	0.879	ttcaAAAAAtgtatg
HvGSK3.1	P$TGA1A_Q2_01	TGA1A	2886	2908	1	1	0.92	tgtATGACaaacctaatttttc
HvGSK3.1	P$KNOX3_01	KNOX3	2886	2898	1	1	0.963	tgtaTGACAaac
HvGSK3.1	P$ATH1_01	ATH1	2890	2898	1	1	0.909	TGACAaac
HvGSK3.1	P$WRKY11_Q2	WRKY11	2927	2935	1	1	0.999	tTTGACca
HvGSK3.1	P$ZAP1_01	ZAP1	2928	2938	1	1	0.862	TTGACcatta
HvGSK3.1	P$WEREWOLF_Q2_01	WEREWOLF	2944	2953	1	1	0.897	tACTAActt
HvGSK3.1	P$MYBAS1_01	MYBAS1	2944	2955	1	1	0.977	taCTAACttac
HvGSK3.1	P$ABI3_01	ABI3	2956	2965	1	1	0.873	tgGCATGga
HvGSK3.1	P$KNOX3_01	KNOX3	2971	2983	1	1	0.964	tgaaTGACAtca
HvGSK3.1	P$ATH1_01	ATH1	2975	2983	1	1	0.941	TGACAtca
HvGSK3.1	P$PHV_02	HV	2976	2991	1	1	0.881	gacATCATtatattt
HvGSK3.1	P$AT3G51080_01	AT3G51080	2996	3003	1	1	1	GGAAAaa
HvGSK3.1	P$ARR2_01	ARR2	2998	3008	1	1	0.912	aaaaATCTTt
HvGSK3.1	P$O2_Q4	O2	3004	3015	1	1	0.984	ctttCATGTca
HvGSK3.1	P$O2_Q4	O2	3009	3020	1	1	0.884	atgtCATGTgt
HvGSK3.1	P$AMS_01	AMS	3011	3021	1	1	0.869	gtCATGTgta
HvGSK3.1	P$GT1_Q6_01	GT1	3021	3033	1	1	0.913	TTTTTacttatt
HvGSK3.1	P$ATHB7_01	ATHB7	3053	3063	1	1	0.914	aaAATCAtag
HvGSK3.1	P$HAT1_01	HAT1	3053	3063	1	1	0.97	aaAATCAtag
HvGSK3.1	P$SPF1_Q2	SPF1	3057	3067	1	1	0.919	tcATAGTcaa
HvGSK3.1	P$WRKY40_03	WRKY40	3059	3069	1	1	0.997	atAGTCAaag
HvGSK3.1	P$WRKY18_02	WRKY18	3059	3069	1	1	0.981	ataGTCAAag
HvGSK3.1	P$WRKY21_02	WRKY21	3059	3069	1	1	0.973	ataGTCAAag
HvGSK3.1	P$WRKY48_02	WRKY48	3059	3069	1	1	0.995	ataGTCAAag
HvGSK3.1	P$WRKY57_01	WRKY57	3059	3069	1	1	0.986	ataGTCAAag
HvGSK3.1	P$WRKY60_01	WRKY60	3059	3070	1	1	0.914	ataGTCAAagt
HvGSK3.1	P$WRKY15_01	WRKY15	3060	3070	1	1	0.982	taGTCAAagt
HvGSK3.1	P$WRKY2_01	WRKY2	3060	3068	1	1	0.94	taGTCAAa
HvGSK3.1	P$WRKY25_02	WRKY25	3060	3068	1	1	0.921	taGTCAAa
HvGSK3.1	P$WRKY40_01	WRKY40	3060	3068	1	1	0.996	taGTCAAa
HvGSK3.1	P$WRKY43_02	WRKY43	3060	3070	1	1	0.991	taGTCAAagt
HvGSK3.1	P$WRKY62_01	WRKY62	3060	3068	1	1	0.923	taGTCAAa
HvGSK3.1	P$WRKY63_01	WRKY63	3060	3068	1	1	0.905	taGTCAAa
HvGSK3.1	P$WRKY75_01	WRKY75	3060	3068	1	1	0.977	taGTCAAa
HvGSK3.1	P$WRKY8_01	WRKY8	3060	3069	1	1	0.992	taGTCAAag
HvGSK3.1	P$WRKY23_01	WRKY23	3061	3069	1	1	0.928	aGTCAAag
HvGSK3.1	P$WRKY30_01	WRKY30	3061	3071	1	1	0.922	aGTCAAagtc
HvGSK3.1	P$WRKY18_Q2	WRKY18	3062	3071	1	1	0.978	GTCAAagtc
HvGSK3.1	P$SBF1_01	SBF1	3068	3082	1	1	0.877	gtcgtaTTAAAaac
HvGSK3.1	P$ATHB7_01	ATHB7	3086	3096	1	1	0.874	aaAATCAagg
HvGSK3.1	P$HAT1_01	HAT1	3086	3096	1	1	0.87	aaAATCAagg
HvGSK3.1	P$ARR2_01	ARR2	3094	3104	1	1	0.943	gggcATCTTa
HvGSK3.1	P$MYB24_01	MYB24	3107	3116	1	1	0.862	tttTTAGGa
HvGSK3.1	P$PBF_01	BF	3114	3125	1	1	0.963	gacAAAAGact
HvGSK3.1	P$DOF_Q2	DOF	3114	3125	1	1	0.922	gacAAAAGact
HvGSK3.1	P$CDF2_01	CDF2	3115	3125	1	1	0.941	acAAAAGact
HvGSK3.1	P$CDF3_01	CDF3	3116	3125	1	1	0.965	cAAAAGact
HvGSK3.1	P$MYB24_01	MYB24	3131	3140	1	1	0.883	taaTTAGGg
HvGSK3.1	P$BZIP68_01	BZIP68	3140	3149	1	1	0.935	tctCGTGGa
HvGSK3.1	P$SED_Q2	SED	3158	3168	1	1	0.951	tcgaCCTTTt
HvGSK3.1	P$PBF_Q2_01	BF	3162	3168	1	1	1	CCTTTt
HvGSK3.1	P$EDT1_01	EDT1	3188	3198	1	1	0.945	aagTTAATga
HvGSK3.1	P$LIM1_01	LIM1	3218	3230	1	1	0.945	CCACCacaacta
HvGSK3.1	P$GAMYB_Q2	GAMYB	3218	3231	1	1	0.895	ccaccACAACtat
HvGSK3.1	P$C1_Q2	C1	3223	3234	1	1	0.937	acAACTAtggc
HvGSK3.1	P$MYB24_01	MYB24	3236	3245	1	1	0.939	tgtTTAGGt
HvGSK3.1	P$MYB131_01	MYB131	3236	3247	1	1	0.908	tgtTTAGGttc
HvGSK3.1	P$O2_Q4	O2	3254	3265	1	1	0.858	tgaaCATGTcc
HvGSK3.1	P$MYB1L_01	MYB1L	3261	3271	1	1	0.942	gtCCCTAacc
HvGSK3.1	P$TRB2_01	TRB2	3261	3269	1	1	0.935	gtCCCTAa
HvGSK3.1	P$MYBAS1_01	MYBAS1	3263	3274	1	1	0.957	ccCTAACcgtg
HvGSK3.1	P$WRKY25_01	WRKY25	3263	3272	1	1	0.886	cccTAACCg
HvGSK3.1	P$AT1G69310_01	AT1G69310	3263	3272	1	1	0.866	cccTAACCg
HvGSK3.1	P$O2_Q4	O2	3282	3293	1	1	0.868	acatCATGTct
HvGSK3.1	P$ATHSFA1D_01	ATHSFA1D	3293	3299	1	1	0.941	tCTACA
HvGSK3.1	P$HMG1_01	HMG1	3301	3310	1	1	0.975	GTTGTcatc
HvGSK3.1	P$HBP1B_Q6	HBP1B	3304	3318	1	1	0.897	gtcatctaCGTCAt
HvGSK3.1	P$HBPA1_Q6_01	HBPA1	3308	3318	1	1	0.876	tctaCGTCAt
HvGSK3.1	P$TGA1A_01	TGA1A	3310	3317	1	1	1	tACGTCa
HvGSK3.1	P$TGA2_Q2	TGA2	3311	3321	1	1	0.94	aCGTCAtcat

HvGSK3.1	P$PHV_02	HV	3313	3328	1	1	0.87	gtcATCATcataatg
HvGSK3.1	P$ATSPL8_01	ATSPL8	3360	3376	1	1	0.896	catatTGTACcggaag
HvGSK3.1	P$MYB89_01	MYB89	3365	3376	1	1	0.868	tgTACCGgaag
HvGSK3.1	P$SBF1_01	SBF1	3375	3389	1	1	0.853	ggcattTTAAAaat
HvGSK3.1	P$MYBAS1_01	MYBAS1	3391	3402	1	1	0.946	ccCCAACcatg
HvGSK3.1	P$AT4G36620_01	AT4G36620	3392	3400	1	1	0.885	cccAACCA
HvGSK3.1	P$GAMYB_01	GAMYB	3394	3402	1	1	0.878	CAACCatg
HvGSK3.1	P$HSFA4A_01	HSFA4A	3404	3410	1	1	0.91	gCTATT
HvGSK3.1	P$ATHSFA1D_01	ATHSFA1D	3421	3427	1	1	0.941	tCTACA
HvGSK3.1	P$SPF1_Q2	SPF1	3427	3437	1	1	0.883	ttATAGTtat
HvGSK3.1	P$ATHSFA1D_01	ATHSFA1D	3436	3442	1	1	0.941	tCTACA
HvGSK3.1	P$ARR1_01	ARR1	3466	3476	1	1	0.953	acaGAATCag
HvGSK3.1	P$HAT1_01	HAT1	3468	3478	1	1	0.852	agAATCAgag
HvGSK3.1	P$REF6_01	REF6	3469	3480	1	1	0.854	gaatCAGAGcc
HvGSK3.1	P$O2_04	O2	3475	3488	1	1	0.874	gaGCCACttcata
HvGSK3.1	P$O2_Q4	O2	3484	3495	1	1	0.934	cataCATGTcg
HvGSK3.1	P$ARF8_01	ARF8	3488	3497	1	1	0.952	caTGTCGta
HvGSK3.1	P$ATSPL3_01	ATSPL3	3488	3504	1	1	0.94	catgtCGTACtcggga
HvGSK3.1	P$SMZ_01	SMZ	3490	3498	1	1	0.896	tgTCGTAc
HvGSK3.1	P$GT1_Q6	GT1	3517	3524	1	1	1	GTGAAta
HvGSK3.1	P$ATHB6_01	ATHB6	3518	3527	1	1	0.894	tgAATAAcc
HvGSK3.1	P$MYB3R5_01	MYB3R5	3530	3545	1	1	0.944	ccaatttcaCCGTTt
HvGSK3.1	P$MYB3R1_01	MYB3R1	3531	3546	1	1	0.954	caatttcaCCGTTtt
HvGSK3.1	P$MYB3R4_01	MYB3R4	3531	3546	1	1	0.95	caatttcaCCGTTtt
HvGSK3.1	P$SQUA_01	SQUA	3539	3549	1	1	0.875	ccgTTTTTac
HvGSK3.1	P$ATHSFA1D_01	ATHSFA1D	3547	3553	1	1	1	aCTACA
HvGSK3.1	P$AT4G01720_01	AT4G01720	3559	3568	1	1	0.913	gGTAAAatc
HvGSK3.1	P$PEND_02	END	3568	3578	1	1	0.856	tgTTCTTctg
HvGSK3.1	P$SPF1_Q2	SPF1	3578	3588	1	1	0.924	ttATAGTata
HvGSK3.1	P$ATSPL8_01	ATSPL8	3592	3608	1	1	0.912	gatcgTGTACtttgac
HvGSK3.1	P$WRKY11_01	WRKY11	3599	3613	1	1	0.889	tactTTGACttatt
HvGSK3.1	P$WRKY11_Q2	WRKY11	3602	3610	1	1	0.931	tTTGACtt
HvGSK3.1	P$HSFA2_01	HSFA2	3631	3637	1	1	0.941	CCAAAc
HvGSK3.1	P$ESR1_01	ESR1	3651	3661	1	1	0.895	aGGCCGgcct
HvGSK3.1	P$ERF3_01	ERF3	3651	3661	1	1	0.945	aGGCCGgcct
HvGSK3.1	P$ERF4_01	ERF4	3651	3661	1	1	0.892	aGGCCGgcct
HvGSK3.1	P$ERF3_02	ERF3	3651	3661	1	1	0.935	aGGCCGgcct
HvGSK3.1	P$ERF2_04	ERF2	3651	3661	1	1	0.943	aGGCCGgcct
HvGSK3.1	P$ERF3_03	ERF3	3651	3661	1	1	0.943	aGGCCGgcct
HvGSK3.1	P$ATERF12_01	ATERF12	3651	3661	1	1	0.916	aGGCCGgcct
HvGSK3.1	P$AT1G24590_01	AT1G24590	3651	3661	1	1	0.888	aGGCCGgcct
HvGSK3.1	P$ATERF11_01	ATERF11	3651	3661	1	1	0.9	aGGCCGgcct
HvGSK3.1	P$ATERF9_01	ATERF9	3651	3661	1	1	0.884	aGGCCGgcct
HvGSK3.1	P$AT1G44830_01	AT1G44830	3651	3661	1	1	0.957	aGGCCGgcct
HvGSK3.1	P$ATERF8_01	ATERF8	3651	3661	1	1	0.89	aGGCCGgcct
HvGSK3.1	P$AT1G53910_01	AT1G53910	3651	3661	1	1	0.938	aGGCCGgcct
HvGSK3.1	P$ERF6_02	ERF6	3651	3661	1	1	0.981	agGCCGGcct
HvGSK3.1	P$ERF105_02	ERF105	3652	3660	1	1	0.944	gGCCGGcc
HvGSK3.1	P$CBF1_02	CBF1	3664	3674	1	1	0.85	agcCCGCCga
HvGSK3.1	P$ERF4_05	ERF4	3664	3679	1	1	0.883	agccCGCCGacacga
HvGSK3.1	P$CBF3_02	CBF3	3665	3679	1	1	0.972	gcccgCCGACacga
HvGSK3.1	P$RRTF1_02	RRTF1	3666	3676	1	1	0.874	ccCGCCGaca
HvGSK3.1	P$ERF112_02	ERF112	3666	3676	1	1	0.985	ccCGCCGaca
HvGSK3.1	P$CBF1_04	CBF1	3666	3678	1	1	0.979	cccgCCGACacg
HvGSK3.1	P$AT3G63350_01	AT3G63350	3667	3673	1	1	0.866	CCGCCg
HvGSK3.1	P$CRF4_01	CRF4	3667	3675	1	1	0.932	cCGCCGac
HvGSK3.1	P$ERF4_04	ERF4	3667	3675	1	1	0.957	cCGCCGac
HvGSK3.1	P$ERF069_01	ERF069	3667	3676	1	1	0.992	cCGCCGaca
HvGSK3.1	P$ERF11_01	ERF11	3667	3677	1	1	0.995	cCGCCGacac
HvGSK3.1	P$ERF5_02	ERF5	3667	3677	1	1	0.931	ccGCCGAcac
HvGSK3.1	P$ERF1_04	ERF1	3667	3677	1	1	0.918	ccGCCGAcac
HvGSK3.1	P$DREB1G_02	DREB1G	3667	3677	1	1	0.989	ccgCCGACac
HvGSK3.1	P$AT1G77200_03	AT1G77200	3667	3681	1	1	0.949	ccgCCGACacgacc
HvGSK3.1	P$ERF8_01	ERF8	3668	3678	1	1	0.989	CGCCGacacg
HvGSK3.1	P$ERF3_04	ERF3	3668	3676	1	1	0.966	CGCCGaca
HvGSK3.1	P$ARF1_01	ARF1	3669	3677	1	1	0.957	gCCGACac
HvGSK3.1	P$ARF5_01	ARF5	3669	3677	1	1	0.99	gCCGACac
HvGSK3.1	P$DREB1B_01	DREB1B	3670	3675	1	1	1	CCGAC
HvGSK3.1	P$HBPA1_Q6_01	HBPA1	3677	3687	1	1	0.878	gaccCGTCAt
HvGSK3.1	P$AT5G04240_01	AT5G04240	3692	3698	1	1	0.939	aGGCAC
HvGSK3.1	P$OSRR22_01	OSRR22	3696	3706	1	1	0.864	acGATACgac
HvGSK3.1	P$AT3G20750_01	AT3G20750	3706	3714	1	1	0.857	tTAAACag
HvGSK3.1	P$LEC2_01	LEC2	3715	3726	1	1	0.958	ccCATGCgaga
HvGSK3.1	P$ABF2_01	ABF2	3721	3734	1	1	0.964	cgagaCACGTagc
HvGSK3.1	P$O2_Q4	O2	3722	3733	1	1	0.872	gagaCACGTag
HvGSK3.1	P$GBP_Q6	GBP	3723	3735	1	1	0.962	agaCACGTagcc
HvGSK3.1	P$GBF_Q2	GBF	3723	3735	1	1	0.855	agaCACGTagcc
HvGSK3.1	P$ABI5_01	ABI5	3723	3733	1	1	0.963	agaCACGTag

HvGSK3.1	P$ABF4_01	ABF4	3723	3735	1	1	0.935	agaCACGTagcc
HvGSK3.1	P$EMBP1_Q2	EMBP1	3724	3734	1	1	0.92	gaCACGTagc
HvGSK3.1	P$CPRF3_Q2	CPRF3	3724	3734	1	1	0.931	gaCACGTagc
HvGSK3.1	P$CPRF2_Q2	CPRF2	3724	3734	1	1	0.947	gaCACGTagc
HvGSK3.1	P$O2_02	O2	3724	3734	1	1	0.957	gaCACGTagc
HvGSK3.1	P$TGA1B_Q2	TGA1B	3724	3734	1	1	0.948	gaCACGTagc
HvGSK3.1	P$TGA1A_Q2	TGA1A	3724	3734	1	1	0.968	gaCACGTagc
HvGSK3.1	P$CPRF1_01	CPRF1	3724	3734	1	1	0.853	gaCACGTagc
HvGSK3.1	P$CPRF3_01	CPRF3	3724	3734	1	1	0.931	gaCACGTagc
HvGSK3.1	P$CPRF2_01	CPRF2	3724	3734	1	1	0.947	gaCACGTagc
HvGSK3.1	P$TGA1B_01	TGA1B	3724	3734	1	1	0.937	gaCACGTagc
HvGSK3.1	P$BEE2_01	BEE2	3724	3734	1	1	0.915	gaCACGTagc
HvGSK3.1	P$BIM2_01	BIM2	3724	3734	1	1	0.852	gaCACGTagc
HvGSK3.1	P$BIM3_01	BIM3	3724	3734	1	1	0.887	gaCACGTagc
HvGSK3.1	P$PHYPA143875_02	HYPA143875	3724	3734	1	1	0.875	gaCACGTagc
HvGSK3.1	P$SPT_01	SPT	3724	3733	1	1	0.914	gaCACGTag
HvGSK3.1	P$GBF1F_Q2	GBF1F	3724	3735	1	1	0.898	gaCACGTagcc
HvGSK3.1	P$RITA1_01	RITA1	3725	3732	1	1	0.974	aCACGTa
HvGSK3.1	P$OCSBF1_01	OCSBF1	3726	3731	1	1	1	CACGT
HvGSK3.1	P$AT3G63350_01	AT3G63350	3734	3740	1	1	0.882	CCGCCa
HvGSK3.1	P$BZR1_01	BZR1	3745	3751	1	1	0.897	CGTGCc
HvGSK3.1	P$ABI3_01	ABI3	3765	3774	1	1	0.953	ggGCATGct
HvGSK3.1	P$FUS3_01	FUS3	3766	3775	1	1	0.944	gGCATGctg
HvGSK3.1	P$LEC2_01	LEC2	3766	3777	1	1	0.94	ggCATGCtgac
HvGSK3.1	P$CBF3_02	CBF3	3771	3785	1	1	0.973	gctgaCCGACacga
HvGSK3.1	P$CBF1_04	CBF1	3772	3784	1	1	0.963	ctgaCCGACacg
HvGSK3.1	P$DREB1G_02	DREB1G	3773	3783	1	1	0.913	tgaCCGACac
HvGSK3.1	P$AT1G77200_03	AT1G77200	3773	3787	1	1	0.942	tgaCCGACacgaca
HvGSK3.1	P$ARF1_01	ARF1	3775	3783	1	1	1	aCCGACac
HvGSK3.1	P$ARF5_01	ARF5	3775	3783	1	1	0.985	aCCGACac
HvGSK3.1	P$DREB1B_01	DREB1B	3776	3781	1	1	1	CCGAC
HvGSK3.1	P$NAC92_01	NAC92	3777	3789	1	1	0.99	cgACACGacacg
HvGSK3.1	P$MYB3R1_01	MYB3R1	3783	3798	1	1	0.854	gacacggtCCGTTtt
HvGSK3.1	P$SQUA_01	SQUA	3791	3801	1	1	0.889	ccgTTTTTat
HvGSK3.1	P$GT1_Q6_01	GT1	3801	3813	1	1	0.962	TTTTTatttaca
HvGSK3.1	P$GT1_Q6_01	GT1	3815	3827	1	1	0.881	TTTGTatatata
HvGSK3.1	P$GAMYB_Q2	GAMYB	3823	3836	1	1	0.887	tatatACAACata
HvGSK3.1	P$RAV1_01	RAV1	3826	3838	1	1	0.959	ataCAACAtata
HvGSK3.1	P$ATHB6_01	ATHB6	3837	3846	1	1	0.973	aaAATAAtc
HvGSK3.1	P$ATHB5_04	ATHB5	3837	3848	1	1	0.886	aaAATAAtcta
HvGSK3.1	P$ATHB1_03	ATHB1	3837	3848	1	1	0.894	aaAATAAtcta
HvGSK3.1	P$ATHB16_01	ATHB16	3838	3846	1	1	0.86	aAATAAtc
HvGSK3.1	P$ATHB6_01	ATHB6	3845	3854	1	1	0.914	ctAATAAat
HvGSK3.1	P$ATHB7_01	ATHB7	3849	3859	1	1	0.875	taAATCAaaa
HvGSK3.1	P$HAT1_01	HAT1	3849	3859	1	1	0.865	taAATCAaaa
HvGSK3.1	P$HSFA2_01	HSFA2	3863	3869	1	1	1	CCAAAa
HvGSK3.1	P$C1_Q2	C1	3866	3877	1	1	0.936	aaAACTAacta
HvGSK3.1	P$WEREWOLF_Q2_01	WEREWOLF	3868	3877	1	1	0.928	aACTAActa
HvGSK3.1	P$MYBAS1_01	MYBAS1	3868	3879	1	1	0.949	aaCTAACtaaa
HvGSK3.1	P$C1_Q2	C1	3870	3881	1	1	0.968	ctAACTAaaag
HvGSK3.1	P$PBF_01	BF	3873	3884	1	1	0.966	actAAAAGcag
HvGSK3.1	P$DOF_Q2	DOF	3873	3884	1	1	0.951	actAAAAGcag
HvGSK3.1	P$DOF2_01	DOF2	3873	3884	1	1	0.994	actaAAAGCag
HvGSK3.1	P$DOF3_01	DOF3	3873	3884	1	1	0.986	actaAAAGCag
HvGSK3.1	P$CDF2_01	CDF2	3874	3884	1	1	0.961	ctAAAAGcag
HvGSK3.1	P$CDF3_01	CDF3	3875	3884	1	1	0.978	tAAAAGcag
HvGSK3.1	P$RRTF1_05	RRTF1	3899	3914	1	1	0.912	agcgtgtCGGCGgat
HvGSK3.1	P$ARF8_01	ARF8	3901	3910	1	1	0.994	cgTGTCGgc
HvGSK3.1	P$DREB1A_04	DREB1A	3902	3912	1	1	0.982	gtGTCGGcgg
HvGSK3.1	P$ERF039_01	ERF039	3902	3912	1	1	0.99	gtGTCGGcgg
HvGSK3.1	P$PHYPA182268_05	HYPA182268	3902	3912	1	1	0.888	gtGTCGGcgg
HvGSK3.1	P$ERF043_01	ERF043	3903	3911	1	1	0.953	tGTCGGcg
HvGSK3.1	P$PHYPA173530_04	HYPA173530	3903	3911	1	1	0.918	tGTCGGcg
HvGSK3.1	P$PHYPA28324_10	HYPA28324	3903	3911	1	1	0.94	tGTCGGcg
HvGSK3.1	P$AT1G28160_02	AT1G28160	3903	3918	1	1	0.876	tgtCGGCGgatcagc
HvGSK3.1	P$RAP26_06	RAP26	3903	3918	1	1	0.872	tgtCGGCGgatcagc
HvGSK3.1	P$AT1G68550_03	AT1G68550	3903	3912	1	1	0.959	tgtCGGCGg
HvGSK3.1	P$ERF1_Q2	ERF1	3907	3915	1	1	0.892	GGCGGatc
HvGSK3.1	P$MYB3R1_01	MYB3R1	3910	3925	1	1	0.869	ggatcagcCCGTTta
HvGSK3.1	P$MYB3R4_01	MYB3R4	3910	3925	1	1	0.857	ggatcagcCCGTTta
HvGSK3.1	P$BZR1_01	BZR1	3930	3936	1	1	0.897	CGTGCc
HvGSK3.1	P$ERF019_01	ERF019	3931	3941	1	1	0.855	gTGCCGtgct
HvGSK3.1	P$CBF1_03	CBF1	3931	3941	1	1	0.865	gTGCCGtgct
HvGSK3.1	P$ANAC042_01	ANAC042	3932	3952	1	1	0.96	tGCCGTgcttgggccgggag
HvGSK3.1	P$ANAC094_01	ANAC094	3932	3950	1	1	0.924	tGCCGTgcttgggccggg
HvGSK3.1	P$BZR1_01	BZR1	3935	3941	1	1	0.902	CGTGCt
HvGSK3.1	P$CBNAC_01	CBNAC	3936	3942	1	1	0.979	gTGCTT
HvGSK3.1	P$CBNAC_02	CBNAC	3936	3952	1	1	0.894	gTGCTTgggccgggag

HvGSK3.1	P$HMG1_01	HMG1	3952	3961	1	1	0.865	GTTGTgcac
HvGSK3.1	P$CBF3_02	CBF3	3965	3979	1	1	0.941	ccagcCCGACatgg
HvGSK3.1	P$CBF1_04	CBF1	3966	3978	1	1	0.935	cagcCCGACatg
HvGSK3.1	P$DREB1G_02	DREB1G	3967	3977	1	1	0.895	agcCCGACat
HvGSK3.1	P$AT1G77200_03	AT1G77200	3967	3981	1	1	0.866	agcCCGACatggcc
HvGSK3.1	P$ARF1_01	ARF1	3969	3977	1	1	0.931	cCCGACat
HvGSK3.1	P$ARF5_01	ARF5	3969	3977	1	1	0.983	cCCGACat
HvGSK3.1	P$DREB1B_01	DREB1B	3970	3975	1	1	1	CCGAC
HvGSK3.1	P$BZR1_01	BZR1	3992	3998	1	1	0.897	CGTGCc
HvGSK4.1	P$O2_Q4	O2	3	14	1	1	0.87	atcaCATGTgg
HvGSK4.1	P$AMS_01	AMS	5	15	1	1	0.885	caCATGTggt
HvGSK4.1	P$O2_Q4	O2	12	23	1	1	0.861	ggtaCATGTta
HvGSK4.1	P$WEREWOLF_Q2	WEREWOLF	17	26	1	1	0.96	atGTTAGta
HvGSK4.1	P$WRKY18_02	WRKY18	27	37	1	1	0.998	atgGTCAAcc
HvGSK4.1	P$WRKY21_02	WRKY21	27	37	1	1	0.996	atgGTCAAcc
HvGSK4.1	P$WRKY48_02	WRKY48	27	37	1	1	1	atgGTCAAcc
HvGSK4.1	P$WRKY57_01	WRKY57	27	37	1	1	0.986	atgGTCAAcc
HvGSK4.1	P$WRKY60_01	WRKY60	27	38	1	1	0.996	atgGTCAAccg
HvGSK4.1	P$WRKY15_01	WRKY15	28	38	1	1	0.998	tgGTCAAccg
HvGSK4.1	P$WRKY2_01	WRKY2	28	36	1	1	0.998	tgGTCAAc
HvGSK4.1	P$WRKY25_02	WRKY25	28	36	1	1	0.994	tgGTCAAc
HvGSK4.1	P$WRKY40_01	WRKY40	28	36	1	1	1	tgGTCAAc
HvGSK4.1	P$WRKY43_02	WRKY43	28	38	1	1	0.983	tgGTCAAccg
HvGSK4.1	P$WRKY62_01	WRKY62	28	36	1	1	1	tgGTCAAc
HvGSK4.1	P$WRKY63_01	WRKY63	28	36	1	1	0.999	tgGTCAAc
HvGSK4.1	P$WRKY75_01	WRKY75	28	36	1	1	0.998	tgGTCAAc
HvGSK4.1	P$WRKY8_01	WRKY8	28	37	1	1	0.999	tgGTCAAcc
HvGSK4.1	P$WRKY23_01	WRKY23	29	37	1	1	0.925	gGTCAAcc
HvGSK4.1	P$WRKY30_01	WRKY30	29	39	1	1	0.994	gGTCAAccga
HvGSK4.1	P$WRKY18_Q2	WRKY18	30	39	1	1	0.927	GTCAAccga
HvGSK4.1	P$GAMYB_01	GAMYB	32	40	1	1	0.926	CAACCgat
HvGSK4.1	P$EDT1_01	EDT1	36	46	1	1	0.949	cgaTTAATgt
HvGSK4.1	P$ATSPL8_01	ATSPL8	38	54	1	1	0.929	attaaTGTACggtcat
HvGSK4.1	P$SPL15_01	SPL15	39	53	1	1	0.911	ttaatGTACGgtca
HvGSK4.1	P$SPL11_01	SPL11	40	52	1	1	0.965	taatGTACGgtc
HvGSK4.1	P$SPL5_02	SPL5	40	52	1	1	0.906	taatGTACGgtc
HvGSK4.1	P$BHLH28_01	BHLH28	40	52	1	1	0.961	taatGTACGgtc
HvGSK4.1	P$SPL5_01	SPL5	42	51	1	1	0.998	atGTACGgt
HvGSK4.1	P$POPTR_01	OPTR	43	50	1	1	0.999	tGTACGg
HvGSK4.1	P$SPL12_01	SPL12	43	51	1	1	1	tGTACGgt
HvGSK4.1	P$SPL4_01	SPL4	43	52	1	1	0.999	tGTACGgtc
HvGSK4.1	P$AT3G60580_01	AT3G60580	50	57	1	1	0.851	tcATCCC
HvGSK4.1	P$SED_Q2	SED	55	65	1	1	0.937	cccaCCTTTg
HvGSK4.1	P$PBF_Q2_01	BF	59	65	1	1	0.988	CCTTTg
HvGSK4.1	P$PBF_01	BF	80	91	1	1	0.969	gatAAAAGgaa
HvGSK4.1	P$DOF_Q2	DOF	80	91	1	1	0.949	gatAAAAGgaa
HvGSK4.1	P$CDF2_01	CDF2	81	91	1	1	0.956	atAAAAGgaa
HvGSK4.1	P$CDF3_01	CDF3	82	91	1	1	0.973	tAAAAGgaa
HvGSK4.1	P$PBF_Q2	BF	83	89	1	1	1	aAAAGG
HvGSK4.1	P$PBF_01	BF	95	106	1	1	0.976	aaaAAAAGaac
HvGSK4.1	P$DOF_Q2	DOF	95	106	1	1	0.999	aaaAAAAGaac
HvGSK4.1	P$CDF2_01	CDF2	96	106	1	1	0.978	aaAAAAGaac
HvGSK4.1	P$CDF3_01	CDF3	97	106	1	1	0.975	aAAAAGaac
HvGSK4.1	P$HMG1_01	HMG1	118	127	1	1	0.896	GTTGTggtg
HvGSK4.1	P$ATSPL3_01	ATSPL3	171	187	1	1	0.952	cactaCGTACcgtctc
HvGSK4.1	P$SPL14_03	SPL14	174	185	1	1	0.877	taCGTACcgtc
HvGSK4.1	P$MYB89_01	MYB89	176	187	1	1	0.851	cgTACCGtctc
HvGSK4.1	P$ABF2_01	ABF2	183	196	1	1	0.901	tctccCACGTcct
HvGSK4.1	P$O2_Q4	O2	184	195	1	1	0.927	ctccCACGTcc
HvGSK4.1	P$GBP_Q6	GBP	185	197	1	1	0.91	tccCACGTcctc
HvGSK4.1	P$ABI5_01	ABI5	185	195	1	1	0.906	tccCACGTcc
HvGSK4.1	P$ABF4_01	ABF4	185	197	1	1	0.881	tccCACGTcctc
HvGSK4.1	P$EMBP1_Q2	EMBP1	186	196	1	1	0.876	ccCACGTcct
HvGSK4.1	P$CPRF3_Q2	CPRF3	186	196	1	1	0.949	ccCACGTcct
HvGSK4.1	P$CPRF2_Q2	CPRF2	186	196	1	1	0.955	ccCACGTcct
HvGSK4.1	P$O2_02	O2	186	196	1	1	0.961	ccCACGTcct
HvGSK4.1	P$TGA1B_Q2	TGA1B	186	196	1	1	0.914	ccCACGTcct
HvGSK4.1	P$TGA1A_Q2	TGA1A	186	196	1	1	0.979	ccCACGTcct
HvGSK4.1	P$CPRF3_01	CPRF3	186	196	1	1	0.96	ccCACGTcct
HvGSK4.1	P$CPRF2_01	CPRF2	186	196	1	1	0.956	ccCACGTcct
HvGSK4.1	P$TGA1B_01	TGA1B	186	196	1	1	0.882	ccCACGTcct
HvGSK4.1	P$BEE2_01	BEE2	186	196	1	1	0.907	ccCACGTcct
HvGSK4.1	P$BIM3_01	BIM3	186	196	1	1	0.879	ccCACGTcct
HvGSK4.1	P$PHYPA143875_02	HYPA143875	186	196	1	1	0.873	ccCACGTcct
HvGSK4.1	P$SPT_01	SPT	186	195	1	1	0.948	ccCACGTcc
HvGSK4.1	P$GBF1F_Q2	GBF1F	186	197	1	1	0.929	ccCACGTcctc
HvGSK4.1	P$RITA1_01	RITA1	187	194	1	1	0.976	cCACGTc
HvGSK4.1	P$OCSBF1_01	OCSBF1	188	193	1	1	1	CACGT

HvGSK4.1	P$TGA1A_01	TGA1A	188	195	1	1	0.861	cACGTCc
HvGSK4.1	P$GL15_01	GL15	200	210	1	1	0.988	cgtgtCCCCC
HvGSK4.1	P$MYBAS1_01	MYBAS1	236	247	1	1	0.994	tcCCAACcgcc
HvGSK4.1	P$AT2G33710_01	AT2G33710	236	251	1	1	0.877	tcccaacCGCCGtcg
HvGSK4.1	P$GAMYB_01	GAMYB	239	247	1	1	1	CAACCgcc
HvGSK4.1	P$ERF4_05	ERF4	239	254	1	1	0.892	caacCGCCGtcgggg
HvGSK4.1	P$ERF112_02	ERF112	241	251	1	1	0.98	acCGCCGtcg
HvGSK4.1	P$AT3G63350_01	AT3G63350	242	248	1	1	0.866	CCGCCg
HvGSK4.1	P$CRF4_01	CRF4	242	250	1	1	0.936	cCGCCGtc
HvGSK4.1	P$ERF4_04	ERF4	242	250	1	1	0.964	cCGCCGtc
HvGSK4.1	P$ERF069_01	ERF069	242	251	1	1	0.994	cCGCCGtcg
HvGSK4.1	P$ERF11_01	ERF11	242	252	1	1	0.989	cCGCCGtcgg
HvGSK4.1	P$ERF8_01	ERF8	243	253	1	1	0.985	CGCCGtcggg
HvGSK4.1	P$ERF3_04	ERF3	243	251	1	1	0.952	CGCCGtcg
HvGSK4.1	P$DREB1A_04	DREB1A	245	255	1	1	0.929	ccGTCGGgga
HvGSK4.1	P$ERF039_01	ERF039	245	255	1	1	0.948	ccGTCGGgga
HvGSK4.1	P$PHYPA182268_05	HYPA182268	245	255	1	1	0.868	ccGTCGGgga
HvGSK4.1	P$PHYPA173530_04	HYPA173530	246	254	1	1	0.858	cGTCGGgg
HvGSK4.1	P$PHYPA28324_10	HYPA28324	246	254	1	1	0.897	cGTCGGgg
HvGSK4.1	P$HSF3_01	HSF3	248	254	1	1	0.94	tCGGGG
HvGSK4.1	P$ARF8_01	ARF8	254	263	1	1	0.95	agTGTCGtc
HvGSK4.1	P$DREB1A_04	DREB1A	258	268	1	1	0.931	tcGTCGGgca
HvGSK4.1	P$ERF039_01	ERF039	258	268	1	1	0.946	tcGTCGGgca
HvGSK4.1	P$DOF2_01	DOF2	263	274	1	1	0.979	gggcAAAGCgc
HvGSK4.1	P$DOF3_01	DOF3	263	274	1	1	0.985	gggcAAAGCgc
HvGSK4.1	P$RRTF1_05	RRTF1	276	291	1	1	0.882	tcggcatCGGCGgtg
HvGSK4.1	P$AT1G28160_02	AT1G28160	280	295	1	1	0.915	catCGGCGgtggcag
HvGSK4.1	P$RAP26_06	RAP26	280	295	1	1	0.904	catCGGCGgtggcag
HvGSK4.1	P$AT1G68550_03	AT1G68550	280	289	1	1	0.955	catCGGCGg
HvGSK4.1	P$RAP21_02	RAP21	282	295	1	1	0.942	tcggCGGTGgcag
HvGSK4.1	P$ERF1_Q2	ERF1	284	292	1	1	0.861	GGCGGtgg
HvGSK4.1	P$RAV1_02	RAV1	317	329	1	1	0.909	aggACCTGgcgc
HvGSK4.1	P$AT1G53910_02	AT1G53910	320	341	1	1	0.939	acctggcgcggGCGGCggggc
HvGSK4.1	P$RRTF1_05	RRTF1	325	340	1	1	0.899	gcgcgggCGGCGggg
HvGSK4.1	P$AT1G28160_02	AT1G28160	329	344	1	1	0.948	gggCGGCGgggcgtc
HvGSK4.1	P$RAP26_06	RAP26	329	344	1	1	0.913	gggCGGCGgggcgtc
HvGSK4.1	P$AT1G68550_03	AT1G68550	329	338	1	1	0.999	gggCGGCGg
HvGSK4.1	P$ERF1_Q2	ERF1	330	338	1	1	0.951	GGCGGcgg
HvGSK4.1	P$E2L_Q2	E2L	332	339	1	1	0.928	cGGCGGg
HvGSK4.1	P$HSF3_01	HSF3	334	340	1	1	0.945	gCGGGG
HvGSK4.1	P$BZIP68_01	BZIP68	340	349	1	1	0.935	cgtCGTGGa
HvGSK4.1	P$UIF1_01	UIF1	347	357	1	1	0.856	gagGATTCag
HvGSK4.1	P$PIL5_01	IL5	363	377	1	1	0.869	acgaagggACGTGa
HvGSK4.1	P$O2_Q2	O2	365	378	1	1	0.874	gaaggGACGTgaa
HvGSK4.1	P$ABZ1_01	ABZ1	366	380	1	1	0.868	aagggACGTGaatc
HvGSK4.1	P$TGA1B_01	TGA1B	368	378	1	1	0.928	ggGACGTgaa
HvGSK4.1	P$HBP1A_Q2	HBP1A	368	378	1	1	0.903	gggACGTGaa
HvGSK4.1	P$TAF1_Q2	TAF1	368	378	1	1	0.937	gggACGTGaa
HvGSK4.1	P$EMBP1_02	EMBP1	368	378	1	1	0.868	gggACGTGaa
HvGSK4.1	P$TAF1_01	TAF1	368	378	1	1	0.952	gggACGTGaa
HvGSK4.1	P$TRAB1_Q2	TRAB1	369	380	1	1	0.872	ggACGTGaatc
HvGSK4.1	P$GBF1_01	GBF1	369	377	1	1	0.92	ggACGTGa
HvGSK4.1	P$BIM1_02	BIM1	369	379	1	1	0.947	ggACGTGaat
HvGSK4.1	P$ABF4_02	ABF4	369	379	1	1	0.948	ggACGTGaat
HvGSK4.1	P$ABI5_Q2	ABI5	371	377	1	1	0.936	ACGTGa
HvGSK4.1	P$ARR1_01	ARR1	372	382	1	1	0.943	cgtGAATCcc
HvGSK4.1	P$AT3G60580_01	AT3G60580	375	382	1	1	0.932	gaATCCC
HvGSK4.1	P$PIL5_01	IL5	384	398	1	1	0.989	tggtggtcACGTGg
HvGSK4.1	P$PIF3_01	IF3	385	403	1	1	0.918	ggtggtCACGTGggggggc
HvGSK4.1	P$PIF3_02	IF3	386	403	1	1	0.911	gtggtCACGTGgggggc
HvGSK4.1	P$HY5_02	HY5	386	402	1	1	0.869	gtggtCACGTGgggggg
HvGSK4.1	P$ABF2_01	ABF2	386	399	1	1	0.939	gtggtCACGTGggg
HvGSK4.1	P$ABF_Q2	ABF	386	403	1	1	0.863	gtggtcACGTGgggggc
HvGSK4.1	P$BZR1_02	BZR1	387	401	1	1	0.939	tggtCACGTggggg
HvGSK4.1	P$HBI1_01	HBI1	387	399	1	1	0.942	tggtCACGTggg
HvGSK4.1	P$ABZ1_01	ABZ1	387	401	1	1	0.989	tggtcACGTGgggg
HvGSK4.1	P$GBP_Q6	GBP	388	400	1	1	0.939	ggtCACGTgggg
HvGSK4.1	P$PIF3_03	IF3	388	398	1	1	0.935	ggtCACGTgg
HvGSK4.1	P$ABI5_01	ABI5	388	398	1	1	0.963	ggtCACGTgg
HvGSK4.1	P$ABF4_01	ABF4	388	400	1	1	0.959	ggtCACGTgggg
HvGSK4.1	P$GBF1_Q2_01	GBF1	388	399	1	1	0.891	ggtcACGTGgg
HvGSK4.1	P$ALFIN1_Q2	ALFIN1	388	403	1	1	0.918	ggtcacGTGGGgggc
HvGSK4.1	P$EMBP1_Q2	EMBP1	389	399	1	1	0.944	gtCACGTggg
HvGSK4.1	P$CPRF_Q2	CPRF	389	399	1	1	0.941	gtCACGTggg
HvGSK4.1	P$CPRF3_Q2	CPRF3	389	399	1	1	0.992	gtCACGTggg
HvGSK4.1	P$CPRF2_Q2	CPRF2	389	399	1	1	1	gtCACGTggg
HvGSK4.1	P$O2_02	O2	389	399	1	1	0.972	gtCACGTggg
HvGSK4.1	P$TGA1B_Q2	TGA1B	389	399	1	1	0.951	gtCACGTggg

HvGSK4.1	P$TGA1A_Q2	TGA1A	389	399	1	1	0.987	gtCACGTggg
HvGSK4.1	P$CPRF1_01	CPRF1	389	399	1	1	0.941	gtCACGTggg
HvGSK4.1	P$CPRF3_01	CPRF3	389	399	1	1	0.991	gtCACGTggg
HvGSK4.1	P$CPRF2_01	CPRF2	389	399	1	1	1	gtCACGTggg
HvGSK4.1	P$TGA1B_01	TGA1B	389	399	1	1	0.936	gtCACGTggg
HvGSK4.1	P$BES1_01	BES1	389	400	1	1	0.963	gtCACGTgggg
HvGSK4.1	P$PIF3_04	IF3	389	399	1	1	0.885	gtCACGTggg
HvGSK4.1	P$BEE2_01	BEE2	389	399	1	1	0.999	gtCACGTggg
HvGSK4.1	P$BIM2_01	BIM2	389	399	1	1	0.996	gtCACGTggg
HvGSK4.1	P$BIM3_01	BIM3	389	399	1	1	0.992	gtCACGTggg
HvGSK4.1	P$PHYPA143875_02	HYPA143875	389	399	1	1	0.996	gtCACGTggg
HvGSK4.1	P$PHYPA72483_07	HYPA72483	389	399	1	1	0.999	gtCACGTggg
HvGSK4.1	P$SPT_01	SPT	389	398	1	1	0.974	gtCACGTgg
HvGSK4.1	P$GBF1F_Q2	GBF1F	389	400	1	1	0.89	gtCACGTgggg
HvGSK4.1	P$HBP1A_Q2	HBP1A	389	399	1	1	0.944	gtcACGTGgg
HvGSK4.1	P$TAF1_Q2	TAF1	389	399	1	1	0.98	gtcACGTGgg
HvGSK4.1	P$EMBP1_02	EMBP1	389	399	1	1	0.945	gtcACGTGgg
HvGSK4.1	P$TAF1_01	TAF1	389	399	1	1	0.993	gtcACGTGgg
HvGSK4.1	P$PIF1_01	IF1	389	399	1	1	0.999	gtcACGTGgg
HvGSK4.1	P$RITA1_01	RITA1	390	397	1	1	1	tCACGTg
HvGSK4.1	P$BHLH66_01	BHLH66	390	398	1	1	0.936	tCACGTgg
HvGSK4.1	P$PIF5_01	IF5	390	398	1	1	1	tCACGTgg
HvGSK4.1	P$MYC2_01	MYC2	390	398	1	1	0.942	tCACGTgg
HvGSK4.1	P$MYC3_01	MYC3	390	398	1	1	0.99	tCACGTgg
HvGSK4.1	P$BHLH34_01	BHLH34	390	398	1	1	0.972	tCACGTgg
HvGSK4.1	P$PHYPA48267_08	HYPA48267	390	398	1	1	0.972	tCACGTgg
HvGSK4.1	P$OJ1058_01	OJ1058	390	398	1	1	0.946	tCACGTgg
HvGSK4.1	P$UNE10_01	UNE10	390	398	1	1	0.98	tCACGTgg
HvGSK4.1	P$BHLH3_01	BHLH3	390	398	1	1	0.939	tCACGTgg
HvGSK4.1	P$HY5_01	HY5	390	400	1	1	0.951	tcACGTGggg
HvGSK4.1	P$GBF1_01	GBF1	390	398	1	1	0.987	tcACGTGg
HvGSK4.1	P$MYC4_01	MYC4	390	398	1	1	0.948	tcACGTGg
HvGSK4.1	P$BIM1_02	BIM1	390	400	1	1	0.995	tcACGTGggg
HvGSK4.1	P$BHLH13_01	BHLH13	390	398	1	1	0.933	tcACGTGg
HvGSK4.1	P$ABF4_02	ABF4	390	400	1	1	0.973	tcACGTGggg
HvGSK4.1	P$BZIP68_01	BZIP68	390	399	1	1	0.979	tcaCGTGGg
HvGSK4.1	P$OCSBF1_01	OCSBF1	391	396	1	1	1	CACGT
HvGSK4.1	P$PIF4_01	IF4	391	399	1	1	0.977	CACGTggg
HvGSK4.1	P$CPRF1_Q2	CPRF1	391	401	1	1	0.962	cACGTGgggg
HvGSK4.1	P$ABI5_Q2	ABI5	392	398	1	1	1	ACGTGg
HvGSK4.1	P$HSF3_01	HSF3	405	411	1	1	0.94	tCGGGG
HvGSK4.1	P$GATA8_01	GATA8	432	441	1	1	0.99	tcGATCTat
HvGSK4.1	P$HSFA4A_01	HSFA4A	436	442	1	1	0.914	tCTATT
HvGSK4.1	P$GATA9_01	GATA9	441	452	1	1	0.893	ttcAGATCagg
HvGSK4.1	P$AGP1_01	AGP1	442	452	1	1	0.872	tcAGATCagg
HvGSK4.1	P$GATA10_01	GATA10	443	451	1	1	0.881	cAGATCag
HvGSK4.1	P$ARR10_01	ARR10	444	451	1	1	0.934	AGATCag
HvGSK4.1	P$DREB1E_01	DREB1E	449	459	1	1	0.876	aGGCCGtcgg
HvGSK4.1	P$AT1G53910_01	AT1G53910	449	459	1	1	0.917	aGGCCGtcgg
HvGSK4.1	P$ANAC094_01	ANAC094	450	468	1	1	0.876	gGCCGTcggcgcctcggg
HvGSK4.1	P$DREB1A_04	DREB1A	452	462	1	1	0.967	ccGTCGGcgc
HvGSK4.1	P$ERF039_01	ERF039	452	462	1	1	0.983	ccGTCGGcgc
HvGSK4.1	P$PHYPA182268_05	HYPA182268	452	462	1	1	0.878	ccGTCGGcgc
HvGSK4.1	P$PHYPA173530_04	HYPA173530	453	461	1	1	0.875	cGTCGGcg
HvGSK4.1	P$PHYPA28324_10	HYPA28324	453	461	1	1	0.92	cGTCGGcg
HvGSK4.1	P$AT1G68550_03	AT1G68550	453	462	1	1	0.96	cgtCGGCGc
HvGSK4.1	P$E2L_Q2	E2L	465	472	1	1	0.928	gGGCGGg
HvGSK4.1	P$P_01		476	485	1	1	0.88	ggCTACCct
HvGSK4.1	P$ARF8_01	ARF8	496	505	1	1	0.953	tgTGTCGcg
HvGSK4.1	P$AT1G53910_02	AT1G53910	510	531	1	1	0.936	caggaacggtgGCGGCgcgat
HvGSK4.1	P$RRTF1_05	RRTF1	515	530	1	1	0.906	acggtggCGGCGcga
HvGSK4.1	P$E2L_Q2	E2L	519	526	1	1	0.91	tGGCGGc
HvGSK4.1	P$AT1G68550_03	AT1G68550	519	528	1	1	1	tggCGGCGc
HvGSK4.1	P$ERF1_Q2	ERF1	520	528	1	1	0.957	GGCGGcgc
HvGSK4.1	P$ARR1_01	ARR1	529	539	1	1	0.996	atcGAATCtg
HvGSK4.1	P$OJ1581_01	OJ1581	536	546	1	1	0.99	ctGGGCCcag
HvGSK4.1	P$TCP2_01	TCP2	536	546	1	1	0.991	ctGGGCCcag
HvGSK4.1	P$AMS_01	AMS	541	551	1	1	0.909	ccCAGGTgtg
HvGSK4.1	P$ALFIN1_Q2	ALFIN1	542	557	1	1	0.878	ccaggtGTGGGcctt
HvGSK4.1	P$TCP11_01	TCP11	547	559	1	1	0.989	tGTGGGccttgg
HvGSK4.1	P$PCF5_01	CF5	555	565	1	1	0.919	ttGGTCCcta
HvGSK4.1	P$MYB1L_01	MYB1L	558	568	1	1	0.942	gtCCCTAgat
HvGSK4.1	P$TRB2_01	TRB2	558	566	1	1	0.932	gtCCCTAg
HvGSK4.1	P$RRTF1_05	RRTF1	567	582	1	1	0.869	ttgtggtCGGCGcag
HvGSK4.1	P$DREB1A_04	DREB1A	570	580	1	1	0.968	tgGTCGGcgc
HvGSK4.1	P$ERF039_01	ERF039	570	580	1	1	0.98	tgGTCGGcgc
HvGSK4.1	P$PHYPA182268_05	HYPA182268	570	580	1	1	0.884	tgGTCGGcgc
HvGSK4.1	P$PHYPA173530_04	HYPA173530	571	579	1	1	0.894	gGTCGGcg

HvGSK4.1	P$PHYPA28324_10	HYPA28324	571	579	1	1	0.929	gGTCGGcg
HvGSK4.1	P$AT1G28160_02	AT1G28160	571	586	1	1	0.851	ggtCGGCGcagtggc
HvGSK4.1	P$AT1G68550_03	AT1G68550	571	580	1	1	0.96	ggtCGGCGc
HvGSK4.1	P$E2L_Q2	E2L	582	589	1	1	0.892	tGGCGGa
HvGSK4.1	P$NAC92_01	NAC92	586	598	1	1	0.99	ggACACGacacg
HvGSK4.1	P$HBP1B_Q6	HBP1B	588	602	1	1	0.906	acacgacaCGTCAg
HvGSK4.1	P$ABF2_01	ABF2	589	602	1	1	0.952	cacgaCACGTcag
HvGSK4.1	P$O2_Q4	O2	590	601	1	1	0.916	acgaCACGTca
HvGSK4.1	P$BZR1_02	BZR1	590	604	1	1	0.868	acgaCACGTcagta
HvGSK4.1	P$NAC92_01	NAC92	591	603	1	1	0.989	cgACACGtcagt
HvGSK4.1	P$GBP_Q6	GBP	591	603	1	1	0.9	cgaCACGTcagt
HvGSK4.1	P$ABI5_01	ABI5	591	601	1	1	0.957	cgaCACGTca
HvGSK4.1	P$GBF6_01	GBF6	591	606	1	1	0.895	cgaCACGTcagtagt
HvGSK4.1	P$ABF4_01	ABF4	591	603	1	1	0.913	cgaCACGTcagt
HvGSK4.1	P$EMBP1_Q2	EMBP1	592	602	1	1	0.925	gaCACGTcag
HvGSK4.1	P$CPRF3_Q2	CPRF3	592	602	1	1	0.966	gaCACGTcag
HvGSK4.1	P$CPRF2_Q2	CPRF2	592	602	1	1	0.965	gaCACGTcag
HvGSK4.1	P$O2_02	O2	592	602	1	1	0.952	gaCACGTcag
HvGSK4.1	P$TGA1B_Q2	TGA1B	592	602	1	1	0.974	gaCACGTcag
HvGSK4.1	P$TGA1A_Q2	TGA1A	592	602	1	1	0.992	gaCACGTcag
HvGSK4.1	P$CPRF3_01	CPRF3	592	602	1	1	0.979	gaCACGTcag
HvGSK4.1	P$CPRF2_01	CPRF2	592	602	1	1	0.966	gaCACGTcag
HvGSK4.1	P$TGA1B_01	TGA1B	592	602	1	1	0.936	gaCACGTcag
HvGSK4.1	P$BEE2_01	BEE2	592	602	1	1	0.906	gaCACGTcag
HvGSK4.1	P$BIM3_01	BIM3	592	602	1	1	0.876	gaCACGTcag
HvGSK4.1	P$PHYPA143875_02	HYPA143875	592	602	1	1	0.871	gaCACGTcag
HvGSK4.1	P$SPT_01	SPT	592	601	1	1	0.917	gaCACGTca
HvGSK4.1	P$GBF1F_Q2	GBF1F	592	603	1	1	0.882	gaCACGTcagt
HvGSK4.1	P$BZIP43_01	BZIP43	592	604	1	1	0.86	gaCACGTcagta
HvGSK4.1	P$BZIP48_01	BZIP48	592	606	1	1	0.888	gaCACGTcagtagt
HvGSK4.1	P$HBPA1_Q6_01	HBPA1	592	602	1	1	0.981	gacaCGTCAg
HvGSK4.1	P$RITA1_01	RITA1	593	600	1	1	0.963	aCACGTc
HvGSK4.1	P$OCSBF1_01	OCSBF1	594	599	1	1	1	CACGT
HvGSK4.1	P$TGA1A_01	TGA1A	594	601	1	1	0.989	cACGTCa
HvGSK4.1	P$DREB1A_04	DREB1A	606	616	1	1	0.929	tgGTCGGatc
HvGSK4.1	P$ERF039_01	ERF039	606	616	1	1	0.941	tgGTCGGatc
HvGSK4.1	P$PHYPA28324_10	HYPA28324	607	615	1	1	0.869	gGTCGGat
HvGSK4.1	P$UIF1_01	UIF1	619	629	1	1	0.978	cgaGATTCta
HvGSK4.1	P$P_01		624	633	1	1	0.88	ttCTACCct
HvGSK4.1	P$GATA15_01	GATA15	635	644	1	1	0.999	tcTGATCca
HvGSK4.1	P$HSF3_01	HSF3	661	667	1	1	1	cCGGGG
HvGSK4.1	P$AT3G51080_01	AT3G51080	665	672	1	1	0.89	GGAAAac
HvGSK4.1	P$MYB1L_01	MYB1L	669	679	1	1	1	aaCCCTAgat
HvGSK4.1	P$TRB2_01	TRB2	669	677	1	1	0.971	aaCCCTAg
HvGSK4.1	P$GATA9_01	GATA9	672	683	1	1	0.992	cctAGATCtcc
HvGSK4.1	P$AGP1_01	AGP1	673	683	1	1	0.933	ctAGATCtcc
HvGSK4.1	P$GATA10_01	GATA10	674	682	1	1	0.954	tAGATCtc
HvGSK4.1	P$GATA11_01	GATA11	674	682	1	1	0.969	taGATCTc
HvGSK4.1	P$GATA8_01	GATA8	674	683	1	1	0.99	taGATCTcc
HvGSK4.1	P$ARR10_01	ARR10	675	682	1	1	0.913	AGATCtc
HvGSK4.1	P$RAP21_02	RAP21	696	709	1	1	0.945	atggCGGTGcttt
HvGSK4.1	P$E2L_Q2	E2L	697	704	1	1	0.892	tGGCGGt
HvGSK4.1	P$ERF1_Q2	ERF1	698	706	1	1	0.867	GGCGGtgc
HvGSK4.1	P$CBNAC_01	CBNAC	702	708	1	1	0.979	gTGCTT
HvGSK4.1	P$ARF8_01	ARF8	709	718	1	1	0.95	tgTGTCGta
HvGSK4.1	P$TCP24_01	TCP24	757	769	1	1	0.885	gaagGGACCaag
HvGSK4.1	P$TCP4_01	TCP4	760	768	1	1	0.856	gGGACCaa
HvGSK4.1	P$TCP5_01	TCP5	760	768	1	1	0.94	gGGACCaa
HvGSK4.1	P$ARALY496250_03	ARALY496250	760	768	1	1	0.877	gGGACCaa
HvGSK4.1	P$CBF3_02	CBF3	768	782	1	1	0.952	gaacgCCGACggcg
HvGSK4.1	P$ERF112_02	ERF112	769	779	1	1	0.966	aaCGCCGacg
HvGSK4.1	P$CBF1_04	CBF1	769	781	1	1	0.951	aacgCCGACggc
HvGSK4.1	P$CRF4_01	CRF4	770	778	1	1	0.92	aCGCCGac
HvGSK4.1	P$ERF4_04	ERF4	770	778	1	1	0.946	aCGCCGac
HvGSK4.1	P$ERF069_01	ERF069	770	779	1	1	0.99	aCGCCGacg
HvGSK4.1	P$ERF11_01	ERF11	770	780	1	1	0.986	aCGCCGacgg
HvGSK4.1	P$ERF5_02	ERF5	770	780	1	1	0.898	acGCCGAcgg
HvGSK4.1	P$ERF1_04	ERF1	770	780	1	1	0.884	acGCCGAcgg
HvGSK4.1	P$DREB1G_02	DREB1G	770	780	1	1	0.904	acgCCGACgg
HvGSK4.1	P$RRTF1_05	RRTF1	770	785	1	1	0.853	acgccgaCGGCGcac
HvGSK4.1	P$ERF8_01	ERF8	771	781	1	1	0.982	CGCCGacggc
HvGSK4.1	P$ERF3_04	ERF3	771	779	1	1	0.949	CGCCGacg
HvGSK4.1	P$ARF1_01	ARF1	772	780	1	1	0.854	gCCGACgg
HvGSK4.1	P$ARF5_01	ARF5	772	780	1	1	0.897	gCCGACgg
HvGSK4.1	P$DREB1B_01	DREB1B	773	778	1	1	1	CCGAC
HvGSK4.1	P$AT1G68550_03	AT1G68550	774	783	1	1	0.97	cgaCGGCGc
HvGSK4.1	P$HSFA1E_01	HSFA1E	776	782	1	1	0.87	aCGGCG
HvGSK4.1	P$ARF8_01	ARF8	798	807	1	1	0.955	acTGTCGat

HvGSK4.1	P$HAT1_01	HAT1	808	818	1	1	0.856	aaAATCAcgc
HvGSK4.1	P$CBF3_02	CBF3	812	826	1	1	0.961	tcacgCCGACtatg
HvGSK4.1	P$ERF112_02	ERF112	813	823	1	1	0.964	caCGCCGact
HvGSK4.1	P$CBF1_04	CBF1	813	825	1	1	0.951	cacgCCGACtat
HvGSK4.1	P$CRF4_01	CRF4	814	822	1	1	0.92	aCGCCGac
HvGSK4.1	P$ERF4_04	ERF4	814	822	1	1	0.946	aCGCCGac
HvGSK4.1	P$ERF069_01	ERF069	814	823	1	1	0.99	aCGCCGact
HvGSK4.1	P$ERF11_01	ERF11	814	824	1	1	0.988	aCGCCGacta
HvGSK4.1	P$ERF5_02	ERF5	814	824	1	1	0.947	acGCCGActa
HvGSK4.1	P$ERF1_04	ERF1	814	824	1	1	0.938	acGCCGActa
HvGSK4.1	P$DREB1G_02	DREB1G	814	824	1	1	0.912	acgCCGACta
HvGSK4.1	P$ERF8_01	ERF8	815	825	1	1	0.984	CGCCGactat
HvGSK4.1	P$ERF3_04	ERF3	815	823	1	1	0.939	CGCCGact
HvGSK4.1	P$ARF5_01	ARF5	816	824	1	1	0.903	gCCGACta
HvGSK4.1	P$DREB1B_01	DREB1B	817	822	1	1	1	CCGAC
HvGSK4.1	P$ARF8_01	ARF8	853	862	1	1	0.952	gaTGTCGtt
HvGSK4.1	P$DRE1C_01	DRE1C	854	862	1	1	0.862	ATGTCgtt
HvGSK4.1	P$ARF8_01	ARF8	864	873	1	1	0.958	ctTGTCGag
HvGSK4.1	P$TGA2_Q2	TGA2	891	901	1	1	0.881	gCGTCAttag
HvGSK4.1	P$MYB24_01	MYB24	894	903	1	1	0.929	tcaTTAGGc
HvGSK4.1	P$MYB1L_01	MYB1L	921	931	1	1	1	aaCCCTAgat
HvGSK4.1	P$TRB2_01	TRB2	921	929	1	1	0.971	aaCCCTAg
HvGSK4.1	P$GATA9_01	GATA9	924	935	1	1	1	cctAGATCtgg
HvGSK4.1	P$AGP1_01	AGP1	925	935	1	1	0.945	ctAGATCtgg
HvGSK4.1	P$GATA10_01	GATA10	926	934	1	1	1	tAGATCtg
HvGSK4.1	P$GATA11_01	GATA11	926	934	1	1	1	taGATCTg
HvGSK4.1	P$GATA8_01	GATA8	926	935	1	1	0.999	taGATCTgg
HvGSK4.1	P$ARR10_01	ARR10	927	934	1	1	0.978	AGATCtg
HvGSK4.1	P$AZF3_01	AZF3	965	976	1	1	0.851	cAGTATcgctt
HvGSK4.1	P$AG_02	AG	975	991	1	1	0.856	TTTCCtgttgggggat
HvGSK4.1	P$AGL15_03	AGL15	975	990	1	1	0.892	TTTCCtgttggggga
HvGSK4.1	P$P_01		1042	1051	1	1	0.921	atCTACCgc
HvGSK4.1	P$CBF3_02	CBF3	1050	1064	1	1	0.958	cagggCCGACgatg
HvGSK4.1	P$CBF1_04	CBF1	1051	1063	1	1	0.95	agggCCGACgat
HvGSK4.1	P$CBF2_02	CBF2	1052	1063	1	1	0.922	gGGCCGacgat
HvGSK4.1	P$ERF3_02	ERF3	1052	1062	1	1	0.857	gGGCCGacga
HvGSK4.1	P$DREB1G_01	DREB1G	1052	1062	1	1	0.872	gGGCCGacga
HvGSK4.1	P$DREB1E_01	DREB1E	1052	1062	1	1	0.91	gGGCCGacga
HvGSK4.1	P$ERF2_04	ERF2	1052	1062	1	1	0.866	gGGCCGacga
HvGSK4.1	P$ERF3_03	ERF3	1052	1062	1	1	0.865	gGGCCGacga
HvGSK4.1	P$ATERF12_01	ATERF12	1052	1062	1	1	0.868	gGGCCGacga
HvGSK4.1	P$AT1G53910_01	AT1G53910	1052	1062	1	1	0.952	gGGCCGacga
HvGSK4.1	P$ERF5_02	ERF5	1052	1062	1	1	0.961	ggGCCGAcga
HvGSK4.1	P$ERF1_04	ERF1	1052	1062	1	1	0.961	ggGCCGAcga
HvGSK4.1	P$AT2G20350_01	AT2G20350	1052	1062	1	1	0.85	ggGCCGAcga
HvGSK4.1	P$DREB1G_02	DREB1G	1052	1062	1	1	0.9	gggCCGACga
HvGSK4.1	P$ARF1_01	ARF1	1054	1062	1	1	0.855	gCCGACga
HvGSK4.1	P$ARF5_01	ARF5	1054	1062	1	1	0.903	gCCGACga
HvGSK4.1	P$DREB1B_01	DREB1B	1055	1060	1	1	1	CCGAC
HvGSK4.1	P$ABI3_01	ABI3	1072	1081	1	1	0.884	cgGCATGgc
HvGSK4.1	P$AT5G04240_01	AT5G04240	1077	1083	1	1	0.938	tGGCAC
HvGSK4.1	P$RRTF1_05	RRTF1	1086	1101	1	1	0.914	ggagtttCGGCGacg
HvGSK4.1	P$AT1G68550_03	AT1G68550	1090	1099	1	1	0.951	tttCGGCGa
HvGSK4.1	P$CMTA2_01	CMTA2	1099	1108	1	1	0.99	cgaaCGCGT
HvGSK4.1	P$CAMTA1_02	CAMTA1	1099	1111	1	1	0.93	cgaaCGCGTttg
HvGSK4.1	P$CMTA3_01	CMTA3	1102	1111	1	1	0.985	aCGCGTttg
HvGSK4.1	P$PIL5_01	IL5	1110	1124	1	1	0.986	ggatggacACGTGc
HvGSK4.1	P$PIF3_01	IF3	1111	1129	1	1	0.915	gatggaCACGTgcatgat
HvGSK4.1	P$HY5_02	HY5	1112	1128	1	1	0.87	atggaCACGTgcatga
HvGSK4.1	P$ABF2_01	ABF2	1112	1125	1	1	0.996	atggaCACGTgca
HvGSK4.1	P$BZR1_02	BZR1	1113	1127	1	1	0.933	tggaCACGTgcatg
HvGSK4.1	P$HBI1_01	HBI1	1113	1125	1	1	0.941	tggaCACGTgca
HvGSK4.1	P$ABZ1_01	ABZ1	1113	1127	1	1	0.907	tggacACGTGcatg
HvGSK4.1	P$GBP_Q6	GBP	1114	1126	1	1	0.917	ggaCACGTgcat
HvGSK4.1	P$PIF3_03	IF3	1114	1124	1	1	0.931	ggaCACGTgc
HvGSK4.1	P$ABI5_01	ABI5	1114	1124	1	1	0.998	ggaCACGTgc
HvGSK4.1	P$ABF4_01	ABF4	1114	1126	1	1	0.977	ggaCACGTgcat
HvGSK4.1	P$BZR1_03	BZR1	1114	1134	1	1	0.899	ggacACGTGcatgatggtgg
HvGSK4.1	P$EMBP1_Q2	EMBP1	1115	1125	1	1	0.95	gaCACGTgca
HvGSK4.1	P$CPRF_Q2	CPRF	1115	1125	1	1	0.943	gaCACGTgca
HvGSK4.1	P$CPRF3_Q2	CPRF3	1115	1125	1	1	0.977	gaCACGTgca
HvGSK4.1	P$CPRF2_Q2	CPRF2	1115	1125	1	1	0.983	gaCACGTgca
HvGSK4.1	P$O2_02	O2	1115	1125	1	1	0.946	gaCACGTgca
HvGSK4.1	P$TGA1B_Q2	TGA1B	1115	1125	1	1	0.918	gaCACGTgca
HvGSK4.1	P$TGA1A_Q2	TGA1A	1115	1125	1	1	0.977	gaCACGTgca
HvGSK4.1	P$CPRF1_01	CPRF1	1115	1125	1	1	0.971	gaCACGTgca
HvGSK4.1	P$CPRF3_01	CPRF3	1115	1125	1	1	0.977	gaCACGTgca
HvGSK4.1	P$CPRF2_01	CPRF2	1115	1125	1	1	0.983	gaCACGTgca

HvGSK4.1	P$TGA1B_01	TGA1B	1115	1125	1	1	0.92	gaCACGTgca
HvGSK4.1	P$BES1_01	BES1	1115	1126	1	1	0.96	gaCACGTgcat
HvGSK4.1	P$BEE2_01	BEE2	1115	1125	1	1	0.999	gaCACGTgca
HvGSK4.1	P$BHLH104_01	BHLH104	1115	1125	1	1	0.875	gaCACGTgca
HvGSK4.1	P$BIM2_01	BIM2	1115	1125	1	1	0.997	gaCACGTgca
HvGSK4.1	P$BIM3_01	BIM3	1115	1125	1	1	0.995	gaCACGTgca
HvGSK4.1	P$PHYPA143875_02	HYPA143875	1115	1125	1	1	0.998	gaCACGTgca
HvGSK4.1	P$PHYPA72483_07	HYPA72483	1115	1125	1	1	0.998	gaCACGTgca
HvGSK4.1	P$SPT_01	SPT	1115	1124	1	1	0.969	gaCACGTgc
HvGSK4.1	P$GBF1F_Q2	GBF1F	1115	1126	1	1	0.892	gaCACGTgcat
HvGSK4.1	P$TSAR1_01	TSAR1	1115	1125	1	1	0.89	gaCACGTgca
HvGSK4.1	P$HBP1A_Q2	HBP1A	1115	1125	1	1	0.925	gacACGTGca
HvGSK4.1	P$TAF1_Q2	TAF1	1115	1125	1	1	0.954	gacACGTGca
HvGSK4.1	P$EMBP1_02	EMBP1	1115	1125	1	1	0.945	gacACGTGca
HvGSK4.1	P$TAF1_01	TAF1	1115	1125	1	1	0.972	gacACGTGca
HvGSK4.1	P$PIF1_01	IF1	1115	1125	1	1	0.985	gacACGTGca
HvGSK4.1	P$ABF3_01	ABF3	1116	1124	1	1	0.875	ACACGtgc
HvGSK4.1	P$RITA1_01	RITA1	1116	1123	1	1	0.984	aCACGTg
HvGSK4.1	P$BHLH66_01	BHLH66	1116	1124	1	1	0.962	aCACGTgc
HvGSK4.1	P$PIF5_01	IF5	1116	1124	1	1	0.917	aCACGTgc
HvGSK4.1	P$MYC2_01	MYC2	1116	1124	1	1	0.981	aCACGTgc
HvGSK4.1	P$MYC3_01	MYC3	1116	1124	1	1	0.997	aCACGTgc
HvGSK4.1	P$BHLH34_01	BHLH34	1116	1124	1	1	0.964	aCACGTgc
HvGSK4.1	P$PHYPA48267_08	HYPA48267	1116	1124	1	1	0.996	aCACGTgc
HvGSK4.1	P$OJ1058_01	OJ1058	1116	1124	1	1	0.964	aCACGTgc
HvGSK4.1	P$UNE10_01	UNE10	1116	1124	1	1	0.994	aCACGTgc
HvGSK4.1	P$BHLH3_01	BHLH3	1116	1124	1	1	0.98	aCACGTgc
HvGSK4.1	P$AIB_01	AIB	1116	1124	1	1	0.899	aCACGTgc
HvGSK4.1	P$GBF1_01	GBF1	1116	1124	1	1	0.927	acACGTGc
HvGSK4.1	P$MYC4_01	MYC4	1116	1124	1	1	1	acACGTGc
HvGSK4.1	P$BIM1_02	BIM1	1116	1126	1	1	0.997	acACGTGcat
HvGSK4.1	P$BHLH13_01	BHLH13	1116	1124	1	1	0.987	acACGTGc
HvGSK4.1	P$ABF4_02	ABF4	1116	1126	1	1	0.952	acACGTGcat
HvGSK4.1	P$OCSBF1_01	OCSBF1	1117	1122	1	1	1	CACGT
HvGSK4.1	P$PIF4_01	IF4	1117	1125	1	1	0.964	CACGTgca
HvGSK4.1	P$ABI5_Q2	ABI5	1118	1124	1	1	0.936	ACGTGc
HvGSK4.1	P$BZR1_01	BZR1	1119	1125	1	1	0.915	CGTGCa
HvGSK4.1	P$ABI3_01	ABI3	1120	1129	1	1	0.866	gtGCATGat
HvGSK4.1	P$CBF1_01	CBF1	1143	1153	1	1	0.863	gTGCCGaggt
HvGSK4.1	P$CBF1_03	CBF1	1143	1153	1	1	0.886	gTGCCGaggt
HvGSK4.1	P$TGA1_01	TGA1	1155	1166	1	1	0.939	cgtTGACGgca
HvGSK4.1	P$WRKY11_Q2	WRKY11	1156	1164	1	1	0.9	gTTGACgg
HvGSK4.1	P$TGA7_01	TGA7	1156	1166	1	1	0.89	gtTGACGgca
HvGSK4.1	P$TGA5_01	TGA5	1157	1165	1	1	0.856	tTGACGgc
HvGSK4.1	P$KNOX3_01	KNOX3	1180	1192	1	1	0.999	gatgTGACAgtt
HvGSK4.1	P$ATMYB77_01	ATMYB77	1181	1194	1	1	0.936	atgtgaCAGTTcc
HvGSK4.1	P$ATH1_01	ATH1	1184	1192	1	1	0.987	TGACAgtt
HvGSK4.1	P$AT1G53910_02	AT1G53910	1211	1232	1	1	0.945	ggtggaagatgGCGGCgacaa
HvGSK4.1	P$RRTF1_05	RRTF1	1216	1231	1	1	0.933	aagatggCGGCGaca
HvGSK4.1	P$E2L_Q2	E2L	1220	1227	1	1	0.91	tGGCGGc
HvGSK4.1	P$AT1G28160_02	AT1G28160	1220	1235	1	1	0.853	tggCGGCGacaacgg
HvGSK4.1	P$RAP26_06	RAP26	1220	1235	1	1	0.856	tggCGGCGacaacgg
HvGSK4.1	P$AT1G68550_03	AT1G68550	1220	1229	1	1	0.994	tggCGGCGa
HvGSK4.1	P$ERF1_Q2	ERF1	1221	1229	1	1	0.94	GGCGGcga
HvGSK4.1	P$GAMYB_Q2	GAMYB	1223	1236	1	1	0.943	cggcgACAACggg
HvGSK4.1	P$AT5G54070_01	AT5G54070	1228	1234	1	1	0.958	aCAACG
HvGSK4.1	P$ERF112_02	ERF112	1238	1248	1	1	0.929	tgCGCCGgac
HvGSK4.1	P$CRF4_01	CRF4	1239	1247	1	1	0.875	gCGCCGga
HvGSK4.1	P$ERF4_04	ERF4	1239	1247	1	1	0.87	gCGCCGga
HvGSK4.1	P$ERF069_01	ERF069	1239	1248	1	1	0.989	gCGCCGgac
HvGSK4.1	P$ERF11_01	ERF11	1239	1249	1	1	0.97	gCGCCGgacc
HvGSK4.1	P$ERF8_01	ERF8	1240	1250	1	1	0.946	CGCCGgaccg
HvGSK4.1	P$AT1G68550_03	AT1G68550	1245	1254	1	1	0.949	gacCGGCGt
HvGSK4.1	P$ALFIN1_Q2	ALFIN1	1246	1261	1	1	0.854	accggcGTGGGaccc
HvGSK4.1	P$HSFA1E_01	HSFA1E	1247	1253	1	1	1	cCGGCG
HvGSK4.1	P$BZIP68_01	BZIP68	1248	1257	1	1	0.929	cggCGTGGg
HvGSK4.1	P$TCP15_01	TCP15	1252	1262	1	1	1	GTGGGacccg
HvGSK4.1	P$TCP20_01	TCP20	1252	1262	1	1	0.992	GTGGGacccg
HvGSK4.1	P$TCP11_02	TCP11	1252	1262	1	1	0.868	GTGGGacccg
HvGSK4.1	P$TCP7_01	TCP7	1252	1263	1	1	0.95	GTGGGacccgg
HvGSK4.1	P$PCF5_01	CF5	1259	1269	1	1	0.896	ccGGTCCcgg
HvGSK4.1	P$AT3G18100_01	AT3G18100	1310	1321	1	1	0.919	tggTGCGAtgt
HvGSK4.1	P$MRP1_Q2	MRP1	1318	1330	1	1	0.852	tgTCTATttggt
HvGSK4.1	P$HSFA4A_01	HSFA4A	1320	1326	1	1	0.914	tCTATT
HvGSK4.1	P$AT5G04240_01	AT5G04240	1346	1352	1	1	0.976	cGGCAC
HvGSK4.1	P$RAV1_01	RAV1	1374	1386	1	1	0.954	tagCAACAggtg
HvGSK4.1	P$AMS_01	AMS	1378	1388	1	1	0.876	aaCAGGTgtt
HvGSK4.1	P$ARR18_01	ARR18	1388	1401	1	1	0.888	gcctAGATAgcgg

HvGSK4.1	P$MYB24_01	MYB24	1400	1409	1	1	0.907	gctTTAGGc
HvGSK4.1	P$SBF1_01	SBF1	1440	1454	1	1	0.941	gagtaaTTAATaaa
HvGSK4.1	P$EDT1_01	EDT1	1443	1453	1	1	0.892	taaTTAATaa
HvGSK4.1	P$ATHB6_01	ATHB6	1446	1455	1	1	0.91	ttAATAAaa
HvGSK4.1	P$HMG1_01	HMG1	1457	1466	1	1	0.874	GTTGTaacc
HvGSK4.1	P$HAHB4_01	HAHB4	1471	1480	1	1	0.949	tAATGAtgc
HvGSK4.1	P$PCF2_01	CF2	1480	1490	1	1	0.891	ccgagCCCAC
HvGSK4.1	P$TCP19_01	TCP19	1480	1490	1	1	0.911	ccgagCCCAC
HvGSK4.1	P$TCP20L_01	TCP20L	1481	1490	1	1	0.893	cgagCCCAC
HvGSK4.1	P$OSI_01	OSI	1482	1490	1	1	0.859	gagCCCAC
HvGSK4.1	P$TCP20_02	TCP20	1482	1492	1	1	0.916	gagCCCACcg
HvGSK4.1	P$ARALY495258_02	ARALY495258	1482	1490	1	1	0.956	gagCCCAC
HvGSK4.1	P$ARALY493022_04	ARALY493022	1482	1490	1	1	0.85	gagCCCAC
HvGSK4.1	P$ARALY484486_05	ARALY484486	1482	1490	1	1	0.956	gagCCCAC
HvGSK4.1	P$CBF3_02	CBF3	1484	1498	1	1	0.958	gcccaCCGACtgct
HvGSK4.1	P$CBF1_04	CBF1	1485	1497	1	1	0.938	cccaCCGACtgc
HvGSK4.1	P$AT1G77200_03	AT1G77200	1486	1500	1	1	0.896	ccaCCGACtgctcc
HvGSK4.1	P$ARF1_01	ARF1	1488	1496	1	1	0.887	aCCGACtg
HvGSK4.1	P$ARF5_01	ARF5	1488	1496	1	1	0.891	aCCGACtg
HvGSK4.1	P$DREB1B_01	DREB1B	1489	1494	1	1	1	CCGAC
HvGSK4.1	P$MYB3R5_01	MYB3R5	1494	1509	1	1	0.907	tgctcctagCCGTTg
HvGSK4.1	P$MYB3R1_01	MYB3R1	1495	1510	1	1	0.92	gctcctagCCGTTgg
HvGSK4.1	P$MYB3R4_01	MYB3R4	1495	1510	1	1	0.943	gctcctagCCGTTgg
HvGSK4.1	P$AT1G19490_01	AT1G19490	1538	1547	1	1	0.895	GGTTTaaaa
HvGSK4.1	P$AT4G36620_01	AT4G36620	1542	1550	1	1	0.915	taaAACCA
HvGSK4.1	P$HSFA2_01	HSFA2	1547	1553	1	1	0.941	CCAAAc
HvGSK4.1	P$AT4G12750_01	AT4G12750	1548	1558	1	1	0.912	caaACCGAga
HvGSK4.1	P$DOF1_01	DOF1	1556	1567	1	1	0.975	gacTAAAGggt
HvGSK4.1	P$PBF_Q2	BF	1559	1565	1	1	0.986	tAAAGG
HvGSK4.1	P$WRKY18_02	WRKY18	1562	1572	1	1	0.995	aggGTCAAat
HvGSK4.1	P$WRKY21_02	WRKY21	1562	1572	1	1	0.969	aggGTCAAat
HvGSK4.1	P$WRKY48_02	WRKY48	1562	1572	1	1	0.998	aggGTCAAat
HvGSK4.1	P$WRKY57_01	WRKY57	1562	1572	1	1	0.973	aggGTCAAat
HvGSK4.1	P$WRKY60_01	WRKY60	1562	1573	1	1	0.971	aggGTCAAatg
HvGSK4.1	P$WRKY15_01	WRKY15	1563	1573	1	1	0.985	ggGTCAAatg
HvGSK4.1	P$WRKY2_01	WRKY2	1563	1571	1	1	0.989	ggGTCAAa
HvGSK4.1	P$WRKY25_02	WRKY25	1563	1571	1	1	0.966	ggGTCAAa
HvGSK4.1	P$WRKY40_01	WRKY40	1563	1571	1	1	1	ggGTCAAa
HvGSK4.1	P$WRKY43_02	WRKY43	1563	1573	1	1	0.975	ggGTCAAatg
HvGSK4.1	P$WRKY62_01	WRKY62	1563	1571	1	1	0.9	ggGTCAAa
HvGSK4.1	P$WRKY63_01	WRKY63	1563	1571	1	1	0.989	ggGTCAAa
HvGSK4.1	P$WRKY75_01	WRKY75	1563	1571	1	1	0.975	ggGTCAAa
HvGSK4.1	P$WRKY8_01	WRKY8	1563	1572	1	1	0.991	ggGTCAAat
HvGSK4.1	P$WRKY30_01	WRKY30	1564	1574	1	1	0.917	gGTCAAatga
HvGSK4.1	P$WRKY18_Q2	WRKY18	1565	1574	1	1	0.969	GTCAAatga
HvGSK4.1	P$PBF_Q2	BF	1572	1578	1	1	0.965	gAAAGG
HvGSK4.1	P$RAV1_02	RAV1	1606	1618	1	1	0.91	cgaACCTGtttt
HvGSK4.1	P$SQUA_01	SQUA	1611	1621	1	1	0.871	ctgTTTTTaa
HvGSK4.1	P$GT1_Q6_02	GT1	1611	1623	1	1	0.978	ctgtttTTAACt
HvGSK4.1	P$GATA15_01	GATA15	1621	1630	1	1	0.999	ctTGATCat
HvGSK4.1	P$ARR2_01	ARR2	1656	1666	1	1	0.941	ttgcATCTTa
HvGSK4.1	P$AT2G26880_01	AT2G26880	1661	1675	1	1	0.863	tcttataTTTAAat
HvGSK4.1	P$SHP2_01	SHP2	1663	1674	1	1	0.868	ttataTTTAAa
HvGSK4.1	P$AT1G77950_01	AT1G77950	1663	1674	1	1	0.855	ttataTTTAAa
HvGSK4.1	P$SBF1_01	SBF1	1663	1677	1	1	0.904	ttatatTTAAAtac
HvGSK4.1	P$MYB1L_01	MYB1L	1674	1684	1	1	0.991	taCCCTAtgt
HvGSK4.1	P$TRB2_01	TRB2	1674	1682	1	1	0.959	taCCCTAt
HvGSK4.1	P$ID1_01	ID1	1729	1740	1	1	0.917	tTTGTCtattt
HvGSK4.1	P$MRP1_Q2	MRP1	1731	1743	1	1	0.871	tgTCTATttttt
HvGSK4.1	P$HSFA4A_01	HSFA4A	1733	1739	1	1	0.914	tCTATT
HvGSK4.1	P$SQUA_01	SQUA	1734	1744	1	1	0.915	ctaTTTTTtt
HvGSK4.1	P$PBF_01	BF	1746	1757	1	1	0.962	ttgAAAAGgag
HvGSK4.1	P$DOF_Q2	DOF	1746	1757	1	1	0.955	ttgAAAAGgag
HvGSK4.1	P$CDF2_01	CDF2	1747	1757	1	1	0.954	tgAAAAGgag
HvGSK4.1	P$CDF3_01	CDF3	1748	1757	1	1	0.973	gAAAAGgag
HvGSK4.1	P$PBF_Q2	BF	1749	1755	1	1	1	aAAAGG
HvGSK4.1	P$WRKY11_Q2	WRKY11	1766	1774	1	1	0.976	cTTGACct
HvGSK4.1	P$ZAP1_01	ZAP1	1767	1777	1	1	0.873	TTGACctctg
HvGSK4.1	P$AT2G15660_01	AT2G15660	1808	1819	1	1	0.866	TTCTCaagaaa
HvGSK4.1	P$BPC1_Q2	BPC1	1814	1820	1	1	1	AGAAAg
HvGSK4.1	P$ARR18_01	ARR18	1819	1832	1	1	0.942	gtttAGATAagaa
HvGSK4.1	P$PEND_01	END	1826	1834	1	1	1	tAAGAAgt
HvGSK4.1	P$PH4_01	H4	1833	1842	1	1	0.854	tatACCCCc
HvGSK4.1	P$C1_Q2	C1	1843	1854	1	1	0.979	caAACTAcaag
HvGSK4.1	P$ATHSFA1D_01	ATHSFA1D	1846	1852	1	1	1	aCTACA
HvGSK4.1	P$ATHB6_01	ATHB6	1883	1892	1	1	0.909	gaAATAAat
HvGSK4.1	P$PDF2_01	DF2	1883	1894	1	1	0.863	gaaaTAAATaa
HvGSK4.1	P$ATHB6_01	ATHB6	1887	1896	1	1	0.979	taAATAAtt

HvGSK4.1	P$ATHB5_04	ATHB5	1887	1898	1	1	0.958	taAATAAtttt
HvGSK4.1	P$ATHB1_03	ATHB1	1887	1898	1	1	0.974	taAATAAtttt
HvGSK4.1	P$ATHB16_01	ATHB16	1888	1896	1	1	0.953	aAATAAtt
HvGSK4.1	P$ATHSFA1D_01	ATHSFA1D	1909	1915	1	1	0.985	cCTACA
HvGSK4.1	P$ATHB6_01	ATHB6	1914	1923	1	1	0.91	atAATAAaa
HvGSK4.1	P$PBF_01	BF	1917	1928	1	1	0.982	ataAAAAGtgt
HvGSK4.1	P$DOF_Q2	DOF	1917	1928	1	1	0.984	ataAAAAGtgt
HvGSK4.1	P$CDF2_01	CDF2	1918	1928	1	1	0.998	taAAAAGtgt
HvGSK4.1	P$CDF3_01	CDF3	1919	1928	1	1	0.999	aAAAAGtgt
HvGSK4.1	P$ARF8_01	ARF8	1923	1932	1	1	0.993	agTGTCGga
HvGSK4.1	P$DREB1A_04	DREB1A	1924	1934	1	1	0.941	gtGTCGGact
HvGSK4.1	P$ERF039_01	ERF039	1924	1934	1	1	0.95	gtGTCGGact
HvGSK4.1	P$ERF043_01	ERF043	1925	1933	1	1	0.912	tGTCGGac
HvGSK4.1	P$PHYPA173530_04	HYPA173530	1925	1933	1	1	0.855	tGTCGGac
HvGSK4.1	P$PHYPA28324_10	HYPA28324	1925	1933	1	1	0.868	tGTCGGac
HvGSK4.1	P$O2_Q4	O2	1930	1941	1	1	0.908	gactCATGTct
HvGSK4.1	P$DRE1C_01	DRE1C	1935	1943	1	1	0.862	ATGTCtgc
HvGSK4.1	P$CBNAC_01	CBNAC	1942	1948	1	1	0.973	cTGCTT
HvGSK4.1	P$ASR1_01	ASR1	1980	1985	1	1	1	ACCCA
HvGSK4.1	P$TRB2_01	TRB2	1992	2000	1	1	0.925	acCCCTAt
HvGSK4.1	P$MYB1L_01	MYB1L	1992	2002	1	1	0.947	acCCCTAtta
HvGSK4.1	P$HSFA4A_01	HSFA4A	1995	2001	1	1	0.964	cCTATT
HvGSK4.1	P$GATA15_01	GATA15	2034	2043	1	1	0.999	ttTGATCgt
HvGSK4.1	P$ATHSFA1D_01	ATHSFA1D	2054	2060	1	1	0.941	tCTACA
HvGSK4.1	P$SPF1_Q2	SPF1	2126	2136	1	1	0.856	caATAGTgtg
HvGSK4.1	P$KNOX3_01	KNOX3	2130	2142	1	1	0.976	agtgTGACAatg
HvGSK4.1	P$ATH1_01	ATH1	2134	2142	1	1	0.907	TGACAatg
HvGSK4.1	P$SBF1_01	SBF1	2155	2169	1	1	0.939	tggtgcTTAAAata
HvGSK4.1	P$CBNAC_01	CBNAC	2157	2163	1	1	0.979	gTGCTT
HvGSK4.1	P$CBNAC_02	CBNAC	2157	2173	1	1	0.879	gTGCTTaaaatagcgg
HvGSK4.1	P$SEP3_01	wrz-03	2169	2180	1	1	0.871	gcggtTTTTGg
HvGSK4.1	P$SQUA_01	SQUA	2170	2180	1	1	0.891	cggTTTTTgg
HvGSK4.1	P$GT1_Q6_01	GT1	2173	2185	1	1	0.893	TTTTTggtcaaa
HvGSK4.1	P$WRKY18_02	WRKY18	2176	2186	1	1	0.996	ttgGTCAAaa
HvGSK4.1	P$WRKY21_02	WRKY21	2176	2186	1	1	0.968	ttgGTCAAaa
HvGSK4.1	P$WRKY48_02	WRKY48	2176	2186	1	1	0.998	ttgGTCAAaa
HvGSK4.1	P$WRKY57_01	WRKY57	2176	2186	1	1	0.973	ttgGTCAAaa
HvGSK4.1	P$WRKY60_01	WRKY60	2176	2187	1	1	0.977	ttgGTCAAaaa
HvGSK4.1	P$WRKY15_01	WRKY15	2177	2187	1	1	0.985	tgGTCAAaaa
HvGSK4.1	P$WRKY2_01	WRKY2	2177	2185	1	1	0.989	tgGTCAAa
HvGSK4.1	P$WRKY25_02	WRKY25	2177	2185	1	1	0.973	tgGTCAAa
HvGSK4.1	P$WRKY40_01	WRKY40	2177	2185	1	1	1	tgGTCAAa
HvGSK4.1	P$WRKY43_02	WRKY43	2177	2187	1	1	0.978	tgGTCAAaaa
HvGSK4.1	P$WRKY62_01	WRKY62	2177	2185	1	1	0.957	tgGTCAAa
HvGSK4.1	P$WRKY63_01	WRKY63	2177	2185	1	1	0.99	tgGTCAAa
HvGSK4.1	P$WRKY75_01	WRKY75	2177	2185	1	1	0.975	tgGTCAAa
HvGSK4.1	P$WRKY8_01	WRKY8	2177	2186	1	1	0.992	tgGTCAAaa
HvGSK4.1	P$WRKY23_01	WRKY23	2178	2186	1	1	0.854	gGTCAAaa
HvGSK4.1	P$WRKY30_01	WRKY30	2178	2188	1	1	0.916	gGTCAAaaac
HvGSK4.1	P$WRKY18_Q2	WRKY18	2179	2188	1	1	0.938	GTCAAaaac
HvGSK4.1	P$PBF_Q2	BF	2209	2215	1	1	0.965	gAAAGG
HvGSK4.1	P$AT1G14580_01	AT1G14580	2214	2225	1	1	0.935	gcatcGGGATa
HvGSK4.1	P$AT5G66730_01	AT5G66730	2214	2225	1	1	0.919	gcatcGGGATa
HvGSK4.1	P$PDF2_01	DF2	2219	2230	1	1	0.915	gggaTAAATga
HvGSK4.1	P$NAC078_01	NAC078	2233	2250	1	1	0.86	gttgaagagCAAGAaag
HvGSK4.1	P$ALFIN1_Q2	ALFIN1	2243	2258	1	1	0.882	aagaaaGTGGGccga
HvGSK4.1	P$BPC1_Q2	BPC1	2244	2250	1	1	1	AGAAAg
HvGSK4.1	P$TCP11_01	TCP11	2248	2260	1	1	0.954	aGTGGGccgaag
HvGSK4.1	P$AT1G53910_01	AT1G53910	2251	2261	1	1	0.859	gGGCCGaaga
HvGSK4.1	P$AT2G41690_01	AT2G41690	2254	2260	1	1	0.988	CCGAAg
HvGSK4.1	P$PEND_01	END	2256	2264	1	1	0.874	gAAGAAat
HvGSK4.1	P$BPC1_Q2	BPC1	2258	2264	1	1	0.99	AGAAAt
HvGSK4.1	P$ARR18_01	ARR18	2272	2285	1	1	0.907	ctcaAGATAtccc
HvGSK4.1	P$AT3G60580_01	AT3G60580	2278	2285	1	1	0.951	atATCCC
HvGSK4.1	P$MYB24_01	MYB24	2310	2319	1	1	0.884	ataTTAGGa
HvGSK4.1	P$CBF1_01	CBF1	2342	2352	1	1	0.861	cTGCCGgacc
HvGSK4.1	P$ERF019_01	ERF019	2342	2352	1	1	0.871	cTGCCGgacc
HvGSK4.1	P$JERF3_01	JERF3	2342	2352	1	1	0.885	cTGCCGgacc
HvGSK4.1	P$CEF1_01	CEF1	2342	2352	1	1	0.886	cTGCCGgacc
HvGSK4.1	P$JERF1_01	JERF1	2342	2352	1	1	0.891	cTGCCGgacc
HvGSK4.1	P$CBF1_03	CBF1	2342	2352	1	1	0.898	cTGCCGgacc
HvGSK4.1	P$AT1G33760_01	AT1G33760	2342	2352	1	1	0.867	cTGCCGgacc
HvGSK4.1	P$LEC2_01	LEC2	2349	2360	1	1	0.94	acCATGCtggt
HvGSK4.1	P$ARF8_01	ARF8	2363	2372	1	1	0.952	tgTGTCGag
HvGSK4.1	P$ABF2_01	ABF2	2371	2384	1	1	0.958	gcacaCACGTaca
HvGSK4.1	P$O2_Q4	O2	2372	2383	1	1	0.855	cacaCACGTac
HvGSK4.1	P$GBP_Q6	GBP	2373	2385	1	1	0.885	acaCACGTacat
HvGSK4.1	P$ABI5_01	ABI5	2373	2383	1	1	0.932	acaCACGTac

HvGSK4.1	P$ABF4_01	ABF4	2373	2385	1	1	0.911	acaCACGTacat
HvGSK4.1	P$ATSPL3_01	ATSPL3	2373	2389	1	1	0.965	acacaCGTACattgac
HvGSK4.1	P$CPRF3_Q2	CPRF3	2374	2384	1	1	0.905	caCACGTaca
HvGSK4.1	P$CPRF2_Q2	CPRF2	2374	2384	1	1	0.933	caCACGTaca
HvGSK4.1	P$O2_02	O2	2374	2384	1	1	0.936	caCACGTaca
HvGSK4.1	P$TGA1B_Q2	TGA1B	2374	2384	1	1	0.877	caCACGTaca
HvGSK4.1	P$TGA1A_Q2	TGA1A	2374	2384	1	1	0.966	caCACGTaca
HvGSK4.1	P$CPRF3_01	CPRF3	2374	2384	1	1	0.915	caCACGTaca
HvGSK4.1	P$CPRF2_01	CPRF2	2374	2384	1	1	0.934	caCACGTaca
HvGSK4.1	P$BEE2_01	BEE2	2374	2384	1	1	0.916	caCACGTaca
HvGSK4.1	P$BIM2_01	BIM2	2374	2384	1	1	0.855	caCACGTaca
HvGSK4.1	P$BIM3_01	BIM3	2374	2384	1	1	0.891	caCACGTaca
HvGSK4.1	P$PHYPA143875_02	HYPA143875	2374	2384	1	1	0.877	caCACGTaca
HvGSK4.1	P$SPT_01	SPT	2374	2383	1	1	0.917	caCACGTac
HvGSK4.1	P$RITA1_01	RITA1	2375	2382	1	1	0.974	aCACGTa
HvGSK4.1	P$OCSBF1_01	OCSBF1	2376	2381	1	1	1	CACGT
HvGSK4.1	P$SPL14_03	SPL14	2376	2387	1	1	0.851	caCGTACattg
HvGSK4.1	P$WRKY11_Q2	WRKY11	2383	2391	1	1	0.978	aTTGACct
HvGSK4.1	P$ZAP1_01	ZAP1	2384	2394	1	1	0.893	TTGACctaca
HvGSK4.1	P$ATHSFA1D_01	ATHSFA1D	2388	2394	1	1	0.985	cCTACA
HvGSK4.1	P$ARR18_01	ARR18	2394	2407	1	1	0.888	taatAGATAgtgt
HvGSK4.1	P$SPF1_Q2	SPF1	2398	2408	1	1	0.856	agATAGTgtc
HvGSK4.1	P$CBF3_02	CBF3	2402	2416	1	1	0.915	agtgtCCGACtcat
HvGSK4.1	P$CBF1_04	CBF1	2403	2415	1	1	0.901	gtgtCCGACtca
HvGSK4.1	P$AT1G15360_01	AT1G15360	2404	2414	1	1	0.889	tgTCCGActc
HvGSK4.1	P$ARF5_01	ARF5	2406	2414	1	1	0.88	tCCGACtc
HvGSK4.1	P$DREB1B_01	DREB1B	2407	2412	1	1	1	CCGAC
HvGSK4.1	P$O2_Q4	O2	2409	2420	1	1	0.908	gactCATGTct
HvGSK4.1	P$DRE1C_01	DRE1C	2414	2422	1	1	0.862	ATGTCtgc
HvGSK4.1	P$ASR1_01	ASR1	2459	2464	1	1	1	ACCCA
HvGSK4.1	P$MYB1L_01	MYB1L	2471	2481	1	1	0.947	acCCCTAtta
HvGSK4.1	P$TRB2_01	TRB2	2471	2479	1	1	0.925	acCCCTAt
HvGSK4.1	P$HSFA4A_01	HSFA4A	2474	2480	1	1	0.964	cCTATT
HvGSK4.1	P$GATA15_01	GATA15	2513	2522	1	1	0.999	ttTGATCgt
HvGSK4.1	P$ATHSFA1D_01	ATHSFA1D	2533	2539	1	1	0.941	tCTACA
HvGSK4.1	P$GAMYB_Q2	GAMYB	2546	2559	1	1	0.901	cgcttACAACtta
HvGSK4.1	P$SPF1_Q2	SPF1	2605	2615	1	1	0.856	caATAGTgtg
HvGSK4.1	P$KNOX3_01	KNOX3	2609	2621	1	1	0.975	agtgTGACAaca
HvGSK4.1	P$GAMYB_Q2	GAMYB	2610	2623	1	1	0.913	gtgtgACAACatt
HvGSK4.1	P$ATH1_01	ATH1	2613	2621	1	1	0.927	TGACAaca
HvGSK4.1	P$RAV1_01	RAV1	2613	2625	1	1	0.943	tgaCAACAttat
HvGSK4.1	P$HSFA4A_01	HSFA4A	2624	2630	1	1	0.914	tCTATT
HvGSK4.1	P$SBF1_01	SBF1	2634	2648	1	1	0.93	tgatgcTTAAAata
HvGSK4.1	P$CBNAC_01	CBNAC	2636	2642	1	1	0.968	aTGCTT
HvGSK4.1	P$CBNAC_02	CBNAC	2636	2652	1	1	0.875	aTGCTTaaaatagcgg
HvGSK4.1	P$PBF_01	BF	2659	2670	1	1	0.951	gcaAAAAGatg
HvGSK4.1	P$DOF_Q2	DOF	2659	2670	1	1	0.98	gcaAAAAGatg
HvGSK4.1	P$CDF2_01	CDF2	2660	2670	1	1	0.973	caAAAAGatg
HvGSK4.1	P$CDF3_01	CDF3	2661	2670	1	1	0.973	aAAAAGatg
HvGSK4.1	P$RAMOSA1_01	RAMOSA1	2682	2696	1	1	0.852	gagagtGAGAGgga
HvGSK4.1	P$AT1G14580_01	AT1G14580	2693	2704	1	1	0.935	ggatcGGGATa
HvGSK4.1	P$AT5G66730_01	AT5G66730	2693	2704	1	1	0.919	ggatcGGGATa
HvGSK4.1	P$PDF2_01	DF2	2698	2709	1	1	0.915	gggaTAAATga
HvGSK4.1	P$NAC078_01	NAC078	2712	2729	1	1	0.86	gttgaagagCAAGAaag
HvGSK4.1	P$ALFIN1_Q2	ALFIN1	2722	2737	1	1	0.882	aagaaaGTGGGccga
HvGSK4.1	P$BPC1_Q2	BPC1	2723	2729	1	1	1	AGAAAg
HvGSK4.1	P$TCP11_01	TCP11	2727	2739	1	1	0.954	aGTGGGccgaag
HvGSK4.1	P$AT1G53910_01	AT1G53910	2730	2740	1	1	0.859	gGGCCGaaga
HvGSK4.1	P$AT2G41690_01	AT2G41690	2733	2739	1	1	0.988	CCGAAg
HvGSK4.1	P$PEND_01	END	2735	2743	1	1	0.874	gAAGAAat
HvGSK4.1	P$BPC1_Q2	BPC1	2737	2743	1	1	0.99	AGAAAt
HvGSK4.1	P$KNOX3_01	KNOX3	2738	2750	1	1	0.969	gaaaTGACAatt
HvGSK4.1	P$ATH1_01	ATH1	2742	2750	1	1	0.919	TGACAatt
HvGSK4.1	P$ARR18_01	ARR18	2751	2764	1	1	0.906	ctccAGATAtccc
HvGSK4.1	P$AT3G60580_01	AT3G60580	2757	2764	1	1	0.951	atATCCC
HvGSK4.1	P$AT4G36620_01	AT4G36620	2772	2780	1	1	0.976	ttgAACCA
HvGSK4.1	P$MYB24_01	MYB24	2789	2798	1	1	0.884	ataTTAGGa
HvGSK4.1	P$LEC2_01	LEC2	2828	2839	1	1	0.94	acCATGCtggt
HvGSK4.1	P$GAMYB_Q2	GAMYB	2844	2857	1	1	0.944	ggtcgACAACgca
HvGSK4.1	P$AT5G54070_01	AT5G54070	2849	2855	1	1	0.958	aCAACG
HvGSK4.1	P$ABF2_01	ABF2	2850	2863	1	1	0.906	caacgCACGTaca
HvGSK4.1	P$O2_Q4	O2	2851	2862	1	1	0.854	aacgCACGTac
HvGSK4.1	P$HBI1_01	HBI1	2851	2863	1	1	0.869	aacgCACGTaca
HvGSK4.1	P$GBP_Q6	GBP	2852	2864	1	1	0.88	acgCACGTacat
HvGSK4.1	P$ABI5_01	ABI5	2852	2862	1	1	0.889	acgCACGTac
HvGSK4.1	P$ABF4_01	ABF4	2852	2864	1	1	0.872	acgCACGTacat
HvGSK4.1	P$ATSPL3_01	ATSPL3	2852	2868	1	1	0.939	acgcaCGTACatcagt
HvGSK4.1	P$CPRF3_Q2	CPRF3	2853	2863	1	1	0.896	cgCACGTaca

HvGSK4.1	P$CPRF2_Q2	CPRF2	2853	2863	1	1	0.923	cgCACGTaca
HvGSK4.1	P$O2_02	O2	2853	2863	1	1	0.928	cgCACGTaca
HvGSK4.1	P$TGA1B_Q2	TGA1B	2853	2863	1	1	0.868	cgCACGTaca
HvGSK4.1	P$TGA1A_Q2	TGA1A	2853	2863	1	1	0.964	cgCACGTaca
HvGSK4.1	P$CPRF3_01	CPRF3	2853	2863	1	1	0.904	cgCACGTaca
HvGSK4.1	P$CPRF2_01	CPRF2	2853	2863	1	1	0.924	cgCACGTaca
HvGSK4.1	P$BEE2_01	BEE2	2853	2863	1	1	0.917	cgCACGTaca
HvGSK4.1	P$BIM2_01	BIM2	2853	2863	1	1	0.857	cgCACGTaca
HvGSK4.1	P$BIM3_01	BIM3	2853	2863	1	1	0.896	cgCACGTaca
HvGSK4.1	P$PHYPA143875_02	HYPA143875	2853	2863	1	1	0.879	cgCACGTaca
HvGSK4.1	P$SPT_01	SPT	2853	2862	1	1	0.928	cgCACGTac
HvGSK4.1	P$TSAR1_01	TSAR1	2853	2863	1	1	0.894	cgCACGTaca
HvGSK4.1	P$BHLH78_01	BHLH78	2854	2862	1	1	0.875	GCACGtac
HvGSK4.1	P$RITA1_01	RITA1	2854	2861	1	1	0.964	gCACGTa
HvGSK4.1	P$BHLH3_01	BHLH3	2854	2862	1	1	0.85	gCACGTac
HvGSK4.1	P$AIB_01	AIB	2854	2862	1	1	0.87	gCACGTac
HvGSK4.1	P$OCSBF1_01	OCSBF1	2855	2860	1	1	1	CACGT
HvGSK4.1	P$ATHB7_01	ATHB7	2871	2881	1	1	0.913	caAATCAtcg
HvGSK4.1	P$HAT1_01	HAT1	2871	2881	1	1	0.972	caAATCAtcg
HvGSK4.1	P$SBF1_01	SBF1	2880	2894	1	1	0.889	gtccccTTAATaaa
HvGSK4.1	P$ATHB6_01	ATHB6	2886	2895	1	1	0.91	ttAATAAaa
HvGSK4.1	P$MADSB_Q2	MADSB	2887	2902	1	1	0.858	taatAAAAAtataaa
HvGSK4.1	P$AP1_01	AP1	2889	2902	1	1	0.859	atAAAAAtataaa
HvGSK4.1	P$PBF_01	BF	2896	2907	1	1	0.976	tatAAAAGcta
HvGSK4.1	P$DOF_Q2	DOF	2896	2907	1	1	0.929	tatAAAAGcta
HvGSK4.1	P$DOF2_01	DOF2	2896	2907	1	1	0.992	tataAAAGCta
HvGSK4.1	P$DOF3_01	DOF3	2896	2907	1	1	0.989	tataAAAGCta
HvGSK4.1	P$CDF2_01	CDF2	2897	2907	1	1	0.959	atAAAAGcta
HvGSK4.1	P$CDF3_01	CDF3	2898	2907	1	1	0.977	tAAAAGcta
HvGSK4.1	P$P_01		2902	2911	1	1	0.923	agCTACCca
HvGSK4.1	P$ASR1_01	ASR1	2906	2911	1	1	1	ACCCA
HvGSK4.1	P$ATHB4_02	ATHB4	2910	2920	1	1	0.875	acATCATtgt
HvGSK4.1	P$O2_Q2	O2	2918	2931	1	1	0.929	gtggtGACGTcga
HvGSK4.1	P$TGA1_01	TGA1	2919	2930	1	1	0.994	tggTGACGtcg
HvGSK4.1	P$STF1_02	STF1	2920	2932	1	1	0.961	ggTGACGtcgat
HvGSK4.1	P$TGA3_Q2	TGA3	2920	2929	1	1	0.953	ggTGACGtc
HvGSK4.1	P$TGA6_01	TGA6	2920	2930	1	1	0.975	ggTGACGtcg
HvGSK4.1	P$TGA7_01	TGA7	2920	2930	1	1	0.971	ggTGACGtcg
HvGSK4.1	P$BZIP14_01	BZIP14	2920	2930	1	1	0.955	ggTGACGtcg
HvGSK4.1	P$STF1_01	STF1	2920	2932	1	1	0.98	ggtGACGTcgat
HvGSK4.1	P$TGA5_01	TGA5	2921	2929	1	1	0.995	gTGACGtc
HvGSK4.1	P$TGA1B_01	TGA1B	2921	2931	1	1	0.936	gtGACGTcga
HvGSK4.1	P$TGA1A_01	TGA1A	2923	2930	1	1	0.857	gACGTCg
HvGSK4.1	P$ARR2_01	ARR2	2928	2938	1	1	0.885	cgatATCTTc
HvGSK4.1	P$ATMYB77_01	ATMYB77	2931	2944	1	1	0.857	tatcttCGGTTat
HvGSK4.1	P$SED_Q2	SED	2948	2958	1	1	0.999	attcCCTTTt
HvGSK4.1	P$PBF_Q2_01	BF	2952	2958	1	1	1	CCTTTt
HvGSK4.1	P$MYB3_01	MYB3	2975	2986	1	1	0.902	tgaTAGGTttc
HvGSK4.1	P$MYB4_01	MYB4	2976	2984	1	1	0.871	gaTAGGTt
HvGSK4.1	P$ATMYB15_Q2	ATMYB15	3003	3009	1	1	1	TAACAa
HvGSK4.1	P$ARR18_01	ARR18	3019	3032	1	1	0.913	cacaAGATAtaac
HvGSK4.1	P$WRKY48_01	WRKY48	3025	3034	1	1	0.865	atatAACAA
HvGSK4.1	P$ATMYB15_Q2	ATMYB15	3028	3034	1	1	1	TAACAa
HvGSK4.1	P$PCF2_01	CF2	3042	3052	1	1	0.893	tggatCCCAC
HvGSK4.1	P$TCP19_01	TCP19	3042	3052	1	1	0.92	tggatCCCAC
HvGSK4.1	P$AT3G60580_01	AT3G60580	3043	3050	1	1	0.902	ggATCCC
HvGSK4.1	P$TCP20L_01	TCP20L	3043	3052	1	1	0.893	ggatCCCAC
HvGSK4.1	P$TCP20_02	TCP20	3044	3054	1	1	0.907	gatCCCACta
HvGSK4.1	P$ARALY495258_02	ARALY495258	3044	3052	1	1	0.918	gatCCCAC
HvGSK4.1	P$ARALY484486_05	ARALY484486	3044	3052	1	1	0.918	gatCCCAC
HvGSK4.1	P$ATHSFA1D_01	ATHSFA1D	3050	3056	1	1	1	aCTACA
HvGSK4.1	P$AT5G04240_01	AT5G04240	3059	3065	1	1	0.976	cGGCAC
HvGSK4.1	P$BZR1_01	BZR1	3081	3087	1	1	0.902	CGTGCt
HvGSK4.1	P$RAV1_01	RAV1	3096	3108	1	1	0.975	atgCAACAttaa
HvGSK4.1	P$LEC2_01	LEC2	3106	3117	1	1	0.978	aaCATGCatgc
HvGSK4.1	P$FUS3_Q2	FUS3	3107	3118	1	1	0.984	aCATGCatgca
HvGSK4.1	P$ABI3_01	ABI3	3109	3118	1	1	0.97	atGCATGca
HvGSK4.1	P$FUS3_01	FUS3	3110	3119	1	1	0.965	tGCATGcaa
HvGSK4.1	P$LEC2_01	LEC2	3110	3121	1	1	0.99	tgCATGCaaaa
HvGSK4.1	P$SBF1_01	SBF1	3128	3142	1	1	0.853	gtttctTTAATcac
HvGSK4.1	P$EDT1_01	EDT1	3131	3141	1	1	0.853	tctTTAATca
HvGSK4.1	P$ATHB7_01	ATHB7	3134	3144	1	1	0.856	ttAATCAcat
HvGSK4.1	P$HAT1_01	HAT1	3134	3144	1	1	0.862	ttAATCAcat
HvGSK4.1	P$LEC2_01	LEC2	3168	3179	1	1	0.98	gaCATGCagca
HvGSK4.1	P$PDF2_01	DF2	3175	3186	1	1	0.872	agcaTAAATcc
HvGSK4.1	P$AT3G60580_01	AT3G60580	3180	3187	1	1	0.883	aaATCCC
HvGSK4.1	P$PEND_02	END	3186	3196	1	1	0.853	ccTTCTTttt
HvGSK4.1	P$ABI3_01	ABI3	3199	3208	1	1	0.959	acGCATGca

HvGSK4.1	P$FUS3_01	FUS3	3200	3209	1	1	0.982	cGCATGcat
HvGSK4.1	P$LEC2_01	LEC2	3200	3211	1	1	0.979	cgCATGCatgc
HvGSK4.1	P$FUS3_Q2	FUS3	3201	3212	1	1	1	gCATGCatgca
HvGSK4.1	P$ABI3_01	ABI3	3203	3212	1	1	0.97	atGCATGca
HvGSK4.1	P$FUS3_01	FUS3	3204	3213	1	1	0.965	tGCATGcaa
HvGSK4.1	P$LEC2_01	LEC2	3204	3215	1	1	0.984	tgCATGCaatg
HvGSK4.1	P$FUS3_Q2	FUS3	3205	3216	1	1	0.851	gCATGCaatga
HvGSK4.1	P$TGA1_02	TGA1	3208	3223	1	1	0.946	tgcaatgACGTCatc
HvGSK4.1	P$HBP1B_Q6	HBP1B	3208	3222	1	1	0.856	tgcaatgaCGTCAt
HvGSK4.1	P$O2_Q2	O2	3209	3222	1	1	0.893	gcaatGACGTcat
HvGSK4.1	P$TGA1_01	TGA1	3210	3221	1	1	0.981	caaTGACGtca
HvGSK4.1	P$TGA7_02	TGA7	3210	3227	1	1	0.972	caatgACGTCatcaaca
HvGSK4.1	P$STF1_02	STF1	3211	3223	1	1	0.994	aaTGACGtcatc
HvGSK4.1	P$TGA2_01	TGA2	3211	3224	1	1	0.986	aaTGACGtcatca
HvGSK4.1	P$TGA3_Q2	TGA3	3211	3220	1	1	0.927	aaTGACGtc
HvGSK4.1	P$TGA6_01	TGA6	3211	3221	1	1	1	aaTGACGtca
HvGSK4.1	P$TGA7_01	TGA7	3211	3221	1	1	0.99	aaTGACGtca
HvGSK4.1	P$TGA5_02	TGA5	3211	3225	1	1	0.998	aaTGACGtcatcaa
HvGSK4.1	P$TGA6_02	TGA6	3211	3226	1	1	0.998	aaTGACGtcatcaac
HvGSK4.1	P$BZIP14_01	BZIP14	3211	3221	1	1	0.969	aaTGACGtca
HvGSK4.1	P$STF1_01	STF1	3211	3223	1	1	0.982	aatGACGTcatc
HvGSK4.1	P$TGA5_01	TGA5	3212	3220	1	1	1	aTGACGtc
HvGSK4.1	P$TGA1B_01	TGA1B	3212	3222	1	1	0.925	atGACGTcat
HvGSK4.1	P$TGA1A_01	TGA1A	3214	3221	1	1	0.975	gACGTCa
HvGSK4.1	P$TGA2_Q2	TGA2	3215	3225	1	1	0.969	aCGTCAtcaa
HvGSK4.1	P$RAV1_01	RAV1	3219	3231	1	1	0.924	catCAACAttcc
HvGSK4.1	P$SED_Q2	SED	3226	3236	1	1	0.985	attcCCTTTa
HvGSK4.1	P$PBF_Q2_01	BF	3230	3236	1	1	0.998	CCTTTa
HvGSK4.1	P$EDT1_01	EDT1	3230	3240	1	1	0.955	cctTTAATgt
HvGSK4.1	P$PBF_01	BF	3247	3258	1	1	0.949	tcaAAAAGttt
HvGSK4.1	P$DOF_Q2	DOF	3247	3258	1	1	0.975	tcaAAAAGttt
HvGSK4.1	P$CDF2_01	CDF2	3248	3258	1	1	0.994	caAAAAGttt
HvGSK4.1	P$CDF3_01	CDF3	3249	3258	1	1	0.989	aAAAAGttt
HvGSK4.1	P$SBF1_01	SBF1	3266	3280	1	1	0.855	aaacgaTTAATttg
HvGSK4.1	P$EDT1_01	EDT1	3269	3279	1	1	0.865	cgaTTAATtt
HvGSK4.1	P$GATA15_01	GATA15	3276	3285	1	1	0.999	ttTGATCga
HvGSK4.1	P$O2_03	O2	3283	3293	1	1	0.91	GATGAtctgt
HvGSK4.1	P$GATA15_01	GATA15	3283	3292	1	1	0.999	gaTGATCtg
HvGSK4.1	P$GATA11_01	GATA11	3284	3292	1	1	0.866	atGATCTg
HvGSK4.1	P$GATA8_01	GATA8	3284	3293	1	1	0.985	atGATCTgt
HvGSK4.1	P$ID1_01	ID1	3300	3311	1	1	0.866	aTTGTCtttat
HvGSK4.1	P$GATA9_01	GATA9	3315	3326	1	1	0.88	atgAGATCatc
HvGSK4.1	P$AGP1_01	AGP1	3316	3326	1	1	0.859	tgAGATCatc
HvGSK4.1	P$ARR10_01	ARR10	3318	3325	1	1	0.913	AGATCat
HvGSK4.1	P$AT3G60580_01	AT3G60580	3321	3328	1	1	0.851	tcATCCC
HvGSK4.1	P$KNOX3_01	KNOX3	3328	3340	1	1	0.959	atatTGACAtat
HvGSK4.1	P$WRKY11_Q2	WRKY11	3330	3338	1	1	0.903	aTTGACat
HvGSK4.1	P$ATH1_01	ATH1	3332	3340	1	1	0.935	TGACAtat
HvGSK4.1	P$KNOX3_01	KNOX3	3338	3350	1	1	0.959	atttTGACAact
HvGSK4.1	P$GAMYB_Q2	GAMYB	3339	3352	1	1	0.902	ttttgACAACttt
HvGSK4.1	P$WRKY11_Q2	WRKY11	3340	3348	1	1	0.924	tTTGACaa
HvGSK4.1	P$ATH1_01	ATH1	3342	3350	1	1	0.926	TGACAact
HvGSK4.1	P$MYB3R5_01	MYB3R5	3347	3362	1	1	0.881	acttttttCCGTTc
HvGSK4.1	P$SQUA_01	SQUA	3348	3358	1	1	0.855	cttTTTTTcc
HvGSK4.1	P$MYB3R1_01	MYB3R1	3348	3363	1	1	0.9	ctttttttCCGTTca
HvGSK4.1	P$MYB3R4_01	MYB3R4	3348	3363	1	1	0.899	ctttttttCCGTTca
HvGSK4.1	P$ATHB1_01	ATHB1	3368	3382	1	1	0.888	gccacATTATtgca
HvGSK4.1	P$ATHB5_01	ATHB5	3371	3380	1	1	0.862	acaTTATTg
HvGSK4.1	P$ID1_01	ID1	3386	3397	1	1	0.871	cTTGTCatatt
HvGSK4.1	P$GT1_Q6	GT1	3398	3405	1	1	0.912	GTAAAca
HvGSK4.1	P$CBNAC_01	CBNAC	3410	3416	1	1	1	tTGCTT
HvGSK4.1	P$CBNAC_02	CBNAC	3410	3426	1	1	0.883	tTGCTTgataaaatat
HvGSK4.1	P$C1_Q2	C1	3424	3435	1	1	0.92	atAACTAaaat
HvGSK4.1	P$O2_Q4	O2	3433	3444	1	1	0.885	atgcCATGTtt
HvGSK4.1	P$HMG1_01	HMG1	3470	3479	1	1	0.936	GTTGTcatt
HvGSK4.1	P$ID1_01	ID1	3470	3481	1	1	0.873	gTTGTCatttt
HvGSK4.1	P$SQUA_01	SQUA	3475	3485	1	1	0.859	catTTTTTat
HvGSK4.1	P$MYBPH3_02	MYBPH3	3503	3516	1	1	0.859	gcaattTAGTTat
HvGSK4.1	P$PBF_01	BF	3546	3557	1	1	0.982	atgAAAAGcaa
HvGSK4.1	P$DOF_Q2	DOF	3546	3557	1	1	0.959	atgAAAAGcaa
HvGSK4.1	P$DOF2_01	DOF2	3546	3557	1	1	0.992	atgaAAAGCaa
HvGSK4.1	P$DOF3_01	DOF3	3546	3557	1	1	0.982	atgaAAAGCaa
HvGSK4.1	P$CDF2_01	CDF2	3547	3557	1	1	0.961	tgAAAAGcaa
HvGSK4.1	P$CDF3_01	CDF3	3548	3557	1	1	0.979	gAAAAGcaa
HvGSK4.1	P$RAV1_01	RAV1	3557	3569	1	1	0.972	gtgCAACAgtta
HvGSK4.1	P$ATMYB77_01	ATMYB77	3557	3570	1	1	0.891	gtgcaaCAGTTat
HvGSK4.1	P$KNOX3_01	KNOX3	3597	3609	1	1	0.969	aataTGACAagt
HvGSK4.1	P$ATH1_01	ATH1	3601	3609	1	1	0.931	TGACAagt

HvGSK4.1	P$RAV1_01	RAV1	3611	3623	1	1	0.956	gtgCAACAatgt
HvGSK4.1	P$SQUA_01	SQUA	3635	3645	1	1	0.871	cgaTTTTTtt
HvGSK4.1	P$GT1_Q6_01	GT1	3638	3650	1	1	0.875	TTTTTttttgtc
HvGSK4.1	P$GT1_Q6_01	GT1	3641	3653	1	1	0.923	TTTTTtgtcaaa
HvGSK4.1	P$WRKY18_02	WRKY18	3644	3654	1	1	0.945	tttGTCAAaa
HvGSK4.1	P$WRKY21_02	WRKY21	3644	3654	1	1	0.955	tttGTCAAaa
HvGSK4.1	P$WRKY48_02	WRKY48	3644	3654	1	1	0.987	tttGTCAAaa
HvGSK4.1	P$WRKY57_01	WRKY57	3644	3654	1	1	0.962	tttGTCAAaa
HvGSK4.1	P$WRKY60_01	WRKY60	3644	3655	1	1	0.898	tttGTCAAaac
HvGSK4.1	P$WRKY15_01	WRKY15	3645	3655	1	1	0.964	ttGTCAAaac
HvGSK4.1	P$WRKY2_01	WRKY2	3645	3653	1	1	0.909	ttGTCAAa
HvGSK4.1	P$WRKY25_02	WRKY25	3645	3653	1	1	0.892	ttGTCAAa
HvGSK4.1	P$WRKY40_01	WRKY40	3645	3653	1	1	0.977	ttGTCAAa
HvGSK4.1	P$WRKY43_02	WRKY43	3645	3655	1	1	0.955	ttGTCAAaac
HvGSK4.1	P$WRKY62_01	WRKY62	3645	3653	1	1	0.895	ttGTCAAa
HvGSK4.1	P$WRKY63_01	WRKY63	3645	3653	1	1	0.886	ttGTCAAa
HvGSK4.1	P$WRKY75_01	WRKY75	3645	3653	1	1	0.936	ttGTCAAa
HvGSK4.1	P$WRKY8_01	WRKY8	3645	3654	1	1	0.98	ttGTCAAaa
HvGSK4.1	P$WRKY30_01	WRKY30	3646	3656	1	1	0.899	tGTCAAaaca
HvGSK4.1	P$WRKY18_Q2	WRKY18	3647	3656	1	1	0.95	GTCAAaaca
HvGSK4.1	P$O2_Q4	O2	3650	3661	1	1	0.937	aaaaCATGTca
HvGSK4.1	P$WRKY18_02	WRKY18	3654	3664	1	1	0.945	catGTCAAta
HvGSK4.1	P$WRKY21_02	WRKY21	3654	3664	1	1	0.949	catGTCAAta
HvGSK4.1	P$WRKY48_02	WRKY48	3654	3664	1	1	0.986	catGTCAAta
HvGSK4.1	P$WRKY57_01	WRKY57	3654	3664	1	1	0.959	catGTCAAta
HvGSK4.1	P$WRKY60_01	WRKY60	3654	3665	1	1	0.883	catGTCAAtat
HvGSK4.1	P$WRKY15_01	WRKY15	3655	3665	1	1	0.958	atGTCAAtat
HvGSK4.1	P$WRKY2_01	WRKY2	3655	3663	1	1	0.902	atGTCAAt
HvGSK4.1	P$WRKY25_02	WRKY25	3655	3663	1	1	0.879	atGTCAAt
HvGSK4.1	P$WRKY40_01	WRKY40	3655	3663	1	1	0.977	atGTCAAt
HvGSK4.1	P$WRKY43_02	WRKY43	3655	3665	1	1	0.954	atGTCAAtat
HvGSK4.1	P$WRKY63_01	WRKY63	3655	3663	1	1	0.885	atGTCAAt
HvGSK4.1	P$WRKY75_01	WRKY75	3655	3663	1	1	0.927	atGTCAAt
HvGSK4.1	P$WRKY8_01	WRKY8	3655	3664	1	1	0.977	atGTCAAta
HvGSK4.1	P$WRKY30_01	WRKY30	3656	3666	1	1	0.903	tGTCAAtatg
HvGSK4.1	P$WRKY18_Q2	WRKY18	3657	3666	1	1	0.964	GTCAAtatg
HvGSK4.1	P$GATA8_01	GATA8	3665	3674	1	1	0.988	ggGATCTag
HvGSK4.1	P$GATA9_01	GATA9	3677	3688	1	1	0.978	cgaAGATCtca
HvGSK4.1	P$AGP1_01	AGP1	3678	3688	1	1	0.926	gaAGATCtca
HvGSK4.1	P$GATA10_01	GATA10	3679	3687	1	1	0.918	aAGATCtc
HvGSK4.1	P$GATA11_01	GATA11	3679	3687	1	1	0.949	aaGATCTc
HvGSK4.1	P$GATA8_01	GATA8	3679	3688	1	1	0.991	aaGATCTca
HvGSK4.1	P$ARR10_01	ARR10	3680	3687	1	1	0.913	AGATCtc
HvGSK4.1	P$GT1_Q6	GT1	3705	3712	1	1	0.971	GTGAAaa
HvGSK4.1	P$GATA9_01	GATA9	3710	3721	1	1	0.887	aacAGATCatt
HvGSK4.1	P$AGP1_01	AGP1	3711	3721	1	1	0.865	acAGATCatt
HvGSK4.1	P$ARR10_01	ARR10	3713	3720	1	1	0.913	AGATCat
HvGSK4.1	P$ATHB4_02	ATHB4	3713	3723	1	1	0.872	agATCATtga
HvGSK4.1	P$GATA15_01	GATA15	3718	3727	1	1	0.999	atTGATCaa
HvGSK4.1	P$ARR18_01	ARR18	3737	3750	1	1	0.897	ttagAGATAaaac
HvGSK4.1	P$SBF1_01	SBF1	3747	3761	1	1	0.855	aactttTTAAAttt
HvGSK4.1	P$AT1G59810_01	AT1G59810	3750	3766	1	1	0.881	ttttTAAATttcaaac
HvGSK4.1	P$AGL20_01	AGL20	3752	3764	1	1	0.898	ttTAAATttcaa
HvGSK4.1	P$AGL12_01	AGL12	3752	3764	1	1	0.937	tttAAATTtcaa
HvGSK4.1	P$AT2G26320_01	AT2G26320	3753	3764	1	1	0.864	TTAAAtttcaa
HvGSK4.1	P$ARR2_01	ARR2	3772	3782	1	1	0.972	gaggATCTTg
HvGSK4.1	P$GATA8_01	GATA8	3773	3782	1	1	0.977	agGATCTtg
HvGSK4.1	P$KNOX3_01	KNOX3	3779	3791	1	1	0.961	ttgaTGACAtca
HvGSK4.1	P$ATH1_01	ATH1	3783	3791	1	1	0.941	TGACAtca
HvGSK4.1	P$ATHB4_02	ATHB4	3785	3795	1	1	0.877	acATCATtgc
HvGSK4.1	P$ABI3_01	ABI3	3791	3800	1	1	0.98	ttGCATGca
HvGSK4.1	P$FUS3_01	FUS3	3792	3801	1	1	0.965	tGCATGcat
HvGSK4.1	P$LEC2_01	LEC2	3792	3803	1	1	0.981	tgCATGCatga
HvGSK4.1	P$FUS3_Q2	FUS3	3793	3804	1	1	0.97	gCATGCatgat
HvGSK4.1	P$IDEF1_Q2	IDEF1	3794	3806	1	1	0.874	CATGCatgattg
HvGSK4.1	P$ABI3_01	ABI3	3795	3804	1	1	0.864	atGCATGat
HvGSK4.1	P$RAMOSA1_01	RAMOSA1	3805	3819	1	1	0.886	ggtagaGAGAGaaa
HvGSK4.1	P$BPC1_Q2	BPC1	3814	3820	1	1	1	AGAAAg
HvGSK4.1	P$AT5G04240_01	AT5G04240	3826	3832	1	1	0.976	cGGCAC
HvGSK4.1	P$AMS_01	AMS	3831	3841	1	1	0.878	caCATGTgct
HvGSK4.1	P$CBNAC_01	CBNAC	3836	3842	1	1	0.979	gTGCTT
HvGSK4.1	P$ABI3_01	ABI3	3840	3849	1	1	0.873	ttGCATGac
HvGSK4.1	P$AT3G18650_01	AT3G18650	3846	3857	1	1	0.898	gacttTTGTAt
HvGSK4.1	P$GT1_Q6_01	GT1	3850	3862	1	1	0.974	TTTGTatttaat
HvGSK4.1	P$SBF1_01	SBF1	3851	3865	1	1	0.912	ttgtatTTAATtaa
HvGSK4.1	P$EDT1_01	EDT1	3854	3864	1	1	0.892	tatTTAATta
HvGSK4.1	P$GT1_Q6_02	GT1	3855	3867	1	1	0.855	atttaaTTAACa
HvGSK4.1	P$ATMYB15_Q2	ATMYB15	3862	3868	1	1	1	TAACAa

HvGSK4.1	P$ABI3_01	ABI3	3876	3885	1	1	0.863	taGCATGat
HvGSK4.1	P$LEC2_01	LEC2	3890	3901	1	1	0.978	caCATGCagcg
HvGSK4.1	P$AT3G63350_01	AT3G63350	3902	3908	1	1	0.882	CCGCCa
HvGSK4.1	P$NAC92_01	NAC92	3905	3917	1	1	0.961	ccACACGaaaag
HvGSK4.1	P$PBF_01	BF	3909	3920	1	1	0.965	acgAAAAGtcc
HvGSK4.1	P$DOF_Q2	DOF	3909	3920	1	1	0.945	acgAAAAGtcc
HvGSK4.1	P$CDF2_01	CDF2	3910	3920	1	1	0.965	cgAAAAGtcc
HvGSK4.1	P$CDF3_01	CDF3	3911	3920	1	1	0.983	gAAAAGtcc
HvGSK4.1	P$AT3G20750_01	AT3G20750	3969	3977	1	1	0.888	aTAAACag
HvGSK4.1	P$AT2G26880_01	AT2G26880	3977	3991	1	1	0.868	ctttattTTTAAac
HvGSK4.1	P$SBF1_01	SBF1	3979	3993	1	1	0.872	ttatttTTAAAcat
HvGSK4.1	P$AT3G20750_01	AT3G20750	3985	3993	1	1	0.969	tTAAACat
OsSK11	P$AT1G14580_01	AT1G14580	2	13	1	1	0.933	ggatgGGGATa
OsSK11	P$AT5G66730_01	AT5G66730	2	13	1	1	0.918	ggatgGGGATa
OsSK11	P$AT4G01720_01	AT4G01720	14	23	1	1	0.867	aGTAAAatc
OsSK11	P$ATHB7_01	ATHB7	17	27	1	1	0.882	aaAATCAata
OsSK11	P$HAT1_01	HAT1	17	27	1	1	0.876	aaAATCAata
OsSK11	P$SBF1_01	SBF1	23	37	1	1	0.872	aatataTTAAAaac
OsSK11	P$PBF_01	BF	43	54	1	1	0.979	aagAAAAGaaa
OsSK11	P$DOF_Q2	DOF	43	54	1	1	0.963	aagAAAAGaaa
OsSK11	P$BPC1_Q2	BPC1	44	50	1	1	0.997	AGAAAa
OsSK11	P$CDF2_01	CDF2	44	54	1	1	0.951	agAAAAGaaa
OsSK11	P$CDF3_01	CDF3	45	54	1	1	0.971	gAAAAGaaa
OsSK11	P$BPC1_Q2	BPC1	49	55	1	1	0.997	AGAAAa
OsSK11	P$ATHB6_01	ATHB6	51	60	1	1	0.975	aaAATAAta
OsSK11	P$ATHB5_04	ATHB5	51	62	1	1	0.888	aaAATAAtaat
OsSK11	P$ATHB1_03	ATHB1	51	62	1	1	0.891	aaAATAAtaat
OsSK11	P$ATHB16_01	ATHB16	52	60	1	1	0.867	aAATAAta
OsSK11	P$ATHB6_01	ATHB6	54	63	1	1	0.981	atAATAAta
OsSK11	P$ATHB5_04	ATHB5	54	65	1	1	0.91	atAATAAtaga
OsSK11	P$ATHB1_03	ATHB1	54	65	1	1	0.905	atAATAAtaga
OsSK11	P$ATHB16_01	ATHB16	55	63	1	1	0.915	tAATAAta
OsSK11	P$AT4G36620_01	AT4G36620	64	72	1	1	0.907	agcAACCA
OsSK11	P$GAMYB_01	GAMYB	66	74	1	1	0.886	CAACCaga
OsSK11	P$GATA9_01	GATA9	68	79	1	1	0.992	accAGATCtag
OsSK11	P$AGP1_01	AGP1	69	79	1	1	0.997	ccAGATCtag
OsSK11	P$GATA10_01	GATA10	70	78	1	1	0.916	cAGATCta
OsSK11	P$GATA11_01	GATA11	70	78	1	1	0.939	caGATCTa
OsSK11	P$GATA8_01	GATA8	70	79	1	1	1	caGATCTag
OsSK11	P$ARR10_01	ARR10	71	78	1	1	0.913	AGATCta
OsSK11	P$MRP1_Q2	MRP1	91	103	1	1	0.872	gcTCTATcttta
OsSK11	P$ARR2_01	ARR2	92	102	1	1	0.86	ctctATCTTt
OsSK11	P$PBF_Q2	BF	115	121	1	1	0.965	gAAAGG
OsSK11	P$SQUA_01	SQUA	121	131	1	1	0.895	ctcTTTTTag
OsSK11	P$MYBAS1_01	MYBAS1	144	155	1	1	0.953	caCCAACcggg
OsSK11	P$GAMYB_01	GAMYB	147	155	1	1	0.92	CAACCggg
OsSK11	P$DOF1_01	DOF1	154	165	1	1	0.981	gacTAAAGatg
OsSK11	P$TEIL_01	TEIL	162	170	1	1	0.925	ATGTAttt
OsSK11	P$RAV1_02	RAV1	184	196	1	1	0.997	ttcACCTGggac
OsSK11	P$DOF1_01	DOF1	193	204	1	1	0.986	gacTAAAGatc
OsSK11	P$GATA9_01	GATA9	196	207	1	1	0.884	taaAGATCgtc
OsSK11	P$AGP1_01	AGP1	197	207	1	1	0.855	aaAGATCgtc
OsSK11	P$ARR10_01	ARR10	199	206	1	1	0.913	AGATCgt
OsSK11	P$ATHSFA1D_01	ATHSFA1D	238	244	1	1	1	aCTACA
OsSK11	P$DOF1_01	DOF1	260	271	1	1	0.983	gagTAAAGccc
OsSK11	P$DOF2_01	DOF2	260	271	1	1	0.994	gagtAAAGCcc
OsSK11	P$DOF3_01	DOF3	260	271	1	1	0.997	gagtAAAGCcc
OsSK11	P$SED_Q2	SED	266	276	1	1	0.916	agccCCTTTc
OsSK11	P$PBF_Q2_01	BF	270	276	1	1	0.985	CCTTTc
OsSK11	P$LIM1_01	LIM1	277	289	1	1	0.896	CCACCagtggaa
OsSK11	P$BHLH64_02	BHLH64	279	285	1	1	1	ACCAGt
OsSK11	P$C1_Q2	C1	285	296	1	1	0.92	ggAACTAaaca
OsSK11	P$ARR1_01	ARR1	314	324	1	1	0.978	catGAATCtt
OsSK11	P$ARR2_01	ARR2	315	325	1	1	0.966	atgaATCTTt
OsSK11	P$AG_02	AG	325	341	1	1	0.852	TTACCctgttctgtaa
OsSK11	P$PBF_01	BF	336	347	1	1	0.958	tgtAAAAGgaa
OsSK11	P$DOF_Q2	DOF	336	347	1	1	0.944	tgtAAAAGgaa
OsSK11	P$CDF2_01	CDF2	337	347	1	1	0.955	gtAAAAGgaa
OsSK11	P$CDF3_01	CDF3	338	347	1	1	0.973	tAAAAGgaa
OsSK11	P$PBF_Q2	BF	339	345	1	1	1	aAAAGG
OsSK11	P$AT2G41690_01	AT2G41690	358	364	1	1	1	CCGAAc
OsSK11	P$MYB3R5_01	MYB3R5	359	374	1	1	0.858	cgaacccatCCGTTt
OsSK11	P$MYB3R1_01	MYB3R1	360	375	1	1	0.874	gaacccatCCGTTtt
OsSK11	P$MYB3R4_01	MYB3R4	360	375	1	1	0.865	gaacccatCCGTTtt
OsSK11	P$ASR1_01	ASR1	362	367	1	1	1	ACCCA
OsSK11	P$PBF_01	BF	385	396	1	1	0.962	tgcAAAAGaaa
OsSK11	P$DOF_Q2	DOF	385	396	1	1	0.936	tgcAAAAGaaa
OsSK11	P$CDF2_01	CDF2	386	396	1	1	0.947	gcAAAAGaaa

OsSK11	P$CDF3_01	CDF3	387	396	1	1	0.969	cAAAAGaaa
OsSK11	P$PBF_01	BF	390	401	1	1	0.979	aagAAAAGtaa
OsSK11	P$DOF_Q2	DOF	390	401	1	1	0.963	aagAAAAGtaa
OsSK11	P$BPC1_Q2	BPC1	391	397	1	1	0.997	AGAAAa
OsSK11	P$CDF2_01	CDF2	391	401	1	1	0.972	agAAAAGtaa
OsSK11	P$CDF3_01	CDF3	392	401	1	1	0.986	gAAAAGtaa
OsSK11	P$GT1_Q6_01	GT1	401	413	1	1	0.875	TTTGTaaataaa
OsSK11	P$GT1_Q6	GT1	404	411	1	1	1	GTAAAta
OsSK11	P$ATHB6_01	ATHB6	405	414	1	1	0.905	taAATAAaa
OsSK11	P$GT1_Q6_01	GT1	428	440	1	1	0.871	TTTTTagtgatc
OsSK11	P$GATA15_01	GATA15	433	442	1	1	0.999	agTGATCta
OsSK11	P$GATA8_01	GATA8	434	443	1	1	0.987	gtGATCTaa
OsSK11	P$PBF_01	BF	438	449	1	1	0.97	tctAAAAGcaa
OsSK11	P$DOF_Q2	DOF	438	449	1	1	0.945	tctAAAAGcaa
OsSK11	P$DOF2_01	DOF2	438	449	1	1	0.993	tctaAAAGCaa
OsSK11	P$DOF3_01	DOF3	438	449	1	1	0.986	tctaAAAGCaa
OsSK11	P$CDF2_01	CDF2	439	449	1	1	0.962	ctAAAAGcaa
OsSK11	P$CDF3_01	CDF3	440	449	1	1	0.978	tAAAAGcaa
OsSK11	P$PBF_Q2	BF	446	452	1	1	0.958	cAAAGG
OsSK11	P$AT3G51080_01	AT3G51080	454	461	1	1	0.893	GGAAAat
OsSK11	P$ATHB6_01	ATHB6	456	465	1	1	0.902	aaAATAAac
OsSK11	P$AT3G20750_01	AT3G20750	459	467	1	1	0.938	aTAAACtt
OsSK11	P$HSFA2_01	HSFA2	481	487	1	1	0.922	CCAAAt
OsSK11	P$ATHB7_01	ATHB7	482	492	1	1	0.873	caAATCAact
OsSK11	P$HAT1_01	HAT1	482	492	1	1	0.866	caAATCAact
OsSK11	P$ATHSFA1D_01	ATHSFA1D	491	497	1	1	0.941	tCTACA
OsSK11	P$RIN_Q2	RIN	493	504	1	1	0.861	tacaTTTAAgg
OsSK11	P$MADSB_Q2	MADSB	502	517	1	1	0.874	ggctAAAAAtttaaa
OsSK11	P$AGL12_01	AGL12	511	523	1	1	0.852	tttAAATTttgg
OsSK11	P$PBF_01	BF	542	553	1	1	0.956	gcgAAAAGacg
OsSK11	P$DOF_Q2	DOF	542	553	1	1	0.943	gcgAAAAGacg
OsSK11	P$CDF2_01	CDF2	543	553	1	1	0.943	cgAAAAGacg
OsSK11	P$CDF3_01	CDF3	544	553	1	1	0.966	gAAAAGacg
OsSK11	P$SBF1_01	SBF1	584	598	1	1	0.861	atcaatTTAATaaa
OsSK11	P$EDT1_01	EDT1	587	597	1	1	0.883	aatTTAATaa
OsSK11	P$ATHB6_01	ATHB6	590	599	1	1	0.914	ttAATAAat
OsSK11	P$PDF2_01	DF2	590	601	1	1	0.904	ttaaTAAATgt
OsSK11	P$TEIL_01	TEIL	597	605	1	1	0.925	ATGTAttt
OsSK11	P$ATHB6_01	ATHB6	609	618	1	1	0.908	tgAATAAgt
OsSK11	P$MYB24_01	MYB24	614	623	1	1	0.923	aagTTAGGa
OsSK11	P$MYBAS1_01	MYBAS1	623	634	1	1	0.952	agCTAACatga
OsSK11	P$GT1_Q6_02	GT1	638	650	1	1	0.968	gttttcTTAACa
OsSK11	P$AT1G66560_01	AT1G66560	641	651	1	1	0.964	ttcTTAACat
OsSK11	P$WRKY21_01	WRKY21	642	651	1	1	0.937	tcTTAACat
OsSK11	P$WRKY43_01	WRKY43	642	651	1	1	0.927	tcTTAACat
OsSK11	P$AT4G11070_01	AT4G11070	642	651	1	1	0.992	tcTTAACat
OsSK11	P$AT1G18860_01	AT1G18860	642	651	1	1	0.94	tcTTAACat
OsSK11	P$AT1G64000_01	AT1G64000	642	651	1	1	0.922	tcTTAACat
OsSK11	P$WRKY6_01	WRKY6	642	651	1	1	0.877	tcTTAACat
OsSK11	P$AT1G66600_01	AT1G66600	642	651	1	1	0.962	tcTTAACat
OsSK11	P$AT1G68150_01	AT1G68150	642	651	1	1	0.909	tcTTAACat
OsSK11	P$AT5G41570_01	AT5G41570	642	651	1	1	0.922	tcTTAACat
OsSK11	P$AT5G15130_01	AT5G15130	642	651	1	1	0.936	tcTTAACat
OsSK11	P$WRKY46_01	WRKY46	642	651	1	1	0.974	tcTTAACat
OsSK11	P$AT2G24570_01	AT2G24570	642	651	1	1	0.929	tcTTAACat
OsSK11	P$WRKY7_01	WRKY7	642	651	1	1	0.935	tcTTAACat
OsSK11	P$ABF4_Q2	ABF4	645	659	1	1	0.921	taacatACGTGtag
OsSK11	P$ABZ1_01	ABZ1	646	660	1	1	0.897	aacatACGTGtagt
OsSK11	P$BZR1_03	BZR1	647	667	1	1	0.859	acatACGTGtagtaagtttt
OsSK11	P$HBP1A_Q2	HBP1A	648	658	1	1	0.865	catACGTGta
OsSK11	P$TAF1_Q2	TAF1	648	658	1	1	0.916	catACGTGta
OsSK11	P$EMBP1_02	EMBP1	648	658	1	1	0.918	catACGTGta
OsSK11	P$TAF1_01	TAF1	648	658	1	1	0.941	catACGTGta
OsSK11	P$GBF1_01	GBF1	649	657	1	1	0.973	atACGTGt
OsSK11	P$BIM1_02	BIM1	649	659	1	1	0.945	atACGTGtag
OsSK11	P$ABF4_02	ABF4	649	659	1	1	0.994	atACGTGtag
OsSK11	P$ABI5_Q2	ABI5	651	657	1	1	0.979	ACGTGt
OsSK11	P$GATA15_01	GATA15	675	684	1	1	0.999	tgTGATCca
OsSK11	P$ARF8_01	ARF8	684	693	1	1	0.956	tcTGTCGac
OsSK11	P$AZF2_01	AZF2	692	704	1	1	0.992	cgattgAAGTAg
OsSK11	P$GAMYB_Q2	GAMYB	703	716	1	1	0.917	gacagACAACcat
OsSK11	P$AT4G36620_01	AT4G36620	707	715	1	1	0.903	gacAACCA
OsSK11	P$GAMYB_01	GAMYB	709	717	1	1	0.878	CAACCatg
OsSK11	P$PDF2_01	DF2	714	725	1	1	0.9	atggTAAATgc
OsSK11	P$PBF_01	BF	735	746	1	1	0.991	caaAAAAGaga
OsSK11	P$DOF_Q2	DOF	735	746	1	1	0.984	caaAAAAGaga
OsSK11	P$CDF2_01	CDF2	736	746	1	1	0.979	aaAAAAGaga
OsSK11	P$CDF3_01	CDF3	737	746	1	1	0.984	aAAAAGaga

OsSK11	P$ABI3_01	ABI3	749	758	1	1	0.953	ggGCATGct
OsSK11	P$FUS3_01	FUS3	750	759	1	1	0.943	gGCATGctt
OsSK11	P$LEC2_01	LEC2	750	761	1	1	0.949	ggCATGCttat
OsSK11	P$IDEF1_Q2	IDEF1	752	764	1	1	0.852	CATGCttatggc
OsSK11	P$CBNAC_01	CBNAC	753	759	1	1	0.968	aTGCTT
OsSK11	P$CBNAC_02	CBNAC	753	769	1	1	0.857	aTGCTTatggcatgct
OsSK11	P$ABI3_01	ABI3	760	769	1	1	0.962	tgGCATGct
OsSK11	P$FUS3_01	FUS3	761	770	1	1	0.943	gGCATGcta
OsSK11	P$LEC2_01	LEC2	761	772	1	1	0.942	ggCATGCtaaa
OsSK11	P$MYB1L_01	MYB1L	779	789	1	1	0.977	caCCCTAcgc
OsSK11	P$TRB2_01	TRB2	779	787	1	1	0.949	caCCCTAc
OsSK11	P$NAC083_01	NAC083	783	793	1	1	0.985	ctACGCAaga
OsSK11	P$GATA9_01	GATA9	787	798	1	1	0.988	gcaAGATCttt
OsSK11	P$AGP1_01	AGP1	788	798	1	1	0.938	caAGATCttt
OsSK11	P$ARR2_01	ARR2	788	798	1	1	0.9	caagATCTTt
OsSK11	P$GATA10_01	GATA10	789	797	1	1	0.912	aAGATCtt
OsSK11	P$GATA11_01	GATA11	789	797	1	1	0.949	aaGATCTt
OsSK11	P$GATA8_01	GATA8	789	798	1	1	0.987	aaGATCTtt
OsSK11	P$ARR10_01	ARR10	790	797	1	1	0.956	AGATCtt
OsSK11	P$MYB24_01	MYB24	794	803	1	1	0.862	cttTTAGGa
OsSK11	P$ARF8_01	ARF8	806	815	1	1	0.956	tcTGTCGac
OsSK11	P$GAMYB_Q2	GAMYB	816	829	1	1	0.875	actttACAACgcg
OsSK11	P$AT5G54070_01	AT5G54070	821	827	1	1	0.958	aCAACG
OsSK11	P$TRAB1_Q2	TRAB1	822	833	1	1	0.86	caACGCGatgc
OsSK11	P$MYB3R5_01	MYB3R5	877	892	1	1	0.877	agaggaaagCCGTTg
OsSK11	P$DOF2_01	DOF2	878	889	1	1	0.979	gaggAAAGCcg
OsSK11	P$DOF3_01	DOF3	878	889	1	1	0.993	gaggAAAGCcg
OsSK11	P$MYB3R1_01	MYB3R1	878	893	1	1	0.875	gaggaaagCCGTTgc
OsSK11	P$MYB3R4_01	MYB3R4	878	893	1	1	0.886	gaggaaagCCGTTgc
OsSK11	P$AGL1_02	AGL1	888	906	1	1	0.943	gTTGCCttttagggcaag
OsSK11	P$SED_Q2	SED	888	898	1	1	0.996	gttgCCTTTt
OsSK11	P$PBF_Q2_01	BF	892	898	1	1	1	CCTTTt
OsSK11	P$MYB24_01	MYB24	893	902	1	1	0.859	cttTTAGGg
OsSK11	P$ARR18_01	ARR18	906	919	1	1	0.886	gcttAGATAacta
OsSK11	P$PIL5_01	IL5	911	925	1	1	0.852	gataactaACGTGg
OsSK11	P$C1_Q2	C1	912	923	1	1	0.937	atAACTAacgt
OsSK11	P$WEREWOLF_Q2_01	WEREWOLF	914	923	1	1	0.94	aACTAAcgt
OsSK11	P$MYBAS1_01	MYBAS1	914	925	1	1	0.943	aaCTAACgtgg
OsSK11	P$ABZ1_01	ABZ1	914	928	1	1	0.934	aactaACGTGgagg
OsSK11	P$GBF1_Q2_01	GBF1	915	926	1	1	0.869	actaACGTGga
OsSK11	P$HBP1A_Q2	HBP1A	916	926	1	1	0.876	ctaACGTGga
OsSK11	P$TAF1_Q2	TAF1	916	926	1	1	0.933	ctaACGTGga
OsSK11	P$EMBP1_02	EMBP1	916	926	1	1	0.933	ctaACGTGga
OsSK11	P$TAF1_01	TAF1	916	926	1	1	0.958	ctaACGTGga
OsSK11	P$HY5_01	HY5	917	927	1	1	0.96	taACGTGgag
OsSK11	P$GBF1_01	GBF1	917	925	1	1	0.971	taACGTGg
OsSK11	P$BIM1_02	BIM1	917	927	1	1	0.945	taACGTGgag
OsSK11	P$ABF4_02	ABF4	917	927	1	1	0.982	taACGTGgag
OsSK11	P$BZIP68_01	BZIP68	917	926	1	1	0.987	taaCGTGGa
OsSK11	P$CPRF1_Q2	CPRF1	918	928	1	1	0.934	aACGTGgagg
OsSK11	P$ABI5_Q2	ABI5	919	925	1	1	1	ACGTGg
OsSK11	P$CBNAC_01	CBNAC	935	941	1	1	1	tTGCTT
OsSK11	P$CBNAC_02	CBNAC	935	951	1	1	0.892	tTGCTTtgtagggtaa
OsSK11	P$ARR18_01	ARR18	952	965	1	1	0.888	gcctAGATAgcca
OsSK11	P$MYBAS1_01	MYBAS1	960	971	1	1	0.943	agCCAACatag
OsSK11	P$RAV1_01	RAV1	960	972	1	1	0.962	agcCAACAtaga
OsSK11	P$C1_Q2	C1	1025	1036	1	1	0.916	agAACTAgata
OsSK11	P$ARR18_01	ARR18	1027	1040	1	1	0.944	aactAGATActtc
OsSK11	P$AMS_01	AMS	1037	1047	1	1	0.876	ttCATGTgca
OsSK11	P$MYBAS1_01	MYBAS1	1045	1056	1	1	0.952	caCCAACaccg
OsSK11	P$RAV1_01	RAV1	1045	1057	1	1	0.921	cacCAACAccga
OsSK11	P$AT4G12750_01	AT4G12750	1049	1059	1	1	0.914	aacACCGAtc
OsSK11	P$AT5G67190_01	AT5G67190	1050	1060	1	1	0.936	ACACCgatca
OsSK11	P$MADSB_Q2	MADSB	1056	1071	1	1	0.861	atcaAAAAAtagaat
OsSK11	P$AP1_01	AP1	1058	1071	1	1	0.868	caAAAAAtagaat
OsSK11	P$RIN_01	RIN	1058	1068	1	1	0.921	caaaaAATAG
OsSK11	P$SED_Q2	SED	1079	1089	1	1	0.883	tataCCTTTg
OsSK11	P$PBF_Q2_01	BF	1083	1089	1	1	0.988	CCTTTg
OsSK11	P$WRKY11_Q2	WRKY11	1088	1096	1	1	0.975	gTTGACcg
OsSK11	P$ZAP1_01	ZAP1	1089	1099	1	1	0.939	TTGACcgaaa
OsSK11	P$AT2G41690_01	AT2G41690	1093	1099	1	1	0.978	CCGAAa
OsSK11	P$GAMYB_Q2	GAMYB	1101	1114	1	1	0.904	tctaaACAACttg
OsSK11	P$BHLH112_01	BHLH112	1106	1115	1	1	0.934	acaACTTGa
OsSK11	P$TGA1_01	TGA1	1109	1120	1	1	0.926	actTGACGaca
OsSK11	P$WRKY11_Q2	WRKY11	1110	1118	1	1	0.921	cTTGACga
OsSK11	P$TGA7_01	TGA7	1110	1120	1	1	0.89	ctTGACGaca
OsSK11	P$TGA5_01	TGA5	1111	1119	1	1	0.856	tTGACGac
OsSK11	P$GAMYB_Q2	GAMYB	1130	1143	1	1	0.869	ggttcACAACgtt

OsSK11	P$AT5G54070_01	AT5G54070	1135	1141	1	1	0.958	aCAACG
OsSK11	P$ARR1_01	ARR1	1157	1167	1	1	0.987	caaGAATCtt
OsSK11	P$ARR2_01	ARR2	1158	1168	1	1	0.983	aagaATCTTg
OsSK11	P$ABZ1_01	ABZ1	1180	1194	1	1	0.919	ggtgaACGTGttta
OsSK11	P$TAF1_Q2	TAF1	1182	1192	1	1	0.914	tgaACGTGtt
OsSK11	P$TAF1_01	TAF1	1182	1192	1	1	0.94	tgaACGTGtt
OsSK11	P$GBF1_01	GBF1	1183	1191	1	1	0.949	gaACGTGt
OsSK11	P$BIM1_02	BIM1	1183	1193	1	1	0.947	gaACGTGttt
OsSK11	P$ABF4_02	ABF4	1183	1193	1	1	0.986	gaACGTGttt
OsSK11	P$ABI5_Q2	ABI5	1185	1191	1	1	0.979	ACGTGt
OsSK11	P$SBF1_01	SBF1	1185	1199	1	1	0.878	acgtgtTTAAAttt
OsSK11	P$ARR18_01	ARR18	1203	1216	1	1	0.94	caatAGATAagtc
OsSK11	P$C1_Q2	C1	1218	1229	1	1	0.93	aaAACTAggag
OsSK11	P$CMTA2_01	CMTA2	1225	1234	1	1	0.993	ggagCGCGT
OsSK11	P$CAMTA1_02	CAMTA1	1225	1237	1	1	0.936	ggagCGCGTaga
OsSK11	P$CMTA3_01	CMTA3	1228	1237	1	1	0.982	gCGCGTaga
OsSK11	P$RIN_Q2	RIN	1239	1250	1	1	0.959	cactTTTAAgg
OsSK11	P$GAMYB_Q2	GAMYB	1251	1264	1	1	0.937	cgtcgACAACcta
OsSK11	P$GAMYB_01	GAMYB	1257	1265	1	1	0.868	CAACCtag
OsSK11	P$ARR18_01	ARR18	1261	1274	1	1	0.896	ctagAGATAgagg
OsSK11	P$CBF3_02	CBF3	1271	1285	1	1	0.948	aggagCCGACggga
OsSK11	P$CBF1_04	CBF1	1272	1284	1	1	0.943	ggagCCGACggg
OsSK11	P$ERF5_02	ERF5	1273	1283	1	1	0.89	gaGCCGAcgg
OsSK11	P$ERF1_04	ERF1	1273	1283	1	1	0.879	gaGCCGAcgg
OsSK11	P$DREB1G_02	DREB1G	1273	1283	1	1	0.901	gagCCGACgg
OsSK11	P$ARF1_01	ARF1	1275	1283	1	1	0.854	gCCGACgg
OsSK11	P$ARF5_01	ARF5	1275	1283	1	1	0.897	gCCGACgg
OsSK11	P$DREB1B_01	DREB1B	1276	1281	1	1	1	CCGAC
OsSK11	P$RAMOSA1_01	RAMOSA1	1282	1296	1	1	0.89	ggaggaGAGAGaga
OsSK11	P$RAMOSA1_01	RAMOSA1	1284	1298	1	1	0.901	aggagaGAGAGaaa
OsSK11	P$BPC1_Q2	BPC1	1293	1299	1	1	1	AGAAAg
OsSK11	P$KNOX3_01	KNOX3	1329	1341	1	1	0.967	gcaaTGACActa
OsSK11	P$ATH1_01	ATH1	1333	1341	1	1	0.913	TGACActa
OsSK11	P$CBF3_02	CBF3	1358	1372	1	1	0.974	agccaCCGACagtg
OsSK11	P$CBF1_04	CBF1	1359	1371	1	1	0.965	gccaCCGACagt
OsSK11	P$DREB1G_02	DREB1G	1360	1370	1	1	0.916	ccaCCGACag
OsSK11	P$AT1G77200_03	AT1G77200	1360	1374	1	1	0.992	ccaCCGACagtgag
OsSK11	P$ARF1_01	ARF1	1362	1370	1	1	1	aCCGACag
OsSK11	P$ARF5_01	ARF5	1362	1370	1	1	0.988	aCCGACag
OsSK11	P$DREB1B_01	DREB1B	1363	1368	1	1	1	CCGAC
OsSK11	P$AMS_01	AMS	1388	1398	1	1	0.909	ctCAGGTgag
OsSK11	P$GAMYB_Q2	GAMYB	1394	1407	1	1	0.912	tgagaACAACgac
OsSK11	P$AT5G54070_01	AT5G54070	1399	1405	1	1	0.958	aCAACG
OsSK11	P$ARR18_01	ARR18	1427	1440	1	1	0.949	ggagAGATAcatg
OsSK11	P$BPC1_Q2	BPC1	1440	1446	1	1	0.99	AGAAAc
OsSK11	P$TGA1_01	TGA1	1447	1458	1	1	0.933	atgTGACGata
OsSK11	P$TGA7_01	TGA7	1448	1458	1	1	0.895	tgTGACGata
OsSK11	P$TGA5_01	TGA5	1449	1457	1	1	0.864	gTGACGat
OsSK11	P$NAC043_01	NAC043	1460	1470	1	1	0.916	gtaACGTAgg
OsSK11	P$BPC1_Q2	BPC1	1471	1477	1	1	0.997	AGAAAa
OsSK11	P$HMG1_01	HMG1	1480	1489	1	1	0.913	GTTGTtatg
OsSK11	P$TEIL_01	TEIL	1486	1494	1	1	0.882	ATGTAatt
OsSK11	P$SED_Q2	SED	1494	1504	1	1	0.91	tgttCCTTTt
OsSK11	P$PBF_Q2_01	BF	1498	1504	1	1	1	CCTTTt
OsSK11	P$HSFA4A_01	HSFA4A	1503	1509	1	1	0.914	tCTATT
OsSK11	P$SED_Q2	SED	1507	1517	1	1	0.964	ttggCCTTTa
OsSK11	P$PBF_Q2_01	BF	1511	1517	1	1	0.998	CCTTTa
OsSK11	P$SEP3_01	wrz-03	1521	1532	1	1	0.873	taattTTTTGg
OsSK11	P$BPC1_Q2	BPC1	1546	1552	1	1	0.997	AGAAAa
OsSK11	P$ATHB4_02	ATHB4	1569	1579	1	1	0.851	atATCATttg
OsSK11	P$ABF2_01	ABF2	1581	1594	1	1	0.918	gccacCACGTatt
OsSK11	P$LIM1_01	LIM1	1582	1594	1	1	0.905	CCACCacgtatt
OsSK11	P$BZR1_02	BZR1	1582	1596	1	1	0.861	ccacCACGTattat
OsSK11	P$GBP_Q6	GBP	1583	1595	1	1	0.913	cacCACGTatta
OsSK11	P$ABI5_01	ABI5	1583	1593	1	1	0.913	cacCACGTat
OsSK11	P$ABF4_01	ABF4	1583	1595	1	1	0.895	cacCACGTatta
OsSK11	P$EMBP1_Q2	EMBP1	1584	1594	1	1	0.881	acCACGTatt
OsSK11	P$CPRF3_Q2	CPRF3	1584	1594	1	1	0.916	acCACGTatt
OsSK11	P$CPRF2_Q2	CPRF2	1584	1594	1	1	0.945	acCACGTatt
OsSK11	P$O2_02	O2	1584	1594	1	1	0.969	acCACGTatt
OsSK11	P$TGA1B_Q2	TGA1B	1584	1594	1	1	0.888	acCACGTatt
OsSK11	P$TGA1A_Q2	TGA1A	1584	1594	1	1	0.964	acCACGTatt
OsSK11	P$CPRF3_01	CPRF3	1584	1594	1	1	0.923	acCACGTatt
OsSK11	P$CPRF2_01	CPRF2	1584	1594	1	1	0.947	acCACGTatt
OsSK11	P$TGA1B_01	TGA1B	1584	1594	1	1	0.861	acCACGTatt
OsSK11	P$BEE2_01	BEE2	1584	1594	1	1	0.915	acCACGTatt
OsSK11	P$BIM2_01	BIM2	1584	1594	1	1	0.852	acCACGTatt
OsSK11	P$BIM3_01	BIM3	1584	1594	1	1	0.887	acCACGTatt

OsSK11	P$PHYPA143875_02	HYPA143875	1584	1594	1	1	0.875	acCACGTatt
OsSK11	P$SPT_01	SPT	1584	1593	1	1	0.942	acCACGTat
OsSK11	P$GBF1F_Q2	GBF1F	1584	1595	1	1	0.937	acCACGTatta
OsSK11	P$RITA1_01	RITA1	1585	1592	1	1	0.987	cCACGTa
OsSK11	P$OCSBF1_01	OCSBF1	1586	1591	1	1	1	CACGT
OsSK11	P$CBF1_01	CBF1	1594	1604	1	1	0.88	aTGCCGaaga
OsSK11	P$JERF1_01	JERF1	1594	1604	1	1	0.872	aTGCCGaaga
OsSK11	P$CBF1_03	CBF1	1594	1604	1	1	0.914	aTGCCGaaga
OsSK11	P$AT2G41690_01	AT2G41690	1597	1603	1	1	0.988	CCGAAg
OsSK11	P$PEND_01	END	1599	1607	1	1	0.874	gAAGAAat
OsSK11	P$BPC1_Q2	BPC1	1601	1607	1	1	0.99	AGAAAt
OsSK11	P$PEND_02	END	1605	1615	1	1	0.881	atTTCTTaag
OsSK11	P$MYB3_01	MYB3	1630	1641	1	1	0.93	cagTAGGTagc
OsSK11	P$MYB4_01	MYB4	1631	1639	1	1	0.95	agTAGGTa
OsSK11	P$ARR18_01	ARR18	1664	1677	1	1	0.952	tcccAGATAcaat
OsSK11	P$MYBAS1_01	MYBAS1	1687	1698	1	1	0.984	ctCCAACaacc
OsSK11	P$RAV1_01	RAV1	1687	1699	1	1	0.918	ctcCAACAacct
OsSK11	P$GAMYB_Q2	GAMYB	1687	1700	1	1	0.96	ctccaACAACctc
OsSK11	P$GAMYB_01	GAMYB	1693	1701	1	1	0.933	CAACCtcc
OsSK11	P$GL15_01	GL15	1700	1710	1	1	0.854	cttatCCCCC
OsSK11	P$AT3G60580_01	AT3G60580	1701	1708	1	1	0.95	ttATCCC
OsSK11	P$SBF1_01	SBF1	1712	1726	1	1	0.892	tctaagTTAAAttt
OsSK11	P$AGL20_01	AGL20	1717	1729	1	1	0.883	gtTAAATtttag
OsSK11	P$AGL12_01	AGL12	1717	1729	1	1	0.945	gttAAATTttag
OsSK11	P$AT2G26320_01	AT2G26320	1718	1729	1	1	0.931	TTAAAttttag
OsSK11	P$SEP3_01	wrz-03	1727	1738	1	1	0.883	agcaaTTTTGg
OsSK11	P$MYBAS1_01	MYBAS1	1750	1761	1	1	0.986	ctCCAACagac
OsSK11	P$RAV1_01	RAV1	1750	1762	1	1	0.963	ctcCAACAgact
OsSK11	P$HSFA2_01	HSFA2	1776	1782	1	1	0.933	CCAAAg
OsSK11	P$WRKY44_01	WRKY44	1780	1789	1	1	0.871	AGTCCtcct
OsSK11	P$AG_01	AG	1793	1811	1	1	0.875	ctagCCAAAtttggccag
OsSK11	P$AGL1_01	AGL1	1793	1811	1	1	0.852	ctagCCAAAtttggccag
OsSK11	P$AG_03	AG	1793	1811	1	1	0.875	ctagCCAAAtttggccag
OsSK11	P$HSFA2_01	HSFA2	1797	1803	1	1	0.922	CCAAAt
OsSK11	P$TCP14_01	TCP14	1822	1842	1	1	0.889	tagccaattGTGGGccccac
OsSK11	P$TCP11_01	TCP11	1830	1842	1	1	0.998	tGTGGGccccac
OsSK11	P$TCP15_01	TCP15	1831	1841	1	1	1	GTGGGcccca
OsSK11	P$TCP20_01	TCP20	1831	1841	1	1	0.99	GTGGGcccca
OsSK11	P$TCP11_02	TCP11	1831	1841	1	1	0.998	GTGGGcccca
OsSK11	P$TCP7_01	TCP7	1831	1842	1	1	0.998	GTGGGccccac
OsSK11	P$OJ1581_01	OJ1581	1831	1841	1	1	0.994	gtGGGCCcca
OsSK11	P$TCP2_01	TCP2	1831	1841	1	1	0.974	gtGGGCCcca
OsSK11	P$PCF2_01	CF2	1832	1842	1	1	1	tgggcCCCAC
OsSK11	P$TCP19_01	TCP19	1832	1842	1	1	0.998	tgggcCCCAC
OsSK11	P$TCP20L_01	TCP20L	1833	1842	1	1	0.997	gggcCCCAC
OsSK11	P$OSI_01	OSI	1834	1842	1	1	0.959	ggcCCCAC
OsSK11	P$TCP20_02	TCP20	1834	1844	1	1	0.997	ggcCCCACgc
OsSK11	P$ARALY495258_02	ARALY495258	1834	1842	1	1	1	ggcCCCAC
OsSK11	P$ARALY493022_04	ARALY493022	1834	1842	1	1	0.973	ggcCCCAC
OsSK11	P$ARALY484486_05	ARALY484486	1834	1842	1	1	1	ggcCCCAC
OsSK11	P$ERF4_05	ERF4	1837	1852	1	1	0.869	cccaCGCCGacagca
OsSK11	P$CBF3_02	CBF3	1838	1852	1	1	0.968	ccacgCCGACagca
OsSK11	P$RRTF1_02	RRTF1	1839	1849	1	1	0.855	caCGCCGaca
OsSK11	P$ERF112_02	ERF112	1839	1849	1	1	0.974	caCGCCGaca
OsSK11	P$CBF1_04	CBF1	1839	1851	1	1	0.972	cacgCCGACagc
OsSK11	P$CRF4_01	CRF4	1840	1848	1	1	0.92	aCGCCGac
OsSK11	P$ERF4_04	ERF4	1840	1848	1	1	0.946	aCGCCGac
OsSK11	P$ERF069_01	ERF069	1840	1849	1	1	0.991	aCGCCGaca
OsSK11	P$ERF11_01	ERF11	1840	1850	1	1	0.993	aCGCCGacag
OsSK11	P$ERF5_02	ERF5	1840	1850	1	1	0.936	acGCCGAcag
OsSK11	P$ERF1_04	ERF1	1840	1850	1	1	0.923	acGCCGAcag
OsSK11	P$DREB1G_02	DREB1G	1840	1850	1	1	0.99	acgCCGACag
OsSK11	P$AT1G77200_03	AT1G77200	1840	1854	1	1	0.937	acgCCGACagcatc
OsSK11	P$ERF8_01	ERF8	1841	1851	1	1	0.989	CGCCGacagc
OsSK11	P$ERF3_04	ERF3	1841	1849	1	1	0.966	CGCCGaca
OsSK11	P$ARF1_01	ARF1	1842	1850	1	1	0.957	gCCGACag
OsSK11	P$ARF5_01	ARF5	1842	1850	1	1	0.993	gCCGACag
OsSK11	P$DREB1B_01	DREB1B	1843	1848	1	1	1	CCGAC
OsSK11	P$AT3G60580_01	AT3G60580	1849	1856	1	1	0.901	gcATCCC
OsSK11	P$SED_Q2	SED	1857	1867	1	1	0.98	ctccCCTTTc
OsSK11	P$PBF_Q2_01	BF	1861	1867	1	1	0.985	CCTTTc
OsSK11	P$AT3G63350_01	AT3G63350	1872	1878	1	1	0.882	CCGCCa
OsSK11	P$MYBAS1_01	MYBAS1	1873	1884	1	1	0.992	cgCCAACtgtc
OsSK11	P$ARF8_01	ARF8	1878	1887	1	1	0.953	acTGTCGtc
OsSK11	P$WRKY18_02	WRKY18	1884	1894	1	1	0.947	gtcGTCAAga
OsSK11	P$WRKY21_02	WRKY21	1884	1894	1	1	0.945	gtcGTCAAga
OsSK11	P$WRKY48_02	WRKY48	1884	1894	1	1	0.987	gtcGTCAAga
OsSK11	P$WRKY57_01	WRKY57	1884	1894	1	1	0.952	gtcGTCAAga

OsSK11	P$WRKY60_01	WRKY60	1884	1895	1	1	0.889	gtcGTCAAgat
OsSK11	P$WRKY15_01	WRKY15	1885	1895	1	1	0.958	tcGTCAAgat
OsSK11	P$WRKY2_01	WRKY2	1885	1893	1	1	0.903	tcGTCAAg
OsSK11	P$WRKY25_02	WRKY25	1885	1893	1	1	0.887	tcGTCAAg
OsSK11	P$WRKY40_01	WRKY40	1885	1893	1	1	0.981	tcGTCAAg
OsSK11	P$WRKY43_02	WRKY43	1885	1895	1	1	0.952	tcGTCAAgat
OsSK11	P$WRKY62_01	WRKY62	1885	1893	1	1	0.87	tcGTCAAg
OsSK11	P$WRKY63_01	WRKY63	1885	1893	1	1	0.888	tcGTCAAg
OsSK11	P$WRKY75_01	WRKY75	1885	1893	1	1	0.92	tcGTCAAg
OsSK11	P$WRKY8_01	WRKY8	1885	1894	1	1	0.977	tcGTCAAga
OsSK11	P$WRKY30_01	WRKY30	1886	1896	1	1	0.901	cGTCAAgatg
OsSK11	P$WRKY18_Q2	WRKY18	1887	1896	1	1	0.984	GTCAAgatg
OsSK11	P$ARF8_01	ARF8	1907	1916	1	1	0.955	gcTGTCGag
OsSK11	P$HMG1_01	HMG1	1916	1925	1	1	0.919	GTTGTcgtt
OsSK11	P$ARF8_01	ARF8	1916	1925	1	1	0.956	gtTGTCGtt
OsSK11	P$CBF1_01	CBF1	1923	1933	1	1	0.859	tTGCCGgacg
OsSK11	P$ERF019_01	ERF019	1923	1933	1	1	0.878	tTGCCGgacg
OsSK11	P$JERF1_01	JERF1	1923	1933	1	1	0.85	tTGCCGgacg
OsSK11	P$CBF1_03	CBF1	1923	1933	1	1	0.891	tTGCCGgacg
OsSK11	P$AT1G33760_01	AT1G33760	1923	1933	1	1	0.858	tTGCCGgacg
OsSK11	P$ERF73_01	ERF73	1924	1945	1	1	0.912	tgccggaCGCCGccgtcgccc
OsSK11	P$ERF4_05	ERF4	1927	1942	1	1	0.9	cggaCGCCGccgtcg
OsSK11	P$AT2G33710_01	AT2G33710	1927	1942	1	1	0.938	cggacgcCGCCGtcg
OsSK11	P$ERF73_01	ERF73	1927	1948	1	1	0.86	cggacgcCGCCGtcgcccgcc
OsSK11	P$ABI4_01	ABI4	1928	1939	1	1	0.854	ggacgCCGCCg
OsSK11	P$RRTF1_02	RRTF1	1929	1939	1	1	0.92	gaCGCCGccg
OsSK11	P$RAP26_03	RAP26	1929	1939	1	1	0.956	gaCGCCGccg
OsSK11	P$RAP210_04	RAP210	1929	1939	1	1	0.917	gaCGCCGccg
OsSK11	P$ERF112_02	ERF112	1929	1939	1	1	0.981	gaCGCCGccg
OsSK11	P$CRF4_01	CRF4	1930	1938	1	1	0.978	aCGCCGcc
OsSK11	P$ERF4_04	ERF4	1930	1938	1	1	0.988	aCGCCGcc
OsSK11	P$ERF069_01	ERF069	1930	1939	1	1	0.998	aCGCCGccg
OsSK11	P$ERF11_01	ERF11	1930	1940	1	1	0.991	aCGCCGccgt
OsSK11	P$ABI4_03	ABI4	1930	1940	1	1	0.954	acGCCGCcgt
OsSK11	P$WRAF1_01	WRAF1	1930	1940	1	1	0.866	acGCCGCcgt
OsSK11	P$PTI5_01	TI5	1930	1940	1	1	0.918	acGCCGCcgt
OsSK11	P$DREBI5_01	DREBI5	1930	1940	1	1	0.944	acGCCGCcgt
OsSK11	P$AT2G47520_01	AT2G47520	1930	1940	1	1	0.932	acGCCGCcgt
OsSK11	P$DREB2B_01	DREB2B	1930	1940	1	1	0.945	acGCCGCcgt
OsSK11	P$CRF1_02	CRF1	1930	1940	1	1	0.896	acGCCGCcgt
OsSK11	P$OPBP1_01	OPBP1	1930	1940	1	1	0.919	acGCCGCcgt
OsSK11	P$ATERF14_01	ATERF14	1930	1940	1	1	0.906	acGCCGCcgt
OsSK11	P$DREB2A_02	DREB2A	1930	1940	1	1	0.856	acGCCGCcgt
OsSK11	P$ERF1_02	ERF1	1930	1940	1	1	0.9	acGCCGCcgt
OsSK11	P$ERF4_02	ERF4	1930	1940	1	1	0.963	acGCCGCcgt
OsSK11	P$AT5G25390_01	AT5G25390	1930	1940	1	1	0.91	acGCCGCcgt
OsSK11	P$EREBP1_01	EREBP1	1930	1940	1	1	0.914	acGCCGCcgt
OsSK11	P$CBF3_01	CBF3	1930	1940	1	1	0.89	acGCCGCcgt
OsSK11	P$DREBII1_01	DREBII1	1930	1940	1	1	0.924	acGCCGCcgt
OsSK11	P$TSRF1_01	TSRF1	1930	1940	1	1	0.91	acGCCGCcgt
OsSK11	P$DRF13_01	DRF13	1930	1940	1	1	0.896	acGCCGCcgt
OsSK11	P$ERF4_03	ERF4	1930	1940	1	1	0.857	acGCCGCcgt
OsSK11	P$ERF2_03	ERF2	1930	1940	1	1	0.908	acGCCGCcgt
OsSK11	P$ERF1B_03	ERF1B	1930	1940	1	1	0.921	acGCCGCcgt
OsSK11	P$RAP26_02	RAP26	1930	1940	1	1	0.855	acGCCGCcgt
OsSK11	P$CBF5_01	CBF5	1930	1940	1	1	0.902	acGCCGCcgt
OsSK11	P$CBF16_01	CBF16	1930	1940	1	1	0.92	acGCCGCcgt
OsSK11	P$CBF17_01	CBF17	1930	1940	1	1	0.948	acGCCGCcgt
OsSK11	P$ERF1_05	ERF1	1930	1940	1	1	0.929	acGCCGCcgt
OsSK11	P$AT5G25190_01	AT5G25190	1930	1940	1	1	0.928	acGCCGCcgt
OsSK11	P$ERF105_01	ERF105	1930	1940	1	1	0.915	acGCCGCcgt
OsSK11	P$CBF_01	CBF	1930	1940	1	1	0.948	acGCCGCcgt
OsSK11	P$AT5G11190_01	AT5G11190	1930	1940	1	1	0.905	acGCCGCcgt
OsSK11	P$AT1G68550_01	AT1G68550	1930	1940	1	1	0.981	acGCCGCcgt
OsSK11	P$AT1G77640_01	AT1G77640	1930	1940	1	1	0.88	acGCCGCcgt
OsSK11	P$ERF016_01	ERF016	1930	1940	1	1	0.89	acGCCGCcgt
OsSK11	P$AT3G61630_01	AT3G61630	1930	1940	1	1	0.969	acGCCGCcgt
OsSK11	P$AT5G43410_01	AT5G43410	1930	1940	1	1	0.912	acGCCGCcgt
OsSK11	P$AT5G07310_01	AT5G07310	1930	1940	1	1	0.922	acGCCGCcgt
OsSK11	P$AT3G16280_01	AT3G16280	1930	1940	1	1	0.851	acGCCGCcgt
OsSK11	P$DREB1A_03	DREB1A	1930	1940	1	1	0.895	acGCCGCcgt
OsSK11	P$AT1G49120_01	AT1G49120	1930	1940	1	1	0.88	acGCCGCcgt
OsSK11	P$DREB2D_01	DREB2D	1930	1940	1	1	0.903	acGCCGCcgt
OsSK11	P$AT3G25890_01	AT3G25890	1930	1940	1	1	0.891	acGCCGCcgt
OsSK11	P$AT4G23750_01	AT4G23750	1930	1940	1	1	0.857	acGCCGCcgt
OsSK11	P$AT4G27950_01	AT4G27950	1930	1940	1	1	0.889	acGCCGCcgt
OsSK11	P$RRTF1_01	RRTF1	1930	1940	1	1	0.951	acGCCGCcgt
OsSK11	P$CRF2_01	CRF2	1930	1938	1	1	0.985	acGCCGCc

OsSK11	P$ERF096_01	ERF096	1930	1940	1	1	0.998	acGCCGCcgt
OsSK11	P$ERF098_01	ERF098	1930	1938	1	1	0.999	acGCCGCc
OsSK11	P$DREB2C_01	DREB2C	1930	1940	1	1	0.913	acgCCGCCgt
OsSK11	P$CBF1_02	CBF1	1930	1940	1	1	0.897	acgCCGCCgt
OsSK11	P$CBF2_03	CBF2	1930	1940	1	1	0.96	acgcCGCCGt
OsSK11	P$ERF4_05	ERF4	1930	1945	1	1	0.923	acgcCGCCGtcgccc
OsSK11	P$ERF8_01	ERF8	1931	1941	1	1	0.993	CGCCGccgtc
OsSK11	P$ERF3_04	ERF3	1931	1939	1	1	0.983	CGCCGccg
OsSK11	P$OS05G0497200_01	OS05G0497200	1931	1939	1	1	1	CGCCGccg
OsSK11	P$ERF1B_06	ERF1B	1931	1939	1	1	0.985	cGCCGCcg
OsSK11	P$ERF7_02	ERF7	1931	1941	1	1	0.995	cGCCGCcgtc
OsSK11	P$ERF094_01	ERF094	1931	1939	1	1	1	cGCCGCcg
OsSK11	P$ERF2_01	ERF2	1931	1938	1	1	1	cgCCGCC
OsSK11	P$ERF13_02	ERF13	1931	1939	1	1	0.996	cgCCGCCg
OsSK11	P$RRTF1_02	RRTF1	1932	1942	1	1	0.851	gcCGCCGtcg
OsSK11	P$RAP26_03	RAP26	1932	1942	1	1	0.852	gcCGCCGtcg
OsSK11	P$ERF112_02	ERF112	1932	1942	1	1	0.982	gcCGCCGtcg
OsSK11	P$AT3G63350_01	AT3G63350	1933	1939	1	1	0.866	CCGCCg
OsSK11	P$CRF4_01	CRF4	1933	1941	1	1	0.936	cCGCCGtc
OsSK11	P$ERF4_04	ERF4	1933	1941	1	1	0.964	cCGCCGtc
OsSK11	P$ERF069_01	ERF069	1933	1942	1	1	0.994	cCGCCGtcg
OsSK11	P$ERF11_01	ERF11	1933	1943	1	1	0.989	cCGCCGtcgc
OsSK11	P$ERF8_01	ERF8	1934	1944	1	1	0.985	CGCCGtcgcc
OsSK11	P$ERF3_04	ERF3	1934	1942	1	1	0.952	CGCCGtcg
OsSK11	P$AT2G33710_01	AT2G33710	1937	1952	1	1	0.859	cgtcgccCGCCGtcg
OsSK11	P$ERF4_05	ERF4	1940	1955	1	1	0.898	cgcccCGCCGtcgtcg
OsSK11	P$ERF112_02	ERF112	1942	1952	1	1	0.98	ccCGCCGtcg
OsSK11	P$AT3G63350_01	AT3G63350	1943	1949	1	1	0.866	CCGCCg
OsSK11	P$CRF4_01	CRF4	1943	1951	1	1	0.936	cCGCCGtc
OsSK11	P$ERF4_04	ERF4	1943	1951	1	1	0.964	cCGCCGtc
OsSK11	P$ERF069_01	ERF069	1943	1952	1	1	0.994	cCGCCGtcg
OsSK11	P$ERF11_01	ERF11	1943	1953	1	1	0.99	cCGCCGtcgt
OsSK11	P$ERF8_01	ERF8	1944	1954	1	1	0.986	CGCCGtcgtc
OsSK11	P$ERF3_04	ERF3	1944	1952	1	1	0.952	CGCCGtcg
OsSK11	P$ANAC042_01	ANAC042	1944	1964	1	1	0.972	cGCCGTcgtcgcgccggcgc
OsSK11	P$ANAC094_01	ANAC094	1944	1962	1	1	0.934	cGCCGTcgtcgcgccggc
OsSK11	P$RRTF1_05	RRTF1	1951	1966	1	1	0.857	gtcgcgcCGGCGcca
OsSK11	P$RRTF1_02	RRTF1	1953	1963	1	1	0.866	cgCGCCGgcg
OsSK11	P$RAP26_03	RAP26	1953	1963	1	1	0.875	cgCGCCGgcg
OsSK11	P$RAP210_04	RAP210	1953	1963	1	1	0.891	cgCGCCGgcg
OsSK11	P$ERF112_02	ERF112	1953	1963	1	1	0.973	cgCGCCGgcg
OsSK11	P$CRF4_01	CRF4	1954	1962	1	1	0.942	gCGCCGgc
OsSK11	P$ERF4_04	ERF4	1954	1962	1	1	0.953	gCGCCGgc
OsSK11	P$ERF069_01	ERF069	1954	1963	1	1	0.993	gCGCCGgcg
OsSK11	P$ERF11_01	ERF11	1954	1964	1	1	0.987	gCGCCGgcgc
OsSK11	P$ERF6_02	ERF6	1954	1964	1	1	0.99	gcGCCGGcgc
OsSK11	P$ERF8_01	ERF8	1955	1965	1	1	0.982	CGCCGgcgcc
OsSK11	P$ERF3_04	ERF3	1955	1963	1	1	0.956	CGCCGgcg
OsSK11	P$ERF105_02	ERF105	1955	1963	1	1	0.97	cGCCGGcg
OsSK11	P$AT1G68550_03	AT1G68550	1955	1964	1	1	0.96	cgcCGGCGc
OsSK11	P$HSFA1E_01	HSFA1E	1957	1963	1	1	1	cCGGCG
OsSK11	P$CRF3_02	CRF3	1957	1967	1	1	0.874	ccggCGCCAg
OsSK11	P$BBM_01	BBM	1958	1968	1	1	0.861	cGGCGCcaga
OsSK11	P$E2FA_02	E2FA	1958	1968	1	1	0.988	cggCGCCAga
OsSK11	P$GATA9_01	GATA9	1962	1973	1	1	0.905	gccAGATCcac
OsSK11	P$AGP1_01	AGP1	1963	1973	1	1	0.936	ccAGATCcac
OsSK11	P$ARR10_01	ARR10	1965	1972	1	1	0.934	AGATCca
OsSK11	P$HSFA4A_01	HSFA4A	1971	1977	1	1	1	aCTATT
OsSK11	P$CBF1_01	CBF1	1975	1985	1	1	0.859	tTGCCGgacg
OsSK11	P$ERF019_01	ERF019	1975	1985	1	1	0.878	tTGCCGgacg
OsSK11	P$JERF1_01	JERF1	1975	1985	1	1	0.85	tTGCCGgacg
OsSK11	P$CBF1_03	CBF1	1975	1985	1	1	0.891	tTGCCGgacg
OsSK11	P$AT1G33760_01	AT1G33760	1975	1985	1	1	0.858	tTGCCGgacg
OsSK11	P$ERF73_01	ERF73	1976	1997	1	1	0.911	tgccggaCGCCGccgtcgtcg
OsSK11	P$ERF4_05	ERF4	1979	1994	1	1	0.9	cggaCGCCGccgtcg
OsSK11	P$AT2G33710_01	AT2G33710	1979	1994	1	1	0.938	cggacgcCGCCGtcg
OsSK11	P$ERF73_01	ERF73	1979	2000	1	1	0.863	cggacgcCGCCGtcgtcgtgc
OsSK11	P$ABI4_01	ABI4	1980	1991	1	1	0.854	ggacgCCGCCg
OsSK11	P$RRTF1_02	RRTF1	1981	1991	1	1	0.92	gaCGCCGccg
OsSK11	P$RAP26_03	RAP26	1981	1991	1	1	0.956	gaCGCCGccg
OsSK11	P$RAP210_04	RAP210	1981	1991	1	1	0.917	gaCGCCGccg
OsSK11	P$ERF112_02	ERF112	1981	1991	1	1	0.981	gaCGCCGccg
OsSK11	P$CRF4_01	CRF4	1982	1990	1	1	0.978	aCGCCGcc
OsSK11	P$ERF4_04	ERF4	1982	1990	1	1	0.988	aCGCCGcc
OsSK11	P$ERF069_01	ERF069	1982	1991	1	1	0.998	aCGCCGccg
OsSK11	P$ERF11_01	ERF11	1982	1992	1	1	0.991	aCGCCGccgt
OsSK11	P$ABI4_03	ABI4	1982	1992	1	1	0.954	acGCCGCcgt
OsSK11	P$WRAF1_01	WRAF1	1982	1992	1	1	0.866	acGCCGCcgt

OsSK11	P$PTI5_01	TI5	1982	1992	1	1	0.918	acGCCGCcgt
OsSK11	P$DREBI5_01	DREBI5	1982	1992	1	1	0.944	acGCCGCcgt
OsSK11	P$AT2G47520_01	AT2G47520	1982	1992	1	1	0.932	acGCCGCcgt
OsSK11	P$DREB2B_01	DREB2B	1982	1992	1	1	0.945	acGCCGCcgt
OsSK11	P$CRF1_02	CRF1	1982	1992	1	1	0.896	acGCCGCcgt
OsSK11	P$OPBP1_01	OPBP1	1982	1992	1	1	0.919	acGCCGCcgt
OsSK11	P$ATERF14_01	ATERF14	1982	1992	1	1	0.906	acGCCGCcgt
OsSK11	P$DREB2A_02	DREB2A	1982	1992	1	1	0.856	acGCCGCcgt
OsSK11	P$ERF1_02	ERF1	1982	1992	1	1	0.9	acGCCGCcgt
OsSK11	P$ERF4_02	ERF4	1982	1992	1	1	0.963	acGCCGCcgt
OsSK11	P$AT5G25390_01	AT5G25390	1982	1992	1	1	0.91	acGCCGCcgt
OsSK11	P$EREBP1_01	EREBP1	1982	1992	1	1	0.914	acGCCGCcgt
OsSK11	P$CBF3_01	CBF3	1982	1992	1	1	0.89	acGCCGCcgt
OsSK11	P$DREBII1_01	DREBII1	1982	1992	1	1	0.924	acGCCGCcgt
OsSK11	P$TSRF1_01	TSRF1	1982	1992	1	1	0.91	acGCCGCcgt
OsSK11	P$DRF13_01	DRF13	1982	1992	1	1	0.896	acGCCGCcgt
OsSK11	P$ERF4_03	ERF4	1982	1992	1	1	0.857	acGCCGCcgt
OsSK11	P$ERF2_03	ERF2	1982	1992	1	1	0.908	acGCCGCcgt
OsSK11	P$ERF1B_03	ERF1B	1982	1992	1	1	0.921	acGCCGCcgt
OsSK11	P$RAP26_02	RAP26	1982	1992	1	1	0.855	acGCCGCcgt
OsSK11	P$CBF5_01	CBF5	1982	1992	1	1	0.902	acGCCGCcgt
OsSK11	P$CBF16_01	CBF16	1982	1992	1	1	0.92	acGCCGCcgt
OsSK11	P$CBF17_01	CBF17	1982	1992	1	1	0.948	acGCCGCcgt
OsSK11	P$ERF1_05	ERF1	1982	1992	1	1	0.929	acGCCGCcgt
OsSK11	P$AT5G25190_01	AT5G25190	1982	1992	1	1	0.928	acGCCGCcgt
OsSK11	P$ERF105_01	ERF105	1982	1992	1	1	0.915	acGCCGCcgt
OsSK11	P$CBF_01	CBF	1982	1992	1	1	0.948	acGCCGCcgt
OsSK11	P$AT5G11190_01	AT5G11190	1982	1992	1	1	0.905	acGCCGCcgt
OsSK11	P$AT1G68550_01	AT1G68550	1982	1992	1	1	0.981	acGCCGCcgt
OsSK11	P$AT1G77640_01	AT1G77640	1982	1992	1	1	0.88	acGCCGCcgt
OsSK11	P$ERF016_01	ERF016	1982	1992	1	1	0.89	acGCCGCcgt
OsSK11	P$AT3G61630_01	AT3G61630	1982	1992	1	1	0.969	acGCCGCcgt
OsSK11	P$AT5G43410_01	AT5G43410	1982	1992	1	1	0.912	acGCCGCcgt
OsSK11	P$AT5G07310_01	AT5G07310	1982	1992	1	1	0.922	acGCCGCcgt
OsSK11	P$AT3G16280_01	AT3G16280	1982	1992	1	1	0.851	acGCCGCcgt
OsSK11	P$DREB1A_03	DREB1A	1982	1992	1	1	0.895	acGCCGCcgt
OsSK11	P$AT1G49120_01	AT1G49120	1982	1992	1	1	0.88	acGCCGCcgt
OsSK11	P$DREB2D_01	DREB2D	1982	1992	1	1	0.903	acGCCGCcgt
OsSK11	P$AT3G25890_01	AT3G25890	1982	1992	1	1	0.891	acGCCGCcgt
OsSK11	P$AT4G23750_01	AT4G23750	1982	1992	1	1	0.857	acGCCGCcgt
OsSK11	P$AT4G27950_01	AT4G27950	1982	1992	1	1	0.889	acGCCGCcgt
OsSK11	P$RRTF1_01	RRTF1	1982	1992	1	1	0.951	acGCCGCcgt
OsSK11	P$CRF2_01	CRF2	1982	1990	1	1	0.985	acGCCGCc
OsSK11	P$ERF096_01	ERF096	1982	1992	1	1	0.998	acGCCGCcgt
OsSK11	P$ERF098_01	ERF098	1982	1990	1	1	0.999	acGCCGCc
OsSK11	P$DREB2C_01	DREB2C	1982	1992	1	1	0.913	acgCCGCCgt
OsSK11	P$CBF1_02	CBF1	1982	1992	1	1	0.897	acgCCGCCgt
OsSK11	P$CBF2_03	CBF2	1982	1992	1	1	0.96	acgcCGCCGt
OsSK11	P$ERF4_05	ERF4	1982	1997	1	1	0.926	acgcCGCCGtcgtcg
OsSK11	P$ERF8_01	ERF8	1983	1993	1	1	0.993	CGCCGccgtc
OsSK11	P$ERF3_04	ERF3	1983	1991	1	1	0.983	CGCCGccg
OsSK11	P$OS05G0497200_01	OS05G0497200	1983	1991	1	1	1	CGCCGccg
OsSK11	P$ERF1B_06	ERF1B	1983	1991	1	1	0.985	cGCCGCcg
OsSK11	P$ERF7_02	ERF7	1983	1993	1	1	0.995	cGCCGCcgtc
OsSK11	P$ERF094_01	ERF094	1983	1991	1	1	1	cGCCGCcg
OsSK11	P$ERF2_01	ERF2	1983	1990	1	1	1	cgCCGCC
OsSK11	P$ERF13_02	ERF13	1983	1991	1	1	0.996	cgCCGCCg
OsSK11	P$RRTF1_02	RRTF1	1984	1994	1	1	0.851	gcCGCCGtcg
OsSK11	P$RAP26_03	RAP26	1984	1994	1	1	0.852	gcCGCCGtcg
OsSK11	P$ERF112_02	ERF112	1984	1994	1	1	0.982	gcCGCCGtcg
OsSK11	P$AT3G63350_01	AT3G63350	1985	1991	1	1	0.866	CCGCCg
OsSK11	P$CRF4_01	CRF4	1985	1993	1	1	0.936	cCGCCGtc
OsSK11	P$ERF4_04	ERF4	1985	1993	1	1	0.964	cCGCCGtc
OsSK11	P$ERF069_01	ERF069	1985	1994	1	1	0.994	cCGCCGtcg
OsSK11	P$ERF11_01	ERF11	1985	1995	1	1	0.99	cCGCCGtcgt
OsSK11	P$ERF8_01	ERF8	1986	1996	1	1	0.986	CGCCGtcgtc
OsSK11	P$ERF3_04	ERF3	1986	1994	1	1	0.952	CGCCGtcg
OsSK11	P$BZR1_01	BZR1	1995	2001	1	1	0.897	CGTGCc
OsSK11	P$GATA9_01	GATA9	2004	2015	1	1	0.909	gccAGATCcga
OsSK11	P$AGP1_01	AGP1	2005	2015	1	1	0.891	ccAGATCcga
OsSK11	P$GATA10_01	GATA10	2006	2014	1	1	0.892	cAGATCcg
OsSK11	P$ARR10_01	ARR10	2007	2014	1	1	1	AGATCcg
OsSK11	P$ERF4_05	ERF4	2024	2039	1	1	0.859	gttgCGCCGtcgcca
OsSK11	P$RRTF1_02	RRTF1	2026	2036	1	1	0.874	tgCGCCGtcg
OsSK11	P$RAP26_03	RAP26	2026	2036	1	1	0.876	tgCGCCGtcg
OsSK11	P$RAP210_04	RAP210	2026	2036	1	1	0.889	tgCGCCGtcg
OsSK11	P$ERF112_02	ERF112	2026	2036	1	1	0.976	tgCGCCGtcg
OsSK11	P$CRF4_01	CRF4	2027	2035	1	1	0.945	gCGCCGtc

OsSK11	P$ERF4_04	ERF4	2027	2035	1	1	0.954	gCGCCGtc
OsSK11	P$ERF069_01	ERF069	2027	2036	1	1	0.994	gCGCCGtcg
OsSK11	P$ERF11_01	ERF11	2027	2037	1	1	0.989	gCGCCGtcgc
OsSK11	P$ERF8_01	ERF8	2028	2038	1	1	0.985	CGCCGtcgcc
OsSK11	P$ERF3_04	ERF3	2028	2036	1	1	0.952	CGCCGtcg
OsSK11	P$CRF3_02	CRF3	2030	2040	1	1	0.891	ccgtCGCCAg
OsSK11	P$RRTF1_05	RRTF1	2040	2055	1	1	0.868	ccgtcgtCGGCGctg
OsSK11	P$DREB1A_04	DREB1A	2043	2053	1	1	0.969	tcGTCGGcgc
OsSK11	P$ERF039_01	ERF039	2043	2053	1	1	0.983	tcGTCGGcgc
OsSK11	P$PHYPA182268_05	HYPA182268	2043	2053	1	1	0.879	tcGTCGGcgc
OsSK11	P$PHYPA173530_04	HYPA173530	2044	2052	1	1	0.875	cGTCGGcg
OsSK11	P$PHYPA28324_10	HYPA28324	2044	2052	1	1	0.92	cGTCGGcg
OsSK11	P$AT1G28160_02	AT1G28160	2044	2059	1	1	0.85	cgtCGGCGctgttgt
OsSK11	P$AT1G68550_03	AT1G68550	2044	2053	1	1	0.96	cgtCGGCGc
OsSK11	P$WRKY18_02	WRKY18	2054	2064	1	1	0.947	gttGTCAAcc
OsSK11	P$WRKY21_02	WRKY21	2054	2064	1	1	0.983	gttGTCAAcc
OsSK11	P$WRKY48_02	WRKY48	2054	2064	1	1	0.989	gttGTCAAcc
OsSK11	P$WRKY57_01	WRKY57	2054	2064	1	1	0.973	gttGTCAAcc
OsSK11	P$WRKY60_01	WRKY60	2054	2065	1	1	0.916	gttGTCAAccg
OsSK11	P$WRKY15_01	WRKY15	2055	2065	1	1	0.977	ttGTCAAccg
OsSK11	P$WRKY2_01	WRKY2	2055	2063	1	1	0.919	ttGTCAAc
OsSK11	P$WRKY25_02	WRKY25	2055	2063	1	1	0.913	ttGTCAAc
OsSK11	P$WRKY40_01	WRKY40	2055	2063	1	1	0.977	ttGTCAAc
OsSK11	P$WRKY43_02	WRKY43	2055	2065	1	1	0.961	ttGTCAAccg
OsSK11	P$WRKY62_01	WRKY62	2055	2063	1	1	0.938	ttGTCAAc
OsSK11	P$WRKY63_01	WRKY63	2055	2063	1	1	0.895	ttGTCAAc
OsSK11	P$WRKY75_01	WRKY75	2055	2063	1	1	0.959	ttGTCAAc
OsSK11	P$WRKY8_01	WRKY8	2055	2064	1	1	0.987	ttGTCAAcc
OsSK11	P$WRKY23_01	WRKY23	2056	2064	1	1	0.899	tGTCAAcc
OsSK11	P$WRKY30_01	WRKY30	2056	2066	1	1	0.977	tGTCAAccgt
OsSK11	P$WRKY18_Q2	WRKY18	2057	2066	1	1	0.927	GTCAAccgt
OsSK11	P$GAMYB_01	GAMYB	2059	2067	1	1	0.99	CAACCgtc
OsSK11	P$CBF3_02	CBF3	2082	2096	1	1	0.915	gttgcCCGACgccg
OsSK11	P$CBF1_04	CBF1	2083	2095	1	1	0.906	ttgcCCGACgcc
OsSK11	P$AT2G33710_01	AT2G33710	2084	2099	1	1	0.865	tgcccgaCGCCGcgc
OsSK11	P$ARF5_01	ARF5	2086	2094	1	1	0.884	cCCGACgc
OsSK11	P$DREB1B_01	DREB1B	2087	2092	1	1	1	CCGAC
OsSK11	P$CBF2_03	CBF2	2087	2097	1	1	0.952	ccgaCGCCGc
OsSK11	P$ERF112_02	ERF112	2089	2099	1	1	0.919	gaCGCCGcgc
OsSK11	P$CRF4_01	CRF4	2090	2098	1	1	0.909	aCGCCGcg
OsSK11	P$ERF4_04	ERF4	2090	2098	1	1	0.895	aCGCCGcg
OsSK11	P$ERF069_01	ERF069	2090	2099	1	1	0.994	aCGCCGcgc
OsSK11	P$ERF11_01	ERF11	2090	2100	1	1	0.961	aCGCCGcgcc
OsSK11	P$ATERF14_01	ATERF14	2090	2100	1	1	0.885	acGCCGCgcc
OsSK11	P$ERF4_02	ERF4	2090	2100	1	1	0.883	acGCCGCgcc
OsSK11	P$AT5G25390_01	AT5G25390	2090	2100	1	1	0.859	acGCCGCgcc
OsSK11	P$DREB1A_01	DREB1A	2090	2100	1	1	0.868	acGCCGCgcc
OsSK11	P$ERF4_03	ERF4	2090	2100	1	1	0.904	acGCCGCgcc
OsSK11	P$AT5G25190_01	AT5G25190	2090	2100	1	1	0.852	acGCCGCgcc
OsSK11	P$AT3G61630_01	AT3G61630	2090	2100	1	1	0.871	acGCCGCgcc
OsSK11	P$AT5G43410_01	AT5G43410	2090	2100	1	1	0.885	acGCCGCgcc
OsSK11	P$AT3G16280_01	AT3G16280	2090	2100	1	1	0.896	acGCCGCgcc
OsSK11	P$CRF2_01	CRF2	2090	2098	1	1	0.936	acGCCGCg
OsSK11	P$ERF098_01	ERF098	2090	2098	1	1	0.894	acGCCGCg
OsSK11	P$ERF8_01	ERF8	2091	2101	1	1	0.942	CGCCGcgccg
OsSK11	P$ERF7_02	ERF7	2091	2101	1	1	0.941	cGCCGCgccg
OsSK11	P$ERF4_05	ERF4	2092	2107	1	1	0.883	gccgCGCCGtcgcta
OsSK11	P$RRTF1_02	RRTF1	2094	2104	1	1	0.874	cgCGCCGtcg
OsSK11	P$RAP26_03	RAP26	2094	2104	1	1	0.876	cgCGCCGtcg
OsSK11	P$RAP210_04	RAP210	2094	2104	1	1	0.89	cgCGCCGtcg
OsSK11	P$ERF112_02	ERF112	2094	2104	1	1	0.977	cgCGCCGtcg
OsSK11	P$CRF4_01	CRF4	2095	2103	1	1	0.945	gCGCCGtc
OsSK11	P$ERF4_04	ERF4	2095	2103	1	1	0.954	gCGCCGtc
OsSK11	P$ERF069_01	ERF069	2095	2104	1	1	0.994	gCGCCGtcg
OsSK11	P$ERF11_01	ERF11	2095	2105	1	1	0.989	gCGCCGtcgc
OsSK11	P$ERF8_01	ERF8	2096	2106	1	1	0.986	CGCCGtcgct
OsSK11	P$ERF3_04	ERF3	2096	2104	1	1	0.952	CGCCGtcg
OsSK11	P$AT2G33710_01	AT2G33710	2111	2126	1	1	0.882	tcgtcgaCGCCGtcg
OsSK11	P$CBF2_03	CBF2	2114	2124	1	1	0.957	tcgaCGCCGt
OsSK11	P$ERF4_05	ERF4	2114	2129	1	1	0.877	tcgaCGCCGtcgcca
OsSK11	P$ERF112_02	ERF112	2116	2126	1	1	0.97	gaCGCCGtcg
OsSK11	P$CRF4_01	CRF4	2117	2125	1	1	0.924	aCGCCGtc
OsSK11	P$ERF4_04	ERF4	2117	2125	1	1	0.952	aCGCCGtc
OsSK11	P$ERF069_01	ERF069	2117	2126	1	1	0.993	aCGCCGtcg
OsSK11	P$ERF11_01	ERF11	2117	2127	1	1	0.988	aCGCCGtcgc
OsSK11	P$ERF8_01	ERF8	2118	2128	1	1	0.985	CGCCGtcgcc
OsSK11	P$ERF3_04	ERF3	2118	2126	1	1	0.952	CGCCGtcg
OsSK11	P$CRF3_02	CRF3	2120	2130	1	1	0.891	ccgtCGCCAg

OsSK11	P$UIF1_01	UIF1	2126	2136	1	1	0.972	ccaGATTCgt
OsSK11	P$ARF8_01	ARF8	2157	2166	1	1	0.953	ccTGTCGtc
OsSK11	P$TGA2_Q2	TGA2	2164	2174	1	1	0.959	tCGTCAtcgc
OsSK11	P$LEC2_01	LEC2	2172	2183	1	1	0.942	gcCATGCcccg
OsSK11	P$RRTF1_05	RRTF1	2174	2189	1	1	0.86	catgcccCGGCGgat
OsSK11	P$AT1G28160_02	AT1G28160	2178	2193	1	1	0.858	cccCGGCGgattcgg
OsSK11	P$RAP26_06	RAP26	2178	2193	1	1	0.854	cccCGGCGgattcgg
OsSK11	P$AT1G68550_03	AT1G68550	2178	2187	1	1	0.955	cccCGGCGg
OsSK11	P$HSFA1E_01	HSFA1E	2180	2186	1	1	1	cCGGCG
OsSK11	P$ERF1_Q2	ERF1	2182	2190	1	1	0.887	GGCGGatt
OsSK11	P$AT1G53910_01	AT1G53910	2190	2200	1	1	0.919	cGGCCGgtgc
OsSK11	P$RAP21_02	RAP21	2190	2203	1	1	0.903	cggcCGGTGcggc
OsSK11	P$ERF4_05	ERF4	2212	2227	1	1	0.861	aggaCGCCGtcatct
OsSK11	P$RRTF1_02	RRTF1	2214	2224	1	1	0.866	gaCGCCGtca
OsSK11	P$ERF112_02	ERF112	2214	2224	1	1	0.977	gaCGCCGtca
OsSK11	P$CRF4_01	CRF4	2215	2223	1	1	0.924	aCGCCGtc
OsSK11	P$ERF4_04	ERF4	2215	2223	1	1	0.952	aCGCCGtc
OsSK11	P$ERF069_01	ERF069	2215	2224	1	1	0.994	aCGCCGtca
OsSK11	P$ERF11_01	ERF11	2215	2225	1	1	0.996	aCGCCGtcat
OsSK11	P$ERF8_01	ERF8	2216	2226	1	1	0.993	CGCCGtcatc
OsSK11	P$ERF3_04	ERF3	2216	2224	1	1	0.969	CGCCGtca
OsSK11	P$TGA2_Q2	TGA2	2218	2228	1	1	0.958	cCGTCAtctc
OsSK11	P$CBF1_01	CBF1	2232	2242	1	1	0.935	cTGCCGtcgt
OsSK11	P$DREB6_01	DREB6	2232	2242	1	1	0.852	cTGCCGtcgt
OsSK11	P$JERF3_01	JERF3	2232	2242	1	1	0.897	cTGCCGtcgt
OsSK11	P$DREB1_01	DREB1	2232	2242	1	1	0.853	cTGCCGtcgt
OsSK11	P$CEF1_01	CEF1	2232	2242	1	1	0.899	cTGCCGtcgt
OsSK11	P$JERF1_01	JERF1	2232	2242	1	1	0.927	cTGCCGtcgt
OsSK11	P$CBF1_03	CBF1	2232	2242	1	1	0.94	cTGCCGtcgt
OsSK11	P$DREB1F_01	DREB1F	2232	2242	1	1	0.947	cTGCCGtcgt
OsSK11	P$AT1G33760_01	AT1G33760	2232	2242	1	1	0.865	cTGCCGtcgt
OsSK11	P$ERF73_01	ERF73	2238	2259	1	1	0.895	tcgtgttCGCCGcccgtgcca
OsSK11	P$ERF4_05	ERF4	2241	2256	1	1	0.878	tgttCGCCGcccgtg
OsSK11	P$ABI4_01	ABI4	2242	2253	1	1	0.862	gttcgCCGCCc
OsSK11	P$RRTF1_02	RRTF1	2243	2253	1	1	0.901	ttCGCCGccc
OsSK11	P$RAP26_03	RAP26	2243	2253	1	1	0.919	ttCGCCGccc
OsSK11	P$RAP210_04	RAP210	2243	2253	1	1	0.922	ttCGCCGccc
OsSK11	P$ERF112_02	ERF112	2243	2253	1	1	0.977	ttCGCCGccc
OsSK11	P$CRF4_01	CRF4	2244	2252	1	1	0.978	tCGCCGcc
OsSK11	P$ERF4_04	ERF4	2244	2252	1	1	0.986	tCGCCGcc
OsSK11	P$ERF069_01	ERF069	2244	2253	1	1	0.998	tCGCCGccc
OsSK11	P$ERF11_01	ERF11	2244	2254	1	1	0.989	tCGCCGcccg
OsSK11	P$ERF13_01	ERF13	2244	2254	1	1	0.975	tcGCCGCccg
OsSK11	P$ABI4_03	ABI4	2244	2254	1	1	0.922	tcGCCGCccg
OsSK11	P$WRAF1_01	WRAF1	2244	2254	1	1	0.861	tcGCCGCccg
OsSK11	P$PTI5_01	TI5	2244	2254	1	1	0.947	tcGCCGCccg
OsSK11	P$DREBI5_01	DREBI5	2244	2254	1	1	0.896	tcGCCGCccg
OsSK11	P$AT2G47520_01	AT2G47520	2244	2254	1	1	0.938	tcGCCGCccg
OsSK11	P$DREB2B_01	DREB2B	2244	2254	1	1	0.885	tcGCCGCccg
OsSK11	P$CRF1_02	CRF1	2244	2254	1	1	0.976	tcGCCGCccg
OsSK11	P$OPBP1_01	OPBP1	2244	2254	1	1	0.933	tcGCCGCccg
OsSK11	P$ATERF14_01	ATERF14	2244	2254	1	1	0.954	tcGCCGCccg
OsSK11	P$DREB2A_02	DREB2A	2244	2254	1	1	0.93	tcGCCGCccg
OsSK11	P$ERF1_02	ERF1	2244	2254	1	1	0.91	tcGCCGCccg
OsSK11	P$ERF4_02	ERF4	2244	2254	1	1	0.999	tcGCCGCccg
OsSK11	P$AT5G25390_01	AT5G25390	2244	2254	1	1	0.995	tcGCCGCccg
OsSK11	P$EREBP1_01	EREBP1	2244	2254	1	1	0.924	tcGCCGCccg
OsSK11	P$CBF3_01	CBF3	2244	2254	1	1	0.874	tcGCCGCccg
OsSK11	P$DREBII1_01	DREBII1	2244	2254	1	1	0.88	tcGCCGCccg
OsSK11	P$DREB1A_01	DREB1A	2244	2254	1	1	0.972	tcGCCGCccg
OsSK11	P$TSRF1_01	TSRF1	2244	2254	1	1	0.925	tcGCCGCccg
OsSK11	P$DRF13_01	DRF13	2244	2254	1	1	0.929	tcGCCGCccg
OsSK11	P$ERF4_03	ERF4	2244	2254	1	1	1	tcGCCGCccg
OsSK11	P$ERF2_03	ERF2	2244	2254	1	1	0.921	tcGCCGCccg
OsSK11	P$ERF1B_03	ERF1B	2244	2254	1	1	0.946	tcGCCGCccg
OsSK11	P$RAP26_02	RAP26	2244	2254	1	1	0.854	tcGCCGCccg
OsSK11	P$CBF16_01	CBF16	2244	2254	1	1	0.875	tcGCCGCccg
OsSK11	P$DREB1A_02	DREB1A	2244	2254	1	1	0.889	tcGCCGCccg
OsSK11	P$CBF17_01	CBF17	2244	2254	1	1	0.896	tcGCCGCccg
OsSK11	P$BD1_01	BD1	2244	2254	1	1	0.873	tcGCCGCccg
OsSK11	P$ERF1_05	ERF1	2244	2254	1	1	0.998	tcGCCGCccg
OsSK11	P$AT5G25190_01	AT5G25190	2244	2254	1	1	0.998	tcGCCGCccg
OsSK11	P$ERF105_01	ERF105	2244	2254	1	1	0.968	tcGCCGCccg
OsSK11	P$CBF_01	CBF	2244	2254	1	1	0.901	tcGCCGCccg
OsSK11	P$AT5G11190_01	AT5G11190	2244	2254	1	1	0.987	tcGCCGCccg
OsSK11	P$AT1G68550_01	AT1G68550	2244	2254	1	1	0.983	tcGCCGCccg
OsSK11	P$AT1G77640_01	AT1G77640	2244	2254	1	1	0.952	tcGCCGCccg
OsSK11	P$AT1G28160_01	AT1G28160	2244	2254	1	1	0.883	tcGCCGCccg

OsSK11	P$AT3G61630_01	AT3G61630	2244	2254	1	1	0.999	tcGCCGCccg
OsSK11	P$AT5G43410_01	AT5G43410	2244	2254	1	1	0.955	tcGCCGCccg
OsSK11	P$AT5G07310_01	AT5G07310	2244	2254	1	1	0.922	tcGCCGCccg
OsSK11	P$AT3G16280_01	AT3G16280	2244	2254	1	1	0.896	tcGCCGCccg
OsSK11	P$DREB1A_03	DREB1A	2244	2254	1	1	0.878	tcGCCGCccg
OsSK11	P$AT1G49120_01	AT1G49120	2244	2254	1	1	0.992	tcGCCGCccg
OsSK11	P$DREB2D_01	DREB2D	2244	2254	1	1	0.891	tcGCCGCccg
OsSK11	P$LEP_01	LEP	2244	2254	1	1	0.877	tcGCCGCccg
OsSK11	P$AT3G25890_01	AT3G25890	2244	2254	1	1	0.896	tcGCCGCccg
OsSK11	P$AT4G23750_01	AT4G23750	2244	2254	1	1	0.969	tcGCCGCccg
OsSK11	P$AT4G27950_01	AT4G27950	2244	2254	1	1	0.982	tcGCCGCccg
OsSK11	P$RRTF1_01	RRTF1	2244	2254	1	1	0.955	tcGCCGCccg
OsSK11	P$CEJ1_02	CEJ1	2244	2254	1	1	0.885	tcGCCGCccg
OsSK11	P$CRF2_01	CRF2	2244	2252	1	1	0.988	tcGCCGCc
OsSK11	P$ERF096_01	ERF096	2244	2254	1	1	0.997	tcGCCGCccg
OsSK11	P$ERF098_01	ERF098	2244	2252	1	1	0.999	tcGCCGCc
OsSK11	P$CBF1_02	CBF1	2244	2254	1	1	0.877	tcgCCGCCcg
OsSK11	P$ERF38_02	ERF38	2244	2254	1	1	0.854	tcgcCGCCCg
OsSK11	P$ERF8_01	ERF8	2245	2255	1	1	0.992	CGCCGcccgt
OsSK11	P$ERF3_04	ERF3	2245	2253	1	1	0.968	CGCCGccc
OsSK11	P$OS05G0497200_01	OS05G0497200	2245	2253	1	1	0.964	CGCCGccc
OsSK11	P$ERF1B_06	ERF1B	2245	2253	1	1	0.977	cGCCGCcc
OsSK11	P$ERF7_02	ERF7	2245	2255	1	1	0.994	cGCCGCccgt
OsSK11	P$ERF094_01	ERF094	2245	2253	1	1	0.992	cGCCGCcc
OsSK11	P$ERF2_01	ERF2	2245	2252	1	1	1	cgCCGCC
OsSK11	P$ERF13_02	ERF13	2245	2253	1	1	0.992	cgCCGCCc
OsSK11	P$AT3G63350_01	AT3G63350	2247	2253	1	1	1	CCGCCc
OsSK11	P$BZR1_01	BZR1	2252	2258	1	1	0.897	CGTGCc
OsSK11	P$CMTA2_01	CMTA2	2266	2275	1	1	0.995	acccCGCGT
OsSK11	P$CAMTA1_02	CAMTA1	2266	2278	1	1	0.884	acccCGCGTcca
OsSK11	P$CMTA3_01	CMTA3	2269	2278	1	1	1	cCGCGTcca
OsSK11	P$LEC2_01	LEC2	2274	2285	1	1	0.958	tcCATGCgagc
OsSK11	P$AT1G53910_02	AT1G53910	2283	2304	1	1	0.861	gctcggcccgaGCGGCggcgg
OsSK11	P$AT1G53910_02	AT1G53910	2286	2307	1	1	0.975	cggcccgagcgGCGGCggcgg
OsSK11	P$RRTF1_05	RRTF1	2288	2303	1	1	0.902	gcccgagCGGCGgcg
OsSK11	P$AT1G53910_02	AT1G53910	2289	2310	1	1	0.967	cccgagcggcgGCGGCggcgg
OsSK11	P$RRTF1_05	RRTF1	2291	2306	1	1	0.927	cgagcggCGGCGgcg
OsSK11	P$AT1G28160_02	AT1G28160	2292	2307	1	1	0.973	gagCGGCGgcggcgg
OsSK11	P$RAP26_06	RAP26	2292	2307	1	1	0.963	gagCGGCGgcggcgg
OsSK11	P$AT1G68550_03	AT1G68550	2292	2301	1	1	0.995	gagCGGCGg
OsSK11	P$AT1G53910_02	AT1G53910	2292	2313	1	1	0.962	gagcggcggcgGCGGCggatg
OsSK11	P$RRTF1_05	RRTF1	2294	2309	1	1	0.925	gcggcggCGGCGgcg
OsSK11	P$AT1G28160_02	AT1G28160	2295	2310	1	1	0.997	cggCGGCGgcggcgg
OsSK11	P$RAP26_06	RAP26	2295	2310	1	1	0.987	cggCGGCGgcggcgg
OsSK11	P$AT1G68550_03	AT1G68550	2295	2304	1	1	0.999	cggCGGCGg
OsSK11	P$ERF1_Q2	ERF1	2296	2304	1	1	0.951	GGCGGcgg
OsSK11	P$RRTF1_05	RRTF1	2297	2312	1	1	0.895	gcggcggCGGCGgat
OsSK11	P$AT1G28160_02	AT1G28160	2298	2313	1	1	0.991	cggCGGCGgcggatg
OsSK11	P$RAP26_06	RAP26	2298	2313	1	1	0.976	cggCGGCGgcggatg
OsSK11	P$AT1G68550_03	AT1G68550	2298	2307	1	1	0.999	cggCGGCGg
OsSK11	P$ERF1_Q2	ERF1	2299	2307	1	1	0.951	GGCGGcgg
OsSK11	P$AT1G28160_02	AT1G28160	2301	2316	1	1	0.919	cggCGGCGgatggcg
OsSK11	P$RAP26_06	RAP26	2301	2316	1	1	0.914	cggCGGCGgatggcg
OsSK11	P$AT1G68550_03	AT1G68550	2301	2310	1	1	0.999	cggCGGCGg
OsSK11	P$ERF1_Q2	ERF1	2302	2310	1	1	0.951	GGCGGcgg
OsSK11	P$ERF1_Q2	ERF1	2305	2313	1	1	0.887	GGCGGatg
OsSK11	P$AT1G53910_02	AT1G53910	2310	2331	1	1	0.943	atggcgatgggGCGGCggcag
OsSK11	P$AT1G53910_02	AT1G53910	2313	2334	1	1	0.881	gcgatggggcgGCGGCagcgg
OsSK11	P$RRTF1_05	RRTF1	2315	2330	1	1	0.901	gatggggCGGCGgca
OsSK11	P$AT1G28160_02	AT1G28160	2319	2334	1	1	0.931	gggCGGCGgcagcgg
OsSK11	P$RAP26_06	RAP26	2319	2334	1	1	0.933	gggCGGCGgcagcgg
OsSK11	P$AT1G68550_03	AT1G68550	2319	2328	1	1	0.999	gggCGGCGg
OsSK11	P$AT1G53910_02	AT1G53910	2319	2340	1	1	0.902	gggcggcggcaGCGGCggcgg
OsSK11	P$ERF1_Q2	ERF1	2320	2328	1	1	0.951	GGCGGcgg
OsSK11	P$AT1G53910_02	AT1G53910	2322	2343	1	1	0.972	cggcggcagcgGCGGCggatg
OsSK11	P$ERF1_Q2	ERF1	2323	2331	1	1	0.951	GGCGGcag
OsSK11	P$RRTF1_05	RRTF1	2324	2339	1	1	0.917	gcggcagCGGCGgcg
OsSK11	P$RRTF1_05	RRTF1	2327	2342	1	1	0.9	gcagcggCGGCGgat
OsSK11	P$AT1G28160_02	AT1G28160	2328	2343	1	1	0.973	cagCGGCGgcggatg
OsSK11	P$RAP26_06	RAP26	2328	2343	1	1	0.959	cagCGGCGgcggatg
OsSK11	P$AT1G68550_03	AT1G68550	2328	2337	1	1	0.995	cagCGGCGg
OsSK11	P$AT1G28160_02	AT1G28160	2331	2346	1	1	0.914	cggCGGCGgatggct
OsSK11	P$RAP26_06	RAP26	2331	2346	1	1	0.901	cggCGGCGgatggct
OsSK11	P$AT1G68550_03	AT1G68550	2331	2340	1	1	0.999	cggCGGCGg
OsSK11	P$ERF1_Q2	ERF1	2332	2340	1	1	0.951	GGCGGcgg
OsSK11	P$ERF1_Q2	ERF1	2335	2343	1	1	0.887	GGCGGatg
OsSK11	P$AT1G53910_02	AT1G53910	2342	2363	1	1	0.976	ggctctcggtgGCGGCggcgg
OsSK11	P$PHYPA64121_06	HYPA64121	2344	2357	1	1	0.867	ctcTCGGTggcgg

OsSK11	P$AT1G53910_02	AT1G53910	2345	2366	1	1	0.957	tctcggtggcgGCGGCggatg
OsSK11	P$RRTF1_05	RRTF1	2347	2362	1	1	0.964	tcggtggCGGCGgcg
OsSK11	P$RRTF1_05	RRTF1	2350	2365	1	1	0.896	gtggcggCGGCGgat
OsSK11	P$E2L_Q2	E2L	2351	2358	1	1	0.91	tGGCGGc
OsSK11	P$AT1G28160_02	AT1G28160	2351	2366	1	1	0.994	tggCGGCGgcggatg
OsSK11	P$RAP26_06	RAP26	2351	2366	1	1	0.988	tggCGGCGgcggatg
OsSK11	P$AT1G68550_03	AT1G68550	2351	2360	1	1	0.999	tggCGGCGg
OsSK11	P$ERF1_Q2	ERF1	2352	2360	1	1	0.951	GGCGGcgg
OsSK11	P$AT1G28160_02	AT1G28160	2354	2369	1	1	0.919	cggCGGCGgatggcg
OsSK11	P$RAP26_06	RAP26	2354	2369	1	1	0.914	cggCGGCGgatggcg
OsSK11	P$AT1G68550_03	AT1G68550	2354	2363	1	1	0.999	cggCGGCGg
OsSK11	P$ERF1_Q2	ERF1	2355	2363	1	1	0.951	GGCGGcgg
OsSK11	P$ERF1_Q2	ERF1	2358	2366	1	1	0.887	GGCGGatg
OsSK11	P$AT1G53910_02	AT1G53910	2363	2384	1	1	0.957	atggcgatgggGCGGCggcgg
OsSK11	P$AT1G53910_02	AT1G53910	2366	2387	1	1	0.969	gcgatggggcgGCGGCggcgg
OsSK11	P$RRTF1_05	RRTF1	2368	2383	1	1	0.924	gatggggCGGCGgcg
OsSK11	P$AT1G53910_02	AT1G53910	2369	2390	1	1	0.957	atggggcggcgGCGGCggcac
OsSK11	P$RRTF1_05	RRTF1	2371	2386	1	1	0.925	ggggcggCGGCGgcg
OsSK11	P$AT1G28160_02	AT1G28160	2372	2387	1	1	0.991	gggCGGCGgcggcgg
OsSK11	P$RAP26_06	RAP26	2372	2387	1	1	0.98	gggCGGCGgcggcgg
OsSK11	P$AT1G68550_03	AT1G68550	2372	2381	1	1	0.999	gggCGGCGg
OsSK11	P$AT1G53910_02	AT1G53910	2372	2393	1	1	0.853	gggcggcggcgGCGGCacctg
OsSK11	P$ERF1_Q2	ERF1	2373	2381	1	1	0.951	GGCGGcgg
OsSK11	P$RRTF1_05	RRTF1	2374	2389	1	1	0.902	gcggcggCGGCGgca
OsSK11	P$AT1G28160_02	AT1G28160	2375	2390	1	1	0.984	cggCGGCGgcggcac
OsSK11	P$RAP26_06	RAP26	2375	2390	1	1	0.961	cggCGGCGgcggcac
OsSK11	P$AT1G68550_03	AT1G68550	2375	2384	1	1	0.999	cggCGGCGg
OsSK11	P$ERF1_Q2	ERF1	2376	2384	1	1	0.951	GGCGGcgg
OsSK11	P$AT1G28160_02	AT1G28160	2378	2393	1	1	0.921	cggCGGCGgcacctg
OsSK11	P$RAP26_06	RAP26	2378	2393	1	1	0.909	cggCGGCGgcacctg
OsSK11	P$AT1G68550_03	AT1G68550	2378	2387	1	1	0.999	cggCGGCGg
OsSK11	P$ERF1_Q2	ERF1	2379	2387	1	1	0.951	GGCGGcgg
OsSK11	P$ERF1_Q2	ERF1	2382	2390	1	1	0.957	GGCGGcac
OsSK11	P$AT5G04240_01	AT5G04240	2384	2390	1	1	0.976	cGGCAC
OsSK11	P$RAV1_02	RAV1	2385	2397	1	1	0.982	ggcACCTGttgc
OsSK11	P$REF6_01	REF6	2401	2412	1	1	0.85	taagCAGAGtg
OsSK11	P$PBF_01	BF	2418	2429	1	1	0.97	aggAAAAGggg
OsSK11	P$DOF_Q2	DOF	2418	2429	1	1	0.947	aggAAAAGggg
OsSK11	P$AT3G51080_01	AT3G51080	2419	2426	1	1	0.918	GGAAAag
OsSK11	P$CDF2_01	CDF2	2419	2429	1	1	0.956	ggAAAAGggg
OsSK11	P$CDF3_01	CDF3	2420	2429	1	1	0.982	gAAAAGggg
OsSK11	P$PBF_Q2	BF	2421	2427	1	1	1	aAAAGG
OsSK11	P$BPC1_Q2	BPC1	2445	2451	1	1	1	AGAAAg
OsSK11	P$PBF_Q2	BF	2446	2452	1	1	0.965	gAAAGG
OsSK11	P$KNOX3_01	KNOX3	2459	2471	1	1	0.978	agacTGACAcgt
OsSK11	P$PIL5_01	IL5	2459	2473	1	1	0.988	agactgacACGTGg
OsSK11	P$PIF3_01	IF3	2460	2478	1	1	0.934	gactgaCACGTGgggtccc
OsSK11	P$TCP14_01	TCP14	2460	2480	1	1	0.893	gactgacacGTGGGtcccac
OsSK11	P$PIF3_02	IF3	2461	2478	1	1	0.92	actgaCACGTgggtccc
OsSK11	P$HY5_02	HY5	2461	2477	1	1	0.909	actgaCACGTgggtcc
OsSK11	P$ABF2_01	ABF2	2461	2474	1	1	0.999	actgaCACGTggg
OsSK11	P$O2_Q4	O2	2462	2473	1	1	0.875	ctgaCACGTgg
OsSK11	P$BZR1_02	BZR1	2462	2476	1	1	0.972	ctgaCACGTgggtc
OsSK11	P$ABF3_Q2	ABF3	2462	2476	1	1	0.913	ctgaCACGTgggtc
OsSK11	P$HBI1_01	HBI1	2462	2474	1	1	0.935	ctgaCACGTggg
OsSK11	P$ABZ1_01	ABZ1	2462	2476	1	1	0.938	ctgacACGTGggtc
OsSK11	P$ATH1_01	ATH1	2463	2471	1	1	0.924	TGACAcgt
OsSK11	P$BIM1_01	BIM1	2463	2475	1	1	0.924	tgACACGtgggt
OsSK11	P$GBP_Q6	GBP	2463	2475	1	1	0.926	tgaCACGTgggt
OsSK11	P$GBF_Q2	GBF	2463	2475	1	1	0.863	tgaCACGTgggt
OsSK11	P$PIF3_03	IF3	2463	2473	1	1	0.935	tgaCACGTgg
OsSK11	P$ABI5_01	ABI5	2463	2473	1	1	1	tgaCACGTgg
OsSK11	P$ABF4_01	ABF4	2463	2475	1	1	0.998	tgaCACGTgggt
OsSK11	P$GBF1_Q2_01	GBF1	2463	2474	1	1	0.892	tgacACGTGgg
OsSK11	P$BZR1_03	BZR1	2463	2483	1	1	0.887	tgacACGTGggtcccactgt
OsSK11	P$ALFIN1_Q2	ALFIN1	2463	2478	1	1	0.867	tgacacGTGGGtccc
OsSK11	P$EMBP1_Q2	EMBP1	2464	2474	1	1	0.964	gaCACGTggg
OsSK11	P$CPRF_Q2	CPRF	2464	2474	1	1	0.968	gaCACGTggg
OsSK11	P$CPRF3_Q2	CPRF3	2464	2474	1	1	0.988	gaCACGTggg
OsSK11	P$CPRF2_Q2	CPRF2	2464	2474	1	1	0.996	gaCACGTggg
OsSK11	P$O2_02	O2	2464	2474	1	1	0.964	gaCACGTggg
OsSK11	P$TGA1B_Q2	TGA1B	2464	2474	1	1	0.946	gaCACGTggg
OsSK11	P$TGA1A_Q2	TGA1A	2464	2474	1	1	0.986	gaCACGTggg
OsSK11	P$CPRF1_01	CPRF1	2464	2474	1	1	0.971	gaCACGTggg
OsSK11	P$CPRF3_01	CPRF3	2464	2474	1	1	0.986	gaCACGTggg
OsSK11	P$CPRF2_01	CPRF2	2464	2474	1	1	0.997	gaCACGTggg
OsSK11	P$TGA1B_01	TGA1B	2464	2474	1	1	0.928	gaCACGTggg
OsSK11	P$BES1_01	BES1	2464	2475	1	1	0.977	gaCACGTgggt

OsSK11	P$PIF3_04	IF3	2464	2474	1	1	0.885	gaCACGTggg
OsSK11	P$BEE2_01	BEE2	2464	2474	1	1	0.999	gaCACGTggg
OsSK11	P$BIM2_01	BIM2	2464	2474	1	1	0.994	gaCACGTggg
OsSK11	P$BIM3_01	BIM3	2464	2474	1	1	0.991	gaCACGTggg
OsSK11	P$PHYPA143875_02	HYPA143875	2464	2474	1	1	0.997	gaCACGTggg
OsSK11	P$PHYPA72483_07	HYPA72483	2464	2474	1	1	0.998	gaCACGTggg
OsSK11	P$SPT_01	SPT	2464	2473	1	1	0.969	gaCACGTgg
OsSK11	P$GBF1F_Q2	GBF1F	2464	2475	1	1	0.895	gaCACGTgggt
OsSK11	P$HBP1A_Q2	HBP1A	2464	2474	1	1	0.945	gacACGTGgg
OsSK11	P$TAF1_Q2	TAF1	2464	2474	1	1	0.98	gacACGTGgg
OsSK11	P$EMBP1_02	EMBP1	2464	2474	1	1	0.973	gacACGTGgg
OsSK11	P$TAF1_01	TAF1	2464	2474	1	1	0.991	gacACGTGgg
OsSK11	P$PIF1_01	IF1	2464	2474	1	1	1	gacACGTGgg
OsSK11	P$ABF3_01	ABF3	2465	2473	1	1	0.875	ACACGtgg
OsSK11	P$RITA1_01	RITA1	2465	2472	1	1	0.984	aCACGTg
OsSK11	P$BHLH66_01	BHLH66	2465	2473	1	1	0.93	aCACGTgg
OsSK11	P$PIF5_01	IF5	2465	2473	1	1	0.93	aCACGTgg
OsSK11	P$MYC2_01	MYC2	2465	2473	1	1	0.953	aCACGTgg
OsSK11	P$MYC3_01	MYC3	2465	2473	1	1	0.994	aCACGTgg
OsSK11	P$BHLH34_01	BHLH34	2465	2473	1	1	0.972	aCACGTgg
OsSK11	P$PHYPA48267_08	HYPA48267	2465	2473	1	1	0.971	aCACGTgg
OsSK11	P$OJ1058_01	OJ1058	2465	2473	1	1	1	aCACGTgg
OsSK11	P$UNE10_01	UNE10	2465	2473	1	1	0.98	aCACGTgg
OsSK11	P$BHLH3_01	BHLH3	2465	2473	1	1	0.952	aCACGTgg
OsSK11	P$TRAB1_Q2	TRAB1	2465	2476	1	1	0.925	acACGTGggtc
OsSK11	P$HY5_01	HY5	2465	2475	1	1	0.946	acACGTGggt
OsSK11	P$GBF1_01	GBF1	2465	2473	1	1	0.973	acACGTGg
OsSK11	P$MYC4_01	MYC4	2465	2473	1	1	0.953	acACGTGg
OsSK11	P$BIM1_02	BIM1	2465	2475	1	1	0.995	acACGTGggt
OsSK11	P$BHLH13_01	BHLH13	2465	2473	1	1	0.947	acACGTGg
OsSK11	P$ABF4_02	ABF4	2465	2475	1	1	0.973	acACGTGggt
OsSK11	P$BZIP68_01	BZIP68	2465	2474	1	1	0.978	acaCGTGGg
OsSK11	P$OCSBF1_01	OCSBF1	2466	2471	1	1	1	CACGT
OsSK11	P$PIF4_01	IF4	2466	2474	1	1	0.977	CACGTggg
OsSK11	P$CPRF1_Q2	CPRF1	2466	2476	1	1	0.962	cACGTGggtc
OsSK11	P$ABI5_Q2	ABI5	2467	2473	1	1	1	ACGTGg
OsSK11	P$TCP15_01	TCP15	2469	2479	1	1	0.999	GTGGGtccca
OsSK11	P$TCP20_01	TCP20	2469	2479	1	1	0.99	GTGGGtccca
OsSK11	P$TCP11_02	TCP11	2469	2479	1	1	0.868	GTGGGtccca
OsSK11	P$TCP7_01	TCP7	2469	2480	1	1	0.999	GTGGGtcccac
OsSK11	P$PCF5_01	CF5	2470	2480	1	1	0.886	tgGGTCCcac
OsSK11	P$PCF2_01	CF2	2470	2480	1	1	1	tgggtCCCAC
OsSK11	P$TCP19_01	TCP19	2470	2480	1	1	0.995	tgggtCCCAC
OsSK11	P$TCP20L_01	TCP20L	2471	2480	1	1	0.994	gggtCCCAC
OsSK11	P$OSI_01	OSI	2472	2480	1	1	0.959	ggtCCCAC
OsSK11	P$TCP20_02	TCP20	2472	2482	1	1	0.991	ggtCCCACtg
OsSK11	P$ARALY495258_02	ARALY495258	2472	2480	1	1	0.963	ggtCCCAC
OsSK11	P$ARALY493022_04	ARALY493022	2472	2480	1	1	0.946	ggtCCCAC
OsSK11	P$ARALY484486_05	ARALY484486	2472	2480	1	1	0.963	ggtCCCAC
OsSK11	P$ATHB6_01	ATHB6	2489	2498	1	1	0.976	caAATAAta
OsSK11	P$ATHB5_04	ATHB5	2489	2500	1	1	0.893	caAATAAtaga
OsSK11	P$ATHB1_03	ATHB1	2489	2500	1	1	0.902	caAATAAtaga
OsSK11	P$RIN_01	RIN	2489	2499	1	1	0.864	caaatAATAG
OsSK11	P$ATHB16_01	ATHB16	2490	2498	1	1	0.867	aAATAAta
OsSK11	P$PDF2_01	DF2	2522	2533	1	1	0.859	ggagTAAATaa
OsSK11	P$GT1_Q6	GT1	2525	2532	1	1	1	GTAAAta
OsSK11	P$ATHB6_01	ATHB6	2526	2535	1	1	0.908	taAATAAga
OsSK11	P$WRKY11_Q2	WRKY11	2536	2544	1	1	0.927	tTTGACtg
OsSK11	P$HSFA2_01	HSFA2	2566	2572	1	1	0.922	CCAAAt
OsSK11	P$MYBAS1_01	MYBAS1	2613	2624	1	1	0.949	ctCTAACaaaa
OsSK11	P$WRKY48_01	WRKY48	2613	2622	1	1	0.882	ctctAACAA
OsSK11	P$ATMYB15_Q2	ATMYB15	2616	2622	1	1	1	TAACAa
OsSK11	P$HAT1_01	HAT1	2620	2630	1	1	0.852	aaAATCAcaa
OsSK11	P$AT3G60580_01	AT3G60580	2627	2634	1	1	0.905	caATCCC
OsSK11	P$MYB89_01	MYB89	2652	2663	1	1	0.917	agTACCGctag
OsSK11	P$AT3G01030_01	AT3G01030	2655	2664	1	1	0.905	ACCGCtagg
OsSK11	P$RAV1_02	RAV1	2661	2673	1	1	0.896	aggACCTGtttg
OsSK11	P$KNOX3_01	KNOX3	2667	2679	1	1	0.968	tgttTGACAtag
OsSK11	P$WRKY11_Q2	WRKY11	2669	2677	1	1	0.903	tTTGACat
OsSK11	P$ATH1_01	ATH1	2671	2679	1	1	0.924	TGACAtag
OsSK11	P$GT1_Q6_02	GT1	2688	2700	1	1	0.852	ctaagtTTAACt
OsSK11	P$GATA15_01	GATA15	2726	2735	1	1	0.999	gtTGATCag
OsSK11	P$AT5G61590_01	AT5G61590	2744	2754	1	1	0.909	GCTCCaccta
OsSK11	P$RAV1_02	RAV1	2778	2790	1	1	0.916	tcaACCTGctcc
OsSK11	P$GAMYB_01	GAMYB	2779	2787	1	1	0.919	CAACCtgc
OsSK11	P$HSFA4A_01	HSFA4A	2794	2800	1	1	0.964	cCTATT
OsSK11	P$SBF1_01	SBF1	2794	2808	1	1	0.862	cctattTTAAAatg
OsSK11	P$AT3G20750_01	AT3G20750	2899	2907	1	1	0.912	tTAAACgt

OsSK11	P$NAC043_01	NAC043	2900	2910	1	1	0.888	taaACGTAaa
OsSK11	P$NAC025_01	NAC025	2902	2910	1	1	0.907	aACGTAaa
OsSK11	P$NAC6_01	NAC6	2903	2909	1	1	1	aCGTAA
OsSK11	P$C1_Q2	C1	2906	2917	1	1	0.936	taAACTAatga
OsSK11	P$KNOX3_01	KNOX3	2910	2922	1	1	0.959	ctaaTGACAata
OsSK11	P$ATH1_01	ATH1	2914	2922	1	1	0.92	TGACAata
OsSK11	P$ASR1_01	ASR1	2921	2926	1	1	1	ACCCA
OsSK11	P$C1_Q2	C1	2937	2948	1	1	0.942	tgAACTAattc
OsSK11	P$ARR1_01	ARR1	2952	2962	1	1	0.978	gatGAATCta
OsSK11	P$HSFA4A_01	HSFA4A	2958	2964	1	1	0.914	tCTATT
OsSK11	P$EDT1_01	EDT1	2969	2979	1	1	0.898	taaTTAATcc
OsSK11	P$ATHSFA1D_01	ATHSFA1D	2997	3003	1	1	0.94	gCTACA
OsSK11	P$GT1_Q6	GT1	3003	3010	1	1	0.912	GTAAAca
OsSK11	P$AT3G20750_01	AT3G20750	3003	3011	1	1	0.943	gTAAACat
OsSK11	P$AMS_01	AMS	3006	3016	1	1	0.868	aaCATGTgct
OsSK11	P$SBF1_01	SBF1	3019	3033	1	1	0.86	tatggaTTAATtag
OsSK11	P$EDT1_01	EDT1	3022	3032	1	1	0.859	ggaTTAATta
OsSK11	P$MYB24_01	MYB24	3026	3035	1	1	0.963	taaTTAGGt
OsSK11	P$MYB131_01	MYB131	3026	3037	1	1	0.94	taaTTAGGttt
OsSK11	P$MYB4_01	MYB4	3028	3036	1	1	0.862	atTAGGTt
OsSK11	P$SBF1_01	SBF1	3029	3043	1	1	0.897	ttaggtTTAAAaaa
OsSK11	P$AT1G19490_01	AT1G19490	3032	3041	1	1	0.895	GGTTTaaaa
OsSK11	P$SBF1_01	SBF1	3092	3106	1	1	0.882	ctatgtTTAATact
OsSK11	P$EDT1_01	EDT1	3095	3105	1	1	0.861	tgtTTAATac
OsSK11	P$WRKY18_02	WRKY18	3113	3123	1	1	0.944	agtGTCAAat
OsSK11	P$WRKY21_02	WRKY21	3113	3123	1	1	0.956	agtGTCAAat
OsSK11	P$WRKY48_02	WRKY48	3113	3123	1	1	0.987	agtGTCAAat
OsSK11	P$WRKY57_01	WRKY57	3113	3123	1	1	0.963	agtGTCAAat
OsSK11	P$WRKY60_01	WRKY60	3113	3124	1	1	0.892	agtGTCAAata
OsSK11	P$WRKY15_01	WRKY15	3114	3124	1	1	0.964	gtGTCAAata
OsSK11	P$WRKY2_01	WRKY2	3114	3122	1	1	0.909	gtGTCAAa
OsSK11	P$WRKY25_02	WRKY25	3114	3122	1	1	0.885	gtGTCAAa
OsSK11	P$WRKY40_01	WRKY40	3114	3122	1	1	0.977	gtGTCAAa
OsSK11	P$WRKY43_02	WRKY43	3114	3124	1	1	0.952	gtGTCAAata
OsSK11	P$WRKY63_01	WRKY63	3114	3122	1	1	0.885	gtGTCAAa
OsSK11	P$WRKY75_01	WRKY75	3114	3122	1	1	0.935	gtGTCAAa
OsSK11	P$WRKY8_01	WRKY8	3114	3123	1	1	0.979	gtGTCAAat
OsSK11	P$WRKY30_01	WRKY30	3115	3125	1	1	0.899	tGTCAAatat
OsSK11	P$WRKY18_Q2	WRKY18	3116	3125	1	1	0.954	GTCAAatat
OsSK11	P$PBF_01	BF	3123	3134	1	1	0.993	attAAAAGaga
OsSK11	P$DOF_Q2	DOF	3123	3134	1	1	0.94	attAAAAGaga
OsSK11	P$CDF2_01	CDF2	3124	3134	1	1	0.952	ttAAAAGaga
OsSK11	P$CDF3_01	CDF3	3125	3134	1	1	0.979	tAAAAGaga
OsSK11	P$GT1_Q6	GT1	3174	3181	1	1	0.971	GTGAAaa
OsSK11	P$ATHB6_01	ATHB6	3177	3186	1	1	0.979	aaAATAAtt
OsSK11	P$ATHB5_04	ATHB5	3177	3188	1	1	0.957	aaAATAAtttt
OsSK11	P$ATHB1_03	ATHB1	3177	3188	1	1	0.974	aaAATAAtttt
OsSK11	P$ATHB16_01	ATHB16	3178	3186	1	1	0.953	aAATAAtt
OsSK11	P$TGA1_01	TGA1	3203	3214	1	1	0.936	ggaTGACGatc
OsSK11	P$TGA7_01	TGA7	3204	3214	1	1	0.854	gaTGACGatc
OsSK11	P$TGA5_01	TGA5	3205	3213	1	1	0.869	aTGACGat
OsSK11	P$ATSPL8_01	ATSPL8	3210	3226	1	1	0.872	gatccTGTACcgagca
OsSK11	P$MYB89_01	MYB89	3215	3226	1	1	0.952	tgTACCGagca
OsSK11	P$ABF2_01	ABF2	3233	3246	1	1	0.897	actagCACGTtcc
OsSK11	P$HBI1_01	HBI1	3234	3246	1	1	0.869	ctagCACGTtcc
OsSK11	P$GBP_Q6	GBP	3235	3247	1	1	0.95	tagCACGTtcca
OsSK11	P$ABI5_01	ABI5	3235	3245	1	1	0.884	tagCACGTtc
OsSK11	P$ABF4_01	ABF4	3235	3247	1	1	0.866	tagCACGTtcca
OsSK11	P$CPRF3_Q2	CPRF3	3236	3246	1	1	0.901	agCACGTtcc
OsSK11	P$CPRF2_Q2	CPRF2	3236	3246	1	1	0.917	agCACGTtcc
OsSK11	P$O2_02	O2	3236	3246	1	1	0.913	agCACGTtcc
OsSK11	P$TGA1B_Q2	TGA1B	3236	3246	1	1	0.89	agCACGTtcc
OsSK11	P$TGA1A_Q2	TGA1A	3236	3246	1	1	0.959	agCACGTtcc
OsSK11	P$CPRF3_01	CPRF3	3236	3246	1	1	0.906	agCACGTtcc
OsSK11	P$CPRF2_01	CPRF2	3236	3246	1	1	0.918	agCACGTtcc
OsSK11	P$TGA1B_01	TGA1B	3236	3246	1	1	0.883	agCACGTtcc
OsSK11	P$BEE2_01	BEE2	3236	3246	1	1	0.921	agCACGTtcc
OsSK11	P$BIM2_01	BIM2	3236	3246	1	1	0.858	agCACGTtcc
OsSK11	P$BIM3_01	BIM3	3236	3246	1	1	0.893	agCACGTtcc
OsSK11	P$PHYPA143875_02	HYPA143875	3236	3246	1	1	0.889	agCACGTtcc
OsSK11	P$SPT_01	SPT	3236	3245	1	1	0.925	agCACGTtc
OsSK11	P$TSAR1_01	TSAR1	3236	3246	1	1	0.908	agCACGTtcc
OsSK11	P$BHLH78_01	BHLH78	3237	3245	1	1	0.875	GCACGttc
OsSK11	P$RITA1_01	RITA1	3237	3244	1	1	0.943	gCACGTt
OsSK11	P$MYC2_01	MYC2	3237	3245	1	1	0.861	gCACGTtc
OsSK11	P$MYC3_01	MYC3	3237	3245	1	1	0.859	gCACGTtc
OsSK11	P$BHLH3_01	BHLH3	3237	3245	1	1	0.856	gCACGTtc
OsSK11	P$AIB_01	AIB	3237	3245	1	1	0.878	gCACGTtc

OsSK11	P$OCSBF1_01	OCSBF1	3238	3243	1	1	1	CACGT
OsSK11	P$HSFA2_01	HSFA2	3254	3260	1	1	1	CCAAAa
OsSK11	P$PBF_01	BF	3254	3265	1	1	0.954	ccaAAAAGgta
OsSK11	P$DOF_Q2	DOF	3254	3265	1	1	0.978	ccaAAAAGgta
OsSK11	P$CDF2_01	CDF2	3255	3265	1	1	0.979	caAAAAGgta
OsSK11	P$CDF3_01	CDF3	3256	3265	1	1	0.977	aAAAAGgta
OsSK11	P$PBF_Q2	BF	3257	3263	1	1	1	aAAAGG
OsSK11	P$ATHSFA1D_01	ATHSFA1D	3264	3270	1	1	1	aCTACA
OsSK11	P$SED_Q2	SED	3275	3285	1	1	0.967	ttgcCCTTTc
OsSK11	P$PBF_Q2_01	BF	3279	3285	1	1	0.985	CCTTTc
OsSK11	P$ATSPL8_01	ATSPL8	3285	3301	1	1	0.897	catccTGTACagcctc
OsSK11	P$ARR1_01	ARR1	3327	3337	1	1	0.942	tttGAATCaa
OsSK11	P$ATHB7_01	ATHB7	3329	3339	1	1	0.876	tgAATCAagc
OsSK11	P$HAT1_01	HAT1	3329	3339	1	1	0.869	tgAATCAagc
OsSK11	P$ATSPL8_01	ATSPL8	3335	3351	1	1	0.925	aagcgTGTACtactac
OsSK11	P$P_01		3348	3357	1	1	0.876	taCTACCca
OsSK11	P$ASR1_01	ASR1	3352	3357	1	1	1	ACCCA
OsSK11	P$ASR1_01	ASR1	3389	3394	1	1	1	ACCCA
OsSK11	P$AT3G60580_01	AT3G60580	3391	3398	1	1	0.873	ccATCCC
OsSK11	P$LIM1_01	LIM1	3396	3408	1	1	0.859	CCACCtccacca
OsSK11	P$LIM1_01	LIM1	3402	3414	1	1	0.969	CCACCaccacca
OsSK11	P$LOB_01	LOB	3404	3425	1	1	0.882	accaccaccacctgCGCCGca
OsSK11	P$LIM1_01	LIM1	3405	3417	1	1	0.976	CCACCaccacct
OsSK11	P$LIM1_01	LIM1	3408	3420	1	1	0.947	CCACCacctgcg
OsSK11	P$RAV1_02	RAV1	3410	3422	1	1	0.988	accACCTGcgcc
OsSK11	P$LIM1_01	LIM1	3411	3423	1	1	0.86	CCACCtgcgccg
OsSK11	P$AT2G33710_01	AT2G33710	3411	3426	1	1	0.916	ccacctgCGCCGcaa
OsSK11	P$ERF73_01	ERF73	3411	3432	1	1	0.891	ccacctgCGCCGcaagcatca
OsSK11	P$ERF4_05	ERF4	3414	3429	1	1	0.866	cctgCGCCGcaagca
OsSK11	P$RRTF1_02	RRTF1	3416	3426	1	1	0.938	tgCGCCGcaa
OsSK11	P$RAP26_03	RAP26	3416	3426	1	1	0.874	tgCGCCGcaa
OsSK11	P$RAP210_04	RAP210	3416	3426	1	1	0.871	tgCGCCGcaa
OsSK11	P$ERF112_02	ERF112	3416	3426	1	1	0.954	tgCGCCGcaa
OsSK11	P$CRF4_01	CRF4	3417	3425	1	1	0.933	gCGCCGca
OsSK11	P$ERF4_04	ERF4	3417	3425	1	1	0.907	gCGCCGca
OsSK11	P$ERF069_01	ERF069	3417	3426	1	1	0.996	gCGCCGcaa
OsSK11	P$ERF11_01	ERF11	3417	3427	1	1	0.983	gCGCCGcaag
OsSK11	P$PTI5_01	TI5	3417	3427	1	1	0.876	gcGCCGCaag
OsSK11	P$ATERF14_01	ATERF14	3417	3427	1	1	0.853	gcGCCGCaag
OsSK11	P$ERF4_02	ERF4	3417	3427	1	1	0.852	gcGCCGCaag
OsSK11	P$AT3G61630_01	AT3G61630	3417	3427	1	1	0.865	gcGCCGCaag
OsSK11	P$AT5G43410_01	AT5G43410	3417	3427	1	1	0.861	gcGCCGCaag
OsSK11	P$CRF2_01	CRF2	3417	3425	1	1	0.951	gcGCCGCa
OsSK11	P$ERF098_01	ERF098	3417	3425	1	1	0.9	gcGCCGCa
OsSK11	P$ERF8_01	ERF8	3418	3428	1	1	0.962	CGCCGcaagc
OsSK11	P$ERF3_04	ERF3	3418	3426	1	1	0.87	CGCCGcaa
OsSK11	P$ERF7_02	ERF7	3418	3428	1	1	0.961	cGCCGCaagc
OsSK11	P$LOB_01	LOB	3462	3483	1	1	0.916	tcctcctcccctgcCGCCGgt
OsSK11	P$AT2G33710_01	AT2G33710	3469	3484	1	1	0.861	cccctgcCGCCGgtg
OsSK11	P$CBF1_01	CBF1	3472	3482	1	1	0.992	cTGCCGccgg
OsSK11	P$ERF019_01	ERF019	3472	3482	1	1	0.903	cTGCCGccgg
OsSK11	P$DREB6_01	DREB6	3472	3482	1	1	0.957	cTGCCGccgg
OsSK11	P$JERF3_01	JERF3	3472	3482	1	1	0.926	cTGCCGccgg
OsSK11	P$DREB1_01	DREB1	3472	3482	1	1	0.957	cTGCCGccgg
OsSK11	P$CEF1_01	CEF1	3472	3482	1	1	0.926	cTGCCGccgg
OsSK11	P$JERF1_01	JERF1	3472	3482	1	1	0.957	cTGCCGccgg
OsSK11	P$CBF1_03	CBF1	3472	3482	1	1	0.985	cTGCCGccgg
OsSK11	P$AT1G71450_01	AT1G71450	3472	3482	1	1	0.885	cTGCCGccgg
OsSK11	P$DREB1F_01	DREB1F	3472	3482	1	1	0.996	cTGCCGccgg
OsSK11	P$AT1G33760_01	AT1G33760	3472	3482	1	1	0.932	cTGCCGccgg
OsSK11	P$AT1G71520_01	AT1G71520	3472	3482	1	1	0.867	cTGCCGccgg
OsSK11	P$AT4G28140_01	AT4G28140	3472	3482	1	1	0.888	cTGCCGccgg
OsSK11	P$DREB1E_02	DREB1E	3472	3482	1	1	0.95	cTGCCGccgg
OsSK11	P$ORA47_01	ORA47	3472	3482	1	1	0.904	cTGCCGccgg
OsSK11	P$DREB2F_01	DREB2F	3472	3482	1	1	0.908	cTGCCGccgg
OsSK11	P$ERF13_01	ERF13	3472	3482	1	1	0.866	ctGCCGCcgg
OsSK11	P$ABI4_03	ABI4	3472	3482	1	1	0.964	ctGCCGCcgg
OsSK11	P$WRAF1_01	WRAF1	3472	3482	1	1	0.879	ctGCCGCcgg
OsSK11	P$PTI5_01	TI5	3472	3482	1	1	0.927	ctGCCGCcgg
OsSK11	P$DREBI5_01	DREBI5	3472	3482	1	1	0.982	ctGCCGCcgg
OsSK11	P$AT2G47520_01	AT2G47520	3472	3482	1	1	0.949	ctGCCGCcgg
OsSK11	P$DREB2B_01	DREB2B	3472	3482	1	1	0.956	ctGCCGCcgg
OsSK11	P$CRF1_02	CRF1	3472	3482	1	1	0.914	ctGCCGCcgg
OsSK11	P$AT1G77200_01	AT1G77200	3472	3482	1	1	0.861	ctGCCGCcgg
OsSK11	P$OPBP1_01	OPBP1	3472	3482	1	1	0.901	ctGCCGCcgg
OsSK11	P$ATERF14_01	ATERF14	3472	3482	1	1	0.908	ctGCCGCcgg
OsSK11	P$DREB2A_02	DREB2A	3472	3482	1	1	0.928	ctGCCGCcgg
OsSK11	P$ERF1_02	ERF1	3472	3482	1	1	0.939	ctGCCGCcgg

OsSK11	P$ERF4_02	ERF4	3472	3482	1	1	0.939	ctGCCGCcgg
OsSK11	P$AT5G25390_01	AT5G25390	3472	3482	1	1	0.913	ctGCCGCcgg
OsSK11	P$EREBP1_01	EREBP1	3472	3482	1	1	0.936	ctGCCGCcgg
OsSK11	P$CBF3_01	CBF3	3472	3482	1	1	0.983	ctGCCGCcgg
OsSK11	P$DREBII1_01	DREBII1	3472	3482	1	1	0.983	ctGCCGCcgg
OsSK11	P$TSRF1_01	TSRF1	3472	3482	1	1	0.89	ctGCCGCcgg
OsSK11	P$DRF13_01	DRF13	3472	3482	1	1	0.967	ctGCCGCcgg
OsSK11	P$ERF2_03	ERF2	3472	3482	1	1	0.938	ctGCCGCcgg
OsSK11	P$ERF1B_03	ERF1B	3472	3482	1	1	0.897	ctGCCGCcgg
OsSK11	P$RAP26_02	RAP26	3472	3482	1	1	0.87	ctGCCGCcgg
OsSK11	P$CBF5_01	CBF5	3472	3482	1	1	0.98	ctGCCGCcgg
OsSK11	P$CBF16_01	CBF16	3472	3482	1	1	0.982	ctGCCGCcgg
OsSK11	P$CBF17_01	CBF17	3472	3482	1	1	0.984	ctGCCGCcgg
OsSK11	P$ERF1_05	ERF1	3472	3482	1	1	0.907	ctGCCGCcgg
OsSK11	P$AT5G25190_01	AT5G25190	3472	3482	1	1	0.916	ctGCCGCcgg
OsSK11	P$ERF105_01	ERF105	3472	3482	1	1	0.925	ctGCCGCcgg
OsSK11	P$CBF_01	CBF	3472	3482	1	1	0.982	ctGCCGCcgg
OsSK11	P$AT5G11190_01	AT5G11190	3472	3482	1	1	0.915	ctGCCGCcgg
OsSK11	P$AT1G68550_01	AT1G68550	3472	3482	1	1	0.997	ctGCCGCcgg
OsSK11	P$AT1G77640_01	AT1G77640	3472	3482	1	1	0.893	ctGCCGCcgg
OsSK11	P$ERF016_01	ERF016	3472	3482	1	1	0.999	ctGCCGCcgg
OsSK11	P$AT3G61630_01	AT3G61630	3472	3482	1	1	0.965	ctGCCGCcgg
OsSK11	P$AT5G43410_01	AT5G43410	3472	3482	1	1	0.916	ctGCCGCcgg
OsSK11	P$TINY2_02	TINY2	3472	3482	1	1	0.85	ctGCCGCcgg
OsSK11	P$AT5G07310_01	AT5G07310	3472	3482	1	1	0.932	ctGCCGCcgg
OsSK11	P$AT3G16280_01	AT3G16280	3472	3482	1	1	0.864	ctGCCGCcgg
OsSK11	P$DREB1A_03	DREB1A	3472	3482	1	1	0.983	ctGCCGCcgg
OsSK11	P$AT1G49120_01	AT1G49120	3472	3482	1	1	0.871	ctGCCGCcgg
OsSK11	P$DREB2D_01	DREB2D	3472	3482	1	1	0.97	ctGCCGCcgg
OsSK11	P$AT3G25890_01	AT3G25890	3472	3482	1	1	0.985	ctGCCGCcgg
OsSK11	P$AT4G23750_01	AT4G23750	3472	3482	1	1	0.891	ctGCCGCcgg
OsSK11	P$AT4G27950_01	AT4G27950	3472	3482	1	1	0.903	ctGCCGCcgg
OsSK11	P$RRTF1_01	RRTF1	3472	3482	1	1	0.923	ctGCCGCcgg
OsSK11	P$CEJ1_02	CEJ1	3472	3482	1	1	0.875	ctGCCGCcgg
OsSK11	P$ERF1_Q2_01	ERF1	3472	3486	1	1	0.873	ctGCCGCcggtgac
OsSK11	P$CRF2_01	CRF2	3472	3480	1	1	0.933	ctGCCGCc
OsSK11	P$ERF096_01	ERF096	3472	3482	1	1	0.983	ctGCCGCcgg
OsSK11	P$ERF098_01	ERF098	3472	3480	1	1	0.997	ctGCCGCc
OsSK11	P$DREB2C_01	DREB2C	3472	3482	1	1	0.958	ctgCCGCCgg
OsSK11	P$CBF1_02	CBF1	3472	3482	1	1	0.887	ctgCCGCCgg
OsSK11	P$CBF2_03	CBF2	3472	3482	1	1	0.865	ctgcCGCCGg
OsSK11	P$ERF1B_06	ERF1B	3473	3481	1	1	0.946	tGCCGCcg
OsSK11	P$ERF7_02	ERF7	3473	3483	1	1	0.984	tGCCGCcggt
OsSK11	P$ERF094_01	ERF094	3473	3481	1	1	0.979	tGCCGCcg
OsSK11	P$ERF2_01	ERF2	3473	3480	1	1	0.929	tgCCGCC
OsSK11	P$ERF13_02	ERF13	3473	3481	1	1	0.978	tgCCGCCg
OsSK11	P$ERF112_02	ERF112	3474	3484	1	1	0.921	gcCGCCGgtg
OsSK11	P$AT3G63350_01	AT3G63350	3475	3481	1	1	0.866	CCGCCg
OsSK11	P$CRF4_01	CRF4	3475	3483	1	1	0.861	cCGCCGgt
OsSK11	P$ERF4_04	ERF4	3475	3483	1	1	0.869	cCGCCGgt
OsSK11	P$ERF069_01	ERF069	3475	3484	1	1	0.989	cCGCCGgtg
OsSK11	P$ERF11_01	ERF11	3475	3485	1	1	0.965	cCGCCGgtga
OsSK11	P$RAP21_02	RAP21	3475	3488	1	1	0.904	ccgcCGGTGaccg
OsSK11	P$ERF8_01	ERF8	3476	3486	1	1	0.941	CGCCGgtgac
OsSK11	P$MYB3R5_01	MYB3R5	3476	3491	1	1	0.889	cgccggtgaCCGTTc
OsSK11	P$MYB3R1_01	MYB3R1	3477	3492	1	1	0.918	gccggtgaCCGTTcc
OsSK11	P$MYB3R4_01	MYB3R4	3477	3492	1	1	0.921	gccggtgaCCGTTcc
OsSK11	P$GATA9_01	GATA9	3526	3537	1	1	0.9	cccAGATCaga
OsSK11	P$AGP1_01	AGP1	3527	3537	1	1	0.869	ccAGATCaga
OsSK11	P$GATA10_01	GATA10	3528	3536	1	1	0.881	cAGATCag
OsSK11	P$ARR10_01	ARR10	3529	3536	1	1	0.934	AGATCag
OsSK11	P$GATA9_01	GATA9	3531	3542	1	1	0.985	atcAGATCtaa
OsSK11	P$AGP1_01	AGP1	3532	3542	1	1	1	tcAGATCtaa
OsSK11	P$GATA10_01	GATA10	3533	3541	1	1	0.916	cAGATCta
OsSK11	P$GATA11_01	GATA11	3533	3541	1	1	0.939	caGATCTa
OsSK11	P$GATA8_01	GATA8	3533	3542	1	1	1	caGATCTaa
OsSK11	P$ARR10_01	ARR10	3534	3541	1	1	0.913	AGATCta
OsSK11	P$MYBAS1_01	MYBAS1	3536	3547	1	1	0.948	atCTAACcccg
OsSK11	P$CBF3_02	CBF3	3539	3553	1	1	0.913	taaccCCGACccaa
OsSK11	P$CBF1_04	CBF1	3540	3552	1	1	0.899	aaccCCGACcca
OsSK11	P$ARF5_01	ARF5	3543	3551	1	1	0.912	cCCGACcc
OsSK11	P$DREB1B_01	DREB1B	3544	3549	1	1	1	CCGAC
OsSK11	P$ASR1_01	ASR1	3547	3552	1	1	1	ACCCA
OsSK11	P$MYBAS1_01	MYBAS1	3547	3558	1	1	0.962	acCCAACccca
OsSK11	P$GAMYB_01	GAMYB	3550	3558	1	1	0.893	CAACCcca
OsSK11	P$MYBAS1_01	MYBAS1	3553	3564	1	1	0.945	ccCCAACcagg
OsSK11	P$AT4G36620_01	AT4G36620	3554	3562	1	1	0.885	cccAACCA
OsSK11	P$GAMYB_01	GAMYB	3556	3564	1	1	0.873	CAACCagg

OsSK11	P$UIF1_01	UIF1	3565	3575	1	1	0.973	gcaGATTCgt
OsSK11	P$AT3G62240_01	AT3G62240	3570	3580	1	1	0.871	ttcgtCATTC
OsSK11	P$GL15_01	GL15	3576	3586	1	1	0.854	attctCCCCC
OsSK11	P$SED_Q2	SED	3586	3596	1	1	0.909	cacaCCTTTa
OsSK11	P$PBF_Q2_01	BF	3590	3596	1	1	0.998	CCTTTa
OsSK11	P$ARR1_01	ARR1	3596	3606	1	1	0.995	ttcGAATCtt
OsSK11	P$ARR2_01	ARR2	3597	3607	1	1	0.997	tcgaATCTTg
OsSK11	P$SED_Q2	SED	3606	3616	1	1	0.942	gcgtCCTTTg
OsSK11	P$PBF_Q2_01	BF	3610	3616	1	1	0.988	CCTTTg
OsSK11	P$GT1_Q6	GT1	3621	3628	1	1	0.971	GTAAAaa
OsSK11	P$MYBAS1_01	MYBAS1	3648	3659	1	1	0.962	caCCAACacca
OsSK11	P$RAV1_01	RAV1	3648	3660	1	1	0.933	cacCAACAccaa
OsSK11	P$MYBAS1_01	MYBAS1	3654	3665	1	1	0.952	caCCAACaata
OsSK11	P$RAV1_01	RAV1	3654	3666	1	1	0.949	cacCAACAataa
OsSK11	P$ATHB6_01	ATHB6	3659	3668	1	1	0.997	acAATAAtg
OsSK11	P$ATHB5_04	ATHB5	3659	3670	1	1	0.922	acAATAAtgac
OsSK11	P$ATHB1_03	ATHB1	3659	3670	1	1	0.902	acAATAAtgac
OsSK11	P$ATHB16_01	ATHB16	3660	3668	1	1	0.878	cAATAAtg
OsSK11	P$TGA1_01	TGA1	3686	3697	1	1	0.932	cacTGACGcgt
OsSK11	P$TGA7_01	TGA7	3687	3697	1	1	0.863	acTGACGcgt
OsSK11	P$TGA5_01	TGA5	3688	3696	1	1	0.877	cTGACGcg
OsSK11	P$CMTA2_01	CMTA2	3688	3697	1	1	0.988	ctgaCGCGT
OsSK11	P$CAMTA1_02	CAMTA1	3688	3700	1	1	0.911	ctgaCGCGTggg
OsSK11	P$ALFIN1_Q2	ALFIN1	3689	3704	1	1	0.885	tgacgcGTGGGccat
OsSK11	P$CMTA3_01	CMTA3	3691	3700	1	1	0.985	aCGCGTggg
OsSK11	P$BZIP68_01	BZIP68	3691	3700	1	1	0.928	acgCGTGGg
OsSK11	P$TCP11_01	TCP11	3694	3706	1	1	0.954	cGTGGGccatcg
OsSK11	P$OJ1581_01	OJ1581	3712	3722	1	1	0.99	atGGGCCcca
OsSK11	P$TCP2_01	TCP2	3712	3722	1	1	0.97	atGGGCCcca
OsSK11	P$PCF2_01	CF2	3713	3723	1	1	1	tgggcCCCAC
OsSK11	P$TCP19_01	TCP19	3713	3723	1	1	0.998	tgggcCCCAC
OsSK11	P$TCP20L_01	TCP20L	3714	3723	1	1	0.997	gggcCCCAC
OsSK11	P$OSI_01	OSI	3715	3723	1	1	0.959	ggcCCCAC
OsSK11	P$TCP20_02	TCP20	3715	3725	1	1	0.997	ggcCCCACtc
OsSK11	P$ARALY495258_02	ARALY495258	3715	3723	1	1	1	ggcCCCAC
OsSK11	P$ARALY493022_04	ARALY493022	3715	3723	1	1	0.973	ggcCCCAC
OsSK11	P$ARALY484486_05	ARALY484486	3715	3723	1	1	1	ggcCCCAC
OsSK11	P$HSF3_01	HSF3	3738	3744	1	1	0.969	aCGGGG
OsSK11	P$WRKY11_Q2	WRKY11	3777	3785	1	1	0.974	tTTGACcg
OsSK11	P$ZAP1_01	ZAP1	3778	3788	1	1	0.933	TTGACcggtc
OsSK11	P$WRKY18_02	WRKY18	3782	3792	1	1	0.996	ccgGTCAAaa
OsSK11	P$WRKY21_02	WRKY21	3782	3792	1	1	0.968	ccgGTCAAaa
OsSK11	P$WRKY48_02	WRKY48	3782	3792	1	1	0.998	ccgGTCAAaa
OsSK11	P$WRKY57_01	WRKY57	3782	3792	1	1	0.971	ccgGTCAAaa
OsSK11	P$WRKY60_01	WRKY60	3782	3793	1	1	0.978	ccgGTCAAaag
OsSK11	P$WRKY15_01	WRKY15	3783	3793	1	1	0.985	cgGTCAAaag
OsSK11	P$WRKY2_01	WRKY2	3783	3791	1	1	0.991	cgGTCAAa
OsSK11	P$WRKY25_02	WRKY25	3783	3791	1	1	0.979	cgGTCAAa
OsSK11	P$WRKY40_01	WRKY40	3783	3791	1	1	1	cgGTCAAa
OsSK11	P$WRKY43_02	WRKY43	3783	3793	1	1	0.977	cgGTCAAaag
OsSK11	P$WRKY62_01	WRKY62	3783	3791	1	1	0.91	cgGTCAAa
OsSK11	P$WRKY63_01	WRKY63	3783	3791	1	1	0.991	cgGTCAAa
OsSK11	P$WRKY75_01	WRKY75	3783	3791	1	1	0.975	cgGTCAAa
OsSK11	P$WRKY8_01	WRKY8	3783	3792	1	1	0.992	cgGTCAAaa
OsSK11	P$WRKY23_01	WRKY23	3784	3792	1	1	0.854	gGTCAAaa
OsSK11	P$WRKY30_01	WRKY30	3784	3794	1	1	0.916	gGTCAAaagg
OsSK11	P$WRKY18_Q2	WRKY18	3785	3794	1	1	0.954	GTCAAaagg
OsSK11	P$PBF_01	BF	3785	3796	1	1	0.96	gtcAAAAGgcg
OsSK11	P$DOF_Q2	DOF	3785	3796	1	1	0.922	gtcAAAAGgcg
OsSK11	P$CDF2_01	CDF2	3786	3796	1	1	0.944	tcAAAAGgcg
OsSK11	P$CDF3_01	CDF3	3787	3796	1	1	0.968	cAAAAGgcg
OsSK11	P$PBF_Q2	BF	3788	3794	1	1	1	aAAAGG
OsSK11	P$BZIP68_01	BZIP68	3791	3800	1	1	0.947	aggCGTGGc
OsSK11	P$AT4G36620_01	AT4G36620	3801	3809	1	1	0.89	acgAACCA
OsSK11	P$HSFA2_01	HSFA2	3806	3812	1	1	0.941	CCAAAc
OsSK11	P$E2L_Q2	E2L	3816	3823	1	1	0.867	aGGCGGc
OsSK11	P$ERF1_Q2	ERF1	3817	3825	1	1	0.951	GGCGGcag
OsSK11	P$TCP14_01	TCP14	3819	3839	1	1	0.929	cggcagcagGTGGGccccac
OsSK11	P$ALFIN1_Q2	ALFIN1	3822	3837	1	1	0.866	cagcagGTGGGcccc
OsSK11	P$AMS_01	AMS	3823	3833	1	1	0.909	agCAGGTggg
OsSK11	P$TCP11_01	TCP11	3827	3839	1	1	0.991	gGTGGGccccac
OsSK11	P$TCP15_01	TCP15	3828	3838	1	1	1	GTGGGcccca
OsSK11	P$TCP20_01	TCP20	3828	3838	1	1	0.99	GTGGGcccca
OsSK11	P$TCP11_02	TCP11	3828	3838	1	1	0.998	GTGGGcccca
OsSK11	P$TCP7_01	TCP7	3828	3839	1	1	0.998	GTGGGccccac
OsSK11	P$OJ1581_01	OJ1581	3828	3838	1	1	0.994	gtGGGCCcca
OsSK11	P$TCP2_01	TCP2	3828	3838	1	1	0.974	gtGGGCCcca
OsSK11	P$PCF2_01	CF2	3829	3839	1	1	1	tgggcCCCAC

OsSK11	P$TCP19_01	TCP19	3829	3839	1	1	0.998	tgggcCCCAC
OsSK11	P$TCP20L_01	TCP20L	3830	3839	1	1	0.997	gggcCCCAC
OsSK11	P$OSI_01	OSI	3831	3839	1	1	0.959	ggcCCCAC
OsSK11	P$TCP20_02	TCP20	3831	3841	1	1	0.998	ggcCCCACct
OsSK11	P$ARALY495258_02	ARALY495258	3831	3839	1	1	1	ggcCCCAC
OsSK11	P$ARALY493022_04	ARALY493022	3831	3839	1	1	0.973	ggcCCCAC
OsSK11	P$ARALY484486_05	ARALY484486	3831	3839	1	1	1	ggcCCCAC
OsSK11	P$RAV1_02	RAV1	3834	3846	1	1	0.98	cccACCTGtctg
OsSK11	P$ALFIN1_Q2	ALFIN1	3839	3854	1	1	0.862	ctgtctGTGGGcgtg
OsSK11	P$TCP11_01	TCP11	3844	3856	1	1	0.851	tGTGGGcgtggg
OsSK11	P$ALFIN1_Q2	ALFIN1	3845	3860	1	1	0.872	gtgggcGTGGGcccc
OsSK11	P$BZIP68_01	BZIP68	3847	3856	1	1	0.925	gggCGTGGg
OsSK11	P$TCP11_01	TCP11	3850	3862	1	1	0.958	cGTGGGccccgc
OsSK11	P$TCP15_01	TCP15	3851	3861	1	1	0.999	GTGGGccccg
OsSK11	P$TCP20_01	TCP20	3851	3861	1	1	0.992	GTGGGccccg
OsSK11	P$TCP11_02	TCP11	3851	3861	1	1	0.998	GTGGGccccg
OsSK11	P$TCP7_01	TCP7	3851	3862	1	1	0.96	GTGGGccccgc
OsSK11	P$OJ1581_01	OJ1581	3851	3861	1	1	0.991	gtGGGCCccg
OsSK11	P$TCP2_01	TCP2	3851	3861	1	1	0.974	gtGGGCCccg
OsSK11	P$CMTA2_01	CMTA2	3855	3864	1	1	0.995	gccccCGCGT
OsSK11	P$CAMTA1_02	CAMTA1	3855	3867	1	1	0.875	gccccCGCGTcct
OsSK11	P$CMTA3_01	CMTA3	3858	3867	1	1	1	cCGCGTcct
OsSK11	P$ALFIN1_Q2	ALFIN1	3870	3885	1	1	0.874	gtggtgGTGGGtgct
OsSK11	P$CBNAC_01	CBNAC	3880	3886	1	1	0.979	gTGCTT
OsSK11	P$CBNAC_02	CBNAC	3880	3896	1	1	0.861	gTGCTTtgactgcacc
OsSK11	P$WRKY11_Q2	WRKY11	3884	3892	1	1	0.927	tTTGACtg
OsSK11	P$ATHB7_01	ATHB7	3914	3924	1	1	0.921	gaAATCAttt
OsSK11	P$HAT1_01	HAT1	3914	3924	1	1	0.982	gaAATCAttt
OsSK11	P$ATHB4_02	ATHB4	3915	3925	1	1	0.926	aaATCATttc
OsSK11	P$PHYPA64121_06	HYPA64121	3920	3933	1	1	0.879	attTCGGTttcgg
OsSK11	P$RAMOSA1_01	RAMOSA1	3930	3944	1	1	0.94	cggagaGAGAGaga
OsSK11	P$RAMOSA1_01	RAMOSA1	3932	3946	1	1	1	gagagaGAGAGaga
OsSK11	P$RAMOSA1_01	RAMOSA1	3934	3948	1	1	0.97	gagagaGAGAGagg
OsSK11	P$RAMOSA1_01	RAMOSA1	3936	3950	1	1	0.876	gagagaGAGAGgat
OsSK11	P$GT1_Q6_01	GT1	3949	3961	1	1	0.858	TTTTTttttcct
OsSK11	P$CBF3_02	CBF3	3983	3997	1	1	0.928	gcgtcCCGACaagc
OsSK11	P$CBF1_04	CBF1	3984	3996	1	1	0.923	cgtcCCGACaag
OsSK11	P$DREB1G_02	DREB1G	3985	3995	1	1	0.903	gtcCCGACaa
OsSK11	P$AT1G77200_03	AT1G77200	3985	3999	1	1	0.874	gtcCCGACaagcaa
OsSK11	P$ARF1_01	ARF1	3987	3995	1	1	0.931	cCCGACaa
OsSK11	P$ARF5_01	ARF5	3987	3995	1	1	0.99	cCCGACaa
OsSK11	P$DREB1B_01	DREB1B	3988	3993	1	1	1	CCGAC
Os05g0134000	P$PIL5_01	IL5	1	15	1	1	0.964	gttgatgcACGTGg
Os05g0134000	P$PIF3_01	IF3	2	20	1	1	0.892	ttgatgCACGTGgttaat
Os05g0134000	P$PIF3_02	IF3	3	20	1	1	0.91	tgatgCACGTGgttaat
Os05g0134000	P$ABF2_01	ABF2	3	16	1	1	0.943	tgatgCACGTggt
Os05g0134000	P$O2_Q4	O2	4	15	1	1	0.869	gatgCACGTgg
Os05g0134000	P$BZR1_02	BZR1	4	18	1	1	0.973	gatgCACGTggtta
Os05g0134000	P$HBI1_01	HBI1	4	16	1	1	0.994	gatgCACGTggt
Os05g0134000	P$ABZ1_01	ABZ1	4	18	1	1	0.944	gatgcACGTGgtta
Os05g0134000	P$GBP_Q6	GBP	5	17	1	1	0.906	atgCACGTggtt
Os05g0134000	P$PIF3_03	IF3	5	15	1	1	0.946	atgCACGTgg
Os05g0134000	P$ABI5_01	ABI5	5	15	1	1	0.931	atgCACGTgg
Os05g0134000	P$ABF4_01	ABF4	5	17	1	1	0.958	atgCACGTggtt
Os05g0134000	P$GBF1_Q2_01	GBF1	5	16	1	1	0.875	atgcACGTGgt
Os05g0134000	P$BZR1_03	BZR1	5	25	1	1	0.868	atgcACGTGgttaatggaga
Os05g0134000	P$TSAR2_01	TSAR2	6	16	1	1	0.869	tGCACGtggt
Os05g0134000	P$EMBP1_Q2	EMBP1	6	16	1	1	0.89	tgCACGTggt
Os05g0134000	P$CPRF_Q2	CPRF	6	16	1	1	0.937	tgCACGTggt
Os05g0134000	P$CPRF3_Q2	CPRF3	6	16	1	1	0.973	tgCACGTggt
Os05g0134000	P$CPRF2_Q2	CPRF2	6	16	1	1	0.985	tgCACGTggt
Os05g0134000	P$O2_02	O2	6	16	1	1	0.962	tgCACGTggt
Os05g0134000	P$TGA1B_Q2	TGA1B	6	16	1	1	0.894	tgCACGTggt
Os05g0134000	P$TGA1A_Q2	TGA1A	6	16	1	1	0.974	tgCACGTggt
Os05g0134000	P$CPRF1_01	CPRF1	6	16	1	1	0.941	tgCACGTggt
Os05g0134000	P$CPRF3_01	CPRF3	6	16	1	1	0.974	tgCACGTggt
Os05g0134000	P$CPRF2_01	CPRF2	6	16	1	1	0.987	tgCACGTggt
Os05g0134000	P$TGA1B_01	TGA1B	6	16	1	1	0.882	tgCACGTggt
Os05g0134000	P$BES1_01	BES1	6	17	1	1	0.971	tgCACGTggtt
Os05g0134000	P$PIF3_04	IF3	6	16	1	1	0.882	tgCACGTggt
Os05g0134000	P$BEE2_01	BEE2	6	16	1	1	0.999	tgCACGTggt
Os05g0134000	P$BIM2_01	BIM2	6	16	1	1	0.997	tgCACGTggt
Os05g0134000	P$BIM3_01	BIM3	6	16	1	1	0.995	tgCACGTggt
Os05g0134000	P$PHYPA143875_02	HYPA143875	6	16	1	1	0.998	tgCACGTggt
Os05g0134000	P$PHYPA72483_07	HYPA72483	6	16	1	1	0.998	tgCACGTggt
Os05g0134000	P$SPT_01	SPT	6	15	1	1	0.98	tgCACGTgg
Os05g0134000	P$GBF1F_Q2	GBF1F	6	17	1	1	0.861	tgCACGTggtt
Os05g0134000	P$TSAR1_01	TSAR1	6	16	1	1	0.892	tgCACGTggt

Os05g0134000	P$HBP1A_Q2	HBP1A	6	16	1	1	0.915	tgcACGTGgt
Os05g0134000	P$TAF1_Q2	TAF1	6	16	1	1	0.968	tgcACGTGgt
Os05g0134000	P$EMBP1_02	EMBP1	6	16	1	1	0.929	tgcACGTGgt
Os05g0134000	P$TAF1_01	TAF1	6	16	1	1	0.98	tgcACGTGgt
Os05g0134000	P$PIF1_01	IF1	6	16	1	1	0.986	tgcACGTGgt
Os05g0134000	P$BHLH78_01	BHLH78	7	15	1	1	0.875	GCACGtgg
Os05g0134000	P$RITA1_01	RITA1	7	14	1	1	0.974	gCACGTg
Os05g0134000	P$BHLH66_01	BHLH66	7	15	1	1	0.968	gCACGTgg
Os05g0134000	P$PIF5_01	IF5	7	15	1	1	0.93	gCACGTgg
Os05g0134000	P$MYC2_01	MYC2	7	15	1	1	0.972	gCACGTgg
Os05g0134000	P$MYC3_01	MYC3	7	15	1	1	0.997	gCACGTgg
Os05g0134000	P$BHLH34_01	BHLH34	7	15	1	1	1	gCACGTgg
Os05g0134000	P$PHYPA48267_08	HYPA48267	7	15	1	1	0.971	gCACGTgg
Os05g0134000	P$OJ1058_01	OJ1058	7	15	1	1	0.946	gCACGTgg
Os05g0134000	P$UNE10_01	UNE10	7	15	1	1	0.977	gCACGTgg
Os05g0134000	P$BHLH3_01	BHLH3	7	15	1	1	0.971	gCACGTgg
Os05g0134000	P$AIB_01	AIB	7	15	1	1	0.892	gCACGTgg
Os05g0134000	P$HY5_01	HY5	7	17	1	1	0.947	gcACGTGgtt
Os05g0134000	P$GBF1_01	GBF1	7	15	1	1	0.959	gcACGTGg
Os05g0134000	P$MYC4_01	MYC4	7	15	1	1	0.953	gcACGTGg
Os05g0134000	P$BIM1_02	BIM1	7	17	1	1	0.995	gcACGTGgtt
Os05g0134000	P$BHLH13_01	BHLH13	7	15	1	1	0.96	gcACGTGg
Os05g0134000	P$ABF4_02	ABF4	7	17	1	1	0.974	gcACGTGgtt
Os05g0134000	P$BZIP68_01	BZIP68	7	16	1	1	0.974	gcaCGTGGt
Os05g0134000	P$OCSBF1_01	OCSBF1	8	13	1	1	1	CACGT
Os05g0134000	P$PIF4_01	IF4	8	16	1	1	0.966	CACGTggt
Os05g0134000	P$CPRF1_Q2	CPRF1	8	18	1	1	0.953	cACGTGgtta
Os05g0134000	P$ABI5_Q2	ABI5	9	15	1	1	1	ACGTGg
Os05g0134000	P$EDT1_01	EDT1	12	22	1	1	0.901	tggTTAATgg
Os05g0134000	P$UIF1_01	UIF1	20	30	1	1	0.979	ggaGATTCca
Os05g0134000	P$WRKY40_03	WRKY40	69	79	1	1	0.994	ggAGTCAaat
Os05g0134000	P$WRKY18_02	WRKY18	69	79	1	1	0.978	ggaGTCAAat
Os05g0134000	P$WRKY21_02	WRKY21	69	79	1	1	0.969	ggaGTCAAat
Os05g0134000	P$WRKY48_02	WRKY48	69	79	1	1	0.995	ggaGTCAAat
Os05g0134000	P$WRKY57_01	WRKY57	69	79	1	1	0.981	ggaGTCAAat
Os05g0134000	P$WRKY60_01	WRKY60	69	80	1	1	0.903	ggaGTCAAatt
Os05g0134000	P$WRKY15_01	WRKY15	70	80	1	1	0.979	gaGTCAAatt
Os05g0134000	P$WRKY2_01	WRKY2	70	78	1	1	0.94	gaGTCAAa
Os05g0134000	P$WRKY25_02	WRKY25	70	78	1	1	0.914	gaGTCAAa
Os05g0134000	P$WRKY40_01	WRKY40	70	78	1	1	0.996	gaGTCAAa
Os05g0134000	P$WRKY43_02	WRKY43	70	80	1	1	0.988	gaGTCAAatt
Os05g0134000	P$WRKY62_01	WRKY62	70	78	1	1	0.866	gaGTCAAa
Os05g0134000	P$WRKY63_01	WRKY63	70	78	1	1	0.904	gaGTCAAa
Os05g0134000	P$WRKY75_01	WRKY75	70	78	1	1	0.976	gaGTCAAa
Os05g0134000	P$WRKY8_01	WRKY8	70	79	1	1	0.99	gaGTCAAat
Os05g0134000	P$WRKY23_01	WRKY23	71	79	1	1	0.88	aGTCAAat
Os05g0134000	P$WRKY30_01	WRKY30	71	81	1	1	0.915	aGTCAAattt
Os05g0134000	P$WRKY18_Q2	WRKY18	72	81	1	1	0.993	GTCAAattt
Os05g0134000	P$AGL1_01	AGL1	86	104	1	1	0.881	gagtCCAAAtttggtacg
Os05g0134000	P$HSFA2_01	HSFA2	90	96	1	1	0.922	CCAAAt
Os05g0134000	P$SPL11_01	SPL11	95	107	1	1	0.889	tttgGTACGatt
Os05g0134000	P$SPL5_01	SPL5	97	106	1	1	0.973	tgGTACGat
Os05g0134000	P$POPTR_01	OPTR	98	105	1	1	0.944	gGTACGa
Os05g0134000	P$SPL12_01	SPL12	98	106	1	1	0.982	gGTACGat
Os05g0134000	P$SPL4_01	SPL4	98	107	1	1	0.994	gGTACGatt
Os05g0134000	P$DOF1_01	DOF1	111	122	1	1	0.979	atgTAAAGttt
Os05g0134000	P$ATSPL8_01	ATSPL8	123	139	1	1	0.896	tttctTGTACtagagg
Os05g0134000	P$MYB24_01	MYB24	150	159	1	1	0.862	tgtTTAGGa
Os05g0134000	P$AZF3_01	AZF3	163	174	1	1	0.908	tAGTATgagtt
Os05g0134000	P$BZIP68_01	BZIP68	176	185	1	1	0.942	gttCGTGGc
Os05g0134000	P$MYB24_01	MYB24	183	192	1	1	0.907	gctTTAGGc
Os05g0134000	P$P_01		193	202	1	1	0.952	gcCTACCtc
Os05g0134000	P$MYB24_01	MYB24	269	278	1	1	0.92	gagTTAGGg
Os05g0134000	P$ATSPL8_01	ATSPL8	313	329	1	1	0.943	gttggTGTACtttgta
Os05g0134000	P$GT1_Q6	GT1	326	333	1	1	0.912	GTAAAca
Os05g0134000	P$REF6_01	REF6	329	340	1	1	0.868	aacaCAGAGag
Os05g0134000	P$BPC1_Q2	BPC1	338	344	1	1	0.99	AGAAAc
Os05g0134000	P$ATHB6_01	ATHB6	342	351	1	1	0.926	acAATAAag
Os05g0134000	P$DOF1_01	DOF1	343	354	1	1	0.975	caaTAAAGttg
Os05g0134000	P$HMG1_01	HMG1	350	359	1	1	0.975	GTTGTcatc
Os05g0134000	P$MYBAS1_01	MYBAS1	402	413	1	1	0.984	gtCTAACcggt
Os05g0134000	P$AT5G04240_01	AT5G04240	412	418	1	1	0.938	tGGCAC
Os05g0134000	P$CBF3_02	CBF3	414	428	1	1	0.954	gcacaCCGACggtc
Os05g0134000	P$CBF1_04	CBF1	415	427	1	1	0.938	cacaCCGACggt
Os05g0134000	P$RAV2_01	RAV2	416	425	1	1	0.858	acACCGAcg
Os05g0134000	P$AT1G77200_03	AT1G77200	416	430	1	1	0.894	acaCCGACggtcag
Os05g0134000	P$ARF1_01	ARF1	418	426	1	1	0.897	aCCGACgg
Os05g0134000	P$ARF5_01	ARF5	418	426	1	1	0.891	aCCGACgg

Os05g0134000	P$DREB1B_01	DREB1B	419	424	1	1	1	CCGAC
Os05g0134000	P$RRTF1_05	RRTF1	448	463	1	1	0.902	gtcagacCGGCGggg
Os05g0134000	P$AT1G28160_02	AT1G28160	452	467	1	1	0.889	gacCGGCGgggtaca
Os05g0134000	P$RAP26_06	RAP26	452	467	1	1	0.856	gacCGGCGgggtaca
Os05g0134000	P$AT1G68550_03	AT1G68550	452	461	1	1	0.955	gacCGGCGg
Os05g0134000	P$HSFA1E_01	HSFA1E	454	460	1	1	1	cCGGCG
Os05g0134000	P$E2L_Q2	E2L	455	462	1	1	0.928	cGGCGGg
Os05g0134000	P$HSF3_01	HSF3	457	463	1	1	0.945	gCGGGG
Os05g0134000	P$ARF8_01	ARF8	480	489	1	1	0.993	ggTGTCGgt
Os05g0134000	P$DREB1A_04	DREB1A	481	491	1	1	0.978	gtGTCGGtga
Os05g0134000	P$ERF039_01	ERF039	481	491	1	1	1	gtGTCGGtga
Os05g0134000	P$PHYPA182268_05	HYPA182268	481	491	1	1	0.999	gtGTCGGtga
Os05g0134000	P$PHYPA64121_06	HYPA64121	481	494	1	1	0.978	gtgTCGGTgacag
Os05g0134000	P$RAP21_02	RAP21	481	494	1	1	0.976	gtgtCGGTGacag
Os05g0134000	P$ERF043_01	ERF043	482	490	1	1	1	tGTCGGtg
Os05g0134000	P$PHYPA173530_04	HYPA173530	482	490	1	1	1	tGTCGGtg
Os05g0134000	P$PHYPA28324_10	HYPA28324	482	490	1	1	1	tGTCGGtg
Os05g0134000	P$KNOX3_01	KNOX3	484	496	1	1	0.989	tcggTGACAggc
Os05g0134000	P$ATH1_01	ATH1	488	496	1	1	0.986	TGACAggc
Os05g0134000	P$GATA9_01	GATA9	502	513	1	1	0.98	agcAGATCtcg
Os05g0134000	P$AGP1_01	AGP1	503	513	1	1	0.936	gcAGATCtcg
Os05g0134000	P$GATA10_01	GATA10	504	512	1	1	0.916	cAGATCtc
Os05g0134000	P$GATA11_01	GATA11	504	512	1	1	0.944	caGATCTc
Os05g0134000	P$GATA8_01	GATA8	504	513	1	1	0.992	caGATCTcg
Os05g0134000	P$ARR10_01	ARR10	505	512	1	1	0.913	AGATCtc
Os05g0134000	P$UIF1_01	UIF1	524	534	1	1	0.979	ggaGATTCaa
Os05g0134000	P$RAV1_01	RAV1	528	540	1	1	0.906	attCAACAccgg
Os05g0134000	P$AT4G12750_01	AT4G12750	564	574	1	1	0.873	cagACCGAtc
Os05g0134000	P$RAV2_01	RAV2	565	574	1	1	0.861	agACCGAtc
Os05g0134000	P$GATA8_01	GATA8	568	577	1	1	0.983	ccGATCTca
Os05g0134000	P$PBF_01	BF	604	615	1	1	0.947	gctAAAAGttt
Os05g0134000	P$DOF_Q2	DOF	604	615	1	1	0.931	gctAAAAGttt
Os05g0134000	P$CDF2_01	CDF2	605	615	1	1	0.968	ctAAAAGttt
Os05g0134000	P$CDF3_01	CDF3	606	615	1	1	0.984	tAAAAGttt
Os05g0134000	P$SOC1_01	SOC1	611	626	1	1	0.915	gttttcggtTTTTGg
Os05g0134000	P$PHYPA64121_06	HYPA64121	612	625	1	1	0.879	tttTCGGTtttg
Os05g0134000	P$SEP3_01	wrz-03	615	626	1	1	0.873	tcggtTTTTGg
Os05g0134000	P$SQUA_01	SQUA	616	626	1	1	0.891	cggTTTTTgg
Os05g0134000	P$AT4G36620_01	AT4G36620	630	638	1	1	1	atcAACCA
Os05g0134000	P$GAMYB_01	GAMYB	632	640	1	1	0.869	CAACCatt
Os05g0134000	P$PH4_01	H4	638	647	1	1	0.935	ttcACCCCc
Os05g0134000	P$ATMYB77_01	ATMYB77	677	690	1	1	0.853	atcctaCAGTTcc
Os05g0134000	P$ATHSFA1D_01	ATHSFA1D	679	685	1	1	0.985	cCTACA
Os05g0134000	P$DOF1_01	DOF1	693	704	1	1	0.984	ataTAAAGcat
Os05g0134000	P$DOF2_01	DOF2	693	704	1	1	0.998	atatAAAGCat
Os05g0134000	P$DOF3_01	DOF3	693	704	1	1	0.979	atatAAAGCat
Os05g0134000	P$ABI3_01	ABI3	710	719	1	1	0.959	aaGCATGca
Os05g0134000	P$FUS3_01	FUS3	711	720	1	1	0.955	aGCATGcaa
Os05g0134000	P$LEC2_01	LEC2	711	722	1	1	0.992	agCATGCaatt
Os05g0134000	P$FUS3_Q2	FUS3	712	723	1	1	0.851	gCATGCaatta
Os05g0134000	P$C1_Q2	C1	720	731	1	1	0.952	ttAACTAtgac
Os05g0134000	P$KNOX3_01	KNOX3	723	735	1	1	0.968	actaTGACAtct
Os05g0134000	P$ATH1_01	ATH1	727	735	1	1	0.939	TGACAtct
Os05g0134000	P$ARR2_01	ARR2	727	737	1	1	0.871	tgacATCTTt
Os05g0134000	P$SBF1_01	SBF1	735	749	1	1	0.872	tttctaTTAAAtta
Os05g0134000	P$HSFA4A_01	HSFA4A	737	743	1	1	0.914	tCTATT
Os05g0134000	P$ATHB1_01	ATHB1	740	754	1	1	0.952	attaaATTATtact
Os05g0134000	P$ATHB5_01	ATHB5	743	752	1	1	0.912	aaaTTATTa
Os05g0134000	P$SQUA_01	SQUA	752	762	1	1	0.892	cttTTTTTaa
Os05g0134000	P$HSFA4A_01	HSFA4A	787	793	1	1	0.964	cCTATT
Os05g0134000	P$AZF3_01	AZF3	805	816	1	1	0.961	tAGTATttatt
Os05g0134000	P$MYB24_01	MYB24	811	820	1	1	0.884	ttaTTAGGa
Os05g0134000	P$GATA15_01	GATA15	818	827	1	1	0.999	gaTGATCca
Os05g0134000	P$HSFA2_01	HSFA2	824	830	1	1	0.941	CCAAAc
Os05g0134000	P$C1_Q2	C1	825	836	1	1	0.979	caAACTAcaag
Os05g0134000	P$ATHSFA1D_01	ATHSFA1D	828	834	1	1	1	aCTACA
Os05g0134000	P$ARR18_01	ARR18	830	843	1	1	0.889	tacaAGATAataa
Os05g0134000	P$ATHB6_01	ATHB6	836	845	1	1	0.981	atAATAAta
Os05g0134000	P$ATHB5_04	ATHB5	836	847	1	1	0.905	atAATAAtaat
Os05g0134000	P$ATHB1_03	ATHB1	836	847	1	1	0.894	atAATAAtaat
Os05g0134000	P$ATHB16_01	ATHB16	837	845	1	1	0.915	tAATAAta
Os05g0134000	P$ATHB6_01	ATHB6	839	848	1	1	0.984	atAATAAtt
Os05g0134000	P$ATHB5_04	ATHB5	839	850	1	1	0.975	atAATAAttat
Os05g0134000	P$ATHB1_03	ATHB1	839	850	1	1	0.978	atAATAAttat
Os05g0134000	P$ATHB16_01	ATHB16	840	848	1	1	1	tAATAAtt
Os05g0134000	P$ATHB1_01	ATHB1	840	854	1	1	0.933	taataATTATttct
Os05g0134000	P$ATHB5_01	ATHB5	843	852	1	1	0.92	taaTTATTt
Os05g0134000	P$AT3G20750_01	AT3G20750	905	913	1	1	0.958	aTAAACct

Os05g0134000	P$SED_Q2	SED	906	916	1	1	0.911	taaaCCTTTt
Os05g0134000	P$PBF_Q2_01	BF	910	916	1	1	1	CCTTTt
Os05g0134000	P$ATHB6_01	ATHB6	933	942	1	1	0.908	aaAATAAat
Os05g0134000	P$ATHB6_01	ATHB6	937	946	1	1	0.909	taAATAAat
Os05g0134000	P$HSFA4A_01	HSFA4A	945	951	1	1	0.914	tCTATT
Os05g0134000	P$O2_Q4	O2	953	964	1	1	0.879	caagCATGTaa
Os05g0134000	P$ABI3_01	ABI3	954	963	1	1	0.855	aaGCATGta
Os05g0134000	P$SPF1_Q2	SPF1	961	971	1	1	0.921	taATAGTtaa
Os05g0134000	P$SBF1_01	SBF1	961	975	1	1	0.91	taatagTTAATgtt
Os05g0134000	P$EDT1_01	EDT1	964	974	1	1	0.947	tagTTAATgt
Os05g0134000	P$SBF1_01	SBF1	968	982	1	1	0.902	taatgtTTAATtaa
Os05g0134000	P$EDT1_01	EDT1	971	981	1	1	0.855	tgtTTAATta
Os05g0134000	P$SBF1_01	SBF1	972	986	1	1	0.866	gtttaaTTAATcat
Os05g0134000	P$EDT1_01	EDT1	975	985	1	1	0.884	taaTTAATca
Os05g0134000	P$ATHB7_01	ATHB7	978	988	1	1	0.924	ttAATCAtgt
Os05g0134000	P$HAT1_01	HAT1	978	988	1	1	0.985	ttAATCAtgt
Os05g0134000	P$PHYPA64121_06	HYPA64121	990	1003	1	1	0.867	atcTCGGTtgcgc
Os05g0134000	P$SBF1_01	SBF1	1001	1015	1	1	0.899	gcgctaTTAAAaag
Os05g0134000	P$HSFA4A_01	HSFA4A	1003	1009	1	1	0.91	gCTATT
Os05g0134000	P$PBF_01	BF	1007	1018	1	1	0.965	ttaAAAAGttc
Os05g0134000	P$DOF_Q2	DOF	1007	1018	1	1	0.975	ttaAAAAGttc
Os05g0134000	P$CDF2_01	CDF2	1008	1018	1	1	0.993	taAAAAGttc
Os05g0134000	P$CDF3_01	CDF3	1009	1018	1	1	0.99	aAAAAGttc
Os05g0134000	P$GAMYB_Q2	GAMYB	1015	1028	1	1	0.899	ttcacACAACttc
Os05g0134000	P$TGA1_01	TGA1	1041	1052	1	1	0.929	gccTGACGccc
Os05g0134000	P$TGA7_01	TGA7	1042	1052	1	1	0.881	ccTGACGccc
Os05g0134000	P$TGA5_01	TGA5	1043	1051	1	1	0.877	cTGACGcc
Os05g0134000	P$ATMYB77_01	ATMYB77	1051	1064	1	1	0.867	cagcttCAGTTag
Os05g0134000	P$GATA9_01	GATA9	1059	1070	1	1	0.891	gttAGATCatg
Os05g0134000	P$AGP1_01	AGP1	1060	1070	1	1	0.86	ttAGATCatg
Os05g0134000	P$GATA10_01	GATA10	1061	1069	1	1	0.868	tAGATCat
Os05g0134000	P$ARR10_01	ARR10	1062	1069	1	1	0.913	AGATCat
Os05g0134000	P$O2_Q4	O2	1062	1073	1	1	0.898	agatCATGTca
Os05g0134000	P$SBF1_01	SBF1	1069	1083	1	1	0.898	gtcaggTTAATtag
Os05g0134000	P$AZF3_01	AZF3	1080	1091	1	1	0.937	tAGTATacttt
Os05g0134000	P$SBF1_01	SBF1	1087	1101	1	1	0.85	ctttgaTTAATtga
Os05g0134000	P$EDT1_01	EDT1	1090	1100	1	1	0.854	tgaTTAATtg
Os05g0134000	P$EDT1_01	EDT1	1098	1108	1	1	0.854	tgaTTAATtg
Os05g0134000	P$MYB24_01	MYB24	1113	1122	1	1	0.967	tggTTAGGc
Os05g0134000	P$UIF1_01	UIF1	1133	1143	1	1	0.856	cagGATTCca
Os05g0134000	P$SBF1_01	SBF1	1154	1168	1	1	0.859	agatgaTTAAAaga
Os05g0134000	P$PBF_01	BF	1159	1170	1	1	0.982	attAAAAGagt
Os05g0134000	P$DOF_Q2	DOF	1159	1170	1	1	0.938	attAAAAGagt
Os05g0134000	P$CDF2_01	CDF2	1160	1170	1	1	0.951	ttAAAAGagt
Os05g0134000	P$CDF3_01	CDF3	1161	1170	1	1	0.978	tAAAAGagt
Os05g0134000	P$WRKY40_03	WRKY40	1165	1175	1	1	0.995	agAGTCAata
Os05g0134000	P$WRKY18_02	WRKY18	1165	1175	1	1	0.978	agaGTCAAta
Os05g0134000	P$WRKY21_02	WRKY21	1165	1175	1	1	0.962	agaGTCAAta
Os05g0134000	P$WRKY48_02	WRKY48	1165	1175	1	1	0.993	agaGTCAAta
Os05g0134000	P$WRKY57_01	WRKY57	1165	1175	1	1	0.978	agaGTCAAta
Os05g0134000	P$WRKY60_01	WRKY60	1165	1176	1	1	0.894	agaGTCAAtat
Os05g0134000	P$WRKY15_01	WRKY15	1166	1176	1	1	0.974	gaGTCAAtat
Os05g0134000	P$WRKY2_01	WRKY2	1166	1174	1	1	0.935	gaGTCAAt
Os05g0134000	P$WRKY25_02	WRKY25	1166	1174	1	1	0.91	gaGTCAAt
Os05g0134000	P$WRKY40_01	WRKY40	1166	1174	1	1	0.996	gaGTCAAt
Os05g0134000	P$WRKY43_02	WRKY43	1166	1176	1	1	0.987	gaGTCAAtat
Os05g0134000	P$WRKY63_01	WRKY63	1166	1174	1	1	0.904	gaGTCAAt
Os05g0134000	P$WRKY75_01	WRKY75	1166	1174	1	1	0.967	gaGTCAAt
Os05g0134000	P$WRKY8_01	WRKY8	1166	1175	1	1	0.988	gaGTCAAta
Os05g0134000	P$WRKY23_01	WRKY23	1167	1175	1	1	0.871	aGTCAAta
Os05g0134000	P$WRKY30_01	WRKY30	1167	1177	1	1	0.919	aGTCAAtata
Os05g0134000	P$WRKY18_Q2	WRKY18	1168	1177	1	1	0.968	GTCAAtata
Os05g0134000	P$WRKY_Q2	WRKY	1175	1186	1	1	0.857	tattttAGTCA
Os05g0134000	P$WRKY40_03	WRKY40	1179	1189	1	1	0.995	ttAGTCAatt
Os05g0134000	P$WRKY18_02	WRKY18	1179	1189	1	1	0.978	ttaGTCAAtt
Os05g0134000	P$WRKY21_02	WRKY21	1179	1189	1	1	0.963	ttaGTCAAtt
Os05g0134000	P$WRKY48_02	WRKY48	1179	1189	1	1	0.993	ttaGTCAAtt
Os05g0134000	P$WRKY57_01	WRKY57	1179	1189	1	1	0.977	ttaGTCAAtt
Os05g0134000	P$WRKY60_01	WRKY60	1179	1190	1	1	0.9	ttaGTCAAtta
Os05g0134000	P$WRKY15_01	WRKY15	1180	1190	1	1	0.974	taGTCAAtta
Os05g0134000	P$WRKY2_01	WRKY2	1180	1188	1	1	0.935	taGTCAAt
Os05g0134000	P$WRKY25_02	WRKY25	1180	1188	1	1	0.917	taGTCAAt
Os05g0134000	P$WRKY40_01	WRKY40	1180	1188	1	1	0.996	taGTCAAt
Os05g0134000	P$WRKY43_02	WRKY43	1180	1190	1	1	0.986	taGTCAAtta
Os05g0134000	P$WRKY62_01	WRKY62	1180	1188	1	1	0.897	taGTCAAt
Os05g0134000	P$WRKY63_01	WRKY63	1180	1188	1	1	0.905	taGTCAAt
Os05g0134000	P$WRKY75_01	WRKY75	1180	1188	1	1	0.968	taGTCAAt
Os05g0134000	P$WRKY8_01	WRKY8	1180	1189	1	1	0.988	taGTCAAtt

Os05g0134000	P$WRKY23_01	WRKY23	1181	1189	1	1	0.862	aGTCAAtt
Os05g0134000	P$WRKY30_01	WRKY30	1181	1191	1	1	0.92	aGTCAAttac
Os05g0134000	P$WRKY18_Q2	WRKY18	1182	1191	1	1	0.936	GTCAAttac
Os05g0134000	P$KNOX3_01	KNOX3	1187	1199	1	1	0.986	ttacTGACAgat
Os05g0134000	P$SIZF2_01	SIZF2	1188	1198	1	1	0.883	tacTGACAga
Os05g0134000	P$ATH1_01	ATH1	1191	1199	1	1	0.99	TGACAgat
Os05g0134000	P$GATA9_01	GATA9	1192	1203	1	1	0.889	gacAGATCgaa
Os05g0134000	P$AGP1_01	AGP1	1193	1203	1	1	0.913	acAGATCgaa
Os05g0134000	P$ARR10_01	ARR10	1195	1202	1	1	0.869	AGATCga
Os05g0134000	P$O2_Q2	O2	1201	1214	1	1	0.876	aagacGACGTacg
Os05g0134000	P$STF1_01	STF1	1203	1215	1	1	0.86	gacGACGTacgg
Os05g0134000	P$ATSPL3_01	ATSPL3	1203	1219	1	1	0.959	gacgaCGTACggttta
Os05g0134000	P$SPL15_01	SPL15	1204	1218	1	1	0.863	acgacGTACGgttt
Os05g0134000	P$SPL11_01	SPL11	1205	1217	1	1	0.986	cgacGTACGgtt
Os05g0134000	P$SPL5_02	SPL5	1205	1217	1	1	0.881	cgacGTACGgtt
Os05g0134000	P$BHLH28_01	BHLH28	1205	1217	1	1	0.944	cgacGTACGgtt
Os05g0134000	P$SPL1_01	SPL1	1206	1216	1	1	0.892	gaCGTACggt
Os05g0134000	P$SPL14_03	SPL14	1206	1217	1	1	0.853	gaCGTACggtt
Os05g0134000	P$SPL5_01	SPL5	1207	1216	1	1	0.997	acGTACGgt
Os05g0134000	P$POPTR_01	OPTR	1208	1215	1	1	1	cGTACGg
Os05g0134000	P$SPL12_01	SPL12	1208	1216	1	1	0.999	cGTACGgt
Os05g0134000	P$SPL4_01	SPL4	1208	1217	1	1	1	cGTACGgtt
Os05g0134000	P$AT1G19490_01	AT1G19490	1213	1222	1	1	0.909	GGTTTactc
Os05g0134000	P$AMS_01	AMS	1227	1237	1	1	0.861	taCAGGTgta
Os05g0134000	P$TEIL_01	TEIL	1239	1247	1	1	0.931	ATGTAtgt
Os05g0134000	P$O2_Q2	O2	1254	1267	1	1	0.881	ggcctGACGTata
Os05g0134000	P$TGA1_01	TGA1	1255	1266	1	1	0.98	gccTGACGtat
Os05g0134000	P$STF1_02	STF1	1256	1268	1	1	0.919	ccTGACGtatat
Os05g0134000	P$TGA3_Q2	TGA3	1256	1265	1	1	0.956	ccTGACGta
Os05g0134000	P$TGA6_01	TGA6	1256	1266	1	1	0.959	ccTGACGtat
Os05g0134000	P$TGA7_01	TGA7	1256	1266	1	1	0.936	ccTGACGtat
Os05g0134000	P$BZIP14_01	BZIP14	1256	1266	1	1	0.983	ccTGACGtat
Os05g0134000	P$STF1_01	STF1	1256	1268	1	1	0.943	cctGACGTatat
Os05g0134000	P$TGA5_01	TGA5	1257	1265	1	1	0.994	cTGACGta
Os05g0134000	P$NAC043_01	NAC043	1257	1267	1	1	0.89	ctgACGTAta
Os05g0134000	P$ATSPL8_01	ATSPL8	1270	1286	1	1	0.938	ttcgtTGTACaaaaaa
Os05g0134000	P$KNOX3_01	KNOX3	1313	1325	1	1	0.975	ggagTGACAaac
Os05g0134000	P$ATH1_01	ATH1	1317	1325	1	1	0.909	TGACAaac
Os05g0134000	P$GAMYB_Q2	GAMYB	1318	1331	1	1	0.932	gacaaACAACacg
Os05g0134000	P$RAV1_01	RAV1	1321	1333	1	1	0.911	aaaCAACAcggc
Os05g0134000	P$NAC92_01	NAC92	1324	1336	1	1	0.986	caACACGgcacg
Os05g0134000	P$AT5G04240_01	AT5G04240	1329	1335	1	1	0.976	cGGCAC
Os05g0134000	P$NAC080_01	NAC080	1331	1339	1	1	0.97	gCACGCaa
Os05g0134000	P$NAC083_01	NAC083	1331	1341	1	1	0.964	gcACGCAagg
Os05g0134000	P$SBF1_01	SBF1	1356	1370	1	1	0.858	agttcaTTAATtat
Os05g0134000	P$EDT1_01	EDT1	1359	1369	1	1	0.876	tcaTTAATta
Os05g0134000	P$ARR1_01	ARR1	1372	1382	1	1	0.956	ataGAATCct
Os05g0134000	P$MYBAS1_01	MYBAS1	1386	1397	1	1	0.983	ttCCAACtcct
Os05g0134000	P$SQUA_01	SQUA	1433	1443	1	1	0.867	caaTTTTTtt
Os05g0134000	P$GT1_Q6_01	GT1	1436	1448	1	1	0.93	TTTTTtttaaaa
Os05g0134000	P$SBF1_01	SBF1	1436	1450	1	1	0.929	ttttttTTAAAaaa
Os05g0134000	P$GT1_Q6_01	GT1	1437	1449	1	1	0.86	TTTTTttaaaaa
Os05g0134000	P$ATHB6_01	ATHB6	1448	1457	1	1	0.975	aaAATAAta
Os05g0134000	P$ATHB5_04	ATHB5	1448	1459	1	1	0.886	aaAATAAtatg
Os05g0134000	P$ATHB1_03	ATHB1	1448	1459	1	1	0.885	aaAATAAtatg
Os05g0134000	P$ATHB16_01	ATHB16	1449	1457	1	1	0.867	aAATAAta
Os05g0134000	P$MYB118_01	MYB118	1462	1479	1	1	0.855	ctactatatGTTACata
Os05g0134000	P$PEND_02	END	1482	1492	1	1	0.895	agTTCTTgtc
Os05g0134000	P$SPF1_Q2	SPF1	1504	1514	1	1	0.869	ttATAGTtgt
Os05g0134000	P$HMG1_01	HMG1	1509	1518	1	1	0.89	GTTGTgtgc
Os05g0134000	P$PDF2_01	DF2	1518	1529	1	1	0.9	attcTAAATgc
Os05g0134000	P$AT1G14580_01	AT1G14580	1531	1542	1	1	0.894	agatgGGGATg
Os05g0134000	P$AT5G66730_01	AT5G66730	1531	1542	1	1	0.878	agatgGGGATg
Os05g0134000	P$ATHB7_01	ATHB7	1541	1551	1	1	0.92	gtAATCAtct
Os05g0134000	P$HAT1_01	HAT1	1541	1551	1	1	0.982	gtAATCAtct
Os05g0134000	P$BPC1_Q2	BPC1	1560	1566	1	1	0.997	AGAAAa
Os05g0134000	P$KNOX3_01	KNOX3	1573	1585	1	1	0.986	gatgTGACAact
Os05g0134000	P$GAMYB_Q2	GAMYB	1574	1587	1	1	0.946	atgtgACAACtga
Os05g0134000	P$ATH1_01	ATH1	1577	1585	1	1	0.926	TGACAact
Os05g0134000	P$C1_Q2	C1	1599	1610	1	1	0.93	taAACTAgaat
Os05g0134000	P$RAV1_01	RAV1	1614	1626	1	1	0.953	ctgCAACAggga
Os05g0134000	P$MYBAS1_01	MYBAS1	1631	1642	1	1	0.958	gtCTAACttca
Os05g0134000	P$ARR2_01	ARR2	1639	1649	1	1	0.925	tcaaATCTTg
Os05g0134000	P$HMG1_01	HMG1	1655	1664	1	1	0.898	GTTGTcata
Os05g0134000	P$ARR2_01	ARR2	1729	1739	1	1	0.886	tgatATCTTt
Os05g0134000	P$SBF1_01	SBF1	1743	1757	1	1	0.87	attgaaTTAAAatt
Os05g0134000	P$MYB24_01	MYB24	1752	1761	1	1	0.883	aaaTTAGGg
Os05g0134000	P$WRKY18_02	WRKY18	1757	1767	1	1	0.996	aggGTCAAgt

Os05g0134000	P$WRKY21_02	WRKY21	1757	1767	1	1	0.959	aggGTCAAgt
Os05g0134000	P$WRKY48_02	WRKY48	1757	1767	1	1	0.996	aggGTCAAgt
Os05g0134000	P$WRKY57_01	WRKY57	1757	1767	1	1	0.966	aggGTCAAgt
Os05g0134000	P$WRKY60_01	WRKY60	1757	1768	1	1	0.962	aggGTCAAgtt
Os05g0134000	P$WRKY15_01	WRKY15	1758	1768	1	1	0.977	ggGTCAAgtt
Os05g0134000	P$WRKY2_01	WRKY2	1758	1766	1	1	0.982	ggGTCAAg
Os05g0134000	P$WRKY25_02	WRKY25	1758	1766	1	1	0.961	ggGTCAAg
Os05g0134000	P$WRKY40_01	WRKY40	1758	1766	1	1	1	ggGTCAAg
Os05g0134000	P$WRKY43_02	WRKY43	1758	1768	1	1	0.969	ggGTCAAgtt
Os05g0134000	P$WRKY62_01	WRKY62	1758	1766	1	1	0.875	ggGTCAAg
Os05g0134000	P$WRKY63_01	WRKY63	1758	1766	1	1	0.992	ggGTCAAg
Os05g0134000	P$WRKY75_01	WRKY75	1758	1766	1	1	0.958	ggGTCAAg
Os05g0134000	P$WRKY8_01	WRKY8	1758	1767	1	1	0.987	ggGTCAAgt
Os05g0134000	P$WRKY30_01	WRKY30	1759	1769	1	1	0.915	gGTCAAgtta
Os05g0134000	P$WRKY18_Q2	WRKY18	1760	1769	1	1	1	GTCAAgtta
Os05g0134000	P$GAMYB_Q2	GAMYB	1765	1778	1	1	0.887	gttagACAACttt
Os05g0134000	P$DOF1_01	DOF1	1781	1792	1	1	0.97	ttgTAAAGatg
Os05g0134000	P$GT1_Q6	GT1	1783	1790	1	1	0.912	GTAAAga
Os05g0134000	P$SPF1_Q2	SPF1	1802	1812	1	1	0.856	gtATAGTgat
Os05g0134000	P$GATA15_01	GATA15	1806	1815	1	1	0.999	agTGATCta
Os05g0134000	P$GATA8_01	GATA8	1807	1816	1	1	0.986	gtGATCTat
Os05g0134000	P$SPF1_Q2	SPF1	1826	1836	1	1	0.856	gtATAGTgat
Os05g0134000	P$GATA15_01	GATA15	1830	1839	1	1	0.999	agTGATCta
Os05g0134000	P$GATA8_01	GATA8	1831	1840	1	1	0.986	gtGATCTat
Os05g0134000	P$ATHSFA1D_01	ATHSFA1D	1843	1849	1	1	0.94	gCTACA
Os05g0134000	P$ARR2_01	ARR2	1844	1854	1	1	0.87	ctacATCTTc
Os05g0134000	P$GATA15_01	GATA15	1867	1876	1	1	1	caTGATCgg
Os05g0134000	P$MYB3_01	MYB3	1885	1896	1	1	0.862	tcaTAGGTagg
Os05g0134000	P$P_01		1898	1907	1	1	0.878	ggCTACCtt
Os05g0134000	P$SED_Q2	SED	1899	1909	1	1	0.956	gctaCCTTTa
Os05g0134000	P$PBF_Q2_01	BF	1903	1909	1	1	0.998	CCTTTa
Os05g0134000	P$ARR18_01	ARR18	1907	1920	1	1	0.895	tacaAGATAgata
Os05g0134000	P$ARR18_01	ARR18	1911	1924	1	1	0.95	agatAGATAcata
Os05g0134000	P$ATHB6_01	ATHB6	1942	1951	1	1	0.91	atAATAAaa
Os05g0134000	P$PBF_Q2	BF	1957	1963	1	1	0.965	gAAAGG
Os05g0134000	P$HAHB4_01	HAHB4	1973	1982	1	1	0.94	tAATGAtat
Os05g0134000	P$PBF_01	BF	1989	2000	1	1	0.96	ctcAAAAGatg
Os05g0134000	P$DOF_Q2	DOF	1989	2000	1	1	0.923	ctcAAAAGatg
Os05g0134000	P$CDF2_01	CDF2	1990	2000	1	1	0.942	tcAAAAGatg
Os05g0134000	P$CDF3_01	CDF3	1991	2000	1	1	0.967	cAAAAGatg
Os05g0134000	P$ABI3_01	ABI3	2016	2025	1	1	0.969	taGCATGca
Os05g0134000	P$FUS3_01	FUS3	2017	2026	1	1	0.955	aGCATGcat
Os05g0134000	P$LEC2_01	LEC2	2017	2028	1	1	0.987	agCATGCatac
Os05g0134000	P$FUS3_Q2	FUS3	2018	2029	1	1	0.914	gCATGCatacc
Os05g0134000	P$ATHB7_01	ATHB7	2027	2037	1	1	0.932	ccAATCActg
Os05g0134000	P$HAT1_01	HAT1	2027	2037	1	1	0.882	ccAATCActg
Os05g0134000	P$SED_Q2	SED	2040	2050	1	1	0.952	cctcCCTTTa
Os05g0134000	P$PBF_Q2_01	BF	2044	2050	1	1	0.998	CCTTTa
Os05g0134000	P$GT1_Q6_01	GT1	2055	2067	1	1	0.853	TTTTTtttttca
Os05g0134000	P$GT1_Q6_01	GT1	2056	2068	1	1	0.861	TTTTTttttcaa
Os05g0134000	P$GT1_Q6_01	GT1	2057	2069	1	1	0.954	TTTTTtttcaaa
Os05g0134000	P$MADSA_Q2	MADSA	2060	2076	1	1	0.865	tttttcAAAATgtaaa
Os05g0134000	P$TEIL_01	TEIL	2069	2077	1	1	0.879	ATGTAaat
Os05g0134000	P$AGL12_01	AGL12	2084	2096	1	1	0.899	gtgAAATTtgat
Os05g0134000	P$ATHB7_01	ATHB7	2123	2133	1	1	0.855	atAATCAcag
Os05g0134000	P$HAT1_01	HAT1	2123	2133	1	1	0.862	atAATCAcag
Os05g0134000	P$TEIL_01	TEIL	2175	2183	1	1	0.922	ATGTAtat
Os05g0134000	P$ARR18_01	ARR18	2182	2195	1	1	0.942	tcgaAGATActtg
Os05g0134000	P$ATMYB77_01	ATMYB77	2190	2203	1	1	0.872	acttgtCAGTTta
Os05g0134000	P$SBF1_01	SBF1	2194	2208	1	1	0.885	gtcagtTTAAAatt
Os05g0134000	P$C1_Q2	C1	2212	2223	1	1	0.939	taAACTAtttt
Os05g0134000	P$HSFA4A_01	HSFA4A	2215	2221	1	1	1	aCTATT
Os05g0134000	P$SQUA_01	SQUA	2216	2226	1	1	0.9	ctaTTTTTta
Os05g0134000	P$SBF1_01	SBF1	2217	2231	1	1	0.874	tattttTTAAAaca
Os05g0134000	P$ATMYB15_Q2	ATMYB15	2231	2237	1	1	0.865	TAACAg
Os05g0134000	P$ABZ1_01	ABZ1	2242	2256	1	1	0.85	cacaaACGTGctcg
Os05g0134000	P$BZR1_03	BZR1	2243	2263	1	1	0.851	acaaACGTGctcggtagatt
Os05g0134000	P$TAF1_Q2	TAF1	2244	2254	1	1	0.908	caaACGTGct
Os05g0134000	P$EMBP1_02	EMBP1	2244	2254	1	1	0.894	caaACGTGct
Os05g0134000	P$TAF1_01	TAF1	2244	2254	1	1	0.94	caaACGTGct
Os05g0134000	P$GBF1_01	GBF1	2245	2253	1	1	0.911	aaACGTGc
Os05g0134000	P$MYC4_01	MYC4	2245	2253	1	1	0.863	aaACGTGc
Os05g0134000	P$BIM1_02	BIM1	2245	2255	1	1	0.949	aaACGTGctc
Os05g0134000	P$BHLH13_01	BHLH13	2245	2253	1	1	0.856	aaACGTGc
Os05g0134000	P$ABF4_02	ABF4	2245	2255	1	1	0.948	aaACGTGctc
Os05g0134000	P$ABI5_Q2	ABI5	2247	2253	1	1	0.936	ACGTGc
Os05g0134000	P$BZR1_01	BZR1	2248	2254	1	1	0.902	CGTGCt
Os05g0134000	P$GT1_Q6	GT1	2268	2275	1	1	0.971	GTAAAaa

Os05g0134000	P$DOF43_01	DOF43	2270	2281	1	1	0.905	aaaaatACTTT
Os05g0134000	P$MYB1L_01	MYB1L	2286	2296	1	1	0.992	taCCCTAcat
Os05g0134000	P$TRB2_01	TRB2	2286	2294	1	1	0.959	taCCCTAc
Os05g0134000	P$ATHSFA1D_01	ATHSFA1D	2289	2295	1	1	0.985	cCTACA
Os05g0134000	P$ABI3_01	ABI3	2302	2311	1	1	0.965	gtGCATGct
Os05g0134000	P$FUS3_01	FUS3	2303	2312	1	1	0.955	tGCATGctt
Os05g0134000	P$LEC2_01	LEC2	2303	2314	1	1	0.945	tgCATGCtttt
Os05g0134000	P$CBNAC_01	CBNAC	2306	2312	1	1	0.968	aTGCTT
Os05g0134000	P$CBNAC_02	CBNAC	2306	2322	1	1	0.871	aTGCTTtgtgctgat
Os05g0134000	P$GT1_Q6_01	GT1	2311	2323	1	1	0.851	TTTGTgctgatt
Os05g0134000	P$SBF1_01	SBF1	2315	2329	1	1	0.867	tgctgaTTAAAagc
Os05g0134000	P$PBF_01	BF	2320	2331	1	1	0.982	attAAAAGcaa
Os05g0134000	P$DOF_Q2	DOF	2320	2331	1	1	0.95	attAAAAGcaa
Os05g0134000	P$DOF2_01	DOF2	2320	2331	1	1	0.994	attaAAAGCaa
Os05g0134000	P$DOF3_01	DOF3	2320	2331	1	1	0.982	attaAAAGCaa
Os05g0134000	P$CDF2_01	CDF2	2321	2331	1	1	0.961	ttAAAAGcaa
Os05g0134000	P$CDF3_01	CDF3	2322	2331	1	1	0.978	tAAAAGcaa
Os05g0134000	P$RAV1_01	RAV1	2325	2337	1	1	0.957	aagCAACAatca
Os05g0134000	P$ATHB7_01	ATHB7	2330	2340	1	1	0.95	acAATCAaaa
Os05g0134000	P$HAT1_01	HAT1	2330	2340	1	1	0.881	acAATCAaaa
Os05g0134000	P$ATHB7_01	ATHB7	2345	2355	1	1	0.924	ttAATCAtag
Os05g0134000	P$HAT1_01	HAT1	2345	2355	1	1	0.98	ttAATCAtag
Os05g0134000	P$ATHB4_02	ATHB4	2346	2356	1	1	0.884	taATCATagt
Os05g0134000	P$SPF1_Q2	SPF1	2349	2359	1	1	0.919	tcATAGTcaa
Os05g0134000	P$WRKY40_03	WRKY40	2351	2361	1	1	0.996	atAGTCAata
Os05g0134000	P$WRKY18_02	WRKY18	2351	2361	1	1	0.979	ataGTCAAta
Os05g0134000	P$WRKY21_02	WRKY21	2351	2361	1	1	0.962	ataGTCAAta
Os05g0134000	P$WRKY48_02	WRKY48	2351	2361	1	1	0.993	ataGTCAAta
Os05g0134000	P$WRKY57_01	WRKY57	2351	2361	1	1	0.979	ataGTCAAta
Os05g0134000	P$WRKY60_01	WRKY60	2351	2362	1	1	0.9	ataGTCAAtat
Os05g0134000	P$WRKY15_01	WRKY15	2352	2362	1	1	0.974	taGTCAAtat
Os05g0134000	P$WRKY2_01	WRKY2	2352	2360	1	1	0.935	taGTCAAt
Os05g0134000	P$WRKY25_02	WRKY25	2352	2360	1	1	0.917	taGTCAAt
Os05g0134000	P$WRKY40_01	WRKY40	2352	2360	1	1	0.996	taGTCAAt
Os05g0134000	P$WRKY43_02	WRKY43	2352	2362	1	1	0.988	taGTCAAtat
Os05g0134000	P$WRKY62_01	WRKY62	2352	2360	1	1	0.897	taGTCAAt
Os05g0134000	P$WRKY63_01	WRKY63	2352	2360	1	1	0.905	taGTCAAt
Os05g0134000	P$WRKY75_01	WRKY75	2352	2360	1	1	0.968	taGTCAAt
Os05g0134000	P$WRKY8_01	WRKY8	2352	2361	1	1	0.988	taGTCAAta
Os05g0134000	P$WRKY23_01	WRKY23	2353	2361	1	1	0.871	aGTCAAta
Os05g0134000	P$WRKY30_01	WRKY30	2353	2363	1	1	0.919	aGTCAAtatg
Os05g0134000	P$WRKY18_Q2	WRKY18	2354	2363	1	1	0.964	GTCAAtatg
Os05g0134000	P$MYBAS1_01	MYBAS1	2361	2372	1	1	0.95	tgCTAACtggg
Os05g0134000	P$HMG1_01	HMG1	2387	2396	1	1	0.992	GTTGTtttc
Os05g0134000	P$GATA15_01	GATA15	2396	2405	1	1	1	ttTGATCaa
Os05g0134000	P$AT5G54070_01	AT5G54070	2401	2407	1	1	0.91	tCAACG
Os05g0134000	P$AT4G36620_01	AT4G36620	2404	2412	1	1	0.89	acgAACCA
Os05g0134000	P$AT3G60580_01	AT3G60580	2409	2416	1	1	0.873	ccATCCC
Os05g0134000	P$SPF1_Q2	SPF1	2423	2433	1	1	0.881	tcATAGTctc
Os05g0134000	P$GATA15_01	GATA15	2433	2442	1	1	0.999	tgTGATCcc
Os05g0134000	P$AT3G60580_01	AT3G60580	2435	2442	1	1	0.852	tgATCCC
Os05g0134000	P$CBF3_02	CBF3	2435	2449	1	1	0.917	tgatcCCGACcaaa
Os05g0134000	P$CBF1_04	CBF1	2436	2448	1	1	0.897	gatcCCGACcaa
Os05g0134000	P$ARF5_01	ARF5	2439	2447	1	1	0.921	cCCGACca
Os05g0134000	P$DREB1B_01	DREB1B	2440	2445	1	1	1	CCGAC
Os05g0134000	P$HSFA2_01	HSFA2	2444	2450	1	1	0.922	CCAAAt
Os05g0134000	P$PEND_01	END	2457	2465	1	1	0.972	tAAGAAat
Os05g0134000	P$BPC1_Q2	BPC1	2459	2465	1	1	0.99	AGAAAt
Os05g0134000	P$SPF1_Q2	SPF1	2514	2524	1	1	0.906	caATAGTgat
Os05g0134000	P$GATA15_01	GATA15	2518	2527	1	1	0.999	agTGATCta
Os05g0134000	P$GATA8_01	GATA8	2519	2528	1	1	0.987	gtGATCTaa
Os05g0134000	P$MYB24_01	MYB24	2525	2534	1	1	0.932	taaTTAGGc
Os05g0134000	P$ERF1_Q2	ERF1	2531	2539	1	1	0.887	GGCGGatg
Os05g0134000	P$EDT1_01	EDT1	2547	2557	1	1	0.863	ataTTAATta
Os05g0134000	P$MYB24_01	MYB24	2551	2560	1	1	0.932	taaTTAGGc
Os05g0134000	P$GT1_Q6_01	GT1	2570	2582	1	1	0.873	TTTGTttttgtt
Os05g0134000	P$ALFIN1_Q2	ALFIN1	2597	2612	1	1	0.851	ggcagaGTGGGttcc
Os05g0134000	P$TCP7_01	TCP7	2603	2614	1	1	0.918	GTGGGttccat
Os05g0134000	P$LEC2_01	LEC2	2609	2620	1	1	0.938	tcCATGCtccg
Os05g0134000	P$CBF3_02	CBF3	2612	2626	1	1	0.918	atgctCCGACcatg
Os05g0134000	P$CBF1_04	CBF1	2613	2625	1	1	0.902	tgctCCGACcat
Os05g0134000	P$ARF5_01	ARF5	2616	2624	1	1	0.918	tCCGACca
Os05g0134000	P$DREB1B_01	DREB1B	2617	2622	1	1	1	CCGAC
Os05g0134000	P$KNOX3_01	KNOX3	2620	2632	1	1	0.972	accaTGACAgtc
Os05g0134000	P$ATH1_01	ATH1	2624	2632	1	1	0.974	TGACAgtc
Os05g0134000	P$ATHB7_01	ATHB7	2644	2654	1	1	1	ccAATCAttt
Os05g0134000	P$HAT1_01	HAT1	2644	2654	1	1	0.999	ccAATCAttt
Os05g0134000	P$ATHB4_02	ATHB4	2645	2655	1	1	0.953	caATCATttg

Os05g0134000	P$CBNAC_01	CBNAC	2652	2658	1	1	1	tTGCTT
Os05g0134000	P$CBNAC_02	CBNAC	2652	2668	1	1	0.862	tTGCTTtcattttgtt
Os05g0134000	P$CBNAC_01	CBNAC	2673	2679	1	1	1	tTGCTT
Os05g0134000	P$RAV1_01	RAV1	2698	2710	1	1	0.934	tctCAACAgttt
Os05g0134000	P$AT3G18650_01	AT3G18650	2714	2725	1	1	0.881	gatttTTGTAt
Os05g0134000	P$AT5G26170_01	AT5G26170	2725	2734	1	1	0.867	gcTCAACtt
Os05g0134000	P$LEC2_01	LEC2	2739	2750	1	1	0.986	atCATGCatac
Os05g0134000	P$FUS3_Q2	FUS3	2740	2751	1	1	0.922	tCATGCataca
Os05g0134000	P$NAC92_01	NAC92	2746	2758	1	1	0.97	atACACGcaatt
Os05g0134000	P$NAC080_01	NAC080	2748	2756	1	1	1	aCACGCaa
Os05g0134000	P$NAC083_01	NAC083	2748	2758	1	1	0.972	acACGCAatt
Os05g0134000	P$SEP3_01	wrz-03	2752	2763	1	1	0.863	gcaatTTTTGt
Os05g0134000	P$SQUA_01	SQUA	2753	2763	1	1	0.933	caaTTTTTgt
Os05g0134000	P$HSFA2_01	HSFA2	2765	2771	1	1	0.941	CCAAAc
Os05g0134000	P$AT4G36620_01	AT4G36620	2765	2773	1	1	0.878	ccaAACCA
Os05g0134000	P$LIM1_01	LIM1	2770	2782	1	1	0.931	CCACCagatatg
Os05g0134000	P$ARR18_01	ARR18	2771	2784	1	1	0.956	caccAGATAtgta
Os05g0134000	P$ATHB7_01	ATHB7	2836	2846	1	1	0.952	ccAATCAaat
Os05g0134000	P$HAT1_01	HAT1	2836	2846	1	1	0.883	ccAATCAaat
Os05g0134000	P$AT5G04240_01	AT5G04240	2857	2863	1	1	0.938	tGGCAC
Os05g0134000	P$AMS_01	AMS	2879	2889	1	1	0.856	gaCATGTggt
Os05g0134000	P$GT1_01	GT1	2887	2895	1	1	0.977	gtTAACCt
Os05g0134000	P$SBF1_01	SBF1	2899	2913	1	1	0.887	tattttTTAATtaa
Os05g0134000	P$GT1_Q6_01	GT1	2902	2914	1	1	0.943	TTTTTaattaag
Os05g0134000	P$EDT1_01	EDT1	2902	2912	1	1	0.857	tttTTAATta
Os05g0134000	P$ARR18_01	ARR18	2908	2921	1	1	0.904	attaAGATAtcta
Os05g0134000	P$MRP1_Q2	MRP1	2915	2927	1	1	0.942	taTCTATttgca
Os05g0134000	P$HSFA4A_01	HSFA4A	2917	2923	1	1	0.914	tCTATT
Os05g0134000	P$KNOX3_01	KNOX3	2932	2944	1	1	0.968	aataTGACAaat
Os05g0134000	P$ATH1_01	ATH1	2936	2944	1	1	0.922	TGACAaat
Os05g0134000	P$GATA15_01	GATA15	2941	2950	1	1	1	aaTGATCat
Os05g0134000	P$ATHB1_01	ATHB1	2945	2959	1	1	0.895	atcatATTATtgca
Os05g0134000	P$ATHB5_01	ATHB5	2948	2957	1	1	0.851	ataTTATTg
Os05g0134000	P$WRKY48_01	WRKY48	2966	2975	1	1	0.865	atatAACAA
Os05g0134000	P$ATMYB15_Q2	ATMYB15	2969	2975	1	1	1	TAACAa
Os05g0134000	P$ATHB6_01	ATHB6	2991	3000	1	1	0.905	gaAATAAaa
Os05g0134000	P$PBF_01	BF	3006	3017	1	1	0.975	tgaAAAAGtgc
Os05g0134000	P$DOF_Q2	DOF	3006	3017	1	1	0.978	tgaAAAAGtgc
Os05g0134000	P$CDF2_01	CDF2	3007	3017	1	1	0.999	gaAAAAGtgc
Os05g0134000	P$CDF3_01	CDF3	3008	3017	1	1	1	aAAAAGtgc
Os05g0134000	P$PBF_Q2	BF	3016	3022	1	1	0.958	cAAAGG
Os05g0134000	P$SQUA_01	SQUA	3032	3042	1	1	0.886	caaTTTTTaa
Os05g0134000	P$SBF1_01	SBF1	3032	3046	1	1	0.914	caatttTTAATatg
Os05g0134000	P$MYB118_01	MYB118	3048	3065	1	1	0.918	ggacattacGTTACgaa
Os05g0134000	P$GAMYB_Q2	GAMYB	3059	3072	1	1	0.929	tacgaACAACcat
Os05g0134000	P$AT4G36620_01	AT4G36620	3063	3071	1	1	0.926	aacAACCA
Os05g0134000	P$GAMYB_01	GAMYB	3065	3073	1	1	0.891	CAACCata
Os05g0134000	P$HSFA4A_01	HSFA4A	3073	3079	1	1	0.964	cCTATT
Os05g0134000	P$LEC2_01	LEC2	3095	3106	1	1	0.933	aaCATGCtgca
Os05g0134000	P$ATHB5_01	ATHB5	3103	3112	1	1	0.852	gcaTTATTc
Os05g0134000	P$MYBAS1_01	MYBAS1	3117	3128	1	1	0.955	cgCCAACatga
Os05g0134000	P$RAV1_01	RAV1	3117	3129	1	1	0.938	cgcCAACAtgaa
Os05g0134000	P$GATA9_01	GATA9	3126	3137	1	1	0.884	gaaAGATCatc
Os05g0134000	P$AGP1_01	AGP1	3127	3137	1	1	0.855	aaAGATCatc
Os05g0134000	P$ARR10_01	ARR10	3129	3136	1	1	0.913	AGATCat
Os05g0134000	P$SBF1_01	SBF1	3132	3146	1	1	0.899	tcatccTTAAAtaa
Os05g0134000	P$PDF2_01	DF2	3135	3146	1	1	0.851	tcctTAAATaa
Os05g0134000	P$ATHB6_01	ATHB6	3139	3148	1	1	0.908	taAATAAga
Os05g0134000	P$BPC1_Q2	BPC1	3147	3153	1	1	0.997	AGAAAa
Os05g0134000	P$ATHB6_01	ATHB6	3150	3159	1	1	0.9	aaAATAAca
Os05g0134000	P$GT1_Q6_01	GT1	3166	3178	1	1	0.892	TTTGTcttgact
Os05g0134000	P$ID1_01	ID1	3166	3177	1	1	0.861	tTTGTCttgac
Os05g0134000	P$WRKY11_Q2	WRKY11	3171	3179	1	1	0.95	cTTGACta
Os05g0134000	P$CBNAC_01	CBNAC	3180	3186	1	1	0.968	aTGCTT
Os05g0134000	P$EDT1_01	EDT1	3191	3201	1	1	0.943	tacTTAATga
Os05g0134000	P$MRP1_Q2	MRP1	3203	3215	1	1	0.929	taTCTATatctt
Os05g0134000	P$ARR2_01	ARR2	3206	3216	1	1	0.885	ctatATCTTg
Os05g0134000	P$C1_Q2	C1	3216	3227	1	1	0.956	aaAACTAtctg
Os05g0134000	P$PEND_02	END	3230	3240	1	1	0.911	agTTCTTttt
Os05g0134000	P$RIN_Q2	RIN	3234	3245	1	1	0.987	ctttTTTAAgg
Os05g0134000	P$PBF_Q2	BF	3257	3263	1	1	0.958	cAAAGG
Os05g0134000	P$AT1G19490_01	AT1G19490	3261	3270	1	1	0.872	GGTTTcatc
Os05g0134000	P$ERF112_02	ERF112	3269	3279	1	1	0.918	ctCGCCGata
Os05g0134000	P$ERF069_01	ERF069	3270	3279	1	1	0.987	tCGCCGata
Os05g0134000	P$ERF11_01	ERF11	3270	3280	1	1	0.971	tCGCCGatag
Os05g0134000	P$ERF8_01	ERF8	3271	3281	1	1	0.947	CGCCGatagc
Os05g0134000	P$ARR2_01	ARR2	3279	3289	1	1	0.892	gcctATCTTa
Os05g0134000	P$MYB24_01	MYB24	3283	3292	1	1	0.853	atcTTAGGa

Os05g0134000	P$AT4G36620_01	AT4G36620	3288	3296	1	1	0.888	aggAACCA
Os05g0134000	P$BHLH64_02	BHLH64	3292	3298	1	1	1	ACCAGt
Os05g0134000	P$EDT1_01	EDT1	3296	3306	1	1	0.938	gttTTAATga
Os05g0134000	P$KNOX3_01	KNOX3	3299	3311	1	1	0.963	ttaaTGACActg
Os05g0134000	P$ATH1_01	ATH1	3303	3311	1	1	0.901	TGACActg
Os05g0134000	P$AT5G04240_01	AT5G04240	3320	3326	1	1	1	gGGCAC
Os05g0134000	P$HSFA4A_01	HSFA4A	3328	3334	1	1	0.914	tCTATT
Os05g0134000	P$WRKY18_02	WRKY18	3351	3361	1	1	0.947	tatGTCAAca
Os05g0134000	P$WRKY21_02	WRKY21	3351	3361	1	1	0.982	tatGTCAAca
Os05g0134000	P$WRKY48_02	WRKY48	3351	3361	1	1	0.989	tatGTCAAca
Os05g0134000	P$WRKY57_01	WRKY57	3351	3361	1	1	0.976	tatGTCAAca
Os05g0134000	P$WRKY60_01	WRKY60	3351	3362	1	1	0.908	tatGTCAAcat
Os05g0134000	P$WRKY15_01	WRKY15	3352	3362	1	1	0.977	atGTCAAcat
Os05g0134000	P$WRKY2_01	WRKY2	3352	3360	1	1	0.917	atGTCAAc
Os05g0134000	P$WRKY25_02	WRKY25	3352	3360	1	1	0.904	atGTCAAc
Os05g0134000	P$WRKY40_01	WRKY40	3352	3360	1	1	0.977	atGTCAAc
Os05g0134000	P$WRKY43_02	WRKY43	3352	3362	1	1	0.964	atGTCAAcat
Os05g0134000	P$WRKY62_01	WRKY62	3352	3360	1	1	0.89	atGTCAAc
Os05g0134000	P$WRKY63_01	WRKY63	3352	3360	1	1	0.894	atGTCAAc
Os05g0134000	P$WRKY75_01	WRKY75	3352	3360	1	1	0.959	atGTCAAc
Os05g0134000	P$WRKY8_01	WRKY8	3352	3361	1	1	0.987	atGTCAAca
Os05g0134000	P$WRKY23_01	WRKY23	3353	3361	1	1	0.899	tGTCAAca
Os05g0134000	P$WRKY30_01	WRKY30	3353	3363	1	1	0.974	tGTCAAcatg
Os05g0134000	P$RAV1_01	RAV1	3353	3365	1	1	0.916	tgtCAACAtgta
Os05g0134000	P$WRKY18_Q2	WRKY18	3354	3363	1	1	0.957	GTCAAcatg
Os05g0134000	P$O2_Q4	O2	3355	3366	1	1	0.852	tcaaCATGTaa
Os05g0134000	P$WRKY48_01	WRKY48	3360	3369	1	1	0.871	atgtAACAA
Os05g0134000	P$GAMYB_Q2	GAMYB	3360	3373	1	1	0.939	atgtaACAACtat
Os05g0134000	P$ATMYB15_Q2	ATMYB15	3363	3369	1	1	1	TAACAa
Os05g0134000	P$C1_Q2	C1	3365	3376	1	1	0.927	acAACTAtatt
Os05g0134000	P$ABZ1_01	ABZ1	3385	3399	1	1	0.896	tacagACGTGaaag
Os05g0134000	P$TGA1B_01	TGA1B	3387	3397	1	1	0.879	caGACGTgaa
Os05g0134000	P$HBP1A_Q2	HBP1A	3387	3397	1	1	0.884	cagACGTGaa
Os05g0134000	P$TAF1_Q2	TAF1	3387	3397	1	1	0.931	cagACGTGaa
Os05g0134000	P$EMBP1_02	EMBP1	3387	3397	1	1	0.922	cagACGTGaa
Os05g0134000	P$TAF1_01	TAF1	3387	3397	1	1	0.951	cagACGTGaa
Os05g0134000	P$GBF1_01	GBF1	3388	3396	1	1	0.934	agACGTGa
Os05g0134000	P$BIM1_02	BIM1	3388	3398	1	1	0.947	agACGTGaaa
Os05g0134000	P$ABF4_02	ABF4	3388	3398	1	1	0.953	agACGTGaaa
Os05g0134000	P$ABI5_Q2	ABI5	3390	3396	1	1	0.936	ACGTGa
Os05g0134000	P$ABF2_01	ABF2	3399	3412	1	1	0.949	aattaCACGTtat
Os05g0134000	P$O2_Q4	O2	3400	3411	1	1	0.922	attaCACGTta
Os05g0134000	P$BZR1_02	BZR1	3400	3414	1	1	0.86	attaCACGTtatta
Os05g0134000	P$NAC92_01	NAC92	3401	3413	1	1	0.95	ttACACGttatt
Os05g0134000	P$GBP_Q6	GBP	3401	3413	1	1	0.883	ttaCACGTtatt
Os05g0134000	P$ABI5_01	ABI5	3401	3411	1	1	0.931	ttaCACGTta
Os05g0134000	P$ABF4_01	ABF4	3401	3413	1	1	0.906	ttaCACGTtatt
Os05g0134000	P$EMBP1_Q2	EMBP1	3402	3412	1	1	0.856	taCACGTtat
Os05g0134000	P$CPRF3_Q2	CPRF3	3402	3412	1	1	0.919	taCACGTtat
Os05g0134000	P$CPRF2_Q2	CPRF2	3402	3412	1	1	0.939	taCACGTtat
Os05g0134000	P$O2_02	O2	3402	3412	1	1	0.945	taCACGTtat
Os05g0134000	P$TGA1B_Q2	TGA1B	3402	3412	1	1	0.899	taCACGTtat
Os05g0134000	P$TGA1A_Q2	TGA1A	3402	3412	1	1	0.969	taCACGTtat
Os05g0134000	P$CPRF3_01	CPRF3	3402	3412	1	1	0.93	taCACGTtat
Os05g0134000	P$CPRF2_01	CPRF2	3402	3412	1	1	0.941	taCACGTtat
Os05g0134000	P$TGA1B_01	TGA1B	3402	3412	1	1	0.85	taCACGTtat
Os05g0134000	P$BEE2_01	BEE2	3402	3412	1	1	0.92	taCACGTtat
Os05g0134000	P$BIM2_01	BIM2	3402	3412	1	1	0.855	taCACGTtat
Os05g0134000	P$BIM3_01	BIM3	3402	3412	1	1	0.885	taCACGTtat
Os05g0134000	P$PHYPA143875_02	HYPA143875	3402	3412	1	1	0.885	taCACGTtat
Os05g0134000	P$SPT_01	SPT	3402	3411	1	1	0.913	taCACGTta
Os05g0134000	P$GBF1F_Q2	GBF1F	3402	3413	1	1	0.855	taCACGTtatt
Os05g0134000	P$RITA1_01	RITA1	3403	3410	1	1	0.953	aCACGTt
Os05g0134000	P$OCSBF1_01	OCSBF1	3404	3409	1	1	1	CACGT
Os05g0134000	P$BPC1_Q2	BPC1	3420	3426	1	1	0.997	AGAAAa
Os05g0134000	P$ATHB6_01	ATHB6	3422	3431	1	1	0.905	aaAATAAag
Os05g0134000	P$DOF1_01	DOF1	3423	3434	1	1	0.985	aaaTAAAGttt
Os05g0134000	P$GAMYB_Q2	GAMYB	3441	3454	1	1	0.877	ataacACAACggt
Os05g0134000	P$AT5G54070_01	AT5G54070	3446	3452	1	1	0.958	aCAACG
Os05g0134000	P$BPC1_Q2	BPC1	3455	3461	1	1	1	AGAAAg
Os05g0134000	P$PBF_Q2	BF	3456	3462	1	1	0.965	gAAAGG
Os05g0134000	P$ARF8_01	ARF8	3464	3473	1	1	0.958	atTGTCGaa
Os05g0134000	P$ATHB6_01	ATHB6	3481	3490	1	1	0.903	gaAATAAac
Os05g0134000	P$SBF1_01	SBF1	3491	3505	1	1	0.915	gctaagTTAAAaac
Os05g0134000	P$C1_Q2	C1	3500	3511	1	1	0.934	aaAACTAcgaa
Os05g0134000	P$PBF_01	BF	3507	3518	1	1	0.955	cgaAAAAGaag
Os05g0134000	P$DOF_Q2	DOF	3507	3518	1	1	0.994	cgaAAAAGaag
Os05g0134000	P$CDF2_01	CDF2	3508	3518	1	1	0.976	gaAAAAGaag

Os05g0134000	P$CDF3_01	CDF3	3509	3518	1	1	0.974	aAAAAGaag
Os05g0134000	P$PEND_01	END	3511	3519	1	1	0.891	aAAGAAgt
Os05g0134000	P$ATHB6_01	ATHB6	3551	3560	1	1	0.921	acAATAAca
Os05g0134000	P$HSFA4A_01	HSFA4A	3559	3565	1	1	1	aCTATT
Os05g0134000	P$RAV1_01	RAV1	3563	3575	1	1	0.923	tttCAACAtcaa
Os05g0134000	P$PEND_01	END	3572	3580	1	1	0.854	cAAGAAct
Os05g0134000	P$KNOX3_01	KNOX3	3583	3595	1	1	0.963	agtaTGACAtaa
Os05g0134000	P$ATH1_01	ATH1	3587	3595	1	1	0.937	TGACAtaa
Os05g0134000	P$ATHB6_01	ATHB6	3640	3649	1	1	0.908	caAATAAat
Os05g0134000	P$MYBAS1_01	MYBAS1	3654	3665	1	1	0.957	agCCAACcaca
Os05g0134000	P$AT4G36620_01	AT4G36620	3655	3663	1	1	0.886	gccAACCA
Os05g0134000	P$GAMYB_01	GAMYB	3657	3665	1	1	0.901	CAACCaca
Os05g0134000	P$HSFA2_01	HSFA2	3683	3689	1	1	1	CCAAAa
Os05g0134000	P$LIM1_01	LIM1	3695	3707	1	1	0.948	CCACCacaaaat
Os05g0134000	P$PBF_Q2	BF	3730	3736	1	1	0.958	cAAAGG
Os05g0134000	P$GT1_Q6	GT1	3735	3742	1	1	0.971	GTAAAaa
Os05g0134000	P$HAT1_01	HAT1	3767	3777	1	1	0.852	aaAATCAgag
Os05g0134000	P$REF6_01	REF6	3768	3779	1	1	0.862	aaatCAGAGag
Os05g0134000	P$OJ1581_01	OJ1581	3779	3789	1	1	0.99	atGGGCCcca
Os05g0134000	P$TCP2_01	TCP2	3779	3789	1	1	0.97	atGGGCCcca
Os05g0134000	P$PCF2_01	CF2	3780	3790	1	1	1	tgggcCCCAC
Os05g0134000	P$TCP19_01	TCP19	3780	3790	1	1	0.998	tgggcCCCAC
Os05g0134000	P$TCP20L_01	TCP20L	3781	3790	1	1	0.997	gggcCCCAC
Os05g0134000	P$OSI_01	OSI	3782	3790	1	1	0.959	ggcCCCAC
Os05g0134000	P$TCP20_02	TCP20	3782	3792	1	1	0.998	ggcCCCACac
Os05g0134000	P$ARALY495258_02	ARALY495258	3782	3790	1	1	1	ggcCCCAC
Os05g0134000	P$ARALY493022_04	ARALY493022	3782	3790	1	1	0.973	ggcCCCAC
Os05g0134000	P$ARALY484486_05	ARALY484486	3782	3790	1	1	1	ggcCCCAC
Os05g0134000	P$NAC92_01	NAC92	3786	3798	1	1	0.943	ccACACGagatg
Os05g0134000	P$BBM_01	BBM	3802	3812	1	1	0.863	tGGCGCcacg
Os05g0134000	P$E2FA_02	E2FA	3802	3812	1	1	0.997	tggCGCCAcg
Os05g0134000	P$SED_Q2	SED	3817	3827	1	1	0.916	ggaaCCTTTc
Os05g0134000	P$PBF_Q2_01	BF	3821	3827	1	1	0.985	CCTTTc
Os05g0134000	P$AT5G54070_01	AT5G54070	3825	3831	1	1	0.91	tCAACG
Os05g0134000	P$WRKY18_02	WRKY18	3828	3838	1	1	0.997	acgGTCAAac
Os05g0134000	P$WRKY21_02	WRKY21	3828	3838	1	1	0.969	acgGTCAAac
Os05g0134000	P$WRKY48_02	WRKY48	3828	3838	1	1	0.998	acgGTCAAac
Os05g0134000	P$WRKY57_01	WRKY57	3828	3838	1	1	0.974	acgGTCAAac
Os05g0134000	P$WRKY60_01	WRKY60	3828	3839	1	1	0.98	acgGTCAAaca
Os05g0134000	P$WRKY15_01	WRKY15	3829	3839	1	1	0.985	cgGTCAAaca
Os05g0134000	P$WRKY2_01	WRKY2	3829	3837	1	1	0.991	cgGTCAAa
Os05g0134000	P$WRKY25_02	WRKY25	3829	3837	1	1	0.979	cgGTCAAa
Os05g0134000	P$WRKY40_01	WRKY40	3829	3837	1	1	1	cgGTCAAa
Os05g0134000	P$WRKY43_02	WRKY43	3829	3839	1	1	0.976	cgGTCAAaca
Os05g0134000	P$WRKY62_01	WRKY62	3829	3837	1	1	0.91	cgGTCAAa
Os05g0134000	P$WRKY63_01	WRKY63	3829	3837	1	1	0.991	cgGTCAAa
Os05g0134000	P$WRKY75_01	WRKY75	3829	3837	1	1	0.975	cgGTCAAa
Os05g0134000	P$WRKY8_01	WRKY8	3829	3838	1	1	0.992	cgGTCAAac
Os05g0134000	P$WRKY23_01	WRKY23	3830	3838	1	1	0.854	gGTCAAac
Os05g0134000	P$WRKY30_01	WRKY30	3830	3840	1	1	0.918	gGTCAAacag
Os05g0134000	P$WRKY18_Q2	WRKY18	3831	3840	1	1	0.927	GTCAAacag
Os05g0134000	P$SBF1_01	SBF1	3839	3853	1	1	0.882	gacaagTTAATtac
Os05g0134000	P$EDT1_01	EDT1	3842	3852	1	1	0.861	aagTTAATta
Os05g0134000	P$SBF1_01	SBF1	3847	3861	1	1	0.861	aattacTTAAAaca
Os05g0134000	P$SBF1_01	SBF1	3856	3870	1	1	0.882	aaacaaTTAATaat
Os05g0134000	P$EDT1_01	EDT1	3859	3869	1	1	0.894	caaTTAATaa
Os05g0134000	P$ATHB6_01	ATHB6	3862	3871	1	1	0.978	ttAATAAtc
Os05g0134000	P$ATHB5_04	ATHB5	3862	3873	1	1	0.904	ttAATAAtcaa
Os05g0134000	P$ATHB1_03	ATHB1	3862	3873	1	1	0.898	ttAATAAtcaa
Os05g0134000	P$ATHB16_01	ATHB16	3863	3871	1	1	0.908	tAATAAtc
Os05g0134000	P$ATHB7_01	ATHB7	3865	3875	1	1	0.89	atAATCAatt
Os05g0134000	P$HAT1_01	HAT1	3865	3875	1	1	0.886	atAATCAatt
Os05g0134000	P$EDT1_01	EDT1	3870	3880	1	1	0.901	caaTTAATta
Os05g0134000	P$SBF1_01	SBF1	3871	3885	1	1	0.873	aattaaTTAATtaa
Os05g0134000	P$EDT1_01	EDT1	3874	3884	1	1	0.899	taaTTAATta
Os05g0134000	P$SBF1_01	SBF1	3875	3889	1	1	0.873	aattaaTTAATtaa
Os05g0134000	P$EDT1_01	EDT1	3878	3888	1	1	0.899	taaTTAATta
Os05g0134000	P$SBF1_01	SBF1	3879	3893	1	1	0.873	aattaaTTAATtaa
Os05g0134000	P$EDT1_01	EDT1	3882	3892	1	1	0.899	taaTTAATta
Os05g0134000	P$SBF1_01	SBF1	3883	3897	1	1	0.873	aattaaTTAATtaa
Os05g0134000	P$EDT1_01	EDT1	3886	3896	1	1	0.899	taaTTAATta
Os05g0134000	P$SBF1_01	SBF1	3887	3901	1	1	0.862	aattaaTTAATact
Os05g0134000	P$EDT1_01	EDT1	3890	3900	1	1	0.906	taaTTAATac
Os05g0134000	P$GT1_Q6	GT1	3905	3912	1	1	0.912	GTAAAca
Os05g0134000	P$AT3G20750_01	AT3G20750	3905	3913	1	1	0.943	gTAAACat
Os05g0134000	P$AGL15_03	AGL15	3918	3933	1	1	0.865	TTTCCcttttttta
Os05g0134000	P$SED_Q2	SED	3918	3928	1	1	0.981	tttcCCTTTt
Os05g0134000	P$PBF_Q2_01	BF	3922	3928	1	1	1	CCTTTt

Os05g0134000	P$SQUA_01	SQUA	3922	3932	1	1	0.877	cctTTTTTtt
Os05g0134000	P$SQUA_01	SQUA	3923	3933	1	1	0.859	cttTTTTTta
Os05g0134000	P$ARF8_01	ARF8	3940	3949	1	1	0.953	tgTGTCGca
Os05g0134000	P$AT5G61590_01	AT5G61590	3987	3997	1	1	0.961	GCTCCtcctt
OsSK12	P$KNOX3_01	KNOX3	49	61	1	1	0.979	ggacTGACAagt
OsSK12	P$ATH1_01	ATH1	53	61	1	1	0.931	TGACAagt
OsSK12	P$KNOX3_01	KNOX3	85	97	1	1	0.981	gggcTGACAttt
OsSK12	P$ATH1_01	ATH1	89	97	1	1	0.933	TGACAttt
OsSK12	P$KNOX3_01	KNOX3	96	108	1	1	0.981	tgtcTGACAtga
OsSK12	P$ATH1_01	ATH1	100	108	1	1	0.946	TGACAtga
OsSK12	P$AT2G41690_01	AT2G41690	108	114	1	1	0.974	CCGAAt
OsSK12	P$GAMYB_Q2	GAMYB	113	126	1	1	0.874	tttacACAACttt
OsSK12	P$NAC083_01	NAC083	126	136	1	1	0.991	ttACGCAatt
OsSK12	P$ASR1_01	ASR1	138	143	1	1	1	ACCCA
OsSK12	P$BPC1_Q2	BPC1	183	189	1	1	0.997	AGAAAa
OsSK12	P$PBF_01	BF	185	196	1	1	0.971	aaaAAAAGttc
OsSK12	P$DOF_Q2	DOF	185	196	1	1	0.982	aaaAAAAGttc
OsSK12	P$CDF2_01	CDF2	186	196	1	1	0.995	aaAAAAGttc
OsSK12	P$CDF3_01	CDF3	187	196	1	1	0.99	aAAAAGttc
OsSK12	P$AT2G41690_01	AT2G41690	209	215	1	1	1	CCGAAc
OsSK12	P$C1_Q2	C1	210	221	1	1	0.958	cgAACTAattc
OsSK12	P$AT1G59530_01	AT1G59530	224	238	1	1	0.87	ggatcacttAACCC
OsSK12	P$AT1G66560_01	AT1G66560	228	238	1	1	0.942	cacTTAACcc
OsSK12	P$WRKY21_01	WRKY21	229	238	1	1	0.949	acTTAACcc
OsSK12	P$AT2G34830_01	AT2G34830	229	238	1	1	0.952	acTTAACcc
OsSK12	P$AT1G18860_01	AT1G18860	229	238	1	1	0.964	acTTAACcc
OsSK12	P$AT1G64000_01	AT1G64000	229	238	1	1	0.937	acTTAACcc
OsSK12	P$AT4G22070_01	AT4G22070	229	238	1	1	0.946	acTTAACcc
OsSK12	P$WRKY6_01	WRKY6	229	238	1	1	0.943	acTTAACcc
OsSK12	P$AT1G66600_01	AT1G66600	229	238	1	1	0.94	acTTAACcc
OsSK12	P$AT1G68150_01	AT1G68150	229	238	1	1	0.96	acTTAACcc
OsSK12	P$AT5G41570_01	AT5G41570	229	238	1	1	0.936	acTTAACcc
OsSK12	P$AT1G69810_01	AT1G69810	229	238	1	1	0.963	acTTAACcc
OsSK12	P$AT5G15130_01	AT5G15130	229	238	1	1	0.965	acTTAACcc
OsSK12	P$WRKY46_01	WRKY46	229	238	1	1	0.88	acTTAACcc
OsSK12	P$AT1G30650_01	AT1G30650	229	238	1	1	0.952	acTTAACcc
OsSK12	P$AT2G24570_01	AT2G24570	229	238	1	1	0.949	acTTAACcc
OsSK12	P$AT4G23550_01	AT4G23550	229	238	1	1	0.952	acTTAACcc
OsSK12	P$WRKY7_01	WRKY7	229	238	1	1	0.953	acTTAACcc
OsSK12	P$WRKY25_01	WRKY25	229	238	1	1	0.969	actTAACCc
OsSK12	P$WRKY33_01	WRKY33	229	238	1	1	0.883	actTAACCc
OsSK12	P$AT1G29860_01	AT1G29860	229	238	1	1	0.919	actTAACCc
OsSK12	P$AT3G62340_01	AT3G62340	229	238	1	1	0.907	actTAACCc
OsSK12	P$AT1G69310_01	AT1G69310	229	238	1	1	0.933	actTAACCc
OsSK12	P$WRKY26_01	WRKY26	229	238	1	1	0.887	actTAACCc
OsSK12	P$GT1_01	GT1	230	238	1	1	0.948	ctTAACCc
OsSK12	P$C1_Q2	C1	238	249	1	1	0.939	tgAACTAtttt
OsSK12	P$HSFA4A_01	HSFA4A	241	247	1	1	1	aCTATT
OsSK12	P$SQUA_01	SQUA	242	252	1	1	0.931	ctaTTTTTtg
OsSK12	P$SEP3_01	wrz-03	242	253	1	1	0.869	ctattTTTTGg
OsSK12	P$PH4_01	H4	259	268	1	1	0.854	tttACCCCt
OsSK12	P$C1_Q2	C1	268	279	1	1	0.943	taAACTAttaa
OsSK12	P$SBF1_01	SBF1	269	283	1	1	0.882	aaactaTTAAAaat
OsSK12	P$HSFA4A_01	HSFA4A	271	277	1	1	1	aCTATT
OsSK12	P$HAHB4_01	HAHB4	279	288	1	1	0.858	aAATGAttc
OsSK12	P$UIF1_01	UIF1	280	290	1	1	0.863	aatGATTCag
OsSK12	P$SBF1_01	SBF1	292	306	1	1	0.924	tactccTTAATaac
OsSK12	P$ATHB6_01	ATHB6	298	307	1	1	0.906	ttAATAAca
OsSK12	P$ATMYB15_Q2	ATMYB15	302	308	1	1	0.865	TAACAg
OsSK12	P$HSFA4A_01	HSFA4A	309	315	1	1	0.914	tCTATT
OsSK12	P$SQUA_01	SQUA	310	320	1	1	0.915	ctaTTTTTtt
OsSK12	P$GT1_Q6_01	GT1	315	327	1	1	0.96	TTTTTtgttact
OsSK12	P$PBF_01	BF	331	342	1	1	0.971	tatAAAAGtta
OsSK12	P$DOF_Q2	DOF	331	342	1	1	0.932	tatAAAAGtta
OsSK12	P$CDF2_01	CDF2	332	342	1	1	0.968	atAAAAGtta
OsSK12	P$CDF3_01	CDF3	333	342	1	1	0.984	tAAAAGtta
OsSK12	P$RIN_Q2	RIN	341	352	1	1	0.852	atatTTTAAgc
OsSK12	P$KNOX3_01	KNOX3	370	382	1	1	0.973	tagaTGACAcct
OsSK12	P$ATH1_01	ATH1	374	382	1	1	0.919	TGACAcct
OsSK12	P$SED_Q2	SED	375	385	1	1	0.914	gacaCCTTTa
OsSK12	P$PBF_Q2_01	BF	379	385	1	1	0.998	CCTTTa
OsSK12	P$CBNAC_01	CBNAC	424	430	1	1	1	tTGCTT
OsSK12	P$CBNAC_02	CBNAC	424	440	1	1	0.93	tTGCTTgaactttaaa
OsSK12	P$AT2G26880_01	AT2G26880	427	441	1	1	0.872	cttgaacTTTAAaa
OsSK12	P$AT1G77950_01	AT1G77950	429	440	1	1	0.88	tgaacTTTAAa
OsSK12	P$SBF1_01	SBF1	429	443	1	1	0.879	tgaactTTAAAatg
OsSK12	P$ATHB7_01	ATHB7	451	461	1	1	0.856	taAATCAgtt
OsSK12	P$HAT1_01	HAT1	451	461	1	1	0.862	taAATCAgtt

OsSK12	P$GT1_Q6_02	GT1	455	467	1	1	0.948	tcagttTTAACt
OsSK12	P$CBNAC_01	CBNAC	465	471	1	1	0.973	cTGCTT
OsSK12	P$CBNAC_02	CBNAC	465	481	1	1	0.867	cTGCTTccaacgtaca
OsSK12	P$MYBAS1_01	MYBAS1	469	480	1	1	0.975	ttCCAACgtac
OsSK12	P$ATSPL3_01	ATSPL3	470	486	1	1	0.954	tccaaCGTACataaac
OsSK12	P$AT5G54070_01	AT5G54070	471	477	1	1	1	cCAACG
OsSK12	P$SPL14_03	SPL14	473	484	1	1	0.862	aaCGTACataa
OsSK12	P$AT3G20750_01	AT3G20750	480	488	1	1	0.888	aTAAACag
OsSK12	P$BPC1_Q2	BPC1	490	496	1	1	0.997	AGAAAa
OsSK12	P$PBF_01	BF	502	513	1	1	0.967	aaaAAAAGttg
OsSK12	P$DOF_Q2	DOF	502	513	1	1	0.984	aaaAAAAGttg
OsSK12	P$CDF2_01	CDF2	503	513	1	1	0.994	aaAAAAGttg
OsSK12	P$CDF3_01	CDF3	504	513	1	1	0.989	aAAAAGttg
OsSK12	P$C1_Q2	C1	530	541	1	1	0.927	gcAACTAtatt
OsSK12	P$P_01		541	550	1	1	0.879	ttCTACCaa
OsSK12	P$HSFA2_01	HSFA2	546	552	1	1	0.922	CCAAAt
OsSK12	P$ARR18_01	ARR18	569	582	1	1	0.897	ctatAGATAaaaa
OsSK12	P$AP1_01	AP1	582	595	1	1	0.853	caAAAAAgagaga
OsSK12	P$PBF_01	BF	582	593	1	1	0.991	caaAAAAGaga
OsSK12	P$DOF_Q2	DOF	582	593	1	1	0.984	caaAAAAGaga
OsSK12	P$CDF2_01	CDF2	583	593	1	1	0.979	aaAAAAGaga
OsSK12	P$CDF3_01	CDF3	584	593	1	1	0.984	aAAAAGaga
OsSK12	P$ARR18_01	ARR18	588	601	1	1	0.888	agagAGATAgcgt
OsSK12	P$ARR2_01	ARR2	625	635	1	1	0.903	caatATCTTg
OsSK12	P$ASR1_01	ASR1	649	654	1	1	1	ACCCA
OsSK12	P$MYBAS1_01	MYBAS1	649	660	1	1	0.952	acCCAACaata
OsSK12	P$RAV1_01	RAV1	649	661	1	1	0.946	accCAACAatag
OsSK12	P$RIN_01	RIN	651	661	1	1	0.92	ccaacAATAG
OsSK12	P$SPF1_Q2	SPF1	655	665	1	1	0.883	caATAGTtta
OsSK12	P$SPF1_Q2	SPF1	685	695	1	1	0.875	aaATAGTtcc
OsSK12	P$GT1_Q6_01	GT1	709	721	1	1	0.861	TTTTTttttcaa
OsSK12	P$GT1_Q6_01	GT1	710	722	1	1	0.954	TTTTTtttcaaa
OsSK12	P$GAMYB_Q2	GAMYB	734	747	1	1	0.887	attctACAACgtt
OsSK12	P$ATHSFA1D_01	ATHSFA1D	736	742	1	1	0.941	tCTACA
OsSK12	P$AT5G54070_01	AT5G54070	739	745	1	1	0.958	aCAACG
OsSK12	P$SBF1_01	SBF1	742	756	1	1	0.884	acgttgTTAAAcat
OsSK12	P$AT3G20750_01	AT3G20750	748	756	1	1	0.969	tTAAACat
OsSK12	P$MYB118_01	MYB118	749	766	1	1	0.854	taaacatagGTTACatc
OsSK12	P$ANTL_01	ANTL	757	767	1	1	0.878	gGTTACatca
OsSK12	P$AT3G20750_01	AT3G20750	767	775	1	1	0.969	tTAAACat
OsSK12	P$AT3G20750_01	AT3G20750	781	789	1	1	0.882	gTAAACtt
OsSK12	P$PDF2_01	DF2	791	802	1	1	0.897	atatTAAATga
OsSK12	P$ARR18_01	ARR18	797	810	1	1	0.91	aatgAGATAtaca
OsSK12	P$C1_Q2	C1	831	842	1	1	0.947	tcAACTAtaaa
OsSK12	P$GAMYB_Q2	GAMYB	836	849	1	1	0.933	tataaACAACacc
OsSK12	P$AT3G20750_01	AT3G20750	837	845	1	1	0.907	aTAAACaa
OsSK12	P$RAV1_01	RAV1	839	851	1	1	0.922	aaaCAACAccac
OsSK12	P$BPC1_Q2	BPC1	863	869	1	1	1	AGAAAg
OsSK12	P$PEND_02	END	877	887	1	1	0.891	aaTTCTTgta
OsSK12	P$GAMYB_Q2	GAMYB	884	897	1	1	0.934	gtaaaACAACaca
OsSK12	P$RAV1_01	RAV1	887	899	1	1	0.944	aaaCAACAcacc
OsSK12	P$HSFA4A_01	HSFA4A	897	903	1	1	0.964	cCTATT
OsSK12	P$RAV1_01	RAV1	920	932	1	1	0.943	catCAACAaagg
OsSK12	P$PBF_Q2	BF	926	932	1	1	0.958	cAAAGG
OsSK12	P$RAV1_02	RAV1	944	956	1	1	0.91	aatACCTGtgta
OsSK12	P$SED_Q2	SED	956	966	1	1	0.985	gttcCCTTTa
OsSK12	P$PBF_Q2_01	BF	960	966	1	1	0.998	CCTTTa
OsSK12	P$FLC_01	FLC	975	996	1	1	0.901	acattccacaagtAGAAAatg
OsSK12	P$AT5G04340_01	AT5G04340	984	996	1	1	0.851	aagtAGAAAatg
OsSK12	P$BPC1_Q2	BPC1	988	994	1	1	0.997	AGAAAa
OsSK12	P$PBF_Q2	BF	1000	1006	1	1	0.958	cAAAGG
OsSK12	P$WRKY18_02	WRKY18	1002	1012	1	1	0.999	aagGTCAAgg
OsSK12	P$WRKY21_02	WRKY21	1002	1012	1	1	0.963	aagGTCAAgg
OsSK12	P$WRKY48_02	WRKY48	1002	1012	1	1	0.996	aagGTCAAgg
OsSK12	P$WRKY57_01	WRKY57	1002	1012	1	1	0.971	aagGTCAAgg
OsSK12	P$WRKY60_01	WRKY60	1002	1013	1	1	0.968	aagGTCAAggt
OsSK12	P$WRKY15_01	WRKY15	1003	1013	1	1	0.98	agGTCAAggt
OsSK12	P$WRKY2_01	WRKY2	1003	1011	1	1	0.98	agGTCAAg
OsSK12	P$WRKY25_02	WRKY25	1003	1011	1	1	0.959	agGTCAAg
OsSK12	P$WRKY40_01	WRKY40	1003	1011	1	1	1	agGTCAAg
OsSK12	P$WRKY43_02	WRKY43	1003	1013	1	1	0.974	agGTCAAggt
OsSK12	P$WRKY62_01	WRKY62	1003	1011	1	1	0.884	agGTCAAg
OsSK12	P$WRKY63_01	WRKY63	1003	1011	1	1	0.992	agGTCAAg
OsSK12	P$WRKY75_01	WRKY75	1003	1011	1	1	0.959	agGTCAAg
OsSK12	P$WRKY8_01	WRKY8	1003	1012	1	1	0.989	agGTCAAgg
OsSK12	P$WRKY23_01	WRKY23	1004	1012	1	1	0.875	gGTCAAgg
OsSK12	P$WRKY30_01	WRKY30	1004	1014	1	1	0.922	gGTCAAggtt
OsSK12	P$WRKY18_Q2	WRKY18	1005	1014	1	1	0.989	GTCAAggtt

OsSK12	P$DRE1C_01	DRE1C	1035	1043	1	1	0.862	ATGTCagc
OsSK12	P$ABI3_01	ABI3	1039	1048	1	1	0.989	caGCATGca
OsSK12	P$FUS3_01	FUS3	1040	1049	1	1	0.956	aGCATGcag
OsSK12	P$LEC2_01	LEC2	1040	1051	1	1	0.994	agCATGCagat
OsSK12	P$FUS3_Q2	FUS3	1041	1052	1	1	0.865	gCATGCagata
OsSK12	P$ARR18_01	ARR18	1043	1056	1	1	0.888	atgcAGATAatca
OsSK12	P$ATHB7_01	ATHB7	1049	1059	1	1	0.882	atAATCAaga
OsSK12	P$HAT1_01	HAT1	1049	1059	1	1	0.881	atAATCAaga
OsSK12	P$GATA9_01	GATA9	1053	1064	1	1	0.895	tcaAGATCaac
OsSK12	P$AGP1_01	AGP1	1054	1064	1	1	0.903	caAGATCaac
OsSK12	P$ARR10_01	ARR10	1056	1063	1	1	0.869	AGATCaa
OsSK12	P$GAMYB_01	GAMYB	1060	1068	1	1	1	CAACCgcc
OsSK12	P$AT5G46350_01	AT5G46350	1062	1071	1	1	0.871	ACCGCccta
OsSK12	P$AT3G63350_01	AT3G63350	1063	1069	1	1	1	CCGCCc
OsSK12	P$MYB1L_01	MYB1L	1064	1074	1	1	0.97	cgCCCTAgaa
OsSK12	P$TRB2_01	TRB2	1064	1072	1	1	0.987	cgCCCTAg
OsSK12	P$BPC1_Q2	BPC1	1070	1076	1	1	0.997	AGAAAa
OsSK12	P$NAC078_Q2	NAC078	1075	1090	1	1	0.881	actTGGAGaacaaat
OsSK12	P$DREB1A_04	DREB1A	1105	1115	1	1	0.931	tcGTCGGacg
OsSK12	P$ERF039_01	ERF039	1105	1115	1	1	0.944	tcGTCGGacg
OsSK12	P$AT5G04240_01	AT5G04240	1134	1140	1	1	0.939	aGGCAC
OsSK12	P$GAMYB_01	GAMYB	1147	1155	1	1	0.925	CAACCgtg
OsSK12	P$GATA15_01	GATA15	1151	1160	1	1	1	cgTGATCaa
OsSK12	P$HSFA2_01	HSFA2	1161	1167	1	1	0.922	CCAAAt
OsSK12	P$RAV1_02	RAV1	1181	1193	1	1	0.985	ttcACCTGctgg
OsSK12	P$E2L_Q2	E2L	1193	1200	1	1	0.957	aGGCGGg
OsSK12	P$MYBAS1_01	MYBAS1	1208	1219	1	1	0.973	gtCCAACaaat
OsSK12	P$RAV1_01	RAV1	1208	1220	1	1	0.958	gtcCAACAaatc
OsSK12	P$CBNAC_01	CBNAC	1230	1236	1	1	0.979	gTGCTT
OsSK12	P$CBNAC_02	CBNAC	1230	1246	1	1	0.89	gTGCTTttcttgctag
OsSK12	P$ABI3_01	ABI3	1245	1254	1	1	0.852	ggGCATGag
OsSK12	P$GATA9_01	GATA9	1249	1260	1	1	0.883	atgAGATCgac
OsSK12	P$AGP1_01	AGP1	1250	1260	1	1	0.908	tgAGATCgac
OsSK12	P$ARR10_01	ARR10	1252	1259	1	1	0.869	AGATCga
OsSK12	P$GATA9_01	GATA9	1277	1288	1	1	0.888	gtgAGATCcca
OsSK12	P$AGP1_01	AGP1	1278	1288	1	1	0.873	tgAGATCcca
OsSK12	P$GATA10_01	GATA10	1279	1287	1	1	0.85	gAGATCcc
OsSK12	P$ARR10_01	ARR10	1280	1287	1	1	0.934	AGATCcc
OsSK12	P$AT3G60580_01	AT3G60580	1280	1287	1	1	0.852	agATCCC
OsSK12	P$RAV1_01	RAV1	1286	1298	1	1	0.92	catCAACAgctc
OsSK12	P$PEND_01	END	1297	1305	1	1	0.854	cAAGAAct
OsSK12	P$C1_Q2	C1	1299	1310	1	1	0.957	agAACTAccac
OsSK12	P$P_01		1301	1310	1	1	0.92	aaCTACCac
OsSK12	P$AT2G41690_01	AT2G41690	1311	1317	1	1	0.988	CCGAAg
OsSK12	P$AT3G20750_01	AT3G20750	1319	1327	1	1	0.943	aTAAACgt
OsSK12	P$HBPA1_Q6_01	HBPA1	1320	1330	1	1	0.866	taaaCGTCAc
OsSK12	P$TGA1A_01	TGA1A	1322	1329	1	1	0.989	aACGTCa
OsSK12	P$SBF1_01	SBF1	1382	1396	1	1	0.897	gtcagaTTAAAatg
OsSK12	P$ATSPL8_01	ATSPL8	1389	1405	1	1	0.966	taaaaTGTACtaaata
OsSK12	P$PDF2_01	DF2	1395	1406	1	1	0.854	gtacTAAATat
OsSK12	P$SBF1_01	SBF1	1399	1413	1	1	0.872	taaataTTAATtta
OsSK12	P$EDT1_01	EDT1	1402	1412	1	1	0.863	ataTTAATtt
OsSK12	P$SBF1_01	SBF1	1409	1423	1	1	0.942	tttatgTTAATatt
OsSK12	P$LEC2_01	LEC2	1434	1445	1	1	0.994	ttCATGCatat
OsSK12	P$FUS3_Q2	FUS3	1435	1446	1	1	0.897	tCATGCatatt
OsSK12	P$GT1_Q6_01	GT1	1465	1477	1	1	0.868	TTTTTtagtaat
OsSK12	P$GT1_Q6_01	GT1	1466	1478	1	1	0.857	TTTTTagtaata
OsSK12	P$LEC2_01	LEC2	1487	1498	1	1	0.981	ttCATGCaaca
OsSK12	P$RAV1_01	RAV1	1490	1502	1	1	0.954	atgCAACAagaa
OsSK12	P$BPC1_Q2	BPC1	1498	1504	1	1	0.997	AGAAAa
OsSK12	P$ARR18_01	ARR18	1507	1520	1	1	0.94	cataAGATAagaa
OsSK12	P$PEND_01	END	1514	1522	1	1	0.892	tAAGAAaa
OsSK12	P$BPC1_Q2	BPC1	1516	1522	1	1	0.997	AGAAAa
OsSK12	P$ATHB6_01	ATHB6	1519	1528	1	1	0.975	aaAATAAta
OsSK12	P$ATHB5_04	ATHB5	1519	1530	1	1	0.888	aaAATAAtaat
OsSK12	P$ATHB1_03	ATHB1	1519	1530	1	1	0.891	aaAATAAtaat
OsSK12	P$ATHB16_01	ATHB16	1520	1528	1	1	0.867	aAATAAta
OsSK12	P$ATHB6_01	ATHB6	1522	1531	1	1	0.978	atAATAAtc
OsSK12	P$ATHB5_04	ATHB5	1522	1533	1	1	0.904	atAATAAtcaa
OsSK12	P$ATHB1_03	ATHB1	1522	1533	1	1	0.898	atAATAAtcaa
OsSK12	P$ATHB16_01	ATHB16	1523	1531	1	1	0.908	tAATAAtc
OsSK12	P$ATHB7_01	ATHB7	1525	1535	1	1	0.882	atAATCAaaa
OsSK12	P$HAT1_01	HAT1	1525	1535	1	1	0.876	atAATCAaaa
OsSK12	P$MYB3_01	MYB3	1535	1546	1	1	0.9	cagTAGGTtca
OsSK12	P$MYB4_01	MYB4	1536	1544	1	1	0.937	agTAGGTt
OsSK12	P$RAV1_01	RAV1	1541	1553	1	1	0.958	gttCAACAtaat
OsSK12	P$ATHB6_01	ATHB6	1548	1557	1	1	0.913	atAATAAat
OsSK12	P$RAV1_02	RAV1	1561	1573	1	1	0.924	tttACCTGgtgc

OsSK12	P$ARR18_01	ARR18	1570	1583	1	1	0.905	tgcaAGATAtcct
OsSK12	P$SED_Q2	SED	1576	1586	1	1	0.999	atatCCTTTt
OsSK12	P$PBF_Q2_01	BF	1580	1586	1	1	1	CCTTTt
OsSK12	P$ATSPL8_01	ATSPL8	1580	1596	1	1	0.882	cctttTGTACcagcga
OsSK12	P$GATA15_01	GATA15	1607	1616	1	1	0.999	ccTGATCca
OsSK12	P$AT3G60580_01	AT3G60580	1635	1642	1	1	0.972	ctATCCC
OsSK12	P$HSFA2_01	HSFA2	1640	1646	1	1	0.933	CCAAAg
OsSK12	P$AT3G20750_01	AT3G20750	1650	1658	1	1	0.907	aTAAACaa
OsSK12	P$GATA9_01	GATA9	1667	1678	1	1	0.992	tccAGATCtag
OsSK12	P$AGP1_01	AGP1	1668	1678	1	1	0.997	ccAGATCtag
OsSK12	P$GATA10_01	GATA10	1669	1677	1	1	0.916	cAGATCta
OsSK12	P$GATA11_01	GATA11	1669	1677	1	1	0.939	caGATCTa
OsSK12	P$GATA8_01	GATA8	1669	1678	1	1	1	caGATCTag
OsSK12	P$ARR10_01	ARR10	1670	1677	1	1	0.913	AGATCta
OsSK12	P$SEP3_01	wrz-03	1681	1692	1	1	0.852	aatgtTTTTGg
OsSK12	P$ANTL_01	ANTL	1693	1703	1	1	0.985	tGTTACatct
OsSK12	P$GATA11_01	GATA11	1714	1722	1	1	0.878	agGATCTg
OsSK12	P$GATA8_01	GATA8	1714	1723	1	1	0.987	agGATCTga
OsSK12	P$ARR18_01	ARR18	1737	1750	1	1	0.94	agtaAGATAgggc
OsSK12	P$ABI3_01	ABI3	1746	1755	1	1	0.852	ggGCATGag
OsSK12	P$RAV1_01	RAV1	1752	1764	1	1	0.944	gagCAACAtcct
OsSK12	P$WRKY25_01	WRKY25	1771	1780	1	1	0.928	acaTAACCt
OsSK12	P$WRKY33_01	WRKY33	1771	1780	1	1	0.851	acaTAACCt
OsSK12	P$AT1G29860_01	AT1G29860	1771	1780	1	1	0.867	acaTAACCt
OsSK12	P$AT3G62340_01	AT3G62340	1771	1780	1	1	0.853	acaTAACCt
OsSK12	P$AT1G69310_01	AT1G69310	1771	1780	1	1	0.911	acaTAACCt
OsSK12	P$WRKY26_01	WRKY26	1771	1780	1	1	0.855	acaTAACCt
OsSK12	P$RAV1_02	RAV1	1773	1785	1	1	0.919	ataACCTGcgct
OsSK12	P$MYBAS1_01	MYBAS1	1789	1800	1	1	0.977	atCCAACtcgc
OsSK12	P$WRKY18_02	WRKY18	1823	1833	1	1	0.947	gctGTCAAca
OsSK12	P$WRKY21_02	WRKY21	1823	1833	1	1	0.982	gctGTCAAca
OsSK12	P$WRKY48_02	WRKY48	1823	1833	1	1	0.989	gctGTCAAca
OsSK12	P$WRKY57_01	WRKY57	1823	1833	1	1	0.972	gctGTCAAca
OsSK12	P$WRKY60_01	WRKY60	1823	1834	1	1	0.915	gctGTCAAcaa
OsSK12	P$WRKY15_01	WRKY15	1824	1834	1	1	0.976	ctGTCAAcaa
OsSK12	P$WRKY2_01	WRKY2	1824	1832	1	1	0.92	ctGTCAAc
OsSK12	P$WRKY25_02	WRKY25	1824	1832	1	1	0.919	ctGTCAAc
OsSK12	P$WRKY40_01	WRKY40	1824	1832	1	1	0.977	ctGTCAAc
OsSK12	P$WRKY43_02	WRKY43	1824	1834	1	1	0.962	ctGTCAAcaa
OsSK12	P$WRKY62_01	WRKY62	1824	1832	1	1	0.89	ctGTCAAc
OsSK12	P$WRKY63_01	WRKY63	1824	1832	1	1	0.896	ctGTCAAc
OsSK12	P$WRKY75_01	WRKY75	1824	1832	1	1	0.959	ctGTCAAc
OsSK12	P$WRKY8_01	WRKY8	1824	1833	1	1	0.986	ctGTCAAca
OsSK12	P$WRKY23_01	WRKY23	1825	1833	1	1	0.899	tGTCAAca
OsSK12	P$WRKY30_01	WRKY30	1825	1835	1	1	0.974	tGTCAAcaag
OsSK12	P$RAV1_01	RAV1	1825	1837	1	1	0.909	tgtCAACAagtt
OsSK12	P$WRKY18_Q2	WRKY18	1826	1835	1	1	0.918	GTCAAcaag
OsSK12	P$SED_Q2	SED	1837	1847	1	1	0.939	tcctCCTTTc
OsSK12	P$PBF_Q2_01	BF	1841	1847	1	1	0.985	CCTTTc
OsSK12	P$AT3G18650_01	AT3G18650	1848	1859	1	1	0.851	tatacTTGTAg
OsSK12	P$ATHB6_01	ATHB6	1859	1868	1	1	0.923	acAATAAac
OsSK12	P$AT3G20750_01	AT3G20750	1862	1870	1	1	0.938	aTAAACtt
OsSK12	P$BZR1_01	BZR1	1874	1880	1	1	0.902	CGTGCt
OsSK12	P$ATSPL8_01	ATSPL8	1874	1890	1	1	0.881	cgtgcTGTACagcagt
OsSK12	P$HSFA2_01	HSFA2	1892	1898	1	1	0.933	CCAAAg
OsSK12	P$HSFA4A_01	HSFA4A	1937	1943	1	1	0.914	tCTATT
OsSK12	P$AT3G62240_01	AT3G62240	1972	1982	1	1	0.911	acggcCATTC
OsSK12	P$ARR2_01	ARR2	1990	2000	1	1	0.858	atttATCTTc
OsSK12	P$WRKY11_Q2	WRKY11	1999	2007	1	1	0.925	cTTGACtg
OsSK12	P$AT4G04450_01	AT4G04450	2027	2036	1	1	0.865	aatTTAGCt
OsSK12	P$ATHSFA1D_01	ATHSFA1D	2033	2039	1	1	0.94	gCTACA
OsSK12	P$WRKY25_01	WRKY25	2048	2057	1	1	0.906	acaTAACCa
OsSK12	P$AT1G69310_01	AT1G69310	2048	2057	1	1	0.874	acaTAACCa
OsSK12	P$AT4G36620_01	AT4G36620	2049	2057	1	1	0.892	catAACCA
OsSK12	P$RAV1_02	RAV1	2053	2065	1	1	0.987	accACCTGctcc
OsSK12	P$LIM1_01	LIM1	2054	2066	1	1	0.858	CCACCtgctcca
OsSK12	P$DOF1_01	DOF1	2079	2090	1	1	0.985	cacTAAAGttc
OsSK12	P$ATHB6_01	ATHB6	2099	2108	1	1	1	tcAATAAtt
OsSK12	P$ATHB5_04	ATHB5	2099	2110	1	1	0.993	tcAATAAtttg
OsSK12	P$ATHB1_03	ATHB1	2099	2110	1	1	0.982	tcAATAAtttg
OsSK12	P$ATHB16_01	ATHB16	2100	2108	1	1	0.963	cAATAAtt
OsSK12	P$AT5G46010_01	AT5G46010	2113	2124	1	1	0.884	GTATGcatttc
OsSK12	P$LIM1_01	LIM1	2123	2135	1	1	0.928	CCACCatcaaag
OsSK12	P$RIN_Q2	RIN	2131	2142	1	1	0.855	aaagTTTAAga
OsSK12	P$REF6_01	REF6	2138	2149	1	1	0.884	aagaCAGAGaa
OsSK12	P$ARR2_01	ARR2	2179	2189	1	1	0.958	gtgtATCTTg
OsSK12	P$ATSPL8_01	ATSPL8	2182	2198	1	1	0.964	tatctTGTACttcttt
OsSK12	P$PEND_02	END	2194	2204	1	1	0.857	ctTTCTTctg

OsSK12	P$C1_Q2	C1	2222	2233	1	1	0.927	atAACTAgaac
OsSK12	P$GAMYB_Q2	GAMYB	2235	2248	1	1	0.922	caagaACAACaaa
OsSK12	P$RAV1_01	RAV1	2238	2250	1	1	0.948	gaaCAACAaaca
OsSK12	P$REF6_01	REF6	2244	2255	1	1	0.984	caaaCAGAGta
OsSK12	P$ARR18_01	ARR18	2251	2264	1	1	0.894	agtaAGATAaatt
OsSK12	P$AGL12_01	AGL12	2256	2268	1	1	0.869	gatAAATTtcag
OsSK12	P$BPC1_Q2	BPC1	2266	2272	1	1	0.997	AGAAAa
OsSK12	P$C1_Q2	C1	2268	2279	1	1	0.94	aaAACTAtaag
OsSK12	P$PBF_01	BF	2285	2296	1	1	0.958	tgcAAAAGgca
OsSK12	P$DOF_Q2	DOF	2285	2296	1	1	0.918	tgcAAAAGgca
OsSK12	P$CDF2_01	CDF2	2286	2296	1	1	0.945	gcAAAAGgca
OsSK12	P$CDF3_01	CDF3	2287	2296	1	1	0.969	cAAAAGgca
OsSK12	P$PBF_Q2	BF	2288	2294	1	1	1	aAAAGG
OsSK12	P$ATHB7_01	ATHB7	2293	2303	1	1	0.93	gcAATCAgtg
OsSK12	P$HAT1_01	HAT1	2293	2303	1	1	0.881	gcAATCAgtg
OsSK12	P$AT4G36620_01	AT4G36620	2300	2308	1	1	0.957	gtgAACCA
OsSK12	P$PEND_01	END	2316	2324	1	1	0.892	tAAGAAac
OsSK12	P$ATMYB77_01	ATMYB77	2317	2330	1	1	0.872	aagaaaCAGTTtc
OsSK12	P$BPC1_Q2	BPC1	2318	2324	1	1	0.99	AGAAAc
OsSK12	P$ATMYB77_01	ATMYB77	2333	2346	1	1	0.852	aacccaCAGTTtt
OsSK12	P$ASR1_01	ASR1	2334	2339	1	1	1	ACCCA
OsSK12	P$PEND_01	END	2352	2360	1	1	0.892	tAAGAAaa
OsSK12	P$BPC1_Q2	BPC1	2354	2360	1	1	0.997	AGAAAa
OsSK12	P$AT4G36620_01	AT4G36620	2355	2363	1	1	0.896	gaaAACCA
OsSK12	P$GAMYB_Q2	GAMYB	2367	2380	1	1	0.91	tttgcACAACaaa
OsSK12	P$RAV1_01	RAV1	2370	2382	1	1	0.952	gcaCAACAaatt
OsSK12	P$RAV1_01	RAV1	2396	2408	1	1	0.958	atgCAACAtgat
OsSK12	P$ARR18_01	ARR18	2406	2419	1	1	0.959	atgaAGATAtgaa
OsSK12	P$AZF3_01	AZF3	2417	2428	1	1	0.867	aAGTATatcat
OsSK12	P$HSFA2_01	HSFA2	2431	2437	1	1	0.933	CCAAAg
OsSK12	P$AZF3_01	AZF3	2434	2445	1	1	0.867	aAGTATatcat
OsSK12	P$RAV1_02	RAV1	2458	2470	1	1	0.985	cacACCTGcagc
OsSK12	P$O2_Q4	O2	2505	2516	1	1	0.873	aataCATGTtg
OsSK12	P$ARR1_01	ARR1	2527	2537	1	1	0.942	actGAATCga
OsSK12	P$NAC92_01	NAC92	2539	2551	1	1	0.957	agACACGgaaac
OsSK12	P$ATMYB15_01	ATMYB15	2565	2578	1	1	0.878	ttgtGGTAGgttg
OsSK12	P$MYB3_01	MYB3	2568	2579	1	1	0.971	tggTAGGTtgc
OsSK12	P$MYB4_01	MYB4	2569	2577	1	1	0.976	ggTAGGTt
OsSK12	P$HSFA2_01	HSFA2	2590	2596	1	1	0.933	CCAAAg
OsSK12	P$ARR18_01	ARR18	2598	2611	1	1	0.909	aatgAGATAtatg
OsSK12	P$O2_Q4	O2	2607	2618	1	1	0.92	tatgCATGTaa
OsSK12	P$ABI3_01	ABI3	2608	2617	1	1	0.866	atGCATGta
OsSK12	P$HSFA2_01	HSFA2	2629	2635	1	1	0.933	CCAAAg
OsSK12	P$CBNAC_01	CBNAC	2659	2665	1	1	0.968	aTGCTT
OsSK12	P$PIL5_01	IL5	2675	2689	1	1	0.862	ttcctgtaACGTGt
OsSK12	P$ABF4_Q2	ABF4	2677	2691	1	1	0.866	cctgtaACGTGtga
OsSK12	P$ABZ1_01	ABZ1	2678	2692	1	1	0.9	ctgtaACGTGtgag
OsSK12	P$HBP1A_Q2	HBP1A	2680	2690	1	1	0.877	gtaACGTGtg
OsSK12	P$TAF1_Q2	TAF1	2680	2690	1	1	0.926	gtaACGTGtg
OsSK12	P$EMBP1_02	EMBP1	2680	2690	1	1	0.86	gtaACGTGtg
OsSK12	P$TAF1_01	TAF1	2680	2690	1	1	0.953	gtaACGTGtg
OsSK12	P$GBF1_01	GBF1	2681	2689	1	1	0.978	taACGTGt
OsSK12	P$BIM1_02	BIM1	2681	2691	1	1	0.947	taACGTGtga
OsSK12	P$ABF4_02	ABF4	2681	2691	1	1	0.985	taACGTGtga
OsSK12	P$ABI5_Q2	ABI5	2683	2689	1	1	0.979	ACGTGt
OsSK12	P$ARR2_01	ARR2	2698	2708	1	1	0.953	ctggATCTTg
OsSK12	P$GATA11_01	GATA11	2699	2707	1	1	0.867	tgGATCTt
OsSK12	P$GATA8_01	GATA8	2699	2708	1	1	0.977	tgGATCTTg
OsSK12	P$MYB1L_01	MYB1L	2721	2731	1	1	0.984	caCCCTAata
OsSK12	P$TRB2_01	TRB2	2721	2729	1	1	0.965	caCCCTAa
OsSK12	P$O2_Q4	O2	2743	2754	1	1	0.871	ttagCATGTat
OsSK12	P$ABI3_01	ABI3	2744	2753	1	1	0.865	taGCATGta
OsSK12	P$C1_Q2	C1	2752	2763	1	1	0.947	atAACTAtcaa
OsSK12	P$C1_Q2	C1	2763	2774	1	1	0.933	ttAACTAgact
OsSK12	P$DOF1_01	DOF1	2770	2781	1	1	0.983	gacTAAAGtac
OsSK12	P$GAMYB_Q2	GAMYB	2774	2787	1	1	0.855	aaagtACAACaag
OsSK12	P$RAV1_01	RAV1	2777	2789	1	1	0.924	gtaCAACAagat
OsSK12	P$SBF1_01	SBF1	2786	2800	1	1	0.852	gatgggTTAAAcaa
OsSK12	P$WRKY48_01	WRKY48	2791	2800	1	1	0.876	gttaAACAA
OsSK12	P$AT3G20750_01	AT3G20750	2792	2800	1	1	0.876	tTAAACaa
OsSK12	P$AZF3_01	AZF3	2805	2816	1	1	0.882	gAGTATgtagt
OsSK12	P$TEIL_01	TEIL	2809	2817	1	1	0.883	ATGTAgtt
OsSK12	P$AT3G18650_01	AT3G18650	2829	2840	1	1	0.851	ggaaaTTGTAt
OsSK12	P$WRKY18_02	WRKY18	2874	2884	1	1	0.946	agtGTCAAct
OsSK12	P$WRKY21_02	WRKY21	2874	2884	1	1	0.983	agtGTCAAct
OsSK12	P$WRKY48_02	WRKY48	2874	2884	1	1	0.989	agtGTCAAct
OsSK12	P$WRKY57_01	WRKY57	2874	2884	1	1	0.974	agtGTCAAct
OsSK12	P$WRKY60_01	WRKY60	2874	2885	1	1	0.908	agtGTCAActg

OsSK12	P$WRKY15_01	WRKY15	2875	2885	1	1	0.976	gtGTCAActg
OsSK12	P$WRKY2_01	WRKY2	2875	2883	1	1	0.919	gtGTCAAc
OsSK12	P$WRKY25_02	WRKY25	2875	2883	1	1	0.906	gtGTCAAc
OsSK12	P$WRKY40_01	WRKY40	2875	2883	1	1	0.977	gtGTCAAc
OsSK12	P$WRKY43_02	WRKY43	2875	2885	1	1	0.96	gtGTCAActg
OsSK12	P$WRKY62_01	WRKY62	2875	2883	1	1	0.881	gtGTCAAc
OsSK12	P$WRKY63_01	WRKY63	2875	2883	1	1	0.894	gtGTCAAc
OsSK12	P$WRKY75_01	WRKY75	2875	2883	1	1	0.958	gtGTCAAc
OsSK12	P$WRKY8_01	WRKY8	2875	2884	1	1	0.986	gtGTCAAct
OsSK12	P$WRKY23_01	WRKY23	2876	2884	1	1	0.889	tGTCAAct
OsSK12	P$WRKY30_01	WRKY30	2876	2886	1	1	0.975	tGTCAActgt
OsSK12	P$WRKY18_Q2	WRKY18	2877	2886	1	1	0.949	GTCAActgt
OsSK12	P$WRKY18_02	WRKY18	2881	2891	1	1	0.949	actGTCAAcg
OsSK12	P$WRKY21_02	WRKY21	2881	2891	1	1	0.986	actGTCAAcg
OsSK12	P$WRKY48_02	WRKY48	2881	2891	1	1	0.989	actGTCAAcg
OsSK12	P$WRKY57_01	WRKY57	2881	2891	1	1	0.976	actGTCAAcg
OsSK12	P$WRKY60_01	WRKY60	2881	2892	1	1	0.92	actGTCAAcgt
OsSK12	P$WRKY15_01	WRKY15	2882	2892	1	1	0.979	ctGTCAAcgt
OsSK12	P$WRKY2_01	WRKY2	2882	2890	1	1	0.92	ctGTCAAc
OsSK12	P$WRKY25_02	WRKY25	2882	2890	1	1	0.919	ctGTCAAc
OsSK12	P$WRKY40_01	WRKY40	2882	2890	1	1	0.977	ctGTCAAc
OsSK12	P$WRKY43_02	WRKY43	2882	2892	1	1	0.962	ctGTCAAcgt
OsSK12	P$WRKY62_01	WRKY62	2882	2890	1	1	0.89	ctGTCAAc
OsSK12	P$WRKY63_01	WRKY63	2882	2890	1	1	0.896	ctGTCAAc
OsSK12	P$WRKY75_01	WRKY75	2882	2890	1	1	0.959	ctGTCAAc
OsSK12	P$WRKY8_01	WRKY8	2882	2891	1	1	0.988	ctGTCAAcg
OsSK12	P$WRKY23_01	WRKY23	2883	2891	1	1	0.938	tGTCAAcg
OsSK12	P$WRKY30_01	WRKY30	2883	2893	1	1	0.982	tGTCAAcgtg
OsSK12	P$ABZ1_01	ABZ1	2883	2897	1	1	0.898	tgtcaACGTGccgt
OsSK12	P$WRKY18_Q2	WRKY18	2884	2893	1	1	0.957	GTCAAcgtg
OsSK12	P$GBF1_Q2_01	GBF1	2884	2895	1	1	0.92	gtcaACGTGcc
OsSK12	P$AT5G54070_01	AT5G54070	2885	2891	1	1	0.91	tCAACG
OsSK12	P$HBP1A_Q2	HBP1A	2885	2895	1	1	0.903	tcaACGTGcc
OsSK12	P$TAF1_Q2	TAF1	2885	2895	1	1	0.926	tcaACGTGcc
OsSK12	P$TAF1_01	TAF1	2885	2895	1	1	0.949	tcaACGTGcc
OsSK12	P$HY5_01	HY5	2886	2896	1	1	0.856	caACGTGccg
OsSK12	P$GBF1_01	GBF1	2886	2894	1	1	0.908	caACGTGc
OsSK12	P$MYC4_01	MYC4	2886	2894	1	1	0.86	caACGTGc
OsSK12	P$BIM1_02	BIM1	2886	2896	1	1	0.95	caACGTGccg
OsSK12	P$ABF4_02	ABF4	2886	2896	1	1	0.957	caACGTGccg
OsSK12	P$OSBZ8_Q6	OSBZ8	2888	2902	1	1	0.893	ACGTGccgtgaaac
OsSK12	P$ABI5_Q2	ABI5	2888	2894	1	1	0.936	ACGTGc
OsSK12	P$BZR1_01	BZR1	2889	2895	1	1	0.897	CGTGCc
OsSK12	P$JERF1_01	JERF1	2890	2900	1	1	0.864	gTGCCGtgaa
OsSK12	P$C1_Q2	C1	2897	2908	1	1	0.924	gaAACTAtgat
OsSK12	P$SPF1_Q2	SPF1	2904	2914	1	1	0.883	tgATAGTtat
OsSK12	P$WRKY11_01	WRKY11	2920	2934	1	1	0.896	actcTTGACtttga
OsSK12	P$WRKY11_Q2	WRKY11	2923	2931	1	1	0.929	cTTGACtt
OsSK12	P$SBF1_01	SBF1	2938	2952	1	1	0.927	gtttggTTAAAagc
OsSK12	P$PBF_01	BF	2943	2954	1	1	0.966	gttAAAAGcct
OsSK12	P$DOF_Q2	DOF	2943	2954	1	1	0.927	gttAAAAGcct
OsSK12	P$DOF2_01	DOF2	2943	2954	1	1	0.988	gttaAAAGCct
OsSK12	P$DOF3_01	DOF3	2943	2954	1	1	0.985	gttaAAAGCct
OsSK12	P$CDF2_01	CDF2	2944	2954	1	1	0.954	ttAAAAGcct
OsSK12	P$CDF3_01	CDF3	2945	2954	1	1	0.974	tAAAAGcct
OsSK12	P$EDF3_02	EDF3	2955	2964	1	1	1	tGACCGagg
OsSK12	P$RAV2_01	RAV2	2955	2964	1	1	1	tgACCGAgg
OsSK12	P$SBF1_01	SBF1	2960	2974	1	1	0.895	gaggaaTTAAAaat
OsSK12	P$PEND_01	END	2988	2996	1	1	0.901	gAAGAAgt
OsSK12	P$HSFA2_01	HSFA2	3000	3006	1	1	0.933	CCAAAg
OsSK12	P$SBF1_01	SBF1	3004	3018	1	1	0.89	agttcaTTAAAaaa
OsSK12	P$AMS_01	AMS	3037	3047	1	1	0.867	tcCATGTgca
OsSK12	P$ATHB6_01	ATHB6	3055	3064	1	1	0.923	gcAATAAac
OsSK12	P$AT3G20750_01	AT3G20750	3058	3066	1	1	0.938	aTAAACtt
OsSK12	P$ERF38_02	ERF38	3070	3080	1	1	0.866	ttatCGCCCg
OsSK12	P$CBNAC_01	CBNAC	3085	3091	1	1	0.973	cTGCTT
OsSK12	P$CBNAC_02	CBNAC	3085	3101	1	1	0.88	cTGCTTagttcctctg
OsSK12	P$EDF3_02	EDF3	3099	3108	1	1	1	tGACCGagg
OsSK12	P$RAV2_01	RAV2	3099	3108	1	1	1	tgACCGAgg
OsSK12	P$SBF1_01	SBF1	3104	3118	1	1	0.895	gaggaaTTAAAatt
OsSK12	P$PEND_01	END	3132	3140	1	1	0.901	gAAGAAgt
OsSK12	P$HSFA2_01	HSFA2	3144	3150	1	1	0.933	CCAAAg
OsSK12	P$SBF1_01	SBF1	3148	3162	1	1	0.89	agttcaTTAAAaaa
OsSK12	P$AMS_01	AMS	3182	3192	1	1	0.867	tcCATGTgca
OsSK12	P$BPC1_Q2	BPC1	3191	3197	1	1	0.997	AGAAAa
OsSK12	P$ATHB6_01	ATHB6	3200	3209	1	1	0.923	gcAATAAac
OsSK12	P$AT3G20750_01	AT3G20750	3203	3211	1	1	0.938	aTAAACtt
OsSK12	P$CBNAC_01	CBNAC	3230	3236	1	1	0.973	cTGCTT

OsSK12	P$CBNAC_02	CBNAC	3230	3246	1	1	0.88	cTGCTTagttccgtat
OsSK12	P$CBNAC_01	CBNAC	3244	3250	1	1	0.968	aTGCTT
OsSK12	P$OSRR22_01	OSRR22	3261	3271	1	1	0.882	agGATACgga
OsSK12	P$AMS_01	AMS	3270	3280	1	1	0.899	atCATGTgtt
OsSK12	P$C1_Q2	C1	3291	3302	1	1	0.938	ggAACTAacaa
OsSK12	P$WEREWOLF_Q2_01	WEREWOLF	3293	3302	1	1	0.991	aACTAAcaa
OsSK12	P$MYBAS1_01	MYBAS1	3293	3304	1	1	0.939	aaCTAACaaag
OsSK12	P$ATMYB15_Q2	ATMYB15	3296	3302	1	1	1	TAACAa
OsSK12	P$ARR1_01	ARR1	3300	3310	1	1	0.948	aaaGAATCgg
OsSK12	P$PBF_Q2	BF	3334	3340	1	1	0.958	cAAAGG
OsSK12	P$WRKY18_02	WRKY18	3336	3346	1	1	0.998	aagGTCAAca
OsSK12	P$WRKY21_02	WRKY21	3336	3346	1	1	0.995	aagGTCAAca
OsSK12	P$WRKY48_02	WRKY48	3336	3346	1	1	1	aagGTCAAca
OsSK12	P$WRKY57_01	WRKY57	3336	3346	1	1	0.988	aagGTCAAca
OsSK12	P$WRKY60_01	WRKY60	3336	3347	1	1	0.988	aagGTCAAcaa
OsSK12	P$WRKY15_01	WRKY15	3337	3347	1	1	0.997	agGTCAAcaa
OsSK12	P$WRKY2_01	WRKY2	3337	3345	1	1	0.997	agGTCAAc
OsSK12	P$WRKY25_02	WRKY25	3337	3345	1	1	0.985	agGTCAAc
OsSK12	P$WRKY40_01	WRKY40	3337	3345	1	1	1	agGTCAAc
OsSK12	P$WRKY43_02	WRKY43	3337	3347	1	1	0.987	agGTCAAcaa
OsSK12	P$WRKY62_01	WRKY62	3337	3345	1	1	0.952	agGTCAAc
OsSK12	P$WRKY63_01	WRKY63	3337	3345	1	1	0.999	agGTCAAc
OsSK12	P$WRKY75_01	WRKY75	3337	3345	1	1	0.999	agGTCAAc
OsSK12	P$WRKY8_01	WRKY8	3337	3346	1	1	0.999	agGTCAAca
OsSK12	P$WRKY23_01	WRKY23	3338	3346	1	1	0.925	gGTCAAca
OsSK12	P$WRKY30_01	WRKY30	3338	3348	1	1	0.992	gGTCAAcaac
OsSK12	P$RAV1_01	RAV1	3338	3350	1	1	0.905	ggtCAACAacgc
OsSK12	P$GAMYB_Q2	GAMYB	3338	3351	1	1	0.938	ggtcaACAACgct
OsSK12	P$WRKY18_Q2	WRKY18	3339	3348	1	1	0.918	GTCAAcaac
OsSK12	P$AT5G54070_01	AT5G54070	3343	3349	1	1	0.958	aCAACG
OsSK12	P$ATHSFA1D_01	ATHSFA1D	3348	3354	1	1	0.94	gCTACA
OsSK12	P$AT3G20750_01	AT3G20750	3360	3368	1	1	0.885	cTAAACgt
OsSK12	P$PHV_02	HV	3382	3397	1	1	0.864	ttcATCATttggtgt
OsSK12	P$ATHB4_02	ATHB4	3383	3393	1	1	0.878	tcATCATttg
OsSK12	P$ATMYB77_01	ATMYB77	3402	3415	1	1	0.887	ggatggCGGTTct
OsSK12	P$E2L_Q2	E2L	3405	3412	1	1	0.892	tGGCGGt
OsSK12	P$ERF1_Q2	ERF1	3406	3414	1	1	0.91	GGCGGttc
OsSK12	P$ARR1_01	ARR1	3429	3439	1	1	0.954	ataGAATCca
OsSK12	P$ASR1_01	ASR1	3441	3446	1	1	1	ACCCA
OsSK12	P$HSFA2_01	HSFA2	3443	3449	1	1	1	CCAAAa
OsSK12	P$PEND_01	END	3456	3464	1	1	0.956	tAAGAAct
OsSK12	P$HSFA2_01	HSFA2	3464	3470	1	1	0.933	CCAAAg
OsSK12	P$PDF2_01	DF2	3466	3477	1	1	0.878	aaagTAAATga
OsSK12	P$HAHB4_01	HAHB4	3471	3480	1	1	0.86	aAATGAtta
OsSK12	P$GATA9_01	GATA9	3478	3489	1	1	0.978	tagAGATCtag
OsSK12	P$AGP1_01	AGP1	3479	3489	1	1	0.988	agAGATCtag
OsSK12	P$GATA10_01	GATA10	3480	3488	1	1	0.92	gAGATCta
OsSK12	P$GATA11_01	GATA11	3480	3488	1	1	0.944	gaGATCTa
OsSK12	P$GATA8_01	GATA8	3480	3489	1	1	1	gaGATCTag
OsSK12	P$ARR10_01	ARR10	3481	3488	1	1	0.913	AGATCta
OsSK12	P$REF6_01	REF6	3489	3500	1	1	0.873	gaagCAGAGaa
OsSK12	P$DOF1_01	DOF1	3503	3514	1	1	0.974	ctgTAAAGaaa
OsSK12	P$GT1_Q6	GT1	3505	3512	1	1	0.912	GTAAAga
OsSK12	P$PI_02	I	3508	3522	1	1	0.899	aagaaaacGGAAAa
OsSK12	P$BPC1_Q2	BPC1	3509	3515	1	1	0.997	AGAAAa
OsSK12	P$AT3G51080_01	AT3G51080	3516	3523	1	1	1	GGAAAaa
OsSK12	P$AP1_01	AP1	3516	3529	1	1	0.854	ggAAAAAaagtaa
OsSK12	P$PBF_01	BF	3518	3529	1	1	0.979	aaaAAAAGtaa
OsSK12	P$DOF_Q2	DOF	3518	3529	1	1	1	aaaAAAAGtaa
OsSK12	P$CDF2_01	CDF2	3519	3529	1	1	0.998	aaAAAAGtaa
OsSK12	P$CDF3_01	CDF3	3520	3529	1	1	0.991	aAAAAGtaa
OsSK12	P$AT3G18650_01	AT3G18650	3525	3536	1	1	0.851	gtaaaTTGTAc
OsSK12	P$ATSPL8_01	ATSPL8	3526	3542	1	1	0.934	taaatTGTACatgtac
OsSK12	P$ATSPL8_01	ATSPL8	3532	3548	1	1	0.89	gtacaTGTACacgaga
OsSK12	P$NAC92_01	NAC92	3538	3550	1	1	0.944	gtACACGagaaa
OsSK12	P$BPC1_Q2	BPC1	3545	3551	1	1	0.99	AGAAAc
OsSK12	P$C1_Q2	C1	3546	3557	1	1	0.93	gaAACTAgaag
OsSK12	P$BHLH112_01	BHLH112	3583	3592	1	1	0.935	gcaACTTGc
OsSK12	P$ABI3_01	ABI3	3588	3597	1	1	0.98	ttGCATGca
OsSK12	P$FUS3_01	FUS3	3589	3598	1	1	0.966	tGCATGcag
OsSK12	P$LEC2_01	LEC2	3589	3600	1	1	0.98	tgCATGCagcg
OsSK12	P$FUS3_Q2	FUS3	3590	3601	1	1	0.851	gCATGCagcga
OsSK12	P$RAV1_02	RAV1	3612	3624	1	1	0.984	ctcACCTGcttg
OsSK12	P$AT1G53910_02	AT1G53910	3615	3636	1	1	0.85	acctgcttgccGCGGCgtggt
OsSK12	P$CBNAC_01	CBNAC	3617	3623	1	1	0.973	cTGCTT
OsSK12	P$RRTF1_05	RRTF1	3620	3635	1	1	0.858	cttgccgCGGCGtgg
OsSK12	P$CBF1_01	CBF1	3621	3631	1	1	0.919	tTGCCGcggc
OsSK12	P$ERF019_01	ERF019	3621	3631	1	1	0.864	tTGCCGcggc

OsSK12	P$JERF3_01	JERF3	3621	3631	1	1	0.881	tTGCCGcggc
OsSK12	P$CEF1_01	CEF1	3621	3631	1	1	0.879	tTGCCGcggc
OsSK12	P$JERF1_01	JERF1	3621	3631	1	1	0.903	tTGCCGcggc
OsSK12	P$CBF1_03	CBF1	3621	3631	1	1	0.937	tTGCCGcggc
OsSK12	P$DREB1F_01	DREB1F	3621	3631	1	1	0.867	tTGCCGcggc
OsSK12	P$AT1G33760_01	AT1G33760	3621	3631	1	1	0.882	tTGCCGcggc
OsSK12	P$AT4G28140_01	AT4G28140	3621	3631	1	1	0.884	tTGCCGcggc
OsSK12	P$ORA47_01	ORA47	3621	3631	1	1	0.889	tTGCCGcggc
OsSK12	P$ABI4_03	ABI4	3621	3631	1	1	0.875	ttGCCGCggc
OsSK12	P$DREBI5_01	DREBI5	3621	3631	1	1	0.917	ttGCCGCggc
OsSK12	P$AT1G77200_01	AT1G77200	3621	3631	1	1	0.867	ttGCCGCggc
OsSK12	P$ERF1_02	ERF1	3621	3631	1	1	0.859	ttGCCGCggc
OsSK12	P$EREBP1_01	EREBP1	3621	3631	1	1	0.858	ttGCCGCggc
OsSK12	P$CBF3_01	CBF3	3621	3631	1	1	0.883	ttGCCGCggc
OsSK12	P$DREBII1_01	DREBII1	3621	3631	1	1	0.915	ttGCCGCggc
OsSK12	P$ERF2_03	ERF2	3621	3631	1	1	0.852	ttGCCGCggc
OsSK12	P$DBF2_01	DBF2	3621	3631	1	1	0.858	ttGCCGCggc
OsSK12	P$CBF5_01	CBF5	3621	3631	1	1	0.915	ttGCCGCggc
OsSK12	P$CBF16_01	CBF16	3621	3631	1	1	0.915	ttGCCGCggc
OsSK12	P$CBF17_01	CBF17	3621	3631	1	1	0.916	ttGCCGCggc
OsSK12	P$CBF_01	CBF	3621	3631	1	1	0.917	ttGCCGCggc
OsSK12	P$ERF016_01	ERF016	3621	3631	1	1	0.907	ttGCCGCggc
OsSK12	P$TINY2_02	TINY2	3621	3631	1	1	0.862	ttGCCGCggc
OsSK12	P$AT3G16280_01	AT3G16280	3621	3631	1	1	0.863	ttGCCGCggc
OsSK12	P$DREB1A_03	DREB1A	3621	3631	1	1	0.882	ttGCCGCggc
OsSK12	P$CRF2_01	CRF2	3621	3629	1	1	0.872	ttGCCGCg
OsSK12	P$ERF098_01	ERF098	3621	3629	1	1	0.892	ttGCCGCg
OsSK12	P$ERF7_02	ERF7	3622	3632	1	1	0.931	tGCCGCggcg
OsSK12	P$AT1G28160_02	AT1G28160	3624	3639	1	1	0.858	ccgCGGCGtggttgc
OsSK12	P$AT1G68550_03	AT1G68550	3624	3633	1	1	0.989	ccgCGGCGt
OsSK12	P$BZIP68_01	BZIP68	3627	3636	1	1	0.927	cggCGTGGt
OsSK12	P$CBF1_01	CBF1	3635	3645	1	1	0.992	tTGCCGccgg
OsSK12	P$ERF019_01	ERF019	3635	3645	1	1	0.903	tTGCCGccgg
OsSK12	P$DREB6_01	DREB6	3635	3645	1	1	0.957	tTGCCGccgg
OsSK12	P$JERF3_01	JERF3	3635	3645	1	1	0.926	tTGCCGccgg
OsSK12	P$DREB1_01	DREB1	3635	3645	1	1	0.957	tTGCCGccgg
OsSK12	P$CEF1_01	CEF1	3635	3645	1	1	0.926	tTGCCGccgg
OsSK12	P$JERF1_01	JERF1	3635	3645	1	1	0.958	tTGCCGccgg
OsSK12	P$CBF1_03	CBF1	3635	3645	1	1	0.985	tTGCCGccgg
OsSK12	P$AT1G71450_01	AT1G71450	3635	3645	1	1	0.885	tTGCCGccgg
OsSK12	P$DREB1F_01	DREB1F	3635	3645	1	1	0.996	tTGCCGccgg
OsSK12	P$AT1G33760_01	AT1G33760	3635	3645	1	1	0.932	tTGCCGccgg
OsSK12	P$AT1G71520_01	AT1G71520	3635	3645	1	1	0.868	tTGCCGccgg
OsSK12	P$AT4G28140_01	AT4G28140	3635	3645	1	1	0.888	tTGCCGccgg
OsSK12	P$DREB1E_02	DREB1E	3635	3645	1	1	0.95	tTGCCGccgg
OsSK12	P$ORA47_01	ORA47	3635	3645	1	1	0.905	tTGCCGccgg
OsSK12	P$DREB2F_01	DREB2F	3635	3645	1	1	0.908	tTGCCGccgg
OsSK12	P$ERF13_01	ERF13	3635	3645	1	1	0.866	ttGCCGCcgg
OsSK12	P$ABI4_03	ABI4	3635	3645	1	1	0.964	ttGCCGCcgg
OsSK12	P$WRAF1_01	WRAF1	3635	3645	1	1	0.879	ttGCCGCcgg
OsSK12	P$PTI5_01	TI5	3635	3645	1	1	0.927	ttGCCGCcgg
OsSK12	P$DREBI5_01	DREBI5	3635	3645	1	1	0.982	ttGCCGCcgg
OsSK12	P$AT2G47520_01	AT2G47520	3635	3645	1	1	0.949	ttGCCGCcgg
OsSK12	P$DREB2B_01	DREB2B	3635	3645	1	1	0.957	ttGCCGCcgg
OsSK12	P$CRF1_02	CRF1	3635	3645	1	1	0.914	ttGCCGCcgg
OsSK12	P$AT1G77200_01	AT1G77200	3635	3645	1	1	0.861	ttGCCGCcgg
OsSK12	P$OPBP1_01	OPBP1	3635	3645	1	1	0.901	ttGCCGCcgg
OsSK12	P$ATERF14_01	ATERF14	3635	3645	1	1	0.908	ttGCCGCcgg
OsSK12	P$DREB2A_02	DREB2A	3635	3645	1	1	0.928	ttGCCGCcgg
OsSK12	P$ERF1_02	ERF1	3635	3645	1	1	0.939	ttGCCGCcgg
OsSK12	P$ERF4_02	ERF4	3635	3645	1	1	0.939	ttGCCGCcgg
OsSK12	P$AT5G25390_01	AT5G25390	3635	3645	1	1	0.913	ttGCCGCcgg
OsSK12	P$EREBP1_01	EREBP1	3635	3645	1	1	0.936	ttGCCGCcgg
OsSK12	P$CBF3_01	CBF3	3635	3645	1	1	0.983	ttGCCGCcgg
OsSK12	P$DREBII1_01	DREBII1	3635	3645	1	1	0.983	ttGCCGCcgg
OsSK12	P$TSRF1_01	TSRF1	3635	3645	1	1	0.89	ttGCCGCcgg
OsSK12	P$DRF13_01	DRF13	3635	3645	1	1	0.968	ttGCCGCcgg
OsSK12	P$ERF2_03	ERF2	3635	3645	1	1	0.938	ttGCCGCcgg
OsSK12	P$ERF1B_03	ERF1B	3635	3645	1	1	0.897	ttGCCGCcgg
OsSK12	P$RAP26_02	RAP26	3635	3645	1	1	0.87	ttGCCGCcgg
OsSK12	P$CBF5_01	CBF5	3635	3645	1	1	0.98	ttGCCGCcgg
OsSK12	P$CBF16_01	CBF16	3635	3645	1	1	0.982	ttGCCGCcgg
OsSK12	P$CBF17_01	CBF17	3635	3645	1	1	0.984	ttGCCGCcgg
OsSK12	P$ERF1_05	ERF1	3635	3645	1	1	0.907	ttGCCGCcgg
OsSK12	P$AT5G25190_01	AT5G25190	3635	3645	1	1	0.916	ttGCCGCcgg
OsSK12	P$ERF105_01	ERF105	3635	3645	1	1	0.925	ttGCCGCcgg
OsSK12	P$CBF_01	CBF	3635	3645	1	1	0.982	ttGCCGCcgg
OsSK12	P$AT5G11190_01	AT5G11190	3635	3645	1	1	0.916	ttGCCGCcgg

OsSK12	P$AT1G68550_01	AT1G68550	3635	3645	1	1	0.997	ttGCCGCcgg
OsSK12	P$AT1G77640_01	AT1G77640	3635	3645	1	1	0.894	ttGCCGCcgg
OsSK12	P$ERF016_01	ERF016	3635	3645	1	1	1	ttGCCGCcgg
OsSK12	P$AT3G61630_01	AT3G61630	3635	3645	1	1	0.965	ttGCCGCcgg
OsSK12	P$AT5G43410_01	AT5G43410	3635	3645	1	1	0.916	ttGCCGCcgg
OsSK12	P$TINY2_02	TINY2	3635	3645	1	1	0.85	ttGCCGCcgg
OsSK12	P$AT5G07310_01	AT5G07310	3635	3645	1	1	0.932	ttGCCGCcgg
OsSK12	P$AT3G16280_01	AT3G16280	3635	3645	1	1	0.864	ttGCCGCcgg
OsSK12	P$DREB1A_03	DREB1A	3635	3645	1	1	0.983	ttGCCGCcgg
OsSK12	P$AT1G49120_01	AT1G49120	3635	3645	1	1	0.871	ttGCCGCcgg
OsSK12	P$DREB2D_01	DREB2D	3635	3645	1	1	0.97	ttGCCGCcgg
OsSK12	P$AT3G25890_01	AT3G25890	3635	3645	1	1	0.985	ttGCCGCcgg
OsSK12	P$AT4G23750_01	AT4G23750	3635	3645	1	1	0.891	ttGCCGCcgg
OsSK12	P$AT4G27950_01	AT4G27950	3635	3645	1	1	0.903	ttGCCGCcgg
OsSK12	P$RRTF1_01	RRTF1	3635	3645	1	1	0.923	ttGCCGCcgg
OsSK12	P$CEJ1_02	CEJ1	3635	3645	1	1	0.875	ttGCCGCcgg
OsSK12	P$ERF1_Q2_01	ERF1	3635	3649	1	1	0.876	ttGCCGCcgggaaa
OsSK12	P$CRF2_01	CRF2	3635	3643	1	1	0.921	ttGCCGCc
OsSK12	P$ERF096_01	ERF096	3635	3645	1	1	0.983	ttGCCGCcgg
OsSK12	P$ERF098_01	ERF098	3635	3643	1	1	0.996	ttGCCGCc
OsSK12	P$DREB2C_01	DREB2C	3635	3645	1	1	0.959	ttgCCGCCgg
OsSK12	P$CBF1_02	CBF1	3635	3645	1	1	0.89	ttgCCGCCgg
OsSK12	P$CBF2_03	CBF2	3635	3645	1	1	0.88	ttgcCGCCGg
OsSK12	P$ERF1B_06	ERF1B	3636	3644	1	1	0.946	tGCCGCcg
OsSK12	P$ERF7_02	ERF7	3636	3646	1	1	0.984	tGCCGCcggg
OsSK12	P$ERF094_01	ERF094	3636	3644	1	1	0.979	tGCCGCcg
OsSK12	P$ERF2_01	ERF2	3636	3643	1	1	0.929	tgCCGCC
OsSK12	P$ERF13_02	ERF13	3636	3644	1	1	0.978	tgCCGCCg
OsSK12	P$ERF112_02	ERF112	3637	3647	1	1	0.925	gcCGCCGgga
OsSK12	P$E2L_Q2_01	E2L	3637	3651	1	1	0.938	gccgcCGGGAaaaa
OsSK12	P$AT3G63350_01	AT3G63350	3638	3644	1	1	0.866	CCGCCg
OsSK12	P$CRF4_01	CRF4	3638	3646	1	1	0.863	cCGCCGgg
OsSK12	P$ERF4_04	ERF4	3638	3646	1	1	0.869	cCGCCGgg
OsSK12	P$ERF069_01	ERF069	3638	3647	1	1	0.99	cCGCCGgga
OsSK12	P$ERF11_01	ERF11	3638	3648	1	1	0.966	cCGCCGggaa
OsSK12	P$ERF8_01	ERF8	3639	3649	1	1	0.941	CGCCGggaaa
OsSK12	P$AT3G51080_01	AT3G51080	3644	3651	1	1	1	GGAAAaa
OsSK12	P$PBF_01	BF	3644	3655	1	1	0.974	ggaAAAAGcgg
OsSK12	P$DOF_Q2	DOF	3644	3655	1	1	0.979	ggaAAAAGcgg
OsSK12	P$DOF2_01	DOF2	3644	3655	1	1	0.989	ggaaAAAGCgg
OsSK12	P$DOF3_01	DOF3	3644	3655	1	1	0.988	ggaaAAAGCgg
OsSK12	P$CDF2_01	CDF2	3645	3655	1	1	0.989	gaAAAAGcgg
OsSK12	P$CDF3_01	CDF3	3646	3655	1	1	0.991	aAAAAGcgg
OsSK12	P$GATA9_01	GATA9	3652	3663	1	1	0.982	cggAGATCtgc
OsSK12	P$AGP1_01	AGP1	3653	3663	1	1	0.94	ggAGATCtgc
OsSK12	P$GATA10_01	GATA10	3654	3662	1	1	0.966	gAGATCtg
OsSK12	P$GATA11_01	GATA11	3654	3662	1	1	0.98	gaGATCTg
OsSK12	P$GATA8_01	GATA8	3654	3663	1	1	0.998	gaGATCTgc
OsSK12	P$ARR10_01	ARR10	3655	3662	1	1	0.978	AGATCtg
OsSK12	P$ALFIN1_Q2	ALFIN1	3657	3672	1	1	0.876	atctgcGTGGGtttg
OsSK12	P$BZIP68_01	BZIP68	3659	3668	1	1	0.929	ctgCGTGGg
OsSK12	P$BPC1_Q2	BPC1	3673	3679	1	1	0.99	AGAAAt
OsSK12	P$FAR1_01	FAR1	3679	3694	1	1	0.881	atgcgACGCGctgca
OsSK12	P$FHY3_01	FHY3	3681	3693	1	1	0.89	gcgACGCGctgc
OsSK12	P$TRAB1_Q2	TRAB1	3682	3693	1	1	0.896	cgACGCGctgc
OsSK12	P$TSAR2_01	TSAR2	3701	3711	1	1	0.93	cGCACGggcg
OsSK12	P$BHLH78_01	BHLH78	3702	3710	1	1	0.875	GCACGggc
OsSK12	P$ERF1_Q2	ERF1	3707	3715	1	1	0.94	GGCGGcaa
OsSK12	P$AT5G54070_01	AT5G54070	3711	3717	1	1	0.915	gCAACG
OsSK12	P$AT5G04240_01	AT5G04240	3729	3735	1	1	0.939	aGGCAC
OsSK12	P$AT2G33710_01	AT2G33710	3740	3755	1	1	0.89	gcggcagCGCCGccg
OsSK12	P$ERF73_01	ERF73	3740	3761	1	1	0.945	gcggcagCGCCGccgcctccg
OsSK12	P$ERF4_05	ERF4	3743	3758	1	1	0.932	gcagCGCCGccgcct
OsSK12	P$AT2G33710_01	AT2G33710	3743	3758	1	1	0.964	gcagcgcCGCCGcct
OsSK12	P$ERF73_01	ERF73	3743	3764	1	1	0.969	gcagcgcCGCCGcctccgcag
OsSK12	P$ABI4_01	ABI4	3744	3755	1	1	0.913	cagcgCCGCCg
OsSK12	P$RRTF1_02	RRTF1	3745	3755	1	1	0.964	agCGCCGccg
OsSK12	P$RAP26_03	RAP26	3745	3755	1	1	0.994	agCGCCGccg
OsSK12	P$RAP210_04	RAP210	3745	3755	1	1	0.976	agCGCCGccg
OsSK12	P$ERF112_02	ERF112	3745	3755	1	1	0.988	agCGCCGccg
OsSK12	P$CRF4_01	CRF4	3746	3754	1	1	1	gCGCCGcc
OsSK12	P$ERF4_04	ERF4	3746	3754	1	1	0.99	gCGCCGcc
OsSK12	P$ERF069_01	ERF069	3746	3755	1	1	0.999	gCGCCGccg
OsSK12	P$ERF11_01	ERF11	3746	3756	1	1	0.992	gCGCCGccgc
OsSK12	P$ABI4_03	ABI4	3746	3756	1	1	0.979	gcGCCGCcgc
OsSK12	P$WRAF1_01	WRAF1	3746	3756	1	1	0.942	gcGCCGCcgc
OsSK12	P$WRAF2_01	WRAF2	3746	3756	1	1	0.886	gcGCCGCcgc
OsSK12	P$PTI5_01	TI5	3746	3756	1	1	0.919	gcGCCGCcgc

OsSK12	P$DREBI5_01	DREBI5	3746	3756	1	1	0.956	gcGCCGCcgc
OsSK12	P$AT2G47520_01	AT2G47520	3746	3756	1	1	0.96	gcGCCGCcgc
OsSK12	P$DREB2B_01	DREB2B	3746	3756	1	1	0.916	gcGCCGCcgc
OsSK12	P$CRF1_02	CRF1	3746	3756	1	1	0.916	gcGCCGCcgc
OsSK12	P$OPBP1_01	OPBP1	3746	3756	1	1	0.917	gcGCCGCcgc
OsSK12	P$ATERF14_01	ATERF14	3746	3756	1	1	0.913	gcGCCGCcgc
OsSK12	P$ERF112_01	ERF112	3746	3756	1	1	0.923	gcGCCGCcgc
OsSK12	P$DREB2A_02	DREB2A	3746	3756	1	1	0.859	gcGCCGCcgc
OsSK12	P$ERF1_02	ERF1	3746	3756	1	1	0.948	gcGCCGCcgc
OsSK12	P$ERF4_02	ERF4	3746	3756	1	1	0.961	gcGCCGCcgc
OsSK12	P$AT5G25390_01	AT5G25390	3746	3756	1	1	0.911	gcGCCGCcgc
OsSK12	P$EREBP1_01	EREBP1	3746	3756	1	1	0.964	gcGCCGCcgc
OsSK12	P$ERF110_02	ERF110	3746	3756	1	1	0.953	gcGCCGCcgc
OsSK12	P$CBF3_01	CBF3	3746	3756	1	1	0.896	gcGCCGCcgc
OsSK12	P$DREBII1_01	DREBII1	3746	3756	1	1	0.934	gcGCCGCcgc
OsSK12	P$DREB1A_01	DREB1A	3746	3756	1	1	0.875	gcGCCGCcgc
OsSK12	P$TSRF1_01	TSRF1	3746	3756	1	1	0.908	gcGCCGCcgc
OsSK12	P$DRF13_01	DRF13	3746	3756	1	1	0.901	gcGCCGCcgc
OsSK12	P$ERF4_03	ERF4	3746	3756	1	1	0.855	gcGCCGCcgc
OsSK12	P$ERF2_03	ERF2	3746	3756	1	1	0.954	gcGCCGCcgc
OsSK12	P$ERF1B_03	ERF1B	3746	3756	1	1	0.918	gcGCCGCcgc
OsSK12	P$RAP26_02	RAP26	3746	3756	1	1	0.919	gcGCCGCcgc
OsSK12	P$CBF5_01	CBF5	3746	3756	1	1	0.913	gcGCCGCcgc
OsSK12	P$CBF16_01	CBF16	3746	3756	1	1	0.931	gcGCCGCcgc
OsSK12	P$CBF17_01	CBF17	3746	3756	1	1	0.958	gcGCCGCcgc
OsSK12	P$ERF1_05	ERF1	3746	3756	1	1	0.928	gcGCCGCcgc
OsSK12	P$AT5G25190_01	AT5G25190	3746	3756	1	1	0.927	gcGCCGCcgc
OsSK12	P$ERF105_01	ERF105	3746	3756	1	1	0.916	gcGCCGCcgc
OsSK12	P$CBF_01	CBF	3746	3756	1	1	0.959	gcGCCGCcgc
OsSK12	P$AT5G11190_01	AT5G11190	3746	3756	1	1	0.905	gcGCCGCcgc
OsSK12	P$AT1G68550_01	AT1G68550	3746	3756	1	1	0.98	gcGCCGCcgc
OsSK12	P$AT1G77640_01	AT1G77640	3746	3756	1	1	0.878	gcGCCGCcgc
OsSK12	P$ERF016_01	ERF016	3746	3756	1	1	0.893	gcGCCGCcgc
OsSK12	P$AT3G61630_01	AT3G61630	3746	3756	1	1	0.988	gcGCCGCcgc
OsSK12	P$AT5G43410_01	AT5G43410	3746	3756	1	1	0.92	gcGCCGCcgc
OsSK12	P$AT5G07310_01	AT5G07310	3746	3756	1	1	0.961	gcGCCGCcgc
OsSK12	P$AT3G16280_01	AT3G16280	3746	3756	1	1	0.868	gcGCCGCcgc
OsSK12	P$DREB1A_03	DREB1A	3746	3756	1	1	0.902	gcGCCGCcgc
OsSK12	P$AT1G49120_01	AT1G49120	3746	3756	1	1	0.881	gcGCCGCcgc
OsSK12	P$DREB2D_01	DREB2D	3746	3756	1	1	0.926	gcGCCGCcgc
OsSK12	P$AT3G25890_01	AT3G25890	3746	3756	1	1	0.897	gcGCCGCcgc
OsSK12	P$AT4G23750_01	AT4G23750	3746	3756	1	1	0.86	gcGCCGCcgc
OsSK12	P$AT4G27950_01	AT4G27950	3746	3756	1	1	0.894	gcGCCGCcgc
OsSK12	P$RRTF1_01	RRTF1	3746	3756	1	1	0.959	gcGCCGCcgc
OsSK12	P$ERF1_Q2_01	ERF1	3746	3760	1	1	0.897	gcGCCGCcgcctcc
OsSK12	P$CRF2_01	CRF2	3746	3754	1	1	0.995	gcGCCGCc
OsSK12	P$ERF096_01	ERF096	3746	3756	1	1	0.998	gcGCCGCcgc
OsSK12	P$ERF098_01	ERF098	3746	3754	1	1	1	gcGCCGCc
OsSK12	P$DREB2C_01	DREB2C	3746	3756	1	1	0.933	gcgCCGCCgc
OsSK12	P$CBF1_02	CBF1	3746	3756	1	1	0.865	gcgCCGCCgc
OsSK12	P$CBF2_03	CBF2	3746	3756	1	1	0.963	gcgcCGCCGc
OsSK12	P$ERF4_05	ERF4	3746	3761	1	1	0.965	gcgcCGCCGcctccg
OsSK12	P$ERF8_01	ERF8	3747	3757	1	1	0.992	CGCCGccgcc
OsSK12	P$ERF3_04	ERF3	3747	3755	1	1	0.983	CGCCGccg
OsSK12	P$OS05G0497200_01	OS05G0497200	3747	3755	1	1	1	CGCCGccg
OsSK12	P$ERF1B_06	ERF1B	3747	3755	1	1	0.985	cGCCGCcg
OsSK12	P$ERF7_02	ERF7	3747	3757	1	1	0.994	cGCCGCcgcc
OsSK12	P$ERF094_01	ERF094	3747	3755	1	1	1	cGCCGCcg
OsSK12	P$ERF2_01	ERF2	3747	3754	1	1	1	cgCCGCC
OsSK12	P$ERF13_02	ERF13	3747	3755	1	1	0.996	cgCCGCCg
OsSK12	P$ABI4_01	ABI4	3747	3758	1	1	0.85	cgccgCCGCCt
OsSK12	P$RRTF1_02	RRTF1	3748	3758	1	1	0.93	gcCGCCGcct
OsSK12	P$RAP26_03	RAP26	3748	3758	1	1	0.943	gcCGCCGcct
OsSK12	P$RAP210_04	RAP210	3748	3758	1	1	0.948	gcCGCCGcct
OsSK12	P$ERF112_02	ERF112	3748	3758	1	1	0.991	gcCGCCGcct
OsSK12	P$AT3G63350_01	AT3G63350	3749	3755	1	1	0.866	CCGCCg
OsSK12	P$CRF4_01	CRF4	3749	3757	1	1	0.99	cCGCCGcc
OsSK12	P$ERF4_04	ERF4	3749	3757	1	1	1	cCGCCGcc
OsSK12	P$ERF069_01	ERF069	3749	3758	1	1	0.999	cCGCCGcct
OsSK12	P$ERF11_01	ERF11	3749	3759	1	1	0.993	cCGCCGcctc
OsSK12	P$ABI4_03	ABI4	3749	3759	1	1	0.92	ccGCCGCctc
OsSK12	P$WRAF1_01	WRAF1	3749	3759	1	1	0.932	ccGCCGCctc
OsSK12	P$WRAF2_01	WRAF2	3749	3759	1	1	0.884	ccGCCGCctc
OsSK12	P$PTI5_01	TI5	3749	3759	1	1	0.919	ccGCCGCctc
OsSK12	P$DREBI5_01	DREBI5	3749	3759	1	1	0.88	ccGCCGCctc
OsSK12	P$AT2G47520_01	AT2G47520	3749	3759	1	1	0.964	ccGCCGCctc
OsSK12	P$DREB2B_01	DREB2B	3749	3759	1	1	0.862	ccGCCGCctc
OsSK12	P$CRF1_02	CRF1	3749	3759	1	1	0.909	ccGCCGCctc

OsSK12	P$OPBP1_01	OPBP1	3749	3759	1	1	0.913	ccGCCGCctc
OsSK12	P$ATERF14_01	ATERF14	3749	3759	1	1	0.913	ccGCCGCctc
OsSK12	P$ERF112_01	ERF112	3749	3759	1	1	0.915	ccGCCGCctc
OsSK12	P$ERF1_02	ERF1	3749	3759	1	1	0.963	ccGCCGCctc
OsSK12	P$ERF4_02	ERF4	3749	3759	1	1	0.951	ccGCCGCctc
OsSK12	P$AT5G25390_01	AT5G25390	3749	3759	1	1	0.902	ccGCCGCctc
OsSK12	P$EREBP1_01	EREBP1	3749	3759	1	1	0.98	ccGCCGCctc
OsSK12	P$ERF110_02	ERF110	3749	3759	1	1	0.949	ccGCCGCctc
OsSK12	P$CBF3_01	CBF3	3749	3759	1	1	0.85	ccGCCGCctc
OsSK12	P$DREBII1_01	DREBII1	3749	3759	1	1	0.863	ccGCCGCctc
OsSK12	P$DREB1A_01	DREB1A	3749	3759	1	1	0.873	ccGCCGCctc
OsSK12	P$TSRF1_01	TSRF1	3749	3759	1	1	0.904	ccGCCGCctc
OsSK12	P$DRF13_01	DRF13	3749	3759	1	1	0.877	ccGCCGCctc
OsSK12	P$ERF4_03	ERF4	3749	3759	1	1	0.855	ccGCCGCctc
OsSK12	P$ERF2_03	ERF2	3749	3759	1	1	0.973	ccGCCGCctc
OsSK12	P$ERF1B_03	ERF1B	3749	3759	1	1	0.916	ccGCCGCctc
OsSK12	P$RAP26_02	RAP26	3749	3759	1	1	0.913	ccGCCGCctc
OsSK12	P$CBF16_01	CBF16	3749	3759	1	1	0.858	ccGCCGCctc
OsSK12	P$CBF17_01	CBF17	3749	3759	1	1	0.879	ccGCCGCctc
OsSK12	P$ERF1_05	ERF1	3749	3759	1	1	0.912	ccGCCGCctc
OsSK12	P$AT5G25190_01	AT5G25190	3749	3759	1	1	0.913	ccGCCGCctc
OsSK12	P$ERF105_01	ERF105	3749	3759	1	1	0.907	ccGCCGCctc
OsSK12	P$CBF_01	CBF	3749	3759	1	1	0.884	ccGCCGCctc
OsSK12	P$AT5G11190_01	AT5G11190	3749	3759	1	1	0.896	ccGCCGCctc
OsSK12	P$AT1G68550_01	AT1G68550	3749	3759	1	1	0.967	ccGCCGCctc
OsSK12	P$AT1G77640_01	AT1G77640	3749	3759	1	1	0.878	ccGCCGCctc
OsSK12	P$AT3G61630_01	AT3G61630	3749	3759	1	1	0.982	ccGCCGCctc
OsSK12	P$AT5G43410_01	AT5G43410	3749	3759	1	1	0.919	ccGCCGCctc
OsSK12	P$AT5G07310_01	AT5G07310	3749	3759	1	1	0.953	ccGCCGCctc
OsSK12	P$DREB1A_03	DREB1A	3749	3759	1	1	0.855	ccGCCGCctc
OsSK12	P$AT1G49120_01	AT1G49120	3749	3759	1	1	0.88	ccGCCGCctc
OsSK12	P$DREB2D_01	DREB2D	3749	3759	1	1	0.883	ccGCCGCctc
OsSK12	P$AT3G25890_01	AT3G25890	3749	3759	1	1	0.889	ccGCCGCctc
OsSK12	P$AT4G23750_01	AT4G23750	3749	3759	1	1	0.855	ccGCCGCctc
OsSK12	P$AT4G27950_01	AT4G27950	3749	3759	1	1	0.891	ccGCCGCctc
OsSK12	P$RRTF1_01	RRTF1	3749	3759	1	1	0.955	ccGCCGCctc
OsSK12	P$ERF1_Q2_01	ERF1	3749	3763	1	1	0.904	ccGCCGCctccgca
OsSK12	P$CRF2_01	CRF2	3749	3757	1	1	1	ccGCCGCc
OsSK12	P$ERF096_01	ERF096	3749	3759	1	1	0.998	ccGCCGCctc
OsSK12	P$ERF098_01	ERF098	3749	3757	1	1	1	ccGCCGCc
OsSK12	P$CBF1_02	CBF1	3749	3759	1	1	0.859	ccgCCGCCtc
OsSK12	P$ERF8_01	ERF8	3750	3760	1	1	0.992	CGCCGcctcc
OsSK12	P$ERF3_04	ERF3	3750	3758	1	1	0.973	CGCCGcct
OsSK12	P$OS05G0497200_01	OS05G0497200	3750	3758	1	1	0.964	CGCCGcct
OsSK12	P$ERF1B_06	ERF1B	3750	3758	1	1	0.98	cGCCGCct
OsSK12	P$ERF7_02	ERF7	3750	3760	1	1	0.994	cGCCGCctcc
OsSK12	P$ERF094_01	ERF094	3750	3758	1	1	0.994	cGCCGCct
OsSK12	P$ERF2_01	ERF2	3750	3757	1	1	1	cgCCGCC
OsSK12	P$ERF13_02	ERF13	3750	3758	1	1	0.993	cgCCGCCt
OsSK12	P$AT3G63350_01	AT3G63350	3752	3758	1	1	0.867	CCGCCt
OsSK12	P$AT1G53910_02	AT1G53910	3776	3797	1	1	0.931	cattttccctgGCGGCgccgc
OsSK12	P$RRTF1_05	RRTF1	3781	3796	1	1	0.93	tccctggCGGCGccg
OsSK12	P$AT2G33710_01	AT2G33710	3784	3799	1	1	0.887	ctggcggCGCCGcac
OsSK12	P$ERF73_01	ERF73	3784	3805	1	1	0.86	ctggcggCGCCGcaccgacga
OsSK12	P$E2L_Q2	E2L	3785	3792	1	1	0.91	tGGCGGc
OsSK12	P$AT1G28160_02	AT1G28160	3785	3800	1	1	0.901	tggCGGCGccgcacc
OsSK12	P$RAP26_06	RAP26	3785	3800	1	1	0.875	tggCGGCGccgcacc
OsSK12	P$AT1G68550_03	AT1G68550	3785	3794	1	1	1	tggCGGCGc
OsSK12	P$ERF1_Q2	ERF1	3786	3794	1	1	0.957	GGCGGcgc
OsSK12	P$CBF2_03	CBF2	3787	3797	1	1	0.977	gcggCGCCGc
OsSK12	P$RRTF1_02	RRTF1	3789	3799	1	1	0.897	ggCGCCGcac
OsSK12	P$RAP26_03	RAP26	3789	3799	1	1	0.863	ggCGCCGcac
OsSK12	P$RAP210_04	RAP210	3789	3799	1	1	0.882	ggCGCCGcac
OsSK12	P$ERF112_02	ERF112	3789	3799	1	1	0.947	ggCGCCGcac
OsSK12	P$CRF4_01	CRF4	3790	3798	1	1	0.933	gCGCCGca
OsSK12	P$ERF4_04	ERF4	3790	3798	1	1	0.907	gCGCCGca
OsSK12	P$ERF069_01	ERF069	3790	3799	1	1	0.995	gCGCCGcac
OsSK12	P$ERF11_01	ERF11	3790	3800	1	1	0.975	gCGCCGcacc
OsSK12	P$ATERF14_01	ATERF14	3790	3800	1	1	0.884	gcGCCGCacc
OsSK12	P$ERF4_02	ERF4	3790	3800	1	1	0.883	gcGCCGCacc
OsSK12	P$AT5G25390_01	AT5G25390	3790	3800	1	1	0.858	gcGCCGCacc
OsSK12	P$DREB1A_01	DREB1A	3790	3800	1	1	0.869	gcGCCGCacc
OsSK12	P$ERF4_03	ERF4	3790	3800	1	1	0.903	gcGCCGCacc
OsSK12	P$AT5G25190_01	AT5G25190	3790	3800	1	1	0.851	gcGCCGCacc
OsSK12	P$ABR1_01	ABR1	3790	3800	1	1	0.881	gcGCCGCacc
OsSK12	P$AT3G61630_01	AT3G61630	3790	3800	1	1	0.87	gcGCCGCacc
OsSK12	P$AT5G43410_01	AT5G43410	3790	3800	1	1	0.885	gcGCCGCacc
OsSK12	P$AT3G16280_01	AT3G16280	3790	3800	1	1	0.895	gcGCCGCacc

OsSK12	P$CRF2_01	CRF2	3790	3798	1	1	0.951	gcGCCGCa
OsSK12	P$ERF098_01	ERF098	3790	3798	1	1	0.9	gcGCCGCa
OsSK12	P$ERF8_01	ERF8	3791	3801	1	1	0.955	CGCCGcaccg
OsSK12	P$ERF7_02	ERF7	3791	3801	1	1	0.955	cGCCGCaccg
OsSK12	P$CBF3_02	CBF3	3793	3807	1	1	0.967	ccgcaCCGACgacg
OsSK12	P$CBF1_04	CBF1	3794	3806	1	1	0.945	cgcaCCGACgac
OsSK12	P$RAV2_01	RAV2	3795	3804	1	1	0.858	gcACCGAcg
OsSK12	P$AT1G77200_03	AT1G77200	3795	3809	1	1	0.897	gcaCCGACgacgac
OsSK12	P$ARF1_01	ARF1	3797	3805	1	1	0.897	aCCGACga
OsSK12	P$ARF5_01	ARF5	3797	3805	1	1	0.898	aCCGACga
OsSK12	P$DREB1B_01	DREB1B	3798	3803	1	1	1	CCGAC
OsSK12	P$WRKY18_02	WRKY18	3817	3827	1	1	0.998	gggGTCAAgg
OsSK12	P$WRKY21_02	WRKY21	3817	3827	1	1	0.963	gggGTCAAgg
OsSK12	P$WRKY48_02	WRKY48	3817	3827	1	1	0.996	gggGTCAAgg
OsSK12	P$WRKY57_01	WRKY57	3817	3827	1	1	0.965	gggGTCAAgg
OsSK12	P$WRKY60_01	WRKY60	3817	3828	1	1	0.967	gggGTCAAggg
OsSK12	P$WRKY15_01	WRKY15	3818	3828	1	1	0.98	ggGTCAAggg
OsSK12	P$WRKY2_01	WRKY2	3818	3826	1	1	0.982	ggGTCAAg
OsSK12	P$WRKY25_02	WRKY25	3818	3826	1	1	0.961	ggGTCAAg
OsSK12	P$WRKY40_01	WRKY40	3818	3826	1	1	1	ggGTCAAg
OsSK12	P$WRKY43_02	WRKY43	3818	3828	1	1	0.971	ggGTCAAggg
OsSK12	P$WRKY62_01	WRKY62	3818	3826	1	1	0.875	ggGTCAAg
OsSK12	P$WRKY63_01	WRKY63	3818	3826	1	1	0.992	ggGTCAAg
OsSK12	P$WRKY75_01	WRKY75	3818	3826	1	1	0.958	ggGTCAAg
OsSK12	P$WRKY8_01	WRKY8	3818	3827	1	1	0.989	ggGTCAAgg
OsSK12	P$WRKY23_01	WRKY23	3819	3827	1	1	0.875	gGTCAAgg
OsSK12	P$WRKY30_01	WRKY30	3819	3829	1	1	0.922	gGTCAAggga
OsSK12	P$WRKY18_Q2	WRKY18	3820	3829	1	1	0.965	GTCAAggga
OsSK12	P$AT1G53910_02	AT1G53910	3821	3842	1	1	0.887	tcaagggaaaaGCGGCggagt
OsSK12	P$PBF_01	BF	3825	3836	1	1	0.974	gggAAAAGcgg
OsSK12	P$DOF_Q2	DOF	3825	3836	1	1	0.942	gggAAAAGcgg
OsSK12	P$DOF2_01	DOF2	3825	3836	1	1	0.988	gggaAAAGCgg
OsSK12	P$DOF3_01	DOF3	3825	3836	1	1	0.988	gggaAAAGCgg
OsSK12	P$AT3G51080_01	AT3G51080	3826	3833	1	1	0.918	GGAAAag
OsSK12	P$CDF2_01	CDF2	3826	3836	1	1	0.963	ggAAAAGcgg
OsSK12	P$RRTF1_05	RRTF1	3826	3841	1	1	0.952	ggaaaagCGGCGgag
OsSK12	P$CDF3_01	CDF3	3827	3836	1	1	0.987	gAAAAGcgg
OsSK12	P$AT1G28160_02	AT1G28160	3830	3845	1	1	0.953	aagCGGCGgagtgag
OsSK12	P$RAP26_06	RAP26	3830	3845	1	1	0.928	aagCGGCGgagtgag
OsSK12	P$AT1G68550_03	AT1G68550	3830	3839	1	1	0.995	aagCGGCGg
OsSK12	P$KNOX3_01	KNOX3	3845	3857	1	1	0.99	tgagTGACAgtg
OsSK12	P$SIZF2_01	SIZF2	3846	3856	1	1	0.931	gagTGACAgt
OsSK12	P$ATH1_01	ATH1	3849	3857	1	1	0.975	TGACAgtg
OsSK12	P$PIF3_02	IF3	3869	3886	1	1	0.865	tgggcCACGTcgcaggc
OsSK12	P$HY5_02	HY5	3869	3885	1	1	0.852	tgggcCACGTcgcagg
OsSK12	P$ABF2_01	ABF2	3869	3882	1	1	0.906	tgggcCACGTcgc
OsSK12	P$O2_Q4	O2	3870	3881	1	1	0.888	gggcCACGTcg
OsSK12	P$GBP_Q6	GBP	3871	3883	1	1	0.986	ggcCACGTcgca
OsSK12	P$GBF_Q2	GBF	3871	3883	1	1	0.925	ggcCACGTcgca
OsSK12	P$ABI5_01	ABI5	3871	3881	1	1	0.938	ggcCACGTcg
OsSK12	P$ABF4_01	ABF4	3871	3883	1	1	0.905	ggcCACGTcgca
OsSK12	P$EMBP1_Q2	EMBP1	3872	3882	1	1	0.965	gcCACGTcgc
OsSK12	P$CPRF_Q2	CPRF	3872	3882	1	1	0.855	gcCACGTcgc
OsSK12	P$CPRF3_Q2	CPRF3	3872	3882	1	1	0.979	gcCACGTcgc
OsSK12	P$CPRF2_Q2	CPRF2	3872	3882	1	1	0.969	gcCACGTcgc
OsSK12	P$O2_02	O2	3872	3882	1	1	0.985	gcCACGTcgc
OsSK12	P$TGA1B_Q2	TGA1B	3872	3882	1	1	0.979	gcCACGTcgc
OsSK12	P$TGA1A_Q2	TGA1A	3872	3882	1	1	0.979	gcCACGTcgc
OsSK12	P$CG1_Q6	CG1	3872	3883	1	1	0.871	gcCACGTcgca
OsSK12	P$CPRF1_01	CPRF1	3872	3882	1	1	0.853	gcCACGTcgc
OsSK12	P$CPRF3_01	CPRF3	3872	3882	1	1	0.978	gcCACGTcgc
OsSK12	P$CPRF2_01	CPRF2	3872	3882	1	1	0.969	gcCACGTcgc
OsSK12	P$TGA1B_01	TGA1B	3872	3882	1	1	0.984	gcCACGTcgc
OsSK12	P$PIF3_04	IF3	3872	3882	1	1	0.87	gcCACGTcgc
OsSK12	P$BEE2_01	BEE2	3872	3882	1	1	0.906	gcCACGTcgc
OsSK12	P$BIM3_01	BIM3	3872	3882	1	1	0.875	gcCACGTcgc
OsSK12	P$PHYPA143875_02	HYPA143875	3872	3882	1	1	0.872	gcCACGTcgc
OsSK12	P$SPT_01	SPT	3872	3881	1	1	0.945	gcCACGTcg
OsSK12	P$GBF1F_Q2	GBF1F	3872	3883	1	1	0.987	gcCACGTcgca
OsSK12	P$RITA1_01	RITA1	3873	3880	1	1	0.976	cCACGTc
OsSK12	P$OCSBF1_01	OCSBF1	3874	3879	1	1	1	CACGT
OsSK12	P$TGA1A_01	TGA1A	3874	3881	1	1	0.871	cACGTCg
OsSK12	P$MYB3R1_01	MYB3R1	3877	3892	1	1	0.852	gtcgcaggCCGTTtc
OsSK12	P$MYB3R4_01	MYB3R4	3877	3892	1	1	0.85	gtcgcaggCCGTTtc
OsSK12	P$AT1G53910_01	AT1G53910	3882	3892	1	1	0.895	aGGCCGtttc
OsSK12	P$HSF3_01	HSF3	3890	3896	1	1	0.94	tCGGGG
OsSK12	P$AT1G53910_02	AT1G53910	3945	3966	1	1	0.875	cctgcgcatgaGCGGCggcgg
OsSK12	P$ABI3_01	ABI3	3948	3957	1	1	0.852	gcGCATGag

OsSK12	P$AT1G53910_02	AT1G53910	3948	3969	1	1	0.955	gcgcatgagcgGCGGCggcta
OsSK12	P$FUS3_01	FUS3	3949	3958	1	1	0.858	cGCATGagc
OsSK12	P$RRTF1_05	RRTF1	3950	3965	1	1	0.918	gcatgagCGGCGgcg
OsSK12	P$RRTF1_05	RRTF1	3953	3968	1	1	0.904	tgagcggCGGCGgct
OsSK12	P$AT1G28160_02	AT1G28160	3954	3969	1	1	0.962	gagCGGCGgcggcta
OsSK12	P$RAP26_06	RAP26	3954	3969	1	1	0.939	gagCGGCGgcggcta
OsSK12	P$AT1G68550_03	AT1G68550	3954	3963	1	1	0.995	gagCGGCGg
OsSK12	P$AT1G28160_02	AT1G28160	3957	3972	1	1	0.926	cggCGGCGgctacag
OsSK12	P$RAP26_06	RAP26	3957	3972	1	1	0.915	cggCGGCGgctacag
OsSK12	P$AT1G68550_03	AT1G68550	3957	3966	1	1	0.999	cggCGGCGg
OsSK12	P$ERF1_Q2	ERF1	3958	3966	1	1	0.951	GGCGGcgg
OsSK12	P$ERF1_Q2	ERF1	3961	3969	1	1	0.983	GGCGGcta
OsSK12	P$ATHSFA1D_01	ATHSFA1D	3965	3971	1	1	0.94	gCTACA
OsSK12	P$ARR1_01	ARR1	3975	3985	1	1	0.944	ttgGAATCcc
OsSK12	P$AT3G60580_01	AT3G60580	3978	3985	1	1	0.932	gaATCCC
OsSK13	P$ATHSFA1D_01	ATHSFA1D	7	13	1	1	0.985	cCTACA
OsSK13	P$PEND_01	END	14	22	1	1	0.874	gAAGAAat
OsSK13	P$BPC1_Q2	BPC1	16	22	1	1	0.99	AGAAAt
OsSK13	P$ABI3_01	ABI3	21	30	1	1	0.882	ttGCATGaa
OsSK13	P$ATHB6_01	ATHB6	29	38	1	1	0.9	aaAATAAca
OsSK13	P$ATMYB15_Q2	ATMYB15	33	39	1	1	0.865	TAACAg
OsSK13	P$MYB24_01	MYB24	38	47	1	1	0.883	gaaTTAGGg
OsSK13	P$DOF1_01	DOF1	49	60	1	1	0.987	attTAAAGtgc
OsSK13	P$C1_Q2	C1	83	94	1	1	0.913	agAACTAgggt
OsSK13	P$PBF_Q2	BF	113	119	1	1	0.958	cAAAGG
OsSK13	P$AT5G66730_01	AT5G66730	114	125	1	1	0.889	aaaggGGGATc
OsSK13	P$UIF1_01	UIF1	137	147	1	1	0.981	ataGATTCtg
OsSK13	P$ERF105_01	ERF105	144	154	1	1	0.917	ctGCCGCcac
OsSK13	P$CBF_01	CBF	144	154	1	1	0.916	ctGCCGCcac
OsSK13	P$AT5G11190_01	AT5G11190	144	154	1	1	0.906	ctGCCGCcac
OsSK13	P$AT1G68550_01	AT1G68550	144	154	1	1	0.988	ctGCCGCcac
OsSK13	P$AT1G77640_01	AT1G77640	144	154	1	1	0.908	ctGCCGCcac
OsSK13	P$ERF016_01	ERF016	144	154	1	1	0.903	ctGCCGCcac
OsSK13	P$AT3G61630_01	AT3G61630	144	154	1	1	0.965	ctGCCGCcac
OsSK13	P$AT5G25190_01	AT5G25190	144	154	1	1	0.903	ctGCCGCcac
OsSK13	P$ERF1_05	ERF1	144	154	1	1	0.891	ctGCCGCcac
OsSK13	P$CBF17_01	CBF17	144	154	1	1	0.913	ctGCCGCcac
OsSK13	P$CBF16_01	CBF16	144	154	1	1	0.919	ctGCCGCcac
OsSK13	P$CBF5_01	CBF5	144	154	1	1	0.911	ctGCCGCcac
OsSK13	P$RAP26_02	RAP26	144	154	1	1	1	ctGCCGCcac
OsSK13	P$ERF1B_03	ERF1B	144	154	1	1	0.925	ctGCCGCcac
OsSK13	P$ERF2_03	ERF2	144	154	1	1	0.981	ctGCCGCcac
OsSK13	P$DRF13_01	DRF13	144	154	1	1	0.945	ctGCCGCcac
OsSK13	P$TSRF1_01	TSRF1	144	154	1	1	0.934	ctGCCGCcac
OsSK13	P$AT5G43410_01	AT5G43410	144	154	1	1	0.955	ctGCCGCcac
OsSK13	P$RAP26L_02	RAP26L	144	154	1	1	1	ctGCCGCcac
OsSK13	P$DREB2C_01	DREB2C	144	154	1	1	0.874	ctgCCGCCac
OsSK13	P$ERF098_01	ERF098	144	152	1	1	0.997	ctGCCGCc
OsSK13	P$ERF096_01	ERF096	144	154	1	1	0.985	ctGCCGCcac
OsSK13	P$CRF2_01	CRF2	144	152	1	1	0.933	ctGCCGCc
OsSK13	P$ERF1_Q2_01	ERF1	144	158	1	1	0.954	ctGCCGCcacgaat
OsSK13	P$CEJ1_02	CEJ1	144	154	1	1	0.895	ctGCCGCcac
OsSK13	P$RRTF1_01	RRTF1	144	154	1	1	0.982	ctGCCGCcac
OsSK13	P$AT4G27950_01	AT4G27950	144	154	1	1	0.913	ctGCCGCcac
OsSK13	P$AT4G23750_01	AT4G23750	144	154	1	1	0.888	ctGCCGCcac
OsSK13	P$AT3G25890_01	AT3G25890	144	154	1	1	0.978	ctGCCGCcac
OsSK13	P$DREB2D_01	DREB2D	144	154	1	1	0.947	ctGCCGCcac
OsSK13	P$AT1G49120_01	AT1G49120	144	154	1	1	0.873	ctGCCGCcac
OsSK13	P$DREB1A_03	DREB1A	144	154	1	1	0.945	ctGCCGCcac
OsSK13	P$AT3G16280_01	AT3G16280	144	154	1	1	0.86	ctGCCGCcac
OsSK13	P$AT5G07310_01	AT5G07310	144	154	1	1	1	ctGCCGCcac
OsSK13	P$CBF1_02	CBF1	144	154	1	1	0.901	ctgCCGCCac
OsSK13	P$DREB1A_01	DREB1A	144	154	1	1	0.858	ctGCCGCcac
OsSK13	P$CBF1_01	CBF1	144	154	1	1	0.944	cTGCCGccac
OsSK13	P$RAP210_02	RAP210	144	154	1	1	0.873	cTGCCGccac
OsSK13	P$DREB2F_01	DREB2F	144	154	1	1	0.918	cTGCCGccac
OsSK13	P$ORA47_01	ORA47	144	154	1	1	0.875	cTGCCGccac
OsSK13	P$DREB1E_02	DREB1E	144	154	1	1	0.928	cTGCCGccac
OsSK13	P$AT4G28140_01	AT4G28140	144	154	1	1	0.986	cTGCCGccac
OsSK13	P$AT1G71520_01	AT1G71520	144	154	1	1	0.864	cTGCCGccac
OsSK13	P$AT1G33760_01	AT1G33760	144	154	1	1	0.916	cTGCCGccac
OsSK13	P$DREB1F_01	DREB1F	144	154	1	1	0.946	cTGCCGccac
OsSK13	P$AT1G71450_01	AT1G71450	144	154	1	1	0.883	cTGCCGccac
OsSK13	P$CBF1_03	CBF1	144	154	1	1	0.937	cTGCCGccac
OsSK13	P$JERF1_01	JERF1	144	154	1	1	1	cTGCCGccac
OsSK13	P$CEF1_01	CEF1	144	154	1	1	0.983	cTGCCGccac
OsSK13	P$DREB1_01	DREB1	144	154	1	1	0.901	cTGCCGccac
OsSK13	P$JERF3_01	JERF3	144	154	1	1	0.982	cTGCCGccac

OsSK13	P$DREB6_01	DREB6	144	154	1	1	0.902	cTGCCGccac
OsSK13	P$ERF019_01	ERF019	144	154	1	1	0.899	cTGCCGccac
OsSK13	P$ABI4_03	ABI4	144	154	1	1	0.929	ctGCCGCcac
OsSK13	P$WRAF1_01	WRAF1	144	154	1	1	1	ctGCCGCcac
OsSK13	P$DREBII1_01	DREBII1	144	154	1	1	0.919	ctGCCGCcac
OsSK13	P$CBF3_01	CBF3	144	154	1	1	0.945	ctGCCGCcac
OsSK13	P$ERF110_02	ERF110	144	154	1	1	0.994	ctGCCGCcac
OsSK13	P$EREBP1_01	EREBP1	144	154	1	1	0.985	ctGCCGCcac
OsSK13	P$AT5G25390_01	AT5G25390	144	154	1	1	0.904	ctGCCGCcac
OsSK13	P$ERF4_02	ERF4	144	154	1	1	0.938	ctGCCGCcac
OsSK13	P$ERF1_02	ERF1	144	154	1	1	0.986	ctGCCGCcac
OsSK13	P$DREB2A_02	DREB2A	144	154	1	1	0.913	ctGCCGCcac
OsSK13	P$ERF112_01	ERF112	144	154	1	1	0.997	ctGCCGCcac
OsSK13	P$ATERF14_01	ATERF14	144	154	1	1	0.946	ctGCCGCcac
OsSK13	P$WRAF2_01	WRAF2	144	154	1	1	1	ctGCCGCcac
OsSK13	P$PTI5_01	TI5	144	154	1	1	0.992	ctGCCGCcac
OsSK13	P$DREBI5_01	DREBI5	144	154	1	1	0.915	ctGCCGCcac
OsSK13	P$AT2G47520_01	AT2G47520	144	154	1	1	0.974	ctGCCGCcac
OsSK13	P$DREB2B_01	DREB2B	144	154	1	1	0.901	ctGCCGCcac
OsSK13	P$CRF1_02	CRF1	144	154	1	1	0.918	ctGCCGCcac
OsSK13	P$OPBP1_01	OPBP1	144	154	1	1	0.938	ctGCCGCcac
OsSK13	P$ERF1B_06	ERF1B	145	153	1	1	0.961	tGCCGCca
OsSK13	P$ERF7_02	ERF7	145	155	1	1	0.988	tGCCGCcacg
OsSK13	P$ERF094_01	ERF094	145	153	1	1	0.974	tGCCGCca
OsSK13	P$ERF2_01	ERF2	145	152	1	1	0.929	tgCCGCC
OsSK13	P$ERF13_02	ERF13	145	153	1	1	0.982	tgCCGCCa
OsSK13	P$E2FA_02	E2FA	145	155	1	1	0.851	tgcCGCCAcg
OsSK13	P$AT3G63350_01	AT3G63350	147	153	1	1	0.882	CCGCCa
OsSK13	P$RIN_01	RIN	150	160	1	1	0.898	ccacgAATAG
OsSK13	P$ASR1_01	ASR1	166	171	1	1	1	ACCCA
OsSK13	P$TGA1B_01	TGA1B	192	202	1	1	0.899	aaGACGTatc
OsSK13	P$ARR2_01	ARR2	195	205	1	1	0.989	acgtATCTTa
OsSK13	P$GT1_Q6	GT1	207	214	1	1	0.912	GTAAAca
OsSK13	P$BPC1_Q2	BPC1	226	232	1	1	0.997	AGAAAa
OsSK13	P$GATA8_01	GATA8	241	250	1	1	0.99	tcGATCTac
OsSK13	P$ATHSFA1D_01	ATHSFA1D	245	251	1	1	0.941	tCTACA
OsSK13	P$ARR18_01	ARR18	326	339	1	1	0.911	cttcAGATAtaca
OsSK13	P$ARR18_01	ARR18	339	352	1	1	0.957	gaccAGATAtgca
OsSK13	P$WRKY11_01	WRKY11	360	374	1	1	0.854	agatTTGACtattg
OsSK13	P$WRKY11_Q2	WRKY11	363	371	1	1	0.952	tTTGACta
OsSK13	P$AT3G18650_01	AT3G18650	366	377	1	1	0.94	gactaTTGTAa
OsSK13	P$HSFA4A_01	HSFA4A	367	373	1	1	1	aCTATT
OsSK13	P$TGA1_01	TGA1	379	390	1	1	0.909	tttTGACGaac
OsSK13	P$WRKY11_Q2	WRKY11	380	388	1	1	0.924	tTTGACga
OsSK13	P$TGA5_01	TGA5	381	389	1	1	0.856	tTGACGaa
OsSK13	P$ARR18_01	ARR18	394	407	1	1	0.889	atgaAGATAacaa
OsSK13	P$ZAT1_01	ZAT1	397	408	1	1	0.868	aagataACAAA
OsSK13	P$ATMYB15_Q2	ATMYB15	401	407	1	1	1	TAACAa
OsSK13	P$ATHB6_01	ATHB6	414	423	1	1	0.969	agAATAAtc
OsSK13	P$ATHB5_04	ATHB5	414	425	1	1	0.882	agAATAAtcaa
OsSK13	P$ATHB1_03	ATHB1	414	425	1	1	0.887	agAATAAtcaa
OsSK13	P$ATHB16_01	ATHB16	415	423	1	1	0.855	gAATAAtc
OsSK13	P$HAT1_01	HAT1	417	427	1	1	0.876	atAATCAaca
OsSK13	P$ATHB7_01	ATHB7	417	427	1	1	0.88	atAATCAaca
OsSK13	P$RAV1_01	RAV1	419	431	1	1	0.953	aatCAACAaaat
OsSK13	P$EDT1_01	EDT1	439	449	1	1	0.881	gaaTTAATca
OsSK13	P$ATHB7_01	ATHB7	442	452	1	1	0.854	ttAATCAcca
OsSK13	P$HAT1_01	HAT1	442	452	1	1	0.863	ttAATCAcca
OsSK13	P$ABF2_01	ABF2	445	458	1	1	0.903	atcacCACGTcct
OsSK13	P$O2_Q4	O2	446	457	1	1	0.869	tcacCACGTcc
OsSK13	P$ABF4_01	ABF4	447	459	1	1	0.881	cacCACGTcctt
OsSK13	P$ABI5_01	ABI5	447	457	1	1	0.906	cacCACGTcc
OsSK13	P$GBP_Q6	GBP	447	459	1	1	0.89	cacCACGTcctt
OsSK13	P$CPRF2_01	CPRF2	448	458	1	1	0.956	acCACGTcct
OsSK13	P$TGA1B_01	TGA1B	448	458	1	1	0.901	acCACGTcct
OsSK13	P$BEE2_01	BEE2	448	458	1	1	0.907	acCACGTcct
OsSK13	P$BIM3_01	BIM3	448	458	1	1	0.879	acCACGTcct
OsSK13	P$PHYPA143875_02	HYPA143875	448	458	1	1	0.873	acCACGTcct
OsSK13	P$SPT_01	SPT	448	457	1	1	0.945	acCACGTcc
OsSK13	P$GBF1F_Q2	GBF1F	448	459	1	1	0.917	acCACGTcctt
OsSK13	P$CPRF3_01	CPRF3	448	458	1	1	0.96	acCACGTcct
OsSK13	P$EMBP1_Q2	EMBP1	448	458	1	1	0.88	acCACGTcct
OsSK13	P$CPRF3_Q2	CPRF3	448	458	1	1	0.949	acCACGTcct
OsSK13	P$CPRF2_Q2	CPRF2	448	458	1	1	0.954	acCACGTcct
OsSK13	P$O2_02	O2	448	458	1	1	0.961	acCACGTcct
OsSK13	P$TGA1B_Q2	TGA1B	448	458	1	1	0.907	acCACGTcct
OsSK13	P$TGA1A_Q2	TGA1A	448	458	1	1	0.973	acCACGTcct
OsSK13	P$RITA1_01	RITA1	449	456	1	1	0.976	cCACGTc

OsSK13	P$OCSBF1_01	OCSBF1	450	455	1	1	1	CACGT
OsSK13	P$TGA1A_01	TGA1A	450	457	1	1	0.861	cACGTCc
OsSK13	P$SED_Q2	SED	451	461	1	1	0.968	acgtCCTTTt
OsSK13	P$RIN_Q2	RIN	454	465	1	1	0.878	tcctTTTAAgg
OsSK13	P$PBF_Q2_01	BF	455	461	1	1	1	CCTTTt
OsSK13	P$ATSPL8_01	ATSPL8	473	489	1	1	0.939	ttgagTGTACtagctc
OsSK13	P$CBNAC_01	CBNAC	499	505	1	1	1	tTGCTT
OsSK13	P$CBNAC_02	CBNAC	499	515	1	1	0.88	tTGCTTcactgcactg
OsSK13	P$MYB3R5_01	MYB3R5	538	553	1	1	0.872	ccagcgctgCCGTTg
OsSK13	P$MYB3R1_01	MYB3R1	539	554	1	1	0.873	cagcgctgCCGTTga
OsSK13	P$MYB3R4_01	MYB3R4	539	554	1	1	0.882	cagcgctgCCGTTga
OsSK13	P$ORA47_01	ORA47	544	554	1	1	0.886	cTGCCGttga
OsSK13	P$AT1G71520_01	AT1G71520	544	554	1	1	0.896	cTGCCGttga
OsSK13	P$AT1G33760_01	AT1G33760	544	554	1	1	0.868	cTGCCGttga
OsSK13	P$CBF1_03	CBF1	544	554	1	1	0.906	cTGCCGttga
OsSK13	P$JERF1_01	JERF1	544	554	1	1	0.89	cTGCCGttga
OsSK13	P$DREBIII4_01	DREBIII4	544	554	1	1	0.893	cTGCCGttga
OsSK13	P$ERF019_01	ERF019	544	554	1	1	0.9	cTGCCGttga
OsSK13	P$CBF1_01	CBF1	544	554	1	1	0.879	cTGCCGttga
OsSK13	P$HSFA4A_01	HSFA4A	599	605	1	1	0.91	gCTATT
OsSK13	P$CBF1_03	CBF1	625	635	1	1	0.857	tTGCCGgaat
OsSK13	P$AT3G60580_01	AT3G60580	658	665	1	1	0.883	aaATCCC
OsSK13	P$TRB2_01	TRB2	660	668	1	1	0.928	atCCCTAt
OsSK13	P$MYB1L_01	MYB1L	660	670	1	1	0.952	atCCCTAttt
OsSK13	P$HSFA4A_01	HSFA4A	663	669	1	1	0.964	cCTATT
OsSK13	P$AT1G66560_01	AT1G66560	674	684	1	1	0.878	cccTTAACaa
OsSK13	P$WRKY48_01	WRKY48	675	684	1	1	0.852	ccttAACAA
OsSK13	P$WRKY7_01	WRKY7	675	684	1	1	0.86	ccTTAACaa
OsSK13	P$AT5G15130_01	AT5G15130	675	684	1	1	0.897	ccTTAACaa
OsSK13	P$AT5G41570_01	AT5G41570	675	684	1	1	0.852	ccTTAACaa
OsSK13	P$AT1G68150_01	AT1G68150	675	684	1	1	0.865	ccTTAACaa
OsSK13	P$AT1G66600_01	AT1G66600	675	684	1	1	0.881	ccTTAACaa
OsSK13	P$AT1G64000_01	AT1G64000	675	684	1	1	0.852	ccTTAACaa
OsSK13	P$AT1G18860_01	AT1G18860	675	684	1	1	0.902	ccTTAACaa
OsSK13	P$WRKY21_01	WRKY21	675	684	1	1	0.856	ccTTAACaa
OsSK13	P$ATMYB15_Q2	ATMYB15	678	684	1	1	1	TAACAa
OsSK13	P$TCP16_03	TCP16	686	697	1	1	0.946	tccgggTCCAC
OsSK13	P$BHLH112_01	BHLH112	692	701	1	1	1	tccACTTGc
OsSK13	P$CBNAC_01	CBNAC	697	703	1	1	1	tTGCTT
OsSK13	P$CBNAC_02	CBNAC	697	713	1	1	0.909	tTGCTTccgtcaaaaa
OsSK13	P$WRKY60_01	WRKY60	702	713	1	1	0.898	tccGTCAAaaa
OsSK13	P$WRKY57_01	WRKY57	702	712	1	1	0.959	tccGTCAAaa
OsSK13	P$WRKY48_02	WRKY48	702	712	1	1	0.989	tccGTCAAaa
OsSK13	P$WRKY21_02	WRKY21	702	712	1	1	0.954	tccGTCAAaa
OsSK13	P$WRKY18_02	WRKY18	702	712	1	1	0.946	tccGTCAAaa
OsSK13	P$WRKY8_01	WRKY8	703	712	1	1	0.981	ccGTCAAaa
OsSK13	P$WRKY75_01	WRKY75	703	711	1	1	0.937	ccGTCAAa
OsSK13	P$WRKY63_01	WRKY63	703	711	1	1	0.887	ccGTCAAa
OsSK13	P$WRKY43_02	WRKY43	703	713	1	1	0.957	ccGTCAAaaa
OsSK13	P$WRKY40_01	WRKY40	703	711	1	1	0.981	ccGTCAAa
OsSK13	P$WRKY25_02	WRKY25	703	711	1	1	0.898	ccGTCAAa
OsSK13	P$WRKY2_01	WRKY2	703	711	1	1	0.912	ccGTCAAa
OsSK13	P$WRKY15_01	WRKY15	703	713	1	1	0.965	ccGTCAAaaa
OsSK13	P$WRKY30_01	WRKY30	704	714	1	1	0.902	cGTCAAaaac
OsSK13	P$WRKY18_Q2	WRKY18	705	714	1	1	0.938	GTCAAaaac
OsSK13	P$AMS_01	AMS	711	721	1	1	0.865	aaCAGGTgta
OsSK13	P$DOF3_01	DOF3	740	751	1	1	0.991	aaaaAAAGCag
OsSK13	P$DOF2_01	DOF2	740	751	1	1	0.994	aaaaAAAGCag
OsSK13	P$DOF_Q2	DOF	740	751	1	1	0.997	aaaAAAAGcag
OsSK13	P$PBF_01	BF	740	751	1	1	0.977	aaaAAAAGcag
OsSK13	P$CDF2_01	CDF2	741	751	1	1	0.988	aaAAAAGcag
OsSK13	P$CDF3_01	CDF3	742	751	1	1	0.983	aAAAAGcag
OsSK13	P$HSFA2_01	HSFA2	754	760	1	1	1	CCAAAa
OsSK13	P$AT4G01720_01	AT4G01720	776	785	1	1	0.913	gGTAAAatc
OsSK13	P$HAT1_01	HAT1	779	789	1	1	0.852	aaAATCAcaa
OsSK13	P$ARR18_01	ARR18	787	800	1	1	0.957	aattAGATAtgaa
OsSK13	P$AT1G14580_01	AT1G14580	799	810	1	1	0.942	aaataGGGATt
OsSK13	P$AT5G66730_01	AT5G66730	799	810	1	1	0.936	aaataGGGATt
OsSK13	P$SED_Q2	SED	809	819	1	1	0.897	tgctCCTTTa
OsSK13	P$PBF_Q2_01	BF	813	819	1	1	0.998	CCTTTa
OsSK13	P$MYB24_01	MYB24	813	822	1	1	0.938	cctTTAGGt
OsSK13	P$MYB131_01	MYB131	813	824	1	1	0.901	cctTTAGGttc
OsSK13	P$SED_Q2	SED	827	837	1	1	0.908	tgggCCTTTt
OsSK13	P$RIN_Q2	RIN	831	842	1	1	0.987	ccttTTTAAgg
OsSK13	P$PBF_Q2_01	BF	831	837	1	1	1	CCTTTt
OsSK13	P$NAC92_01	NAC92	877	889	1	1	0.947	gaACACGgtaac
OsSK13	P$UIF1_01	UIF1	886	896	1	1	0.853	aacGATTCcg
OsSK13	P$ABF2_01	ABF2	898	911	1	1	0.888	tccctCACGTtaa

OsSK13	P$GBP_Q6	GBP	900	912	1	1	0.882	cctCACGTtaat
OsSK13	P$ABI5_01	ABI5	900	910	1	1	0.89	cctCACGTta
OsSK13	P$ABF4_01	ABF4	900	912	1	1	0.867	cctCACGTtaat
OsSK13	P$CPRF3_01	CPRF3	901	911	1	1	0.935	ctCACGTtaa
OsSK13	P$CPRF2_01	CPRF2	901	911	1	1	0.944	ctCACGTtaa
OsSK13	P$BEE2_01	BEE2	901	911	1	1	0.92	ctCACGTtaa
OsSK13	P$BIM2_01	BIM2	901	911	1	1	0.857	ctCACGTtaa
OsSK13	P$BIM3_01	BIM3	901	911	1	1	0.887	ctCACGTtaa
OsSK13	P$PHYPA143875_02	HYPA143875	901	911	1	1	0.885	ctCACGTtaa
OsSK13	P$SPT_01	SPT	901	910	1	1	0.921	ctCACGTta
OsSK13	P$TGA1A_Q2	TGA1A	901	911	1	1	0.976	ctCACGTtaa
OsSK13	P$TGA1B_Q2	TGA1B	901	911	1	1	0.91	ctCACGTtaa
OsSK13	P$O2_02	O2	901	911	1	1	0.948	ctCACGTtaa
OsSK13	P$CPRF2_Q2	CPRF2	901	911	1	1	0.943	ctCACGTtaa
OsSK13	P$CPRF3_Q2	CPRF3	901	911	1	1	0.923	ctCACGTtaa
OsSK13	P$RITA1_01	RITA1	902	909	1	1	0.969	tCACGTt
OsSK13	P$OCSBF1_01	OCSBF1	903	908	1	1	1	CACGT
OsSK13	P$GT1_Q6	GT1	918	925	1	1	0.912	GTGAAga
OsSK13	P$AT3G60580_01	AT3G60580	945	952	1	1	0.905	caATCCC
OsSK13	P$BZR1_01	BZR1	951	957	1	1	0.902	CGTGCt
OsSK13	P$C1_Q2	C1	961	972	1	1	0.932	taAACTAattt
OsSK13	P$HSFA2_01	HSFA2	978	984	1	1	0.922	CCAAAt
OsSK13	P$GT1_Q6_02	GT1	984	996	1	1	0.945	accgtcTTAACt
OsSK13	P$AT1G66560_01	AT1G66560	987	997	1	1	0.895	gtcTTAACtg
OsSK13	P$WRKY7_01	WRKY7	988	997	1	1	0.884	tcTTAACtg
OsSK13	P$AT2G24570_01	AT2G24570	988	997	1	1	0.87	tcTTAACtg
OsSK13	P$WRKY46_01	WRKY46	988	997	1	1	0.908	tcTTAACtg
OsSK13	P$AT5G15130_01	AT5G15130	988	997	1	1	0.895	tcTTAACtg
OsSK13	P$AT5G41570_01	AT5G41570	988	997	1	1	0.875	tcTTAACtg
OsSK13	P$AT1G68150_01	AT1G68150	988	997	1	1	0.859	tcTTAACtg
OsSK13	P$AT1G66600_01	AT1G66600	988	997	1	1	0.891	tcTTAACtg
OsSK13	P$AT1G64000_01	AT1G64000	988	997	1	1	0.875	tcTTAACtg
OsSK13	P$AT1G18860_01	AT1G18860	988	997	1	1	0.899	tcTTAACtg
OsSK13	P$AT4G11070_01	AT4G11070	988	997	1	1	0.949	tcTTAACtg
OsSK13	P$WRKY43_01	WRKY43	988	997	1	1	0.878	tcTTAACtg
OsSK13	P$WRKY21_01	WRKY21	988	997	1	1	0.879	tcTTAACtg
OsSK13	P$ATSPL8_01	ATSPL8	990	1006	1	1	0.927	ttaacTGTACattgag
OsSK13	P$AT3G60580_01	AT3G60580	1005	1012	1	1	1	gtATCCC
OsSK13	P$ATSPL8_01	ATSPL8	1024	1040	1	1	0.92	aaaacTGTACattgag
OsSK13	P$ATHB7_01	ATHB7	1041	1051	1	1	0.922	atAATCAtgg
OsSK13	P$HAT1_01	HAT1	1041	1051	1	1	0.986	atAATCAtgg
OsSK13	P$ATHB4_02	ATHB4	1042	1052	1	1	0.879	taATCATgga
OsSK13	P$ARR2_01	ARR2	1062	1072	1	1	0.985	ccggATCTTc
OsSK13	P$GATA8_01	GATA8	1063	1072	1	1	0.976	cgGATCTtc
OsSK13	P$WRKY44_01	WRKY44	1082	1091	1	1	0.94	AGTCCatct
OsSK13	P$SBF1_01	SBF1	1096	1110	1	1	0.862	ctgcatTTAATtat
OsSK13	P$EDT1_01	EDT1	1099	1109	1	1	0.894	catTTAATta
OsSK13	P$GATA15_01	GATA15	1107	1116	1	1	0.999	taTGATCtg
OsSK13	P$GATA11_01	GATA11	1108	1116	1	1	0.866	atGATCTg
OsSK13	P$GATA8_01	GATA8	1108	1117	1	1	0.985	atGATCTgc
OsSK13	P$ARR2_01	ARR2	1115	1125	1	1	0.893	gcctATCTTt
OsSK13	P$O2_Q4	O2	1147	1158	1	1	0.891	tttgCATGTgg
OsSK13	P$ABI3_01	ABI3	1148	1157	1	1	0.867	ttGCATGtg
OsSK13	P$AMS_01	AMS	1149	1159	1	1	0.894	tgCATGTggt
OsSK13	P$ATHB1_01	ATHB1	1175	1189	1	1	0.97	ctcaaATTATtgtt
OsSK13	P$ATHB5_01	ATHB5	1178	1187	1	1	0.929	aaaTTATTg
OsSK13	P$SBF1_01	SBF1	1181	1195	1	1	0.871	ttattgTTAAAcct
OsSK13	P$AT3G20750_01	AT3G20750	1187	1195	1	1	0.927	tTAAACct
OsSK13	P$AT4G36620_01	AT4G36620	1218	1226	1	1	0.896	tgaAACCA
OsSK13	P$CBNAC_02	CBNAC	1236	1252	1	1	0.897	cTGCTTattttttcg
OsSK13	P$CBNAC_01	CBNAC	1236	1242	1	1	0.973	cTGCTT
OsSK13	P$LEC2_01	LEC2	1257	1268	1	1	0.947	tcCATGCtcct
OsSK13	P$MYB24_01	MYB24	1270	1279	1	1	1	tagTTAGGt
OsSK13	P$MYB131_01	MYB131	1270	1281	1	1	0.956	tagTTAGGtta
OsSK13	P$MYB3_01	MYB3	1271	1282	1	1	0.918	agtTAGGTtaa
OsSK13	P$SBF1_01	SBF1	1272	1286	1	1	0.905	gttaggTTAATtta
OsSK13	P$MYB4_01	MYB4	1272	1280	1	1	0.901	gtTAGGTt
OsSK13	P$HAT1_01	HAT1	1300	1310	1	1	0.887	gcAATCAagc
OsSK13	P$ATHB7_01	ATHB7	1300	1310	1	1	0.95	gcAATCAagc
OsSK13	P$PEND_01	END	1331	1339	1	1	0.892	tAAGAAac
OsSK13	P$BPC1_Q2	BPC1	1333	1339	1	1	0.99	AGAAAc
OsSK13	P$AT4G36620_01	AT4G36620	1333	1341	1	1	0.9	agaAACCA
OsSK13	P$HSFA2_01	HSFA2	1338	1344	1	1	0.922	CCAAAt
OsSK13	P$BPC1_Q2	BPC1	1344	1350	1	1	0.99	AGAAAt
OsSK13	P$AT2G15660_01	AT2G15660	1366	1377	1	1	0.965	TTCTCatggaa
OsSK13	P$GATA9_01	GATA9	1386	1397	1	1	0.894	accAGATCgtc
OsSK13	P$AGP1_01	AGP1	1387	1397	1	1	0.865	ccAGATCgtc
OsSK13	P$ARR10_01	ARR10	1389	1396	1	1	0.913	AGATCgt

OsSK13	P$LEC2_01	LEC2	1425	1436	1	1	0.994	gtCATGCaaat
OsSK13	P$ERF3_01	ERF3	1444	1454	1	1	0.861	gGGCCGagcc
OsSK13	P$AT1G53910_01	AT1G53910	1444	1454	1	1	0.879	gGGCCGagcc
OsSK13	P$AT5G67000_01	AT5G67000	1444	1454	1	1	0.923	ggGCCGAgcc
OsSK13	P$AT2G20350_01	AT2G20350	1444	1454	1	1	0.903	ggGCCGAgcc
OsSK24	P$ASR1_01	ASR1	47	52	1	1	1	ACCCA
OsSK24	P$BZR1_01	BZR1	64	70	1	1	1	CGTGCg
OsSK24	P$TRB2_01	TRB2	69	77	1	1	0.985	ggCCCTAg
OsSK24	P$MYB1L_01	MYB1L	69	79	1	1	0.968	ggCCCTAgca
OsSK24	P$HSF3_01	HSF3	90	96	1	1	0.969	aCGGGG
OsSK24	P$AT5G04240_01	AT5G04240	106	112	1	1	0.938	tGGCAC
OsSK24	P$ATMYB77_01	ATMYB77	120	133	1	1	0.913	tggtggCAGTTgg
OsSK24	P$AT1G53910_02	AT1G53910	127	148	1	1	0.891	agttggaggtgGCGGCtgcat
OsSK24	P$E2L_Q2	E2L	136	143	1	1	0.91	tGGCGGc
OsSK24	P$ERF1_Q2	ERF1	137	145	1	1	0.994	GGCGGctg
OsSK24	P$GAMYB_Q2	GAMYB	156	169	1	1	0.939	gcgtaACAACaag
OsSK24	P$ATMYB15_Q2	ATMYB15	159	165	1	1	1	TAACAa
OsSK24	P$RAV1_01	RAV1	159	171	1	1	0.923	taaCAACAagag
OsSK24	P$HMG1_01	HMG1	177	186	1	1	0.924	GTTGTgctg
OsSK24	P$AT3G61630_01	AT3G61630	186	196	1	1	0.945	gtGCCGCcat
OsSK24	P$ERF016_01	ERF016	186	196	1	1	0.899	gtGCCGCcat
OsSK24	P$AT1G77640_01	AT1G77640	186	196	1	1	0.908	gtGCCGCcat
OsSK24	P$AT1G68550_01	AT1G68550	186	196	1	1	0.987	gtGCCGCcat
OsSK24	P$AT5G11190_01	AT5G11190	186	196	1	1	0.906	gtGCCGCcat
OsSK24	P$CBF_01	CBF	186	196	1	1	0.905	gtGCCGCcat
OsSK24	P$ERF105_01	ERF105	186	196	1	1	0.916	gtGCCGCcat
OsSK24	P$AT5G25190_01	AT5G25190	186	196	1	1	0.903	gtGCCGCcat
OsSK24	P$ERF1_05	ERF1	186	196	1	1	0.892	gtGCCGCcat
OsSK24	P$CBF17_01	CBF17	186	196	1	1	0.903	gtGCCGCcat
OsSK24	P$CBF16_01	CBF16	186	196	1	1	0.908	gtGCCGCcat
OsSK24	P$CBF5_01	CBF5	186	196	1	1	0.899	gtGCCGCcat
OsSK24	P$RAP26_02	RAP26	186	196	1	1	0.935	gtGCCGCcat
OsSK24	P$ERF1B_03	ERF1B	186	196	1	1	0.928	gtGCCGCcat
OsSK24	P$ERF2_03	ERF2	186	196	1	1	0.935	gtGCCGCcat
OsSK24	P$DRF13_01	DRF13	186	196	1	1	0.938	gtGCCGCcat
OsSK24	P$AT5G43410_01	AT5G43410	186	196	1	1	0.947	gtGCCGCcat
OsSK24	P$RAP26L_02	RAP26L	186	196	1	1	0.982	gtGCCGCcat
OsSK24	P$CBF1_02	CBF1	186	196	1	1	0.93	gtgCCGCCat
OsSK24	P$DREB2C_01	DREB2C	186	196	1	1	0.855	gtgCCGCCat
OsSK24	P$ERF098_01	ERF098	186	194	1	1	0.997	gtGCCGCc
OsSK24	P$ERF096_01	ERF096	186	196	1	1	0.985	gtGCCGCcat
OsSK24	P$CRF2_01	CRF2	186	194	1	1	0.928	gtGCCGCc
OsSK24	P$ERF1_Q2_01	ERF1	186	200	1	1	0.895	gtGCCGCcatggaa
OsSK24	P$CEJ1_02	CEJ1	186	196	1	1	0.868	gtGCCGCcat
OsSK24	P$RRTF1_01	RRTF1	186	196	1	1	0.973	gtGCCGCcat
OsSK24	P$AT4G27950_01	AT4G27950	186	196	1	1	0.907	gtGCCGCcat
OsSK24	P$AT4G23750_01	AT4G23750	186	196	1	1	0.885	gtGCCGCcat
OsSK24	P$AT3G25890_01	AT3G25890	186	196	1	1	0.971	gtGCCGCcat
OsSK24	P$DREB2D_01	DREB2D	186	196	1	1	0.923	gtGCCGCcat
OsSK24	P$AT1G49120_01	AT1G49120	186	196	1	1	0.871	gtGCCGCcat
OsSK24	P$DREB1A_03	DREB1A	186	196	1	1	0.937	gtGCCGCcat
OsSK24	P$AT5G07310_01	AT5G07310	186	196	1	1	0.96	gtGCCGCcat
OsSK24	P$TSRF1_01	TSRF1	186	196	1	1	0.935	gtGCCGCcat
OsSK24	P$DREBII1_01	DREBII1	186	196	1	1	0.909	gtGCCGCcat
OsSK24	P$ORA47_01	ORA47	186	196	1	1	0.873	gTGCCGccat
OsSK24	P$DREB1E_02	DREB1E	186	196	1	1	0.919	gTGCCGccat
OsSK24	P$AT4G28140_01	AT4G28140	186	196	1	1	0.905	gTGCCGccat
OsSK24	P$AT1G71520_01	AT1G71520	186	196	1	1	0.86	gTGCCGccat
OsSK24	P$AT1G33760_01	AT1G33760	186	196	1	1	0.907	gTGCCGccat
OsSK24	P$DREB1F_01	DREB1F	186	196	1	1	0.941	gTGCCGccat
OsSK24	P$AT1G71450_01	AT1G71450	186	196	1	1	0.877	gTGCCGccat
OsSK24	P$CBF1_03	CBF1	186	196	1	1	0.932	gTGCCGccat
OsSK24	P$JERF1_01	JERF1	186	196	1	1	0.957	gTGCCGccat
OsSK24	P$CEF1_01	CEF1	186	196	1	1	0.923	gTGCCGccat
OsSK24	P$DREB1_01	DREB1	186	196	1	1	0.914	gTGCCGccat
OsSK24	P$JERF3_01	JERF3	186	196	1	1	0.923	gTGCCGccat
OsSK24	P$DREB6_01	DREB6	186	196	1	1	0.915	gTGCCGccat
OsSK24	P$ERF019_01	ERF019	186	196	1	1	0.896	gTGCCGccat
OsSK24	P$CBF1_01	CBF1	186	196	1	1	0.94	gTGCCGccat
OsSK24	P$DREB2F_01	DREB2F	186	196	1	1	0.892	gTGCCGccat
OsSK24	P$ABI4_03	ABI4	186	196	1	1	0.903	gtGCCGCcat
OsSK24	P$WRAF1_01	WRAF1	186	196	1	1	0.922	gtGCCGCcat
OsSK24	P$CBF3_01	CBF3	186	196	1	1	0.939	gtGCCGCcat
OsSK24	P$ERF110_02	ERF110	186	196	1	1	0.874	gtGCCGCcat
OsSK24	P$EREBP1_01	EREBP1	186	196	1	1	0.934	gtGCCGCcat
OsSK24	P$AT5G25390_01	AT5G25390	186	196	1	1	0.903	gtGCCGCcat
OsSK24	P$ERF4_02	ERF4	186	196	1	1	0.939	gtGCCGCcat
OsSK24	P$ERF1_02	ERF1	186	196	1	1	0.937	gtGCCGCcat

OsSK24	P$DREB2A_02	DREB2A	186	196	1	1	0.908	gtGCCGCcat
OsSK24	P$ERF112_01	ERF112	186	196	1	1	0.906	gtGCCGCcat
OsSK24	P$ATERF14_01	ATERF14	186	196	1	1	0.938	gtGCCGCcat
OsSK24	P$WRAF2_01	WRAF2	186	196	1	1	0.892	gtGCCGCcat
OsSK24	P$PTI5_01	TI5	186	196	1	1	0.99	gtGCCGCcat
OsSK24	P$DREBI5_01	DREBI5	186	196	1	1	0.904	gtGCCGCcat
OsSK24	P$AT2G47520_01	AT2G47520	186	196	1	1	0.946	gtGCCGCcat
OsSK24	P$DREB2B_01	DREB2B	186	196	1	1	0.929	gtGCCGCcat
OsSK24	P$CRF1_02	CRF1	186	196	1	1	0.898	gtGCCGCcat
OsSK24	P$OPBP1_01	OPBP1	186	196	1	1	0.94	gtGCCGCcat
OsSK24	P$E2FA_02	E2FA	187	197	1	1	0.852	tgcCGCCAtg
OsSK24	P$ERF13_02	ERF13	187	195	1	1	0.982	tgCCGCCa
OsSK24	P$ERF2_01	ERF2	187	194	1	1	0.929	tgCCGCC
OsSK24	P$ERF094_01	ERF094	187	195	1	1	0.974	tGCCGCca
OsSK24	P$ERF7_02	ERF7	187	197	1	1	0.989	tGCCGCcatg
OsSK24	P$ERF1B_06	ERF1B	187	195	1	1	0.961	tGCCGCca
OsSK24	P$AT3G63350_01	AT3G63350	189	195	1	1	0.882	CCGCCa
OsSK24	P$DOF2_01	DOF2	194	205	1	1	0.982	atggAAAGCcg
OsSK24	P$DOF3_01	DOF3	194	205	1	1	0.976	atggAAAGCcg
OsSK24	P$RAP21_02	RAP21	199	212	1	1	0.907	aagcCGGTGtggg
OsSK24	P$ALFIN1_Q2	ALFIN1	201	216	1	1	0.884	gccggtGTGGGagag
OsSK24	P$RAMOSA1_01	RAMOSA1	211	225	1	1	0.897	gagaggGAGAGaga
OsSK24	P$RAMOSA1_01	RAMOSA1	213	227	1	1	0.922	gagggaGAGAGagc
OsSK24	P$ERF73_01	ERF73	227	248	1	1	0.857	agagcccCGCCGtcgtcttgc
OsSK24	P$AT2G33710_01	AT2G33710	227	242	1	1	0.901	agagcccCGCCGtcg
OsSK24	P$ABI4_01	ABI4	228	239	1	1	0.883	gagccCCGCCg
OsSK24	P$ERF4_05	ERF4	230	245	1	1	0.925	gcccCGCCGtcgtct
OsSK24	P$ERF112_02	ERF112	232	242	1	1	0.98	ccCGCCGtcg
OsSK24	P$ERF11_01	ERF11	233	243	1	1	0.99	cCGCCGtcgt
OsSK24	P$ERF069_01	ERF069	233	242	1	1	0.994	cCGCCGtcg
OsSK24	P$ERF4_04	ERF4	233	241	1	1	0.964	cCGCCGtc
OsSK24	P$CRF4_01	CRF4	233	241	1	1	0.936	cCGCCGtc
OsSK24	P$AT3G63350_01	AT3G63350	233	239	1	1	0.866	CCGCCg
OsSK24	P$ANAC094_01	ANAC094	234	252	1	1	0.926	cGCCGTcgtcttgccgcg
OsSK24	P$ANAC042_01	ANAC042	234	254	1	1	0.94	cGCCGTcgtcttgccgcgcc
OsSK24	P$ERF3_04	ERF3	234	242	1	1	0.952	CGCCGtcg
OsSK24	P$ERF8_01	ERF8	234	244	1	1	0.986	CGCCGtcgtc
OsSK24	P$AT2G33710_01	AT2G33710	243	258	1	1	0.853	cttgccgCGCCGctc
OsSK24	P$ERF73_01	ERF73	243	264	1	1	0.855	cttgccgCGCCGctcctcctc
OsSK24	P$CBF5_01	CBF5	244	254	1	1	0.86	ttGCCGCgcc
OsSK24	P$DBF2_01	DBF2	244	254	1	1	0.861	ttGCCGCgcc
OsSK24	P$ERF2_03	ERF2	244	254	1	1	0.863	ttGCCGCgcc
OsSK24	P$ERF4_03	ERF4	244	254	1	1	0.889	ttGCCGCgcc
OsSK24	P$DRF13_01	DRF13	244	254	1	1	0.863	ttGCCGCgcc
OsSK24	P$AT2G44940_01	AT2G44940	244	254	1	1	0.863	ttGCCGCgcc
OsSK24	P$DREB1A_01	DREB1A	244	254	1	1	0.853	ttGCCGCgcc
OsSK24	P$DREBII1_01	DREBII1	244	254	1	1	0.869	ttGCCGCgcc
OsSK24	P$CBF3_01	CBF3	244	254	1	1	0.869	ttGCCGCgcc
OsSK24	P$CBF16_01	CBF16	244	254	1	1	0.869	ttGCCGCgcc
OsSK24	P$CBF17_01	CBF17	244	254	1	1	0.862	ttGCCGCgcc
OsSK24	P$ERF098_01	ERF098	244	252	1	1	0.892	ttGCCGCg
OsSK24	P$CRF2_01	CRF2	244	252	1	1	0.872	ttGCCGCg
OsSK24	P$CEJ1_02	CEJ1	244	254	1	1	0.871	ttGCCGCgcc
OsSK24	P$AT4G23750_01	AT4G23750	244	254	1	1	0.862	ttGCCGCgcc
OsSK24	P$DREB1A_03	DREB1A	244	254	1	1	0.867	ttGCCGCgcc
OsSK24	P$AT3G16280_01	AT3G16280	244	254	1	1	0.908	ttGCCGCgcc
OsSK24	P$AT5G43410_01	AT5G43410	244	254	1	1	0.89	ttGCCGCgcc
OsSK24	P$AT5G11190_01	AT5G11190	244	254	1	1	0.861	ttGCCGCgcc
OsSK24	P$CBF_01	CBF	244	254	1	1	0.868	ttGCCGCgcc
OsSK24	P$EREBP1_01	EREBP1	244	254	1	1	0.866	ttGCCGCgcc
OsSK24	P$CBF1_01	CBF1	244	254	1	1	0.899	tTGCCGcgcc
OsSK24	P$AT1G33760_01	AT1G33760	244	254	1	1	0.913	tTGCCGcgcc
OsSK24	P$DREB1F_01	DREB1F	244	254	1	1	0.855	tTGCCGcgcc
OsSK24	P$AT1G71450_01	AT1G71450	244	254	1	1	0.854	tTGCCGcgcc
OsSK24	P$CBF1_03	CBF1	244	254	1	1	0.917	tTGCCGcgcc
OsSK24	P$JERF1_01	JERF1	244	254	1	1	0.903	tTGCCGcgcc
OsSK24	P$CEF1_01	CEF1	244	254	1	1	0.889	tTGCCGcgcc
OsSK24	P$JERF3_01	JERF3	244	254	1	1	0.89	tTGCCGcgcc
OsSK24	P$ERF019_01	ERF019	244	254	1	1	0.911	tTGCCGcgcc
OsSK24	P$AT1G71520_01	AT1G71520	244	254	1	1	0.878	tTGCCGcgcc
OsSK24	P$AT4G28140_01	AT4G28140	244	254	1	1	0.91	tTGCCGcgcc
OsSK24	P$ORA47_01	ORA47	244	254	1	1	0.899	tTGCCGcgcc
OsSK24	P$AT5G25390_01	AT5G25390	244	254	1	1	0.861	ttGCCGCgcc
OsSK24	P$ERF4_02	ERF4	244	254	1	1	0.859	ttGCCGCgcc
OsSK24	P$ERF1_02	ERF1	244	254	1	1	0.867	ttGCCGCgcc
OsSK24	P$DREB2A_02	DREB2A	244	254	1	1	0.863	ttGCCGCgcc
OsSK24	P$ATERF14_01	ATERF14	244	254	1	1	0.889	ttGCCGCgcc
OsSK24	P$AT1G77200_01	AT1G77200	244	254	1	1	0.879	ttGCCGCgcc

OsSK24	P$DREBI5_01	DREBI5	244	254	1	1	0.867	ttGCCGCgcc
OsSK24	P$RAP210_02	RAP210	244	254	1	1	0.86	tTGCCGcgcc
OsSK24	P$DREB2F_01	DREB2F	244	254	1	1	0.892	tTGCCGcgcc
OsSK24	P$ERF7_02	ERF7	245	255	1	1	0.93	tGCCGCgccg
OsSK24	P$ERF112_02	ERF112	248	258	1	1	0.929	cgCGCCGctc
OsSK24	P$RAP210_04	RAP210	248	258	1	1	0.887	cgCGCCGctc
OsSK24	P$RAP26_03	RAP26	248	258	1	1	0.852	cgCGCCGctc
OsSK24	P$RRTF1_02	RRTF1	248	258	1	1	0.873	cgCGCCGctc
OsSK24	P$DREB1A_01	DREB1A	249	259	1	1	0.868	gcGCCGCtcc
OsSK24	P$AT2G44940_01	AT2G44940	249	259	1	1	0.926	gcGCCGCtcc
OsSK24	P$ERF4_03	ERF4	249	259	1	1	0.919	gcGCCGCtcc
OsSK24	P$DBF2_01	DBF2	249	259	1	1	0.948	gcGCCGCtcc
OsSK24	P$AT5G25190_01	AT5G25190	249	259	1	1	0.851	gcGCCGCtcc
OsSK24	P$ABR1_01	ABR1	249	259	1	1	1	gcGCCGCtcc
OsSK24	P$AT5G11190_01	AT5G11190	249	259	1	1	0.851	gcGCCGCtcc
OsSK24	P$AT3G61630_01	AT3G61630	249	259	1	1	0.884	gcGCCGCtcc
OsSK24	P$AT5G43410_01	AT5G43410	249	259	1	1	0.908	gcGCCGCtcc
OsSK24	P$TINY2_02	TINY2	249	259	1	1	0.908	gcGCCGCtcc
OsSK24	P$AT3G16280_01	AT3G16280	249	259	1	1	0.986	gcGCCGCtcc
OsSK24	P$CRF2_01	CRF2	249	257	1	1	0.949	gcGCCGCt
OsSK24	P$ERF098_01	ERF098	249	257	1	1	0.895	gcGCCGCt
OsSK24	P$EREBP1_01	EREBP1	249	259	1	1	0.855	gcGCCGCtcc
OsSK24	P$DREBIII1_01	DREBIII1	249	259	1	1	0.932	gcGCCGCtcc
OsSK24	P$CRF4_01	CRF4	249	257	1	1	0.929	gCGCCGct
OsSK24	P$ERF4_04	ERF4	249	257	1	1	0.897	gCGCCGct
OsSK24	P$ERF069_01	ERF069	249	258	1	1	0.994	gCGCCGctc
OsSK24	P$ERF11_01	ERF11	249	259	1	1	0.97	gCGCCGctcc
OsSK24	P$ERF13_01	ERF13	249	259	1	1	0.85	gcGCCGCtcc
OsSK24	P$PTI5_01	TI5	249	259	1	1	0.852	gcGCCGCtcc
OsSK24	P$AT1G77200_01	AT1G77200	249	259	1	1	0.941	gcGCCGCtcc
OsSK24	P$ATERF14_01	ATERF14	249	259	1	1	0.908	gcGCCGCtcc
OsSK24	P$DREBIII3_01	DREBIII3	249	259	1	1	0.931	gcGCCGCtcc
OsSK24	P$DREBIII2_01	DREBIII2	249	259	1	1	0.93	gcGCCGCtcc
OsSK24	P$ERF4_02	ERF4	249	259	1	1	0.901	gcGCCGCtcc
OsSK24	P$AT5G25390_01	AT5G25390	249	259	1	1	0.86	gcGCCGCtcc
OsSK24	P$ERF8_01	ERF8	250	260	1	1	0.95	CGCCGctcct
OsSK24	P$ERF7_02	ERF7	250	260	1	1	0.947	cGCCGCtcct
OsSK24	P$AT5G61590_01	AT5G61590	254	264	1	1	0.897	GCTCCtcctc
OsSK24	P$BZIP68_01	BZIP68	274	283	1	1	0.926	cacCGTGGt
OsSK24	P$AT5G61890_01	AT5G61890	292	302	1	1	0.9	aaGCCGGcta
OsSK24	P$ERF6_02	ERF6	292	302	1	1	0.979	aaGCCGGcta
OsSK24	P$ERF105_02	ERF105	293	301	1	1	0.929	aGCCGGct
OsSK24	P$BPC1_Q2	BPC1	305	311	1	1	1	AGAAAg
OsSK24	P$PBF_Q2	BF	306	312	1	1	0.965	gAAAGG
OsSK24	P$DOF1_01	DOF1	311	322	1	1	0.983	ggcTAAAGccc
OsSK24	P$DOF2_01	DOF2	311	322	1	1	0.993	ggctAAAGCcc
OsSK24	P$DOF3_01	DOF3	311	322	1	1	0.987	ggctAAAGCcc
OsSK24	P$AGL1_01	AGL1	316	334	1	1	0.87	aagcCCAAAtggggaaga
OsSK24	P$HSFA2_01	HSFA2	320	326	1	1	0.922	CCAAAt
OsSK24	P$BPC1_Q2	BPC1	333	339	1	1	0.99	AGAAAt
OsSK24	P$BPC1_Q2	BPC1	342	348	1	1	1	AGAAAg
OsSK24	P$PBF_Q2	BF	343	349	1	1	0.965	gAAAGG
OsSK24	P$ATHB6_01	ATHB6	356	365	1	1	0.909	taAATAAgg
OsSK24	P$KNOX3_01	KNOX3	374	386	1	1	0.965	agaaTGACAtgt
OsSK24	P$TCP14_01	TCP14	375	395	1	1	0.944	gaatgacatGTGGGtcccac
OsSK24	P$O2_Q4	O2	377	388	1	1	0.881	atgaCATGTgg
OsSK24	P$ATH1_01	ATH1	378	386	1	1	0.944	TGACAtgt
OsSK24	P$ALFIN1_Q2	ALFIN1	378	393	1	1	0.867	tgacatGTGGGtccc
OsSK24	P$AMS_01	AMS	379	389	1	1	0.867	gaCATGTggg
OsSK24	P$TCP11_01	TCP11	383	395	1	1	0.859	tGTGGGtcccac
OsSK24	P$TCP7_01	TCP7	384	395	1	1	0.999	GTGGGtcccac
OsSK24	P$TCP15_01	TCP15	384	394	1	1	0.999	GTGGGtccca
OsSK24	P$TCP20_01	TCP20	384	394	1	1	0.99	GTGGGtccca
OsSK24	P$TCP11_02	TCP11	384	394	1	1	0.868	GTGGGtccca
OsSK24	P$TCP19_01	TCP19	385	395	1	1	0.995	tgggtCCCAC
OsSK24	P$PCF2_01	CF2	385	395	1	1	1	tgggtCCCAC
OsSK24	P$PCF5_01	CF5	385	395	1	1	0.886	tgGGTCCcac
OsSK24	P$TCP20L_01	TCP20L	386	395	1	1	0.994	gggtCCCAC
OsSK24	P$ARALY484486_05	ARALY484486	387	395	1	1	0.963	ggtCCCAC
OsSK24	P$ARALY493022_04	ARALY493022	387	395	1	1	0.946	ggtCCCAC
OsSK24	P$ARALY495258_02	ARALY495258	387	395	1	1	0.963	ggtCCCAC
OsSK24	P$TCP20_02	TCP20	387	397	1	1	0.991	ggtCCCACat
OsSK24	P$OSI_01	OSI	387	395	1	1	0.959	ggtCCCAC
OsSK24	P$LEC2_01	LEC2	392	403	1	1	0.938	caCATGCtgac
OsSK24	P$AT5G26170_01	AT5G26170	401	410	1	1	0.877	acTCAACgg
OsSK24	P$AT5G54070_01	AT5G54070	403	409	1	1	0.91	tCAACG
OsSK24	P$LEC2_01	LEC2	420	431	1	1	0.938	gaCATGCcacg
OsSK24	P$HBP1B_Q6	HBP1B	421	435	1	1	0.913	acatgccaCGTCAg

OsSK24	P$ABF2_01	ABF2	422	435	1	1	0.907	catgcCACGTcag
OsSK24	P$O2_Q4	O2	423	434	1	1	0.982	atgcCACGTca
OsSK24	P$O2_04	O2	423	436	1	1	0.977	atGCCACgtcagc
OsSK24	P$ABF4_01	ABF4	424	436	1	1	0.885	tgcCACGTcagc
OsSK24	P$GBP_Q6	GBP	424	436	1	1	0.928	tgcCACGTcagc
OsSK24	P$ABI5_01	ABI5	424	434	1	1	0.936	tgcCACGTca
OsSK24	P$GBF6_01	GBF6	424	439	1	1	0.984	tgcCACGTcagcgaa
OsSK24	P$BEE2_01	BEE2	425	435	1	1	0.906	gcCACGTcag
OsSK24	P$BIM3_01	BIM3	425	435	1	1	0.876	gcCACGTcag
OsSK24	P$PHYPA143875_02	HYPA143875	425	435	1	1	0.871	gcCACGTcag
OsSK24	P$SPT_01	SPT	425	434	1	1	0.945	gcCACGTca
OsSK24	P$GBF1F_Q2	GBF1F	425	436	1	1	0.978	gcCACGTcagc
OsSK24	P$BZIP43_01	BZIP43	425	437	1	1	0.98	gcCACGTcagcg
OsSK24	P$BZIP48_01	BZIP48	425	439	1	1	0.982	gcCACGTcagcgaa
OsSK24	P$HBPA1_Q6_01	HBPA1	425	435	1	1	0.986	gccaCGTCAg
OsSK24	P$TGA1B_01	TGA1B	425	435	1	1	0.936	gcCACGTcag
OsSK24	P$CPRF2_01	CPRF2	425	435	1	1	0.969	gcCACGTcag
OsSK24	P$EMBP1_Q2	EMBP1	425	435	1	1	0.959	gcCACGTcag
OsSK24	P$CPRF3_Q2	CPRF3	425	435	1	1	0.971	gcCACGTcag
OsSK24	P$CPRF2_Q2	CPRF2	425	435	1	1	0.969	gcCACGTcag
OsSK24	P$O2_02	O2	425	435	1	1	0.979	gcCACGTcag
OsSK24	P$CPRF3_01	CPRF3	425	435	1	1	0.979	gcCACGTcag
OsSK24	P$TGA1A_Q2	TGA1A	425	435	1	1	0.992	gcCACGTcag
OsSK24	P$TGA1B_Q2	TGA1B	425	435	1	1	0.979	gcCACGTcag
OsSK24	P$RITA1_01	RITA1	426	433	1	1	0.976	cCACGTc
OsSK24	P$OCSBF1_01	OCSBF1	427	432	1	1	1	CACGT
OsSK24	P$TGA1A_01	TGA1A	427	434	1	1	0.989	cACGTCa
OsSK24	P$TGA2_Q2	TGA2	428	438	1	1	0.878	aCGTCAgcga
OsSK24	P$C1_Q2	C1	437	448	1	1	0.947	aaAACTAccat
OsSK24	P$P_01		439	448	1	1	0.92	aaCTACCat
OsSK24	P$MYB24_01	MYB24	453	462	1	1	0.918	ctgTTAGGg
OsSK24	P$WRKY60_01	WRKY60	461	472	1	1	0.903	ggaGTCAAatt
OsSK24	P$WRKY57_01	WRKY57	461	471	1	1	0.981	ggaGTCAAat
OsSK24	P$WRKY48_02	WRKY48	461	471	1	1	0.995	ggaGTCAAat
OsSK24	P$WRKY21_02	WRKY21	461	471	1	1	0.969	ggaGTCAAat
OsSK24	P$WRKY18_02	WRKY18	461	471	1	1	0.978	ggaGTCAAat
OsSK24	P$WRKY40_03	WRKY40	461	471	1	1	0.994	ggAGTCAaat
OsSK24	P$WRKY8_01	WRKY8	462	471	1	1	0.99	gaGTCAAat
OsSK24	P$WRKY75_01	WRKY75	462	470	1	1	0.976	gaGTCAAa
OsSK24	P$WRKY63_01	WRKY63	462	470	1	1	0.904	gaGTCAAa
OsSK24	P$WRKY62_01	WRKY62	462	470	1	1	0.866	gaGTCAAa
OsSK24	P$WRKY43_02	WRKY43	462	472	1	1	0.988	gaGTCAAatt
OsSK24	P$WRKY40_01	WRKY40	462	470	1	1	0.996	gaGTCAAa
OsSK24	P$WRKY25_02	WRKY25	462	470	1	1	0.914	gaGTCAAa
OsSK24	P$WRKY2_01	WRKY2	462	470	1	1	0.94	gaGTCAAa
OsSK24	P$WRKY15_01	WRKY15	462	472	1	1	0.979	gaGTCAAatt
OsSK24	P$WRKY23_01	WRKY23	463	471	1	1	0.88	aGTCAAat
OsSK24	P$WRKY30_01	WRKY30	463	473	1	1	0.915	aGTCAAatta
OsSK24	P$WRKY18_Q2	WRKY18	464	473	1	1	0.993	GTCAAatta
OsSK24	P$BHLH64_02	BHLH64	474	480	1	1	1	ACCAGt
OsSK24	P$SPF1_Q2	SPF1	481	491	1	1	0.869	taATAGTtgg
OsSK24	P$ARR18_01	ARR18	494	507	1	1	0.904	gttaAGATAtctg
OsSK24	P$RIN_Q2	RIN	548	559	1	1	0.885	tataTTTAAgg
OsSK24	P$DOF1_01	DOF1	560	571	1	1	0.984	agtTAAAGtgg
OsSK24	P$MYBAS1_01	MYBAS1	576	587	1	1	0.947	ttCCAACacaa
OsSK24	P$RAV1_01	RAV1	576	588	1	1	0.966	ttcCAACAcaaa
OsSK24	P$SBF1_01	SBF1	590	604	1	1	0.851	tataccTTAAAaga
OsSK24	P$PBF_01	BF	595	606	1	1	0.985	cttAAAAGagc
OsSK24	P$DOF_Q2	DOF	595	606	1	1	0.935	cttAAAAGagc
OsSK24	P$CDF2_01	CDF2	596	606	1	1	0.951	ttAAAAGagc
OsSK24	P$CDF3_01	CDF3	597	606	1	1	0.979	tAAAAGagc
OsSK24	P$LEC2_01	LEC2	615	626	1	1	0.941	taCATGCctta
OsSK24	P$AMS_01	AMS	637	647	1	1	0.962	gcCATGTggc
OsSK24	P$ATMYB15_Q2	ATMYB15	650	656	1	1	0.865	TAACAg
OsSK24	P$CBF3_02	CBF3	677	691	1	1	0.915	tcttcCCGACccat
OsSK24	P$CBF1_04	CBF1	678	690	1	1	0.899	cttcCCGACcca
OsSK24	P$ARF5_01	ARF5	681	689	1	1	0.912	cCCGACcc
OsSK24	P$DREB1B_01	DREB1B	682	687	1	1	1	CCGAC
OsSK24	P$ASR1_01	ASR1	685	690	1	1	1	ACCCA
OsSK24	P$ASR1_01	ASR1	703	708	1	1	1	ACCCA
OsSK24	P$HSFA2_01	HSFA2	705	711	1	1	0.933	CCAAAg
OsSK24	P$NAC6_01	NAC6	727	733	1	1	0.854	tCGTAA
OsSK24	P$TSAR2_01	TSAR2	768	778	1	1	0.93	cGCACGagct
OsSK24	P$BHLH78_01	BHLH78	769	777	1	1	0.875	GCACGagc
OsSK24	P$E2FA_02	E2FA	786	796	1	1	0.985	cagCGCCAgt
OsSK24	P$E2FA_02	E2FA	794	804	1	1	0.986	gtgCGCCAct
OsSK24	P$ATHB6_01	ATHB6	807	816	1	1	0.981	gtAATAAta
OsSK24	P$ATHB5_04	ATHB5	807	818	1	1	0.9	gtAATAAtatc

OsSK24	P$ATHB1_03	ATHB1	807	818	1	1	0.885	gtAATAAtatc
OsSK24	P$ATHB16_01	ATHB16	808	816	1	1	0.915	tAATAAta
OsSK24	P$AT3G51080_01	AT3G51080	828	835	1	1	1	GGAAAaa
OsSK24	P$PBF_01	BF	828	839	1	1	0.961	ggaAAAAGaaa
OsSK24	P$DOF_Q2	DOF	828	839	1	1	0.994	ggaAAAAGaaa
OsSK24	P$FLC_01	FLC	829	850	1	1	0.876	gaaaaagaaaaagAGAAAaaa
OsSK24	P$CDF2_01	CDF2	829	839	1	1	0.977	gaAAAAGaaa
OsSK24	P$CDF3_01	CDF3	830	839	1	1	0.975	aAAAAGaaa
OsSK24	P$BPC1_Q2	BPC1	834	840	1	1	0.997	AGAAAa
OsSK24	P$AP1_01	AP1	834	847	1	1	0.923	agAAAAAgagaaa
OsSK24	P$PBF_01	BF	834	845	1	1	0.982	agaAAAAGaga
OsSK24	P$DOF_Q2	DOF	834	845	1	1	0.985	agaAAAAGaga
OsSK24	P$CDF2_01	CDF2	835	845	1	1	0.979	gaAAAAGaga
OsSK24	P$CDF3_01	CDF3	836	845	1	1	0.984	aAAAAGaga
OsSK24	P$FLC_01	FLC	839	860	1	1	0.885	aagagaaaaaaaaAGAAAaaa
OsSK24	P$BPC1_Q2	BPC1	842	848	1	1	0.997	AGAAAa
OsSK24	P$AP1_01	AP1	844	857	1	1	0.939	aaAAAAAaagaaa
OsSK24	P$AP1_01	AP1	845	858	1	1	0.859	aaAAAAAagaaaa
OsSK24	P$PBF_01	BF	846	857	1	1	0.979	aaaAAAAGaaa
OsSK24	P$DOF_Q2	DOF	846	857	1	1	1	aaaAAAAGaaa
OsSK24	P$CDF2_01	CDF2	847	857	1	1	0.978	aaAAAAGaaa
OsSK24	P$CDF3_01	CDF3	848	857	1	1	0.975	aAAAAGaaa
OsSK24	P$BPC1_Q2	BPC1	852	858	1	1	0.997	AGAAAa
OsSK24	P$PBF_01	BF	854	865	1	1	0.992	aaaAAAAGagc
OsSK24	P$DOF_Q2	DOF	854	865	1	1	0.986	aaaAAAAGagc
OsSK24	P$CDF2_01	CDF2	855	865	1	1	0.979	aaAAAAGagc
OsSK24	P$CDF3_01	CDF3	856	865	1	1	0.984	aAAAAGagc
OsSK24	P$ARF8_01	ARF8	864	873	1	1	0.953	cgTGTCGct
OsSK22	P$MYB24_01	MYB24	2	11	1	1	0.883	taaTTAGGg
OsSK22	P$OJ1581_01	OJ1581	6	16	1	1	0.968	taGGGCCctt
OsSK22	P$TCP2_01	TCP2	6	16	1	1	0.941	taGGGCCctt
OsSK22	P$SED_Q2	SED	8	18	1	1	0.901	gggcCCTTTg
OsSK22	P$PBF_Q2_01	BF	12	18	1	1	0.988	CCTTTg
OsSK22	P$AT3G51080_01	AT3G51080	22	29	1	1	1	GGAAAaa
OsSK22	P$BPC1_Q2	BPC1	35	41	1	1	0.99	AGAAAt
OsSK22	P$EDT1_01	EDT1	49	59	1	1	0.854	attTTAATcc
OsSK22	P$AT3G51080_01	AT3G51080	63	70	1	1	0.893	GGAAAat
OsSK22	P$SED_Q2	SED	78	88	1	1	0.912	aaggCCTTTa
OsSK22	P$PBF_Q2_01	BF	82	88	1	1	0.998	CCTTTa
OsSK22	P$ARR1_01	ARR1	97	107	1	1	0.946	attGAATCat
OsSK22	P$ATHB7_01	ATHB7	99	109	1	1	0.915	tgAATCAtat
OsSK22	P$HAT1_01	HAT1	99	109	1	1	0.969	tgAATCAtat
OsSK22	P$SED_Q2	SED	110	120	1	1	0.954	ttatCCTTTg
OsSK22	P$PBF_Q2_01	BF	114	120	1	1	0.988	CCTTTg
OsSK22	P$ARR18_01	ARR18	143	156	1	1	0.903	tcatAGATAtttt
OsSK22	P$BPC1_Q2	BPC1	158	164	1	1	0.997	AGAAAa
OsSK22	P$GAMYB_01	GAMYB	178	186	1	1	0.86	CAACCtct
OsSK22	P$SED_Q2	SED	192	202	1	1	0.953	ttttCCTTTg
OsSK22	P$PBF_Q2_01	BF	196	202	1	1	0.988	CCTTTg
OsSK22	P$AT5G26170_01	AT5G26170	212	221	1	1	0.931	tcTCAACcg
OsSK22	P$GAMYB_01	GAMYB	215	223	1	1	0.926	CAACCgat
OsSK22	P$PCF5_01	CF5	238	248	1	1	0.889	gtGGTCCaaa
OsSK22	P$HSFA2_01	HSFA2	243	249	1	1	0.941	CCAAAc
OsSK22	P$CBNAC_01	CBNAC	263	269	1	1	0.979	gTGCTT
OsSK22	P$CBNAC_02	CBNAC	263	279	1	1	0.871	gTGCTTttcatgtatt
OsSK22	P$O2_Q4	O2	267	278	1	1	0.914	ttttCATGTat
OsSK22	P$TEIL_01	TEIL	272	280	1	1	0.925	ATGTAttt
OsSK22	P$ARR2_01	ARR2	280	290	1	1	0.866	tgtaATCTTc
OsSK22	P$HSFA4A_01	HSFA4A	307	313	1	1	0.964	cCTATT
OsSK22	P$HSFA4A_01	HSFA4A	329	335	1	1	0.964	cCTATT
OsSK22	P$PBF_01	BF	358	369	1	1	0.982	tttAAAAGggc
OsSK22	P$DOF_Q2	DOF	358	369	1	1	0.932	tttAAAAGggc
OsSK22	P$CDF2_01	CDF2	359	369	1	1	0.956	ttAAAAGggc
OsSK22	P$CDF3_01	CDF3	360	369	1	1	0.983	tAAAAGggc
OsSK22	P$PBF_Q2	BF	361	367	1	1	1	aAAAGG
OsSK22	P$OJ1581_01	OJ1581	363	373	1	1	0.967	aaGGGCCctt
OsSK22	P$TCP2_01	TCP2	363	373	1	1	0.942	aaGGGCCctt
OsSK22	P$SBF1_01	SBF1	365	379	1	1	0.89	gggcccTTAATatg
OsSK22	P$ABI3_01	ABI3	394	403	1	1	0.862	taGCATGac
OsSK22	P$P_01		435	444	1	1	0.878	ctCTACCct
OsSK22	P$MYB1L_01	MYB1L	438	448	1	1	0.992	taCCCTAcca
OsSK22	P$TRB2_01	TRB2	438	446	1	1	0.959	taCCCTAc
OsSK22	P$P_01		440	449	1	1	0.95	ccCTACCat
OsSK22	P$GAMYB_Q2	GAMYB	445	458	1	1	0.902	ccatcACAACcaa
OsSK22	P$AT4G36620_01	AT4G36620	449	457	1	1	0.902	cacAACCA
OsSK22	P$GAMYB_01	GAMYB	451	459	1	1	0.879	CAACCaat
OsSK22	P$ATSPL8_01	ATSPL8	459	475	1	1	0.902	agagtTGTACtgaact
OsSK22	P$HMG1_01	HMG1	462	471	1	1	0.933	GTTGTactg

OsSK22	P$SED_Q2	SED	491	501	1	1	0.91	tggcCCTTTt
OsSK22	P$PBF_Q2_01	BF	495	501	1	1	1	CCTTTt
OsSK22	P$ATHB1_01	ATHB1	501	515	1	1	0.945	gtaaaATTATttta
OsSK22	P$ATHB5_01	ATHB5	504	513	1	1	0.915	aaaTTATTt
OsSK22	P$PBF_01	BF	528	539	1	1	0.965	tcgAAAAGtaa
OsSK22	P$DOF_Q2	DOF	528	539	1	1	0.957	tcgAAAAGtaa
OsSK22	P$CDF2_01	CDF2	529	539	1	1	0.971	cgAAAAGtaa
OsSK22	P$CDF3_01	CDF3	530	539	1	1	0.986	gAAAAGtaa
OsSK22	P$MYB80_01	MYB80	541	552	1	1	0.855	tgGAATAtgga
OsSK22	P$TGA1_01	TGA1	559	570	1	1	0.935	tctTGACGcca
OsSK22	P$WRKY11_Q2	WRKY11	560	568	1	1	0.897	cTTGACgc
OsSK22	P$TGA7_01	TGA7	560	570	1	1	0.902	ctTGACGcca
OsSK22	P$TGA5_01	TGA5	561	569	1	1	0.87	tTGACGcc
OsSK22	P$E2FA_02	E2FA	562	572	1	1	0.85	tgaCGCCAaa
OsSK22	P$HSFA2_01	HSFA2	567	573	1	1	0.933	CCAAAg
OsSK22	P$GT1_Q6_01	GT1	573	585	1	1	0.89	TTTTTatatacg
OsSK22	P$HBPA1_Q6_01	HBPA1	583	593	1	1	0.868	cgaaCGTCAg
OsSK22	P$TGA1A_01	TGA1A	585	592	1	1	0.989	aACGTCa
OsSK22	P$PCF5_01	CF5	601	611	1	1	0.885	tgGGTCCcca
OsSK22	P$PCF2_01	CF2	602	612	1	1	0.891	gggtcCCCAC
OsSK22	P$TCP19_01	TCP19	602	612	1	1	0.911	gggtcCCCAC
OsSK22	P$TCP20L_01	TCP20L	603	612	1	1	0.889	ggtcCCCAC
OsSK22	P$TCP20_02	TCP20	604	614	1	1	0.909	gtcCCCACat
OsSK22	P$ARALY495258_02	ARALY495258	604	612	1	1	0.933	gtcCCCAC
OsSK22	P$ARALY484486_05	ARALY484486	604	612	1	1	0.933	gtcCCCAC
OsSK22	P$WEREWOLF_Q2	WEREWOLF	612	621	1	1	0.949	atGTTAGtg
OsSK22	P$GATA15_01	GATA15	617	626	1	1	0.999	agTGATCca
OsSK22	P$ABF2_01	ABF2	619	632	1	1	0.915	tgatcCACGTaaa
OsSK22	P$O2_Q4	O2	620	631	1	1	0.933	gatcCACGTaa
OsSK22	P$GBP_Q6	GBP	621	633	1	1	0.922	atcCACGTaaaa
OsSK22	P$ABI5_01	ABI5	621	631	1	1	0.916	atcCACGTaa
OsSK22	P$ABF4_01	ABF4	621	633	1	1	0.885	atcCACGTaaaa
OsSK22	P$EMBP1_Q2	EMBP1	622	632	1	1	0.892	tcCACGTaaa
OsSK22	P$CPRF3_Q2	CPRF3	622	632	1	1	0.926	tcCACGTaaa
OsSK22	P$CPRF2_Q2	CPRF2	622	632	1	1	0.949	tcCACGTaaa
OsSK22	P$O2_02	O2	622	632	1	1	0.988	tcCACGTaaa
OsSK22	P$TGA1B_Q2	TGA1B	622	632	1	1	0.903	tcCACGTaaa
OsSK22	P$TGA1A_Q2	TGA1A	622	632	1	1	0.969	tcCACGTaaa
OsSK22	P$CPRF3_01	CPRF3	622	632	1	1	0.932	tcCACGTaaa
OsSK22	P$CPRF2_01	CPRF2	622	632	1	1	0.95	tcCACGTaaa
OsSK22	P$BEE2_01	BEE2	622	632	1	1	0.915	tcCACGTaaa
OsSK22	P$BIM2_01	BIM2	622	632	1	1	0.854	tcCACGTaaa
OsSK22	P$BIM3_01	BIM3	622	632	1	1	0.888	tcCACGTaaa
OsSK22	P$PHYPA143875_02	HYPA143875	622	632	1	1	0.875	tcCACGTaaa
OsSK22	P$SPT_01	SPT	622	631	1	1	0.941	tcCACGTaa
OsSK22	P$GBF1F_Q2	GBF1F	622	633	1	1	0.954	tcCACGTaaaa
OsSK22	P$NAC043_01	NAC043	622	632	1	1	0.856	tccACGTAaa
OsSK22	P$RITA1_01	RITA1	623	630	1	1	0.987	cCACGTa
OsSK22	P$OCSBF1_01	OCSBF1	624	629	1	1	1	CACGT
OsSK22	P$NAC025_01	NAC025	624	632	1	1	0.95	cACGTAaa
OsSK22	P$NAC6_01	NAC6	625	631	1	1	1	aCGTAA
OsSK22	P$PBF_01	BF	626	637	1	1	0.955	cgtAAAAGaag
OsSK22	P$DOF_Q2	DOF	626	637	1	1	0.947	cgtAAAAGaag
OsSK22	P$CDF2_01	CDF2	627	637	1	1	0.95	gtAAAAGaag
OsSK22	P$CDF3_01	CDF3	628	637	1	1	0.969	tAAAAGaag
OsSK22	P$PEND_01	END	630	638	1	1	0.891	aAAGAAgt
OsSK22	P$GT1_Q6_02	GT1	631	643	1	1	0.85	aagaagTTAACg
OsSK22	P$MYB76_01	MYB76	635	648	1	1	0.893	agttAACGGcccc
OsSK22	P$AT5G55020_01	AT5G55020	636	648	1	1	0.862	gttAACGGcccc
OsSK22	P$AT1G26780_01	AT1G26780	636	648	1	1	0.85	gttAACGGcccc
OsSK22	P$SED_Q2	SED	642	652	1	1	0.902	ggccCCTTTg
OsSK22	P$PBF_Q2_01	BF	646	652	1	1	0.988	CCTTTg
OsSK22	P$DOF1_01	DOF1	652	663	1	1	0.986	aatTAAAGgat
OsSK22	P$PBF_Q2	BF	655	661	1	1	0.986	tAAAGG
OsSK22	P$UIF1_01	UIF1	679	689	1	1	0.856	tagGATTCaa
OsSK22	P$ATHSFA1D_01	ATHSFA1D	691	697	1	1	0.985	cCTACA
OsSK22	P$AGL27_01	AGL27	703	717	1	1	0.855	tTTTCTatttggcc
OsSK22	P$MRP1_Q2	MRP1	704	716	1	1	0.852	ttTCTATttggc
OsSK22	P$HSFA4A_01	HSFA4A	706	712	1	1	0.914	tCTATT
OsSK22	P$SED_Q2	SED	712	722	1	1	0.883	tggcCCTTTg
OsSK22	P$PBF_Q2_01	BF	716	722	1	1	0.988	CCTTTg
OsSK22	P$PBF_Q2	BF	725	731	1	1	0.958	cAAAGG
OsSK22	P$DOF2_01	DOF2	727	738	1	1	0.982	aaggAAAGCtt
OsSK22	P$DOF3_01	DOF3	727	738	1	1	0.984	aaggAAAGCtt
OsSK22	P$HSFA2_01	HSFA2	739	745	1	1	0.922	CCAAAt
OsSK22	P$KNOX3_01	KNOX3	761	773	1	1	0.969	ggaaTGACAcat
OsSK22	P$ATH1_01	ATH1	765	773	1	1	0.915	TGACAcat
OsSK22	P$O2_Q4	O2	771	782	1	1	0.892	attgCATGTgg

OsSK22	P$ABI3_01	ABI3	772	781	1	1	0.867	ttGCATGtg
OsSK22	P$AMS_01	AMS	773	783	1	1	0.884	tgCATGTgga
OsSK22	P$MYBAS1_01	MYBAS1	806	817	1	1	0.988	ctCCAACttct
OsSK22	P$AT3G51080_01	AT3G51080	818	825	1	1	0.893	GGAAAat
OsSK22	P$SED_Q2	SED	823	833	1	1	0.971	atttCCTTTg
OsSK22	P$PBF_Q2_01	BF	827	833	1	1	0.988	CCTTTg
OsSK22	P$PEND_02	END	902	912	1	1	0.912	caTTCTTatc
OsSK22	P$ARR1_01	ARR1	910	920	1	1	0.954	tcaGAATCct
OsSK22	P$HSFA4A_01	HSFA4A	930	936	1	1	0.964	cCTATT
OsSK22	P$HSFA4A_01	HSFA4A	938	944	1	1	0.914	tCTATT
OsSK22	P$SQUA_01	SQUA	939	949	1	1	0.915	ctaTTTTTtt
OsSK22	P$GT1_Q6_01	GT1	942	954	1	1	0.96	TTTTTtttcaac
OsSK22	P$GAMYB_01	GAMYB	950	958	1	1	0.935	CAACCctc
OsSK22	P$PBF_Q2	BF	962	968	1	1	0.958	cAAAGG
OsSK22	P$ATHB6_01	ATHB6	971	980	1	1	0.908	taAATAAga
OsSK22	P$C1_Q2	C1	980	991	1	1	0.945	tgAACTActtt
OsSK22	P$BBM_01	BBM	995	1005	1	1	0.948	gGGCGCcaac
OsSK22	P$PLT1_01	LT1	995	1005	1	1	0.918	gGGCGCcaac
OsSK22	P$E2FA_02	E2FA	995	1005	1	1	0.992	gggCGCCAac
OsSK22	P$MYBAS1_01	MYBAS1	998	1009	1	1	0.982	cgCCAACacac
OsSK22	P$RAV1_01	RAV1	998	1010	1	1	0.955	cgcCAACAcaca
OsSK22	P$GAMYB_Q2	GAMYB	1002	1015	1	1	0.884	aacacACAACatg
OsSK22	P$RAV1_01	RAV1	1005	1017	1	1	0.922	acaCAACAtgtg
OsSK22	P$AMS_01	AMS	1009	1019	1	1	0.886	aaCATGTgtg
OsSK22	P$AT4G36620_01	AT4G36620	1018	1026	1	1	0.884	ggcAACCA
OsSK22	P$GAMYB_01	GAMYB	1020	1028	1	1	0.888	CAACCaag
OsSK22	P$MYB24_01	MYB24	1025	1034	1	1	0.923	aagTTAGGa
OsSK22	P$ATSPL8_01	ATSPL8	1039	1055	1	1	0.937	gtatgTGTACatgatg
OsSK22	P$CBF1_01	CBF1	1052	1062	1	1	0.853	aTGCCGagca
OsSK22	P$ERF019_01	ERF019	1052	1062	1	1	0.897	aTGCCGagca
OsSK22	P$JERF1_01	JERF1	1052	1062	1	1	0.872	aTGCCGagca
OsSK22	P$CBF1_03	CBF1	1052	1062	1	1	0.88	aTGCCGagca
OsSK22	P$AT1G33760_01	AT1G33760	1052	1062	1	1	0.873	aTGCCGagca
OsSK22	P$AT1G71520_01	AT1G71520	1052	1062	1	1	0.881	aTGCCGagca
OsSK22	P$ALFIN1_Q2	ALFIN1	1080	1095	1	1	0.867	tgtagtGTGGGatcg
OsSK22	P$MYBAS1_01	MYBAS1	1106	1117	1	1	0.953	gaCCAACccta
OsSK22	P$GAMYB_01	GAMYB	1109	1117	1	1	0.883	CAACCcta
OsSK22	P$MYB1L_01	MYB1L	1110	1120	1	1	0.992	aaCCCTAcaa
OsSK22	P$TRB2_01	TRB2	1110	1118	1	1	0.957	aaCCCTAc
OsSK22	P$ATHSFA1D_01	ATHSFA1D	1113	1119	1	1	0.985	cCTACA
OsSK22	P$CBNAC_01	CBNAC	1119	1125	1	1	0.968	aTGCTT
OsSK22	P$CBNAC_02	CBNAC	1119	1135	1	1	0.854	aTGCTTgtgcatgcgg
OsSK22	P$ABI3_01	ABI3	1125	1134	1	1	0.962	gtGCATGcg
OsSK22	P$FUS3_01	FUS3	1126	1135	1	1	0.981	tGCATGcgg
OsSK22	P$LEC2_01	LEC2	1126	1137	1	1	0.973	tgCATGCggat
OsSK22	P$ARF8_01	ARF8	1134	1143	1	1	0.956	gaTGTCGct
OsSK22	P$DRE1C_01	DRE1C	1135	1143	1	1	0.862	ATGTCgct
OsSK22	P$AT5G04240_01	AT5G04240	1163	1169	1	1	0.938	tGGCAC
OsSK22	P$PIL5_01	IL5	1166	1180	1	1	0.851	cacgctcgACGTGc
OsSK22	P$O2_Q2	O2	1168	1181	1	1	0.867	cgctcGACGTgct
OsSK22	P$ABZ1_01	ABZ1	1169	1183	1	1	0.885	gctcgACGTGcttg
OsSK22	P$TGA1B_01	TGA1B	1171	1181	1	1	0.882	tcGACGTgct
OsSK22	P$HBP1A_Q2	HBP1A	1171	1181	1	1	0.87	tcgACGTGct
OsSK22	P$TAF1_Q2	TAF1	1171	1181	1	1	0.929	tcgACGTGct
OsSK22	P$EMBP1_02	EMBP1	1171	1181	1	1	0.857	tcgACGTGct
OsSK22	P$TAF1_01	TAF1	1171	1181	1	1	0.953	tcgACGTGct
OsSK22	P$GBF1_01	GBF1	1172	1180	1	1	0.93	cgACGTGc
OsSK22	P$MYC4_01	MYC4	1172	1180	1	1	0.852	cgACGTGc
OsSK22	P$BIM1_02	BIM1	1172	1182	1	1	0.947	cgACGTGctt
OsSK22	P$ABF4_02	ABF4	1172	1182	1	1	0.948	cgACGTGctt
OsSK22	P$ABI5_Q2	ABI5	1174	1180	1	1	0.936	ACGTGc
OsSK22	P$BZR1_01	BZR1	1175	1181	1	1	0.902	CGTGCt
OsSK22	P$CBNAC_01	CBNAC	1176	1182	1	1	0.979	gTGCTT
OsSK22	P$CBNAC_02	CBNAC	1176	1192	1	1	0.859	gTGCTTgacgccaact
OsSK22	P$TGA1_01	TGA1	1178	1189	1	1	0.933	gctTGACGcca
OsSK22	P$WRKY11_Q2	WRKY11	1179	1187	1	1	0.897	cTTGACgc
OsSK22	P$TGA7_01	TGA7	1179	1189	1	1	0.902	ctTGACGcca
OsSK22	P$TGA5_01	TGA5	1180	1188	1	1	0.87	tTGACGcc
OsSK22	P$MYBAS1_01	MYBAS1	1184	1195	1	1	0.98	cgCCAACtaat
OsSK22	P$C1_Q2	C1	1186	1197	1	1	0.953	ccAACTAatgt
OsSK22	P$ABI4_01	ABI4	1206	1217	1	1	0.913	gttcaCCGCCa
OsSK22	P$AT3G01030_01	AT3G01030	1210	1219	1	1	0.909	ACCGCcatc
OsSK22	P$AT3G63350_01	AT3G63350	1211	1217	1	1	0.882	CCGCCa
OsSK22	P$PCF5_01	CF5	1236	1246	1	1	0.866	gcGGTCCaat
OsSK22	P$LFY_Q2	LFY	1239	1250	1	1	0.854	gtCCAATgagt
OsSK22	P$PDF2_01	DF2	1246	1257	1	1	0.887	gagtTAAATgg
OsSK22	P$ATHB6_01	ATHB6	1290	1299	1	1	0.909	gaAATAAgg
OsSK22	P$CDC5_01	CDC5	1296	1307	1	1	0.867	aggTCAGCgcc

OsSK22	P$AT5G04240_01	AT5G04240	1315	1321	1	1	0.976	cGGCAC
OsSK22	P$MYB1L_01	MYB1L	1323	1333	1	1	0.956	acCCCTAgcc
OsSK22	P$TRB2_01	TRB2	1323	1331	1	1	0.939	acCCCTAg
OsSK22	P$RRTF1_05	RRTF1	1408	1423	1	1	0.868	tggacctCGGCGcca
OsSK22	P$AT1G68550_03	AT1G68550	1412	1421	1	1	0.956	cctCGGCGc
OsSK22	P$BBM_01	BBM	1415	1425	1	1	0.861	cGGCGCcaaa
OsSK22	P$E2FA_02	E2FA	1415	1425	1	1	0.993	cggCGCCAaa
OsSK22	P$HSFA2_01	HSFA2	1420	1426	1	1	0.941	CCAAAc
OsSK22	P$UIF1_01	UIF1	1437	1447	1	1	0.856	gagGATTCaa
OsSK22	P$RAV1_02	RAV1	1444	1456	1	1	0.919	caaACCTGaaaa
OsSK22	P$PBF_01	BF	1449	1460	1	1	0.973	ctgAAAAGtaa
OsSK22	P$DOF_Q2	DOF	1449	1460	1	1	0.958	ctgAAAAGtaa
OsSK22	P$CDF2_01	CDF2	1450	1460	1	1	0.97	tgAAAAGtaa
OsSK22	P$CDF3_01	CDF3	1451	1460	1	1	0.986	gAAAAGtaa
OsSK22	P$ATHB7_01	ATHB7	1480	1490	1	1	0.874	aaAATCAagt
OsSK22	P$HAT1_01	HAT1	1480	1490	1	1	0.869	aaAATCAagt
OsSK22	P$AT5G04340_01	AT5G04340	1490	1502	1	1	0.907	ttttAGAAAagg
OsSK22	P$PBF_01	BF	1493	1504	1	1	0.98	tagAAAAGggg
OsSK22	P$DOF_Q2	DOF	1493	1504	1	1	0.944	tagAAAAGggg
OsSK22	P$BPC1_Q2	BPC1	1494	1500	1	1	0.997	AGAAAa
OsSK22	P$CDF2_01	CDF2	1494	1504	1	1	0.957	agAAAAGggg
OsSK22	P$CDF3_01	CDF3	1495	1504	1	1	0.982	gAAAAGggg
OsSK22	P$PBF_Q2	BF	1496	1502	1	1	1	aAAAGG
OsSK22	P$GT1_Q6	GT1	1511	1518	1	1	0.971	GTAAAaa
OsSK22	P$ATHB6_01	ATHB6	1515	1524	1	1	0.908	aaAATAAat
OsSK22	P$PDF2_01	DF2	1515	1526	1	1	0.863	aaaaTAAATcc
OsSK22	P$ARR1_01	ARR1	1533	1543	1	1	0.945	gggGAATCaa
OsSK22	P$ATHB7_01	ATHB7	1535	1545	1	1	0.873	ggAATCAaaa
OsSK22	P$HAT1_01	HAT1	1535	1545	1	1	0.867	ggAATCAaaa
OsSK22	P$AT4G36620_01	AT4G36620	1540	1548	1	1	0.895	caaAACCA
OsSK22	P$HSFA2_01	HSFA2	1545	1551	1	1	0.941	CCAAAc
OsSK22	P$AT2G41690_01	AT2G41690	1560	1566	1	1	0.978	CCGAAa
OsSK22	P$AT4G36620_01	AT4G36620	1562	1570	1	1	0.896	gaaAACCA
OsSK22	P$GATA9_01	GATA9	1578	1589	1	1	0.894	gccAGATCatg
OsSK22	P$AGP1_01	AGP1	1579	1589	1	1	0.865	ccAGATCatg
OsSK22	P$ARR10_01	ARR10	1581	1588	1	1	0.913	AGATCat
OsSK22	P$MYBAS1_01	MYBAS1	1589	1600	1	1	0.989	gcCCAACcccc
OsSK22	P$GAMYB_01	GAMYB	1592	1600	1	1	0.945	CAACCccc
OsSK22	P$MYBAS1_01	MYBAS1	1596	1607	1	1	0.985	ccCCAACattc
OsSK22	P$RAV1_01	RAV1	1596	1608	1	1	0.94	cccCAACAttca
OsSK22	P$HSFA2_01	HSFA2	1610	1616	1	1	1	CCAAAa
OsSK22	P$ASR1_01	ASR1	1615	1620	1	1	1	ACCCA
OsSK22	P$HSFA2_01	HSFA2	1617	1623	1	1	1	CCAAAa
OsSK22	P$WRKY11_Q2	WRKY11	1631	1639	1	1	0.974	aTTGACcg
OsSK22	P$ZAP1_01	ZAP1	1632	1642	1	1	0.948	TTGACcgaat
OsSK22	P$AT2G41690_01	AT2G41690	1636	1642	1	1	0.974	CCGAAt
OsSK22	P$AT3G60580_01	AT3G60580	1643	1650	1	1	0.873	ccATCCC
OsSK22	P$TGA1_01	TGA1	1650	1661	1	1	0.925	atcTGACGact
OsSK22	P$TGA7_01	TGA7	1651	1661	1	1	0.879	tcTGACGact
OsSK22	P$TGA5_01	TGA5	1652	1660	1	1	0.863	cTGACGac
OsSK22	P$MYBAS1_01	MYBAS1	1661	1672	1	1	0.943	gaCCAACattg
OsSK22	P$RAV1_01	RAV1	1661	1673	1	1	0.94	gacCAACAttgt
OsSK22	P$TCP24_01	TCP24	1690	1702	1	1	0.892	tgcaGGACCacc
OsSK22	P$ARALY897773_01	ARALY897773	1691	1701	1	1	0.87	gcaggACCAC
OsSK22	P$TCP4_01	TCP4	1693	1701	1	1	0.951	aGGACCac
OsSK22	P$TCP5_01	TCP5	1693	1701	1	1	0.866	aGGACCac
OsSK22	P$ARALY496250_03	ARALY496250	1693	1701	1	1	0.877	aGGACCac
OsSK22	P$LIM1_01	LIM1	1697	1709	1	1	0.918	CCACCaccaatc
OsSK22	P$LIM1_01	LIM1	1700	1712	1	1	0.91	CCACCaatcagc
OsSK22	P$ATHB7_01	ATHB7	1703	1713	1	1	0.922	ccAATCAgct
OsSK22	P$HAT1_01	HAT1	1703	1713	1	1	0.87	ccAATCAgct
OsSK22	P$ATSPL8_01	ATSPL8	1718	1734	1	1	0.901	ggttcTGTACacagtg
OsSK22	P$WRKY18_02	WRKY18	1743	1753	1	1	0.998	ttgGTCAAag
OsSK22	P$WRKY21_02	WRKY21	1743	1753	1	1	0.973	ttgGTCAAag
OsSK22	P$WRKY48_02	WRKY48	1743	1753	1	1	0.998	ttgGTCAAag
OsSK22	P$WRKY57_01	WRKY57	1743	1753	1	1	0.975	ttgGTCAAag
OsSK22	P$WRKY60_01	WRKY60	1743	1754	1	1	0.982	ttgGTCAAagc
OsSK22	P$WRKY15_01	WRKY15	1744	1754	1	1	0.987	tgGTCAAagc
OsSK22	P$WRKY2_01	WRKY2	1744	1752	1	1	0.989	tgGTCAAa
OsSK22	P$WRKY25_02	WRKY25	1744	1752	1	1	0.973	tgGTCAAa
OsSK22	P$WRKY40_01	WRKY40	1744	1752	1	1	1	tgGTCAAa
OsSK22	P$WRKY43_02	WRKY43	1744	1754	1	1	0.978	tgGTCAAagc
OsSK22	P$WRKY62_01	WRKY62	1744	1752	1	1	0.957	tgGTCAAa
OsSK22	P$WRKY63_01	WRKY63	1744	1752	1	1	0.99	tgGTCAAa
OsSK22	P$WRKY75_01	WRKY75	1744	1752	1	1	0.975	tgGTCAAa
OsSK22	P$WRKY8_01	WRKY8	1744	1753	1	1	0.993	tgGTCAAag
OsSK22	P$WRKY23_01	WRKY23	1745	1753	1	1	0.893	gGTCAAag
OsSK22	P$WRKY30_01	WRKY30	1745	1755	1	1	0.924	gGTCAAagca

OsSK22	P$DOF2_01	DOF2	1745	1756	1	1	0.98	ggtcAAAGCat
OsSK22	P$DOF3_01	DOF3	1745	1756	1	1	0.982	ggtcAAAGCat
OsSK22	P$WRKY18_Q2	WRKY18	1746	1755	1	1	0.95	GTCAAagca
OsSK22	P$CBF3_02	CBF3	1751	1765	1	1	0.944	agcatCCGACatgt
OsSK22	P$CBF1_04	CBF1	1752	1764	1	1	0.94	gcatCCGACatg
OsSK22	P$DREB1G_02	DREB1G	1753	1763	1	1	0.895	catCCGACat
OsSK22	P$AT1G77200_03	AT1G77200	1753	1767	1	1	0.878	catCCGACatgtgg
OsSK22	P$TCP14_01	TCP14	1754	1774	1	1	0.91	atccgacatGTGGGgcccac
OsSK22	P$ARF1_01	ARF1	1755	1763	1	1	0.931	tCCGACat
OsSK22	P$ARF5_01	ARF5	1755	1763	1	1	0.979	tCCGACat
OsSK22	P$DREB1B_01	DREB1B	1756	1761	1	1	1	CCGAC
OsSK22	P$ALFIN1_Q2	ALFIN1	1757	1772	1	1	0.93	cgacatGTGGGgccc
OsSK22	P$AMS_01	AMS	1758	1768	1	1	0.867	gaCATGTggg
OsSK22	P$TCP11_01	TCP11	1762	1774	1	1	0.859	tGTGGGgcccac
OsSK22	P$TCP15_01	TCP15	1763	1773	1	1	0.999	GTGGGgccca
OsSK22	P$TCP20_01	TCP20	1763	1773	1	1	0.991	GTGGGgccca
OsSK22	P$TCP11_02	TCP11	1763	1773	1	1	0.868	GTGGGgccca
OsSK22	P$TCP7_01	TCP7	1763	1774	1	1	0.999	GTGGGgcccac
OsSK22	P$OJ1581_01	OJ1581	1764	1774	1	1	0.994	tgGGGCCcac
OsSK22	P$TCP2_01	TCP2	1764	1774	1	1	0.974	tgGGGCCcac
OsSK22	P$PCF2_01	CF2	1764	1774	1	1	1	tggggCCCAC
OsSK22	P$TCP19_01	TCP19	1764	1774	1	1	1	tggggCCCAC
OsSK22	P$TCP20L_01	TCP20L	1765	1774	1	1	1	ggggCCCAC
OsSK22	P$OSI_01	OSI	1766	1774	1	1	1	gggCCCAC
OsSK22	P$TCP20_02	TCP20	1766	1776	1	1	1	gggCCCACat
OsSK22	P$ARALY495258_02	ARALY495258	1766	1774	1	1	1	gggCCCAC
OsSK22	P$ARALY493022_04	ARALY493022	1766	1774	1	1	1	gggCCCAC
OsSK22	P$ARALY484486_05	ARALY484486	1766	1774	1	1	1	gggCCCAC
OsSK22	P$CBNAC_01	CBNAC	1784	1790	1	1	0.979	gTGCTT
OsSK22	P$ATHSFA1D_01	ATHSFA1D	1791	1797	1	1	0.94	gCTACA
OsSK22	P$ATHSFA1D_01	ATHSFA1D	1798	1804	1	1	0.985	cCTACA
OsSK22	P$O2_Q4	O2	1819	1830	1	1	0.902	gatgCATGTga
OsSK22	P$ABI3_01	ABI3	1820	1829	1	1	0.857	atGCATGtg
OsSK22	P$AMS_01	AMS	1821	1831	1	1	0.894	tgCATGTgag
OsSK22	P$GL15_01	GL15	1842	1852	1	1	0.854	attttCCCCC
OsSK22	P$PEND_02	END	1851	1861	1	1	0.853	ccTTCTTttt
OsSK22	P$SPL14_02	SPL14	1864	1883	1	1	0.93	ttttttCCGTActgatttc
OsSK22	P$ATSPL3_01	ATSPL3	1866	1882	1	1	0.985	ttttcCGTACtgattt
OsSK22	P$SPL3_01	SPL3	1869	1877	1	1	0.998	tCCGTAct
OsSK22	P$SPL1_01	SPL1	1869	1879	1	1	0.891	tcCGTACtga
OsSK22	P$SPL14_03	SPL14	1869	1880	1	1	0.988	tcCGTACtgat
OsSK22	P$SPL14_01	SPL14	1870	1877	1	1	0.944	CCGTAct
OsSK22	P$ATHB6_01	ATHB6	1914	1923	1	1	0.923	gcAATAAac
OsSK22	P$AT3G20750_01	AT3G20750	1917	1925	1	1	0.907	aTAAACaa
OsSK22	P$CBF1_01	CBF1	1925	1935	1	1	0.94	gTGCCGccat
OsSK22	P$ERF019_01	ERF019	1925	1935	1	1	0.896	gTGCCGccat
OsSK22	P$DREB6_01	DREB6	1925	1935	1	1	0.915	gTGCCGccat
OsSK22	P$JERF3_01	JERF3	1925	1935	1	1	0.923	gTGCCGccat
OsSK22	P$DREB1_01	DREB1	1925	1935	1	1	0.914	gTGCCGccat
OsSK22	P$CEF1_01	CEF1	1925	1935	1	1	0.923	gTGCCGccat
OsSK22	P$JERF1_01	JERF1	1925	1935	1	1	0.957	gTGCCGccat
OsSK22	P$CBF1_03	CBF1	1925	1935	1	1	0.932	gTGCCGccat
OsSK22	P$AT1G71450_01	AT1G71450	1925	1935	1	1	0.877	gTGCCGccat
OsSK22	P$DREB1F_01	DREB1F	1925	1935	1	1	0.941	gTGCCGccat
OsSK22	P$AT1G33760_01	AT1G33760	1925	1935	1	1	0.907	gTGCCGccat
OsSK22	P$AT1G71520_01	AT1G71520	1925	1935	1	1	0.86	gTGCCGccat
OsSK22	P$AT4G28140_01	AT4G28140	1925	1935	1	1	0.905	gTGCCGccat
OsSK22	P$DREB1E_02	DREB1E	1925	1935	1	1	0.919	gTGCCGccat
OsSK22	P$ORA47_01	ORA47	1925	1935	1	1	0.873	gTGCCGccat
OsSK22	P$DREB2F_01	DREB2F	1925	1935	1	1	0.892	gTGCCGccat
OsSK22	P$ABI4_03	ABI4	1925	1935	1	1	0.903	gtGCCGCcat
OsSK22	P$WRAF1_01	WRAF1	1925	1935	1	1	0.922	gtGCCGCcat
OsSK22	P$WRAF2_01	WRAF2	1925	1935	1	1	0.892	gtGCCGCcat
OsSK22	P$PTI5_01	TI5	1925	1935	1	1	0.99	gtGCCGCcat
OsSK22	P$DREBI5_01	DREBI5	1925	1935	1	1	0.904	gtGCCGCcat
OsSK22	P$AT2G47520_01	AT2G47520	1925	1935	1	1	0.946	gtGCCGCcat
OsSK22	P$DREB2B_01	DREB2B	1925	1935	1	1	0.929	gtGCCGCcat
OsSK22	P$CRF1_02	CRF1	1925	1935	1	1	0.898	gtGCCGCcat
OsSK22	P$OPBP1_01	OPBP1	1925	1935	1	1	0.94	gtGCCGCcat
OsSK22	P$ATERF14_01	ATERF14	1925	1935	1	1	0.938	gtGCCGCcat
OsSK22	P$ERF112_01	ERF112	1925	1935	1	1	0.906	gtGCCGCcat
OsSK22	P$DREB2A_02	DREB2A	1925	1935	1	1	0.908	gtGCCGCcat
OsSK22	P$ERF1_02	ERF1	1925	1935	1	1	0.937	gtGCCGCcat
OsSK22	P$ERF4_02	ERF4	1925	1935	1	1	0.939	gtGCCGCcat
OsSK22	P$AT5G25390_01	AT5G25390	1925	1935	1	1	0.903	gtGCCGCcat
OsSK22	P$EREBP1_01	EREBP1	1925	1935	1	1	0.934	gtGCCGCcat
OsSK22	P$ERF110_02	ERF110	1925	1935	1	1	0.874	gtGCCGCcat
OsSK22	P$CBF3_01	CBF3	1925	1935	1	1	0.939	gtGCCGCcat

OsSK22	P$DREBII1_01	DREBII1	1925	1935	1	1	0.909	gtGCCGCcat
OsSK22	P$TSRF1_01	TSRF1	1925	1935	1	1	0.935	gtGCCGCcat
OsSK22	P$DRF13_01	DRF13	1925	1935	1	1	0.938	gtGCCGCcat
OsSK22	P$ERF2_03	ERF2	1925	1935	1	1	0.935	gtGCCGCcat
OsSK22	P$ERF1B_03	ERF1B	1925	1935	1	1	0.928	gtGCCGCcat
OsSK22	P$RAP26_02	RAP26	1925	1935	1	1	0.935	gtGCCGCcat
OsSK22	P$CBF5_01	CBF5	1925	1935	1	1	0.899	gtGCCGCcat
OsSK22	P$CBF16_01	CBF16	1925	1935	1	1	0.908	gtGCCGCcat
OsSK22	P$CBF17_01	CBF17	1925	1935	1	1	0.903	gtGCCGCcat
OsSK22	P$ERF1_05	ERF1	1925	1935	1	1	0.892	gtGCCGCcat
OsSK22	P$AT5G25190_01	AT5G25190	1925	1935	1	1	0.903	gtGCCGCcat
OsSK22	P$ERF105_01	ERF105	1925	1935	1	1	0.916	gtGCCGCcat
OsSK22	P$CBF_01	CBF	1925	1935	1	1	0.905	gtGCCGCcat
OsSK22	P$AT5G11190_01	AT5G11190	1925	1935	1	1	0.906	gtGCCGCcat
OsSK22	P$AT1G68550_01	AT1G68550	1925	1935	1	1	0.987	gtGCCGCcat
OsSK22	P$AT1G77640_01	AT1G77640	1925	1935	1	1	0.908	gtGCCGCcat
OsSK22	P$ERF016_01	ERF016	1925	1935	1	1	0.899	gtGCCGCcat
OsSK22	P$AT3G61630_01	AT3G61630	1925	1935	1	1	0.945	gtGCCGCcat
OsSK22	P$AT5G43410_01	AT5G43410	1925	1935	1	1	0.947	gtGCCGCcat
OsSK22	P$RAP26L_02	RAP26L	1925	1935	1	1	0.982	gtGCCGCcat
OsSK22	P$AT5G07310_01	AT5G07310	1925	1935	1	1	0.96	gtGCCGCcat
OsSK22	P$DREB1A_03	DREB1A	1925	1935	1	1	0.937	gtGCCGCcat
OsSK22	P$AT1G49120_01	AT1G49120	1925	1935	1	1	0.871	gtGCCGCcat
OsSK22	P$DREB2D_01	DREB2D	1925	1935	1	1	0.923	gtGCCGCcat
OsSK22	P$AT3G25890_01	AT3G25890	1925	1935	1	1	0.971	gtGCCGCcat
OsSK22	P$AT4G23750_01	AT4G23750	1925	1935	1	1	0.885	gtGCCGCcat
OsSK22	P$AT4G27950_01	AT4G27950	1925	1935	1	1	0.907	gtGCCGCcat
OsSK22	P$RRTF1_01	RRTF1	1925	1935	1	1	0.973	gtGCCGCcat
OsSK22	P$CEJ1_02	CEJ1	1925	1935	1	1	0.868	gtGCCGCcat
OsSK22	P$CRF2_01	CRF2	1925	1933	1	1	0.928	gtGCCGCc
OsSK22	P$ERF096_01	ERF096	1925	1935	1	1	0.985	gtGCCGCcat
OsSK22	P$ERF098_01	ERF098	1925	1933	1	1	0.997	gtGCCGCc
OsSK22	P$DREB2C_01	DREB2C	1925	1935	1	1	0.855	gtgCCGCCat
OsSK22	P$CBF1_02	CBF1	1925	1935	1	1	0.93	gtgCCGCCat
OsSK22	P$ERF1B_06	ERF1B	1926	1934	1	1	0.961	tGCCGCca
OsSK22	P$ERF7_02	ERF7	1926	1936	1	1	0.989	tGCCGCcatg
OsSK22	P$ERF094_01	ERF094	1926	1934	1	1	0.974	tGCCGCca
OsSK22	P$ERF2_01	ERF2	1926	1933	1	1	0.929	tgCCGCC
OsSK22	P$ERF13_02	ERF13	1926	1934	1	1	0.982	tgCCGCCa
OsSK22	P$E2FA_02	E2FA	1926	1936	1	1	0.852	tgcCGCCAtg
OsSK22	P$AT3G63350_01	AT3G63350	1928	1934	1	1	0.882	CCGCCa
OsSK22	P$O2_Q4	O2	1928	1939	1	1	0.888	ccgcCATGTcg
OsSK22	P$ARF8_01	ARF8	1932	1941	1	1	0.956	caTGTCGct
OsSK22	P$DRE1C_01	DRE1C	1933	1941	1	1	0.862	ATGTCgct
OsSK22	P$WRKY11_Q2	WRKY11	1975	1983	1	1	0.974	tTTGACcg
OsSK22	P$ZAP1_01	ZAP1	1976	1986	1	1	0.935	TTGACcgcct
OsSK22	P$AT3G01030_01	AT3G01030	1979	1988	1	1	0.852	ACCGCctac
OsSK22	P$AT3G63350_01	AT3G63350	1980	1986	1	1	0.867	CCGCCt
OsSK22	P$ATHSFA1D_01	ATHSFA1D	1983	1989	1	1	0.985	cCTACA
OsSK22	P$ASR1_01	ASR1	1990	1995	1	1	1	ACCCA
OsSK22	P$AT4G36620_01	AT4G36620	2006	2014	1	1	0.903	tgcAACCA
OsSK22	P$GAMYB_01	GAMYB	2008	2016	1	1	0.879	CAACCaat
OsSK22	P$E2FA_02	E2FA	2022	2032	1	1	0.987	ctgCGCCAcg
OsSK22	P$NAC080_01	NAC080	2027	2035	1	1	0.888	cCACGCca
OsSK22	P$DOF2_01	DOF2	2036	2047	1	1	0.983	ccccAAAGCta
OsSK22	P$DOF3_01	DOF3	2036	2047	1	1	0.979	ccccAAAGCta
OsSK22	P$HSFA2_01	HSFA2	2038	2044	1	1	0.933	CCAAAg
OsSK22	P$HSFA4A_01	HSFA4A	2043	2049	1	1	0.91	gCTATT
OsSK22	P$WEREWOLF_Q2_01	WEREWOLF	2051	2060	1	1	0.906	tACTAAcca
OsSK22	P$MYBAS1_01	MYBAS1	2051	2062	1	1	0.954	taCTAACcaca
OsSK22	P$AT4G36620_01	AT4G36620	2052	2060	1	1	0.899	actAACCA
OsSK22	P$BPC6_01	BPC6	2060	2081	1	1	0.875	cacccctcTCTCTctctcta
OsSK22	P$ATHSFA1D_01	ATHSFA1D	2077	2083	1	1	0.941	tCTACA
OsSK22	P$RIN_Q2	RIN	2115	2126	1	1	0.855	aattTTTAAgt
OsSK22	P$WRKY_Q2	WRKY	2117	2128	1	1	0.901	tttttaAGTCA
OsSK22	P$MYB1L_01	MYB1L	2179	2189	1	1	0.933	gtCCCTAtca
OsSK22	P$TRB2_01	TRB2	2179	2187	1	1	0.918	gtCCCTAt
OsSK22	P$FBP24_01	FBP24	2216	2234	1	1	0.86	atatTAAACgtagactat
OsSK22	P$AT3G20750_01	AT3G20750	2219	2227	1	1	0.912	tTAAACgt
OsSK22	P$SBF1_01	SBF1	2227	2241	1	1	0.864	agactaTTAATaaa
OsSK22	P$HSFA4A_01	HSFA4A	2229	2235	1	1	1	aCTATT
OsSK22	P$EDT1_01	EDT1	2230	2240	1	1	0.859	ctaTTAATaa
OsSK22	P$ATHB6_01	ATHB6	2233	2242	1	1	0.91	ttAATAAaa
OsSK22	P$ASR1_01	ASR1	2241	2246	1	1	1	ACCCA
OsSK22	P$ARR2_01	ARR2	2248	2258	1	1	0.885	cataATCTTa
OsSK22	P$ARR1_01	ARR1	2271	2281	1	1	0.994	gacGAATCta
OsSK22	P$HSFA4A_01	HSFA4A	2277	2283	1	1	0.914	tCTATT
OsSK22	P$WRKY_Q2	WRKY	2288	2299	1	1	0.882	taattaAGTCA

OsSK22	P$NTL9_01	NTL9	2291	2300	1	1	0.887	tTAAGTcat
OsSK22	P$GT1_Q6	GT1	2322	2329	1	1	0.912	GTAAAca
OsSK22	P$AT3G20750_01	AT3G20750	2322	2330	1	1	0.943	gTAAACat
OsSK22	P$SBF1_01	SBF1	2338	2352	1	1	0.86	tatggaTTAATtag
OsSK22	P$EDT1_01	EDT1	2341	2351	1	1	0.859	ggaTTAATta
OsSK22	P$SBF1_01	SBF1	2348	2362	1	1	0.903	ttagacTTAAAaaa
OsSK22	P$GT1_Q6_01	GT1	2399	2411	1	1	0.861	TTTGTaaatagt
OsSK22	P$GT1_Q6	GT1	2402	2409	1	1	1	GTAAAta
OsSK22	P$SPF1_Q2	SPF1	2404	2414	1	1	0.945	aaATAGTcta
OsSK22	P$SBF1_01	SBF1	2411	2425	1	1	0.875	ctatatTTAATact
OsSK22	P$EDT1_01	EDT1	2414	2424	1	1	0.898	tatTTAATac
OsSK22	P$SBF1_01	SBF1	2431	2445	1	1	0.897	tagtgtTTAAAaca
OsSK22	P$REF6_01	REF6	2439	2450	1	1	0.984	aaaaCAGAGac
OsSK22	P$DOF1_01	DOF1	2447	2458	1	1	0.98	gacTAAAGtta
OsSK22	P$GATA15_01	GATA15	2462	2471	1	1	0.999	caTGATCca
OsSK22	P$HSFA2_01	HSFA2	2468	2474	1	1	0.941	CCAAAc
OsSK22	P$MYB305_Q3	MYB305	2472	2485	1	1	0.96	acaccACCTAaca
OsSK22	P$MYB61_01	MYB61	2474	2489	1	1	0.908	accACCTAacatgga
OsSK22	P$MYBAS1_01	MYBAS1	2477	2488	1	1	0.945	acCTAACatgg
OsSK22	P$MYB24_01	MYB24	2489	2498	1	1	0.932	caaTTAGGc
OsSK22	P$ATSPL8_01	ATSPL8	2493	2509	1	1	0.936	taggcTGTACtacaac
OsSK22	P$GAMYB_Q2	GAMYB	2499	2512	1	1	0.876	gtactACAACctt
OsSK22	P$ATHSFA1D_01	ATHSFA1D	2501	2507	1	1	1	aCTACA
OsSK22	P$SED_Q2	SED	2504	2514	1	1	0.971	acaaCCTTTt
OsSK22	P$PBF_Q2_01	BF	2508	2514	1	1	1	CCTTTt
OsSK22	P$PIL5_01	IL5	2514	2528	1	1	0.856	gccaagctACGTGt
OsSK22	P$ABF4_Q2	ABF4	2516	2530	1	1	0.894	caagctACGTGtac
OsSK22	P$ABZ1_01	ABZ1	2517	2531	1	1	0.894	aagctACGTGtaca
OsSK22	P$HBP1A_Q2	HBP1A	2519	2529	1	1	0.892	gctACGTGta
OsSK22	P$TAF1_Q2	TAF1	2519	2529	1	1	0.936	gctACGTGta
OsSK22	P$EMBP1_02	EMBP1	2519	2529	1	1	0.864	gctACGTGta
OsSK22	P$TAF1_01	TAF1	2519	2529	1	1	0.953	gctACGTGta
OsSK22	P$GBF1_01	GBF1	2520	2528	1	1	0.97	ctACGTGt
OsSK22	P$BIM1_02	BIM1	2520	2530	1	1	0.945	ctACGTGtac
OsSK22	P$ABF4_02	ABF4	2520	2530	1	1	0.993	ctACGTGtac
OsSK22	P$ATSPL8_01	ATSPL8	2520	2536	1	1	0.894	ctacgTGTACaggtaa
OsSK22	P$ABI5_Q2	ABI5	2522	2528	1	1	0.979	ACGTGt
OsSK22	P$HMG1_01	HMG1	2547	2556	1	1	0.975	GTTGTcatc
OsSK22	P$O2_Q4	O2	2579	2590	1	1	0.895	atatCATGTga
OsSK22	P$AMS_01	AMS	2581	2591	1	1	0.879	atCATGTgaa
OsSK22	P$MYB3R5_01	MYB3R5	2584	2599	1	1	0.899	atgtgaaaaCCGTTa
OsSK22	P$MYB3R1_01	MYB3R1	2585	2600	1	1	0.929	tgtgaaaaCCGTTaa
OsSK22	P$MYB3R4_01	MYB3R4	2585	2600	1	1	0.916	tgtgaaaaCCGTTaa
OsSK22	P$GT1_Q6	GT1	2586	2593	1	1	0.971	GTGAAaa
OsSK22	P$SBF1_01	SBF1	2590	2604	1	1	0.882	aaaccgTTAAAtaa
OsSK22	P$ATHB6_01	ATHB6	2597	2606	1	1	0.911	taAATAAgt
OsSK22	P$GT1_Q6	GT1	2604	2611	1	1	0.971	GTGAAaa
OsSK22	P$AT2G26880_01	AT2G26880	2619	2633	1	1	0.859	aattataTTTAAaa
OsSK22	P$SHP2_01	SHP2	2621	2632	1	1	0.868	ttataTTTAAa
OsSK22	P$AT1G77950_01	AT1G77950	2621	2632	1	1	0.855	ttataTTTAAa
OsSK22	P$SBF1_01	SBF1	2621	2635	1	1	0.892	ttatatTTAAAagt
OsSK22	P$PBF_01	BF	2626	2637	1	1	0.965	tttAAAAGttc
OsSK22	P$DOF_Q2	DOF	2626	2637	1	1	0.929	tttAAAAGttc
OsSK22	P$CDF2_01	CDF2	2627	2637	1	1	0.967	ttAAAAGttc
OsSK22	P$CDF3_01	CDF3	2628	2637	1	1	0.985	tAAAAGttc
OsSK22	P$AGL4_01	AGL4	2637	2649	1	1	0.922	tcTAAATttata
OsSK22	P$SBF1_01	SBF1	2649	2663	1	1	0.859	taaaaaTTAATaga
OsSK22	P$EDT1_01	EDT1	2652	2662	1	1	0.881	aaaTTAATag
OsSK22	P$ARR18_01	ARR18	2658	2671	1	1	0.896	atagAGATAaata
OsSK22	P$AT5G26170_01	AT5G26170	2709	2718	1	1	0.924	acTCAACtt
OsSK22	P$PBF_01	BF	2731	2742	1	1	0.976	tatAAAAGaca
OsSK22	P$DOF_Q2	DOF	2731	2742	1	1	0.932	tatAAAAGaca
OsSK22	P$CDF2_01	CDF2	2732	2742	1	1	0.945	atAAAAGaca
OsSK22	P$CDF3_01	CDF3	2733	2742	1	1	0.966	tAAAAGaca
OsSK22	P$MYB24_01	MYB24	2743	2752	1	1	0.939	attTTAGGt
OsSK22	P$MYB131_01	MYB131	2743	2754	1	1	0.913	attTTAGGtga
OsSK22	P$MYB3_01	MYB3	2744	2755	1	1	0.855	tttTAGGTgaa
OsSK22	P$MYB4_01	MYB4	2745	2753	1	1	0.867	ttTAGGTg
OsSK22	P$GT1_Q6	GT1	2750	2757	1	1	1	GTGAAta
OsSK22	P$SPF1_Q2	SPF1	2752	2762	1	1	0.906	gaATAGTgtt
OsSK22	P$ATHB1_01	ATHB1	2759	2773	1	1	0.85	gttctATTATtttt
OsSK22	P$HSFA4A_01	HSFA4A	2761	2767	1	1	0.914	tCTATT
OsSK22	P$ATHB5_01	ATHB5	2762	2771	1	1	0.909	ctaTTATTt
OsSK22	P$AT2G41690_01	AT2G41690	2776	2782	1	1	0.978	CCGAAa
OsSK22	P$SEP3_01	wrz-03	2785	2796	1	1	0.869	ggcatTTTTGt
OsSK22	P$AP2B_01	AP2B	2793	2803	1	1	0.907	tgttACTCCc
OsSK22	P$HSFA2_01	HSFA2	2801	2807	1	1	0.922	CCAAAt
OsSK22	P$ATHB6_01	ATHB6	2802	2811	1	1	0.906	caAATAAag

OsSK22	P$DOF1_01	DOF1	2803	2814	1	1	0.985	aaaTAAAGttg
OsSK22	P$GT1_Q6_02	GT1	2811	2823	1	1	0.852	ttgagtTTAACt
OsSK22	P$GT1_Q6	GT1	2832	2839	1	1	1	GTGAAta
OsSK22	P$LEC2_01	LEC2	2842	2853	1	1	0.944	ttCATGCctac
OsSK22	P$ATHSFA1D_01	ATHSFA1D	2848	2854	1	1	0.985	cCTACA
OsSK22	P$GT1_Q6_01	GT1	2868	2880	1	1	0.875	TTTTTcatgaat
OsSK22	P$FBP24_01	FBP24	2880	2898	1	1	0.878	ttatTAAACttttagttc
OsSK22	P$AT3G20750_01	AT3G20750	2883	2891	1	1	0.907	tTAAACtt
OsSK22	P$PIL5_01	IL5	2898	2912	1	1	0.963	cgattttcACGTGt
OsSK22	P$PIF3_01	IF3	2899	2917	1	1	0.885	gattttCACGTgttttca
OsSK22	P$ABF2_01	ABF2	2900	2913	1	1	0.938	attttCACGTgtt
OsSK22	P$ABF4_Q2	ABF4	2900	2914	1	1	0.915	attttcACGTGttt
OsSK22	P$O2_Q4	O2	2901	2912	1	1	0.901	ttttCACGTgt
OsSK22	P$BZR1_02	BZR1	2901	2915	1	1	0.956	ttttCACGTgtttt
OsSK22	P$HBI1_01	HBI1	2901	2913	1	1	0.941	ttttCACGTgtt
OsSK22	P$ABZ1_01	ABZ1	2901	2915	1	1	0.929	ttttcACGTGtttt
OsSK22	P$GBP_Q6	GBP	2902	2914	1	1	0.902	tttCACGTgttt
OsSK22	P$PIF3_03	IF3	2902	2912	1	1	0.93	tttCACGTgt
OsSK22	P$ABI5_01	ABI5	2902	2912	1	1	0.937	tttCACGTgt
OsSK22	P$ABF4_01	ABF4	2902	2914	1	1	0.949	tttCACGTgttt
OsSK22	P$BZR1_03	BZR1	2902	2922	1	1	0.869	tttcACGTGttttcacttat
OsSK22	P$EMBP1_Q2	EMBP1	2903	2913	1	1	0.885	ttCACGTgtt
OsSK22	P$CPRF_Q2	CPRF	2903	2913	1	1	0.928	ttCACGTgtt
OsSK22	P$CPRF3_Q2	CPRF3	2903	2913	1	1	0.982	ttCACGTgtt
OsSK22	P$CPRF2_Q2	CPRF2	2903	2913	1	1	0.995	ttCACGTgtt
OsSK22	P$O2_02	O2	2903	2913	1	1	0.966	ttCACGTgtt
OsSK22	P$TGA1B_Q2	TGA1B	2903	2913	1	1	0.901	ttCACGTgtt
OsSK22	P$TGA1A_Q2	TGA1A	2903	2913	1	1	0.979	ttCACGTgtt
OsSK22	P$CPRF1_01	CPRF1	2903	2913	1	1	0.941	ttCACGTgtt
OsSK22	P$CPRF3_01	CPRF3	2903	2913	1	1	0.989	ttCACGTgtt
OsSK22	P$CPRF2_01	CPRF2	2903	2913	1	1	0.997	ttCACGTgtt
OsSK22	P$TGA1B_01	TGA1B	2903	2913	1	1	0.898	ttCACGTgtt
OsSK22	P$BES1_01	BES1	2903	2914	1	1	0.943	ttCACGTgttt
OsSK22	P$BEE2_01	BEE2	2903	2913	1	1	0.999	ttCACGTgtt
OsSK22	P$BIM2_01	BIM2	2903	2913	1	1	0.996	ttCACGTgtt
OsSK22	P$BIM3_01	BIM3	2903	2913	1	1	0.992	ttCACGTgtt
OsSK22	P$PHYPA143875_02	HYPA143875	2903	2913	1	1	0.996	ttCACGTgtt
OsSK22	P$PHYPA72483_07	HYPA72483	2903	2913	1	1	0.998	ttCACGTgtt
OsSK22	P$SPT_01	SPT	2903	2912	1	1	0.973	ttCACGTgt
OsSK22	P$GBF1F_Q2	GBF1F	2903	2914	1	1	0.863	ttCACGTgttt
OsSK22	P$HBP1A_Q2	HBP1A	2903	2913	1	1	0.902	ttcACGTGtt
OsSK22	P$TAF1_Q2	TAF1	2903	2913	1	1	0.955	ttcACGTGtt
OsSK22	P$EMBP1_02	EMBP1	2903	2913	1	1	0.906	ttcACGTGtt
OsSK22	P$TAF1_01	TAF1	2903	2913	1	1	0.973	ttcACGTGtt
OsSK22	P$PIF1_01	IF1	2903	2913	1	1	0.976	ttcACGTGtt
OsSK22	P$RITA1_01	RITA1	2904	2911	1	1	1	tCACGTg
OsSK22	P$BHLH66_01	BHLH66	2904	2912	1	1	0.93	tCACGTgt
OsSK22	P$PIF5_01	IF5	2904	2912	1	1	0.975	tCACGTgt
OsSK22	P$MYC2_01	MYC2	2904	2912	1	1	0.95	tCACGTgt
OsSK22	P$MYC3_01	MYC3	2904	2912	1	1	0.989	tCACGTgt
OsSK22	P$BHLH34_01	BHLH34	2904	2912	1	1	0.967	tCACGTgt
OsSK22	P$PHYPA48267_08	HYPA48267	2904	2912	1	1	0.975	tCACGTgt
OsSK22	P$OJ1058_01	OJ1058	2904	2912	1	1	0.946	tCACGTgt
OsSK22	P$UNE10_01	UNE10	2904	2912	1	1	0.982	tCACGTgt
OsSK22	P$BHLH3_01	BHLH3	2904	2912	1	1	0.949	tCACGTgt
OsSK22	P$GBF1_01	GBF1	2904	2912	1	1	0.994	tcACGTGt
OsSK22	P$MYC4_01	MYC4	2904	2912	1	1	0.955	tcACGTGt
OsSK22	P$BIM1_02	BIM1	2904	2914	1	1	0.996	tcACGTGttt
OsSK22	P$BHLH13_01	BHLH13	2904	2912	1	1	0.942	tcACGTGt
OsSK22	P$ABF4_02	ABF4	2904	2914	1	1	0.99	tcACGTGttt
OsSK22	P$OCSBF1_01	OCSBF1	2905	2910	1	1	1	CACGT
OsSK22	P$PIF4_01	IF4	2905	2913	1	1	0.947	CACGTgtt
OsSK22	P$ABI5_Q2	ABI5	2906	2912	1	1	0.979	ACGTGt
OsSK22	P$GAMYB_Q2	GAMYB	2962	2975	1	1	0.855	gaagtACAACata
OsSK22	P$RAV1_01	RAV1	2965	2977	1	1	0.953	gtaCAACAtagt
OsSK22	P$SPF1_Q2	SPF1	2970	2980	1	1	0.9	acATAGTtca
OsSK22	P$O2_Q4	O2	2997	3008	1	1	0.91	aagtCATGTaa
OsSK22	P$CBNAC_01	CBNAC	3030	3036	1	1	1	tTGCTT
OsSK22	P$CBNAC_02	CBNAC	3030	3046	1	1	0.907	tTGCTTacatacaatt
OsSK22	P$ALFIN1_Q2	ALFIN1	3041	3056	1	1	0.853	caattgGTGGGataa
OsSK22	P$PBF_01	BF	3052	3063	1	1	0.972	ataAAAAGgca
OsSK22	P$DOF_Q2	DOF	3052	3063	1	1	0.981	ataAAAAGgca
OsSK22	P$CDF2_01	CDF2	3053	3063	1	1	0.974	taAAAAGgca
OsSK22	P$CDF3_01	CDF3	3054	3063	1	1	0.975	aAAAAGgca
OsSK22	P$PBF_Q2	BF	3055	3061	1	1	1	aAAAGG
OsSK22	P$AT5G04240_01	AT5G04240	3058	3064	1	1	0.939	aGGCAC
OsSK22	P$ATHB6_01	ATHB6	3086	3095	1	1	0.908	aaAATAAat
OsSK22	P$PDF2_01	DF2	3086	3097	1	1	0.904	aaaaTAAATgt

OsSK22	P$AT5G04240_01	AT5G04240	3114	3120	1	1	0.938	tGGCAC
OsSK22	P$AGL3_01	AGL3	3152	3170	1	1	0.856	agctCCATAaattgttgg
OsSK22	P$AGL3_02	AGL3	3152	3170	1	1	0.862	agctCCATAaattgttgg
OsSK22	P$AGL2_01	AGL2	3152	3170	1	1	0.874	agctCCATAaattgttgg
OsSK22	P$AGL3_03	AGL3	3152	3170	1	1	0.862	agctCCATAaattgttgg
OsSK22	P$GT1_Q6_01	GT1	3177	3189	1	1	0.932	TTTGTgattaca
OsSK22	P$ATHB6_01	ATHB6	3192	3201	1	1	0.984	atAATAAtt
OsSK22	P$ATHB5_04	ATHB5	3192	3203	1	1	0.98	atAATAAttga
OsSK22	P$ATHB1_03	ATHB1	3192	3203	1	1	0.989	atAATAAttga
OsSK22	P$ATHB16_01	ATHB16	3193	3201	1	1	1	tAATAAtt
OsSK22	P$ARR1_01	ARR1	3202	3212	1	1	0.944	attGAATCag
OsSK22	P$ATHB7_01	ATHB7	3204	3214	1	1	0.856	tgAATCAgtt
OsSK22	P$HAT1_01	HAT1	3204	3214	1	1	0.862	tgAATCAgtt
OsSK22	P$GT1_Q6_01	GT1	3212	3224	1	1	0.937	TTTGTtcttatt
OsSK22	P$PEND_02	END	3214	3224	1	1	0.931	tgTTCTTatt
OsSK22	P$BPC1_Q2	BPC1	3228	3234	1	1	0.997	AGAAAa
OsSK22	P$ATHB6_01	ATHB6	3230	3239	1	1	0.905	aaAATAAaa
OsSK22	P$ATHB6_01	ATHB6	3239	3248	1	1	0.902	aaAATAAac
OsSK22	P$AT3G20750_01	AT3G20750	3242	3250	1	1	0.865	aTAAACca
OsSK22	P$AT4G36620_01	AT4G36620	3242	3250	1	1	0.993	ataAACCA
OsSK22	P$GT1_Q6_02	GT1	3251	3263	1	1	0.88	tataatTTAACt
OsSK22	P$HSFA2_01	HSFA2	3271	3277	1	1	1	CCAAAa
OsSK22	P$C1_Q2	C1	3273	3284	1	1	0.914	aaAACTAgtct
OsSK22	P$GATA11_01	GATA11	3283	3291	1	1	0.898	tgGATCTg
OsSK22	P$GATA8_01	GATA8	3283	3292	1	1	0.986	tgGATCTgt
OsSK22	P$MYB24_01	MYB24	3292	3301	1	1	0.941	aatTTAGGt
OsSK22	P$MYB131_01	MYB131	3292	3303	1	1	0.939	aatTTAGGttg
OsSK22	P$HMG1_01	HMG1	3299	3308	1	1	0.915	GTTGTgcta
OsSK22	P$DOF1_01	DOF1	3314	3325	1	1	0.979	gccTAAAGaaa
OsSK22	P$PBF_01	BF	3319	3330	1	1	0.972	aagAAAAGaag
OsSK22	P$DOF_Q2	DOF	3319	3330	1	1	0.963	aagAAAAGaag
OsSK22	P$BPC1_Q2	BPC1	3320	3326	1	1	0.997	AGAAAa
OsSK22	P$CDF2_01	CDF2	3320	3330	1	1	0.95	agAAAAGaag
OsSK22	P$CDF3_01	CDF3	3321	3330	1	1	0.97	gAAAAGaag
OsSK22	P$PEND_01	END	3323	3331	1	1	0.891	aAAGAAgt
OsSK22	P$ATHB7_01	ATHB7	3352	3362	1	1	0.884	taAATCAata
OsSK22	P$HAT1_01	HAT1	3352	3362	1	1	0.876	taAATCAata
OsSK22	P$ARR2_01	ARR2	3364	3374	1	1	0.862	gaccATCTTc
OsSK22	P$AGL9_01	AGL9	3370	3385	1	1	0.866	cttctaaAATGGgca
OsSK22	P$AT5G04240_01	AT5G04240	3380	3386	1	1	1	gGGCAC
OsSK22	P$GAMYB_01	GAMYB	3392	3400	1	1	0.948	CAACCgaa
OsSK22	P$AT2G41690_01	AT2G41690	3395	3401	1	1	0.978	CCGAAa
OsSK22	P$SBF1_01	SBF1	3423	3437	1	1	0.924	caatccTTAAAaaa
OsSK22	P$C1_Q2	C1	3434	3445	1	1	0.929	aaAACTAcatt
OsSK22	P$ATHSFA1D_01	ATHSFA1D	3437	3443	1	1	1	aCTACA
OsSK22	P$CCA1_Q5	CCA1	3441	3458	1	1	0.882	cattgAAAATatcctaa
OsSK22	P$AT3G60580_01	AT3G60580	3456	3463	1	1	0.883	aaATCCC
OsSK22	P$HSFA2_01	HSFA2	3461	3467	1	1	1	CCAAAa
OsSK22	P$AGL20_01	AGL20	3466	3478	1	1	0.896	atTAAATtttaa
OsSK22	P$AGL12_01	AGL12	3466	3478	1	1	0.933	attAAATTttaa
OsSK22	P$AT2G26880_01	AT2G26880	3466	3480	1	1	0.858	attaaatTTTAAaa
OsSK22	P$AT2G26320_01	AT2G26320	3467	3478	1	1	0.948	TTAAAttttaa
OsSK22	P$SBF1_01	SBF1	3468	3482	1	1	0.894	taaattTTAAAatc
OsSK22	P$SEP3_01	wrz-03	3480	3491	1	1	0.916	tctaaTTTTGg
OsSK22	P$MYB1L_01	MYB1L	3524	3534	1	1	0.967	ggCCCTAaat
OsSK22	P$TRB2_01	TRB2	3524	3532	1	1	0.988	ggCCCTAa
OsSK22	P$PDF2_01	DF2	3525	3536	1	1	0.858	gcccTAAATca
OsSK22	P$ATHB7_01	ATHB7	3529	3539	1	1	0.873	taAATCAacc
OsSK22	P$HAT1_01	HAT1	3529	3539	1	1	0.864	taAATCAacc
OsSK22	P$AT4G36620_01	AT4G36620	3532	3540	1	1	1	atcAACCA
OsSK22	P$GAMYB_01	GAMYB	3534	3542	1	1	0.878	CAACCatg
OsSK22	P$AT4G04450_01	AT4G04450	3540	3549	1	1	0.901	tgtTTAGCt
OsSK22	P$ATHSFA1D_01	ATHSFA1D	3581	3587	1	1	0.94	gCTACA
OsSK22	P$GT1_Q6_01	GT1	3609	3621	1	1	0.906	TTTTTattaaaa
OsSK22	P$SBF1_01	SBF1	3609	3623	1	1	0.941	tttttaTTAAAaaa
OsSK22	P$ATHB1_01	ATHB1	3618	3632	1	1	0.937	aaaaaATTATttta
OsSK22	P$ATHB5_01	ATHB5	3621	3630	1	1	0.915	aaaTTATTt
OsSK22	P$RIN_Q2	RIN	3624	3635	1	1	0.883	ttatTTTAAgt
OsSK22	P$PBF_01	BF	3636	3647	1	1	0.962	tgcAAAAGcta
OsSK22	P$DOF_Q2	DOF	3636	3647	1	1	0.917	tgcAAAAGcta
OsSK22	P$DOF2_01	DOF2	3636	3647	1	1	0.99	tgcaAAAGCta
OsSK22	P$DOF3_01	DOF3	3636	3647	1	1	0.979	tgcaAAAGCta
OsSK22	P$CDF2_01	CDF2	3637	3647	1	1	0.955	gcAAAAGcta
OsSK22	P$CDF3_01	CDF3	3638	3647	1	1	0.976	cAAAAGcta
OsSK22	P$SBF1_01	SBF1	3645	3659	1	1	0.903	taatgtTTAATtag
OsSK22	P$EDT1_01	EDT1	3648	3658	1	1	0.855	tgtTTAATta
OsSK22	P$ATHSFA1D_01	ATHSFA1D	3660	3666	1	1	0.985	cCTACA
OsSK22	P$ATHB6_01	ATHB6	3666	3675	1	1	0.978	ctAATAAtc

OsSK22	P$ATHB5_04	ATHB5	3666	3677	1	1	0.898	ctAATAAtcct
OsSK22	P$ATHB1_03	ATHB1	3666	3677	1	1	0.887	ctAATAAtcct
OsSK22	P$ATHB16_01	ATHB16	3667	3675	1	1	0.908	tAATAAtc
OsSK22	P$CBF1_01	CBF1	3688	3698	1	1	0.934	tTGCCGctga
OsSK22	P$ERF019_01	ERF019	3688	3698	1	1	0.953	tTGCCGctga
OsSK22	P$DREB6_01	DREB6	3688	3698	1	1	0.873	tTGCCGctga
OsSK22	P$DREBIII4_01	DREBIII4	3688	3698	1	1	0.94	tTGCCGctga
OsSK22	P$JERF3_01	JERF3	3688	3698	1	1	0.867	tTGCCGctga
OsSK22	P$DREB1_01	DREB1	3688	3698	1	1	0.874	tTGCCGctga
OsSK22	P$CEF1_01	CEF1	3688	3698	1	1	0.864	tTGCCGctga
OsSK22	P$JERF1_01	JERF1	3688	3698	1	1	0.919	tTGCCGctga
OsSK22	P$CBF1_03	CBF1	3688	3698	1	1	0.952	tTGCCGctga
OsSK22	P$DREB1F_01	DREB1F	3688	3698	1	1	0.878	tTGCCGctga
OsSK22	P$AT1G33760_01	AT1G33760	3688	3698	1	1	0.936	tTGCCGctga
OsSK22	P$AT1G71520_01	AT1G71520	3688	3698	1	1	0.96	tTGCCGctga
OsSK22	P$DREB1E_02	DREB1E	3688	3698	1	1	0.864	tTGCCGctga
OsSK22	P$ORA47_01	ORA47	3688	3698	1	1	0.988	tTGCCGctga
OsSK22	P$ABI4_03	ABI4	3688	3698	1	1	0.896	ttGCCGCtga
OsSK22	P$DREBI5_01	DREBI5	3688	3698	1	1	0.939	ttGCCGCtga
OsSK22	P$DREB2E_01	DREB2E	3688	3699	1	1	0.852	ttGCCGCtgat
OsSK22	P$DREB2B_01	DREB2B	3688	3698	1	1	0.864	ttGCCGCtga
OsSK22	P$AT1G77200_01	AT1G77200	3688	3698	1	1	0.987	ttGCCGCtga
OsSK22	P$ATERF14_01	ATERF14	3688	3698	1	1	0.865	ttGCCGCtga
OsSK22	P$DREBIII3_01	DREBIII3	3688	3698	1	1	0.976	ttGCCGCtga
OsSK22	P$DREBIII2_01	DREBIII2	3688	3698	1	1	0.975	ttGCCGCtga
OsSK22	P$DREBIII1_01	DREBIII1	3688	3698	1	1	0.975	ttGCCGCtga
OsSK22	P$CBF3_01	CBF3	3688	3698	1	1	0.895	ttGCCGCtga
OsSK22	P$DREBII1_01	DREBII1	3688	3698	1	1	0.937	ttGCCGCtga
OsSK22	P$AT2G44940_01	AT2G44940	3688	3698	1	1	0.974	ttGCCGCtga
OsSK22	P$DBF2_01	DBF2	3688	3698	1	1	0.995	ttGCCGCtga
OsSK22	P$CBF5_01	CBF5	3688	3698	1	1	0.939	ttGCCGCtga
OsSK22	P$CBF16_01	CBF16	3688	3698	1	1	0.938	ttGCCGCtga
OsSK22	P$CBF17_01	CBF17	3688	3698	1	1	0.936	ttGCCGCtga
OsSK22	P$CBF_01	CBF	3688	3698	1	1	0.938	ttGCCGCtga
OsSK22	P$ERF016_01	ERF016	3688	3698	1	1	0.921	ttGCCGCtga
OsSK22	P$AT5G43410_01	AT5G43410	3688	3698	1	1	0.872	ttGCCGCtga
OsSK22	P$TINY2_02	TINY2	3688	3698	1	1	0.999	ttGCCGCtga
OsSK22	P$AT3G16280_01	AT3G16280	3688	3698	1	1	0.942	ttGCCGCtga
OsSK22	P$DREB1A_03	DREB1A	3688	3698	1	1	0.894	ttGCCGCtga
OsSK22	P$DREB2D_01	DREB2D	3688	3698	1	1	0.851	ttGCCGCtga
OsSK22	P$CRF2_01	CRF2	3688	3696	1	1	0.875	ttGCCGCt
OsSK22	P$ERF098_01	ERF098	3688	3696	1	1	0.891	ttGCCGCt
OsSK22	P$ERF7_02	ERF7	3689	3699	1	1	0.939	tGCCGCtgat
OsSK22	P$WRKY40_03	WRKY40	3703	3713	1	1	0.996	ttAGTCAaaa
OsSK22	P$WRKY18_02	WRKY18	3703	3713	1	1	0.979	ttaGTCAAaa
OsSK22	P$WRKY21_02	WRKY21	3703	3713	1	1	0.968	ttaGTCAAaa
OsSK22	P$WRKY48_02	WRKY48	3703	3713	1	1	0.995	ttaGTCAAaa
OsSK22	P$WRKY57_01	WRKY57	3703	3713	1	1	0.982	ttaGTCAAaa
OsSK22	P$WRKY60_01	WRKY60	3703	3714	1	1	0.909	ttaGTCAAaac
OsSK22	P$WRKY15_01	WRKY15	3704	3714	1	1	0.98	taGTCAAaac
OsSK22	P$WRKY2_01	WRKY2	3704	3712	1	1	0.94	taGTCAAa
OsSK22	P$WRKY25_02	WRKY25	3704	3712	1	1	0.921	taGTCAAa
OsSK22	P$WRKY40_01	WRKY40	3704	3712	1	1	0.996	taGTCAAa
OsSK22	P$WRKY43_02	WRKY43	3704	3714	1	1	0.991	taGTCAAaac
OsSK22	P$WRKY62_01	WRKY62	3704	3712	1	1	0.923	taGTCAAa
OsSK22	P$WRKY63_01	WRKY63	3704	3712	1	1	0.905	taGTCAAa
OsSK22	P$WRKY75_01	WRKY75	3704	3712	1	1	0.977	taGTCAAa
OsSK22	P$WRKY8_01	WRKY8	3704	3713	1	1	0.991	taGTCAAaa
OsSK22	P$WRKY23_01	WRKY23	3705	3713	1	1	0.889	aGTCAAaa
OsSK22	P$WRKY30_01	WRKY30	3705	3715	1	1	0.914	aGTCAAaacc
OsSK22	P$WRKY18_Q2	WRKY18	3706	3715	1	1	0.946	GTCAAaacc
OsSK22	P$GATA15_01	GATA15	3714	3723	1	1	0.999	ccTGATCcg
OsSK22	P$AT2G41690_01	AT2G41690	3720	3726	1	1	1	CCGAAc
OsSK22	P$ABF2_01	ABF2	3723	3736	1	1	0.909	aactgCACGTaag
OsSK22	P$O2_Q4	O2	3724	3735	1	1	0.882	actgCACGTaa
OsSK22	P$BZR1_02	BZR1	3724	3738	1	1	0.866	actgCACGTaagaa
OsSK22	P$HBI1_01	HBI1	3724	3736	1	1	0.865	actgCACGTaag
OsSK22	P$GBP_Q6	GBP	3725	3737	1	1	0.908	ctgCACGTaaga
OsSK22	P$ABI5_01	ABI5	3725	3735	1	1	0.894	ctgCACGTaa
OsSK22	P$ABF4_01	ABF4	3725	3737	1	1	0.875	ctgCACGTaaga
OsSK22	P$CPRF3_Q2	CPRF3	3726	3736	1	1	0.909	tgCACGTaag
OsSK22	P$CPRF2_Q2	CPRF2	3726	3736	1	1	0.936	tgCACGTaag
OsSK22	P$O2_02	O2	3726	3736	1	1	0.95	tgCACGTaag
OsSK22	P$TGA1B_Q2	TGA1B	3726	3736	1	1	0.903	tgCACGTaag
OsSK22	P$TGA1A_Q2	TGA1A	3726	3736	1	1	0.975	tgCACGTaag
OsSK22	P$CPRF3_01	CPRF3	3726	3736	1	1	0.92	tgCACGTaag
OsSK22	P$CPRF2_01	CPRF2	3726	3736	1	1	0.937	tgCACGTaag
OsSK22	P$BEE2_01	BEE2	3726	3736	1	1	0.916	tgCACGTaag

OsSK22	P$BIM2_01	BIM2	3726	3736	1	1	0.857	tgCACGTaag
OsSK22	P$BIM3_01	BIM3	3726	3736	1	1	0.893	tgCACGTaag
OsSK22	P$PHYPA143875_02	HYPA143875	3726	3736	1	1	0.877	tgCACGTaag
OsSK22	P$SPT_01	SPT	3726	3735	1	1	0.925	tgCACGTaa
OsSK22	P$GBF1F_Q2	GBF1F	3726	3737	1	1	0.861	tgCACGTaaga
OsSK22	P$NAC043_01	NAC043	3726	3736	1	1	0.856	tgcACGTAag
OsSK22	P$RITA1_01	RITA1	3727	3734	1	1	0.964	gCACGTa
OsSK22	P$OCSBF1_01	OCSBF1	3728	3733	1	1	1	CACGT
OsSK22	P$NAC025_01	NAC025	3728	3736	1	1	0.95	cACGTAag
OsSK22	P$NAC6_01	NAC6	3729	3735	1	1	1	aCGTAA
OsSK22	P$PEND_01	END	3732	3740	1	1	0.875	tAAGAAcg
OsSK22	P$WRKY18_02	WRKY18	3737	3747	1	1	0.997	acgGTCAAga
OsSK22	P$WRKY21_02	WRKY21	3737	3747	1	1	0.958	acgGTCAAga
OsSK22	P$WRKY48_02	WRKY48	3737	3747	1	1	0.996	acgGTCAAga
OsSK22	P$WRKY57_01	WRKY57	3737	3747	1	1	0.966	acgGTCAAga
OsSK22	P$WRKY60_01	WRKY60	3737	3748	1	1	0.969	acgGTCAAgaa
OsSK22	P$WRKY15_01	WRKY15	3738	3748	1	1	0.977	cgGTCAAgaa
OsSK22	P$WRKY2_01	WRKY2	3738	3746	1	1	0.983	cgGTCAAg
OsSK22	P$WRKY25_02	WRKY25	3738	3746	1	1	0.974	cgGTCAAg
OsSK22	P$WRKY40_01	WRKY40	3738	3746	1	1	1	cgGTCAAg
OsSK22	P$WRKY43_02	WRKY43	3738	3748	1	1	0.972	cgGTCAAgaa
OsSK22	P$WRKY62_01	WRKY62	3738	3746	1	1	0.884	cgGTCAAg
OsSK22	P$WRKY63_01	WRKY63	3738	3746	1	1	0.993	cgGTCAAg
OsSK22	P$WRKY75_01	WRKY75	3738	3746	1	1	0.959	cgGTCAAg
OsSK22	P$WRKY8_01	WRKY8	3738	3747	1	1	0.987	cgGTCAAga
OsSK22	P$WRKY30_01	WRKY30	3739	3749	1	1	0.914	gGTCAAgaaa
OsSK22	P$WRKY18_Q2	WRKY18	3740	3749	1	1	0.949	GTCAAgaaa
OsSK22	P$BPC1_Q2	BPC1	3744	3750	1	1	0.99	AGAAAc
OsSK22	P$AT4G36620_01	AT4G36620	3744	3752	1	1	0.9	agaAACCA
OsSK22	P$ATMYB77_01	ATMYB77	3749	3762	1	1	0.867	ccatttCGGTTac
OsSK22	P$PHYPA64121_06	HYPA64121	3751	3764	1	1	0.879	attTCGGTtacat
OsSK22	P$ANTL_01	ANTL	3756	3766	1	1	0.878	gGTTACatca
OsSK22	P$GAMYB_Q2	GAMYB	3762	3775	1	1	0.927	atcacACAACaca
OsSK22	P$RAV1_01	RAV1	3765	3777	1	1	0.946	acaCAACAcagc
OsSK22	P$LEC2_01	LEC2	3784	3795	1	1	0.933	ctCATGCtgtc
OsSK22	P$LEC2_01	LEC2	3792	3803	1	1	0.939	gtCATGCttgt
OsSK22	P$CBNAC_01	CBNAC	3795	3801	1	1	0.968	aTGCTT
OsSK22	P$CBNAC_02	CBNAC	3795	3811	1	1	0.858	aTGCTTgtggtgcacc
OsSK22	P$MYB305_Q3	MYB305	3803	3816	1	1	0.888	ggtgcACCTAgca
OsSK22	P$MYB61_01	MYB61	3805	3820	1	1	0.889	tgcACCTAgcaattc
OsSK22	P$LIM1_01	LIM1	3871	3883	1	1	0.905	CCACCagctgtg
OsSK22	P$ATSPL8_01	ATSPL8	3876	3892	1	1	0.909	agctgTGTACactgca
OsSK22	P$MYBAS1_01	MYBAS1	3903	3914	1	1	0.984	agCTAACcact
OsSK22	P$GT1_01	GT1	3904	3912	1	1	0.872	gcTAACCa
OsSK22	P$AT4G36620_01	AT4G36620	3904	3912	1	1	0.876	gctAACCA
OsSK22	P$C1_Q2	C1	3921	3932	1	1	0.933	aaAACTAtagc
OsSK22	P$ATMYB15_Q2	ATMYB15	3940	3946	1	1	0.865	TAACAg
OsSK22	P$ASR1_01	ASR1	3956	3961	1	1	1	ACCCA
OsSK22	P$LIM1_01	LIM1	3958	3970	1	1	0.856	CCACCttcttct
OsSK22	P$PEND_02	END	3961	3971	1	1	0.869	ccTTCTTctt
OsSK22	P$WRKY18_02	WRKY18	3969	3979	1	1	0.998	ttgGTCAAag
OsSK22	P$WRKY21_02	WRKY21	3969	3979	1	1	0.973	ttgGTCAAag
OsSK22	P$WRKY48_02	WRKY48	3969	3979	1	1	0.998	ttgGTCAAag
OsSK22	P$WRKY57_01	WRKY57	3969	3979	1	1	0.975	ttgGTCAAag
OsSK22	P$WRKY60_01	WRKY60	3969	3980	1	1	0.982	ttgGTCAAagc
OsSK22	P$WRKY15_01	WRKY15	3970	3980	1	1	0.987	tgGTCAAagc
OsSK22	P$WRKY2_01	WRKY2	3970	3978	1	1	0.989	tgGTCAAa
OsSK22	P$WRKY25_02	WRKY25	3970	3978	1	1	0.973	tgGTCAAa
OsSK22	P$WRKY40_01	WRKY40	3970	3978	1	1	1	tgGTCAAa
OsSK22	P$WRKY43_02	WRKY43	3970	3980	1	1	0.978	tgGTCAAagc
OsSK22	P$WRKY62_01	WRKY62	3970	3978	1	1	0.957	tgGTCAAa
OsSK22	P$WRKY63_01	WRKY63	3970	3978	1	1	0.99	tgGTCAAa
OsSK22	P$WRKY75_01	WRKY75	3970	3978	1	1	0.975	tgGTCAAa
OsSK22	P$WRKY8_01	WRKY8	3970	3979	1	1	0.993	tgGTCAAag
OsSK22	P$WRKY23_01	WRKY23	3971	3979	1	1	0.893	gGTCAAag
OsSK22	P$WRKY30_01	WRKY30	3971	3981	1	1	0.924	gGTCAAagcc
OsSK22	P$DOF2_01	DOF2	3971	3982	1	1	0.98	ggtcAAAGCca
OsSK22	P$DOF3_01	DOF3	3971	3982	1	1	0.985	ggtcAAAGCca
OsSK22	P$WRKY18_Q2	WRKY18	3972	3981	1	1	0.946	GTCAAagcc
OsSK22	P$AT3G60580_01	AT3G60580	3983	3990	1	1	0.873	ccATCCC
OsSK22	P$LIM1_01	LIM1	3988	4000	1	1	0.947	CCACCactcacc
OsSK22	P$ASR1_01	ASR1	3997	4002	1	1	1	ACCCA
OsSK23	P$ARR18_01	ARR18	5	18	1	1	0.89	ctatAGATAataa
OsSK23	P$ATHB6_01	ATHB6	11	20	1	1	0.913	atAATAAat
OsSK23	P$ATHB6_01	ATHB6	15	24	1	1	0.909	taAATAAgg
OsSK23	P$ALFIN1_Q2	ALFIN1	33	48	1	1	0.888	atatgcGTGGGcttt
OsSK23	P$BZIP68_01	BZIP68	35	44	1	1	0.927	atgCGTGGg
OsSK23	P$ATHB6_01	ATHB6	61	70	1	1	0.981	atAATAAta

OsSK23	P$ATHB5_04	ATHB5	61	72	1	1	0.909	atAATAAtaca
OsSK23	P$ATHB1_03	ATHB1	61	72	1	1	0.9	atAATAAtaca
OsSK23	P$ATHB16_01	ATHB16	62	70	1	1	0.915	tAATAAta
OsSK23	P$ATHB6_01	ATHB6	69	78	1	1	0.926	acAATAAaa
OsSK23	P$BZIP68_01	BZIP68	89	98	1	1	0.936	accCGTGGa
OsSK23	P$PBF_01	BF	103	114	1	1	0.944	tcgAAAAGgtt
OsSK23	P$DOF_Q2	DOF	103	114	1	1	0.937	tcgAAAAGgtt
OsSK23	P$CDF2_01	CDF2	104	114	1	1	0.952	cgAAAAGgtt
OsSK23	P$CDF3_01	CDF3	105	114	1	1	0.972	gAAAAGgtt
OsSK23	P$PBF_Q2	BF	106	112	1	1	1	aAAAGG
OsSK23	P$GT1_Q6_01	GT1	114	126	1	1	0.867	TTTTTtttttac
OsSK23	P$GT1_Q6_01	GT1	115	127	1	1	0.993	TTTTTttttacc
OsSK23	P$GT1_01	GT1	132	140	1	1	0.935	atTAACCt
OsSK23	P$PBF_Q2	BF	159	165	1	1	0.965	gAAAGG
OsSK23	P$SBF1_01	SBF1	183	197	1	1	0.869	ttagaaTTAATttc
OsSK23	P$EDT1_01	EDT1	186	196	1	1	0.896	gaaTTAATtt
OsSK23	P$HSFA4A_01	HSFA4A	213	219	1	1	1	aCTATT
OsSK23	P$O2_Q4	O2	227	238	1	1	0.914	tttgCATGTta
OsSK23	P$ABI3_01	ABI3	228	237	1	1	0.87	ttGCATGtt
OsSK23	P$WEREWOLF_Q2	WEREWOLF	232	241	1	1	0.96	atGTTAGta
OsSK23	P$WRKY40_03	WRKY40	261	271	1	1	0.995	ctAGTCAagg
OsSK23	P$WRKY18_02	WRKY18	261	271	1	1	0.982	ctaGTCAAgg
OsSK23	P$WRKY21_02	WRKY21	261	271	1	1	0.963	ctaGTCAAgg
OsSK23	P$WRKY48_02	WRKY48	261	271	1	1	0.993	ctaGTCAAgg
OsSK23	P$WRKY57_01	WRKY57	261	271	1	1	0.976	ctaGTCAAgg
OsSK23	P$WRKY60_01	WRKY60	261	272	1	1	0.905	ctaGTCAAggg
OsSK23	P$WRKY15_01	WRKY15	262	272	1	1	0.975	taGTCAAggg
OsSK23	P$WRKY2_01	WRKY2	262	270	1	1	0.933	taGTCAAg
OsSK23	P$WRKY25_02	WRKY25	262	270	1	1	0.916	taGTCAAg
OsSK23	P$WRKY40_01	WRKY40	262	270	1	1	0.996	taGTCAAg
OsSK23	P$WRKY43_02	WRKY43	262	272	1	1	0.985	taGTCAAggg
OsSK23	P$WRKY62_01	WRKY62	262	270	1	1	0.897	taGTCAAg
OsSK23	P$WRKY63_01	WRKY63	262	270	1	1	0.908	taGTCAAg
OsSK23	P$WRKY75_01	WRKY75	262	270	1	1	0.96	taGTCAAg
OsSK23	P$WRKY8_01	WRKY8	262	271	1	1	0.988	taGTCAAgg
OsSK23	P$WRKY23_01	WRKY23	263	271	1	1	0.91	aGTCAAgg
OsSK23	P$WRKY30_01	WRKY30	263	273	1	1	0.92	aGTCAAgggt
OsSK23	P$WRKY18_Q2	WRKY18	264	273	1	1	0.965	GTCAAgggt
OsSK23	P$ASR1_01	ASR1	282	287	1	1	1	ACCCA
OsSK23	P$MYB3_01	MYB3	284	295	1	1	0.861	ccaTAGGTatg
OsSK23	P$RAV1_01	RAV1	302	314	1	1	0.956	ctgCAACAgctg
OsSK23	P$ATHSFA1D_01	ATHSFA1D	313	319	1	1	0.94	gCTACA
OsSK23	P$ARR1_01	ARR1	318	328	1	1	0.94	aatGAATCga
OsSK23	P$SED_Q2	SED	386	396	1	1	0.927	tctcCCTTTg
OsSK23	P$PBF_Q2_01	BF	390	396	1	1	0.988	CCTTTg
OsSK23	P$O2_Q4	O2	394	405	1	1	0.856	tgttCATGTag
OsSK23	P$TEIL_01	TEIL	399	407	1	1	0.883	ATGTAgtt
OsSK23	P$ABF2_01	ABF2	417	430	1	1	0.951	gaagaCACGTtaa
OsSK23	P$O2_Q4	O2	418	429	1	1	0.888	aagaCACGTta
OsSK23	P$BZR1_02	BZR1	418	432	1	1	0.863	aagaCACGTtaaga
OsSK23	P$NAC92_01	NAC92	419	431	1	1	0.95	agACACGttaag
OsSK23	P$GBP_Q6	GBP	419	431	1	1	0.907	agaCACGTtaag
OsSK23	P$ABI5_01	ABI5	419	429	1	1	0.957	agaCACGTta
OsSK23	P$ABF4_01	ABF4	419	431	1	1	0.907	agaCACGTtaag
OsSK23	P$EMBP1_Q2	EMBP1	420	430	1	1	0.913	gaCACGTtaa
OsSK23	P$CPRF3_Q2	CPRF3	420	430	1	1	0.926	gaCACGTtaa
OsSK23	P$CPRF2_Q2	CPRF2	420	430	1	1	0.94	gaCACGTtaa
OsSK23	P$O2_02	O2	420	430	1	1	0.942	gaCACGTtaa
OsSK23	P$TGA1B_Q2	TGA1B	420	430	1	1	0.927	gaCACGTtaa
OsSK23	P$TGA1A_Q2	TGA1A	420	430	1	1	0.971	gaCACGTtaa
OsSK23	P$CPRF3_01	CPRF3	420	430	1	1	0.934	gaCACGTtaa
OsSK23	P$CPRF2_01	CPRF2	420	430	1	1	0.941	gaCACGTtaa
OsSK23	P$TGA1B_01	TGA1B	420	430	1	1	0.888	gaCACGTtaa
OsSK23	P$BEE2_01	BEE2	420	430	1	1	0.92	gaCACGTtaa
OsSK23	P$BIM2_01	BIM2	420	430	1	1	0.855	gaCACGTtaa
OsSK23	P$BIM3_01	BIM3	420	430	1	1	0.885	gaCACGTtaa
OsSK23	P$PHYPA143875_02	HYPA143875	420	430	1	1	0.885	gaCACGTtaa
OsSK23	P$SPT_01	SPT	420	429	1	1	0.914	gaCACGTta
OsSK23	P$GBF1F_Q2	GBF1F	420	431	1	1	0.886	gaCACGTtaag
OsSK23	P$RITA1_01	RITA1	421	428	1	1	0.953	aCACGTt
OsSK23	P$OCSBF1_01	OCSBF1	422	427	1	1	1	CACGT
OsSK23	P$PEND_01	END	427	435	1	1	0.892	tAAGAAaa
OsSK23	P$BPC1_Q2	BPC1	429	435	1	1	0.997	AGAAAa
OsSK23	P$SBF1_01	SBF1	431	445	1	1	0.869	aaaatgTTAAAaga
OsSK23	P$PBF_01	BF	436	447	1	1	0.956	gttAAAAGatt
OsSK23	P$DOF_Q2	DOF	436	447	1	1	0.93	gttAAAAGatt
OsSK23	P$CDF2_01	CDF2	437	447	1	1	0.946	ttAAAAGatt
OsSK23	P$CDF3_01	CDF3	438	447	1	1	0.968	tAAAAGatt

OsSK23	P$MRP1_Q2	MRP1	445	457	1	1	0.865	ttTCTATgtcct
OsSK23	P$AT4G00870_01	AT4G00870	452	466	1	1	0.912	gtCCTCGttaatat
OsSK23	P$SBF1_01	SBF1	453	467	1	1	0.95	tcctcgTTAATata
OsSK23	P$ATHSFA1D_01	ATHSFA1D	477	483	1	1	1	aCTACA
OsSK23	P$PEND_01	END	498	506	1	1	0.877	tAAGAAtc
OsSK23	P$ARR1_01	ARR1	498	508	1	1	0.95	taaGAATCaa
OsSK23	P$ATHB7_01	ATHB7	500	510	1	1	0.873	agAATCAaat
OsSK23	P$HAT1_01	HAT1	500	510	1	1	0.864	agAATCAaat
OsSK23	P$MYB80_01	MYB80	509	520	1	1	0.988	tgGAATAttct
OsSK23	P$ARR18_01	ARR18	531	544	1	1	0.943	atctAGATAggga
OsSK23	P$MYB3_01	MYB3	542	553	1	1	0.93	gagTAGGTaag
OsSK23	P$MYB4_01	MYB4	543	551	1	1	0.95	agTAGGTa
OsSK23	P$GATA9_01	GATA9	548	559	1	1	0.883	gtaAGATCaca
OsSK23	P$ARR10_01	ARR10	551	558	1	1	0.869	AGATCac
OsSK23	P$PEND_02	END	569	579	1	1	0.973	agTTCTTata
OsSK23	P$ALFIN1_Q2	ALFIN1	586	601	1	1	0.897	atggagGTGGGtgat
OsSK23	P$C1_Q2	C1	620	631	1	1	0.927	atAACTAtgat
OsSK23	P$TEIL_01	TEIL	629	637	1	1	0.887	ATGTAagt
OsSK23	P$AZF3_01	AZF3	633	644	1	1	0.891	aAGTATcagtt
OsSK23	P$SEP3_01	wrz-03	641	652	1	1	0.904	gttttTTTTGg
OsSK23	P$ATHB6_01	ATHB6	661	670	1	1	0.913	gtAATAAga
OsSK23	P$HMG1_01	HMG1	680	689	1	1	0.948	GTTGTcttt
OsSK23	P$REF6_01	REF6	690	701	1	1	0.861	gagaCAGAGat
OsSK23	P$ARR18_01	ARR18	693	706	1	1	0.94	acagAGATAggcc
OsSK23	P$GATA9_01	GATA9	709	720	1	1	0.898	tttAGATCcca
OsSK23	P$AGP1_01	AGP1	710	720	1	1	0.875	ttAGATCcca
OsSK23	P$GATA10_01	GATA10	711	719	1	1	0.884	tAGATCcc
OsSK23	P$PCF2_01	CF2	711	721	1	1	0.892	tagatCCCAC
OsSK23	P$TCP19_01	TCP19	711	721	1	1	0.91	tagatCCCAC
OsSK23	P$ARR10_01	ARR10	712	719	1	1	0.934	AGATCcc
OsSK23	P$AT3G60580_01	AT3G60580	712	719	1	1	0.852	agATCCC
OsSK23	P$TCP20L_01	TCP20L	712	721	1	1	0.884	agatCCCAC
OsSK23	P$TCP20_02	TCP20	713	723	1	1	0.908	gatCCCACcc
OsSK23	P$ARALY495258_02	ARALY495258	713	721	1	1	0.918	gatCCCAC
OsSK23	P$ARALY484486_05	ARALY484486	713	721	1	1	0.918	gatCCCAC
OsSK23	P$MYB1L_01	MYB1L	718	728	1	1	0.984	caCCCTAaaa
OsSK23	P$TRB2_01	TRB2	718	726	1	1	0.965	caCCCTAa
OsSK23	P$LEC2_01	LEC2	734	745	1	1	0.942	acCATGCcata
OsSK23	P$SBF1_01	SBF1	747	761	1	1	0.86	gaatgtTTAAAcac
OsSK23	P$AP2A_01	AP2A	752	762	1	1	0.881	tttaaACACC
OsSK23	P$AT3G20750_01	AT3G20750	753	761	1	1	0.857	tTAAACac
OsSK23	P$MYB61_01	MYB61	756	771	1	1	0.86	aacACCTAcatgaag
OsSK23	P$ATHSFA1D_01	ATHSFA1D	760	766	1	1	0.985	cCTACA
OsSK23	P$SBF1_01	SBF1	767	781	1	1	0.853	gaagtaTTAAAtat
OsSK23	P$AZF3_01	AZF3	768	779	1	1	0.872	aAGTATtaaat
OsSK23	P$PDF2_01	DF2	770	781	1	1	0.872	gtatTAAATat
OsSK23	P$GT1_Q6	GT1	784	791	1	1	0.971	GTAAAaa
OsSK23	P$ATHB6_01	ATHB6	788	797	1	1	0.903	aaAATAAct
OsSK23	P$C1_Q2	C1	791	802	1	1	0.935	atAACTAattg
OsSK23	P$ATSPL8_01	ATSPL8	795	811	1	1	0.912	ctaatTGTACagattg
OsSK23	P$ARR1_01	ARR1	825	835	1	1	0.995	gacGAATCtt
OsSK23	P$ARR2_01	ARR2	826	836	1	1	0.998	acgaATCTTt
OsSK23	P$RIN_Q2	RIN	831	842	1	1	0.877	tcttTTTAAgc
OsSK23	P$GATA15_01	GATA15	852	861	1	1	0.999	caTGATCtg
OsSK23	P$GATA11_01	GATA11	853	861	1	1	0.866	atGATCTg
OsSK23	P$GATA8_01	GATA8	853	862	1	1	0.987	atGATCTga
OsSK23	P$KNOX3_01	KNOX3	855	867	1	1	0.982	gatcTGACAatg
OsSK23	P$ATH1_01	ATH1	859	867	1	1	0.907	TGACAatg
OsSK23	P$ATHSFA1D_01	ATHSFA1D	871	877	1	1	0.94	gCTACA
OsSK23	P$FBP24_01	FBP24	874	892	1	1	0.86	acagTAAACatttactaa
OsSK23	P$GT1_Q6	GT1	877	884	1	1	0.912	GTAAAca
OsSK23	P$AT3G20750_01	AT3G20750	877	885	1	1	0.943	gTAAACat
OsSK23	P$TGA1_01	TGA1	889	900	1	1	0.935	taaTGACGgat
OsSK23	P$TGA7_01	TGA7	890	900	1	1	0.873	aaTGACGgat
OsSK23	P$TGA5_01	TGA5	891	899	1	1	0.87	aTGACGga
OsSK23	P$EDT1_01	EDT1	896	906	1	1	0.859	ggaTTAATta
OsSK23	P$SBF1_01	SBF1	903	917	1	1	0.955	ttatgcTTAATaaa
OsSK23	P$CBNAC_01	CBNAC	905	911	1	1	0.968	aTGCTT
OsSK23	P$CBNAC_02	CBNAC	905	921	1	1	0.892	aTGCTTaataaatttg
OsSK23	P$ATHB6_01	ATHB6	909	918	1	1	0.914	ttAATAAat
OsSK23	P$KNOX3_01	KNOX3	930	942	1	1	0.986	ttacTGACAgat
OsSK23	P$SIZF2_01	SIZF2	931	941	1	1	0.883	tacTGACAga
OsSK23	P$ATH1_01	ATH1	934	942	1	1	0.99	TGACAgat
OsSK23	P$UIF1_01	UIF1	936	946	1	1	0.978	acaGATTCtg
OsSK23	P$GT1_Q6_01	GT1	955	967	1	1	0.955	TTTTTtattagt
OsSK23	P$MYB1L_01	MYB1L	986	996	1	1	0.976	caCCCTAtat
OsSK23	P$TRB2_01	TRB2	986	994	1	1	0.949	caCCCTAt
OsSK23	P$ATHB6_01	ATHB6	1010	1019	1	1	0.997	tcAATAAta

OsSK23	P$ATHB5_04	ATHB5	1010	1021	1	1	0.924	tcAATAAtaat
OsSK23	P$ATHB1_03	ATHB1	1010	1021	1	1	0.905	tcAATAAtaat
OsSK23	P$ATHB16_01	ATHB16	1011	1019	1	1	0.878	cAATAAta
OsSK23	P$ATHB6_01	ATHB6	1013	1022	1	1	0.981	atAATAAta
OsSK23	P$ATHB5_04	ATHB5	1013	1024	1	1	0.905	atAATAAtaat
OsSK23	P$ATHB1_03	ATHB1	1013	1024	1	1	0.894	atAATAAtaat
OsSK23	P$ATHB16_01	ATHB16	1014	1022	1	1	0.915	tAATAAta
OsSK23	P$ATHB6_01	ATHB6	1016	1025	1	1	0.981	atAATAAta
OsSK23	P$ATHB5_04	ATHB5	1016	1027	1	1	0.909	atAATAAtata
OsSK23	P$ATHB1_03	ATHB1	1016	1027	1	1	0.901	atAATAAtata
OsSK23	P$ATHB16_01	ATHB16	1017	1025	1	1	0.915	tAATAAta
OsSK23	P$ATSPL8_01	ATSPL8	1044	1060	1	1	0.938	tttctTGTACtgtttg
OsSK23	P$GATA15_01	GATA15	1056	1065	1	1	0.999	ttTGATCat
OsSK23	P$PHV_02	HV	1057	1072	1	1	0.851	ttgATCATtcgtctt
OsSK23	P$ATHB4_02	ATHB4	1058	1068	1	1	0.871	tgATCATtcg
OsSK23	P$PEND_01	END	1074	1082	1	1	0.892	tAAGAAaa
OsSK23	P$BPC1_Q2	BPC1	1076	1082	1	1	0.997	AGAAAa
OsSK23	P$ATHB1_01	ATHB1	1081	1095	1	1	0.946	atagaATTATtatt
OsSK23	P$ATHB5_01	ATHB5	1084	1093	1	1	0.919	gaaTTATTa
OsSK23	P$SBF1_01	SBF1	1116	1130	1	1	0.853	tattatTTAAAgta
OsSK23	P$DOF1_01	DOF1	1120	1131	1	1	0.988	attTAAAGtac
OsSK23	P$EDT1_01	EDT1	1129	1139	1	1	0.851	actTTAATca
OsSK23	P$ATHB7_01	ATHB7	1132	1142	1	1	0.924	ttAATCAtaa
OsSK23	P$HAT1_01	HAT1	1132	1142	1	1	0.981	ttAATCAtaa
OsSK23	P$PHV_02	HV	1132	1147	1	1	0.903	ttaATCATaactttt
OsSK23	P$ATHB4_02	ATHB4	1133	1143	1	1	0.854	taATCATaac
OsSK23	P$SQUA_01	SQUA	1147	1157	1	1	0.862	cgtTTTTTat
OsSK23	P$ATSPL8_01	ATSPL8	1155	1171	1	1	0.968	atattTGTACaaattt
OsSK23	P$SBF1_01	SBF1	1166	1180	1	1	0.897	aattttTTAATaag
OsSK23	P$GT1_Q6_01	GT1	1168	1180	1	1	0.897	TTTTTtaataag
OsSK23	P$GT1_Q6_01	GT1	1169	1181	1	1	0.863	TTTTTaataaga
OsSK23	P$ATHB6_01	ATHB6	1172	1181	1	1	0.913	ttAATAAga
OsSK23	P$WRKY18_02	WRKY18	1184	1194	1	1	0.997	gtgGTCAAac
OsSK23	P$WRKY21_02	WRKY21	1184	1194	1	1	0.969	gtgGTCAAac
OsSK23	P$WRKY48_02	WRKY48	1184	1194	1	1	0.998	gtgGTCAAac
OsSK23	P$WRKY57_01	WRKY57	1184	1194	1	1	0.973	gtgGTCAAac
OsSK23	P$WRKY60_01	WRKY60	1184	1195	1	1	0.979	gtgGTCAAaca
OsSK23	P$WRKY15_01	WRKY15	1185	1195	1	1	0.985	tgGTCAAaca
OsSK23	P$WRKY2_01	WRKY2	1185	1193	1	1	0.989	tgGTCAAa
OsSK23	P$WRKY25_02	WRKY25	1185	1193	1	1	0.973	tgGTCAAa
OsSK23	P$WRKY40_01	WRKY40	1185	1193	1	1	1	tgGTCAAa
OsSK23	P$WRKY43_02	WRKY43	1185	1195	1	1	0.976	tgGTCAAaca
OsSK23	P$WRKY62_01	WRKY62	1185	1193	1	1	0.957	tgGTCAAa
OsSK23	P$WRKY63_01	WRKY63	1185	1193	1	1	0.99	tgGTCAAa
OsSK23	P$WRKY75_01	WRKY75	1185	1193	1	1	0.975	tgGTCAAa
OsSK23	P$WRKY8_01	WRKY8	1185	1194	1	1	0.992	tgGTCAAac
OsSK23	P$WRKY23_01	WRKY23	1186	1194	1	1	0.854	gGTCAAac
OsSK23	P$WRKY30_01	WRKY30	1186	1196	1	1	0.918	gGTCAAacag
OsSK23	P$WRKY18_Q2	WRKY18	1187	1196	1	1	0.927	GTCAAacag
OsSK23	P$RIN_Q2_01	RIN	1197	1209	1	1	0.902	gcaagcAAAAGa
OsSK23	P$PBF_01	BF	1200	1211	1	1	0.957	agcAAAAGatc
OsSK23	P$DOF_Q2	DOF	1200	1211	1	1	0.924	agcAAAAGatc
OsSK23	P$CDF2_01	CDF2	1201	1211	1	1	0.944	gcAAAAGatc
OsSK23	P$CDF3_01	CDF3	1202	1211	1	1	0.968	cAAAAGatc
OsSK23	P$GATA9_01	GATA9	1203	1214	1	1	0.887	aaaAGATCaaa
OsSK23	P$AGP1_01	AGP1	1204	1214	1	1	0.903	aaAGATCaaa
OsSK23	P$ARR10_01	ARR10	1206	1213	1	1	0.869	AGATCaa
OsSK23	P$SED_Q2	SED	1212	1222	1	1	0.916	aaatCCTTTa
OsSK23	P$PBF_Q2_01	BF	1216	1222	1	1	0.998	CCTTTa
OsSK23	P$HSF3_01	HSF3	1231	1237	1	1	0.969	aCGGGG
OsSK23	P$RAV1_02	RAV1	1239	1251	1	1	0.919	agtACCTGatgt
OsSK23	P$JERF3_01	JERF3	1254	1264	1	1	0.862	aTGCCGaaac
OsSK23	P$CEF1_01	CEF1	1254	1264	1	1	0.863	aTGCCGaaac
OsSK23	P$JERF1_01	JERF1	1254	1264	1	1	0.883	aTGCCGaaac
OsSK23	P$CBF1_03	CBF1	1254	1264	1	1	0.852	aTGCCGaaac
OsSK23	P$AT2G41690_01	AT2G41690	1257	1263	1	1	0.978	CCGAAa
OsSK23	P$AGL3_01	AGL3	1266	1284	1	1	0.879	tacaCCATAtagagtgtg
OsSK23	P$AGL2_01	AGL2	1266	1284	1	1	0.859	tacaCCATAtagagtgtg
OsSK23	P$ATMYB77_01	ATMYB77	1288	1301	1	1	0.926	aaatggCGGTTgg
OsSK23	P$E2L_Q2	E2L	1291	1298	1	1	0.892	tGGCGGt
OsSK23	P$ERF1_Q2	ERF1	1292	1300	1	1	0.905	GGCGGttg
OsSK23	P$AT3G51080_01	AT3G51080	1299	1306	1	1	1	GGAAAaa
OsSK23	P$SPF1_Q2	SPF1	1315	1325	1	1	0.949	gaATAGTatt
OsSK23	P$E2L1_Q2	E2L1	1321	1333	1	1	0.871	taTTGGCgacat
OsSK23	P$ARR1_01	ARR1	1351	1361	1	1	0.945	tggGAATCaa
OsSK23	P$ATHB7_01	ATHB7	1353	1363	1	1	0.882	ggAATCAatc
OsSK23	P$HAT1_01	HAT1	1353	1363	1	1	0.877	ggAATCAatc
OsSK23	P$ATHB7_01	ATHB7	1357	1367	1	1	0.96	tcAATCAata

OsSK23	P$HAT1_01	HAT1	1357	1367	1	1	0.892	tcAATCAata
OsSK23	P$ATHB6_01	ATHB6	1361	1370	1	1	0.997	tcAATAAta
OsSK23	P$ATHB5_04	ATHB5	1361	1372	1	1	0.92	tcAATAAtatc
OsSK23	P$ATHB1_03	ATHB1	1361	1372	1	1	0.897	tcAATAAtatc
OsSK23	P$ATHB16_01	ATHB16	1362	1370	1	1	0.878	cAATAAta
OsSK23	P$ATHB7_01	ATHB7	1378	1388	1	1	0.96	ccAATCAatt
OsSK23	P$HAT1_01	HAT1	1378	1388	1	1	0.894	ccAATCAatt
OsSK23	P$AZF2_01	AZF2	1392	1404	1	1	0.854	actttgAAGTAc
OsSK23	P$GT1_Q6	GT1	1416	1423	1	1	0.971	GTAAAaa
OsSK23	P$MYB118_01	MYB118	1423	1440	1	1	0.91	cttgtctacGTTACcta
OsSK23	P$ANTL_01	ANTL	1431	1441	1	1	0.874	cGTTACctat
OsSK23	P$O2_Q2	O2	1438	1451	1	1	0.883	tatgaGACGTtga
OsSK23	P$TGA1B_01	TGA1B	1441	1451	1	1	0.88	gaGACGTtga
OsSK23	P$WRKY11_01	WRKY11	1443	1457	1	1	0.86	gacgTTGACatgca
OsSK23	P$KNOX3_01	KNOX3	1444	1456	1	1	0.96	acgtTGACAtgc
OsSK23	P$WRKY11_Q2	WRKY11	1446	1454	1	1	0.904	gTTGACat
OsSK23	P$ATH1_01	ATH1	1448	1456	1	1	0.932	TGACAtgc
OsSK23	P$LEC2_01	LEC2	1449	1460	1	1	0.983	gaCATGCaata
OsSK23	P$DOF1_01	DOF1	1494	1505	1	1	0.975	tagTAAAGtaa
OsSK23	P$ARR18_01	ARR18	1505	1518	1	1	0.904	ttgtAGATAttta
OsSK23	P$RIN_Q2	RIN	1510	1521	1	1	0.873	gataTTTAAga
OsSK23	P$DOF1_01	DOF1	1522	1533	1	1	0.973	tagTAAAGtag
OsSK23	P$SBF1_01	SBF1	1565	1579	1	1	0.877	tctcttTTAAAata
OsSK23	P$ATHB6_01	ATHB6	1573	1582	1	1	0.979	aaAATAAtt
OsSK23	P$ATHB5_04	ATHB5	1573	1584	1	1	0.962	aaAATAAttca
OsSK23	P$ATHB1_03	ATHB1	1573	1584	1	1	0.981	aaAATAAttca
OsSK23	P$ATHB16_01	ATHB16	1574	1582	1	1	0.953	aAATAAtt
OsSK23	P$LEC2_01	LEC2	1598	1609	1	1	0.988	acCATGCagct
OsSK23	P$HSFA2_01	HSFA2	1618	1624	1	1	0.941	CCAAAc
OsSK23	P$TCP24_01	TCP24	1621	1633	1	1	0.89	aacaGGACCacg
OsSK23	P$ARALY897773_01	ARALY897773	1622	1632	1	1	0.869	acaggACCAC
OsSK23	P$TCP4_01	TCP4	1624	1632	1	1	0.951	aGGACCac
OsSK23	P$TCP5_01	TCP5	1624	1632	1	1	0.866	aGGACCac
OsSK23	P$ARALY496250_03	ARALY496250	1624	1632	1	1	0.877	aGGACCac
OsSK23	P$NAC080_01	NAC080	1628	1636	1	1	0.954	cCACGCaa
OsSK23	P$NAC083_01	NAC083	1628	1638	1	1	0.967	ccACGCAatt
OsSK23	P$ATSPL8_01	ATSPL8	1636	1652	1	1	0.951	ttcacTGTACttgtat
OsSK23	P$RAV2_01	RAV2	1658	1667	1	1	0.861	cgACCGAtc
OsSK23	P$NAC080_01	NAC080	1673	1681	1	1	0.954	cCACGCaa
OsSK23	P$NAC083_01	NAC083	1673	1683	1	1	0.964	ccACGCAaaa
OsSK23	P$ABZ1_01	ABZ1	1677	1691	1	1	0.873	gcaaaACGTGcgcg
OsSK23	P$TAF1_Q2	TAF1	1679	1689	1	1	0.908	aaaACGTGcg
OsSK23	P$EMBP1_02	EMBP1	1679	1689	1	1	0.891	aaaACGTGcg
OsSK23	P$TAF1_01	TAF1	1679	1689	1	1	0.941	aaaACGTGcg
OsSK23	P$GBF1_01	GBF1	1680	1688	1	1	0.911	aaACGTGc
OsSK23	P$MYC4_01	MYC4	1680	1688	1	1	0.863	aaACGTGc
OsSK23	P$BIM1_02	BIM1	1680	1690	1	1	0.949	aaACGTGcgc
OsSK23	P$BHLH13_01	BHLH13	1680	1688	1	1	0.856	aaACGTGc
OsSK23	P$ABF4_02	ABF4	1680	1690	1	1	0.943	aaACGTGcgc
OsSK23	P$ABI5_Q2	ABI5	1682	1688	1	1	0.936	ACGTGc
OsSK23	P$BZR1_01	BZR1	1683	1689	1	1	1	CGTGCg
OsSK23	P$SPL15_01	SPL15	1716	1730	1	1	0.934	caggaGTACGgagt
OsSK23	P$SPL11_01	SPL11	1717	1729	1	1	0.976	aggaGTACGgag
OsSK23	P$SPL5_02	SPL5	1717	1729	1	1	0.958	aggaGTACGgag
OsSK23	P$BHLH28_01	BHLH28	1717	1729	1	1	0.974	aggaGTACGgag
OsSK23	P$SPL5_01	SPL5	1719	1728	1	1	0.998	gaGTACGga
OsSK23	P$POPTR_01	OPTR	1720	1727	1	1	0.999	aGTACGg
OsSK23	P$SPL12_01	SPL12	1720	1728	1	1	0.999	aGTACGga
OsSK23	P$SPL4_01	SPL4	1720	1729	1	1	0.999	aGTACGgag
OsSK23	P$ARR18_01	ARR18	1726	1739	1	1	0.887	gagtAGATAacag
OsSK23	P$ALFIN1_Q2	ALFIN1	1732	1747	1	1	0.861	ataacaGTGGGaaga
OsSK23	P$ATMYB15_Q2	ATMYB15	1733	1739	1	1	0.865	TAACAg
OsSK23	P$PEND_01	END	1742	1750	1	1	0.859	gAAGAAtt
OsSK23	P$DOF1_01	DOF1	1751	1762	1	1	0.98	tctTAAAGacc
OsSK23	P$ARR18_01	ARR18	1776	1789	1	1	0.894	aagcAGATAgaaa
OsSK23	P$BPC1_Q2	BPC1	1784	1790	1	1	0.99	AGAAAt
OsSK23	P$UIF1_01	UIF1	1813	1823	1	1	0.979	ggaGATTCta
OsSK23	P$SPF1_Q2	SPF1	1827	1837	1	1	0.935	gaATAGTagt
OsSK23	P$RAV1_02	RAV1	1854	1866	1	1	0.91	aatACCTGcaaa
OsSK23	P$PBF_01	BF	1860	1871	1	1	0.954	tgcAAAAGgac
OsSK23	P$DOF_Q2	DOF	1860	1871	1	1	0.933	tgcAAAAGgac
OsSK23	P$CDF2_01	CDF2	1861	1871	1	1	0.952	gcAAAAGgac
OsSK23	P$CDF3_01	CDF3	1862	1871	1	1	0.973	cAAAAGgac
OsSK23	P$PBF_Q2	BF	1863	1869	1	1	1	aAAAGG
OsSK23	P$SED_Q2	SED	1866	1876	1	1	0.926	aggaCCTTTt
OsSK23	P$PBF_Q2_01	BF	1870	1876	1	1	1	CCTTTt
OsSK23	P$PEND_01	END	1883	1891	1	1	0.877	tAAGAAtg
OsSK23	P$PEND_01	END	1899	1907	1	1	0.877	tAAGAAta

OsSK23	P$ATHB6_01	ATHB6	1901	1910	1	1	0.901	agAATAAaa
OsSK23	P$UIF1_01	UIF1	1916	1926	1	1	0.857	tagGATTCtg
OsSK23	P$KNOX3_01	KNOX3	1920	1932	1	1	0.981	attcTGACAgaa
OsSK23	P$ATH1_01	ATH1	1924	1932	1	1	0.991	TGACAgaa
OsSK23	P$MADSA_Q2	MADSA	1924	1940	1	1	0.861	tgacagAAAATgcaag
OsSK23	P$BPC1_Q2	BPC1	1928	1934	1	1	0.997	AGAAAa
OsSK23	P$HAHB4_01	HAHB4	1948	1957	1	1	0.874	aAATGAttg
OsSK23	P$C1_Q2	C1	1957	1968	1	1	0.978	caAACTAcagg
OsSK23	P$ATHSFA1D_01	ATHSFA1D	1960	1966	1	1	1	aCTACA
OsSK23	P$AT3G51080_01	AT3G51080	1966	1973	1	1	1	GGAAAaa
OsSK23	P$BPC1_Q2	BPC1	1976	1982	1	1	0.997	AGAAAa
OsSK23	P$PEND_01	END	1999	2007	1	1	0.92	tAAGAAga
OsSK23	P$ARR1_01	ARR1	2010	2020	1	1	0.948	caaGAATCag
OsSK23	P$HAT1_01	HAT1	2012	2022	1	1	0.851	agAATCAgat
OsSK23	P$ARR18_01	ARR18	2014	2027	1	1	0.94	aatcAGATAagag
OsSK23	P$ARR18_01	ARR18	2023	2036	1	1	0.895	agagAGATAgact
OsSK23	P$BPC1_Q2	BPC1	2039	2045	1	1	0.997	AGAAAa
OsSK23	P$PBF_01	BF	2048	2059	1	1	0.944	tctAAAAGgtt
OsSK23	P$DOF_Q2	DOF	2048	2059	1	1	0.928	tctAAAAGgtt
OsSK23	P$CDF2_01	CDF2	2049	2059	1	1	0.952	ctAAAAGgtt
OsSK23	P$CDF3_01	CDF3	2050	2059	1	1	0.971	tAAAAGgtt
OsSK23	P$PBF_Q2	BF	2051	2057	1	1	1	aAAAGG
OsSK23	P$GATA9_01	GATA9	2080	2091	1	1	0.98	gtaAGATCttt
OsSK23	P$AGP1_01	AGP1	2081	2091	1	1	0.942	taAGATCttt
OsSK23	P$ARR2_01	ARR2	2081	2091	1	1	0.899	taagATCTTt
OsSK23	P$GATA10_01	GATA10	2082	2090	1	1	0.912	aAGATCtt
OsSK23	P$GATA11_01	GATA11	2082	2090	1	1	0.949	aaGATCTt
OsSK23	P$GATA8_01	GATA8	2082	2091	1	1	0.987	aaGATCTtt
OsSK23	P$ARR10_01	ARR10	2083	2090	1	1	0.956	AGATCtt
OsSK23	P$PBF_Q2	BF	2118	2124	1	1	0.958	cAAAGG
OsSK23	P$UIF1_01	UIF1	2130	2140	1	1	0.856	tagGATTCaa
OsSK23	P$GAMYB_01	GAMYB	2137	2145	1	1	0.935	CAACCctc
OsSK23	P$HSFA4A_01	HSFA4A	2143	2149	1	1	0.914	tCTATT
OsSK23	P$PBF_Q2	BF	2150	2156	1	1	0.958	cAAAGG
OsSK23	P$SBF1_01	SBF1	2175	2189	1	1	0.889	tgaaaaTTAAAatc
OsSK23	P$CCA1_Q5	CCA1	2188	2205	1	1	0.878	ccttgAAAATttctatg
OsSK23	P$MRP1_Q2	MRP1	2197	2209	1	1	0.871	ttTCTATgtttt
OsSK23	P$AG_02	AG	2208	2224	1	1	0.861	TTTCCtccagaggaaa
OsSK23	P$PBF_Q2	BF	2220	2226	1	1	0.965	gAAAGG
OsSK23	P$SPF1_Q2	SPF1	2242	2252	1	1	0.92	acATAGTtgt
OsSK23	P$O2_Q4	O2	2255	2266	1	1	0.866	tattCATGTtc
OsSK23	P$RRTF1_05	RRTF1	2274	2289	1	1	0.919	ttcctgtCGGCGggg
OsSK23	P$ARF8_01	ARF8	2276	2285	1	1	0.997	ccTGTCGgc
OsSK23	P$DREB1A_04	DREB1A	2277	2287	1	1	0.981	ctGTCGGcgg
OsSK23	P$ERF039_01	ERF039	2277	2287	1	1	0.99	ctGTCGGcgg
OsSK23	P$PHYPA182268_05	HYPA182268	2277	2287	1	1	0.888	ctGTCGGcgg
OsSK23	P$ERF043_01	ERF043	2278	2286	1	1	0.953	tGTCGGcg
OsSK23	P$PHYPA173530_04	HYPA173530	2278	2286	1	1	0.918	tGTCGGcg
OsSK23	P$PHYPA28324_10	HYPA28324	2278	2286	1	1	0.94	tGTCGGcg
OsSK23	P$AT1G28160_02	AT1G28160	2278	2293	1	1	0.921	tgtCGGCGggatac
OsSK23	P$RAP26_06	RAP26	2278	2293	1	1	0.902	tgtCGGCGggatac
OsSK23	P$AT1G68550_03	AT1G68550	2278	2287	1	1	0.959	tgtCGGCGg
OsSK23	P$E2L_Q2	E2L	2281	2288	1	1	0.928	cGGCGGg
OsSK23	P$HSF3_01	HSF3	2283	2289	1	1	0.945	gCGGGG
OsSK23	P$SPL14_01	SPL14	2293	2300	1	1	0.851	CCGTAga
OsSK23	P$AT4G12750_01	AT4G12750	2296	2306	1	1	0.864	tagACCGAat
OsSK23	P$AT2G41690_01	AT2G41690	2300	2306	1	1	0.974	CCGAAt
OsSK23	P$SPF1_Q2	SPF1	2302	2312	1	1	0.898	gaATAGTatg
OsSK23	P$SPL11_01	SPL11	2317	2329	1	1	0.903	tgggGTACGttg
OsSK23	P$SPL5_01	SPL5	2319	2328	1	1	0.973	ggGTACGtt
OsSK23	P$POPTR_01	OPTR	2320	2327	1	1	0.931	gGTACGt
OsSK23	P$SPL12_01	SPL12	2320	2328	1	1	0.977	gGTACGtt
OsSK23	P$SPL4_01	SPL4	2320	2329	1	1	0.992	gGTACGttg
OsSK23	P$GATA15_01	GATA15	2333	2342	1	1	0.999	gaTGATCta
OsSK23	P$GATA8_01	GATA8	2334	2343	1	1	0.986	atGATCTat
OsSK23	P$ATHSFA1D_01	ATHSFA1D	2348	2354	1	1	0.941	tCTACA
OsSK23	P$ZAT1_01	ZAT1	2356	2367	1	1	0.858	gagcaaACAAA
OsSK23	P$PBF_01	BF	2362	2373	1	1	0.968	acaAAAAGaca
OsSK23	P$DOF_Q2	DOF	2362	2373	1	1	0.984	acaAAAAGaca
OsSK23	P$CDF2_01	CDF2	2363	2373	1	1	0.971	caAAAAGaca
OsSK23	P$CDF3_01	CDF3	2364	2373	1	1	0.971	aAAAAGaca
OsSK23	P$PEND_01	END	2371	2379	1	1	0.856	cAAGAAtt
OsSK23	P$NAC6_01	NAC6	2396	2402	1	1	0.854	tCGTAA
OsSK23	P$ARR18_01	ARR18	2397	2410	1	1	0.887	cgtaAGATAatag
OsSK23	P$MYB1L_01	MYB1L	2408	2418	1	1	0.985	agCCCTAatc
OsSK23	P$TRB2_01	TRB2	2408	2416	1	1	0.998	agCCCTAa
OsSK23	P$SPF1_Q2	SPF1	2447	2457	1	1	0.954	acATAGTaca
OsSK23	P$BPC1_Q2	BPC1	2459	2465	1	1	0.997	AGAAAa

OsSK23	P$GAMYB_Q2	GAMYB	2459	2472	1	1	0.928	agaaaACAACtga
OsSK23	P$P_01		2478	2487	1	1	0.882	ggCTACCag
OsSK23	P$GATA9_01	GATA9	2486	2497	1	1	0.897	gcgAGATCctt
OsSK23	P$AGP1_01	AGP1	2487	2497	1	1	0.878	cgAGATCctt
OsSK23	P$ARR10_01	ARR10	2489	2496	1	1	0.978	AGATCct
OsSK23	P$O2_Q2	O2	2506	2519	1	1	0.886	gtctcGACGTgat
OsSK23	P$ABZ1_01	ABZ1	2507	2521	1	1	0.891	tctcgACGTGatct
OsSK23	P$TGA1B_01	TGA1B	2509	2519	1	1	0.898	tcGACGTgat
OsSK23	P$HBP1A_Q2	HBP1A	2509	2519	1	1	0.88	tcgACGTGat
OsSK23	P$TAF1_Q2	TAF1	2509	2519	1	1	0.942	tcgACGTGat
OsSK23	P$EMBP1_02	EMBP1	2509	2519	1	1	0.857	tcgACGTGat
OsSK23	P$TAF1_01	TAF1	2509	2519	1	1	0.958	tcgACGTGat
OsSK23	P$GBF1_01	GBF1	2510	2518	1	1	0.93	cgACGTGa
OsSK23	P$BIM1_02	BIM1	2510	2520	1	1	0.948	cgACGTGatc
OsSK23	P$ABF4_02	ABF4	2510	2520	1	1	0.949	cgACGTGatc
OsSK23	P$ABI5_Q2	ABI5	2512	2518	1	1	0.936	ACGTGa
OsSK23	P$GATA15_01	GATA15	2513	2522	1	1	0.999	cgTGATCta
OsSK23	P$GATA8_01	GATA8	2514	2523	1	1	0.986	gtGATCTac
OsSK23	P$CBNAC_01	CBNAC	2550	2556	1	1	1	tTGCTT
OsSK23	P$CBNAC_02	CBNAC	2550	2566	1	1	0.865	tTGCTTctatggtggc
OsSK23	P$E2L_Q2	E2L	2562	2569	1	1	1	tGGCGGg
OsSK23	P$ERF1_Q2	ERF1	2563	2571	1	1	0.887	GGCGGgtt
OsSK23	P$ATHB1_01	ATHB1	2576	2590	1	1	0.864	gtgctATTATttga
OsSK23	P$HSFA4A_01	HSFA4A	2578	2584	1	1	0.91	gCTATT
OsSK23	P$ATHB5_01	ATHB5	2579	2588	1	1	0.909	ctaTTATTt
OsSK23	P$TCP16_01	TCP16	2594	2604	1	1	0.871	ctGGACCctt
OsSK23	P$TEIL_01	TEIL	2611	2619	1	1	0.925	ATGTAttt
OsSK23	P$ARR1_01	ARR1	2623	2633	1	1	0.943	cgtGAATCcc
OsSK23	P$AT3G60580_01	AT3G60580	2626	2633	1	1	0.932	gaATCCC
OsSK23	P$AT1G34190_Q2	AT1G34190	2633	2646	1	1	0.937	cttagtCTCCAag
OsSK23	P$AT3G10500_Q2	AT3G10500	2633	2646	1	1	0.937	cttagtCTCCAag
OsSK23	P$ANAC013_Q2	ANAC013	2633	2647	1	1	0.91	cttagtctCCAAGt
OsSK23	P$AGL15_01	AGL15	2668	2684	1	1	0.905	ttacatgaTATAGtat
OsSK23	P$SPF1_Q2	SPF1	2675	2685	1	1	0.936	atATAGTata
OsSK23	P$MYBAS1_01	MYBAS1	2686	2697	1	1	0.98	tcCTAACcctt
OsSK23	P$WRKY25_01	WRKY25	2686	2695	1	1	0.923	tccTAACCc
OsSK23	P$WRKY33_01	WRKY33	2686	2695	1	1	0.945	tccTAACCc
OsSK23	P$AT1G29860_01	AT1G29860	2686	2695	1	1	0.892	tccTAACCc
OsSK23	P$AT3G62340_01	AT3G62340	2686	2695	1	1	0.922	tccTAACCc
OsSK23	P$AT1G69310_01	AT1G69310	2686	2695	1	1	0.913	tccTAACCc
OsSK23	P$WRKY26_01	WRKY26	2686	2695	1	1	0.945	tccTAACCc
OsSK23	P$SED_Q2	SED	2689	2699	1	1	0.913	taacCCTTTt
OsSK23	P$PBF_Q2_01	BF	2693	2699	1	1	1	CCTTTt
OsSK23	P$ID1_01	ID1	2727	2738	1	1	0.868	cTTGTCgattt
OsSK23	P$ARF8_01	ARF8	2727	2736	1	1	0.958	ctTGTCGat
OsSK23	P$PEND_02	END	2735	2745	1	1	0.914	ttTTCTTatc
OsSK23	P$O2_Q2	O2	2773	2786	1	1	0.851	ggtgtGACGTatc
OsSK23	P$TGA1_01	TGA1	2774	2785	1	1	0.977	gtgTGACGtat
OsSK23	P$STF1_02	STF1	2775	2787	1	1	0.937	tgTGACGtatct
OsSK23	P$TGA3_Q2	TGA3	2775	2784	1	1	0.965	tgTGACGta
OsSK23	P$TGA6_01	TGA6	2775	2785	1	1	0.959	tgTGACGtat
OsSK23	P$TGA7_01	TGA7	2775	2785	1	1	0.959	tgTGACGtat
OsSK23	P$BZIP14_01	BZIP14	2775	2785	1	1	0.991	tgTGACGtat
OsSK23	P$STF1_01	STF1	2775	2787	1	1	0.953	tgtGACGTatct
OsSK23	P$TGA5_01	TGA5	2776	2784	1	1	0.995	gTGACGta
OsSK23	P$TGA1B_01	TGA1B	2776	2786	1	1	0.945	gtGACGTatc
OsSK23	P$NAC043_01	NAC043	2776	2786	1	1	0.89	gtgACGTAtc
OsSK23	P$SBF1_01	SBF1	2803	2817	1	1	0.894	gtagaaTTAATata
OsSK23	P$EDT1_01	EDT1	2806	2816	1	1	0.889	gaaTTAATat
OsSK23	P$WRKY25_01	WRKY25	2828	2837	1	1	0.884	ccgTAACCa
OsSK23	P$AT1G69310_01	AT1G69310	2828	2837	1	1	0.857	ccgTAACCa
OsSK23	P$AT4G36620_01	AT4G36620	2829	2837	1	1	0.874	cgtAACCA
OsSK23	P$KNOX3_01	KNOX3	2833	2845	1	1	0.968	accaTGACAcct
OsSK23	P$ATH1_01	ATH1	2837	2845	1	1	0.919	TGACAcct
OsSK23	P$ATHSFA1D_01	ATHSFA1D	2846	2852	1	1	0.985	cCTACA
OsSK23	P$SED_Q2	SED	2857	2867	1	1	0.965	ttcgCCTTTc
OsSK23	P$PBF_Q2_01	BF	2861	2867	1	1	0.985	CCTTTc
OsSK23	P$SED_Q2	SED	2876	2886	1	1	0.929	ggatCCTTTt
OsSK23	P$GT1_Q6_02	GT1	2879	2891	1	1	0.975	tcctttTTAACa
OsSK23	P$PBF_Q2_01	BF	2880	2886	1	1	1	CCTTTt
OsSK23	P$PEND_02	END	2928	2938	1	1	0.914	ctTTCTTatc
OsSK23	P$ARR2_01	ARR2	2931	2941	1	1	0.891	tcttATCTTt
OsSK23	P$HSFA2_01	HSFA2	2969	2975	1	1	1	CCAAAa
OsSK23	P$SBF1_01	SBF1	2989	3003	1	1	0.904	atttttTTAAAaaa
OsSK23	P$GT1_Q6_01	GT1	2990	3002	1	1	0.86	TTTTTttaaaaa
OsSK23	P$ATHB7_01	ATHB7	3025	3035	1	1	0.913	aaAATCAtaa
OsSK23	P$HAT1_01	HAT1	3025	3035	1	1	0.97	aaAATCAtaa
OsSK23	P$ATHB6_01	ATHB6	3031	3040	1	1	0.981	atAATAAta

OsSK23	P$ATHB5_04	ATHB5	3031	3042	1	1	0.905	atAATAAtaat
OsSK23	P$ATHB1_03	ATHB1	3031	3042	1	1	0.894	atAATAAtaat
OsSK23	P$ATHB16_01	ATHB16	3032	3040	1	1	0.915	tAATAAta
OsSK23	P$ATHB6_01	ATHB6	3034	3043	1	1	0.981	atAATAAta
OsSK23	P$ATHB5_04	ATHB5	3034	3045	1	1	0.904	atAATAAtatt
OsSK23	P$ATHB1_03	ATHB1	3034	3045	1	1	0.892	atAATAAtatt
OsSK23	P$ATHB16_01	ATHB16	3035	3043	1	1	0.915	tAATAAta
OsSK23	P$WRKY40_03	WRKY40	3045	3055	1	1	0.995	agAGTCAatt
OsSK23	P$WRKY18_02	WRKY18	3045	3055	1	1	0.978	agaGTCAAtt
OsSK23	P$WRKY21_02	WRKY21	3045	3055	1	1	0.963	agaGTCAAtt
OsSK23	P$WRKY48_02	WRKY48	3045	3055	1	1	0.993	agaGTCAAtt
OsSK23	P$WRKY57_01	WRKY57	3045	3055	1	1	0.978	agaGTCAAtt
OsSK23	P$WRKY60_01	WRKY60	3045	3056	1	1	0.894	agaGTCAAttg
OsSK23	P$WRKY15_01	WRKY15	3046	3056	1	1	0.974	gaGTCAAttg
OsSK23	P$WRKY2_01	WRKY2	3046	3054	1	1	0.935	gaGTCAAt
OsSK23	P$WRKY25_02	WRKY25	3046	3054	1	1	0.91	gaGTCAAt
OsSK23	P$WRKY40_01	WRKY40	3046	3054	1	1	0.996	gaGTCAAt
OsSK23	P$WRKY43_02	WRKY43	3046	3056	1	1	0.985	gaGTCAAttg
OsSK23	P$WRKY63_01	WRKY63	3046	3054	1	1	0.904	gaGTCAAt
OsSK23	P$WRKY75_01	WRKY75	3046	3054	1	1	0.967	gaGTCAAt
OsSK23	P$WRKY8_01	WRKY8	3046	3055	1	1	0.987	gaGTCAAtt
OsSK23	P$WRKY23_01	WRKY23	3047	3055	1	1	0.862	aGTCAAtt
OsSK23	P$WRKY30_01	WRKY30	3047	3057	1	1	0.92	aGTCAAttgc
OsSK23	P$WRKY18_Q2	WRKY18	3048	3057	1	1	0.952	GTCAAttgc
OsSK23	P$SPL11_01	SPL11	3060	3072	1	1	0.901	ggtgGTACGcaa
OsSK23	P$SPL5_01	SPL5	3062	3071	1	1	0.964	tgGTACGca
OsSK23	P$POPTR_01	OPTR	3063	3070	1	1	0.929	gGTACGc
OsSK23	P$SPL12_01	SPL12	3063	3071	1	1	0.971	gGTACGca
OsSK23	P$SPL4_01	SPL4	3063	3072	1	1	0.988	gGTACGcaa
OsSK23	P$NAC083_01	NAC083	3064	3074	1	1	0.985	gtACGCAaac
OsSK23	P$AT3G20750_01	AT3G20750	3099	3107	1	1	0.938	aTAAACtt
OsSK23	P$MYB3R5_01	MYB3R5	3107	3122	1	1	0.885	gtgaaatatCCGTTt
OsSK23	P$MYB3R1_01	MYB3R1	3108	3123	1	1	0.912	tgaaatatCCGTTtc
OsSK23	P$MYB3R4_01	MYB3R4	3108	3123	1	1	0.913	tgaaatatCCGTTtc
OsSK23	P$BZR1_01	BZR1	3144	3150	1	1	0.915	CGTGCa
OsSK23	P$HSFA2_01	HSFA2	3158	3164	1	1	1	CCAAAa
OsSK23	P$CBNAC_01	CBNAC	3175	3181	1	1	1	tTGCTT
OsSK23	P$CBNAC_02	CBNAC	3175	3191	1	1	0.858	tTGCTTggtttttctt
OsSK23	P$GT1_Q6_01	GT1	3183	3195	1	1	0.899	TTTTTcttcatc
OsSK23	P$HSFA4A_01	HSFA4A	3220	3226	1	1	0.914	tCTATT
OsSK23	P$SQUA_01	SQUA	3221	3231	1	1	0.915	ctaTTTTTTtt
OsSK23	P$GT1_Q6_01	GT1	3226	3238	1	1	0.863	TTTTTtattgaa
OsSK23	P$GT1_Q6_01	GT1	3227	3239	1	1	0.911	TTTTTattgaag
OsSK23	P$O2_Q4	O2	3240	3251	1	1	0.896	tgtgCATGTct
OsSK23	P$ABI3_01	ABI3	3241	3250	1	1	0.859	gtGCATGtc
OsSK23	P$GAMYB_01	GAMYB	3256	3264	1	1	0.924	CAACCttc
OsSK23	P$ARR18_01	ARR18	3262	3275	1	1	0.909	tctcAGATAtatg
OsSK23	P$SBF1_01	SBF1	3270	3284	1	1	0.852	atatgtTTAATcaa
OsSK23	P$ATHB7_01	ATHB7	3276	3286	1	1	0.882	ttAATCAacc
OsSK23	P$HAT1_01	HAT1	3276	3286	1	1	0.875	ttAATCAacc
OsSK23	P$GAMYB_01	GAMYB	3281	3289	1	1	0.86	CAACCtct
OsSK23	P$ATHB7_01	ATHB7	3288	3298	1	1	0.884	taAATCAata
OsSK23	P$HAT1_01	HAT1	3288	3298	1	1	0.876	taAATCAata
OsSK23	P$ATHB6_01	ATHB6	3292	3301	1	1	0.929	tcAATAAga
OsSK23	P$O2_Q4	O2	3302	3313	1	1	0.876	tagtCATGTag
OsSK23	P$SED_Q2	SED	3312	3322	1	1	0.968	gcgtCCTTTt
OsSK23	P$PBF_Q2_01	BF	3316	3322	1	1	1	CCTTTt
OsSK23	P$HSFA2_01	HSFA2	3357	3363	1	1	1	CCAAAa
OsSK23	P$BZR1_01	BZR1	3380	3386	1	1	0.915	CGTGCa
OsSK23	P$MYB61_01	MYB61	3392	3407	1	1	0.884	tgcACCTActgacaa
OsSK23	P$KNOX3_01	KNOX3	3397	3409	1	1	0.975	ctacTGACAaat
OsSK23	P$ATH1_01	ATH1	3401	3409	1	1	0.922	TGACAaat
OsSK23	P$ATSPL8_01	ATSPL8	3407	3423	1	1	0.896	attcgTGTACtgatga
OsSK23	P$GT1_Q6_02	GT1	3446	3458	1	1	0.994	agcattTTAACa
OsSK23	P$WRKY48_01	WRKY48	3450	3459	1	1	1	ttttAACAA
OsSK23	P$ATMYB15_Q2	ATMYB15	3453	3459	1	1	1	TAACAa
OsSK23	P$AT3G51080_01	AT3G51080	3470	3477	1	1	1	GGAAAaa
OsSK23	P$ARR1_01	ARR1	3490	3500	1	1	0.98	aatGAATCtg
OsSK23	P$SED_Q2	SED	3516	3526	1	1	0.957	acatCCTTTc
OsSK23	P$PBF_Q2_01	BF	3520	3526	1	1	0.985	CCTTTc
OsSK23	P$AT2G38090_01	AT2G38090	3520	3532	1	1	0.974	cctTTCGTaggt
OsSK23	P$MYB3_01	MYB3	3524	3535	1	1	0.901	tcgTAGGTtac
OsSK23	P$MYB4_01	MYB4	3525	3533	1	1	0.917	cgTAGGTt
OsSK23	P$ANTL_01	ANTL	3529	3539	1	1	0.864	gGTTACcttt
OsSK23	P$SED_Q2	SED	3530	3540	1	1	0.997	gttaCCTTTt
OsSK23	P$PBF_Q2_01	BF	3534	3540	1	1	1	CCTTTt
OsSK23	P$SQUA_01	SQUA	3534	3544	1	1	0.893	cctTTTTTTtg
OsSK23	P$SEP3_01	wrz-03	3534	3545	1	1	0.962	cctttTTTTGg

OsSK23	P$SQUA_01	SQUA	3535	3545	1	1	0.955	cttTTTTTgg
OsSK23	P$ATSPL3_01	ATSPL3	3552	3568	1	1	0.982	gattaCGTACttaatt
OsSK23	P$NAC043_01	NAC043	3553	3563	1	1	0.922	attACGTACt
OsSK23	P$SPL14_03	SPL14	3555	3566	1	1	0.877	taCGTACttaa
OsSK23	P$WEREWOLF_Q2	WEREWOLF	3601	3610	1	1	0.906	gaGTTAGta
OsSK23	P$SPL11_01	SPL11	3603	3615	1	1	0.906	gttaGTACGaac
OsSK23	P$BHLH28_01	BHLH28	3603	3615	1	1	0.857	gttaGTACGaac
OsSK23	P$SPL5_01	SPL5	3605	3614	1	1	0.972	taGTACGaa
OsSK23	P$POPTR_01	OPTR	3606	3613	1	1	0.943	aGTACGa
OsSK23	P$SPL12_01	SPL12	3606	3614	1	1	0.982	aGTACGaa
OsSK23	P$SPL4_01	SPL4	3606	3615	1	1	0.994	aGTACGaac
OsSK23	P$SPL11_01	SPL11	3614	3626	1	1	0.908	caaaGTACGttc
OsSK23	P$SPL5_01	SPL5	3616	3625	1	1	0.972	aaGTACGtt
OsSK23	P$POPTR_01	OPTR	3617	3624	1	1	0.93	aGTACGt
OsSK23	P$SPL12_01	SPL12	3617	3625	1	1	0.977	aGTACGtt
OsSK23	P$SPL4_01	SPL4	3617	3626	1	1	0.992	aGTACGttc
OsSK23	P$C1_Q2	C1	3625	3636	1	1	0.959	cgAACTAaacc
OsSK23	P$AT3G20750_01	AT3G20750	3629	3637	1	1	0.9	cTAAACct
OsSK23	P$MYB52_01	MYB52	3645	3657	1	1	0.9	agtAACGGaaag
OsSK23	P$AT1G26780_01	AT1G26780	3645	3657	1	1	0.86	agtAACGGaaag
OsSK23	P$DOF2_01	DOF2	3649	3660	1	1	0.983	acggAAAGCta
OsSK23	P$DOF3_01	DOF3	3649	3660	1	1	0.981	acggAAAGCta
OsSK23	P$ATHSFA1D_01	ATHSFA1D	3656	3662	1	1	0.94	gCTACA
OsSK23	P$ATHB6_01	ATHB6	3660	3669	1	1	0.973	caAATAAtc
OsSK23	P$ATHB5_04	ATHB5	3660	3671	1	1	0.881	caAATAAtctt
OsSK23	P$ATHB1_03	ATHB1	3660	3671	1	1	0.885	caAATAAtctt
OsSK23	P$ATHB16_01	ATHB16	3661	3669	1	1	0.86	aAATAAtc
OsSK23	P$ARR2_01	ARR2	3662	3672	1	1	0.884	aataATCTTc
OsSK23	P$HSFA2_01	HSFA2	3691	3697	1	1	0.922	CCAAAt
OsSK23	P$DOF1_01	DOF1	3697	3708	1	1	0.97	tccTAAAGggg
OsSK23	P$PBF_Q2	BF	3700	3706	1	1	0.986	tAAAGG
OsSK23	P$AT4G36620_01	AT4G36620	3707	3715	1	1	0.969	gtaAACCA
OsSK23	P$BPC1_Q2	BPC1	3714	3720	1	1	0.997	AGAAAa
OsSK23	P$AP1_01	AP1	3714	3727	1	1	0.89	agAAAAAaaggaa
OsSK23	P$AP1_01	AP1	3715	3728	1	1	0.921	gaAAAAAaggaaa
OsSK23	P$PI_02	I	3715	3729	1	1	0.937	gaaaaaaaGGAAAa
OsSK23	P$PBF_01	BF	3716	3727	1	1	0.974	aaaAAAAGgaa
OsSK23	P$DOF_Q2	DOF	3716	3727	1	1	0.999	aaaAAAAGgaa
OsSK23	P$CDF2_01	CDF2	3717	3727	1	1	0.982	aaAAAAGgaa
OsSK23	P$CDF3_01	CDF3	3718	3727	1	1	0.978	aAAAAGgaa
OsSK23	P$PBF_Q2	BF	3719	3725	1	1	1	aAAAGG
OsSK23	P$PBF_01	BF	3722	3733	1	1	0.954	aggAAAAGatg
OsSK23	P$DOF_Q2	DOF	3722	3733	1	1	0.944	aggAAAAGatg
OsSK23	P$AT3G51080_01	AT3G51080	3723	3730	1	1	0.918	GGAAAag
OsSK23	P$CDF2_01	CDF2	3723	3733	1	1	0.946	ggAAAAGatg
OsSK23	P$CDF3_01	CDF3	3724	3733	1	1	0.968	gAAAAGatg
OsSK23	P$TGA1_01	TGA1	3728	3739	1	1	0.95	agaTGACGgaa
OsSK23	P$TGA7_01	TGA7	3729	3739	1	1	0.897	gaTGACGgaa
OsSK23	P$TGA5_01	TGA5	3730	3738	1	1	0.87	aTGACGga
OsSK23	P$AT3G60580_01	AT3G60580	3743	3750	1	1	0.905	caATCCC
OsSK23	P$ABI3_01	ABI3	3749	3758	1	1	0.883	ccGCATGgg
OsSK23	P$FUS3_01	FUS3	3750	3759	1	1	0.856	cGCATGgga
OsSK23	P$UIF1_01	UIF1	3756	3766	1	1	0.979	ggaGATTCca
OsSK23	P$E2FA_02	E2FA	3765	3775	1	1	0.989	aagCGCCAga
OsSK23	P$KNOX3_01	KNOX3	3772	3784	1	1	0.986	agacTGACAgat
OsSK23	P$SIZF2_01	SIZF2	3773	3783	1	1	0.885	gacTGACAga
OsSK23	P$ATH1_01	ATH1	3776	3784	1	1	0.99	TGACAgat
OsSK23	P$WRKY11_Q2	WRKY11	3782	3790	1	1	0.978	aTTGACct
OsSK23	P$ZAP1_01	ZAP1	3783	3793	1	1	0.909	TTGACcttgt
OsSK23	P$GL15_01	GL15	3788	3798	1	1	0.996	cttgtCCCCC
OsSK23	P$MYB1L_01	MYB1L	3847	3857	1	1	0.936	ctCCCTAtag
OsSK23	P$TRB2_01	TRB2	3847	3855	1	1	0.92	ctCCCTAt
OsSK23	P$AT4G00870_01	AT4G00870	3871	3885	1	1	0.891	gaCCTCGcttcgga
OsSK23	P$CBF3_02	CBF3	3884	3898	1	1	0.948	acaagCCGACcccg
OsSK23	P$CBF1_04	CBF1	3885	3897	1	1	0.941	caagCCGACccc
OsSK23	P$AT5G67000_01	AT5G67000	3886	3896	1	1	0.902	aaGCCGAccc
OsSK23	P$ERF5_02	ERF5	3886	3896	1	1	0.915	aaGCCGAccc
OsSK23	P$ERF1_04	ERF1	3886	3896	1	1	0.905	aaGCCGAccc
OsSK23	P$DREB1G_02	DREB1G	3886	3896	1	1	0.901	aagCCGACcc
OsSK23	P$ARF5_01	ARF5	3888	3896	1	1	0.922	gCCGACcc
OsSK23	P$DREB1B_01	DREB1B	3889	3894	1	1	1	CCGAC
OsSK23	P$AT3G63350_01	AT3G63350	3895	3901	1	1	0.867	CCGCCt
OsSK23	P$HSFA4A_01	HSFA4A	3901	3907	1	1	0.91	gCTATT
OsSK23	P$E2F_Q2	E2F	3903	3914	1	1	0.913	tatTTCCCgaa
OsSK23	P$AT2G41690_01	AT2G41690	3909	3915	1	1	0.978	CCGAAa
OsSK23	P$RAP210_04	RAP210	3936	3946	1	1	0.853	aaCGCCGtct
OsSK23	P$ERF112_02	ERF112	3936	3946	1	1	0.965	aaCGCCGtct
OsSK23	P$CRF4_01	CRF4	3937	3945	1	1	0.924	aCGCCGtc

OsSK23	P$ERF4_04	ERF4	3937	3945	1	1	0.952	aCGCCGtc
OsSK23	P$ERF069_01	ERF069	3937	3946	1	1	0.993	aCGCCGtct
OsSK23	P$ERF11_01	ERF11	3937	3947	1	1	0.99	aCGCCGtctt
OsSK23	P$ERF8_01	ERF8	3938	3948	1	1	0.986	CGCCGtcttc
OsSK23	P$ERF3_04	ERF3	3938	3946	1	1	0.943	CGCCGtct
OsSK23	P$ATHB6_01	ATHB6	3970	3979	1	1	0.902	ggAATAAaa
OsSK23	P$AP1_01	AP1	3973	3986	1	1	0.859	atAAAAAaacaaa
OsSK23	P$PI_02	I	3980	3994	1	1	0.917	aacaaagaGGAAAa
OsSK23	P$AT3G51080_01	AT3G51080	3988	3995	1	1	1	GGAAAaa
OsSK23	P$AP1_01	AP1	3989	4002	1	1	0.857	gaAAAAAaaggaa
OsSK23	P$PBF_01	BF	3991	4002	1	1	0.974	aaaAAAAGgaa
OsSK23	P$DOF_Q2	DOF	3991	4002	1	1	0.999	aaaAAAAGgaa
OsSK23	P$CDF2_01	CDF2	3992	4002	1	1	0.982	aaAAAAGgaa
OsSK23	P$CDF3_01	CDF3	3993	4002	1	1	0.978	aAAAAGgaa
OsSK23	P$PBF_Q2	BF	3994	4000	1	1	1	aAAAGG
OsSK21	P$ERF6_02	ERF6	3	13	1	1	0.978	taGCCGGcct
OsSK21	P$ERF105_02	ERF105	4	12	1	1	0.929	aGCCGGcc
OsSK21	P$MYB24_01	MYB24	22	31	1	1	0.862	attTTAGGa
OsSK21	P$UIF1_01	UIF1	26	36	1	1	0.856	tagGATTCaa
OsSK21	P$ABF2_01	ABF2	33	46	1	1	0.95	caagaCACGTtcc
OsSK21	P$BZR1_02	BZR1	34	48	1	1	0.854	aagaCACGTtccaa
OsSK21	P$GBP_Q6	GBP	35	47	1	1	0.96	agaCACGTtcca
OsSK21	P$ABI5_01	ABI5	35	45	1	1	0.957	agaCACGTtc
OsSK21	P$ABF4_01	ABF4	35	47	1	1	0.906	agaCACGTtcca
OsSK21	P$EMBP1_Q2	EMBP1	36	46	1	1	0.906	gaCACGTtcc
OsSK21	P$CPRF3_Q2	CPRF3	36	46	1	1	0.918	gaCACGTtcc
OsSK21	P$CPRF2_Q2	CPRF2	36	46	1	1	0.928	gaCACGTtcc
OsSK21	P$O2_02	O2	36	46	1	1	0.923	gaCACGTtcc
OsSK21	P$TGA1B_Q2	TGA1B	36	46	1	1	0.927	gaCACGTtcc
OsSK21	P$TGA1A_Q2	TGA1A	36	46	1	1	0.964	gaCACGTtcc
OsSK21	P$CG1_Q6	CG1	36	47	1	1	0.855	gaCACGTtcca
OsSK21	P$CPRF3_01	CPRF3	36	46	1	1	0.92	gaCACGTtcc
OsSK21	P$CPRF2_01	CPRF2	36	46	1	1	0.928	gaCACGTtcc
OsSK21	P$TGA1B_01	TGA1B	36	46	1	1	0.929	gaCACGTtcc
OsSK21	P$BEE2_01	BEE2	36	46	1	1	0.92	gaCACGTtcc
OsSK21	P$BIM2_01	BIM2	36	46	1	1	0.855	gaCACGTtcc
OsSK21	P$BIM3_01	BIM3	36	46	1	1	0.888	gaCACGTtcc
OsSK21	P$PHYPA143875_02	HYPA143875	36	46	1	1	0.887	gaCACGTtcc
OsSK21	P$SPT_01	SPT	36	45	1	1	0.914	gaCACGTtc
OsSK21	P$GBF1F_Q2	GBF1F	36	47	1	1	0.899	gaCACGTtcca
OsSK21	P$RITA1_01	RITA1	37	44	1	1	0.953	aCACGTt
OsSK21	P$MYC3_01	MYC3	37	45	1	1	0.856	aCACGTtc
OsSK21	P$OCSBF1_01	OCSBF1	38	43	1	1	1	CACGT
OsSK21	P$MYBAS1_01	MYBAS1	42	53	1	1	0.977	ttCCAACgttt
OsSK21	P$AT5G54070_01	AT5G54070	44	50	1	1	1	cCAACG
OsSK21	P$SBF1_01	SBF1	45	59	1	1	0.885	caacgtTTAAAaag
OsSK21	P$PBF_01	BF	51	62	1	1	0.965	ttaAAAAGatc
OsSK21	P$DOF_Q2	DOF	51	62	1	1	0.975	ttaAAAAGatc
OsSK21	P$CDF2_01	CDF2	52	62	1	1	0.973	taAAAAGatc
OsSK21	P$CDF3_01	CDF3	53	62	1	1	0.974	aAAAAGatc
OsSK21	P$GATA9_01	GATA9	54	65	1	1	0.98	aaaAGATCttt
OsSK21	P$AGP1_01	AGP1	55	65	1	1	0.938	aaAGATCttt
OsSK21	P$ARR2_01	ARR2	55	65	1	1	0.899	aaagATCTTt
OsSK21	P$GATA10_01	GATA10	56	64	1	1	0.912	aAGATCtt
OsSK21	P$GATA11_01	GATA11	56	64	1	1	0.949	aaGATCTt
OsSK21	P$GATA8_01	GATA8	56	65	1	1	0.987	aaGATCTtt
OsSK21	P$ARR10_01	ARR10	57	64	1	1	0.956	AGATCtt
OsSK21	P$SBF1_01	SBF1	57	71	1	1	0.855	agatctTTAAAtat
OsSK21	P$AGL27_01	AGL27	92	106	1	1	0.853	gTTTCTcttttttt
OsSK21	P$SQUA_01	SQUA	98	108	1	1	0.886	cttTTTTTcg
OsSK21	P$PHYPA64121_06	HYPA64121	102	115	1	1	0.874	tttTCGGTcgttc
OsSK21	P$PHYPA64121_06	HYPA64121	149	162	1	1	0.9	attTCGGTaccgc
OsSK21	P$MYB89_01	MYB89	154	165	1	1	0.912	ggTACCGcata
OsSK21	P$SED_Q2	SED	166	176	1	1	0.895	cacaCCTTTg
OsSK21	P$PBF_Q2_01	BF	170	176	1	1	0.988	CCTTTg
OsSK21	P$AT4G01720_01	AT4G01720	176	185	1	1	0.913	gGTAAAatc
OsSK21	P$ATHB7_01	ATHB7	179	189	1	1	0.914	aaAATCAtgg
OsSK21	P$HAT1_01	HAT1	179	189	1	1	0.975	aaAATCAtgg
OsSK21	P$AT3G51080_01	AT3G51080	187	194	1	1	0.89	GGAAAac
OsSK21	P$C1_Q2	C1	189	200	1	1	0.933	aaAACTAcaga
OsSK21	P$ATHSFA1D_01	ATHSFA1D	192	198	1	1	1	aCTACA
OsSK21	P$ATHB6_01	ATHB6	197	206	1	1	0.902	agAATAAag
OsSK21	P$DOF1_01	DOF1	198	209	1	1	0.976	gaaTAAAGacg
OsSK21	P$ARR18_01	ARR18	214	227	1	1	0.894	tataAGATAaaga
OsSK21	P$DOF1_01	DOF1	218	229	1	1	0.986	agaTAAAGaaa
OsSK21	P$BPC1_Q2	BPC1	224	230	1	1	0.997	AGAAAa
OsSK21	P$AP1_01	AP1	224	237	1	1	0.858	agAAAAAaagaca
OsSK21	P$PBF_01	BF	226	237	1	1	0.979	aaaAAAAGaca

OsSK21	P$DOF_Q2	DOF	226	237	1	1	0.984	aaaAAAAGaca
OsSK21	P$CDF2_01	CDF2	227	237	1	1	0.971	aaAAAAGaca
OsSK21	P$CDF3_01	CDF3	228	237	1	1	0.971	aAAAAGaca
OsSK21	P$GT1_Q6_02	GT1	253	265	1	1	0.871	atgaagTTAACt
OsSK21	P$SBF1_01	SBF1	286	300	1	1	0.9	actctgTTAAAaat
OsSK21	P$HMG1_01	HMG1	300	309	1	1	0.912	GTTGTaata
OsSK21	P$ATHB6_01	ATHB6	303	312	1	1	0.984	gtAATAAtt
OsSK21	P$ATHB5_04	ATHB5	303	314	1	1	0.974	gtAATAAtttt
OsSK21	P$ATHB1_03	ATHB1	303	314	1	1	0.975	gtAATAAtttt
OsSK21	P$ATHB16_01	ATHB16	304	312	1	1	1	tAATAAtt
OsSK21	P$ATHB6_01	ATHB6	326	335	1	1	0.917	ctAATAAgt
OsSK21	P$AT4G00870_01	AT4G00870	348	362	1	1	0.883	taCCTCGtatttta
OsSK21	P$SBF1_01	SBF1	353	367	1	1	0.853	cgtattTTAAAaat
OsSK21	P$AT2G02070_01	AT2G02070	379	388	1	1	0.857	atgtGTCGA
OsSK21	P$ARF8_01	ARF8	380	389	1	1	0.952	tgTGTCGat
OsSK21	P$GAMYB_Q2	GAMYB	386	399	1	1	0.867	gatatACAACtgt
OsSK21	P$LEC2_01	LEC2	403	414	1	1	0.993	atCATGCactt
OsSK21	P$FUS3_Q2	FUS3	404	415	1	1	0.863	tCATGCactta
OsSK21	P$SBF1_01	SBF1	406	420	1	1	0.899	atgcacTTAATatt
OsSK21	P$EDT1_01	EDT1	409	419	1	1	0.853	cacTTAATat
OsSK21	P$AT4G00870_01	AT4G00870	420	434	1	1	0.859	ttCCTCGtgtaata
OsSK21	P$ATHB6_01	ATHB6	428	437	1	1	0.906	gtAATAAca
OsSK21	P$ATMYB15_Q2	ATMYB15	432	438	1	1	1	TAACAa
OsSK21	P$CBF3_02	CBF3	440	454	1	1	0.961	agtcaCCGACtgtc
OsSK21	P$CBF1_04	CBF1	441	453	1	1	0.941	gtcaCCGACtgt
OsSK21	P$AT1G77200_03	AT1G77200	442	456	1	1	0.892	tcaCCGACtgtcac
OsSK21	P$ARF1_01	ARF1	444	452	1	1	0.887	aCCGACtg
OsSK21	P$ARF5_01	ARF5	444	452	1	1	0.891	aCCGACtg
OsSK21	P$DREB1B_01	DREB1B	445	450	1	1	1	CCGAC
OsSK21	P$HSFA2_01	HSFA2	455	461	1	1	1	CCAAAa
OsSK21	P$GAMYB_01	GAMYB	472	480	1	1	0.985	CAACCggc
OsSK21	P$ATHB6_01	ATHB6	486	495	1	1	0.905	tgAATAAga
OsSK21	P$PEND_01	END	490	498	1	1	0.892	tAAGAAaa
OsSK21	P$BPC1_Q2	BPC1	492	498	1	1	0.997	AGAAAa
OsSK21	P$HAT1_01	HAT1	498	508	1	1	0.851	aaAATCAcct
OsSK21	P$TEIL_01	TEIL	519	527	1	1	0.925	ATGTAttt
OsSK21	P$LEC2_01	LEC2	529	540	1	1	0.938	atCATGCtgaa
OsSK21	P$ATHB6_01	ATHB6	537	546	1	1	0.905	gaAATAAaa
OsSK21	P$PBF_01	BF	540	551	1	1	0.97	ataAAAAGtag
OsSK21	P$DOF_Q2	DOF	540	551	1	1	0.999	ataAAAAGtag
OsSK21	P$CDF2_01	CDF2	541	551	1	1	0.996	taAAAAGtag
OsSK21	P$CDF3_01	CDF3	542	551	1	1	0.99	aAAAAGtag
OsSK21	P$ANTL_01	ANTL	549	559	1	1	0.936	aGTTACtact
OsSK21	P$AGL4_01	AGL4	556	568	1	1	0.857	acTAAATtgaat
OsSK21	P$AT5G04240_01	AT5G04240	572	578	1	1	0.939	aGGCAC
OsSK21	P$JERF1_01	JERF1	584	594	1	1	0.873	aTGCCGaata
OsSK21	P$CBF1_03	CBF1	584	594	1	1	0.859	aTGCCGaata
OsSK21	P$AT2G41690_01	AT2G41690	587	593	1	1	0.974	CCGAAt
OsSK21	P$BPC1_Q2	BPC1	611	617	1	1	0.99	AGAAAt
OsSK21	P$MYB80_01	MYB80	628	639	1	1	0.861	cgGAATAtgtt
OsSK21	P$PHYPA64121_06	HYPA64121	639	652	1	1	0.9	attTCGGTaactt
OsSK21	P$GT1_Q6_01	GT1	650	662	1	1	0.881	TTTGTgttaata
OsSK21	P$SBF1_01	SBF1	650	664	1	1	0.929	tttgtgTTAATaaa
OsSK21	P$ATHB6_01	ATHB6	656	665	1	1	0.911	ttAATAAag
OsSK21	P$DOF1_01	DOF1	657	668	1	1	0.979	taaTAAAGaca
OsSK21	P$GT1_Q6_02	GT1	669	681	1	1	0.854	tactacTTAACc
OsSK21	P$AT1G66560_01	AT1G66560	672	682	1	1	0.953	tacTTAACca
OsSK21	P$WRKY21_01	WRKY21	673	682	1	1	0.956	acTTAACca
OsSK21	P$AT2G34830_01	AT2G34830	673	682	1	1	0.958	acTTAACca
OsSK21	P$AT1G18860_01	AT1G18860	673	682	1	1	0.973	acTTAACca
OsSK21	P$AT1G64000_01	AT1G64000	673	682	1	1	0.94	acTTAACca
OsSK21	P$AT4G22070_01	AT4G22070	673	682	1	1	0.952	acTTAACca
OsSK21	P$WRKY6_01	WRKY6	673	682	1	1	0.95	acTTAACca
OsSK21	P$AT1G66600_01	AT1G66600	673	682	1	1	0.951	acTTAACca
OsSK21	P$AT1G68150_01	AT1G68150	673	682	1	1	0.967	acTTAACca
OsSK21	P$AT5G41570_01	AT5G41570	673	682	1	1	0.939	acTTAACca
OsSK21	P$AT1G69810_01	AT1G69810	673	682	1	1	0.969	acTTAACca
OsSK21	P$AT5G15130_01	AT5G15130	673	682	1	1	0.973	acTTAACca
OsSK21	P$WRKY46_01	WRKY46	673	682	1	1	0.881	acTTAACca
OsSK21	P$AT1G30650_01	AT1G30650	673	682	1	1	0.958	acTTAACca
OsSK21	P$AT2G24570_01	AT2G24570	673	682	1	1	0.955	acTTAACca
OsSK21	P$AT4G23550_01	AT4G23550	673	682	1	1	0.958	acTTAACca
OsSK21	P$WRKY7_01	WRKY7	673	682	1	1	0.959	acTTAACca
OsSK21	P$WRKY25_01	WRKY25	673	682	1	1	0.971	actTAACCa
OsSK21	P$WRKY33_01	WRKY33	673	682	1	1	0.885	actTAACCa
OsSK21	P$AT1G29860_01	AT1G29860	673	682	1	1	0.922	actTAACCa
OsSK21	P$AT3G62340_01	AT3G62340	673	682	1	1	0.907	actTAACCa
OsSK21	P$AT1G69310_01	AT1G69310	673	682	1	1	0.936	actTAACCa

OsSK21	P$WRKY26_01	WRKY26	673	682	1	1	0.889	actTAACCa
OsSK21	P$GT1_01	GT1	674	682	1	1	0.968	ctTAACCa
OsSK21	P$AT4G36620_01	AT4G36620	674	682	1	1	0.966	cttAACCA
OsSK21	P$ARR18_01	ARR18	684	697	1	1	0.912	attgAGATAtaaa
OsSK21	P$SPF1_Q2	SPF1	716	726	1	1	0.924	tcATAGTatc
OsSK21	P$AT5G26170_01	AT5G26170	741	750	1	1	0.931	tcTCAACcc
OsSK21	P$GAMYB_01	GAMYB	744	752	1	1	0.893	CAACCcca
OsSK21	P$SQUA_01	SQUA	750	760	1	1	0.867	caaTTTTTtt
OsSK21	P$GT1_Q6_01	GT1	754	766	1	1	0.909	TTTTTtatcatg
OsSK21	P$GATA15_01	GATA15	762	771	1	1	1	caTGATCat
OsSK21	P$PHV_02	HV	763	778	1	1	0.929	atgATCATtaatatt
OsSK21	P$ATHB4_02	ATHB4	764	774	1	1	0.877	tgATCATtaa
OsSK21	P$SBF1_01	SBF1	764	778	1	1	0.925	tgatcaTTAATatt
OsSK21	P$EDT1_01	EDT1	767	777	1	1	0.868	tcaTTAATat
OsSK21	P$ATHB6_01	ATHB6	790	799	1	1	0.909	taAATAAat
OsSK21	P$ATHB6_01	ATHB6	794	803	1	1	0.909	taAATAAat
OsSK21	P$PDF2_01	DF2	794	805	1	1	0.867	taaaTAAATtc
OsSK21	P$TGA1A_Q2_01	TGA1A	813	835	1	1	0.912	cacATGACttagtttatatttc
OsSK21	P$HSFA4A_01	HSFA4A	875	881	1	1	0.914	tCTATT
OsSK21	P$ARR1_01	ARR1	887	897	1	1	0.948	agtGAATCat
OsSK21	P$ATHB7_01	ATHB7	889	899	1	1	0.913	tgAATCAtct
OsSK21	P$HAT1_01	HAT1	889	899	1	1	0.969	tgAATCAtct
OsSK21	P$ARR2_01	ARR2	891	901	1	1	0.86	aatcATCTTg
OsSK21	P$GATA9_01	GATA9	917	928	1	1	0.883	gagAGATCaaa
OsSK21	P$AGP1_01	AGP1	918	928	1	1	0.905	agAGATCaaa
OsSK21	P$ARR10_01	ARR10	920	927	1	1	0.869	AGATCaa
OsSK21	P$ATHB7_01	ATHB7	976	986	1	1	0.914	aaAATCAtag
OsSK21	P$HAT1_01	HAT1	976	986	1	1	0.97	aaAATCAtag
OsSK21	P$ARR18_01	ARR18	980	993	1	1	0.94	tcatAGATAagta
OsSK21	P$SBF1_01	SBF1	999	1013	1	1	0.894	gtagccTTAAAaaa
OsSK21	P$PBF_01	BF	1006	1017	1	1	0.981	taaAAAAGcaa
OsSK21	P$DOF_Q2	DOF	1006	1017	1	1	0.992	taaAAAAGcaa
OsSK21	P$DOF2_01	DOF2	1006	1017	1	1	0.993	taaaAAAGCaa
OsSK21	P$DOF3_01	DOF3	1006	1017	1	1	0.991	taaaAAAGCaa
OsSK21	P$CDF2_01	CDF2	1007	1017	1	1	0.989	aaAAAAGcaa
OsSK21	P$CDF3_01	CDF3	1008	1017	1	1	0.984	aAAAAGcaa
OsSK21	P$C1_Q2	C1	1013	1024	1	1	0.931	gcAACTAatac
OsSK21	P$CBNAC_01	CBNAC	1040	1046	1	1	0.973	cTGCTT
OsSK21	P$CBNAC_02	CBNAC	1040	1056	1	1	0.901	cTGCTTgttaattaaa
OsSK21	P$SBF1_01	SBF1	1041	1055	1	1	0.908	tgcttgTTAATtaa
OsSK21	P$SBF1_01	SBF1	1045	1059	1	1	0.869	tgttaaTTAAAaca
OsSK21	P$GAMYB_01	GAMYB	1067	1075	1	1	0.925	CAACCgtg
OsSK21	P$LEC2_01	LEC2	1106	1117	1	1	0.941	ttCATGCtaaa
OsSK21	P$DOF1_01	DOF1	1110	1121	1	1	0.976	tgcTAAAGact
OsSK21	P$ARR2_01	ARR2	1123	1133	1	1	0.975	ccgcATCTTt
OsSK21	P$EDT1_01	EDT1	1128	1138	1	1	0.853	tctTTAATct
OsSK21	P$ARR2_01	ARR2	1130	1140	1	1	0.866	tttaATCTTc
OsSK21	P$PEND_02	END	1143	1153	1	1	0.854	gtTTCTTttg
OsSK21	P$SEP3_01	wrz-03	1143	1154	1	1	0.862	gtttcTTTTGg
OsSK21	P$MYB1L_01	MYB1L	1152	1162	1	1	0.968	ggCCCTAgtt
OsSK21	P$TRB2_01	TRB2	1152	1160	1	1	0.985	ggCCCTAg
OsSK21	P$GT1_Q6_01	GT1	1160	1172	1	1	0.918	TTTTTtttaatc
OsSK21	P$SBF1_01	SBF1	1160	1174	1	1	0.862	ttttttTTAATcta
OsSK21	P$ARR18_01	ARR18	1169	1182	1	1	0.954	atctAGATAcaac
OsSK21	P$GAMYB_Q2	GAMYB	1172	1185	1	1	0.917	tagatACAACaaa
OsSK21	P$RAV1_01	RAV1	1175	1187	1	1	0.961	ataCAACAaaaa
OsSK21	P$GAMYB_Q2	GAMYB	1185	1198	1	1	0.911	aaaaaACAACaat
OsSK21	P$RAV1_01	RAV1	1188	1200	1	1	0.934	aaaCAACAatta
OsSK21	P$ATHB7_01	ATHB7	1213	1223	1	1	0.922	acAATCAcat
OsSK21	P$HAT1_01	HAT1	1213	1223	1	1	0.868	acAATCAcat
OsSK21	P$SBF1_01	SBF1	1218	1232	1	1	0.861	cacattTTAATaaa
OsSK21	P$ATHB6_01	ATHB6	1224	1233	1	1	0.91	ttAATAAaa
OsSK21	P$GAMYB_Q2	GAMYB	1256	1269	1	1	0.871	aatatACAACtta
OsSK21	P$MADSB_Q2	MADSB	1286	1301	1	1	0.874	ttctAAAAAtgtacc
OsSK21	P$ATSPL8_01	ATSPL8	1290	1306	1	1	0.959	aaaaaTGTACcacatc
OsSK21	P$TEIL_01	TEIL	1294	1302	1	1	0.881	ATGTAcca
OsSK21	P$PEND_02	END	1308	1318	1	1	0.863	taTTCTTctt
OsSK21	P$WRKY11_Q2	WRKY11	1315	1323	1	1	0.95	cTTGACta
OsSK21	P$GAMYB_01	GAMYB	1327	1335	1	1	0.883	CAACCcta
OsSK21	P$MYB1L_01	MYB1L	1328	1338	1	1	0.999	aaCCCTAaca
OsSK21	P$TRB2_01	TRB2	1328	1336	1	1	0.973	aaCCCTAa
OsSK21	P$MYBAS1_01	MYBAS1	1330	1341	1	1	0.985	ccCTAACattt
OsSK21	P$GAMYB_Q2	GAMYB	1345	1358	1	1	0.878	ctgatACAACctt
OsSK21	P$GAMYB_01	GAMYB	1351	1359	1	1	0.871	CAACCtta
OsSK21	P$PEND_01	END	1357	1365	1	1	0.892	tAAGAAag
OsSK21	P$DOF2_01	DOF2	1357	1368	1	1	0.984	taagAAAGCaa
OsSK21	P$DOF3_01	DOF3	1357	1368	1	1	0.987	taagAAAGCaa
OsSK21	P$BPC1_Q2	BPC1	1359	1365	1	1	1	AGAAAg

OsSK21	P$RAV1_01	RAV1	1362	1374	1	1	0.957	aagCAACAatca
OsSK21	P$ATHB7_01	ATHB7	1367	1377	1	1	0.922	acAATCAcga
OsSK21	P$HAT1_01	HAT1	1367	1377	1	1	0.873	acAATCAcga
OsSK21	P$AT4G36620_01	AT4G36620	1386	1394	1	1	0.915	taaAACCA
OsSK21	P$SBF1_01	SBF1	1392	1406	1	1	0.907	cacatgTTAATaaa
OsSK21	P$ATHB6_01	ATHB6	1398	1407	1	1	0.91	ttAATAAaa
OsSK21	P$NAC043_01	NAC043	1411	1421	1	1	0.894	actACGTAac
OsSK21	P$NAC025_01	NAC025	1413	1421	1	1	1	tACGTAac
OsSK21	P$NAC6_01	NAC6	1414	1420	1	1	1	aCGTAA
OsSK21	P$WRKY25_01	WRKY25	1414	1423	1	1	0.906	acgTAACCg
OsSK21	P$AT1G69310_01	AT1G69310	1414	1423	1	1	0.873	acgTAACCg
OsSK21	P$C1_Q2	C1	1427	1438	1	1	0.943	tgAACTAtaca
OsSK21	P$PDF2_01	DF2	1447	1458	1	1	0.865	tccaTAAATtt
OsSK21	P$LEC2_01	LEC2	1456	1467	1	1	0.941	ttCATGCtaaa
OsSK21	P$DOF1_01	DOF1	1460	1471	1	1	0.976	tgcTAAAGacg
OsSK21	P$ATSPL3_01	ATSPL3	1464	1480	1	1	0.987	aaagaCGTACcacatt
OsSK21	P$TGA1B_01	TGA1B	1465	1475	1	1	0.891	aaGACGTacc
OsSK21	P$HSFA4A_01	HSFA4A	1480	1486	1	1	0.914	tCTATT
OsSK21	P$HSFA4A_01	HSFA4A	1493	1499	1	1	1	aCTATT
OsSK21	P$MYBAS1_01	MYBAS1	1504	1515	1	1	0.986	ccCCAACtttt
OsSK21	P$GAMYB_Q2	GAMYB	1519	1532	1	1	0.878	ctgatACAACctt
OsSK21	P$GAMYB_01	GAMYB	1525	1533	1	1	0.871	CAACCtta
OsSK21	P$PEND_01	END	1531	1539	1	1	0.892	tAAGAAag
OsSK21	P$BPC1_Q2	BPC1	1533	1539	1	1	1	AGAAAg
OsSK21	P$BPC1_Q2	BPC1	1537	1543	1	1	0.99	AGAAAc
OsSK21	P$ATHB7_01	ATHB7	1541	1551	1	1	0.99	acAATCAtga
OsSK21	P$HAT1_01	HAT1	1541	1551	1	1	0.991	acAATCAtga
OsSK21	P$ARR18_01	ARR18	1553	1566	1	1	0.895	aaacAGATAaaac
OsSK21	P$AT4G36620_01	AT4G36620	1560	1568	1	1	0.915	taaAACCA
OsSK21	P$SBF1_01	SBF1	1566	1580	1	1	0.907	cacatgTTAATata
OsSK21	P$MYB3R5_01	MYB3R5	1586	1601	1	1	0.972	actatgtaaCCGTTg
OsSK21	P$MYB3R1_01	MYB3R1	1587	1602	1	1	0.981	ctatgtaaCCGTTgt
OsSK21	P$MYB3R4_01	MYB3R4	1587	1602	1	1	0.99	ctatgtaaCCGTTgt
OsSK21	P$MYBAS1_01	MYBAS1	1609	1620	1	1	0.938	taCCAACtaag
OsSK21	P$C1_Q2	C1	1611	1622	1	1	0.957	ccAACTAagca
OsSK21	P$LEC2_01	LEC2	1631	1642	1	1	0.941	ttCATGCtaaa
OsSK21	P$DOF1_01	DOF1	1635	1646	1	1	0.976	tgcTAAAGatg
OsSK21	P$ATSPL8_01	ATSPL8	1639	1655	1	1	0.949	aaagaTGTACtacatc
OsSK21	P$ATHSFA1D_01	ATHSFA1D	1647	1653	1	1	1	aCTACA
OsSK21	P$HSFA4A_01	HSFA4A	1655	1661	1	1	0.914	tCTATT
OsSK21	P$PEND_02	END	1657	1667	1	1	0.863	taTTCTTctt
OsSK21	P$MYBAS1_01	MYBAS1	1678	1689	1	1	0.983	gcCCAACtttt
OsSK21	P$ARR2_01	ARR2	1697	1707	1	1	0.884	tacaATCTTa
OsSK21	P$PEND_01	END	1705	1713	1	1	0.892	tAAGAAag
OsSK21	P$DOF2_01	DOF2	1705	1716	1	1	0.984	taagAAAGCaa
OsSK21	P$DOF3_01	DOF3	1705	1716	1	1	0.987	taagAAAGCaa
OsSK21	P$BPC1_Q2	BPC1	1707	1713	1	1	1	AGAAAg
OsSK21	P$RAV1_01	RAV1	1710	1722	1	1	0.963	aagCAACAatta
OsSK21	P$PIL5_01	IL5	1733	1747	1	1	0.954	acaaaatcACGTGt
OsSK21	P$PIF3_01	IF3	1734	1752	1	1	0.895	caaaatCACGTgttaaca
OsSK21	P$HAT1_01	HAT1	1735	1745	1	1	0.856	aaAATCAcgt
OsSK21	P$ABF2_01	ABF2	1735	1748	1	1	0.938	aaaatCACGTgtt
OsSK21	P$ABF4_Q2	ABF4	1735	1749	1	1	0.92	aaaatcACGTGtta
OsSK21	P$BZR1_02	BZR1	1736	1750	1	1	0.989	aaatCACGTgttaa
OsSK21	P$HBI1_01	HBI1	1736	1748	1	1	0.939	aaatCACGTgtt
OsSK21	P$ABZ1_01	ABZ1	1736	1750	1	1	0.904	aaatcACGTGttaa
OsSK21	P$GBP_Q6	GBP	1737	1749	1	1	0.932	aatCACGTgtta
OsSK21	P$PIF3_03	IF3	1737	1747	1	1	0.932	aatCACGTgt
OsSK21	P$ABI5_01	ABI5	1737	1747	1	1	0.933	aatCACGTgt
OsSK21	P$ABF4_01	ABF4	1737	1749	1	1	0.949	aatCACGTgtta
OsSK21	P$BZR1_03	BZR1	1737	1757	1	1	0.859	aatcACGTGttaacaaaaat
OsSK21	P$EMBP1_Q2	EMBP1	1738	1748	1	1	0.873	atCACGTgtt
OsSK21	P$CPRF_Q2	CPRF	1738	1748	1	1	0.901	atCACGTgtt
OsSK21	P$CPRF3_Q2	CPRF3	1738	1748	1	1	0.98	atCACGTgtt
OsSK21	P$CPRF2_Q2	CPRF2	1738	1748	1	1	0.995	atCACGTgtt
OsSK21	P$O2_02	O2	1738	1748	1	1	0.958	atCACGTgtt
OsSK21	P$TGA1B_Q2	TGA1B	1738	1748	1	1	0.908	atCACGTgtt
OsSK21	P$TGA1A_Q2	TGA1A	1738	1748	1	1	0.98	atCACGTgtt
OsSK21	P$CPRF1_01	CPRF1	1738	1748	1	1	0.912	atCACGTgtt
OsSK21	P$CPRF3_01	CPRF3	1738	1748	1	1	0.987	atCACGTgtt
OsSK21	P$CPRF2_01	CPRF2	1738	1748	1	1	0.997	atCACGTgtt
OsSK21	P$TGA1B_01	TGA1B	1738	1748	1	1	0.917	atCACGTgtt
OsSK21	P$BES1_01	BES1	1738	1749	1	1	0.954	atCACGTgtta
OsSK21	P$BEE2_01	BEE2	1738	1748	1	1	0.999	atCACGTgtt
OsSK21	P$BIM2_01	BIM2	1738	1748	1	1	0.996	atCACGTgtt
OsSK21	P$BIM3_01	BIM3	1738	1748	1	1	0.992	atCACGTgtt
OsSK21	P$PHYPA143875_02	HYPA143875	1738	1748	1	1	0.996	atCACGTgtt
OsSK21	P$PHYPA72483_07	HYPA72483	1738	1748	1	1	0.999	atCACGTgtt

OsSK21	P$SPT_01	SPT	1738	1747	1	1	0.974	atCACGTgt
OsSK21	P$GBF1F_Q2	GBF1F	1738	1749	1	1	0.857	atCACGTgtta
OsSK21	P$HBP1A_Q2	HBP1A	1738	1748	1	1	0.902	atcACGTGtt
OsSK21	P$TAF1_Q2	TAF1	1738	1748	1	1	0.957	atcACGTGtt
OsSK21	P$EMBP1_02	EMBP1	1738	1748	1	1	0.905	atcACGTGtt
OsSK21	P$TAF1_01	TAF1	1738	1748	1	1	0.974	atcACGTGtt
OsSK21	P$PIF1_01	IF1	1738	1748	1	1	0.977	atcACGTGtt
OsSK21	P$RITA1_01	RITA1	1739	1746	1	1	1	tCACGTg
OsSK21	P$BHLH66_01	BHLH66	1739	1747	1	1	0.93	tCACGTgt
OsSK21	P$PIF5_01	IF5	1739	1747	1	1	0.975	tCACGTgt
OsSK21	P$MYC2_01	MYC2	1739	1747	1	1	0.95	tCACGTgt
OsSK21	P$MYC3_01	MYC3	1739	1747	1	1	0.989	tCACGTgt
OsSK21	P$BHLH34_01	BHLH34	1739	1747	1	1	0.967	tCACGTgt
OsSK21	P$PHYPA48267_08	HYPA48267	1739	1747	1	1	0.975	tCACGTgt
OsSK21	P$OJ1058_01	OJ1058	1739	1747	1	1	0.946	tCACGTgt
OsSK21	P$UNE10_01	UNE10	1739	1747	1	1	0.982	tCACGTgt
OsSK21	P$BHLH3_01	BHLH3	1739	1747	1	1	0.949	tCACGTgt
OsSK21	P$GBF1_01	GBF1	1739	1747	1	1	0.994	tcACGTGt
OsSK21	P$MYC4_01	MYC4	1739	1747	1	1	0.955	tcACGTGt
OsSK21	P$BIM1_02	BIM1	1739	1749	1	1	0.996	tcACGTGtta
OsSK21	P$BHLH13_01	BHLH13	1739	1747	1	1	0.942	tcACGTGt
OsSK21	P$ABF4_02	ABF4	1739	1749	1	1	0.991	tcACGTGtta
OsSK21	P$OCSBF1_01	OCSBF1	1740	1745	1	1	1	CACGT
OsSK21	P$PIF4_01	IF4	1740	1748	1	1	0.947	CACGTgtt
OsSK21	P$GT1_Q6_02	GT1	1740	1752	1	1	0.941	cacgtgTTAACa
OsSK21	P$ABI5_Q2	ABI5	1741	1747	1	1	0.979	ACGTGt
OsSK21	P$WRKY48_01	WRKY48	1744	1753	1	1	0.872	tgttAACAA
OsSK21	P$ATMYB15_Q2	ATMYB15	1747	1753	1	1	1	TAACAa
OsSK21	P$NAC043_01	NAC043	1760	1770	1	1	0.89	tctACGTAac
OsSK21	P$NAC025_01	NAC025	1762	1770	1	1	1	tACGTAac
OsSK21	P$NAC6_01	NAC6	1763	1769	1	1	1	aCGTAA
OsSK21	P$WRKY25_01	WRKY25	1763	1772	1	1	0.906	acgTAACCg
OsSK21	P$AT1G69310_01	AT1G69310	1763	1772	1	1	0.873	acgTAACCg
OsSK21	P$TGA2_Q2	TGA2	1769	1779	1	1	0.87	cCGTCAttga
OsSK21	P$ATHB7_01	ATHB7	1802	1812	1	1	0.913	aaAATCAtgc
OsSK21	P$HAT1_01	HAT1	1802	1812	1	1	0.974	aaAATCAtgc
OsSK21	P$LEC2_01	LEC2	1805	1816	1	1	0.94	atCATGCtaaa
OsSK21	P$DOF1_01	DOF1	1809	1820	1	1	0.976	tgcTAAAGacg
OsSK21	P$ATSPL3_01	ATSPL3	1813	1829	1	1	0.979	aaagaCGTACcgtata
OsSK21	P$TGA1B_01	TGA1B	1814	1824	1	1	0.891	aaGACGTacc
OsSK21	P$MYB89_01	MYB89	1818	1829	1	1	0.908	cgTACCGtata
OsSK21	P$SPL14_01	SPL14	1822	1829	1	1	0.851	CCGTAta
OsSK21	P$MYBAS1_01	MYBAS1	1843	1854	1	1	0.98	tcCCAACcttt
OsSK21	P$SED_Q2	SED	1845	1855	1	1	0.965	ccaaCCTTTt
OsSK21	P$PBF_Q2_01	BF	1849	1855	1	1	1	CCTTTt
OsSK21	P$NAC6_01	NAC6	1856	1862	1	1	0.854	tCGTAA
OsSK21	P$GAMYB_Q2	GAMYB	1858	1871	1	1	0.872	gtaatACAACcta
OsSK21	P$GAMYB_01	GAMYB	1864	1872	1	1	0.881	CAACCtaa
OsSK21	P$DOF1_01	DOF1	1866	1877	1	1	0.991	accTAAAGaaa
OsSK21	P$DOF2_01	DOF2	1870	1881	1	1	0.986	aaagAAAGCaa
OsSK21	P$DOF3_01	DOF3	1870	1881	1	1	0.99	aaagAAAGCaa
OsSK21	P$BPC1_Q2	BPC1	1872	1878	1	1	1	AGAAAg
OsSK21	P$RAV1_01	RAV1	1875	1887	1	1	0.957	aagCAACAatca
OsSK21	P$ATHB7_01	ATHB7	1880	1890	1	1	0.922	acAATCAcga
OsSK21	P$HAT1_01	HAT1	1880	1890	1	1	0.873	acAATCAcga
OsSK21	P$AT4G36620_01	AT4G36620	1907	1915	1	1	0.915	taaAACCA
OsSK21	P$SBF1_01	SBF1	1913	1927	1	1	0.915	catatgTTAATaaa
OsSK21	P$ATHB6_01	ATHB6	1919	1928	1	1	0.91	ttAATAAaa
OsSK21	P$GT1_Q6_02	GT1	1932	1944	1	1	0.857	ttctacTTAACa
OsSK21	P$AT1G66560_01	AT1G66560	1935	1945	1	1	0.909	tacTTAACag
OsSK21	P$WRKY21_01	WRKY21	1936	1945	1	1	0.884	acTTAACag
OsSK21	P$AT2G34830_01	AT2G34830	1936	1945	1	1	0.887	acTTAACag
OsSK21	P$AT1G18860_01	AT1G18860	1936	1945	1	1	0.96	acTTAACag
OsSK21	P$AT1G64000_01	AT1G64000	1936	1945	1	1	0.857	acTTAACag
OsSK21	P$AT4G22070_01	AT4G22070	1936	1945	1	1	0.894	acTTAACag
OsSK21	P$WRKY6_01	WRKY6	1936	1945	1	1	0.89	acTTAACag
OsSK21	P$AT1G66600_01	AT1G66600	1936	1945	1	1	0.909	acTTAACag
OsSK21	P$AT1G68150_01	AT1G68150	1936	1945	1	1	0.929	acTTAACag
OsSK21	P$AT5G41570_01	AT5G41570	1936	1945	1	1	0.856	acTTAACag
OsSK21	P$AT1G69810_01	AT1G69810	1936	1945	1	1	0.934	acTTAACag
OsSK21	P$AT5G15130_01	AT5G15130	1936	1945	1	1	0.96	acTTAACag
OsSK21	P$WRKY46_01	WRKY46	1936	1945	1	1	0.861	acTTAACag
OsSK21	P$AT1G30650_01	AT1G30650	1936	1945	1	1	0.891	acTTAACag
OsSK21	P$AT2G24570_01	AT2G24570	1936	1945	1	1	0.881	acTTAACag
OsSK21	P$AT4G23550_01	AT4G23550	1936	1945	1	1	0.885	acTTAACag
OsSK21	P$WRKY7_01	WRKY7	1936	1945	1	1	0.886	acTTAACag
OsSK21	P$ATMYB15_Q2	ATMYB15	1939	1945	1	1	0.865	TAACAg
OsSK21	P$LEC2_01	LEC2	1978	1989	1	1	0.941	ttCATGCtaaa

OsSK21	P$DOF1_01	DOF1	1982	1993	1	1	0.976	tgcTAAAGatg
OsSK21	P$ATSPL8_01	ATSPL8	1986	2002	1	1	0.944	aaagaTGTACaacatc
OsSK21	P$GAMYB_Q2	GAMYB	1989	2002	1	1	0.854	gatgtACAACatc
OsSK21	P$RAV1_01	RAV1	1992	2004	1	1	0.92	gtaCAACAtctc
OsSK21	P$HSFA4A_01	HSFA4A	2002	2008	1	1	0.914	tCTATT
OsSK21	P$PEND_02	END	2004	2014	1	1	0.863	taTTCTTctt
OsSK21	P$WRKY11_01	WRKY11	2008	2022	1	1	0.862	cttcTTGACttatt
OsSK21	P$WRKY11_Q2	WRKY11	2011	2019	1	1	0.929	cTTGACtt
OsSK21	P$AT3G60580_01	AT3G60580	2036	2043	1	1	0.95	ttATCCC
OsSK21	P$GAMYB_Q2	GAMYB	2041	2054	1	1	0.871	ccgatACAACctt
OsSK21	P$GAMYB_01	GAMYB	2047	2055	1	1	0.871	CAACCtta
OsSK21	P$PEND_01	END	2053	2061	1	1	0.892	tAAGAAag
OsSK21	P$DOF2_01	DOF2	2053	2064	1	1	0.984	taagAAAGCaa
OsSK21	P$DOF3_01	DOF3	2053	2064	1	1	0.987	taagAAAGCaa
OsSK21	P$BPC1_Q2	BPC1	2055	2061	1	1	1	AGAAAg
OsSK21	P$RAV1_01	RAV1	2058	2070	1	1	0.963	aagCAACAatta
OsSK21	P$AT4G12750_01	AT4G12750	2074	2084	1	1	0.943	gaaACCGAta
OsSK21	P$SPL14_02	SPL14	2082	2101	1	1	0.946	taaaaaCCGTAcgatgata
OsSK21	P$ATSPL3_01	ATSPL3	2084	2100	1	1	0.978	aaaacCGTACgatgat
OsSK21	P$SPL11_01	SPL11	2086	2098	1	1	0.914	aaccGTACGatg
OsSK21	P$SPL3_01	SPL3	2087	2095	1	1	0.972	aCCGTAcg
OsSK21	P$SPL1_01	SPL1	2087	2097	1	1	0.897	acCGTACgat
OsSK21	P$SPL14_03	SPL14	2087	2098	1	1	0.967	acCGTACgatg
OsSK21	P$SPL14_01	SPL14	2088	2095	1	1	0.967	CCGTAcg
OsSK21	P$SPL5_01	SPL5	2088	2097	1	1	0.971	ccGTACGat
OsSK21	P$POPTR_01	OPTR	2089	2096	1	1	0.944	cGTACGa
OsSK21	P$SPL12_01	SPL12	2089	2097	1	1	0.982	cGTACGat
OsSK21	P$SPL4_01	SPL4	2089	2098	1	1	0.994	cGTACGatg
OsSK21	P$MRP1_Q2	MRP1	2108	2120	1	1	0.879	ttTCTATataac
OsSK21	P$AT3G01030_01	AT3G01030	2118	2127	1	1	0.89	ACCGCcatt
OsSK21	P$AT3G63350_01	AT3G63350	2119	2125	1	1	0.882	CCGCCa
OsSK21	P$RAV1_01	RAV1	2144	2156	1	1	0.954	catCAACAaaat
OsSK21	P$LEC2_01	LEC2	2155	2166	1	1	0.945	ttCATGCcaaa
OsSK21	P$HSFA2_01	HSFA2	2161	2167	1	1	0.933	CCAAAg
OsSK21	P$ATSPL3_01	ATSPL3	2163	2179	1	1	0.987	aaagaCGTACcacatc
OsSK21	P$TGA1B_01	TGA1B	2164	2174	1	1	0.891	aaGACGTacc
OsSK21	P$TGA2_Q2	TGA2	2184	2194	1	1	0.929	tCGTCAtcca
OsSK21	P$ASR1_01	ASR1	2195	2200	1	1	1	ACCCA
OsSK21	P$ATHB7_01	ATHB7	2210	2220	1	1	0.892	ctAATCAata
OsSK21	P$HAT1_01	HAT1	2210	2220	1	1	0.889	ctAATCAata
OsSK21	P$LIM1_01	LIM1	2220	2232	1	1	0.977	CCACCaaagact
OsSK21	P$HSFA2_01	HSFA2	2223	2229	1	1	0.933	CCAAAg
OsSK21	P$C1_Q2	C1	2237	2248	1	1	0.936	taAACTAagaa
OsSK21	P$PEND_01	END	2242	2250	1	1	0.892	tAAGAAac
OsSK21	P$BPC1_Q2	BPC1	2244	2250	1	1	0.99	AGAAAc
OsSK21	P$LEC2_01	LEC2	2275	2286	1	1	0.994	ctCATGCaaat
OsSK21	P$AT3G60580_01	AT3G60580	2308	2315	1	1	0.852	acATCCC
OsSK21	P$BPC1_Q2	BPC1	2325	2331	1	1	0.997	AGAAAa
OsSK21	P$CCA1_Q5	CCA1	2333	2350	1	1	0.873	cttcaAAAATatcatac
OsSK21	P$ATMYB77_01	ATMYB77	2350	2363	1	1	0.887	tgagcaCAGTTat
OsSK21	P$ATMYB77_01	ATMYB77	2375	2388	1	1	0.887	tcagcaCAGTTat
OsSK21	P$PHYPA64121_06	HYPA64121	2384	2397	1	1	0.889	ttaTCGGTaaaac
OsSK21	P$AT4G36620_01	AT4G36620	2391	2399	1	1	0.915	taaAACCA
OsSK21	P$HSFA2_01	HSFA2	2412	2418	1	1	0.941	CCAAAc
OsSK21	P$DOF2_01	DOF2	2417	2428	1	1	0.981	ctccAAAGCcg
OsSK21	P$DOF3_01	DOF3	2417	2428	1	1	0.974	ctccAAAGCcg
OsSK21	P$HSFA2_01	HSFA2	2419	2425	1	1	0.933	CCAAAg
OsSK21	P$ERF6_02	ERF6	2422	2432	1	1	0.979	aaGCCGGcct
OsSK21	P$ERF105_02	ERF105	2423	2431	1	1	0.929	aGCCGGcc
OsSK21	P$GAMYB_Q2	GAMYB	2456	2469	1	1	0.905	acaccACAACacc
OsSK21	P$RAV1_01	RAV1	2459	2471	1	1	0.909	ccaCAACAcccc
OsSK21	P$WRKY18_02	WRKY18	2497	2507	1	1	0.996	ccgGTCAAaa
OsSK21	P$WRKY21_02	WRKY21	2497	2507	1	1	0.968	ccgGTCAAaa
OsSK21	P$WRKY48_02	WRKY48	2497	2507	1	1	0.998	ccgGTCAAaa
OsSK21	P$WRKY57_01	WRKY57	2497	2507	1	1	0.971	ccgGTCAAaa
OsSK21	P$WRKY60_01	WRKY60	2497	2508	1	1	0.978	ccgGTCAAaag
OsSK21	P$WRKY15_01	WRKY15	2498	2508	1	1	0.985	cgGTCAAaag
OsSK21	P$WRKY2_01	WRKY2	2498	2506	1	1	0.991	cgGTCAAa
OsSK21	P$WRKY25_02	WRKY25	2498	2506	1	1	0.979	cgGTCAAa
OsSK21	P$WRKY40_01	WRKY40	2498	2506	1	1	1	cgGTCAAa
OsSK21	P$WRKY43_02	WRKY43	2498	2508	1	1	0.977	cgGTCAAaag
OsSK21	P$WRKY62_01	WRKY62	2498	2506	1	1	0.91	cgGTCAAa
OsSK21	P$WRKY63_01	WRKY63	2498	2506	1	1	0.991	cgGTCAAa
OsSK21	P$WRKY75_01	WRKY75	2498	2506	1	1	0.975	cgGTCAAa
OsSK21	P$WRKY8_01	WRKY8	2498	2507	1	1	0.992	cgGTCAAaa
OsSK21	P$WRKY23_01	WRKY23	2499	2507	1	1	0.854	gGTCAAaa
OsSK21	P$WRKY30_01	WRKY30	2499	2509	1	1	0.916	gGTCAAagc
OsSK21	P$WRKY18_Q2	WRKY18	2500	2509	1	1	0.954	GTCAAagc

OsSK21	P$PBF_01	BF	2500	2511	1	1	0.973	gtcAAAAGcac
OsSK21	P$DOF_Q2	DOF	2500	2511	1	1	0.935	gtcAAAAGcac
OsSK21	P$DOF2_01	DOF2	2500	2511	1	1	0.988	gtcaAAAGCac
OsSK21	P$DOF3_01	DOF3	2500	2511	1	1	0.987	gtcaAAAGCac
OsSK21	P$CDF2_01	CDF2	2501	2511	1	1	0.957	tcAAAAGcac
OsSK21	P$CDF3_01	CDF3	2502	2511	1	1	0.978	cAAAAGcac
OsSK21	P$LIM1_01	LIM1	2512	2524	1	1	0.965	CCACCaccatcc
OsSK21	P$LIM1_01	LIM1	2515	2527	1	1	0.978	CCACCatccccc
OsSK21	P$AT3G60580_01	AT3G60580	2518	2525	1	1	0.873	ccATCCC
OsSK21	P$DOF2_01	DOF2	2526	2537	1	1	0.983	ccgcAAAGCca
OsSK21	P$DOF3_01	DOF3	2526	2537	1	1	0.982	ccgcAAAGCca
OsSK21	P$MYBAS1_01	MYBAS1	2539	2550	1	1	0.983	caCCAACgctc
OsSK21	P$AT5G54070_01	AT5G54070	2541	2547	1	1	1	cCAACG
OsSK21	P$CDC5_01	CDC5	2545	2556	1	1	0.856	cgcTCAGCagc
OsSK21	P$ABI4_03	ABI4	2558	2568	1	1	0.894	caGCCGCctc
OsSK21	P$WRAF1_01	WRAF1	2558	2568	1	1	0.926	caGCCGCctc
OsSK21	P$WRAF2_01	WRAF2	2558	2568	1	1	0.879	caGCCGCctc
OsSK21	P$PTI5_01	TI5	2558	2568	1	1	0.911	caGCCGCctc
OsSK21	P$DREBI5_01	DREBI5	2558	2568	1	1	0.887	caGCCGCctc
OsSK21	P$AT2G47520_01	AT2G47520	2558	2568	1	1	0.957	caGCCGCctc
OsSK21	P$CRF1_02	CRF1	2558	2568	1	1	0.902	caGCCGCctc
OsSK21	P$OPBP1_01	OPBP1	2558	2568	1	1	0.886	caGCCGCctc
OsSK21	P$ATERF14_01	ATERF14	2558	2568	1	1	0.905	caGCCGCctc
OsSK21	P$ERF112_01	ERF112	2558	2568	1	1	0.91	caGCCGCctc
OsSK21	P$ERF1_02	ERF1	2558	2568	1	1	0.963	caGCCGCctc
OsSK21	P$ERF4_02	ERF4	2558	2568	1	1	0.92	caGCCGCctc
OsSK21	P$AT5G25390_01	AT5G25390	2558	2568	1	1	0.874	caGCCGCctc
OsSK21	P$EREBP1_01	EREBP1	2558	2568	1	1	0.974	caGCCGCctc
OsSK21	P$ERF110_02	ERF110	2558	2568	1	1	0.943	caGCCGCctc
OsSK21	P$CBF3_01	CBF3	2558	2568	1	1	0.85	caGCCGCctc
OsSK21	P$DREBII1_01	DREBII1	2558	2568	1	1	0.862	caGCCGCctc
OsSK21	P$DREB1A_01	DREB1A	2558	2568	1	1	0.853	caGCCGCctc
OsSK21	P$TSRF1_01	TSRF1	2558	2568	1	1	0.874	caGCCGCctc
OsSK21	P$DRF13_01	DRF13	2558	2568	1	1	0.863	caGCCGCctc
OsSK21	P$ERF2_03	ERF2	2558	2568	1	1	0.966	caGCCGCctc
OsSK21	P$ERF1B_03	ERF1B	2558	2568	1	1	0.882	caGCCGCctc
OsSK21	P$RAP26_02	RAP26	2558	2568	1	1	0.906	caGCCGCctc
OsSK21	P$CBF16_01	CBF16	2558	2568	1	1	0.857	caGCCGCctc
OsSK21	P$CBF17_01	CBF17	2558	2568	1	1	0.885	caGCCGCctc
OsSK21	P$ERF1_05	ERF1	2558	2568	1	1	0.872	caGCCGCctc
OsSK21	P$AT5G25190_01	AT5G25190	2558	2568	1	1	0.883	caGCCGCctc
OsSK21	P$ERF105_01	ERF105	2558	2568	1	1	0.9	caGCCGCctc
OsSK21	P$CBF_01	CBF	2558	2568	1	1	0.89	caGCCGCctc
OsSK21	P$AT5G11190_01	AT5G11190	2558	2568	1	1	0.867	caGCCGCctc
OsSK21	P$AT1G68550_01	AT1G68550	2558	2568	1	1	0.944	caGCCGCctc
OsSK21	P$AT1G77640_01	AT1G77640	2558	2568	1	1	0.879	caGCCGCctc
OsSK21	P$AT3G61630_01	AT3G61630	2558	2568	1	1	0.953	caGCCGCctc
OsSK21	P$AT5G43410_01	AT5G43410	2558	2568	1	1	0.911	caGCCGCctc
OsSK21	P$AT5G07310_01	AT5G07310	2558	2568	1	1	0.944	caGCCGCctc
OsSK21	P$DREB1A_03	DREB1A	2558	2568	1	1	0.854	caGCCGCctc
OsSK21	P$AT1G49120_01	AT1G49120	2558	2568	1	1	0.86	caGCCGCctc
OsSK21	P$DREB2D_01	DREB2D	2558	2568	1	1	0.867	caGCCGCctc
OsSK21	P$AT3G25890_01	AT3G25890	2558	2568	1	1	0.886	caGCCGCctc
OsSK21	P$AT4G23750_01	AT4G23750	2558	2568	1	1	0.854	caGCCGCctc
OsSK21	P$AT4G27950_01	AT4G27950	2558	2568	1	1	0.884	caGCCGCctc
OsSK21	P$RRTF1_01	RRTF1	2558	2568	1	1	0.928	caGCCGCctc
OsSK21	P$ERF1_Q2_01	ERF1	2558	2572	1	1	0.915	caGCCGCctccctc
OsSK21	P$CRF2_01	CRF2	2558	2566	1	1	0.94	caGCCGCc
OsSK21	P$ERF096_01	ERF096	2558	2568	1	1	0.991	caGCCGCctc
OsSK21	P$ERF098_01	ERF098	2558	2566	1	1	0.998	caGCCGCc
OsSK21	P$CBF1_02	CBF1	2558	2568	1	1	0.859	cagCCGCCtc
OsSK21	P$ERF1B_06	ERF1B	2559	2567	1	1	0.971	aGCCGCct
OsSK21	P$ERF7_02	ERF7	2559	2569	1	1	0.987	aGCCGCctcc
OsSK21	P$ERF094_01	ERF094	2559	2567	1	1	0.989	aGCCGCct
OsSK21	P$ERF2_01	ERF2	2559	2566	1	1	0.922	agCCGCC
OsSK21	P$ERF13_02	ERF13	2559	2567	1	1	0.983	agCCGCCt
OsSK21	P$AT3G63350_01	AT3G63350	2561	2567	1	1	0.867	CCGCCt
OsSK21	P$CDC5_01	CDC5	2575	2586	1	1	0.863	agcTCAGCtca
OsSK21	P$CDC5_01	CDC5	2580	2591	1	1	0.863	agcTCAGCtcc
OsSK21	P$ABI3_01	ABI3	2594	2603	1	1	0.893	ccGCATGga
OsSK21	P$ERF73_01	ERF73	2598	2619	1	1	0.912	atggaggCGCCGccggggccg
OsSK21	P$CBF2_03	CBF2	2601	2611	1	1	0.872	gaggCGCCGc
OsSK21	P$ERF4_05	ERF4	2601	2616	1	1	0.896	gaggCGCCGccgggg
OsSK21	P$AT2G33710_01	AT2G33710	2601	2616	1	1	0.886	gaggcgcCGCCGggg
OsSK21	P$ABI4_01	ABI4	2602	2613	1	1	0.914	aggcgCCGCCg
OsSK21	P$RRTF1_02	RRTF1	2603	2613	1	1	0.967	ggCGCCGccg
OsSK21	P$RAP26_03	RAP26	2603	2613	1	1	1	ggCGCCGccg
OsSK21	P$RAP210_04	RAP210	2603	2613	1	1	0.977	ggCGCCGccg

OsSK21	P$ERF112_02	ERF112	2603	2613	1	1	0.99	ggCGCCGccg
OsSK21	P$CRF4_01	CRF4	2604	2612	1	1	1	gCGCCGcc
OsSK21	P$ERF4_04	ERF4	2604	2612	1	1	0.99	gCGCCGcc
OsSK21	P$ERF069_01	ERF069	2604	2613	1	1	0.999	gCGCCGccg
OsSK21	P$ERF11_01	ERF11	2604	2614	1	1	0.992	gCGCCGccgg
OsSK21	P$ERF13_01	ERF13	2604	2614	1	1	0.858	gcGCCGCcgg
OsSK21	P$ABI4_03	ABI4	2604	2614	1	1	0.955	gcGCCGCcgg
OsSK21	P$WRAF1_01	WRAF1	2604	2614	1	1	0.865	gcGCCGCcgg
OsSK21	P$PTI5_01	TI5	2604	2614	1	1	0.92	gcGCCGCcgg
OsSK21	P$DREBI5_01	DREBI5	2604	2614	1	1	0.946	gcGCCGCcgg
OsSK21	P$AT2G47520_01	AT2G47520	2604	2614	1	1	0.936	gcGCCGCcgg
OsSK21	P$DREB2B_01	DREB2B	2604	2614	1	1	0.916	gcGCCGCcgg
OsSK21	P$CRF1_02	CRF1	2604	2614	1	1	0.908	gcGCCGCcgg
OsSK21	P$OPBP1_01	OPBP1	2604	2614	1	1	0.923	gcGCCGCcgg
OsSK21	P$ATERF14_01	ATERF14	2604	2614	1	1	0.904	gcGCCGCcgg
OsSK21	P$DREB2A_02	DREB2A	2604	2614	1	1	0.86	gcGCCGCcgg
OsSK21	P$ERF1_02	ERF1	2604	2614	1	1	0.902	gcGCCGCcgg
OsSK21	P$ERF4_02	ERF4	2604	2614	1	1	0.963	gcGCCGCcgg
OsSK21	P$AT5G25390_01	AT5G25390	2604	2614	1	1	0.909	gcGCCGCcgg
OsSK21	P$EREBP1_01	EREBP1	2604	2614	1	1	0.916	gcGCCGCcgg
OsSK21	P$CBF3_01	CBF3	2604	2614	1	1	0.888	gcGCCGCcgg
OsSK21	P$DREBII1_01	DREBII1	2604	2614	1	1	0.926	gcGCCGCcgg
OsSK21	P$DREB1A_01	DREB1A	2604	2614	1	1	0.851	gcGCCGCcgg
OsSK21	P$TSRF1_01	TSRF1	2604	2614	1	1	0.914	gcGCCGCcgg
OsSK21	P$DRF13_01	DRF13	2604	2614	1	1	0.899	gcGCCGCcgg
OsSK21	P$ERF4_03	ERF4	2604	2614	1	1	0.863	gcGCCGCcgg
OsSK21	P$ERF2_03	ERF2	2604	2614	1	1	0.911	gcGCCGCcgg
OsSK21	P$ERF1B_03	ERF1B	2604	2614	1	1	0.922	gcGCCGCcgg
OsSK21	P$RAP26_02	RAP26	2604	2614	1	1	0.852	gcGCCGCcgg
OsSK21	P$CBF5_01	CBF5	2604	2614	1	1	0.904	gcGCCGCcgg
OsSK21	P$CBF16_01	CBF16	2604	2614	1	1	0.922	gcGCCGCcgg
OsSK21	P$CBF17_01	CBF17	2604	2614	1	1	0.95	gcGCCGCcgg
OsSK21	P$ERF1_05	ERF1	2604	2614	1	1	0.927	gcGCCGCcgg
OsSK21	P$AT5G25190_01	AT5G25190	2604	2614	1	1	0.926	gcGCCGCcgg
OsSK21	P$ERF105_01	ERF105	2604	2614	1	1	0.918	gcGCCGCcgg
OsSK21	P$CBF_01	CBF	2604	2614	1	1	0.95	gcGCCGCcgg
OsSK21	P$AT5G11190_01	AT5G11190	2604	2614	1	1	0.904	gcGCCGCcgg
OsSK21	P$AT1G68550_01	AT1G68550	2604	2614	1	1	0.98	gcGCCGCcgg
OsSK21	P$AT1G77640_01	AT1G77640	2604	2614	1	1	0.875	gcGCCGCcgg
OsSK21	P$ERF016_01	ERF016	2604	2614	1	1	0.899	gcGCCGCcgg
OsSK21	P$AT3G61630_01	AT3G61630	2604	2614	1	1	0.99	gcGCCGCcgg
OsSK21	P$AT5G43410_01	AT5G43410	2604	2614	1	1	0.91	gcGCCGCcgg
OsSK21	P$AT5G07310_01	AT5G07310	2604	2614	1	1	0.916	gcGCCGCcgg
OsSK21	P$AT3G16280_01	AT3G16280	2604	2614	1	1	0.851	gcGCCGCcgg
OsSK21	P$DREB1A_03	DREB1A	2604	2614	1	1	0.893	gcGCCGCcgg
OsSK21	P$AT1G49120_01	AT1G49120	2604	2614	1	1	0.883	gcGCCGCcgg
OsSK21	P$DREB2D_01	DREB2D	2604	2614	1	1	0.905	gcGCCGCcgg
OsSK21	P$AT3G25890_01	AT3G25890	2604	2614	1	1	0.891	gcGCCGCcgg
OsSK21	P$AT4G23750_01	AT4G23750	2604	2614	1	1	0.86	gcGCCGCcgg
OsSK21	P$AT4G27950_01	AT4G27950	2604	2614	1	1	0.891	gcGCCGCcgg
OsSK21	P$RRTF1_01	RRTF1	2604	2614	1	1	0.94	gcGCCGCcgg
OsSK21	P$CRF2_01	CRF2	2604	2612	1	1	0.995	gcGCCGCc
OsSK21	P$ERF096_01	ERF096	2604	2614	1	1	0.998	gcGCCGCcgg
OsSK21	P$ERF098_01	ERF098	2604	2612	1	1	1	gcGCCGCc
OsSK21	P$DREB2C_01	DREB2C	2604	2614	1	1	0.913	gcgCCGCCgg
OsSK21	P$CBF1_02	CBF1	2604	2614	1	1	0.865	gcgCCGCCgg
OsSK21	P$CBF2_03	CBF2	2604	2614	1	1	0.957	gcgcCGCCGg
OsSK21	P$ERF8_01	ERF8	2605	2615	1	1	0.991	CGCCGccggg
OsSK21	P$ERF3_04	ERF3	2605	2613	1	1	0.983	CGCCGccg
OsSK21	P$OS05G0497200_01	OS05G0497200	2605	2613	1	1	1	CGCCGccg
OsSK21	P$ERF1B_06	ERF1B	2605	2613	1	1	0.985	cGCCGCcg
OsSK21	P$ERF7_02	ERF7	2605	2615	1	1	0.994	cGCCGCcggg
OsSK21	P$ERF094_01	ERF094	2605	2613	1	1	1	cGCCGCcg
OsSK21	P$ERF2_01	ERF2	2605	2612	1	1	1	cgCCGCC
OsSK21	P$ERF13_02	ERF13	2605	2613	1	1	0.996	cgCCGCCg
OsSK21	P$ERF112_02	ERF112	2606	2616	1	1	0.918	gcCGCCGggg
OsSK21	P$AT3G63350_01	AT3G63350	2607	2613	1	1	0.866	CCGCCg
OsSK21	P$CRF4_01	CRF4	2607	2615	1	1	0.863	cCGCCGgg
OsSK21	P$ERF4_04	ERF4	2607	2615	1	1	0.869	cCGCCGgg
OsSK21	P$ERF069_01	ERF069	2607	2616	1	1	0.989	cCGCCGggg
OsSK21	P$ERF11_01	ERF11	2607	2617	1	1	0.958	cCGCCGgggc
OsSK21	P$ERF8_01	ERF8	2608	2618	1	1	0.933	CGCCGgggcc
OsSK21	P$HSF3_01	HSF3	2610	2616	1	1	1	cCGGGG
OsSK21	P$AT1G53910_01	AT1G53910	2613	2623	1	1	0.892	gGGCCGgagc
OsSK21	P$FAR1_01	FAR1	2630	2645	1	1	0.884	gctggACGCGccacc
OsSK21	P$FHY3_01	FHY3	2632	2644	1	1	0.858	tggACGCGccac
OsSK21	P$TRAB1_Q2	TRAB1	2633	2644	1	1	0.906	ggACGCGccac
OsSK21	P$E2FA_02	E2FA	2635	2645	1	1	0.991	acgCGCCAcc

OsSK21	P$ABI4_01	ABI4	2638	2649	1	1	0.941	cgccaCCGCCc
OsSK21	P$AT5G46350_01	AT5G46350	2642	2651	1	1	0.854	ACCGCcccc
OsSK21	P$AT3G01030_01	AT3G01030	2642	2651	1	1	0.856	ACCGCcccc
OsSK21	P$AT3G63350_01	AT3G63350	2643	2649	1	1	1	CCGCCc
OsSK21	P$AT2G33710_01	AT2G33710	2644	2659	1	1	0.946	cgcccccCGCCGccg
OsSK21	P$ERF73_01	ERF73	2644	2665	1	1	0.975	cgcccccCGCCGccgtggcgg
OsSK21	P$ERF4_05	ERF4	2647	2662	1	1	0.975	ccccCGCCGccgtgg
OsSK21	P$AT2G33710_01	AT2G33710	2647	2662	1	1	0.943	cccccgcCGCCGtgg
OsSK21	P$ABI4_01	ABI4	2648	2659	1	1	0.853	ccccgCCGCCg
OsSK21	P$RRTF1_02	RRTF1	2649	2659	1	1	0.934	ccCGCCGccg
OsSK21	P$RAP26_03	RAP26	2649	2659	1	1	0.967	ccCGCCGccg
OsSK21	P$RAP210_04	RAP210	2649	2659	1	1	0.924	ccCGCCGccg
OsSK21	P$ERF112_02	ERF112	2649	2659	1	1	0.992	ccCGCCGccg
OsSK21	P$AT3G63350_01	AT3G63350	2650	2656	1	1	0.866	CCGCCg
OsSK21	P$CRF4_01	CRF4	2650	2658	1	1	0.99	cCGCCGcc
OsSK21	P$ERF4_04	ERF4	2650	2658	1	1	1	cCGCCGcc
OsSK21	P$ERF069_01	ERF069	2650	2659	1	1	0.999	cCGCCGccg
OsSK21	P$ERF11_01	ERF11	2650	2660	1	1	0.993	cCGCCGccgt
OsSK21	P$ABI4_03	ABI4	2650	2660	1	1	0.954	ccGCCGCcgt
OsSK21	P$WRAF1_01	WRAF1	2650	2660	1	1	0.865	ccGCCGCcgt
OsSK21	P$PTI5_01	TI5	2650	2660	1	1	0.918	ccGCCGCcgt
OsSK21	P$DREBI5_01	DREBI5	2650	2660	1	1	0.944	ccGCCGCcgt
OsSK21	P$AT2G47520_01	AT2G47520	2650	2660	1	1	0.931	ccGCCGCcgt
OsSK21	P$DREB2B_01	DREB2B	2650	2660	1	1	0.944	ccGCCGCcgt
OsSK21	P$CRF1_02	CRF1	2650	2660	1	1	0.895	ccGCCGCcgt
OsSK21	P$OPBP1_01	OPBP1	2650	2660	1	1	0.918	ccGCCGCcgt
OsSK21	P$ATERF14_01	ATERF14	2650	2660	1	1	0.906	ccGCCGCcgt
OsSK21	P$DREB2A_02	DREB2A	2650	2660	1	1	0.855	ccGCCGCcgt
OsSK21	P$ERF1_02	ERF1	2650	2660	1	1	0.9	ccGCCGCcgt
OsSK21	P$ERF4_02	ERF4	2650	2660	1	1	0.962	ccGCCGCcgt
OsSK21	P$AT5G25390_01	AT5G25390	2650	2660	1	1	0.91	ccGCCGCcgt
OsSK21	P$EREBP1_01	EREBP1	2650	2660	1	1	0.913	ccGCCGCcgt
OsSK21	P$CBF3_01	CBF3	2650	2660	1	1	0.889	ccGCCGCcgt
OsSK21	P$DREBII1_01	DREBII1	2650	2660	1	1	0.924	ccGCCGCcgt
OsSK21	P$TSRF1_01	TSRF1	2650	2660	1	1	0.909	ccGCCGCcgt
OsSK21	P$DRF13_01	DRF13	2650	2660	1	1	0.895	ccGCCGCcgt
OsSK21	P$ERF4_03	ERF4	2650	2660	1	1	0.856	ccGCCGCcgt
OsSK21	P$ERF2_03	ERF2	2650	2660	1	1	0.908	ccGCCGCcgt
OsSK21	P$ERF1B_03	ERF1B	2650	2660	1	1	0.921	ccGCCGCcgt
OsSK21	P$RAP26_02	RAP26	2650	2660	1	1	0.854	ccGCCGCcgt
OsSK21	P$CBF5_01	CBF5	2650	2660	1	1	0.902	ccGCCGCcgt
OsSK21	P$CBF16_01	CBF16	2650	2660	1	1	0.92	ccGCCGCcgt
OsSK21	P$CBF17_01	CBF17	2650	2660	1	1	0.948	ccGCCGCcgt
OsSK21	P$ERF1_05	ERF1	2650	2660	1	1	0.928	ccGCCGCcgt
OsSK21	P$AT5G25190_01	AT5G25190	2650	2660	1	1	0.927	ccGCCGCcgt
OsSK21	P$ERF105_01	ERF105	2650	2660	1	1	0.915	ccGCCGCcgt
OsSK21	P$CBF_01	CBF	2650	2660	1	1	0.948	ccGCCGCcgt
OsSK21	P$AT5G11190_01	AT5G11190	2650	2660	1	1	0.905	ccGCCGCcgt
OsSK21	P$AT1G68550_01	AT1G68550	2650	2660	1	1	0.98	ccGCCGCcgt
OsSK21	P$AT1G77640_01	AT1G77640	2650	2660	1	1	0.878	ccGCCGCcgt
OsSK21	P$ERF016_01	ERF016	2650	2660	1	1	0.89	ccGCCGCcgt
OsSK21	P$AT3G61630_01	AT3G61630	2650	2660	1	1	0.969	ccGCCGCcgt
OsSK21	P$AT5G43410_01	AT5G43410	2650	2660	1	1	0.912	ccGCCGCcgt
OsSK21	P$AT5G07310_01	AT5G07310	2650	2660	1	1	0.922	ccGCCGCcgt
OsSK21	P$AT3G16280_01	AT3G16280	2650	2660	1	1	0.851	ccGCCGCcgt
OsSK21	P$DREB1A_03	DREB1A	2650	2660	1	1	0.894	ccGCCGCcgt
OsSK21	P$AT1G49120_01	AT1G49120	2650	2660	1	1	0.879	ccGCCGCcgt
OsSK21	P$DREB2D_01	DREB2D	2650	2660	1	1	0.903	ccGCCGCcgt
OsSK21	P$AT3G25890_01	AT3G25890	2650	2660	1	1	0.89	ccGCCGCcgt
OsSK21	P$AT4G23750_01	AT4G23750	2650	2660	1	1	0.857	ccGCCGCcgt
OsSK21	P$AT4G27950_01	AT4G27950	2650	2660	1	1	0.888	ccGCCGCcgt
OsSK21	P$RRTF1_01	RRTF1	2650	2660	1	1	0.951	ccGCCGCcgt
OsSK21	P$CRF2_01	CRF2	2650	2658	1	1	1	ccGCCGCc
OsSK21	P$ERF096_01	ERF096	2650	2660	1	1	0.998	ccGCCGCcgt
OsSK21	P$ERF098_01	ERF098	2650	2658	1	1	1	ccGCCGCc
OsSK21	P$DREB2C_01	DREB2C	2650	2660	1	1	0.912	ccgCCGCCgt
OsSK21	P$CBF1_02	CBF1	2650	2660	1	1	0.894	ccgCCGCCgt
OsSK21	P$CBF2_03	CBF2	2650	2660	1	1	0.949	ccgcCGCCGt
OsSK21	P$AT1G53910_02	AT1G53910	2650	2671	1	1	0.972	ccgccgccgtgGCGGCggcgg
OsSK21	P$ERF8_01	ERF8	2651	2661	1	1	0.992	CGCCGccgtg
OsSK21	P$ERF3_04	ERF3	2651	2659	1	1	0.983	CGCCGccg
OsSK21	P$OS05G0497200_01	OS05G0497200	2651	2659	1	1	1	CGCCGccg
OsSK21	P$ERF1B_06	ERF1B	2651	2659	1	1	0.985	cGCCGCcg
OsSK21	P$ERF7_02	ERF7	2651	2661	1	1	0.995	cGCCGCcgtg
OsSK21	P$ERF094_01	ERF094	2651	2659	1	1	1	cGCCGCcg
OsSK21	P$ERF2_01	ERF2	2651	2658	1	1	1	cgCCGCC
OsSK21	P$ERF13_02	ERF13	2651	2659	1	1	0.996	cgCCGCCg
OsSK21	P$ERF112_02	ERF112	2652	2662	1	1	0.923	gcCGCCGtgg

OsSK21	P$AT3G63350_01	AT3G63350	2653	2659	1	1	0.866	CCGCCg
OsSK21	P$CRF4_01	CRF4	2653	2661	1	1	0.866	cCGCCGtg
OsSK21	P$ERF4_04	ERF4	2653	2661	1	1	0.871	cCGCCGtg
OsSK21	P$ERF069_01	ERF069	2653	2662	1	1	0.989	cCGCCGtgg
OsSK21	P$ERF11_01	ERF11	2653	2663	1	1	0.961	cCGCCGtggc
OsSK21	P$AT1G53910_02	AT1G53910	2653	2674	1	1	0.972	ccgccgtggcgGCGGCggcgg
OsSK21	P$ERF8_01	ERF8	2654	2664	1	1	0.936	CGCCGtggcg
OsSK21	P$ANAC042_01	ANAC042	2654	2674	1	1	0.922	cGCCGTggcggcggcggcgg
OsSK21	P$ANAC094_01	ANAC094	2654	2672	1	1	0.91	cGCCGTggcggcggcggc
OsSK21	P$BZIP68_01	BZIP68	2654	2663	1	1	0.948	cgcCGTGGc
OsSK21	P$RRTF1_05	RRTF1	2655	2670	1	1	0.964	gccgtggCGGCGgcg
OsSK21	P$AT1G53910_02	AT1G53910	2656	2677	1	1	0.972	ccgtggcggcgGCGGCggcga
OsSK21	P$RRTF1_05	RRTF1	2658	2673	1	1	0.926	gtggcggCGGCGgcg
OsSK21	P$E2L_Q2	E2L	2659	2666	1	1	0.91	tGGCGGc
OsSK21	P$AT1G28160_02	AT1G28160	2659	2674	1	1	1	tggCGGCGgcggcgg
OsSK21	P$RAP26_06	RAP26	2659	2674	1	1	0.999	tggCGGCGgcggcgg
OsSK21	P$AT1G68550_03	AT1G68550	2659	2668	1	1	0.999	tggCGGCGg
OsSK21	P$AT1G53910_02	AT1G53910	2659	2680	1	1	0.935	tggcggcggcgGCGGCgaccg
OsSK21	P$ERF1_Q2	ERF1	2660	2668	1	1	0.951	GGCGGcgg
OsSK21	P$RRTF1_05	RRTF1	2661	2676	1	1	0.925	gcggcggCGGCGgcg
OsSK21	P$AT1G28160_02	AT1G28160	2662	2677	1	1	0.992	cggCGGCGgcggcga
OsSK21	P$RAP26_06	RAP26	2662	2677	1	1	0.975	cggCGGCGgcggcga
OsSK21	P$AT1G68550_03	AT1G68550	2662	2671	1	1	0.999	cggCGGCGg
OsSK21	P$ERF1_Q2	ERF1	2663	2671	1	1	0.951	GGCGGcgg
OsSK21	P$RRTF1_05	RRTF1	2664	2679	1	1	0.862	gcggcggCGGCGacc
OsSK21	P$AT1G28160_02	AT1G28160	2665	2680	1	1	0.983	cggCGGCGgcgaccg
OsSK21	P$RAP26_06	RAP26	2665	2680	1	1	0.958	cggCGGCGgcgaccg
OsSK21	P$AT1G68550_03	AT1G68550	2665	2674	1	1	0.999	cggCGGCGg
OsSK21	P$ERF1_Q2	ERF1	2666	2674	1	1	0.951	GGCGGcgg
OsSK21	P$AT1G68550_03	AT1G68550	2668	2677	1	1	0.993	cggCGGCGa
OsSK21	P$ERF1_Q2	ERF1	2669	2677	1	1	0.94	GGCGGcga
OsSK21	P$AT2G33710_01	AT2G33710	2671	2686	1	1	0.85	cggcgacCGCCGgca
OsSK21	P$ERF4_05	ERF4	2674	2689	1	1	0.896	cgacCGCCGgcatca
OsSK21	P$AT3G01030_01	AT3G01030	2676	2685	1	1	0.921	ACCGCcggc
OsSK21	P$RRTF1_02	RRTF1	2676	2686	1	1	0.872	acCGCCGgca
OsSK21	P$ERF112_02	ERF112	2676	2686	1	1	0.982	acCGCCGgca
OsSK21	P$AT3G63350_01	AT3G63350	2677	2683	1	1	0.866	CCGCCg
OsSK21	P$CRF4_01	CRF4	2677	2685	1	1	0.932	cCGCCGgc
OsSK21	P$ERF4_04	ERF4	2677	2685	1	1	0.963	cCGCCGgc
OsSK21	P$ERF069_01	ERF069	2677	2686	1	1	0.994	cCGCCGgca
OsSK21	P$ERF11_01	ERF11	2677	2687	1	1	0.995	cCGCCGgcat
OsSK21	P$ERF6_02	ERF6	2677	2687	1	1	0.983	ccGCCGGcat
OsSK21	P$ERF8_01	ERF8	2678	2688	1	1	0.99	CGCCGgcatc
OsSK21	P$ERF3_04	ERF3	2678	2686	1	1	0.974	CGCCGgca
OsSK21	P$ERF105_02	ERF105	2678	2686	1	1	0.936	cGCCGGca
OsSK21	P$ALFIN1_Q2	ALFIN1	2698	2713	1	1	0.96	taacccGTGGGgagg
OsSK21	P$BZIP68_01	BZIP68	2700	2709	1	1	0.927	accCGTGGg
OsSK21	P$GATA9_01	GATA9	2719	2730	1	1	0.996	tccAGATCtgg
OsSK21	P$AGP1_01	AGP1	2720	2730	1	1	0.952	ccAGATCtgg
OsSK21	P$GATA10_01	GATA10	2721	2729	1	1	0.962	cAGATCtg
OsSK21	P$GATA11_01	GATA11	2721	2729	1	1	0.975	caGATCTg
OsSK21	P$GATA8_01	GATA8	2721	2730	1	1	0.999	caGATCTgg
OsSK21	P$ARR10_01	ARR10	2722	2729	1	1	0.978	AGATCtg
OsSK21	P$ARF8_01	ARF8	2730	2739	1	1	0.993	ggTGTCGgg
OsSK21	P$DREB1A_04	DREB1A	2731	2741	1	1	0.942	gtGTCGGggt
OsSK21	P$ERF039_01	ERF039	2731	2741	1	1	0.954	gtGTCGGggt
OsSK21	P$PHYPA182268_05	HYPA182268	2731	2741	1	1	0.878	gtGTCGGggt
OsSK21	P$ERF043_01	ERF043	2732	2740	1	1	0.929	tGTCGGgg
OsSK21	P$PHYPA173530_04	HYPA173530	2732	2740	1	1	0.9	tGTCGGgg
OsSK21	P$PHYPA28324_10	HYPA28324	2732	2740	1	1	0.917	tGTCGGgg
OsSK21	P$HSF3_01	HSF3	2734	2740	1	1	0.94	tCGGGG
OsSK21	P$GATA11_01	GATA11	2740	2748	1	1	0.898	tgGATCTg
OsSK21	P$GATA8_01	GATA8	2740	2749	1	1	0.988	tgGATCTgg
OsSK21	P$CBF1_01	CBF1	2760	2770	1	1	0.854	gTGCCGatct
OsSK21	P$ERF019_01	ERF019	2760	2770	1	1	0.919	gTGCCGatct
OsSK21	P$DREBIII4_01	DREBIII4	2760	2770	1	1	0.926	gTGCCGatct
OsSK21	P$JERF1_01	JERF1	2760	2770	1	1	0.865	gTGCCGatct
OsSK21	P$CBF1_03	CBF1	2760	2770	1	1	0.875	gTGCCGatct
OsSK21	P$AT1G33760_01	AT1G33760	2760	2770	1	1	0.863	gTGCCGatct
OsSK21	P$AT1G71520_01	AT1G71520	2760	2770	1	1	0.866	gTGCCGatct
OsSK21	P$ORA47_01	ORA47	2760	2770	1	1	0.901	gTGCCGatct
OsSK21	P$AT2G20350_01	AT2G20350	2760	2770	1	1	0.89	gtGCCGAtct
OsSK21	P$GATA11_01	GATA11	2763	2771	1	1	0.86	ccGATCTg
OsSK21	P$GATA8_01	GATA8	2763	2772	1	1	0.99	ccGATCTgc
OsSK21	P$PHYPA64121_06	HYPA64121	2774	2787	1	1	0.878	gttTCGGTggtcg
OsSK21	P$AT1G53910_02	AT1G53910	2809	2830	1	1	0.857	tccggtgtctcGCGGCgccgt
OsSK21	P$RRTF1_05	RRTF1	2814	2829	1	1	0.918	tgtctcgCGGCGccg
OsSK21	P$AT2G33710_01	AT2G33710	2817	2832	1	1	0.864	ctcgcggCGCCGttt

OsSK21	P$AT1G28160_02	AT1G28160	2818	2833	1	1	0.883	tcgCGGCGccgtttt
OsSK21	P$RAP26_06	RAP26	2818	2833	1	1	0.86	tcgCGGCGccgtttt
OsSK21	P$AT1G68550_03	AT1G68550	2818	2827	1	1	0.996	tcgCGGCGc
OsSK21	P$CBF2_03	CBF2	2820	2830	1	1	0.968	gcggCGCCGt
OsSK21	P$ERF112_02	ERF112	2822	2832	1	1	0.92	ggCGCCGttt
OsSK21	P$CRF4_01	CRF4	2823	2831	1	1	0.874	gCGCCGtt
OsSK21	P$ERF4_04	ERF4	2823	2831	1	1	0.861	gCGCCGtt
OsSK21	P$ERF069_01	ERF069	2823	2832	1	1	0.989	gCGCCGttt
OsSK21	P$ERF11_01	ERF11	2823	2833	1	1	0.97	gCGCCGtttt
OsSK21	P$ERF8_01	ERF8	2824	2834	1	1	0.945	CGCCGttttt
OsSK21	P$SQUA_01	SQUA	2826	2836	1	1	0.856	ccgTTTTTtt
OsSK21	P$GT1_Q6_01	GT1	2845	2857	1	1	0.873	TTTTTttttggt
OsSK21	P$SEP3_01	wrz-03	2845	2856	1	1	0.906	tttttTTTTGg
OsSK21	P$E2L_Q2	E2L	2882	2889	1	1	0.928	gGGCGGg
OsSK21	P$HSF3_01	HSF3	2884	2890	1	1	0.945	gCGGGG
OsSK21	P$CBF2_03	CBF2	2890	2900	1	1	0.947	gcgaCGCCGt
OsSK21	P$ERF112_02	ERF112	2892	2902	1	1	0.917	gaCGCCGtga
OsSK21	P$CRF4_01	CRF4	2893	2901	1	1	0.854	aCGCCGtg
OsSK21	P$ERF4_04	ERF4	2893	2901	1	1	0.859	aCGCCGtg
OsSK21	P$ERF069_01	ERF069	2893	2902	1	1	0.99	aCGCCGtga
OsSK21	P$ERF11_01	ERF11	2893	2903	1	1	0.966	aCGCCGtgac
OsSK21	P$ERF8_01	ERF8	2894	2904	1	1	0.943	CGCCGtgacg
OsSK21	P$TGA1_01	TGA1	2896	2907	1	1	0.939	ccgTGACGggt
OsSK21	P$TGA7_01	TGA7	2897	2907	1	1	0.873	cgTGACGggt
OsSK21	P$TGA5_01	TGA5	2898	2906	1	1	0.864	gTGACGgg
OsSK21	P$LIM1_01	LIM1	2917	2929	1	1	0.963	CCACCaccatcg
OsSK21	P$LIM1_01	LIM1	2920	2932	1	1	0.944	CCACCatcggcg
OsSK21	P$RRTF1_05	RRTF1	2920	2935	1	1	0.867	ccaccatCGGCGgca
OsSK21	P$AT1G28160_02	AT1G28160	2924	2939	1	1	0.859	catCGGCGgcaagaa
OsSK21	P$AT1G68550_03	AT1G68550	2924	2933	1	1	0.955	catCGGCGg
OsSK21	P$ERF1_Q2	ERF1	2928	2936	1	1	0.94	GGCGGcaa
OsSK21	P$RRTF1_05	RRTF1	2932	2947	1	1	0.859	gcaagaaCGGCGagc
OsSK21	P$AT1G68550_03	AT1G68550	2936	2945	1	1	0.96	gaaCGGCGa
OsSK21	P$HSFA1E_01	HSFA1E	2938	2944	1	1	0.87	aCGGCG
OsSK21	P$AT2G41690_01	AT2G41690	2946	2952	1	1	0.988	CCGAAg
OsSK21	P$HSF3_01	HSF3	2966	2972	1	1	0.94	tCGGGG
OsSK21	P$PEND_02	END	2985	2995	1	1	0.864	tgTTCTTctt
OsSK21	P$ATSPL8_01	ATSPL8	2992	3008	1	1	0.892	ctttgTGTACagtgga
OsSK21	P$SEP3_01	wrz-03	3012	3023	1	1	0.91	gctagTTTTGg
OsSK21	P$E2L1_Q2	E2L1	3017	3029	1	1	0.947	ttTTGGCgccct
OsSK21	P$ERF38_02	ERF38	3019	3029	1	1	0.926	ttggCGCCCt
OsSK21	P$CBNAC_01	CBNAC	3033	3039	1	1	1	tTGCTT
OsSK21	P$CBNAC_02	CBNAC	3033	3049	1	1	0.864	tTGCTTccttgctcgt
OsSK21	P$NAC6_01	NAC6	3045	3051	1	1	0.854	tCGTAA
OsSK21	P$ATMYB15_Q2	ATMYB15	3048	3054	1	1	1	TAACAa
OsSK21	P$ATHB7_01	ATHB7	3050	3060	1	1	0.93	acAATCActt
OsSK21	P$HAT1_01	HAT1	3050	3060	1	1	0.879	acAATCActt
OsSK21	P$MYB3R1_01	MYB3R1	3081	3096	1	1	0.863	ctcttaagCCGTTtc
OsSK21	P$MYB3R4_01	MYB3R4	3081	3096	1	1	0.864	ctcttaagCCGTTtc
OsSK21	P$GATA15_01	GATA15	3116	3125	1	1	0.999	gcTGATCaa
OsSK21	P$MRP1_Q2	MRP1	3131	3143	1	1	0.888	taTCTATgtgct
OsSK21	P$DREB1A_04	DREB1A	3146	3156	1	1	0.931	tcGTCGGgga
OsSK21	P$ERF039_01	ERF039	3146	3156	1	1	0.948	tcGTCGGgga
OsSK21	P$PHYPA182268_05	HYPA182268	3146	3156	1	1	0.869	tcGTCGGgga
OsSK21	P$PHYPA173530_04	HYPA173530	3147	3155	1	1	0.858	cGTCGGgg
OsSK21	P$PHYPA28324_10	HYPA28324	3147	3155	1	1	0.897	cGTCGGgg
OsSK21	P$HSF3_01	HSF3	3149	3155	1	1	0.94	tCGGGG
OsSK21	P$ARR1_01	ARR1	3151	3161	1	1	0.94	gggGAATCgc
OsSK21	P$ALFIN1_Q2	ALFIN1	3161	3176	1	1	0.858	aattttGTGGGtttg
OsSK21	P$ARR18_01	ARR18	3181	3194	1	1	0.952	gactAGATAcagt
OsSK21	P$OSRR22_01	OSRR22	3184	3194	1	1	0.86	taGATACagt
OsSK21	P$ATMYB77_01	ATMYB77	3184	3197	1	1	0.864	tagataCAGTTgt
OsSK21	P$HMG1_01	HMG1	3192	3201	1	1	0.919	GTTGTcgtt
OsSK21	P$ID1_01	ID1	3192	3203	1	1	0.905	gTTGTCgtttt
OsSK21	P$ARF8_01	ARF8	3192	3201	1	1	0.956	gtTGTCGtt
OsSK21	P$GT1_Q6	GT1	3208	3215	1	1	0.912	GTAAAca
OsSK21	P$AT3G20750_01	AT3G20750	3208	3216	1	1	0.85	gTAAACaa
OsSK21	P$WRKY11_Q2	WRKY11	3223	3231	1	1	0.927	tTTGACtg
OsSK21	P$NAC043_01	NAC043	3236	3246	1	1	0.922	attACGTAgt
OsSK21	P$PDF2_01	DF2	3254	3265	1	1	0.905	caatTAAATgt
OsSK21	P$ATSPL8_01	ATSPL8	3286	3302	1	1	0.966	gataaTGTACatttta
OsSK21	P$TEIL_01	TEIL	3290	3298	1	1	0.94	ATGTAcat
OsSK21	P$MYB24_01	MYB24	3296	3305	1	1	0.862	attTTAGGa
OsSK21	P$ABI3_01	ABI3	3304	3313	1	1	0.853	aaGCATGat
OsSK21	P$AGL27_01	AGL27	3312	3326	1	1	0.899	tTTTCTttttctgt
OsSK21	P$HSFA4A_01	HSFA4A	3328	3334	1	1	0.91	gCTATT
OsSK21	P$SPF1_Q2	SPF1	3334	3344	1	1	0.919	tcATAGTcta
OsSK21	P$ML1_01	ML1	3361	3373	1	1	0.871	ggtaaTAAATgc

OsSK21	P$ATHB6_01	ATHB6	3362	3371	1	1	0.914	gtAATAAat
OsSK21	P$PDF2_01	DF2	3362	3373	1	1	0.971	gtaaTAAATgc
OsSK21	P$CBNAC_01	CBNAC	3369	3375	1	1	0.968	aTGCTT
OsSK21	P$CBNAC_02	CBNAC	3369	3385	1	1	0.906	aTGCTTcacttctaat
OsSK21	P$WRKY_Q2	WRKY	3386	3397	1	1	0.941	ttagttAGTCA
OsSK21	P$WEREWOLF_Q2	WEREWOLF	3387	3396	1	1	0.906	taGTTAGtc
OsSK21	P$GAMYB_Q2	GAMYB	3414	3427	1	1	0.873	gatgcACAACata
OsSK21	P$RAV1_01	RAV1	3417	3429	1	1	0.955	gcaCAACAtaga
OsSK21	P$ARR1_01	ARR1	3424	3434	1	1	0.953	ataGAATCcg
OsSK21	P$WRKY18_02	WRKY18	3430	3440	1	1	0.946	tccGTCAAtc
OsSK21	P$WRKY21_02	WRKY21	3430	3440	1	1	0.949	tccGTCAAtc
OsSK21	P$WRKY48_02	WRKY48	3430	3440	1	1	0.987	tccGTCAAtc
OsSK21	P$WRKY57_01	WRKY57	3430	3440	1	1	0.954	tccGTCAAtc
OsSK21	P$WRKY60_01	WRKY60	3430	3441	1	1	0.891	tccGTCAAtct
OsSK21	P$WRKY15_01	WRKY15	3431	3441	1	1	0.96	ccGTCAAtct
OsSK21	P$WRKY2_01	WRKY2	3431	3439	1	1	0.907	ccGTCAAt
OsSK21	P$WRKY25_02	WRKY25	3431	3439	1	1	0.894	ccGTCAAt
OsSK21	P$WRKY40_01	WRKY40	3431	3439	1	1	0.981	ccGTCAAt
OsSK21	P$WRKY43_02	WRKY43	3431	3441	1	1	0.953	ccGTCAAtct
OsSK21	P$WRKY63_01	WRKY63	3431	3439	1	1	0.887	ccGTCAAt
OsSK21	P$WRKY75_01	WRKY75	3431	3439	1	1	0.928	ccGTCAAt
OsSK21	P$WRKY8_01	WRKY8	3431	3440	1	1	0.978	ccGTCAAtc
OsSK21	P$WRKY30_01	WRKY30	3432	3442	1	1	0.91	cGTCAAtctt
OsSK21	P$WRKY18_Q2	WRKY18	3433	3442	1	1	0.957	GTCAAtctt
OsSK21	P$ARR2_01	ARR2	3433	3443	1	1	0.868	gtcaATCTTg
OsSK21	P$ARR18_01	ARR18	3439	3452	1	1	0.905	cttgAGATAttgt
OsSK21	P$LEC2_01	LEC2	3461	3472	1	1	0.967	ctCATGCgatt
OsSK21	P$SQUA_01	SQUA	3467	3477	1	1	0.871	cgaTTTTTtt
OsSK21	P$GT1_Q6_01	GT1	3471	3483	1	1	0.95	TTTTTtcttacc
OsSK21	P$AT1G66560_01	AT1G66560	3480	3490	1	1	0.857	accTTAACtg
OsSK21	P$AT1G18860_01	AT1G18860	3481	3490	1	1	0.887	ccTTAACtg
OsSK21	P$AT1G64000_01	AT1G64000	3481	3490	1	1	0.851	ccTTAACtg
OsSK21	P$AT1G66600_01	AT1G66600	3481	3490	1	1	0.859	ccTTAACtg
OsSK21	P$AT5G41570_01	AT5G41570	3481	3490	1	1	0.851	ccTTAACtg
OsSK21	P$AT5G15130_01	AT5G15130	3481	3490	1	1	0.882	ccTTAACtg
OsSK21	P$MYBPH3_02	MYBPH3	3492	3505	1	1	0.86	gacaatTAGTTat
OsSK21	P$E2L_Q2	E2L	3506	3513	1	1	0.892	tGGCGGa
OsSK21	P$SPL11_01	SPL11	3549	3561	1	1	0.887	ccagGTACGatg
OsSK21	P$SPL5_01	SPL5	3551	3560	1	1	0.971	agGTACGat
OsSK21	P$POPTR_01	OPTR	3552	3559	1	1	0.944	gGTACGa
OsSK21	P$SPL12_01	SPL12	3552	3560	1	1	0.982	gGTACGat
OsSK21	P$SPL4_01	SPL4	3552	3561	1	1	0.994	gGTACGatg
OsSK21	P$CBNAC_01	CBNAC	3558	3564	1	1	0.968	aTGCTT
OsSK21	P$ABI3_01	ABI3	3576	3585	1	1	0.963	atGCATGct
OsSK21	P$FUS3_01	FUS3	3577	3586	1	1	0.955	tGCATGctt
OsSK21	P$LEC2_01	LEC2	3577	3588	1	1	0.941	tgCATGCttac
OsSK21	P$CBNAC_01	CBNAC	3580	3586	1	1	0.968	aTGCTT
OsSK21	P$CBNAC_02	CBNAC	3580	3596	1	1	0.89	aTGCTTaccgtgtatt
OsSK21	P$MYB89_01	MYB89	3583	3594	1	1	0.903	ctTACCGtgta
OsSK21	P$ATSPL8_01	ATSPL8	3590	3606	1	1	0.974	tgtatTGTACattttt
OsSK21	P$SQUA_01	SQUA	3599	3609	1	1	0.878	catTTTTTga
OsSK21	P$GT1_Q6_01	GT1	3601	3613	1	1	0.873	TTTTTtgatatg
OsSK21	P$ARR1_01	ARR1	3613	3623	1	1	0.942	tttGAATCaa
OsSK21	P$ATHB7_01	ATHB7	3615	3625	1	1	0.875	tgAATCAaac
OsSK21	P$HAT1_01	HAT1	3615	3625	1	1	0.864	tgAATCAaac
OsSK21	P$MYB24_01	MYB24	3639	3648	1	1	0.909	catTTAGGc
OsSK21	P$CBNAC_01	CBNAC	3651	3657	1	1	0.979	gTGCTT
OsSK21	P$CBNAC_02	CBNAC	3651	3667	1	1	0.865	gTGCTTggagacgggg
OsSK21	P$HSF3_01	HSF3	3661	3667	1	1	0.969	aCGGGG
OsSK21	P$MYB118_01	MYB118	3695	3712	1	1	0.9	aggaccggcGTTACaag
OsSK21	P$AT1G68550_03	AT1G68550	3697	3706	1	1	0.949	gacCGGCGt
OsSK21	P$HSFA1E_01	HSFA1E	3699	3705	1	1	1	cCGGCG
OsSK21	P$CBNAC_01	CBNAC	3771	3777	1	1	0.973	cTGCTT
OsSK21	P$CBNAC_02	CBNAC	3771	3787	1	1	0.855	cTGCTTcttctcaacc
OsSK21	P$PEND_02	END	3773	3783	1	1	0.864	gcTTCTTctc
OsSK21	P$AT5G26170_01	AT5G26170	3779	3788	1	1	0.937	tcTCAACca
OsSK21	P$AT4G36620_01	AT4G36620	3780	3788	1	1	0.976	ctcAACCA
OsSK21	P$GAMYB_01	GAMYB	3782	3790	1	1	0.901	CAACCaca
OsSK21	P$O2_03	O2	3796	3806	1	1	0.885	GATGAgctgt
OsSK21	P$ARR1_01	ARR1	3807	3817	1	1	0.98	cctGAATCtt
OsSK21	P$ARR2_01	ARR2	3808	3818	1	1	0.965	ctgaATCTTg
OsSK21	P$SPL11_01	SPL11	3821	3833	1	1	0.899	tggaGTACGtcc
OsSK21	P$SPL5_01	SPL5	3823	3832	1	1	0.973	gaGTACGtc
OsSK21	P$POPTR_01	OPTR	3824	3831	1	1	0.93	aGTACGt
OsSK21	P$SPL12_01	SPL12	3824	3832	1	1	0.977	aGTACGtc
OsSK21	P$SPL4_01	SPL4	3824	3833	1	1	0.992	aGTACGtcc
OsSK21	P$TGA1A_01	TGA1A	3826	3833	1	1	0.872	tACGTCc
OsSK21	P$P_01		3841	3850	1	1	0.874	ctCTACCgt

OsSK21	P$MYB89_01	MYB89	3842	3853	1	1	0.863	tcTACCGtgtg
OsSK21	P$ATHSFA1D_01	ATHSFA1D	3860	3866	1	1	1	aCTACA
OsSK21	P$MYBAS1_01	MYBAS1	3870	3881	1	1	0.974	tgCTAACcagc
OsSK21	P$GT1_01	GT1	3871	3879	1	1	0.872	gcTAACCa
OsSK21	P$AT4G36620_01	AT4G36620	3871	3879	1	1	0.876	gctAACCA
OsSK21	P$GATA15_01	GATA15	3888	3897	1	1	0.999	atTGATCta
OsSK21	P$GATA8_01	GATA8	3889	3898	1	1	0.986	ttGATCTat
OsSK21	P$LIM1_01	LIM1	3928	3940	1	1	0.917	CCACCatgttaa
OsSK21	P$GT1_Q6_02	GT1	3930	3942	1	1	0.984	accatgTTAACa
OsSK21	P$AP2A_01	AP2A	3934	3944	1	1	0.881	tgttaACACC
OsSK21	P$CBNAC_01	CBNAC	3959	3965	1	1	0.968	aTGCTT
OsSK21	P$CBNAC_01	CBNAC	3969	3975	1	1	1	tTGCTT
OsSK21	P$CBNAC_02	CBNAC	3969	3985	1	1	0.882	tTGCTTctctaaagat
OsSK21	P$DOF1_01	DOF1	3975	3986	1	1	0.979	ctcTAAAGata
OsSK21	P$ARR18_01	ARR18	3977	3990	1	1	0.945	ctaaAGATActtt
OsSK21	P$DOF43_01	DOF43	3979	3990	1	1	0.999	aaagatACTTT
OsSK21	P$OSRR22_01	OSRR22	3980	3990	1	1	0.857	aaGATACttt
OsSK21	P$CBNAC_01	CBNAC	3994	4000	1	1	0.968	aTGCTT
OsSK31	P$SPF1_Q2	SPF1	21	31	1	1	0.932	acATAGTgaa
OsSK31	P$SBF1_01	SBF1	44	58	1	1	0.881	aaaaaaTTAAAaaa
OsSK31	P$ATHB7_01	ATHB7	56	66	1	1	0.873	aaAATCAaat
OsSK31	P$HAT1_01	HAT1	56	66	1	1	0.864	aaAATCAaat
OsSK31	P$MYB24_01	MYB24	63	72	1	1	0.864	aatTTAGGa
OsSK31	P$KNOX3_01	KNOX3	75	87	1	1	0.976	aatcTGACAtcc
OsSK31	P$ATH1_01	ATH1	79	87	1	1	0.927	TGACAtcc
OsSK31	P$WRKY11_01	WRKY11	85	99	1	1	0.862	ccagTTGACtcccc
OsSK31	P$WRKY11_Q2	WRKY11	88	96	1	1	0.928	gTTGACtc
OsSK31	P$AP2B_01	AP2B	88	98	1	1	0.93	gttgACTCCc
OsSK31	P$FAR1_01	FAR1	98	113	1	1	0.85	cacccACGCGgccgt
OsSK31	P$ASR1_01	ASR1	99	104	1	1	1	ACCCA
OsSK31	P$FHY3_01	FHY3	100	112	1	1	0.859	cccACGCGgccg
OsSK31	P$DREB2C_02	DREB2C	104	114	1	1	0.867	cgCGGCCgtc
OsSK31	P$PTI6_01	TI6	106	116	1	1	0.932	cGGCCGtcac
OsSK31	P$TSI1_01	TSI1	106	116	1	1	0.925	cGGCCGtcac
OsSK31	P$AT1G53910_01	AT1G53910	106	116	1	1	0.963	cGGCCGtcac
OsSK31	P$RRTF1_05	RRTF1	110	125	1	1	0.859	cgtcacaCGGCGcca
OsSK31	P$AT1G68550_03	AT1G68550	114	123	1	1	0.966	acaCGGCGc
OsSK31	P$HSFA1E_01	HSFA1E	116	122	1	1	0.87	aCGGCG
OsSK31	P$BBM_01	BBM	117	127	1	1	0.875	cGGCGCcagc
OsSK31	P$E2FA_02	E2FA	117	127	1	1	0.987	cggCGCCAgc
OsSK31	P$E2FA_02	E2FA	123	133	1	1	0.988	cagCGCCAcg
OsSK31	P$FHY3_01	FHY3	127	139	1	1	0.858	gccACGCGgccc
OsSK31	P$TRAB1_Q2	TRAB1	128	139	1	1	0.893	ccACGCGgccc
OsSK31	P$ESR1_01	ESR1	161	171	1	1	0.898	cGGCCGgccc
OsSK31	P$ERF3_01	ERF3	161	171	1	1	0.95	cGGCCGgccc
OsSK31	P$ERF4_01	ERF4	161	171	1	1	0.894	cGGCCGgccc
OsSK31	P$ERF3_02	ERF3	161	171	1	1	0.937	cGGCCGgccc
OsSK31	P$ERF2_04	ERF2	161	171	1	1	0.945	cGGCCGgccc
OsSK31	P$ERF3_03	ERF3	161	171	1	1	0.945	cGGCCGgccc
OsSK31	P$ATERF12_01	ATERF12	161	171	1	1	0.918	cGGCCGgccc
OsSK31	P$AT1G24590_01	AT1G24590	161	171	1	1	0.888	cGGCCGgccc
OsSK31	P$ATERF11_01	ATERF11	161	171	1	1	0.904	cGGCCGgccc
OsSK31	P$ATERF9_01	ATERF9	161	171	1	1	0.887	cGGCCGgccc
OsSK31	P$AT1G44830_01	AT1G44830	161	171	1	1	0.956	cGGCCGgccc
OsSK31	P$ATERF8_01	ATERF8	161	171	1	1	0.892	cGGCCGgccc
OsSK31	P$AT1G53910_01	AT1G53910	161	171	1	1	0.987	cGGCCGgccc
OsSK31	P$ERF6_02	ERF6	161	171	1	1	0.982	cgGCCGGccc
OsSK31	P$ERF105_02	ERF105	162	170	1	1	0.944	gGCCGGcc
OsSK31	P$KNOX3_01	KNOX3	184	196	1	1	0.973	cacaTGACAtgg
OsSK31	P$ATH1_01	ATH1	188	196	1	1	0.933	TGACAtgg
OsSK31	P$AT5G46350_01	AT5G46350	211	220	1	1	0.859	ACCGCtcga
OsSK31	P$AT3G01030_01	AT3G01030	211	220	1	1	0.853	ACCGCtcga
OsSK31	P$KNOX3_01	KNOX3	225	237	1	1	0.976	tgacTGACAagg
OsSK31	P$ATH1_01	ATH1	229	237	1	1	0.919	TGACAagg
OsSK31	P$BPC1_Q2	BPC1	246	252	1	1	0.99	AGAAAc
OsSK31	P$RRTF1_05	RRTF1	260	275	1	1	0.901	atcacgcCGGCGgcc
OsSK31	P$ERF112_02	ERF112	262	272	1	1	0.964	caCGCCGgcg
OsSK31	P$CRF4_01	CRF4	263	271	1	1	0.92	aCGCCGgc
OsSK31	P$ERF4_04	ERF4	263	271	1	1	0.951	aCGCCGgc
OsSK31	P$ERF069_01	ERF069	263	272	1	1	0.992	aCGCCGgcg
OsSK31	P$ERF11_01	ERF11	263	273	1	1	0.985	aCGCCGgcgg
OsSK31	P$ERF6_02	ERF6	263	273	1	1	0.991	acGCCGGcgg
OsSK31	P$ERF8_01	ERF8	264	274	1	1	0.982	CGCCGgcggc
OsSK31	P$ERF3_04	ERF3	264	272	1	1	0.956	CGCCGgcg
OsSK31	P$ERF105_02	ERF105	264	272	1	1	0.97	cGCCGGcg
OsSK31	P$AT1G28160_02	AT1G28160	264	279	1	1	0.881	cgcCGGCGgccacga
OsSK31	P$RAP26_06	RAP26	264	279	1	1	0.868	cgcCGGCGgccacga
OsSK31	P$AT1G68550_03	AT1G68550	264	273	1	1	0.959	cgcCGGCGg

OsSK31	P$HSFA1E_01	HSFA1E	266	272	1	1	1	cCGGCG
OsSK31	P$ERF1_Q2	ERF1	268	276	1	1	0.929	GGCGGcca
OsSK31	P$DREB2C_02	DREB2C	268	278	1	1	0.855	ggCGGCCacg
OsSK31	P$RRTF1_05	RRTF1	283	298	1	1	0.864	tcgtccaCGGCGtcg
OsSK31	P$AT1G28160_02	AT1G28160	287	302	1	1	0.866	ccaCGGCGtcggcaa
OsSK31	P$AT1G68550_03	AT1G68550	287	296	1	1	0.96	ccaCGGCGt
OsSK31	P$HSFA1E_01	HSFA1E	289	295	1	1	0.87	aCGGCG
OsSK31	P$DREB1A_04	DREB1A	292	302	1	1	0.969	gcGTCGGcaa
OsSK31	P$ERF039_01	ERF039	292	302	1	1	0.982	gcGTCGGcaa
OsSK31	P$PHYPA28324_10	HYPA28324	293	301	1	1	0.879	cGTCGGca
OsSK31	P$AT1G53910_02	AT1G53910	301	322	1	1	0.855	aggcagcggccGCGGCgagag
OsSK31	P$DREB2C_02	DREB2C	305	315	1	1	0.95	agCGGCCgcg
OsSK31	P$AT1G53910_01	AT1G53910	307	317	1	1	0.905	cGGCCGcggc
OsSK31	P$DREBI5_01	DREBI5	307	317	1	1	0.884	cgGCCGCggc
OsSK31	P$AT2G47520_01	AT2G47520	307	317	1	1	0.866	cgGCCGCggc
OsSK31	P$ATERF14_01	ATERF14	307	317	1	1	0.868	cgGCCGCggc
OsSK31	P$ERF1_02	ERF1	307	317	1	1	0.856	cgGCCGCggc
OsSK31	P$DREBII1_01	DREBII1	307	317	1	1	0.882	cgGCCGCggc
OsSK31	P$CBF16_01	CBF16	307	317	1	1	0.877	cgGCCGCggc
OsSK31	P$CBF17_01	CBF17	307	317	1	1	0.884	cgGCCGCggc
OsSK31	P$CBF_01	CBF	307	317	1	1	0.887	cgGCCGCggc
OsSK31	P$AT3G61630_01	AT3G61630	307	317	1	1	0.863	cgGCCGCggc
OsSK31	P$AT5G43410_01	AT5G43410	307	317	1	1	0.873	cgGCCGCggc
OsSK31	P$CRF2_01	CRF2	307	315	1	1	0.89	cgGCCGCg
OsSK31	P$ERF098_01	ERF098	307	315	1	1	0.893	cgGCCGCg
OsSK31	P$ERF7_02	ERF7	308	318	1	1	0.931	gGCCGCggcg
OsSK31	P$ICE1_01	ICE1	308	321	1	1	0.869	ggCCGCGgcgaga
OsSK31	P$AT1G68550_03	AT1G68550	310	319	1	1	0.989	ccgCGGCGa
OsSK31	P$WRKY11_Q2	WRKY11	324	332	1	1	0.972	cTTGACcg
OsSK31	P$ZAP1_01	ZAP1	325	335	1	1	0.93	TTGACcggac
OsSK31	P$AT1G68550_03	AT1G68550	337	346	1	1	0.989	cagCGGCGa
OsSK31	P$GATA9_01	GATA9	346	357	1	1	0.99	acaAGATCtag
OsSK31	P$AGP1_01	AGP1	347	357	1	1	0.987	caAGATCtag
OsSK31	P$GATA10_01	GATA10	348	356	1	1	0.918	aAGATCta
OsSK31	P$GATA11_01	GATA11	348	356	1	1	0.944	aaGATCTa
OsSK31	P$GATA8_01	GATA8	348	357	1	1	1	aaGATCTag
OsSK31	P$ARR10_01	ARR10	349	356	1	1	0.913	AGATCta
OsSK31	P$ATHSFA1D_01	ATHSFA1D	381	387	1	1	1	aCTACA
OsSK31	P$HSFA2_01	HSFA2	411	417	1	1	1	CCAAAa
OsSK31	P$ATHB6_01	ATHB6	415	424	1	1	0.973	aaAATAAtc
OsSK31	P$ATHB5_04	ATHB5	415	426	1	1	0.886	aaAATAAtcca
OsSK31	P$ATHB1_03	ATHB1	415	426	1	1	0.893	aaAATAAtcca
OsSK31	P$ATHB16_01	ATHB16	416	424	1	1	0.86	aAATAAtc
OsSK31	P$HSFA2_01	HSFA2	423	429	1	1	1	CCAAAa
OsSK31	P$SHP2_01	SHP2	438	449	1	1	0.863	ttgaaTTTAAt
OsSK31	P$AT1G77950_01	AT1G77950	438	449	1	1	0.87	ttgaaTTTAAt
OsSK31	P$SBF1_01	SBF1	438	452	1	1	0.879	ttgaatTTAATttt
OsSK31	P$EDT1_01	EDT1	441	451	1	1	0.891	aatTTAATtt
OsSK31	P$AT2G40210_01	AT2G40210	441	457	1	1	0.913	aatttaatTTTAAagt
OsSK31	P$AT2G26880_01	AT2G26880	442	456	1	1	0.944	atttaatTTTAAag
OsSK31	P$AGL65_01	AGL65	443	455	1	1	0.852	tttaatTTTAAa
OsSK31	P$SHP2_01	SHP2	444	455	1	1	0.877	ttaatTTTAAa
OsSK31	P$AT1G77950_01	AT1G77950	444	455	1	1	0.942	ttaatTTTAAa
OsSK31	P$DOF1_01	DOF1	448	459	1	1	0.973	tttTAAAGtta
OsSK31	P$AT2G26320_01	AT2G26320	450	461	1	1	0.921	TTAAAgttaat
OsSK31	P$SBF1_01	SBF1	450	464	1	1	0.917	ttaaagTTAATttg
OsSK31	P$EDT1_01	EDT1	453	463	1	1	0.861	aagTTAATtt
OsSK31	P$ATHB6_01	ATHB6	462	471	1	1	0.902	tgAATAAgc
OsSK31	P$AT2G40210_01	AT2G40210	468	484	1	1	0.854	agctaaaaTTTAAatt
OsSK31	P$AT2G26880_01	AT2G26880	469	483	1	1	0.861	gctaaaaTTTAAat
OsSK31	P$SHP2_01	SHP2	471	482	1	1	0.864	taaaaTTTAAa
OsSK31	P$AT1G77950_01	AT1G77950	471	482	1	1	0.855	taaaaTTTAAa
OsSK31	P$SBF1_01	SBF1	471	485	1	1	0.862	taaaatTTAAAttt
OsSK31	P$SBF1_01	SBF1	509	523	1	1	0.925	ttgaagTTAATttt
OsSK31	P$EDT1_01	EDT1	512	522	1	1	0.861	aagTTAATtt
OsSK31	P$CBNAC_01	CBNAC	526	532	1	1	0.968	aTGCTT
OsSK31	P$CBNAC_02	CBNAC	526	542	1	1	0.9	aTGCTTctaacataat
OsSK31	P$MYBAS1_01	MYBAS1	530	541	1	1	0.947	ttCTAACataa
OsSK31	P$GT1_Q6_01	GT1	541	553	1	1	0.873	TTTTTttttgtt
OsSK31	P$GT1_Q6_01	GT1	544	556	1	1	0.968	TTTTTtgttaat
OsSK31	P$GT1_Q6_01	GT1	545	557	1	1	0.879	TTTTTgttaatg
OsSK31	P$SBF1_01	SBF1	545	559	1	1	0.91	tttttgTTAATgat
OsSK31	P$EDT1_01	EDT1	548	558	1	1	0.911	ttgTTAATga
OsSK31	P$HAHB4_01	HAHB4	552	561	1	1	0.965	tAATGAtgg
OsSK31	P$PDF2_01	DF2	571	582	1	1	0.88	gctaTAAATaa
OsSK31	P$ATHB6_01	ATHB6	575	584	1	1	0.909	taAATAAat
OsSK31	P$PBF_01	BF	585	596	1	1	0.961	tatAAAAGttt
OsSK31	P$DOF_Q2	DOF	585	596	1	1	0.929	tatAAAAGttt

OsSK31	P$CDF2_01	CDF2	586	596	1	1	0.968	atAAAAGttt
OsSK31	P$CDF3_01	CDF3	587	596	1	1	0.984	tAAAAGttt
OsSK31	P$SBF1_01	SBF1	600	614	1	1	0.865	tataaaTTAATttt
OsSK31	P$EDT1_01	EDT1	603	613	1	1	0.898	aaaTTAATtt
OsSK31	P$SBF1_01	SBF1	614	628	1	1	0.921	tattttTTAATaaa
OsSK31	P$GT1_Q6_01	GT1	616	628	1	1	0.899	TTTTTtaataaa
OsSK31	P$GT1_Q6_01	GT1	617	629	1	1	0.886	TTTTTaataaat
OsSK31	P$ATHB6_01	ATHB6	620	629	1	1	0.914	ttAATAAat
OsSK31	P$ATHB6_01	ATHB6	624	633	1	1	0.905	taAATAAaa
OsSK31	P$GAMYB_01	GAMYB	637	645	1	1	0.926	CAACCgat
OsSK31	P$P_01		649	658	1	1	0.953	gcCTACCac
OsSK31	P$AT3G60580_01	AT3G60580	677	684	1	1	0.873	ccATCCC
OsSK31	P$ZML1_01	ZML1	710	725	1	1	0.864	tactCATCAtcatca
OsSK31	P$PHV_02	HV	712	727	1	1	0.854	ctcATCATcatcact
OsSK31	P$PHV_02	HV	715	730	1	1	0.855	atcATCATcactcgt
OsSK31	P$DREB1A_04	DREB1A	726	736	1	1	0.931	tcGTCGGaca
OsSK31	P$ERF039_01	ERF039	726	736	1	1	0.944	tcGTCGGaca
OsSK31	P$LEC2_01	LEC2	732	743	1	1	0.933	gaCATGCtacg
OsSK31	P$PIL5_01	IL5	732	746	1	1	0.865	gacatgctACGTGc
OsSK31	P$ABZ1_01	ABZ1	735	749	1	1	0.877	atgctACGTGccgt
OsSK31	P$GBF1_Q2_01	GBF1	736	747	1	1	0.92	tgctACGTGcc
OsSK31	P$HBP1A_Q2	HBP1A	737	747	1	1	0.928	gctACGTGcc
OsSK31	P$TAF1_Q2	TAF1	737	747	1	1	0.936	gctACGTGcc
OsSK31	P$TAF1_01	TAF1	737	747	1	1	0.952	gctACGTGcc
OsSK31	P$HY5_01	HY5	738	748	1	1	0.858	ctACGTGccg
OsSK31	P$GBF1_01	GBF1	738	746	1	1	0.918	ctACGTGc
OsSK31	P$MYC4_01	MYC4	738	746	1	1	0.852	ctACGTGc
OsSK31	P$BIM1_02	BIM1	738	748	1	1	0.949	ctACGTGccg
OsSK31	P$ABF4_02	ABF4	738	748	1	1	0.957	ctACGTGccg
OsSK31	P$ABI5_Q2	ABI5	740	746	1	1	0.936	ACGTGc
OsSK31	P$BZR1_01	BZR1	741	747	1	1	0.897	CGTGCc
OsSK31	P$ERF019_01	ERF019	742	752	1	1	0.855	gTGCCGtgct
OsSK31	P$CBF1_03	CBF1	742	752	1	1	0.865	gTGCCGtgct
OsSK31	P$BZR1_01	BZR1	746	752	1	1	0.902	CGTGCt
OsSK31	P$CBNAC_01	CBNAC	747	753	1	1	0.979	gTGCTT
OsSK31	P$CBNAC_02	CBNAC	747	763	1	1	0.866	gTGCTTgctctcgccg
OsSK31	P$ERF73_01	ERF73	751	772	1	1	0.852	ttgctctCGCCGctgtcacca
OsSK31	P$ERF112_02	ERF112	756	766	1	1	0.924	ctCGCCGctg
OsSK31	P$CRF4_01	CRF4	757	765	1	1	0.907	tCGCCGct
OsSK31	P$ERF4_04	ERF4	757	765	1	1	0.892	tCGCCGct
OsSK31	P$ERF069_01	ERF069	757	766	1	1	0.994	tCGCCGctg
OsSK31	P$ERF11_01	ERF11	757	767	1	1	0.969	tCGCCGctgt
OsSK31	P$ABI4_03	ABI4	757	767	1	1	0.85	tcGCCGCtgt
OsSK31	P$DREBI5_01	DREBI5	757	767	1	1	0.884	tcGCCGCtgt
OsSK31	P$AT1G77200_01	AT1G77200	757	767	1	1	0.913	tcGCCGCtgt
OsSK31	P$ATERF14_01	ATERF14	757	767	1	1	0.85	tcGCCGCtgt
OsSK31	P$DREBIII3_01	DREBIII3	757	767	1	1	0.896	tcGCCGCtgt
OsSK31	P$DREBIII2_01	DREBIII2	757	767	1	1	0.894	tcGCCGCtgt
OsSK31	P$ERF4_02	ERF4	757	767	1	1	0.867	tcGCCGCtgt
OsSK31	P$DREBIII1_01	DREBIII1	757	767	1	1	0.896	tcGCCGCtgt
OsSK31	P$DREBII1_01	DREBII1	757	767	1	1	0.861	tcGCCGCtgt
OsSK31	P$AT2G44940_01	AT2G44940	757	767	1	1	0.885	tcGCCGCtgt
OsSK31	P$DBF2_01	DBF2	757	767	1	1	0.929	tcGCCGCtgt
OsSK31	P$CBF16_01	CBF16	757	767	1	1	0.858	tcGCCGCtgt
OsSK31	P$CBF17_01	CBF17	757	767	1	1	0.884	tcGCCGCtgt
OsSK31	P$CBF_01	CBF	757	767	1	1	0.886	tcGCCGCtgt
OsSK31	P$AT3G61630_01	AT3G61630	757	767	1	1	0.857	tcGCCGCtgt
OsSK31	P$AT5G43410_01	AT5G43410	757	767	1	1	0.856	tcGCCGCtgt
OsSK31	P$TINY2_02	TINY2	757	767	1	1	0.934	tcGCCGCtgt
OsSK31	P$AT3G16280_01	AT3G16280	757	767	1	1	0.924	tcGCCGCtgt
OsSK31	P$CRF2_01	CRF2	757	765	1	1	0.941	tcGCCGCt
OsSK31	P$ERF098_01	ERF098	757	765	1	1	0.894	tcGCCGCt
OsSK31	P$ERF8_01	ERF8	758	768	1	1	0.951	CGCCGctgtc
OsSK31	P$ERF7_02	ERF7	758	768	1	1	0.948	cGCCGCtgtc
OsSK31	P$O2_Q4	O2	766	777	1	1	0.907	tcacCATGTca
OsSK31	P$ATHB7_01	ATHB7	791	801	1	1	0.95	ccAATCAacg
OsSK31	P$HAT1_01	HAT1	791	801	1	1	0.883	ccAATCAacg
OsSK31	P$RRTF1_05	RRTF1	792	807	1	1	0.903	caatcaaCGGCGgtg
OsSK31	P$MYB76_01	MYB76	793	806	1	1	0.892	aatcAACGGcggt
OsSK31	P$AT4G27900_01	AT4G27900	793	815	1	1	0.863	aatcAACGGcggtgactgtcgt
OsSK31	P$AT1G69560_01	AT1G69560	794	806	1	1	0.891	atcAACGGcggt
OsSK31	P$AT5G54070_01	AT5G54070	795	801	1	1	0.91	tCAACG
OsSK31	P$AT1G28160_02	AT1G28160	796	811	1	1	0.933	caaCGGCGgtgactg
OsSK31	P$RAP26_06	RAP26	796	811	1	1	0.909	caaCGGCGgtgactg
OsSK31	P$AT1G68550_03	AT1G68550	796	805	1	1	0.966	caaCGGCGg
OsSK31	P$HSFA1E_01	HSFA1E	798	804	1	1	0.87	aCGGCG
OsSK31	P$RAP21_02	RAP21	798	811	1	1	0.926	acggCGGTGactg
OsSK31	P$ARF8_01	ARF8	807	816	1	1	0.953	acTGTCGtc

OsSK31	P$ATHB4_02	ATHB4	834	844	1	1	0.876	gcATCATtgg
OsSK31	P$SEP3_01	wrz-03	842	853	1	1	0.852	ggcatTTTTGc
OsSK31	P$AT3G63350_01	AT3G63350	860	866	1	1	0.867	CCGCCt
OsSK31	P$ATHB9_01	ATHB9	898	917	1	1	0.961	tcccgtaATGATtgccggt
OsSK31	P$HAHB4_01	HAHB4	903	912	1	1	1	tAATGAttg
OsSK31	P$CBF1_01	CBF1	909	919	1	1	0.861	tTGCCGgtcg
OsSK31	P$ERF019_01	ERF019	909	919	1	1	0.936	tTGCCGgtcg
OsSK31	P$DREBIII4_01	DREBIII4	909	919	1	1	0.907	tTGCCGgtcg
OsSK31	P$JERF3_01	JERF3	909	919	1	1	0.853	tTGCCGgtcg
OsSK31	P$CEF1_01	CEF1	909	919	1	1	0.853	tTGCCGgtcg
OsSK31	P$JERF1_01	JERF1	909	919	1	1	0.876	tTGCCGgtcg
OsSK31	P$CBF1_03	CBF1	909	919	1	1	0.884	tTGCCGgtcg
OsSK31	P$AT1G33760_01	AT1G33760	909	919	1	1	0.885	tTGCCGgtcg
OsSK31	P$AT1G71520_01	AT1G71520	909	919	1	1	0.893	tTGCCGgtcg
OsSK31	P$ORA47_01	ORA47	909	919	1	1	0.9	tTGCCGgtcg
OsSK31	P$KNOX3_01	KNOX3	932	944	1	1	0.968	gcctTGACAtga
OsSK31	P$WRKY11_Q2	WRKY11	934	942	1	1	0.901	cTTGACat
OsSK31	P$ATH1_01	ATH1	936	944	1	1	0.946	TGACAtga
OsSK31	P$AT5G54070_01	AT5G54070	947	953	1	1	0.91	tCAACG
OsSK31	P$ASR1_01	ASR1	967	972	1	1	1	ACCCA
OsSK31	P$GATA15_01	GATA15	970	979	1	1	0.999	caTGATCcg
OsSK31	P$BZIP68_01	BZIP68	974	983	1	1	0.935	atcCGTGGa
OsSK31	P$ATHB6_01	ATHB6	984	993	1	1	0.975	cgAATAAtt
OsSK31	P$ATHB5_04	ATHB5	984	995	1	1	0.959	cgAATAAttga
OsSK31	P$ATHB1_03	ATHB1	984	995	1	1	0.978	cgAATAAttga
OsSK31	P$ATHB16_01	ATHB16	985	993	1	1	0.947	gAATAAtt
OsSK31	P$SED_Q2	SED	1014	1024	1	1	0.989	ctggCCTTTt
OsSK31	P$PBF_Q2_01	BF	1018	1024	1	1	1	CCTTTt
OsSK31	P$MYBAS1_01	MYBAS1	1034	1045	1	1	0.988	tcCCAACtccc
OsSK31	P$GT1_Q6	GT1	1047	1054	1	1	1	GTGAAta
OsSK31	P$ATMYB77_01	ATMYB77	1054	1067	1	1	0.9	taaggtCGGTTgt
OsSK31	P$DREB1A_04	DREB1A	1056	1066	1	1	0.981	agGTCGGttg
OsSK31	P$ERF039_01	ERF039	1056	1066	1	1	0.988	agGTCGGttg
OsSK31	P$PHYPA182268_05	HYPA182268	1056	1066	1	1	0.941	agGTCGGttg
OsSK31	P$PHYPA64121_06	HYPA64121	1056	1069	1	1	0.864	aggTCGGTtgtga
OsSK31	P$ERF043_01	ERF043	1057	1065	1	1	0.865	gGTCGGtt
OsSK31	P$PHYPA173530_04	HYPA173530	1057	1065	1	1	0.948	gGTCGGtt
OsSK31	P$PHYPA28324_10	HYPA28324	1057	1065	1	1	0.951	gGTCGGtt
OsSK31	P$KNOX3_01	KNOX3	1062	1074	1	1	0.982	gttgTGACAtat
OsSK31	P$ATH1_01	ATH1	1066	1074	1	1	0.935	TGACAtat
OsSK31	P$PDF2_01	DF2	1080	1091	1	1	0.86	actaTAAATat
OsSK31	P$SPF1_Q2	SPF1	1119	1129	1	1	0.936	ttATAGTatt
OsSK31	P$TEIL_01	TEIL	1132	1140	1	1	0.863	ATGTAtca
OsSK31	P$SPL11_01	SPL11	1168	1180	1	1	0.883	tggaGTACGagg
OsSK31	P$SPL5_01	SPL5	1170	1179	1	1	0.971	gaGTACGag
OsSK31	P$POPTR_01	OPTR	1171	1178	1	1	0.943	aGTACGa
OsSK31	P$SPL12_01	SPL12	1171	1179	1	1	0.981	aGTACGag
OsSK31	P$SPL4_01	SPL4	1171	1180	1	1	0.994	aGTACGagg
OsSK31	P$GATA9_01	GATA9	1177	1188	1	1	0.88	aggAGATCatc
OsSK31	P$AGP1_01	AGP1	1178	1188	1	1	0.852	ggAGATCatc
OsSK31	P$ARR10_01	ARR10	1180	1187	1	1	0.913	AGATCat
OsSK31	P$UIF1_01	UIF1	1203	1213	1	1	0.859	catGATTCtt
OsSK31	P$MYB24_01	MYB24	1216	1225	1	1	0.899	agcTTAGGc
OsSK31	P$AT5G04240_01	AT5G04240	1221	1227	1	1	0.939	aGGCAC
OsSK31	P$BPC1_Q2	BPC1	1228	1234	1	1	0.997	AGAAAa
OsSK31	P$AT1G68550_03	AT1G68550	1243	1252	1	1	0.949	gccCGGCGa
OsSK31	P$HSFA1E_01	HSFA1E	1245	1251	1	1	1	cCGGCG
OsSK31	P$ATHB6_01	ATHB6	1257	1266	1	1	0.9	aaAATAAca
OsSK31	P$GATA15_01	GATA15	1265	1274	1	1	0.999	atTGATCga
OsSK31	P$SEP3_01	wrz-03	1270	1281	1	1	0.876	tcgagTTTTGg
OsSK31	P$ANTL_01	ANTL	1296	1306	1	1	0.862	tGTTACcgcg
OsSK31	P$MYB89_01	MYB89	1297	1308	1	1	0.896	gtTACCGcggg
OsSK31	P$E2L_Q2_01	E2L	1299	1313	1	1	0.944	taccgCGGGAaaaa
OsSK31	P$AT3G51080_01	AT3G51080	1306	1313	1	1	1	GGAAAaa
OsSK31	P$ATHB6_01	ATHB6	1311	1320	1	1	0.905	aaAATAAaa
OsSK31	P$TGA1_02	TGA1	1315	1330	1	1	0.863	taaaatcACGTCagt
OsSK31	P$HAT1_01	HAT1	1316	1326	1	1	0.856	aaAATCAcgt
OsSK31	P$ABF2_01	ABF2	1316	1329	1	1	0.89	aaaatCACGTcag
OsSK31	P$O2_Q4	O2	1317	1328	1	1	0.937	aaatCACGTca
OsSK31	P$TGA7_02	TGA7	1317	1334	1	1	0.879	aaatcACGTCagtgatt
OsSK31	P$GBP_Q6	GBP	1318	1330	1	1	0.897	aatCACGTcagt
OsSK31	P$ABI5_01	ABI5	1318	1328	1	1	0.89	aatCACGTca
OsSK31	P$ABF4_01	ABF4	1318	1330	1	1	0.873	aatCACGTcagt
OsSK31	P$CPRF3_Q2	CPRF3	1319	1329	1	1	0.963	atCACGTcag
OsSK31	P$CPRF2_Q2	CPRF2	1319	1329	1	1	0.968	atCACGTcag
OsSK31	P$O2_02	O2	1319	1329	1	1	0.958	atCACGTcag
OsSK31	P$TGA1B_Q2	TGA1B	1319	1329	1	1	0.95	atCACGTcag
OsSK31	P$TGA1A_Q2	TGA1A	1319	1329	1	1	0.992	atCACGTcag

OsSK31	P$CPRF3_01	CPRF3	1319	1329	1	1	0.981	atCACGTcag
OsSK31	P$CPRF2_01	CPRF2	1319	1329	1	1	0.969	atCACGTcag
OsSK31	P$TGA1B_01	TGA1B	1319	1329	1	1	0.906	atCACGTcag
OsSK31	P$BEE2_01	BEE2	1319	1329	1	1	0.906	atCACGTcag
OsSK31	P$BIM3_01	BIM3	1319	1329	1	1	0.877	atCACGTcag
OsSK31	P$PHYPA143875_02	HYPA143875	1319	1329	1	1	0.871	atCACGTcag
OsSK31	P$SPT_01	SPT	1319	1328	1	1	0.921	atCACGTca
OsSK31	P$HBPA1_Q6_01	HBPA1	1319	1329	1	1	0.916	atcaCGTCAg
OsSK31	P$RITA1_01	RITA1	1320	1327	1	1	0.98	tCACGTc
OsSK31	P$OCSBF1_01	OCSBF1	1321	1326	1	1	1	CACGT
OsSK31	P$TGA1A_01	TGA1A	1321	1328	1	1	0.989	cACGTCa
OsSK31	P$HSFA2_01	HSFA2	1342	1348	1	1	0.941	CCAAAc
OsSK31	P$MYBAS1_01	MYBAS1	1359	1370	1	1	0.952	cgCTAACaaaa
OsSK31	P$ATMYB15_Q2	ATMYB15	1362	1368	1	1	1	TAACAa
OsSK31	P$GATA15_01	GATA15	1408	1417	1	1	0.999	acTGATCga
OsSK31	P$AT3G01030_01	AT3G01030	1418	1427	1	1	0.926	ACCGCgaga
OsSK31	P$SBF1_01	SBF1	1423	1437	1	1	0.894	gagattTTAATata
OsSK31	P$SPF1_Q2	SPF1	1434	1444	1	1	0.894	atATAGTgaa
OsSK31	P$GT1_Q6	GT1	1439	1446	1	1	0.971	GTGAAaa
OsSK31	P$ATHB7_01	ATHB7	1486	1496	1	1	0.873	aaAATCAaac
OsSK31	P$HAT1_01	HAT1	1486	1496	1	1	0.864	aaAATCAaac
OsSK31	P$PDF2_01	DF2	1494	1505	1	1	0.854	acgaTAAATtt
OsSK31	P$HSFA2_01	HSFA2	1507	1513	1	1	0.922	CCAAAt
OsSK31	P$ATHB7_01	ATHB7	1514	1524	1	1	0.884	ttAATCAaag
OsSK31	P$HAT1_01	HAT1	1514	1524	1	1	0.875	ttAATCAaag
OsSK31	P$MYBPH3_02	MYBPH3	1519	1532	1	1	0.989	caaagtTAGTTaa
OsSK31	P$WEREWOLF_Q2	WEREWOLF	1521	1530	1	1	0.919	aaGTTAGtt
OsSK31	P$SBF1_01	SBF1	1522	1536	1	1	0.926	agttagTTAAAat
OsSK31	P$TGA1_01	TGA1	1533	1544	1	1	0.92	aatTGACGaaa
OsSK31	P$WRKY11_Q2	WRKY11	1534	1542	1	1	0.924	aTTGACga
OsSK31	P$TGA7_01	TGA7	1534	1544	1	1	0.872	atTGACGaaa
OsSK31	P$TGA5_01	TGA5	1535	1543	1	1	0.856	tTGACGaa
OsSK31	P$AT2G41690_01	AT2G41690	1557	1563	1	1	0.988	CCGAAg
OsSK31	P$GATA9_01	GATA9	1563	1574	1	1	0.986	ttaAGATCtga
OsSK31	P$AGP1_01	AGP1	1564	1574	1	1	0.946	taAGATCtga
OsSK31	P$GATA10_01	GATA10	1565	1573	1	1	0.964	aAGATCtg
OsSK31	P$GATA11_01	GATA11	1565	1573	1	1	0.98	aaGATCTg
OsSK31	P$GATA8_01	GATA8	1565	1574	1	1	0.999	aaGATCTga
OsSK31	P$ARR10_01	ARR10	1566	1573	1	1	0.978	AGATCtg
OsSK31	P$AMS_01	AMS	1583	1593	1	1	0.868	caCATGTgca
OsSK31	P$SPF1_Q2	SPF1	1600	1610	1	1	0.881	ttATAGTgta
OsSK31	P$ATSPL8_01	ATSPL8	1601	1617	1	1	0.936	tatagTGTACcgttct
OsSK31	P$MYB3R5_01	MYB3R5	1601	1616	1	1	0.895	tatagtgtaCCGTTc
OsSK31	P$MYB3R1_01	MYB3R1	1602	1617	1	1	0.909	atagtgtaCCGTTct
OsSK31	P$MYB3R4_01	MYB3R4	1602	1617	1	1	0.892	atagtgtaCCGTTct
OsSK31	P$AT3G51080_01	AT3G51080	1617	1624	1	1	0.89	GGAAAac
OsSK31	P$AT4G36620_01	AT4G36620	1618	1626	1	1	0.896	gaaAACCA
OsSK31	P$MYBAS1_01	MYBAS1	1621	1632	1	1	0.987	aaCCAACttct
OsSK31	P$ATHSFA1D_01	ATHSFA1D	1629	1635	1	1	0.941	tCTACA
OsSK31	P$O2_03	O2	1635	1645	1	1	0.93	GATGAattgg
OsSK31	P$GAMYB_Q2	GAMYB	1640	1653	1	1	0.916	attggACAACtga
OsSK31	P$HSFA2_01	HSFA2	1656	1662	1	1	0.922	CCAAAt
OsSK31	P$ATHB1_01	ATHB1	1668	1682	1	1	0.94	tcggaATTATtatt
OsSK31	P$ATHB5_01	ATHB5	1671	1680	1	1	0.919	gaaTTATTa
OsSK31	P$GAMYB_Q2	GAMYB	1681	1694	1	1	0.894	tttttACAACggg
OsSK31	P$AT5G54070_01	AT5G54070	1686	1692	1	1	0.958	aCAACG
OsSK31	P$HSF3_01	HSF3	1689	1695	1	1	0.969	aCGGGG
OsSK31	P$PIL5_01	IL5	1691	1705	1	1	0.853	gggggtatACGTGt
OsSK31	P$ABF4_Q2	ABF4	1693	1707	1	1	0.903	gggtatACGTGtgg
OsSK31	P$ABZ1_01	ABZ1	1694	1708	1	1	0.939	ggtatACGTGtggc
OsSK31	P$HBP1A_Q2	HBP1A	1696	1706	1	1	0.863	tatACGTGtg
OsSK31	P$TAF1_Q2	TAF1	1696	1706	1	1	0.916	tatACGTGtg
OsSK31	P$EMBP1_02	EMBP1	1696	1706	1	1	0.901	tatACGTGtg
OsSK31	P$TAF1_01	TAF1	1696	1706	1	1	0.941	tatACGTGtg
OsSK31	P$TRAB1_Q2	TRAB1	1697	1708	1	1	0.986	atACGTGtggc
OsSK31	P$GBF1_01	GBF1	1697	1705	1	1	0.973	atACGTGt
OsSK31	P$BIM1_02	BIM1	1697	1707	1	1	0.946	atACGTGtgg
OsSK31	P$ABF4_02	ABF4	1697	1707	1	1	0.985	atACGTGtgg
OsSK31	P$ABI5_Q2	ABI5	1699	1705	1	1	0.979	ACGTGt
OsSK31	P$PIL5_01	IL5	1721	1735	1	1	0.954	gtatatgcACGTGt
OsSK31	P$PIF3_01	IF3	1722	1740	1	1	0.855	tatatgCACGTgtgtctt
OsSK31	P$ABF2_01	ABF2	1723	1736	1	1	0.944	atatgCACGTgtg
OsSK31	P$ABF4_Q2	ABF4	1723	1737	1	1	0.909	atatgcACGTGtgt
OsSK31	P$O2_Q4	O2	1724	1735	1	1	0.877	tatgCACGTgt
OsSK31	P$BZR1_02	BZR1	1724	1738	1	1	0.963	tatgCACGTgtgtc
OsSK31	P$HBI1_01	HBI1	1724	1736	1	1	0.993	tatgCACGTgtg
OsSK31	P$ABZ1_01	ABZ1	1724	1738	1	1	0.907	tatgcACGTGtgtc
OsSK31	P$GBP_Q6	GBP	1725	1737	1	1	0.9	atgCACGTgtgt

OsSK31	P$PIF3_03	IF3	1725	1735	1	1	0.945	atgCACGTgt
OsSK31	P$ABI5_01	ABI5	1725	1735	1	1	0.931	atgCACGTgt
OsSK31	P$ABF4_01	ABF4	1725	1737	1	1	0.949	atgCACGTgtgt
OsSK31	P$BZR1_03	BZR1	1725	1745	1	1	0.9	atgcACGTGtgtcttcctgt
OsSK31	P$TSAR2_01	TSAR2	1726	1736	1	1	0.87	tGCACGtgtg
OsSK31	P$EMBP1_Q2	EMBP1	1726	1736	1	1	0.89	tgCACGTgtg
OsSK31	P$CPRF_Q2	CPRF	1726	1736	1	1	0.923	tgCACGTgtg
OsSK31	P$CPRF3_Q2	CPRF3	1726	1736	1	1	0.969	tgCACGTgtg
OsSK31	P$CPRF2_Q2	CPRF2	1726	1736	1	1	0.982	tgCACGTgtg
OsSK31	P$O2_02	O2	1726	1736	1	1	0.949	tgCACGTgtg
OsSK31	P$TGA1B_Q2	TGA1B	1726	1736	1	1	0.894	tgCACGTgtg
OsSK31	P$TGA1A_Q2	TGA1A	1726	1736	1	1	0.981	tgCACGTgtg
OsSK31	P$CPRF1_01	CPRF1	1726	1736	1	1	0.941	tgCACGTgtg
OsSK31	P$CPRF3_01	CPRF3	1726	1736	1	1	0.974	tgCACGTgtg
OsSK31	P$CPRF2_01	CPRF2	1726	1736	1	1	0.983	tgCACGTgtg
OsSK31	P$TGA1B_01	TGA1B	1726	1736	1	1	0.863	tgCACGTgtg
OsSK31	P$BES1_01	BES1	1726	1737	1	1	0.986	tgCACGTgtgt
OsSK31	P$BEE2_01	BEE2	1726	1736	1	1	0.999	tgCACGTgtg
OsSK31	P$BIM2_01	BIM2	1726	1736	1	1	0.997	tgCACGTgtg
OsSK31	P$BIM3_01	BIM3	1726	1736	1	1	0.995	tgCACGTgtg
OsSK31	P$PHYPA143875_02	HYPA143875	1726	1736	1	1	0.998	tgCACGTgtg
OsSK31	P$PHYPA72483_07	HYPA72483	1726	1736	1	1	0.998	tgCACGTgtg
OsSK31	P$SPT_01	SPT	1726	1735	1	1	0.98	tgCACGTgt
OsSK31	P$GBF1F_Q2	GBF1F	1726	1737	1	1	0.863	tgCACGTgtgt
OsSK31	P$TSAR1_01	TSAR1	1726	1736	1	1	0.89	tgCACGTgtg
OsSK31	P$HBP1A_Q2	HBP1A	1726	1736	1	1	0.905	tgcACGTGtg
OsSK31	P$TAF1_Q2	TAF1	1726	1736	1	1	0.95	tgcACGTGtg
OsSK31	P$EMBP1_02	EMBP1	1726	1736	1	1	0.871	tgcACGTGtg
OsSK31	P$TAF1_01	TAF1	1726	1736	1	1	0.968	tgcACGTGtg
OsSK31	P$PIF1_01	IF1	1726	1736	1	1	0.977	tgcACGTGtg
OsSK31	P$BHLH78_01	BHLH78	1727	1735	1	1	0.875	GCACGtgt
OsSK31	P$RITA1_01	RITA1	1727	1734	1	1	0.974	gCACGTg
OsSK31	P$BHLH66_01	BHLH66	1727	1735	1	1	0.962	gCACGTgt
OsSK31	P$PIF5_01	IF5	1727	1735	1	1	0.904	gCACGTgt
OsSK31	P$MYC2_01	MYC2	1727	1735	1	1	0.981	gCACGTgt
OsSK31	P$MYC3_01	MYC3	1727	1735	1	1	0.997	gCACGTgt
OsSK31	P$BHLH34_01	BHLH34	1727	1735	1	1	0.995	gCACGTgt
OsSK31	P$PHYPA48267_08	HYPA48267	1727	1735	1	1	0.974	gCACGTgt
OsSK31	P$OJ1058_01	OJ1058	1727	1735	1	1	0.946	gCACGTgt
OsSK31	P$UNE10_01	UNE10	1727	1735	1	1	0.979	gCACGTgt
OsSK31	P$BHLH3_01	BHLH3	1727	1735	1	1	0.98	gCACGTgt
OsSK31	P$AIB_01	AIB	1727	1735	1	1	0.899	gCACGTgt
OsSK31	P$TRAB1_Q2	TRAB1	1727	1738	1	1	0.965	gcACGTGtgtc
OsSK31	P$GBF1_01	GBF1	1727	1735	1	1	0.965	gcACGTGt
OsSK31	P$MYC4_01	MYC4	1727	1735	1	1	0.96	gcACGTGt
OsSK31	P$BIM1_02	BIM1	1727	1737	1	1	0.995	gcACGTGtgt
OsSK31	P$BHLH13_01	BHLH13	1727	1735	1	1	0.968	gcACGTGt
OsSK31	P$ABF4_02	ABF4	1727	1737	1	1	0.981	gcACGTGtgt
OsSK31	P$OCSBF1_01	OCSBF1	1728	1733	1	1	1	CACGT
OsSK31	P$PIF4_01	IF4	1728	1736	1	1	0.958	CACGTgtg
OsSK31	P$ABI5_Q2	ABI5	1729	1735	1	1	0.979	ACGTGt
OsSK31	P$AT1G19490_01	AT1G19490	1752	1761	1	1	0.898	GGTTTaggc
OsSK31	P$MYB24_01	MYB24	1752	1761	1	1	0.908	ggtTTAGGc
OsSK31	P$MYBAS1_01	MYBAS1	1778	1789	1	1	0.988	ctCCAACtgat
OsSK31	P$MYB24_01	MYB24	1786	1795	1	1	0.941	gatTTAGGt
OsSK31	P$MYB131_01	MYB131	1786	1797	1	1	0.925	gatTTAGGttt
OsSK31	P$PHV_02	HV	1805	1820	1	1	0.852	tttATCATtgttcac
OsSK31	P$ATHB4_02	ATHB4	1806	1816	1	1	0.924	ttATCATtgt
OsSK31	P$PDF2_01	DF2	1823	1834	1	1	0.893	cataTAAATgt
OsSK31	P$SBF1_01	SBF1	1827	1841	1	1	0.873	taaatgTTAATgaa
OsSK31	P$EDT1_01	EDT1	1830	1840	1	1	0.91	atgTTAATga
OsSK31	P$ARR1_01	ARR1	1835	1845	1	1	0.981	aatGAATCta
OsSK31	P$UIF1_01	UIF1	1860	1870	1	1	0.975	ctaGATTCat
OsSK31	P$AT3G20750_01	AT3G20750	1894	1902	1	1	1	aTAAACat
OsSK31	P$SBF1_01	SBF1	1914	1928	1	1	0.932	tatatgTTAATata
OsSK31	P$SBF1_01	SBF1	1928	1942	1	1	0.932	tatatgTTAATata
OsSK31	P$GT1_Q6_02	GT1	1946	1958	1	1	0.991	tatataTTAACa
OsSK31	P$GT1_Q6_02	GT1	1958	1970	1	1	0.972	tatttaTTAACa
OsSK31	P$ID1_01	ID1	1971	1982	1	1	0.852	aTTGTCctgtt
OsSK31	P$BPC1_Q2	BPC1	2006	2012	1	1	0.99	AGAAAt
OsSK31	P$PEND_02	END	2009	2019	1	1	0.972	aaTTCTTata
OsSK31	P$AT3G01140_01	AT3G01140	2023	2034	1	1	0.866	gaAACGGagga
OsSK31	P$ABI3_01	ABI3	2055	2064	1	1	0.959	acGCATGca
OsSK31	P$FUS3_01	FUS3	2056	2065	1	1	0.981	cGCATGcaa
OsSK31	P$LEC2_01	LEC2	2056	2067	1	1	0.982	cgCATGCaaca
OsSK31	P$RAV1_01	RAV1	2059	2071	1	1	0.982	atgCAACAcata
OsSK31	P$EDT1_01	EDT1	2091	2101	1	1	0.891	taaTTAATtg
OsSK31	P$GT1_Q6_02	GT1	2095	2107	1	1	0.97	taattgTTAACt

OsSK31	P$SBF1_01	SBF1	2104	2118	1	1	0.887	acttttTTAAAaaa
OsSK31	P$ATHB6_01	ATHB6	2114	2123	1	1	0.979	aaAATAAtt
OsSK31	P$ATHB5_04	ATHB5	2114	2125	1	1	0.963	aaAATAAttaa
OsSK31	P$ATHB1_03	ATHB1	2114	2125	1	1	0.984	aaAATAAttaa
OsSK31	P$ATHB16_01	ATHB16	2115	2123	1	1	0.953	aAATAAtt
OsSK31	P$EDT1_01	EDT1	2118	2128	1	1	0.984	taaTTAATgt
OsSK31	P$TEIL_01	TEIL	2124	2132	1	1	0.882	ATGTAatt
OsSK31	P$AT2G26880_01	AT2G26880	2124	2138	1	1	0.853	atgtaatTTTAAag
OsSK31	P$DOF1_01	DOF1	2130	2141	1	1	0.97	tttTAAAGtat
OsSK31	P$AZF3_01	AZF3	2135	2146	1	1	0.985	aAGTATttttt
OsSK31	P$GT1_Q6_01	GT1	2140	2152	1	1	0.887	TTTTTtctaaaa
OsSK31	P$PBF_01	BF	2146	2157	1	1	0.957	ctaAAAAGatt
OsSK31	P$DOF_Q2	DOF	2146	2157	1	1	0.976	ctaAAAAGatt
OsSK31	P$CDF2_01	CDF2	2147	2157	1	1	0.973	taAAAAGatt
OsSK31	P$CDF3_01	CDF3	2148	2157	1	1	0.973	aAAAAGatt
OsSK31	P$SBF1_01	SBF1	2167	2181	1	1	0.894	tatcgtTTAATaat
OsSK31	P$ATHB6_01	ATHB6	2173	2182	1	1	0.984	ttAATAAtt
OsSK31	P$ATHB5_04	ATHB5	2173	2184	1	1	0.972	ttAATAAtttc
OsSK31	P$ATHB1_03	ATHB1	2173	2184	1	1	0.97	ttAATAAtttc
OsSK31	P$ATHB16_01	ATHB16	2174	2182	1	1	1	tAATAAtt
OsSK31	P$ATHB6_01	ATHB6	2185	2194	1	1	0.979	gaAATAAtt
OsSK31	P$ATHB5_04	ATHB5	2185	2196	1	1	0.956	gaAATAAtttg
OsSK31	P$ATHB1_03	ATHB1	2185	2196	1	1	0.968	gaAATAAtttg
OsSK31	P$ATHB16_01	ATHB16	2186	2194	1	1	0.953	aAATAAtt
OsSK31	P$O2_Q4	O2	2203	2214	1	1	0.852	tgcgCATGTaa
OsSK31	P$ABI3_01	ABI3	2204	2213	1	1	0.857	gcGCATGta
OsSK31	P$PIL5_01	IL5	2217	2231	1	1	0.868	agctgggaACGTGg
OsSK31	P$ABZ1_01	ABZ1	2220	2234	1	1	0.967	tgggaACGTGgagt
OsSK31	P$GBF1_Q2_01	GBF1	2221	2232	1	1	0.883	gggaACGTGga
OsSK31	P$HBP1A_Q2	HBP1A	2222	2232	1	1	0.896	ggaACGTGga
OsSK31	P$TAF1_Q2	TAF1	2222	2232	1	1	0.939	ggaACGTGga
OsSK31	P$EMBP1_02	EMBP1	2222	2232	1	1	0.907	ggaACGTGga
OsSK31	P$TAF1_01	TAF1	2222	2232	1	1	0.958	ggaACGTGga
OsSK31	P$PIF1_01	IF1	2222	2232	1	1	0.851	ggaACGTGga
OsSK31	P$HY5_01	HY5	2223	2233	1	1	0.952	gaACGTGgag
OsSK31	P$GBF1_01	GBF1	2223	2231	1	1	0.943	gaACGTGg
OsSK31	P$BIM1_02	BIM1	2223	2233	1	1	0.945	gaACGTGgag
OsSK31	P$ABF4_02	ABF4	2223	2233	1	1	0.978	gaACGTGgag
OsSK31	P$BZIP68_01	BZIP68	2223	2232	1	1	0.984	gaaCGTGGa
OsSK31	P$CPRF1_Q2	CPRF1	2224	2234	1	1	0.934	aACGTGgagt
OsSK31	P$ABI5_Q2	ABI5	2225	2231	1	1	1	ACGTGg
OsSK31	P$GT1_Q6	GT1	2232	2239	1	1	0.912	GTAAAca
OsSK31	P$DOF1_01	DOF1	2237	2248	1	1	0.972	cagTAAAGggg
OsSK31	P$PBF_Q2	BF	2240	2246	1	1	0.986	tAAAGG
OsSK31	P$HSF3_01	HSF3	2263	2269	1	1	1	cCGGGG
OsSK31	P$FAR1_01	FAR1	2271	2286	1	1	0.863	gcgggACGCGcggac
OsSK31	P$FHY3_01	FHY3	2273	2285	1	1	0.852	gggACGCGcgga
OsSK31	P$BBM_01	BBM	2287	2297	1	1	0.933	gGGCGCcacg
OsSK31	P$PLT1_01	LT1	2287	2297	1	1	0.902	gGGCGCcacg
OsSK31	P$E2FA_02	E2FA	2287	2297	1	1	0.99	gggCGCCAcg
OsSK31	P$FAR1_01	FAR1	2289	2304	1	1	0.968	gcgccACGCGcgcgc
OsSK31	P$FHY3_01	FHY3	2291	2303	1	1	0.96	gccACGCGcgcg
OsSK31	P$HSFA2_01	HSFA2	2317	2323	1	1	0.922	CCAAAt
OsSK31	P$AT1G53910_02	AT1G53910	2334	2355	1	1	0.87	cgagacagacaGCGGCgcgga
OsSK31	P$RRTF1_05	RRTF1	2339	2354	1	1	0.906	cagacagCGGCGcgg
OsSK31	P$AT1G28160_02	AT1G28160	2343	2358	1	1	0.864	cagCGGCGcggaact
OsSK31	P$AT1G68550_03	AT1G68550	2343	2352	1	1	0.996	cagCGGCGc
OsSK31	P$E2FA_02	E2FA	2380	2390	1	1	0.988	gagCGCCAcc
OsSK31	P$MYB305_Q3	MYB305	2382	2395	1	1	0.911	gcgccACCTAtcg
OsSK31	P$MYB61_01	MYB61	2384	2399	1	1	0.882	gccACCTAtcgccgc
OsSK31	P$RAP210_04	RAP210	2391	2401	1	1	0.852	atCGCCGctt
OsSK31	P$ERF112_02	ERF112	2391	2401	1	1	0.921	atCGCCGctt
OsSK31	P$CRF4_01	CRF4	2392	2400	1	1	0.907	tCGCCGct
OsSK31	P$ERF4_04	ERF4	2392	2400	1	1	0.892	tCGCCGct
OsSK31	P$ERF069_01	ERF069	2392	2401	1	1	0.993	tCGCCGctt
OsSK31	P$ERF11_01	ERF11	2392	2402	1	1	0.97	tCGCCGcttc
OsSK31	P$AT1G77200_01	AT1G77200	2392	2402	1	1	0.9	tcGCCGCttc
OsSK31	P$ATERF14_01	ATERF14	2392	2402	1	1	0.858	tcGCCGCttc
OsSK31	P$DREBIII3_01	DREBIII3	2392	2402	1	1	0.89	tcGCCGCttc
OsSK31	P$DREBIII2_01	DREBIII2	2392	2402	1	1	0.888	tcGCCGCttc
OsSK31	P$ERF4_02	ERF4	2392	2402	1	1	0.855	tcGCCGCttc
OsSK31	P$DREBIII1_01	DREBIII1	2392	2402	1	1	0.89	tcGCCGCttc
OsSK31	P$EREBP1_01	EREBP1	2392	2402	1	1	0.863	tcGCCGCttc
OsSK31	P$AT2G44940_01	AT2G44940	2392	2402	1	1	0.88	tcGCCGCttc
OsSK31	P$ERF2_03	ERF2	2392	2402	1	1	0.858	tcGCCGCttc
OsSK31	P$DBF2_01	DBF2	2392	2402	1	1	0.914	tcGCCGCttc
OsSK31	P$AT3G61630_01	AT3G61630	2392	2402	1	1	0.87	tcGCCGCttc
OsSK31	P$AT5G43410_01	AT5G43410	2392	2402	1	1	0.863	tcGCCGCttc

OsSK31	P$TINY2_02	TINY2	2392	2402	1	1	0.889	tcGCCGCttc
OsSK31	P$AT3G16280_01	AT3G16280	2392	2402	1	1	0.92	tcGCCGCttc
OsSK31	P$CRF2_01	CRF2	2392	2400	1	1	0.941	tcGCCGCt
OsSK31	P$ERF098_01	ERF098	2392	2400	1	1	0.894	tcGCCGCt
OsSK31	P$ERF8_01	ERF8	2393	2403	1	1	0.95	CGCCGcttcg
OsSK31	P$ERF7_02	ERF7	2393	2403	1	1	0.947	cGCCGCttcg
OsSK31	P$DREB1E_01	DREB1E	2402	2412	1	1	0.876	gGGCCGtcgg
OsSK31	P$AT1G53910_01	AT1G53910	2402	2412	1	1	0.916	gGGCCGtcgg
OsSK31	P$DREB1A_04	DREB1A	2405	2415	1	1	0.927	ccGTCGGatc
OsSK31	P$ERF039_01	ERF039	2405	2415	1	1	0.944	ccGTCGGatc
OsSK31	P$PHYPA28324_10	HYPA28324	2406	2414	1	1	0.86	cGTCGGat
OsSK31	P$ALFIN1_Q2	ALFIN1	2442	2457	1	1	0.858	gaccgtGTGGGcccc
OsSK31	P$TCP11_01	TCP11	2447	2459	1	1	0.998	tGTGGGcccctg
OsSK31	P$TCP15_01	TCP15	2448	2458	1	1	0.999	GTGGGcccct
OsSK31	P$TCP20_01	TCP20	2448	2458	1	1	0.991	GTGGGcccct
OsSK31	P$TCP11_02	TCP11	2448	2458	1	1	1	GTGGGcccct
OsSK31	P$TCP7_01	TCP7	2448	2459	1	1	0.944	GTGGGcccctg
OsSK31	P$OJ1581_01	OJ1581	2448	2458	1	1	0.992	gtGGGCCcct
OsSK31	P$TCP2_01	TCP2	2448	2458	1	1	0.974	gtGGGCCcct
OsSK31	P$ANT_01	ANT	2461	2475	1	1	0.869	cgcgggtCCCGAcg
OsSK31	P$PCF5_01	CF5	2463	2473	1	1	0.885	cgGGTCCcga
OsSK31	P$CBF3_02	CBF3	2464	2478	1	1	0.907	gggtcCCGACggtg
OsSK31	P$CBF1_04	CBF1	2465	2477	1	1	0.896	ggtcCCGACggt
OsSK31	P$ARF5_01	ARF5	2468	2476	1	1	0.887	cCCGACgg
OsSK31	P$DREB1B_01	DREB1B	2469	2474	1	1	1	CCGAC
OsSK31	P$RAP21_02	RAP21	2469	2482	1	1	0.936	ccgaCGGTGgtgg
OsSK31	P$ATHB9_01	ATHB9	2477	2496	1	1	0.871	ggtggcaATGATtaggctc
OsSK31	P$HAHB4_01	HAHB4	2482	2491	1	1	0.854	cAATGAtta
OsSK31	P$MYB24_01	MYB24	2485	2494	1	1	0.93	tgaTTAGGc
OsSK31	P$RAV1_02	RAV1	2495	2507	1	1	0.979	cgcACCTGcggg
OsSK31	P$HSF3_01	HSF3	2502	2508	1	1	0.945	gCGGGG
OsSK31	P$WRKY11_Q2	WRKY11	2509	2517	1	1	0.927	tTTGACtg
OsSK31	P$AT4G00870_01	AT4G00870	2529	2543	1	1	0.891	ctCCTCGcccatgc
OsSK31	P$LEC2_01	LEC2	2536	2547	1	1	0.95	ccCATGCccaa
OsSK31	P$HSFA2_01	HSFA2	2543	2549	1	1	1	CCAAAa
OsSK31	P$CBNAC_01	CBNAC	2562	2568	1	1	1	tTGCTT
OsSK31	P$CBNAC_02	CBNAC	2562	2578	1	1	0.9	tTGCTTttgcgctgtg
OsSK31	P$CBNAC_01	CBNAC	2618	2624	1	1	0.973	cTGCTT
OsSK31	P$MYB1L_01	MYB1L	2648	2658	1	1	0.937	ctCCCTAcac
OsSK31	P$TRB2_01	TRB2	2648	2656	1	1	0.92	ctCCCTAc
OsSK31	P$ATHSFA1D_01	ATHSFA1D	2651	2657	1	1	0.985	cCTACA
OsSK31	P$KNOX3_01	KNOX3	2655	2667	1	1	0.966	cacaTGACAatc
OsSK31	P$ATH1_01	ATH1	2659	2667	1	1	0.906	TGACAatc
OsSK31	P$REF6_01	REF6	2694	2705	1	1	0.968	agaaCAGAGga
OsSK31	P$SPF1_Q2	SPF1	2704	2714	1	1	0.882	aaATAGTtgg
OsSK31	P$P_01		2733	2742	1	1	0.876	gtCTACCga
OsSK31	P$GT1_Q6_01	GT1	2754	2766	1	1	0.886	TTTTTttgtaag
OsSK31	P$GT1_Q6_01	GT1	2755	2767	1	1	0.885	TTTTTtgtaagt
OsSK31	P$SBF1_01	SBF1	2770	2784	1	1	0.914	caatatTTAAAatt
OsSK31	P$SQUA_01	SQUA	2788	2798	1	1	0.859	catTTTTTat
OsSK31	P$PCF5_01	CF5	2806	2816	1	1	0.92	ttGGTCCcat
OsSK31	P$ARR1_01	ARR1	2827	2837	1	1	0.952	ataGAATCag
OsSK31	P$HAT1_01	HAT1	2829	2839	1	1	0.851	agAATCAgat
OsSK31	P$ARR18_01	ARR18	2831	2844	1	1	0.956	aatcAGATAtgac
OsSK31	P$KNOX3_01	KNOX3	2836	2848	1	1	0.975	gataTGACAtat
OsSK31	P$ATH1_01	ATH1	2840	2848	1	1	0.935	TGACAtat
OsSK31	P$ARR1_01	ARR1	2856	2866	1	1	0.981	aatGAATCta
OsSK31	P$HSFA2_01	HSFA2	3003	3009	1	1	0.933	CCAAAg
OsSK31	P$ATHB6_01	ATHB6	3013	3022	1	1	0.923	gcAATAAac
OsSK31	P$SEP3_01	wrz-03	3032	3043	1	1	0.91	catatTTTTGg
OsSK31	P$MYBAS1_01	MYBAS1	3049	3060	1	1	0.974	tgCCAACaaat
OsSK31	P$RAV1_01	RAV1	3049	3061	1	1	0.96	tgcCAACAaata
OsSK31	P$RIN_01	RIN	3052	3062	1	1	0.899	caacaAATAG
OsSK31	P$ARR2_01	ARR2	3084	3094	1	1	0.877	tactATCTTt
OsSK31	P$MYB3_01	MYB3	3102	3113	1	1	0.929	aagTAGGTata
OsSK31	P$MYB4_01	MYB4	3103	3111	1	1	0.95	agTAGGTa
OsSK31	P$C1_Q2	C1	3125	3136	1	1	0.933	taAACTAatct
OsSK31	P$SBF1_01	SBF1	3135	3149	1	1	0.914	tagctaTTAAAaaa
OsSK31	P$HSFA4A_01	HSFA4A	3137	3143	1	1	0.91	gCTATT
OsSK31	P$PBF_01	BF	3143	3154	1	1	0.964	aaaAAAAGatt
OsSK31	P$DOF_Q2	DOF	3143	3154	1	1	0.981	aaaAAAAGatt
OsSK31	P$CDF2_01	CDF2	3144	3154	1	1	0.974	aaAAAAGatt
OsSK31	P$CDF3_01	CDF3	3145	3154	1	1	0.973	aAAAAGatt
OsSK31	P$CBF3_02	CBF3	3153	3167	1	1	0.971	tatcaCCGACtatt
OsSK31	P$CBF1_04	CBF1	3154	3166	1	1	0.947	atcaCCGACtat
OsSK31	P$AT1G77200_03	AT1G77200	3155	3169	1	1	0.896	tcaCCGACtattta
OsSK31	P$ARF1_01	ARF1	3157	3165	1	1	0.887	aCCGACta
OsSK31	P$ARF5_01	ARF5	3157	3165	1	1	0.898	aCCGACta

OsSK31	P$DREB1B_01	DREB1B	3158	3163	1	1	1	CCGAC
OsSK31	P$HSFA4A_01	HSFA4A	3161	3167	1	1	1	aCTATT
OsSK31	P$AZF3_01	AZF3	3167	3178	1	1	0.975	tAGTATctttt
OsSK31	P$ARR2_01	ARR2	3167	3177	1	1	0.976	tagtATCTTt
OsSK31	P$O2_Q4	O2	3178	3189	1	1	0.873	gaaaCATGTga
OsSK31	P$AMS_01	AMS	3180	3190	1	1	0.866	aaCATGTgat
OsSK31	P$PEND_01	END	3190	3198	1	1	0.892	tAAGAAaa
OsSK31	P$BPC1_Q2	BPC1	3192	3198	1	1	0.997	AGAAAa
OsSK31	P$BPC1_Q2	BPC1	3207	3213	1	1	0.99	AGAAAt
OsSK31	P$C1_Q2	C1	3265	3276	1	1	0.939	taAACTAttgt
OsSK31	P$HSFA4A_01	HSFA4A	3268	3274	1	1	1	aCTATT
OsSK31	P$MADSA_Q2	MADSA	3287	3303	1	1	0.858	ataggaAAAATgtaga
OsSK31	P$AT3G51080_01	AT3G51080	3290	3297	1	1	1	GGAAAaa
OsSK31	P$BPC1_Q2	BPC1	3300	3306	1	1	0.99	AGAAAt
OsSK31	P$FLC_01	FLC	3308	3329	1	1	0.886	tagacacataaaaAGAAAaac
OsSK31	P$AP1_01	AP1	3315	3328	1	1	0.851	atAAAAAgaaaaa
OsSK31	P$PBF_01	BF	3315	3326	1	1	0.977	ataAAAAGaaa
OsSK31	P$DOF_Q2	DOF	3315	3326	1	1	0.999	ataAAAAGaaa
OsSK31	P$CDF2_01	CDF2	3316	3326	1	1	0.976	taAAAAGaaa
OsSK31	P$CDF3_01	CDF3	3317	3326	1	1	0.975	aAAAAGaaa
OsSK31	P$BPC1_Q2	BPC1	3321	3327	1	1	0.997	AGAAAa
OsSK31	P$DOF1_01	DOF1	3393	3404	1	1	0.97	tttTAAAGgaa
OsSK31	P$PBF_Q2	BF	3396	3402	1	1	0.986	tAAAGG
OsSK31	P$AGL27_01	AGL27	3429	3443	1	1	0.912	aTTTCTattttttt
OsSK31	P$MRP1_Q2	MRP1	3430	3442	1	1	0.871	ttTCTATttttt
OsSK31	P$HSFA4A_01	HSFA4A	3432	3438	1	1	0.914	tCTATT
OsSK31	P$SQUA_01	SQUA	3433	3443	1	1	0.915	ctaTTTTTtt
OsSK31	P$GT1_Q6_01	GT1	3436	3448	1	1	0.853	TTTTTtttttca
OsSK31	P$GT1_Q6_01	GT1	3437	3449	1	1	0.861	TTTTTttttcaa
OsSK31	P$GT1_Q6_01	GT1	3438	3450	1	1	0.954	TTTTTtttcaaa
OsSK31	P$SED_Q2	SED	3450	3460	1	1	0.897	tgagCCTTTa
OsSK31	P$PBF_Q2_01	BF	3454	3460	1	1	0.998	CCTTTa
OsSK31	P$ARR1_01	ARR1	3457	3467	1	1	0.986	ttaGAATCtc
OsSK31	P$MYB24_01	MYB24	3489	3498	1	1	0.859	ggtTTAGGg
OsSK31	P$MADSB_Q2	MADSB	3508	3523	1	1	0.858	tcacAAAAAttccaa
OsSK31	P$HSFA2_01	HSFA2	3519	3525	1	1	0.922	CCAAAt
OsSK31	P$EDF3_02	EDF3	3525	3534	1	1	1	cGACCGagg
OsSK31	P$RAV2_01	RAV2	3525	3534	1	1	1	cgACCGAgg
OsSK31	P$MYBAS1_01	MYBAS1	3539	3550	1	1	0.973	ttCCAACcatt
OsSK31	P$AT4G36620_01	AT4G36620	3540	3548	1	1	0.905	tccAACCA
OsSK31	P$GAMYB_01	GAMYB	3542	3550	1	1	0.869	CAACCatt
OsSK31	P$SED_Q2	SED	3547	3557	1	1	0.996	attgCCTTTt
OsSK31	P$PBF_Q2_01	BF	3551	3557	1	1	1	CCTTTt
OsSK31	P$SQUA_01	SQUA	3551	3561	1	1	0.862	cctTTTTTta
OsSK31	P$SQUA_01	SQUA	3552	3562	1	1	0.892	cttTTTTTac
OsSK31	P$SBF1_01	SBF1	3576	3590	1	1	0.887	gtttttTTAATtta
OsSK31	P$EDT1_01	EDT1	3579	3589	1	1	0.857	tttTTAATtt
OsSK31	P$SBF1_01	SBF1	3597	3611	1	1	0.882	attataTTAATatt
OsSK31	P$EDT1_01	EDT1	3600	3610	1	1	0.855	ataTTAATat
OsSK31	P$SBF1_01	SBF1	3603	3617	1	1	0.869	ttaataTTAAAact
OsSK31	P$EDT1_01	EDT1	3622	3632	1	1	0.938	gttTTAATga
OsSK31	P$ARR1_01	ARR1	3627	3637	1	1	0.944	aatGAATCaa
OsSK31	P$ATHB7_01	ATHB7	3629	3639	1	1	0.884	tgAATCAatt
OsSK31	P$HAT1_01	HAT1	3629	3639	1	1	0.875	tgAATCAatt
OsSK31	P$MYBAS1_01	MYBAS1	3638	3649	1	1	0.973	taCCAACcagt
OsSK31	P$AT4G36620_01	AT4G36620	3639	3647	1	1	0.909	accAACCA
OsSK31	P$GAMYB_01	GAMYB	3641	3649	1	1	0.864	CAACCagt
OsSK31	P$BHLH64_02	BHLH64	3643	3649	1	1	1	ACCAGt
OsSK31	P$PBF_01	BF	3661	3672	1	1	0.962	tttAAAAGatg
OsSK31	P$DOF_Q2	DOF	3661	3672	1	1	0.93	tttAAAAGatg
OsSK31	P$CDF2_01	CDF2	3662	3672	1	1	0.946	ttAAAAGatg
OsSK31	P$CDF3_01	CDF3	3663	3672	1	1	0.968	tAAAAGatg
OsSK31	P$P_01		3670	3679	1	1	0.879	tgCTACCaa
OsSK31	P$MYBAS1_01	MYBAS1	3678	3689	1	1	0.993	atCCAACtgcc
OsSK31	P$ATHSFA1D_01	ATHSFA1D	3690	3696	1	1	0.985	cCTACA
OsSK31	P$MYB1L_01	MYB1L	3702	3712	1	1	0.948	acCCCTActa
OsSK31	P$TRB2_01	TRB2	3702	3710	1	1	0.925	acCCCTAc
OsSK31	P$MYB3_01	MYB3	3710	3721	1	1	0.901	tagTAGGTcat
OsSK31	P$MYB4_01	MYB4	3711	3719	1	1	0.937	agTAGGTc
OsSK31	P$GT1_Q6	GT1	3728	3735	1	1	0.971	GTAAAaa
OsSK31	P$GAMYB_Q2	GAMYB	3767	3780	1	1	0.878	caaatACAACaac
OsSK31	P$RAV1_01	RAV1	3770	3782	1	1	0.911	ataCAACAacgc
OsSK31	P$GAMYB_Q2	GAMYB	3770	3783	1	1	0.942	atacaACAACgcc
OsSK31	P$AT5G54070_01	AT5G54070	3775	3781	1	1	0.958	aCAACG
OsSK31	P$ZAT1_01	ZAT1	3790	3801	1	1	0.854	gagcacACAAA
OsSK31	P$HSFA2_01	HSFA2	3807	3813	1	1	0.941	CCAAAc
OsSK31	P$RRTF1_05	RRTF1	3816	3831	1	1	0.897	gccatacCGGCGacc
OsSK31	P$AT1G68550_03	AT1G68550	3820	3829	1	1	0.95	tacCGGCGa

OsSK31	P$HSFA1E_01	HSFA1E	3822	3828	1	1	1	cCGGCG
OsSK31	P$MYBAS1_01	MYBAS1	3827	3838	1	1	0.983	gaCCAACaacc
OsSK31	P$RAV1_01	RAV1	3827	3839	1	1	0.92	gacCAACAacca
OsSK31	P$GAMYB_Q2	GAMYB	3827	3840	1	1	0.977	gaccaACAACcaa
OsSK31	P$AT4G36620_01	AT4G36620	3831	3839	1	1	0.926	aacAACCA
OsSK31	P$RIN_Q2_01	RIN	3832	3844	1	1	0.876	acaaccAAAAGg
OsSK31	P$GAMYB_01	GAMYB	3833	3841	1	1	0.901	CAACCaaa
OsSK31	P$AP3_01	AP3	3834	3849	1	1	0.859	aacCAAAAggagaga
OsSK31	P$PBF_01	BF	3835	3846	1	1	0.956	accAAAAGgag
OsSK31	P$DOF_Q2	DOF	3835	3846	1	1	0.943	accAAAAGgag
OsSK31	P$HSFA2_01	HSFA2	3836	3842	1	1	1	CCAAAa
OsSK31	P$CDF2_01	CDF2	3836	3846	1	1	0.951	ccAAAAGgag
OsSK31	P$CDF3_01	CDF3	3837	3846	1	1	0.972	cAAAAGgag
OsSK31	P$PBF_Q2	BF	3838	3844	1	1	1	aAAAGG
OsSK31	P$RAMOSA1_01	RAMOSA1	3839	3853	1	1	0.889	aaaggaGAGAGaga
OsSK31	P$RAMOSA1_01	RAMOSA1	3841	3855	1	1	0.864	aggagaGAGAGaac
OsSK31	P$PBF_01	BF	3857	3868	1	1	0.974	cacAAAAGcta
OsSK31	P$DOF_Q2	DOF	3857	3868	1	1	0.922	cacAAAAGcta
OsSK31	P$DOF2_01	DOF2	3857	3868	1	1	0.992	cacaAAAGCta
OsSK31	P$DOF3_01	DOF3	3857	3868	1	1	0.989	cacaAAAGCta
OsSK31	P$CDF2_01	CDF2	3858	3868	1	1	0.956	acAAAAGcta
OsSK31	P$CDF3_01	CDF3	3859	3868	1	1	0.976	cAAAAGcta
OsSK31	P$RIN_Q2_01	RIN	3867	3879	1	1	0.919	actaaaAAAAGc
OsSK31	P$PBF_01	BF	3870	3881	1	1	0.981	aaaAAAAGcac
OsSK31	P$DOF_Q2	DOF	3870	3881	1	1	0.996	aaaAAAAGcac
OsSK31	P$DOF2_01	DOF2	3870	3881	1	1	0.994	aaaaAAAGCac
OsSK31	P$DOF3_01	DOF3	3870	3881	1	1	0.993	aaaaAAAGCac
OsSK31	P$CDF2_01	CDF2	3871	3881	1	1	0.989	aaAAAAGcac
OsSK31	P$CDF3_01	CDF3	3872	3881	1	1	0.984	aAAAAGcac
OsSK31	P$BPC1_Q2	BPC1	3883	3889	1	1	0.99	AGAAAt
OsSK31	P$LIM1_01	LIM1	3918	3930	1	1	0.871	CCACCtccccct
OsSK31	P$LIM1_01	LIM1	3935	3947	1	1	0.969	CCACCaccacca
OsSK31	P$LIM1_01	LIM1	3938	3950	1	1	0.976	CCACCaccacct
OsSK31	P$LIM1_01	LIM1	3941	3953	1	1	0.976	CCACCacctccc
OsSK31	P$BPC6_01	BPC6	3962	3983	1	1	0.859	ctctccatcTCTCTctccctc
OsSK31	P$SQUA_01	SQUA	3984	3994	1	1	0.867	caaTTTTTtt
OsSK31	P$AT3G63350_01	AT3G63350	3994	4000	1	1	0.867	CCGCCt
OsSK41	P$AZF3_01	AZF3	3	14	1	1	0.875	cAGTATctact
OsSK41	P$SED_Q2	SED	11	21	1	1	0.956	acttCCTTTc
OsSK41	P$PBF_Q2_01	BF	15	21	1	1	0.985	CCTTTc
OsSK41	P$MYB3R5_01	MYB3R5	18	33	1	1	0.924	ttcacataaCCGTTt
OsSK41	P$MYB3R1_01	MYB3R1	19	34	1	1	0.95	tcacataaCCGTTtc
OsSK41	P$MYB3R4_01	MYB3R4	19	34	1	1	0.95	tcacataaCCGTTtc
OsSK41	P$AT1G69310_01	AT1G69310	21	30	1	1	0.871	acaTAACCg
OsSK41	P$WRKY25_01	WRKY25	21	30	1	1	0.901	acaTAACCg
OsSK41	P$ARR1_01	ARR1	39	49	1	1	0.985	gaaGAATCtg
OsSK41	P$SEP3_01	wrz-03	58	69	1	1	0.878	acaaaTTTTGg
OsSK41	P$KNOX3_01	KNOX3	122	134	1	1	0.974	ctttTGACAgag
OsSK41	P$WRKY11_Q2	WRKY11	124	132	1	1	0.899	tTTGACag
OsSK41	P$ZAP1_01	ZAP1	125	135	1	1	0.874	TTGACagaga
OsSK41	P$ATH1_01	ATH1	126	134	1	1	0.978	TGACAgag
OsSK41	P$GATA9_01	GATA9	129	140	1	1	0.883	cagAGATCaat
OsSK41	P$AGP1_01	AGP1	130	140	1	1	0.905	agAGATCaat
OsSK41	P$ARR10_01	ARR10	132	139	1	1	0.869	AGATCaa
OsSK41	P$DOF3_01	DOF3	154	165	1	1	0.979	attaAAAGCta
OsSK41	P$DOF2_01	DOF2	154	165	1	1	0.993	attaAAAGCta
OsSK41	P$DOF_Q2	DOF	154	165	1	1	0.934	attAAAAGcta
OsSK41	P$PBF_01	BF	154	165	1	1	0.977	attAAAAGcta
OsSK41	P$CDF2_01	CDF2	155	165	1	1	0.958	ttAAAAGcta
OsSK41	P$CDF3_01	CDF3	156	165	1	1	0.977	tAAAAGcta
OsSK41	P$LEC2_01	LEC2	178	189	1	1	0.984	caCATGCagag
OsSK41	P$MYBAS1_01	MYBAS1	193	204	1	1	0.986	agCTAACagat
OsSK41	P$ATMYB15_Q2	ATMYB15	196	202	1	1	0.865	TAACAg
OsSK41	P$GL15_01	GL15	201	211	1	1	0.94	gatgaCCCCC
OsSK41	P$PH4_01	H4	202	211	1	1	0.867	atgACCCCc
OsSK41	P$SED_Q2	SED	205	215	1	1	0.944	accCCTTTg
OsSK41	P$PBF_Q2_01	BF	209	215	1	1	0.988	CCTTTg
OsSK41	P$SPF1_Q2	SPF1	214	224	1	1	0.906	gaATAGTgat
OsSK41	P$AT1G49120_01	AT1G49120	283	293	1	1	0.863	taGCCGCctg
OsSK41	P$AT5G07310_01	AT5G07310	283	293	1	1	0.9	taGCCGCctg
OsSK41	P$AT5G43410_01	AT5G43410	283	293	1	1	0.902	taGCCGCctg
OsSK41	P$AT3G61630_01	AT3G61630	283	293	1	1	0.955	taGCCGCctg
OsSK41	P$AT1G77640_01	AT1G77640	283	293	1	1	0.875	taGCCGCctg
OsSK41	P$AT1G68550_01	AT1G68550	283	293	1	1	0.945	taGCCGCctg
OsSK41	P$AT5G11190_01	AT5G11190	283	293	1	1	0.866	taGCCGCctg
OsSK41	P$CBF_01	CBF	283	293	1	1	0.881	taGCCGCctg
OsSK41	P$AT3G25890_01	AT3G25890	283	293	1	1	0.88	taGCCGCctg
OsSK41	P$AT4G23750_01	AT4G23750	283	293	1	1	0.855	taGCCGCctg

OsSK41	P$CBF1_02	CBF1	283	293	1	1	0.862	tagCCGCCtg
OsSK41	P$ERF098_01	ERF098	283	291	1	1	0.997	taGCCGCc
OsSK41	P$ERF096_01	ERF096	283	293	1	1	0.991	taGCCGCctg
OsSK41	P$CRF2_01	CRF2	283	291	1	1	0.928	taGCCGCc
OsSK41	P$ERF1_Q2_01	ERF1	283	297	1	1	0.904	taGCCGCctgtgag
OsSK41	P$RRTF1_01	RRTF1	283	293	1	1	0.91	taGCCGCctg
OsSK41	P$AT4G27950_01	AT4G27950	283	293	1	1	0.882	taGCCGCctg
OsSK41	P$ERF105_01	ERF105	283	293	1	1	0.902	taGCCGCctg
OsSK41	P$AT5G25190_01	AT5G25190	283	293	1	1	0.882	taGCCGCctg
OsSK41	P$ERF1_02	ERF1	283	293	1	1	0.917	taGCCGCctg
OsSK41	P$ATERF14_01	ATERF14	283	293	1	1	0.895	taGCCGCctg
OsSK41	P$OPBP1_01	OPBP1	283	293	1	1	0.892	taGCCGCctg
OsSK41	P$CRF1_02	CRF1	283	293	1	1	0.895	taGCCGCctg
OsSK41	P$AT2G47520_01	AT2G47520	283	293	1	1	0.934	taGCCGCctg
OsSK41	P$DREBI5_01	DREBI5	283	293	1	1	0.878	taGCCGCctg
OsSK41	P$PTI5_01	TI5	283	293	1	1	0.912	taGCCGCctg
OsSK41	P$ABI4_03	ABI4	283	293	1	1	0.87	taGCCGCctg
OsSK41	P$ERF4_02	ERF4	283	293	1	1	0.923	taGCCGCctg
OsSK41	P$AT5G25390_01	AT5G25390	283	293	1	1	0.873	taGCCGCctg
OsSK41	P$ERF1_05	ERF1	283	293	1	1	0.872	taGCCGCctg
OsSK41	P$CBF17_01	CBF17	283	293	1	1	0.878	taGCCGCctg
OsSK41	P$ERF1B_03	ERF1B	283	293	1	1	0.886	taGCCGCctg
OsSK41	P$ERF2_03	ERF2	283	293	1	1	0.924	taGCCGCctg
OsSK41	P$DRF13_01	DRF13	283	293	1	1	0.861	taGCCGCctg
OsSK41	P$TSRF1_01	TSRF1	283	293	1	1	0.881	taGCCGCctg
OsSK41	P$DREBII1_01	DREBII1	283	293	1	1	0.853	taGCCGCctg
OsSK41	P$EREBP1_01	EREBP1	283	293	1	1	0.926	taGCCGCctg
OsSK41	P$ERF13_02	ERF13	284	292	1	1	0.983	agCCGCCt
OsSK41	P$ERF2_01	ERF2	284	291	1	1	0.922	agCCGCC
OsSK41	P$ERF094_01	ERF094	284	292	1	1	0.989	aGCCGCct
OsSK41	P$ERF7_02	ERF7	284	294	1	1	0.988	aGCCGCctgt
OsSK41	P$ERF1B_06	ERF1B	284	292	1	1	0.971	aGCCGCct
OsSK41	P$AT3G63350_01	AT3G63350	286	292	1	1	0.867	CCGCCt
OsSK41	P$TGA1A_Q2_01	TGA1A	298	320	1	1	0.856	cccATGACctggtcaacgaatg
OsSK41	P$RAV1_02	RAV1	301	313	1	1	0.91	atgACCTGgtca
OsSK41	P$WRKY60_01	WRKY60	306	317	1	1	0.999	ctgGTCAAcga
OsSK41	P$WRKY57_01	WRKY57	306	316	1	1	0.985	ctgGTCAAcg
OsSK41	P$WRKY48_02	WRKY48	306	316	1	1	1	ctgGTCAAcg
OsSK41	P$WRKY21_02	WRKY21	306	316	1	1	1	ctgGTCAAcg
OsSK41	P$WRKY18_02	WRKY18	306	316	1	1	1	ctgGTCAAcg
OsSK41	P$WRKY8_01	WRKY8	307	316	1	1	1	tgGTCAAcg
OsSK41	P$WRKY75_01	WRKY75	307	315	1	1	0.998	tgGTCAAc
OsSK41	P$WRKY63_01	WRKY63	307	315	1	1	0.999	tgGTCAAc
OsSK41	P$WRKY62_01	WRKY62	307	315	1	1	1	tgGTCAAc
OsSK41	P$WRKY43_02	WRKY43	307	317	1	1	0.985	tgGTCAAcga
OsSK41	P$WRKY40_01	WRKY40	307	315	1	1	1	tgGTCAAc
OsSK41	P$WRKY25_02	WRKY25	307	315	1	1	0.994	tgGTCAAc
OsSK41	P$WRKY2_01	WRKY2	307	315	1	1	0.998	tgGTCAAc
OsSK41	P$WRKY15_01	WRKY15	307	317	1	1	1	tgGTCAAcga
OsSK41	P$WRKY23_01	WRKY23	308	316	1	1	0.964	gGTCAAcg
OsSK41	P$WRKY30_01	WRKY30	308	318	1	1	0.999	gGTCAAcgaa
OsSK41	P$WRKY18_Q2	WRKY18	309	318	1	1	0.922	GTCAAcgaa
OsSK41	P$AT5G54070_01	AT5G54070	310	316	1	1	0.91	tCAACG
OsSK41	P$TGA1_01	TGA1	315	326	1	1	0.938	gaaTGACGgga
OsSK41	P$TGA7_01	TGA7	316	326	1	1	0.887	aaTGACGgga
OsSK41	P$TGA5_01	TGA5	317	325	1	1	0.869	aTGACGgg
OsSK41	P$ABI3_01	ABI3	337	346	1	1	0.863	taGCATGat
OsSK41	P$ABI3_01	ABI3	346	355	1	1	0.861	gaGCATGaa
OsSK41	P$PHYPA64121_06	HYPA64121	365	378	1	1	0.867	atcTCGGTggacc
OsSK41	P$SED_Q2	SED	372	382	1	1	0.896	tggaCCTTTc
OsSK41	P$PBF_Q2_01	BF	376	382	1	1	0.985	CCTTTc
OsSK41	P$GT1_Q6_01	GT1	393	405	1	1	0.863	TTTGTtactacc
OsSK41	P$P_01		398	407	1	1	0.876	taCTACCac
OsSK41	P$BHLH112_01	BHLH112	402	411	1	1	1	accACTTGt
OsSK41	P$PEND_02	END	409	419	1	1	0.865	gtTTCTTgtt
OsSK41	P$HSFA4A_01	HSFA4A	419	425	1	1	0.914	tCTATT
OsSK41	P$AT4G16610_01	AT4G16610	425	435	1	1	1	catTCGGGca
OsSK41	P$O2_Q4	O2	435	446	1	1	0.906	tagcCATGTga
OsSK41	P$AMS_01	AMS	437	447	1	1	0.952	gcCATGTgac
OsSK41	P$GATA9_01	GATA9	513	524	1	1	0.988	ggcAGATCtgt
OsSK41	P$AGP1_01	AGP1	514	524	1	1	0.948	gcAGATCtgt
OsSK41	P$GATA8_01	GATA8	515	524	1	1	0.998	caGATCTgt
OsSK41	P$GATA11_01	GATA11	515	523	1	1	0.975	caGATCTg
OsSK41	P$GATA10_01	GATA10	515	523	1	1	0.962	cAGATCtg
OsSK41	P$ARR10_01	ARR10	516	523	1	1	0.978	AGATCtg
OsSK41	P$ATMYB15_Q2	ATMYB15	527	533	1	1	0.865	TAACAg
OsSK41	P$ATHSFA1D_01	ATHSFA1D	539	545	1	1	1	aCTACA
OsSK41	P$GAMYB_Q2	GAMYB	542	555	1	1	0.878	acattACAACata

OsSK41	P$RAV1_01	RAV1	545	557	1	1	0.952	ttaCAACAtaca
OsSK41	P$ARR18_01	ARR18	555	568	1	1	0.943	cagtAGATActac
OsSK41	P$ATHSFA1D_01	ATHSFA1D	563	569	1	1	1	aCTACA
OsSK41	P$BPC1_Q2	BPC1	569	575	1	1	0.99	AGAAAc
OsSK41	P$ARF8_01	ARF8	631	640	1	1	0.956	atTGTCGtt
OsSK41	P$ID1_01	ID1	631	642	1	1	0.855	aTTGTCgttta
OsSK41	P$GAMYB_Q2	GAMYB	636	649	1	1	0.882	cgtttACAACtag
OsSK41	P$C1_Q2	C1	641	652	1	1	0.928	acAACTAgtac
OsSK41	P$AT4G36620_01	AT4G36620	654	662	1	1	0.883	gagAACCA
OsSK41	P$ATMYB77_01	ATMYB77	656	669	1	1	0.902	gaaccaCGGTTat
OsSK41	P$AT3G25990_01	AT3G25990	657	678	1	1	0.856	aaccacGGTTAtgtacagaag
OsSK41	P$ATSPL8_01	ATSPL8	663	679	1	1	0.897	ggttaTGTACagaagt
OsSK41	P$GT1_Q6_01	GT1	678	690	1	1	0.885	TTTTTcttaaag
OsSK41	P$SBF1_01	SBF1	678	692	1	1	0.873	tttttcTTAAAgaa
OsSK41	P$DOF1_01	DOF1	682	693	1	1	0.977	tctTAAAGaaa
OsSK41	P$BPC1_Q2	BPC1	688	694	1	1	0.997	AGAAAa
OsSK41	P$RAV1_02	RAV1	692	704	1	1	0.911	aaaACCTGcatg
OsSK41	P$ABI3_01	ABI3	697	706	1	1	0.894	ctGCATGat
OsSK41	P$GATA15_01	GATA15	700	709	1	1	0.999	caTGATCct
OsSK41	P$ATSPL8_01	ATSPL8	703	719	1	1	0.9	gatccTGTACactcca
OsSK41	P$SPF1_Q2	SPF1	716	726	1	1	0.862	ccATAGTtct
OsSK41	P$ATHSFA1D_01	ATHSFA1D	757	763	1	1	0.94	gCTACA
OsSK41	P$GT1_Q6_01	GT1	768	780	1	1	0.86	TTTGTtttttcc
OsSK41	P$E2F_Q2	E2F	773	784	1	1	0.976	tttTTCCCgca
OsSK41	P$EDT1_01	EDT1	784	794	1	1	0.926	ttgTTAATgc
OsSK41	P$KNOX3_01	KNOX3	796	808	1	1	0.976	ctgcTGACAagt
OsSK41	P$ATH1_01	ATH1	800	808	1	1	0.931	TGACAagt
OsSK41	P$AT4G36620_01	AT4G36620	820	828	1	1	0.866	gcgAACCA
OsSK41	P$LEC2_01	LEC2	824	835	1	1	0.987	acCATGCatgt
OsSK41	P$FUS3_Q2	FUS3	825	836	1	1	0.97	cCATGCatgtt
OsSK41	P$O2_Q4	O2	826	837	1	1	0.86	catgCATGTtg
OsSK41	P$IDEF1_Q2	IDEF1	826	838	1	1	0.905	CATGCatgttgt
OsSK41	P$ABI3_01	ABI3	827	836	1	1	0.859	atGCATGtt
OsSK41	P$P_01		842	851	1	1	0.876	ttCTACCga
OsSK41	P$AT2G41690_01	AT2G41690	847	853	1	1	0.978	CCGAAa
OsSK41	P$AT5G04240_01	AT5G04240	859	865	1	1	0.938	tGGCAC
OsSK41	P$LEC2_01	LEC2	862	873	1	1	0.977	caCATGCaggc
OsSK41	P$FUS3_Q2	FUS3	863	874	1	1	0.917	aCATGCaggct
OsSK41	P$ID1_01	ID1	874	885	1	1	0.941	tTTGTCgattt
OsSK41	P$ARF8_01	ARF8	874	883	1	1	0.958	ttTGTCGat
OsSK41	P$PEND_02	END	881	891	1	1	0.894	atTTCTTttc
OsSK41	P$ATMYB77_01	ATMYB77	925	938	1	1	0.88	gatttgCGGTTag
OsSK41	P$MYB24_01	MYB24	931	940	1	1	0.918	cggTTAGGg
OsSK41	P$SBF1_01	SBF1	956	970	1	1	0.852	ctaaaaTTAAAagt
OsSK41	P$PBF_01	BF	961	972	1	1	0.961	attAAAAGttt
OsSK41	P$DOF_Q2	DOF	961	972	1	1	0.934	attAAAAGttt
OsSK41	P$CDF2_01	CDF2	962	972	1	1	0.967	ttAAAAGttt
OsSK41	P$CDF3_01	CDF3	963	972	1	1	0.984	tAAAAGttt
OsSK41	P$WRKY11_Q2	WRKY11	969	977	1	1	0.952	tTTGACta
OsSK41	P$DOF1_01	DOF1	986	997	1	1	0.976	gttTAAAGaaa
OsSK41	P$MADSA_Q2	MADSA	992	1008	1	1	0.872	agaaaaAAAATtgaaa
OsSK41	P$BPC1_Q2	BPC1	992	998	1	1	0.997	AGAAAa
OsSK41	P$MADSB_Q2	MADSB	993	1008	1	1	0.861	gaaaAAAAAttgaaa
OsSK41	P$AP1_01	AP1	995	1008	1	1	0.899	aaAAAAAttgaaa
OsSK41	P$PBF_01	BF	1038	1049	1	1	0.95	tggAAAAGatg
OsSK41	P$DOF_Q2	DOF	1038	1049	1	1	0.938	tggAAAAGatg
OsSK41	P$AT3G51080_01	AT3G51080	1039	1046	1	1	0.918	GGAAAag
OsSK41	P$CDF2_01	CDF2	1039	1049	1	1	0.946	ggAAAAGatg
OsSK41	P$CDF3_01	CDF3	1040	1049	1	1	0.968	gAAAAGatg
OsSK41	P$BPC1_Q2	BPC1	1058	1064	1	1	0.997	AGAAAa
OsSK41	P$PBF_01	BF	1058	1069	1	1	0.961	agaAAAAGtta
OsSK41	P$DOF_Q2	DOF	1058	1069	1	1	0.981	agaAAAAGtta
OsSK41	P$CDF2_01	CDF2	1059	1069	1	1	0.994	gaAAAAGtta
OsSK41	P$CDF3_01	CDF3	1060	1069	1	1	0.989	aAAAAGtta
OsSK41	P$MYB24_01	MYB24	1063	1072	1	1	0.923	aagTTAGGa
OsSK41	P$ANAC013_01	ANAC013	1076	1092	1	1	0.859	CTTGGaagagaagaat
OsSK41	P$ATERF14_01	ATERF14	1111	1121	1	1	0.868	aaGCCGCact
OsSK41	P$ERF4_02	ERF4	1111	1121	1	1	0.855	aaGCCGCact
OsSK41	P$ERF4_03	ERF4	1111	1121	1	1	0.885	aaGCCGCact
OsSK41	P$AT5G43410_01	AT5G43410	1111	1121	1	1	0.869	aaGCCGCact
OsSK41	P$AT3G16280_01	AT3G16280	1111	1121	1	1	0.859	aaGCCGCact
OsSK41	P$CRF2_01	CRF2	1111	1119	1	1	0.881	aaGCCGCa
OsSK41	P$ERF098_01	ERF098	1111	1119	1	1	0.898	aaGCCGCa
OsSK41	P$ERF7_02	ERF7	1112	1122	1	1	0.95	aGCCGCactt
OsSK41	P$AT1G66560_01	AT1G66560	1117	1127	1	1	0.953	cacTTAACca
OsSK41	P$WRKY21_01	WRKY21	1118	1127	1	1	0.956	acTTAACca
OsSK41	P$AT2G24570_01	AT2G24570	1118	1127	1	1	0.955	acTTAACca
OsSK41	P$AT4G23550_01	AT4G23550	1118	1127	1	1	0.958	acTTAACca

OsSK41	P$WRKY7_01	WRKY7	1118	1127	1	1	0.959	acTTAACca
OsSK41	P$WRKY25_01	WRKY25	1118	1127	1	1	0.971	actTAACCa
OsSK41	P$WRKY33_01	WRKY33	1118	1127	1	1	0.885	actTAACCa
OsSK41	P$AT1G29860_01	AT1G29860	1118	1127	1	1	0.922	actTAACCa
OsSK41	P$AT3G62340_01	AT3G62340	1118	1127	1	1	0.907	actTAACCa
OsSK41	P$AT1G69310_01	AT1G69310	1118	1127	1	1	0.936	actTAACCa
OsSK41	P$WRKY26_01	WRKY26	1118	1127	1	1	0.889	actTAACCa
OsSK41	P$AT1G30650_01	AT1G30650	1118	1127	1	1	0.958	acTTAACca
OsSK41	P$WRKY46_01	WRKY46	1118	1127	1	1	0.881	acTTAACca
OsSK41	P$AT5G15130_01	AT5G15130	1118	1127	1	1	0.973	acTTAACca
OsSK41	P$AT2G34830_01	AT2G34830	1118	1127	1	1	0.958	acTTAACca
OsSK41	P$AT1G18860_01	AT1G18860	1118	1127	1	1	0.973	acTTAACca
OsSK41	P$AT1G64000_01	AT1G64000	1118	1127	1	1	0.94	acTTAACca
OsSK41	P$AT4G22070_01	AT4G22070	1118	1127	1	1	0.952	acTTAACca
OsSK41	P$WRKY6_01	WRKY6	1118	1127	1	1	0.95	acTTAACca
OsSK41	P$AT1G66600_01	AT1G66600	1118	1127	1	1	0.951	acTTAACca
OsSK41	P$AT1G68150_01	AT1G68150	1118	1127	1	1	0.967	acTTAACca
OsSK41	P$AT5G41570_01	AT5G41570	1118	1127	1	1	0.939	acTTAACca
OsSK41	P$AT1G69810_01	AT1G69810	1118	1127	1	1	0.969	acTTAACca
OsSK41	P$AT4G36620_01	AT4G36620	1119	1127	1	1	0.966	cttAACCA
OsSK41	P$GT1_01	GT1	1119	1127	1	1	0.968	ctTAACCa
OsSK41	P$ARR1_01	ARR1	1134	1144	1	1	0.98	gctGAATCtt
OsSK41	P$ARR2_01	ARR2	1135	1145	1	1	0.965	ctgaATCTTg
OsSK41	P$RAV1_01	RAV1	1164	1176	1	1	0.959	tagCAACAttgc
OsSK41	P$AGL1_02	AGL1	1171	1189	1	1	0.948	aTTGCCcaatctgggata
OsSK41	P$SBF1_01	SBF1	1206	1220	1	1	0.855	gatataTTAATtta
OsSK41	P$EDT1_01	EDT1	1209	1219	1	1	0.863	ataTTAATtt
OsSK41	P$EDT1_01	EDT1	1214	1224	1	1	0.891	aatTTAATtt
OsSK41	P$SED_Q2	SED	1263	1273	1	1	0.984	atagCCTTTa
OsSK41	P$PBF_Q2_01	BF	1267	1273	1	1	0.998	CCTTTa
OsSK41	P$ATHB6_01	ATHB6	1277	1286	1	1	0.899	tgAATAAac
OsSK41	P$C1_Q2	C1	1281	1292	1	1	0.955	taAACTActgc
OsSK41	P$DOF43_01	DOF43	1303	1314	1	1	0.887	aatgctACTTT
OsSK41	P$PBF_01	BF	1313	1324	1	1	0.973	tagAAAAGtcc
OsSK41	P$DOF_Q2	DOF	1313	1324	1	1	0.94	tagAAAAGtcc
OsSK41	P$CDF2_01	CDF2	1314	1324	1	1	0.965	agAAAAGtcc
OsSK41	P$BPC1_Q2	BPC1	1314	1320	1	1	0.997	AGAAAa
OsSK41	P$CDF3_01	CDF3	1315	1324	1	1	0.983	gAAAAGtcc
OsSK41	P$SEP3_01	wrz-03	1329	1340	1	1	0.862	aggttTTTTGg
OsSK41	P$GT1_Q6_01	GT1	1332	1344	1	1	0.855	TTTTTtggtact
OsSK41	P$DOF3_01	DOF3	1365	1376	1	1	0.98	ttgaAAAGCaa
OsSK41	P$DOF2_01	DOF2	1365	1376	1	1	0.991	ttgaAAAGCaa
OsSK41	P$DOF_Q2	DOF	1365	1376	1	1	0.953	ttgAAAAGcaa
OsSK41	P$PBF_01	BF	1365	1376	1	1	0.979	ttgAAAAGcaa
OsSK41	P$CDF2_01	CDF2	1366	1376	1	1	0.961	tgAAAAGcaa
OsSK41	P$CDF3_01	CDF3	1367	1376	1	1	0.979	gAAAAGcaa
OsSK41	P$AT5G54070_01	AT5G54070	1372	1378	1	1	0.915	gCAACG
OsSK41	P$PBF_01	BF	1390	1401	1	1	0.975	attAAAAGcag
OsSK41	P$DOF_Q2	DOF	1390	1401	1	1	0.95	attAAAAGcag
OsSK41	P$DOF2_01	DOF2	1390	1401	1	1	0.993	attaAAAGCag
OsSK41	P$DOF3_01	DOF3	1390	1401	1	1	0.98	attaAAAGCag
OsSK41	P$CDF2_01	CDF2	1391	1401	1	1	0.96	ttAAAAGcag
OsSK41	P$CDF3_01	CDF3	1392	1401	1	1	0.978	tAAAAGcag
OsSK41	P$REF6_01	REF6	1394	1405	1	1	0.866	aaagCAGAGct
OsSK41	P$AT1G66560_01	AT1G66560	1401	1411	1	1	0.858	agcTTAACtc
OsSK41	P$AT1G18860_01	AT1G18860	1402	1411	1	1	0.888	gcTTAACtc
OsSK41	P$AT1G66600_01	AT1G66600	1402	1411	1	1	0.859	gcTTAACtc
OsSK41	P$AT1G68150_01	AT1G68150	1402	1411	1	1	0.852	gcTTAACtc
OsSK41	P$AT5G15130_01	AT5G15130	1402	1411	1	1	0.884	gcTTAACtc
OsSK41	P$PBF_01	BF	1427	1438	1	1	0.957	cctAAAAGtcg
OsSK41	P$DOF_Q2	DOF	1427	1438	1	1	0.934	cctAAAAGtcg
OsSK41	P$CDF2_01	CDF2	1428	1438	1	1	0.964	ctAAAAGtcg
OsSK41	P$CDF3_01	CDF3	1429	1438	1	1	0.981	tAAAAGtcg
OsSK41	P$HMG1_01	HMG1	1444	1453	1	1	0.95	GTTGTaatt
OsSK41	P$RAV1_01	RAV1	1451	1463	1	1	0.961	ttgCAACAttgg
OsSK41	P$ABI3_01	ABI3	1468	1477	1	1	0.863	tgGCATGat
OsSK41	P$UIF1_01	UIF1	1471	1481	1	1	0.86	catGATTCca
OsSK41	P$WRKY48_01	WRKY48	1485	1494	1	1	0.914	tttaAACAA
OsSK41	P$AT3G20750_01	AT3G20750	1486	1494	1	1	0.876	tTAAACaa
OsSK41	P$P_01		1504	1513	1	1	0.875	caCTACCcc
OsSK41	P$HAT1_01	HAT1	1531	1541	1	1	0.852	aaAATCAcaa
OsSK41	P$DOF_Q2	DOF	1544	1555	1	1	0.98	agaAAAAGttc
OsSK41	P$PBF_01	BF	1544	1555	1	1	0.957	agaAAAAGttc
OsSK41	P$BPC1_Q2	BPC1	1544	1550	1	1	0.997	AGAAAa
OsSK41	P$CDF2_01	CDF2	1545	1555	1	1	0.994	gaAAAAGttc
OsSK41	P$CDF3_01	CDF3	1546	1555	1	1	0.99	aAAAAGttc
OsSK41	P$AP2B_01	AP2B	1551	1561	1	1	0.868	gttcACTCCc
OsSK41	P$WRKY60_01	WRKY60	1560	1571	1	1	0.92	caaGTCAAcac

OsSK41	P$WRKY57_01	WRKY57	1560	1570	1	1	0.995	caaGTCAAca
OsSK41	P$WRKY48_02	WRKY48	1560	1570	1	1	0.997	caaGTCAAca
OsSK41	P$WRKY21_02	WRKY21	1560	1570	1	1	0.995	caaGTCAAca
OsSK41	P$WRKY18_02	WRKY18	1560	1570	1	1	0.981	caaGTCAAca
OsSK41	P$WRKY40_03	WRKY40	1560	1570	1	1	0.998	caAGTCAaca
OsSK41	P$WRKY8_01	WRKY8	1561	1570	1	1	0.998	aaGTCAAca
OsSK41	P$WRKY75_01	WRKY75	1561	1569	1	1	1	aaGTCAAc
OsSK41	P$WRKY63_01	WRKY63	1561	1569	1	1	0.914	aaGTCAAc
OsSK41	P$WRKY62_01	WRKY62	1561	1569	1	1	0.918	aaGTCAAc
OsSK41	P$WRKY43_02	WRKY43	1561	1571	1	1	1	aaGTCAAcac
OsSK41	P$WRKY40_01	WRKY40	1561	1569	1	1	0.996	aaGTCAAc
OsSK41	P$WRKY25_02	WRKY25	1561	1569	1	1	0.933	aaGTCAAc
OsSK41	P$WRKY2_01	WRKY2	1561	1569	1	1	0.948	aaGTCAAc
OsSK41	P$WRKY15_01	WRKY15	1561	1571	1	1	0.992	aaGTCAAcac
OsSK41	P$RAV1_01	RAV1	1562	1574	1	1	0.918	agtCAACActcc
OsSK41	P$WRKY30_01	WRKY30	1562	1572	1	1	0.99	aGTCAAcact
OsSK41	P$WRKY23_01	WRKY23	1562	1570	1	1	0.961	aGTCAAca
OsSK41	P$WRKY18_Q2	WRKY18	1563	1572	1	1	0.93	GTCAAcact
OsSK41	P$WRKY60_01	WRKY60	1572	1583	1	1	0.891	cctGTCAAtca
OsSK41	P$WRKY57_01	WRKY57	1572	1582	1	1	0.955	cctGTCAAtc
OsSK41	P$WRKY48_02	WRKY48	1572	1582	1	1	0.986	cctGTCAAtc
OsSK41	P$WRKY21_02	WRKY21	1572	1582	1	1	0.95	cctGTCAAtc
OsSK41	P$WRKY18_02	WRKY18	1572	1582	1	1	0.945	cctGTCAAtc
OsSK41	P$WRKY8_01	WRKY8	1573	1582	1	1	0.977	ctGTCAAtc
OsSK41	P$WRKY75_01	WRKY75	1573	1581	1	1	0.927	ctGTCAAt
OsSK41	P$WRKY63_01	WRKY63	1573	1581	1	1	0.887	ctGTCAAt
OsSK41	P$WRKY43_02	WRKY43	1573	1583	1	1	0.95	ctGTCAAtca
OsSK41	P$WRKY40_01	WRKY40	1573	1581	1	1	0.977	ctGTCAAt
OsSK41	P$WRKY25_02	WRKY25	1573	1581	1	1	0.894	ctGTCAAt
OsSK41	P$WRKY2_01	WRKY2	1573	1581	1	1	0.906	ctGTCAAt
OsSK41	P$WRKY15_01	WRKY15	1573	1583	1	1	0.959	ctGTCAAtca
OsSK41	P$WRKY30_01	WRKY30	1574	1584	1	1	0.905	tGTCAAtcaa
OsSK41	P$WRKY18_Q2	WRKY18	1575	1584	1	1	0.917	GTCAAtcaa
OsSK41	P$HAT1_01	HAT1	1576	1586	1	1	0.892	tcAATCAatc
OsSK41	P$ATHB7_01	ATHB7	1576	1586	1	1	0.96	tcAATCAatc
OsSK41	P$HSFA2_01	HSFA2	1601	1607	1	1	0.922	CCAAAt
OsSK41	P$WRKY11_Q2	WRKY11	1605	1613	1	1	0.927	aTTGACtg
OsSK41	P$HSFA2_01	HSFA2	1616	1622	1	1	0.941	CCAAAc
OsSK41	P$HSFA2_01	HSFA2	1623	1629	1	1	1	CCAAAa
OsSK41	P$HAT1_01	HAT1	1635	1645	1	1	0.866	caAATCAaat
OsSK41	P$ATHB7_01	ATHB7	1635	1645	1	1	0.875	caAATCAaat
OsSK41	P$GAMYB_Q2	GAMYB	1661	1674	1	1	0.924	agctcACAACcca
OsSK41	P$GAMYB_01	GAMYB	1667	1675	1	1	0.893	CAACCcaa
OsSK41	P$ASR1_01	ASR1	1669	1674	1	1	1	ACCCA
OsSK41	P$SQUA_01	SQUA	1672	1682	1	1	0.884	caaTTTTTtg
OsSK41	P$GT1_Q6_01	GT1	1676	1688	1	1	0.854	TTTTTgtttgct
OsSK41	P$CBNAC_01	CBNAC	1683	1689	1	1	1	tTGCTT
OsSK41	P$GL15_01	GL15	1686	1696	1	1	0.854	cttctCCCCC
OsSK41	P$GL15_01	GL15	1698	1708	1	1	0.858	catctCCCCC
OsSK41	P$PDF2_01	DF2	1736	1747	1	1	0.865	tccaTAAATtt
OsSK41	P$LEC2_01	LEC2	1758	1769	1	1	0.953	tcCATGCcctt
AtSK11	P$WRKY11_Q2	WRKY11	11	19	1	1	0.952	aTTGACta
AtSK11	P$NTL9_01	NTL9	16	25	1	1	0.895	cTAAGTaat
AtSK11	P$ARR2_01	ARR2	21	31	1	1	0.903	taatATCTTa
AtSK11	P$SPL14_02	SPL14	27	46	1	1	0.927	cttaatCCGTAcctttttgt
AtSK11	P$ATSPL3_01	ATSPL3	29	45	1	1	0.986	taatcCGTACcttttg
AtSK11	P$SPL14_03	SPL14	32	43	1	1	0.99	tcCGTACcttt
AtSK11	P$SPL3_01	SPL3	32	40	1	1	0.998	tCCGTAcc
AtSK11	P$SPL14_01	SPL14	33	40	1	1	0.944	CCGTAcc
AtSK11	P$SED_Q2	SED	34	44	1	1	0.922	cgtaCCTTTt
AtSK11	P$PBF_Q2_01	BF	38	44	1	1	1	CCTTTt
AtSK11	P$ID1_01	ID1	41	52	1	1	0.854	tTTGTCgaaaa
AtSK11	P$ARF8_01	ARF8	41	50	1	1	0.958	ttTGTCGaa
AtSK11	P$AP1_01	AP1	49	62	1	1	0.891	aaAAAAAtagtaa
AtSK11	P$SPF1_Q2	SPF1	53	63	1	1	1	aaATAGTaat
AtSK11	P$ATHB1_03	ATHB1	58	69	1	1	0.968	gtAATAAttcc
AtSK11	P$ATHB5_04	ATHB5	58	69	1	1	0.971	gtAATAAttcc
AtSK11	P$ATHB6_01	ATHB6	58	67	1	1	0.984	gtAATAAtt
AtSK11	P$ATHB16_01	ATHB16	59	67	1	1	1	tAATAAtt
AtSK11	P$ARR1_01	ARR1	72	82	1	1	0.986	ccaGAATCtc
AtSK11	P$RAV1_01	RAV1	103	115	1	1	0.932	tttCAACAttta
AtSK11	P$EDT1_01	EDT1	109	119	1	1	0.908	catTTAATtc
AtSK11	P$PDF2_01	DF2	132	143	1	1	0.878	tttaTAAATga
AtSK11	P$ATSPL8_01	ATSPL8	140	156	1	1	0.914	tgaaaTGTACatggaa
AtSK11	P$TEIL_01	TEIL	144	152	1	1	0.94	ATGTAcat
AtSK11	P$ATHB6_01	ATHB6	152	161	1	1	0.902	ggAATAAaa
AtSK11	P$ATHB6_01	ATHB6	157	166	1	1	0.905	aaAATAAag
AtSK11	P$DOF1_01	DOF1	158	169	1	1	0.989	aaaTAAAGaat

AtSK11	P$GT1_Q6_01	GT1	182	194	1	1	0.852	TTTTTtcctaac
AtSK11	P$MYBAS1_01	MYBAS1	187	198	1	1	0.951	tcCTAACtata
AtSK11	P$C1_Q2	C1	189	200	1	1	0.962	ctAACTAtaaa
AtSK11	P$PDF2_01	DF2	192	203	1	1	0.852	actaTAAATta
AtSK11	P$PDF2_01	DF2	197	208	1	1	0.898	aaatTAAATgt
AtSK11	P$ATHB1_03	ATHB1	222	233	1	1	0.986	ccAATAAttgg
AtSK11	P$ATHB5_04	ATHB5	222	233	1	1	0.994	ccAATAAttgg
AtSK11	P$ATHB6_01	ATHB6	222	231	1	1	1	ccAATAAtt
AtSK11	P$ATHB16_01	ATHB16	223	231	1	1	0.963	cAATAAtt
AtSK11	P$ATHB1_01	ATHB1	234	248	1	1	0.966	gatgaATTATtgag
AtSK11	P$ATHB5_01	ATHB5	237	246	1	1	0.936	gaaTTATTg
AtSK11	P$ARR18_01	ARR18	242	255	1	1	0.94	attgAGATAggtt
AtSK11	P$MYB3_01	MYB3	246	257	1	1	0.903	agaTAGGTtac
AtSK11	P$MYB4_01	MYB4	247	255	1	1	0.871	gaTAGGTt
AtSK11	P$ANTL_01	ANTL	251	261	1	1	0.936	gGTTACtact
AtSK11	P$ARR18_01	ARR18	259	272	1	1	0.941	ctttAGATAggga
AtSK11	P$ROM_Q2	ROM	278	287	1	1	0.878	ctCACCTca
AtSK11	P$ATHB6_01	ATHB6	291	300	1	1	0.91	atAATAAaa
AtSK11	P$PBF_01	BF	296	307	1	1	0.988	aaaAAAAGtgg
AtSK11	P$DOF_Q2	DOF	296	307	1	1	0.988	aaaAAAAGtgg
AtSK11	P$CDF2_01	CDF2	297	307	1	1	0.999	aaAAAAGtgg
AtSK11	P$CDF3_01	CDF3	298	307	1	1	0.999	aAAAAGtgg
AtSK11	P$AGL27_01	AGL27	317	331	1	1	0.866	tTTTCTtttttgtt
AtSK11	P$SEP3_01	wrz-03	319	330	1	1	0.874	ttcttTTTTGt
AtSK11	P$CBF3_02	CBF3	332	346	1	1	0.934	cctctCCGACacgt
AtSK11	P$CBF1_04	CBF1	333	345	1	1	0.932	ctctCCGACacg
AtSK11	P$AT1G77200_03	AT1G77200	334	348	1	1	0.92	tctCCGACacgttt
AtSK11	P$DREB1G_02	DREB1G	334	344	1	1	0.896	tctCCGACac
AtSK11	P$ARF1_01	ARF1	336	344	1	1	0.931	tCCGACac
AtSK11	P$ARF5_01	ARF5	336	344	1	1	0.977	tCCGACac
AtSK11	P$ABF2_01	ABF2	336	349	1	1	0.953	tccgaCACGTttg
AtSK11	P$ABF3_Q2	ABF3	337	351	1	1	0.905	ccgaCACGTttgac
AtSK11	P$BZR1_02	BZR1	337	351	1	1	0.859	ccgaCACGTttgac
AtSK11	P$DREB1B_01	DREB1B	337	342	1	1	1	CCGAC
AtSK11	P$ABF4_01	ABF4	338	350	1	1	0.916	cgaCACGTttga
AtSK11	P$ABI5_01	ABI5	338	348	1	1	0.958	cgaCACGTtt
AtSK11	P$GBP_Q6	GBP	338	350	1	1	0.917	cgaCACGTttga
AtSK11	P$CPRF2_01	CPRF2	339	349	1	1	0.938	gaCACGTttg
AtSK11	P$TGA1B_01	TGA1B	339	349	1	1	0.88	gaCACGTttg
AtSK11	P$BEE2_01	BEE2	339	349	1	1	0.919	gaCACGTttg
AtSK11	P$BIM2_01	BIM2	339	349	1	1	0.853	gaCACGTttg
AtSK11	P$BIM3_01	BIM3	339	349	1	1	0.884	gaCACGTttg
AtSK11	P$PHYPA143875_02	HYPA143875	339	349	1	1	0.885	gaCACGTttg
AtSK11	P$SPT_01	SPT	339	348	1	1	0.914	gaCACGTtt
AtSK11	P$GBF1F_Q2	GBF1F	339	350	1	1	0.902	gaCACGTttga
AtSK11	P$CPRF3_01	CPRF3	339	349	1	1	0.927	gaCACGTttg
AtSK11	P$EMBP1_Q2	EMBP1	339	349	1	1	0.917	gaCACGTttg
AtSK11	P$CPRF3_Q2	CPRF3	339	349	1	1	0.919	gaCACGTttg
AtSK11	P$CPRF2_Q2	CPRF2	339	349	1	1	0.937	gaCACGTttg
AtSK11	P$O2_02	O2	339	349	1	1	0.93	gaCACGTttg
AtSK11	P$TGA1B_Q2	TGA1B	339	349	1	1	0.913	gaCACGTttg
AtSK11	P$TGA1A_Q2	TGA1A	339	349	1	1	0.971	gaCACGTttg
AtSK11	P$MYC3_01	MYC3	340	348	1	1	0.853	aCACGTtt
AtSK11	P$RITA1_01	RITA1	340	347	1	1	0.953	aCACGTt
AtSK11	P$ABF3_01	ABF3	340	348	1	1	0.875	ACACGttt
AtSK11	P$OCSBF1_01	OCSBF1	341	346	1	1	1	CACGT
AtSK11	P$WRKY11_Q2	WRKY11	345	353	1	1	0.974	tTTGACcc
AtSK11	P$ZAP1_01	ZAP1	346	356	1	1	0.907	TTGACccaac
AtSK11	P$ASR1_01	ASR1	349	354	1	1	1	ACCCA
AtSK11	P$MYBAS1_01	MYBAS1	349	360	1	1	0.997	acCCAACggcc
AtSK11	P$AT5G54070_01	AT5G54070	351	357	1	1	1	cCAACG
AtSK11	P$MYB24_01	MYB24	390	399	1	1	0.938	tctTTAGGt
AtSK11	P$MYB131_01	MYB131	390	401	1	1	0.943	tctTTAGGtat
AtSK11	P$MYB3_01	MYB3	391	402	1	1	0.877	cttTAGGTata
AtSK11	P$MYB4_01	MYB4	392	400	1	1	0.855	ttTAGGTa
AtSK11	P$PBF_01	BF	407	418	1	1	0.974	tttAAAAGtca
AtSK11	P$DOF_Q2	DOF	407	418	1	1	0.93	tttAAAAGtca
AtSK11	P$CDF2_01	CDF2	408	418	1	1	0.964	ttAAAAGtca
AtSK11	P$CDF3_01	CDF3	409	418	1	1	0.982	tAAAAGtca
AtSK11	P$WRKY60_01	WRKY60	411	422	1	1	0.903	aaaGTCAAaat
AtSK11	P$WRKY57_01	WRKY57	411	421	1	1	0.987	aaaGTCAAaa
AtSK11	P$WRKY48_02	WRKY48	411	421	1	1	0.995	aaaGTCAAaa
AtSK11	P$WRKY21_02	WRKY21	411	421	1	1	0.968	aaaGTCAAaa
AtSK11	P$WRKY18_02	WRKY18	411	421	1	1	0.979	aaaGTCAAaa
AtSK11	P$WRKY40_03	WRKY40	411	421	1	1	1	aaAGTCAaaa
AtSK11	P$WRKY8_01	WRKY8	412	421	1	1	0.991	aaGTCAAaa
AtSK11	P$WRKY75_01	WRKY75	412	420	1	1	0.977	aaGTCAAa
AtSK11	P$WRKY63_01	WRKY63	412	420	1	1	0.905	aaGTCAAa

AtSK11	P$WRKY62_01	WRKY62	412	420	1	1	0.875	aaGTCAAa
AtSK11	P$WRKY43_02	WRKY43	412	422	1	1	0.992	aaGTCAAaat
AtSK11	P$WRKY40_01	WRKY40	412	420	1	1	0.996	aaGTCAAa
AtSK11	P$WRKY25_02	WRKY25	412	420	1	1	0.912	aaGTCAAa
AtSK11	P$WRKY2_01	WRKY2	412	420	1	1	0.939	aaGTCAAa
AtSK11	P$WRKY15_01	WRKY15	412	422	1	1	0.98	aaGTCAAaat
AtSK11	P$WRKY23_01	WRKY23	413	421	1	1	0.889	aGTCAAaa
AtSK11	P$WRKY30_01	WRKY30	413	423	1	1	0.914	aGTCAAaatt
AtSK11	P$WRKY18_Q2	WRKY18	414	423	1	1	0.982	GTCAAaatt
AtSK11	P$PEND_02	END	432	442	1	1	0.863	taTTCTTctt
AtSK11	P$ATHB6_01	ATHB6	443	452	1	1	0.909	taAATAAat
AtSK11	P$PDF2_01	DF2	443	454	1	1	0.867	taaaTAAATtc
AtSK11	P$GT1_Q6_02	GT1	467	479	1	1	0.978	atgtttTTAACt
AtSK11	P$C1_Q2	C1	473	484	1	1	0.966	ttAACTAcaag
AtSK11	P$ATHSFA1D_01	ATHSFA1D	476	482	1	1	1	aCTACA
AtSK11	P$BPC1_Q2	BPC1	482	488	1	1	0.997	AGAAAa
AtSK11	P$BPC1_Q2	BPC1	494	500	1	1	0.99	AGAAAc
AtSK11	P$EDT1_01	EDT1	508	518	1	1	0.938	gttTTAATgt
AtSK11	P$ATSPL8_01	ATSPL8	510	526	1	1	0.938	tttaaTGTACcaatca
AtSK11	P$TEIL_01	TEIL	514	522	1	1	0.881	ATGTAcca
AtSK11	P$ATHB7_01	ATHB7	519	529	1	1	0.924	ccAATCAcat
AtSK11	P$HAT1_01	HAT1	519	529	1	1	0.87	ccAATCAcat
AtSK11	P$SBF1_01	SBF1	522	536	1	1	0.872	atcacaTTAAAata
AtSK11	P$ATHB1_03	ATHB1	530	541	1	1	0.893	aaAATAAtggg
AtSK11	P$ATHB5_04	ATHB5	530	541	1	1	0.889	aaAATAAtggg
AtSK11	P$ATHB6_01	ATHB6	530	539	1	1	0.976	aaAATAAtg
AtSK11	P$ATHB16_01	ATHB16	531	539	1	1	0.867	aAATAAtg
AtSK11	P$ATMYB15_Q2	ATMYB15	550	556	1	1	1	TAACAa
AtSK11	P$SBF1_01	SBF1	552	566	1	1	0.861	acaattTTAAAaaa
AtSK11	P$ATSPL8_01	ATSPL8	562	578	1	1	0.958	aaaacTGTACaaaatt
AtSK11	P$SBF1_01	SBF1	589	603	1	1	0.85	acagttTTAATatt
AtSK11	P$SBF1_01	SBF1	617	631	1	1	0.899	actttaTTAAAaac
AtSK11	P$MYB118_01	MYB118	622	639	1	1	0.971	attaaaaacGTTACaaa
AtSK11	P$ATSPL3_01	ATSPL3	667	683	1	1	0.969	tttttCGTACagctaa
AtSK11	P$SMZ_01	SMZ	669	677	1	1	0.876	ttTCGTAc
AtSK11	P$SPL14_03	SPL14	670	681	1	1	0.887	ttCGTACagct
AtSK11	P$DOF1_01	DOF1	677	688	1	1	0.985	agcTAAAGttt
AtSK11	P$MYBAS1_01	MYBAS1	692	703	1	1	0.977	ctCCAACtaac
AtSK11	P$C1_Q2	C1	694	705	1	1	0.987	ccAACTAacag
AtSK11	P$MYBAS1_01	MYBAS1	696	707	1	1	0.993	aaCTAACagct
AtSK11	P$WEREWOLF_Q2_01	WEREWOLF	696	705	1	1	1	aACTAAcag
AtSK11	P$ATMYB15_Q2	ATMYB15	699	705	1	1	0.865	TAACAg
AtSK11	P$ATHB6_01	ATHB6	723	732	1	1	0.994	ccAATAAtc
AtSK11	P$ATHB5_04	ATHB5	723	734	1	1	0.917	ccAATAAtcct
AtSK11	P$ATHB1_03	ATHB1	723	734	1	1	0.897	ccAATAAtcct
AtSK11	P$ATHB16_01	ATHB16	724	732	1	1	0.871	cAATAAtc
AtSK11	P$ATHB6_01	ATHB6	732	741	1	1	0.914	ctAATAAat
AtSK11	P$ATHB6_01	ATHB6	736	745	1	1	0.911	taAATAAgt
AtSK11	P$ATHB5_01	ATHB5	752	761	1	1	0.856	gcaTTATTt
AtSK11	P$SEP3_01	wrz-03	773	784	1	1	0.895	atctcTTTTGg
AtSK11	P$HMG1_01	HMG1	787	796	1	1	0.924	GTTGTgttg
AtSK11	P$BPC1_Q2	BPC1	799	805	1	1	0.997	AGAAAa
AtSK11	P$PBF_01	BF	805	816	1	1	0.961	tatAAAAGttt
AtSK11	P$DOF_Q2	DOF	805	816	1	1	0.929	tatAAAAGttt
AtSK11	P$CDF2_01	CDF2	806	816	1	1	0.968	atAAAAGttt
AtSK11	P$CDF3_01	CDF3	807	816	1	1	0.984	tAAAAGttt
AtSK11	P$WRKY60_01	WRKY60	818	829	1	1	0.978	atgGTCAAatc
AtSK11	P$WRKY57_01	WRKY57	818	828	1	1	0.975	atgGTCAAat
AtSK11	P$WRKY48_02	WRKY48	818	828	1	1	0.998	atgGTCAAat
AtSK11	P$WRKY21_02	WRKY21	818	828	1	1	0.969	atgGTCAAat
AtSK11	P$WRKY18_02	WRKY18	818	828	1	1	0.996	atgGTCAAat
AtSK11	P$WRKY8_01	WRKY8	819	828	1	1	0.992	tgGTCAAat
AtSK11	P$WRKY75_01	WRKY75	819	827	1	1	0.975	tgGTCAAa
AtSK11	P$WRKY63_01	WRKY63	819	827	1	1	0.99	tgGTCAAa
AtSK11	P$WRKY62_01	WRKY62	819	827	1	1	0.957	tgGTCAAa
AtSK11	P$WRKY43_02	WRKY43	819	829	1	1	0.976	tgGTCAAatc
AtSK11	P$WRKY40_01	WRKY40	819	827	1	1	1	tgGTCAAa
AtSK11	P$WRKY25_02	WRKY25	819	827	1	1	0.973	tgGTCAAa
AtSK11	P$WRKY2_01	WRKY2	819	827	1	1	0.989	tgGTCAAa
AtSK11	P$WRKY15_01	WRKY15	819	829	1	1	0.985	tgGTCAAatc
AtSK11	P$WRKY30_01	WRKY30	820	830	1	1	0.917	gGTCAAatct
AtSK11	P$WRKY18_Q2	WRKY18	821	830	1	1	0.961	GTCAAatct
AtSK11	P$DOF_Q2	DOF	829	840	1	1	0.982	tcaAAAAGagg
AtSK11	P$PBF_01	BF	829	840	1	1	0.974	tcaAAAAGagg
AtSK11	P$CDF2_01	CDF2	830	840	1	1	0.978	caAAAAGagg
AtSK11	P$CDF3_01	CDF3	831	840	1	1	0.983	aAAAAGagg
AtSK11	P$ZAT1_01	ZAT1	834	845	1	1	0.987	aagaggACAAA
AtSK11	P$C1_Q2	C1	843	854	1	1	0.927	aaAACTAaaac

AtSK11	P$AT4G36620_01	AT4G36620	848	856	1	1	0.915	taaAACCA
AtSK11	P$TEIL_01	TEIL	856	864	1	1	0.939	ATGTAact
AtSK11	P$WEREWOLF_Q2_01	WEREWOLF	865	874	1	1	0.897	tACTAActt
AtSK11	P$MYBAS1_01	MYBAS1	865	876	1	1	0.949	taCTAACttcg
AtSK11	P$ATHB1_01	ATHB1	880	894	1	1	0.85	gtcccATTATtttt
AtSK11	P$ATHB5_01	ATHB5	883	892	1	1	0.92	ccaTTATTt
AtSK11	P$PEND_01	END	908	916	1	1	0.864	aAAGAAat
AtSK11	P$BPC1_Q2	BPC1	910	916	1	1	0.99	AGAAAt
AtSK11	P$EDT1_01	EDT1	935	945	1	1	0.889	gatTTAATtt
AtSK11	P$TRB2_01	TRB2	953	961	1	1	0.949	caCCCTAc
AtSK11	P$MYB1L_01	MYB1L	953	963	1	1	0.977	caCCCTAcca
AtSK11	P$P_01		955	964	1	1	0.95	ccCTACCaa
AtSK11	P$HSFA2_01	HSFA2	960	966	1	1	1	CCAAAa
AtSK11	P$AP1_01	AP1	960	973	1	1	0.886	ccAAAAAaaaaaa
AtSK11	P$EDT1_01	EDT1	975	985	1	1	0.889	gatTTAATtt
AtSK11	P$SPF1_Q2	SPF1	998	1008	1	1	0.949	atATAGTaat
AtSK11	P$ATHB6_01	ATHB6	1003	1012	1	1	0.981	gtAATAAta
AtSK11	P$ATHB5_04	ATHB5	1003	1014	1	1	0.909	gtAATAAtaaa
AtSK11	P$ATHB1_03	ATHB1	1003	1014	1	1	0.901	gtAATAAtaaa
AtSK11	P$ATHB16_01	ATHB16	1004	1012	1	1	0.915	tAATAAta
AtSK11	P$ATHB6_01	ATHB6	1006	1015	1	1	0.91	atAATAAaa
AtSK12	P$SEP3_01	wrz-03	2	13	1	1	0.868	agcatTTTTGt
AtSK12	P$SHP2_01	SHP2	47	58	1	1	0.868	ttcaaTTTAAc
AtSK12	P$AT1G77950_01	AT1G77950	47	58	1	1	0.852	ttcaaTTTAAc
AtSK12	P$GT1_Q6_02	GT1	47	59	1	1	0.88	ttcaatTTAACa
AtSK12	P$WRKY48_01	WRKY48	51	60	1	1	0.964	atttAACAA
AtSK12	P$ATMYB15_Q2	ATMYB15	54	60	1	1	1	TAACAa
AtSK12	P$DOF2_01	DOF2	54	65	1	1	0.982	taacAAAGCct
AtSK12	P$DOF3_01	DOF3	54	65	1	1	0.985	taacAAAGCct
AtSK12	P$MYBAS1_01	MYBAS1	61	72	1	1	0.956	gcCTAACtcta
AtSK12	P$MYBAS1_01	MYBAS1	67	78	1	1	0.979	ctCTAACatgt
AtSK12	P$O2_Q4	O2	69	80	1	1	0.903	ctaaCATGTaa
AtSK12	P$AT3G20750_01	AT3G20750	76	84	1	1	0.887	gTAAACgt
AtSK12	P$PHYPA64121_06	HYPA64121	88	101	1	1	0.889	ttcTCGGTagata
AtSK12	P$ARR18_01	ARR18	92	105	1	1	0.955	cggtAGATAtgtt
AtSK12	P$PEND_02	END	108	118	1	1	0.909	caTTCTTata
AtSK12	P$PDF2_01	DF2	112	123	1	1	0.884	cttaTAAATgg
AtSK12	P$PHYPA182268_05	HYPA182268	120	130	1	1	0.884	tgGTCGGcga
AtSK12	P$ERF039_01	ERF039	120	130	1	1	0.98	tgGTCGGcga
AtSK12	P$DREB1A_04	DREB1A	120	130	1	1	0.969	tgGTCGGcga
AtSK12	P$AT1G68550_03	AT1G68550	121	130	1	1	0.953	ggtCGGCGa
AtSK12	P$PHYPA28324_10	HYPA28324	121	129	1	1	0.929	gGTCGGcg
AtSK12	P$PHYPA173530_04	HYPA173530	121	129	1	1	0.894	gGTCGGcg
AtSK12	P$SQUA_01	SQUA	148	158	1	1	0.87	cttTTTTTct
AtSK12	P$PIL5_01	IL5	166	180	1	1	0.983	atctcgacACGTGg
AtSK12	P$PIF3_01	IF3	167	185	1	1	0.933	tctcgaCACGTGgccccg
AtSK12	P$ABF_Q2	ABF	168	185	1	1	0.965	ctcgacACGTGgccccg
AtSK12	P$ABF2_01	ABF2	168	181	1	1	0.998	ctcgaCACGTggc
AtSK12	P$HY5_02	HY5	168	184	1	1	0.974	ctcgaCACGTggcccc
AtSK12	P$PIF3_02	IF3	168	185	1	1	0.929	ctcgaCACGTGgccccg
AtSK12	P$ABZ1_01	ABZ1	169	183	1	1	0.955	tcgacACGTGgccc
AtSK12	P$HBI1_01	HBI1	169	181	1	1	0.935	tcgaCACGTggc
AtSK12	P$BZR1_02	BZR1	169	183	1	1	0.959	tcgaCACGTggccc
AtSK12	P$ABF1_04	ABF1	170	189	1	1	0.934	cgacACGTGgccccggttc
AtSK12	P$GBF1_Q2_01	GBF1	170	181	1	1	0.989	cgacACGTGgc
AtSK12	P$BZR1_03	BZR1	170	190	1	1	0.877	cgacACGTGgccccggttca
AtSK12	P$ABF4_01	ABF4	170	182	1	1	1	cgaCACGTggcc
AtSK12	P$BIM1_01	BIM1	170	182	1	1	0.954	cgACACGtggcc
AtSK12	P$GBP_Q6	GBP	170	182	1	1	0.978	cgaCACGTggcc
AtSK12	P$GBF_Q2	GBF	170	182	1	1	0.923	cgaCACGTggcc
AtSK12	P$PIF3_03	IF3	170	180	1	1	0.931	cgaCACGTgg
AtSK12	P$ABI5_01	ABI5	170	180	1	1	1	cgaCACGTgg
AtSK12	P$EMBP1_02	EMBP1	171	181	1	1	0.973	gacACGTGgc
AtSK12	P$TAF1_01	TAF1	171	181	1	1	0.992	gacACGTGgc
AtSK12	P$TAF1_Q2	TAF1	171	181	1	1	0.99	gacACGTGgc
AtSK12	P$HBP1A_Q2	HBP1A	171	181	1	1	0.996	gacACGTGgc
AtSK12	P$PIF1_01	IF1	171	181	1	1	1	gacACGTGgc
AtSK12	P$EMBP1_Q2	EMBP1	171	181	1	1	0.966	gaCACGTggc
AtSK12	P$CPRF1_01	CPRF1	171	181	1	1	1	gaCACGTggc
AtSK12	P$CG1_Q6	CG1	171	182	1	1	0.975	gaCACGTggcc
AtSK12	P$TGA1A_Q2	TGA1A	171	181	1	1	0.982	gaCACGTggc
AtSK12	P$TGA1B_Q2	TGA1B	171	181	1	1	0.968	gaCACGTggc
AtSK12	P$O2_02	O2	171	181	1	1	0.965	gaCACGTggc
AtSK12	P$CPRF2_Q2	CPRF2	171	181	1	1	0.997	gaCACGTggc
AtSK12	P$CPRF3_Q2	CPRF3	171	181	1	1	0.995	gaCACGTggc
AtSK12	P$CPRF_Q2	CPRF	171	181	1	1	0.991	gaCACGTggc
AtSK12	P$CPRF3_01	CPRF3	171	181	1	1	0.99	gaCACGTggc
AtSK12	P$CPRF2_01	CPRF2	171	181	1	1	0.997	gaCACGTggc

AtSK12	P$GBF1F_Q2	GBF1F	171	182	1	1	0.91	gaCACGTggcc
AtSK12	P$SPT_01	SPT	171	180	1	1	0.969	gaCACGTgg
AtSK12	P$PHYPA72483_07	HYPA72483	171	181	1	1	0.998	gaCACGTggc
AtSK12	P$PHYPA143875_02	HYPA143875	171	181	1	1	0.997	gaCACGTggc
AtSK12	P$BIM3_01	BIM3	171	181	1	1	0.991	gaCACGTggc
AtSK12	P$BIM2_01	BIM2	171	181	1	1	0.994	gaCACGTggc
AtSK12	P$BHLH104_01	BHLH104	171	181	1	1	0.869	gaCACGTggc
AtSK12	P$BEE2_01	BEE2	171	181	1	1	0.999	gaCACGTggc
AtSK12	P$PIF3_04	IF3	171	181	1	1	0.886	gaCACGTggc
AtSK12	P$BES1_01	BES1	171	182	1	1	0.981	gaCACGTggcc
AtSK12	P$TGA1B_01	TGA1B	171	181	1	1	0.984	gaCACGTggc
AtSK12	P$MYC4_01	MYC4	172	180	1	1	0.953	acACGTGg
AtSK12	P$GBF1_01	GBF1	172	180	1	1	0.973	acACGTGg
AtSK12	P$BIM1_02	BIM1	172	182	1	1	0.996	acACGTGgcc
AtSK12	P$BHLH13_01	BHLH13	172	180	1	1	0.947	acACGTGg
AtSK12	P$ABF4_02	ABF4	172	182	1	1	0.988	acACGTGgcc
AtSK12	P$BZIP68_01	BZIP68	172	181	1	1	0.998	acaCGTGGc
AtSK12	P$HY5_01	HY5	172	182	1	1	0.993	acACGTGgcc
AtSK12	P$ABF3_01	ABF3	172	180	1	1	0.875	ACACGtgg
AtSK12	P$RITA1_01	RITA1	172	179	1	1	0.984	aCACGTg
AtSK12	P$BHLH66_01	BHLH66	172	180	1	1	0.93	aCACGTgg
AtSK12	P$PIF5_01	IF5	172	180	1	1	0.93	aCACGTgg
AtSK12	P$MYC2_01	MYC2	172	180	1	1	0.953	aCACGTgg
AtSK12	P$MYC3_01	MYC3	172	180	1	1	0.994	aCACGTgg
AtSK12	P$TRAB1_Q2	TRAB1	172	183	1	1	0.893	acACGTGgccc
AtSK12	P$BHLH3_01	BHLH3	172	180	1	1	0.952	aCACGTgg
AtSK12	P$UNE10_01	UNE10	172	180	1	1	0.98	aCACGTgg
AtSK12	P$OJ1058_01	OJ1058	172	180	1	1	1	aCACGTgg
AtSK12	P$PHYPA48267_08	HYPA48267	172	180	1	1	0.971	aCACGTgg
AtSK12	P$BHLH34_01	BHLH34	172	180	1	1	0.972	aCACGTgg
AtSK12	P$GBF1_Q2	GBF1	173	182	1	1	0.959	cACGTGgcc
AtSK12	P$CPRF1_Q2	CPRF1	173	183	1	1	0.991	cACGTGgccc
AtSK12	P$PIF4_01	IF4	173	181	1	1	1	CACGTggc
AtSK12	P$OCSBF1_01	OCSBF1	173	178	1	1	1	CACGT
AtSK12	P$ABI5_Q2	ABI5	174	180	1	1	1	ACGTGg
AtSK12	P$ARR2_01	ARR2	193	203	1	1	0.867	tgtaATCTTt
AtSK12	P$SEP3_01	wrz-03	196	207	1	1	0.852	aatctTTTTGg
AtSK12	P$MYB80_01	MYB80	204	215	1	1	0.992	tgGAATAttcc
AtSK12	P$O2_Q4	O2	230	241	1	1	0.89	tatgCATGTat
AtSK12	P$ABI3_01	ABI3	231	240	1	1	0.866	atGCATGta
AtSK12	P$TEIL_01	TEIL	235	243	1	1	0.931	ATGTAtgt
AtSK12	P$TEIL_01	TEIL	239	247	1	1	0.931	ATGTAtgt
AtSK12	P$TEIL_01	TEIL	243	251	1	1	0.922	ATGTAtat
AtSK12	P$C1_Q2	C1	268	279	1	1	0.957	aaAACTAcccc
AtSK12	P$P_01		270	279	1	1	0.921	aaCTACCcc
AtSK12	P$MYB1L_01	MYB1L	274	284	1	1	0.948	acCCCTActt
AtSK12	P$TRB2_01	TRB2	274	282	1	1	0.925	acCCCTAc
AtSK12	P$ARF8_01	ARF8	282	291	1	1	0.956	ttTGTCGtg
AtSK12	P$ID1_01	ID1	282	293	1	1	0.961	tTTGTCgtgtt
AtSK12	P$PCF2_01	CF2	298	308	1	1	0.889	ctgtcCCCAC
AtSK12	P$TCP19_01	TCP19	298	308	1	1	0.904	ctgtcCCCAC
AtSK12	P$TCP20L_01	TCP20L	299	308	1	1	0.884	tgtcCCCAC
AtSK12	P$ARALY484486_05	ARALY484486	300	308	1	1	0.933	gtcCCCAC
AtSK12	P$ARALY495258_02	ARALY495258	300	308	1	1	0.933	gtcCCCAC
AtSK12	P$TCP20_02	TCP20	300	310	1	1	0.909	gtcCCCACtt
AtSK12	P$ID1_01	ID1	316	327	1	1	0.889	tTTGTCcaaat
AtSK12	P$HSFA2_01	HSFA2	321	327	1	1	0.922	CCAAAt
AtSK12	P$ASR1_01	ASR1	337	342	1	1	1	ACCCA
AtSK12	P$SBF1_01	SBF1	343	357	1	1	0.863	ccttctTTAATtat
AtSK12	P$EDT1_01	EDT1	346	356	1	1	0.868	tctTTAATta
AtSK12	P$ATHB1_01	ATHB1	347	361	1	1	0.945	ctttaATTATtaat
AtSK12	P$ATHB5_01	ATHB5	350	359	1	1	0.917	taaTTATTa
AtSK12	P$SBF1_01	SBF1	350	364	1	1	0.942	taattaTTAATatt
AtSK12	P$EDT1_01	EDT1	353	363	1	1	0.857	ttaTTAATat
AtSK12	P$SBF1_01	SBF1	374	388	1	1	0.879	tttcgaTTAATtta
AtSK12	P$EDT1_01	EDT1	377	387	1	1	0.865	cgaTTAATtt
AtSK12	P$RIN_Q2	RIN	380	391	1	1	0.902	ttaaTTTAAga
AtSK12	P$PEND_01	END	386	394	1	1	0.956	tAAGAAct
AtSK12	P$C1_Q2	C1	388	399	1	1	0.919	agAACTAatta
AtSK12	P$C1_Q2	C1	399	410	1	1	0.942	taAACTAtata
AtSK12	P$C1_Q2	C1	464	475	1	1	0.932	tgAACTAgata
AtSK12	P$ARR18_01	ARR18	466	479	1	1	0.944	aactAGATActca
AtSK12	P$AT3G18650_01	AT3G18650	492	503	1	1	0.968	gacaaTTGTAt
AtSK12	P$KNOX3_01	KNOX3	498	510	1	1	0.972	tgtaTGACAttt
AtSK12	P$ATH1_01	ATH1	502	510	1	1	0.933	TGACAttt
AtSK12	P$MYB24_01	MYB24	505	514	1	1	0.864	catTTAGGa
AtSK12	P$ATHB1_01	ATHB1	513	527	1	1	0.976	aagtaATTATtgga
AtSK12	P$ATHB5_01	ATHB5	516	525	1	1	0.933	taaTTATTg

AtSK12	P$PEND_01	END	533	541	1	1	1	tAAGAAgt
AtSK12	P$WRKY48_01	WRKY48	546	555	1	1	0.964	atttAACAA
AtSK12	P$ATMYB15_Q2	ATMYB15	549	555	1	1	1	TAACAa
AtSK12	P$DOF3_01	DOF3	550	561	1	1	0.993	aacaAAAGCac
AtSK12	P$DOF2_01	DOF2	550	561	1	1	0.992	aacaAAAGCac
AtSK12	P$DOF_Q2	DOF	550	561	1	1	0.941	aacAAAAGcac
AtSK12	P$PBF_01	BF	550	561	1	1	0.98	aacAAAAGcac
AtSK12	P$CDF2_01	CDF2	551	561	1	1	0.959	acAAAAGcac
AtSK12	P$CDF3_01	CDF3	552	561	1	1	0.978	cAAAAGcac
AtSK12	P$BPC1_Q2	BPC1	566	572	1	1	0.997	AGAAAa
AtSK12	P$HSFA2_01	HSFA2	578	584	1	1	1	CCAAAa
AtSK12	P$ATHB7_01	ATHB7	590	600	1	1	0.89	gtAATCAata
AtSK12	P$HAT1_01	HAT1	590	600	1	1	0.889	gtAATCAata
AtSK12	P$ATHB6_01	ATHB6	594	603	1	1	0.926	tcAATAAaa
AtSK12	P$ATHB6_01	ATHB6	601	610	1	1	0.905	aaAATAAag
AtSK12	P$DOF1_01	DOF1	602	613	1	1	0.99	aaaTAAAGttc
AtSK12	P$AT4G04450_01	AT4G04450	622	631	1	1	0.866	catTTAGCt
AtSK12	P$KNOX3_01	KNOX3	636	648	1	1	0.965	acttTGACAttg
AtSK12	P$WRKY11_Q2	WRKY11	638	646	1	1	0.903	tTTGACat
AtSK12	P$ATH1_01	ATH1	640	648	1	1	0.921	TGACAttg
AtSK12	P$WRKY11_01	WRKY11	650	664	1	1	0.864	agtaTTGACttctt
AtSK12	P$WRKY11_Q2	WRKY11	653	661	1	1	0.931	aTTGACtt
AtSK12	P$SBF1_01	SBF1	661	675	1	1	0.914	ctttatTTAAAaag
AtSK12	P$PBF_01	BF	667	678	1	1	0.969	ttaAAAAGtta
AtSK12	P$DOF_Q2	DOF	667	678	1	1	0.977	ttaAAAAGtta
AtSK12	P$CDF2_01	CDF2	668	678	1	1	0.993	taAAAAGtta
AtSK12	P$CDF3_01	CDF3	669	678	1	1	0.989	aAAAAGtta
AtSK12	P$MYB24_01	MYB24	672	681	1	1	0.923	aagTTAGGa
AtSK12	P$BPC1_Q2	BPC1	681	687	1	1	0.997	AGAAAa
AtSK12	P$TEIL_01	TEIL	698	706	1	1	0.94	ATGTAgct
AtSK12	P$PBF_01	BF	707	718	1	1	0.962	ctaAAAAGaat
AtSK12	P$DOF_Q2	DOF	707	718	1	1	0.992	ctaAAAAGaat
AtSK12	P$CDF2_01	CDF2	708	718	1	1	0.976	taAAAAGaat
AtSK12	P$CDF3_01	CDF3	709	718	1	1	0.974	aAAAAGaat
AtSK12	P$ARR1_01	ARR1	711	721	1	1	0.953	aaaGAATCaa
AtSK12	P$ATHB7_01	ATHB7	713	723	1	1	0.882	agAATCAata
AtSK12	P$HAT1_01	HAT1	713	723	1	1	0.876	agAATCAata
AtSK12	P$ATHB1_03	ATHB1	717	728	1	1	0.98	tcAATAAttcc
AtSK12	P$ATHB5_04	ATHB5	717	728	1	1	0.991	tcAATAAttcc
AtSK12	P$ATHB6_01	ATHB6	717	726	1	1	1	tcAATAAtt
AtSK12	P$ATHB16_01	ATHB16	718	726	1	1	0.963	cAATAAtt
AtSK12	P$PDF2_01	DF2	732	743	1	1	0.896	aaaaTAAATga
AtSK12	P$ATHB6_01	ATHB6	732	741	1	1	0.908	aaAATAAat
AtSK12	P$ATHB1_01	ATHB1	772	786	1	1	0.93	gctaaATTATttat
AtSK12	P$ATHB5_01	ATHB5	775	784	1	1	0.915	aaaTTATTt
AtSK12	P$SBF1_01	SBF1	782	796	1	1	0.944	ttatggTTAATaca
AtSK12	P$DRE1C_01	DRE1C	800	808	1	1	0.862	ATGTCagt
AtSK12	P$MYBAS1_01	MYBAS1	806	817	1	1	0.977	gtCCAACctat
AtSK12	P$GAMYB_01	GAMYB	809	817	1	1	0.86	CAACCtat
AtSK12	P$WRKY48_01	WRKY48	813	822	1	1	0.881	ctatAACAA
AtSK12	P$GAMYB_Q2	GAMYB	813	826	1	1	0.922	ctataACAACtta
AtSK12	P$ATMYB15_Q2	ATMYB15	816	822	1	1	1	TAACAa
AtSK12	P$ARR18_01	ARR18	824	837	1	1	0.895	tagtAGATAaaaa
AtSK12	P$PBF_01	BF	834	845	1	1	0.977	aaaAAAAGcag
AtSK12	P$DOF_Q2	DOF	834	845	1	1	0.997	aaaAAAAGcag
AtSK12	P$DOF2_01	DOF2	834	845	1	1	0.994	aaaaAAAGCag
AtSK12	P$DOF3_01	DOF3	834	845	1	1	0.991	aaaaAAAGCag
AtSK12	P$CDF2_01	CDF2	835	845	1	1	0.988	aaAAAAGcag
AtSK12	P$CDF3_01	CDF3	836	845	1	1	0.983	aAAAAGcag
AtSK12	P$REF6_01	REF6	838	849	1	1	0.881	aaagCAGAGaa
AtSK12	P$BPC1_Q2	BPC1	848	854	1	1	0.997	AGAAAa
AtSK12	P$AZF2_01	AZF2	854	866	1	1	0.905	acgtttAAGTAg
AtSK12	P$MYB3_01	MYB3	860	871	1	1	0.929	aagTAGGTata
AtSK12	P$MYB4_01	MYB4	861	869	1	1	0.95	agTAGGTa
AtSK12	P$ATHB6_01	ATHB6	877	886	1	1	0.902	cgAATAAag
AtSK12	P$DOF1_01	DOF1	878	889	1	1	0.973	gaaTAAAGtgg
AtSK12	P$ALFIN1_Q2	ALFIN1	879	894	1	1	0.882	aataaaGTGGGaggg
AtSK12	P$DOF_Q2	DOF	900	911	1	1	0.938	ggcAAAAGaag
AtSK12	P$PBF_01	BF	900	911	1	1	0.953	ggcAAAAGaag
AtSK12	P$CDF2_01	CDF2	901	911	1	1	0.946	gcAAAAGaag
AtSK12	P$CDF3_01	CDF3	902	911	1	1	0.968	cAAAAGaag
AtSK12	P$PEND_01	END	904	912	1	1	0.891	aAAGAAgt
AtSK13	P$ARR1_01	ARR1	18	28	1	1	0.946	attGAATCat
AtSK13	P$HAT1_01	HAT1	20	30	1	1	0.98	tgAATCAttt
AtSK13	P$ATHB7_01	ATHB7	20	30	1	1	0.923	tgAATCAttt
AtSK13	P$ATHB4_02	ATHB4	21	31	1	1	0.924	gaATCATttt
AtSK13	P$SPF1_Q2	SPF1	30	40	1	1	0.894	taATAGTgac
AtSK13	P$HSFA4A_01	HSFA4A	38	44	1	1	1	aCTATT

AtSK13	P$GT1_Q6_01	GT1	42	54	1	1	0.873	TTTGTttttggt
AtSK13	P$SEP3_01	wrz-03	42	53	1	1	0.852	tttgtTTTTGg
AtSK13	P$GT1_Q6_01	GT1	46	58	1	1	0.899	TTTTTggtcaac
AtSK13	P$WRKY60_01	WRKY60	49	60	1	1	0.994	ttgGTCAActt
AtSK13	P$WRKY57_01	WRKY57	49	59	1	1	0.984	ttgGTCAAct
AtSK13	P$WRKY48_02	WRKY48	49	59	1	1	1	ttgGTCAAct
AtSK13	P$WRKY21_02	WRKY21	49	59	1	1	0.996	ttgGTCAAct
AtSK13	P$WRKY18_02	WRKY18	49	59	1	1	0.997	ttgGTCAAct
AtSK13	P$WRKY8_01	WRKY8	50	59	1	1	0.998	tgGTCAAct
AtSK13	P$WRKY75_01	WRKY75	50	58	1	1	0.998	tgGTCAAc
AtSK13	P$WRKY63_01	WRKY63	50	58	1	1	0.999	tgGTCAAc
AtSK13	P$WRKY62_01	WRKY62	50	58	1	1	1	tgGTCAAc
AtSK13	P$WRKY43_02	WRKY43	50	60	1	1	0.983	tgGTCAActt
AtSK13	P$WRKY40_01	WRKY40	50	58	1	1	1	tgGTCAAc
AtSK13	P$WRKY25_02	WRKY25	50	58	1	1	0.994	tgGTCAAc
AtSK13	P$WRKY2_01	WRKY2	50	58	1	1	0.998	tgGTCAAc
AtSK13	P$WRKY15_01	WRKY15	50	60	1	1	0.997	tgGTCAActt
AtSK13	P$WRKY23_01	WRKY23	51	59	1	1	0.916	gGTCAAct
AtSK13	P$WRKY30_01	WRKY30	51	61	1	1	0.992	gGTCAActtc
AtSK13	P$WRKY18_Q2	WRKY18	52	61	1	1	0.969	GTCAActtc
AtSK13	P$EDT1_01	EDT1	62	72	1	1	0.948	ataTTAATgt
AtSK13	P$LEC2_01	LEC2	74	85	1	1	0.98	taCATGCatcg
AtSK13	P$FUS3_Q2	FUS3	75	86	1	1	0.878	aCATGCatcgt
AtSK13	P$IDEF1_Q2	IDEF1	76	88	1	1	0.901	CATGCatcgtgt
AtSK13	P$HMG1_01	HMG1	86	95	1	1	0.873	GTTGTatac
AtSK13	P$AT3G18650_01	AT3G18650	90	101	1	1	0.856	tatacTTGTAt
AtSK13	P$ANAC059_01	ANAC059	93	110	1	1	0.87	acttgtattgcACGCAa
AtSK13	P$NAC083_01	NAC083	102	112	1	1	0.967	gcACGCAatt
AtSK13	P$NAC080_01	NAC080	102	110	1	1	0.97	gCACGCaa
AtSK13	P$GT1_Q6	GT1	114	121	1	1	1	GTGAAta
AtSK13	P$TGA1_02	TGA1	114	129	1	1	0.854	gtgaataACGTCaat
AtSK13	P$ATHB6_01	ATHB6	115	124	1	1	0.898	tgAATAAcg
AtSK13	P$TGA7_02	TGA7	116	133	1	1	0.875	gaataACGTCaatcgtg
AtSK13	P$WRKY60_01	WRKY60	120	131	1	1	0.884	aacGTCAAtcg
AtSK13	P$WRKY57_01	WRKY57	120	130	1	1	0.96	aacGTCAAtc
AtSK13	P$WRKY48_02	WRKY48	120	130	1	1	0.987	aacGTCAAtc
AtSK13	P$WRKY21_02	WRKY21	120	130	1	1	0.949	aacGTCAAtc
AtSK13	P$WRKY18_02	WRKY18	120	130	1	1	0.946	aacGTCAAtc
AtSK13	P$TGA1A_01	TGA1A	120	127	1	1	0.989	aACGTCa
AtSK13	P$WRKY8_01	WRKY8	121	130	1	1	0.978	acGTCAAtc
AtSK13	P$WRKY75_01	WRKY75	121	129	1	1	0.928	acGTCAAt
AtSK13	P$WRKY63_01	WRKY63	121	129	1	1	0.885	acGTCAAt
AtSK13	P$WRKY43_02	WRKY43	121	131	1	1	0.955	acGTCAAtcg
AtSK13	P$WRKY40_01	WRKY40	121	129	1	1	0.981	acGTCAAt
AtSK13	P$WRKY25_02	WRKY25	121	129	1	1	0.879	acGTCAAt
AtSK13	P$WRKY2_01	WRKY2	121	129	1	1	0.904	acGTCAAt
AtSK13	P$WRKY15_01	WRKY15	121	131	1	1	0.96	acGTCAAtcg
AtSK13	P$WRKY30_01	WRKY30	122	132	1	1	0.91	cGTCAAtcgt
AtSK13	P$WRKY18_Q2	WRKY18	123	132	1	1	0.933	GTCAAtcgt
AtSK13	P$ATSPL8_01	ATSPL8	126	142	1	1	0.941	aatcgTGTACgaaatt
AtSK13	P$BHLH28_01	BHLH28	128	140	1	1	0.869	tcgtGTACGaaa
AtSK13	P$SPL11_01	SPL11	128	140	1	1	0.905	tcgtGTACGaaa
AtSK13	P$SPL5_01	SPL5	130	139	1	1	0.972	gtGTACGaa
AtSK13	P$POPTR_01	OPTR	131	138	1	1	0.943	tGTACGa
AtSK13	P$SPL12_01	SPL12	131	139	1	1	0.983	tGTACGaa
AtSK13	P$SPL4_01	SPL4	131	140	1	1	0.994	tGTACGaaa
AtSK13	P$ARF8_01	ARF8	148	157	1	1	0.956	ttTGTCGta
AtSK13	P$ID1_01	ID1	148	159	1	1	0.902	tTTGTCgtaac
AtSK13	P$GT1_Q6_01	GT1	148	160	1	1	0.856	TTTGTcgtaacc
AtSK13	P$WRKY26_01	WRKY26	152	161	1	1	0.935	tcgTAACCa
AtSK13	P$AT1G69310_01	AT1G69310	152	161	1	1	0.904	tcgTAACCa
AtSK13	P$AT3G62340_01	AT3G62340	152	161	1	1	0.908	tcgTAACCa
AtSK13	P$AT1G29860_01	AT1G29860	152	161	1	1	0.887	tcgTAACCa
AtSK13	P$WRKY33_01	WRKY33	152	161	1	1	0.935	tcgTAACCa
AtSK13	P$WRKY25_01	WRKY25	152	161	1	1	0.918	tcgTAACCa
AtSK13	P$NAC6_01	NAC6	152	158	1	1	0.854	tCGTAA
AtSK13	P$AT4G36620_01	AT4G36620	153	161	1	1	0.874	cgtAACCA
AtSK13	P$AT3G60580_01	AT3G60580	189	196	1	1	0.883	aaATCCC
AtSK13	P$ATHB1_01	ATHB1	199	213	1	1	0.939	tggcaATTATttat
AtSK13	P$ATHB5_01	ATHB5	202	211	1	1	0.987	caaTTATTt
AtSK13	P$AT1G59810_01	AT1G59810	227	243	1	1	0.895	aattTAAATtcaaaaa
AtSK13	P$AGL20_01	AGL20	229	241	1	1	0.897	ttTAAATtcaaa
AtSK13	P$PBF_01	BF	236	247	1	1	0.977	tcaAAAAGtgc
AtSK13	P$DOF_Q2	DOF	236	247	1	1	0.981	tcaAAAAGtgc
AtSK13	P$CDF2_01	CDF2	237	247	1	1	1	caAAAAGtgc
AtSK13	P$CDF3_01	CDF3	238	247	1	1	1	aAAAAGtgc
AtSK13	P$ATHB4_02	ATHB4	259	269	1	1	0.887	ttATCATtta
AtSK13	P$RIN_Q2	RIN	261	272	1	1	0.853	atcaTTTAAga

AtSK13	P$BPC1_Q2	BPC1	271	277	1	1	1	AGAAAg
AtSK13	P$RIN_Q2	RIN	294	305	1	1	0.865	ttcaTTTAAgg
AtSK13	P$AT4G00870_01	AT4G00870	308	322	1	1	0.861	atCCTCGtgtaaga
AtSK13	P$MYBAS1_01	MYBAS1	325	336	1	1	0.991	caCCAACggtc
AtSK13	P$AT5G54070_01	AT5G54070	327	333	1	1	1	cCAACG
AtSK13	P$ARR1_01	ARR1	333	343	1	1	0.957	gtcGAATCag
AtSK13	P$HAT1_01	HAT1	335	345	1	1	0.854	cgAATCAgac
AtSK13	P$NAC92_01	NAC92	341	353	1	1	0.989	agACACGacatc
AtSK13	P$C1_Q2	C1	359	370	1	1	0.958	cgAACTAaatc
AtSK13	P$ARR2_01	ARR2	363	373	1	1	0.894	ctaaATCTTg
AtSK13	P$ARF8_01	ARF8	369	378	1	1	0.96	ctTGTCGct
AtSK13	P$ID1_01	ID1	369	380	1	1	0.904	cTTGTCgcttt
AtSK13	P$O2_03	O2	384	394	1	1	0.955	GATGActtgg
AtSK13	P$NAC6_01	NAC6	399	405	1	1	0.854	tCGTAA
AtSK13	P$SED_Q2	SED	410	420	1	1	0.997	atttCCTTTt
AtSK13	P$AGL15_03	AGL15	411	426	1	1	0.866	TTTCCttttctgaca
AtSK13	P$PBF_Q2_01	BF	414	420	1	1	1	CCTTTt
AtSK13	P$KNOX3_01	KNOX3	417	429	1	1	0.974	tttcTGACAaaa
AtSK13	P$ATH1_01	ATH1	421	429	1	1	0.923	TGACAaaa
AtSK13	P$GAMYB_Q2	GAMYB	423	436	1	1	0.895	acaaaACAACatt
AtSK13	P$RAV1_01	RAV1	426	438	1	1	0.943	aaaCAACAttac
AtSK13	P$GT1_Q6_02	GT1	437	449	1	1	0.881	cacaatTTAACa
AtSK13	P$WRKY48_01	WRKY48	441	450	1	1	0.964	atttAACAA
AtSK13	P$ATMYB15_Q2	ATMYB15	444	450	1	1	1	TAACAa
AtSK13	P$AT4G12750_01	AT4G12750	449	459	1	1	0.883	aatACCGAaa
AtSK13	P$AT2G41690_01	AT2G41690	453	459	1	1	0.978	CCGAAa
AtSK13	P$PBF_01	BF	454	465	1	1	0.977	cgaAAAAGtga
AtSK13	P$DOF_Q2	DOF	454	465	1	1	0.981	cgaAAAAGtga
AtSK13	P$CDF2_01	CDF2	455	465	1	1	0.999	gaAAAAGtga
AtSK13	P$CDF3_01	CDF3	456	465	1	1	1	aAAAAGtga
AtSK13	P$GT1_Q6	GT1	461	468	1	1	0.912	GTGAAga
AtSK13	P$ARR1_01	ARR1	469	479	1	1	0.987	cgaGAATCtg
AtSK13	P$ATSPL8_01	ATSPL8	474	490	1	1	0.909	atctgTGTACgccacg
AtSK13	P$SPL11_01	SPL11	476	488	1	1	0.884	ctgtGTACGcca
AtSK13	P$SPL5_01	SPL5	478	487	1	1	0.965	gtGTACGcc
AtSK13	P$POPTR_01	OPTR	479	486	1	1	0.928	tGTACGc
AtSK13	P$SPL12_01	SPL12	479	487	1	1	0.971	tGTACGcc
AtSK13	P$SPL4_01	SPL4	479	488	1	1	0.988	tGTACGcca
AtSK13	P$AT1G66560_01	AT1G66560	493	503	1	1	0.914	cgcTTAACgt
AtSK13	P$WRKY7_01	WRKY7	494	503	1	1	0.889	gcTTAACgt
AtSK13	P$AT2G24570_01	AT2G24570	494	503	1	1	0.887	gcTTAACgt
AtSK13	P$WRKY46_01	WRKY46	494	503	1	1	0.907	gcTTAACgt
AtSK13	P$AT5G15130_01	AT5G15130	494	503	1	1	0.918	gcTTAACgt
AtSK13	P$AT5G41570_01	AT5G41570	494	503	1	1	0.89	gcTTAACgt
AtSK13	P$AT1G68150_01	AT1G68150	494	503	1	1	0.89	gcTTAACgt
AtSK13	P$AT1G66600_01	AT1G66600	494	503	1	1	0.917	gcTTAACgt
AtSK13	P$WRKY6_01	WRKY6	494	503	1	1	0.864	gcTTAACgt
AtSK13	P$AT1G64000_01	AT1G64000	494	503	1	1	0.89	gcTTAACgt
AtSK13	P$AT1G18860_01	AT1G18860	494	503	1	1	0.923	gcTTAACgt
AtSK13	P$WRKY21_01	WRKY21	494	503	1	1	0.89	gcTTAACgt
AtSK13	P$MYBAS1_01	MYBAS1	512	523	1	1	0.977	ctCTAACtagt
AtSK13	P$C1_Q2	C1	514	525	1	1	0.965	ctAACTAgtag
AtSK13	P$AT1G15360_01	AT1G15360	526	536	1	1	0.939	cgTCCGAtta
AtSK13	P$MYBAS1_01	MYBAS1	559	570	1	1	0.985	atCTAACcgtc
AtSK13	P$WRKY60_01	WRKY60	564	575	1	1	0.889	accGTCAAtta
AtSK13	P$WRKY57_01	WRKY57	564	574	1	1	0.956	accGTCAAtt
AtSK13	P$WRKY48_02	WRKY48	564	574	1	1	0.987	accGTCAAtt
AtSK13	P$WRKY21_02	WRKY21	564	574	1	1	0.949	accGTCAAtt
AtSK13	P$WRKY18_02	WRKY18	564	574	1	1	0.945	accGTCAAtt
AtSK13	P$WRKY8_01	WRKY8	565	574	1	1	0.977	ccGTCAAtt
AtSK13	P$WRKY75_01	WRKY75	565	573	1	1	0.928	ccGTCAAt
AtSK13	P$WRKY63_01	WRKY63	565	573	1	1	0.887	ccGTCAAt
AtSK13	P$WRKY43_02	WRKY43	565	575	1	1	0.953	ccGTCAAtta
AtSK13	P$WRKY40_01	WRKY40	565	573	1	1	0.981	ccGTCAAt
AtSK13	P$WRKY25_02	WRKY25	565	573	1	1	0.894	ccGTCAAt
AtSK13	P$WRKY2_01	WRKY2	565	573	1	1	0.907	ccGTCAAt
AtSK13	P$WRKY15_01	WRKY15	565	575	1	1	0.959	ccGTCAAtta
AtSK13	P$WRKY30_01	WRKY30	566	576	1	1	0.908	cGTCAAttag
AtSK13	P$WRKY18_Q2	WRKY18	567	576	1	1	0.936	GTCAAttag
AtSK13	P$AT1G66560_01	AT1G66560	590	600	1	1	0.942	cacTTAACcg
AtSK13	P$AT1G30650_01	AT1G30650	591	600	1	1	0.95	acTTAACcg
AtSK13	P$AT2G24570_01	AT2G24570	591	600	1	1	0.947	acTTAACcg
AtSK13	P$AT4G23550_01	AT4G23550	591	600	1	1	0.95	acTTAACcg
AtSK13	P$WRKY7_01	WRKY7	591	600	1	1	0.95	acTTAACcg
AtSK13	P$WRKY25_01	WRKY25	591	600	1	1	0.966	actTAACCg
AtSK13	P$WRKY33_01	WRKY33	591	600	1	1	0.88	actTAACCg
AtSK13	P$AT1G29860_01	AT1G29860	591	600	1	1	0.918	actTAACCg
AtSK13	P$AT3G62340_01	AT3G62340	591	600	1	1	0.902	actTAACCg

AtSK13	P$AT1G69310_01	AT1G69310	591	600	1	1	0.932	actTAACCg
AtSK13	P$WRKY26_01	WRKY26	591	600	1	1	0.884	actTAACCg
AtSK13	P$WRKY46_01	WRKY46	591	600	1	1	0.887	acTTAACcg
AtSK13	P$AT5G15130_01	AT5G15130	591	600	1	1	0.966	acTTAACcg
AtSK13	P$WRKY21_01	WRKY21	591	600	1	1	0.947	acTTAACcg
AtSK13	P$AT2G34830_01	AT2G34830	591	600	1	1	0.95	acTTAACcg
AtSK13	P$AT1G18860_01	AT1G18860	591	600	1	1	0.966	acTTAACcg
AtSK13	P$AT1G64000_01	AT1G64000	591	600	1	1	0.935	acTTAACcg
AtSK13	P$AT4G22070_01	AT4G22070	591	600	1	1	0.948	acTTAACcg
AtSK13	P$WRKY6_01	WRKY6	591	600	1	1	0.945	acTTAACcg
AtSK13	P$AT1G66600_01	AT1G66600	591	600	1	1	0.94	acTTAACcg
AtSK13	P$AT1G68150_01	AT1G68150	591	600	1	1	0.959	acTTAACcg
AtSK13	P$AT5G41570_01	AT5G41570	591	600	1	1	0.934	acTTAACcg
AtSK13	P$AT1G69810_01	AT1G69810	591	600	1	1	0.962	acTTAACcg
AtSK13	P$GT1_01	GT1	592	600	1	1	0.946	ctTAACCg
AtSK13	P$ARR18_01	ARR18	602	615	1	1	0.904	ggaaAGATAttca
AtSK13	P$AT4G36620_01	AT4G36620	613	621	1	1	0.895	caaAACCA
AtSK13	P$RIN_Q2_01	RIN	617	629	1	1	0.948	accattAAAAGc
AtSK13	P$DOF3_01	DOF3	620	631	1	1	0.98	attaAAAGCag
AtSK13	P$DOF2_01	DOF2	620	631	1	1	0.993	attaAAAGCag
AtSK13	P$DOF_Q2	DOF	620	631	1	1	0.95	attAAAAGcag
AtSK13	P$PBF_01	BF	620	631	1	1	0.975	attAAAAGcag
AtSK13	P$CDF2_01	CDF2	621	631	1	1	0.96	ttAAAAGcag
AtSK13	P$CDF3_01	CDF3	622	631	1	1	0.978	tAAAAGcag
AtSK13	P$WRKY60_01	WRKY60	627	638	1	1	0.926	gcaGTCAAcac
AtSK13	P$WRKY57_01	WRKY57	627	637	1	1	0.992	gcaGTCAAca
AtSK13	P$WRKY48_02	WRKY48	627	637	1	1	0.997	gcaGTCAAca
AtSK13	P$WRKY21_02	WRKY21	627	637	1	1	0.995	gcaGTCAAca
AtSK13	P$WRKY18_02	WRKY18	627	637	1	1	0.981	gcaGTCAAca
AtSK13	P$WRKY40_03	WRKY40	627	637	1	1	0.994	gcAGTCAaca
AtSK13	P$WRKY8_01	WRKY8	628	637	1	1	0.997	caGTCAAca
AtSK13	P$WRKY75_01	WRKY75	628	636	1	1	1	caGTCAAc
AtSK13	P$WRKY63_01	WRKY63	628	636	1	1	0.915	caGTCAAc
AtSK13	P$WRKY62_01	WRKY62	628	636	1	1	0.918	caGTCAAc
AtSK13	P$WRKY43_02	WRKY43	628	638	1	1	0.998	caGTCAAcac
AtSK13	P$WRKY40_01	WRKY40	628	636	1	1	0.996	caGTCAAc
AtSK13	P$WRKY25_02	WRKY25	628	636	1	1	0.948	caGTCAAc
AtSK13	P$WRKY2_01	WRKY2	628	636	1	1	0.951	caGTCAAc
AtSK13	P$WRKY15_01	WRKY15	628	638	1	1	0.992	caGTCAAcac
AtSK13	P$RAV1_01	RAV1	629	641	1	1	0.954	agtCAACAcaaa
AtSK13	P$WRKY30_01	WRKY30	629	639	1	1	0.99	aGTCAAcaca
AtSK13	P$WRKY23_01	WRKY23	629	637	1	1	0.961	aGTCAAca
AtSK13	P$WRKY18_Q2	WRKY18	630	639	1	1	0.93	GTCAAcaca
AtSK13	P$PIL5_01	IL5	635	649	1	1	0.857	cacaaatgACGTGa
AtSK13	P$O2_Q2	O2	637	650	1	1	0.92	caaatGACGTgag
AtSK13	P$TGA1_01	TGA1	638	649	1	1	0.98	aaaTGACGtga
AtSK13	P$ABZ1_01	ABZ1	638	652	1	1	0.88	aaatgACGTGagtc
AtSK13	P$STF1_01	STF1	639	651	1	1	0.968	aatGACGTgagt
AtSK13	P$BZIP14_01	BZIP14	639	649	1	1	0.961	aaTGACGtga
AtSK13	P$TGA6_02	TGA6	639	654	1	1	0.879	aaTGACGtgagtctt
AtSK13	P$TGA5_02	TGA5	639	653	1	1	0.867	aaTGACGtgagtct
AtSK13	P$TGA7_01	TGA7	639	649	1	1	0.963	aaTGACGtga
AtSK13	P$TGA6_01	TGA6	639	649	1	1	0.977	aaTGACGtga
AtSK13	P$TGA3_Q2	TGA3	639	648	1	1	0.974	aaTGACGtg
AtSK13	P$STF1_02	STF1	639	651	1	1	0.975	aaTGACGtgagt
AtSK13	P$EMBP1_02	EMBP1	640	650	1	1	0.876	atgACGTGag
AtSK13	P$TAF1_01	TAF1	640	650	1	1	0.954	atgACGTGag
AtSK13	P$TGA5_01	TGA5	640	648	1	1	0.999	aTGACGtg
AtSK13	P$TGA1B_01	TGA1B	640	650	1	1	0.906	atGACGTgag
AtSK13	P$HBP1A_Q2	HBP1A	640	650	1	1	0.88	atgACGTGag
AtSK13	P$TAF1_Q2	TAF1	640	650	1	1	0.932	atgACGTGag
AtSK13	P$ABF4_02	ABF4	641	651	1	1	0.944	tgACGTGagt
AtSK13	P$BIM1_02	BIM1	641	651	1	1	0.948	tgACGTGagt
AtSK13	P$GBF1_01	GBF1	641	649	1	1	0.948	tgACGTGa
AtSK13	P$TRAB1_Q2	TRAB1	641	652	1	1	0.93	tgACGTGagtc
AtSK13	P$ABI5_Q2	ABI5	643	649	1	1	0.936	ACGTGa
AtSK22	P$GT1_Q6_01	GT1	9	21	1	1	0.879	TTTTTgtatagt
AtSK22	P$CBNAC_01	CBNAC	36	42	1	1	0.973	cTGCTT
AtSK22	P$SBF1_01	SBF1	37	51	1	1	0.876	tgcttgTTAATctt
AtSK22	P$ARR2_01	ARR2	42	52	1	1	0.868	gttaATCTTa
AtSK22	P$NAC6_01	NAC6	57	63	1	1	0.854	tCGTAA
AtSK22	P$C1_Q2	C1	60	71	1	1	0.93	taAACTAgtct
AtSK22	P$AT4G04450_01	AT4G04450	82	91	1	1	0.867	tttTTAGCc
AtSK22	P$SED_Q2	SED	86	96	1	1	0.883	tagcCCTTTg
AtSK22	P$PBF_Q2_01	BF	90	96	1	1	0.988	CCTTTg
AtSK22	P$AT2G28700_01	AT2G28700	98	112	1	1	0.9	actTAAATtttatc
AtSK22	P$AGL20_01	AGL20	99	111	1	1	0.886	ctTAAATtttat
AtSK22	P$AGL12_01	AGL12	99	111	1	1	0.925	cttAAATTttat

AtSK22	P$AT2G26320_01	AT2G26320	100	111	1	1	0.965	TTAAAttttat
AtSK22	P$ARR2_01	ARR2	105	115	1	1	0.859	ttttATCTTt
AtSK22	P$MYBAS1_01	MYBAS1	122	133	1	1	0.96	tgCTAACtgta
AtSK22	P$PEND_02	END	132	142	1	1	0.902	aaTTCTTttg
AtSK22	P$WEREWOLF_Q2	WEREWOLF	143	152	1	1	0.895	tgGTTAGtg
AtSK22	P$PBF_01	BF	155	166	1	1	0.952	tgaAAAAGaat
AtSK22	P$DOF_Q2	DOF	155	166	1	1	0.989	tgaAAAAGaat
AtSK22	P$CDF2_01	CDF2	156	166	1	1	0.977	gaAAAAGaat
AtSK22	P$CDF3_01	CDF3	157	166	1	1	0.974	aAAAAGaat
AtSK22	P$ARR1_01	ARR1	159	169	1	1	0.989	aaaGAATCta
AtSK22	P$C1_Q2	C1	172	183	1	1	0.942	ttAACTAttgt
AtSK22	P$HSFA4A_01	HSFA4A	175	181	1	1	1	aCTATT
AtSK22	P$PBF_Q2	BF	187	193	1	1	0.958	cAAAGG
AtSK22	P$RAV1_01	RAV1	190	202	1	1	0.959	aggCAACActtc
AtSK22	P$DOF1_01	DOF1	203	214	1	1	0.972	ggcTAAAGgga
AtSK22	P$PBF_Q2	BF	206	212	1	1	0.986	tAAAGG
AtSK22	P$RIN_Q2_01	RIN	213	225	1	1	0.912	acaaagAAAAGt
AtSK22	P$PBF_01	BF	216	227	1	1	0.969	aagAAAAGtct
AtSK22	P$DOF_Q2	DOF	216	227	1	1	0.944	aagAAAAGtct
AtSK22	P$BPC1_Q2	BPC1	217	223	1	1	0.997	AGAAAa
AtSK22	P$CDF2_01	CDF2	217	227	1	1	0.965	agAAAAGtct
AtSK22	P$CDF3_01	CDF3	218	227	1	1	0.982	gAAAAGtct
AtSK22	P$SBF1_01	SBF1	234	248	1	1	0.921	ttaagcTTAATatc
AtSK22	P$AZF3_01	AZF3	268	279	1	1	0.898	gAGTATtcatt
AtSK22	P$ATHB1_01	ATHB1	285	299	1	1	0.86	ataccATTATtatt
AtSK22	P$ATHB5_01	ATHB5	288	297	1	1	0.917	ccaTTATTa
AtSK22	P$EDT1_01	EDT1	308	318	1	1	0.968	tatTTAATgg
AtSK22	P$MYBAS1_01	MYBAS1	320	331	1	1	0.948	ttCTAACtcaa
AtSK22	P$AT5G26170_01	AT5G26170	325	334	1	1	0.924	acTCAACtt
AtSK22	P$MYBPH3_02	MYBPH3	335	348	1	1	0.86	gtaattTAGTTat
AtSK22	P$PHV_02	HV	343	358	1	1	0.907	gttATCATtaaatgc
AtSK22	P$ATHB4_02	ATHB4	344	354	1	1	0.892	ttATCATtaa
AtSK22	P$SBF1_01	SBF1	344	358	1	1	0.869	ttatcaTTAAAtgc
AtSK22	P$ML1_01	ML1	346	358	1	1	0.927	atcatTAAATgc
AtSK22	P$PDF2_01	DF2	347	358	1	1	0.958	tcatTAAATgc
AtSK22	P$HDG9_01	HDG9	347	361	1	1	0.896	tcatTAAATgctaa
AtSK22	P$HDG7_01	HDG7	347	358	1	1	0.968	tCATTAaatgc
AtSK22	P$CBNAC_01	CBNAC	385	391	1	1	0.979	gTGCTT
AtSK22	P$CBNAC_02	CBNAC	385	401	1	1	0.867	gTGCTTtatcaattaa
AtSK22	P$SBF1_01	SBF1	391	405	1	1	0.899	tatcaaTTAAAaag
AtSK22	P$PBF_01	BF	397	408	1	1	0.967	ttaAAAAGtag
AtSK22	P$DOF_Q2	DOF	397	408	1	1	0.993	ttaAAAAGtag
AtSK22	P$CDF2_01	CDF2	398	408	1	1	0.996	taAAAAGtag
AtSK22	P$CDF3_01	CDF3	399	408	1	1	0.99	aAAAAGtag
AtSK22	P$MYB3R5_01	MYB3R5	412	427	1	1	0.916	ctcattggaCCGTTa
AtSK22	P$MYB3R4_01	MYB3R4	413	428	1	1	0.93	tcattggaCCGTTac
AtSK22	P$MYB3R1_01	MYB3R1	413	428	1	1	0.948	tcattggaCCGTTac
AtSK22	P$MYB118_01	MYB118	414	431	1	1	0.968	cattggaccGTTACatt
AtSK22	P$FAR1_01	FAR1	427	442	1	1	0.984	cattcACGCGctcta
AtSK22	P$FHY3_01	FHY3	429	441	1	1	0.999	ttcACGCGctct
AtSK23	P$PEND_01	END	13	21	1	1	0.87	cAAGAAat
AtSK23	P$BPC1_Q2	BPC1	15	21	1	1	0.99	AGAAAt
AtSK23	P$GATA15_01	GATA15	25	34	1	1	1	taTGATCgt
AtSK23	P$AT3G62240_01	AT3G62240	29	39	1	1	0.871	atcgtCATTC
AtSK23	P$AMS_01	AMS	40	50	1	1	0.877	tcCATGTgct
AtSK23	P$SED_Q2	SED	81	91	1	1	0.952	ccatCCTTTa
AtSK23	P$PBF_Q2_01	BF	85	91	1	1	0.998	CCTTTa
AtSK23	P$AGL12_01	AGL12	87	99	1	1	0.861	tttAAATTggac
AtSK23	P$HSFA2_01	HSFA2	98	104	1	1	0.922	CCAAAt
AtSK23	P$PBF_01	BF	104	115	1	1	0.977	attAAAAGtca
AtSK23	P$DOF_Q2	DOF	104	115	1	1	0.936	attAAAAGtca
AtSK23	P$CDF2_01	CDF2	105	115	1	1	0.964	ttAAAAGtca
AtSK23	P$CDF3_01	CDF3	106	115	1	1	0.982	tAAAAGtca
AtSK23	P$WRKY60_01	WRKY60	108	119	1	1	0.903	aaaGTCAAata
AtSK23	P$WRKY57_01	WRKY57	108	118	1	1	0.987	aaaGTCAAat
AtSK23	P$WRKY48_02	WRKY48	108	118	1	1	0.995	aaaGTCAAat
AtSK23	P$WRKY21_02	WRKY21	108	118	1	1	0.969	aaaGTCAAat
AtSK23	P$WRKY18_02	WRKY18	108	118	1	1	0.978	aaaGTCAAat
AtSK23	P$WRKY40_03	WRKY40	108	118	1	1	1	aaAGTCAaat
AtSK23	P$WRKY8_01	WRKY8	109	118	1	1	0.991	aaGTCAAat
AtSK23	P$WRKY75_01	WRKY75	109	117	1	1	0.977	aaGTCAAa
AtSK23	P$WRKY63_01	WRKY63	109	117	1	1	0.905	aaGTCAAa
AtSK23	P$WRKY62_01	WRKY62	109	117	1	1	0.875	aaGTCAAa
AtSK23	P$WRKY43_02	WRKY43	109	119	1	1	0.99	aaGTCAAata
AtSK23	P$WRKY40_01	WRKY40	109	117	1	1	0.996	aaGTCAAa
AtSK23	P$WRKY25_02	WRKY25	109	117	1	1	0.912	aaGTCAAa
AtSK23	P$WRKY2_01	WRKY2	109	117	1	1	0.939	aaGTCAAa
AtSK23	P$WRKY15_01	WRKY15	109	119	1	1	0.98	aaGTCAAata

AtSK23	P$WRKY30_01	WRKY30	110	120	1	1	0.915	aGTCAAatat
AtSK23	P$WRKY23_01	WRKY23	110	118	1	1	0.88	aGTCAAat
AtSK23	P$WRKY18_Q2	WRKY18	111	120	1	1	0.954	GTCAAatat
AtSK23	P$SQUA_01	SQUA	145	155	1	1	0.867	caaTTTTTtt
AtSK23	P$GT1_Q6_01	GT1	149	161	1	1	0.888	TTTTTttctaaa
AtSK23	P$GT1_Q6_01	GT1	150	162	1	1	0.887	TTTTTtctaaaa
AtSK23	P$KNOX3_01	KNOX3	158	170	1	1	0.961	aaaaTGACAaaa
AtSK23	P$ATH1_01	ATH1	162	170	1	1	0.923	TGACAaaa
AtSK23	P$UIF1_01	UIF1	175	185	1	1	0.996	gaaGATTCaa
AtSK23	P$UIF1_01	UIF1	182	192	1	1	0.995	caaGATTCag
AtSK23	P$BPC1_Q2	BPC1	190	196	1	1	0.997	AGAAAa
AtSK23	P$NAC043_01	NAC043	198	208	1	1	0.89	catACGTAac
AtSK23	P$NAC025_01	NAC025	200	208	1	1	1	tACGTAac
AtSK23	P$NAC6_01	NAC6	201	207	1	1	1	aCGTAA
AtSK23	P$NAC043_01	NAC043	203	213	1	1	0.994	gtaACGTAag
AtSK23	P$NAC025_01	NAC025	205	213	1	1	0.907	aACGTAag
AtSK23	P$NAC6_01	NAC6	206	212	1	1	1	aCGTAA
AtSK23	P$ATHB6_01	ATHB6	247	256	1	1	0.902	cgAATAAaa
AtSK23	P$SBF1_01	SBF1	254	268	1	1	0.915	aaactgTTAAAaaa
AtSK23	P$C1_Q2	C1	265	276	1	1	0.914	aaAACTAgaat
AtSK23	P$AT3G20750_01	AT3G20750	283	291	1	1	0.85	gTAAACaa
AtSK23	P$GT1_Q6	GT1	283	290	1	1	0.912	GTAAACa
AtSK23	P$ARR18_01	ARR18	298	311	1	1	0.894	tagtAGATAgatt
AtSK23	P$DOF1_01	DOF1	307	318	1	1	0.98	gatTAAAGagt
AtSK23	P$SBF1_01	SBF1	314	328	1	1	0.93	gagtttTTAAAatt
AtSK23	P$GAMYB_Q2	GAMYB	339	352	1	1	0.907	ccaaaACAACtat
AtSK23	P$HSFA2_01	HSFA2	339	345	1	1	1	CCAAAa
AtSK23	P$C1_Q2	C1	344	355	1	1	0.931	acAACTAttaa
AtSK23	P$HSFA4A_01	HSFA4A	347	353	1	1	1	aCTATT
AtSK23	P$EDT1_01	EDT1	348	358	1	1	0.851	ctaTTAATct
AtSK23	P$HMG1_01	HMG1	369	378	1	1	0.925	GTTGTtgtt
AtSK23	P$WEREWOLF_Q2	WEREWOLF	373	382	1	1	0.991	ttGTTAGtt
AtSK23	P$ASR1_01	ASR1	383	388	1	1	1	ACCCA
AtSK23	P$HSFA2_01	HSFA2	385	391	1	1	0.922	CCAAAt
AtSK23	P$PDF2_01	DF2	420	431	1	1	0.862	acaaTAAATtg
AtSK23	P$ATHB6_01	ATHB6	420	429	1	1	0.929	acAATAAat
AtSK23	P$ATHB1_01	ATHB1	435	449	1	1	0.862	atggtATTATtacg
AtSK23	P$ATHB7_01	ATHB7	472	482	1	1	0.932	ccAATCActg
AtSK23	P$HAT1_01	HAT1	472	482	1	1	0.882	ccAATCActg
AtSK23	P$CBNAC_02	CBNAC	481	497	1	1	0.933	gTGCTTattaaataat
AtSK23	P$CBNAC_01	CBNAC	481	487	1	1	0.979	gTGCTT
AtSK23	P$SBF1_01	SBF1	482	496	1	1	0.873	tgcttaTTAAAtaa
AtSK23	P$ATHB1_03	ATHB1	489	500	1	1	0.888	taAATAAtgac
AtSK23	P$ATHB5_04	ATHB5	489	500	1	1	0.886	taAATAAtgac
AtSK23	P$ATHB6_01	ATHB6	489	498	1	1	0.976	taAATAAtg
AtSK23	P$ATHB16_01	ATHB16	490	498	1	1	0.867	aAATAAtg
AtSK23	P$SBF1_01	SBF1	497	511	1	1	0.915	gactacTTAATaaa
AtSK23	P$EDT1_01	EDT1	500	510	1	1	0.851	tacTTAATaa
AtSK23	P$ATHB6_01	ATHB6	503	512	1	1	0.908	ttAATAAac
AtSK23	P$AT3G20750_01	AT3G20750	506	514	1	1	0.888	aTAAACag
AtSK23	P$ARR1_01	ARR1	534	544	1	1	0.949	taaGAATCac
AtSK23	P$PEND_01	END	534	542	1	1	0.877	tAAGAAtc
AtSK23	P$HAT1_01	HAT1	536	546	1	1	0.856	agAATCAcgc
AtSK23	P$FAR1_01	FAR1	537	552	1	1	0.98	gaatcACGCGctaaa
AtSK23	P$FHY3_01	FHY3	539	551	1	1	0.999	atcACGCGctaa
AtSK23	P$AT3G20750_01	AT3G20750	547	555	1	1	0.88	cTAAACtt
AtSK23	P$CAMTA1_02	CAMTA1	552	564	1	1	0.902	cttaCGCGTggg
AtSK23	P$CMTA2_01	CMTA2	552	561	1	1	0.988	cttaCGCGT
AtSK23	P$ALFIN1_Q2	ALFIN1	553	568	1	1	0.861	ttacgcGTGGGtttc
AtSK23	P$CBT_01	CBT	554	569	1	1	0.864	taCGCGTgggtttcc
AtSK23	P$BZIP68_01	BZIP68	555	564	1	1	0.928	acgCGTGGg
AtSK23	P$CMTA3_01	CMTA3	555	564	1	1	0.985	aCGCGTggg
AtSK31	P$GT1_Q6_01	GT1	1	13	1	1	0.861	TTTTTtgttgat
AtSK31	P$GT1_Q6_01	GT1	2	14	1	1	0.887	TTTTTgttgatg
AtSK31	P$SBF1_01	SBF1	13	27	1	1	0.949	gtgtcaTTAATaaa
AtSK31	P$EDT1_01	EDT1	16	26	1	1	0.868	tcaTTAATaa
AtSK31	P$ATHB6_01	ATHB6	19	28	1	1	0.914	ttAATAAat
AtSK31	P$ATHB6_01	ATHB6	23	32	1	1	0.909	taAATAAat
AtSK31	P$ATHB6_01	ATHB6	27	36	1	1	0.904	taAATAAct
AtSK31	P$C1_Q2	C1	30	41	1	1	0.944	atAACTAtcat
AtSK31	P$SBF1_01	SBF1	34	48	1	1	0.934	ctatcaTTAATaat
AtSK31	P$ATHB4_02	ATHB4	34	44	1	1	0.881	ctATCATtaa
AtSK31	P$EDT1_01	EDT1	37	47	1	1	0.868	tcaTTAATaa
AtSK31	P$ATHB1_03	ATHB1	40	51	1	1	0.976	ttAATAAttgg
AtSK31	P$ATHB5_04	ATHB5	40	51	1	1	0.975	ttAATAAttgg
AtSK31	P$ATHB6_01	ATHB6	40	49	1	1	0.984	ttAATAAtt
AtSK31	P$ATHB16_01	ATHB16	41	49	1	1	1	tAATAAtt
AtSK31	P$AGL20_01	AGL20	64	76	1	1	0.894	ttTAAATtttat

AtSK31	P$AGL12_01	AGL12	64	76	1	1	0.933	tttAAATTttat
AtSK31	P$AT2G26320_01	AT2G26320	65	76	1	1	0.965	TTAAAttttat
AtSK31	P$SQUA_01	SQUA	91	101	1	1	0.867	caaTTTTTtt
AtSK31	P$GT1_Q6_01	GT1	95	107	1	1	0.86	TTTTTtttttcc
AtSK31	P$GT1_Q6_01	GT1	96	108	1	1	0.853	TTTTTttttcca
AtSK31	P$EDT1_01	EDT1	113	123	1	1	0.882	aatTTAATtg
AtSK31	P$PHV_02	HV	122	137	1	1	0.93	gttATCATtatgatg
AtSK31	P$ATHB4_02	ATHB4	123	133	1	1	0.892	ttATCATtat
AtSK31	P$ARR2_01	ARR2	168	178	1	1	0.888	gtatATCTTt
AtSK31	P$GT1_Q6	GT1	189	196	1	1	1	GTAAAta
AtSK31	P$ATHB6_01	ATHB6	199	208	1	1	0.975	aaAATAAta
AtSK31	P$ATHB5_04	ATHB5	199	210	1	1	0.892	aaAATAAtata
AtSK31	P$ATHB1_03	ATHB1	199	210	1	1	0.898	aaAATAAtata
AtSK31	P$ATHB16_01	ATHB16	200	208	1	1	0.867	aAATAAta
AtSK31	P$AT3G51080_01	AT3G51080	217	224	1	1	1	GGAAAaa
AtSK31	P$HSFA4A_01	HSFA4A	226	232	1	1	0.914	tCTATT
AtSK31	P$AGL27_01	AGL27	235	249	1	1	0.853	gTTTCTcttttttt
AtSK31	P$SQUA_01	SQUA	241	251	1	1	0.906	cttTTTTTat
AtSK31	P$GT1_Q6_01	GT1	242	254	1	1	0.905	TTTTTttatata
AtSK31	P$GT1_Q6_01	GT1	244	256	1	1	0.897	TTTTTatataag
AtSK31	P$C1_Q2	C1	255	266	1	1	0.93	gcAACTAttta
AtSK31	P$HSFA4A_01	HSFA4A	258	264	1	1	1	aCTATT
AtSK31	P$EDT1_01	EDT1	260	270	1	1	0.968	tatTTAATgg
AtSK31	P$KNOX3_01	KNOX3	271	283	1	1	0.97	aataTGACAtat
AtSK31	P$TGA1A_Q2_01	TGA1A	271	293	1	1	0.923	aatATGACAtataataataatt
AtSK31	P$ATH1_01	ATH1	275	283	1	1	0.935	TGACAtat
AtSK31	P$ATHB6_01	ATHB6	281	290	1	1	0.981	atAATAAta
AtSK31	P$ATHB5_04	ATHB5	281	292	1	1	0.905	atAATAAtaat
AtSK31	P$ATHB1_03	ATHB1	281	292	1	1	0.894	atAATAAtaat
AtSK31	P$ATHB16_01	ATHB16	282	290	1	1	0.915	tAATAAta
AtSK31	P$ATHB6_01	ATHB6	284	293	1	1	0.984	atAATAAtt
AtSK31	P$ATHB5_04	ATHB5	284	295	1	1	0.979	atAATAAttta
AtSK31	P$ATHB1_03	ATHB1	284	295	1	1	0.985	atAATAAttta
AtSK31	P$ATHB16_01	ATHB16	285	293	1	1	1	tAATAAtt
AtSK31	P$SBF1_01	SBF1	286	300	1	1	0.861	aataatTTAAAaaa
AtSK31	P$ARR18_01	ARR18	298	311	1	1	0.888	aattAGATAgtat
AtSK31	P$SPF1_Q2	SPF1	302	312	1	1	0.949	agATAGTatt
AtSK31	P$HSFA4A_01	HSFA4A	322	328	1	1	1	aCTATT
AtSK31	P$AT3G51080_01	AT3G51080	331	338	1	1	0.893	GGAAAat
AtSK31	P$ATHB6_01	ATHB6	343	352	1	1	0.908	aaAATAAat
AtSK31	P$ATHB6_01	ATHB6	347	356	1	1	0.909	taAATAAat
AtSK31	P$PDF2_01	DF2	347	358	1	1	0.856	taaaTAAATcc
AtSK31	P$AT3G60580_01	AT3G60580	352	359	1	1	0.883	aaATCCC
AtSK31	P$MYBAS1_01	MYBAS1	355	366	1	1	0.942	tcCCAACtatg
AtSK31	P$C1_Q2	C1	357	368	1	1	0.96	ccAACTAtgat
AtSK32b	P$ABI3_01	ABI3	3	12	1	1	0.875	ttGCATGat
AtSK32b	P$NAC043_01	NAC043	18	28	1	1	0.918	cttACGTAtt
AtSK32b	P$AT3G63350_01	AT3G63350	28	34	1	1	0.882	CCGCCa
AtSK32b	P$HBP1B_Q6	HBP1B	40	54	1	1	0.956	ttgcgccaCGTCAt
AtSK32b	P$E2FA_02	E2FA	40	50	1	1	0.993	ttgCGCCAcg
AtSK32b	P$ABF2_01	ABF2	41	54	1	1	0.906	tgcgcCACGTcat
AtSK32b	P$O2_Q4	O2	42	53	1	1	0.921	gcgcCACGTca
AtSK32b	P$O2_04	O2	42	55	1	1	0.93	gcGCCACgtcatt
AtSK32b	P$ABF4_01	ABF4	43	55	1	1	0.885	cgcCACGTcatt
AtSK32b	P$GBF6_01	GBF6	43	58	1	1	0.896	cgcCACGTcattccc
AtSK32b	P$GBP_Q6	GBP	43	55	1	1	0.901	cgcCACGTcatt
AtSK32b	P$ABI5_01	ABI5	43	53	1	1	0.936	cgcCACGTca
AtSK32b	P$BEE2_01	BEE2	44	54	1	1	0.906	gcCACGTcat
AtSK32b	P$BIM3_01	BIM3	44	54	1	1	0.876	gcCACGTcat
AtSK32b	P$PHYPA143875_02	HYPA143875	44	54	1	1	0.871	gcCACGTcat
AtSK32b	P$SPT_01	SPT	44	53	1	1	0.945	gcCACGTca
AtSK32b	P$GBF1F_Q2	GBF1F	44	55	1	1	0.966	gcCACGTcatt
AtSK32b	P$BZIP43_01	BZIP43	44	56	1	1	0.853	gcCACGTcattc
AtSK32b	P$BZIP48_01	BZIP48	44	58	1	1	0.896	gcCACGTcattccc
AtSK32b	P$HBPA1_Q6_01	HBPA1	44	54	1	1	0.979	gccaCGTCAt
AtSK32b	P$TGA1B_01	TGA1B	44	54	1	1	0.954	gcCACGTcat
AtSK32b	P$CPRF2_01	CPRF2	44	54	1	1	0.969	gcCACGTcat
AtSK32b	P$CPRF3_01	CPRF3	44	54	1	1	0.979	gcCACGTcat
AtSK32b	P$EMBP1_Q2	EMBP1	44	54	1	1	0.957	gcCACGTcat
AtSK32b	P$CPRF3_Q2	CPRF3	44	54	1	1	0.971	gcCACGTcat
AtSK32b	P$CPRF2_Q2	CPRF2	44	54	1	1	0.968	gcCACGTcat
AtSK32b	P$O2_02	O2	44	54	1	1	0.98	gcCACGTcat
AtSK32b	P$TGA1B_Q2	TGA1B	44	54	1	1	0.971	gcCACGTcat
AtSK32b	P$TGA1A_Q2	TGA1A	44	54	1	1	0.986	gcCACGTcat
AtSK32b	P$RITA1_01	RITA1	45	52	1	1	0.976	cCACGTc
AtSK32b	P$AT3G62240_01	AT3G62240	46	56	1	1	0.899	cacgtCATTC
AtSK32b	P$TGA1A_01	TGA1A	46	53	1	1	0.989	cACGTCa
AtSK32b	P$OCSBF1_01	OCSBF1	46	51	1	1	1	CACGT

AtSK32b	P$TGA2_Q2	TGA2	47	57	1	1	0.941	aCGTCAttcc
AtSK32b	P$KNOX3_01	KNOX3	55	67	1	1	0.975	ccctTGACAgac
AtSK32b	P$WRKY11_Q2	WRKY11	57	65	1	1	0.897	cTTGACag
AtSK32b	P$ATH1_01	ATH1	59	67	1	1	0.977	TGACAgac
AtSK32b	P$AT4G36620_01	AT4G36620	74	82	1	1	0.98	atgAACCA
AtSK32b	P$MYB118_01	MYB118	76	93	1	1	0.923	gaaccagacGTTACgcc
AtSK32b	P$TGA1B_01	TGA1B	80	90	1	1	0.888	caGACGTtac
AtSK32b	P$ERF4_05	ERF4	85	100	1	1	0.855	gttaCGCCGtcgttt
AtSK32b	P$ERF112_02	ERF112	87	97	1	1	0.968	taCGCCGtcg
AtSK32b	P$ERF11_01	ERF11	88	98	1	1	0.989	aCGCCGtcgt
AtSK32b	P$ERF069_01	ERF069	88	97	1	1	0.993	aCGCCGtcg
AtSK32b	P$ERF4_04	ERF4	88	96	1	1	0.952	aCGCCGtc
AtSK32b	P$CRF4_01	CRF4	88	96	1	1	0.924	aCGCCGtc
AtSK32b	P$ERF8_01	ERF8	89	99	1	1	0.987	CGCCGtcgtt
AtSK32b	P$ERF3_04	ERF3	89	97	1	1	0.952	CGCCGtcg
AtSK32b	P$HMG1_01	HMG1	141	150	1	1	0.953	GTTGTtttt
AtSK32b	P$RIN_Q2	RIN	143	154	1	1	0.867	tgttTTTAAgt
AtSK32b	P$DOF_Q2	DOF	161	172	1	1	0.96	aggAAAAGaaa
AtSK32b	P$PBF_01	BF	161	172	1	1	0.966	aggAAAAGaaa
AtSK32b	P$AT3G51080_01	AT3G51080	162	169	1	1	0.918	GGAAAag
AtSK32b	P$CDF2_01	CDF2	162	172	1	1	0.95	ggAAAAGaaa
AtSK32b	P$CDF3_01	CDF3	163	172	1	1	0.971	gAAAAGaaa
AtSK32b	P$BPC1_Q2	BPC1	167	173	1	1	0.997	AGAAAa
AtSK32b	P$NAC043_01	NAC043	170	180	1	1	0.891	aaaACGTAag
AtSK32b	P$NAC025_01	NAC025	172	180	1	1	0.907	aACGTAag
AtSK32b	P$NAC6_01	NAC6	173	179	1	1	1	aCGTAA
AtSK32b	P$SBF1_01	SBF1	183	197	1	1	0.87	gtgaatTTAAAtag
AtSK32b	P$ATSPL8_01	ATSPL8	193	209	1	1	0.888	atagcTGTACggtggt
AtSK32b	P$SPL15_01	SPL15	194	208	1	1	0.9	tagctGTACGgtgg
AtSK32b	P$BHLH28_01	BHLH28	195	207	1	1	0.958	agctGTACGgtg
AtSK32b	P$SPL5_02	SPL5	195	207	1	1	0.896	agctGTACGgtg
AtSK32b	P$SPL11_01	SPL11	195	207	1	1	0.965	agctGTACGgtg
AtSK32b	P$SPL5_01	SPL5	197	206	1	1	0.998	ctGTACGgt
AtSK32b	P$SPL4_01	SPL4	198	207	1	1	0.999	tGTACGgtg
AtSK32b	P$SPL12_01	SPL12	198	206	1	1	1	tGTACGgt
AtSK32b	P$POPTR_01	OPTR	198	205	1	1	0.999	tGTACGg
AtSK32b	P$ALFIN1_Q2	ALFIN1	201	216	1	1	0.873	acggtgGTGGGcttt
AtSK32b	P$DOF1_01	DOF1	217	228	1	1	0.98	tttTAAAGccc
AtSK32b	P$DOF2_01	DOF2	217	228	1	1	0.997	ttttAAAGCcc
AtSK32b	P$DOF3_01	DOF3	217	228	1	1	0.978	ttttAAAGCcc
AtSK32b	P$MYB3R4_01	MYB3R4	224	239	1	1	0.851	gccctggcCCGTTat
AtSK32b	P$MYB3R1_01	MYB3R1	224	239	1	1	0.86	gccctggcCCGTTat
AtSK32b	P$ATHB9_01	ATHB9	237	256	1	1	0.856	atggctaATGATtcctaga
AtSK32b	P$HAHB4_01	HAHB4	242	251	1	1	0.984	tAATGAttc
AtSK32b	P$UIF1_01	UIF1	243	253	1	1	0.863	aatGATTCct
AtSK32b	P$GATA9_01	GATA9	250	261	1	1	0.994	cctAGATCtta
AtSK32b	P$ARR2_01	ARR2	251	261	1	1	0.881	ctagATCTTa
AtSK32b	P$AGP1_01	AGP1	251	261	1	1	0.941	ctAGATCtta
AtSK32b	P$GATA8_01	GATA8	252	261	1	1	0.989	taGATCTta
AtSK32b	P$GATA11_01	GATA11	252	260	1	1	0.969	taGATCTt
AtSK32b	P$GATA10_01	GATA10	252	260	1	1	0.949	tAGATCtt
AtSK32b	P$ARR10_01	ARR10	253	260	1	1	0.956	AGATCtt
AtSK32b	P$EDT1_01	EDT1	263	273	1	1	0.866	acaTTAATtg
AtSK32b	P$GAMYB_Q2	GAMYB	273	286	1	1	0.921	ggcctACAACaat
AtSK32b	P$ATHSFA1D_01	ATHSFA1D	275	281	1	1	0.985	cCTACA
AtSK32b	P$RAV1_01	RAV1	276	288	1	1	0.94	ctaCAACAatat
AtSK32b	P$O2_Q4	O2	284	295	1	1	0.857	atatCATGTgc
AtSK32b	P$AMS_01	AMS	286	296	1	1	0.983	atCATGTgcc
AtSK32b	P$ATHB1_01	ATHB1	291	305	1	1	0.852	gtgccATTATttat
AtSK32b	P$ATHB5_01	ATHB5	294	303	1	1	0.92	ccaTTATTt
AtSK32b	P$MYB3R5_01	MYB3R5	296	311	1	1	0.897	attatttatCCGTTc
AtSK32b	P$MYB3R1_01	MYB3R1	297	312	1	1	0.915	ttatttatCCGTTca
AtSK32b	P$MYB3R4_01	MYB3R4	297	312	1	1	0.917	ttatttatCCGTTca
AtSK32b	P$ATHB6_01	ATHB6	341	350	1	1	0.911	ctAATAAgc
AtSK32b	P$GT1_Q6	GT1	361	368	1	1	0.971	GTGAAaa
AtSK32b	P$EDT1_01	EDT1	372	382	1	1	0.893	catTTAATcc
AtSK32b	P$AT3G60580_01	AT3G60580	376	383	1	1	0.882	taATCCC
AtSK32b	P$MYBAS1_01	MYBAS1	381	392	1	1	0.986	ccCCAACtttc
AtSK32b	P$MADSB_Q2	MADSB	389	404	1	1	0.869	ttcaAAAAAtggtca
AtSK32b	P$MYB3R5_01	MYB3R5	420	435	1	1	0.912	cgtatgtggCCGTTt
AtSK32b	P$MYB3R1_01	MYB3R1	421	436	1	1	0.927	gtatgtggCCGTTta
AtSK32b	P$MYB3R4_01	MYB3R4	421	436	1	1	0.934	gtatgtggCCGTTta
AtSK32b	P$AT1G53910_01	AT1G53910	426	436	1	1	0.876	tGGCCGttta
AtSK32b	P$C1_Q2	C1	441	452	1	1	0.949	tgAACTAaagg
AtSK32b	P$DOF1_01	DOF1	443	454	1	1	0.987	aacTAAAGgtt
AtSK32b	P$PBF_Q2	BF	446	452	1	1	0.986	tAAAGG
AtSK32b	P$WRKY11_01	WRKY11	448	462	1	1	0.881	aaggTTGACtaatt
AtSK32b	P$WRKY11_Q2	WRKY11	451	459	1	1	0.953	gTTGACta

AtSK32b	P$AT3G20750_01	AT3G20750	464	472	1	1	0.969	tTAAACat
AtSK32b	P$ARR2_01	ARR2	469	479	1	1	0.873	catgATCTTt
AtSK32b	P$GATA15_01	GATA15	469	478	1	1	0.999	caTGATCtt
AtSK32b	P$GATA8_01	GATA8	470	479	1	1	0.975	atGATCTtt
AtSK32b	P$AT2G38090_01	AT2G38090	474	486	1	1	0.912	tctTTCGTtgac
AtSK32b	P$WRKY11_Q2	WRKY11	480	488	1	1	1	gTTGACca
AtSK32b	P$ZAP1_01	ZAP1	481	491	1	1	0.878	TTGACcagac
AtSK32b	P$MYB3R5_01	MYB3R5	495	510	1	1	0.878	atagacatgCCGTTa
AtSK32b	P$MYB3R1_01	MYB3R1	496	511	1	1	0.885	tagacatgCCGTTat
AtSK32b	P$MYB3R4_01	MYB3R4	496	511	1	1	0.878	tagacatgCCGTTat
AtSK32b	P$LEC2_01	LEC2	498	509	1	1	0.947	gaCATGCcgtt
AtSK32b	P$ERF019_01	ERF019	501	511	1	1	0.87	aTGCCGttat
AtSK32b	P$JERF1_01	JERF1	501	511	1	1	0.858	aTGCCGttat
AtSK32b	P$ORA47_01	ORA47	501	511	1	1	0.856	aTGCCGttat
AtSK32b	P$MYB23_01	MYB23	515	528	1	1	0.864	cattAACGGaact
AtSK32b	P$AT3G09370_01	AT3G09370	516	528	1	1	0.869	atTAACGgaact
AtSK32b	P$AT5G11510_01	AT5G11510	516	528	1	1	0.872	atTAACGgaact
AtSK32b	P$MYB52_01	MYB52	516	528	1	1	0.89	attAACGGaact
AtSK32b	P$AT1G26780_01	AT1G26780	516	528	1	1	0.871	attAACGGaact
AtSK32b	P$MYB3R5_01	MYB3R5	527	542	1	1	0.897	ttctaatgtCCGTTa
AtSK32b	P$MYB3R1_01	MYB3R1	528	543	1	1	0.928	tctaatgtCCGTTat
AtSK32b	P$MYB3R4_01	MYB3R4	528	543	1	1	0.926	tctaatgtCCGTTat
AtSK32b	P$DRE1C_01	DRE1C	532	540	1	1	0.862	ATGTCcgt
AtSK32b	P$MYB3R5_01	MYB3R5	537	552	1	1	0.863	cgttatcatCCGTTa
AtSK32b	P$MYB3R4_01	MYB3R4	538	553	1	1	0.883	gttatcatCCGTTaa
AtSK32b	P$MYB3R1_01	MYB3R1	538	553	1	1	0.894	gttatcatCCGTTaa
AtSK32b	P$SBF1_01	SBF1	543	557	1	1	0.883	catccgTTAATttt
AtSK32b	P$WRKY_Q2	WRKY	554	565	1	1	0.886	tttattAGTCA
AtSK32b	P$WRKY60_01	WRKY60	558	569	1	1	0.9	ttaGTCAAgtt
AtSK32b	P$WRKY57_01	WRKY57	558	568	1	1	0.975	ttaGTCAAgt
AtSK32b	P$WRKY48_02	WRKY48	558	568	1	1	0.993	ttaGTCAAgt
AtSK32b	P$WRKY21_02	WRKY21	558	568	1	1	0.959	ttaGTCAAgt
AtSK32b	P$WRKY18_02	WRKY18	558	568	1	1	0.979	ttaGTCAAgt
AtSK32b	P$WRKY40_03	WRKY40	558	568	1	1	0.995	ttAGTCAagt
AtSK32b	P$WRKY8_01	WRKY8	559	568	1	1	0.986	taGTCAAgt
AtSK32b	P$WRKY75_01	WRKY75	559	567	1	1	0.96	taGTCAAg
AtSK32b	P$WRKY63_01	WRKY63	559	567	1	1	0.908	taGTCAAg
AtSK32b	P$WRKY62_01	WRKY62	559	567	1	1	0.897	taGTCAAg
AtSK32b	P$WRKY43_02	WRKY43	559	569	1	1	0.983	taGTCAAgtt
AtSK32b	P$WRKY40_01	WRKY40	559	567	1	1	0.996	taGTCAAg
AtSK32b	P$WRKY25_02	WRKY25	559	567	1	1	0.916	taGTCAAg
AtSK32b	P$WRKY2_01	WRKY2	559	567	1	1	0.933	taGTCAAg
AtSK32b	P$WRKY15_01	WRKY15	559	569	1	1	0.972	taGTCAAgtt
AtSK32b	P$WRKY30_01	WRKY30	560	570	1	1	0.913	aGTCAAgttt
AtSK32b	P$WRKY23_01	WRKY23	560	568	1	1	0.862	aGTCAAgt
AtSK32b	P$WRKY18_Q2	WRKY18	561	570	1	1	1	GTCAAgttt
AtSK32b	P$AT3G20750_01	AT3G20750	585	593	1	1	0.969	tTAAACat
AtSK32b	P$PEND_02	END	595	605	1	1	1	acTTCTTatt
AtSK32b	P$AT3G20750_01	AT3G20750	624	632	1	1	0.887	gTAAACgt
AtSK32b	P$WRKY11_01	WRKY11	627	641	1	1	0.882	aacgTTGACtacca
AtSK32b	P$WRKY11_Q2	WRKY11	630	638	1	1	0.953	gTTGACta
AtSK32b	P$P_01		633	642	1	1	0.876	gaCTACCat
AtSK32b	P$RIN_Q2	RIN	637	648	1	1	0.853	accaTTTAAga
AtSK32b	P$WRKY11_Q2	WRKY11	660	668	1	1	1	gTTGACca
AtSK32b	P$ZAP1_01	ZAP1	661	671	1	1	0.921	TTGACcaggc
AtSK32b	P$HSFA2_01	HSFA2	670	676	1	1	1	CCAAAa
AtSK32b	P$O2_Q4	O2	676	687	1	1	0.924	tagaCATGTct
AtSK32b	P$BPC1_Q2	BPC1	702	708	1	1	0.99	AGAAAc
AtSK32b	P$SBF1_01	SBF1	705	719	1	1	0.866	aactcgTTAATgac
AtSK32b	P$EDT1_01	EDT1	708	718	1	1	0.923	tcgTTAATga
AtSK32b	P$HSFA2_01	HSFA2	730	736	1	1	1	CCAAAa
AtSK32b	P$GAMYB_Q2	GAMYB	730	743	1	1	0.899	ccaaaACAACgtc
AtSK32b	P$AT5G54070_01	AT5G54070	735	741	1	1	0.958	aCAACG
AtSK32b	P$TGA1A_01	TGA1A	737	744	1	1	0.871	aACGTCg
AtSK32b	P$ATHB6_01	ATHB6	748	757	1	1	0.969	agAATAAtc
AtSK32b	P$ATHB5_04	ATHB5	748	759	1	1	0.883	agAATAAtcga
AtSK32b	P$ATHB1_03	ATHB1	748	759	1	1	0.89	agAATAAtcga
AtSK32b	P$ATHB16_01	ATHB16	749	757	1	1	0.855	gAATAAtc
AtSK32b	P$DOF1_01	DOF1	763	774	1	1	0.984	acaTAAAGatt
AtSK32b	P$PBF_01	BF	796	807	1	1	0.949	tcaAAAAGttt
AtSK32b	P$DOF_Q2	DOF	796	807	1	1	0.975	tcaAAAAGttt
AtSK32b	P$CDF2_01	CDF2	797	807	1	1	0.994	caAAAAGttt
AtSK32b	P$CDF3_01	CDF3	798	807	1	1	0.989	aAAAAGttt
AtSK32b	P$ASR1_01	ASR1	815	820	1	1	1	ACCCA
AtSK32b	P$SPF1_Q2	SPF1	823	833	1	1	0.883	acATAGTttg
AtSK32b	P$ARR18_01	ARR18	835	848	1	1	0.889	atagAGATAaccg
AtSK32b	P$ATHB6_01	ATHB6	867	876	1	1	0.906	caAATAAag
AtSK32b	P$DOF1_01	DOF1	868	879	1	1	0.982	aaaTAAAGgtg

AtSK32b	P$PBF_Q2	BF	871	877	1	1	0.986	tAAAGG
AtSK32b	P$SBF1_01	SBF1	893	907	1	1	0.85	tttgttTTAAAttt
AtSK32b	P$GT1_Q6_01	GT1	893	905	1	1	0.934	TTTGTtttaaat
AtSK32b	P$PDF2_01	DF2	896	907	1	1	0.854	gtttTAAATtt
AtSK32b	P$AT1G59810_01	AT1G59810	896	912	1	1	0.882	gtttTAAATtttaaaa
AtSK32b	P$AT2G26880_01	AT2G26880	898	912	1	1	0.861	tttaaatTTTAAaa
AtSK32b	P$AGL12_01	AGL12	898	910	1	1	0.937	tttAAATTttaa
AtSK32b	P$AGL20_01	AGL20	898	910	1	1	0.898	ttTAAATtttaa
AtSK32b	P$AT2G26320_01	AT2G26320	899	910	1	1	0.948	TTAAAttttaa
AtSK32b	P$PBF_01	BF	905	916	1	1	0.969	tttAAAAGgaa
AtSK32b	P$DOF_Q2	DOF	905	916	1	1	0.945	tttAAAAGgaa
AtSK32b	P$CDF2_01	CDF2	906	916	1	1	0.954	ttAAAAGgaa
AtSK32b	P$CDF3_01	CDF3	907	916	1	1	0.973	tAAAAGgaa
AtSK32b	P$PBF_Q2	BF	908	914	1	1	1	aAAAGG
AtSK32b	P$ARR1_01	ARR1	910	920	1	1	0.948	aagGAATCat
AtSK32b	P$HAT1_01	HAT1	912	922	1	1	0.977	ggAATCAtgg
AtSK32b	P$ATHB7_01	ATHB7	912	922	1	1	0.914	ggAATCAtgg
AtSK32b	P$BPC1_Q2	BPC1	973	979	1	1	0.99	AGAAAt
AtSK32b	P$BPC1_Q2	BPC1	1005	1011	1	1	0.997	AGAAAa
AtSK32b	P$MYB24_01	MYB24	1050	1059	1	1	0.859	attTTAGGg
AtSK32b	P$WRKY48_01	WRKY48	1072	1081	1	1	0.852	cgttAACAA
AtSK32b	P$ATMYB15_Q2	ATMYB15	1075	1081	1	1	1	TAACAa
AtSK32b	P$PEND_02	END	1099	1109	1	1	0.889	agTTCTTtta
AtSK32b	P$EDT1_01	EDT1	1103	1113	1	1	0.943	cttTTAATga
AtSK32b	P$KNOX3_01	KNOX3	1113	1125	1	1	0.954	atgaTGACAaca
AtSK32b	P$GAMYB_Q2	GAMYB	1114	1127	1	1	0.896	tgatgACAACatg
AtSK32b	P$ATH1_01	ATH1	1117	1125	1	1	0.927	TGACAaca
AtSK32b	P$RAV1_01	RAV1	1117	1129	1	1	0.921	tgaCAACAtgtt
AtSK32b	P$SEP3_01	wrz-03	1126	1137	1	1	0.853	gtttaTTTTGg
AtSK32b	P$WRKY60_01	WRKY60	1138	1149	1	1	0.999	ttgGTCAAcga
AtSK32b	P$WRKY57_01	WRKY57	1138	1148	1	1	0.986	ttgGTCAAcg
AtSK32b	P$WRKY48_02	WRKY48	1138	1148	1	1	1	ttgGTCAAcg
AtSK32b	P$WRKY21_02	WRKY21	1138	1148	1	1	1	ttgGTCAAcg
AtSK32b	P$WRKY18_02	WRKY18	1138	1148	1	1	1	ttgGTCAAcg
AtSK32b	P$WRKY8_01	WRKY8	1139	1148	1	1	1	tgGTCAAcg
AtSK32b	P$WRKY75_01	WRKY75	1139	1147	1	1	0.998	tgGTCAAc
AtSK32b	P$WRKY63_01	WRKY63	1139	1147	1	1	0.999	tgGTCAAc
AtSK32b	P$WRKY62_01	WRKY62	1139	1147	1	1	1	tgGTCAAc
AtSK32b	P$WRKY43_02	WRKY43	1139	1149	1	1	0.985	tgGTCAAcga
AtSK32b	P$WRKY40_01	WRKY40	1139	1147	1	1	1	tgGTCAAc
AtSK32b	P$WRKY25_02	WRKY25	1139	1147	1	1	0.994	tgGTCAAc
AtSK32b	P$WRKY2_01	WRKY2	1139	1147	1	1	0.998	tgGTCAAc
AtSK32b	P$WRKY15_01	WRKY15	1139	1149	1	1	1	tgGTCAAcga
AtSK32b	P$WRKY23_01	WRKY23	1140	1148	1	1	0.964	gGTCAAcg
AtSK32b	P$WRKY30_01	WRKY30	1140	1150	1	1	0.999	gGTCAAcgaa
AtSK32b	P$WRKY18_Q2	WRKY18	1141	1150	1	1	0.922	GTCAAcgaa
AtSK32b	P$AT5G54070_01	AT5G54070	1142	1148	1	1	0.91	tCAACG
AtSK32b	P$O2_Q4	O2	1151	1162	1	1	0.863	ttctCATGTtt
AtSK32b	P$PDF2_01	DF2	1158	1169	1	1	0.9	gtttTAAATgg
AtSK32b	P$GT1_Q6_02	GT1	1166	1178	1	1	0.85	tggtagTTAACt
AtSK32b	P$SBF1_01	SBF1	1184	1198	1	1	0.928	tcatgtTTAAAatg
AtSK32b	P$DOF1_01	DOF1	1203	1214	1	1	0.979	ataTAAAGtcg
AtSK32b	P$SBF1_01	SBF1	1212	1226	1	1	0.903	cgatgtTTAAAatg
AtSK32b	P$WRKY11_Q2	WRKY11	1240	1248	1	1	0.95	cTTGACta
AtSK32b	P$ATHB6_01	ATHB6	1245	1254	1	1	0.911	ctAATAAag
AtSK32b	P$DOF1_01	DOF1	1246	1257	1	1	0.973	taaTAAAGtcg
AtSK32b	P$MYB23_01	MYB23	1255	1268	1	1	0.913	cgttAACGGatga
AtSK32b	P$AT3G09370_01	AT3G09370	1256	1268	1	1	0.854	gtTAACGgatga
AtSK32b	P$ATMYB15_Q2	ATMYB15	1268	1274	1	1	0.865	TAACAg
AtSK32b	P$MYB3R5_01	MYB3R5	1277	1292	1	1	0.86	ttagaagttCCGTTa
AtSK32b	P$MYB3R4_01	MYB3R4	1278	1293	1	1	0.863	tagaagttCCGTTaa
AtSK32b	P$MYB3R1_01	MYB3R1	1278	1293	1	1	0.878	tagaagttCCGTTaa
AtSK32b	P$SBF1_01	SBF1	1283	1297	1	1	0.865	gttccgTTAATgac
AtSK32b	P$EDT1_01	EDT1	1286	1296	1	1	0.925	ccgTTAATga
AtSK32b	P$AT5G11510_01	AT5G11510	1298	1310	1	1	0.87	gaTAACGgcatg
AtSK32b	P$O2_Q4	O2	1302	1313	1	1	0.879	acggCATGTct
AtSK32b	P$ABI3_01	ABI3	1303	1312	1	1	0.876	cgGCATGtc
AtSK32b	P$MRP1_Q2	MRP1	1308	1320	1	1	0.852	tgTCTATtttgg
AtSK32b	P$SEP3_01	wrz-03	1309	1320	1	1	0.887	gtctaTTTTGg
AtSK32b	P$HSFA4A_01	HSFA4A	1310	1316	1	1	0.914	tCTATT
AtSK32b	P$ATHB1_03	ATHB1	1329	1340	1	1	0.878	cgAATAAtcat
AtSK32b	P$ATHB5_04	ATHB5	1329	1340	1	1	0.878	cgAATAAtcat
AtSK32b	P$ATHB6_01	ATHB6	1329	1338	1	1	0.969	cgAATAAtc
AtSK32b	P$ATHB16_01	ATHB16	1330	1338	1	1	0.855	gAATAAtc
AtSK32b	P$ATHB7_01	ATHB7	1332	1342	1	1	0.922	atAATCAtgt
AtSK32b	P$HAT1_01	HAT1	1332	1342	1	1	0.985	atAATCAtgt
AtSK32b	P$DOF_Q2	DOF	1342	1353	1	1	0.93	tttAAAAGtta
AtSK32b	P$PBF_01	BF	1342	1353	1	1	0.969	tttAAAAGtta

AtSK32b	P$CDF2_01	CDF2	1343	1353	1	1	0.967	ttAAAAGtta
AtSK32b	P$CDF3_01	CDF3	1344	1353	1	1	0.984	tAAAAGtta
AtSK32b	P$MYBPH3_02	MYBPH3	1345	1358	1	1	0.997	aaaagtTAGTTaa
AtSK32b	P$WEREWOLF_Q2	WEREWOLF	1347	1356	1	1	0.919	aaGTTAGtt
AtSK32b	P$WRKY11_01	WRKY11	1389	1403	1	1	0.856	gaagTTGACgtata
AtSK32b	P$O2_Q2	O2	1390	1403	1	1	0.953	aagttGACGTata
AtSK32b	P$TGA1_01	TGA1	1391	1402	1	1	0.977	agtTGACGtat
AtSK32b	P$STF1_01	STF1	1392	1404	1	1	0.963	gttGACGTataa
AtSK32b	P$BZIP14_01	BZIP14	1392	1402	1	1	0.982	gtTGACGtat
AtSK32b	P$TGA7_01	TGA7	1392	1402	1	1	0.925	gtTGACGtat
AtSK32b	P$TGA6_01	TGA6	1392	1402	1	1	0.958	gtTGACGtat
AtSK32b	P$TGA3_Q2	TGA3	1392	1401	1	1	0.97	gtTGACGta
AtSK32b	P$STF1_02	STF1	1392	1404	1	1	0.94	gtTGACGtataa
AtSK32b	P$WRKY11_Q2	WRKY11	1392	1400	1	1	0.904	gTTGACgt
AtSK32b	P$NAC043_01	NAC043	1393	1403	1	1	0.89	ttgACGTAta
AtSK32b	P$TGA5_01	TGA5	1393	1401	1	1	0.986	tTGACGta
AtSK32b	P$SQUA_01	SQUA	1409	1419	1	1	0.934	ccaTTTTTtg
AtSK32b	P$SQUA_01	SQUA	1410	1420	1	1	0.878	catTTTTTga
AtSK32b	P$EDT1_01	EDT1	1428	1438	1	1	0.85	ggaTTAATtg
AtSK32b	P$KNOX3_01	KNOX3	1432	1444	1	1	0.97	taatTGACAtct
AtSK32b	P$WRKY11_Q2	WRKY11	1434	1442	1	1	0.903	aTTGACat
AtSK32b	P$ATH1_01	ATH1	1436	1444	1	1	0.939	TGACAtct
AtSK32b	P$ARR2_01	ARR2	1436	1446	1	1	0.871	tgacATCTTt
AtSK32b	P$ATHB7_01	ATHB7	1463	1473	1	1	0.932	tcAATCAgta
AtSK32b	P$HAT1_01	HAT1	1463	1473	1	1	0.88	tcAATCAgta
AtSK32b	P$ATHB6_01	ATHB6	1470	1479	1	1	0.903	gtAATAAcc
AtSK32b	P$AT4G36620_01	AT4G36620	1472	1480	1	1	0.916	aatAACCA
AtSK32b	P$ARR18_01	ARR18	1496	1509	1	1	0.887	cctcAGATAgtaa
AtSK32b	P$SPF1_Q2	SPF1	1500	1510	1	1	0.936	agATAGTaaa
AtSK32b	P$C1_Q2	C1	1506	1517	1	1	0.954	taAACTAacca
AtSK32b	P$MYBAS1_01	MYBAS1	1508	1519	1	1	0.975	aaCTAACcaat
AtSK32b	P$WEREWOLF_Q2_01	WEREWOLF	1508	1517	1	1	0.928	aACTAAcca
AtSK32b	P$AT4G36620_01	AT4G36620	1509	1517	1	1	0.899	actAACCA
AtSK32b	P$SBF1_01	SBF1	1592	1606	1	1	0.879	tgataaTTAATagt
AtSK32b	P$EDT1_01	EDT1	1595	1605	1	1	0.883	taaTTAATag
AtSK32b	P$SPF1_Q2	SPF1	1599	1609	1	1	0.921	taATAGTtta
AtSK32b	P$SBF1_01	SBF1	1600	1614	1	1	0.868	aatagtTTAAAatt
AtSK32b	P$REF6_01	REF6	1615	1626	1	1	0.937	tgaaCAGAGac
AtSK32b	P$ATHB1_01	ATHB1	1621	1635	1	1	0.852	gagacATTATtaaa
AtSK32b	P$SBF1_01	SBF1	1624	1638	1	1	0.908	acattaTTAAAaat
AtSK32b	P$CCA1_Q5	CCA1	1628	1645	1	1	0.851	tattaAAAATgtctaaa
AtSK32b	P$AP1_01	AP1	1640	1653	1	1	0.896	ctAAAAAcagtaa
AtSK32b	P$PEND_01	END	1650	1658	1	1	0.972	tAAGAAat
AtSK32b	P$BPC1_Q2	BPC1	1652	1658	1	1	0.99	AGAAAt
AtSK32b	P$PBF_01	BF	1681	1692	1	1	0.972	gcaAAAAGagg
AtSK32b	P$DOF_Q2	DOF	1681	1692	1	1	0.984	gcaAAAAGagg
AtSK32b	P$CDF2_01	CDF2	1682	1692	1	1	0.978	caAAAAGagg
AtSK32b	P$CDF3_01	CDF3	1683	1692	1	1	0.983	aAAAAGagg
AtSK32b	P$WRKY18_02	WRKY18	1695	1705	1	1	0.998	gtgGTCAAag
AtSK32b	P$WRKY21_02	WRKY21	1695	1705	1	1	0.973	gtgGTCAAag
AtSK32b	P$WRKY48_02	WRKY48	1695	1705	1	1	0.998	gtgGTCAAag
AtSK32b	P$WRKY57_01	WRKY57	1695	1705	1	1	0.974	gtgGTCAAag
AtSK32b	P$WRKY60_01	WRKY60	1695	1706	1	1	0.983	gtgGTCAAaga
AtSK32b	P$WRKY62_01	WRKY62	1696	1704	1	1	0.957	tgGTCAAa
AtSK32b	P$WRKY63_01	WRKY63	1696	1704	1	1	0.99	tgGTCAAa
AtSK32b	P$WRKY75_01	WRKY75	1696	1704	1	1	0.975	tgGTCAAa
AtSK32b	P$WRKY8_01	WRKY8	1696	1705	1	1	0.993	tgGTCAAag
AtSK32b	P$WRKY43_02	WRKY43	1696	1706	1	1	0.977	tgGTCAAaga
AtSK32b	P$WRKY40_01	WRKY40	1696	1704	1	1	1	tgGTCAAa
AtSK32b	P$WRKY25_02	WRKY25	1696	1704	1	1	0.973	tgGTCAAa
AtSK32b	P$WRKY2_01	WRKY2	1696	1704	1	1	0.989	tgGTCAAa
AtSK32b	P$WRKY15_01	WRKY15	1696	1706	1	1	0.987	tgGTCAAaga
AtSK32b	P$WRKY30_01	WRKY30	1697	1707	1	1	0.924	gGTCAAagat
AtSK32b	P$WRKY23_01	WRKY23	1697	1705	1	1	0.893	gGTCAAag
AtSK32b	P$WRKY18_Q2	WRKY18	1698	1707	1	1	0.942	GTCAAagat
AtSK32b	P$WRKY60_01	WRKY60	1705	1716	1	1	0.978	atgGTCAAatg
AtSK32b	P$WRKY57_01	WRKY57	1705	1715	1	1	0.975	atgGTCAAat
AtSK32b	P$WRKY48_02	WRKY48	1705	1715	1	1	0.998	atgGTCAAat
AtSK32b	P$WRKY21_02	WRKY21	1705	1715	1	1	0.969	atgGTCAAat
AtSK32b	P$WRKY18_02	WRKY18	1705	1715	1	1	0.996	atgGTCAAat
AtSK32b	P$WRKY75_01	WRKY75	1706	1714	1	1	0.975	tgGTCAAa
AtSK32b	P$WRKY63_01	WRKY63	1706	1714	1	1	0.99	tgGTCAAa
AtSK32b	P$WRKY62_01	WRKY62	1706	1714	1	1	0.957	tgGTCAAa
AtSK32b	P$WRKY43_02	WRKY43	1706	1716	1	1	0.976	tgGTCAAatg
AtSK32b	P$WRKY40_01	WRKY40	1706	1714	1	1	1	tgGTCAAa
AtSK32b	P$WRKY25_02	WRKY25	1706	1714	1	1	0.973	tgGTCAAa
AtSK32b	P$WRKY2_01	WRKY2	1706	1714	1	1	0.989	tgGTCAAa
AtSK32b	P$WRKY15_01	WRKY15	1706	1716	1	1	0.985	tgGTCAAatg

AtSK32b	P$WRKY8_01	WRKY8	1706	1715	1	1	0.992	tgGTCAAat
AtSK32b	P$WRKY30_01	WRKY30	1707	1717	1	1	0.917	gGTCAAatga
AtSK32b	P$WRKY18_Q2	WRKY18	1708	1717	1	1	0.969	GTCAAatga
AtSK32b	P$ATHB6_01	ATHB6	1716	1725	1	1	0.975	aaAATAAta
AtSK32b	P$ATHB5_04	ATHB5	1716	1727	1	1	0.891	aaAATAAtaca
AtSK32b	P$ATHB1_03	ATHB1	1716	1727	1	1	0.897	aaAATAAtaca
AtSK32b	P$ATHB16_01	ATHB16	1717	1725	1	1	0.867	aAATAAta
AtSK32b	P$CMTA2_01	CMTA2	1737	1746	1	1	0.997	attcCGCGT
AtSK32b	P$CAMTA1_02	CAMTA1	1737	1749	1	1	0.909	attcCGCGTggg
AtSK32b	P$CBT_01	CBT	1739	1754	1	1	0.855	tcCGCGTgggcttag
AtSK32b	P$BZIP68_01	BZIP68	1740	1749	1	1	0.929	ccgCGTGGg
AtSK32b	P$CMTA3_01	CMTA3	1740	1749	1	1	1	cCGCGTggg
AtSK32b	P$ATHB7_01	ATHB7	1756	1766	1	1	0.998	gcAATCAttc
AtSK32b	P$HAT1_01	HAT1	1756	1766	1	1	0.999	gcAATCAttc
AtSK32b	P$ATHB4_02	ATHB4	1757	1767	1	1	0.946	caATCATtca
AtSK32b	P$HSFA2_01	HSFA2	1773	1779	1	1	0.922	CCAAAt
AtSK32b	P$SED_Q2	SED	1775	1785	1	1	0.929	aaatCCTTTt
AtSK32b	P$PBF_Q2_01	BF	1779	1785	1	1	1	CCTTTt
AtSK32a	P$PHV_02	HV	10	25	1	1	0.869	attATCATttttttg
AtSK32a	P$ATHB4_02	ATHB4	11	21	1	1	0.887	ttATCATttt
AtSK32a	P$SEP3_01	wrz-03	15	26	1	1	0.907	catttTTTTGg
AtSK32a	P$GT1_Q6_01	GT1	19	31	1	1	0.915	TTTTTggttata
AtSK32a	P$ATHB7_01	ATHB7	29	39	1	1	0.876	taAATCAagg
AtSK32a	P$HAT1_01	HAT1	29	39	1	1	0.87	taAATCAagg
AtSK32a	P$AT4G36620_01	AT4G36620	37	45	1	1	0.884	ggcAACCA
AtSK32a	P$GAMYB_01	GAMYB	39	47	1	1	0.886	CAACCaga
AtSK32a	P$PBF_Q2	BF	86	92	1	1	0.965	gAAAGG
AtSK32a	P$SED_Q2	SED	105	115	1	1	0.983	gtgtCCTTTa
AtSK32a	P$PBF_Q2_01	BF	109	115	1	1	0.998	CCTTTa
AtSK32a	P$EDT1_01	EDT1	113	123	1	1	0.976	tatTTAATgt
AtSK32a	P$ATHB6_01	ATHB6	147	156	1	1	0.929	tcAATAAga
AtSK32a	P$DOF_Q2	DOF	156	167	1	1	0.953	tggAAAAGgaa
AtSK32a	P$PBF_01	BF	156	167	1	1	0.958	tggAAAAGgaa
AtSK32a	P$AT3G51080_01	AT3G51080	157	164	1	1	0.918	GGAAAag
AtSK32a	P$CDF2_01	CDF2	157	167	1	1	0.955	ggAAAAGgaa
AtSK32a	P$CDF3_01	CDF3	158	167	1	1	0.974	gAAAAGgaa
AtSK32a	P$PBF_Q2	BF	159	165	1	1	1	aAAAGG
AtSK32a	P$DYT1_01	DYT1	179	193	1	1	0.96	agtgaaaCACGTga
AtSK32a	P$PIL5_01	IL5	179	193	1	1	0.968	agtgaaacACGTGa
AtSK32a	P$PIF3_01	IF3	180	198	1	1	0.929	gtgaaaCACGTgagtgca
AtSK32a	P$ABF2_01	ABF2	181	194	1	1	0.995	tgaaaCACGTgag
AtSK32a	P$ABZ1_01	ABZ1	182	196	1	1	0.879	gaaacACGTGagtg
AtSK32a	P$HBI1_01	HBI1	182	194	1	1	0.934	gaaaCACGTgag
AtSK32a	P$BZR1_02	BZR1	182	196	1	1	0.953	gaaaCACGTgagtg
AtSK32a	P$O2_Q4	O2	182	193	1	1	0.873	gaaaCACGTga
AtSK32a	P$BZR1_03	BZR1	183	203	1	1	0.885	aaacACGTGagtgcatgaat
AtSK32a	P$ABF4_01	ABF4	183	195	1	1	0.979	aaaCACGTgagt
AtSK32a	P$NAC92_01	NAC92	183	195	1	1	0.943	aaACACGtgagt
AtSK32a	P$GBP_Q6	GBP	183	195	1	1	0.911	aaaCACGTgagt
AtSK32a	P$PIF3_03	IF3	183	193	1	1	0.94	aaaCACGTga
AtSK32a	P$ABI5_01	ABI5	183	193	1	1	0.968	aaaCACGTga
AtSK32a	P$TAF1_01	TAF1	184	194	1	1	0.973	aacACGTGag
AtSK32a	P$EMBP1_02	EMBP1	184	194	1	1	0.925	aacACGTGag
AtSK32a	P$TAF1_Q2	TAF1	184	194	1	1	0.959	aacACGTGag
AtSK32a	P$PIF1_01	IF1	184	194	1	1	0.981	aacACGTGag
AtSK32a	P$HBP1A_Q2	HBP1A	184	194	1	1	0.909	aacACGTGag
AtSK32a	P$SPT_01	SPT	184	193	1	1	0.969	aaCACGTga
AtSK32a	P$TGA1A_Q2	TGA1A	184	194	1	1	0.992	aaCACGTgag
AtSK32a	P$TGA1B_Q2	TGA1B	184	194	1	1	0.939	aaCACGTgag
AtSK32a	P$O2_02	O2	184	194	1	1	0.958	aaCACGTgag
AtSK32a	P$CPRF2_Q2	CPRF2	184	194	1	1	0.995	aaCACGTgag
AtSK32a	P$CPRF3_Q2	CPRF3	184	194	1	1	0.98	aaCACGTgag
AtSK32a	P$CPRF_Q2	CPRF	184	194	1	1	0.895	aaCACGTgag
AtSK32a	P$EMBP1_Q2	EMBP1	184	194	1	1	0.892	aaCACGTgag
AtSK32a	P$CPRF1_01	CPRF1	184	194	1	1	0.912	aaCACGTgag
AtSK32a	P$PHYPA72483_07	HYPA72483	184	194	1	1	0.999	aaCACGTgag
AtSK32a	P$PHYPA143875_02	HYPA143875	184	194	1	1	0.996	aaCACGTgag
AtSK32a	P$BIM3_01	BIM3	184	194	1	1	0.992	aaCACGTgag
AtSK32a	P$BIM2_01	BIM2	184	194	1	1	0.996	aaCACGTgag
AtSK32a	P$BEE2_01	BEE2	184	194	1	1	0.999	aaCACGTgag
AtSK32a	P$BES1_01	BES1	184	195	1	1	0.974	aaCACGTgagt
AtSK32a	P$TGA1B_01	TGA1B	184	194	1	1	0.898	aaCACGTgag
AtSK32a	P$CPRF2_01	CPRF2	184	194	1	1	0.997	aaCACGTgag
AtSK32a	P$CPRF3_01	CPRF3	184	194	1	1	0.987	aaCACGTgag
AtSK32a	P$MYC4_01	MYC4	185	193	1	1	0.949	acACGTGa
AtSK32a	P$BIM1_02	BIM1	185	195	1	1	0.999	acACGTGagt
AtSK32a	P$BHLH13_01	BHLH13	185	193	1	1	0.945	acACGTGa
AtSK32a	P$ABF4_02	ABF4	185	195	1	1	0.944	acACGTGagt

AtSK32a	P$GBF1_01	GBF1	185	193	1	1	0.927	acACGTGa
AtSK32a	P$ABF3_01	ABF3	185	193	1	1	0.875	ACACGtga
AtSK32a	P$RITA1_01	RITA1	185	192	1	1	0.984	aCACGTg
AtSK32a	P$BHLH66_01	BHLH66	185	193	1	1	0.917	aCACGTga
AtSK32a	P$PIF5_01	IF5	185	193	1	1	0.899	aCACGTga
AtSK32a	P$MYC2_01	MYC2	185	193	1	1	0.95	aCACGTga
AtSK32a	P$MYC3_01	MYC3	185	193	1	1	0.989	aCACGTga
AtSK32a	P$BHLH34_01	BHLH34	185	193	1	1	0.963	aCACGTga
AtSK32a	P$PHYPA48267_08	HYPA48267	185	193	1	1	0.974	aCACGTga
AtSK32a	P$OJ1058_01	OJ1058	185	193	1	1	0.964	aCACGTga
AtSK32a	P$UNE10_01	UNE10	185	193	1	1	0.986	aCACGTga
AtSK32a	P$BHLH3_01	BHLH3	185	193	1	1	0.949	aCACGTga
AtSK32a	P$PIF4_01	IF4	186	194	1	1	0.95	CACGTgag
AtSK32a	P$OCSBF1_01	OCSBF1	186	191	1	1	1	CACGT
AtSK32a	P$ABI5_Q2	ABI5	187	193	1	1	0.936	ACGTGa
AtSK32a	P$ABI3_01	ABI3	193	202	1	1	0.873	gtGCATGaa
AtSK32a	P$AT3G60580_01	AT3G60580	210	217	1	1	0.905	caATCCC
AtSK32a	P$SPF1_Q2	SPF1	217	227	1	1	0.883	ttATAGTtat
AtSK32a	P$LEC2_01	LEC2	231	242	1	1	0.984	tcCATGCaatg
AtSK32a	P$GAMYB_Q2	GAMYB	240	253	1	1	0.918	tgtagACAACtac
AtSK32a	P$C1_Q2	C1	245	256	1	1	0.944	acAACTAcaac
AtSK32a	P$GAMYB_Q2	GAMYB	246	259	1	1	0.908	caactACAACgaa
AtSK32a	P$ATHSFA1D_01	ATHSFA1D	248	254	1	1	1	aCTACA
AtSK32a	P$AT5G54070_01	AT5G54070	251	257	1	1	0.958	aCAACG
AtSK32a	P$PBF_Q2	BF	256	262	1	1	0.965	gAAAGG
AtSK32a	P$MYB24_01	MYB24	267	276	1	1	0.962	tgaTTAGGt
AtSK32a	P$MYB131_01	MYB131	267	278	1	1	0.917	tgaTTAGGttg
AtSK32a	P$MYB4_01	MYB4	269	277	1	1	0.862	atTAGGTt
AtSK32a	P$GATA9_01	GATA9	304	315	1	1	0.91	cctAGATCcaa
AtSK32a	P$AGP1_01	AGP1	305	315	1	1	0.928	ctAGATCcaa
AtSK32a	P$GATA10_01	GATA10	306	314	1	1	0.884	tAGATCca
AtSK32a	P$ARR10_01	ARR10	307	314	1	1	0.934	AGATCca
AtSK32a	P$HSFA2_01	HSFA2	311	317	1	1	0.933	CCAAAg
AtSK32a	P$PBF_Q2	BF	312	318	1	1	0.958	cAAAGG
AtSK32a	P$DOF1_01	DOF1	356	367	1	1	0.989	atcTAAAGcct
AtSK32a	P$DOF2_01	DOF2	356	367	1	1	0.995	atctAAAGCct
AtSK32a	P$DOF3_01	DOF3	356	367	1	1	0.979	atctAAAGCct
AtSK32a	P$C1_Q2	C1	371	382	1	1	0.949	ttAACTActct
AtSK32a	P$HSFA4A_01	HSFA4A	379	385	1	1	0.914	tCTATT
AtSK32a	P$P_01		387	396	1	1	0.879	ggCTACCaa
AtSK32a	P$TCP2_01	TCP2	416	426	1	1	0.854	ttGGGCCaat
AtSK32a	P$ATHB7_01	ATHB7	421	431	1	1	1	ccAATCAtta
AtSK32a	P$HAT1_01	HAT1	421	431	1	1	1	ccAATCAtta
AtSK32a	P$PHV_02	HV	421	436	1	1	0.887	ccaATCATtaactaa
AtSK32a	P$ATHB4_02	ATHB4	422	432	1	1	0.955	caATCATtaa
AtSK32a	P$C1_Q2	C1	428	439	1	1	0.935	ttAACTAattt
AtSK32a	P$AZF3_01	AZF3	464	475	1	1	0.9	gAGTATatact
AtSK32a	P$ATHB1_01	ATHB1	470	484	1	1	0.878	atactATTATtaca
AtSK32a	P$HSFA4A_01	HSFA4A	472	478	1	1	1	aCTATT
AtSK32a	P$ATHB5_01	ATHB5	473	482	1	1	0.906	ctaTTATTa
AtSK32a	P$ATHB1_03	ATHB1	488	499	1	1	0.903	acAATAAtatt
AtSK32a	P$ATHB5_04	ATHB5	488	499	1	1	0.923	acAATAAtatt
AtSK32a	P$ATHB6_01	ATHB6	488	497	1	1	0.996	acAATAAta
AtSK32a	P$ATHB16_01	ATHB16	489	497	1	1	0.878	cAATAAta
AtSK32a	P$ATHB1_01	ATHB1	491	505	1	1	0.85	ataatATTATtatt
AtSK32a	P$ATHB1_01	ATHB1	494	508	1	1	0.868	atattATTATtaca
AtSK32a	P$AT2G41690_01	AT2G41690	514	520	1	1	0.978	CCGAAa
AtSK32a	P$PBF_01	BF	518	529	1	1	0.985	aagAAAAGagt
AtSK32a	P$DOF_Q2	DOF	518	529	1	1	0.948	aagAAAAGagt
AtSK32a	P$CDF2_01	CDF2	519	529	1	1	0.953	agAAAAGagt
AtSK32a	P$BPC1_Q2	BPC1	519	525	1	1	0.997	AGAAAa
AtSK32a	P$CDF3_01	CDF3	520	529	1	1	0.979	gAAAAGagt
AtSK32a	P$WRKY60_01	WRKY60	524	535	1	1	0.903	agaGTCAAaag
AtSK32a	P$WRKY57_01	WRKY57	524	534	1	1	0.983	agaGTCAAaa
AtSK32a	P$WRKY48_02	WRKY48	524	534	1	1	0.995	agaGTCAAaa
AtSK32a	P$WRKY21_02	WRKY21	524	534	1	1	0.968	agaGTCAAaa
AtSK32a	P$WRKY18_02	WRKY18	524	534	1	1	0.979	agaGTCAAaa
AtSK32a	P$WRKY40_03	WRKY40	524	534	1	1	0.996	agAGTCAaaa
AtSK32a	P$WRKY8_01	WRKY8	525	534	1	1	0.991	gaGTCAAaa
AtSK32a	P$WRKY75_01	WRKY75	525	533	1	1	0.976	gaGTCAAa
AtSK32a	P$WRKY63_01	WRKY63	525	533	1	1	0.904	gaGTCAAa
AtSK32a	P$WRKY62_01	WRKY62	525	533	1	1	0.866	gaGTCAAa
AtSK32a	P$WRKY43_02	WRKY43	525	535	1	1	0.99	gaGTCAAaag
AtSK32a	P$WRKY40_01	WRKY40	525	533	1	1	0.996	gaGTCAAa
AtSK32a	P$WRKY25_02	WRKY25	525	533	1	1	0.914	gaGTCAAa
AtSK32a	P$WRKY2_01	WRKY2	525	533	1	1	0.94	gaGTCAAa
AtSK32a	P$WRKY15_01	WRKY15	525	535	1	1	0.98	gaGTCAAaag
AtSK32a	P$WRKY30_01	WRKY30	526	536	1	1	0.914	aGTCAAaagg

AtSK32a	P$WRKY23_01	WRKY23	526	534	1	1	0.889	aGTCAAaa
AtSK32a	P$WRKY18_Q2	WRKY18	527	536	1	1	0.954	GTCAAagg
AtSK32a	P$PBF_01	BF	527	538	1	1	0.967	gtcAAAAGgaa
AtSK32a	P$DOF_Q2	DOF	527	538	1	1	0.938	gtcAAAAGgaa
AtSK32a	P$CDF2_01	CDF2	528	538	1	1	0.951	tcAAAAGgaa
AtSK32a	P$CDF3_01	CDF3	529	538	1	1	0.972	cAAAAGgaa
AtSK32a	P$PBF_Q2	BF	530	536	1	1	1	aAAAGG
AtSK32a	P$WRKY60_01	WRKY60	537	548	1	1	0.905	agaGTCAAact
AtSK32a	P$WRKY57_01	WRKY57	537	547	1	1	0.983	agaGTCAAac
AtSK32a	P$WRKY48_02	WRKY48	537	547	1	1	0.995	agaGTCAAac
AtSK32a	P$WRKY21_02	WRKY21	537	547	1	1	0.969	agaGTCAAac
AtSK32a	P$WRKY18_02	WRKY18	537	547	1	1	0.979	agaGTCAAac
AtSK32a	P$WRKY40_03	WRKY40	537	547	1	1	0.996	agAGTCAaac
AtSK32a	P$WRKY8_01	WRKY8	538	547	1	1	0.991	gaGTCAAac
AtSK32a	P$WRKY75_01	WRKY75	538	546	1	1	0.976	gaGTCAAa
AtSK32a	P$WRKY63_01	WRKY63	538	546	1	1	0.904	gaGTCAAa
AtSK32a	P$WRKY62_01	WRKY62	538	546	1	1	0.866	gaGTCAAa
AtSK32a	P$WRKY43_02	WRKY43	538	548	1	1	0.988	gaGTCAAact
AtSK32a	P$WRKY40_01	WRKY40	538	546	1	1	0.996	gaGTCAAa
AtSK32a	P$WRKY25_02	WRKY25	538	546	1	1	0.914	gaGTCAAa
AtSK32a	P$WRKY2_01	WRKY2	538	546	1	1	0.94	gaGTCAAa
AtSK32a	P$WRKY15_01	WRKY15	538	548	1	1	0.98	gaGTCAAact
AtSK32a	P$WRKY30_01	WRKY30	539	549	1	1	0.916	aGTCAAactt
AtSK32a	P$WRKY23_01	WRKY23	539	547	1	1	0.889	aGTCAAac
AtSK32a	P$WRKY18_Q2	WRKY18	540	549	1	1	0.971	GTCAAactt
AtSK32a	P$AT1G15360_01	AT1G15360	546	556	1	1	0.882	ctTCCGAata
AtSK32a	P$AT2G41690_01	AT2G41690	549	555	1	1	0.974	CCGAAt
AtSK32a	P$ATHB1_03	ATHB1	550	561	1	1	0.871	cgAATAAtccg
AtSK32a	P$ATHB5_04	ATHB5	550	561	1	1	0.876	cgAATAAtccg
AtSK32a	P$ATHB6_01	ATHB6	550	559	1	1	0.969	cgAATAAtc
AtSK32a	P$ATHB16_01	ATHB16	551	559	1	1	0.855	gAATAAtc
AtSK32a	P$AT1G66560_01	AT1G66560	563	573	1	1	0.914	cgcTTAACgt
AtSK32a	P$WRKY7_01	WRKY7	564	573	1	1	0.889	gcTTAACgt
AtSK32a	P$AT2G24570_01	AT2G24570	564	573	1	1	0.887	gcTTAACgt
AtSK32a	P$WRKY46_01	WRKY46	564	573	1	1	0.907	gcTTAACgt
AtSK32a	P$AT5G15130_01	AT5G15130	564	573	1	1	0.918	gcTTAACgt
AtSK32a	P$AT5G41570_01	AT5G41570	564	573	1	1	0.89	gcTTAACgt
AtSK32a	P$AT1G68150_01	AT1G68150	564	573	1	1	0.89	gcTTAACgt
AtSK32a	P$AT1G66600_01	AT1G66600	564	573	1	1	0.917	gcTTAACgt
AtSK32a	P$WRKY6_01	WRKY6	564	573	1	1	0.864	gcTTAACgt
AtSK32a	P$AT1G64000_01	AT1G64000	564	573	1	1	0.89	gcTTAACgt
AtSK32a	P$AT1G18860_01	AT1G18860	564	573	1	1	0.923	gcTTAACgt
AtSK32a	P$WRKY21_01	WRKY21	564	573	1	1	0.89	gcTTAACgt
AtSK32a	P$MYB3R5_01	MYB3R5	574	589	1	1	0.911	cgatcgccaCCGTTg
AtSK32a	P$MYB3R4_01	MYB3R4	575	590	1	1	0.913	gatcgccaCCGTTga
AtSK32a	P$MYB3R1_01	MYB3R1	575	590	1	1	0.916	gatcgccaCCGTTga
AtSK32a	P$EDT1_01	EDT1	625	635	1	1	0.906	tatTTAATtc
AtSK32a	P$SQUA_01	SQUA	634	644	1	1	0.918	ccaTTTTTtt
AtSK41	P$CBF1_01	CBF1	20	30	1	1	0.861	tTGCCGaaca
AtSK41	P$ERF019_01	ERF019	20	30	1	1	0.897	tTGCCGaaca
AtSK41	P$JERF1_01	JERF1	20	30	1	1	0.872	tTGCCGaaca
AtSK41	P$CBF1_03	CBF1	20	30	1	1	0.894	tTGCCGaaca
AtSK41	P$AT1G33760_01	AT1G33760	20	30	1	1	0.873	tTGCCGaaca
AtSK41	P$AT1G71520_01	AT1G71520	20	30	1	1	0.881	tTGCCGaaca
AtSK41	P$AT2G41690_01	AT2G41690	23	29	1	1	1	CCGAAc
AtSK41	P$HAHB4_01	HAHB4	30	39	1	1	0.874	aAATGAttg
AtSK41	P$PEND_01	END	44	52	1	1	0.901	gAAGAAgt
AtSK41	P$PIL5_01	IL5	49	63	1	1	0.964	agtgaaccACGTGt
AtSK41	P$PIF3_01	IF3	50	68	1	1	0.898	gtgaacCACGTgtgtaaa
AtSK41	P$AT4G36620_01	AT4G36620	50	58	1	1	0.957	gtgAACCA
AtSK41	P$ABF2_01	ABF2	51	64	1	1	0.951	tgaacCACGTgtg
AtSK41	P$ABF4_Q2	ABF4	51	65	1	1	0.914	tgaaccACGTGtgt
AtSK41	P$ABZ1_01	ABZ1	52	66	1	1	0.904	gaaccACGTGtgta
AtSK41	P$HBI1_01	HBI1	52	64	1	1	0.939	gaacCACGTgtg
AtSK41	P$BZR1_02	BZR1	52	66	1	1	0.987	gaacCACGTgtgta
AtSK41	P$O2_Q4	O2	52	63	1	1	0.854	gaacCACGTgt
AtSK41	P$BZR1_03	BZR1	53	73	1	1	0.87	aaccACGTGtgtaaatagga
AtSK41	P$ABF4_01	ABF4	53	65	1	1	0.959	aacCACGTgtgt
AtSK41	P$ABI5_01	ABI5	53	63	1	1	0.949	aacCACGTgt
AtSK41	P$PIF3_03	IF3	53	63	1	1	0.989	aacCACGTgt
AtSK41	P$GBP_Q6	GBP	53	65	1	1	0.913	aacCACGTgtgt
AtSK41	P$EMBP1_02	EMBP1	54	64	1	1	0.869	accACGTGtg
AtSK41	P$TAF1_Q2	TAF1	54	64	1	1	0.965	accACGTGtg
AtSK41	P$HBP1A_Q2	HBP1A	54	64	1	1	0.913	accACGTGtg
AtSK41	P$TAF1_01	TAF1	54	64	1	1	0.981	accACGTGtg
AtSK41	P$PIF1_01	IF1	54	64	1	1	0.978	accACGTGtg
AtSK41	P$CPRF1_01	CPRF1	54	64	1	1	0.941	acCACGTgtg
AtSK41	P$TGA1A_Q2	TGA1A	54	64	1	1	0.984	acCACGTgtg

AtSK41	P$TGA1B_Q2	TGA1B	54	64	1	1	0.915	acCACGTgtg
AtSK41	P$O2_02	O2	54	64	1	1	0.976	acCACGTgtg
AtSK41	P$CPRF2_Q2	CPRF2	54	64	1	1	0.995	acCACGTgtg
AtSK41	P$CPRF3_Q2	CPRF3	54	64	1	1	0.98	acCACGTgtg
AtSK41	P$CPRF_Q2	CPRF	54	64	1	1	0.932	acCACGTgtg
AtSK41	P$EMBP1_Q2	EMBP1	54	64	1	1	0.929	acCACGTgtg
AtSK41	P$CPRF3_01	CPRF3	54	64	1	1	0.982	acCACGTgtg
AtSK41	P$GBF1F_Q2	GBF1F	54	65	1	1	0.934	acCACGTgtgt
AtSK41	P$SPT_01	SPT	54	63	1	1	0.997	acCACGTgt
AtSK41	P$PHYPA72483_07	HYPA72483	54	64	1	1	0.997	acCACGTgtg
AtSK41	P$PHYPA143875_02	HYPA143875	54	64	1	1	0.997	acCACGTgtg
AtSK41	P$BIM3_01	BIM3	54	64	1	1	0.991	acCACGTgtg
AtSK41	P$BIM2_01	BIM2	54	64	1	1	0.994	acCACGTgtg
AtSK41	P$BEE2_01	BEE2	54	64	1	1	0.999	acCACGTgtg
AtSK41	P$PIF3_04	IF3	54	64	1	1	0.882	acCACGTgtg
AtSK41	P$BES1_01	BES1	54	65	1	1	0.961	acCACGTgtgt
AtSK41	P$TGA1B_01	TGA1B	54	64	1	1	0.89	acCACGTgtg
AtSK41	P$CPRF2_01	CPRF2	54	64	1	1	0.997	acCACGTgtg
AtSK41	P$MYC4_01	MYC4	55	63	1	1	0.956	ccACGTGt
AtSK41	P$BIM1_02	BIM1	55	65	1	1	0.995	ccACGTGtgt
AtSK41	P$BHLH13_01	BHLH13	55	63	1	1	0.947	ccACGTGt
AtSK41	P$ABF4_02	ABF4	55	65	1	1	0.985	ccACGTGtgt
AtSK41	P$GBF1_01	GBF1	55	63	1	1	0.976	ccACGTGt
AtSK41	P$RITA1_01	RITA1	55	62	1	1	0.997	cCACGTg
AtSK41	P$BHLH66_01	BHLH66	55	63	1	1	0.917	cCACGTgt
AtSK41	P$PIF5_01	IF5	55	63	1	1	0.931	cCACGTgt
AtSK41	P$MYC2_01	MYC2	55	63	1	1	0.953	cCACGTgt
AtSK41	P$MYC3_01	MYC3	55	63	1	1	0.994	cCACGTgt
AtSK41	P$BHLH3_01	BHLH3	55	63	1	1	0.952	cCACGTgt
AtSK41	P$UNE10_01	UNE10	55	63	1	1	0.988	cCACGTgt
AtSK41	P$OJ1058_01	OJ1058	55	63	1	1	0.973	cCACGTgt
AtSK41	P$PHYPA48267_08	HYPA48267	55	63	1	1	0.978	cCACGTgt
AtSK41	P$BHLH34_01	BHLH34	55	63	1	1	0.961	cCACGTgt
AtSK41	P$OCSBF1_01	OCSBF1	56	61	1	1	1	CACGT
AtSK41	P$PIF4_01	IF4	56	64	1	1	0.958	CACGTgtg
AtSK41	P$ABI5_Q2	ABI5	57	63	1	1	0.979	ACGTGt
AtSK41	P$GT1_Q6	GT1	63	70	1	1	1	GTAAAta
AtSK41	P$TEIL_01	TEIL	76	84	1	1	0.94	ATGTAgct
AtSK41	P$GAMYB_Q2	GAMYB	89	102	1	1	0.889	tatccACAACttt
AtSK41	P$LIM1_01	LIM1	103	115	1	1	0.946	CCACCagaaaat
AtSK41	P$BPC1_Q2	BPC1	108	114	1	1	0.997	AGAAAa
AtSK41	P$AGL65_01	AGL65	123	135	1	1	0.91	gctaaaTTTAAt
AtSK41	P$AGL12_01	AGL12	123	135	1	1	0.906	gctAAATTtaat
AtSK41	P$AGL4_01	AGL4	123	135	1	1	0.993	gcTAAATttaat
AtSK41	P$AGL20_01	AGL20	123	135	1	1	0.916	gcTAAATttaat
AtSK41	P$SHP2_01	SHP2	124	135	1	1	0.863	ctaaaTTTAAt
AtSK41	P$AT1G77950_01	AT1G77950	124	135	1	1	0.87	ctaaaTTTAAt
AtSK41	P$SBF1_01	SBF1	124	138	1	1	0.853	ctaaatTTAATtat
AtSK41	P$EDT1_01	EDT1	127	137	1	1	0.89	aatTTAATta
AtSK41	P$SBF1_01	SBF1	170	184	1	1	0.942	gtaatgTTAATaag
AtSK41	P$ATHB6_01	ATHB6	176	185	1	1	0.917	ttAATAAgt
AtSK41	P$SPL11_01	SPL11	179	191	1	1	0.907	ataaGTACGttc
AtSK41	P$SPL5_01	SPL5	181	190	1	1	0.972	aaGTACGtt
AtSK41	P$SPL4_01	SPL4	182	191	1	1	0.992	aGTACGttc
AtSK41	P$SPL12_01	SPL12	182	190	1	1	0.977	aGTACGtt
AtSK41	P$POPTR_01	OPTR	182	189	1	1	0.93	aGTACGt
AtSK41	P$DOF2_01	DOF2	196	207	1	1	0.983	catgAAAGCct
AtSK41	P$DOF3_01	DOF3	196	207	1	1	0.985	catgAAAGCct
AtSK41	P$SED_Q2	SED	200	210	1	1	0.928	aaagCCTTTt
AtSK41	P$GT1_Q6_02	GT1	202	214	1	1	0.957	agccttTTAACa
AtSK41	P$PBF_Q2_01	BF	204	210	1	1	1	CCTTTt
AtSK41	P$ATHB4_02	ATHB4	222	232	1	1	0.867	cgATCATtat
AtSK41	P$SBF1_01	SBF1	244	258	1	1	0.927	gtttgtTTAATaaa
AtSK41	P$ATHB6_01	ATHB6	250	259	1	1	0.91	ttAATAAaa
AtSK41	P$ATHB4_02	ATHB4	285	295	1	1	0.871	tcATCATtca
AtSK41	P$AT1G53910_01	AT1G53910	308	318	1	1	0.874	gGGCCGgaga
AtSK41	P$ATHB7_01	ATHB7	326	336	1	1	0.95	gcAATCAaaa
AtSK41	P$HAT1_01	HAT1	326	336	1	1	0.883	gcAATCAaaa
AtSK41	P$RIN_Q2_01	RIN	326	338	1	1	0.896	gcaatcAAAAGc
AtSK41	P$DOF3_01	DOF3	329	340	1	1	0.98	atcaAAAGCct
AtSK41	P$DOF2_01	DOF2	329	340	1	1	0.99	atcaAAAGCct
AtSK41	P$DOF_Q2	DOF	329	340	1	1	0.922	atcAAAAGcct
AtSK41	P$PBF_01	BF	329	340	1	1	0.971	atcAAAAGcct
AtSK41	P$CDF2_01	CDF2	330	340	1	1	0.951	tcAAAAGcct
AtSK41	P$CDF3_01	CDF3	331	340	1	1	0.973	cAAAAGcct
AtSK41	P$MYBAS1_01	MYBAS1	336	347	1	1	0.981	gcCTAACacat
AtSK41	P$AT4G04450_01	AT4G04450	344	353	1	1	0.866	catTTAGCt
AtSK41	P$MYBAS1_01	MYBAS1	357	368	1	1	0.955	atCCAACtaca

AtSK41	P$C1_Q2	C1	359	370	1	1	0.969	ccAACTAcata
AtSK41	P$ATHSFA1D_01	ATHSFA1D	362	368	1	1	1	aCTACA
AtSK41	P$NAC6_01	NAC6	382	388	1	1	0.854	tCGTAA
AtSK41	P$ATHB7_01	ATHB7	385	395	1	1	0.924	taAATCAttg
AtSK41	P$HAT1_01	HAT1	385	395	1	1	0.981	taAATCAttg
AtSK41	P$ATHB4_02	ATHB4	386	396	1	1	0.961	aaATCATtgt
AtSK41	P$HMG1_01	HMG1	399	408	1	1	0.925	GTTGTtgtt
AtSK41	P$WRKY60_01	WRKY60	407	418	1	1	0.994	ttgGTCAAcac
AtSK41	P$WRKY57_01	WRKY57	407	417	1	1	0.984	ttgGTCAAca
AtSK41	P$WRKY48_02	WRKY48	407	417	1	1	1	ttgGTCAAca
AtSK41	P$WRKY21_02	WRKY21	407	417	1	1	0.995	ttgGTCAAca
AtSK41	P$WRKY18_02	WRKY18	407	417	1	1	0.998	ttgGTCAAca
AtSK41	P$WRKY8_01	WRKY8	408	417	1	1	0.999	tgGTCAAca
AtSK41	P$WRKY75_01	WRKY75	408	416	1	1	0.998	tgGTCAAc
AtSK41	P$WRKY63_01	WRKY63	408	416	1	1	0.999	tgGTCAAc
AtSK41	P$WRKY62_01	WRKY62	408	416	1	1	1	tgGTCAAc
AtSK41	P$WRKY43_02	WRKY43	408	418	1	1	0.985	tgGTCAAcac
AtSK41	P$WRKY40_01	WRKY40	408	416	1	1	1	tgGTCAAc
AtSK41	P$WRKY25_02	WRKY25	408	416	1	1	0.994	tgGTCAAc
AtSK41	P$WRKY2_01	WRKY2	408	416	1	1	0.998	tgGTCAAc
AtSK41	P$WRKY15_01	WRKY15	408	418	1	1	0.997	tgGTCAAcac
AtSK41	P$WRKY23_01	WRKY23	409	417	1	1	0.925	gGTCAAca
AtSK41	P$WRKY30_01	WRKY30	409	419	1	1	0.992	gGTCAAcact
AtSK41	P$RAV1_01	RAV1	409	421	1	1	0.934	ggtCAACActaa
AtSK41	P$WRKY18_Q2	WRKY18	410	419	1	1	0.93	GTCAAcact
AtSK41	P$ATHB7_01	ATHB7	419	429	1	1	0.922	aaAATCAttg
AtSK41	P$HAT1_01	HAT1	419	429	1	1	0.981	aaAATCAttg
AtSK41	P$ATHB4_02	ATHB4	420	430	1	1	0.961	aaATCATtgt
AtSK41	P$SBF1_01	SBF1	423	437	1	1	0.888	tcattgTTAAAtgg
AtSK41	P$PDF2_01	DF2	426	437	1	1	0.858	ttgtTAAATgg
AtSK41	P$DOF_Q2	DOF	462	473	1	1	0.942	agcAAAAGaaa
AtSK41	P$PBF_01	BF	462	473	1	1	0.966	agcAAAAGaaa
AtSK41	P$CDF2_01	CDF2	463	473	1	1	0.947	gcAAAAGaaa
AtSK41	P$CDF3_01	CDF3	464	473	1	1	0.969	cAAAAGaaa
AtSK41	P$DOF2_01	DOF2	466	477	1	1	0.986	aaagAAAGCaa
AtSK41	P$DOF3_01	DOF3	466	477	1	1	0.99	aaagAAAGCaa
AtSK41	P$BPC1_Q2	BPC1	468	474	1	1	1	AGAAAg
AtSK41	P$DOF43_01	DOF43	470	481	1	1	0.957	aaagcaACTTT
AtSK41	P$SED_Q2	SED	479	489	1	1	0.98	ttagCCTTTt
AtSK41	P$PBF_Q2_01	BF	483	489	1	1	1	CCTTTt
AtSK41	P$MYB24_01	MYB24	485	494	1	1	0.939	tttTTAGGt
AtSK41	P$MYB131_01	MYB131	485	496	1	1	0.918	tttTTAGGtta
AtSK41	P$LIM1_01	LIM1	501	513	1	1	0.966	CCACCactccct
AtSK41	P$MYB1L_01	MYB1L	507	517	1	1	0.936	ctCCCTAttt
AtSK41	P$TRB2_01	TRB2	507	515	1	1	0.92	ctCCCTAt
AtSK41	P$HSFA4A_01	HSFA4A	510	516	1	1	0.964	cCTATT
AtSK41	P$SED_Q2	SED	569	579	1	1	0.91	tagcCCTTTt
AtSK41	P$SQUA_01	SQUA	572	582	1	1	0.865	cccTTTTTtg
AtSK41	P$SEP3_01	wrz-03	572	583	1	1	0.913	cccttTTTTGc
AtSK41	P$PBF_Q2_01	BF	573	579	1	1	1	CCTTTt
AtSK41	P$SQUA_01	SQUA	573	583	1	1	0.928	cctTTTTTgc
AtSK41	P$GT1_Q6_01	GT1	576	588	1	1	0.907	TTTTTgcttatt
AtSK41	P$CBNAC_02	CBNAC	579	595	1	1	0.862	tTGCTTattctaatta
AtSK41	P$CBNAC_01	CBNAC	579	585	1	1	1	tTGCTT
AtSK41	P$GT1_Q6	GT1	595	602	1	1	1	GTAAAta
AtSK41	P$ATHB6_01	ATHB6	596	605	1	1	0.908	taAATAAga
AtSK41	P$GATA9_01	GATA9	599	610	1	1	0.883	ataAGATCacg
AtSK41	P$ARR10_01	ARR10	602	609	1	1	0.869	AGATCac
AtSK41	P$FHY3_01	FHY3	604	616	1	1	0.853	atcACGCGgaga
AtSK41	P$AT4G36620_01	AT4G36620	628	636	1	1	0.898	agtAACCA
AtSK41	P$DOF3_01	DOF3	631	642	1	1	0.99	aaccAAAGCaa
AtSK41	P$DOF2_01	DOF2	631	642	1	1	0.984	aaccAAAGCaa
AtSK41	P$HSFA2_01	HSFA2	633	639	1	1	0.933	CCAAAg
AtSK41	P$ABI3_01	ABI3	643	652	1	1	0.963	gtGCATGcc
AtSK41	P$LEC2_01	LEC2	644	655	1	1	0.947	tgCATGCctaa
AtSK41	P$FUS3_01	FUS3	644	653	1	1	0.952	tGCATGcct
AtSK41	P$MYBAS1_01	MYBAS1	649	660	1	1	0.982	gcCTAACgtgt
AtSK41	P$ABZ1_01	ABZ1	649	663	1	1	0.904	gcctaACGTGtatt
AtSK41	P$TAF1_01	TAF1	651	661	1	1	0.946	ctaACGTGta
AtSK41	P$EMBP1_02	EMBP1	651	661	1	1	0.882	ctaACGTGta
AtSK41	P$HBP1A_Q2	HBP1A	651	661	1	1	0.859	ctaACGTGta
AtSK41	P$TAF1_Q2	TAF1	651	661	1	1	0.916	ctaACGTGta
AtSK41	P$ABF4_02	ABF4	652	662	1	1	0.993	taACGTGtat
AtSK41	P$BIM1_02	BIM1	652	662	1	1	0.946	taACGTGtat
AtSK41	P$GBF1_01	GBF1	652	660	1	1	0.978	taACGTGt
AtSK41	P$ABI5_Q2	ABI5	654	660	1	1	0.979	ACGTGt
AtSK41	P$ATMYB46_Q6	ATMYB46	673	683	1	1	0.915	CTCACcaaag
AtSK41	P$DOF2_01	DOF2	675	686	1	1	0.984	caccAAAGCaa

AtSK41	P$DOF3_01	DOF3	675	686	1	1	0.988	caccAAAGCaa
AtSK41	P$HSFA2_01	HSFA2	677	683	1	1	0.933	CCAAAg
AtSK41	P$ATHB6_01	ATHB6	685	694	1	1	0.979	aaAATAAtt
AtSK41	P$ATHB5_04	ATHB5	685	696	1	1	0.963	aaAATAAttaa
AtSK41	P$ATHB1_03	ATHB1	685	696	1	1	0.984	aaAATAAttaa
AtSK41	P$ATHB16_01	ATHB16	686	694	1	1	0.953	aAATAAtt
AtSK41	P$GT1_Q6_02	GT1	686	698	1	1	0.862	aaataaTTAACt
AtSK41	P$GT1_Q6	GT1	706	713	1	1	0.971	GTGAAaa
AtSK41	P$TEIL_01	TEIL	718	726	1	1	0.925	ATGTAttt
AtSK41	P$GATA15_01	GATA15	723	732	1	1	1	ttTGATCaa
AtSK41	P$ARR2_01	ARR2	727	737	1	1	0.867	atcaATCTTt
AtSK41	P$AT2G38090_01	AT2G38090	733	745	1	1	0.87	cttTTCGTattt
AtSK41	P$AT4G36620_01	AT4G36620	763	771	1	1	0.915	taaAACCA
AtSK41	P$ARR18_01	ARR18	772	785	1	1	0.896	ttgcAGATAaaaa
AtSK41	P$SBF1_01	SBF1	796	810	1	1	0.883	taagctTTAATatc
AtSK41	P$EDT1_01	EDT1	799	809	1	1	0.858	gctTTAATat
AtSK41	P$ATHB6_01	ATHB6	811	820	1	1	0.91	ctAATAAaa
AtSK41	P$PBF_01	BF	849	860	1	1	0.961	acaAAAAGaag
AtSK41	P$DOF_Q2	DOF	849	860	1	1	1	acaAAAAGaag
AtSK41	P$CDF2_01	CDF2	850	860	1	1	0.977	caAAAAGaag
AtSK41	P$CDF3_01	CDF3	851	860	1	1	0.974	aAAAAGaag
AtSK41	P$END_01	END	853	861	1	1	0.891	aAAGAAgt
AtSK41	P$MYB305_Q3	MYB305	873	886	1	1	0.896	gccttACCTAact
AtSK41	P$MYB61_01	MYB61	875	890	1	1	0.882	cttACCTAactgtcg
AtSK41	P$MYBAS1_01	MYBAS1	878	889	1	1	0.991	acCTAACtgtc
AtSK41	P$ARF8_01	ARF8	883	892	1	1	0.956	acTGTCGac
AtSK41	P$DOF1_01	DOF1	892	903	1	1	0.982	aagTAAAGgcg
AtSK41	P$PBF_Q2	BF	895	901	1	1	0.986	tAAAGG
AtSK41	P$ATHB6_01	ATHB6	914	923	1	1	0.903	atAATAAcc
AtSK41	P$AT4G36620_01	AT4G36620	916	924	1	1	0.916	aatAACCA
AtSK41	P$ATHB1_01	ATHB1	950	964	1	1	0.936	ttgaaATTATtttt
AtSK41	P$ATHB5_01	ATHB5	953	962	1	1	0.915	aaaTTATTt
AtSK41	P$MYB1L_01	MYB1L	964	974	1	1	0.978	tgCCCTAcaa
AtSK41	P$TRB2_01	TRB2	964	972	1	1	0.984	tgCCCTAc
AtSK41	P$GAMYB_Q2	GAMYB	965	978	1	1	0.919	gccctACAACagt
AtSK41	P$ATHSFA1D_01	ATHSFA1D	967	973	1	1	0.985	cCTACA
AtSK41	P$RAV1_01	RAV1	968	980	1	1	0.949	ctaCAACAgtat
AtSK41	P$EDT1_01	EDT1	978	988	1	1	0.869	attTTAATtc
AtSK41	P$BPC1_Q2	BPC1	991	997	1	1	0.997	AGAAAa
AtSK41	P$HMG1_01	HMG1	1012	1021	1	1	0.915	GTTGTttta
AtSK41	P$ATHB7_01	ATHB7	1031	1041	1	1	0.873	aaAATCAaaa
AtSK41	P$HAT1_01	HAT1	1031	1041	1	1	0.865	aaAATCAaaa
AtSK41	P$DOF_Q2	DOF	1034	1045	1	1	0.926	atcAAAAGgca
AtSK41	P$PBF_01	BF	1034	1045	1	1	0.972	atcAAAAGgca
AtSK41	P$CDF2_01	CDF2	1035	1045	1	1	0.944	tcAAAAGgca
AtSK41	P$CDF3_01	CDF3	1036	1045	1	1	0.969	cAAAAGgca
AtSK41	P$PBF_Q2	BF	1037	1043	1	1	1	aAAAGG
AtSK41	P$AT5G04240_01	AT5G04240	1040	1046	1	1	0.939	aGGCAC
AtSK41	P$AT3G60580_01	AT3G60580	1058	1065	1	1	0.883	aaATCCC
AtSK41	P$KNOX3_01	KNOX3	1097	1109	1	1	0.958	gtgtTGACAaaa
AtSK41	P$WRKY11_Q2	WRKY11	1099	1107	1	1	0.925	gTTGACaa
AtSK41	P$ATH1_01	ATH1	1101	1109	1	1	0.923	TGACAaaa
AtSK41	P$HAT1_01	HAT1	1132	1142	1	1	0.865	gtAATCAcaa
AtSK41	P$ATHB7_01	ATHB7	1132	1142	1	1	0.854	gtAATCAcaa
AtSK41	P$HMG1_01	HMG1	1145	1154	1	1	0.992	GTTGTtttc
AtSK41	P$ATHB6_01	ATHB6	1178	1187	1	1	0.912	gaAATAAgt
AtSK41	P$ATSPL8_01	ATSPL8	1182	1198	1	1	0.936	taagtTGTACaccaaa
AtSK41	P$HMG1_01	HMG1	1185	1194	1	1	0.873	GTTGTacac
AtSK41	P$HSFA2_01	HSFA2	1193	1199	1	1	1	CCAAAa
AtSK41	P$PBF_01	BF	1193	1204	1	1	0.948	ccaAAAAGttt
AtSK41	P$DOF_Q2	DOF	1193	1204	1	1	0.977	ccaAAAAGttt
AtSK41	P$CDF2_01	CDF2	1194	1204	1	1	0.994	caAAAAGttt
AtSK41	P$CDF3_01	CDF3	1195	1204	1	1	0.989	aAAAAGttt
AtSK41	P$MYB80_01	MYB80	1202	1213	1	1	0.947	ttGAATAtccg
AtSK41	P$MYB118_01	MYB118	1204	1221	1	1	0.853	gaatatccgGTTACaaa
AtSK41	P$ATMYB77_01	ATMYB77	1205	1218	1	1	0.869	aatatcCGGTTac
AtSK41	P$MYBPH3_01	MYBPH3	1205	1218	1	1	0.891	aatatcCGGTTac
AtSK41	P$MYB24_01	MYB24	1220	1229	1	1	0.964	aaaTTAGGt
AtSK41	P$MYB131_01	MYB131	1220	1231	1	1	0.984	aaaTTAGGtag
AtSK41	P$MYB3_01	MYB3	1221	1232	1	1	0.877	aatTAGGTagg
AtSK41	P$MYB4_01	MYB4	1222	1230	1	1	0.875	atTAGGTa
AtSK41	P$HMG1_01	HMG1	1256	1265	1	1	0.913	GTTGTtatg
AtSK41	P$SBF1_01	SBF1	1276	1290	1	1	0.882	catccaTTAAAaag
AtSK41	P$PBF_01	BF	1282	1293	1	1	0.963	ttaAAAAGtat
AtSK41	P$DOF_Q2	DOF	1282	1293	1	1	0.99	ttaAAAAGtat
AtSK41	P$CDF2_01	CDF2	1283	1293	1	1	0.997	taAAAAGtat
AtSK41	P$CDF3_01	CDF3	1284	1293	1	1	0.99	aAAAAGtat
AtSK41	P$AZF3_01	AZF3	1287	1298	1	1	0.863	aAGTATagtat

AtSK42	P$ARR1_01	ARR1	76	86	1	1	0.946	agtGAATCcc
AtSK42	P$AT3G60580_01	AT3G60580	79	86	1	1	0.932	gaATCCC
AtSK42	P$MYB1L_01	MYB1L	81	91	1	1	0.952	atCCCTAtat
AtSK42	P$TRB2_01	TRB2	81	89	1	1	0.928	atCCCTAt
AtSK42	P$GT1_Q6_02	GT1	107	119	1	1	0.964	tgattgTTAACa
AtSK42	P$ATHB6_01	ATHB6	138	147	1	1	0.927	ccAATAAag
AtSK42	P$DOF1_01	DOF1	139	150	1	1	0.982	caaTAAAGcca
AtSK42	P$DOF2_01	DOF2	139	150	1	1	0.999	caatAAAGCca
AtSK42	P$DOF3_01	DOF3	139	150	1	1	0.991	caatAAAGCca
AtSK42	P$AT3G60580_01	AT3G60580	149	156	1	1	0.852	acATCCC
AtSK42	P$BHLH112_01	BHLH112	153	162	1	1	1	cccACTTGa
AtSK42	P$SED_Q2	SED	185	195	1	1	0.896	tatgCCTTTa
AtSK42	P$MYB24_01	MYB24	189	198	1	1	0.907	cctTTAGGc
AtSK42	P$PBF_Q2_01	BF	189	195	1	1	0.998	CCTTTa
AtSK42	P$ATSPL3_01	ATSPL3	194	210	1	1	0.949	aggctCGTACctttcc
AtSK42	P$SMZ_01	SMZ	196	204	1	1	0.935	gcTCGTAc
AtSK42	P$SED_Q2	SED	199	209	1	1	0.909	cgtaCCTTTc
AtSK42	P$PBF_Q2_01	BF	203	209	1	1	0.985	CCTTTc
AtSK42	P$NAC043_01	NAC043	220	230	1	1	0.888	gcaACGTAaa
AtSK42	P$AT5G54070_01	AT5G54070	220	226	1	1	0.915	gCAACG
AtSK42	P$NAC025_01	NAC025	222	230	1	1	0.907	aACGTAaa
AtSK42	P$NAC6_01	NAC6	223	229	1	1	1	aCGTAA
AtSK42	P$AT3G20750_01	AT3G20750	225	233	1	1	0.887	gTAAACgt
AtSK42	P$TGA1A_01	TGA1A	228	235	1	1	0.871	aACGTCt
AtSK42	P$ANAC013_Q2	ANAC013	230	244	1	1	0.853	cgtcttccCCAAGc
AtSK42	P$PEND_01	END	267	275	1	1	0.901	gAAGAAgt
AtSK42	P$CBNAC_02	CBNAC	282	298	1	1	0.896	aTGCTTatttgttttg
AtSK42	P$CBNAC_01	CBNAC	282	288	1	1	0.968	aTGCTT
AtSK42	P$ARR2_01	ARR2	297	307	1	1	0.887	gtatATCTTc
AtSK42	P$GATA15_01	GATA15	311	320	1	1	1	ggTGATCat
AtSK42	P$HV_02	HV	312	327	1	1	0.86	gtgATCATtcctctg
AtSK42	P$ATHB4_02	ATHB4	313	323	1	1	0.871	tgATCATtcc
AtSK42	P$TGA1A_01	TGA1A	339	346	1	1	0.882	tACGTCg
AtSK42	P$DOF3_01	DOF3	345	356	1	1	0.995	gatgAAAGCca
AtSK42	P$DOF2_01	DOF2	345	356	1	1	0.981	gatgAAAGCca
AtSK42	P$BPC1_Q2	BPC1	355	361	1	1	0.99	AGAAAt
AtSK42	P$LEC2_01	LEC2	371	382	1	1	0.999	gcCATGCacat
AtSK42	P$FUS3_Q2	FUS3	372	383	1	1	0.87	cCATGCacatt
AtSK42	P$MYB118_01	MYB118	377	394	1	1	0.853	cacatttcgGTTACaga
AtSK42	P$ATMYB77_01	ATMYB77	378	391	1	1	0.867	acatttCGGTTac
AtSK42	P$PHYPA64121_06	HYPA64121	380	393	1	1	0.879	attTCGGTtacag
AtSK42	P$AT5G04240_01	AT5G04240	396	402	1	1	0.938	tGGCAC
AtSK42	P$BPC1_Q2	BPC1	424	430	1	1	1	AGAAAg
AtSK42	P$PBF_Q2	BF	425	431	1	1	0.965	gAAAGG
AtSK42	P$AT1G66560_01	AT1G66560	441	451	1	1	0.856	agcTTAACgg
AtSK42	P$MYB23_01	MYB23	442	455	1	1	0.852	gcttAACGGaaat
AtSK42	P$AT5G15130_01	AT5G15130	442	451	1	1	0.884	gcTTAACgg
AtSK42	P$AT1G66600_01	AT1G66600	442	451	1	1	0.857	gcTTAACgg
AtSK42	P$AT1G18860_01	AT1G18860	442	451	1	1	0.889	gcTTAACgg
AtSK42	P$AT3G09370_01	AT3G09370	443	455	1	1	0.861	ctTAACGgaaat
AtSK42	P$AT5G11510_01	AT5G11510	443	455	1	1	0.865	ctTAACGgaaat
AtSK42	P$DOF1_01	DOF1	454	465	1	1	0.968	tggTAAAGtat
AtSK42	P$ARR18_01	ARR18	467	480	1	1	0.894	tatgAGATAgaga
AtSK42	P$ARR18_01	ARR18	473	486	1	1	0.889	atagAGATAatgt
AtSK42	P$PEND_02	END	492	502	1	1	0.896	atTTCTTgtc
AtSK42	P$SBF1_01	SBF1	511	525	1	1	0.924	atatccTTAATata
AtSK42	P$DOF1_01	DOF1	520	531	1	1	0.985	ataTAAAGaaa
AtSK42	P$BPC1_Q2	BPC1	526	532	1	1	1	AGAAAg
AtSK42	P$UIF1_01	UIF1	547	557	1	1	0.995	caaGATTCag
AtSK42	P$BPC1_Q2	BPC1	557	563	1	1	0.99	AGAAAc
AtSK42	P$GAMYB_Q2	GAMYB	578	591	1	1	0.931	tggaaACAACtct
AtSK42	P$AT4G36620_01	AT4G36620	594	602	1	1	0.883	cagAACCA
AtSK42	P$HSFA2_01	HSFA2	599	605	1	1	0.941	CCAAAc
AtSK42	P$P_01		615	624	1	1	0.876	taCTACCat
AtSK42	P$AT3G20750_01	AT3G20750	632	640	1	1	0.857	tTAAACac
AtSK42	P$AT1G66560_01	AT1G66560	638	648	1	1	0.958	accTTAACct
AtSK42	P$AT1G30650_01	AT1G30650	639	648	1	1	0.869	ccTTAACct
AtSK42	P$AT2G24570_01	AT2G24570	639	648	1	1	0.961	ccTTAACct
AtSK42	P$AT4G23550_01	AT4G23550	639	648	1	1	0.865	ccTTAACct
AtSK42	P$WRKY7_01	WRKY7	639	648	1	1	0.961	ccTTAACct
AtSK42	P$WRKY25_01	WRKY25	639	648	1	1	0.966	cctTAACCt
AtSK42	P$WRKY33_01	WRKY33	639	648	1	1	0.896	cctTAACCt
AtSK42	P$AT1G29860_01	AT1G29860	639	648	1	1	0.937	cctTAACCt
AtSK42	P$AT3G62340_01	AT3G62340	639	648	1	1	0.908	cctTAACCt
AtSK42	P$AT1G69310_01	AT1G69310	639	648	1	1	0.953	cctTAACCt
AtSK42	P$WRKY26_01	WRKY26	639	648	1	1	0.899	cctTAACCt
AtSK42	P$WRKY46_01	WRKY46	639	648	1	1	0.94	ccTTAACct
AtSK42	P$AT5G15130_01	AT5G15130	639	648	1	1	0.929	ccTTAACct

AtSK42	P$AT1G69810_01	AT1G69810	639	648	1	1	0.869	ccTTAACct
AtSK42	P$WRKY21_01	WRKY21	639	648	1	1	0.961	ccTTAACct
AtSK42	P$AT2G34830_01	AT2G34830	639	648	1	1	0.87	ccTTAACct
AtSK42	P$WRKY43_01	WRKY43	639	648	1	1	0.881	ccTTAACct
AtSK42	P$AT1G18860_01	AT1G18860	639	648	1	1	0.934	ccTTAACct
AtSK42	P$AT1G64000_01	AT1G64000	639	648	1	1	0.976	ccTTAACct
AtSK42	P$AT4G22070_01	AT4G22070	639	648	1	1	0.893	ccTTAACct
AtSK42	P$WRKY6_01	WRKY6	639	648	1	1	0.92	ccTTAACct
AtSK42	P$AT1G66600_01	AT1G66600	639	648	1	1	0.961	ccTTAACct
AtSK42	P$AT1G68150_01	AT1G68150	639	648	1	1	0.928	ccTTAACct
AtSK42	P$AT5G41570_01	AT5G41570	639	648	1	1	0.976	ccTTAACct
AtSK42	P$GT1_01	GT1	640	648	1	1	0.945	ctTAACCt
AtSK42	P$SED_Q2	SED	650	660	1	1	0.97	acttCCTTTt
AtSK42	P$PBF_Q2_01	BF	654	660	1	1	1	CCTTTt
AtSK42	P$SPL11_01	SPL11	680	692	1	1	0.902	ttcgGTACGcat
AtSK42	P$SPL5_01	SPL5	682	691	1	1	0.962	cgGTACGca
AtSK42	P$POPTR_01	OPTR	683	690	1	1	0.929	gGTACGc
AtSK42	P$SPL12_01	SPL12	683	691	1	1	0.971	gGTACGca
AtSK42	P$SPL4_01	SPL4	683	692	1	1	0.988	gGTACGcat
AtSK42	P$SPF1_Q2	SPF1	692	702	1	1	0.928	caATAGTact
AtSK42	P$AT1G69310_01	AT1G69310	708	717	1	1	0.852	ccaTAACCg
AtSK42	P$WRKY25_01	WRKY25	708	717	1	1	0.875	ccaTAACCg
AtSK42	P$BPC1_Q2	BPC1	717	723	1	1	0.99	AGAAAc
AtSK42	P$GAMYB_Q2	GAMYB	735	748	1	1	0.889	ccatcACAACcgc
AtSK42	P$GAMYB_01	GAMYB	741	749	1	1	0.948	CAACCgca
AtSK42	P$AT5G46350_01	AT5G46350	743	752	1	1	0.934	ACCGCacta
AtSK42	P$AT1G66560_01	AT1G66560	761	771	1	1	0.957	gacTTAACtt
AtSK42	P$AT1G69810_01	AT1G69810	762	771	1	1	0.963	acTTAACtt
AtSK42	P$AT5G15130_01	AT5G15130	762	771	1	1	0.987	acTTAACtt
AtSK42	P$WRKY46_01	WRKY46	762	771	1	1	0.917	acTTAACtt
AtSK42	P$AT1G30650_01	AT1G30650	762	771	1	1	0.934	acTTAACtt
AtSK42	P$AT2G24570_01	AT2G24570	762	771	1	1	0.927	acTTAACtt
AtSK42	P$AT4G23550_01	AT4G23550	762	771	1	1	0.928	acTTAACtt
AtSK42	P$WRKY7_01	WRKY7	762	771	1	1	0.923	acTTAACtt
AtSK42	P$AT5G41570_01	AT5G41570	762	771	1	1	0.91	acTTAACtt
AtSK42	P$AT1G68150_01	AT1G68150	762	771	1	1	0.962	acTTAACtt
AtSK42	P$AT1G66600_01	AT1G66600	762	771	1	1	0.959	acTTAACtt
AtSK42	P$WRKY6_01	WRKY6	762	771	1	1	0.946	acTTAACtt
AtSK42	P$AT4G22070_01	AT4G22070	762	771	1	1	0.947	acTTAACtt
AtSK42	P$AT1G64000_01	AT1G64000	762	771	1	1	0.911	acTTAACtt
AtSK42	P$AT1G18860_01	AT1G18860	762	771	1	1	0.987	acTTAACtt
AtSK42	P$AT2G34830_01	AT2G34830	762	771	1	1	0.931	acTTAACtt
AtSK42	P$WRKY21_01	WRKY21	762	771	1	1	0.927	acTTAACtt
AtSK42	P$GAMYB_01	GAMYB	771	779	1	1	0.945	CAACCcac
AtSK42	P$ASR1_01	ASR1	773	778	1	1	1	ACCCA
AtSK42	P$MYB1L_01	MYB1L	779	789	1	1	0.96	ttCCCTAatt
AtSK42	P$TRB2_01	TRB2	779	787	1	1	0.947	ttCCCTAa
AtSK42	P$GAMYB_01	GAMYB	795	803	1	1	0.871	CAACCtta
AtSK42	P$AT3G20750_01	AT3G20750	802	810	1	1	1	aTAAACat
AtSK42	P$AT4G36620_01	AT4G36620	810	818	1	1	0.896	tgaAACCA
AtSK42	P$MYBAS1_01	MYBAS1	813	824	1	1	0.977	aaCCAACcatt
AtSK42	P$AT4G36620_01	AT4G36620	814	822	1	1	0.909	accAACCA
AtSK42	P$GAMYB_01	GAMYB	816	824	1	1	0.869	CAACCatt
AtSK42	P$HSFA4A_01	HSFA4A	833	839	1	1	0.914	tCTATT
AtSK42	P$AG_03	AG	868	886	1	1	0.88	attcCCAAAatcgtcatg
AtSK42	P$AG_01	AG	868	886	1	1	0.88	attcCCAAAatcgtcatg
AtSK42	P$HSFA2_01	HSFA2	872	878	1	1	1	CCAAAa
AtSK42	P$TGA2_Q2	TGA2	878	888	1	1	0.869	tCGTCAtgta
AtSK42	P$TEIL_01	TEIL	883	891	1	1	0.863	ATGTAtca
AtSK42	P$BPC1_Q2	BPC1	895	901	1	1	0.99	AGAAAt
AtSK42	P$ATHSFA1D_01	ATHSFA1D	908	914	1	1	0.94	gCTACA
AtSK42	P$MYBAS1_01	MYBAS1	912	923	1	1	0.954	caCCAACataa
AtSK42	P$RAV1_01	RAV1	912	924	1	1	0.974	cacCAACAtaaa
AtSK42	P$AT3G20750_01	AT3G20750	919	927	1	1	1	aTAAACat
AtSK42	P$ATHB7_01	ATHB7	943	953	1	1	0.852	gtAATCAgct
AtSK42	P$HAT1_01	HAT1	943	953	1	1	0.864	gtAATCAgct
AtSK42	P$SBF1_01	SBF1	968	982	1	1	0.922	ttctctTTAAAaag
AtSK42	P$AT2G15660_01	AT2G15660	968	979	1	1	0.918	TTCTCtttaaa
AtSK42	P$PBF_01	BF	974	985	1	1	0.967	ttaAAAAGacg
AtSK42	P$DOF_Q2	DOF	974	985	1	1	0.977	ttaAAAAGacg
AtSK42	P$CDF2_01	CDF2	975	985	1	1	0.969	taAAAAGacg
AtSK42	P$CDF3_01	CDF3	976	985	1	1	0.97	aAAAAGacg
AtSK42	P$RAV1_01	RAV1	984	996	1	1	0.933	gatCAACAatat
AtSK42	P$DOF3_01	DOF3	992	1003	1	1	0.978	atatAAAGCtc
AtSK42	P$DOF2_01	DOF2	992	1003	1	1	0.998	atatAAAGCtc
AtSK42	P$DOF1_01	DOF1	992	1003	1	1	0.989	ataTAAAGctc
AtSK42	P$GAMYB_Q2	GAMYB	1001	1014	1	1	0.901	tctccACAACatg
AtSK42	P$RAV1_01	RAV1	1004	1016	1	1	0.918	ccaCAACAtgca

AtSK42	P$LEC2_01	LEC2	1008	1019	1	1	0.981	aaCATGCaacc
AtSK42	P$AT4G36620_01	AT4G36620	1012	1020	1	1	0.903	tgcAACCA
AtSK42	P$GAMYB_01	GAMYB	1014	1022	1	1	0.888	CAACCaag
AtSK42	P$LIM1_01	LIM1	1031	1043	1	1	0.975	CCACCacattca
AtSK42	P$MYB305_Q3	MYB305	1057	1070	1	1	0.888	tccatACCTAacg
AtSK42	P$MYB61_01	MYB61	1059	1074	1	1	0.886	catACCTAacgatac
AtSK42	P$MYBAS1_01	MYBAS1	1062	1073	1	1	0.953	acCTAACgata
AtSK42	P$LIM1_01	LIM1	1078	1090	1	1	0.956	CCACCaaacaat
AtSK42	P$HSFA2_01	HSFA2	1081	1087	1	1	0.941	CCAAAc
AtSK42	P$SED_Q2	SED	1087	1097	1	1	0.913	aatgCCTTTc
AtSK42	P$AT2G38090_01	AT2G38090	1091	1103	1	1	0.958	cctTTCGTatcc
AtSK42	P$PBF_Q2_01	BF	1091	1097	1	1	0.985	CCTTTc
AtSK42	P$MYBAS1_01	MYBAS1	1099	1110	1	1	0.976	atCCAACacgt
AtSK42	P$RAV1_01	RAV1	1099	1111	1	1	0.925	atcCAACAcgta
AtSK42	P$ABF2_01	ABF2	1100	1113	1	1	0.962	tccaaCACGTaaa
AtSK42	P$BZR1_02	BZR1	1101	1115	1	1	0.852	ccaaCACGTaaatt
AtSK42	P$ABF4_01	ABF4	1102	1114	1	1	0.914	caaCACGTaaat
AtSK42	P$ABI5_01	ABI5	1102	1112	1	1	0.932	caaCACGTaa
AtSK42	P$GBP_Q6	GBP	1102	1114	1	1	0.888	caaCACGTaaat
AtSK42	P$NAC92_01	NAC92	1102	1114	1	1	0.96	caACACGtaaat
AtSK42	P$CPRF2_01	CPRF2	1103	1113	1	1	0.947	aaCACGTaaa
AtSK42	P$TGA1B_01	TGA1B	1103	1113	1	1	0.85	aaCACGTaaa
AtSK42	P$BEE2_01	BEE2	1103	1113	1	1	0.915	aaCACGTaaa
AtSK42	P$BIM2_01	BIM2	1103	1113	1	1	0.854	aaCACGTaaa
AtSK42	P$BIM3_01	BIM3	1103	1113	1	1	0.888	aaCACGTaaa
AtSK42	P$PHYPA143875_02	HYPA143875	1103	1113	1	1	0.875	aaCACGTaaa
AtSK42	P$SPT_01	SPT	1103	1112	1	1	0.914	aaCACGTaa
AtSK42	P$NAC043_01	NAC043	1103	1113	1	1	0.86	aacACGTAaa
AtSK42	P$CPRF3_01	CPRF3	1103	1113	1	1	0.93	aaCACGTaaa
AtSK42	P$CPRF3_Q2	CPRF3	1103	1113	1	1	0.919	aaCACGTaaa
AtSK42	P$CPRF2_Q2	CPRF2	1103	1113	1	1	0.945	aaCACGTaaa
AtSK42	P$O2_02	O2	1103	1113	1	1	0.953	aaCACGTaaa
AtSK42	P$TGA1B_Q2	TGA1B	1103	1113	1	1	0.905	aaCACGTaaa
AtSK42	P$TGA1A_Q2	TGA1A	1103	1113	1	1	0.971	aaCACGTaaa
AtSK42	P$RITA1_01	RITA1	1104	1111	1	1	0.974	aCACGTa
AtSK42	P$OCSBF1_01	OCSBF1	1105	1110	1	1	1	CACGT
AtSK42	P$NAC025_01	NAC025	1105	1113	1	1	0.95	cACGTAaa
AtSK42	P$NAC6_01	NAC6	1106	1112	1	1	1	aCGTAA
AtSK42	P$ATHB1_03	ATHB1	1127	1138	1	1	0.897	acAATAAtatc
AtSK42	P$ATHB5_04	ATHB5	1127	1138	1	1	0.92	acAATAAtatc
AtSK42	P$ATHB6_01	ATHB6	1127	1136	1	1	0.996	acAATAAta
AtSK42	P$ATHB16_01	ATHB16	1128	1136	1	1	0.878	cAATAAta
AtSK42	P$ARR2_01	ARR2	1152	1162	1	1	0.886	tctgATCTTt
AtSK42	P$GATA15_01	GATA15	1152	1161	1	1	0.999	tcTGATCtt
AtSK42	P$GATA8_01	GATA8	1153	1162	1	1	0.975	ctGATCTtt
AtSK21	P$ATMYB77_01	ATMYB77	1	14	1	1	0.867	ttgcaaCGGTTgg
AtSK21	P$AT5G54070_01	AT5G54070	3	9	1	1	0.915	gCAACG
AtSK21	P$RAP21_02	RAP21	17	30	1	1	0.934	aggaCGGTGgggt
AtSK21	P$ALFIN1_Q2	ALFIN1	17	32	1	1	0.965	aggacgGTGGGgtcg
AtSK21	P$NAC6_01	NAC6	29	35	1	1	0.854	tCGTAA
AtSK21	P$WRKY40_03	WRKY40	32	42	1	1	0.999	taAGTCAaca
AtSK21	P$WRKY18_02	WRKY18	32	42	1	1	0.981	taaGTCAAca
AtSK21	P$WRKY21_02	WRKY21	32	42	1	1	0.995	taaGTCAAca
AtSK21	P$WRKY48_02	WRKY48	32	42	1	1	0.997	taaGTCAAca
AtSK21	P$WRKY57_01	WRKY57	32	42	1	1	0.996	taaGTCAAca
AtSK21	P$WRKY60_01	WRKY60	32	43	1	1	0.92	taaGTCAAcat
AtSK21	P$WRKY15_01	WRKY15	33	43	1	1	0.992	aaGTCAAcat
AtSK21	P$WRKY2_01	WRKY2	33	41	1	1	0.948	aaGTCAAc
AtSK21	P$WRKY25_02	WRKY25	33	41	1	1	0.933	aaGTCAAc
AtSK21	P$WRKY40_01	WRKY40	33	41	1	1	0.996	aaGTCAAc
AtSK21	P$WRKY43_02	WRKY43	33	43	1	1	1	aaGTCAAcat
AtSK21	P$WRKY62_01	WRKY62	33	41	1	1	0.918	aaGTCAAc
AtSK21	P$WRKY63_01	WRKY63	33	41	1	1	0.914	aaGTCAAc
AtSK21	P$WRKY75_01	WRKY75	33	41	1	1	1	aaGTCAAc
AtSK21	P$WRKY8_01	WRKY8	33	42	1	1	0.998	aaGTCAAca
AtSK21	P$WRKY23_01	WRKY23	34	42	1	1	0.961	aGTCAAca
AtSK21	P$WRKY30_01	WRKY30	34	44	1	1	0.99	aGTCAAcata
AtSK21	P$RAV1_01	RAV1	34	46	1	1	0.945	agtCAACAtacg
AtSK21	P$WRKY18_Q2	WRKY18	35	44	1	1	0.962	GTCAAcata
AtSK21	P$NAC043_01	NAC043	40	50	1	1	0.89	catACGTAat
AtSK21	P$NAC025_01	NAC025	42	50	1	1	0.982	tACGTAat
AtSK21	P$NAC6_01	NAC6	43	49	1	1	1	aCGTAA
AtSK21	P$DREB1A_04	DREB1A	52	62	1	1	0.965	tgGTCGGtca
AtSK21	P$ERF039_01	ERF039	52	62	1	1	0.988	tgGTCGGtca
AtSK21	P$PHYPA182268_05	HYPA182268	52	62	1	1	0.941	tgGTCGGtca
AtSK21	P$PHYPA64121_06	HYPA64121	52	65	1	1	0.86	tggTCGGTcaaga
AtSK21	P$ERF043_01	ERF043	53	61	1	1	0.865	gGTCGGtc
AtSK21	P$PHYPA173530_04	HYPA173530	53	61	1	1	0.931	gGTCGGtc

AtSK21	P$PHYPA28324_10	HYPA28324	53	61	1	1	0.939	gGTCGGtc
AtSK21	P$WRKY18_02	WRKY18	55	65	1	1	0.997	tcgGTCAAga
AtSK21	P$WRKY21_02	WRKY21	55	65	1	1	0.958	tcgGTCAAga
AtSK21	P$WRKY48_02	WRKY48	55	65	1	1	0.996	tcgGTCAAga
AtSK21	P$WRKY57_01	WRKY57	55	65	1	1	0.964	tcgGTCAAga
AtSK21	P$WRKY60_01	WRKY60	55	66	1	1	0.969	tcgGTCAAgag
AtSK21	P$WRKY15_01	WRKY15	56	66	1	1	0.977	cgGTCAAgag
AtSK21	P$WRKY2_01	WRKY2	56	64	1	1	0.983	cgGTCAAg
AtSK21	P$WRKY25_02	WRKY25	56	64	1	1	0.974	cgGTCAAg
AtSK21	P$WRKY40_01	WRKY40	56	64	1	1	1	cgGTCAAg
AtSK21	P$WRKY43_02	WRKY43	56	66	1	1	0.972	cgGTCAAgag
AtSK21	P$WRKY62_01	WRKY62	56	64	1	1	0.884	cgGTCAAg
AtSK21	P$WRKY63_01	WRKY63	56	64	1	1	0.993	cgGTCAAg
AtSK21	P$WRKY75_01	WRKY75	56	64	1	1	0.959	cgGTCAAg
AtSK21	P$WRKY8_01	WRKY8	56	65	1	1	0.987	cgGTCAAga
AtSK21	P$WRKY30_01	WRKY30	57	67	1	1	0.914	gGTCAAgaga
AtSK21	P$WRKY18_Q2	WRKY18	58	67	1	1	0.965	GTCAAgaga
AtSK21	P$HSFA4A_01	HSFA4A	107	113	1	1	0.914	tCTATT
AtSK21	P$WRKY18_02	WRKY18	110	120	1	1	0.944	attGTCAAtt
AtSK21	P$WRKY21_02	WRKY21	110	120	1	1	0.95	attGTCAAtt
AtSK21	P$WRKY48_02	WRKY48	110	120	1	1	0.986	attGTCAAtt
AtSK21	P$WRKY57_01	WRKY57	110	120	1	1	0.96	attGTCAAtt
AtSK21	P$WRKY60_01	WRKY60	110	121	1	1	0.889	attGTCAAttt
AtSK21	P$WRKY15_01	WRKY15	111	121	1	1	0.958	ttGTCAAttt
AtSK21	P$WRKY2_01	WRKY2	111	119	1	1	0.904	ttGTCAAt
AtSK21	P$WRKY25_02	WRKY25	111	119	1	1	0.888	ttGTCAAt
AtSK21	P$WRKY40_01	WRKY40	111	119	1	1	0.977	ttGTCAAt
AtSK21	P$WRKY43_02	WRKY43	111	121	1	1	0.95	ttGTCAAttt
AtSK21	P$WRKY62_01	WRKY62	111	119	1	1	0.87	ttGTCAAt
AtSK21	P$WRKY63_01	WRKY63	111	119	1	1	0.886	ttGTCAAt
AtSK21	P$WRKY75_01	WRKY75	111	119	1	1	0.927	ttGTCAAt
AtSK21	P$WRKY8_01	WRKY8	111	120	1	1	0.977	ttGTCAAtt
AtSK21	P$WRKY30_01	WRKY30	112	122	1	1	0.904	tGTCAAttta
AtSK21	P$WRKY18_Q2	WRKY18	113	122	1	1	0.98	GTCAAttta
AtSK21	P$HAHB4_01	HAHB4	128	137	1	1	0.94	tAATGAtgt
AtSK21	P$TEIL_01	TEIL	133	141	1	1	0.879	ATGTAaat
AtSK21	P$GT1_Q6	GT1	135	142	1	1	1	GTAAAta
AtSK21	P$ATHB6_01	ATHB6	136	145	1	1	0.976	taAATAAta
AtSK21	P$ATHB5_04	ATHB5	136	147	1	1	0.887	taAATAAtatt
AtSK21	P$ATHB1_03	ATHB1	136	147	1	1	0.89	taAATAAtatt
AtSK21	P$ATHB16_01	ATHB16	137	145	1	1	0.867	aAATAAta
AtSK21	P$ARR18_01	ARR18	145	158	1	1	0.895	ttttAGATAgatt
AtSK21	P$SBF1_01	SBF1	155	169	1	1	0.878	atttgtTTAATtat
AtSK21	P$EDT1_01	EDT1	158	168	1	1	0.855	tgtTTAATta
AtSK21	P$MADSA_Q2	MADSA	167	183	1	1	0.874	atatctAAAATgaaac
AtSK21	P$SBF1_01	SBF1	184	198	1	1	0.925	tactaaTTAAAatg
AtSK21	P$GT1_Q6	GT1	246	253	1	1	0.971	GTAAAaa
AtSK21	P$HSFA4A_01	HSFA4A	267	273	1	1	0.964	cCTATT
AtSK21	P$ATHB6_01	ATHB6	272	281	1	1	0.897	tgAATAAca
AtSK21	P$ATMYB15_Q2	ATMYB15	276	282	1	1	1	TAACAa
AtSK21	P$GT1_Q6_01	GT1	283	295	1	1	0.867	TTTTTctatagt
AtSK21	P$KNOX3_01	KNOX3	302	314	1	1	0.977	cgatTGACAgtt
AtSK21	P$SIZF2_01	SIZF2	303	313	1	1	0.907	gatTGACAgt
AtSK21	P$ATMYB77_01	ATMYB77	303	316	1	1	0.939	gattgaCAGTTtg
AtSK21	P$WRKY11_Q2	WRKY11	304	312	1	1	0.899	aTTGACag
AtSK21	P$ATH1_01	ATH1	306	314	1	1	0.987	TGACAgtt
AtSK21	P$AT5G04240_01	AT5G04240	314	320	1	1	0.938	tGGCAC
AtSK21	P$SBF1_01	SBF1	324	338	1	1	0.917	attaagTTAATaat
AtSK21	P$EDT1_01	EDT1	327	337	1	1	0.853	aagTTAATaa
AtSK21	P$ATHB6_01	ATHB6	330	339	1	1	0.984	ttAATAAtt
AtSK21	P$ATHB5_04	ATHB5	330	341	1	1	0.972	ttAATAAtttc
AtSK21	P$ATHB1_03	ATHB1	330	341	1	1	0.97	ttAATAAtttc
AtSK21	P$ATHB16_01	ATHB16	331	339	1	1	1	tAATAAtt
AtSK21	P$RIN_Q2	RIN	345	356	1	1	0.859	atatTTTAAgt
AtSK21	P$ATHB6_01	ATHB6	374	383	1	1	0.914	ctAATAAat
AtSK21	P$PDF2_01	DF2	374	385	1	1	0.888	ctaaTAAATtc
AtSK21	P$ABF2_01	ABF2	379	392	1	1	0.902	aaattCACGTaga
AtSK21	P$O2_Q4	O2	380	391	1	1	0.896	aattCACGTag
AtSK21	P$BZR1_02	BZR1	380	394	1	1	0.851	aattCACGTagatc
AtSK21	P$GBP_Q6	GBP	381	393	1	1	0.895	attCACGTagat
AtSK21	P$ABI5_01	ABI5	381	391	1	1	0.9	attCACGTag
AtSK21	P$ABF4_01	ABF4	381	393	1	1	0.893	attCACGTagat
AtSK21	P$CPRF3_Q2	CPRF3	382	392	1	1	0.926	ttCACGTaga
AtSK21	P$CPRF2_Q2	CPRF2	382	392	1	1	0.949	ttCACGTaga
AtSK21	P$O2_02	O2	382	392	1	1	0.973	ttCACGTaga
AtSK21	P$TGA1B_Q2	TGA1B	382	392	1	1	0.881	ttCACGTaga
AtSK21	P$TGA1A_Q2	TGA1A	382	392	1	1	0.962	ttCACGTaga
AtSK21	P$CPRF3_01	CPRF3	382	392	1	1	0.932	ttCACGTaga

AtSK21	P$CPRF2_01	CPRF2	382	392	1	1	0.95	ttCACGTaga
AtSK21	P$BEE2_01	BEE2	382	392	1	1	0.915	ttCACGTaga
AtSK21	P$BIM2_01	BIM2	382	392	1	1	0.854	ttCACGTaga
AtSK21	P$BIM3_01	BIM3	382	392	1	1	0.888	ttCACGTaga
AtSK21	P$PHYPA143875_02	HYPA143875	382	392	1	1	0.875	ttCACGTaga
AtSK21	P$SPT_01	SPT	382	391	1	1	0.918	ttCACGTag
AtSK21	P$NAC043_01	NAC043	382	392	1	1	0.884	ttcACGTAga
AtSK21	P$RITA1_01	RITA1	383	390	1	1	0.99	tCACGTa
AtSK21	P$OCSBF1_01	OCSBF1	384	389	1	1	1	CACGT
AtSK21	P$GATA9_01	GATA9	386	397	1	1	0.891	cgtAGATCgta
AtSK21	P$AGP1_01	AGP1	387	397	1	1	0.853	gtAGATCgta
AtSK21	P$GATA10_01	GATA10	388	396	1	1	0.876	tAGATCgt
AtSK21	P$ATSPL3_01	ATSPL3	388	404	1	1	0.951	tagatCGTACgcacac
AtSK21	P$ARR10_01	ARR10	389	396	1	1	0.913	AGATCgt
AtSK21	P$SMZ_01	SMZ	390	398	1	1	0.85	gaTCGTAc
AtSK21	P$SPL11_01	SPL11	390	402	1	1	0.927	gatcGTACGcac
AtSK21	P$BHLH28_01	BHLH28	390	402	1	1	0.857	gatcGTACGcac
AtSK21	P$SPL14_03	SPL14	391	402	1	1	0.854	atCGTACgcac
AtSK21	P$SPL5_01	SPL5	392	401	1	1	0.964	tcGTACGca
AtSK21	P$POPTR_01	OPTR	393	400	1	1	0.928	cGTACGc
AtSK21	P$SPL12_01	SPL12	393	401	1	1	0.97	cGTACGca
AtSK21	P$SPL4_01	SPL4	393	402	1	1	0.989	cGTACGcac
AtSK21	P$GT1_Q6_01	GT1	406	418	1	1	0.863	TTTGTttacatg
AtSK21	P$LEC2_01	LEC2	412	423	1	1	0.936	taCATGCttta
AtSK21	P$CBNAC_01	CBNAC	415	421	1	1	0.968	aTGCTT
AtSK21	P$CBNAC_02	CBNAC	415	431	1	1	0.863	aTGCTTtaacgtgttg
AtSK21	P$ABZ1_01	ABZ1	418	432	1	1	0.898	ctttaACGTGttgc
AtSK21	P$HBP1A_Q2	HBP1A	420	430	1	1	0.851	ttaACGTGtt
AtSK21	P$TAF1_Q2	TAF1	420	430	1	1	0.918	ttaACGTGtt
AtSK21	P$EMBP1_02	EMBP1	420	430	1	1	0.872	ttaACGTGtt
AtSK21	P$TAF1_01	TAF1	420	430	1	1	0.945	ttaACGTGtt
AtSK21	P$TRAB1_Q2	TRAB1	421	432	1	1	0.905	taACGTGttgc
AtSK21	P$GBF1_01	GBF1	421	429	1	1	0.978	taACGTGt
AtSK21	P$BIM1_02	BIM1	421	431	1	1	0.947	taACGTGttg
AtSK21	P$ABF4_02	ABF4	421	431	1	1	0.99	taACGTGttg
AtSK21	P$ABI5_Q2	ABI5	423	429	1	1	0.979	ACGTGt
AtSK21	P$O2_Q4	O2	427	438	1	1	0.909	gttgCATGTta
AtSK21	P$ABI3_01	ABI3	428	437	1	1	0.87	ttGCATGtt
AtSK21	P$UIF1_01	UIF1	456	466	1	1	0.994	caaGATTCgt
AtSK21	P$BZR1_01	BZR1	463	469	1	1	0.897	CGTGCc
AtSK21	P$P_01		466	475	1	1	0.951	gcCTACCtt
AtSK21	P$AP3_01	AP3	476	491	1	1	0.86	gaaCAAAAgaggcaa
AtSK21	P$PBF_01	BF	477	488	1	1	0.988	aacAAAAGagg
AtSK21	P$DOF_Q2	DOF	477	488	1	1	0.932	aacAAAAGagg
AtSK21	P$CDF2_01	CDF2	478	488	1	1	0.949	acAAAAGagg
AtSK21	P$CDF3_01	CDF3	479	488	1	1	0.977	cAAAAGagg
AtSK21	P$AP3_01	AP3	497	512	1	1	0.865	agaCAAAAgaggcaa
AtSK21	P$PBF_01	BF	498	509	1	1	0.983	gacAAAAGagg
AtSK21	P$DOF_Q2	DOF	498	509	1	1	0.928	gacAAAAGagg
AtSK21	P$CDF2_01	CDF2	499	509	1	1	0.949	acAAAAGagg
AtSK21	P$CDF3_01	CDF3	500	509	1	1	0.977	cAAAAGagg
AtSK21	P$ATHB7_01	ATHB7	516	526	1	1	0.924	ctAATCAtgg
AtSK21	P$HAT1_01	HAT1	516	526	1	1	0.988	ctAATCAtgg
AtSK21	P$ATHB4_02	ATHB4	517	527	1	1	0.882	taATCATggc
AtSK21	P$HSFA2_01	HSFA2	527	533	1	1	0.922	CCAAAt
AtSK21	P$AT3G60580_01	AT3G60580	546	553	1	1	0.902	ggATCCC
AtSK21	P$SPF1_Q2	SPF1	558	568	1	1	0.979	aaATAGTact
AtSK21	P$CBF1_01	CBF1	578	588	1	1	0.941	tTGCCGccag
AtSK21	P$ERF019_01	ERF019	578	588	1	1	0.906	tTGCCGccag
AtSK21	P$DREB6_01	DREB6	578	588	1	1	0.901	tTGCCGccag
AtSK21	P$JERF3_01	JERF3	578	588	1	1	0.927	tTGCCGccag
AtSK21	P$DREB1_01	DREB1	578	588	1	1	0.9	tTGCCGccag
AtSK21	P$CEF1_01	CEF1	578	588	1	1	0.927	tTGCCGccag
AtSK21	P$JERF1_01	JERF1	578	588	1	1	0.958	tTGCCGccag
AtSK21	P$CBF1_03	CBF1	578	588	1	1	0.93	tTGCCGccag
AtSK21	P$AT1G71450_01	AT1G71450	578	588	1	1	0.878	tTGCCGccag
AtSK21	P$DREB1F_01	DREB1F	578	588	1	1	0.948	tTGCCGccag
AtSK21	P$AT1G33760_01	AT1G33760	578	588	1	1	0.907	tTGCCGccag
AtSK21	P$AT1G71520_01	AT1G71520	578	588	1	1	0.865	tTGCCGccag
AtSK21	P$AT4G28140_01	AT4G28140	578	588	1	1	0.899	tTGCCGccag
AtSK21	P$DREB1E_02	DREB1E	578	588	1	1	0.92	tTGCCGccag
AtSK21	P$ORA47_01	ORA47	578	588	1	1	0.878	tTGCCGccag
AtSK21	P$DREB2F_01	DREB2F	578	588	1	1	0.894	tTGCCGccag
AtSK21	P$ERF13_01	ERF13	578	588	1	1	0.862	ttGCCGCcag
AtSK21	P$ABI4_03	ABI4	578	588	1	1	0.905	ttGCCGCcag
AtSK21	P$WRAF1_01	WRAF1	578	588	1	1	0.923	ttGCCGCcag
AtSK21	P$WRAF2_01	WRAF2	578	588	1	1	0.894	ttGCCGCcag
AtSK21	P$PTI5_01	TI5	578	588	1	1	0.993	ttGCCGCcag

AtSK21	P$DREBI5_01	DREBI5	578	588	1	1	0.906	ttGCCGCcag
AtSK21	P$AT2G47520_01	AT2G47520	578	588	1	1	0.951	ttGCCGCcag
AtSK21	P$DREB2B_01	DREB2B	578	588	1	1	0.901	ttGCCGCcag
AtSK21	P$CRF1_02	CRF1	578	588	1	1	0.911	ttGCCGCcag
AtSK21	P$OPBP1_01	OPBP1	578	588	1	1	0.944	ttGCCGCcag
AtSK21	P$ATERF14_01	ATERF14	578	588	1	1	0.936	ttGCCGCcag
AtSK21	P$ERF112_01	ERF112	578	588	1	1	0.906	ttGCCGCcag
AtSK21	P$DREB2A_02	DREB2A	578	588	1	1	0.914	ttGCCGCcag
AtSK21	P$ERF1_02	ERF1	578	588	1	1	0.94	ttGCCGCcag
AtSK21	P$ERF4_02	ERF4	578	588	1	1	0.941	ttGCCGCcag
AtSK21	P$AT5G25390_01	AT5G25390	578	588	1	1	0.903	ttGCCGCcag
AtSK21	P$EREBP1_01	EREBP1	578	588	1	1	0.937	ttGCCGCcag
AtSK21	P$ERF110_02	ERF110	578	588	1	1	0.874	ttGCCGCcag
AtSK21	P$CBF3_01	CBF3	578	588	1	1	0.938	ttGCCGCcag
AtSK21	P$DREBII1_01	DREBII1	578	588	1	1	0.911	ttGCCGCcag
AtSK21	P$TSRF1_01	TSRF1	578	588	1	1	0.94	ttGCCGCcag
AtSK21	P$DRF13_01	DRF13	578	588	1	1	0.943	ttGCCGCcag
AtSK21	P$ERF2_03	ERF2	578	588	1	1	0.938	ttGCCGCcag
AtSK21	P$ERF1B_03	ERF1B	578	588	1	1	0.929	ttGCCGCcag
AtSK21	P$RAP26_02	RAP26	578	588	1	1	0.932	ttGCCGCcag
AtSK21	P$CBF5_01	CBF5	578	588	1	1	0.901	ttGCCGCcag
AtSK21	P$CBF16_01	CBF16	578	588	1	1	0.91	ttGCCGCcag
AtSK21	P$CBF17_01	CBF17	578	588	1	1	0.905	ttGCCGCcag
AtSK21	P$ERF1_05	ERF1	578	588	1	1	0.891	ttGCCGCcag
AtSK21	P$AT5G25190_01	AT5G25190	578	588	1	1	0.902	ttGCCGCcag
AtSK21	P$ERF105_01	ERF105	578	588	1	1	0.92	ttGCCGCcag
AtSK21	P$CBF_01	CBF	578	588	1	1	0.907	ttGCCGCcag
AtSK21	P$AT5G11190_01	AT5G11190	578	588	1	1	0.905	ttGCCGCcag
AtSK21	P$AT1G68550_01	AT1G68550	578	588	1	1	0.988	ttGCCGCcag
AtSK21	P$AT1G77640_01	AT1G77640	578	588	1	1	0.904	ttGCCGCcag
AtSK21	P$ERF016_01	ERF016	578	588	1	1	0.909	ttGCCGCcag
AtSK21	P$AT3G61630_01	AT3G61630	578	588	1	1	0.967	ttGCCGCcag
AtSK21	P$AT5G43410_01	AT5G43410	578	588	1	1	0.946	ttGCCGCcag
AtSK21	P$RAP26L_02	RAP26L	578	588	1	1	0.982	ttGCCGCcag
AtSK21	P$AT5G07310_01	AT5G07310	578	588	1	1	0.955	ttGCCGCcag
AtSK21	P$DREB1A_03	DREB1A	578	588	1	1	0.937	ttGCCGCcag
AtSK21	P$AT1G49120_01	AT1G49120	578	588	1	1	0.875	ttGCCGCcag
AtSK21	P$DREB2D_01	DREB2D	578	588	1	1	0.925	ttGCCGCcag
AtSK21	P$AT3G25890_01	AT3G25890	578	588	1	1	0.973	ttGCCGCcag
AtSK21	P$AT4G23750_01	AT4G23750	578	588	1	1	0.888	ttGCCGCcag
AtSK21	P$AT4G27950_01	AT4G27950	578	588	1	1	0.911	ttGCCGCcag
AtSK21	P$RRTF1_01	RRTF1	578	588	1	1	0.963	ttGCCGCcag
AtSK21	P$CEJ1_02	CEJ1	578	588	1	1	0.871	ttGCCGCcag
AtSK21	P$ERF1_Q2_01	ERF1	578	592	1	1	0.869	ttGCCGCcagcttt
AtSK21	P$CRF2_01	CRF2	578	586	1	1	0.921	ttGCCGCc
AtSK21	P$ERF096_01	ERF096	578	588	1	1	0.985	ttGCCGCcag
AtSK21	P$ERF098_01	ERF098	578	586	1	1	0.996	ttGCCGCc
AtSK21	P$DREB2C_01	DREB2C	578	588	1	1	0.855	ttgCCGCCag
AtSK21	P$CBF1_02	CBF1	578	588	1	1	0.904	ttgCCGCCag
AtSK21	P$ERF1B_06	ERF1B	579	587	1	1	0.961	tGCCGCca
AtSK21	P$ERF7_02	ERF7	579	589	1	1	0.988	tGCCGCcagc
AtSK21	P$ERF094_01	ERF094	579	587	1	1	0.974	tGCCGCca
AtSK21	P$ERF2_01	ERF2	579	586	1	1	0.929	tgCCGCC
AtSK21	P$ERF13_02	ERF13	579	587	1	1	0.982	tgCCGCCa
AtSK21	P$AT3G63350_01	AT3G63350	581	587	1	1	0.882	CCGCCa
AtSK21	P$ID1_01	ID1	590	601	1	1	0.902	tTTGTCgctac
AtSK21	P$ARF8_01	ARF8	590	599	1	1	0.96	ttTGTCGct
AtSK21	P$ARR2_01	ARR2	606	616	1	1	0.885	aacaATCTTt
AtSK21	P$AT2G38090_01	AT2G38090	611	623	1	1	0.993	tctTTCGTattt
AtSK21	P$SBF1_01	SBF1	615	629	1	1	0.862	tcgtatTTAAAgac
AtSK21	P$DOF1_01	DOF1	619	630	1	1	0.986	attTAAAGact
AtSK21	P$ID1_01	ID1	633	644	1	1	0.919	tTTGTCttcct
AtSK21	P$AT1G77950_01	AT1G77950	650	661	1	1	0.853	tttagTTTAAt
AtSK21	P$SBF1_01	SBF1	650	664	1	1	0.902	tttagtTTAATatg
AtSK21	P$GT1_Q6	GT1	680	687	1	1	1	GTAAAta
AtSK21	P$ATHB6_01	ATHB6	681	690	1	1	0.903	taAATAAac
AtSK21	P$CBF3_02	CBF3	695	709	1	1	0.977	agttgCCGACgttt
AtSK21	P$CBF1_04	CBF1	696	708	1	1	0.972	gttgCCGACgtt
AtSK21	P$CBF1_01	CBF1	697	707	1	1	0.939	tTGCCGacgt
AtSK21	P$DREB6_01	DREB6	697	707	1	1	0.854	tTGCCGacgt
AtSK21	P$JERF3_01	JERF3	697	707	1	1	0.903	tTGCCGacgt
AtSK21	P$DREB1_01	DREB1	697	707	1	1	0.854	tTGCCGacgt
AtSK21	P$CEF1_01	CEF1	697	707	1	1	0.905	tTGCCGacgt
AtSK21	P$JERF1_01	JERF1	697	707	1	1	0.935	tTGCCGacgt
AtSK21	P$CBF1_03	CBF1	697	707	1	1	0.942	tTGCCGacgt
AtSK21	P$DREB1F_01	DREB1F	697	707	1	1	0.95	tTGCCGacgt
AtSK21	P$AT1G33760_01	AT1G33760	697	707	1	1	0.866	tTGCCGacgt
AtSK21	P$ERF5_02	ERF5	697	707	1	1	0.902	ttGCCGAcgt

AtSK21	P$ERF1_04	ERF1	697	707	1	1	0.902	ttGCCGAcgt
AtSK21	P$DREB1G_02	DREB1G	697	707	1	1	0.914	ttgCCGACgt
AtSK21	P$ARF1_01	ARF1	699	707	1	1	0.855	gCCGACgt
AtSK21	P$ARF5_01	ARF5	699	707	1	1	0.896	gCCGACgt
AtSK21	P$DREB1B_01	DREB1B	700	705	1	1	1	CCGAC
AtSK21	P$AT3G63350_01	AT3G63350	731	737	1	1	0.882	CCGCCa
AtSK21	P$ARR1_01	ARR1	735	745	1	1	0.957	cacGAATCcg
AtSK21	P$O2_Q4	O2	748	759	1	1	0.892	ttctCATGTta
AtSK21	P$BPC1_Q2	BPC1	758	764	1	1	0.99	AGAAAc
AtSK21	P$ABF2_01	ABF2	764	777	1	1	0.895	ctatgCACGTccg
AtSK21	P$O2_Q4	O2	765	776	1	1	0.926	tatgCACGTcc
AtSK21	P$HBI1_01	HBI1	765	777	1	1	0.87	tatgCACGTccg
AtSK21	P$GBP_Q6	GBP	766	778	1	1	0.915	atgCACGTccga
AtSK21	P$ABI5_01	ABI5	766	776	1	1	0.888	atgCACGTcc
AtSK21	P$ABF4_01	ABF4	766	778	1	1	0.871	atgCACGTccga
AtSK21	P$CPRF3_Q2	CPRF3	767	777	1	1	0.938	tgCACGTccg
AtSK21	P$CPRF2_Q2	CPRF2	767	777	1	1	0.941	tgCACGTccg
AtSK21	P$O2_02	O2	767	777	1	1	0.933	tgCACGTccg
AtSK21	P$TGA1B_Q2	TGA1B	767	777	1	1	0.894	tgCACGTccg
AtSK21	P$TGA1A_Q2	TGA1A	767	777	1	1	0.976	tgCACGTccg
AtSK21	P$CPRF3_01	CPRF3	767	777	1	1	0.951	tgCACGTccg
AtSK21	P$CPRF2_01	CPRF2	767	777	1	1	0.943	tgCACGTccg
AtSK21	P$TGA1B_01	TGA1B	767	777	1	1	0.855	tgCACGTccg
AtSK21	P$BEE2_01	BEE2	767	777	1	1	0.908	tgCACGTccg
AtSK21	P$BIM3_01	BIM3	767	777	1	1	0.883	tgCACGTccg
AtSK21	P$PHYPA143875_02	HYPA143875	767	777	1	1	0.875	tgCACGTccg
AtSK21	P$SPT_01	SPT	767	776	1	1	0.927	tgCACGTcc
AtSK21	P$GBF1F_Q2	GBF1F	767	778	1	1	0.862	tgCACGTccga
AtSK21	P$TSAR1_01	TSAR1	767	777	1	1	0.894	tgCACGTccg
AtSK21	P$BHLH78_01	BHLH78	768	776	1	1	0.875	GCACGtcc
AtSK21	P$RITA1_01	RITA1	768	775	1	1	0.954	gCACGTc
AtSK21	P$BHLH3_01	BHLH3	768	776	1	1	0.851	gCACGTcc
AtSK21	P$AIB_01	AIB	768	776	1	1	0.869	gCACGTcc
AtSK21	P$OCSBF1_01	OCSBF1	769	774	1	1	1	CACGT
AtSK21	P$TGA1A_01	TGA1A	769	776	1	1	0.861	cACGTCc
AtSK21	P$PBF_01	BF	798	809	1	1	0.99	ttcAAAAGaga
AtSK21	P$DOF_Q2	DOF	798	809	1	1	0.925	ttcAAAAGaga
AtSK21	P$CDF2_01	CDF2	799	809	1	1	0.948	tcAAAAGaga
AtSK21	P$CDF3_01	CDF3	800	809	1	1	0.978	cAAAAGaga
AtSK21	P$ARR18_01	ARR18	802	815	1	1	0.91	aaagAGATAtata
AtSK21	P$PEND_02	END	828	838	1	1	0.854	gtTTCTTttg
AtSK21	P$C1_Q2	C1	837	848	1	1	0.931	gcAACTAtaaa
AtSK21	P$PDF2_01	DF2	840	851	1	1	0.906	actaTAAATgg
AtSK21	P$SED_Q2	SED	848	858	1	1	0.881	tgggCCTTTg
AtSK21	P$PBF_Q2_01	BF	852	858	1	1	0.988	CCTTTg
AtSK21	P$ATHB6_01	ATHB6	856	865	1	1	0.905	tgAATAAga
AtSK21	P$PEND_01	END	860	868	1	1	0.875	tAAGAAca
AtSK21	P$ATHB1_01	ATHB1	875	889	1	1	0.859	ttgatATTATttca
AtSK21	P$O2_Q4	O2	883	894	1	1	0.951	atttCATGTcc
AtSK21	P$HSFA2_01	HSFA2	892	898	1	1	0.933	CCAAAg
AtSK21	P$SBF1_01	SBF1	897	911	1	1	0.865	gttttaTTAAActc
AtSK21	P$HMG1_01	HMG1	913	922	1	1	0.873	GTTGTcagc
AtSK21	P$C1_Q2	C1	931	942	1	1	0.956	ttAACTAacaa
AtSK21	P$WEREWOLF_Q2_01	WEREWOLF	933	942	1	1	0.991	aACTAAcaa
AtSK21	P$MYBAS1_01	MYBAS1	933	944	1	1	0.948	aaCTAACaaaa
AtSK21	P$FLC_01	FLC	933	954	1	1	0.926	aactaacaaaaaaAGAAAccg
AtSK21	P$ATMYB15_Q2	ATMYB15	936	942	1	1	1	TAACAa
AtSK21	P$AP3_01	AP3	936	951	1	1	0.876	taaCAAAAaaagaaa
AtSK21	P$AP1_01	AP1	938	951	1	1	0.98	acAAAAAaagaaa
AtSK21	P$PBF_01	BF	940	951	1	1	0.979	aaaAAAAGaaa
AtSK21	P$DOF_Q2	DOF	940	951	1	1	1	aaaAAAAGaaa
AtSK21	P$CDF2_01	CDF2	941	951	1	1	0.978	aaAAAAGaaa
AtSK21	P$CDF3_01	CDF3	942	951	1	1	0.975	aAAAAGaaa
AtSK21	P$BPC1_Q2	BPC1	946	952	1	1	0.99	AGAAAc
AtSK21	P$AT4G12750_01	AT4G12750	947	957	1	1	0.913	gaaACCGAgg
AtSK21	P$RAV2_01	RAV2	948	957	1	1	0.9	aaACCGAgg
AtSK21	P$AT3G51080_01	AT3G51080	955	962	1	1	0.89	GGAAAac
AtSK21	P$C1_Q2	C1	961	972	1	1	0.952	cgAACTAaaga
AtSK21	P$DOF1_01	DOF1	963	974	1	1	0.994	aacTAAAGaat
AtSK21	P$ARR1_01	ARR1	967	977	1	1	0.954	aaaGAATCat
AtSK21	P$ATHB7_01	ATHB7	969	979	1	1	0.921	agAATCAttt
AtSK21	P$HAT1_01	HAT1	969	979	1	1	0.98	agAATCAttt
AtSK21	P$ATHB4_02	ATHB4	970	980	1	1	0.923	gaATCATtta
AtSK21	P$ARR2_01	ARR2	975	985	1	1	0.858	atttATCTTa
AtSK21	P$WRKY18_02	WRKY18	988	998	1	1	0.945	tttGTCAAaa
AtSK21	P$WRKY21_02	WRKY21	988	998	1	1	0.955	tttGTCAAaa
AtSK21	P$WRKY48_02	WRKY48	988	998	1	1	0.987	tttGTCAAaa
AtSK21	P$WRKY57_01	WRKY57	988	998	1	1	0.962	tttGTCAAaa

AtSK21	P$WRKY60_01	WRKY60	988	999	1	1	0.898	tttGTCAAaaa
AtSK21	P$WRKY15_01	WRKY15	989	999	1	1	0.964	ttGTCAAaaa
AtSK21	P$WRKY2_01	WRKY2	989	997	1	1	0.909	ttGTCAAa
AtSK21	P$WRKY25_02	WRKY25	989	997	1	1	0.892	ttGTCAAa
AtSK21	P$WRKY40_01	WRKY40	989	997	1	1	0.977	ttGTCAAa
AtSK21	P$WRKY43_02	WRKY43	989	999	1	1	0.955	ttGTCAAaaa
AtSK21	P$WRKY62_01	WRKY62	989	997	1	1	0.895	ttGTCAAa
AtSK21	P$WRKY63_01	WRKY63	989	997	1	1	0.886	ttGTCAAa
AtSK21	P$WRKY75_01	WRKY75	989	997	1	1	0.936	ttGTCAAa
AtSK21	P$WRKY8_01	WRKY8	989	998	1	1	0.98	ttGTCAAaa
AtSK21	P$WRKY30_01	WRKY30	990	1000	1	1	0.898	tGTCAAaaag
AtSK21	P$WRKY18_Q2	WRKY18	991	1000	1	1	0.938	GTCAAaaag
AtSK21	P$PBF_01	BF	992	1003	1	1	0.974	tcaAAAAGagg
AtSK21	P$DOF_Q2	DOF	992	1003	1	1	0.982	tcaAAAAGagg
AtSK21	P$CDF2_01	CDF2	993	1003	1	1	0.978	caAAAAGagg
AtSK21	P$CDF3_01	CDF3	994	1003	1	1	0.983	aAAAAGagg
AtSK21	P$AT1G14580_01	AT1G14580	996	1007	1	1	0.859	aaagaGGGATa
AtSK21	P$AT5G66730_01	AT5G66730	996	1007	1	1	0.901	aaagaGGGATa
AtSK21	P$ARR18_01	ARR18	1015	1028	1	1	0.897	ctttAGATAgaat
AtSK21	P$PDF2_01	DF2	1024	1035	1	1	0.865	gaatTAAATat
AtSK21	P$SPL14_01	SPL14	1037	1044	1	1	0.851	CCGTAta
AtSK21	P$SBF1_01	SBF1	1061	1075	1	1	0.898	atatggTTAAAcaa
AtSK21	P$WRKY48_01	WRKY48	1066	1075	1	1	0.876	gttaAACAA
AtSK21	P$AT3G20750_01	AT3G20750	1067	1075	1	1	0.876	tTAAACaa
AtSK21	P$ARR1_01	ARR1	1079	1089	1	1	0.987	ccaGAATCta
AtSK21	P$MYBAS1_01	MYBAS1	1084	1095	1	1	0.974	atCTAACtaac
AtSK21	P$C1_Q2	C1	1086	1097	1	1	0.972	ctAACTAacaa
AtSK21	P$WEREWOLF_Q2_01	WEREWOLF	1088	1097	1	1	0.991	aACTAAcaa
AtSK21	P$MYBAS1_01	MYBAS1	1088	1099	1	1	0.975	aaCTAACaaac
AtSK21	P$ATMYB15_Q2	ATMYB15	1091	1097	1	1	1	TAACAa
AtSK21	P$MYBAS1_01	MYBAS1	1108	1119	1	1	0.988	tcCTAACtcct
AtSK21	P$AT3G18650_01	AT3G18650	1124	1135	1	1	0.856	tagacTTGTAa
AtSK21	P$ATSPL3_01	ATSPL3	1130	1146	1	1	0.964	tgtaaCGTACagagaa
AtSK21	P$NAC043_01	NAC043	1131	1141	1	1	0.916	gtaACGTAca
AtSK21	P$SPL14_03	SPL14	1133	1144	1	1	0.861	aaCGTACagag
AtSK21	P$AT3G18650_01	AT3G18650	1143	1154	1	1	0.943	gaagaTTGTAt
AtSK21	P$PBF_01	BF	1173	1184	1	1	0.978	tatAAAAGccc
AtSK21	P$DOF_Q2	DOF	1173	1184	1	1	0.928	tatAAAAGccc
AtSK21	P$DOF2_01	DOF2	1173	1184	1	1	0.992	tataAAAGCcc
AtSK21	P$DOF3_01	DOF3	1173	1184	1	1	0.99	tataAAAGCcc
AtSK21	P$CDF2_01	CDF2	1174	1184	1	1	0.956	atAAAAGccc
AtSK21	P$CDF3_01	CDF3	1175	1184	1	1	0.975	tAAAAGccc
AtSK21	P$PBF_01	BF	1221	1232	1	1	0.954	gctAAAAGttc
AtSK21	P$DOF_Q2	DOF	1221	1232	1	1	0.932	gctAAAAGttc
AtSK21	P$CDF2_01	CDF2	1222	1232	1	1	0.968	ctAAAAGttc
AtSK21	P$CDF3_01	CDF3	1223	1232	1	1	0.985	tAAAAGttc
AtSK21	P$PBF_01	BF	1242	1253	1	1	0.978	ctgAAAAGtgt
AtSK21	P$DOF_Q2	DOF	1242	1253	1	1	0.943	ctgAAAAGtgt
AtSK21	P$CDF2_01	CDF2	1243	1253	1	1	0.972	tgAAAAGtgt
AtSK21	P$CDF3_01	CDF3	1244	1253	1	1	0.994	gAAAAGtgt
AtSK21	P$GT1_Q6	GT1	1251	1258	1	1	0.912	GTGAAca
AtSK21	P$ATSPL8_01	ATSPL8	1281	1297	1	1	0.916	aggacTGTACgttcca
AtSK21	P$SPL11_01	SPL11	1283	1295	1	1	0.909	gactGTACGttc
AtSK21	P$SPL5_01	SPL5	1285	1294	1	1	0.972	ctGTACGtt
AtSK21	P$POPTR_01	OPTR	1286	1293	1	1	0.93	tGTACGt
AtSK21	P$SPL12_01	SPL12	1286	1294	1	1	0.978	tGTACGtt
AtSK21	P$SPL4_01	SPL4	1286	1295	1	1	0.993	tGTACGttc
AtSK21	P$HSFA2_01	HSFA2	1294	1300	1	1	1	CCAAAa
AtSK21	P$ABI3_01	ABI3	1300	1309	1	1	0.89	caGCATGaa
AtSK21	P$AT4G36620_01	AT4G36620	1304	1312	1	1	0.98	atgAACCA
AtSK21	P$GT1_Q6_02	GT1	1318	1330	1	1	0.994	aaaataTTAACa
AtSK21	P$WRKY48_01	WRKY48	1322	1331	1	1	0.872	tattAACAA
AtSK21	P$FLC_01	FLC	1323	1344	1	1	0.868	attaacaaaatatAGAAAaat
AtSK21	P$ATMYB15_Q2	ATMYB15	1325	1331	1	1	1	TAACAa
AtSK21	P$AGL15_01	AGL15	1325	1341	1	1	0.875	taacaaaaTATAGaaa
AtSK21	P$BPC1_Q2	BPC1	1336	1342	1	1	0.997	AGAAAa
AtSK21	P$ATHB6_01	ATHB6	1339	1348	1	1	0.975	aaAATAAta
AtSK21	P$ATHB5_04	ATHB5	1339	1350	1	1	0.892	aaAATAAtaaa
AtSK21	P$ATHB1_03	ATHB1	1339	1350	1	1	0.9	aaAATAAtaaa
AtSK21	P$ATHB16_01	ATHB16	1340	1348	1	1	0.867	aAATAAta
AtSK21	P$ATHB6_01	ATHB6	1342	1351	1	1	0.911	atAATAAag
AtSK21	P$DOF1_01	DOF1	1343	1354	1	1	0.979	taaTAAAGaca
AtSK21	P$REF6_01	REF6	1348	1359	1	1	0.868	aagaCAGAGag
AtSK21	P$PBF_01	BF	1359	1370	1	1	0.979	gctAAAAGaga
AtSK21	P$DOF_Q2	DOF	1359	1370	1	1	0.938	gctAAAAGaga
AtSK21	P$CDF2_01	CDF2	1360	1370	1	1	0.953	ctAAAAGaga
AtSK21	P$CDF3_01	CDF3	1361	1370	1	1	0.979	tAAAAGaga
AtSK21	P$PEND_01	END	1382	1390	1	1	1	tAAGAAgt

AtSK21	P$WRKY11_Q2	WRKY11	1389	1397	1	1	0.952	tTTGACta
AtSK21	P$ATHB6_01	ATHB6	1395	1404	1	1	0.911	taAATAAgt
AtSK21	P$WRKY11_Q2	WRKY11	1411	1419	1	1	0.952	tTTGACta
AtSK21	P$LIM1_01	LIM1	1421	1433	1	1	0.865	CCACCttctccc
AtSK21	P$CBF3_02	CBF3	1429	1443	1	1	0.959	tcccaCCGACtgcc
AtSK21	P$CBF1_04	CBF1	1430	1442	1	1	0.938	cccaCCGACtgc
AtSK21	P$AT1G77200_03	AT1G77200	1431	1445	1	1	0.893	ccaCCGACtgccaa
AtSK21	P$ARF1_01	ARF1	1433	1441	1	1	0.887	aCCGACtg
AtSK21	P$ARF5_01	ARF5	1433	1441	1	1	0.891	aCCGACtg
AtSK21	P$DREB1B_01	DREB1B	1434	1439	1	1	1	CCGAC
AtSK21	P$MYBAS1_01	MYBAS1	1439	1450	1	1	0.977	tgCCAACccat
AtSK21	P$GAMYB_01	GAMYB	1442	1450	1	1	0.871	CAACCcat
AtSK21	P$ASR1_01	ASR1	1444	1449	1	1	1	ACCCA
AtSK21	P$WRKY18_02	WRKY18	1449	1459	1	1	0.946	tccGTCAAta
AtSK21	P$WRKY21_02	WRKY21	1449	1459	1	1	0.948	tccGTCAAta
AtSK21	P$WRKY48_02	WRKY48	1449	1459	1	1	0.987	tccGTCAAta
AtSK21	P$WRKY57_01	WRKY57	1449	1459	1	1	0.954	tccGTCAAta
AtSK21	P$WRKY60_01	WRKY60	1449	1460	1	1	0.889	tccGTCAAtat
AtSK21	P$WRKY15_01	WRKY15	1450	1460	1	1	0.959	ccGTCAAtat
AtSK21	P$WRKY2_01	WRKY2	1450	1458	1	1	0.907	ccGTCAAt
AtSK21	P$WRKY25_02	WRKY25	1450	1458	1	1	0.894	ccGTCAAt
AtSK21	P$WRKY40_01	WRKY40	1450	1458	1	1	0.981	ccGTCAAt
AtSK21	P$WRKY43_02	WRKY43	1450	1460	1	1	0.955	ccGTCAAtat
AtSK21	P$WRKY63_01	WRKY63	1450	1458	1	1	0.887	ccGTCAAt
AtSK21	P$WRKY75_01	WRKY75	1450	1458	1	1	0.928	ccGTCAAt
AtSK21	P$WRKY8_01	WRKY8	1450	1459	1	1	0.978	ccGTCAAta
AtSK21	P$WRKY30_01	WRKY30	1451	1461	1	1	0.908	cGTCAAtatt
AtSK21	P$WRKY18_Q2	WRKY18	1452	1461	1	1	0.968	GTCAAtatt
AtSK21	P$MRP1_Q2	MRP1	1459	1471	1	1	0.951	ttTCTATcttca
AtSK21	P$ARR2_01	ARR2	1460	1470	1	1	0.858	ttctATCTTc
AtSK21	P$SPF1_Q2	SPF1	1511	1521	1	1	0.896	atATAGTttt
AtSK21	P$AZF3_01	AZF3	1531	1542	1	1	0.853	tAGTATggtct
AtSK21	P$DOF1_01	DOF1	1538	1549	1	1	0.975	gtcTAAAGact
AtSK21	P$C1_Q2	C1	1551	1562	1	1	0.919	aaAACTAaata
AtSK21	P$ATHB6_01	ATHB6	1556	1565	1	1	0.905	taAATAAaa
AtSK21	P$HDG9_01	HDG9	1571	1585	1	1	0.853	gaagTAAATgcaaa
AtSK21	P$PDF2_01	DF2	1571	1582	1	1	0.946	gaagTAAATgc
AtSK21	P$C1_Q2	C1	1598	1609	1	1	0.939	taAACTAtatt
AtSK21	P$SBF1_01	SBF1	1644	1658	1	1	0.905	atgtgaTTAAAtac
AtSK21	P$PDF2_01	DF2	1647	1658	1	1	0.875	tgatTAAATac
AtSK21	P$GT1_Q6_01	GT1	1659	1671	1	1	0.885	TTTGTatatatt
AtSK21	P$C1_Q2	C1	1709	1720	1	1	0.936	atAACTAttgc
AtSK21	P$HSFA4A_01	HSFA4A	1712	1718	1	1	1	aCTATT
AtSK21	P$HMG1_01	HMG1	1747	1756	1	1	0.962	GTTGTattt
AtSK21	P$TGA1B_01	TGA1B	1764	1774	1	1	0.85	taGACGTtat
AtSK21	P$TGA1A_Q2_01	TGA1A	1769	1791	1	1	0.888	gttATGACatttgtaagttgta
AtSK21	P$KNOX3_01	KNOX3	1769	1781	1	1	0.968	gttaTGACAttt
AtSK21	P$ATH1_01	ATH1	1773	1781	1	1	0.933	TGACAttt
AtSK21	P$AT3G18650_01	AT3G18650	1774	1785	1	1	0.934	gacatTTGTAa
AtSK21	P$HMG1_01	HMG1	1785	1794	1	1	0.922	GTTGTaatg
AtSK21	P$TEIL_01	TEIL	1791	1799	1	1	0.982	ATGTAtct
AtSK21	P$ARR2_01	ARR2	1791	1801	1	1	0.957	atgtATCTTa
AtSK21	P$EDT1_01	EDT1	1800	1810	1	1	0.983	aaaTTAATgt
AtSK21	P$KNOX3_01	KNOX3	1808	1820	1	1	0.964	gtttTGACAccg
AtSK21	P$AP2A_01	AP2A	1809	1819	1	1	0.958	ttttgACACC
AtSK21	P$WRKY11_Q2	WRKY11	1810	1818	1	1	0.899	tTTGACac
AtSK21	P$ATH1_01	ATH1	1812	1820	1	1	0.908	TGACAccg
AtSK21	P$C1_Q2	C1	1824	1835	1	1	0.944	tcAACTAttat
AtSK21	P$HSFA4A_01	HSFA4A	1827	1833	1	1	1	aCTATT
AtSK21	P$TGA1B_01	TGA1B	1834	1844	1	1	0.882	tgGACGTctt
AtSK21	P$TGA1A_01	TGA1A	1836	1843	1	1	0.857	gACGTCt
AtSK21	P$NAC043_01	NAC043	1843	1853	1	1	0.994	ttaACGTAag
AtSK21	P$NAC025_01	NAC025	1845	1853	1	1	0.907	aACGTAag
AtSK21	P$NAC6_01	NAC6	1846	1852	1	1	1	aCGTAA
AtSK21	P$PEND_01	END	1849	1857	1	1	0.892	tAAGAAaa
AtSK21	P$BPC1_Q2	BPC1	1851	1857	1	1	0.997	AGAAAa
AtSK21	P$DOF43_01	DOF43	1854	1865	1	1	0.862	aaactgACTTT
AtSK21	P$CBNAC_01	CBNAC	1885	1891	1	1	0.973	cTGCTT
AtSK21	P$CBNAC_02	CBNAC	1885	1901	1	1	0.862	cTGCTTtacaaattaa
AtSK21	P$EDT1_01	EDT1	1894	1904	1	1	0.883	aaaTTAATct
AtSK21	P$ARR2_01	ARR2	1896	1906	1	1	0.867	attaATCTTt
AtSK21	P$CBNAC_01	CBNAC	1904	1910	1	1	1	tTGCTT
AtSK21	P$CBNAC_02	CBNAC	1904	1920	1	1	0.887	tTGCTTatataatttt
AtSK21	P$SBF1_01	SBF1	1913	1927	1	1	0.863	taatttTTAATgat
AtSK21	P$GT1_Q6_01	GT1	1916	1928	1	1	0.871	TTTTTaatgata
AtSK21	P$EDT1_01	EDT1	1916	1926	1	1	0.941	tttTTAATga
AtSK21	P$HAHB4_01	HAHB4	1920	1929	1	1	0.951	tAATGAtaa
AtSK21	P$ATHB6_01	ATHB6	1925	1934	1	1	0.913	atAATAAga

AtSK21	P$DOF1_01	DOF1	1932	1943	1	1	0.98	gatTAAAGaat
AtSK21	P$AP1_01	AP1	1946	1959	1	1	0.864	atAAAAAaaaaaa
AtSK21	P$CCA1_Q5	CCA1	1950	1967	1	1	0.851	aaaaaAAAATatatatt
AtSK21	P$SBF1_01	SBF1	1959	1973	1	1	0.852	tatataTTAAAagt
AtSK21	P$PBF_01	BF	1964	1975	1	1	0.977	attAAAAGtaa
AtSK21	P$DOF_Q2	DOF	1964	1975	1	1	0.952	attAAAAGtaa
AtSK21	P$CDF2_01	CDF2	1965	1975	1	1	0.97	ttAAAAGtaa
AtSK21	P$CDF3_01	CDF3	1966	1975	1	1	0.986	tAAAAGtaa
AtSK21	P$TEIL_01	TEIL	1974	1982	1	1	0.922	ATGTAtat
AtSK21	P$HSFA2_01	HSFA2	1990	1996	1	1	0.933	CCAAAg
AtSK21	P$ARR18_01	ARR18	1990	2003	1	1	0.888	ccaaAGATAatct
AtSK21	P$SBF1_01	SBF1	2001	2015	1	1	0.881	ctgaggTTAAAgtt
AtSK21	P$DOF1_01	DOF1	2005	2016	1	1	0.974	ggtTAAAGtta
AtSK21	P$ATHB6_01	ATHB6	2015	2024	1	1	0.996	acAATAAta
AtSK21	P$ATHB5_04	ATHB5	2015	2026	1	1	0.924	acAATAAtaat
AtSK21	P$ATHB1_03	ATHB1	2015	2026	1	1	0.905	acAATAAtaat
AtSK21	P$ATHB16_01	ATHB16	2016	2024	1	1	0.878	cAATAAta
AtSK21	P$ATHB6_01	ATHB6	2018	2027	1	1	0.978	atAATAAtc
AtSK21	P$ATHB5_04	ATHB5	2018	2029	1	1	0.899	atAATAAtcat
AtSK21	P$ATHB1_03	ATHB1	2018	2029	1	1	0.889	atAATAAtcat
AtSK21	P$ATHB16_01	ATHB16	2019	2027	1	1	0.908	tAATAAtc
AtSK21	P$ATHB7_01	ATHB7	2021	2031	1	1	0.922	atAATCAtat
AtSK21	P$HAT1_01	HAT1	2021	2031	1	1	0.98	atAATCAtat
AtSK21	P$BPC1_Q2	BPC1	2031	2037	1	1	0.99	AGAAAc
AtSK21	P$GT1_Q6_02	GT1	2047	2059	1	1	0.877	gcaaaaTTAACt
AtSK21	P$LEC2_01	LEC2	2061	2072	1	1	0.991	gaCATGCaatt
AtSK21	P$KNOX3_01	KNOX3	2079	2091	1	1	0.962	ttctTGACAtta
AtSK21	P$WRKY11_Q2	WRKY11	2081	2089	1	1	0.901	cTTGACat
AtSK21	P$ATH1_01	ATH1	2083	2091	1	1	0.934	TGACAtta
AtSK21	P$MYBPH3_01	MYBPH3	2105	2118	1	1	0.896	gaaactCGGTTat
AtSK21	P$ATMYB77_01	ATMYB77	2105	2118	1	1	0.855	gaaactCGGTTat
AtSK21	P$BPC1_Q2	BPC1	2148	2154	1	1	0.997	AGAAAa
AtSK21	P$ATHB7_01	ATHB7	2150	2160	1	1	0.882	aaAATCAatt
AtSK21	P$HAT1_01	HAT1	2150	2160	1	1	0.875	aaAATCAatt
AtSK21	P$ATHB6_01	ATHB6	2170	2179	1	1	0.925	tcAATAAct
AtSK21	P$GT1_Q6_01	GT1	2178	2190	1	1	0.875	TTTTTagtaaaa
AtSK21	P$C1_Q2	C1	2186	2197	1	1	0.949	aaAACTAactg
AtSK21	P$WEREWOLF_Q2_01	WEREWOLF	2188	2197	1	1	0.937	aACTAActg
AtSK21	P$MYBAS1_01	MYBAS1	2188	2199	1	1	0.96	aaCTAACtgga
AtSK21	P$ARR1_01	ARR1	2205	2215	1	1	0.943	gatGAATCat
AtSK21	P$ATHB7_01	ATHB7	2207	2217	1	1	0.915	tgAATCAtat
AtSK21	P$HAT1_01	HAT1	2207	2217	1	1	0.969	tgAATCAtat
AtSK21	P$AP1_01	AP1	2226	2239	1	1	0.883	acAAAAAaaaaaa
AtSK21	P$C1_Q2	C1	2235	2246	1	1	0.95	aaAACTAacgg
AtSK21	P$WEREWOLF_Q2_01	WEREWOLF	2237	2246	1	1	0.958	aACTAAcgg
AtSK21	P$MYBAS1_01	MYBAS1	2237	2248	1	1	0.951	aaCTAACggag
AtSK21	P$MYB52_01	MYB52	2238	2250	1	1	0.884	actAACGGaggt
AtSK21	P$AT1G69560_01	AT1G69560	2238	2250	1	1	0.861	actAACGGaggt
AtSK21	P$SPF1_Q2	SPF1	2253	2263	1	1	0.883	caATAGTtta
AtSK21	P$CBNAC_01	CBNAC	2269	2275	1	1	0.968	aTGCTT
AtSK21	P$PEND_01	END	2275	2283	1	1	0.874	gAAGAAat
AtSK21	P$BPC1_Q2	BPC1	2277	2283	1	1	0.99	AGAAAt
AtSK21	P$MYB80_01	MYB80	2286	2297	1	1	0.951	aaGAATAtgct
AtSK21	P$CBNAC_01	CBNAC	2292	2298	1	1	0.968	aTGCTT
AtSK21	P$RAV1_02	RAV1	2322	2334	1	1	0.913	gttACCTGcaaa
AtSK21	P$PEND_02	END	2341	2351	1	1	0.912	atTTCTTttt
AtSK21	P$CBF3_02	CBF3	2347	2361	1	1	0.957	ttttgCCGACggtt
AtSK21	P$CBF1_04	CBF1	2348	2360	1	1	0.953	tttgCCGACggt
AtSK21	P$CBF1_01	CBF1	2349	2359	1	1	0.941	tTGCCGacgg
AtSK21	P$ERF019_01	ERF019	2349	2359	1	1	0.858	tTGCCGacgg
AtSK21	P$JERF3_01	JERF3	2349	2359	1	1	0.906	tTGCCGacgg
AtSK21	P$CEF1_01	CEF1	2349	2359	1	1	0.909	tTGCCGacgg
AtSK21	P$JERF1_01	JERF1	2349	2359	1	1	0.936	tTGCCGacgg
AtSK21	P$CBF1_03	CBF1	2349	2359	1	1	0.94	tTGCCGacgg
AtSK21	P$DREB1F_01	DREB1F	2349	2359	1	1	0.957	tTGCCGacgg
AtSK21	P$AT1G33760_01	AT1G33760	2349	2359	1	1	0.865	tTGCCGacgg
AtSK21	P$ERF5_02	ERF5	2349	2359	1	1	0.905	ttGCCGAcgg
AtSK21	P$ERF1_04	ERF1	2349	2359	1	1	0.905	ttGCCGAcgg
AtSK21	P$DREB1G_02	DREB1G	2349	2359	1	1	0.912	ttgCCGACgg
AtSK21	P$ATMYB77_01	ATMYB77	2350	2363	1	1	0.937	tgccgaCGGTTgg
AtSK21	P$ARF1_01	ARF1	2351	2359	1	1	0.854	gCCGACgg
AtSK21	P$ARF5_01	ARF5	2351	2359	1	1	0.897	gCCGACgg
AtSK21	P$DREB1B_01	DREB1B	2352	2357	1	1	1	CCGAC
AtSK21	P$MYBPH3_02	MYBPH3	2361	2374	1	1	0.887	ggctgtTAGTTag
AtSK21	P$WEREWOLF_Q2	WEREWOLF	2363	2372	1	1	1	ctGTTAGtt
AtSK21	P$MYB24_01	MYB24	2367	2376	1	1	0.923	tagTTAGGa
AtSK21	P$AT3G51080_01	AT3G51080	2373	2380	1	1	1	GGAAAaa
AtSK21	P$PBF_01	BF	2373	2384	1	1	0.95	ggaAAAAGaat

AtSK21	P$DOF_Q2	DOF	2373	2384	1	1	0.991	ggaAAAAGaat
AtSK21	P$CDF2_01	CDF2	2374	2384	1	1	0.977	gaAAAAGaat
AtSK21	P$CDF3_01	CDF3	2375	2384	1	1	0.974	aAAAAGaat
AtSK21	P$MYB3R1_01	MYB3R1	2398	2413	1	1	0.859	tttctcttCCGTTtc
AtSK21	P$ATSPL3_01	ATSPL3	2407	2423	1	1	0.959	cgtttCGTACaaaccc
AtSK21	P$SMZ_01	SMZ	2409	2417	1	1	0.876	ttTCGTAc
AtSK21	P$SPL14_03	SPL14	2410	2421	1	1	0.887	ttCGTACaaac
AtSK21	P$ASR1_01	ASR1	2419	2424	1	1	1	ACCCA
AtSK21	P$ATHB7_01	ATHB7	2421	2431	1	1	1	ccAATCAtta
AtSK21	P$HAT1_01	HAT1	2421	2431	1	1	1	ccAATCAtta
AtSK21	P$PHV_02	HV	2421	2436	1	1	0.907	ccaATCATtattctc
AtSK21	P$ATHB4_02	ATHB4	2422	2432	1	1	0.956	caATCATtat
AtSK21	P$GT1_Q6_02	GT1	2440	2452	1	1	0.858	ttatacTTAACt
AtSK21	P$AT1G66560_01	AT1G66560	2443	2453	1	1	0.957	tacTTAACtt
AtSK21	P$WRKY21_01	WRKY21	2444	2453	1	1	0.927	acTTAACtt
AtSK21	P$AT2G34830_01	AT2G34830	2444	2453	1	1	0.931	acTTAACtt
AtSK21	P$AT1G18860_01	AT1G18860	2444	2453	1	1	0.987	acTTAACtt
AtSK21	P$AT1G64000_01	AT1G64000	2444	2453	1	1	0.911	acTTAACtt
AtSK21	P$AT4G22070_01	AT4G22070	2444	2453	1	1	0.947	acTTAACtt
AtSK21	P$WRKY6_01	WRKY6	2444	2453	1	1	0.946	acTTAACtt
AtSK21	P$AT1G66600_01	AT1G66600	2444	2453	1	1	0.959	acTTAACtt
AtSK21	P$AT1G68150_01	AT1G68150	2444	2453	1	1	0.962	acTTAACtt
AtSK21	P$AT5G41570_01	AT5G41570	2444	2453	1	1	0.91	acTTAACtt
AtSK21	P$AT1G69810_01	AT1G69810	2444	2453	1	1	0.963	acTTAACtt
AtSK21	P$AT5G15130_01	AT5G15130	2444	2453	1	1	0.987	acTTAACtt
AtSK21	P$WRKY46_01	WRKY46	2444	2453	1	1	0.917	acTTAACtt
AtSK21	P$AT1G30650_01	AT1G30650	2444	2453	1	1	0.934	acTTAACtt
AtSK21	P$AT2G24570_01	AT2G24570	2444	2453	1	1	0.927	acTTAACtt
AtSK21	P$AT4G23550_01	AT4G23550	2444	2453	1	1	0.928	acTTAACtt
AtSK21	P$WRKY7_01	WRKY7	2444	2453	1	1	0.923	acTTAACtt
AtSK21	P$O2_Q4	O2	2462	2473	1	1	0.921	ttttCATGTta
AtSK21	P$ARR2_01	ARR2	2472	2482	1	1	0.867	atcaATCTTt
AtSK21	P$AGL20_01	AGL20	2480	2492	1	1	0.893	ttTAAATtaaat
AtSK21	P$SBF1_01	SBF1	2480	2494	1	1	0.873	tttaaaTTAAAtat
AtSK21	P$WRKY18_02	WRKY18	2494	2504	1	1	0.948	atcGTCAAtg
AtSK21	P$WRKY21_02	WRKY21	2494	2504	1	1	0.953	atcGTCAAtg
AtSK21	P$WRKY48_02	WRKY48	2494	2504	1	1	0.988	atcGTCAAtg
AtSK21	P$WRKY57_01	WRKY57	2494	2504	1	1	0.959	atcGTCAAtg
AtSK21	P$WRKY60_01	WRKY60	2494	2505	1	1	0.894	atcGTCAAtga
AtSK21	P$WRKY15_01	WRKY15	2495	2505	1	1	0.962	tcGTCAAtga
AtSK21	P$WRKY2_01	WRKY2	2495	2503	1	1	0.905	tcGTCAAt
AtSK21	P$WRKY25_02	WRKY25	2495	2503	1	1	0.888	tcGTCAAt
AtSK21	P$WRKY40_01	WRKY40	2495	2503	1	1	0.981	tcGTCAAt
AtSK21	P$WRKY43_02	WRKY43	2495	2505	1	1	0.955	tcGTCAAtga
AtSK21	P$WRKY62_01	WRKY62	2495	2503	1	1	0.87	tcGTCAAt
AtSK21	P$WRKY63_01	WRKY63	2495	2503	1	1	0.886	tcGTCAAt
AtSK21	P$WRKY75_01	WRKY75	2495	2503	1	1	0.928	tcGTCAAt
AtSK21	P$WRKY8_01	WRKY8	2495	2504	1	1	0.979	tcGTCAAtg
AtSK21	P$WRKY30_01	WRKY30	2496	2506	1	1	0.915	cGTCAAtgac
AtSK21	P$WRKY18_Q2	WRKY18	2497	2506	1	1	0.924	GTCAAtgac
AtSK21	P$TGA1A_Q2_01	TGA1A	2498	2520	1	1	0.909	tcaATGACtgttatgaataatt
AtSK21	P$ATHB6_01	ATHB6	2511	2520	1	1	0.976	tgAATAAtt
AtSK21	P$ATHB5_04	ATHB5	2511	2522	1	1	0.953	tgAATAAttag
AtSK21	P$ATHB1_03	ATHB1	2511	2522	1	1	0.962	tgAATAAttag
AtSK21	P$ATHB16_01	ATHB16	2512	2520	1	1	0.947	gAATAAtt
AtSK21	P$ABF2_01	ABF2	2559	2572	1	1	0.896	tggtgCACGTtat
AtSK21	P$O2_Q4	O2	2560	2571	1	1	0.854	ggtgCACGTta
AtSK21	P$HBI1_01	HBI1	2560	2572	1	1	0.865	ggtgCACGTtat
AtSK21	P$GBP_Q6	GBP	2561	2573	1	1	0.879	gtgCACGTtatt
AtSK21	P$ABI5_01	ABI5	2561	2571	1	1	0.888	gtgCACGTta
AtSK21	P$ABF4_01	ABF4	2561	2573	1	1	0.867	gtgCACGTtatt
AtSK21	P$CPRF3_Q2	CPRF3	2562	2572	1	1	0.909	tgCACGTtat
AtSK21	P$CPRF2_Q2	CPRF2	2562	2572	1	1	0.929	tgCACGTtat
AtSK21	P$O2_02	O2	2562	2572	1	1	0.937	tgCACGTtat
AtSK21	P$TGA1B_Q2	TGA1B	2562	2572	1	1	0.89	tgCACGTtat
AtSK21	P$TGA1A_Q2	TGA1A	2562	2572	1	1	0.966	tgCACGTtat
AtSK21	P$CPRF3_01	CPRF3	2562	2572	1	1	0.92	tgCACGTtat
AtSK21	P$CPRF2_01	CPRF2	2562	2572	1	1	0.931	tgCACGTtat
AtSK21	P$BEE2_01	BEE2	2562	2572	1	1	0.92	tgCACGTtat
AtSK21	P$BIM2_01	BIM2	2562	2572	1	1	0.857	tgCACGTtat
AtSK21	P$BIM3_01	BIM3	2562	2572	1	1	0.89	tgCACGTtat
AtSK21	P$PHYPA143875_02	HYPA143875	2562	2572	1	1	0.887	tgCACGTtat
AtSK21	P$SPT_01	SPT	2562	2571	1	1	0.924	tgCACGTta
AtSK21	P$RITA1_01	RITA1	2563	2570	1	1	0.943	gCACGTt
AtSK21	P$MYC3_01	MYC3	2563	2571	1	1	0.852	gCACGTta
AtSK21	P$OCSBF1_01	OCSBF1	2564	2569	1	1	1	CACGT
AtSK21	P$GATA9_01	GATA9	2599	2610	1	1	0.899	tcaAGATCcct
AtSK21	P$AGP1_01	AGP1	2600	2610	1	1	0.869	caAGATCcct

AtSK21	P$ARR10_01	ARR10	2602	2609	1	1	0.934	AGATCcc
AtSK21	P$AT3G60580_01	AT3G60580	2602	2609	1	1	0.852	agATCCC
AtSK21	P$BPC1_Q2	BPC1	2613	2619	1	1	0.99	AGAAAt
AtSK21	P$ARR2_01	ARR2	2626	2636	1	1	0.885	tataATCTTt
AtSK21	P$TEIL_01	TEIL	2642	2650	1	1	0.883	ATGTAgtt
AtSK21	P$O2_Q4	O2	2655	2666	1	1	0.857	caaaCATGTat
AtSK21	P$P_01		2667	2676	1	1	0.923	atCTACCaa
AtSK21	P$HSFA2_01	HSFA2	2672	2678	1	1	1	CCAAAa
AtSK21	P$ATHB6_01	ATHB6	2674	2683	1	1	0.905	aaAATAAaa
AtSK21	P$PBF_01	BF	2677	2688	1	1	0.985	ataAAAAGggc
AtSK21	P$DOF_Q2	DOF	2677	2688	1	1	0.984	ataAAAAGggc
AtSK21	P$CDF2_01	CDF2	2678	2688	1	1	0.983	taAAAAGggc
AtSK21	P$CDF3_01	CDF3	2679	2688	1	1	0.988	aAAAAGggc
AtSK21	P$PBF_Q2	BF	2680	2686	1	1	1	aAAAGG
AtSK21	P$PBF_01	BF	2686	2697	1	1	0.952	gctAAAAGctt
AtSK21	P$DOF_Q2	DOF	2686	2697	1	1	0.928	gctAAAAGctt
AtSK21	P$DOF2_01	DOF2	2686	2697	1	1	0.989	gctaAAAGCtt
AtSK21	P$DOF3_01	DOF3	2686	2697	1	1	0.988	gctaAAAGCtt
AtSK21	P$CDF2_01	CDF2	2687	2697	1	1	0.958	ctAAAAGctt
AtSK21	P$CDF3_01	CDF3	2688	2697	1	1	0.977	tAAAAGctt
AtSK21	P$SEP3_01	wrz-03	2707	2718	1	1	0.898	accgcTTTTGg
AtSK21	P$ATHSFA1D_01	ATHSFA1D	2731	2737	1	1	0.985	cCTACA
AtSK21	P$SPF1_Q2	SPF1	2740	2750	1	1	0.949	caATAGTatt
AtSK21	P$HSFA4A_01	HSFA4A	2752	2758	1	1	1	aCTATT
AtSK21	P$WRKY48_01	WRKY48	2760	2769	1	1	0.865	atatAACAA
AtSK21	P$ATMYB15_Q2	ATMYB15	2763	2769	1	1	1	TAACAa
AtSK21	P$ARR1_01	ARR1	2766	2776	1	1	0.947	caaGAATCgt
AtSK21	P$ALFIN1_Q2	ALFIN1	2768	2783	1	1	0.869	agaatcGTGGGattt
AtSK21	P$BZIP68_01	BZIP68	2770	2779	1	1	0.924	aatCGTGGg
AtSK21	P$EDT1_01	EDT1	2782	2792	1	1	0.854	tgtTTAATcc
AtSK21	P$SPL14_02	SPL14	2784	2803	1	1	0.977	tttaatCCGTAcaatgaaa
AtSK21	P$ATSPL3_01	ATSPL3	2786	2802	1	1	0.976	taatcCGTACaatgaa
AtSK21	P$SPL3_01	SPL3	2789	2797	1	1	1	tCCGTAca
AtSK21	P$SPL14_03	SPL14	2789	2800	1	1	0.998	tcCGTACaatg
AtSK21	P$SPL14_01	SPL14	2790	2797	1	1	1	CCGTAca
AtSK21	P$FLC_01	FLC	2792	2813	1	1	0.856	gtacaatgaaacaAGAAAgat
AtSK21	P$BPC1_Q2	BPC1	2805	2811	1	1	1	AGAAAg
AtSK21	P$ARR18_01	ARR18	2805	2818	1	1	0.912	agaaAGATAtaca
AtSK21	P$ATHB6_01	ATHB6	2815	2824	1	1	0.924	acAATAAct
AtSK21	P$SBF1_01	SBF1	2817	2831	1	1	0.872	aataacTTAATatc
AtSK21	P$GT1_Q6_01	GT1	2832	2844	1	1	0.867	TTTTTttttggg
AtSK21	P$SEP3_01	wrz-03	2832	2843	1	1	0.906	tttttTTTTGg
AtSK21	P$ARR2_01	ARR2	2844	2854	1	1	0.886	gacaATCTTa
AtSK21	P$SBF1_01	SBF1	2845	2859	1	1	0.872	acaatcTTAATatc
AtSK21	P$ATHB4_02	ATHB4	2854	2864	1	1	0.851	atATCATttc
AtSK21	P$AGL27_01	AGL27	2859	2873	1	1	0.856	aTTTCTaatttcta
AtSK21	P$AT4G36620_01	AT4G36620	2873	2881	1	1	0.902	tagAACCA
AtSK21	P$HSFA2_01	HSFA2	2878	2884	1	1	1	CCAAAa
AtSK21	P$GAMYB_Q2	GAMYB	2888	2901	1	1	0.92	tcataACAACtta
AtSK21	P$ATMYB15_Q2	ATMYB15	2891	2897	1	1	1	TAACAa
AtSK21	P$AT3G20750_01	AT3G20750	2898	2906	1	1	0.907	tTAAACtt
AtSK21	P$GT1_Q6_01	GT1	2908	2920	1	1	0.889	TTTTTtgtgaga
AtSK21	P$BPC1_Q2	BPC1	2917	2923	1	1	0.997	AGAAAa
AtSK21	P$ATHB6_01	ATHB6	2920	2929	1	1	0.9	aaAATAAca
AtSK21	P$GAMYB_Q2	GAMYB	2921	2934	1	1	0.911	aaataACAACtta
AtSK21	P$ATMYB15_Q2	ATMYB15	2924	2930	1	1	1	TAACAa
AtSK21	P$EDT1_01	EDT1	2928	2938	1	1	0.857	aacTTAATta
AtSK21	P$ATHSFA1D_01	ATHSFA1D	2944	2950	1	1	1	aCTACA
AtSK21	P$KNOX3_01	KNOX3	2947	2959	1	1	0.962	acatTGACAatt
AtSK21	P$WRKY11_Q2	WRKY11	2949	2957	1	1	0.924	aTTGACaa
AtSK21	P$ATH1_01	ATH1	2951	2959	1	1	0.919	TGACAatt
AtSK21	P$GT1_Q6_02	GT1	2952	2964	1	1	0.881	gacaatTTAACa
AtSK21	P$ARR2_01	ARR2	2959	2969	1	1	0.887	taacATCTTc
AtSK21	P$AT4G12750_01	AT4G12750	2973	2983	1	1	0.969	aaaACCGAat
AtSK21	P$AT2G41690_01	AT2G41690	2977	2983	1	1	0.974	CCGAAt
AtSK21	P$TEIL_01	TEIL	2981	2989	1	1	0.922	ATGTAtat
AtSK21	P$HSFA4A_01	HSFA4A	3008	3014	1	1	0.91	gCTATT
AtSK21	P$ANTL_02	ANTL	3038	3048	1	1	0.9	tatCGACAtt
AtSK21	P$ATHB6_01	ATHB6	3048	3057	1	1	0.979	taAATAAtt
AtSK21	P$ATHB5_04	ATHB5	3048	3059	1	1	0.964	taAATAAttga
AtSK21	P$ATHB1_03	ATHB1	3048	3059	1	1	0.987	taAATAAttga
AtSK21	P$ATHB16_01	ATHB16	3049	3057	1	1	0.953	aAATAAtt
AtSK21	P$ARR18_01	ARR18	3061	3074	1	1	0.888	ctgaAGATAattc
AtSK21	P$TEIL_01	TEIL	3085	3093	1	1	0.879	ATGTAaat
AtSK21	P$PBF_01	BF	3104	3115	1	1	0.952	gccAAAAGtat
AtSK21	P$DOF_Q2	DOF	3104	3115	1	1	0.938	gccAAAAGtat
AtSK21	P$HSFA2_01	HSFA2	3105	3111	1	1	1	CCAAAa
AtSK21	P$CDF2_01	CDF2	3105	3115	1	1	0.968	ccAAAAGtat

AtSK21	P$CDF3_01	CDF3	3106	3115	1	1	0.984	cAAAAGtat
AtSK21	P$TEIL_01	TEIL	3113	3121	1	1	0.931	ATGTAtgt
AtSK21	P$LEC2_01	LEC2	3121	3132	1	1	0.988	ccCATGCataa
AtSK21	P$FUS3_Q2	FUS3	3122	3133	1	1	0.896	cCATGCataat
AtSK21	P$IDEF1_Q2	IDEF1	3123	3135	1	1	0.859	CATGCataattg
AtSK21	P$WRKY48_01	WRKY48	3131	3140	1	1	0.881	attgAACAA
AtSK21	P$PBF_01	BF	3136	3147	1	1	0.968	acaAAAAGaaa
AtSK21	P$DOF_Q2	DOF	3136	3147	1	1	1	acaAAAAGaaa
AtSK21	P$CDF2_01	CDF2	3137	3147	1	1	0.977	caAAAAGaaa
AtSK21	P$CDF3_01	CDF3	3138	3147	1	1	0.975	aAAAAGaaa
AtSK21	P$PEND_01	END	3140	3148	1	1	0.864	aAAGAAat
AtSK21	P$BPC1_Q2	BPC1	3142	3148	1	1	0.99	AGAAAt
AtSK21	P$SBF1_01	SBF1	3146	3160	1	1	0.882	attataTTAATatt
AtSK21	P$EDT1_01	EDT1	3149	3159	1	1	0.855	ataTTAATat
AtSK21	P$HMG1_01	HMG1	3166	3175	1	1	0.986	GTTGTcttc
AtSK21	P$PHV_02	HV	3191	3206	1	1	0.863	gctATCATtatcatc
AtSK21	P$ATHB4_02	ATHB4	3192	3202	1	1	0.882	ctATCATtat
AtSK21	P$PHV_02	HV	3200	3215	1	1	0.896	atcATCATtgatttt
AtSK21	P$ATHB4_02	ATHB4	3201	3211	1	1	0.912	tcATCATtga
AtSK21	P$AT1G77950_01	AT1G77950	3207	3218	1	1	0.853	ttgatTTTAAt
AtSK21	P$SBF1_01	SBF1	3207	3221	1	1	0.912	ttgattTTAATatt
AtSK21	P$BPC1_Q2	BPC1	3230	3236	1	1	0.997	AGAAAa
AtSK21	P$ATHB6_01	ATHB6	3233	3242	1	1	0.908	aaAATAAat
AtSK21	P$ATHB6_01	ATHB6	3237	3246	1	1	0.903	taAATAAac
AtSK21	P$AT3G20750_01	AT3G20750	3240	3248	1	1	0.907	aTAAACaa
AtSK21	P$DOF1_01	DOF1	3248	3259	1	1	0.985	ataTAAAGaaa
AtSK21	P$BPC1_Q2	BPC1	3254	3260	1	1	0.997	AGAAAa
AtSK21	P$ATHB6_01	ATHB6	3268	3277	1	1	0.932	acAATAAgt
AtSK21	P$DOF1_01	DOF1	3273	3284	1	1	0.993	aagTAAAGcta
AtSK21	P$DOF2_01	DOF2	3273	3284	1	1	0.998	aagtAAAGCta
AtSK21	P$DOF3_01	DOF3	3273	3284	1	1	0.99	aagtAAAGCta
AtSK21	P$ATHSFA1D_01	ATHSFA1D	3280	3286	1	1	0.94	gCTACA
AtSK21	P$HSFA4A_01	HSFA4A	3285	3291	1	1	1	aCTATT
AtSK21	P$MYB24_01	MYB24	3286	3295	1	1	0.962	ctaTTAGGt
AtSK21	P$MYB131_01	MYB131	3286	3297	1	1	0.955	ctaTTAGGtaa
AtSK21	P$MYB3_01	MYB3	3287	3298	1	1	0.877	tatTAGGTaat
AtSK21	P$MYB4_01	MYB4	3288	3296	1	1	0.875	atTAGGTa
AtSK21	P$ATHB6_01	ATHB6	3293	3302	1	1	0.984	gtAATAAtt
AtSK21	P$ATHB5_04	ATHB5	3293	3304	1	1	0.979	gtAATAAttaa
AtSK21	P$ATHB1_03	ATHB1	3293	3304	1	1	0.985	gtAATAAttaa
AtSK21	P$ATHB16_01	ATHB16	3294	3302	1	1	1	tAATAAtt
AtSK21	P$DOF1_01	DOF1	3298	3309	1	1	0.999	aatTAAAGctc
AtSK21	P$DOF2_01	DOF2	3298	3309	1	1	0.999	aattAAAGCtc
AtSK21	P$DOF3_01	DOF3	3298	3309	1	1	0.989	aattAAAGCtc
AtSK21	P$RIN_Q2	RIN	3308	3319	1	1	0.965	cattTTTAAgt
AtSK21	P$EDT1_01	EDT1	3324	3334	1	1	0.855	attTTAATta
AtSK21	P$ATHB6_01	ATHB6	3335	3344	1	1	0.905	aaAATAAaa
AtSK21	P$SBF1_01	SBF1	3341	3355	1	1	0.882	aaaacaTTAAAaag
AtSK21	P$PBF_01	BF	3347	3358	1	1	0.969	ttaAAAAGtta
AtSK21	P$DOF_Q2	DOF	3347	3358	1	1	0.977	ttaAAAAGtta
AtSK21	P$CDF2_01	CDF2	3348	3358	1	1	0.993	taAAAAGtta
AtSK21	P$CDF3_01	CDF3	3349	3358	1	1	0.989	aAAAAGtta
AtSK21	P$ASR1_01	ASR1	3360	3365	1	1	1	ACCCA
AtSK21	P$HSFA2_01	HSFA2	3362	3368	1	1	0.933	CCAAAg
AtSK21	P$BPC1_Q2	BPC1	3366	3372	1	1	0.99	AGAAAc
AtSK21	P$PDF2_01	DF2	3369	3380	1	1	0.93	aacaTAAATgc
AtSK21	P$CBNAC_01	CBNAC	3376	3382	1	1	0.968	aTGCTT
AtSK21	P$WRKY18_02	WRKY18	3394	3404	1	1	0.945	aatGTCAAaa
AtSK21	P$WRKY21_02	WRKY21	3394	3404	1	1	0.955	aatGTCAAaa
AtSK21	P$WRKY48_02	WRKY48	3394	3404	1	1	0.987	aatGTCAAaa
AtSK21	P$WRKY57_01	WRKY57	3394	3404	1	1	0.967	aatGTCAAaa
AtSK21	P$WRKY60_01	WRKY60	3394	3405	1	1	0.892	aatGTCAAaaa
AtSK21	P$WRKY15_01	WRKY15	3395	3405	1	1	0.964	atGTCAAaaa
AtSK21	P$WRKY2_01	WRKY2	3395	3403	1	1	0.908	atGTCAAa
AtSK21	P$WRKY25_02	WRKY25	3395	3403	1	1	0.883	atGTCAAa
AtSK21	P$WRKY40_01	WRKY40	3395	3403	1	1	0.977	atGTCAAa
AtSK21	P$WRKY43_02	WRKY43	3395	3405	1	1	0.957	atGTCAAaaa
AtSK21	P$WRKY63_01	WRKY63	3395	3403	1	1	0.885	atGTCAAa
AtSK21	P$WRKY75_01	WRKY75	3395	3403	1	1	0.936	atGTCAAa
AtSK21	P$WRKY8_01	WRKY8	3395	3404	1	1	0.98	atGTCAAaa
AtSK21	P$WRKY30_01	WRKY30	3396	3406	1	1	0.898	tGTCAAaaaa
AtSK21	P$WRKY18_Q2	WRKY18	3397	3406	1	1	0.942	GTCAAaaaa
AtSK21	P$PBF_01	BF	3399	3410	1	1	0.968	caaAAAAGaag
AtSK21	P$DOF_Q2	DOF	3399	3410	1	1	0.996	caaAAAAGaag
AtSK21	P$CDF2_01	CDF2	3400	3410	1	1	0.977	aaAAAAGaag
AtSK21	P$CDF3_01	CDF3	3401	3410	1	1	0.974	aAAAAGaag
AtSK21	P$PEND_01	END	3403	3411	1	1	0.891	aAAGAAgt
AtSK21	P$LIM1_01	LIM1	3415	3427	1	1	0.929	CCACCaaatggt

AtSK21	P$HSFA2_01	HSFA2	3418	3424	1	1	0.922	CCAAAt
AtSK21	P$WRKY18_02	WRKY18	3422	3432	1	1	0.997	atgGTCAAac
AtSK21	P$WRKY21_02	WRKY21	3422	3432	1	1	0.969	atgGTCAAac
AtSK21	P$WRKY48_02	WRKY48	3422	3432	1	1	0.998	atgGTCAAac
AtSK21	P$WRKY57_01	WRKY57	3422	3432	1	1	0.975	atgGTCAAac
AtSK21	P$WRKY60_01	WRKY60	3422	3433	1	1	0.979	atgGTCAAact
AtSK21	P$WRKY15_01	WRKY15	3423	3433	1	1	0.985	tgGTCAAact
AtSK21	P$WRKY2_01	WRKY2	3423	3431	1	1	0.989	tgGTCAAa
AtSK21	P$WRKY25_02	WRKY25	3423	3431	1	1	0.973	tgGTCAAa
AtSK21	P$WRKY40_01	WRKY40	3423	3431	1	1	1	tgGTCAAa
AtSK21	P$WRKY43_02	WRKY43	3423	3433	1	1	0.976	tgGTCAAact
AtSK21	P$WRKY62_01	WRKY62	3423	3431	1	1	0.957	tgGTCAAa
AtSK21	P$WRKY63_01	WRKY63	3423	3431	1	1	0.99	tgGTCAAa
AtSK21	P$WRKY75_01	WRKY75	3423	3431	1	1	0.975	tgGTCAAa
AtSK21	P$WRKY8_01	WRKY8	3423	3432	1	1	0.992	tgGTCAAac
AtSK21	P$WRKY23_01	WRKY23	3424	3432	1	1	0.854	gGTCAAac
AtSK21	P$WRKY30_01	WRKY30	3424	3434	1	1	0.918	gGTCAAactt
AtSK21	P$WRKY18_Q2	WRKY18	3425	3434	1	1	0.971	GTCAAactt
AtSK21	P$ARR2_01	ARR2	3432	3442	1	1	0.953	ttggATCTTt
AtSK21	P$GATA11_01	GATA11	3433	3441	1	1	0.867	tgGATCTt
AtSK21	P$GATA8_01	GATA8	3433	3442	1	1	0.976	tgGATCTtt
AtSK21	P$KNOX3_01	KNOX3	3437	3449	1	1	0.965	tcttTGACAata
AtSK21	P$WRKY11_Q2	WRKY11	3439	3447	1	1	0.924	tTTGACaa
AtSK21	P$ATH1_01	ATH1	3441	3449	1	1	0.92	TGACAata
AtSK21	P$LEC2_01	LEC2	3462	3473	1	1	0.944	atCATGCccta
AtSK21	P$MYB1L_01	MYB1L	3466	3476	1	1	0.977	tgCCCTAtct
AtSK21	P$TRB2_01	TRB2	3466	3474	1	1	0.984	tgCCCTAt
AtSK21	P$ARR2_01	ARR2	3468	3478	1	1	0.892	ccctATCTTt
AtSK21	P$AGL27_01	AGL27	3475	3489	1	1	0.882	tTTTCTatttatac
AtSK21	P$MRP1_Q2	MRP1	3476	3488	1	1	0.924	ttTCTATttata
AtSK21	P$HSFA4A_01	HSFA4A	3478	3484	1	1	0.914	tCTATT
AtSK21	P$ARR18_01	ARR18	3494	3507	1	1	0.911	ccttAGATAtaat
AtSK21	P$ATHB1_01	ATHB1	3500	3514	1	1	0.957	atataATTATtaaa
AtSK21	P$ATHB5_01	ATHB5	3503	3512	1	1	0.917	taaTTATTa
AtSK21	P$ASR1_01	ASR1	3513	3518	1	1	1	ACCCA
AtSK21	P$HSFA2_01	HSFA2	3515	3521	1	1	1	CCAAAa
AtSK21	P$GT1_Q6_01	GT1	3521	3533	1	1	0.881	TTTGTgttaaga
AtSK21	P$PEND_01	END	3528	3536	1	1	0.892	tAAGAAaa
AtSK21	P$BPC1_Q2	BPC1	3530	3536	1	1	0.997	AGAAAa
AtSK21	P$ASR1_01	ASR1	3543	3548	1	1	1	ACCCA
AtSK21	P$HSFA2_01	HSFA2	3545	3551	1	1	1	CCAAAa
AtSK21	P$GT1_Q6_01	GT1	3551	3563	1	1	0.881	TTTGTgttaaga
AtSK21	P$PEND_01	END	3558	3566	1	1	0.892	tAAGAAaa
AtSK21	P$BPC1_Q2	BPC1	3560	3566	1	1	0.997	AGAAAa
AtSK21	P$ASR1_01	ASR1	3573	3578	1	1	1	ACCCA
AtSK21	P$AP3_01	AP3	3573	3588	1	1	0.872	accCAAAAtttggaa
AtSK21	P$HSFA2_01	HSFA2	3575	3581	1	1	1	CCAAAa
AtSK21	P$MYB80_01	MYB80	3583	3594	1	1	0.854	tgGAATAtata
AtSK21	P$AT3G51080_01	AT3G51080	3608	3615	1	1	1	GGAAAaa
AtSK21	P$ATHB1_01	ATHB1	3612	3626	1	1	0.98	aaaaaATTATtgca
AtSK21	P$ATHB5_01	ATHB5	3615	3624	1	1	0.929	aaaTTATTg
AtSK21	P$PEND_02	END	3630	3640	1	1	0.974	atTTCTTata
AtSK21	P$GATA15_01	GATA15	3660	3669	1	1	1	ttTGATCaa
AtSK21	P$AZF3_01	AZF3	3667	3678	1	1	0.876	aAGTATccatt
AtSK21	P$GAMYB_Q2	GAMYB	3684	3697	1	1	0.882	attttACAACgct
AtSK21	P$AT5G54070_01	AT5G54070	3689	3695	1	1	0.958	aCAACG
AtSK21	P$ARR18_01	ARR18	3693	3706	1	1	0.888	cgctAGATAacga
AtSK21	P$CBNAC_01	CBNAC	3708	3714	1	1	1	tTGCTT
AtSK21	P$CBNAC_02	CBNAC	3708	3724	1	1	0.926	tTGCTTgaagtcgaac
AtSK21	P$GT1_Q6	GT1	3726	3733	1	1	0.912	GTGAAca
AtSK21	P$ATHB6_01	ATHB6	3737	3746	1	1	0.981	atAATAAtg
AtSK21	P$ATHB5_04	ATHB5	3737	3748	1	1	0.91	atAATAAtgta
AtSK21	P$ATHB1_03	ATHB1	3737	3748	1	1	0.904	atAATAAtgta
AtSK21	P$ATHB16_01	ATHB16	3738	3746	1	1	0.915	tAATAAtg
AtSK21	P$C1_Q2	C1	3747	3758	1	1	0.933	aaAACTAactt
AtSK21	P$WEREWOLF_Q2_01	WEREWOLF	3749	3758	1	1	0.919	aACTAActt
AtSK21	P$MYBAS1_01	MYBAS1	3749	3760	1	1	0.979	aaCTAACttgt
AtSK21	P$WRKY11_Q2	WRKY11	3760	3768	1	1	0.952	aTTGACta
AtSK21	P$ARR2_01	ARR2	3764	3774	1	1	0.899	actaATCTTt
AtSK21	P$GT1_Q6_01	GT1	3777	3789	1	1	0.888	TTTTTttacaat
AtSK21	P$ATHB6_01	ATHB6	3784	3793	1	1	0.997	acAATAAtg
AtSK21	P$ATHB5_04	ATHB5	3784	3795	1	1	0.924	acAATAAtgag
AtSK21	P$ATHB1_03	ATHB1	3784	3795	1	1	0.903	acAATAAtgag
AtSK21	P$ATHB16_01	ATHB16	3785	3793	1	1	0.878	cAATAAtg
AtSK21	P$GT1_Q6_02	GT1	3808	3820	1	1	0.975	attttaTTAACt
AtSK21	P$C1_Q2	C1	3814	3825	1	1	0.938	ttAACTAaata
AtSK21	P$GAMYB_Q2	GAMYB	3819	3832	1	1	0.888	taaatACAACgaa
AtSK21	P$AT5G54070_01	AT5G54070	3824	3830	1	1	0.958	aCAACG

AtSK21	P$AT3G20750_01	AT3G20750	3855	3863	1	1	0.938	aTAAACtt
AtSK21	P$SPF1_Q2	SPF1	3865	3875	1	1	0.881	tgATAGTgaa
AtSK21	P$GT1_Q6	GT1	3870	3877	1	1	0.971	GTGAAaa
AtSK21	P$KNOX3_01	KNOX3	3883	3895	1	1	0.962	acatTGACAatt
AtSK21	P$WRKY11_Q2	WRKY11	3885	3893	1	1	0.924	aTTGACaa
AtSK21	P$ATH1_01	ATH1	3887	3895	1	1	0.919	TGACAatt
AtSK21	P$MYBAS1_01	MYBAS1	3901	3912	1	1	0.976	ttCTAACattt
AtSK21	P$WRKY18_02	WRKY18	3927	3937	1	1	0.944	aatGTCAAtt
AtSK21	P$WRKY21_02	WRKY21	3927	3937	1	1	0.95	aatGTCAAtt
AtSK21	P$WRKY48_02	WRKY48	3927	3937	1	1	0.986	aatGTCAAtt
AtSK21	P$WRKY57_01	WRKY57	3927	3937	1	1	0.962	aatGTCAAtt
AtSK21	P$WRKY60_01	WRKY60	3927	3938	1	1	0.883	aatGTCAAttt
AtSK21	P$WRKY15_01	WRKY15	3928	3938	1	1	0.958	atGTCAAttt
AtSK21	P$WRKY2_01	WRKY2	3928	3936	1	1	0.902	atGTCAAt
AtSK21	P$WRKY25_02	WRKY25	3928	3936	1	1	0.879	atGTCAAt
AtSK21	P$WRKY40_01	WRKY40	3928	3936	1	1	0.977	atGTCAAt
AtSK21	P$WRKY43_02	WRKY43	3928	3938	1	1	0.952	atGTCAAttt
AtSK21	P$WRKY63_01	WRKY63	3928	3936	1	1	0.885	atGTCAAt
AtSK21	P$WRKY75_01	WRKY75	3928	3936	1	1	0.927	atGTCAAt
AtSK21	P$WRKY8_01	WRKY8	3928	3937	1	1	0.977	atGTCAAtt
AtSK21	P$WRKY30_01	WRKY30	3929	3939	1	1	0.904	tGTCAAttta
AtSK21	P$WRKY18_Q2	WRKY18	3930	3939	1	1	0.98	GTCAAttta
AtSK21	P$AT4G36620_01	AT4G36620	3943	3951	1	1	0.988	ttaAACCA
AtSK21	P$HSFA2_01	HSFA2	3948	3954	1	1	0.941	CCAAAc
AtSK21	P$UIF1_01	UIF1	3959	3969	1	1	0.859	catGATTCaa
AtSK21	P$AGL12_01	AGL12	3964	3976	1	1	0.876	ttcAAATTtgac
AtSK21	P$WRKY11_Q2	WRKY11	3970	3978	1	1	0.974	tTTGACcg
AtSK21	P$CBF3_02	CBF3	3970	3984	1	1	0.981	tttgaCCGACaagt
AtSK21	P$ZAP1_01	ZAP1	3971	3981	1	1	0.946	TTGACcgaca
AtSK21	P$CBF1_04	CBF1	3971	3983	1	1	0.968	ttgaCCGACaag
AtSK21	P$DREB1G_02	DREB1G	3972	3982	1	1	0.912	tgaCCGACaa
AtSK21	P$AT1G77200_03	AT1G77200	3972	3986	1	1	0.944	tgaCCGACaagtac
AtSK21	P$ARF1_01	ARF1	3974	3982	1	1	1	aCCGACaa
AtSK21	P$ARF5_01	ARF5	3974	3982	1	1	0.994	aCCGACaa
AtSK21	P$DREB1B_01	DREB1B	3975	3980	1	1	1	CCGAC
